# Isolation and NMR Scaling Factors for the Structure Determination of Lobatolide H, a Flexible Sesquiterpene from *Neurolaena lobata* †

**DOI:** 10.3390/ijms24065841

**Published:** 2023-03-19

**Authors:** Tibor Kovács, Ildikó Lajter, Norbert Kúsz, Zsuzsanna Schelz, Noémi Bózsity-Faragó, Anikó Borbás, István Zupkó, Georg Krupitza, Richard Frisch, Judit Hohmann, Andrea Vasas, Attila Mándi

**Affiliations:** 1 Department of Organic Chemistry, University of Debrecen, P.O. Box 400, 4002 Debrecen, Hungary; 2 Doctoral School of Chemistry, University of Debrecen, Egyetem tér 1, 4032 Debrecen, Hungary; 3 Institute of Pharmacognosy, University of Szeged, Eötvös u. 6, 6720 Szeged, Hungary; 4 Institute of Pharmacodynamics and Biopharmacy, University of Szeged, Eötvös u. 6, 6720 Szeged, Hungary; 5 Department of Pharmaceutical Chemistry, University of Debrecen, Egyetem tér 1, 4032 Debrecen, Hungary; 6 Department of Pathology, Medical University of Vienna, Waehringer Guertel 18-20, 1090 Vienna, Austria; 7 Institute for Ethnobiology, Playa Diana, San José GT-170, Guatemala; 8 ELKH-USZ Biologically Active Natural Products Research Group, University of Szeged, Eötvös u. 6, 6720 Szeged, Hungary

**Keywords:** natural product, stereochemistry, NMR shift parameter development, DFT calculations, ECD calculations, antiproliferative activity, antimigratory effect

## Abstract

A new flexible germacranolide (**1**, lobatolide H) was isolated from the aerial parts of *Neurolaena lobata*. The structure elucidation was performed by classical NMR experiments and DFT NMR calculations. Altogether, 80 theoretical level combinations with existing ^13^C NMR scaling factors were tested, and the best performing ones were applied on **1**. ^1^H and ^13^C NMR scaling factors were also developed for two combinations utilizing known exomethylene containing derivatives, and the results were complemented by homonuclear coupling constant (*J*_HH_) and TDDFT-ECD calculations to elucidate the stereochemistry of **1**. Lobatolide H possessed remarkable antiproliferative activity against human cervical tumor cell lines with different HPV status (SiHa and C33A), induced cell cycle disturbance and exhibited a substantial antimigratory effect in SiHa cells.

## 1. Introduction

Sesquiterpene lactones (SLs) constitute a large and diverse group of biologically active plant specialized metabolites that have been identified in several plant families. The greatest numbers are found in the family Asteraceae with over 3000 reported different structures [1,2,3]. They are primarily classified on the basis of their carbocyclic skeletons. From over 40 structural types of sesquiterpene lactones known to date, the most widespread are germacrane, guaiane, eudesmane, and pseudoguaiane [4]. An important structural feature of the SLs is the presence of a *γ*-lactone ring containing in many cases an *α*-methylene group. The biological activity (e.g., cytotoxic [1,5,6,7,8], anti-inflammatory [2,9,10,11]) of SLs is mainly due to the presence of this structural element [12,13,14]. Furthermore, there are also reports on neuroprotective [15,16], antimicrobial [17] or antiparasitic [18,19] activities of SL derivatives.

*Neurolaena lobata* (L.) R.Br. ex Cass. (Asteraceae) is a perennial plant occurring mainly in Central and South America. It is a rich source of SLs; previously 24 sesquiterpenes, among them 22 SLs having germacranolide, *seco*-germacranolide, furanoheliangolide and eudesmanolide skeletons were isolated from the aerial parts of the plant. Several of them possessed remarkable antiproliferative and anti-inflammatory activities [20,21,22,23,24].

Although DFT NMR calculations of ^1^H and ^13^C chemical shifts and the associated statistical methods [25,26] are a powerful tool which allowed the structure elucidation [27,28,29,30] or even structure revision [31,32,33,34] of a large number of natural and synthetic derivatives, they have several limitations [26,33,35,36]. Regardless of one’s aims to apply a scaling factor approach or an inner reference approach, for a successful determination of the relative/absolute configuration, the difference between the experimental values of the sample and computed data of the correct isomer should be small, while the wrong isomers should show larger differences from the experimental data. It is known that some atoms or groups can cause large deviations in the computed values, preventing a good reproduction of the experimental NMR data in the vicinity [37,38]. If there is no chirality center next to the problematic atom or group, one can neglect the highly affected atoms, which is usually applied for the training sets, too [27]. If the problematic region is far from the chirality center(s) to be determined, even truncated models can be applied for the NMR calculations [28,29,39]. There are, however, a few examples in the literature, where even for rather problematic cases, such as halogen atoms, well performing scaling factors could be prepared [40]. Exomethylene groups are less problematic than halogen atoms, but still capable of exerting a distortion of several ppm, which is comparable with the difference between the possible stereoisomers and, hence, thwarts a safe assignment [24,41,42]. For example, in our previous study on lobatolide derivatives, the ^13^C NMR chemical shift data of lobatolide A could be well reproduced, but for most of the other exomethylene containing lobatolides, large differences were found in the vicinity of the exomethylene group preventing utilization of the calculated NMR scaling factors in structure elucidation [24]. Since a large number of level combinations with NMR scaling factors are already available in the literature [27,43], and for some of them, even exomethylene containing derivatives were considered in the training set [41], we hoped to find a few well applicable combinations for germacranolides including the novel flexible natural product (**1**). On the other hand, the vast majority of the combinations found in the literature apply B3LYP or other classical functionals for DFT optimization, and almost all combinations were prepared for gas-phase optimized conformers [44]. These combinations can work well for rigid molecules, but for open chain compounds or macrolides with high flexibility they can be problematic [45,46]. To this end, we developed scaling factors by applying the ωB97XD long-range corrected hybrid functional [47,48] for geometry optimization both in vacuo and with SMD (solvation model based on density) for chloroform.

## 2. Results and Discussion

**Figure 1:**
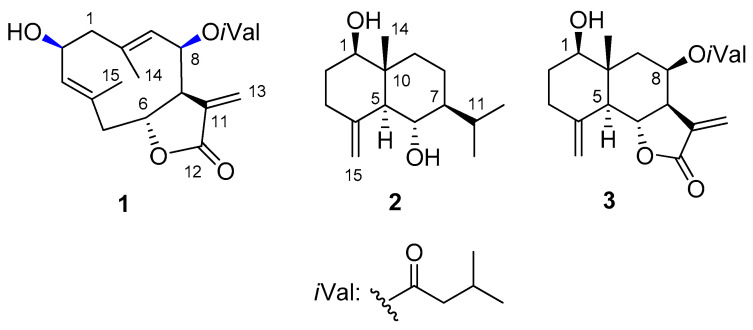
Structures of the novel compound (**1**) and the known NMR test compounds (**2**, volenol, and **3**, 8*β*-isovaleroyloxyreynosin) isolated from *N. lobata*. Blue indicates the problematic chirality centers.

Multistep chromatography of the dichloromethane-soluble phase of the methanolic extract prepared from the aerial parts of *N. lobata* resulted in the isolation of a pure compound (**1**, Figure 1). The structure elucidation of **1** was carried out by one- and two-dimensional NMR spectroscopy (^1^H–^1^H COSY, HSQC, HMBC and NOESY) and HRESIMS experiments and TDDFT-ECD calculations.

Compound **1** (lobatolide H) was isolated as a yellow gum with [*α*]^27^_D_ + 31 (*c* 0.1, CHCl_3_). Its HRESIMS displayed a quasi-molecular ion peak at *m*/*z* 349.2012 [M + H]^+^ (calcd. for 349.2010), indicating the molecular formula C_20_H_28_O_5_. The ^1^H and ^13^C NMR spectra of **1** showed the presence of an isovaleroyl group (Table 1). Additionally, the 1D and 2D NMR spectra (Figures S1–S6) exhibited that this compound is very similar to the germacranolide-type sesquiterpene lactone 2*α*-hydroxy-8*β*-isovaleroyloxycostunolide, isolated previously from *Helianthus gracilentus* (Asteraceae) [49]. The ^13^C NMR data of the two compounds were completely in agreement. Only two differences could be detected between the two compounds. Firstly, the positions of the double bonds differ. These were present at C-3–C-4 and C-9–C-10 in **1**, but at C-4–C-5 and C-1–C-10 in 2α-hydroxy-8*β*-isovaleroyloxycostunolide. Secondly, the orientation of OH-2 group in **1** differs from the proposed *α*-orientation of this substituent in case of the costunolide derivative. The position of the double bond was proved by ^1^H-^1^H COSY correlations. The ^1^H-^1^H COSY spectrum defined two structural fragments with correlated protons: –CH_2_-CH(OH)-CH= (A) (*δ*_H_ 2.72, 2.07, 4.75, 5.25) (C-1–C-3) and –CH_2_–CH(OR)–CH(R)–CH(OR)–CH= (B) (*δ*_H_ 2.76, 2.32, 5.76, 2.93, 5.50, and 4.98) (C-5–C-9). These two structural parts, tertiary methyls (*δ*_H_ 1.79 and 1.53), and quaternary carbons (*δ*_C_ 135.1, 136.4, 142.7, and 169.4) were connected by inspection of the HMBC correlations. The two- and three-bond correlations between the quaternary carbon C-4 and H-2, H-5a, H-6, and H-15; between C-10 and H-1a, H-1b, H-8, and H-14; and between C-7 and H-13a, H-13b revealed the presence of a germacranolide-3,9-diene structure substituted with a hydroxy group at C-2, and an isovaleroyloxy group at C-8.

The *β*-orientation of the 2-OH and 8-isovaleroyl group was indicated by the NOE correlations between H-2/H-1a, H_3_-14; H_3_-14/H-1a, and H-8; and H-8/H-2. The *E* configuration of the olefinic bonds was demonstrated by the NOESY cross-peaks between H-14/H-1*α* and H-9/H-1*β*, and between H-15/H-2*α* and H-3/H-1*β*. All the above data suggested lobatolide H to have the structure **1**.

Although NOE correlations work well in smaller rings, for flexible systems or macrolides one should be careful with the interpretations to avoid misassignments [24,50,51]. Therefore, we tested a large number of available DFT NMR methods, developed parameters for two combinations and augmented the NMR studies with TDDFT-ECD calculations to verify the stereochemistry of **1**. To help the reader navigate between the tested and developed NMR chemical shift scaling factor combinations, we named approach A the test of three known DFT combinations on lobatolide H (**1**), which we used successfully lately on various heterocycles, including lobatolides A and B [24,28,29,52,53]. In approach B we tested 80 available combinations first on the known derivative volenol (**2**, step 1), and then the best performing ones on 8*β*-isovaleroyloxyreynosin (**3**, step 2) and **1** (step 3). In approach C we developed chemical shift scaling factors for two further combinations based on eleven exomethylene containing derivatives and tested them for **2**, **3** and **1**.

**Approach A**

Since in our previous work we could predict the ^13^C chemical shifts of lobatolides A and B with a good approximation but encountered problems for other lobatolide derivatives [24], we tested three DFT combinations with available scaling factors on **1**, namely, the mPW1PW91/6-311+G(2d,p)//B3LYP/6-31+G(d,p) [27], the mPW1PW91/6-311+G(2d,p) SMD/CHCl_3_//B3LYP/6-31+G(d,p) [27] and the mPW1PW91/6-311+G(2d,p) SMD/CHCl_3_//mPW1PW91/6-311+G(2d,p) SMD/CHCl_3_ [54] combinations, which were successfully utilized by us for other derivatives, recently [28,29,52,53]. The absolute configuration of the C-6, C-7 and C-10 chirality centers seems to be biosynthetically conserved in lobatine and related derivatives if applicable; thus, four possible diastereomers of **1** were considered with different configurations at the C-2 and C-8 chirality centers, namely, (2*R*,6*R*,7*S*,8*R*)-**1**, (2*R*,6*R*,7*S*,8*S*)-**1**, (2*S*,6*R*,7*S*,8*R*)-**1** and (2*S*,6*R*,7*S*,8*S*)-**1**. The experimentally most plausible isomer, (2*S*,6*R*,7*S*,8*R*)-**1**, was denoted as **isomer 1**, (2*R*,6*R*,7*S*,8*R*)-**1** as **isomer 2**, (2*R*,6*R*,7*S*,8*S*)-**1** as **isomer 3** and (2*S*,6*R*,7*S*,8*S*)-**1** as **isomer 4**. The results obtained at the three combinations were contradictory (Table 2 and Tables S3–S5). While the mPW1PW91/6-311+G(2d,p)//B3LYP/6-31+G(d,p) level suggested **isomer 2**, the mPW1PW91/6-311+G(2d,p) SMD/CHCl_3_//B3LYP/6-31+G(d,p) level gave similarly good results for **isomer 1** and **isomer 2**, and only **isomer 4** yielded a considerably higher mean absolute error (MAE) value. The mPW1PW91/6-311+G(2d,p) SMD/CHCl_3_//mPW1PW91/6-311+G(2d,p) SMD/CHCl_3_ level gave similar results to mPW1PW91/6-311+G(2d,p)//B3LYP/6-31+G(d,p), preferring **isomer 2** and rating **isomer 1** the second best. The DP4+ [26] results suggested **isomer 2** with two combinations and **isomer 1** with 1 combination as the most plausible structure, but in the latter case, both **isomer 2** and **isomer 3** had considerable possibilities. It is important to note here, that ECD calculations allow exclusion of **isomer 3** (vide infra).

**Approach B**

Parallel with approach A, we also performed a three-step test for a large number of DFT combinations with available NMR chemical shift parameters. In the first evaluation step, 79 combinations were selected from the CHESHIRE Chemical Shift Repository database as of 5 October 2018 [27,43,44,55,56,57]. Basically, all combinations were selected with available ^13^C NMR scaling factors that were developed with reference to experimental data measured in CDCl_3_ and either in gas-phase or based on Gaussian 09 solvent model calculations. (Solvent model implementations in previous versions of the software package were different; therefore, parameters developed with earlier versions are not comparable with calculations obtained by more recent versions). The scaling factors found in the CHESHIRE database are usually developed on a larger set of small, rigid but diverse organic compounds (e.g.*,* a few dozens in Tantillo et al. [27] and Pierens [43] works). Since the CHESHIRE database did not contain the third combination applied in approach A, we supplemented the 79 combinations with the mPW1PW91/6-311+G(2d,p) SMD/CHCl_3_//mPW1PW91/6-311+G(2d,p) SMD/CHCl_3_ method. Thus the 80 combinations in total (see Table S1 in the Supplementary Materials) were tested for volenol (**2**) in the first step. The initial 24 MMFF conformers of (1*R*,5*S*,6*S*,7*S*,10*R*)-**2** were optimized independently at all the given DFT optimization levels (Tables S30–S37) and chemical shifts were computed at the corresponding NMR levels with the GIAO method [58]. The resulting values were Boltzmann averaged and corrected according Equation 1 utilizing the corresponding scaling factors (Table S1). The resulting computed ^13^C NMR chemical shift values were compared with the experimental values of **2** [24,59]. Mean absolute error (MAE) values were calculated from the absolute differences of the calculated and experimental data (see Table S2 in the Supplementary Materials), and the methods were ranked according to their MAE values while taking also the maximum absolute errors (Δδ_max_) into account. The best performing four combinations were chosen for the second evaluation step, namely, mPW1PW91/6-31G(d)//M06-2X/6-31G(d), mPW1PW91/6-31G(d) SMD/CHCl_3_//M06-2X/6-31G(d), M06/6-31G(d)//B3LYP/6-31+G(d,p) and OPBE0/6-31G(d)//B3LYP/6-31+G(d,p). It is interesting to note, that none of the three combinations of approach A were among the best ones for **2**.

*δ* = (*Intercept* − *σ*)/−*Slope* (1)

In the second evaluation step, the four best performing combinations of the first step were tested for 8*β*-isovaleroyloxyreynosin (**3**) that contains two exomethylene moieties, one in a five-membered and one in a six-membered ring. Since **3** contains a chirality center at C-8 with the same substitution pattern as **1** and C-8 was one of the problematic chirality centers in **1**, we aimed to differentiate the two C-8 epimers with the four NMR combinations. The calculations were performed similarly to **2** for the 65 and 92 initial MMFF conformers of (1*R*,5*S*,6*R*,7*R*,8*R*,10*R*)-**3** and (1*R*,5*S*,6*R*,7*R*,8*S*,10*R*)-**3**, respectively. The resulting MAE and DP4+ values indicated the mPW1PW91/6-31G(d) SMD/CHCl_3_//M06-2X/6-31G(d) and the M06/6-31G(d)//B3LYP/6-31+G(d,p) methods to be better than the other two (Table 3 and Tables S6–S9). The mPW1PW91/6-31G(d)//M06-2X/6-31G(d) combination, which is similar to mPW1PW91/6-31G(d) SMD/CHCl_3_//M06-2X/6-31G(d) but lacks the solvent model in the NMR calculation step, showed less difference between the epimers, while the last OPBE0/6-31G(d)//B3LYP/6-31+G(d,p) combination yielded much larger MAE values than the others. Here we want to note that the DP4+ method was developed for 24 combinations utilizing only the B3LYP and mPW1PW91 functionals with various basis sets, and application of the method for chemical shifts calculated at considerably different combinations can be misleading [26,35]. Therefore, the DP4+ values of all other combinations are shown only for information and where contradictory, the MAE values should be considered.

In the third step, the best performing two combinations of step 2 were applied for the above four isomers of **1**. While the mPW1PW91/6-31G(d) SMD/CHCl_3_//M06-2X/6-31G(d) combination showed the best agreement for **isomer 1**, it rated **isomer 2** and **isomer 3** with similar MAE values the second best. The M06/6-31G(d)//B3LYP/6-31+G(d,p) method rated **isomer 4** and **isomer 2** similarly good and yielded considerably higher MAE values for **isomer 1** and **isomer 3** (Table 4, Tables S10 and S11).

**Approach C**

**Figure 2:**
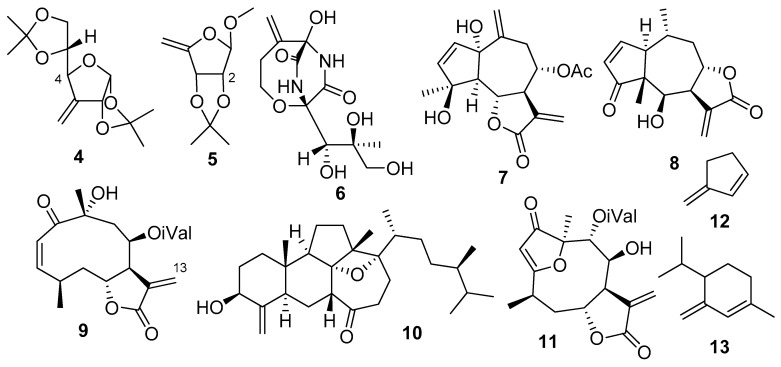
Structures of the compounds considered only in the reference set of the novel NMR shift methods: 3-deoxy-1,2;5,6-di-*O*-isopropylidene-3-*C*-methylene-*α*-D-*ribo*-hexofuranose (**4**), methyl 5-deoxy-2,3-*O*-isopropylidene-*β*-D-*erythro*-pent-4-enofuranoside (**5**), bicyclomycin (**6**), 1*α*,4*β*-dihydroxy-8*α*-acetoxy-guaia-2,10(14),11(13)-triene-6,12-olide (**7**), mexicanin I (**8**), neurolenin A (**9**), swinhoeisterol F (**10**), lobatolide A (**11**), 3-methylenecyclopent-1-ene (**12**), 4-isopropyl-1-methyl-3-methylenecyclohex-1-ene (**13**).

The contradictory results indicate that flexibility of **1** can play a significant role in the low reproducibility of the chemical shift values. If we consider the widely available or the tested 80 combinations, it is obvious that almost all of them use classical (mostly B3LYP) functionals for DFT geometry optimization, and there is only one combination (mPW1PW91/6-311+G(2d,p) SMD/CHCl_3_//B3LYP/6-31+G(d,p) SMD/CHCl_3_) [27] in the CHESHIRE chemical shift database which also applies a solvent model for the optimization level [44]. The additionally tested functional (mPW1PW91/6-311+G(2d,p) SMD/CHCl_3_//mPW1PW91/6-311+G(2d,p) SMD/CHCl_3_) was not part of the database, perhaps due to the very limited reference compound set utilized for creating the chemical shift parameters [54]. Although for small and rigid molecules, which usually constitute the reference set of the NMR scaling parameters, the available level combinations seem to be sufficient, for flexible molecules, however, better performing functionals and consideration of the solvent effect also for the DFT optimization can be crucial to obtaining the low-energy conformers and estimate their Boltzmann populations correctly. To this end, we selected the ωB97XD functional [40,47,48] for DFT optimization level both in vacuo and with SMD solvent model for chloroform, and combined it with the well performing mPW1PW91 functional as an NMR calculation level with the same or no solvent model as in the corresponding DFT optimization step (Tables S38 and S39). As reference set, we have chosen 11 exomethylene containing molecules with different flexibilities (**2**, **4**–**13**, Figure 2 and Figure S14) [23,24,59,60,61,62,63,64,65,66,67,68,69,70,71]. One of the molecules contained no chirality centers (**12**), one had only one chirality center (**13**), while the relative or absolute configuration of the others was known from the literature, secured by X-ray measurements, synthetic or biosynthetic considerations.

**Figure 3:**
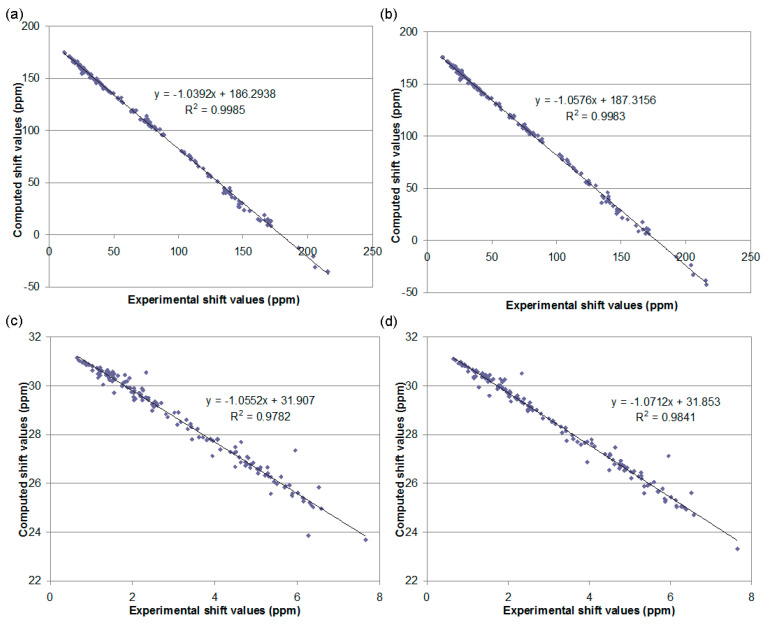
Correlation plots for computed ^13^C and ^1^H NMR data for the reference set: (**a**) ^13^C gas phase, (**b**) ^13^C SMD, (**c**) ^1^H gas phase and (**d**) ^1^H SMD.

As Figure 3 shows, the computed ^13^C NMR data shows an excellent correlation with the experimental chemical shift data for the reference set at both level combinations. On the other hand, larger deviations were observed for the ^1^H shift values of a few protons in both cases. We investigated the most discordant cases, such as H-7 of **2**, H-2 of **5**, H-13a and b of **9** or H-4 of **4**, but no better assignments could be found than those described in the literature. Unfortunately, some of these hydrogens are connected to a chirality center, which limits the application of the ^1^H chemical shift data as a solid proof. Accordingly, we will rely more on the better performing ^13^C parameters and the corrected shift values obtained by these if the results are contradictory.

The novel parameters were first tested back on **2** (Figure S11). The calculated chemical shifts of most carbons both in the gas phase and the SMD combination showed close agreement with the experimental values (Table 5 and Table 6). Only the exomethylene carbons exhibited larger deviations. However, most importantly, the carbons in the vicinity of the exomethylene moiety showed good correlation with the experimental data. The MAE values derived from the difference of the experimental and the computed ^1^H chemical shift data are relatively small (0.18 and 0.19), but larger differences were found for the H-3, H-8 and H-9 hydrogens, and H-7 showed a particularly large difference as this hydrogen was one of the most problematic in the reference set already (see Tables S12 and S13 in the Supplementary Materials).

Thereafter, the novel chemical shift parameters were also tested on the two above epimers of **3** (Figures S12 and S13). The computed ^13^C chemical shift data obtained with both the novel in vacuo and SMD level parameters favored the (1*R*,5*S*,6*R*,7*R*,8*R*,10*R*)-**3** epimer, but the solvent model calculations showed substantially larger difference between the isomers and smaller MAE for the correct one (Table 7, Tables S14 and S15). In contrast to **2**, the results of the ^1^H NMR chemical shift calculations at both levels gave excellent agreement for almost all protons of the correct isomer, and higher differences were found for some characteristic protons in the vicinity of the epimeric center in the wrong stereoisomer (see Tables S16 and S17 in the Supplementary Materials). That is, both the carbon and proton results favored the correct (1*R*,5*S*,6*R*,7*R*,8*R*,10*R*)-**3** epimer at both novel combinations of levels.

Finally, the initial 133, 124, 252 and 201 MMFF conformers of the four possible stereoisomers of **1** were also re-optimized at the ωB97XD/6-31+G(d,p) and ωB97XD/6-31+G(d,p) SMD/CHCl_3_ levels (Figures S7–S10). NMR shift data were calculated for the conformers above 1% Boltzmann distribution at the mPW1PW91/6-311+G(2d,p) and mPW1PW91/6-311+G(2d,p) SMD/CHCl_3_ levels, respectively, and corrected with the novel parameters. The ^13^C data obtained at both combinations of levels favored **isomer 1** (Table 8, Tables S18 and S19), while the ^1^H data suggested **isomer 2** (see Tables S20 and S21 in the Supplementary Materials). As indicated above, the DP4+ statistical analysis gave unexpected results in contrast to the MAE values similar to the second combination in Table 3, due to the large difference in the combinations the method was originally developed for, and thus, the sDP4+ percentages were neglected.

**Coupling constant calculations**

To verify the result of the ^13^C data, homonuclear coupling constants were also calculated with the last SMD combination for key protons in the macrolide ring of the two favored stereoisomers (**isomer 1** and **isomer 2**), and compared with the experimental values (Table 9). The results significantly preferred **isomer 1 in line with the ^13^C calculations of approach C. Especially *J*_1aH-2H_ and *J*_2H-3H_ showed large differences between the calculated data of the two isomers and also in comparison with the experimental values, where isomer 1** reproduced the experimental data well. This also leads to the conclusion that the calculations better reproduced the conformation of the western part of the molecule than the eastern part, and that macrocycles are still a tough target to study even with otherwise well performing DFT functionals. At the suggestion of one of the reviewers, we tested the J-DP4 method [72] for the coupling constant data of the two isomers, resulting in a 98.21% probability for **isomer 1**. However, we have to indicate that similarly to the DP4+ method there are differences in the applied level and the level the method was developed for.

**TDDFT-ECD calculations**

**Figure 4:**
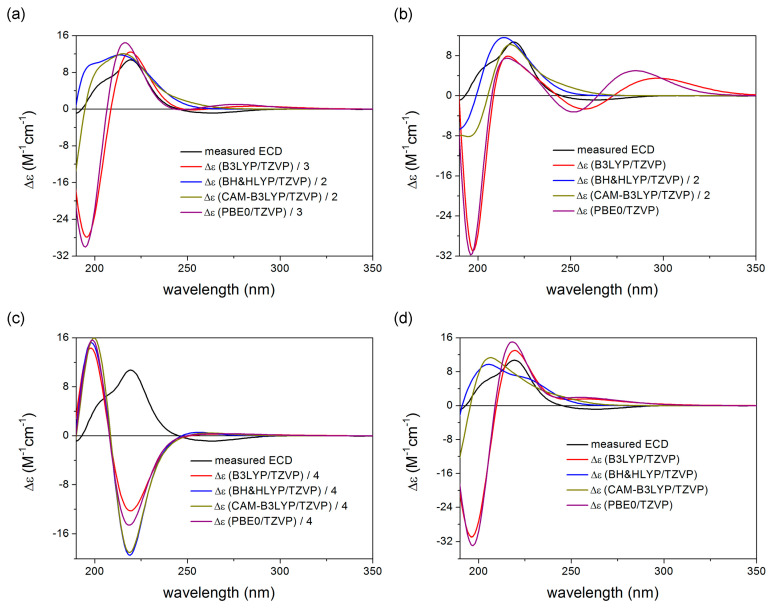
Comparison of the experimental ECD spectrum of **1** with the calculated spectra obtained at various levels of theory for the (**a**) 16 low-energy CAM-B3LYP/TZVP PCM/MeCN conformers of **isomer 1**, (**b**) 10 low-energy CAM-B3LYP/TZVP PCM/MeCN conformers of **isomer 2**, (**c**) 22 low-energy CAM-B3LYP/TZVP PCM/MeCN conformers of **isomer 3**, and (**d**) 25 low-energy CAM-B3LYP/TZVP PCM/MeCN conformers of **isomer 4**.

To elucidate the absolute configuration and further verify the NMR results, TDDFT-ECD calculations [73,74,75] were performed on the MMFF conformers of the four possible isomers of 1. The initial conformers were re-optimized at the CAM-B3LYP/TZVP [76] PCM/MeCN level (Table S40) and rotatory strength values were computed at four different levels similarly to the recently described lobatolides [24]. While the Boltzmann average for three isomers (isomer 1, isomer 2 and isomer 4) gave acceptable agreement with the experimental ECD spectrum (the high wavelength n-π^*^ transitions were reported to be hard to reproduce in several cases in the literature [77,78]), isomer 3 showed a mirror-image relationship (Figure 4). That is, assuming homochirality with the known lobatolides at the conserved chirality centers C-6 and C-7, isomer 3 can be excluded as a possible isomer, and accepting the results of the novel NMR combinations and the coupling constant calculations, the homochiral nature of 1 with the known lobatolides can be verified. Based on the above-described NMR and ECD calculations, the (2*S*,6*R*,7*S*,8*R*) absolute configuration was assigned for 1.

**Antiproliferative activity**

The antiproliferative activity of compound 1 was tested on three cervical cancer cell lines of different human papilloma virus (HPV) of different status (HeLa, SiHa, and C33A) and on non-cancerous (NIH/3T3 (mouse embryonic fibroblast) and MRC-5 (human fibroblast)) cell lines. Cisplatin was used as a positive control. Lobatolide H (1) showed remarkable growth-inhibitory effects against SiHa (IC_50_ 2.82 µM) and C33A (IC_50_ 4.43 µM) cells, whereas it exerted weak activity against HeLa (IC_50_ 16.62 µM) cell line (Table 10). These results were comparable to that of the reference agent cisplatin. According to the calculated IC_50_ values on the two non-cancerous fibroblast cell lines, cancer selectivity could be determined. The best selectivity was obtained in the case of the Siha cells (SI = 5.26).

**Figure 5:**
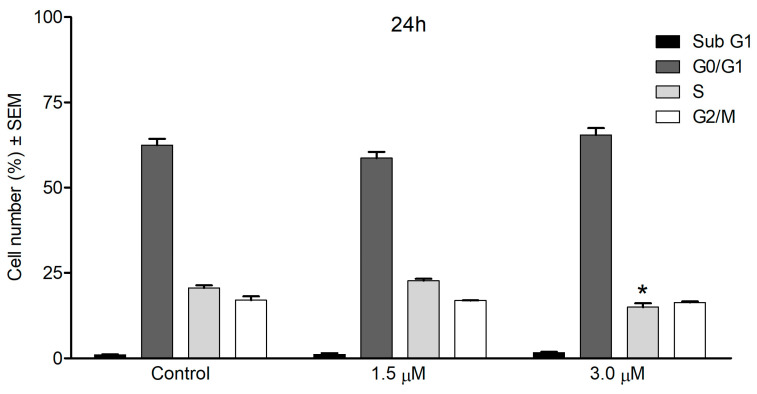

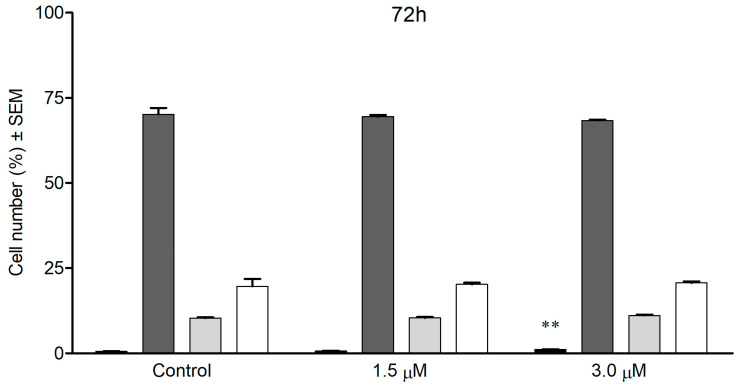
Cell cycle distributions of SiHa cells after treatment with compound **1** for 24 (**upper panel**) or 72 h (**lower panel**). Distribution of cell populations in different cell cycle phases. * and ** indicate *p* < 0.05 and *p* < 0.01, respectively, by means of one-way ANOVA followed by Dunnett’s post hoc test.

The most sensitive SiHa cell line was chosen to further investigate the possible mechanisms behind the antiproliferative effects, and cell-cycle analysis was performed. The cell-cycle analysis showed only slight differences in the distribution of cell cycle phases compared with the untreated control cells. After 24 h, 3 μM of compound **1** elicited a significant depression in the S phase population with no relevant change in other cell phases. After a longer exposure (72 h), the sub G1 population had grown significantly but to a modest extent when treated with 3 μM (Figure 5).

Based on the results of the antiproliferative assay, compound **1** was additionally investigated for its antimetastatic activity. A wound healing assay was performed on the SiHa cell line in 1.5 µM and 3.0 µM concentrations.

**Figure 6:**
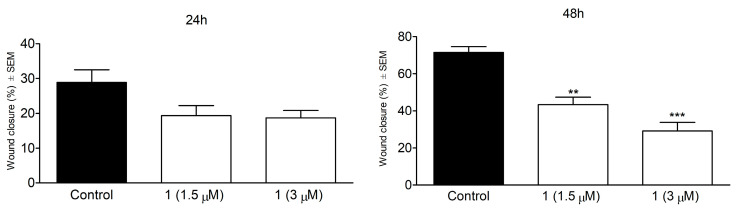

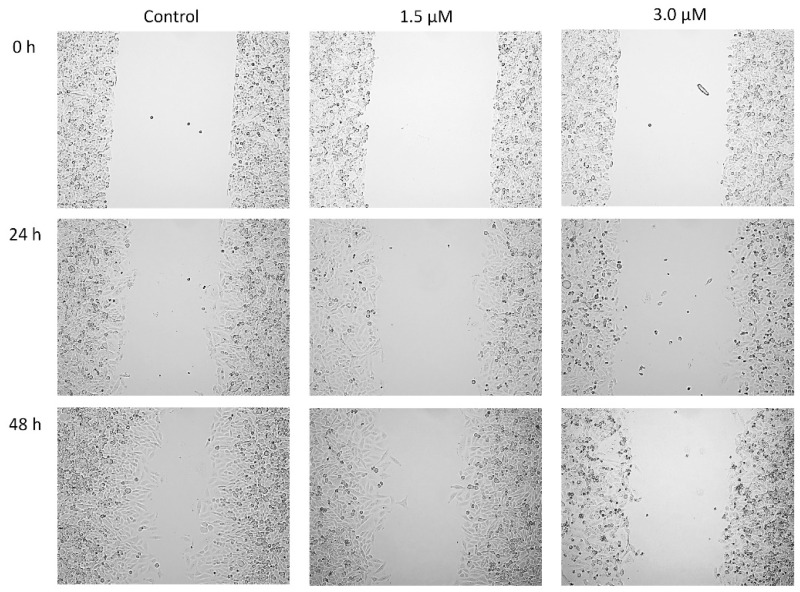
Wound healing assay. Effect of compound **1** on the migration of SiHa cancer cells after 24 and 48 h of incubation (**left and right upper panels**, respectively). ** and *** indicate *p* < 0.01 and *p* < 0.001, respectively, by means of one-way ANOVA followed by Dunnett’s post hoc test. **Lower panel**: representative images of reduced wound healing at 0, 24 and 48 h post-treatment.

Treatment with compound **1** resulted in no significant reduction in the migration of cervical cancer cells (Figure 6). A longer exposure, however, elicited a substantial and concentration-dependent decrease in the closure of the cell-free area, which indicates the inhibition of migration of the treated cells.

## 3. Materials and Methods

### 3.1. General Procedures

The high-resolution MS spectra were acquired on a Thermo Scientific Q-Exactive Plus orbitrap mass spectrometer equipped with an ESI ion source in positive ionization mode. The samples were dissolved in MeOH. Data acquisition and analysis were accomplished with Xcalibur software version 2.0 (Thermo Fisher Scientific). A Bruker Avance DRX 500 spectrometer (500 MHz (^1^H) and 125 MHz (^13^C)) was used for recording the NMR spectra. The signals of the deuterated solvent CDCl_3_ were taken as the reference. 2D NMR data were acquired and processed with standard Bruker software TopSpin 3.6.1. Gradient-enhanced versions were used in the ^1^H–^1^H COSY, HSQC and HMBC experiments. Optical rotations were determined in CHCl_3_ by using a Perkin-Elmer 341 polarimeter. UV and ECD spectra were recorded on a JASCO J-810 spectropolarimeter.

For column chromatography (CC), polyamide (MP Polyamide, 50–160 μm, MP Biomedicals, Irvine, CA, USA) and silica gel (Kieselgel 60, 63–200 μm, Merck, Darmstadt, Germany) were used. Preparative thin-layer chromatography (prep. TLC) was carried out using RP-18 (F_254s_, Merck) pre-coated plates.

### 3.2. Plant Material

*Neurolaena lobata* (L.) R.Br. ex Cass. (Asteraceae) was collected by R. Diaz, and R. O. Frisch (Institute for Ethnobiology, Playa Diana, GT-170 San José/Petén, Guatemala), in the flowering period, in the area of the Chakmamantok rock formation (16 59′16″ N, 89 53′45″ W) in San José, Guatemala. A voucher specimen (No. 813) has been deposited at the Herbarium of Department of Pharmacognosy, University of Szeged, Szeged, Hungary.

### 3.3. Extraction and Isolation

The dried and ground material from the aerial parts of the plant (3.00 kg) was percolated with MeOH (50 L) at room temperature. The extract was concentrated under reduced pressure and solvent–solvent partition was performed with 5 × 1 L petroleum ether (A), 5 × 1 L of CH_2_Cl_2_ (B), and finally with 5 × 1 L of EtOAc (C). The CH_2_Cl_2_ phase (95.4 g) was separated on a polyamide column (287 g) with mixtures of MeOH and H_2_O (1:4, 2:3, 3:2 and 4:1, 3 L of each) as eluents to afford seven fractions (BI–BVII). Fraction BIII (7.6 g) obtained from the polyamide column with MeOH–H_2_O (3:2) was subjected to silica gel VLC, using a gradient system of cyclohexane–EtOAc–EtOH (from 30:5:0 to 30:30:2) to yield 10 fractions (BIII/1–10). Fraction BIII/8 (0.6 g) was rechromatographed on VLC with a gradient system of cyclohexane–EtOAc–EtOH (from 3:1:0 to 30:30:3) and 10 subfractions (BIII/8/1–10) were obtained. Purification of subfraction BIII/8/8 by preparative RP-TLC (MeOH–H_2_O 7:3) resulted in the isolation of compound **1** (28.7 mg).

### 3.4. Physical Characteristics of the New Compound

*Lobatolide H* (**1**): a yellow gum; [*α*]^27^_D_ +31 (*c* 0.1, CHCl_3_); UV (MeCN, λ_max_ (nm)) 204sh, <190; ECD (MeCN, λ (nm) (Δε), c 6.65 × 10^−4^ M): 263 (-0.87), 219 (+10.73), 206sh (+6.41); ^1^H NMR and ^13^C NMR data, see Table 1; HRESIMS *m*/*z* 349.2012 [M + H]^+^ (calcd. for C_20_H_29_O_5_, 349.2015).

### 3.5. Computational Section

Mixed torsional/low-mode conformational searches were carried out by means of the Macromodel 10.8.011 software using the MMFF with an implicit solvent model for CHCl_3_ and applying a 21 kJ/mol energy window [79]. Geometry re-optimizations of the resultant conformers (CAM-B3LYP/TZVP PCM/MeCN for the ECD calculations, ωB97XD/6-31+G(d,p) in vacuo and ωB97XD/6-31+G(d,p) SMD/CHCl_3_ for the novel NMR parameters, see Table S1 for tests of the 80 NMR combinations), TDDFT-ECD (B3LYP/TZVP PCM/MeCN, BH&HLYP/TZVP PCM/MeCN, CAM-B3LYP/TZVP PCM/MeCN and PBE0/TZVP PCM/MeCN), and DFT-NMR calculations (mPW1PW91/6-311+G(2d,p) in vacuo and mPW1PW91/6-311+G(2d,p) SMD/CHCl_3_ for the novel NMR parameters, see Table S1 for test of the 80 NMR combinations) were performed with the Gaussian 09 package [80,81,82]. ECD spectra were generated as sums of Gaussians with 3000 cm^−1^ width at half-height, using dipole-velocity-computed rotational strength values [83]. Computed NMR shift data were corrected with the scaling factors listed in Table S1 or determined in the current work. Boltzmann distributions were estimated from the DFT energies. Visualization of the results was performed by the MOLEKEL 5.4 software package [84].

### 3.6. Antiproliferative MTT Assay

The antiproliferative effects of the isolated compounds were determined in vitro using SiHa (HPV 16+), HeLa (HPV 18+), and C33A (HPV negative) human cervical cell lines, and NIH-3T3 mouse embryonic and MRC-5 human fibroblast cells by means of the MTT ([3-(4,5-dimethylthiazol-2-yl)-2,5-diphenyltetrazolium bromide]) assay. Briefly, a limited number of human cancer cells (5000/well for the SiHa and HeLa cells, 10,000/well in the case of C33A cells) were seeded onto a 96-well microplate and became attached to the bottom of the well overnight. On the second day of the procedure, the test substances were added in two concentrations (10.0, 30.0 µM) in order to obtain preliminary data and then the compounds were applied in serial dilutions (the final concentrations were 0.1, 0.3, 1.0, 3.0, 10.0 and 30.0 µM). After an incubation period of 72 h, the living cells were assayed by the addition of 20 µL of 5 mg/mL MTT solution. After a 4 h incubation, the medium was removed, and the precipitated formazan was dissolved in 100 µL/well of DMSO during a 60 min period of shaking. Finally, the reduced MTT was assayed at 545 nm, using a microplate reader. Untreated cells were taken as the negative control, and cisplatin (Ebewe Pharma GmbH, Unterach, Austria) was used as a reference active compound. All the cell lines were purchased from the European Collection of Cell Cultures (Salisbury, UK). Stock solutions (10 mM) of the tested compounds were prepared with DMSO. The highest DMSO concentration (0.3%) of the medium did not have any substantial effect on the cell proliferation. All in vitro experiments were carried out on two 96-well dishes with at least five parallel wells [24,85].

### 3.7. Cell Cycle Analysis by Flow Cytometry

Cellular DNA content was determined by means of flow cytometric analysis, using a DNA-specific fluorescent dye, propidium iodide (PI). The SiHa cells were seeded in 6-well plates and cultured overnight. The cultured cells were treated with various concentrations (1.5 or 3.0 µM) of the tested compound for 24 h or 72 h. The medium was then removed, and the cells were washed with phosphate-buffered saline (PBS) and trypsinized. The harvested cells were suspended in medium and centrifuged at 1500 rpm for 15 min at 4 °C. The supernatant was then removed and the cells were resuspended in 1 mL of PBS. After the second centrifugation, 1 mL of −20 °C 70% EtOH was added dropwise to the cell pellet. The cells were stored at −20 °C until DNA staining. On the day of measurement, the samples were washed with PBS and suspended in 1 mL of DNA staining buffer containing PI, ribonuclease-A, Triton-X and sodium citrate. After incubation for 1 h at room temperature, protected from light, the samples were analyzed with a Partec CyFlow instrument (Partec GmbH, Münster, Germany). For each experiment, 20,000 events were counted, and the percentages of the cells in the different cell-cycle phases (subG1, G1, S and G2/M) were determined by means of ModFit LT software 3.3.11 (Verity Software House, Topsham, ME, USA) [86,87,88].

### 3.8. Wound Healing Assay

In order to assess the antimetastatic activity of the tested compound, a wound healing assay was performed. The assay was performed with specific wound healing assay chambers (Ibidi GmbH, Martinsried, Germany). SiHa cells were collected and 35,000 cells were seeded into both chambers of the insert. The cells were left to attach to the plate surface during an overnight incubation at 37 °C in 5% CO_2_ atmosphere and then the inserts were removed. Cell debris was removed by a washing step with PBS. Test compounds were added to the wells in increasing concentrations in 2% FBS containing medium for 24 and 48 h. Migration of the cells into the wound site was visualized by a phase-contrast inverted microscope (Axiovert 40, Zeiss, Thornwood, NY, USA). The images were taken with CCD camera at defined intervals and the migration of the cells was calculated as the ratio of wound closure using ImageJ software 1.53a [89].

### 3.9. Statistical Analysis

Statistical analysis of the obtained data was performed by analysis of variance (ANOVA) followed by Dunnett’s test. All analyses were performed with GraphPad Prism 5 (GraphPad Software; San Diego, CA, USA).

## 4. Conclusions

A new flexible, biologically active germacranolide (**1**, lobatolide H) was isolated from the aerial parts of *Neurolaena lobata*. In order to elucidate the relative configuration of the two problematic chirality centers, a large number of known NMR shift parameters with the corresponding theoretical level combinations were tested, and novel parameters were also developed. Although several methods were found among the existing ones which performed well for the rigid test molecules, also verifying the relative configuration of **3** at C-8 that was recently found to be problematic with standard DFT-NMR methods [24], the flexibility of **1** required the development of novel combinations with newer DFT functionals and solvent models, also in the geometry optimization step. The novel ^13^C parameters combined with the coupling constant and TDDFT-ECD calculations allowed elucidation of the relative and absolute configuration of **1**. This example is a warning that similar to ECD [74,90], one should be careful with the DFT optimization level applied for NMR shift calculations of flexible compounds (Tables S22–S29). A novel type functional and solvent model or an independent verification of the results is recommended, if possible. Concerning the anticancer properties of **1**, the antiproliferative action determined on cervical cancer cells is comparable to that of the reference agent cisplatin with less pronounced action against non-cancerous cells. The modest treatment-related change in the cell-cycle distribution of the exposed cells provides limited information on the mechanism of the action, but the increase in the hypodiploid (sub G1) population indicates induction of apoptosis. Moreover, the compound inhibits the migration of SiHa cells in a concentration-dependent way, which may be the base of its antimetastatic action.

## Figures and Tables

**Table 1:** ^1^H and ^13^C NMR data of compound **1** in CDCl_3_ (*δ* in ppm, mult. *J* in Hz).

Position	^1^H	^13^C
1a	2.72 dd (11.0, 5.9)	48.6
1b	2.07 m	
2	4.75 td (9.8, 5.9)	69.2
3	5.25 d (9.8)	133.9
4	-	135.1
5a	2.76 dd (14.3, 5.1)	44.0
5b	2.32 dd (14.3, 1.6)	
6	5.76 brd (5.1)	70.8
7	2.93 d (7.9)	52.9
8	5.50 t (9.9, 7.9)	75.3
9	4.98 brd (9.9)	129.3
10	-	142.7
11	-	136.4
12	-	169.4
13a	6.30 d (3.2)	121.2
13b	5.60 d (3.2)	
14	1.79 s	18.7
15	1.53 s	20.0
8-iVal		
1′	-	171.8
2′	2.15–2.17 m (2H)	43.3
3′	2.05 m	25.4
4′	0.92 d (6.2)	22.3
5′	0.90 d (6.3)	22.3

**Table 2:** MAE and DP4+ results calculated from the ^13^C isotropic shielding constants of the four possible diastereomers of **1** vs. the experimental data of the naturally occurring **1** determined by three usually well performing combinations of theoretical levels.

NMR Level//DFT Optimization Level	MAE (Isomer 1; Isomer 2; Isomer 3, Isomer 4)	sDP4+ (Isomer 1; Isomer 2; Isomer 3, Isomer 4)
mPW1PW91/6-311+G(2d,p)//B3LYP/6-31+G(d,p)	2.32; 2.10; 2.57; 2.49	5.28%; 93.00%; 0.25%; 1.47%
mPW1PW91/6-311+G(2d,p) SMD/CHCl_3_//B3LYP/6-31+G(d,p)	2.06; 2.07; 2.16; 2.34	55.91%; 30.41%; 12.46%; 1.22%
mPW1PW91/6-311+G(2d,p) SMD/CHCl_3_//mPW1PW91/6-311+G(2d,p) SMD/CHCl_3_	2.15; 1.89; 2.38; 2.41	3.40%; 96.26%; 0.19%; 0.16%

**Table 3:** MAE and DP4+ results calculated from the ^13^C isotropic shielding constants of the 8-epimers of **3** vs. the experimental data of naturally occurring **3** determined by the four best performing combinations of theoretical levels.

NMR Level//DFT Optimization Level	MAE [(8*R*)-Epimer; (8*S*)-Epimer]	sDP4+ [(8*R*)-Epimer; (8*S*)-Epimer]
mPW1PW91/6-31G(d)//M06-2X/6-31G(d)	1.63; 1.68	(69.35%; 30.65%)
mPW1PW91/6-31G(d) SMD/CHCl_3_//M06-2X/6-31G(d)	1.40; 1.54	(93.58%; 6.42%)
M06/6-31G(d)//B3LYP/6-31+G(d,p)	1.68; 2.01	(99.13%; 0.87%)
OPBE0/6-31G(d)//B3LYP/6-31+G(d,p)	1.93; 2.35	(99.79%; 0.21%)

**Table 4:** MAE and DP4+ results calculated from the ^13^C isotropic shielding constants of the four possible diastereomers of **1** vs. the experimental data of naturally occurring **1** determined by the two best performing combinations of theoretical levels.

NMR Level//DFT Optimization Level	MAE (Isomer 1; Isomer 2; Isomer 3, Isomer 4)	sDP4+ (Isomer 1; Isomer 2; Isomer 3, Isomer 4)
mPW1PW91/6-31G(d) SMD/CHCl_3_//M06-2X/6-31G(d)	2.42; 2.59; 2.62; 2.85	(90.66% 3.14% 5.16% 1.04%)
M06/6-31G(d)//B3LYP/6-31+G(d,p)	3.12; 2.83; 3.19; 2.76	(2.54%; 69.71%; 9.58%; 18.17%)

**Table 5:** Test of the mPW1PW91/6-311+G(2d,p)//ωB97XD/6-31+G(d,p) level ^13^C parameters for **2**.

Numbering	δ_Exp_ (ppm)	δ_Calc_ (ppm)	Δδ (ppm)
C-1	79.28	78.78	0.50
C-2	32.17	31.26	0.91
C-3	35.34	36.89	1.55
C-4	146.48	152.97	6.49
C-5	56.15	56.06	0.09
C-6	67.25	64.33	2.92
C-7	49.59	48.55	1.04
C-8	18.43	19.65	1.22
C-9	36.55	35.90	0.65
C-10	41.93	44.33	2.40
C-11	26.26	27.81	1.55
C-12	21.32	19.60	1.72
C-13	16.44	15.72	0.72
C-14	11.82	10.57	1.25
C-15	108.03	105.99	2.04
MAE	N/A	N/A	1.67

**Table 6:** Test of the mPW1PW91/6-311+G(2d,p) SMD/CHCl_3_//ωB97XD/6-31+G(d,p) SMD/CHCl_3_ level ^13^C parameters for **2**.

Numbering	δ_Exp_ (ppm)	δ_Calc_ (ppm)	Δδ (ppm)
C-1	79.28	77.99	1.29
C-2	32.17	31.72	0.45
C-3	35.34	36.68	1.34
C-4	146.48	152.90	6.42
C-5	56.15	55.65	0.50
C-6	67.25	64.65	2.60
C-7	49.59	48.49	1.10
C-8	18.43	19.48	1.05
C-9	36.55	35.92	0.63
C-10	41.93	44.70	2.77
C-11	26.26	28.01	1.75
C-12	21.32	19.80	1.52
C-13	16.44	15.27	1.17
C-14	11.82	11.13	0.69
C-15	108.03	104.29	3.74
MAE	N/A	N/A	1.80

**Table 7:** MAE and DP4+ results calculated from the ^13^C isotropic shielding constants of the 8-epimers of **3** vs. the experimental data of naturally occurring **3** determined by the two novel combinations.

NMR Level//DFT Optimization Level	MAE [(8*R*)-Epimer; (8*S*)-Epimer]	sDP4+ [(8*R*)-Epimer; (8*S*)-Epimer]
mPW1PW91/6-311+G(2d,p)//ωB97XD/6-31+G(d,p)	1.82; 1.87	(66.38%; 33.62%)
mPW1PW91/6-311+G(2d,p) SMD/CHCl_3_//ωB97XD/6-31+G(d,p) SMD/CHCl_3_	1.60; 1.91	(99.26%; 0.74%)

**Table 8:** MAE results calculated from the ^13^C isotropic shielding constants of the four possible diastereomers of **1** vs. the experimental data of naturally occurring **1** determined by the two novel combinations.

NMR Level//DFT Optimization Level	MAE (Isomer 1; Isomer 2; Isomer 3, Isomer 4)
mPW1PW91/6-311+G(2d,p)//ωB97XD/6-31+G(d,p)	2.33; 2.48; 2.58; 3.11
mPW1PW91/6-311+G(2d,p) SMD/CHCl_3_//ωB97XD/6-31+G(d,p) SMD/CHCl_3_	2.26; 2.42; 2.48; 3.23

**Table 9:** Comparison of the experimental and computed coupling constant values of **isomer 1** and **isomer 2** of **1** vs. the experimental data of naturally occurring **1** determined by the mPW1PW91/6-311+G(2d,p) SMD/CHCl_3_//ωB97XD/6-31+G(d,p) SMD/CHCl_3_ level. *J*_in_ indicates the coupling constant data of **isomer n**.

Coupling Atoms	*J*_Exp_ (Hz)	*J*_i1_ (Hz)	*J*_i2_ (Hz)	Δ*J*_i1_	Δ*J*_i2_
1aH-2H	5.90	6.62	0.93	0.72	4.97
2H-3H	9.80	10.08	6.51	0.28	3.29
5aH-6H	5.10	6.58	2.82	1.48	2.28
7H-8H	7.90	4.33	3.28	3.57	4.62
8H-9H	9.90	7.36	8.73	2.54	1.17
MAE				1.72	3.27

**Table 10:** Antiproliferative activity (IC50 values) of compound 1. The selectivity indices (SI) were calculated as the ratio of the IC50 value in the non-tumor cells and the IC50 in the cancer cell lines. The compound’s activity towards cancer cells is considered as strongly selective if the selectivity index (SI) value is higher than 6, moderately selective if 3 < SI < 6, slightly selective if 1 < SI < 3, and non-selective if SI is lower than 1.

Compound	IC_50_ Values (µM) [95% CI]				
	HeLa	C33A	SiHa	NIH-3T3	MRC-5
**1**	16.62 [14.55–18.99]	4.43 [3.62–5.43]	2.82 [2.40–3.32]	13.05 [11.44–14.90]	14.82 [13.90–15.80]
cisplatin	12.14 [10.18–14.46]	5.85 [5.37–6.38]	4.29 [3.72–4.95]	5.49 [4.76–6.35]	5.17 [3.88–6.89]
SI for **1** NIH-3T3/cell line	0.79	2.95	4.63		
SI for **1** MRC-5/cell line	0.89	3.35	5.26		

## Data Availability

Not applicable.

## Supplementary Materials

The following supporting information can be downloaded at: https://www.mdpi.com/article/10.3390/ijms24065841/s1. References [23,24,27,43,44,54,55,56,57,59,64,65,66,67,68,69,70,71] are cited in the supplementary materials.

## References

[1] Babaei G., Aliarab A., Abroon S., Rasmi Y., Aziz S.G.G. (2018). Application of sesquiterpene lactone: A new promising way for cancer therapy based on anticancer activity. Biomed. Pharmacother..

[2] Paço A., Brás T., Santos J.O., Sampaio P., Gomes A.C., Duarte M.F. (2022). Anti-Inflammatory and Immunoregulatory Action of Sesquiterpene Lactones. Molecules.

[3] Shulha O., Zidorn C. (2019). Sesquiterpene lactones and their precursors as chemosystematic markers in the tribe Cichorieae of the Asteraceae revisited: An update (2008–2017). Phytochemistry.

[4] Zorrilla J.G., Cala A., Rial C., Mejias F.J.R., Molinillo J.M.G., Varela R.M., Macias F.A. (2020). Synthesis of Active Strigolactone Analogues Based on Eudesmane- and Guaiane-Type Sesquiterpene Lactones. J. Agric. Food Chem..

[5] Laurella L.C., Mirakian N.T., Garcia M.N., Grasso D.H., Sülsen V.P., Papademetrio D.L. (2022). Sesquiterpene Lactones as Promising Candidates for Cancer Therapy: Focus on Pancreatic Cancer. Molecules.

[6] Moujir L., Callies O., Sousa P.M.C., Sharopov F., Seca A.M.L. (2020). Applications of Sesquiterpene Lactones: A Review of Some Potential Success Cases. Appl. Sci..

[7] Liu J., Yang Z., Kong Y., He Y., Xu Y., Cao X. (2019). Antitumor activity of alantolactone in lung cancer cell lines NCI-H1299 and Anip973. J. Food Biochem..

[8] Zhu N.L., Tang C., Xu C., Ke C.Q., Lin G., Jenis J., Yao S., Liu H., Ye Y. (2019). Cytotoxic germacrane-type sesquiterpene lactones from the whole plant of *Carpesium lipskyi*. J. Nat. Prod..

[9] Wang J., Su S., Zhang S., Zhai S., Sheng R., Wu W., Guo R. (2019). Structure-activity relationship and synthetic methodologies of *α*-santonin derivatives with diverse bioactivities: A mini-review. Eur. J. Med. Chem..

[10] Matos M.S., Anastácio J.D., dos Santos C.N. (2021). Sesquiterpene Lactones: Promising Natural Compounds to Fight Inflammation. Pharmaceutics.

[11] Yuan J., Wen X., Ke C.Q., Zhang T., Lin L., Yao S., Goodpaster J.D., Tang C., Ye Y. (2020). Tricarabrols A-C, three anti-inflammatory sesquiterpene lactone trimers featuring a methylene-tethered linkage from *Carpesium faberi*. Org. Chem. Front..

[12] Li Q., Wang Z., Xie Y., Hu H. (2020). Antitumor activity and mechanism of costunolide and dehydrocostus lactone: Two natural sesquiterpene lactones from the Asteraceae family. Biomed. Pharmacother..

[13] Bailly C. (2021). Anticancer targets and signaling pathways activated by britannin and related pseudoguaianolide sesquiterpene lactones. Biomedicines.

[14] Liu X.N., Li H.M., Wang S.P., Zhang J.Z., Liu D.L. (2021). Sesquiterpene lactones of *Aucklandia lappa*: Pharmacology, pharmacokinetics, toxicity, and structure-activity relationship. Chin. Herb. Med..

[15] Ma C., Meng C.W., Zhou Q.M., Peng C., Liu F., Zhang J.W., Zhou F., Xiong L. (2019). New sesquiterpenoids from the stems of *Dendrobium nobile* and their neuroprotective activities. Fitoterapia.

[16] Tang J.J., Huang L.F., Deng J.L., Wang Y.M., Guo C., Peng X.N., Liu Z., Gao J.M. (2022). Cognitive enhancement and neuroprotective effects of 1,6-*O*,*O*-diacetylbritannilactone, a sesquiterpene lactone in 5xFAD Alzheimer’s disease mice model. Redox Biol..

[17] Abdelwahab S.I., Taha M.M.E., Alhazmi H.A., Ahsan W., Rehman Z.U., Bratty M.A., Makeen H. (2019). Phytochemical profiling of costus (*Saussurea lappa* Clarke) root essential oil, and its antimicrobial and toxicological effects. Trop. J. Pharm. Res..

[18] Sülsen V.P., Martino V.S., Sülsen V.P., Martino V.S. (2018). Overview. Sesquiterpene Lactones: Advances in Their Chemistry and Biological Aspects.

[19] Sosa A., Diaz M., Salvatore A., Bardon A., Borkosky S., Vera N. (2019). Insecticidal effects of *Vernonanthura nebularum* against two economically important pest insects. Saudi J. Biol. Sci..

[20] Lajter I., Vasas A., Beni Z., Forgo P., Binder M., Bochkov V., Zupko I., Krupitza G., Frisch R., Kopp B. (2014). Sesquiterpenes from Neurolaena lobata and their antiproliferative and anti-inflammatory activities. J. Nat. Prod..

[21] Passreiter C.M., Wendisch D., Gondol D. (1995). Sesquiterpene lactones from *Neurolaena lobata*. Phytochemistry.

[22] Borges-del-Castillo J., Manresa-Ferrero M.T., Rodríguez-Luis F., Vázquez-Bueno P. (1982). Panama Flora. II. New Sesquiterpene Lactones from *Neurolaena lobata*. J. Nat. Prod..

[23] Manchand P.S., Blount J.F. (1978). Stereostructures of Neurolenins A and B, Novel Germacranolide Sesquiterpenes from *Neurolaena lobata* (L.) R.Br. J. Org. Chem..

[24] Vasas A., Lajter I., Kúsz N., Király S.B., Kovács T., Kurtán T., Bózsity N., Nagy N., Schelz Z., Zupkó I. (2021). Isolation, Structure Determination of Sesquiterpenes from *Neurolaena lobata* and Their Antiproliferative, Cell Cycle Arrest-Inducing and Anti-Invasive Properties against Human Cervical Tumor Cells. Pharmaceutics.

[25] Smith S.G., Goodman J.M. (2010). Assigning Stereochemistry to Single Diastereoisomers by GIAO NMR Calculation: The DP4 Probability. J. Am. Chem. Soc..

[26] Grimblat N., Zanardi M.M., Sarotti A.M. (2015). Beyond DP4: An Improved Probability for the Stereochemical Assignment of Isomeric Compounds using Quantum Chemical Calculations of NMR Shifts. J. Org. Chem..

[27] Lodewyk M.W., Siebert M.R., Tantillo D.J. (2012). Computational Prediction of ^1^H and ^13^C Chemical Shifts: A Useful Tool for Natural Product, Mechanistic, and Synthetic Organic Chemistry. Chem. Rev..

[28] Li W.S., Yan R.J., Yu Y., Shi Z., Mándi A., Shen L., Kurtán T., Wu J. (2020). Determination of the Absolute Configuration of Super-Carbon-Chain Compounds by a Combined Chemical, Spectroscopic, and Computational Approach: Gibbosols A and B. Angew. Chem. Int. Ed..

[29] Jiang Z.P., Sun S.H., Yu Y., Mándi A., Luo J.Y., Yang M.H., Kurtán T., Chen W.H., Shen L., Wu J. (2021). Discovery of benthol A and its challenging stereochemical assignment: Opening up a new window for skeletal diversity of super-carbonchain compounds. Chem. Sci..

[30] Kawka A., Hajdaś G., Kułaga D., Koenig H., Kowalczyk I., Pospieszny T. (2023). Molecular structure, spectral and theoretical study of new type bile acid–sterol conjugates linked via 1,2,3-triazole ring. J. Mol. Struct..

[31] Lodewyk M.W., Soldi C., Jones P.B., Olmstead M.M., Rita J., Shaw J.T., Tantillo D.J. (2012). The Correct Structure of Aquatolide-Experimental Validation of a Theoretically-Predicted Structural Revision. J. Am. Chem. Soc..

[32] Liu Y., Holt T.A., Kutateladze A., Newhouse T.R. (2020). Stereochemical revision of xylogranatin F by GIAO and DU8+ NMR calculations. Chirality.

[33] Sarotti A.M. (2020). In Silico Reassignment of (+)-Diplopyrone by NMR Calculations: Use of a DP4/J-DP4/DP4+/DIP Tandem to Revise Both Relative and Absolute Configuration. J. Org. Chem..

[34] Zhou T., Zheng A., Zhang W., Lu X., Chen H., Tan H. (2022). Concise total syntheses of two flavans and structure revision assisted by quantum NMR calculations. Org. Biomol. Chem..

[35] Marcarino M.O., Cicetti S., Zanardi M.M., Sarotti A.M. (2022). A critical review on the use of DP4+ in the structural elucidation of natural products: The good, the bad and the ugly. A practical guide. Nat. Prod. Rep..

[36] Xiong J., Zhou P.J., Jiang H.W., Huang T., He Y.H., Zhao Z.Y., Zang Y., Choo Y.M., Wang X., Chittiboyina A.G. (2021). Forrestiacids A and B, Pentaterpene Inhibitors of ACL and Lipogenesis: Extending the Limits of Computational NMR Methods in the Structure Assignment of Complex Natural Products. Angew. Chem. Int. Ed..

[37] Dybiec K., Gryff-Keller A. (2009). Remarks on GIAO-DFT predictions of ^13^C chemical shifts. Magn. Reson. Chem..

[38] Autschbach J., Zheng S. (2009). Relativistic Computations of NMR Parameters from First Principles: Theory and Applications. Annu. Rep. NMR Spectrosc..

[39] Forster L.C., Pierens G.K., Garson M.J. (2019). Elucidation of Relative and Absolute Configurations of Highly Rearranged Diterpenoids and Evidence for a Putative Biosynthetic Intermediate from the Australian Nudibranch *Goniobranchus geometricus*. J. Nat. Prod..

[40] Kutateladze A.G., Reddy D.S. (2017). High-Throughput in Silico Structure Validation and Revision of Halogenated Natural Products Is Enabled by Parametric Corrections to DFT-Computed ^13^C NMR Chemical Shifts and Spin−Spin Coupling Constants. J. Org. Chem..

[41] Dračínský M., Buděšínský M., Warżajtis B., Rychlewska U. (2012). Solution and Solid-State Effects on NMR Chemical Shifts in Sesquiterpene Lactones: NMR, X-ray, and Theoretical Methods. J. Phys. Chem. A.

[42] Li W.S., Mándi A., Liu J.J., Shen L., Kurtán T., Wu J. (2019). Xylomolones A−D from the Thai Mangrove *Xylocarpus moluccensis*: Assignment of Absolute Stereostructures and Unveiling a Convergent Strategy for Limonoid Biosynthesis. J. Org. Chem..

[43] Pierens G.K. (2014). ^1^H and ^13^C NMR Scaling Factors for the Calculation of Chemical Shifts in Commonly Used Solvents Using Density Functional Theory. J. Comput. Chem..

[44] CHESHIRE CCAT, The Chemical Shift Repository for Computed NMR Scaling Factors. https://cheshirenmr.info/index.htm.

[45] Bohle F., Grimme S. (2022). Hydrocarbon Macrocycle Conformer Ensembles and ^13^C-NMR Spectra. Angew. Chem. Int. Ed..

[46] Begnini F., Poongavanam V., Atilaw Y., Erdelyi M., Schiesser S., Kihlberg J. (2021). Cell Permeability of Isomeric Macrocycles: Predictions and NMR Studies. ACS Med. Chem. Lett..

[47] Chai J.D., Head-Gordon M. (2008). Long-range corrected hybrid density functionals with damped atom–atom dispersion corrections. Phys. Chem. Chem. Phys..

[48] Brémond É., Savarese M., Su N.Q., Pérez-Jiménez Á.J., Xu X., Sancho-García J.C., Adamo C. (2016). Benchmarking Density Functionals on Structural Parameters of Small-/Medium-Sized Organic Molecules. J. Chem. Theory Comput..

[49] Melek F.R., Gershenzon J., Lee E., Mabry T.J. (1984). Sesquiterpene lactones of *Helianthus gracilentus*. Phytochemistry.

[50] Liang L.F., Lan L.F., Taglialatela-Scafati O., Guo Y.W. (2013). Sartrolides A-G and bissartrolide, new cembranolides from the South China Sea soft coral *Sarcophyton trocheliophorum* Marenzeller. Tetrahedron.

[51] Liang L.F., Kurtán T., Mándi A., Yao L.G., Li J., Lan L.F., Guo Y.W. (2018). Structural, stereochemical, and bioactive studies of cembranoids from Chinese soft coral *Sarcophyton trocheliophorum*. Tetrahedron.

[52] Kicsák M., Mándi A., Varga S., Herczeg M., Batta G., Bényei A., Borbás A., Herczegh P. (2018). Tricyclanos: Conformationally constrained nucleoside analogues with a new heterotricycle obtained from a D-ribofuranose unit. Org. Biomol. Chem..

[53] Mándi A., Wu J., Kurtán T. (2020). TDDFT-ECD and DFT-NMR studies of thaigranatins A–E and granatumin L isolated from *Xylocarpus granatum*. RSC Adv..

[54] Qiu S., de Gussem E., Tehrani K.A., Sergeyev S., Bultinck P., Herrebout W. (2013). Stereochemistry of the Tadalafil Diastereoisomers: A Critical Assessment of Vibrational Circular Dichroism, Electronic Circular Dichroism, and Optical Rotatory Dispersion. J. Med. Chem..

[55] Rablen P.R., Pearlman S.A., Finkbiner J. (1999). A comparison of density functional methods for the estimation of proton chemical shifts with chemical accuracy. J. Phys. Chem. A.

[56] Jain R.J., Bally T., Rablen P.R. (2009). Calculating accurate proton chemical shifts of organic molecules with density functional methods and modest basis sets. J. Org. Chem..

[57] Konstantinov I.A., Broadbelt L.J. (2011). Regression Formulas for Density Functional Theory Calculated ^1^H and ^13^C NMR Chemical Shifts in Toluene-d8. J. Phys. Chem. A.

[58] Ditchfield R. (1974). Self-consistent perturbation theory of diamagnetism. Mol. Phys..

[59] Lee K.H., Min Y.D., Choi S.Z., Kwon H.C., Cho O.R., Lee K.C., Lee K.R. (2004). A new sesquiterpene lactone from *Artemisia rubripes* nakai. Arch. Pharmacal. Res..

[60] Jewell J.S., Szarek W.A. (1969). The light-induced addition of 1,3-dioxolan to unsaturated carbohydrates. Tetrahedron Lett..

[61] Hough L., Otte B. (1966). Furanoid Vinyl Ethers. Chem. Commun..

[62] Miyoshi T., Miyairi N., Aoki H., Kohsaka M., Sakai H.I., Imanaka H. (1972). Bicyclomycin, a new antibiotic I. Taxonomy, isolation and characterization. J. Antibiot..

[63] Domínguez E., Romo J. (1963). Mexicanin—I. A new sesquiterpene lactone related to tenulin. Tetrahedron.

[64] Li J., Tang H., Kurtán T., Mándi A., Zhuang C.L., Su L., Zheng G.L., Zhang W. (2018). Swinhoeisterols from the South China Sea Sponge *Theonella swinhoei*. J. Nat. Prod..

[65] Kobayashi S., Lu C., Hoye T.R., Hillmyer M.A. (2009). Controlled Polymerization of a Cyclic Diene Prepared from the Ring-Closing Metathesis of a Naturally Occurring Monoterpene. J. Am. Chem. Soc..

[66] Nishida T., Satoh K., Kamigaito M. (2020). Biobased polymers via radical homopolymerization and copolymerization of a series of terpenoid derived conjugated dienes with exo-methylene and 6-membered ring. Molecules.

[67] Raghavendra S., Tadiparthi K., Yadav J.S. (2017). Total syntheses of Prelactone V and Prelactone B. Carbohydr. Res..

[68] Doboszewski B., Herdewijn P. (2008). Carbohydrate chiral-pool approach to four enantiomerically pure 2-naphthylmethyl 3-hydroxy-2-methylbutanoates. Tetrahedron.

[69] Ivanova N.A., Valiullina Z.R., Shitikova O.V., Miftakhov M.S. (2007). Reaction of methyl-4-methylene-2,3-*O*-isopropylidene-β-D-ribofuranoside with *N*-bromosuccinimide in aqueous tetrahydrofurane. Russ. J. Org. Chem..

[70] Kohn H., Abuzar S., Korp J.D., Zektzer A.S., Martin G.E. (1988). Structural studies of bicyclomycin. J. Heterocyclic. Chem..

[71] Błoszyk E., Dudek A., Kosturkiewicz Z., Rychłewska U., Daniewski W.M., Gumulka M., Nawrot J., Buděšínský M., Vašíčková S., Holub M. (1989). Sesquiterpene lactones of *Cephalophora aromatica* (HOOK.) SCHRADER and their deterrent activity. The stereostructure of geigerinin. Collect. Czech. Chem. Commun..

[72] Grimblat N., Gavín J.A., Daranas A.H., Sarotti A.M. (2019). Combining the Power of J Coupling and DP4 Analysis on Stereochemical Assignments: The J-DP4 Methods. Org. Lett..

[73] Mándi A., Kurtán T. (2019). Applications of OR/ECD/VCD to the structure elucidation of natural products. Nat. Prod. Rep..

[74] Superchi S., Scafato P., Górecki M., Pescitelli G. (2018). Configuration Determination by Quantum Mechanical Calculation of Chiroptical Spectra: Basics and Applications to Fungal Metabolites. Curr. Med. Chem..

[75] De B.C., Zhang W., Yang C., Mándi A., Huang C., Zhang L., Liu W., Ruszczycky M.W., Zhu Y., Ma M. (2022). Flavin-enabled reductive and oxidative epoxide ring opening reactions. Nat. Commun..

[76] Yanai T., Tew D., Handy N. (2004). A new hybrid exchange–correlation functional using the Coulomb-attenuating method (CAM-B3LYP). Chem. Phys. Lett..

[77] Pecul M., Marchesan D., Ruud K., Coriani S. (2005). Polarizable continuum model study of solvent effects on electronic circular dichroism parameters. J. Chem. Phys..

[78] Szabó K.E., Kun S., Mándi A., Kurtán T., Somsák L. (2017). Glucopyranosylidene-Spiro-Thiazolinones: Synthetic Studies and Determination of Absolute Configuration by TDDFT-ECD Calculations. Molecules.

[79] MacroModel (2015). Schrödinger LLC. http://www.schrodinger.com/MacroModel.

[80] Frisch M.J., Trucks G.W., Schlegel H.B., Scuseria G.E., Robb M.A., Cheeseman J.R., Scalmani G., Barone V., Mennucci B., Petersson G.A. (2010). Gaussian 09, Revision B.01.

[81] Frisch M.J., Trucks G.W., Schlegel H.B., Scuseria G.E., Robb M.A., Cheeseman J.R., Scalmani G., Barone V., Mennucci B., Petersson G.A. (2010). Gaussian 09, Revision C.01.

[82] Frisch M.J., Trucks G.W., Schlegel H.B., Scuseria G.E., Robb M.A., Cheeseman J.R., Scalmani G., Barone V., Mennucci B., Petersson G.A. (2013). Gaussian 09, Revision E.01.

[83] Stephens P.J., Harada N. (2010). ECD cotton effect approximated by the Gaussian curve and other methods. Chirality.

[84] Varetto U. (2009). MOLEKEL, 5.4.

[85] Mosmann T. (1983). Rapid colorimetric assay for cellular growth and survival: Application to proliferation and cytotoxicity assays. J. Immunol. Methods.

[86] Vermes I., Haanen C., Reutelingsperger C. (2000). Flow cytometry of apoptotic cell death. J. Immunol. Methods.

[87] Latif D.A., Gonda T., Vágvölgyi M., Kúsz N., Kulmány Á., Ocsovszki I., Zomborszki P.Z., Zupkó I., Hunyadi A. (2019). Synthesis and In Vitro Antitumor Activity of Naringenin Oxime and Oxime Ether Derivatives. Int. J. Mol. Sci..

[88] Kuo C.Y., Schelz Z., Tóth B., Vasas A., Ocsovszki I., Chang F.R., Hohmann J., Zupkó I., Wang H.C. (2019). Investigation of natural phenanthrenes and the antiproliferative potential of juncusol in cervical cancer cell lines. Phytomedicine.

[89] Behrens J., Kameritsch P., Wallner S., Pohl U., Pogoda K. (2010). The carboxyl tail of Cx43 augments p38 mediated cell migration in a gap junction-independent manner. Eur. J. Cell Biol..

[90] Sun P., Xu D.X., Mándi A., Kurtán T., Li T.J., Schulz B., Zhang W. (2013). Structure, Absolute Configuration, and Conformational Study of 12-Membered Macrolides from the Fungus *Dendrodochium* sp. Associated with the Sea Cucumber *Holothuria nobilis* Selenka. J. Org. Chem..

