# Supplementary material for: Isolation and NMR Scaling Factors for the Structure Determination of Lobatolide H, a Flexible Sesquiterpene from Neurolaena lobata
Source: Int J Mol Sci. 2023 Mar 19;24(6):5841. doi: 10.3390/ijms24065841 (PMC10052924; doi:10.3390/ijms24065841)
Supplement: Supplementary file 1 [file ijms-24-05841-s001.zip › ijms-2208496-supplementary.pdf]

Supporting Information

Isolation and NMR Scaling Factors for the Structure Determination of Lobatolide H, a Flexible Sesquiterpene from *Neurolaena lobata*

Tibor Kovács,[a,b] Ildikó Lajter,[c] Norbert Kúsz,[c] Zsuzsanna Schelz,[d] Noémi Bózsity-Faragó,[d] Anikó Borbás,[e] István Zupkó,[d] Georg Krupitza,[f] Richard Frisch,[g] Judit Hohmann,[c,h] Andrea Vasas,[c,h,]* Attila Mándi,[a,]*

[a] Department of Organic Chemistry, University of Debrecen, P.O.B. 400, 4002 Debrecen, Hungary
[b] Doctoral School of Chemistry, University of Debrecen, Egyetem tér 1, 4032 Debrecen, Hungary
[c] Institute of Pharmacognosy, University of Szeged, 6720 Szeged, Hungary
[d] Institute of Pharmacodynamics and Biopharmacy, University of Szeged, Eötvös u. 6, H-6720 Szeged, Hungary
[e] Department of Pharmaceutical Chemistry, University of Debrecen, Egyetem tér 1, H-4032 Debrecen, Hungary
[f] Clinical Institute of Pathology, Medical University of Vienna, Waehringer Guertel 18-20, A-1090 Vienna, Austria
[g] Institute for Ethnobiology, Playa Diana, GT-170 San José/Petén, Guatemala
[h] ELKH-USZ Biologically Active Natural Products Research Group, University of Szeged, Eötvös u. 6, H-6720 Szeged, Hungary

Table of Contents

NMR spectra of **1** S2
Low-energy conformers of the computed diastereomers of **1**-**3** S6
Tested NMR combinations with available scaling factors found in the literature S11
MAE and $\Delta\delta_{max}$ values obtained for **1**-**3** with the literature combinations S13
MAE and $\Delta\delta_{max}$ values obtained for **1** and **3** with the currently developed combinations S24
Level dependency of Boltzmann populations and conformer dependency of chemical shift data S34
Cartesian coordinates and energies of the low-energy conformers calculated at various levels for **1**-**3** S42
References S409

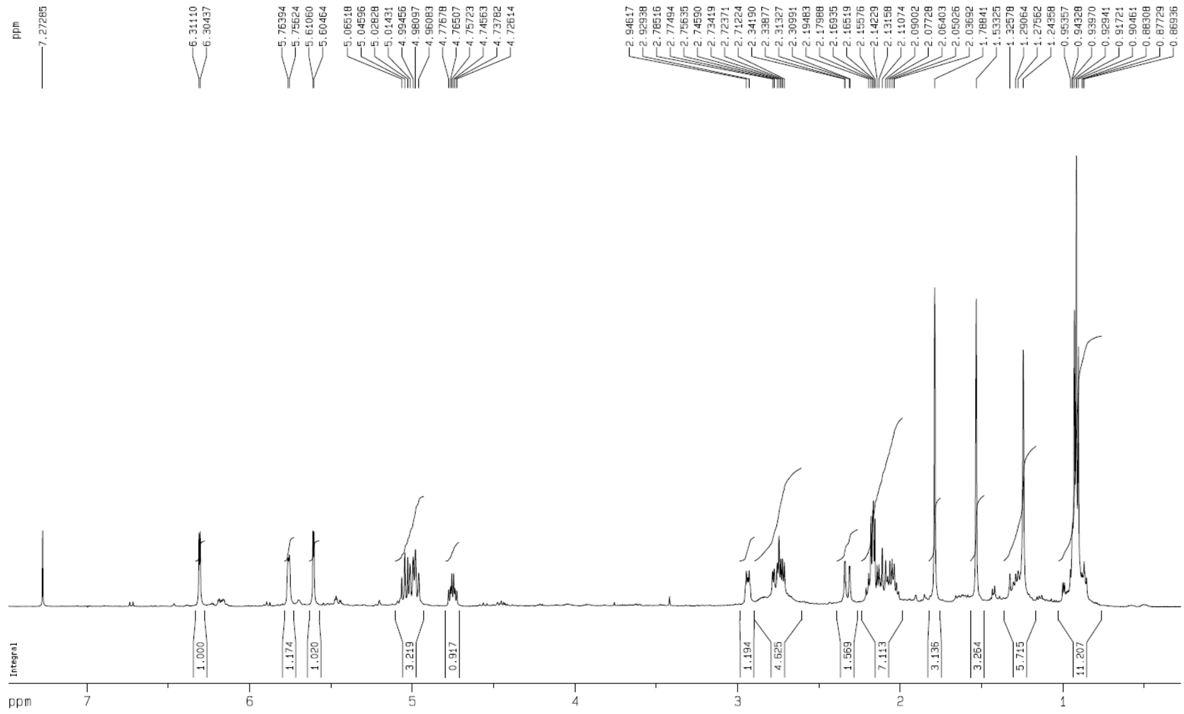

Figure S1. ^{1}H NMR spectrum of **1**

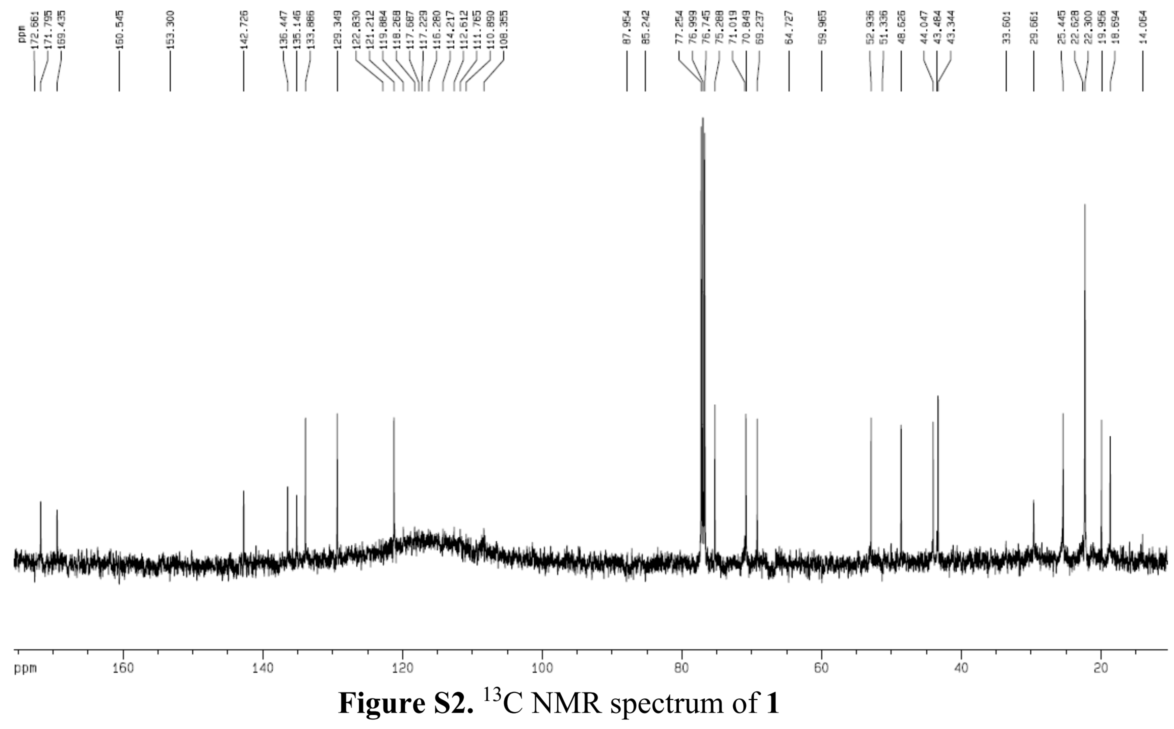

Figure S2. ^{13}C NMR spectrum of **1**

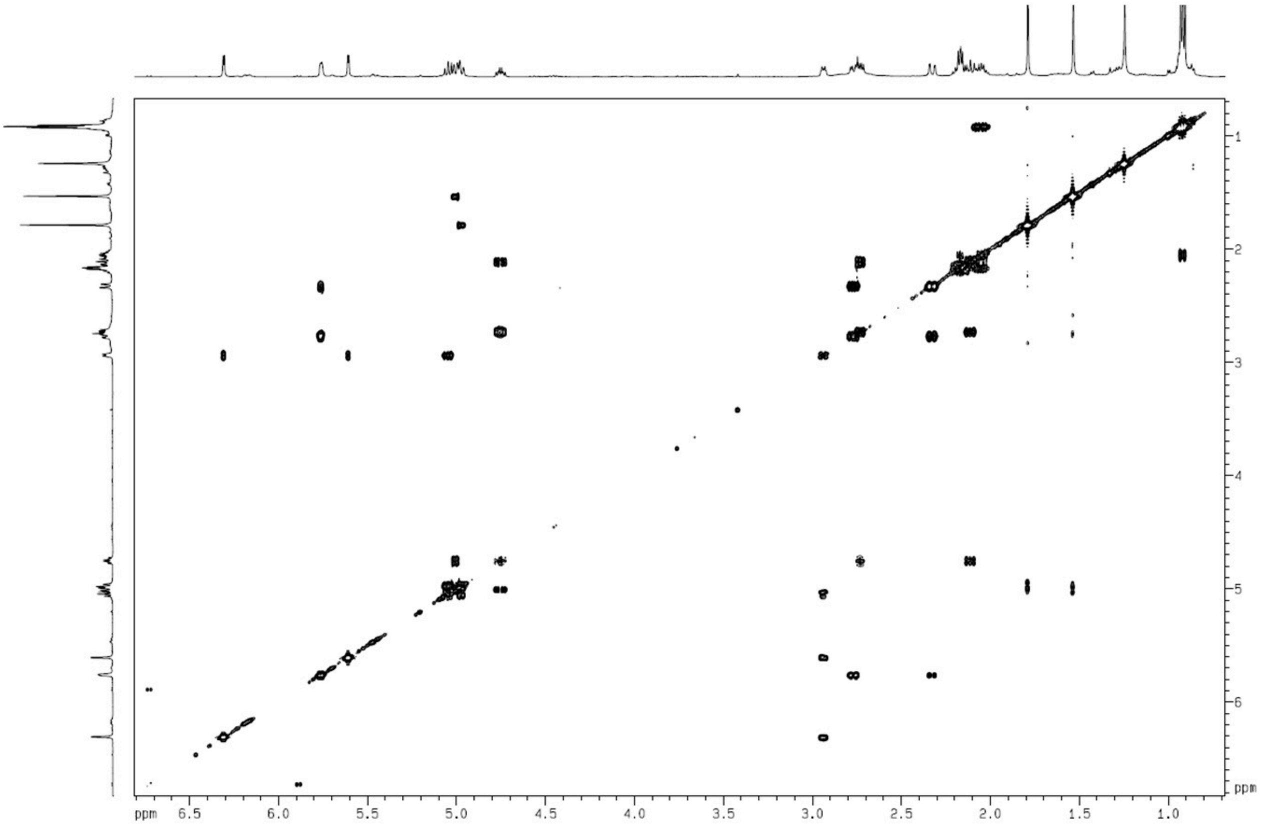

Figure S3. ^{1}H-^{1}H COSY spectrum of **1**

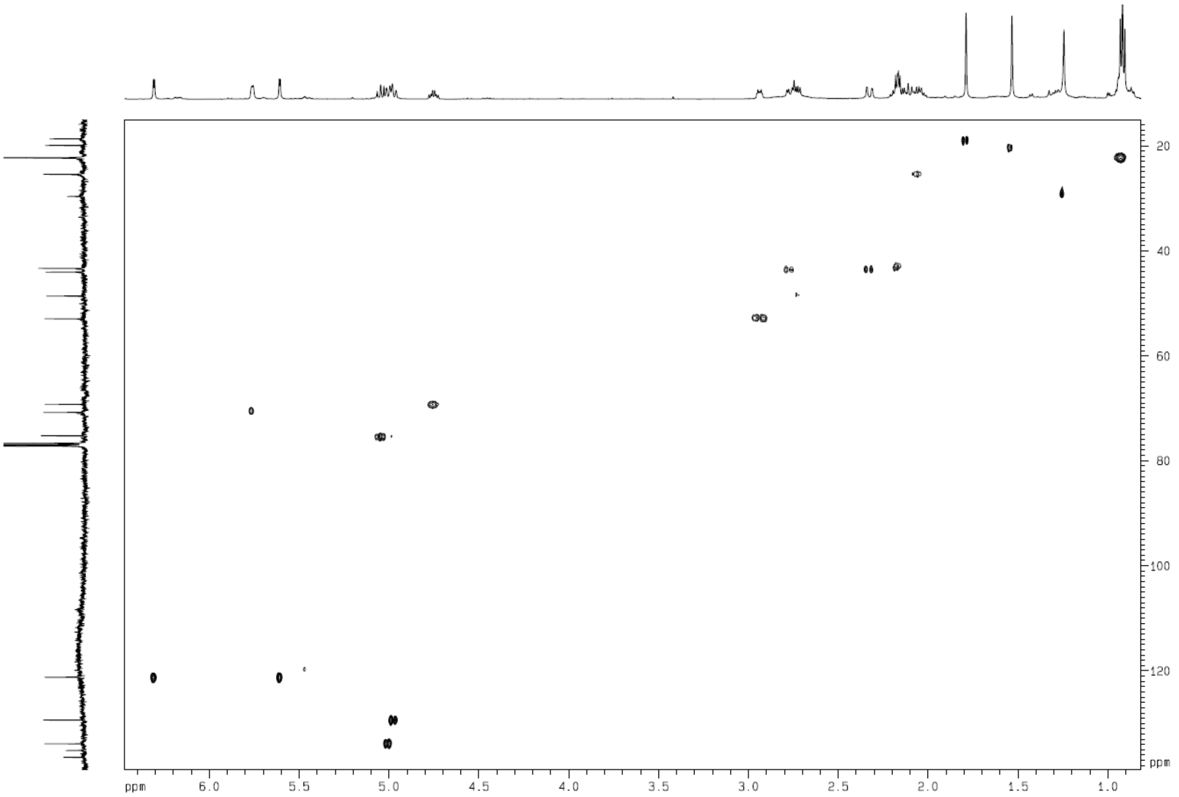

Figure S4. HSQC spectrum of **1**

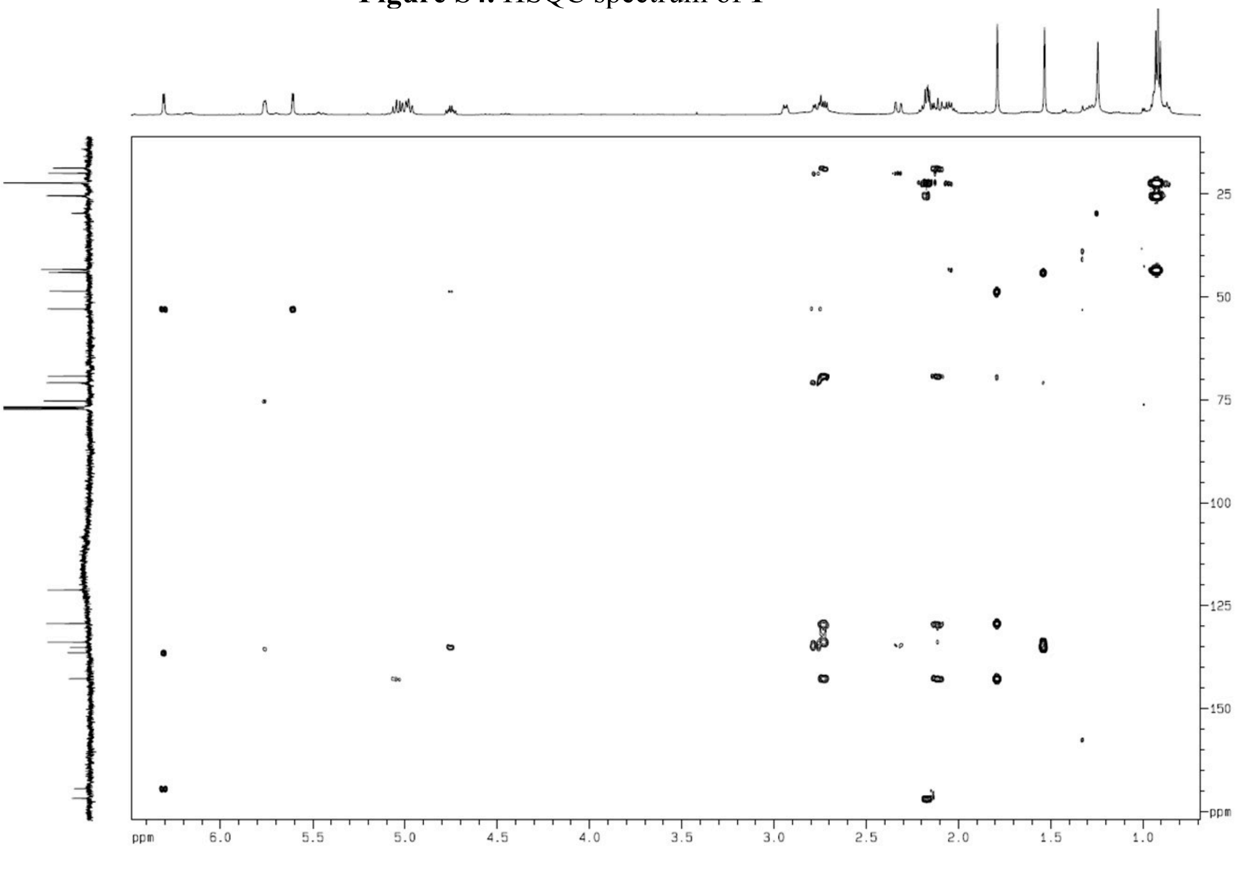

Figure S5. HMBC spectrum of **1**

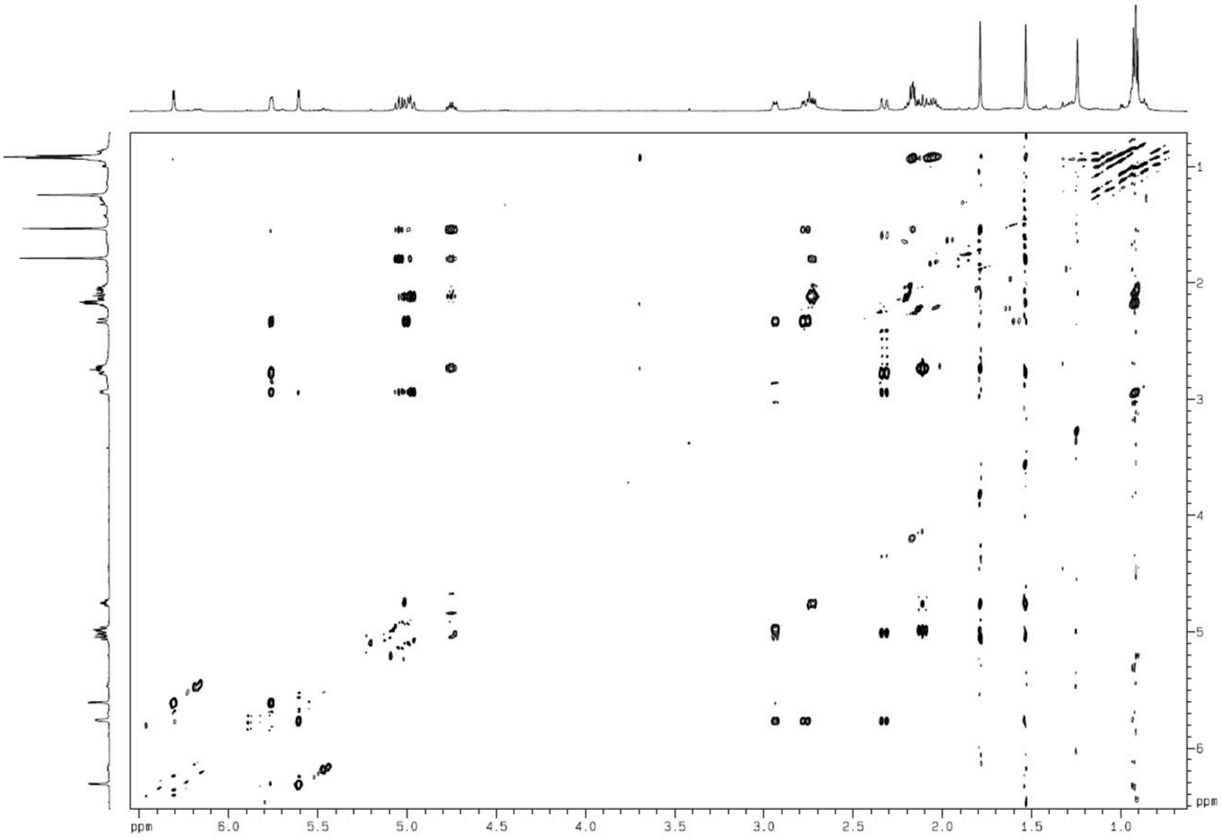

Figure S6. NOESY spectrum of **1**

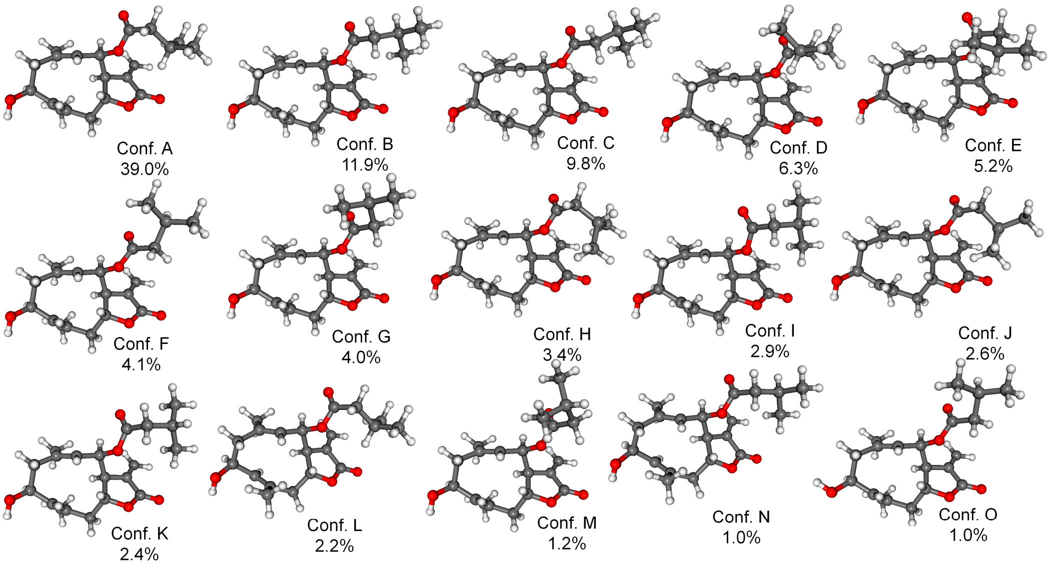

Figure S7. Structure and population of the low-energy (≥ 1%) ωB97XD/6-31+G(d,p) PCM/$CHCl_3$ conformers of (2*S*,6*R*,7*S*,8*R*)-**1**.

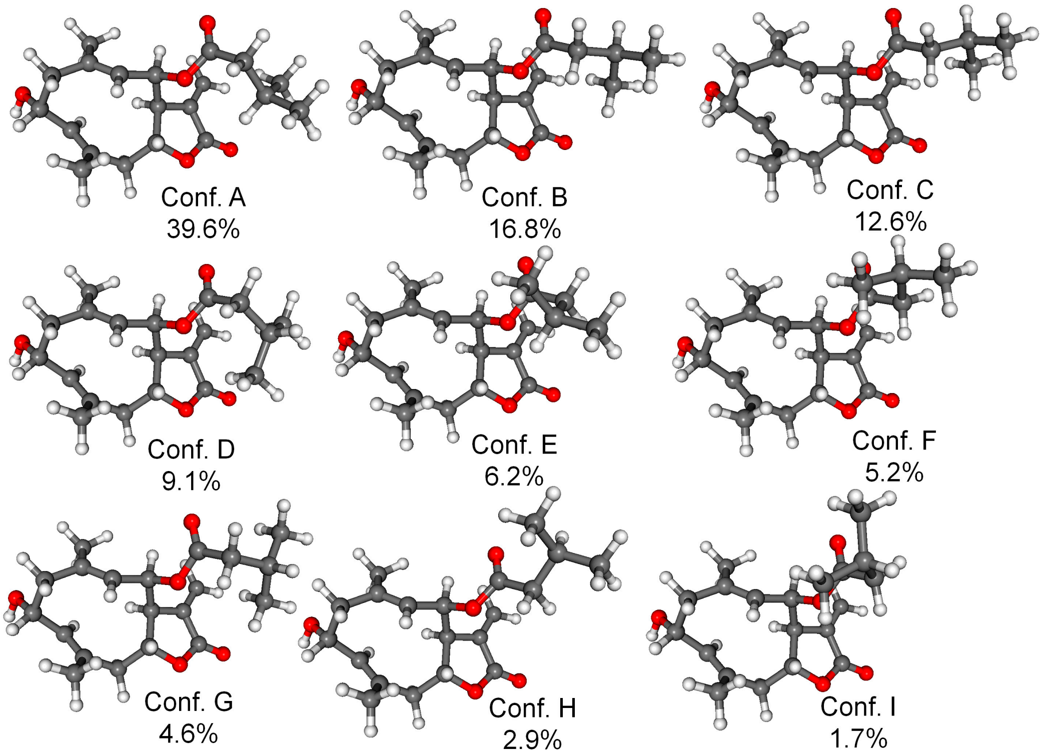

Figure S8. Structure and population of the low-energy (≥ 1%) ωB97XD/6-31+G(d,p) PCM/$CHCl_3$ conformers of (2*R*,6*R*,7*S*,8*R*)-**1**.

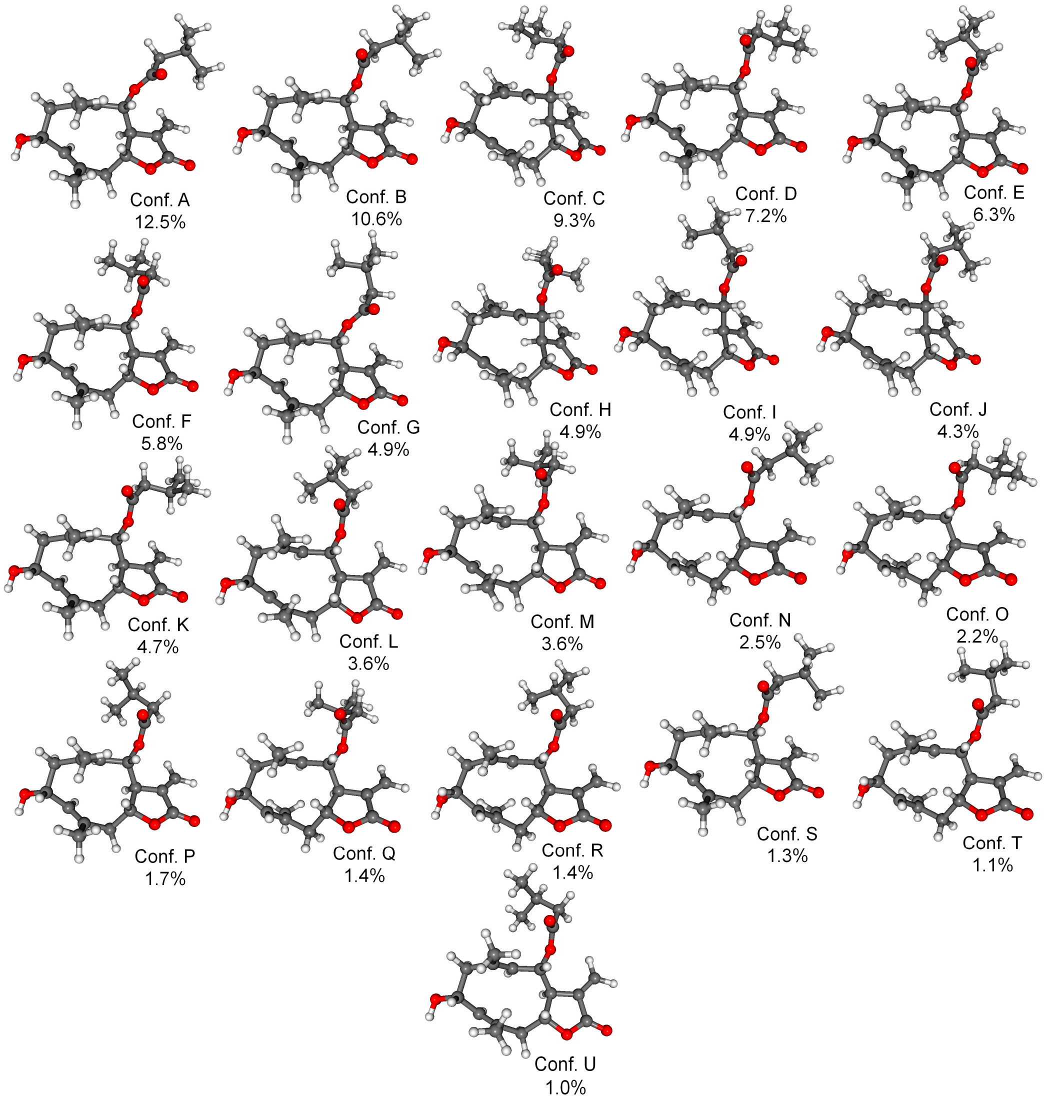

Figure S9. Structure and population of the low-energy (≥ 1%) ωB97XD/6-31+G(d,p) PCM/$CHCl_3$ conformers of (2*R*,6*R*,7*S*,8*S*)-**1**.

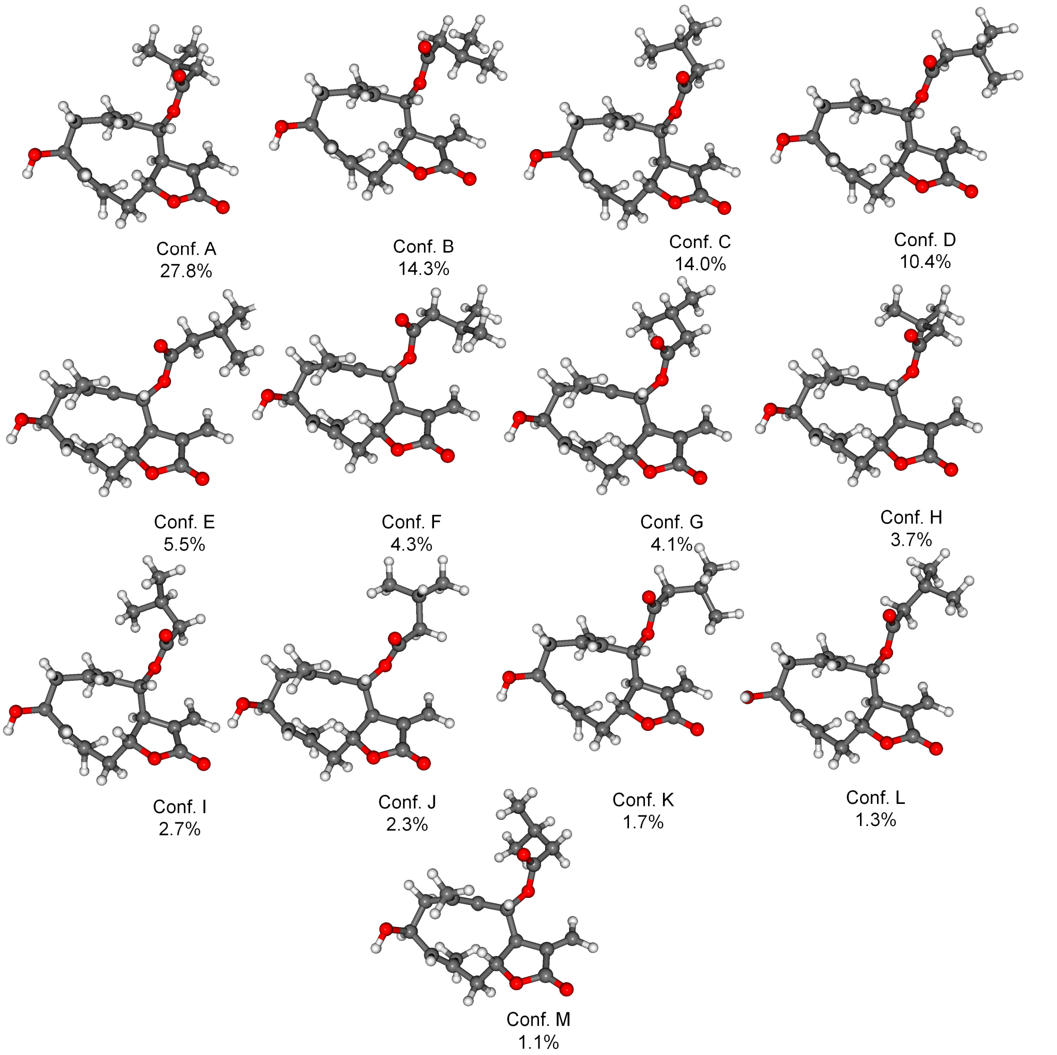

Figure S10. Structure and population of the low-energy (≥ 1%) ωB97XD/6-31+G(d,p) PCM/$CHCl_3$ conformers of (2*S*,6*R*,7*S*,8*S*)-**1**.

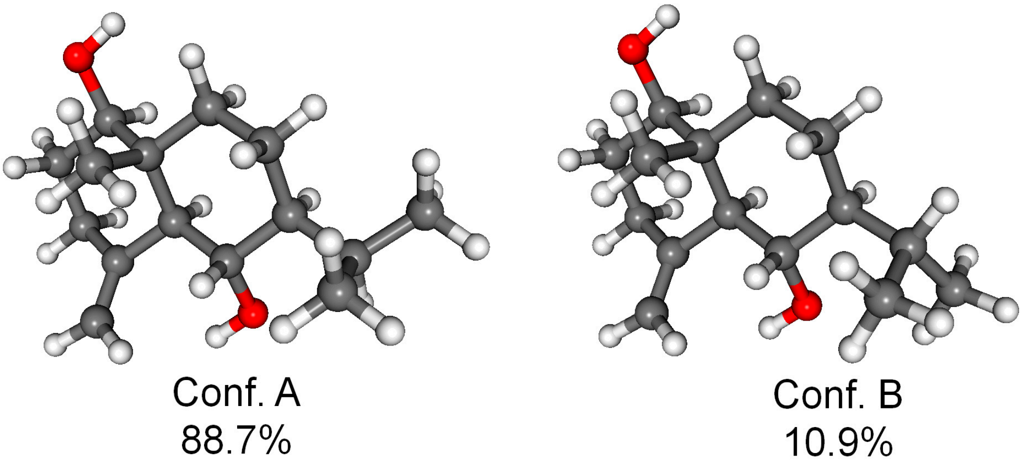

Figure S11. Structure and population of the low-energy (≥ 1%) ωB97XD/6-31+G(d,p) PCM/$CHCl_3$ conformers of (1*R*,5*S*,6*S*,7*S*,10*R*)-**2**.

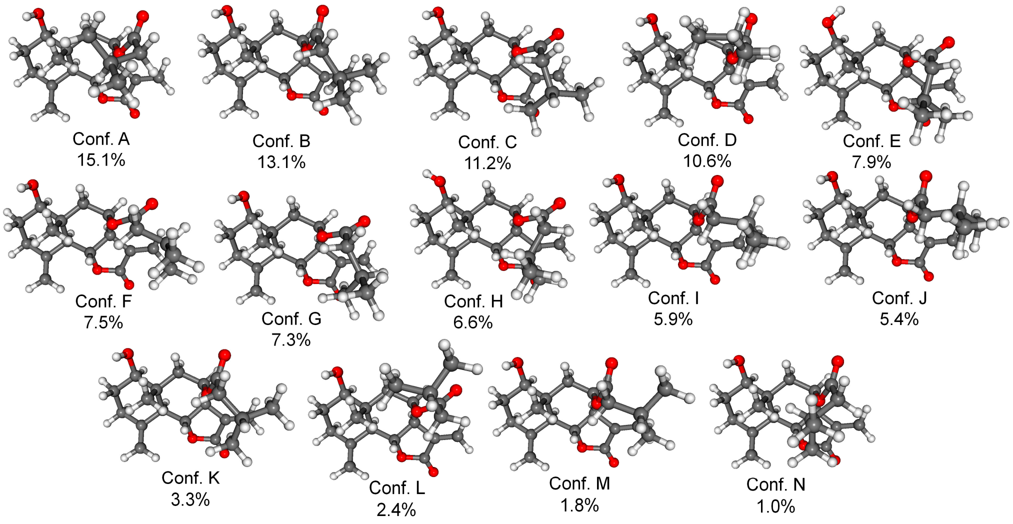

Figure S12. Structure and population of the low-energy (≥ 1%) ωB97X/6-31+G(d,p) PCM/$CHCl_3$ conformers of (1*R*,5*S*,6*R*,7*R*,8*R*,10*R*)-**3**.

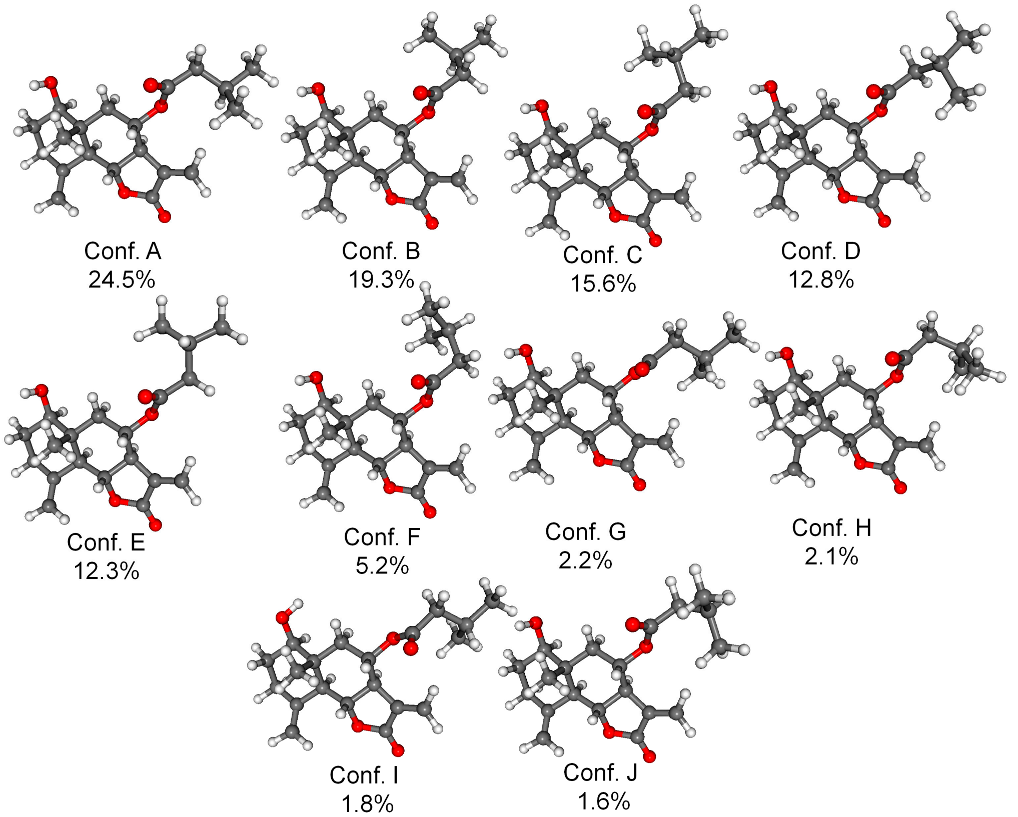

Figure S13. Structure and population of the low-energy (≥ 1%) ωB97X/6-31+G(d,p) PCM/$CHCl_3$ conformers of (1*R*,5*S*,6*R*,7*R*,8*S*,10*R*)-**3**.

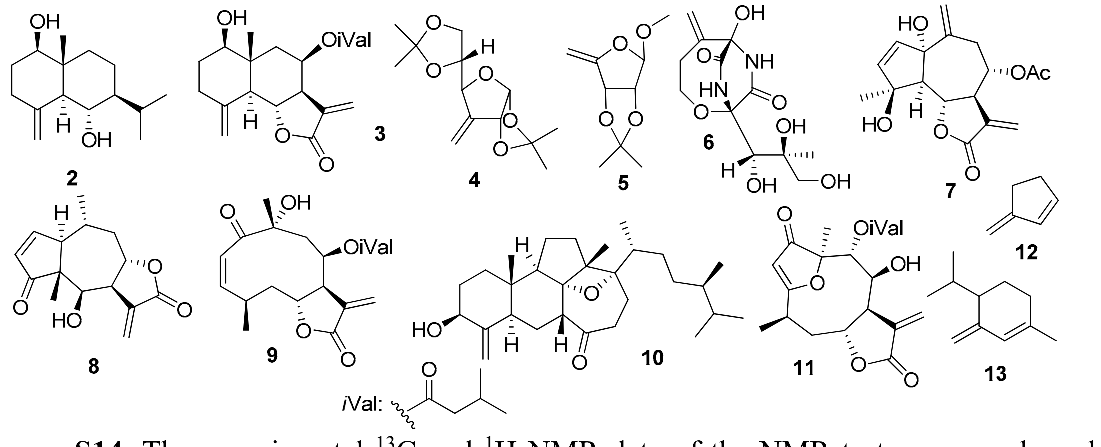

Figure S14. The experimental ^{13}C and ^{1}H NMR data of the NMR test compounds and reference compounds [volenol (**2**), 8*β*-isovaleroyloxyreynosin (**3**), 3-deoxy-1,2;5,6-di-*O*-isopropylidene-3-*C*-methylene-*α*-D-*ribo*-hexofuranose (**4**), methyl 5-deoxy-2,3-*O*-isopropylidene-*β*-D-*erythro*-pent-4-enofuranoside (**5**), bicyclomycin (**6**), 1*α*,4*β*-dihydroxy-8*α*-acetoxy-guaia-2,10(14),11(13)-triene-6,12-olide (**7**), mexicanin I (**8**), neurolenin A (**9**), swinhoeisterol F (**10**), lobatolide A (**11**), 3-methylenecyclopent-1-ene (**12**), 4-isopropyl-1-methyl-3-methylenecyclohex-1-ene (**13**)] can be found in refs. 1-10.

Table S1. Tested NMR combinations with available scaling factors found in the literature.[12-16]

No.	DFT optimizaton level	NMR level	Intercept	Slope
1	B3LYP/6-31G(d)	B3LYP/6-31G(d)	187.4743	-0.9269
2	B3LYP/6-31G(d)	B3LYP/6-31+G(d,p)	189.4397	-0.9468
3	B3LYP/6-31+G(d,p)	B3LYP/6-311+G(2d,p)	180.7713	-1.0311
4	B3LYP/6-31+G(d,p)	B3LYP/aug-cc-pVDZ	181.3782	-1.0228
5	B3LYP/6-31+G(d,p)	mPW1PW91/6-311+G(2d,p)	185.4855	-1.0306
6	B3LYP/6-31+G(d,p)	PBE0/6-311+G(2d,p)	186.2696	-1.0305
7	B3LYP/6-31+G(d,p)	WC04/aug-cc-pVDZ	196.9100	-0.9563
8	B3LYP/6-311+G(2d,p)	B3LYP/6-311+G(2d,p)	181.3782	-1.0228
9	B3LYP/6-311+G(2d,p)	mPW1PW91/6-311+G(2d,p)	186.0684	-1.0226
10	B3LYP/6-311+G(2d,p)	PBE0/6-311+G(2d,p)	186.8488	-1.0226
11	M06-2X/6-31G(d)	M06-2X/6-31G(d)	195.8694	-1.0591
12	M06-2X/6-31G(d)	M06-2X/6-31+G(d,p)	197.1285	-1.0741
13	M06-2X/6-31G(d)	M06-L/6-31G(d)	185.5136	-0.8512
14	M06-2X/6-31G(d)	M06-L/6-31+G(d,p)	187.7662	-0.8665
15	M06-2X/6-31G(d)	mPW1PW91/6-31G(d)	192.1504	-0.9349
16	M06-2X/6-31G(d)	mPW1PW91/6-31+G(d,p)	193.8769	-0.9511
17	M06-2X/6-31+G(d,p)	M06-2X/6-311+G(2d,p)	180.5121	-0.9295
18	M06-2X/6-31+G(d,p)	M06-L/6-311+G(2d,p)	181.0501	-0.9245
19	B3LYP/6-31G(d)	B3LYP/6-31G(d) SMD/$CHCl_3$	188.4418	-0.9449
20	B3LYP/6-31G(d)	B3LYP/6-31+G(d,p) SMD/$CHCl_3$	190.4279	-0.9679
21	B3LYP/6-31+G(d,p)	B3LYP/6-311+G(2d,p) SMD/$CHCl_3$	181.7815	-1.0537
22	B3LYP/6-31+G(d,p)	B3LYP/aug-cc-pVDZ SMD/$CHCl_3$	190.9490	-1.0048
23	B3LYP/6-31+G(d,p)	mPW1PW91/6-311+G(2d,p) SMD/$CHCl_3$	186.5242	-1.0533
24	B3LYP/6-31+G(d,p)	PBE0/6-311+G(2d,p) SMD/$CHCl_3$	187.3123	-1.0533
25	B3LYP/6-31+G(d,p)	VSXC/aug-cc-pVDZ SMD/$CHCl_3$	184.8018	-0.9589
26	B3LYP/6-31+G(d,p)	WC04/aug-cc-pVDZ SMD/$CHCl_3$	197.7614	-0.9760
27	B3LYP/6-311+G(2d,p)	B3LYP/6-311+G(2d,p) SMD/$CHCl_3$	182.3835	-1.0451
28	B3LYP/6-311+G(2d,p)	mPW1PW91/6-311+G(2d,p) SMD/$CHCl_3$	187.1018	-1.0449
29	B3LYP/6-311+G(2d,p)	PBE0/6-311+G(2d,p) SMD/$CHCl_3$	187.8859	-1.0450
30	M06-2X/6-31G(d)	M06-2X/6-31G(d) SMD/$CHCl_3$	196.9784	-1.0801
31	M06-2X/6-31G(d)	M06-2X/6-31+G(d,p) SMD/$CHCl_3$	198.2814	-1.0982
32	M06-2X/6-31G(d)	M06-L/6-31G(d) SMD/$CHCl_3$	186.5207	-0.8684
33	M06-2X/6-31G(d)	M06-L/6-31+G(d,p) SMD/$CHCl_3$	188.7538	-0.8856
34	M06-2X/6-31G(d)	mPW1PW91/6-31G(d) SMD/$CHCl_3$	193.2179	-0.9537
35	M06-2X/6-31G(d)	mPW1PW91/6-31+G(d,p) SMD/$CHCl_3$	194.9643	-0.9726
36	M06-2X/6-31+G(d,p)	M06-2X/6-311+G(2d,p) SMD/$CHCl_3$	189.5548	-1.1746
37	M06-2X/6-31+G(d,p)	M06-L/6-311+G(2d,p) SMD/$CHCl_3$	181.4397	-0.9491
38	M06-2X/6-31+G(d,p)	mPW1PW91/6-311+G(2d,p) SMD/$CHCl_3$	186.7246	-1.0446
39	M06-2X/6-311+G(2d,p)	M06-2X/6-311+G(2d,p) SMD/$CHCl_3$	189.9794	-1.1650
40	M06-2X/6-311+G(2d,p)	M06-L/6-311+G(2d,p) SMD/$CHCl_3$	181.9711	-0.9439
41	M06-2X/6-311+G(2d,p)	mPW1PW91/6-311+G(2d,p) SMD/$CHCl_3$	187.2065	-1.0379
42	B3LYP/6-31+G(d,p) SMD/$CHCl_3$	mPW1PW91/6-311+G(2d,p) SMD/$CHCl_3$	186.2627	-1.0512
43	B3LYP/6-31+G(d,p)	B3LYP/6-31G(d)	186.79	-0.93

44	B3LYP/6-31+G(d,p)	B3LYP/6-31G(d,p)	188.57	-0.94
45	B3LYP/6-31+G(d,p)	B3LYP/6-31+G(d,p)	189.00	-0.95
46	B3LYP/6-31+G(d,p)	B3LYP/6-31++G(d,p)	188.63	-0.95
47	B3LYP/6-31+G(d,p)	B3LYP/6-311G(d)	180.71	-1.02
48	B3LYP/6-31+G(d,p)	B3LYP/6-311G(d,p)	180.51	-1.02
49	B3LYP/6-31+G(d,p)	B3LYP/6-311+G(d,p)	180.62	-1.02
50	B3LYP/6-31+G(d,p)	B3LYP/6-311+G(2d,p)	180.09	-1.03
51	B3LYP/6-31+G(d,p)	B3LYP/6-311++G(d,p)	180.62	-1.03
52	B3LYP/6-31+G(d,p)	B3LYP/cc-pVDZ	189.61	-0.97
53	B3LYP/6-31+G(d,p)	B3LYP/aug-cc-pVDZ	189.19	-0.99
54	B3LYP/6-31+G(d,p)	B3LYP/cc-pVTZ	180.43	-1.03
55	B3LYP/6-31+G(d,p)	BMK/6-31G(d)	194.56	-1.04
56	B3LYP/6-31+G(d,p)	BMK/6-311G(d)	187.69	-1.25
57	B3LYP/6-31+G(d,p)	M06/6-31G(d)	183.90	-0.96
58	B3LYP/6-31+G(d,p)	M06-L/6-31G(d)	184.36	-0.86
59	B3LYP/6-31+G(d,p)	OPBE/6-31G(d)	189.55	-0.90
60	B3LYP/6-31+G(d,p)	VSXC/6-31G(d)	186.87	-0.86
61	B3LYP/6-31+G(d,p)	WC04/6-31G(d)	192.49	-0.90
62	B3LYP/6-31+G(d,p)	B3LYP/6-31G(d)	181.4540	-1.0074
63	B3LYP/6-31+G(d,p)	B3LYP/6-31G(d) PCM/$CHCl_3$	182.1337	-1.0222
64	B3LYP/6-31+G(d,p)	B3LYP/6-311+G(2d,p)	181.0114	-1.0262
65	B3LYP/6-31+G(d,p)	B3LYP/6-311+G(2d,p) PCM/$CHCl_3$	181.7173	-1.0427
66	B3LYP/6-31+G(d,p)	B3LYP/cc-pVDZ	190.7467	-0.9618
67	B3LYP/6-31+G(d,p)	B3LYP/cc-pVDZ PCM/$CHCl_3$	191.3459	-0.9742
68	B3LYP/6-31+G(d,p)	B3LYP/aug-cc-pVDZ	190.3004	-0.9825
69	B3LYP/6-31+G(d,p)	B3LYP/aug-cc-pVDZ PCM/$CHCl_3$	190.9642	-0.9974
70	B3LYP/6-31+G(d,p)	BMK/6-31G(d)	195.0388	-1.0302
71	B3LYP/6-31+G(d,p)	BMK/6-31G(d) PCM/$CHCl_3$	195.7931	-1.0453
72	B3LYP/6-31+G(d,p)	BMK/6-311G(d)	187.9681	-1.1115
73	B3LYP/6-31+G(d,p)	BMK/6-311G(d) PCM/$CHCl_3$	188.7195	-1.1285
74	B3LYP/6-31+G(d,p)	mPW1PW91/6-311+G(2d,p)	185.6419	-1.0255
75	B3LYP/6-31+G(d,p)	mPW1PW91/6-311+G(2d,p) PCM/$CHCl_3$	186.3567	-1.0420
76	B3LYP/6-31+G(d,p)	PBE0/6-311+G(2d,p)	186.4772	-1.0258
77	B3LYP/6-31+G(d,p)	PBE0/6-311+G(2d,p) PCM/$CHCl_3$	187.1937	-1.0423
78	B3LYP/6-31+G(d,p)	WC04/6-31G(d)	193.0729	-0.9007
79	B3LYP/6-31+G(d,p)	WC04/6-31G(d) PCM/$CHCl_3$	193.6390	-0.9124
80	mPW1PW91/6-311+G(2d,p) SMD/$CHCl_3$	mPW1PW91/6-311+G(2d,p) SMD/$CHCl_3$	187.9864	-1.0358

Table S2. Investigated combinations of computational levels with their MAE and $\Delta\delta_{max}$ values obtained for (1*R*,5*S*,6*S*,7*S*,10*R*)-**2**.

No.	DFT optimizaton level	NMR level	MAE	$\Delta\delta_{max}$
1	B3LYP/6-31G(d)	B3LYP/6-31G(d)	2.09	5.88
2	B3LYP/6-31G(d)	B3LYP/6-31+G(d,p)	3.18	8.33
3	B3LYP/6-31+G(d,p)	B3LYP/6-311+G(2d,p)	2.57	9.06
4	B3LYP/6-31+G(d,p)	B3LYP/aug-cc-pVDZ	3.41	9.51
5	B3LYP/6-31+G(d,p)	mPW1PW91/6-311+G(2d,p)	1.76	7.61
6	B3LYP/6-31+G(d,p)	PBE0/6-311+G(2d,p)	1.78	7.76
7	B3LYP/6-31+G(d,p)	WC04/aug-cc-pVDZ	3.07	10.63
8	B3LYP/6-311+G(2d,p)	B3LYP/6-311+G(2d,p)	2.61	9.21
9	B3LYP/6-311+G(2d,p)	mPW1PW91/6-311+G(2d,p)	1.76	7.76
10	B3LYP/6-311+G(2d,p)	PBE0/6-311+G(2d,p)	1.80	7.90
11	M06-2X/6-31G(d)	M06-2X/6-31G(d)	2.26	6.48
12	M06-2X/6-31G(d)	M06-2X/6-31+G(d,p)	2.20	8.56
13	M06-2X/6-31G(d)	M06-L/6-31G(d)	2.48	6.43
14	M06-2X/6-31G(d)	M06-L/6-31+G(d,p)	3.93	6.33
15	M06-2X/6-31G(d)	mPW1PW91/6-31G(d),	1.35	4.78
16	M06-2X/6-31G(d)	mPW1PW91/6-31+G(d,p)	1.84	7.30
17	M06-2X/6-31+G(d,p)	M06-2X/6-311+G(2d,p)	2.29	9.55
18	M06-2X/6-31+G(d,p)	M06-L/6-311+G(2d,p)	1.99	8.20
19	B3LYP/6-31G(d)	B3LYP/6-31G(d) SMD/$CHCl_3$	2.03	5.28
20	B3LYP/6-31G(d)	B3LYP/6-31+G(d,p) SMD/$CHCl_3$	3.14	7.51
21	B3LYP/6-31+G(d,p)	B3LYP/6-311+G(2d,p) SMD/$CHCl_3$	2.52	8.09
22	B3LYP/6-31+G(d,p)	B3LYP/aug-cc-pVDZ SMD/$CHCl_3$	3.34	8.61
23	B3LYP/6-31+G(d,p)	mPW1PW91/6-311+G(2d,p) SMD/$CHCl_3$	1.88	6.60
24	B3LYP/6-31+G(d,p)	PBE0/6-311+G(2d,p) SMD/$CHCl_3$	1.89	6.74
25	B3LYP/6-31+G(d,p)	VSXC/aug-cc-pVDZ SMD/$CHCl_3$	4.29	16.32
26	B3LYP/6-31+G(d,p)	WC04/aug-cc-pVDZ SMD/$CHCl_3$	3.41	10.36
27	B3LYP/6-311+G(2d,p)	B3LYP/6-311+G(2d,p) SMD/$CHCl_3$	2.54	8.22
28	B3LYP/6-311+G(2d,p)	mPW1PW91/6-311+G(2d,p) SMD/$CHCl_3$	1.82	6.73
29	B3LYP/6-311+G(2d,p)	PBE0/6-311+G(2d,p) SMD/$CHCl_3$	1.82	6.87
30	M06-2X/6-31G(d)	M06-2X/6-31G(d) SMD/$CHCl_3$	2.26	5.25
31	M06-2X/6-31G(d)	M06-2X/6-31+G(d,p) SMD/$CHCl_3$	2.08	7.56
32	M06-2X/6-31G(d)	M06-L/6-31G(d) SMD/$CHCl_3$	2.52	6.02
33	M06-2X/6-31G(d)	M06-L/6-31+G(d,p) SMD/$CHCl_3$	3.31	8.26
34	M06-2X/6-31G(d)	mPW1PW91/6-31G(d) SMD/$CHCl_3$	1.30	4.17
35	M06-2X/6-31G(d)	mPW1PW91/6-31+G(d,p) SMD/$CHCl_3$	1.84	6.49
36	M06-2X/6-31+G(d,p)	M06-2X/6-311+G(2d,p) SMD/$CHCl_3$	3.36	12.17
37	M06-2X/6-31+G(d,p)	M06-L/6-311+G(2d,p) SMD/$CHCl_3$	2.48	7.99
38	M06-2X/6-31+G(d,p)	mPW1PW91/6-311+G(2d,p) SMD/$CHCl_3$	1.87	7.33
39	M06-2X/6-311+G(2d,p)	M06-2X/6-311+G(2d,p) SMD/$CHCl_3$	2.14	8.54
40	M06-2X/6-311+G(2d,p)	M06-L/6-311+G(2d,p) SMD/$CHCl_3$	2.11	7.52
41	M06-2X/6-311+G(2d,p)	mPW1PW91/6-311+G(2d,p) SMD/$CHCl_3$	1.82	7.42
42	B3LYP/6-31+G(d,p) SMD/$CHCl_3$	mPW1PW91/6-311+G(2d,p) SMD/$CHCl_3$	1.75	6.69
43	B3LYP/6-31+G(d,p)	B3LYP/6-31G(d)	1.48	5.35

44	B3LYP/6-31+G(d,p)	B3LYP/6-31G(d,p)	1.95	6.06
45	B3LYP/6-31+G(d,p)	B3LYP/6-31+G(d,p)	2.87	8.06
46	B3LYP/6-31+G(d,p)	B3LYP/6-31++G(d,p)	2.65	7.69
47	B3LYP/6-31+G(d,p)	B3LYP/6-311G(d)	2.24	8.48
48	B3LYP/6-31+G(d,p)	B3LYP/6-311G(d,p)	2.26	8.82
49	B3LYP/6-31+G(d,p)	B3LYP/6-311+G(d,p)	2.44	9.90
50	B3LYP/6-31+G(d,p)	B3LYP/6-311+G(2d,p)	2.35	8.57
51	B3LYP/6-31+G(d,p)	B3LYP/6-311++G(d,p)	2.26	8.53
52	B3LYP/6-31+G(d,p)	B3LYP/cc-pVDZ	2.44	6.92
53	B3LYP/6-31+G(d,p)	B3LYP/aug-cc-pVDZ	2.83	7.76
54	B3LYP/6-31+G(d,p)	B3LYP/cc-pVTZ	2.23	7.80
55	B3LYP/6-31+G(d,p)	BMK/6-31G(d)	1.71	7.60
56	B3LYP/6-31+G(d,p)	BMK/6-311G(d)	4.49	10.52
57	B3LYP/6-31+G(d,p)	M06/6-31G(d)	1.32	3.31
58	B3LYP/6-31+G(d,p)	M06-L/6-31G(d)	1.98	5.09
59	B3LYP/6-31+G(d,p)	OPBE/6-31G(d)	1.51	2.99
60	B3LYP/6-31+G(d,p)	VSXC/6-31G(d)	4.85	10.34
61	B3LYP/6-31+G(d,p)	WC04/6-31G(d)	2.95	7.97
62	B3LYP/6-31+G(d,p)	B3LYP/6-31G(d)	8.23	11.61
63	B3LYP/6-31+G(d,p)	B3LYP/6-31G(d) PCM/$CHCl_3$	8.28	12.75
64	B3LYP/6-31+G(d,p)	B3LYP/6-311+G(2d,p)	2.83	10.04
65	B3LYP/6-31+G(d,p)	B3LYP/6-311+G(2d,p) PCM/$CHCl_3$	2.62	8.51
66	B3LYP/6-31+G(d,p)	B3LYP/cc-pVDZ	3.22	9.41
67	B3LYP/6-31+G(d,p)	B3LYP/cc-pVDZ PCM/$CHCl_3$	3.07	8.25
68	B3LYP/6-31+G(d,p)	B3LYP/aug-cc-pVDZ	3.60	10.07
69	B3LYP/6-31+G(d,p)	B3LYP/aug-cc-pVDZ PCM/$CHCl_3$	3.40	8.66
70	B3LYP/6-31+G(d,p)	BMK/6-31G(d)	2.40	9.53
71	B3LYP/6-31+G(d,p)	BMK/6-31G(d) PCM/$CHCl_3$	2.25	8.17
72	B3LYP/6-31+G(d,p)	BMK/6-311G(d)	2.01	11.52
73	B3LYP/6-31+G(d,p)	BMK/6-311G(d) PCM/$CHCl_3$	1.84	10.01
74	B3LYP/6-31+G(d,p)	mPW1PW91/6-311+G(2d,p)	2.00	9.88
75	B3LYP/6-31+G(d,p)	mPW1PW91/6-311+G(2d,p) PCM/$CHCl_3$	1.71	7.03
76	B3LYP/6-31+G(d,p)	PBE0/6-311+G(2d,p)	2.05	10.03
77	B3LYP/6-31+G(d,p)	PBE0/6-311+G(2d,p) PCM/$CHCl_3$	1.77	7.17
78	B3LYP/6-31+G(d,p)	WC04/6-31G(d)	2.79	7.35
79	B3LYP/6-31+G(d,p)	WC04/6-31G(d) PCM/$CHCl_3$	2.87	7.21
80	mPW1PW91/6-311+G(2d,p) SMD/$CHCl_3$	mPW1PW91/6-311+G(2d,p) SMD/$CHCl_3$	1.66	8.50

Table S3. Comparison of the experimental and the mPW1PW91/6-311+G(2d,p) // B3LYP/6-31+G(d,p) ^{13}C NMR data of (2*S*,6*R*,7*S*,8*R*)-**1** (isomer 1), (2*R*,6*R*,7*S*,8*R*)-**1** (isomer 2), (2*R*,6*R*,7*S*,8*S*)-**1** (isomer 3) and (2*S*,6*R*,7*S*,8*S*)-**1** (isomer 4). For a better comparison Δδ values over 2.5 were marked with yellow and those over 5.0 with red. (Supplementary table for Table 2)

Carbon	δ_{Exp} (ppm)	δ_{calc} isomer 1 (ppm)	δ_{calc} isomer 2 (ppm)	δ_{calc} isomer 3 (ppm)	δ_{calc} isomer 4 (ppm)	Δδ isomer 1 (ppm)	Δδ isomer 2 (ppm)	Δδ isomer 3 (ppm)	Δδ isomer 4 (ppm)
C-1	48.60	47.38	47.35	47.63	47.37	1.22	1.25	0.97	1.23
C-2	69.20	68.48	72.76	68.56	70.89	0.72	3.56	0.64	1.69
C-3	133.90	136.99	136.29	136.85	137.06	3.09	2.39	2.95	3.16
C-4	135.10	137.96	134.97	137.92	135.50	2.86	0.13	2.82	0.40
C-5	44.00	41.63	46.98	47.74	41.89	2.37	2.98	3.74	2.11
C-6	70.80	77.08	75.36	79.29	82.05	6.28	4.56	8.49	11.25
C-7	52.90	50.46	53.26	52.72	49.94	2.44	0.36	0.18	2.96
C-8	75.30	70.66	69.64	72.30	72.56	4.64	5.66	3.00	2.74
C-9	129.30	131.59	129.59	131.22	128.98	2.29	0.29	1.92	0.32
C-10	142.70	141.00	141.15	145.51	146.24	1.70	1.55	2.81	3.54
C-11	136.40	138.95	138.88	138.41	138.84	2.55	2.48	2.01	2.44
C-12	169.40	168.07	167.75	167.40	167.51	1.33	1.65	2.00	1.89
C-13	121.20	125.85	125.67	129.57	128.67	4.65	4.47	8.37	7.47
C-14	18.70	17.40	18.16	17.69	18.54	1.30	0.54	1.01	0.16
C-15	20.00	20.97	17.60	16.83	21.09	0.97	2.40	3.17	1.09
C-1'	171.80	173.10	172.98	172.65	172.60	1.30	1.18	0.85	0.80
C-2'	43.30	42.40	42.51	42.73	42.77	0.90	0.79	0.57	0.53
C-3'	25.40	27.51	27.53	27.60	27.80	2.11	2.13	2.20	2.40
C-4'	22.30	20.53	20.47	20.36	20.44	1.77	1.83	1.94	1.86
C-5'	22.30	20.34	20.43	20.63	20.61	1.96	1.87	1.67	1.69
MAE	N/A	N/A	N/A	N/A	N/A	2.32	2.10	2.57	2.49

Table S4. Comparison of the experimental and the mPW1PW91/6-311+G(2d,p) SMD /$CHCl_3$ // B3LYP/6-31+G(d,p) ^{13}C NMR data of (2*S*,6*R*,7*S*,8*R*)-**1** (isomer 1), (2*R*,6*R*,7*S*,8*R*)-**1** (isomer 2), (2*R*,6*R*,7*S*,8*S*)-**1** (isomer 3) and (2*S*,6*R*,7*S*,8*S*)-**1** (isomer 4). For a better comparison Δδ values over 2.5 were marked with yellow and those over 5.0 with red. (Supplementary table for Table 2)

Carbon	δ_{Exp} (ppm)	δ_{calc} isomer 1 (ppm)	δ_{calc} isomer 2 (ppm)	δ_{calc} isomer 3 (ppm)	δ_{calc} isomer 4 (ppm)	Δδ isomer 1 (ppm)	Δδ isomer 2 (ppm)	Δδ isomer 3 (ppm)	Δδ isomer 4 (ppm)
C-1	48.60	46.83	46.96	47.07	46.66	1.77	1.64	1.53	1.94
C-2	69.20	67.77	72.34	68.14	70.46	1.43	3.14	1.06	1.26
C-3	133.90	134.45	134.37	134.16	134.45	0.55	0.47	0.26	0.55
C-4	135.10	136.54	133.84	136.93	134.42	1.44	1.26	1.83	0.68
C-5	44.00	41.47	46.10	46.85	41.59	2.53	2.10	2.85	2.41
C-6	70.80	77.35	75.64	79.30	81.80	6.55	4.84	8.50	11.00
C-7	52.90	50.31	52.94	52.18	49.50	2.59	0.04	0.72	3.40
C-8	75.30	70.72	69.53	71.82	72.18	4.58	5.77	3.48	3.12
C-9	129.30	128.62	126.57	128.85	126.87	0.68	2.73	0.45	2.43
C-10	142.70	140.76	140.70	143.99	144.82	1.94	2.00	1.29	2.12
C-11	136.40	137.27	136.95	136.77	136.97	0.87	0.55	0.37	0.57
C-12	169.40	168.42	168.12	167.81	167.83	0.98	1.28	1.59	1.57
C-13	121.20	126.11	125.57	128.64	127.81	4.91	4.37	7.44	6.61
C-14	18.70	17.36	18.30	17.70	18.60	1.34	0.40	1.00	0.10
C-15	20.00	20.88	17.38	16.87	21.17	0.88	2.62	3.13	1.17
C-1'	171.80	172.41	172.26	171.84	171.97	0.61	0.46	0.04	0.17
C-2'	43.30	42.58	42.58	42.65	42.75	0.72	0.72	0.65	0.55
C-3'	25.40	27.99	28.14	28.32	28.54	2.59	2.74	2.92	3.14
C-4'	22.30	20.26	20.19	20.16	20.23	2.04	2.11	2.14	2.07
C-5'	22.30	20.14	20.22	20.43	20.40	2.16	2.08	1.87	1.90
MAE	N/A	N/A	N/A	N/A	N/A	2.06	2.07	2.16	2.34

Table S5. Comparison of the experimental and the mPW1PW91/6-311+G(2d,p) SMD /$CHCl_3$ // mPW1PW91/6-311+G(2d,p) SMD /$CHCl_3$ ^{13}C NMR data of (2*S*,6*R*,7*S*,8*R*)-**1** (isomer 1), (2*R*,6*R*,7*S*,8*R*)-**1** (isomer 2), (2*R*,6*R*,7*S*,8*S*)-**1** (isomer 3) and (2*S*,6*R*,7*S*,8*S*)-**1** (isomer 4). For a better comparison Δδ values over 2.5 were marked with yellow and those over 5.0 with red. (Supplementary table for Table 2)

Carbon	δ_{Exp} (ppm)	δ_{calc} isomer 1 (ppm)	δ_{calc} isomer 2 (ppm)	δ_{calc} isomer 3 (ppm)	δ_{calc} isomer 4 (ppm)	Δδ isomer 1 (ppm)	Δδ isomer 2 (ppm)	Δδ isomer 3 (ppm)	Δδ isomer 4 (ppm)
C-1	48.60	48.53	48.46	48.23	48.10	0.07	0.14	0.37	0.50
C-2	69.20	67.60	72.83	67.90	69.65	1.60	3.63	1.30	0.45
C-3	133.90	136.96	135.38	136.09	136.34	3.06	1.48	2.19	2.44
C-4	135.10	136.39	134.99	137.49	135.59	1.29	0.11	2.39	0.49
C-5	44.00	41.23	47.03	47.83	41.89	2.77	3.03	3.83	2.11
C-6	70.80	78.46	76.47	79.92	82.14	7.66	5.67	9.12	11.34
C-7	52.90	49.68	52.53	53.22	49.57	3.22	0.37	0.32	3.33
C-8	75.30	72.32	70.60	71.39	71.96	2.98	4.70	3.91	3.34
C-9	129.30	130.41	128.73	130.43	128.69	1.11	0.57	1.13	0.61
C-10	142.70	142.52	141.97	145.03	146.70	0.18	0.73	2.33	4.00
C-11	136.40	139.21	139.02	137.63	138.23	2.81	2.62	1.23	1.83
C-12	169.40	169.88	169.48	169.24	169.33	0.48	0.08	0.16	0.07
C-14	18.70	17.94	18.88	18.14	19.35	0.76	0.18	0.56	0.65
C-13	121.20	127.80	127.21	130.83	129.86	6.60	6.01	9.63	8.66
C-15	20.00	22.00	18.21	17.51	22.10	2.00	1.79	2.49	2.10
C-1'	171.80	173.56	173.40	173.04	173.15	1.76	1.60	1.24	1.35
C-2'	43.30	43.71	43.83	43.95	43.73	0.41	0.53	0.65	0.43
C-3'	25.40	27.87	28.39	28.60	28.29	2.47	2.99	3.20	2.89
C-4'	22.30	21.59	21.47	21.53	21.61	0.71	0.83	0.77	0.69
C-5'	22.30	21.20	21.46	21.48	21.41	1.10	0.84	0.82	0.89
MAE	N/A	N/A	N/A	N/A	N/A	2.15	1.89	2.38	2.41

Table S6. Comparison of the experimental and the mPW1PW91/6-31G(d) // M06-2X/6-31G(d) ^{13}C NMR data of (1*R*,5*S*,6*R*,7*R*,8*R*,10*R*)-**3** and (1*R*,5*S*,6*R*,7*R*,8*S*,10*R*)-**3**. For a better comparison Δδ values over 2.5 were marked with yellow and those over 5.0 with red. (Supplementary table for Table 3)

Carbon	δ_{Exp} (ppm)	δ_{calc} (8*R*)-epimer (ppm)	δ_{calc} (8*S*)-epimer (ppm)	Δδ (8*R*)-epimer (ppm)	Δδ (8*S*)-epimer (ppm)
C-1	78.80	79.24	78.81	0.44	0.01
C-2	31.10	31.78	31.53	0.68	0.43
C-3	33.50	33.88	33.95	0.38	0.45
C-4	142.00	144.08	144.19	2.08	2.19
C-5	53.70	54.53	53.22	0.83	0.48
C-6	75.20	73.49	75.69	1.71	0.49
C-7	52.20	54.57	52.70	2.37	0.50
C-8	65.90	66.65	71.18	0.75	5.28
C-9	40.60	40.28	41.13	0.32	0.53
C-10	42.80	43.43	42.85	0.63	0.05
C-11	134.80	138.03	140.55	3.23	5.75
C-12	170.00	168.47	168.48	1.53	1.52
C-13	119.60	126.66	123.76	7.06	4.16
C-14	13.80	13.73	12.81	0.07	0.99
C-15	111.10	117.12	117.39	6.02	6.29
C-1'	172.30	173.08	172.95	0.78	0.65
C-2'	43.80	43.51	42.63	0.29	1.17
C-3'	25.60	27.38	26.12	1.78	0.52
C-4'	22.60	22.30	21.62	0.30	0.98
C-5'	22.60	21.18	21.41	1.42	1.19
MAE	N/A	N/A	N/A	1.63	1.68

Table S7. Comparison of the experimental and the mPW1PW91/6-31G(d) SMD/$CHCl_3$ // M06-2X/6-31G(d) ^{13}C NMR data of (1*R*,5*S*,6*R*,7*R*,8*R*,10*R*)-**3** and (1*R*,5*S*,6*R*,7*R*,8*S*,10*R*)-**3**. For a better comparison Δδ values over 2.5 were marked with yellow and those over 5.0 with red. (Supplementary table for Table 3)

Carbon	δ_{Exp} (ppm)	δ_{calc} (8*R*)-epimer (ppm)	δ_{calc} (8*S*)-epimer (ppm)	Δδ (8*R*)-epimer (ppm)	Δδ (8*S*)-epimer (ppm)
C-1	78.80	78.21	77.78	0.59	1.02
C-2	31.10	31.66	31.47	0.56	0.37
C-3	33.50	33.83	33.87	0.33	0.37
C-4	142.00	145.98	145.94	3.98	3.94
C-5	53.70	53.85	52.62	0.15	1.08
C-6	75.20	73.88	75.79	1.32	0.59
C-7	52.20	54.14	52.24	1.94	0.04
C-8	65.90	66.93	71.04	1.03	5.14
C-9	40.60	40.14	41.08	0.46	0.48
C-10	42.80	43.85	43.35	1.05	0.55
C-11	134.80	137.25	139.48	2.45	4.68
C-12	170.00	168.92	168.89	1.08	1.11
C-13	119.60	125.85	123.35	6.25	3.75
C-14	13.80	14.18	13.07	0.38	0.73
C-15	111.10	113.03	113.42	1.93	2.32
C-1'	172.30	172.46	172.41	0.16	0.11
C-2'	43.80	43.58	42.66	0.22	1.14
C-3'	25.60	27.89	26.81	2.29	1.21
C-4'	22.60	22.26	21.55	0.34	1.05
C-5'	22.60	21.20	21.40	1.40	1.20
MAE	N/A	N/A	N/A	1.40	1.54

Table S8. Comparison of the experimental and the M06/6-31G(d) // B3LYP/6-31+G(d,p) ^{13}C NMR data of (1*R*,5*S*,6*R*,7*R*,8*R*,10*R*)-**3** and (1*R*,5*S*,6*R*,7*R*,8*S*,10*R*)-**3**. For a better comparison Δδ values over 2.5 were marked with yellow and those over 5.0 with red. (Supplementary table for Table 3)

Carbon	δ_{Exp} (ppm)	δ_{calc} (8*R*)-epimer (ppm)	δ_{calc} (8*S*)-epimer (ppm)	Δδ (8*R*)-epimer (ppm)	Δδ (8*S*)-epimer (ppm)
C-1	78.80	78.21	77.77	0.59	1.03
C-2	31.10	30.05	29.90	1.05	1.20
C-3	33.50	31.94	32.01	1.56	1.49
C-4	142.00	141.61	141.55	0.39	0.45
C-5	53.70	52.22	50.43	1.48	3.27
C-6	75.20	72.79	74.89	2.41	0.31
C-7	52.20	51.23	50.17	0.97	2.03
C-8	65.90	64.55	69.26	1.35	3.36
C-9	40.60	39.84	41.42	0.76	0.82
C-10	42.80	42.35	41.83	0.45	0.97
C-11	134.80	134.37	136.79	0.43	1.99
C-12	170.00	167.08	167.15	2.92	2.85
C-13	119.60	125.14	124.74	5.54	5.14
C-14	13.80	13.44	12.64	0.36	1.16
C-15	111.10	116.71	116.85	5.61	5.75
C-1'	172.30	172.18	171.55	0.12	0.75
C-2'	43.80	40.89	40.82	2.91	2.98
C-3'	25.60	23.60	23.79	2.00	1.81
C-4'	22.60	21.28	21.15	1.32	1.45
C-5'	22.60	21.17	21.25	1.43	1.35
MAE	N/A	N/A	N/A	1.68	2.01

Table S9. Comparison of the experimental and the OPBE0/6-31G(d) // B3LYP/6-31+G(d,p) ^{13}C NMR data of (1*R*,5*S*,6*R*,7*R*,8*R*,10*R*)-**3** and (1*R*,5*S*,6*R*,7*R*,8*S*,10*R*)-**3**. For a better comparison Δδ values over 2.5 were marked with yellow and those over 5.0 with red. (Supplementary table for Table 3)

Carbon	δ_{Exp} (ppm)	δ_{calc} (8*R*)-epimer (ppm)	δ_{calc} (8*S*)-epimer (ppm)	Δδ (8*R*)-epimer (ppm)	Δδ (8*S*)-epimer (ppm)
C-1	78.80	80.80	80.57	2.00	1.77
C-2	31.10	30.68	30.55	0.42	0.55
C-3	33.50	33.73	33.81	0.23	0.31
C-4	142.00	141.53	141.53	0.47	0.47
C-5	53.70	53.44	51.70	0.26	2.00
C-6	75.20	75.11	77.27	0.09	2.07
C-7	52.20	53.79	52.29	1.59	0.09
C-8	65.90	68.60	72.30	2.70	6.40
C-9	40.60	39.27	39.82	1.33	0.78
C-10	42.80	45.31	44.77	2.51	1.97
C-11	134.80	136.56	139.17	1.76	4.37
C-12	170.00	166.04	166.15	3.96	3.85
C-13	119.60	122.17	121.53	2.57	1.93
C-14	13.80	10.79	9.94	3.01	3.86
C-15	111.10	115.49	115.56	4.39	4.46
C-1'	172.30	170.46	170.04	1.84	2.26
C-2'	43.80	40.96	40.90	2.84	2.90
C-3'	25.60	26.10	26.40	0.50	0.80
C-4'	22.60	19.64	19.46	2.96	3.14
C-5'	22.60	19.42	19.67	3.18	2.93
MAE	N/A	N/A	N/A	1.93	2.35

Table S10. Comparison of the experimental and the mPW1PW91/6-31G(d) SMD/$CHCl_3$ // M06-2X/6-31G(d) ^{13}C NMR data of (2*S*,6*R*,7*S*,8*R*)-**1** (isomer 1), (2*R*,6*R*,7*S*,8*R*)-**1** (isomer 2), (2*R*,6*R*,7*S*,8*S*)-**1** (isomer 3) and (2*S*,6*R*,7*S*,8*S*)-**1** (isomer 4). For a better comparison Δδ values over 2.5 were marked with yellow and those over 5.0 with red. (Supplementary table for Table 4)

Carbon	δ_{Exp} (ppm)	δ_{calc} isomer 1 (ppm)	δ_{calc} isomer 2 (ppm)	δ_{calc} isomer 3 (ppm)	δ_{calc} isomer 4 (ppm)	Δδ isomer 1 (ppm)	Δδ isomer 2 (ppm)	Δδ isomer 3 (ppm)	Δδ isomer 4 (ppm)
C-1	48.60	48.03	48.06	48.29	48.48	0.57	0.54	0.31	0.12
C-2	69.20	68.43	72.68	71.61	70.49	0.77	3.48	2.41	1.29
C-3	133.90	135.64	135.73	134.64	136.34	1.74	1.83	0.74	2.44
C-4	135.10	136.36	133.67	136.48	134.34	1.26	1.43	1.38	0.76
C-5	44.00	42.90	47.17	46.38	42.75	1.10	3.17	2.38	1.25
C-6	70.80	78.23	76.31	79.58	82.88	7.43	5.51	8.78	12.08
C-7	52.90	50.15	53.05	50.17	48.95	2.75	0.15	2.73	3.95
C-8	75.30	72.16	70.98	74.18	73.98	3.14	4.32	1.12	1.32
C-9	129.30	129.86	127.58	127.76	128.18	0.56	1.72	1.54	1.12
C-10	142.70	139.74	140.18	146.11	144.76	2.96	2.52	3.41	2.06
C-11	136.40	135.83	135.89	137.60	137.38	0.57	0.51	1.20	0.98
C-12	169.40	168.71	168.30	167.74	167.70	0.69	1.10	1.66	1.70
C-13	121.20	131.98	132.28	130.88	130.93	10.78	11.08	9.68	9.73
C-14	18.70	18.67	19.53	20.33	19.77	0.03	0.83	1.63	1.07
C-15	20.00	21.97	19.29	20.27	22.90	1.97	0.71	0.27	2.90
C-1'	171.80	172.42	172.30	171.90	172.19	0.62	0.50	0.10	0.39
C-2'	43.30	43.47	43.61	42.94	43.08	0.17	0.31	0.36	0.22
C-3'	25.40	27.48	27.82	27.53	27.76	2.08	2.42	2.13	2.36
C-4'	22.30	21.89	21.42	21.44	21.73	0.41	0.88	0.86	0.57
C-5'	22.30	22.35	22.30	22.07	21.75	0.05	0.00	0.23	0.55
MAE	N/A	N/A	N/A	N/A	N/A	2.42	2.59	2.62	2.85

Table S11. Comparison of the experimental and the M06/6-31G(d) // B3LYP/6-31+G(d,p) ^{13}C NMR data of (2*S*,6*R*,7*S*,8*R*)-**1** (isomer 1), (2*R*,6*R*,7*S*,8*R*)-**1** (isomer 2), (2*R*,6*R*,7*S*,8*S*)-**1** (isomer 3) and (2*S*,6*R*,7*S*,8*S*)-**1** (isomer 4). For a better comparison Δδ values over 2.5 were marked with yellow and those over 5.0 with red. (Supplementary table for Table 4)

Carbon	δ_{Exp} (ppm)	δ_{calc} isomer 1 (ppm)	δ_{calc} isomer 2 (ppm)	δ_{calc} isomer 3 (ppm)	δ_{calc} isomer 4 (ppm)	Δδ isomer 1 (ppm)	Δδ isomer 2 (ppm)	Δδ isomer 3 (ppm)	Δδ isomer 4 (ppm)
C-1	48.60	46.43	46.94	46.73	46.71	2.17	1.66	1.87	1.89
C-2	69.20	66.75	71.55	67.12	68.87	2.45	2.35	2.08	0.33
C-3	133.90	135.66	136.06	136.20	136.48	1.76	2.16	2.30	2.58
C-4	135.10	134.88	131.50	134.90	132.61	0.22	3.60	0.20	2.49
C-5	44.00	40.65	45.43	46.88	40.51	3.35	1.43	2.88	3.49
C-6	70.80	76.76	74.92	78.82	81.71	5.96	4.12	8.02	10.91
C-7	52.90	48.06	50.87	49.80	47.09	4.84	2.03	3.10	5.81
C-8	75.30	70.35	69.43	72.62	73.04	4.95	5.87	2.68	2.26
C-9	129.30	131.49	129.63	130.89	128.49	2.19	0.33	1.59	0.81
C-10	142.70	137.05	138.25	142.00	143.43	5.65	4.45	0.70	0.73
C-11	136.40	135.63	135.31	134.57	135.12	0.77	1.09	1.83	1.28
C-12	169.40	166.47	165.87	165.71	165.96	2.93	3.53	3.69	3.44
C-13	121.20	127.98	128.25	132.94	132.11	6.78	7.05	11.74	10.91
C-14	18.70	17.33	17.83	17.53	18.49	1.37	0.87	1.17	0.21
C-15	20.00	21.35	18.09	16.03	20.82	1.35	1.91	3.97	0.82
C-1'	171.80	170.77	170.81	170.90	170.86	1.03	0.99	0.90	0.94
C-2'	43.30	40.43	40.53	40.52	40.74	2.87	2.77	2.78	2.56
C-3'	25.40	23.91	23.54	23.62	23.82	1.49	1.86	1.78	1.58
C-4'	22.30	20.88	20.73	21.02	21.13	1.42	1.57	1.28	1.17
C-5'	22.30	20.86	21.28	21.42	21.24	1.44	1.02	0.88	1.06
MAE	N/A	N/A	N/A	N/A	N/A	3.12	2.83	3.19	2.76

Table S12. Comparison of the experimental and the mPW1PW91/6-311+G(2d,p) // ωB97XD/6-31+G(d,p) ^{1}H NMR data of (1*R*,5*S*,6*S*,7*S*,10*R*)-**2**. For a better comparison Δδ values over 0.3 were marked with yellow and those over 0.6 with red. For the CMAE values of the protons the shielding constants related to the hydrogen atoms in the methyl group were averaged (same for the other ^{1}H NMR tables).[16]

Hydrogen	δ_{Exp} (ppm)	δ_{Calc} (ppm)	Δδ (ppm)
H-1	3.42	3.41	0.01
H-2a	1.86	1.63	0.23
H-2b	1.86	1.87	0.01
H-3a	1.55	2.09	0.54
H-3b	2.07	2.31	0.24
H-5	1.75	1.80	0.05
H-6	3.71	3.82	0.11
H-7	2.33	1.29	1.04
H-8a	1.55	1.35	0.20
H-8b	1.92	1.51	0.41
H-9a	1.21	1.12	0.09
H-9b	1.28	1.76	0.48
H-11	2.24	2.37	0.13
H-12 (3x)	0.87	0.99	0.12
H-13 (3x)	0.95	1.00	0.05
H-14 (3x)	0.71	0.83	0.12
H-15a	5.02	5.05	0.03
H-15b	4.74	4.78	0.04
MAE	N/A	N/A	0.19

Table S13. Comparison of the experimental and the mPW1PW91/6-311+G(2d,p) SMD/$CHCl_3$ // ωB97XD/6-31+G(d,p) SMD/$CHCl_3$ ^{1}H NMR data of (1*R*,5*S*,6*S*,7*S*,10*R*)-**2**. For a better comparison Δδ values over 0.3 were marked with yellow and those over 0.6 with red.

Hydrogen	δ_{Exp} (ppm)	δ_{Calc} (ppm)	Δδ (ppm)
H-1	3.42	3.46	0.04
H-2a	1.86	1.59	0.27
H-2b	1.86	1.82	0.04
H-3a	1.55	2.11	0.56
H-3b	2.07	2.33	0.26
H-5	1.75	1.84	0.09
H-6	3.71	3.87	0.16
H-7	2.33	1.26	1.07
H-8a	1.55	1.32	0.23
H-8b	1.92	1.49	0.43
H-9a	1.21	1.16	0.05
H-9b	1.28	1.78	0.50
H-11	2.24	2.23	0.01
H-12 (3x)	0.87	0.95	0.08
H-13 (3x)	0.95	0.98	0.03
H-14 (3x)	0.71	0.77	0.06
H-15a	5.02	5.01	0.01
H-15b	4.74	4.73	0.01
MAE	N/A	N/A	0.18

Table S14. Comparison of the experimental and the mPW1PW91/6-311+G(2d,p) // ωB97XD/6-31+G(d,p) ^{13}C NMR data of (1*R*,5*S*,6*R*,7*R*,8*R*,10*R*)-**3** and (1*R*,5*S*,6*R*,7*R*,8*S*,10*R*)-**3**. For a better comparison Δδ values over 2.5 were marked with yellow and those over 5.0 with red. (Supplementary table for Table 7)

Carbon	δ_{Exp} (ppm)	δ_{calc} (8*R*)-epimer	δ_{calc} (8*S*)-epimer	Δδ (8*R*)-epimer	Δδ (8*S*)-epimer
C-1	78.80	77.74	77.34	1.06	1.46
C-2	31.10	33.15	33.46	2.05	2.36
C-3	33.50	35.02	35.12	1.52	1.62
C-4	142.00	145.15	145.14	3.15	3.14
C-5	53.70	54.85	53.24	1.15	0.46
C-6	75.20	71.76	74.36	3.44	0.84
C-7	52.20	54.01	52.63	1.81	0.43
C-8	65.90	64.32	68.43	1.58	2.53
C-9	40.60	40.66	41.54	0.06	0.94
C-10	42.80	44.86	44.03	2.06	1.23
C-11	134.80	136.67	140.52	1.87	5.72
C-12	170.00	166.26	166.31	3.74	3.69
C-13	119.60	122.64	120.08	3.04	0.48
C-14	13.80	12.65	11.33	1.15	2.47
C-15	111.10	112.54	112.83	1.44	1.73
C-1'	172.30	170.64	170.71	1.66	1.59
C-2'	43.80	43.59	43.23	0.21	0.57
C-3'	25.60	29.12	29.11	3.52	3.51
C-4'	22.60	21.97	21.44	0.63	1.16
C-5'	22.60	21.27	21.08	1.33	1.52
MAE	N/A	N/A	N/A	1.82	1.87

Table S15. Comparison of the experimental and the mPW1PW91/6-311+G(2d,p) SMD/$CHCl_3$ // ωB97XD/6-31+G(d,p) SMD/$CHCl_3$ ^{13}C NMR data of (1*R*,5*S*,6*R*,7*R*,8*R*,10*R*)-**3** and (1*R*,5*S*,6*R*,7*R*,8*S*,10*R*)-**3**. For a better comparison Δδ values over 2.5 were marked with yellow and those over 5.0 with red. (Supplementary table for Table 7)

Carbon	δ_{Exp} (ppm)	δ_{calc} (8*R*)-epimer	δ_{calc} (8*S*)-epimer	Δδ (8*R*)-epimer	Δδ (8*S*)-epimer
C-1	78.80	77.51	77.35	1.29	1.45
C-2	31.10	32.71	32.86	1.61	1.76
C-3	33.50	35.50	35.66	2.00	2.16
C-4	142.00	147.87	147.33	5.87	5.33
C-5	53.70	53.78	52.42	0.08	1.28
C-6	75.20	73.68	75.76	1.52	0.56
C-7	52.20	52.82	52.47	0.62	0.27
C-8	65.90	65.40	68.45	0.50	2.55
C-9	40.60	40.43	41.66	0.17	1.06
C-10	42.80	45.90	45.11	3.10	2.31
C-11	134.80	136.25	139.09	1.45	4.29
C-12	170.00	168.02	168.04	1.98	1.96
C-13	119.60	120.91	120.30	1.31	0.70
C-14	13.80	13.46	11.55	0.34	2.25
C-15	111.10	108.45	108.51	2.65	2.59
C-1'	172.30	171.35	171.70	0.95	0.60
C-2'	43.80	44.08	43.81	0.28	0.01
C-3'	25.60	29.78	30.06	4.18	4.46
C-4'	22.60	21.78	21.49	0.82	1.11
C-5'	22.60	21.36	21.13	1.24	1.47
MAE	N/A	N/A	N/A	1.60	1.91

Table S16. Comparison of the experimental and the mPW1PW91/6-311+G(2d,p) // ωB97XD/6-31+G(d,p) ^{1}H NMR data of (1*R*,5*S*,6*R*,7*R*,8*R*,10*R*)-**3** and (1*R*,5*S*,6*R*,7*R*,8*S*,10*R*)-**3**. For a better comparison Δδ values over 0.3 were marked with yellow and those over 0.6 with red.

Hydrogen	δ_{Exp} (ppm)	δ_{calc} (8*R*)-epimer (ppm)	δ_{calc} (8*S*)-epimer (ppm)	Δδ (8*R*)-epimer (ppm)	Δδ (8*S*)-epimer (ppm)
H-1	3.50	3.51	3.52	0.01	0.02
H-2a	1.81	1.74	1.72	0.07	0.09
H-2b	1.58	1.55	1.50	0.03	0.08
H-3a	2.35	2.34	2.33	0.01	0.02
H-3b	2.13	2.18	2.15	0.05	0.02
H-5	2.23	2.18	2.11	0.05	0.12
H-6	4.50	4.39	3.90	0.11	0.60
H-7	2.79	2.61	2.72	0.18	0.07
H-8	5.75	5.92	5.10	0.17	0.65
H-9a	2.31	2.40	2.77	0.09	0.46
H-9b	1.56	1.51	1.18	0.05	0.38
H-13a	6.15	6.34	6.24	0.19	0.09
H-13b	5.44	5.90	5.71	0.46	0.27
H-14a	5.01	5.24	5.23	0.23	0.22
H-14b	4.94	5.21	5.19	0.27	0.25
H-15 (3x)	0.96	1.24	0.99	0.28	0.03
H-2'a	2.17	2.22	2.24	0.05	0.07
H-2'b	2.16	2.19	2.17	0.03	0.01
H-3'	2.06	2.09	2.18	0.03	0.12
H-4' (3x)	0.93	0.99	1.05	0.06	0.12
H-5' (3x)	0.92	0.96	1.03	0.04	0.11
CMAE	N/A	N/A	N/A	0.12	0.16

Table S17. Comparison of the experimental and the mPW1PW91/6-311+G(2d,p) SMD/$CHCl_3$ // ωB97XD/6-31+G(d,p) SMD/$CHCl_3$ ^{1}H NMR data of (1*R*,5*S*,6*R*,7*R*,8*R*,10*R*)-**3** and (1*R*,5*S*,6*R*,7*R*,8*S*,10*R*)-**3**. For a better comparison Δδ values over 0.3 were marked with yellow and those over 0.6 with red.

Hydrogen	δ_{Exp} (ppm)	δ_{calc} (8*R*)-epimer (ppm)	δ_{calc} (8*S*)-epimer (ppm)	Δδ (8*R*)-epimer (ppm)	Δδ (8*S*)-epimer (ppm)
H-1	3.50	3.46	3.42	0.04	0.08
H-2a	1.81	1.95	1.97	0.14	0.16
H-2b	1.58	1.57	1.53	0.01	0.05
H-3a	2.35	2.46	2.42	0.11	0.07
H-3b	2.13	2.29	2.27	0.16	0.14
H-5	2.23	2.45	2.38	0.22	0.15
H-6	4.50	4.63	4.21	0.13	0.29
H-7	2.79	2.93	2.98	0.14	0.19
H-8	5.75	5.79	5.21	0.04	0.54
H-9a	2.31	2.33	2.52	0.02	0.21
H-9b	1.56	1.66	1.36	0.10	0.20
H-13a	6.15	6.43	6.36	0.28	0.21
H-13b	5.44	5.83	5.92	0.39	0.48
H-14a	5.01	5.24	5.23	0.23	0.22
H-14b	4.94	5.12	5.05	0.18	0.11
H-15 (3x)	0.96	1.04	0.86	0.08	0.10
H-2'a	2.17	2.30	2.37	0.13	0.20
H-2'b	2.16	2.30	2.29	0.14	0.13
H-3'	2.06	2.17	2.25	0.11	0.19
H-4' (3x)	0.93	1.03	1.07	0.10	0.14
H-5' (3x)	0.92	0.99	1.06	0.07	0.14
CMAE	N/A	N/A	N/A	0.12	0.18

Table S18. Comparison of the experimental and the mPW1PW91/6-311+G(2d,p) // ωB97XD/6-31+G(d,p) ^{13}C NMR data of (2*S*,6*R*,7*S*,8*R*)-**1** (isomer 1), (2*R*,6*R*,7*S*,8*R*)-**1** (isomer 2), (2*R*,6*R*,7*S*,8*S*)-**1** (isomer 3) and (2*S*,6*R*,7*S*,8*S*)-**1** (isomer 4). For a better comparison Δδ values over 2.5 were marked with yellow and those over 5.0 with red. (Supplementary table for Table 8)

Carbon	δ_{Exp} (ppm)	δ_{calc} isomer 1 (ppm)	δ_{calc} isomer 2 (ppm)	δ_{calc} isomer 3 (ppm)	δ_{calc} isomer 4 (ppm)	Δδ isomer 1 (ppm)	Δδ isomer 2 (ppm)	Δδ isomer 3 (ppm)	Δδ isomer 4 (ppm)
C-1	48.60	46.77	46.08	46.72	46.34	1.83	2.52	1.88	2.26
C-2	69.20	66.68	71.23	69.48	67.98	2.52	2.03	0.28	1.22
C-3	133.90	135.65	134.65	133.52	136.61	1.75	0.75	0.38	2.71
C-4	135.10	135.59	134.98	138.17	133.87	0.49	0.12	3.07	1.23
C-5	44.00	42.07	46.79	46.43	41.95	1.93	2.79	2.43	2.05
C-6	70.80	75.81	73.77	77.99	79.54	5.01	2.97	7.19	8.74
C-7	52.90	49.23	51.87	50.53	48.71	3.67	1.03	2.37	4.19
C-8	75.30	68.81	67.68	70.70	70.36	6.49	7.62	4.60	4.94
C-9	129.30	130.05	127.40	128.03	127.67	0.75	1.90	1.27	1.63
C-10	142.70	140.30	140.09	145.65	146.05	2.40	2.61	2.95	3.35
C-11	136.40	136.82	137.00	137.39	137.54	0.42	0.60	0.99	1.14
C-12	169.40	165.94	165.56	165.16	165.30	3.46	3.84	4.24	4.10
C-13	121.20	127.40	127.72	127.76	127.56	6.20	6.52	6.56	6.36
C-14	18.70	17.83	18.60	18.90	19.21	0.87	0.10	0.20	0.51
C-15	20.00	21.50	18.16	19.72	22.21	1.50	1.84	0.28	2.21
C-1'	171.80	170.72	170.65	169.95	169.89	1.08	1.15	1.85	1.91
C-2'	43.30	43.61	43.65	43.55	43.33	0.31	0.35	0.25	0.03
C-3'	25.40	29.58	29.47	28.93	29.35	4.18	4.07	3.53	3.95
C-4'	22.30	20.97	21.11	21.18	21.44	1.33	1.19	1.12	0.86
C-5'	22.30	21.86	21.61	21.43	21.47	0.44	0.69	0.87	0.83
MAE	N/A	N/A	N/A	N/A	N/A	2.33	2.48	2.58	3.11

Table S19. Comparison of the experimental and the mPW1PW91/6-311+G(2d,p) SMD/$CHCl_3$ // ωB97XD/6-31+G(d,p) SMD/$CHCl_3$ ^{13}C NMR data of (2*S*,6*R*,7*S*,8*R*)-**1** (isomer 1), (2*R*,6*R*,7*S*,8*R*)-**1** (isomer 2), (2*R*,6*R*,7*S*,8*S*)-**1** (isomer 3) and (2*S*,6*R*,7*S*,8*S*)-**1** (isomer 4). For a better comparison Δδ values over 2.5 were marked with yellow and those over 5.0 with red. (Supplementary table for Table 8)

Carbon	δ_{Exp} (ppm)	δ_{calc} isomer 1 (ppm)	δ_{calc} isomer 2 (ppm)	δ_{calc} isomer 3 (ppm)	δ_{calc} isomer 4 (ppm)	Δδ isomer 1 (ppm)	Δδ isomer 2 (ppm)	Δδ isomer 3 (ppm)	Δδ isomer 4 (ppm)
C-1	48.60	46.44	45.88	46.06	46.06	2.16	2.72	2.54	2.54
C-2	69.20	66.23	71.22	67.92	67.30	2.97	2.02	1.28	1.90
C-3	133.90	134.81	133.29	133.53	135.28	0.91	0.61	0.37	1.38
C-4	135.10	134.03	134.25	135.28	133.42	1.07	0.85	0.18	1.68
C-5	44.00	40.78	46.05	46.42	41.18	3.22	2.05	2.42	2.82
C-6	70.80	77.55	75.60	78.61	80.11	6.75	4.80	7.81	9.31
C-7	52.90	48.94	51.26	51.60	48.62	3.96	1.64	1.30	4.28
C-8	75.30	71.28	69.48	70.58	70.79	4.02	5.82	4.72	4.51
C-9	129.30	127.74	125.79	126.84	125.74	1.56	3.51	2.46	3.56
C-10	142.70	141.54	140.81	143.93	146.64	1.16	1.89	1.23	3.94
C-11	136.40	136.40	136.65	135.51	136.39	0.00	0.25	0.89	0.01
C-12	169.40	167.28	166.94	166.82	167.11	2.12	2.46	2.58	2.29
C-13	121.20	126.40	127.11	129.14	128.38	5.20	5.91	7.94	7.18
C-14	18.70	18.12	18.79	18.64	19.63	0.58	0.09	0.06	0.93
C-15	20.00	21.80	18.26	18.52	22.12	1.80	1.74	1.48	2.12
C-1'	171.80	171.22	171.20	170.99	171.11	0.58	0.60	0.81	0.69
C-2'	43.30	43.93	44.06	43.90	43.89	0.63	0.76	0.60	0.59
C-3'	25.40	29.88	30.04	29.73	30.00	4.48	4.64	4.33	4.60
C-4'	22.30	21.14	21.21	21.55	21.35	1.16	1.09	0.75	0.95
C-5'	22.30	21.44	21.45	21.43	21.62	0.86	0.85	0.87	0.68
MAE	N/A	N/A	N/A	N/A	N/A	2.26	2.42	2.48	3.23

Table S20. Comparison of the experimental and the mPW1PW91/6-311+G(2d,p) // ωB97XD/6-31+G(d,p) ^{1}H NMR data of (2*S*,6*R*,7*S*,8*R*)-**1** (isomer 1), (2*R*,6*R*,7*S*,8*R*)-**1** (isomer 2), (2*R*,6*R*,7*S*,8*S*)-**1** (isomer 3) and (2*S*,6*R*,7*S*,8*S*)-**1** (isomer 4). For a better comparison Δδ values over 0.3 were marked with yellow and those over 0.6 with red.

Hydrogen	δ_{Exp} (ppm)	δ_{calc} isomer 1 (ppm)	δ_{calc} isomer 2 (ppm)	δ_{calc} isomer 3 (ppm)	δ_{calc} isomer 4 (ppm)	Δδ isomer 1 (ppm)	Δδ isomer 2 (ppm)	Δδ isomer 3 (ppm)	Δδ isomer 4 (ppm)
H-1a	2.02	2.25	2.37	2.32	2.27	0.23	0.35	0.30	0.25
H-1b	2.72	2.68	2.55	2.49	2.56	0.04	0.17	0.23	0.16
H-2	4.75	4.69	4.87	4.70	4.80	0.06	0.12	0.05	0.05
H-3	5.15	5.23	5.28	5.33	5.27	0.08	0.13	0.18	0.12
H-5a	2.76	2.91	2.62	2.78	2.95	0.15	0.14	0.02	0.19
H-5b	2.32	1.68	2.07	2.15	1.73	0.64	0.25	0.17	0.59
H-6	5.76	4.51	4.43	3.91	4.10	1.25	1.33	1.85	1.66
H-7	2.93	2.76	2.78	3.02	2.93	0.17	0.15	0.09	0.00
H-8	5.50	6.34	6.47	5.14	5.05	0.84	0.97	0.36	0.45
H-9	4.98	5.28	4.96	5.04	5.18	0.30	0.02	0.06	0.20
H-13a	6.30	6.50	6.53	6.53	6.50	0.20	0.23	0.23	0.20
H-13b	5.60	5.82	5.94	6.10	5.96	0.22	0.34	0.50	0.36
H-14 (3x)	1.79	1.66	1.89	1.94	1.88	0.13	0.10	0.15	0.09
H-15 (3x)	1.53	1.86	1.65	1.77	1.77	0.33	0.12	0.24	0.24
H-2'	2.16	1.99	2.02	2.14	2.13	0.17	0.14	0.02	0.03
H-2'	2.16	2.14	2.10	2.23	2.18	0.02	0.06	0.07	0.02
H-3'	2.05	1.96	1.98	2.23	2.20	0.09	0.07	0.18	0.15
H-4' (3x)	0.92	0.94	0.91	0.98	0.97	0.02	0.01	0.06	0.05
H-5' (3x)	0.90	0.91	0.92	0.96	0.95	0.01	0.02	0.06	0.05
CMAE	N/A	N/A	N/A	N/A	N/A	0.22	0.19	0.22	0.21

Table S21. Comparison of the experimental and the mPW1PW91/6-311+G(2d,p) SMD/$CHCl_3$ // ωB97XD/6-31+G(d,p) SMD/$CHCl_3$ ^{1}H NMR data of (2*S*,6*R*,7*S*,8*R*)-**1** (isomer 1), (2*R*,6*R*,7*S*,8*R*)-**1** (isomer 2), (2*R*,6*R*,7*S*,8*S*)-**1** (isomer 3) and (2*S*,6*R*,7*S*,8*S*)-**1** (isomer 4). For a better comparison Δδ values over 0.3 were marked with yellow and those over 0.6 with red.

Hydrogen	δ_{Exp} (ppm)	δ_{calc} isomer 1 (ppm)	δ_{calc} isomer 2 (ppm)	δ_{calc} isomer 3 (ppm)	δ_{calc} isomer 4 (ppm)	Δδ isomer 1 (ppm)	Δδ isomer 2 (ppm)	Δδ isomer 3 (ppm)	Δδ isomer 4 (ppm)
H-1a	2.02	2.17	2.45	2.56	2.12	0.15	0.43	0.16	0.10
H-1b	2.72	2.59	2.46	2.18	2.61	0.13	0.26	0.16	0.11
H-2	4.75	4.57	4.87	4.76	4.79	0.18	0.12	0.01	0.04
H-3	5.15	5.10	5.33	5.19	5.21	0.05	0.18	0.04	0.06
H-5a	2.76	2.81	2.60	2.54	2.93	0.05	0.16	0.22	0.17
H-5b	2.32	1.60	2.15	2.39	1.80	0.72	0.17	0.07	0.52
H-6	5.76	4.39	4.57	3.98	4.32	1.37	1.19	1.78	1.44
H-7	2.93	2.67	2.95	3.15	3.15	0.26	0.02	0.22	0.22
H-8	5.50	6.19	6.25	5.24	4.87	0.69	0.75	0.26	0.63
H-9	4.98	5.15	4.96	5.08	5.34	0.17	0.02	0.10	0.36
H-13a	6.30	6.35	6.46	6.51	6.47	0.05	0.16	0.21	0.17
H-13b	5.60	5.69	6.00	6.14	6.05	0.09	0.40	0.54	0.45
H-14 (3x)	1.79	1.58	1.86	1.78	1.84	0.21	0.07	0.01	0.05
H-15 (3x)	1.53	1.79	1.67	1.83	1.81	0.26	0.14	0.30	0.28
H-2'	2.16	1.91	2.08	2.18	2.17	0.25	0.08	0.02	0.01
H-2'	2.16	2.06	2.18	2.26	2.27	0.10	0.02	0.10	0.11
H-3'	2.05	1.88	1.95	2.16	2.17	0.17	0.10	0.11	0.12
H-4' (3x)	0.92	0.88	0.88	0.95	0.94	0.04	0.04	0.03	0.02
H-5' (3x)	0.90	0.85	0.89	0.92	0.92	0.05	0.01	0.02	0.02
CMAE	N/A	N/A	N/A	N/A	N/A	0.23	0.18	0.19	0.21

Table S22. Level dependency of the Boltzmann populations illustrated on the most likely isomer of **1** [(2*S*,6*R*,7*S*,8*R*)-**1,** isomer 1] computed at the mPW1PW91/6-311+G(2d,p) // B3LYP/6-31+G(d,p) (method 1), mPW1PW91/6-311+G(2d,p) SMD/$CHCl_3$ // B3LYP/6-31+G(d,p) (method 2), mPW1PW91/6-311+G(2d,p) SMD/$CHCl_3$ // mPW1PW91/6-311+G(2d,p) SMD/$CHCl_3$ (method 3), mPW1PW91/6-31G(d) SMD/$CHCl_3$ // M06-2X/6-31G(d) (method 4), M06/6-31G(d) // B3LYP/6-31+G(d,p) (method 5), mPW1PW91/6-311+G(2d,p) // ωB97XD/6-31+G(d,p) (method 6) and mPW1PW91/6-311+G(2d,p) SMD/$CHCl_3$ // ωB97XD/6-31+G(d,p) SMD/$CHCl_3$ (method 7). Only DFT conformers ≥ 1% Boltzmann distributions were considered in each case.

Conformers	method 1	method 2	method 3	method 4	method 5	method 6	method 7
Conf. A	24.7 %	24.7 %	13.9 %	26.3 %	24.7 %	41.1 %	39.0 %
Conf. B	21.9 %	21.9 %	13.2 %	12.6 %	21.9 %	13.5 %	11.9 %
Conf. C	10.8 %	10.8 %	12.4 %	9.2 %	10.8 %	7.4 %	9.8 %
Conf. D	7.4 %	7.4 %	9.1 %	4.9 %	7.4 %	5.1 %	6.3 %
Conf. E	4.7 %	4.7 %	6.7 %	4.3 %	4.7 %	5.1 %	5.2 %
Conf. F	4.4 %	4.4 %	4.5 %	4.2 %	4.4 %	4.3 %	4.1 %
Conf. G	3.0 %	3.0 %	4.4 %	4.0 %	3.0 %	3.9 %	4.0 %
Conf. H	3.0 %	3.0 %	4.3 %	3.7 %	3.0 %	3.1 %	3.4 %
Conf. I	2.6 %	2.6 %	4.0 %	3.4 %	2.6 %	2.6 %	2.9 %
Conf. J	2.6 %	2.6 %	3.6 %	3.2 %	2.6 %	2.4 %	2.6 %
Conf. K	2.0 %	2.0 %	3.5 %	3.2 %	2.0 %	2.2 %	2.4 %
Conf. L	1.7 %	1.7 %	3.2 %	2.3 %	1.7 %	2.0 %	2.2 %
Conf. M	1.7 %	1.7 %	3.0 %	1.4 %	1.7 %	1.0 %	1.2 %
Conf. N	1.6 %	1.6 %	2.5 %	1.4 %	1.6 %	1.0 %	1.0 %
Conf. O	1.3 %	1.3 %	2.3 %	1.4 %	1.3 %		1.0 %
Conf. P	1.3 %	1.3 %	1.9 %	1.1 %	1.3 %		
Conf. Q				1.0 %			
Conf. R				1.0 %			

Table S23. Conformer dependency of the ^{13}C chemical shifts at the mPW1PW91/6-311+G(2d,p) // B3LYP/6-31+G(d,p) level, illustrated on the most likely isomer of **1** [(2*S*,6*R*,7*S*,8*R*)-**1,** isomer 1]. For simplicity only the first 6 conformers were included.

Carbon	δ_{Exp} (ppm)	δ_{calc} Conf. A (ppm)	δ_{calc} Conf. B (ppm)	δ_{calc} Conf. C (ppm)	δ_{calc} Conf. D (ppm)	δ_{calc} Conf. E (ppm)	δ_{calc} Conf. F (ppm)
C-1	48.60	46.71	46.71	46.69	46.66	47.62	47.57
C-2	69.20	67.98	67.93	68.05	67.98	71.21	71.23
C-3	133.90	138.21	138.12	138.14	138.23	131.00	131.23
C-4	135.10	137.04	137.12	137.19	136.96	144.54	144.45
C-5	44.00	40.78	40.68	40.87	40.74	47.57	47.62
C-6	70.80	77.36	77.29	77.30	77.43	75.41	75.37
C-7	52.90	50.26	50.03	50.30	50.04	52.46	52.43
C-8	75.30	70.61	71.18	70.31	71.14	69.56	69.78
C-9	129.30	131.75	131.78	131.41	132.00	130.77	130.76
C-10	142.70	141.06	141.22	141.15	141.17	140.92	141.02
C-11	136.40	138.76	139.21	138.51	139.24	138.89	139.21
C-12	169.40	168.11	168.09	168.15	168.39	167.67	167.60
C-13	121.20	126.12	125.56	126.33	125.77	125.75	125.13
C-14	18.70	17.51	17.50	17.51	17.56	16.95	16.87
C-15	20.00	21.37	21.32	21.34	21.40	18.28	18.28
C-1'	171.80	173.35	173.02	172.89	172.96	173.36	173.06
C-2'	43.30	41.90	41.95	43.77	44.35	41.96	42.13
C-3'	25.40	26.71	26.39	29.86	29.86	26.72	26.46
C-4'	22.30	19.61	21.26	22.07	19.52	19.57	21.27
C-5'	22.30	21.25	19.30	19.47	22.53	21.26	19.39

Table S24. Conformer dependency of the ^{13}C chemical shifts at the mPW1PW91/6-311+G(2d,p) SMD/$CHCl_3$ // B3LYP/6-31+G(d,p) level, illustrated on the most likely isomer of **1** [(2*S*,6*R*,7*S*,8*R*)-**1,** isomer 1]. For simplicity only the first 6 conformers were included.

Carbon	δ_{Exp} (ppm)	δ_{calc} Conf. A (ppm)	δ_{calc} Conf. B (ppm)	δ_{calc} Conf. C (ppm)	δ_{calc} Conf. D (ppm)	δ_{calc} Conf. E (ppm)	δ_{calc} Conf. F (ppm)
C-1	48.60	46.27	46.19	46.37	46.48	46.94	47.12
C-2	69.20	67.34	66.86	67.55	67.64	70.32	70.61
C-3	133.90	135.57	135.54	135.23	135.14	129.56	129.91
C-4	135.10	135.90	135.36	136.15	135.65	142.09	142.53
C-5	44.00	40.53	40.99	40.49	40.82	46.44	46.58
C-6	70.80	77.61	77.75	77.27	77.32	76.15	75.63
C-7	52.90	50.06	49.87	50.34	50.02	52.09	52.22
C-8	75.30	70.62	71.26	70.54	71.11	69.54	69.81
C-9	129.30	128.85	129.16	127.97	128.91	128.03	127.43
C-10	142.70	140.62	140.82	141.17	141.22	140.27	140.94
C-11	136.40	137.71	137.15	136.98	136.62	136.99	136.42
C-12	169.40	168.57	168.37	168.49	168.50	168.04	167.89
C-13	121.20	126.66	125.86	126.26	126.18	125.04	125.16
C-14	18.70	17.50	17.42	17.46	17.44	17.02	16.98
C-15	20.00	21.19	21.14	21.27	21.35	18.50	18.51
C-1'	171.80	172.61	172.29	172.46	172.37	172.56	172.26
C-2'	43.30	42.09	42.19	44.04	44.31	42.05	42.33
C-3'	25.40	27.03	26.67	30.53	31.41	27.20	26.75
C-4'	22.30	19.41	20.81	21.76	19.42	19.48	20.86
C-5'	22.30	20.96	19.24	19.25	22.14	20.94	19.25

Table S25. Conformer dependency of the ^{13}C chemical shifts at the mPW1PW91/6-311+G(2d,p) SMD/$CHCl_3$ // mPW1PW91/6-311+G(2d,p) SMD/$CHCl_3$ level, illustrated on the most likely isomer of **1** [(2*S*,6*R*,7*S*,8*R*)-**1,** isomer 1]. For simplicity only the first 6 conformers were included.

Carbon	δ_{Exp} (ppm)	δ_{calc} Conf. A (ppm)	δ_{calc} Conf. B (ppm)	δ_{calc} Conf. C (ppm)	δ_{calc} Conf. D (ppm)	δ_{calc} Conf. E (ppm)	δ_{calc} Conf. F (ppm)
C-1	48.60	47.12	47.08	47.32	47.08	47.28	50.42
C-2	69.20	67.03	67.55	67.21	67.32	67.49	67.76
C-3	133.90	137.60	137.69	137.41	137.20	137.63	136.85
C-4	135.10	136.38	136.66	136.63	136.44	136.44	135.85
C-5	44.00	41.37	41.13	41.62	41.09	41.29	41.17
C-6	70.80	78.60	78.44	78.51	78.27	78.32	78.59
C-7	52.90	49.45	49.83	49.75	49.88	49.39	49.41
C-8	75.30	72.99	71.08	71.70	72.10	74.31	73.14
C-9	129.30	130.56	130.33	130.69	130.08	131.15	130.08
C-10	142.70	142.86	142.29	142.68	142.98	142.63	142.57
C-11	136.40	139.75	139.38	138.71	138.76	139.44	139.78
C-12	169.40	169.88	169.92	169.81	169.81	170.05	169.88
C-13	121.20	127.77	128.27	128.11	127.05	128.19	127.82
C-14	18.70	18.04	18.07	18.07	17.96	17.94	17.77
C-15	20.00	21.77	22.02	21.76	22.09	21.75	22.01
C-1'	171.80	173.47	173.72	173.45	173.54	173.64	173.45
C-2'	43.30	43.17	42.76	43.53	45.07	45.62	43.17
C-3'	25.40	27.07	27.00	26.93	29.67	30.33	27.08
C-4'	22.30	22.15	20.57	22.08	22.70	20.54	22.16
C-5'	22.30	20.48	22.09	20.40	20.35	23.10	20.48

Table S26. Conformer dependency of the ^{13}C chemical shifts at the mPW1PW91/6-31G(d) SMD/$CHCl_3$ // M06-2X/6-31G(d) level, illustrated on the most likely isomer of **1** [(2*S*,6*R*,7*S*,8*R*)-**1,** isomer 1]. For simplicity only the first 6 conformers were included.

Carbon	δ_{Exp} (ppm)	δ_{calc} Conf. A (ppm)	δ_{calc} Conf. B (ppm)	δ_{calc} Conf. C (ppm)	δ_{calc} Conf. D (ppm)	δ_{calc} Conf. E (ppm)	δ_{calc} Conf. F (ppm)
C-1	48.60	47.38	48.71	47.41	47.36	48.56	47.33
C-2	69.20	67.74	70.47	67.57	67.90	70.60	67.76
C-3	133.90	137.47	130.52	137.44	137.65	130.65	137.56
C-4	135.10	134.37	143.10	134.21	134.15	142.87	134.22
C-5	44.00	41.50	47.48	41.09	41.76	47.71	41.71
C-6	70.80	78.84	76.24	79.22	78.68	76.10	78.41
C-7	52.90	49.54	51.87	49.63	50.41	51.92	49.53
C-8	75.30	72.19	71.14	73.16	71.18	71.25	72.50
C-9	129.30	130.72	128.64	129.65	129.53	128.58	130.48
C-10	142.70	139.68	139.74	140.06	140.06	139.68	139.57
C-11	136.40	135.75	135.82	135.59	135.32	135.89	135.72
C-12	169.40	168.85	168.22	169.05	168.84	168.15	168.79
C-13	121.20	132.10	132.17	131.98	131.86	132.93	132.97
C-14	18.70	18.90	18.27	18.80	18.59	18.20	18.70
C-15	20.00	22.79	19.16	22.81	22.95	19.21	22.98
C-1'	171.80	172.32	172.32	172.64	173.44	171.70	172.04
C-2'	43.30	44.61	44.36	42.29	43.11	42.42	42.42
C-3'	25.40	28.62	28.84	24.93	28.14	26.56	26.82
C-4'	22.30	21.99	20.99	22.71	24.70	21.72	21.37
C-5'	22.30	23.61	23.52	20.01	23.96	20.14	20.34

Table S27. Conformer dependency of the ^{13}C chemical shifts at the M06/6-31G(d) // B3LYP/6-31+G(d,p) level, illustrated on the most likely isomer of **1** [(2*S*,6*R*,7*S*,8*R*)-**1,** isomer 1]. For simplicity only the first 6 conformers were included.

Carbon	δ_{Exp} (ppm)	δ_{calc} Conf. A (ppm)	δ_{calc} Conf. B (ppm)	δ_{calc} Conf. C (ppm)	δ_{calc} Conf. D (ppm)	δ_{calc} Conf. E (ppm)	δ_{calc} Conf. F (ppm)
C-1	48.60	45.96	45.57	45.43	45.72	46.58	47.41
C-2	69.20	66.08	65.53	66.99	66.62	70.48	70.57
C-3	133.90	136.68	136.74	136.84	136.68	130.33	130.50
C-4	135.10	133.87	134.39	134.40	134.34	140.56	140.67
C-5	44.00	40.06	39.82	39.84	38.92	46.32	46.16
C-6	70.80	77.06	77.01	76.87	77.82	74.67	74.70
C-7	52.90	47.91	47.63	48.13	47.30	49.97	49.75
C-8	75.30	70.12	70.93	70.42	71.05	68.80	69.29
C-9	129.30	131.63	131.67	131.54	131.66	130.70	130.77
C-10	142.70	137.05	137.09	137.50	137.19	137.33	137.40
C-11	136.40	135.43	136.04	135.13	135.55	135.73	135.67
C-12	169.40	166.42	166.64	166.53	166.38	166.55	166.02
C-13	121.20	128.32	127.60	128.28	127.37	128.88	127.46
C-14	18.70	17.52	17.14	17.89	17.17	17.42	17.01
C-15	20.00	21.92	21.72	21.86	21.90	17.34	17.99
C-1'	171.80	171.17	170.11	171.31	170.59	171.18	170.43
C-2'	43.30	39.60	40.36	41.26	43.28	39.80	40.30
C-3'	25.40	23.11	23.53	26.39	24.91	22.76	22.50
C-4'	22.30	20.05	21.41	21.77	20.71	20.03	22.04
C-5'	22.30	22.13	19.80	18.78	23.69	21.74	19.54

Table S28. Conformer dependency of the ^{13}C chemical shifts at the mPW1PW91/6-311+G(2d,p) // ωB97XD/6-31+G(d,p) level, illustrated on the most likely isomer of **1** [(2*S*,6*R*,7*S*,8*R*)-**1,** isomer 1]. For simplicity only the first 6 conformers were included.

Carbon	δ_{Exp} (ppm)	δ_{calc} Conf. A (ppm)	δ_{calc} Conf. B (ppm)	δ_{calc} Conf. C (ppm)	δ_{calc} Conf. D (ppm)	δ_{calc} Conf. E (ppm)	δ_{calc} Conf. F (ppm)
C-1	48.60	46.64	46.82	47.54	46.38	46.52	46.30
C-2	69.20	65.99	66.32	69.48	66.32	66.04	66.07
C-3	133.90	137.14	137.12	128.54	136.91	137.06	137.51
C-4	135.10	133.67	133.82	144.73	134.07	133.87	133.65
C-5	44.00	40.84	41.07	47.05	41.27	41.24	41.43
C-6	70.80	76.20	76.09	73.66	76.25	75.90	76.78
C-7	52.90	49.05	48.98	50.37	49.29	48.89	48.75
C-8	75.30	69.10	69.22	68.03	68.02	68.90	69.04
C-9	129.30	130.62	129.79	128.64	129.87	130.46	129.93
C-10	142.70	140.38	140.64	139.82	140.17	140.15	139.76
C-11	136.40	137.16	136.10	136.68	136.29	136.22	136.43
C-12	169.40	166.13	165.77	165.55	166.34	166.04	165.88
C-13	121.20	127.26	127.30	128.40	128.28	128.55	126.59
C-14	18.70	18.01	17.92	17.25	17.91	17.95	17.96
C-15	20.00	21.94	22.00	18.90	22.51	22.20	22.15
C-1'	171.80	170.43	171.37	170.64	171.86	170.62	171.34
C-2'	43.30	44.38	43.20	44.41	43.34	42.61	42.38
C-3'	25.40	30.51	28.32	29.68	29.33	29.47	26.27
C-4'	22.30	20.26	22.08	20.02	24.33	21.26	20.19
C-5'	22.30	22.98	20.55	22.91	22.75	19.41	21.77

Table S29. Conformer dependency of the ^{13}C chemical shifts at the mPW1PW91/6-311+G(2d,p) SMD/$CHCl_3$ // ωB97XD/6-31+G(d,p) SMD/$CHCl_3$ level, illustrated on the most likely isomer of **1** [(2*S*,6*R*,7*S*,8*R*)-**1,** isomer 1]. For simplicity only the first 6 conformers were included.

Carbon	δ_{Exp} (ppm)	δ_{calc} Conf. A (ppm)	δ_{calc} Conf. B (ppm)	δ_{calc} Conf. C (ppm)	δ_{calc} Conf. D (ppm)	δ_{calc} Conf. E (ppm)	δ_{calc} Conf. F (ppm)
C-1	48.60	46.46	46.27	46.55	46.33	46.16	46.16
C-2	69.20	65.98	66.32	66.16	65.99	66.08	66.02
C-3	133.90	135.04	135.08	135.18	135.04	134.88	135.60
C-4	135.10	133.59	133.77	133.88	133.73	133.77	133.84
C-5	44.00	40.56	40.57	40.74	40.50	40.25	40.84
C-6	70.80	77.50	77.53	77.77	77.73	77.86	78.33
C-7	52.90	48.97	49.14	49.00	48.75	48.09	48.67
C-8	75.30	71.52	71.29	70.80	70.53	74.08	70.39
C-9	129.30	127.91	127.43	126.96	127.52	128.97	127.65
C-10	142.70	141.71	141.69	141.68	141.40	141.63	140.54
C-11	136.40	136.83	136.16	135.01	136.07	137.25	136.03
C-12	169.40	167.30	167.20	167.17	167.38	167.38	167.39
C-13	121.20	126.67	126.79	126.30	125.78	123.77	127.05
C-14	18.70	18.13	18.23	18.11	18.12	18.23	18.13
C-15	20.00	21.79	21.85	21.85	21.99	21.96	21.98
C-1'	171.80	171.08	171.71	171.22	170.95	171.29	171.49
C-2'	43.30	44.78	43.66	42.72	44.32	44.02	42.69
C-3'	25.40	30.78	29.43	27.69	30.19	29.31	26.87
C-4'	22.30	20.50	22.14	21.75	22.05	21.90	20.07
C-5'	22.30	22.53	20.50	20.11	19.94	20.22	21.68

Table S30. Cartesian coordinates and energies of the low-energy conformers calculated at the B3LYP/6-31G(d) level.

(1*R*,5*S*,6*S*,7*S*,10*R*)-**2**, Conf. A

C	3.472530	0.017669	-0.242346
C	2.942188	1.404164	-0.660815
C	1.586107	1.670814	-0.045274
C	0.569591	0.587712	-0.380103
C	1.060017	-0.787333	0.190201
C	2.438006	-1.083289	-0.481842
C	-0.893396	0.928380	-0.031987
C	-1.857047	-0.195733	-0.455209
C	-1.406687	-1.542158	0.137765
C	0.045695	-1.876773	-0.231440
C	-3.344637	0.161691	-0.178109
C	-4.301665	-0.791231	-0.913032
C	-3.707560	0.241514	1.314146
O	-1.310047	2.104836	-0.730084
C	1.366406	2.723577	0.753381
O	3.028166	-2.300324	-0.026548
H	0.582876	0.483660	-1.478969
C	1.211624	-0.773571	1.724540
H	3.745857	0.024840	0.819023
H	4.382993	-0.228165	-0.800519
H	3.658611	2.184094	-0.380186
H	2.851237	1.432402	-1.757525
H	2.255295	-1.150345	-1.569810
H	-0.978698	1.090184	1.054784
H	-1.758167	-0.265660	-1.550338
H	-2.059475	-2.344534	-0.225468
H	-1.520055	-1.530849	1.228650
H	0.105781	-2.014825	-1.322310
H	0.323259	-2.836921	0.226304
H	-3.489978	1.161108	-0.603909
H	-4.261779	-1.810058	-0.507992
H	-5.338063	-0.446054	-0.817772
H	-4.067177	-0.846678	-1.983197
H	-4.742414	0.583910	1.432846
H	-3.068443	0.946331	1.857698
H	-3.631190	-0.734451	1.809572
H	-0.603786	2.763476	-0.634209
H	0.414662	2.905684	1.246061
H	2.154634	3.442895	0.962539
H	2.451750	-3.029593	-0.301746
H	1.718479	-1.687299	2.049944
H	1.799177	0.081007	2.070220
H	0.242640	-0.722855	2.230103

B3LYP energy = -737.679988196 a.u.

(1*R*,5*S*,6*S*,7*S*,10*R*)-**2**, Conf. B

C	3.471139	0.025713	-0.260205
C	2.935777	1.414311	-0.667956
C	1.585193	1.669399	-0.037006
C	0.569818	0.589215	-0.382021
C	1.053843	-0.794725	0.172444
C	2.439171	-1.090011	-0.482993
C	-0.894148	0.927508	-0.032604
C	-1.860464	-0.192958	-0.460650
C	-1.405267	-1.549582	0.107469
C	0.044742	-1.875651	-0.275486
C	-3.345407	0.163201	-0.167191
C	-4.309130	-0.779071	-0.907057
C	-3.696979	0.222541	1.328836
O	-1.308787	2.109142	-0.723502
C	1.371128	2.708333	0.781015
O	2.970147	-2.359565	-0.103137
H	0.584677	0.493798	-1.481264
C	1.177178	-0.791294	1.710659
H	3.758575	0.049639	0.800710
H	4.377963	-0.217629	-0.825875
H	3.652985	2.195124	-0.391366
H	2.834594	1.444416	-1.763376
H	2.267203	-1.187914	-1.563326
H	-0.979719	1.085065	1.054726
H	-1.770234	-0.247988	-1.557284
H	-2.059251	-2.344719	-0.269401
H	-1.518326	-1.560045	1.198908
H	0.104225	-1.975954	-1.369416
H	0.347030	-2.844726	0.136867
H	-3.493716	1.168527	-0.578048
H	-4.266810	-1.802906	-0.515379
H	-5.344475	-0.434027	-0.799836
H	-4.082280	-0.821230	-1.979429
H	-4.731058	0.562765	1.460369
H	-3.054250	0.920307	1.877509
H	-3.616040	-0.760162	1.809886
H	-0.607288	2.770463	-0.612654
H	0.422235	2.881742	1.282232
H	2.159538	3.426113	0.995052
H	3.294379	-2.280697	0.807844
H	1.850047	-0.008568	2.072703
H	0.211092	-0.628431	2.196168
H	1.548454	-1.761142	2.063275

B3LYP energy = -737.679638075 a.u.

(1*R*,5*S*,6*S*,7*S*,10*R*)-**2**, Conf. C

C	3.467192	0.024608	-0.234428
C	2.940498	1.410593	-0.661072
C	1.584649	1.671424	-0.042888
C	0.570628	0.587972	-0.383369
C	1.055297	-0.793036	0.180401
C	2.432272	-1.084199	-0.475414
C	-0.892918	0.925975	-0.033505
C	-1.858301	-0.195513	-0.460356
C	-1.404498	-1.549161	0.114662

C	0.046673	-1.879167	-0.260720
C	-3.343989	0.161714	-0.171905
C	-4.306006	-0.784255	-0.909349
C	-3.698950	0.228314	1.322993
O	-1.308878	2.106215	-0.726993
C	1.366779	2.718698	0.763351
O	2.899268	-2.340724	0.017431
H	0.584226	0.489024	-1.482321
C	1.187828	-0.784539	1.718166
H	3.735504	0.036211	0.828089
H	4.386430	-0.210001	-0.790552
H	3.656293	2.192138	-0.383200
H	2.848759	1.433873	-1.757734
H	2.258234	-1.160049	-1.564092
H	-0.977677	1.084783	1.053531
H	-1.765488	-0.254251	-1.556687
H	-2.057227	-2.346389	-0.260153
H	-1.520300	-1.553983	1.205605
H	0.109349	-1.991748	-1.353577
H	0.345180	-2.842305	0.166877
H	-3.491735	1.164987	-0.587953
H	-4.264398	-1.806163	-0.512623
H	-5.341690	-0.438867	-0.806147
H	-4.076740	-0.831599	-1.981048
H	-4.733458	0.568904	1.450548
H	-3.057396	0.928812	1.869418
H	-3.618583	-0.751885	1.809143
H	-0.605515	2.766442	-0.622285
H	0.415947	2.896498	1.259191
H	2.154755	3.437531	0.975405
H	3.746313	-2.529349	-0.415165
H	1.809868	0.042030	2.072703
H	0.214002	-0.682937	2.206018
H	1.639301	-1.722205	2.054106

B3LYP energy = -737.679373197 a.u.

(1*R*,5*S*,6*S*,7*S*,10*R*)-**2**, Conf. D

C	3.421318	0.429837	-0.263698
C	2.672649	1.733261	-0.608736
C	1.305456	1.752310	0.039300
C	0.462714	0.542963	-0.343871
C	1.173328	-0.764849	0.149461
C	2.568511	-0.807263	-0.549240
C	-1.029052	0.632290	0.039475
C	-1.818761	-0.599827	-0.438328
C	-1.145497	-1.888944	0.069974
C	0.335137	-1.976251	-0.321591
C	-3.338486	-0.583265	-0.083840
C	-3.635702	-0.099422	1.345174
C	-4.184098	0.196730	-1.104136
O	-1.627465	1.780791	-0.568232
C	0.947750	2.708392	0.906898
O	3.349291	-1.937158	-0.161831
H	0.473162	0.498546	-1.446777
C	1.345671	-0.807651	1.681286
H	3.705592	0.430297	0.794823
H	4.350357	0.357851	-0.840381
H	3.263850	2.602324	-0.299676
H	2.555618	1.796808	-1.701421
H	2.379817	-0.848809	-1.637352
H	-1.114367	0.705801	1.135013
H	-1.742621	-0.601606	-1.536883
H	-1.677919	-2.755041	-0.343058
H	-1.252333	-1.962883	1.160212
H	0.401419	-2.048310	-1.418548
H	0.763224	-2.903910	0.084254
H	-3.663337	-1.633794	-0.141701
H	-3.049384	-0.639658	2.098667
H	-3.423480	0.970396	1.449564
H	-4.694747	-0.251569	1.584493
H	-3.918301	1.256470	-1.106471
H	-5.251852	0.103807	-0.868494
H	-4.033120	-0.190927	-2.119264
H	-1.020521	2.526817	-0.435838
H	-0.005977	2.709168	1.428530
H	1.621471	3.527546	1.146489
H	2.891651	-2.733539	-0.471776
H	2.004542	-1.638854	1.950558
H	1.786479	0.113573	2.071277
H	0.390543	-0.949438	2.195499

B3LYP energy = -737.678973266 a.u.

(1*R*,5*S*,6*S*,7*S*,10*R*)-**2**, Conf. E

C	3.418671	0.435656	-0.281763
C	2.666124	1.740947	-0.614133
C	1.305855	1.749668	0.047710
C	0.463219	0.544176	-0.345535
C	1.168274	-0.772739	0.131560
C	2.570001	-0.815679	-0.551647
C	-1.029648	0.631417	0.037926
C	-1.821953	-0.597099	-0.445704
C	-1.144372	-1.893195	0.041826
C	0.332320	-1.974035	-0.362365
C	-3.339279	-0.582078	-0.081916
C	-3.627773	-0.114884	1.354545
C	-4.191173	0.210419	-1.087328
O	-1.625688	1.785208	-0.562308
C	0.955982	2.692940	0.932357
O	3.298308	-2.003347	-0.240624
H	0.474303	0.508643	-1.448386
C	1.317057	-0.827696	1.666394
H	3.714739	0.452970	0.776960
H	4.343276	0.365700	-0.866275
H	3.258622	2.610162	-0.307598
H	2.539176	1.806578	-1.705243
H	2.396389	-0.886988	-1.633658
H	-1.116164	0.699673	1.133709
H	-1.752774	-0.587351	-1.544609
H	-1.680083	-2.752366	-0.381431
H	-1.248289	-1.984385	1.131646
H	0.392328	-2.011943	-1.460301
H	0.784801	-2.903771	0.000143

H	-3.665135	-1.631562	-0.149892
H	-4.685237	-0.270070	1.598950
H	-3.036574	-0.663925	2.097870
H	-3.415453	0.953862	1.470240
H	-5.257574	0.114757	-0.846413
H	-4.046410	-0.164970	-2.107910
H	-3.925451	1.270208	-1.078859
H	-1.023631	2.532045	-0.413523
H	0.006099	2.686166	1.460976
H	1.630425	3.510187	1.176661
H	3.618864	-1.919518	0.671226
H	0.346312	-0.840567	2.169472
H	1.838697	-1.745229	1.964649
H	1.867952	0.029883	2.063480

B3LYP energy = -737.678639960 a.u.

(1*R*,5*S*,6*S*,7*S*,10*R*)-**2**, Conf. F

C	3.414911	0.434219	-0.258363
C	2.669771	1.738670	-0.609424
C	1.304533	1.751995	0.042519
C	0.463855	0.543092	-0.345727
C	1.169631	-0.771295	0.140727
C	2.562093	-0.810581	-0.543635
C	-1.028458	0.630363	0.037987
C	-1.819560	-0.599412	-0.443809
C	-1.143342	-1.893109	0.050470
C	0.335058	-1.977786	-0.347101
C	-3.337909	-0.583224	-0.083892
C	-3.630267	-0.108500	1.349221
C	-4.187531	0.203351	-1.095842
O	-1.625495	1.782232	-0.566173
C	0.950472	2.702598	0.917515
O	3.229220	-2.000391	-0.119877
H	0.473945	0.503574	-1.448436
C	1.326969	-0.821254	1.675198
H	3.696215	0.437978	0.800849
H	4.349441	0.374459	-0.834783
H	3.260937	2.608718	-0.302950
H	2.551286	1.798407	-1.702054
H	2.381450	-0.860500	-1.632580
H	-1.114532	0.701268	1.133451
H	-1.747684	-0.593079	-1.542650
H	-1.677648	-2.754256	-0.370782
H	-1.250312	-1.979377	1.140139
H	0.399599	-2.026570	-1.444647
H	0.783312	-2.903203	0.030230
H	-3.663205	-1.633221	-0.146976
H	-4.688418	-0.262457	1.591652
H	-3.040764	-0.653529	2.096700
H	-3.418140	0.960810	1.459853
H	-5.254592	0.109042	-0.857076
H	-4.040175	-0.177846	-2.113944
H	-3.921826	1.263172	-1.092644
H	-1.022438	2.529204	-0.422599
H	-0.001103	2.699065	1.442941
H	1.624319	3.521040	1.159419
H	4.086821	-2.032541	-0.571136
H	0.358351	-0.899497	2.177293
H	1.925200	-1.692766	1.955235
H	1.818801	0.072609	2.068816

B3LYP energy = -737.678381843 a.u.

(1*R*,5*S*,6*S*,7*S*,10*R*)-**2**, Conf. G

C	3.485647	-0.013122	-0.188568
C	2.982295	1.374511	-0.622029
C	1.609206	1.684229	-0.060578
C	0.577578	0.609516	-0.384317
C	1.054454	-0.780463	0.173709
C	2.441959	-1.090691	-0.471059
C	-0.884351	0.933172	-0.000667
C	-1.855571	-0.183571	-0.454312
C	-1.407005	-1.550102	0.090750
C	0.048102	-1.866191	-0.275389
C	-3.340363	0.163147	-0.152616
C	-4.305301	-0.725996	-0.953721
C	-3.700684	0.134254	1.342417
O	-1.297027	2.198990	-0.526180
C	1.382134	2.785521	0.661876
O	2.996591	-2.327416	-0.022615
H	0.579004	0.497355	-1.486736
C	1.180477	-0.773835	1.710857
H	3.722123	-0.014102	0.881637
H	4.410521	-0.271549	-0.716915
H	3.699205	2.148408	-0.326407
H	2.930386	1.398544	-1.722739
H	2.285140	-1.136761	-1.564521
H	-0.953200	1.060790	1.085230
H	-1.766555	-0.226592	-1.554763
H	-2.056402	-2.341937	-0.301844
H	-1.527664	-1.573075	1.180246
H	0.118368	-1.983512	-1.368399
H	0.327691	-2.833124	0.165962
H	-3.476308	1.194045	-0.499901
H	-4.081289	-0.696165	-2.027362
H	-4.261508	-1.774191	-0.632798
H	-5.340674	-0.390234	-0.821278
H	-4.725240	0.495332	1.490280
H	-3.041909	0.774650	1.939638
H	-3.651815	-0.880089	1.756934
H	-1.057201	2.217884	-1.466875
H	0.400773	3.047800	1.039202
H	2.188278	3.485902	0.870604
H	2.422161	-3.042101	-0.336667
H	1.741574	0.094078	2.067409
H	0.203618	-0.748586	2.203113
H	1.702956	-1.677632	2.039189

B3LYP energy = -737.677599469 a.u.

(1*R*,5*S*,6*S*,7*S*,10*R*)-**2**, Conf. H

C	3.484783	-0.006220	-0.204953
C	2.977060	1.384433	-0.626286
C	1.608451	1.683664	-0.049735

C	0.578160	0.612414	-0.385484
C	1.048101	-0.786347	0.155780
C	2.442076	-1.096271	-0.473534
C	-0.885388	0.934131	-0.003378
C	-1.858825	-0.180572	-0.458397
C	-1.404636	-1.556847	0.060175
C	0.047939	-1.863097	-0.321029
C	-3.340719	0.163854	-0.139229
C	-4.313216	-0.714190	-0.943439
C	-3.687460	0.113486	1.358474
O	-1.294196	2.202238	-0.526797
C	1.385794	2.772113	0.693029
O	2.935448	-2.385288	-0.105257
H	0.583211	0.510074	-1.488366
C	1.146646	-0.792022	1.695968
H	3.734107	0.006923	0.865910
H	4.406835	-0.261102	-0.740678
H	3.695561	2.158179	-0.333786
H	2.915388	1.411490	-1.726057
H	2.294076	-1.170288	-1.559763
H	-0.956005	1.063380	1.082061
H	-1.779582	-0.209735	-1.560249
H	-2.055540	-2.340433	-0.346305
H	-1.523945	-1.603290	1.149535
H	0.116660	-1.939659	-1.416723
H	0.354943	-2.838468	0.071765
H	-3.480148	1.199644	-0.470677
H	-5.347480	-0.380964	-0.796327
H	-4.099336	-0.669160	-2.018593
H	-4.265145	-1.766709	-0.637992
H	-3.023493	0.745575	1.959032
H	-3.633761	-0.906852	1.757007
H	-4.710746	0.471869	1.521217
H	-1.103783	2.199475	-1.479079
H	0.406334	3.027089	1.080349
H	2.191738	3.471033	0.907574
H	3.275841	-2.319626	0.800742
H	1.550338	-1.749095	2.047505
H	1.782628	0.015024	2.070785
H	0.168683	-0.671156	2.169785

B3LYP energy = -737.677535017 a.u.

(1*R*,5*S*,6*S*,7*S*,10*R*)-**2**, Conf. I

C	3.480204	-0.008381	-0.175414
C	2.983166	1.379399	-0.617207
C	1.608874	1.685341	-0.056757
C	0.579313	0.610999	-0.387529
C	1.048935	-0.785674	0.162018
C	2.435499	-1.090719	-0.465320
C	-0.883252	0.932260	-0.002528
C	-1.856728	-0.181690	-0.458717
C	-1.404317	-1.556952	0.062737
C	0.049404	-1.866631	-0.311405
C	-3.339027	0.163626	-0.142242
C	-4.310557	-0.715453	-0.946519
C	-3.688072	0.115759	1.355000
O	-1.293755	2.200688	-0.524942
C	1.382616	2.783669	0.670275
O	2.862208	-2.368919	0.010052
H	0.581733	0.505575	-1.490147
C	1.153844	-0.787620	1.702354
H	3.708575	-0.006419	0.896497
H	4.415397	-0.255443	-0.699329
H	3.699810	2.153909	-0.322519
H	2.933142	1.398670	-1.717981
H	2.288565	-1.140267	-1.560106
H	-0.951613	1.059584	1.083189
H	-1.775759	-0.211403	-1.560520
H	-2.054116	-2.341279	-0.344271
H	-1.526646	-1.600939	1.151608
H	0.122184	-1.952366	-1.406403
H	0.351647	-2.837799	0.094145
H	-3.477839	1.198896	-0.475534
H	-4.095196	-0.671994	-2.021491
H	-4.262932	-1.767528	-0.639473
H	-5.345043	-0.381964	-0.801305
H	-4.711379	0.475160	1.515666
H	-3.024298	0.748065	1.955434
H	-3.635547	-0.903927	1.755272
H	-1.098257	2.200628	-1.476165
H	0.401072	3.044803	1.047692
H	2.188777	3.483103	0.882464
H	3.739027	-2.541863	-0.366103
H	1.742652	0.057160	2.070809
H	0.170271	-0.720743	2.176299
H	1.628737	-1.713373	2.038923

B3LYP energy = -737.677297250 a.u.

Table S31. Cartesian coordinates and energies of the low-energy conformers calculated at the B3LYP/6-31+G(d,p) *in vacuo* level.

(2*S*,6*R*,7*S*,8*R*)-**1**, Conf. A

C	-3.935987	-1.724823	-0.476646
C	-3.413579	-0.419720	-1.013984
C	-3.402810	0.779099	-0.404271
C	-4.180571	1.122314	0.844962
C	-1.698111	-1.975043	0.703200
C	-2.746581	-2.676002	-0.137067
C	-0.995375	1.514677	-0.853179
C	-0.455115	1.117728	0.557584
C	0.218364	-0.287516	0.625437
C	-0.684325	-1.356416	0.075929
O	-0.226527	2.670883	-1.280109
C	0.605099	3.134250	-0.306823
C	0.481949	2.252969	0.892972
O	1.302826	4.109698	-0.458236
C	1.136154	2.510414	2.028395
C	-2.493048	1.887861	-0.924382
C	-1.971970	-1.879544	2.183535
O	-4.728879	-2.439172	-1.436485
O	1.420067	-0.243680	-0.187413
C	2.603891	-0.580064	0.394117
O	2.705968	-0.935554	1.550184
C	3.748912	-0.405379	-0.581134
C	4.996882	-1.240933	-0.248302
C	4.732406	-2.743808	-0.427393
C	6.184177	-0.781350	-1.107550
H	-4.527641	-1.561870	0.433062
H	-2.845197	-0.531838	-1.938852
H	-4.846324	0.322737	1.175166
H	-4.793646	2.014255	0.662184
H	-2.318388	-3.000809	-1.091352
H	-3.153600	-3.563407	0.361042
H	-3.515102	1.377365	1.680230
H	-1.282009	1.079325	1.273166
H	0.516974	-0.479241	1.658057
H	-0.579496	-1.507101	-0.996699
H	1.055626	1.871880	2.903054
H	1.783996	3.379943	2.090696
H	-2.644969	2.805315	-0.344100
H	-0.779046	0.734045	-1.584163
H	-2.722775	2.136280	-1.966906
H	-2.136255	-2.880252	2.601352
H	-2.885232	-1.304151	2.385403
H	-1.153167	-1.416583	2.739492
H	-5.449723	-1.864388	-1.726784
H	3.386870	-0.616798	-1.593861
H	3.995788	0.665714	-0.565882
H	5.239966	-1.061451	0.806252
H	5.621132	-3.328661	-0.166117
H	3.911268	-3.086048	0.210031
H	4.477847	-2.974560	-1.470037
H	7.085885	-1.353458	-0.863658
H	6.408177	0.279973	-0.950740
H	5.979230	-0.925899	-2.175962

B3LYP energy = -1155.15771223 a.u.

(2*S*,6*R*,7*S*,8*R*)-**1**, Conf. B

C	-4.165774	-1.393181	-0.438742
C	-3.507444	-0.149376	-0.972358
C	-3.344000	1.031116	-0.348967
C	-4.048248	1.444153	0.922644
C	-1.940518	-1.935800	0.664004
C	-3.090119	-2.487095	-0.155642
C	-0.876393	1.491761	-0.843310
C	-0.362506	0.994929	0.545679
C	0.152954	-0.475564	0.557872
C	-0.882954	-1.420795	0.015822
O	0.013949	2.563973	-1.252883
C	0.906045	2.903217	-0.281596
C	0.697286	2.010576	0.897634
O	1.707996	3.796960	-0.419625
C	1.380944	2.168789	2.033774
C	-2.322937	2.035367	-0.875814
C	-2.153195	-1.848782	2.154804
O	-5.070133	-1.987814	-1.381593
O	1.325750	-0.534589	-0.294226
C	2.441499	-1.145989	0.190541
O	2.517627	-1.610743	1.308967
C	3.526414	-1.189823	-0.864955
C	4.945682	-1.404111	-0.312343
C	5.914971	-1.720620	-1.461517
C	5.426107	-0.189931	0.497176
H	-4.702737	-1.178972	0.493865
H	-2.978731	-0.311674	-1.912987
H	-3.338459	1.616716	1.742318
H	-4.789656	0.719600	1.264783
H	-2.732452	-2.839482	-1.129051
H	-3.586460	-3.330384	0.338147
H	-4.563888	2.399843	0.762100
H	-1.177070	1.022723	1.275279
H	0.461776	-0.728257	1.574324
H	-0.832264	-1.551221	-1.063421
H	1.232343	1.524202	2.895126
H	2.121065	2.959836	2.111410
H	-2.355868	2.955087	-0.280380
H	-0.761209	0.713656	-1.599832
H	-2.544634	2.325243	-1.909356
H	-2.424959	-2.833164	2.554770
H	-2.982487	-1.172485	2.402112
H	-1.266390	-1.504530	2.692404
H	-5.724624	-1.324426	-1.637946
H	3.255537	-2.013458	-1.541071
H	3.468092	-0.275096	-1.466426
H	4.909384	-2.269702	0.360789

H	6.926330	-1.897705	-1.080021
H	5.605577	-2.614085	-2.016259
H	5.970609	-0.886854	-2.172743
H	6.429871	-0.365303	0.899508
H	4.761242	0.016488	1.341129
H	5.471660	0.708079	-0.131924

B3LYP energy = -1155.15760025 a.u.

(2*S*,6*R*,7*S*,8*R*)-**1**, Conf. C

C	-3.725681	-1.739254	-0.456201
C	-3.158424	-0.503260	-1.100140
C	-3.198544	0.760098	-0.640491
C	-4.079918	1.243954	0.487440
C	-1.606774	-1.822997	0.950319
C	-2.572001	-2.628475	0.103341
C	-0.761755	1.466682	-0.955122
C	-0.354664	1.258044	0.538427
C	0.299038	-0.123050	0.850486
C	-0.548215	-1.257310	0.347247
O	0.041870	2.569217	-1.453255
C	0.786672	3.156863	-0.476208
C	0.555274	2.432085	0.809027
O	1.497953	4.111030	-0.687727
C	1.108634	2.835171	1.955639
C	-2.247003	1.806603	-1.212323
C	-2.004408	-1.583963	2.385511
O	-4.427094	-2.574796	-1.389203
O	1.581287	-0.170999	0.172520
C	2.690768	-0.443506	0.914446
O	2.663660	-0.638741	2.112550
C	3.930263	-0.464359	0.049206
C	3.960771	-1.615949	-0.985926
C	5.203625	-1.479777	-1.877503
C	3.912554	-2.993336	-0.307887
H	-4.398477	-1.473834	0.369011
H	-2.514397	-0.719225	-1.954434
H	-3.488788	1.608033	1.338028
H	-4.769621	0.482860	0.857222
H	-2.055515	-3.057421	-0.762001
H	-3.023128	-3.456526	0.661873
H	-4.677353	2.098951	0.145366
H	-1.243453	1.308389	1.174453
H	0.485035	-0.183100	1.924562
H	-0.353381	-1.519926	-0.690809
H	0.949632	2.311399	2.893415
H	1.753074	3.709379	1.963543
H	-2.454514	2.788859	-0.772331
H	-0.477109	0.602736	-1.557776
H	-2.378464	1.915349	-2.295012
H	-2.949097	-1.028327	2.453829
H	-1.249046	-1.034635	2.952375
H	-2.171304	-2.542268	2.892513
H	-5.122798	-2.049812	-1.807094
H	3.988841	0.494821	-0.478699
H	4.789247	-0.538208	0.722793
H	3.071928	-1.511733	-1.621412

H	5.223967	-2.267659	-2.638298
H	5.224253	-0.513354	-2.393231
H	6.123591	-1.565438	-1.285819
H	3.925430	-3.792751	-1.056844
H	3.010424	-3.121220	0.299765
H	4.778356	-3.136612	0.350203

B3LYP energy = -1155.15693640 a.u.

(2*S*,6*R*,7*S*,8*R*)-**1**, Conf. D

C	-4.282788	-0.849439	-0.577768
C	-3.413046	0.299655	-1.011188
C	-3.071028	1.389957	-0.301888
C	-3.729105	1.828530	0.985699
C	-2.213150	-1.821083	0.533178
C	-3.410068	-2.122137	-0.346172
C	-0.550256	1.461909	-0.717766
C	-0.168935	0.802935	0.645244
C	0.097353	-0.731209	0.573085
C	-1.061900	-1.455859	-0.054072
O	0.519935	2.390309	-1.040529
C	1.424332	2.516225	-0.030895
C	1.030379	1.606255	1.086114
O	2.371220	3.265101	-0.096492
C	1.690592	1.584505	2.246715
C	-1.884117	2.241929	-0.741849
C	-2.459904	-1.792593	2.021227
O	-5.240985	-1.224652	-1.578320
O	1.272963	-0.936636	-0.251296
C	2.246005	-1.768832	0.217321
O	2.185725	-2.325984	1.294317
C	3.387434	-1.877349	-0.767420
C	4.325823	-0.641705	-0.773257
C	5.020289	-0.442140	0.581545
C	5.355010	-0.782895	-1.904594
H	-4.807379	-0.608193	0.355253
H	-2.895392	0.115626	-1.954203
H	-4.596467	1.224641	1.259268
H	-4.063816	2.869501	0.889733
H	-3.084620	-2.467700	-1.333153
H	-4.053300	-2.900555	0.079979
H	-3.024968	1.812812	1.828167
H	-0.991178	0.921591	1.356909
H	0.323422	-1.093586	1.578198
H	-0.993871	-1.530924	-1.137715
H	1.407706	0.926767	3.063418
H	2.547287	2.236253	2.391264
H	-1.782333	3.115361	-0.087522
H	-0.546003	0.723274	-1.520674
H	-2.024530	2.627695	-1.758105
H	-1.549642	-1.624985	2.601848
H	-2.893138	-2.745925	2.347671
H	-3.183884	-1.012973	2.293664
H	-5.769466	-0.447167	-1.802987
H	3.956729	-2.774344	-0.504826
H	2.966318	-2.011487	-1.769772
H	3.712295	0.243810	-0.981810

H	5.659794	0.446498	0.555537
H	4.301531	-0.312686	1.396570
H	5.650326	-1.305538	0.828749
H	6.011286	0.093075	-1.941259
H	4.868294	-0.878489	-2.881614
H	5.986609	-1.667408	-1.753568

B3LYP energy = -1155.15657770 a.u.

(2*S*,6*R*,7*S*,8*R*)-**1**, Conf. E

C	-4.127182	-1.504969	0.211513
C	-3.682201	-0.063552	0.041386
C	-3.147906	0.542860	-1.033291
C	-3.100452	-0.046823	-2.422662
C	-1.716313	-1.891284	0.954483
C	-2.910008	-2.489984	0.242000
C	-0.910640	1.704150	-0.703383
C	-0.415641	1.192672	0.687885
C	0.199291	-0.235142	0.699218
C	-0.792442	-1.252351	0.217445
O	-0.306069	3.012891	-0.898757
C	0.541239	3.359771	0.107648
C	0.540536	2.275723	1.133221
O	1.159937	4.398413	0.106327
C	1.295772	2.348020	2.232255
C	-2.438717	1.875271	-0.865146
C	-1.761295	-1.889024	2.462473
O	-4.993220	-2.005844	-0.815966
O	1.349304	-0.232396	-0.188427
C	2.547651	-0.636484	0.313178
O	2.702807	-1.020748	1.454215
C	3.639639	-0.496265	-0.726574
C	4.864703	-1.394368	-0.483945
C	4.519741	-2.880152	-0.668112
C	6.018182	-0.974567	-1.407169
H	-4.648702	-1.575294	1.177148
H	-3.657150	0.491750	0.979848
H	-3.631050	0.611446	-3.123487
H	-2.068368	-0.114074	-2.791350
H	-2.656953	-2.704007	-0.800876
H	-3.241606	-3.428176	0.701473
H	-3.558365	-1.034506	-2.470754
H	-1.270621	1.149898	1.373376
H	0.554960	-0.451798	1.707879
H	-0.861588	-1.325173	-0.865881
H	1.312923	1.561996	2.980972
H	1.930329	3.215217	2.389872
H	-2.822413	2.419569	0.005351
H	-0.513835	1.075714	-1.505440
H	-2.595923	2.512288	-1.743405
H	-2.565870	-1.240810	2.838478
H	-0.824917	-1.556649	2.916555
H	-1.975158	-2.897232	2.836509
H	-5.776018	-1.440132	-0.857355
H	3.208409	-0.671069	-1.719149
H	3.937376	0.561848	-0.708021
H	5.179225	-1.244705	0.556272

H	5.393959	-3.509471	-0.468644
H	3.722746	-3.194815	0.012597
H	4.193515	-3.081706	-1.696742
H	6.905659	-1.590620	-1.226433
H	6.300050	0.072723	-1.249200
H	5.743576	-1.091757	-2.463265

B3LYP emergy = -1155.15614961 a.u.

(2*S*,6*R*,7*S*,8*R*)-**1**, Conf. F

C	-4.324611	-1.174471	0.219277
C	-3.722171	0.215600	0.122524
C	-3.156345	0.827453	-0.932883
C	-3.223655	0.330497	-2.357219
C	-1.953078	-1.886277	0.844663
C	-3.228862	-2.290409	0.136838
C	-0.787137	1.697961	-0.620060
C	-0.307086	1.050787	0.718743
C	0.132189	-0.436726	0.624308
C	-0.989081	-1.295530	0.118909
O	-0.040011	2.937039	-0.767549
C	0.876466	3.123805	0.220672
C	0.786251	1.986050	1.182329
O	1.609909	4.084164	0.252418
C	1.585438	1.902951	2.248770
C	-2.289677	2.054286	-0.709915
C	-1.946741	-2.004532	2.348256
O	-5.276504	-1.503415	-0.801767
O	1.247681	-0.506668	-0.303546
C	2.355037	-1.199014	0.078020
O	2.476402	-1.722522	1.166430
C	3.369234	-1.248988	-1.045658
C	4.812231	-1.522809	-0.588627
C	5.698459	-1.836868	-1.803388
C	5.380854	-0.347522	0.221209
H	-4.818049	-1.250744	1.199133
H	-3.598780	0.698322	1.092721
H	-3.792850	-0.594347	-2.448633
H	-2.218798	0.172922	-2.770856
H	-3.031706	-2.453926	-0.927024
H	-3.654662	-3.216192	0.540650
H	-3.698273	1.089488	-2.993323
H	-1.135785	1.066725	1.436831
H	0.488608	-0.758006	1.604373
H	-1.106829	-1.272119	-0.962546
H	1.536913	1.077172	2.952245
H	2.323300	2.679762	2.426653
H	-2.578156	2.580548	0.207217
H	-0.495537	1.076149	-1.470954
H	-2.397960	2.762378	-1.539903
H	-0.970380	-1.791241	2.789224
H	-2.679168	-1.324403	2.806122
H	-2.235202	-3.018843	2.648617
H	-5.989939	-0.851681	-0.774546
H	3.029861	-2.046961	-1.721751
H	3.304426	-0.317776	-1.620389
H	4.788283	-2.405309	0.062622

H	6.724937	-2.055664	-1.490162
H	5.326278	-2.704451	-2.360457
H	5.737710	-0.985689	-2.494881
H	6.401583	-0.564714	0.554469
H	4.777680	-0.144207	1.111153
H	5.416392	0.566573	-0.385105

B3LYP energy = -1155.15608246 a.u.

(2*S*,6*R*,7*S*,8*R*)-**1**, Conf. G

C	-3.950508	-1.711343	-0.469804
C	-3.417129	-0.410261	-1.009755
C	-3.405308	0.792020	-0.409883
C	-4.199478	1.146047	0.825017
C	-1.698294	-1.980015	0.691104
C	-2.752842	-2.671781	-0.148780
C	-0.990767	1.508718	-0.855942
C	-0.456680	1.107692	0.555659
C	0.220379	-0.296245	0.621538
C	-0.678544	-1.366297	0.068246
O	-0.210555	2.657653	-1.281845
C	0.616147	3.119303	-0.303437
C	0.477989	2.242614	0.898226
O	1.321260	4.089734	-0.452548
C	1.119199	2.503122	2.040331
C	-2.485068	1.893806	-0.927281
C	-1.980914	-1.876619	2.169409
O	-4.881487	-2.358228	-1.344642
O	1.423943	-0.247177	-0.188009
C	2.606338	-0.587469	0.394813
O	2.704294	-0.954255	1.547585
C	3.754752	-0.401067	-0.574170
C	5.004619	-1.234105	-0.242371
C	4.747266	-2.736654	-0.434184
C	6.193860	-0.763049	-1.092680
H	-4.521541	-1.544343	0.445656
H	-2.828375	-0.523143	-1.924133
H	-4.816870	2.030280	0.620934
H	-3.546001	1.417681	1.664721
H	-2.317471	-2.990646	-1.104637
H	-3.159342	-3.563776	0.341205
H	-4.866853	0.347736	1.154227
H	-1.287352	1.064893	1.266589
H	0.516782	-0.489588	1.654539
H	-0.566508	-1.518855	-1.003588
H	1.026489	1.868311	2.916518
H	1.767938	3.371635	2.107228
H	-2.629910	2.810522	-0.344175
H	-0.779359	0.726362	-1.586622
H	-2.713247	2.148453	-1.968731
H	-2.166406	-2.872881	2.588666
H	-2.884096	-1.283175	2.364065
H	-1.157136	-1.427377	2.729105
H	-4.482298	-2.444321	-2.221672
H	3.397841	-0.606136	-1.590056
H	3.997144	0.670869	-0.549978
H	5.242310	-1.061893	0.814607

H	5.637326	-3.319707	-0.173470
H	3.924827	-3.087226	0.197018
H	4.498458	-2.960413	-1.479783
H	7.096708	-1.333548	-0.849292
H	6.412978	0.297842	-0.926478
H	5.994222	-0.899881	-2.163116

B3LYP energy = -1155.15573333 a.u.

(2*S*,6*R*,7*S*,8*R*)-**1**, Conf. H

C	-3.953467	-1.708364	-0.483154
C	-3.429450	-0.407545	-1.019381
C	-3.399549	0.783370	-0.399021
C	-4.166453	1.126224	0.856742
C	-1.700519	-1.968075	0.698792
C	-2.750531	-2.659631	-0.146417
C	-0.993150	1.518703	-0.854742
C	-0.451857	1.122028	0.555647
C	0.218841	-0.284178	0.624354
C	-0.685274	-1.350580	0.072816
O	-0.222839	2.674499	-1.282275
C	0.611142	3.135858	-0.310963
C	0.487938	2.255270	0.889648
O	1.311671	4.109370	-0.462997
C	1.143265	2.512351	2.024469
C	-2.490580	1.890662	-0.924144
C	-1.977854	-1.873294	2.178455
O	-4.785332	-2.306306	-1.485424
O	1.422004	-0.243196	-0.186249
C	2.602701	-0.589374	0.395619
O	2.699959	-0.955544	1.548833
C	3.751047	-0.410740	-0.574929
C	4.996344	-1.250905	-0.243957
C	4.729439	-2.752097	-0.433343
C	6.187018	-0.788238	-1.096852
H	-4.541145	-1.542978	0.428241
H	-2.881489	-0.517195	-1.956093
H	-4.779528	2.018735	0.677007
H	-3.496710	1.379617	1.689273
H	-2.320552	-2.975903	-1.103034
H	-3.144880	-3.555120	0.354121
H	-4.834488	0.328826	1.187351
H	-1.278301	1.086139	1.271759
H	0.515110	-0.477635	1.657356
H	-0.581305	-1.498602	-1.000192
H	1.061970	1.874762	2.899784
H	1.792405	3.380985	2.085778
H	-2.641484	2.809843	-0.346319
H	-0.777236	0.738373	-1.586242
H	-2.723369	2.135205	-1.966802
H	-2.150780	-2.872997	2.595645
H	-2.885820	-1.289290	2.379021
H	-1.156082	-1.418175	2.736356
H	-5.066392	-3.180165	-1.181013
H	3.391535	-0.614519	-1.590138
H	3.999837	0.659757	-0.552002
H	5.236803	-1.078733	0.812420

H	5.616245	-3.340361	-0.173164
H	3.905745	-3.096558	0.199593
H	4.477543	-2.975734	-1.478202
H	7.086944	-1.363586	-0.853992
H	6.412556	0.271635	-0.932714
H	5.984862	-0.925580	-2.166736

B3LYP energy = -1155.15571433 a.u.

(2*S*,6*R*,7*S*,8*R*)-**1**, Conf. I

C	-4.174072	-1.383817	-0.431255
C	-3.505895	-0.145735	-0.969277
C	-3.345183	1.039990	-0.358001
C	-4.069217	1.466645	0.896898
C	-1.937971	-1.942422	0.655574
C	-3.091656	-2.486184	-0.163124
C	-0.871986	1.486242	-0.846350
C	-0.364977	0.986994	0.544055
C	0.155120	-0.482071	0.554904
C	-0.876868	-1.430254	0.010555
O	0.025895	2.553143	-1.253565
C	0.911974	2.892240	-0.276650
C	0.691592	2.003381	0.903403
O	1.718125	3.782659	-0.411347
C	1.364660	2.164364	2.045449
C	-2.315263	2.037288	-0.881499
C	-2.156953	-1.849267	2.145218
O	-5.202812	-1.899375	-1.283938
O	1.329208	-0.536278	-0.295668
C	2.445335	-1.147754	0.188683
O	2.519527	-1.618362	1.304690
C	3.532983	-1.183419	-0.864358
C	4.951412	-1.397202	-0.309309
C	5.924071	-1.706392	-1.457642
C	5.427152	-0.185636	0.506813
H	-4.691958	-1.166593	0.504934
H	-2.953718	-0.312597	-1.897829
H	-3.372780	1.651939	1.725466
H	-4.817364	0.746268	1.232382
H	-2.725314	-2.835191	-1.137210
H	-3.588174	-3.333111	0.323855
H	-4.585111	2.418148	0.714513
H	-1.184077	1.010924	1.268807
H	0.463260	-0.735224	1.571490
H	-0.820484	-1.562663	-1.068343
H	1.207034	1.522739	2.907415
H	2.104562	2.955131	2.127912
H	-2.344102	2.955938	-0.284344
H	-0.757744	0.707738	-1.602691
H	-2.533585	2.331908	-1.914523
H	-2.438841	-2.829897	2.547203
H	-2.980787	-1.164585	2.387499
H	-1.269208	-1.511025	2.684883
H	-4.846695	-2.010004	-2.176628
H	3.265937	-2.004274	-1.545409
H	3.473971	-0.265680	-1.461143
H	4.915324	-2.265937	0.359774

H	6.934937	-1.882816	-1.074584
H	5.618121	-2.598160	-2.017058
H	5.979452	-0.869355	-2.165034
H	6.430255	-0.360659	0.910883
H	4.759718	0.015520	1.350000
H	5.472279	0.715270	-0.118124

B3LYP energy = -1155.15560372 a.u.

(2*S*,6*R*,7*S*,8*R*)-**1**, Conf. J

C	-4.178113	-1.379925	-0.444611
C	-3.520051	-0.140204	-0.977932
C	-3.341366	1.031189	-0.345516
C	-4.037144	1.442049	0.931197
C	-1.938884	-1.928237	0.662327
C	-3.087407	-2.473093	-0.162145
C	-0.875304	1.495762	-0.844640
C	-0.360030	1.001346	0.544660
C	0.155165	-0.468802	0.558412
C	-0.880583	-1.413399	0.015324
O	0.014883	2.568375	-1.255630
C	0.908123	2.907931	-0.286388
C	0.700511	2.016814	0.894480
O	1.711164	3.800677	-0.425740
C	1.384803	2.176549	2.029978
C	-2.322518	2.035611	-0.876719
C	-2.155946	-1.840307	2.152348
O	-5.106370	-1.856595	-1.427045
O	1.328519	-0.528740	-0.292616
C	2.439314	-1.151783	0.188212
O	2.509728	-1.629127	1.301793
C	3.526834	-1.190216	-0.864847
C	4.943764	-1.414848	-0.310275
C	5.915074	-1.724812	-1.459535
C	5.426382	-0.209653	0.511236
H	-4.712464	-1.163658	0.488609
H	-3.009594	-0.298097	-1.928760
H	-3.324788	1.612039	1.749406
H	-4.781082	0.719550	1.271921
H	-2.725442	-2.817808	-1.136998
H	-3.570884	-3.326300	0.334250
H	-4.552431	2.398301	0.773312
H	-1.174096	1.030779	1.274638
H	0.462722	-0.722212	1.575121
H	-0.829920	-1.542071	-1.064060
H	1.236337	1.533520	2.892560
H	2.124910	2.967756	2.106121
H	-2.356330	2.957056	-0.283990
H	-0.759052	0.717125	-1.600511
H	-2.547266	2.321374	-1.910651
H	-2.434413	-2.822741	2.552978
H	-2.980400	-1.157401	2.397281
H	-1.267959	-1.501961	2.691584
H	-5.477199	-2.697920	-1.127102
H	3.254810	-2.006721	-1.549128
H	3.473502	-0.269894	-1.458129
H	4.902303	-2.286232	0.355063

H	6.924681	-1.909105	-1.076804
H	5.603914	-2.612125	-2.023120
H	5.975872	-0.884993	-2.163160
H	6.428071	-0.392519	0.915388
H	4.759467	-0.007780	1.354680
H	5.477601	0.693479	-0.110003

B3LYP energy = -1155.15557900 a.u.

(2*S*,6*R*,7*S*,8*R*)-**1**, Conf. K

C	-3.985025	-1.373408	0.246017
C	-3.510817	0.029274	-0.089668
C	-2.883836	0.474455	-1.192721
C	-2.734607	-0.308748	-2.475302
C	-1.647182	-1.650454	1.234346
C	-2.784920	-2.343165	0.514862
C	-0.661375	1.666258	-0.858582
C	-0.276524	1.371043	0.626807
C	0.302471	-0.044131	0.905686
C	-0.663489	-1.114488	0.492711
O	-0.027371	2.929238	-1.203376
C	0.753640	3.419756	-0.202422
C	0.670720	2.498729	0.968516
O	1.383193	4.445613	-0.314706
C	1.363869	2.722708	2.087754
C	-2.170656	1.814769	-1.160774
C	-1.810106	-1.450698	2.720552
O	-4.770668	-2.015700	-0.767409
O	1.526290	-0.185493	0.136594
C	2.662937	-0.539171	0.797749
O	2.712749	-0.717239	1.997989
C	3.824784	-0.680401	-0.159525
C	3.691746	-1.878177	-1.132626
C	4.854031	-1.862924	-2.136332
C	3.623451	-3.217241	-0.383340
H	-4.583895	-1.306218	1.165891
H	-3.551034	0.710818	0.760906
H	-3.200807	-1.292082	-2.418800
H	-1.677601	-0.430710	-2.746443
H	-2.448318	-2.699370	-0.463575
H	-3.163430	-3.208610	1.070636
H	-3.198292	0.243548	-3.303540
H	-1.178324	1.445728	1.246410
H	0.562267	-0.109089	1.963493
H	-0.653169	-1.330935	-0.573565
H	1.326647	2.053802	2.941980
H	2.004069	3.597505	2.154200
H	-2.613062	2.479993	-0.410376
H	-0.214917	0.922107	-1.524082
H	-2.250782	2.319282	-2.130822
H	-0.916839	-1.040500	3.197188
H	-2.649961	-0.777265	2.943557
H	-2.041003	-2.405915	3.206913
H	-5.541328	-1.462101	-0.952131
H	3.902579	0.246905	-0.738960
H	4.729787	-0.784744	0.446270
H	2.756121	-1.745934	-1.691211

H	4.760188	-2.685722	-2.853503
H	4.884137	-0.925385	-2.702660
H	5.817419	-1.977346	-1.624007
H	3.519944	-4.049130	-1.088505
H	2.775317	-3.259069	0.308035
H	4.536072	-3.384825	0.201705

B3LYP energy = -1155.15533922 a.u.

(2*S*,6*R*,7*S*,8*R*)-**1**, Conf. L

C	-4.007674	-1.643548	-0.351965
C	-3.495047	-0.327896	-0.872558
C	-3.407711	0.842552	-0.216041
C	-4.079915	1.149425	1.102081
C	-1.691191	-1.993504	0.635241
C	-2.820175	-2.634639	-0.146545
C	-1.023799	1.546054	-0.818937
C	-0.387912	1.071106	0.526257
C	0.256215	-0.348102	0.476423
C	-0.713399	-1.372379	-0.044483
O	-0.260953	2.705037	-1.250125
C	0.652289	3.104354	-0.322723
C	0.597582	2.170346	0.841500
O	1.360705	4.070798	-0.482353
C	1.337905	2.362361	1.936308
C	-2.513270	1.953316	-0.758516
C	-1.844473	-1.957075	2.135520
O	-4.890061	-2.296865	-1.276742
O	1.388977	-0.288362	-0.427541
C	2.604411	-0.701645	0.030900
O	2.781212	-1.132242	1.151537
C	3.650256	-0.516564	-1.050836
C	5.025351	-1.172115	-0.812204
C	5.792212	-0.548206	0.365451
C	4.930129	-2.701434	-0.686496
H	-4.523568	-1.507607	0.606878
H	-3.003078	-0.411074	-1.842974
H	-4.736527	0.350527	1.451789
H	-4.685031	2.059773	1.001815
H	-2.476810	-2.928200	-1.144250
H	-3.209476	-3.532925	0.346099
H	-3.347573	1.357972	1.893174
H	-1.158118	1.015289	1.301139
H	0.632029	-0.594315	1.471716
H	-0.695187	-1.479600	-1.127287
H	1.305816	1.685945	2.785265
H	2.008316	3.215168	1.990021
H	-2.598438	2.848111	-0.131147
H	-0.882022	0.797495	-1.600149
H	-2.815786	2.251278	-1.769003
H	-2.002019	-2.971445	2.521900
H	-2.723074	-1.370540	2.435402
H	-0.972039	-1.538990	2.643418
H	-5.617108	-1.694389	-1.483305
H	3.212402	-0.878934	-1.988546
H	3.766842	0.567914	-1.183267
H	5.598320	-0.954340	-1.725021

H	6.807283	-0.957606	0.418063
H	5.878547	0.538928	0.251143
H	5.291898	-0.752762	1.315742
H	5.930733	-3.145426	-0.642210
H	4.410678	-3.140623	-1.546748
H	4.393247	-2.990990	0.221864

B3LYP energy = -1155.15521256 a.u.

(2*S*,6*R*,7*S*,8*R*)-**1**, Conf. M

C	-3.605516	-1.944875	-0.294200
C	-3.193522	-0.646137	-0.933247
C	-3.294636	0.593938	-0.422328
C	-4.110863	0.964197	0.794416
C	-1.360753	-1.914716	0.897863
C	-2.341543	-2.763407	0.113607
C	-0.956847	1.506972	-0.920789
C	-0.398214	1.259435	0.516731
C	0.396002	-0.070918	0.688184
C	-0.407835	-1.252262	0.221775
O	-0.292753	2.697328	-1.422979
C	0.485065	3.299521	-0.481120
C	0.431365	2.494228	0.775472
O	1.091630	4.322417	-0.697234
C	1.051861	2.884229	1.891816
C	-2.481602	1.735319	-1.025569
C	-1.642748	-1.742250	2.369514
O	-4.333651	-2.795956	-1.192029
O	1.593666	0.023217	-0.126080
C	2.793175	-0.235470	0.465141
O	2.908900	-0.533219	1.637339
C	3.934051	-0.108763	-0.520788
C	4.542094	-1.469867	-0.962560
C	5.252200	-2.202909	0.184833
C	3.502274	-2.368801	-1.649156
H	-4.209958	-1.762820	0.603373
H	-2.609828	-0.781219	-1.845398
H	-4.709708	0.138629	1.183538
H	-4.795490	1.783953	0.541361
H	-1.882409	-3.120979	-0.814220
H	-2.674305	-3.642429	0.677332
H	-3.477561	1.338026	1.609761
H	-1.225902	1.196811	1.229624
H	0.704972	-0.158579	1.731827
H	-0.292965	-1.466473	-0.838920
H	1.021291	2.300718	2.807214
H	1.620835	3.809332	1.896254
H	-2.720596	2.677945	-0.519786
H	-0.660876	0.702149	-1.595860
H	-2.722578	1.880280	-2.084963
H	-1.711812	-2.722344	2.857017
H	-2.606867	-1.243276	2.535773
H	-0.872117	-1.166049	2.887180
H	-5.099697	-2.307930	-1.522518
H	3.583712	0.438379	-1.400030
H	4.711580	0.486519	-0.030277
H	5.301728	-1.207313	-1.711823

H	5.732445	-3.114847	-0.187189
H	6.026655	-1.575008	0.638932
H	4.550347	-2.483976	0.975806
H	3.978911	-3.269558	-2.050718
H	3.006631	-1.851603	-2.478230
H	2.727182	-2.693407	-0.944761

B3LYP energy = -1155.15515922 a.u.

(2*S*,6*R*,7*S*,8*R*)-**1**, Conf. N

C	4.415594	-0.603243	-0.044446
C	3.575252	0.660371	-0.090788
C	2.857126	1.241154	0.886478
C	2.939729	0.877359	2.349773
C	2.236339	-1.773799	-0.676876
C	3.529553	-1.886384	0.101584
C	0.391874	1.648710	0.423057
C	0.100583	0.813123	-0.864557
C	-0.081718	-0.714340	-0.645826
C	1.149186	-1.316864	-0.033634
O	-0.569071	2.740809	0.429278
C	-1.449230	2.677603	-0.606398
C	-1.107484	1.500783	-1.458035
O	-2.338720	3.483306	-0.752086
C	-1.812031	1.194452	-2.550184
C	1.802591	2.273629	0.529171
C	2.319763	-1.998005	-2.166102
O	5.360950	-0.673099	1.031960
O	-1.205062	-0.903955	0.254572
C	-2.134557	-1.844356	-0.073387
O	-2.097458	-2.490575	-1.100602
C	-3.192072	-1.961853	1.001302
C	-4.117025	-0.724455	1.122326
C	-4.914983	-0.482328	-0.167066
C	-5.051968	-0.893071	2.328879
H	4.960882	-0.669748	-0.997071
H	3.422405	1.038157	-1.102447
H	3.654858	0.077280	2.539497
H	1.958686	0.579106	2.742242
H	3.316342	-1.996673	1.169233
H	4.128398	-2.750729	-0.207661
H	3.246034	1.755877	2.933282
H	0.951209	0.910566	-1.549673
H	-0.334572	-1.176044	-1.602017
H	1.207558	-1.194620	1.046064
H	-1.577007	0.337542	-3.174522
H	-2.659547	1.812182	-2.832486
H	2.038291	2.766741	-0.420908
H	0.172577	1.060660	1.318279
H	1.746974	3.054123	1.297091
H	2.910794	-1.211448	-2.656982
H	1.339399	-2.029692	-2.647168
H	2.826874	-2.946438	-2.378853
H	5.951059	0.090085	0.970104
H	-3.784421	-2.852782	0.772291
H	-2.682533	-2.123173	1.958629
H	-3.480308	0.150493	1.304879

H	-5.544895	0.407973	-0.068606
H	-4.259940	-0.330805	-1.030925
H	-5.567504	-1.335787	-0.389042
H	-5.695680	-0.014770	2.446205
H	-4.488285	-1.022592	3.259702
H	-5.701885	-1.768289	2.204265

B3LYP energy = -1155.15511951 a.u.

(2*S*,6*R*,7*S*,8*R*)-**1**, Conf. O

C	-3.763040	-1.655050	-0.473828
C	-3.143201	-0.456292	-1.131198
C	-3.149715	0.814090	-0.695377
C	-4.043734	1.352511	0.396933
C	-1.667700	-1.763545	0.990516
C	-2.635227	-2.559974	0.138911
C	-0.687361	1.444020	-0.967053
C	-0.323985	1.258345	0.540585
C	0.293157	-0.129143	0.893725
C	-0.572264	-1.255470	0.402246
O	0.162632	2.510934	-1.467860
C	0.895227	3.101564	-0.484402
C	0.606052	2.416258	0.811023
O	1.640334	4.029527	-0.696978
C	1.132555	2.834881	1.964625
C	-2.154836	1.818540	-1.269514
C	-2.103682	-1.454792	2.401036
O	-4.492255	-2.386880	-1.467213
O	1.582479	-0.216075	0.234581
C	2.669270	-0.543096	0.987509
O	2.614581	-0.765145	2.179885
C	3.925241	-0.574103	0.145523
C	3.899431	-1.603218	-1.010066
C	5.178605	-1.473911	-1.849819
C	3.720061	-3.036684	-0.488104
H	-4.445835	-1.347252	0.327679
H	-2.496359	-0.711677	-1.971564
H	-3.465573	1.726527	1.252311
H	-4.765000	0.621594	0.767127
H	-2.108393	-3.021202	-0.703539
H	-3.110743	-3.365392	0.716031
H	-4.609653	2.211413	0.013748
H	-1.227192	1.340994	1.152140
H	0.462943	-0.171245	1.971560
H	-0.354444	-1.559669	-0.619626
H	0.929277	2.339879	2.909572
H	1.798041	3.693166	1.972254
H	-2.344894	2.817078	-0.859310
H	-0.412785	0.558931	-1.543061
H	-2.258063	1.902677	-2.357374
H	-2.339217	-2.383749	2.934957
H	-3.017866	-0.846700	2.414726
H	-1.339982	-0.926005	2.976115
H	-4.834734	-3.199938	-1.071083
H	4.069509	0.430667	-0.270493
H	4.758007	-0.783562	0.823351
H	3.043477	-1.358075	-1.651271

H	5.164521	-2.175128	-2.691320
H	5.290924	-0.462970	-2.256932
H	6.068725	-1.695205	-1.247724
H	3.695721	-3.750970	-1.318372
H	2.789666	-3.153343	0.078241
H	4.548626	-3.319315	0.172919

B3LYP energy = -1155.15492832 a.u.

(2*S*,6*R*,7*S*,8*R*)-**1**, Conf. P

C	-3.764039	-1.656431	-0.456888
C	-3.132491	-0.460404	-1.119160
C	-3.154884	0.819665	-0.711501
C	-4.077212	1.370167	0.350066
C	-1.667030	-1.778528	0.986435
C	-2.640828	-2.574021	0.140676
C	-0.684547	1.429280	-0.966602
C	-0.330652	1.239783	0.542549
C	0.293705	-0.145768	0.893533
C	-0.566324	-1.276543	0.402151
O	0.176341	2.488900	-1.462669
C	0.898777	3.081717	-0.472011
C	0.592533	2.401079	0.821757
O	1.648253	4.007047	-0.679663
C	1.102305	2.824642	1.981086
C	-2.147472	1.817577	-1.275304
C	-2.108758	-1.456359	2.392481
O	-4.610515	-2.416126	-1.327039
O	1.583379	-0.224095	0.234014
C	2.673970	-0.537028	0.988012
O	2.621729	-0.755790	2.180911
C	3.930525	-0.555737	0.146325
C	3.914487	-1.581045	-1.012464
C	5.193044	-1.437815	-1.850948
C	3.747181	-3.017635	-0.495076
H	-4.428652	-1.344230	0.351113
H	-2.445396	-0.719447	-1.929240
H	-3.518679	1.765834	1.208731
H	-4.642286	2.216390	-0.061442
H	-2.110274	-3.038602	-0.700359
H	-3.127722	-3.376532	0.706208
H	-4.800088	0.639962	0.718025
H	-1.238231	1.314715	1.148752
H	0.463902	-0.187482	1.971357
H	-0.340312	-1.587024	-0.616306
H	0.886315	2.332687	2.924789
H	1.766470	3.683859	1.995182
H	-2.331816	2.815156	-0.860437
H	-0.414186	0.542159	-1.541484
H	-2.244010	1.910763	-2.363232
H	-1.340145	-0.939408	2.971783
H	-2.366771	-2.378696	2.926819
H	-3.011637	-0.831338	2.396456
H	-4.124280	-2.625237	-2.136824
H	4.066862	0.451674	-0.266110
H	4.764501	-0.760077	0.824234
H	3.056838	-1.341214	-1.653495

H	5.186039	-2.136461	-2.694706
H	5.296736	-0.424604	-2.254731
H	6.084663	-1.653022	-1.248907
H	3.729244	-3.729752	-1.327396
H	2.817749	-3.143882	0.070809
H	4.577931	-3.295251	0.165279

B3LYP energy = -1155.15491422 a.u.

H	5.160063	-1.204015	0.663898
H	5.522652	-3.397878	-0.466620
H	3.815362	-3.171858	-0.068333
H	4.377900	-2.946561	-1.738721
H	6.996787	-1.387815	-1.030861
H	6.329692	0.251951	-0.998095
H	5.888040	-0.860709	-2.304415

B3LYP energy = -1155.15856156 a.u.

(2*R*,6*R*,7*S*,8*S*)-**1**, Conf. A

C	-4.121283	-1.527050	-0.323972
C	-3.668669	-0.087744	-0.253172
C	-3.128756	0.662441	-1.226991
C	-3.065864	0.250225	-2.678086
C	-1.768696	-1.987613	0.590333
C	-2.910400	-2.520396	-0.250852
C	-0.891053	1.745722	-0.678612
C	-0.449482	1.114042	0.680954
C	0.154702	-0.315737	0.593882
C	-0.811106	-1.278000	-0.031515
O	-0.265461	3.057316	-0.744786
C	0.548549	3.309697	0.316094
C	0.499403	2.145651	1.248130
O	1.177482	4.336866	0.420794
C	1.215255	2.118218	2.375184
C	-2.410821	1.952435	-0.876292
C	-1.900637	-2.139108	2.084304
O	-4.981648	-1.843720	0.781859
O	1.350646	-0.241857	-0.229503
C	2.526094	-0.671683	0.302949
O	2.627595	-1.140714	1.417947
C	3.667807	-0.440898	-0.665099
C	4.911092	-1.306320	-0.399575
C	4.636574	-2.791106	-0.683144
C	6.097956	-0.794057	-1.229234
H	-4.654889	-1.733626	-1.262237
H	-3.648346	0.303703	0.764499
H	-3.465252	1.047184	-3.317491
H	-3.629423	-0.662545	-2.883819
H	-2.559360	-2.685533	-1.274829
H	-3.301708	-3.471408	0.127449
H	-2.030742	0.080888	-3.004586
H	-1.327439	1.023117	1.331671
H	0.454595	-0.626854	1.595761
H	-0.820149	-1.246920	-1.119811
H	1.199204	1.273425	3.056923
H	1.851593	2.962034	2.625588
H	-2.824841	2.395718	0.036721
H	-0.475661	1.180039	-1.517524
H	-2.528889	2.690239	-1.678360
H	-2.791176	-1.615407	2.453873
H	-1.026811	-1.775174	2.629847
H	-2.042402	-3.195606	2.341605
H	-5.742104	-1.246987	0.762621
H	3.297325	-0.579788	-1.687634
H	3.923073	0.624924	-0.579299

(2*R*,6*R*,7*S*,8*S*)-**1**, Conf. B

C	-4.309677	-1.202520	-0.296462
C	-3.703835	0.177507	-0.192265
C	-3.112614	0.902781	-1.155207
C	-3.129130	0.539778	-2.620730
C	-1.995688	-1.941218	0.533908
C	-3.212366	-2.323049	-0.283313
C	-0.759965	1.730757	-0.634870
C	-0.354046	0.996869	0.684129
C	0.094750	-0.480788	0.520136
C	-0.990163	-1.312269	-0.098465
O	-0.005097	2.973926	-0.661652
C	0.854526	3.094275	0.387296
C	0.708262	1.899356	1.268483
O	1.585614	4.047691	0.520166
C	1.441715	1.747072	2.374044
C	-2.254810	2.098241	-0.781107
C	-2.093909	-2.133488	2.025626
O	-5.175546	-1.460966	0.820345
O	1.259627	-0.492601	-0.348939
C	2.354765	-1.190150	0.057288
O	2.429503	-1.758918	1.126919
C	3.423213	-1.183440	-1.016381
C	4.843517	-1.471662	-0.500829
C	5.789573	-1.737500	-1.681607
C	5.366515	-0.327326	0.380547
H	-4.882323	-1.319331	-1.227039
H	-3.615470	0.527138	0.836891
H	-3.784181	-0.306358	-2.840114
H	-2.124492	0.283327	-2.983476
H	-2.910818	-2.498709	-1.321162
H	-3.694247	-3.235403	0.085147
H	-3.465734	1.394253	-3.220819
H	-1.221004	0.965859	1.354683
H	0.399346	-0.862741	1.495719
H	-1.033970	-1.238120	-1.183826
H	1.350742	0.878484	3.019279
H	2.167128	2.508547	2.644993
H	-2.597156	2.546604	0.158689
H	-0.423101	1.164285	-1.507639
H	-2.316753	2.874026	-1.553054
H	-2.921068	-1.545220	2.442377
H	-1.173983	-1.869156	2.552290
H	-2.323102	-3.181652	2.252643
H	-5.866089	-0.784557	0.838768
H	3.122697	-1.951127	-1.743973
H	3.380512	-0.227190	-1.550734

H	4.790895	-2.378688	0.114039
H	6.800269	-1.966155	-1.326705
H	5.448999	-2.584135	-2.289032
H	5.859900	-0.860733	-2.337860
H	6.368124	-0.556908	0.760186
H	4.715355	-0.156891	1.243085
H	5.432901	0.608145	-0.189390

B3LYP energy = -1155.15846845 a.u.

(2*R*,6*R*,7*S*,8*S*)-**1**, Conf. C

C	3.940480	-1.444760	0.337225
C	3.448901	-0.022217	0.466301
C	2.777453	0.537952	1.485217
C	2.565107	-0.122423	2.826157
C	1.726861	-1.767953	-0.919553
C	2.781593	-2.425920	-0.054198
C	0.583227	1.678200	0.865984
C	0.322758	1.287986	-0.625206
C	-0.236152	-0.142755	-0.859165
C	0.685514	-1.185566	-0.300374
O	-0.083782	2.953017	1.079051
C	-0.774535	3.378687	-0.013974
C	-0.589325	2.389980	-1.115405
O	-1.412323	4.405626	-0.017512
C	-1.182349	2.541817	-2.302339
C	2.062415	1.861124	1.279296
C	2.029957	-1.659251	-2.392056
O	4.933093	-1.546418	-0.696057
O	-1.521384	-0.231219	-0.186553
C	-2.600283	-0.631533	-0.913672
O	-2.547750	-0.909956	-2.094419
C	-3.848035	-0.671030	-0.059740
C	-3.798474	-1.701949	1.094269
C	-5.068319	-1.585200	1.949915
C	-3.612548	-3.133401	0.569167
H	4.369088	-1.808415	1.281486
H	3.527644	0.541280	-0.464070
H	3.123678	-1.055140	2.931853
H	1.504507	-0.349764	2.999731
H	2.320413	-2.775519	0.875473
H	3.246922	-3.288674	-0.543700
H	2.873022	0.551283	3.635386
H	1.271826	1.325067	-1.173151
H	-0.407671	-0.277887	-1.928100
H	0.576316	-1.340939	0.771663
H	-1.070425	1.821833	-3.107163
H	-1.814061	3.407608	-2.478108
H	2.566801	2.462382	0.514066
H	0.088223	0.971914	1.538755
H	2.062406	2.447936	2.205220
H	1.216739	-1.206168	-2.963723
H	2.220755	-2.655429	-2.809006
H	2.946094	-1.079589	-2.560608
H	5.664593	-0.951652	-0.481781
H	-3.998872	0.331353	0.359022
H	-4.685737	-0.891134	-0.727989

H	-2.936430	-1.450010	1.725114
H	-5.037478	-2.287133	2.790395
H	-5.185859	-0.575601	2.359116
H	-5.963420	-1.814514	1.358424
H	-3.573881	-3.847725	1.398900
H	-2.687679	-3.242320	-0.007257
H	-4.445812	-3.422176	-0.083128

B3LYP energy = -1155.15771223 a.u.

(2*R*,6*R*,7*S*,8*S*)-**1**, Conf. D

C	4.393831	-0.598803	0.425596
C	3.564503	0.638242	0.172291
C	2.839562	1.350807	1.049563
C	2.887534	1.156181	2.545958
C	2.261141	-1.812035	-0.331419
C	3.503555	-1.884904	0.532698
C	0.395341	1.691021	0.430467
C	0.159816	0.771826	-0.810301
C	-0.046375	-0.735084	-0.494152
C	1.147294	-1.299629	0.219180
O	-0.574027	2.771126	0.332019
C	-1.406430	2.639642	-0.736703
C	-1.013052	1.423061	-1.506056
O	-2.296363	3.425119	-0.966052
C	-1.649148	1.060030	-2.622668
C	1.797176	2.333242	0.545563
C	2.426557	-2.150417	-1.790800
O	5.312192	-0.822207	-0.656147
O	-1.216689	-0.846510	0.359227
C	-2.113055	-1.835237	0.087009
O	-1.998581	-2.602555	-0.846768
C	-3.250712	-1.825931	1.083153
C	-4.244317	-0.652009	0.885899
C	-4.944349	-0.719099	-0.479097
C	-5.266729	-0.641479	2.031929
H	4.960084	-0.516376	1.363793
H	3.440699	0.856226	-0.889015
H	3.675319	0.467121	2.858626
H	1.935280	0.768145	2.932301
H	3.208295	-1.992190	1.581658
H	4.143209	-2.736580	0.275737
H	3.056893	2.116178	3.049160
H	1.043191	0.814973	-1.457899
H	-0.256781	-1.263470	-1.425592
H	1.151063	-1.098638	1.289374
H	-1.370887	0.177487	-3.191086
H	-2.481371	1.656606	-2.984682
H	2.079618	2.735910	-0.434021
H	0.147866	1.160118	1.353825
H	1.703090	3.182547	1.232110
H	3.136287	-1.467371	-2.274416
H	1.483855	-2.127821	-2.342436
H	2.853501	-3.154960	-1.895460
H	5.879259	-0.044295	-0.745523
H	-3.775664	-2.780719	0.982606
H	-2.823956	-1.768149	2.090812

H	-3.670181	0.282069	0.934795
H	-5.624127	0.130347	-0.604184
H	-4.229390	-0.698738	-1.307544
H	-5.533107	-1.640119	-0.571362
H	-5.960076	0.199715	1.925621
H	-4.776197	-0.550346	3.007637
H	-5.860883	-1.563997	2.038257

B3LYP energy = -1155.15750883 a.u.

(2*R*,6*R*,7*S*,8*S*)-**1**, Conf. E

C	-4.135328	-1.511528	-0.298945
C	-3.683687	-0.074635	-0.255447
C	-3.116068	0.642835	-1.236008
C	-3.024595	0.191805	-2.674313
C	-1.763923	-1.985885	0.570228
C	-2.909371	-2.502504	-0.273673
C	-0.889762	1.748714	-0.695917
C	-0.457774	1.119688	0.667583
C	0.152557	-0.307784	0.586334
C	-0.806464	-1.271584	-0.046492
O	-0.270788	3.064034	-0.755925
C	0.533693	3.319388	0.310937
C	0.482555	2.154655	1.242492
O	1.157889	4.348955	0.421382
C	1.189532	2.130150	2.375157
C	-2.408485	1.944042	-0.907669
C	-1.895594	-2.144744	2.063745
O	-4.984211	-1.690258	0.843372
O	1.354833	-0.230698	-0.227346
C	2.523740	-0.672198	0.309277
O	2.614085	-1.154827	1.419537
C	3.674453	-0.435543	-0.646695
C	4.910767	-1.311157	-0.382063
C	4.630207	-2.790743	-0.686201
C	6.107205	-0.795448	-1.195668
H	-4.708607	-1.722651	-1.213056
H	-3.715404	0.347877	0.748004
H	-1.983064	0.041574	-2.989236
H	-3.440091	0.960271	-3.338191
H	-2.571418	-2.632284	-1.307810
H	-3.271363	-3.479704	0.074270
H	-3.563793	-0.739732	-2.861432
H	-1.341892	1.025675	1.309548
H	0.445042	-0.618023	1.590656
H	-0.814029	-1.233593	-1.134277
H	1.171192	1.285452	3.057024
H	1.819933	2.976799	2.630949
H	-2.830982	2.402686	-0.006324
H	-0.461909	1.185983	-1.530601
H	-2.525319	2.665515	-1.724857
H	-2.051315	-3.200865	2.316398
H	-2.774829	-1.606159	2.438865
H	-1.013636	-1.799449	2.608094
H	-5.364358	-2.577836	0.817345
H	3.311426	-0.559515	-1.673812
H	3.934715	0.627694	-0.545518

H	5.151616	-1.223464	0.684574
H	5.510771	-3.405413	-0.469405
H	3.801413	-3.173986	-0.083187
H	4.379903	-2.931769	-1.745819
H	7.001027	-1.396821	-0.997590
H	6.342926	0.246212	-0.949487
H	5.905629	-0.847268	-2.273256

B3LYP energy = -1155.15733917 a.u.

(2*R*,6*R*,7*S*,8*S*)-**1**, Conf. F

C	-4.330708	-1.168988	-0.267699
C	-3.718648	0.205471	-0.182315
C	-3.107576	0.901662	-1.152134
C	-3.113373	0.510636	-2.610733
C	-2.001636	-1.938617	0.499988
C	-3.224715	-2.292314	-0.318178
C	-0.755584	1.733524	-0.644246
C	-0.355859	0.993684	0.673046
C	0.091426	-0.483901	0.503963
C	-0.993204	-1.307307	-0.125418
O	0.002134	2.975442	-0.663232
C	0.858168	3.089452	0.388743
C	0.705490	1.891737	1.265499
O	1.592161	4.039859	0.528048
C	1.432626	1.734887	2.374550
C	-2.249413	2.101402	-0.793798
C	-2.098879	-2.146564	1.989968
O	-5.163468	-1.298955	0.893133
O	1.261455	-0.494557	-0.358085
C	2.350571	-1.199973	0.050002
O	2.414117	-1.780716	1.114057
C	3.428965	-1.185661	-1.013596
C	4.845561	-1.467821	-0.484257
C	5.805133	-1.725857	-1.655785
C	5.353122	-0.322730	0.405106
H	-4.948460	-1.275614	-1.170878
H	-3.666895	0.580811	0.838847
H	-3.768700	-0.338522	-2.818153
H	-2.107708	0.248971	-2.966826
H	-2.937458	-2.423789	-1.367320
H	-3.683546	-3.234087	0.012386
H	-3.449876	1.353568	-3.227149
H	-1.225963	0.960608	1.339340
H	0.388950	-0.872265	1.479171
H	-1.036477	-1.220015	-1.209508
H	1.335988	0.864871	3.017061
H	2.157879	2.494254	2.651757
H	-2.590955	2.560665	0.140900
H	-0.415672	1.170549	-1.518127
H	-2.311400	2.868418	-1.574636
H	-2.345279	-3.193448	2.206913
H	-2.910999	-1.544583	2.416564
H	-1.171278	-1.907406	2.514969
H	-5.640405	-2.137591	0.845767
H	3.139678	-1.953955	-1.745111
H	3.386842	-0.228729	-1.546598

H	4.791272	-2.376588	0.127898
H	6.813332	-1.949901	-1.290942
H	5.475469	-2.572937	-2.268618
H	5.877524	-0.847236	-2.309300
H	6.351813	-0.547818	0.794975
H	4.691857	-0.157548	1.260970
H	5.420468	0.614388	-0.161957

B3LYP energy = -1155.15721714 a.u.

(2*R*,6*R*,7*S*,8*S*)-**1**, Conf. G

C	3.964104	-1.416564	0.330673
C	3.466926	-0.002793	0.488458
C	2.755501	0.515869	1.500097
C	2.505558	-0.189479	2.811773
C	1.732860	-1.765093	-0.896686
C	2.788380	-2.406420	-0.022213
C	0.572422	1.673355	0.884561
C	0.332148	1.286800	-0.610666
C	-0.229826	-0.141289	-0.853549
C	0.684547	-1.185856	-0.286250
O	-0.091527	2.951395	1.089588
C	-0.765441	3.380686	-0.011896
C	-0.568400	2.392813	-1.112412
O	-1.400122	4.409619	-0.023492
C	-1.142891	2.549193	-2.307793
C	2.046017	1.845227	1.319220
C	2.044801	-1.654763	-2.367505
O	4.948097	-1.370876	-0.712050
O	-1.522089	-0.226371	-0.194723
C	-2.591053	-0.639074	-0.929241
O	-2.523363	-0.934457	-2.105139
C	-3.848741	-0.669721	-0.089610
C	-3.812342	-1.691011	1.073494
C	-5.090504	-1.565384	1.915401
C	-3.623077	-3.127061	0.562332
H	4.432681	-1.781257	1.256024
H	3.604620	0.595487	-0.411225
H	2.803406	0.453433	3.649507
H	3.056251	-1.129076	2.898465
H	2.334792	-2.726562	0.922416
H	3.226766	-3.298483	-0.490157
H	1.440265	-0.413730	2.957918
H	1.289737	1.319374	-1.143934
H	-0.390301	-0.274707	-1.924435
H	0.566097	-1.340174	0.784663
H	-1.020812	1.830688	-3.112533
H	-1.768290	3.417871	-2.491667
H	2.560045	2.463290	0.574253
H	0.062789	0.969165	1.548575
H	2.036307	2.410141	2.258813
H	2.945281	-1.049926	-2.531817
H	1.223352	-1.226017	-2.945958
H	2.261487	-2.647636	-2.780791
H	5.356802	-2.241898	-0.797784
H	-4.003408	0.336109	0.319165
H	-4.679075	-0.895207	-0.765222

H	-2.956368	-1.434746	1.710833
H	-5.069379	-2.260361	2.761970
H	-5.210400	-0.552289	2.315125
H	-5.980026	-1.798044	1.316835
H	-3.592402	-3.834362	1.398410
H	-2.693350	-3.241519	-0.005155
H	-4.450767	-3.420657	-0.094894

B3LYP energy = -1155.15649248 a.u.

(2*R*,6*R*,7*S*,8*S*)-**1**, Conf. H

C	4.405662	-0.580871	0.389620
C	3.578503	0.659439	0.169357
C	2.835545	1.328848	1.063141
C	2.868532	1.080315	2.552210
C	2.255742	-1.802348	-0.317380
C	3.498537	-1.860884	0.544822
C	0.394628	1.695413	0.455950
C	0.164837	0.786148	-0.792775
C	-0.048115	-0.722054	-0.487661
C	1.140350	-1.290670	0.230761
O	-0.571287	2.779032	0.358853
C	-1.397304	2.656897	-0.715358
C	-1.001013	1.445601	-1.492135
O	-2.285212	3.444903	-0.944430
C	-1.628111	1.094581	-2.617596
C	1.798550	2.329166	0.584785
C	2.421432	-2.140508	-1.777125
O	5.271293	-0.681036	-0.749646
O	-1.225267	-0.835632	0.355601
C	-2.104940	-1.840427	0.088821
O	-1.968503	-2.620988	-0.831064
C	-3.254292	-1.831564	1.071373
C	-4.252771	-0.665139	0.856003
C	-4.932617	-0.741874	-0.518665
C	-5.291379	-0.657667	1.987352
H	5.014615	-0.498053	1.301315
H	3.493796	0.927665	-0.882931
H	3.640752	0.365310	2.845523
H	1.906700	0.701930	2.923820
H	3.208177	-1.930224	1.599032
H	4.115687	-2.741618	0.320332
H	3.058442	2.018968	3.087694
H	1.053769	0.830837	-1.432581
H	-0.251865	-1.244925	-1.423678
H	1.140925	-1.088901	1.300503
H	-1.346130	0.217275	-3.192341
H	-2.455687	1.696443	-2.981489
H	2.082483	2.753155	-0.385232
H	0.138378	1.158928	1.373706
H	1.708978	3.162843	1.291128
H	1.474529	-2.140756	-2.321766
H	2.867309	-3.137102	-1.883230
H	3.110769	-1.441095	-2.266828
H	5.875763	-1.423411	-0.621194
H	-3.771881	-2.790350	0.970878
H	-2.839275	-1.765068	2.083496

H	-3.685637	0.272880	0.909575
H	-5.617412	0.101614	-0.656031
H	-4.206016	-0.717631	-1.336897
H	-5.512488	-1.667931	-0.616855
H	-5.988824	0.178390	1.868239
H	-4.815375	-0.559617	2.969556
H	-5.879325	-1.584197	1.988754

B3LYP energy = -1155.15630801 a.u.

(2*R*,6*R*,7*S*,8*S*)-**1**, Conf. I

C	-4.125077	-1.529364	-0.169034
C	-3.670853	-0.091971	-0.074834
C	-3.209644	0.702407	-1.054178
C	-3.257745	0.355123	-2.522768
C	-1.708384	-2.012545	0.548757
C	-2.910211	-2.519533	-0.221550
C	-0.940268	1.781612	-0.631081
C	-0.389943	1.092536	0.659115
C	0.206134	-0.328437	0.462171
C	-0.808244	-1.269410	-0.117654
O	-0.331139	3.101747	-0.682456
C	0.563759	3.311651	0.321146
C	0.599952	2.102267	1.194242
O	1.190593	4.339997	0.426241
C	1.413458	2.022393	2.250168
C	-2.472238	1.981640	-0.702754
C	-1.718556	-2.228956	2.040262
O	-4.902519	-1.892289	0.983062
O	1.326831	-0.212171	-0.455173
C	2.519496	-0.756989	-0.086384
O	2.694147	-1.322778	0.972958
C	3.546722	-0.529803	-1.178723
C	4.928072	-1.187096	-0.987952
C	5.698363	-0.618677	0.215284
C	4.848362	-2.721897	-0.943732
H	-4.724816	-1.702105	-1.073470
H	-3.572248	0.256518	0.953883
H	-3.816894	-0.561362	-2.723939
H	-2.249700	0.226579	-2.939794
H	-2.637372	-2.647331	-1.274269
H	-3.270294	-3.484763	0.151606
H	-3.724864	1.169380	-3.090670
H	-1.212719	0.968550	1.373132
H	0.591248	-0.680015	1.420618
H	-0.910812	-1.190432	-1.198708
H	1.459417	1.143827	2.886526
H	2.066785	2.857020	2.487047
H	-2.817826	2.380940	0.257851
H	-0.586193	1.260076	-1.524924
H	-2.655690	2.752816	-1.459965
H	-2.584527	-1.737174	2.500823
H	-0.809659	-1.872062	2.530254
H	-1.823536	-3.297715	2.262405
H	-5.662665	-1.297846	1.042948
H	3.091950	-0.866973	-2.118644
H	3.655920	0.558119	-1.281970

H	5.491866	-0.915782	-1.892234
H	5.206674	-0.876654	1.157179
H	6.716801	-1.022127	0.240740
H	5.775975	0.473582	0.157091
H	4.326888	-3.118841	-1.823177
H	4.320322	-3.064831	-0.049318
H	5.853740	-3.157143	-0.931082

B3LYP energy = -1155.15612817 a.u.

(2*R*,6*R*,7*S*,8*S*)-**1**, Conf. J

C	-3.768356	-1.800205	-0.113357
C	-3.463938	-0.322489	-0.194579
C	-2.985201	0.373012	-1.238494
C	-2.854404	-0.180218	-2.637147
C	-1.393810	-1.923293	0.852027
C	-2.465596	-2.653679	0.069391
C	-0.881558	1.744711	-0.798490
C	-0.394267	1.296976	0.617683
C	0.347254	-0.066587	0.678010
C	-0.514759	-1.179002	0.159336
O	-0.404832	3.106073	-0.984935
C	0.366910	3.543002	0.048101
C	0.438875	2.470151	1.082165
O	0.876086	4.639277	0.058018
C	1.151050	2.619651	2.201946
C	-2.411904	1.761631	-1.022087
C	-1.518521	-1.944946	2.353790
O	-4.607671	-2.082613	1.017760
O	1.536772	0.038550	-0.151071
C	2.730776	-0.316966	0.397355
O	2.852583	-0.699408	1.544264
C	3.861853	-0.180564	-0.599311
C	4.418873	-1.538668	-1.112369
C	5.126668	-2.344242	-0.013193
C	3.337904	-2.373226	-1.816412
H	-4.265794	-2.157975	-1.025595
H	-3.500351	0.174061	0.775662
H	-3.306474	-1.168647	-2.745741
H	-1.802063	-0.262148	-2.941125
H	-2.085846	-2.890521	-0.929983
H	-2.765098	-3.593614	0.546049
H	-3.333023	0.493127	-3.359034
H	-1.266584	1.183544	1.272613
H	0.669037	-0.241229	1.705828
H	-0.525341	-1.253999	-0.926688
H	1.221628	1.842036	2.956320
H	1.694358	3.544550	2.371903
H	-2.883664	2.249679	-0.161392
H	-0.391986	1.152312	-1.576879
H	-2.597278	2.396032	-1.896648
H	-1.528621	-2.981268	2.712678
H	-2.471496	-1.504403	2.672437
H	-0.702454	-1.422762	2.858623
H	-5.423549	-1.570912	0.932623
H	3.518035	0.417908	-1.447189
H	4.665378	0.366199	-0.094229

H	5.172246	-1.266574	-1.864615
H	5.569448	-3.253689	-0.434629
H	5.930203	-1.762451	0.451714
H	4.431099	-2.636819	0.778935
H	3.778218	-3.266901	-2.271745
H	2.840181	-1.802161	-2.608153
H	2.569186	-2.708695	-1.110220

B3LYP energy = -1155.15594692 a.u.

(2*R*,6*R*,7*S*,8*S*)-**1**, Conf. A

C	3.730189	-2.176062	-0.285457
C	3.551200	-0.765398	-0.768269
C	3.631304	0.360791	-0.038832
C	4.264629	0.469536	1.329366
C	1.275480	-2.008637	0.405342
C	2.335874	-2.880289	-0.242237
C	1.552616	1.777787	0.162098
C	0.280540	1.292627	-0.619335
C	-0.303887	-0.021796	-0.020855
C	0.564799	-1.188580	-0.387468
O	1.350838	3.189378	0.443196
C	0.106083	3.621367	0.104455
C	-0.624688	2.506780	-0.566897
O	-0.270273	4.748402	0.325494
C	-1.847177	2.705293	-1.069702
C	2.939718	1.623611	-0.518530
C	1.202005	-2.038508	1.910242
O	4.531238	-2.980180	-1.163940
O	-1.621633	-0.217437	-0.617509
C	-2.542405	-0.878658	0.131816
O	-2.347831	-1.224817	1.279139
C	-3.801713	-1.150518	-0.665857
C	-5.063588	-1.348325	0.191648
C	-5.476258	-0.047202	0.896429
C	-6.208750	-1.898510	-0.671632
H	4.165279	-2.191596	0.721399
H	3.112070	-0.696941	-1.764030
H	4.719393	-0.462409	1.669738
H	3.546022	0.792135	2.093253
H	2.453179	-3.838785	0.275539
H	2.067186	-3.098237	-1.281295
H	5.050011	1.235930	1.306605
H	0.547762	1.088192	-1.663482
H	-0.439126	0.084043	1.056746
H	0.696248	-1.289799	-1.464903
H	-2.386899	1.931024	-1.600025
H	-2.315556	3.677602	-0.945129
H	2.825700	1.625932	-1.607918
H	1.579266	1.288272	1.141328
H	3.520014	2.512248	-0.245677
H	1.058273	-3.070948	2.251473
H	0.378787	-1.442992	2.308572
H	2.140044	-1.689537	2.361966
H	5.391443	-2.552299	-1.269359
H	-3.942547	-0.347624	-1.399092

H	-3.595819	-2.059648	-1.249081
H	-4.821112	-2.090297	0.962272
H	-6.358970	-0.209132	1.524461
H	-4.676784	0.331885	1.540239
H	-5.728151	0.731968	0.165301
H	-7.105912	-2.066961	-0.066355
H	-5.937955	-2.851357	-1.140920
H	-6.476384	-1.195293	-1.470634

B3LYP energy = -1155.15542201 a.u.

(2*R*,6*R*,7*S*,8*S*)-**1**, Conf. B

C	3.199187	-2.645533	-0.369939
C	3.273409	-1.222211	-0.844092
C	3.585524	-0.138131	-0.113317
C	4.269018	-0.163987	1.234896
C	0.857424	-2.013394	0.440548
C	1.699319	-3.068399	-0.253643
C	1.832790	1.659435	0.161844
C	0.460904	1.417113	-0.560915
C	-0.334579	0.239012	0.077982
C	0.276573	-1.071533	-0.321711
O	1.914330	3.086861	0.423726
C	0.761168	3.743938	0.126125
C	-0.196351	2.780972	-0.493002
O	0.615460	4.924682	0.339083
C	-1.380787	3.202487	-0.947303
C	3.135895	1.238091	-0.568711
C	0.853294	-2.032852	1.947382
O	3.783345	-3.580572	-1.288850
O	-1.693646	0.300228	-0.451177
C	-2.689636	-0.149113	0.355989
O	-2.503305	-0.564303	1.481618
C	-4.041210	-0.012599	-0.316412
C	-5.099401	-1.008041	0.191684
C	-6.490798	-0.611068	-0.323423
C	-4.751375	-2.450265	-0.207303
H	3.675501	-2.755216	0.612199
H	2.824553	-1.063455	-1.825219
H	5.180194	0.446510	1.193166
H	4.555694	-1.167616	1.553718
H	1.656394	-4.033901	0.262576
H	1.343089	-3.226287	-1.277074
H	3.644518	0.273207	2.024062
H	0.642152	1.159014	-1.611512
H	-0.391992	0.367789	1.160143
H	0.331697	-1.193284	-1.403637
H	-2.080766	2.539220	-1.439318
H	-1.649263	4.247778	-0.822436
H	2.984339	1.268309	-1.653117
H	1.808120	1.184947	1.148313
H	3.888907	1.994333	-0.319515
H	0.173430	-1.296963	2.380166
H	1.860145	-1.863316	2.351304
H	0.538841	-3.021852	2.302407
H	4.705798	-3.330735	-1.433148
H	-4.374175	1.016933	-0.121401

H	-3.910148	-0.094325	-1.401543
H	-5.103183	-0.948791	1.286920
H	-7.255435	-1.299837	0.051529
H	-6.765796	0.400386	-0.002865
H	-6.530616	-0.637955	-1.419884
H	-5.498193	-3.151282	0.181034
H	-3.776279	-2.752507	0.187200
H	-4.729218	-2.559797	-1.299353

B3LYP energy = -1155.15534206 a.u.

(2*R*,6*R*,7*S*,8*S*)-**1**, Conf. C

C	3.043552	-2.638578	-0.393380
C	3.039857	-1.255536	-0.978686
C	3.445315	-0.120304	-0.384186
C	4.318104	-0.046063	0.847839
C	0.827913	-1.942766	0.676508
C	1.576001	-3.048176	-0.046053
C	1.731840	1.692864	0.007167
C	0.272522	1.410918	-0.496974
C	-0.419808	0.279163	0.320148
C	0.142292	-1.057034	-0.066109
O	1.843320	3.133804	0.161263
C	0.655590	3.775125	-0.006978
C	-0.375763	2.778547	-0.420123
O	0.535742	4.967409	0.150175
C	-1.615526	3.174925	-0.724432
C	2.920887	1.218049	-0.870926
C	1.030978	-1.849783	2.167034
O	3.507764	-3.641738	-1.308778
O	-1.837966	0.305430	-0.022581
C	-2.710535	-0.092141	0.941822
O	-2.368899	-0.402076	2.065179
C	-4.135025	-0.088356	0.431138
C	-4.406135	-1.109161	-0.700988
C	-4.127077	-2.549223	-0.245395
C	-5.848787	-0.956010	-1.204724
H	3.646590	-2.673859	0.522284
H	2.451261	-1.169506	-1.892587
H	4.657586	-1.023442	1.195154
H	3.811500	0.450875	1.684638
H	1.609610	-3.972962	0.540625
H	1.086189	-3.279898	-0.997789
H	5.207631	0.558021	0.627516
H	0.304776	1.088272	-1.545010
H	-0.333040	0.481779	1.388849
H	0.052703	-1.256309	-1.134204
H	-2.374942	2.484425	-1.068638
H	-1.870013	4.226565	-0.626759
H	2.614720	1.166518	-1.921380
H	1.849186	1.284194	1.016371
H	3.693679	1.990850	-0.790119
H	0.783292	-2.810634	2.634238
H	0.406817	-1.086316	2.634592
H	2.081518	-1.645712	2.412878
H	4.399099	-3.403037	-1.596399
H	-4.781731	-0.293704	1.289359

H	-4.364398	0.921124	0.067933
H	-3.726630	-0.872016	-1.529813
H	-4.321497	-3.255811	-1.059606
H	-3.087770	-2.684486	0.071915
H	-4.771072	-2.824638	0.598789
H	-6.049014	-1.649488	-2.028653
H	-6.043766	0.059952	-1.566457
H	-6.569129	-1.172142	-0.405999

B3LYP energy = -1155.15455547 a.u.

(2*R*,6*R*,7*S*,8*S*)-**1**, Conf. D

C	3.996931	-1.753130	-0.366645
C	3.612779	-0.369979	-0.809828
C	3.536631	0.735430	-0.048993
C	4.162615	0.896894	1.317509
C	1.557237	-1.945752	0.373402
C	2.715645	-2.645917	-0.312911
C	1.281937	1.839840	0.204037
C	0.089264	1.185510	-0.579181
C	-0.302410	-0.202110	0.013319
C	0.713334	-1.230888	-0.389518
O	0.876894	3.201866	0.510797
C	-0.414324	3.458243	0.168615
C	-0.974564	2.262615	-0.526774
O	-0.948781	4.516524	0.403667
C	-2.201930	2.298219	-1.054742
C	2.669310	1.900841	-0.488990
C	1.527606	-2.002081	1.878975
O	4.880623	-2.417112	-1.282172
O	-1.595441	-0.568316	-0.555295
C	-2.369697	-1.408379	0.183482
O	-2.054699	-1.805944	1.287421
C	-3.660697	-1.745439	-0.528606
C	-4.839320	-0.810677	-0.141851
C	-6.038835	-1.092311	-1.059359
C	-5.230894	-0.947123	1.336546
H	4.454851	-1.731564	0.629945
H	3.158427	-0.339021	-1.800755
H	4.833472	1.765647	1.309807
H	4.746054	0.029155	1.630078
H	2.977126	-3.588250	0.181316
H	2.457762	-2.879007	-1.351381
H	3.413986	1.096545	2.094345
H	0.383189	1.025158	-1.623589
H	-0.423415	-0.128094	1.095493
H	0.828296	-1.300759	-1.471451
H	-2.617966	1.463613	-1.604641
H	-2.801836	3.195646	-0.931956
H	2.544917	1.916266	-1.577176
H	1.391126	1.347103	1.175539
H	3.122010	2.854464	-0.194668
H	0.633992	-1.541627	2.303416
H	2.413629	-1.520828	2.313579
H	1.548023	-3.048037	2.208389
H	5.674137	-1.875426	-1.387306
H	-3.493708	-1.693077	-1.608682

H	-3.917412	-2.777130	-0.265239
H	-4.515313	0.224510	-0.317694
H	-6.876192	-0.428530	-0.818439
H	-5.784201	-0.943581	-2.114741
H	-6.388796	-2.125255	-0.939915
H	-6.040496	-0.251345	1.582486
H	-4.390288	-0.742408	2.004919
H	-5.583855	-1.963149	1.552061

B3LYP energy = -1155.15370154 a.u.

(2*R*,6*R*,7*S*,8*S*)-**1**, Conf. E

C	3.740633	-2.172039	-0.288553
C	3.561563	-0.765323	-0.771180
C	3.626316	0.355991	-0.035875
C	4.252542	0.463721	1.335459
C	1.269654	-2.002878	0.399503
C	2.331221	-2.869278	-0.252041
C	1.552543	1.784799	0.160121
C	0.277493	1.302548	-0.618270
C	-0.305891	-0.012497	-0.021662
C	0.561596	-1.179008	-0.391609
O	1.356948	3.199269	0.433641
C	0.111236	3.632456	0.102657
C	-0.626880	2.517197	-0.560118
O	-0.262245	4.760819	0.322522
C	-1.853386	2.716627	-1.052632
C	2.938191	1.619477	-0.519128
C	1.197735	-2.035993	1.904392
O	4.608704	-2.870181	-1.189396
O	-1.624671	-0.207579	-0.616873
C	-2.538454	-0.884979	0.125939
O	-2.336486	-1.248957	1.266596
C	-3.800827	-1.149858	-0.669340
C	-5.058920	-1.357333	0.191607
C	-5.469309	-0.063506	0.910995
C	-6.207389	-1.899558	-0.672325
H	4.170417	-2.184884	0.720175
H	3.138031	-0.696888	-1.773078
H	4.714113	-0.466291	1.671675
H	3.531021	0.780118	2.099429
H	2.431294	-3.833924	0.263969
H	2.062355	-3.076035	-1.293615
H	5.034915	1.233175	1.316111
H	0.541845	1.101149	-1.663800
H	-0.439577	0.091197	1.056360
H	0.694884	-1.275748	-1.469160
H	-2.398983	1.942604	-1.577190
H	-2.318774	3.690066	-0.925679
H	2.824847	1.618756	-1.608616
H	1.576455	1.300958	1.142300
H	3.523257	2.506133	-0.249920
H	0.367881	-1.449668	2.302401
H	2.131532	-1.675819	2.356012
H	1.064222	-3.069871	2.245829
H	4.669054	-3.795539	-0.914812
H	-3.945533	-0.339778	-1.393762

H	-3.596841	-2.053080	-1.262372
H	-4.812612	-2.106808	0.953708
H	-6.349510	-0.231971	1.540814
H	-4.667488	0.309517	1.555463
H	-5.724160	0.722829	0.188665
H	-7.102052	-2.074220	-0.065125
H	-5.938170	-2.847684	-1.152008
H	-6.478432	-1.188679	-1.463328

B3LYP energy = -1155.15343546 a.u.

(2*R*,6*R*,7*S*,8*S*)-**1**, Conf. F

C	3.222406	-2.637711	-0.375316
C	3.293346	-1.216730	-0.844840
C	3.579198	-0.135587	-0.102253
C	4.245390	-0.162047	1.254468
C	0.859889	-2.010488	0.430274
C	1.709531	-3.055816	-0.268172
C	1.829797	1.669560	0.162350
C	0.458597	1.424842	-0.560696
C	-0.332332	0.242351	0.074487
C	0.282406	-1.064993	-0.330053
O	1.912405	3.099296	0.413763
C	0.756478	3.752887	0.122169
C	-0.203232	2.786416	-0.488783
O	0.608705	4.933927	0.332762
C	-1.391768	3.204767	-0.935182
C	3.132971	1.240428	-0.561480
C	0.850254	-2.037983	1.936943
O	3.894473	-3.476387	-1.322691
O	-1.692701	0.300182	-0.452449
C	-2.684593	-0.161593	0.352247
O	-2.493583	-0.589482	1.472473
C	-4.038538	-0.020989	-0.314550
C	-5.097330	-1.014702	0.195236
C	-6.489647	-0.612952	-0.313626
C	-4.754755	-2.456984	-0.208335
H	3.693949	-2.746431	0.608738
H	2.867191	-1.060884	-1.835912
H	3.611593	0.270797	2.038733
H	5.155481	0.450610	1.223036
H	1.651237	-4.025421	0.244901
H	1.357510	-3.201773	-1.295122
H	4.534912	-1.165202	1.571874
H	0.641222	1.171186	-1.612212
H	-0.388988	0.366914	1.157180
H	0.344873	-1.179654	-1.412264
H	-2.093603	2.539955	-1.422361
H	-1.661365	4.249649	-0.809256
H	2.986180	1.268658	-1.646616
H	1.801708	1.202974	1.152575
H	3.887944	1.994634	-0.311765
H	1.852441	-1.854325	2.346037
H	0.547746	-3.032503	2.287309
H	0.157436	-1.313877	2.368932
H	3.793400	-4.399586	-1.053005
H	-4.368118	1.009087	-0.116673

H	-3.911526	-0.100477	-1.400380
H	-5.096812	-0.958103	1.290622
H	-7.254727	-1.300341	0.062943
H	-6.760564	0.398579	0.010063
H	-6.533823	-0.637447	-1.409970
H	-5.501972	-3.156881	0.181300
H	-3.778912	-2.762449	0.181830
H	-4.737200	-2.564034	-1.300735

B3LYP energy = -1155.15340035 a.u.

(2*R*,6*R*,7*S*,8*S*)-**1**, Conf. G

C	3.750013	-2.162393	-0.270719
C	3.561916	-0.755968	-0.765277
C	3.637242	0.377183	-0.048427
C	4.284923	0.500656	1.310791
C	1.279392	-2.009495	0.391977
C	2.348385	-2.871906	-0.253405
C	1.545353	1.774911	0.155634
C	0.271423	1.289357	-0.623230
C	-0.308496	-0.028316	-0.027441
C	0.562067	-1.192312	-0.398188
O	1.338718	3.183988	0.445978
C	0.092085	3.613941	0.112488
C	-0.636797	2.500848	-0.563604
O	-0.287722	4.738416	0.340358
C	-1.860379	2.698697	-1.063943
C	2.929858	1.629449	-0.532675
C	1.211639	-2.037852	1.897405
O	4.684483	-2.938315	-1.028439
O	-1.627079	-0.225215	-0.621805
C	-2.545100	-0.889580	0.128350
O	-2.345948	-1.240481	1.273389
C	-3.807722	-1.158084	-0.665222
C	-5.067333	-1.351693	0.196724
C	-5.473111	-0.049153	0.902874
C	-6.217307	-1.897948	-0.662626
H	4.158713	-2.166214	0.741432
H	3.103489	-0.689987	-1.754809
H	5.073028	1.263220	1.267666
H	4.744281	-0.427633	1.654295
H	2.464464	-3.836169	0.253557
H	2.077129	-3.080417	-1.296068
H	3.576169	0.836080	2.078481
H	0.535615	1.089166	-1.669003
H	-0.441420	0.074131	1.050747
H	0.689498	-1.291588	-1.476543
H	-2.398864	1.925796	-1.597540
H	-2.331167	3.669115	-0.933821
H	2.809060	1.623376	-1.621470
H	1.578486	1.279808	1.131888
H	3.503684	2.525261	-0.270440
H	0.377997	-1.457287	2.296004
H	2.143372	-1.668553	2.345859
H	1.089223	-3.071902	2.241692
H	4.478403	-2.848593	-1.969257
H	-3.948707	-0.355150	-1.398341

H	-3.606750	-2.068367	-1.248392
H	-4.824640	-2.094506	0.966456
H	-6.354380	-0.208007	1.533671
H	-4.670342	0.327092	1.544257
H	-5.724536	0.730980	0.172631
H	-7.112881	-2.063425	-0.054219
H	-5.951427	-2.851698	-1.132919
H	-6.485399	-1.193726	-1.460586

B3LYP energy = -1155.15330860 a.u.

(2*R*,6*R*,7*S*,8*S*)-**1**, Conf. H

C	3.207615	-2.644797	-0.353832
C	3.274307	-1.225671	-0.844854
C	3.593209	-0.132446	-0.133061
C	4.304688	-0.143713	1.199787
C	0.852865	-2.010046	0.437917
C	1.697145	-3.064613	-0.253580
C	1.830892	1.655036	0.150072
C	0.452953	1.418167	-0.563598
C	-0.341771	0.240869	0.077521
C	0.265386	-1.071095	-0.323119
O	1.914630	3.080785	0.420633
C	0.761004	3.741070	0.133041
C	-0.201198	2.782985	-0.486653
O	0.617542	4.920697	0.353384
C	-1.386905	3.208833	-0.933485
C	3.127876	1.236895	-0.593803
C	0.863388	-2.020866	1.944933
O	3.926701	-3.584807	-1.158911
O	-1.702176	0.304219	-0.447922
C	-2.695747	-0.149894	0.359871
O	-2.505586	-0.571348	1.482440
C	-4.049558	-0.010402	-0.307314
C	-5.102197	-1.016556	0.191355
C	-6.496417	-0.620389	-0.316586
C	-4.748185	-2.452496	-0.224742
H	3.665141	-2.735804	0.633084
H	2.800517	-1.066629	-1.816497
H	5.222660	0.453121	1.124625
H	4.589738	-1.144921	1.526968
H	1.649553	-4.032976	0.256566
H	1.333943	-3.215794	-1.278062
H	3.704374	0.316740	1.994632
H	0.625872	1.162509	-1.616243
H	-0.395415	0.369570	1.159869
H	0.311005	-1.195548	-1.405435
H	-2.090471	2.549348	-1.425510
H	-1.652947	4.253956	-0.802141
H	2.962877	1.258160	-1.676586
H	1.814102	1.174211	1.133622
H	3.879112	1.999696	-0.360508
H	0.568699	-3.012344	2.309510
H	0.175694	-1.292977	2.378748
H	1.870781	-1.831239	2.338365
H	3.672057	-3.466410	-2.084573
H	-4.386296	1.015096	-0.098195

H	-3.921435	-0.077973	-1.393763
H	-5.104049	-0.969836	1.287181
H	-7.257136	-1.316613	0.052481
H	-6.775291	0.386267	0.015428
H	-6.538431	-0.635662	-1.413196
H	-5.491250	-3.161355	0.156444
H	-3.771199	-2.754921	0.165015
H	-4.727191	-2.549272	-1.318052

B3LYP energy = -1155.15322040 a.u.

(2*R*,6*R*,7*S*,8*S*)-**1**, Conf. I

C	3.783412	-2.112538	0.405991
C	3.694502	-0.600602	0.346545
C	3.431216	0.202770	-0.698613
C	3.451240	-0.222684	-2.145257
C	1.237714	-1.943031	0.726247
C	2.381266	-2.788009	0.202985
C	1.647881	1.698497	0.341237
C	0.345992	1.317610	-0.443493
C	-0.301418	0.020869	0.123483
C	0.593301	-1.151839	-0.148247
O	1.469228	3.062800	0.806780
C	0.255313	3.579813	0.473801
C	-0.497014	2.568031	-0.325897
O	-0.086000	4.693139	0.797482
C	-1.691808	2.858226	-0.849737
C	2.988878	1.636567	-0.419521
C	1.013146	-1.948156	2.216654
O	4.635537	-2.719547	-0.576232
O	-1.566611	-0.157107	-0.580960
C	-2.541703	-0.839016	0.075437
O	-2.436142	-1.215937	1.224626
C	-3.733795	-1.088997	-0.825888
C	-5.052336	-1.344561	-0.076046
C	-5.541243	-0.082874	0.651590
C	-6.119239	-1.872760	-1.046645
H	4.152389	-2.380452	1.406235
H	3.655408	-0.143565	1.336731
H	3.718497	-1.272798	-2.266122
H	4.178051	0.383861	-2.702613
H	2.408442	-3.778500	0.671270
H	2.270394	-2.936314	-0.875460
H	2.478331	-0.043177	-2.621916
H	0.584982	1.131497	-1.497095
H	-0.521788	0.146204	1.185088
H	0.833368	-1.265994	-1.204738
H	-2.239843	2.150797	-1.460219
H	-2.130267	3.833728	-0.659483
H	2.901058	2.209226	-1.350089
H	1.728908	1.086164	1.243904
H	3.725310	2.156780	0.203122
H	0.910969	-2.980442	2.572482
H	0.112284	-1.406946	2.511039
H	1.871677	-1.518741	2.751890
H	5.527854	-2.364668	-0.464563
H	-3.832308	-0.253017	-1.528526

H	-3.469786	-1.965425	-1.435108
H	-4.854657	-2.116295	0.678022
H	-6.466223	-0.286369	1.201966
H	-4.800496	0.278709	1.371215
H	-5.751197	0.724626	-0.061900
H	-7.056358	-2.081092	-0.519374
H	-5.796809	-2.799837	-1.534532
H	-6.338113	-1.138647	-1.832542

B3LYP energy = -1155.15310732 a.u.

(2*R*,6*R*,7*S*,8*S*)-**1**, Conf. J

C	3.340252	-2.548536	0.354277
C	3.516921	-1.045678	0.261943
C	3.331378	-0.226488	-0.787860
C	3.187649	-0.673753	-2.220958
C	0.883552	-1.930035	0.791423
C	1.833435	-2.968330	0.230786
C	1.893028	1.572581	0.301247
C	0.506203	1.411222	-0.409461
C	-0.330262	0.258135	0.218552
C	0.330300	-1.057424	-0.067998
O	1.977072	2.954301	0.741535
C	0.856680	3.670021	0.452105
C	-0.098758	2.792423	-0.287193
O	0.730814	4.831043	0.763389
C	-1.247283	3.279979	-0.766313
C	3.161029	1.266653	-0.523322
C	0.748786	-1.869837	2.291661
O	4.024212	-3.311944	-0.650378
O	-1.639179	0.299838	-0.424752
C	-2.696444	-0.149160	0.300289
O	-2.601425	-0.555385	1.440394
C	-3.988341	-0.023177	-0.482657
C	-5.086411	-1.010141	-0.048542
C	-6.431086	-0.616013	-0.677465
C	-4.711987	-2.457889	-0.400368
H	3.704583	-2.861720	1.342971
H	3.624049	-0.572806	1.239486
H	3.267247	-1.755680	-2.328510
H	3.970002	-0.208719	-2.836102
H	1.709968	-3.942980	0.716800
H	1.643581	-3.106985	-0.837823
H	2.230965	-0.339791	-2.644295
H	0.655094	1.166479	-1.467703
H	-0.473104	0.435769	1.285982
H	0.481938	-1.231084	-1.132789
H	-1.938858	2.670470	-1.335390
H	-1.497481	4.321097	-0.582813
H	3.124511	1.831223	-1.462271
H	1.917040	0.970565	1.214218
H	4.008228	1.659974	0.049495
H	1.694793	-1.577298	2.768355
H	0.500634	-2.864442	2.681418
H	-0.033279	-1.182807	2.619237
H	4.967722	-3.108701	-0.594805
H	-4.334610	1.009353	-0.331378

H	-3.768565	-0.120466	-1.552108
H	-5.177409	-0.937308	1.042121
H	-7.225288	-1.299385	-0.358223
H	-6.728060	0.399244	-0.390132
H	-6.382873	-0.653668	-1.773302
H	-5.489647	-3.152486	-0.064595
H	-3.772741	-2.756519	0.075397
H	-4.602244	-2.581463	-1.485607

B3LYP energy = -1155.15308611 a.u.

(2*R*,6*R*,7*S*,8*S*)-**1**, Conf. K

C	3.504603	-2.363978	-0.272707
C	3.465599	-0.925657	-0.703466
C	3.592650	0.161456	0.076847
C	4.157854	0.165254	1.478984
C	1.039464	-2.018659	0.310093
C	2.056909	-2.949604	-0.323642
C	1.626569	1.734533	0.238790
C	0.361738	1.385524	-0.622815
C	-0.357489	0.102765	-0.107444
C	0.433328	-1.116537	-0.480162
O	1.523381	3.145733	0.570772
C	0.337733	3.691795	0.187912
C	-0.442997	2.667856	-0.566673
O	0.041925	4.836893	0.436813
C	-1.615001	2.985877	-1.125152
C	3.028956	1.493850	-0.382238
C	0.893479	-2.096076	1.808229
O	4.279207	-3.198056	-1.146823
O	-1.653791	0.038024	-0.772824
C	-2.670000	-0.553253	-0.087139
O	-2.558196	-0.948106	1.055089
C	-3.908252	-0.638295	-0.960076
C	-5.208135	-1.116946	-0.282156
C	-5.132483	-2.579012	0.187697
C	-5.663445	-0.175882	0.845003
H	3.887460	-2.454602	0.751243
H	3.090146	-0.783694	-1.717446
H	4.999699	0.867773	1.529758
H	4.521234	-0.812558	1.800123
H	2.069921	-3.933634	0.158147
H	1.823499	-3.104815	-1.382257
H	3.426646	0.511995	2.219992
H	0.666221	1.197356	-1.659762
H	-0.536843	0.179564	0.966167
H	0.606418	-1.189304	-1.554041
H	-2.183672	2.279037	-1.716108
H	-2.009618	3.988337	-0.985355
H	2.969588	1.546008	-1.474716
H	1.566487	1.206739	1.196123
H	3.663997	2.321310	-0.046146
H	0.650655	-3.124528	2.102196
H	0.103596	-1.448802	2.193277
H	1.834596	-1.842201	2.313781
H	5.176858	-2.842526	-1.191162
H	-4.057764	0.352045	-1.408066

H	-3.651823	-1.301523	-1.797541
H	-5.969037	-1.069020	-1.074588
H	-6.110756	-2.912894	0.551056
H	-4.838891	-3.245240	-0.632484
H	-4.409198	-2.696855	0.998970
H	-6.646312	-0.479095	1.222023
H	-5.749543	0.858415	0.490749
H	-4.960256	-0.193270	1.682543

B3LYP energy = -1155.15294253 a.u.

(2*R*,6*R*,7*S*,8*S*)-**1**, Conf. L

C	2.707896	-2.903446	-0.305421
C	2.933797	-1.516081	-0.834648
C	3.401333	-0.453519	-0.157105
C	4.130734	-0.517504	1.165512
C	0.490109	-1.967694	0.556689
C	1.173313	-3.137501	-0.127136
C	1.886165	1.548516	0.115802
C	0.465844	1.456427	-0.547022
C	-0.438092	0.400048	0.156558
C	-0.000558	-0.985583	-0.218367
O	2.151508	2.961748	0.327042
C	1.073445	3.746114	0.056864
C	-0.019727	2.890857	-0.491552
O	1.080587	4.940954	0.238639
C	-1.165536	3.441225	-0.905179
C	3.098713	0.950480	-0.647113
C	0.539037	-1.939620	2.062730
O	3.143006	-3.932584	-1.206227
O	-1.799743	0.606516	-0.326476
C	-2.808668	0.292535	0.529731
O	-2.622188	-0.088620	1.668412
C	-4.167309	0.474235	-0.113161
C	-4.867985	-0.859767	-0.498101
C	-4.040277	-1.675351	-1.502925
C	-5.257757	-1.700267	0.726783
H	3.200920	-3.037211	0.665396
H	2.467986	-1.337073	-1.804329
H	5.109366	-0.030549	1.065707
H	4.301010	-1.537373	1.514577
H	1.033242	-4.074478	0.423415
H	0.764234	-3.282268	-1.132602
H	3.599793	0.024629	1.957954
H	0.571453	1.154223	-1.596427
H	-0.439673	0.567113	1.235089
H	0.003264	-1.144643	-1.296900
H	-1.962922	2.855532	-1.344658
H	-1.301533	4.513831	-0.798946
H	2.906765	0.963024	-1.725442
H	1.842968	1.108251	1.117539
H	3.945442	1.619157	-0.454945
H	1.573285	-1.876982	2.426069
H	0.124811	-2.872870	2.463255
H	-0.033406	-1.115074	2.490965
H	4.082178	-3.799038	-1.391298
H	-4.792075	1.009596	0.609619

H	-4.060433	1.096222	-1.006393
H	-5.796358	-0.553861	-1.000370
H	-4.602383	-2.553133	-1.839406
H	-3.780508	-1.082468	-2.387058
H	-3.106438	-2.032893	-1.053845
H	-5.826604	-2.582955	0.414009
H	-5.880489	-1.126605	1.422007
H	-4.376110	-2.040853	1.278256

B3LYP energy = -1155.15276488 a.u.

(2*R*,6*R*,7*S*,8*S*)-**1**, Conf. M

C	3.062817	-2.628197	-0.401237
C	3.049114	-1.250029	-0.988092
C	3.438804	-0.113613	-0.389110
C	4.310887	-0.033015	0.842778
C	0.833331	-1.935393	0.678644
C	1.584303	-3.034372	-0.049712
C	1.725506	1.703050	0.000870
C	0.263122	1.418116	-0.492664
C	-0.420055	0.283581	0.327074
C	0.144730	-1.050499	-0.062101
O	1.836862	3.145691	0.143617
C	0.646405	3.783814	-0.011742
C	-0.388513	2.783825	-0.408584
O	0.525201	4.976446	0.142709
C	-1.633354	3.177139	-0.695607
C	2.909093	1.221368	-0.879575
C	1.042162	-1.842625	2.168351
O	3.605390	-3.541783	-1.362080
O	-1.840245	0.303787	-0.008852
C	-2.704373	-0.118004	0.952364
O	-2.354242	-0.446685	2.067862
C	-4.132026	-0.114064	0.450089
C	-4.401423	-1.107369	-0.706503
C	-4.107791	-2.556323	-0.289879
C	-5.848242	-0.953749	-1.198009
H	3.667130	-2.655626	0.513361
H	2.469684	-1.171403	-1.907813
H	4.661876	-1.008001	1.185108
H	3.802585	0.458236	1.682103
H	1.607335	-3.960482	0.540695
H	1.092027	-3.259724	-1.002017
H	5.195262	0.577781	0.620566
H	0.289362	1.097901	-1.541684
H	-0.328905	0.486027	1.395484
H	0.054479	-1.248137	-1.130352
H	-2.396386	2.484926	-1.028094
H	-1.888108	4.228531	-0.595906
H	2.598069	1.163718	-1.928312
H	1.848188	1.302589	1.012806
H	3.682538	1.994473	-0.808021
H	2.090096	-1.620852	2.409672
H	0.810928	-2.807306	2.636373
H	0.407470	-1.089742	2.638748
H	3.552921	-4.439846	-1.007066
H	-4.771686	-0.346814	1.306585

H	-4.372083	0.902522	0.114624
H	-3.728861	-0.843221	-1.532795
H	-4.301087	-3.243308	-1.121012
H	-3.065545	-2.690622	0.018245
H	-4.744664	-2.858750	0.550452
H	-6.047756	-1.628294	-2.037680
H	-6.053412	0.069164	-1.533462
H	-6.562177	-1.195252	-0.400792

B3LYP energy = -1155.15260227 a.u.

(2*R*,6*R*,7*S*,8*S*)-**1**, Conf. N

C	3.651075	-2.241431	-0.166429
C	3.491866	-0.787909	-0.540020
C	3.649041	0.291266	0.242431
C	4.256187	0.259883	1.624537
C	1.111337	-2.057737	0.128701
C	2.368574	-2.789082	0.556083
C	1.647266	1.840475	0.391210
C	0.461484	1.142485	-0.361260
C	-0.256673	0.095478	0.544339
C	0.685503	-1.034739	0.891851
O	1.371161	3.266323	0.372549
C	0.217028	3.572405	-0.281824
C	-0.391848	2.311296	-0.798057
O	-0.180502	4.707817	-0.396268
C	-1.488033	2.341202	-1.562212
C	3.064517	1.624187	-0.189234
C	0.546537	-2.464416	-1.210130
O	3.846649	-3.052007	-1.336903
O	-1.442220	-0.350720	-0.161814
C	-2.515757	-0.703973	0.597699
O	-2.537102	-0.617531	1.807046
C	-3.637482	-1.245149	-0.265371
C	-5.039833	-1.079342	0.346674
C	-5.449679	0.399229	0.423016
C	-6.065349	-1.898031	-0.451444
H	4.499060	-2.390442	0.515575
H	3.029841	-0.645593	-1.517267
H	3.518170	0.515372	2.396834
H	5.056910	1.005650	1.704496
H	2.515631	-2.668168	1.633969
H	2.320384	-3.863054	0.343012
H	4.675902	-0.715144	1.881652
H	0.845503	0.605953	-1.234896
H	-0.592301	0.583345	1.463733
H	1.191468	-0.884674	1.844174
H	-1.928605	1.440761	-1.973419
H	-1.956548	3.296637	-1.780699
H	3.018805	1.700841	-1.281522
H	1.644613	1.562360	1.449768
H	3.682866	2.454119	0.171686
H	1.343625	-2.521569	-1.959996
H	-0.242179	-1.800171	-1.561675
H	0.128907	-3.477934	-1.138961
H	4.625730	-2.727741	-1.808875
H	-3.582231	-0.784603	-1.258496

H	-3.416241	-2.312249	-0.411064
H	-4.997697	-1.474239	1.369085
H	-4.744030	0.981989	1.022946
H	-5.499211	0.844563	-0.579209
H	-6.439142	0.504602	0.880902
H	-7.064356	-1.804497	-0.012328
H	-5.806049	-2.962889	-0.468877
H	-6.127296	-1.550872	-1.490790

B3LYP energy = -1155.15257557 a.u.

(2*R*,6*R*,7*S*,8*S*)-**1**, Conf. O

C	2.957775	-2.840985	-0.251443
C	3.176375	-1.382813	-0.574488
C	3.578219	-0.403260	0.250340
C	4.131496	-0.632620	1.636370
C	0.545934	-2.026756	0.048310
C	1.572063	-3.066796	0.452434
C	2.028974	1.595239	0.445335
C	0.712883	1.243434	-0.330963
C	-0.251720	0.380102	0.538296
C	0.374198	-0.960341	0.850418
O	2.122751	3.044766	0.471060
C	1.087262	3.652915	-0.170173
C	0.182757	2.603549	-0.725330
O	0.989535	4.855000	-0.246840
C	-0.868117	2.933415	-1.482612
C	3.352724	1.046537	-0.137478
C	-0.079675	-2.225172	-1.310328
O	2.949621	-3.634814	-1.449285
O	-1.505795	0.269016	-0.182663
C	-2.636411	0.152706	0.566473
O	-2.634542	0.127488	1.778861
C	-3.869973	0.109369	-0.313170
C	-5.082534	-0.578717	0.338372
C	-6.349408	-0.313894	-0.488430
C	-4.842667	-2.085267	0.520612
H	3.733867	-3.224620	0.424492
H	2.784872	-1.098523	-1.551659
H	4.301607	-1.689152	1.854988
H	3.460344	-0.239205	2.411581
H	1.736169	-3.020224	1.533618
H	1.254350	-4.086083	0.204849
H	5.085090	-0.103628	1.756418
H	0.957004	0.659929	-1.224755
H	-0.461692	0.907390	1.473211
H	0.879684	-0.978258	1.814454
H	-1.519184	2.186331	-1.920672
H	-1.079767	3.982723	-1.667918
H	3.340436	1.166796	-1.226730
H	1.944198	1.295182	1.494535
H	4.155526	1.682285	0.253456
H	0.689148	-2.456438	-2.056058
H	-0.666462	-1.368439	-1.639681
H	-0.744244	-3.099408	-1.283185
H	3.790807	-3.507705	-1.908799
H	-4.113725	1.156120	-0.545054

H	-3.612807	-0.363698	-1.268105
H	-5.215162	-0.132705	1.331628
H	-6.552234	0.759236	-0.582064
H	-6.256765	-0.727283	-1.500927
H	-7.223653	-0.779331	-0.020812
H	-5.701300	-2.558947	1.008796
H	-3.961163	-2.279139	1.139433
H	-4.698438	-2.578718	-0.449367

B3LYP energy = -1155.15250379 a.u.

(2*R*,6*R*,7*S*,8*S*)-**1**, Conf. P

C	3.075652	-2.620998	-0.381637
C	3.057494	-1.240773	-0.976018
C	3.454184	-0.097831	-0.393187
C	4.339482	-0.010762	0.827963
C	0.834607	-1.943878	0.662622
C	1.595879	-3.039693	-0.060306
C	1.720703	1.695267	0.002994
C	0.261200	1.409609	-0.499362
C	-0.425252	0.272255	0.315002
C	0.141197	-1.060651	-0.076108
O	1.824877	3.135649	0.167476
C	0.634637	3.772790	0.002828
C	-0.392603	2.774162	-0.416006
O	0.509183	4.963577	0.166536
C	-1.633777	3.166981	-0.719078
C	2.909625	1.231881	-0.881635
C	1.042656	-1.850591	2.152679
O	3.699916	-3.613038	-1.203120
O	-1.844316	0.294664	-0.024093
C	-2.712707	-0.109487	0.941566
O	-2.366073	-0.425851	2.061511
C	-4.139376	-0.103823	0.437058
C	-4.415333	-1.119554	-0.698425
C	-4.134001	-2.561608	-0.250557
C	-5.860318	-0.964509	-1.194877
H	3.655191	-2.638214	0.543233
H	2.452579	-1.154055	-1.881690
H	5.228332	0.586805	0.588431
H	4.683136	-0.984838	1.179723
H	1.626416	-3.971356	0.515339
H	1.106129	-3.261619	-1.017053
H	3.844033	0.498792	1.664002
H	0.292466	1.091161	-1.548749
H	-0.336437	0.471353	1.384161
H	0.049050	-1.257071	-1.144762
H	-2.390309	2.475171	-1.067058
H	-1.892571	4.217083	-0.616315
H	2.597187	1.172902	-1.930009
H	1.843118	1.279759	1.008753
H	3.673648	2.013983	-0.809678
H	0.817150	-2.817143	2.619099
H	0.404570	-1.100962	2.623723
H	2.089405	-1.624145	2.394853
H	3.335056	-3.556570	-2.097212
H	-4.782220	-0.313365	1.297149

H	-4.370626	0.907200	0.079495
H	-3.739661	-0.878367	-1.529283
H	-4.332339	-3.264676	-1.066934
H	-3.093295	-2.698087	0.061717
H	-4.774049	-2.840866	0.595316
H	-6.064487	-1.654683	-2.020621
H	-6.056993	0.052890	-1.551549
H	-6.576869	-1.183823	-0.393646

B3LYP energy = -1155.15242755 a.u.

(2*S*,6*R*,7*S*,8*S*)-**1**, Conf. A

C	-3.836599	-2.079490	0.123347
C	-3.699888	-0.582079	0.000711
C	-3.408741	0.315459	0.955223
C	-3.409236	0.019987	2.433663
C	-1.323283	-1.993837	-0.460983
C	-2.435728	-2.789265	0.193781
C	-1.644495	1.670137	-0.301317
C	-0.312172	1.314995	0.441277
C	0.286496	-0.029205	-0.066831
C	-0.610154	-1.166122	0.322745
O	-1.460006	2.988644	-0.882119
C	-0.222967	3.502513	-0.642231
C	0.545013	2.537462	0.199300
O	0.124251	4.580573	-1.064037
C	1.764438	2.843196	0.652866
C	-2.939031	1.706131	0.536960
C	-1.206419	-2.094840	-1.959299
O	-4.512073	-2.622261	-1.021919
O	1.577359	-0.181579	0.596729
C	2.528557	-0.894506	-0.061781
O	2.383163	-1.325185	-1.187168
C	3.753498	-1.102914	0.805929
C	5.057968	-1.309533	0.016484
C	5.474063	-0.031794	-0.727989
C	6.173737	-1.792443	0.955106
H	-4.388970	-2.360713	1.030648
H	-3.660136	-0.252607	-1.038539
H	-4.019413	0.757772	2.969962
H	-2.396980	0.091272	2.854395
H	-2.562796	-3.776917	-0.262849
H	-2.194833	-2.938096	1.251276
H	-3.798820	-0.973670	2.667551
H	-0.500958	1.202542	1.515513
H	0.463574	0.019768	-1.142629
H	-0.780695	-1.217521	1.398525
H	2.326091	2.172610	1.291908
H	2.209736	3.793848	0.373598
H	-2.779962	2.345639	1.413084
H	-1.791906	0.993693	-1.147338
H	-3.698280	2.201075	-0.079339
H	-0.364551	-1.527806	-2.360287
H	-2.131254	-1.767463	-2.450530
H	-1.067387	-3.144441	-2.245175
H	-5.376412	-2.196265	-1.101638

H	3.843232	-0.266099	1.508325
H	3.539582	-1.991500	1.417220
H	4.867095	-2.090735	-0.729467
H	6.388328	-0.200167	-1.307290
H	4.697380	0.298526	-1.424578
H	5.674296	0.785592	-0.023014
H	7.101790	-1.965871	0.399892
H	5.902760	-2.729599	1.454883
H	6.387598	-1.047524	1.732231

B3LYP energy = -1155.15499413 a.u.

(2*S*,6*R*,7*S*,8*S*)-**1**, Conf. B

C	-3.223395	-2.651700	0.170194
C	-3.430587	-1.158471	0.105460
C	-3.271104	-0.246044	1.076866
C	-3.096447	-0.576221	2.537615
C	-0.838734	-1.990772	-0.567185
C	-1.699036	-3.031238	0.122803
C	-1.947486	1.505974	-0.231098
C	-0.517762	1.436205	0.404858
C	0.321851	0.271620	-0.195486
C	-0.274461	-1.044925	0.203793
O	-2.103886	2.849981	-0.759672
C	-0.995568	3.619170	-0.582300
C	0.029521	2.824694	0.157613
O	-0.928463	4.759825	-0.976075
C	1.184958	3.379985	0.535899
C	-3.151628	1.225866	0.691527
C	-0.807210	-2.022814	-2.072713
O	-3.840181	-3.297622	-0.954507
O	1.659155	0.392155	0.377556
C	2.693734	-0.074965	-0.368605
O	2.559071	-0.546000	-1.479210
C	4.017574	0.113186	0.345662
C	5.060980	-0.970977	0.017482
C	6.438212	-0.562833	0.560758
C	4.635149	-2.343464	0.561096
H	-3.635138	-3.076127	1.096349
H	-3.543335	-0.798798	-0.918286
H	-2.100335	-0.283895	2.896903
H	-3.229767	-1.639407	2.751244
H	-1.636420	-4.009950	-0.365176
H	-1.356977	-3.150301	1.156030
H	-3.819789	-0.016024	3.143734
H	-0.597638	1.257748	1.483723
H	0.405700	0.385157	-1.277756
H	-0.355419	-1.159790	1.285063
H	1.928771	2.833364	1.102960
H	1.387424	4.413374	0.268864
H	-3.072253	1.858065	1.583709
H	-2.004699	0.841079	-1.097375
H	-4.045109	1.557467	0.150223
H	-0.473581	-3.010950	-2.411597
H	-0.131029	-1.280095	-2.499504
H	-1.811731	-1.882387	-2.491309
H	-4.781005	-3.074690	-0.961234

H	4.395607	1.096648	0.032164
H	3.839265	0.175469	1.424924
H	5.123710	-1.041432	-1.075283
H	7.193135	-1.316871	0.313298
H	6.770397	0.393246	0.140201
H	6.418779	-0.460129	1.653301
H	5.374705	-3.109201	0.302961
H	3.672745	-2.659990	0.146618
H	4.547053	-2.321951	1.655084

B3LYP energy = -1155.15492805 a.u.

(2*S*,6*R*,7*S*,8*S*)-**1**, Conf. C

C	-3.185860	-2.545790	0.230233
C	-3.334097	-1.045539	0.263750
C	-3.050980	-0.193351	1.261380
C	-2.755364	-0.609079	2.680311
C	-0.873459	-1.931609	-0.742754
C	-1.693682	-2.977829	-0.012778
C	-1.806359	1.581250	-0.095616
C	-0.317207	1.428683	0.366000
C	0.397667	0.274509	-0.394762
C	-0.197912	-1.042011	0.005903
O	-1.967989	2.956200	-0.535006
C	-0.818512	3.678659	-0.442679
C	0.251764	2.810527	0.132067
O	-0.752635	4.837547	-0.779069
C	1.463578	3.304094	0.404163
C	-2.910230	1.292703	0.942350
C	-0.998120	-1.892655	-2.243158
O	-3.953179	-3.106656	-0.846435
O	1.800286	0.318405	0.004764
C	2.713954	-0.114368	-0.905034
O	2.421688	-0.470527	-2.028267
C	4.115374	-0.086061	-0.334300
C	4.341436	-1.072690	0.837375
C	4.087623	-2.526680	0.412116
C	5.760139	-0.898353	1.398815
H	-3.506846	-3.005143	1.175346
H	-3.526330	-0.627040	-0.725145
H	-1.714455	-0.386941	2.952903
H	-2.926247	-1.674062	2.854503
H	-1.728108	-3.928991	-0.554936
H	-1.242730	-3.167190	0.966710
H	-3.384865	-0.046585	3.381425
H	-0.278069	1.189816	1.435502
H	0.349405	0.447516	-1.471198
H	-0.171894	-1.206551	1.083520
H	2.243574	2.699577	0.851249
H	1.677018	4.343484	0.171429
H	-2.704866	1.869389	1.851798
H	-1.984722	0.965242	-0.981072
H	-3.843479	1.688164	0.525024
H	-0.350266	-1.145428	-2.704595
H	-2.036627	-1.713166	-2.548146
H	-0.725969	-2.870628	-2.658290
H	-4.877352	-2.844432	-0.736584

H	4.799395	-0.312559	-1.157560
H	4.324159	0.934349	0.009863
H	3.625755	-0.814778	1.628782
H	4.249576	-3.208674	1.254056
H	3.063695	-2.675927	0.053768
H	4.768564	-2.822972	-0.395163
H	5.926100	-1.566162	2.251151
H	5.936097	0.128783	1.738053
H	6.515607	-1.135307	0.639410

B3LYP energy = -1155.15408544 a.u.

(2*S*,6*R*,7*S*,8*S*)-**1**, Conf. D

C	-3.871403	-2.051374	0.090306
C	-3.729980	-0.556163	-0.010936
C	-3.401442	0.318756	0.950053
C	-3.370441	-0.000626	2.423451
C	-1.338183	-1.990662	-0.418235
C	-2.465397	-2.756315	0.243374
C	-1.637131	1.678118	-0.294267
C	-0.304503	1.319187	0.447423
C	0.284013	-0.032865	-0.051157
C	-0.617491	-1.159982	0.355402
O	-1.449703	2.998557	-0.870870
C	-0.208566	3.504407	-0.640096
C	0.560288	2.534344	0.195253
O	0.142967	4.580754	-1.063195
C	1.785669	2.832617	0.637602
C	-2.933190	1.712264	0.541626
C	-1.221212	-2.109223	-1.915456
O	-4.539651	-2.460869	-1.110152
O	1.578967	-0.187497	0.604372
C	2.517806	-0.917311	-0.052561
O	2.356639	-1.366454	-1.168799
C	3.752851	-1.118901	0.802341
C	5.050079	-1.312384	-0.002791
C	5.446350	-0.028959	-0.748291
C	6.180844	-1.788397	0.921204
H	-4.477772	-2.341511	0.960358
H	-3.743546	-0.210883	-1.044417
H	-2.355281	0.084434	2.834010
H	-3.739648	-1.004613	2.647130
H	-2.557050	-3.774545	-0.158169
H	-2.258867	-2.848642	1.315326
H	-3.989165	0.715519	2.979284
H	-0.491849	1.217052	1.522966
H	0.453779	0.004884	-1.128536
H	-0.786657	-1.195858	1.431652
H	2.348897	2.159528	1.272541
H	2.234090	3.780265	0.353138
H	-2.775989	2.348331	1.420694
H	-1.785812	1.004405	-1.142652
H	-3.690680	2.208643	-0.075336
H	-1.108195	-3.164712	-2.192544
H	-0.361755	-1.570041	-2.317197
H	-2.134691	-1.758810	-2.411740
H	-4.707638	-3.411183	-1.069355

H	3.843912	-0.284100	1.506747
H	3.553034	-2.012129	1.411627
H	4.856852	-2.093137	-0.748608
H	6.354713	-0.187830	-1.339416
H	4.658510	0.297290	-1.434284
H	5.648339	0.787968	-0.043346
H	7.103729	-1.952185	0.354543
H	5.923784	-2.729322	1.421265
H	6.397446	-1.043952	1.697990

B3LYP energy = -1155.15367498 a.u.

H	4.161498	1.091182	0.603600
H	3.548207	-0.344779	1.408601
H	5.301133	-0.377349	-1.102941
H	7.192892	-1.024710	0.410683
H	6.571328	0.580211	0.823922
H	6.145136	-0.821449	1.821308
H	5.643195	-2.795331	-0.592626
H	3.930028	-2.444551	-0.845436
H	4.560217	-2.668359	0.801121

B3LYP energy = -1155.15364991 a.u.

(2*S*,6*R*,7*S*,8*S*)-**1**, Conf. E

C	-3.051923	-2.694797	-0.049435
C	-3.358899	-1.237901	-0.264640
C	-3.378876	-0.253628	0.651659
C	-3.387856	-0.457879	2.148211
C	-0.610069	-2.006860	-0.153836
C	-1.634415	-3.024890	-0.616489
C	-1.999711	1.501571	-0.580315
C	-0.659179	1.255120	0.188660
C	0.319283	0.356731	-0.629489
C	-0.317861	-0.974326	-0.964044
O	-2.007655	2.904448	-0.955254
C	-0.997910	3.605127	-0.365935
C	-0.156422	2.658283	0.426778
O	-0.868896	4.798353	-0.504480
C	0.834335	3.096189	1.209314
C	-3.298306	1.200829	0.197832
C	-0.105610	-2.183866	1.257488
O	-3.953054	-3.561461	-0.753802
O	1.528472	0.238638	0.165414
C	2.698053	0.074125	-0.512347
O	2.764339	0.003197	-1.720783
C	3.878596	0.041843	0.437529
C	5.089430	-0.747083	-0.092264
C	6.318467	-0.485797	0.790807
C	4.783619	-2.249524	-0.188806
H	-3.072287	-2.942838	1.019041
H	-3.396880	-0.960229	-1.319261
H	-2.479098	-0.056970	2.616318
H	-3.485298	-1.504505	2.443251
H	-1.719665	-3.019472	-1.707980
H	-1.375789	-4.045350	-0.310280
H	-4.227754	0.093951	2.589583
H	-0.852268	0.739613	1.133075
H	0.588601	0.864685	-1.559683
H	-0.723522	-1.009400	-1.973172
H	1.435478	2.420601	1.807518
H	1.050964	4.159821	1.252449
H	-3.350491	1.873707	1.061560
H	-2.015441	0.950725	-1.522146
H	-4.139173	1.463371	-0.454511
H	0.540945	-1.370383	1.585220
H	0.466948	-3.118457	1.327187
H	-0.937512	-2.282124	1.965497
H	-4.855523	-3.356606	-0.474738

(2*S*,6*R*,7*S*,8*S*)-**1**, Conf. F

C	-3.715435	-2.087232	-0.091266
C	-3.651410	-0.595959	-0.280592
C	-3.428188	0.345927	0.653127
C	-3.497283	0.124704	2.145583
C	-1.178920	-2.026376	-0.187627
C	-2.423187	-2.748577	-0.667425
C	-1.653718	1.722125	-0.553647
C	-0.414558	1.144134	0.207122
C	0.310304	0.040960	-0.623913
C	-0.637699	-1.083631	-0.979121
O	-1.316067	3.087938	-0.913137
C	-0.163948	3.511581	-0.320540
C	0.419803	2.377053	0.457135
O	0.254290	4.638053	-0.445921
C	1.489959	2.546248	1.239195
C	-2.985394	1.742214	0.226006
C	-0.740063	-2.348799	1.219991
O	-4.801718	-2.691714	-0.808068
O	1.446927	-0.391496	0.168963
C	2.551357	-0.808634	-0.510463
O	2.637166	-0.793684	-1.719614
C	3.615137	-1.319146	0.440464
C	5.052064	-1.189470	-0.095904
C	5.478851	0.281720	-0.210971
C	6.022434	-1.980514	0.793715
H	-3.798044	-2.340693	0.973002
H	-3.613229	-0.299623	-1.330249
H	-2.521711	0.283311	2.623785
H	-3.851266	-0.870617	2.420965
H	-2.501554	-2.702138	-1.758430
H	-2.427273	-3.806775	-0.380630
H	-4.179603	0.858318	2.594113
H	-0.725783	0.683967	1.148603
H	0.703421	0.478953	-1.545307
H	-1.039709	-0.997125	-1.986605
H	1.906728	1.734848	1.825288
H	1.963944	3.522126	1.293803
H	-2.865810	2.389414	1.102649
H	-1.805942	1.203351	-1.501754
H	-3.733871	2.220388	-0.416391
H	-0.426627	-3.399951	1.274828
H	-1.572076	-2.241635	1.926742
H	0.091764	-1.732454	1.559932
H	-5.625984	-2.274524	-0.523811

H	3.506413	-0.814882	1.407538
H	3.377133	-2.377864	0.617840
H	5.064755	-1.627501	-1.101378
H	6.493518	0.361137	-0.615708
H	4.813591	0.843209	-0.874124
H	5.475394	0.768886	0.772870
H	7.045700	-1.913192	0.409108
H	5.752179	-3.041748	0.840901
H	6.028513	-1.589915	1.819433

B3LYP energy = -1155.15364933 a.u.

(2*S*,6*R*,7*S*,8*S*)-**1**, Conf. G

C	-3.259548	-2.639949	0.142864
C	-3.464813	-1.149048	0.105411
C	-3.260454	-0.253347	1.081776
C	-3.044342	-0.603606	2.532483
C	-0.848673	-1.991192	-0.532514
C	-1.724018	-3.012771	0.164015
C	-1.950789	1.510290	-0.217358
C	-0.515941	1.442838	0.408491
C	0.317330	0.270179	-0.184171
C	-0.281238	-1.040618	0.230225
O	-2.113586	2.856103	-0.740463
C	-1.001354	3.622353	-0.582605
C	0.033536	2.827439	0.143640
O	-0.936514	4.762084	-0.979940
C	1.197061	3.381025	0.498912
C	-3.148983	1.222278	0.710352
C	-0.813511	-2.036495	-2.037852
O	-3.911090	-3.151253	-1.027321
O	1.658180	0.392165	0.380993
C	2.686462	-0.091412	-0.362731
O	2.543586	-0.582868	-1.463578
C	4.014950	0.108119	0.339885
C	5.066539	-0.964817	0.002629
C	6.444196	-0.541918	0.533409
C	4.660245	-2.341580	0.550498
H	-3.715828	-3.087850	1.037318
H	-3.638858	-0.783132	-0.906235
H	-3.153671	-1.672645	2.730655
H	-3.768733	-0.070487	3.161650
H	-1.617310	-4.010821	-0.281760
H	-1.419404	-3.093971	1.213285
H	-2.048843	-0.296417	2.880288
H	-0.589334	1.276785	1.489855
H	0.396170	0.372746	-1.267867
H	-0.363650	-1.143279	1.312163
H	1.948788	2.835845	1.056752
H	1.397548	4.412286	0.222308
H	-3.066198	1.848126	1.606792
H	-2.012678	0.847729	-1.085368
H	-4.045743	1.554722	0.175397
H	-0.114480	-1.316179	-2.466063
H	-1.812045	-1.870110	-2.460968
H	-0.503491	-3.035279	-2.369058
H	-3.867450	-4.116061	-1.016739

H	4.380153	1.096015	0.024936
H	3.844758	0.168434	1.420662
H	5.120300	-1.035359	-1.090608
H	7.204924	-1.287813	0.279167
H	6.762122	0.417603	0.109730
H	6.433532	-0.439189	1.626057
H	5.405478	-3.099603	0.285901
H	3.697440	-2.668039	0.144800
H	4.581969	-2.320510	1.645262

B3LYP energy = -1155.15362784 a.u.

(2*S*,6*R*,7*S*,8*S*)-**1**, Conf. H

C	-4.071865	-1.666499	0.219133
C	-3.735987	-0.208731	0.025219
C	-3.327211	0.688707	0.935806
C	-3.364489	0.469296	2.427119
C	-1.576300	-1.942695	-0.382247
C	-2.778149	-2.555708	0.309643
C	-1.398115	1.729692	-0.373544
C	-0.128284	1.227523	0.393430
C	0.286548	-0.197523	-0.076196
C	-0.749075	-1.189797	0.363526
O	-1.031226	2.981293	-1.013131
C	0.258119	3.341195	-0.768390
C	0.879912	2.322915	0.129295
O	0.749290	4.345638	-1.227211
C	2.114529	2.492910	0.611458
C	-2.675993	1.981786	0.452604
C	-1.491221	-2.115607	-1.875914
O	-4.831502	-2.163162	-0.894108
O	1.558916	-0.498193	0.571397
C	2.366991	-1.394933	-0.056706
O	2.099411	-1.893726	-1.131252
C	3.629588	-1.656298	0.734504
C	4.819485	-0.750769	0.315144
C	5.984344	-0.948426	1.297015
C	5.267442	-1.007674	-1.130893
H	-4.643541	-1.829169	1.143156
H	-3.654927	0.061272	-1.028631
H	-3.873471	1.305420	2.923256
H	-2.351092	0.429960	2.849420
H	-3.041695	-3.533769	-0.107312
H	-2.547599	-2.695672	1.370712
H	-3.878767	-0.452956	2.707971
H	-0.336718	1.168740	1.468002
H	0.446304	-0.207351	-1.155993
H	-0.913142	-1.175912	1.441567
H	2.567387	1.783200	1.293321
H	2.684504	3.368879	0.314763
H	-2.433275	2.636963	1.297466
H	-1.636134	1.041712	-1.189072
H	-3.361607	2.542854	-0.192740
H	-2.379132	-1.703867	-2.371518
H	-1.476691	-3.184821	-2.119956
H	-0.594691	-1.666425	-2.306254
H	-5.629590	-1.625382	-0.987600

H	3.418406	-1.509575	1.798048
H	3.901172	-2.705236	0.574120
H	4.486381	0.293585	0.391798
H	6.828047	-0.301096	1.034347
H	5.689391	-0.716303	2.326556
H	6.342037	-1.985362	1.274823
H	6.083607	-0.329703	-1.403618
H	4.452229	-0.865191	-1.845229
H	5.631899	-2.035771	-1.246993

B3LYP energy = -1155.15336451 a.u.

(2*S*,6*R*,7*S*,8*S*)-**1**, Conf. I

C	-3.476699	-2.209717	0.333710
C	-3.624911	-0.725236	0.139125
C	-3.319647	0.240948	1.023996
C	-3.113513	0.023977	2.503924
C	-1.247311	-1.890814	-0.997263
C	-2.443523	-2.782287	-0.681113
C	-1.731295	1.653131	-0.344033
C	-0.392136	1.291867	0.382797
C	0.264172	0.006502	-0.205173
C	-0.648873	-1.172094	-0.032065
O	-1.565125	2.987319	-0.890281
C	-0.349489	3.527419	-0.599453
C	0.427806	2.551516	0.221938
O	-0.024011	4.630177	-0.971211
C	1.626413	2.870396	0.719663
C	-3.019768	1.642487	0.508755
C	-0.883805	-1.816511	-2.460292
O	-4.685889	-2.945028	0.077402
O	1.495924	-0.195124	0.551493
C	2.512164	-0.833015	-0.086267
O	2.469119	-1.158439	-1.255007
C	3.663810	-1.107553	0.859705
C	5.023933	-1.287687	0.163863
C	5.512591	0.024278	-0.468811
C	6.056801	-1.842253	1.156222
H	-3.137705	-2.424519	1.354519
H	-3.810271	-0.431095	-0.895867
H	-3.351898	-0.990837	2.828874
H	-3.757256	0.712476	3.066193
H	-3.000106	-2.955274	-1.609449
H	-2.123477	-3.773315	-0.334655
H	-2.084361	0.245794	2.816091
H	-0.577019	1.101452	1.444905
H	0.532806	0.172125	-1.249555
H	-0.954557	-1.329211	1.000544
H	2.190570	2.186566	1.342760
H	2.052394	3.844086	0.495249
H	-2.908273	2.355037	1.334198
H	-1.879334	0.994085	-1.203339
H	-3.829812	2.014370	-0.128728
H	-1.661985	-1.294959	-3.034995
H	-0.823376	-2.830311	-2.874820
H	0.076986	-1.331245	-2.637921
H	-5.387591	-2.579297	0.632309

H	3.705571	-0.312821	1.613926
H	3.393531	-2.025006	1.402163
H	4.884256	-2.020864	-0.639952
H	6.467237	-0.126022	-0.984482
H	4.797357	0.408266	-1.202571
H	5.668167	0.795203	0.297165
H	7.023839	-1.997101	0.665856
H	5.737152	-2.802993	1.576043
H	6.216703	-1.147947	1.991015

B3LYP energy = -1155.15305279 a.u.

(2*S*,6*R*,7*S*,8*S*)-**1**, Conf. J

C	-2.835371	-2.722106	0.347594
C	-3.321080	-1.305277	0.207950
C	-3.188897	-0.313500	1.107303
C	-2.854890	-0.509366	2.566882
C	-0.806247	-1.876440	-1.071327
C	-1.748533	-3.024842	-0.726805
C	-2.031915	1.453172	-0.282731
C	-0.608682	1.387606	0.367728
C	0.286170	0.292947	-0.287584
C	-0.331703	-1.063351	-0.112483
O	-2.200358	2.803358	-0.788175
C	-1.119753	3.594823	-0.545109
C	-0.099119	2.801519	0.203433
O	-1.069025	4.749386	-0.898215
C	1.025109	3.371292	0.648136
C	-3.239813	1.129653	0.624443
C	-0.550100	-1.689795	-2.547046
O	-3.860419	-3.704366	0.117495
O	1.567244	0.357109	0.409979
C	2.668779	-0.019829	-0.289943
O	2.635322	-0.371254	-1.451447
C	3.923712	0.105361	0.551337
C	5.053707	-0.853883	0.135928
C	6.363497	-0.465237	0.837305
C	4.682521	-2.316773	0.422861
H	-2.409240	-2.878448	1.346058
H	-3.624179	-1.039879	-0.806900
H	-1.881155	-0.075659	2.830282
H	-2.849419	-1.557914	2.871132
H	-2.294957	-3.308584	-1.633604
H	-1.191879	-3.919546	-0.419814
H	-3.597735	0.011796	3.184189
H	-0.688901	1.143219	1.431888
H	0.459911	0.535993	-1.336975
H	-0.535830	-1.309367	0.927952
H	1.761540	2.820233	1.221084
H	1.210093	4.419738	0.432290
H	-3.254245	1.831472	1.466360
H	-2.072964	0.799828	-1.158109
H	-4.143268	1.319270	0.033962
H	-0.276470	-2.652476	-2.996178
H	0.259486	-0.988746	-2.755275
H	-1.458418	-1.354676	-3.067120
H	-4.598688	-3.518311	0.712873

H	4.260962	1.146043	0.441301
H	3.661699	-0.024723	1.607552
H	5.192280	-0.743924	-0.946556
H	7.180498	-1.127083	0.530489
H	6.658654	0.562977	0.598284
H	6.267534	-0.541749	1.927964
H	5.483540	-2.990966	0.100790
H	3.770063	-2.610415	-0.105439
H	4.524629	-2.477818	1.497203

B3LYP energy = -1155.15295549 a.u.

(2*S*,6*R*,7*S*,8*S*)-**1**, Conf. K

C	-3.224444	-2.533308	0.209444
C	-3.371783	-1.036666	0.267778
C	-3.041748	-0.202404	1.263943
C	-2.695331	-0.640235	2.664914
C	-0.879273	-1.934476	-0.696889
C	-1.710372	-2.957216	0.050853
C	-1.809113	1.583397	-0.081294
C	-0.315661	1.435301	0.370110
C	0.394121	0.274384	-0.384943
C	-0.196991	-1.037525	0.036684
O	-1.977190	2.959457	-0.516198
C	-0.827355	3.681780	-0.441633
C	0.251332	2.815336	0.120339
O	-0.765314	4.840174	-0.781078
C	1.466930	3.310097	0.372652
C	-2.908192	1.286744	0.959593
C	-1.009392	-1.913775	-2.197393
O	-4.012624	-2.958788	-0.909912
O	1.801185	0.323571	-0.001275
C	2.702990	-0.130122	-0.912154
O	2.396461	-0.512328	-2.023166
C	4.111672	-0.089886	-0.360476
C	4.355219	-1.059777	0.821715
C	4.097877	-2.519956	0.420445
C	5.781382	-0.875754	1.360735
H	-3.602778	-3.013030	1.123540
H	-3.627110	-0.611406	-0.702554
H	-2.839823	-1.712188	2.821431
H	-3.319843	-0.107976	3.393806
H	-1.687508	-3.939408	-0.440028
H	-1.300904	-3.088243	1.058542
H	-1.653474	-0.400624	2.917511
H	-0.268508	1.207271	1.441696
H	0.334335	0.435640	-1.462563
H	-0.164747	-1.187484	1.115847
H	2.253571	2.708054	0.811273
H	1.676286	4.348599	0.132333
H	-2.701176	1.857381	1.872566
H	-1.991356	0.968626	-0.967148
H	-3.843395	1.681841	0.546900
H	-2.044040	-1.711343	-2.500463
H	-0.760445	-2.903025	-2.601231
H	-0.343809	-1.189222	-2.669469
H	-4.001847	-3.923581	-0.954985

H	4.785433	-0.326906	-1.189205
H	4.322449	0.935595	-0.033280
H	3.650153	-0.791724	1.619277
H	4.272753	-3.189813	1.269545
H	3.069301	-2.675580	0.078421
H	4.767976	-2.826480	-0.392084
H	5.960828	-1.532275	2.219077
H	5.959992	0.155981	1.684132
H	6.526341	-1.121002	0.593607

B3LYP energy = -1155.15272896 a.u.

(2*S*,6*R*,7*S*,8*S*)-**1**, Conf. L

C	-3.488354	-2.413485	0.079313
C	-3.535356	-0.916055	-0.093313
C	-3.421054	0.042133	0.839697
C	-3.477503	-0.203699	2.326287
C	-0.979913	-2.020689	-0.393750
C	-2.013465	-2.935725	0.234154
C	-1.775704	1.575727	-0.374760
C	-0.450053	1.413455	0.442822
C	0.341656	0.146617	0.004928
C	-0.419230	-1.085815	0.393216
O	-1.729162	2.891483	-0.988213
C	-0.584199	3.568206	-0.700185
C	0.254791	2.731157	0.208123
O	-0.357056	4.672518	-1.135531
C	1.398262	3.202320	0.714593
C	-3.107395	1.468555	0.396425
C	-0.772782	-2.147458	-1.880370
O	-4.035902	-3.073877	-1.072673
O	1.609000	0.183707	0.725432
C	2.678517	-0.410197	0.129126
O	2.633002	-0.900118	-0.979985
C	3.884326	-0.353102	1.049150
C	5.196623	-0.965300	0.520226
C	5.089277	-2.480948	0.284299
C	5.737251	-0.229763	-0.717053
H	-4.042445	-2.734565	0.972100
H	-3.475823	-0.617850	-1.140926
H	-2.513136	0.016370	2.803980
H	-3.747623	-1.232371	2.576230
H	-1.991921	-3.942183	-0.197652
H	-1.807464	-3.026861	1.305571
H	-4.212853	0.462649	2.794959
H	-0.678903	1.301772	1.509331
H	0.557818	0.187961	-1.064181
H	-0.635646	-1.127700	1.461193
H	2.004000	2.624299	1.402160
H	1.733920	4.195913	0.431326
H	-3.077547	2.147019	1.257098
H	-1.791651	0.864495	-1.204996
H	-3.888703	1.846116	-0.273203
H	0.011419	-1.489175	-2.258275
H	-1.703992	-1.953767	-2.427148
H	-0.487853	-3.178264	-2.123332
H	-4.940928	-2.761114	-1.206922

H	4.038905	0.702459	1.308629
H	3.588507	-0.843202	1.985997
H	5.921983	-0.813116	1.332682
H	6.069746	-2.897606	0.028349
H	4.734019	-2.998918	1.183433
H	4.400273	-2.705971	-0.534363
H	6.726495	-0.614883	-0.988103
H	5.841730	0.845328	-0.527125
H	5.074457	-0.362150	-1.576612

B3LYP energy = -1155.15251117 a.u.

(2*S*,6*R*,7*S*,8*S*)-**1**, Conf. M

C	-2.872351	-2.826824	0.093947
C	-3.198445	-1.354905	0.055505
C	-3.119845	-0.449952	1.043638
C	-2.920014	-0.790778	2.498764
C	-0.554472	-1.959346	-0.644619
C	-1.322618	-3.082834	0.024021
C	-1.937856	1.422917	-0.228843
C	-0.508445	1.452691	0.410392
C	0.424450	0.379694	-0.224366
C	-0.055744	-0.992447	0.145699
O	-2.196454	2.759440	-0.735681
C	-1.156111	3.612231	-0.530208
C	-0.077043	2.887801	0.204556
O	-1.178384	4.762807	-0.899383
C	1.023266	3.527417	0.612461
C	-3.119135	1.033079	0.683406
C	-0.537981	-1.947924	-2.150888
O	-3.448294	-3.502991	-1.034720
O	1.751638	0.597807	0.340056
C	2.816268	0.285682	-0.446784
O	2.707524	-0.107169	-1.591146
C	4.128109	0.486610	0.282451
C	4.826367	-0.838319	0.700065
C	3.944216	-1.683543	1.631738
C	5.320613	-1.654010	-0.503770
H	-3.237869	-3.297258	1.017127
H	-3.339921	-0.988121	-0.962094
H	-1.948057	-0.430660	2.862891
H	-2.972586	-1.864505	2.693785
H	-1.187865	-4.040132	-0.491192
H	-0.961474	-3.202136	1.050732
H	-3.682767	-0.297161	3.114255
H	-0.573320	1.235584	1.483007
H	0.489283	0.527542	-1.303744
H	-0.113763	-1.140920	1.224481
H	1.803242	3.029707	1.175867
H	1.143256	4.580201	0.372988
H	-3.094591	1.654708	1.586130
H	-1.940487	0.770256	-1.105940
H	-4.034440	1.300137	0.142893
H	-0.131998	-2.897178	-2.520649
H	0.072669	-1.142623	-2.562863
H	-1.555005	-1.875380	-2.556275
H	-4.403721	-3.354490	-1.029253

H	4.787965	1.039932	-0.394334
H	3.952162	1.098624	1.171620
H	5.709594	-0.522350	1.272723
H	4.496260	-2.557702	1.993449
H	3.613392	-1.108886	2.504179
H	3.050339	-2.048299	1.112448
H	5.879601	-2.532095	-0.161471
H	5.983827	-1.059763	-1.141968
H	4.488651	-1.999618	-1.124792

B3LYP energy = -1155.15245205 a.u.

(2*S*,6*R*,7*S*,8*S*)-**1**, Conf. N

C	-2.703777	-2.868961	-0.074369
C	-3.146633	-1.437899	-0.216726
C	-3.207285	-0.493597	0.739137
C	-3.134072	-0.753566	2.225187
C	-0.345300	-1.957690	-0.287257
C	-1.297742	-3.051577	-0.729054
C	-2.046543	1.414870	-0.492702
C	-0.647047	1.258763	0.189317
C	0.350897	0.488993	-0.730070
C	-0.182549	-0.883692	-1.080085
O	-2.193091	2.823725	-0.812066
C	-1.217456	3.588752	-0.245298
C	-0.255538	2.690477	0.462674
O	-1.200419	4.793030	-0.338960
C	0.734482	3.183101	1.212771
C	-3.268541	0.978876	0.343048
C	0.236690	-2.118665	1.095652
O	-3.564245	-3.788860	-0.761517
O	1.624695	0.455836	-0.034508
C	2.747858	0.503932	-0.807204
O	2.721164	0.575765	-2.017579
C	4.000580	0.441204	0.037686
C	4.329840	-0.983137	0.554615
C	4.559938	-1.973754	-0.596103
C	5.548450	-0.923612	1.487652
H	-2.640858	-3.152622	0.983558
H	-3.255050	-1.125130	-1.256500
H	-3.136826	-1.814963	2.481112
H	-2.240545	-0.298167	2.672407
H	-1.445347	-3.021843	-1.813400
H	-0.931471	-4.052613	-0.472342
H	-3.995028	-0.288121	2.722101
H	-0.736883	0.690381	1.118577
H	0.501691	1.054839	-1.653146
H	-0.631086	-0.928150	-2.070543
H	1.422704	2.540078	1.750039
H	0.861865	4.259006	1.291630
H	-3.323463	1.611857	1.236273
H	-2.070318	0.896569	-1.452670
H	-4.164632	1.196771	-0.249611
H	0.888737	-3.002023	1.118661
H	-0.550673	-2.300876	1.837151
H	0.826485	-1.258855	1.412295
H	-4.464371	-3.672836	-0.428935

H	4.823723	0.809602	-0.581982
H	3.881963	1.114770	0.893513
H	3.468632	-1.328851	1.141897
H	4.778044	-2.973226	-0.204074
H	3.687884	-2.052291	-1.252576
H	5.410809	-1.663176	-1.214537
H	5.781149	-1.916235	1.888322
H	5.374704	-0.251254	2.335439
H	6.435821	-0.565507	0.951165

B3LYP energy = -1155.15228989 a.u.

(2*S*,6*R*,7*S*,8*S*)-**1**, Conf. O

C	-3.883348	-1.768913	-0.006457
C	-3.663235	-0.297263	-0.227025
C	-3.328134	0.633199	0.684445
C	-3.394441	0.448227	2.181934
C	-1.360458	-1.975100	-0.168340
C	-2.684734	-2.573797	-0.600781
C	-1.438042	1.790272	-0.579166
C	-0.256283	1.084116	0.165269
C	0.328237	-0.089458	-0.679060
C	-0.741924	-1.112974	-0.993865
O	-0.956190	3.106797	-0.959289
C	0.242458	3.406733	-0.384215
C	0.706297	2.222781	0.400616
O	0.781105	4.478739	-0.527103
C	1.787641	2.284988	1.182825
C	-2.744742	1.965911	0.223284
C	-0.919973	-2.313919	1.235093
O	-5.047580	-2.264999	-0.682914
O	1.445520	-0.629601	0.074218
C	2.400602	-1.286253	-0.643955
O	2.351913	-1.415332	-1.849219
C	3.513536	-1.791447	0.246485
C	4.664224	-0.766055	0.435474
C	5.649504	-1.289336	1.491588
C	5.384821	-0.452031	-0.883628
H	-3.963422	-1.992703	1.064465
H	-3.611503	-0.025910	-1.282669
H	-2.398751	0.508163	2.640934
H	-3.848964	-0.497595	2.483166
H	-2.787800	-2.541263	-1.690189
H	-2.790630	-3.619862	-0.289892
H	-3.984935	1.259604	2.626761
H	-0.601573	0.660597	1.111967
H	0.728031	0.303608	-1.618209
H	-1.150678	-1.008664	-1.996724
H	2.114942	1.438647	1.776265
H	2.361937	3.205849	1.231020
H	-2.539782	2.610945	1.085601
H	-1.664082	1.283849	-1.518990
H	-3.449526	2.511862	-0.414582
H	-0.032684	-1.764885	1.549647
H	-0.691079	-3.386349	1.297194
H	-1.724101	-2.131250	1.958347
H	-5.813380	-1.754019	-0.388456

H	3.096282	-2.050769	1.224583
H	3.911444	-2.700831	-0.215767
H	4.221231	0.164255	0.816656
H	6.458293	-0.569689	1.658329
H	5.154995	-1.468603	2.453000
H	6.106090	-2.232484	1.166704
H	6.169265	0.295237	-0.721679
H	4.701372	-0.067101	-1.645499
H	5.858840	-1.353567	-1.290655

B3LYP energy = -1155.15212527 a.u.

(1*R*,5*S*,6*S*,7*S*,10*R*)-**2**, Conf. A

C	3.473160	0.018619	-0.253346
C	2.940311	1.406886	-0.664193
C	1.588658	1.670638	-0.038902
C	0.571696	0.589024	-0.375943
C	1.060304	-0.789533	0.194841
C	2.436763	-1.082160	-0.483231
C	-0.892978	0.929983	-0.035054
C	-1.858438	-0.193906	-0.454628
C	-1.407851	-1.538660	0.142088
C	0.043324	-1.876218	-0.228154
C	-3.347243	0.161429	-0.177267
C	-4.303050	-0.788296	-0.919849
C	-3.713035	0.231878	1.315681
O	-1.308184	2.107669	-0.742867
C	1.373652	2.718688	0.770210
O	3.035611	-2.301767	-0.030376
H	0.590112	0.484261	-1.474194
C	1.208040	-0.774140	1.730176
H	3.758956	0.023795	0.804411
H	4.375975	-0.226964	-0.822808
H	3.657438	2.185070	-0.383462
H	2.840886	1.440602	-1.759453
H	2.249064	-1.157838	-1.568152
H	-0.987401	1.106951	1.047219
H	-1.761149	-0.267058	-1.549392
H	-2.061638	-2.340177	-0.218883
H	-1.519659	-1.524652	1.232559
H	0.103083	-2.012213	-1.318810
H	0.317825	-2.836294	0.229490
H	-3.494516	1.162329	-0.597957
H	-4.264607	-1.808841	-0.520467
H	-5.338649	-0.442842	-0.824594
H	-4.066812	-0.836495	-1.989374
H	-4.743516	0.585260	1.434026
H	-3.068696	0.924110	1.868115
H	-3.649894	-0.749155	1.801683
H	-0.626464	2.785181	-0.636343
H	0.427320	2.893661	1.274744
H	2.165030	3.434070	0.978119
H	2.496885	-3.047232	-0.324411
H	1.664130	-1.709438	2.067574
H	1.836605	0.050476	2.073540
H	0.240398	-0.669169	2.228213

B3LYP energy = -737.751540513 a.u.

(1*R*,5*S*,6*S*,7*S*,10*R*)-**2**, Conf. B

C	3.468920	0.024609	-0.242310
C	2.939471	1.411723	-0.664676
C	1.588114	1.671511	-0.036903
C	0.572325	0.590935	-0.379485
C	1.055433	-0.793340	0.183640
C	2.431695	-1.082390	-0.477148
C	-0.892583	0.929020	-0.035947
C	-1.859476	-0.192326	-0.459977
C	-1.404890	-1.544807	0.117433
C	0.044902	-1.877246	-0.259334
C	-3.346355	0.161923	-0.170059
C	-4.307295	-0.780464	-0.915391
C	-3.703112	0.217438	1.325794
O	-1.307613	2.111067	-0.738162
C	1.376410	2.714265	0.779836
O	2.903058	-2.344825	0.011623
H	0.590388	0.491780	-1.477926
C	1.186311	-0.788177	1.722077
H	3.745401	0.036336	0.817785
H	4.382336	-0.210092	-0.806089
H	3.656170	2.191845	-0.388017
H	2.839354	1.438265	-1.759913
H	2.252603	-1.165230	-1.562945
H	-0.986317	1.102040	1.046823
H	-1.768678	-0.253314	-1.556044
H	-2.058481	-2.341215	-0.255253
H	-1.518499	-1.547718	1.208019
H	0.107652	-1.989539	-1.351733
H	0.341288	-2.840153	0.168920
H	-3.496685	1.166990	-0.579866
H	-4.266737	-1.804316	-0.525053
H	-5.342171	-0.435056	-0.811402
H	-4.076772	-0.819807	-1.986537
H	-4.733574	0.567675	1.454150
H	-3.056611	0.905561	1.880981
H	-3.634426	-0.768096	1.801608
H	-0.628674	2.789888	-0.623656
H	0.431257	2.885832	1.287583
H	2.168354	3.428295	0.990492
H	3.735400	-2.558540	-0.429003
H	1.841010	0.011050	2.077683
H	0.216519	-0.645451	2.205785
H	1.597115	-1.742996	2.060620

B3LYP energy = -737.751313607 a.u.

(1*R*,5*S*,6*S*,7*S*,10*R*)-**2**, Conf. C

C	3.471741	0.025438	-0.267935
C	2.934635	1.415894	-0.668959
C	1.587772	1.668915	-0.030121
C	0.571555	0.590761	-0.377553
C	1.054640	-0.796265	0.177374
C	2.437243	-1.087531	-0.486832
C	-0.893981	0.929807	-0.035861
C	-1.861614	-0.191116	-0.459346
C	-1.405641	-1.544719	0.115157
C	0.042527	-1.875252	-0.269665
C	-3.347915	0.162902	-0.166451
C	-4.310147	-0.776711	-0.913548
C	-3.702716	0.213885	1.330041
O	-1.307097	2.111982	-0.738019
C	1.378100	2.703940	0.797003
O	2.972210	-2.362111	-0.114621
H	0.591428	0.494693	-1.476198
C	1.179006	-0.792947	1.716234
H	3.768902	0.046872	0.789689
H	4.371970	-0.216707	-0.843592
H	3.652481	2.194701	-0.391350
H	2.826633	1.451271	-1.762992
H	2.257886	-1.185066	-1.565636
H	-0.989249	1.103706	1.046781
H	-1.772591	-0.251171	-1.555501
H	-2.061178	-2.340158	-0.256005
H	-1.515252	-1.549233	1.206439
H	0.101380	-1.977870	-1.362852
H	0.340578	-2.843748	0.145737
H	-3.498182	1.169307	-0.572981
H	-4.269108	-1.801987	-0.527017
H	-5.344776	-0.431569	-0.806598
H	-4.081415	-0.812160	-1.985151
H	-4.732407	0.565319	1.460904
H	-3.054646	0.899206	1.887006
H	-3.635228	-0.773496	1.802269
H	-0.629932	2.791895	-0.619119
H	0.434031	2.870981	1.308342
H	2.169718	3.417660	1.009991
H	3.325695	-2.303179	0.783125
H	1.887666	-0.042358	2.075610
H	0.221769	-0.580635	2.198627
H	1.501682	-1.777234	2.074353

B3LYP energy = -737.751006437 a.u.

(1*R*,5*S*,6*S*,7*S*,10*R*)-**2**, Conf. D

C	3.424038	0.428413	-0.266814
C	2.675713	1.734706	-0.602804
C	1.311044	1.751761	0.049687
C	0.467192	0.545486	-0.340171
C	1.173530	-0.768282	0.150966
C	2.568826	-0.807439	-0.548849
C	-1.027760	0.638063	0.032072
C	-1.820972	-0.592745	-0.443031
C	-1.148510	-1.883591	0.063226
C	0.331492	-1.973763	-0.328448
C	-3.340682	-0.580057	-0.086493
C	-3.639958	-0.121955	1.351622
C	-4.194614	0.209016	-1.094109
O	-1.620943	1.787435	-0.590318
C	0.956379	2.703599	0.925921
O	3.355161	-1.940686	-0.163701
H	0.485055	0.503888	-1.442639

C	1.337173	-0.814651	1.683921
H	3.717496	0.425676	0.788857
H	4.347125	0.356374	-0.851896
H	3.268173	2.600362	-0.289246
H	2.553282	1.805806	-1.693896
H	2.380668	-0.855408	-1.635130
H	-1.126876	0.727637	1.124179
H	-1.748195	-0.594091	-1.541543
H	-1.682875	-2.746350	-0.352537
H	-1.254985	-1.960141	1.152764
H	0.398521	-2.038118	-1.425376
H	0.754609	-2.904650	0.072733
H	-3.660837	-1.630489	-0.159974
H	-3.046690	-0.667086	2.094981
H	-3.443575	0.948541	1.475041
H	-4.695933	-0.291769	1.589574
H	-3.947608	1.272977	-1.074654
H	-5.260545	0.092732	-0.863510
H	-4.034385	-0.154388	-2.116039
H	-1.038410	2.545956	-0.445795
H	0.006651	2.699171	1.453811
H	1.633871	3.518358	1.167342
H	2.941244	-2.743842	-0.504116
H	1.933655	-1.686912	1.966622
H	1.838085	0.075449	2.071088
H	0.371389	-0.885051	2.191340

B3LYP energy = -737.749907514 a.u.

(1*R*,5*S*,6*S*,7*S*,10*R*)-**2**, Conf. E

C	3.418776	0.432157	-0.257601
C	2.673925	1.738623	-0.604581
C	1.310732	1.752288	0.051184
C	0.467602	0.547194	-0.342633
C	1.169419	-0.772373	0.141264
C	2.563032	-0.809559	-0.543135
C	-1.027149	0.636944	0.031882
C	-1.821263	-0.591320	-0.448835
C	-1.145389	-1.887209	0.041484
C	0.332540	-1.973850	-0.355245
C	-3.339469	-0.580282	-0.086144
C	-3.633354	-0.132132	1.356268
C	-4.198236	0.215332	-1.084565
O	-1.620600	1.790582	-0.584029
C	0.960662	2.699864	0.933831
O	3.231966	-2.006482	-0.124619
H	0.484087	0.510273	-1.444969
C	1.320825	-0.830086	1.676410
H	3.703600	0.434560	0.800363
H	4.349830	0.371875	-0.837824
H	3.266195	2.606111	-0.295577
H	2.550998	1.802526	-1.695898
H	2.382419	-0.864049	-1.630330
H	-1.125466	0.721704	1.124281
H	-1.753095	-0.582942	-1.547619
H	-1.680932	-2.744782	-0.383651
H	-1.252242	-1.977488	1.130239
H	0.399383	-2.017336	-1.452425
H	0.776996	-2.901657	0.019231
H	-3.659534	-1.630242	-0.165176
H	-4.688191	-0.304559	1.597721
H	-3.036167	-0.681854	2.093043
H	-3.437636	0.937815	1.486188
H	-5.263280	0.097187	-0.850427
H	-4.042090	-0.141019	-2.109629
H	-3.951569	1.279325	-1.059093
H	-1.039962	2.549055	-0.432640
H	0.012900	2.692584	1.465090
H	1.639033	3.513540	1.176676
H	4.081051	-2.064614	-0.580975
H	0.348076	-0.862350	2.173907
H	1.876062	-1.728442	1.958795
H	1.852685	0.038431	2.072384

B3LYP energy = -737.749718076 a.u.

(1*R*,5*S*,6*S*,7*S*,10*R*)-**2**, Conf. F

C	3.421786	0.432222	-0.281017
C	2.670658	1.740451	-0.606481
C	1.311656	1.749198	0.057377
C	0.467131	0.547049	-0.341538
C	1.168559	-0.774819	0.134091
C	2.568968	-0.814457	-0.553174
C	-1.028450	0.637672	0.031472
C	-1.823590	-0.590135	-0.448866
C	-1.146732	-1.886878	0.038601
C	0.329285	-1.971720	-0.365582
C	-3.341269	-0.579079	-0.083731
C	-3.632865	-0.134199	1.360115
C	-4.200879	0.219329	-1.079148
O	-1.620228	1.792138	-0.583580
C	0.964106	2.690649	0.947659
O	3.298363	-2.008633	-0.251523
H	0.485082	0.513772	-1.443996
C	1.313912	-0.834666	1.669727
H	3.723877	0.445469	0.775462
H	4.341584	0.362873	-0.872216
H	3.264483	2.606194	-0.295365
H	2.540412	1.812529	-1.696263
H	2.391292	-0.882763	-1.634414
H	-1.128139	0.722469	1.123910
H	-1.757219	-0.581205	-1.547662
H	-1.684306	-2.743770	-0.385116
H	-1.249628	-1.978196	1.127917
H	0.391138	-2.007083	-1.462974
H	0.776089	-2.903664	-0.003271
H	-3.661917	-1.628680	-0.164747
H	-4.687376	-0.306882	1.602672
H	-3.035055	-0.685975	2.094984
H	-3.436916	0.935421	1.492538
H	-5.265582	0.101833	-0.843351
H	-4.046889	-0.135276	-2.105081
H	-3.953114	1.283017	-1.052133
H	-1.039665	2.550317	-0.430197

H	0.016966	2.681100	1.480062
H	1.642725	3.503415	1.192930
H	3.657058	-1.938635	0.643328
H	0.342920	-0.799252	2.169767
H	1.790007	-1.774884	1.970759
H	1.903434	-0.003882	2.066248

B3LYP energy = -737.749394045 a.u.

(1*R*,5*S*,6*S*,7*S*,10*R*)-**2**, Conf. G

C	3.481840	-0.007506	-0.180195
C	2.982249	1.382725	-0.614414
C	1.610776	1.684611	-0.045538
C	0.580888	0.612796	-0.383764
C	1.048605	-0.786897	0.164530
C	2.435144	-1.087912	-0.467497
C	-0.884233	0.937087	-0.012087
C	-1.859117	-0.178290	-0.460879
C	-1.405055	-1.551827	0.064137
C	0.047036	-1.864622	-0.312218
C	-3.341897	0.164346	-0.138540
C	-4.314534	-0.715269	-0.942918
C	-3.687526	0.111525	1.360380
O	-1.291191	2.201793	-0.555951
C	1.385468	2.776459	0.695460
O	2.866414	-2.372802	0.000972
H	0.592554	0.509229	-1.485887
C	1.151052	-0.794660	1.705488
H	3.717462	-0.008267	0.889814
H	4.412071	-0.252471	-0.711857
H	3.698802	2.155374	-0.317240
H	2.925457	1.408955	-1.714044
H	2.283792	-1.141969	-1.559879
H	-0.961625	1.078941	1.071342
H	-1.783762	-0.211232	-1.562194
H	-2.055824	-2.335912	-0.339569
H	-1.524168	-1.593476	1.152858
H	0.119881	-1.948671	-1.406866
H	0.346605	-2.836295	0.092821
H	-3.487343	1.199105	-0.469309
H	-4.100891	-0.669309	-2.017503
H	-4.266958	-1.767194	-0.637147
H	-5.347959	-0.381503	-0.795793
H	-4.707937	0.475846	1.523997
H	-3.020550	0.737713	1.962572
H	-3.640257	-0.909829	1.755535
H	-1.041894	2.234618	-1.489813
H	0.404878	3.027055	1.082228
H	2.191721	3.473845	0.910733
H	3.731349	-2.567458	-0.381955
H	1.772334	0.023448	2.078004
H	0.170000	-0.688517	2.175384
H	1.586306	-1.739360	2.041929

B3LYP energy = -737.748394819 a.u.

(1*R*,5*S*,6*S*,7*S*,10*R*)-**2**, Conf. H

C	3.485718	-0.011023	-0.198581
C	2.979445	1.379745	-0.620495
C	1.609744	1.683007	-0.048543
C	0.578807	0.609436	-0.379416
C	1.054165	-0.783757	0.178295
C	2.440202	-1.088422	-0.473906
C	-0.885832	0.936185	-0.010324
C	-1.858109	-0.182030	-0.456229
C	-1.408759	-1.545608	0.095782
C	0.044634	-1.865832	-0.272855
C	-3.343420	0.163713	-0.151065
C	-4.308950	-0.726291	-0.952514
C	-3.702597	0.131981	1.345233
O	-1.296486	2.196594	-0.560831
C	1.382754	2.776311	0.690056
O	3.004343	-2.328360	-0.030994
H	0.589919	0.498136	-1.481213
C	1.177938	-0.778329	1.716151
H	3.734940	-0.016940	0.868347
H	4.403399	-0.266800	-0.739373
H	3.696315	2.151413	-0.321707
H	2.919481	1.413026	-1.719904
H	2.277692	-1.140484	-1.564667
H	-0.964416	1.078300	1.073425
H	-1.773314	-0.229545	-1.555848
H	-2.059618	-2.337619	-0.291791
H	-1.526220	-1.563743	1.185194
H	0.113814	-1.979730	-1.365788
H	0.320836	-2.833491	0.167056
H	-3.484260	1.193671	-0.497893
H	-4.085539	-0.694655	-2.025579
H	-4.265786	-1.774149	-0.632052
H	-5.343362	-0.389915	-0.819470
H	-4.723946	0.499744	1.494168
H	-3.040592	0.765828	1.944817
H	-3.661170	-0.883740	1.755805
H	-0.953191	2.274067	-1.461318
H	0.402684	3.025059	1.079735
H	2.188044	3.475713	0.901971
H	2.462801	-3.058275	-0.356657
H	1.778466	0.061500	2.072858
H	0.201775	-0.703613	2.202686
H	1.653945	-1.704145	2.052258

B3LYP energy = -737.748331233 a.u.

(1*R*,5*S*,6*R*,7*R*,8*R*,10*R*)-**3**, Conf. A

C	3.983707	-2.245352	0.202929
C	4.546267	-0.854366	0.559131
C	3.455314	0.103996	0.988761
C	2.332854	0.192283	-0.028443
C	1.665649	-1.229329	-0.254382
C	2.802175	-2.154308	-0.770095
C	1.217071	1.193793	0.221331
C	0.291142	1.292242	-0.998544
C	-0.461898	-0.008409	-1.246048
C	0.561381	-1.155807	-1.353116

O	1.650767	2.567158	0.427512
C	0.654920	3.417799	0.012719
C	-0.360386	2.628289	-0.766385
O	2.236146	-3.443956	-1.032222
C	3.509635	0.784105	2.139434
C	-1.539678	3.129255	-1.140564
O	0.672382	4.600143	0.254878
H	2.802785	0.473094	-0.986183
C	1.071066	-1.801157	1.054028
O	-1.392890	-0.266793	-0.158917
C	-2.723998	-0.167204	-0.409787
O	-3.185889	0.115607	-1.497430
C	-3.530342	-0.414225	0.848623
C	-4.978150	-0.868504	0.594570
C	-5.026543	-2.272518	-0.027378
C	-5.786128	-0.811489	1.899556
H	3.659300	-2.766542	1.110103
H	4.774949	-2.858602	-0.249771
H	5.048379	-0.438491	-0.328109
H	5.308898	-0.942664	1.339218
H	3.163699	-1.725367	-1.720885
H	0.643256	0.912297	1.111151
H	0.948963	1.416565	-1.874939
H	-1.045414	0.047650	-2.167468
H	1.048431	-1.036229	-2.330775
H	0.030840	-2.110757	-1.393570
H	2.935760	-4.036358	-1.336306
H	4.349513	0.657984	2.817989
H	2.750776	1.501088	2.431915
H	-2.261516	2.545752	-1.703516
H	-1.796735	4.150190	-0.872913
H	0.172228	-1.261610	1.354488
H	1.783477	-1.752191	1.880500
H	0.794435	-2.847257	0.898159
H	-2.993483	-1.132414	1.479094
H	-3.525339	0.537258	1.399567
H	-5.421731	-0.164653	-0.120381
H	-4.588278	-3.016608	0.650267
H	-6.060776	-2.573768	-0.226262
H	-4.482604	-2.309498	-0.976244
H	-5.795880	0.199169	2.323697
H	-5.367158	-1.487994	2.655339
H	-6.825244	-1.111883	1.727223

B3LYP energy = -1155.18203143 a.u.

(1*R*,5*S*,6*R*,7*R*,8*R*,10*R*)-**3**, Conf. B

C	4.364029	-1.638663	0.313483
C	4.631124	-0.168450	0.695891
C	3.359118	0.560688	1.074428
C	2.285454	0.437518	0.009593
C	1.913987	-1.085121	-0.239494
C	3.228033	-1.768355	-0.708420
C	0.988510	1.207084	0.203492
C	0.118157	1.128441	-1.057704
C	-0.366096	-0.290888	-1.323119
C	0.861392	-1.221811	-1.381926

O	1.143999	2.637179	0.419324
C	0.027582	3.285499	-0.051301
C	-0.781549	2.319161	-0.870935
O	2.933803	-3.140464	-0.996544
C	3.230241	1.234178	2.222888
C	-2.012743	2.588822	-1.309982
O	-0.188703	4.450145	0.180296
H	2.734282	0.806717	-0.928196
C	1.390822	-1.770562	1.044384
O	-1.269485	-0.720520	-0.266849
C	-2.580359	-0.907573	-0.569423
O	-3.058371	-0.684388	-1.663738
C	-3.331965	-1.470780	0.619675
C	-4.847997	-1.211019	0.594901
C	-5.548919	-2.069119	1.658722
C	-5.168249	0.278967	0.788200
H	4.110820	-2.222908	1.204600
H	5.277170	-2.081752	-0.107218
H	5.083900	0.343148	-0.167587
H	5.360671	-0.115691	1.510208
H	3.538060	-1.267908	-1.642082
H	0.439531	0.819385	1.068488
H	0.781666	1.369935	-1.904617
H	-0.915552	-0.347097	-2.265325
H	1.354886	-1.009476	-2.340555
H	0.526012	-2.260715	-1.437710
H	3.747723	-3.584552	-1.267058
H	4.046776	1.269984	2.939540
H	2.335035	1.791298	2.475149
H	-2.580888	1.876809	-1.900411
H	-2.471049	3.543314	-1.067622
H	0.395952	-1.413219	1.312137
H	2.049822	-1.597517	1.898021
H	1.322240	-2.848597	0.875711
H	-3.135057	-2.552518	0.612397
H	-2.877828	-1.087233	1.540666
H	-5.215780	-1.513439	-0.393366
H	-5.366959	-3.138058	1.498582
H	-5.197014	-1.816245	2.666996
H	-6.632028	-1.907585	1.636940
H	-4.696438	0.895596	0.017183
H	-4.820832	0.631607	1.767824
H	-6.248493	0.453016	0.737279

B3LYP energy = -1155.18175660 a.u.

(1*R*,5*S*,6*R*,7*R*,8*R*,10*R*)-**3**, Conf. C

C	3.996486	-2.235884	0.180580
C	4.540827	-0.845630	0.566925
C	3.440172	0.097447	1.003824
C	2.326375	0.193254	-0.021714
C	1.663585	-1.227208	-0.264202
C	2.808754	-2.152062	-0.785325
C	1.211271	1.196681	0.224038
C	0.289655	1.299095	-0.998676
C	-0.461147	-0.001724	-1.253207
C	0.566154	-1.144353	-1.367447

O	1.646475	2.568545	0.434925
C	0.651812	3.421309	0.020131
C	-0.362234	2.634549	-0.763524
O	2.339391	-3.454351	-1.146110
C	3.478356	0.759792	2.165471
C	-1.540789	3.136905	-1.138171
O	0.669363	4.602702	0.266037
H	2.804642	0.478002	-0.974225
C	1.060890	-1.804687	1.036812
O	-1.389208	-0.270807	-0.165390
C	-2.721252	-0.162515	-0.410380
O	-3.185415	0.131912	-1.493683
C	-3.525081	-0.416438	0.848244
C	-4.970046	-0.879915	0.593725
C	-5.009048	-2.282878	-0.031251
C	-5.778144	-0.831065	1.898906
H	3.690118	-2.771899	1.089317
H	4.784142	-2.835275	-0.289205
H	5.045058	-0.412066	-0.310338
H	5.299563	-0.940191	1.350166
H	3.160475	-1.727140	-1.735091
H	0.632783	0.915795	1.111055
H	0.950324	1.426665	-1.872409
H	-1.046278	0.058009	-2.173331
H	1.059222	-1.011654	-2.340106
H	0.039357	-2.100700	-1.426461
H	2.250672	-3.990392	-0.346661
H	4.312826	0.629968	2.849955
H	2.712105	1.467738	2.461033
H	-2.261768	2.555312	-1.704156
H	-1.798057	4.157100	-0.867924
H	0.196983	-1.230101	1.372509
H	1.786276	-1.824676	1.853154
H	0.702892	-2.825402	0.861490
H	-2.983835	-1.131707	1.478256
H	-3.526749	0.534761	1.399687
H	-5.418250	-0.177510	-0.119690
H	-4.565864	-3.025525	0.644884
H	-6.041170	-2.590802	-0.230592
H	-4.465299	-2.314476	-0.980485
H	-5.794538	0.178538	2.325248
H	-5.354795	-1.506549	2.653190
H	-6.815247	-1.137861	1.725938

B3LYP energy = -1155.18150642 a.u.

(1*R*,5*S*,6*R*,7*R*,8*R*,10*R*)-**3**, Conf. D

C	4.045343	-2.132084	0.193156
C	4.571068	-0.729065	0.557814
C	3.454720	0.196848	0.992598
C	2.330934	0.262671	-0.024834
C	1.700867	-1.174808	-0.261935
C	2.862102	-2.067296	-0.780141
C	1.188708	1.232197	0.232057
C	0.259913	1.309454	-0.986746
C	-0.457448	-0.005204	-1.244619
C	0.596918	-1.125075	-1.362699

O	1.582094	2.618003	0.441650
C	0.559697	3.439731	0.033272
C	-0.432607	2.621270	-0.745840
O	2.329876	-3.370140	-1.047106
C	3.489027	0.870657	2.147751
C	-1.633288	3.076165	-1.107308
O	0.541132	4.621261	0.278720
H	2.793232	0.562455	-0.980426
C	1.118328	-1.769384	1.041763
O	-1.386327	-0.251281	-0.154341
C	-2.603370	-0.773183	-0.460243
O	-2.966898	-1.007984	-1.594766
C	-3.403072	-1.045515	0.797498
C	-4.918138	-1.182548	0.571495
C	-5.590869	-1.745780	1.832265
C	-5.551839	0.154709	0.157969
H	3.734683	-2.666790	1.097222
H	4.852605	-2.721750	-0.262486
H	5.062274	-0.294717	-0.326664
H	5.335384	-0.801653	1.337835
H	3.212652	-1.625359	-1.729011
H	0.624781	0.932345	1.121909
H	0.914517	1.459146	-1.861391
H	-1.040684	0.032640	-2.167598
H	1.080494	-0.984387	-2.339408
H	0.093494	-2.094023	-1.413980
H	3.043104	-3.941513	-1.359610
H	4.330992	0.763378	2.826924
H	2.710413	1.564796	2.443581
H	-2.332766	2.468075	-1.672930
H	-1.936083	4.083196	-0.835956
H	0.208844	-1.249772	1.346001
H	1.828186	-1.711573	1.869868
H	0.865191	-2.820232	0.877682
H	-2.998192	-1.978077	1.216197
H	-3.179593	-0.263663	1.533092
H	-5.063833	-1.895663	-0.249284
H	-5.177272	-2.722803	2.107530
H	-5.456711	-1.071691	2.687816
H	-6.667696	-1.870687	1.675992
H	-6.626986	0.036889	-0.015880
H	-5.108338	0.540760	-0.764843
H	-5.423664	0.909242	0.944915

B3LYP energy = -1155.18142467 a.u.

(1*R*,5*S*,6*R*,7*R*,8*R*,10*R*)-**3**, Conf. E

C	4.376046	-1.627006	0.294212
C	4.625503	-0.160589	0.702051
C	3.347436	0.553789	1.086616
C	2.280289	0.438019	0.014907
C	1.912991	-1.082632	-0.248192
C	3.235034	-1.766385	-0.720474
C	0.983727	1.209476	0.205192
C	0.115994	1.134968	-1.057954
C	-0.364717	-0.284447	-1.330843
C	0.866481	-1.209413	-1.395715

O	1.140376	2.638460	0.425309
C	0.023733	3.288312	-0.043750
C	-0.784669	2.324506	-0.867333
O	3.039513	-3.132251	-1.098578
C	3.208030	1.210672	2.243495
C	-2.015513	2.595853	-1.306339
O	-0.193141	4.451819	0.192159
H	2.735423	0.811556	-0.918210
C	1.381933	-1.772500	1.028814
O	-1.264032	-0.725672	-0.275143
C	-2.577380	-0.904691	-0.574186
O	-3.058865	-0.667201	-1.663663
C	-3.326303	-1.479200	0.611276
C	-4.842212	-1.217929	0.593112
C	-5.541146	-2.087122	1.649214
C	-5.160571	0.270169	0.803340
H	4.142067	-2.219516	1.189176
H	5.283421	-2.059059	-0.142156
H	5.076445	0.366087	-0.152993
H	5.352911	-0.113392	1.518690
H	3.536588	-1.274266	-1.654814
H	0.430781	0.821585	1.067632
H	0.780716	1.381008	-1.902586
H	-0.915450	-0.337046	-2.272473
H	1.364059	-0.982795	-2.348697
H	0.536424	-2.249138	-1.470211
H	3.042257	-3.682694	-0.304149
H	4.020693	1.242815	2.964703
H	2.308158	1.759291	2.498204
H	-2.583306	1.886155	-1.899831
H	-2.473708	3.549594	-1.060828
H	0.416161	-1.369408	1.335081
H	2.069071	-1.667613	1.871376
H	1.224360	-2.839926	0.837648
H	-3.130751	-2.561088	0.592834
H	-2.869788	-1.104868	1.534937
H	-5.212607	-1.509277	-0.397470
H	-5.360415	-3.154414	1.476974
H	-5.186903	-1.845424	2.659421
H	-6.624160	-1.924719	1.631661
H	-6.240792	0.445603	0.757676
H	-4.690729	0.894622	0.037451
H	-4.809853	0.611996	1.785618

B3LYP energy = -1155.18124773 a.u.

(1*R*,5*S*,6*R*,7*R*,8*R*,10*R*)-**3**, Conf. F

C	3.468506	-2.621053	-0.021604
C	4.216492	-1.359681	0.455387
C	3.266425	-0.296068	0.964319
C	2.182795	0.039130	-0.044057
C	1.325677	-1.248243	-0.395317
C	2.326519	-2.278150	-0.986144
C	1.213255	1.163366	0.286709
C	0.328802	1.494059	-0.923306
C	-0.594865	0.339125	-1.288511
C	0.261505	-0.926346	-1.488709

O	1.832518	2.439014	0.610669
C	0.972209	3.454119	0.267033
C	-0.132336	2.884140	-0.578619
O	1.587040	-3.446268	-1.362171
C	3.397903	0.269907	2.169104
C	-1.225308	3.574519	-0.910906
O	1.153315	4.597336	0.609104
H	2.702243	0.332245	-0.972253
C	0.635391	-1.839643	0.856038
O	-1.575577	0.121485	-0.237579
C	-2.876952	0.419359	-0.495345
O	-3.265308	0.882008	-1.549998
C	-3.756237	0.093530	0.691218
C	-3.888164	-1.423135	0.979009
C	-4.703942	-1.638782	2.262124
C	-4.510037	-2.176126	-0.206393
H	3.059438	-3.166941	0.835443
H	4.171697	-3.299082	-0.524345
H	4.784341	-0.945335	-0.391953
H	4.948135	-1.621495	1.226175
H	2.760307	-1.825231	-1.894576
H	0.592568	0.891922	1.147719
H	1.011887	1.599022	-1.782732
H	-1.145511	0.557430	-2.206097
H	0.777185	-0.792831	-2.449742
H	-0.394525	-1.791505	-1.615210
H	2.200929	-4.106260	-1.709114
H	4.200384	-0.029561	2.838347
H	2.742240	1.060176	2.517022
H	-2.013398	3.145997	-1.522066
H	-1.341978	4.595061	-0.557588
H	-0.178848	-1.202598	1.203598
H	1.335199	-1.970343	1.684468
H	0.211209	-2.816095	0.607310
H	-3.335508	0.591195	1.572821
H	-4.740935	0.525599	0.489741
H	-2.877533	-1.816811	1.146941
H	-4.244662	-1.135370	3.120276
H	-5.723716	-1.249970	2.150413
H	-4.779762	-2.705337	2.500011
H	-4.581700	-3.247095	0.011944
H	-3.919978	-2.061822	-1.121801
H	-5.521438	-1.807720	-0.417122

B3LYP energy = -1155.18106152 a.u.

(1*R*,5*S*,6*R*,7*R*,8*R*,10*R*)-**3**, Conf. G

C	3.994383	-2.237618	0.204786
C	4.542464	-0.846348	0.577087
C	3.447396	0.109237	1.000891
C	2.331351	0.196559	-0.023422
C	1.671788	-1.228153	-0.250857
C	2.820039	-2.152127	-0.770051
C	1.212941	1.196492	0.220898
C	0.291292	1.292226	-1.002380
C	-0.462075	-0.008047	-1.246510
C	0.564506	-1.155295	-1.346605

O	1.642031	2.571393	0.426799
C	0.646239	3.419386	0.007553
C	-0.364202	2.626965	-0.775399
O	2.386225	-3.496713	-1.005814
C	3.490677	0.789317	2.152060
C	-1.542959	3.124118	-1.155959
O	0.658950	4.601909	0.248192
H	2.806884	0.478456	-0.978494
C	1.080289	-1.807690	1.053833
O	-1.391641	-0.264146	-0.160159
C	-2.724400	-0.180925	-0.416751
O	-3.183476	0.074254	-1.512012
C	-3.531061	-0.407333	0.844684
C	-4.977238	-0.871087	0.598452
C	-5.020872	-2.286217	0.001943
C	-5.785130	-0.793190	1.902419
H	3.668396	-2.770693	1.104513
H	4.780398	-2.846766	-0.253910
H	5.053694	-0.422298	-0.301282
H	5.296943	-0.936601	1.364834
H	3.184073	-1.721306	-1.718374
H	0.636466	0.914227	1.108868
H	0.951531	1.414904	-1.877232
H	-1.044324	0.043435	-2.169117
H	1.046640	-1.036687	-2.328491
H	0.015897	-2.103358	-1.374445
H	1.860723	-3.530911	-1.814776
H	4.325441	0.664306	2.837033
H	2.728295	1.504636	2.439555
H	-2.260514	2.538814	-1.722609
H	-1.804374	4.144607	-0.890942
H	0.165803	-1.290337	1.346679
H	1.785856	-1.739557	1.884373
H	0.834084	-2.862433	0.900412
H	-2.992019	-1.111394	1.489133
H	-3.529118	0.554250	1.377810
H	-5.423322	-0.181717	-0.128942
H	-4.579759	-3.016471	0.692655
H	-6.054081	-2.594668	-0.190802
H	-4.477626	-2.338011	-0.946676
H	-5.798128	0.224882	2.308272
H	-5.363869	-1.454614	2.670139
H	-6.823260	-1.099901	1.735727

B3LYP energy = -1155.18097793 a.u.

(1*R*,5*S*,6*R*,7*R*,8*R*,10*R*)-**3**, Conf. H

C	4.375889	-1.624444	0.314491
C	4.626562	-0.155927	0.709580
C	3.350861	0.568904	1.082422
C	2.282621	0.442439	0.012552
C	1.921163	-1.082607	-0.234671
C	3.246752	-1.761598	-0.707043
C	0.983013	1.208624	0.201802
C	0.115327	1.124845	-1.060893
C	-0.367624	-0.295397	-1.319900
C	0.864280	-1.223491	-1.372743

O	1.132222	2.639874	0.414405
C	0.014855	3.283329	-0.059671
C	-0.788992	2.312702	-0.879957
O	3.092381	-3.162936	-0.960321
C	3.212296	1.242582	2.229672
C	-2.019468	2.577093	-1.323949
O	-0.207103	4.447375	0.168604
H	2.734667	0.812895	-0.923649
C	1.405822	-1.773757	1.047634
O	-1.267012	-0.722869	-0.261977
C	-2.577113	-0.924291	-0.565417
O	-3.050789	-0.725104	-1.665882
C	-3.328625	-1.469276	0.631655
C	-4.845270	-1.212701	0.601160
C	-5.544900	-2.054047	1.679127
C	-5.168198	0.279742	0.769447
H	4.126010	-2.219548	1.199433
H	5.283354	-2.063829	-0.112980
H	5.084700	0.364484	-0.146024
H	5.349089	-0.104397	1.530211
H	3.556499	-1.260289	-1.639631
H	0.433668	0.820337	1.066431
H	0.779568	1.364747	-1.907743
H	-0.916734	-0.358068	-2.262019
H	1.351580	-1.012152	-2.336511
H	0.511180	-2.259767	-1.416256
H	2.623292	-3.292115	-1.793923
H	4.024577	1.280754	2.950949
H	2.314016	1.796915	2.477282
H	-2.583422	1.862778	-1.915697
H	-2.481993	3.530418	-1.085141
H	0.398937	-1.444657	1.307380
H	2.054683	-1.578291	1.903795
H	1.372517	-2.855186	0.885567
H	-3.130133	-2.550757	0.642196
H	-2.876005	-1.070010	1.546582
H	-5.211939	-1.532560	-0.382034
H	-6.628137	-1.894192	1.654613
H	-5.361689	-3.125273	1.536830
H	-5.193346	-1.783923	2.682989
H	-6.248726	0.450728	0.715527
H	-4.697737	0.884676	-0.011643
H	-4.821566	0.649210	1.743056

B3LYP energy = -1155.18069142 a.u.

(1*R*,5*S*,6*R*,7*R*,8*R*,10*R*)-**3**, Conf. I

C	3.965904	-2.221436	0.181186
C	4.528519	-0.836174	0.553894
C	3.444602	0.127049	0.988939
C	2.322834	0.228722	-0.027584
C	1.649176	-1.189109	-0.258746
C	2.785948	-2.123199	-0.785730
C	1.214538	1.236389	0.230643
C	0.283135	1.341670	-0.984164
C	-0.479829	0.052057	-1.232268
C	0.538515	-1.103358	-1.349871

O	1.654201	2.608992	0.434147
C	0.659120	3.463534	0.027456
C	-0.363800	2.677206	-0.745706
O	2.338538	-3.463095	-1.019768
C	3.501407	0.802029	2.142555
C	-1.548155	3.174475	-1.104582
O	0.680347	4.645673	0.268931
H	2.794138	0.511762	-0.984368
C	1.058076	-1.766786	1.046961
O	-1.412243	-0.148778	-0.137974
C	-2.575140	-0.795944	-0.414557
O	-2.868652	-1.193733	-1.523944
C	-3.408024	-0.976167	0.837495
C	-4.915057	-1.143802	0.575239
C	-5.626221	-1.602365	1.857125
C	-5.538711	0.149226	0.028330
H	3.641487	-2.754422	1.081555
H	4.743511	-2.836081	-0.284434
H	5.036839	-0.413441	-0.326799
H	5.288015	-0.935439	1.335739
H	3.147811	-1.694079	-1.735684
H	0.644609	0.957240	1.123444
H	0.938040	1.465692	-1.862607
H	-1.063842	0.102476	-2.154708
H	1.015896	-0.979264	-2.333440
H	-0.016873	-2.046475	-1.387910
H	1.793561	-3.489482	-1.816206
H	4.339670	0.666986	2.821303
H	2.747122	1.522612	2.438266
H	-2.272728	2.592086	-1.665520
H	-1.812090	4.192798	-0.834923
H	0.160885	-1.228400	1.356403
H	1.773533	-1.722625	1.870643
H	0.786041	-2.814318	0.887620
H	-3.012293	-1.872608	1.336365
H	-3.212782	-0.138768	1.517184
H	-5.029963	-1.925511	-0.185711
H	-5.523908	-0.856748	2.655818
H	-6.696634	-1.748593	1.677105
H	-5.217524	-2.549129	2.228637
H	-5.063721	0.458531	-0.907917
H	-5.440971	0.968459	0.752235
H	-6.606359	0.010169	-0.172908

B3LYP energy = -1155.18063980 a.u.

(1*R*,5*S*,6*R*,7*R*,8*R*,10*R*)-**3**, Conf. J

C	4.361831	-1.203177	0.419320
C	4.424041	0.277062	0.847887
C	3.051484	0.836886	1.158404
C	2.068774	0.616539	0.023719
C	1.900670	-0.931269	-0.281318
C	3.314583	-1.440524	-0.675768
C	0.678404	1.218824	0.149152
C	-0.095394	1.070799	-1.167786
C	-0.386818	-0.387018	-1.495228
C	0.947153	-1.159428	-1.494802

O	0.643803	2.650972	0.402902
C	-0.509132	3.174870	-0.130256
C	-1.138752	2.144176	-1.025187
O	3.206306	-2.830066	-1.006773
C	2.771248	1.457802	2.309643
C	-2.354890	2.284703	-1.557014
O	-0.879919	4.297987	0.110696
H	2.526845	1.063639	-0.874829
C	1.383680	-1.709232	0.951266
O	-1.294515	-0.965262	-0.517260
C	-2.543713	-1.319023	-0.922280
O	-2.972478	-1.110080	-2.039984
C	-3.302292	-2.013566	0.188052
C	-3.640739	-1.114804	1.402962
C	-4.578181	0.038031	1.015227
C	-4.251513	-1.969120	2.523964
H	4.127953	-1.838395	1.280392
H	5.346229	-1.520859	0.049141
H	4.862828	0.863879	0.026019
H	5.090278	0.394864	1.708251
H	3.619788	-0.883068	-1.578302
H	0.128343	0.745030	0.969646
H	0.588138	1.410740	-1.963534
H	-0.863419	-0.477936	-2.473721
H	1.471433	-0.858805	-2.412417
H	0.747002	-2.229204	-1.597000
H	4.082868	-3.166697	-1.232896
H	3.531784	1.571579	3.077961
H	1.801056	1.895961	2.515597
H	-2.790508	1.529681	-2.204291
H	-2.937323	3.174913	-1.337111
H	1.456550	-2.782697	0.757331
H	0.338027	-1.480466	1.159950
H	1.962897	-1.481757	1.848900
H	-4.221668	-2.409895	-0.253077
H	-2.692480	-2.859202	0.527902
H	-2.700006	-0.687502	1.773532
H	-4.138691	0.683093	0.248003
H	-5.528817	-0.346075	0.625429
H	-4.800969	0.663862	1.886050
H	-3.570558	-2.767759	2.839090
H	-5.188500	-2.435966	2.195520
H	-4.477327	-1.354550	3.402055

B3LYP energy = -1155.18051777 a.u.

(1*R*,5*S*,6*R*,7*R*,8*R*,10*R*)-**3**, Conf. K

C	3.491452	-2.608474	-0.041247
C	4.221144	-1.344857	0.457495
C	3.259740	-0.294954	0.972497
C	2.180913	0.041662	-0.039954
C	1.329670	-1.246269	-0.400200
C	2.338197	-2.277788	-0.996263
C	1.210526	1.166153	0.287727
C	0.326821	1.496057	-0.922979
C	-0.592843	0.338302	-1.290842
C	0.270253	-0.921474	-1.495516

O	1.828576	2.441793	0.612688
C	0.966386	3.456031	0.269975
C	-0.136904	2.885159	-0.576883
O	1.692709	-3.466761	-1.462286
C	3.377251	0.259278	2.184205
C	-1.230077	3.575001	-0.909603
O	1.145165	4.598928	0.613823
H	2.704407	0.335546	-0.965722
C	0.635714	-1.838382	0.847640
O	-1.571013	0.110874	-0.238727
C	-2.872376	0.416689	-0.489267
O	-3.262409	0.888530	-1.538931
C	-3.749330	0.086573	0.697934
C	-3.895423	-1.431600	0.970093
C	-4.710020	-1.652756	2.253043
C	-4.527756	-2.165982	-0.221511
H	3.101083	-3.168016	0.820016
H	4.192239	-3.272605	-0.559118
H	4.789266	-0.916722	-0.382474
H	4.951632	-1.608547	1.228814
H	2.757385	-1.830708	-1.907755
H	0.588239	0.895929	1.148049
H	1.010562	1.603594	-1.781533
H	-1.145901	0.557074	-2.206821
H	0.791174	-0.777902	-2.452023
H	-0.379690	-1.789350	-1.636419
H	1.541218	-4.057317	-0.712354
H	4.177144	-0.040257	2.856500
H	2.713206	1.041487	2.534703
H	-2.017272	3.146721	-1.521998
H	-1.347622	4.595130	-0.555363
H	-0.145857	-1.179649	1.227982
H	1.340336	-2.024494	1.661366
H	0.145261	-2.783278	0.588211
H	-3.320504	0.570976	1.582974
H	-4.730755	0.529752	0.504988
H	-2.888056	-1.836742	1.131559
H	-4.796914	-2.720980	2.479466
H	-4.243462	-1.163537	3.115439
H	-5.725795	-1.251955	2.148130
H	-3.939146	-2.048246	-1.137409
H	-5.535606	-1.784698	-0.425830
H	-4.610824	-3.238320	-0.013987

B3LYP energy = -1155.18045736 a.u.

(1*R*,5*S*,6*R*,7*R*,8*R*,10*R*)-**3**, Conf. L

C	4.304773	-1.429254	0.398235
C	4.515064	0.067996	0.701302
C	3.213134	0.766285	1.033738
C	2.153339	0.547044	-0.029469
C	1.840223	-0.999065	-0.203228
C	3.183520	-1.657437	-0.622919
C	0.826875	1.274857	0.119833
C	-0.031517	1.094990	-1.139443
C	-0.458062	-0.350112	-1.338078
C	0.806351	-1.234381	-1.347011

O	0.921312	2.720580	0.259160
C	-0.218375	3.295572	-0.247493
C	-0.980313	2.254323	-1.020319
O	2.942290	-3.053271	-0.833925
C	3.049600	1.491648	2.145761
C	-2.216629	2.447147	-1.482147
O	-0.487013	4.459971	-0.077955
H	2.594412	0.884476	-0.982665
C	1.327437	-1.635535	1.109936
O	-1.367737	-0.719166	-0.267070
C	-2.389371	-1.566560	-0.566140
O	-2.594164	-1.998984	-1.682582
C	-3.217361	-1.879368	0.660542
C	-4.089641	-0.696536	1.152629
C	-5.123738	-0.273585	0.098996
C	-4.771937	-1.071561	2.476421
H	4.064447	-1.972114	1.318595
H	5.237085	-1.861302	0.009575
H	4.953585	0.549298	-0.186645
H	5.236412	0.192554	1.515044
H	3.483057	-1.198746	-1.581156
H	0.289183	0.912392	1.002347
H	0.627778	1.321391	-1.993660
H	-0.991363	-0.485224	-2.282107
H	1.299872	-1.048164	-2.311093
H	0.512866	-2.286963	-1.358920
H	3.771589	-3.480081	-1.084684
H	3.859001	1.595298	2.863874
H	2.131756	2.025256	2.365766
H	-2.745691	1.684830	-2.045930
H	-2.723950	3.389466	-1.297635
H	0.320716	-1.292850	1.352736
H	1.973110	-1.400860	1.959170
H	1.292562	-2.722262	0.995406
H	-3.852843	-2.734134	0.410180
H	-2.535337	-2.179170	1.464495
H	-3.420477	0.152637	1.342036
H	-5.714328	0.576303	0.457691
H	-4.652993	0.021517	-0.844368
H	-5.816160	-1.095105	-0.121874
H	-5.374237	-0.237865	2.853297
H	-4.037873	-1.330492	3.247627
H	-5.439912	-1.931926	2.344990

B3LYP energy = -1155.18021605 a.u.

(1*R*,5*S*,6*R*,7*R*,8*R*,10*R*)-**3**, Conf. M

C	3.490881	-2.603210	-0.020807
C	4.217947	-1.338400	0.475343
C	3.258010	-0.281369	0.978566
C	2.180911	0.049476	-0.038046
C	1.336957	-1.244829	-0.394373
C	2.354572	-2.270399	-0.988244
C	1.203520	1.168397	0.287481
C	0.323631	1.494710	-0.926958
C	-0.594858	0.336682	-1.292515
C	0.269934	-0.926025	-1.486085

O	1.812795	2.448086	0.614633
C	0.948834	3.458113	0.266612
C	-0.147624	2.882052	-0.585845
O	1.741820	-3.515653	-1.343259
C	3.374398	0.284636	2.184911
C	-1.242632	3.565682	-0.925022
O	1.120176	4.602411	0.609489
H	2.706127	0.346400	-0.962222
C	0.649729	-1.849065	0.850646
O	-1.574971	0.114598	-0.244419
C	-2.879667	0.398147	-0.509092
O	-3.268074	0.840325	-1.572135
C	-3.757764	0.084174	0.680978
C	-3.879270	-1.428933	0.991922
C	-4.691532	-1.630357	2.279521
C	-4.498205	-2.203908	-0.180810
H	3.080404	-3.163462	0.826328
H	4.193075	-3.272005	-0.529562
H	4.795193	-0.914013	-0.360877
H	4.941251	-1.599641	1.254164
H	2.789906	-1.814003	-1.893451
H	0.579723	0.892632	1.144989
H	1.009863	1.601924	-1.783676
H	-1.144073	0.549871	-2.212270
H	0.781931	-0.789265	-2.450310
H	-0.399915	-1.785238	-1.601906
H	1.242628	-3.409112	-2.162402
H	4.172153	-0.011360	2.861239
H	2.711192	1.070750	2.528053
H	-2.024476	3.133210	-1.541467
H	-1.367667	4.585484	-0.572504
H	-0.185065	-1.234243	1.189955
H	1.345898	-1.960213	1.684536
H	0.257288	-2.839150	0.600801
H	-3.341412	0.598112	1.555199
H	-4.745160	0.506241	0.471848
H	-2.865836	-1.813337	1.164590
H	-4.759918	-2.693599	2.533602
H	-4.233856	-1.111148	3.128991
H	-5.713917	-1.249800	2.163795
H	-4.560290	-3.272212	0.052832
H	-3.912372	-2.097152	-1.100022
H	-5.513277	-1.847249	-0.393839

B3LYP energy = -1155.17997755 a.u.

(1*R*,5*S*,6*R*,7*R*,8*R*,10*R*)-**3**, Conf. N

C	4.377742	-1.178509	0.399290
C	4.415204	0.293596	0.858229
C	3.035474	0.830754	1.174581
C	2.061383	0.619588	0.031254
C	1.903538	-0.925140	-0.291765
C	3.327803	-1.426404	-0.690551
C	0.669501	1.219298	0.152723
C	-0.099612	1.076570	-1.167396
C	-0.383532	-0.380970	-1.504059
C	0.955681	-1.143715	-1.509864

O	0.632037	2.649608	0.414191
C	-0.521314	3.173722	-0.118710
C	-1.146414	2.146352	-1.020845
O	3.328694	-2.794050	-1.110173
C	2.741048	1.424700	2.336452
C	-2.361840	2.287503	-1.554174
O	-0.895633	4.294239	0.128060
H	2.524879	1.075521	-0.860137
C	1.383463	-1.713151	0.931828
O	-1.284544	-0.973314	-0.527416
C	-2.538604	-1.316825	-0.927866
O	-2.974127	-1.092173	-2.039603
C	-3.292719	-2.021204	0.179375
C	-3.631973	-1.130478	1.400130
C	-4.573231	0.021948	1.020473
C	-4.238774	-1.993074	2.516909
H	4.165575	-1.824266	1.262453
H	5.357386	-1.480429	0.012585
H	4.848402	0.900007	0.048024
H	5.078327	0.404574	1.721982
H	3.623503	-0.873454	-1.592289
H	0.115490	0.741313	0.968235
H	0.584922	1.423609	-1.959169
H	-0.861528	-0.468638	-2.482129
H	1.480886	-0.826656	-2.421113
H	0.764826	-2.213502	-1.630878
H	3.355478	-3.363284	-0.329547
H	3.495747	1.533015	3.111272
H	1.764509	1.847470	2.545072
H	-2.794914	1.535573	-2.206653
H	-2.946087	3.175492	-1.330104
H	0.353156	-1.451187	1.174632
H	1.991847	-1.538476	1.822011
H	1.385279	-2.786806	0.712474
H	-4.211818	-2.417326	-0.262475
H	-2.680257	-2.867257	0.513334
H	-2.691943	-0.702510	1.771962
H	-5.523529	-0.362686	0.630488
H	-4.137167	0.672368	0.255874
H	-4.796019	0.642297	1.895178
H	-5.175170	-2.460089	2.187175
H	-3.555541	-2.792137	2.826135
H	-4.464617	-1.384394	3.399050

B3LYP energy = -1155.17996322 a.u.

(1*R*,5*S*,6*R*,7*R*,8*R*,10*R*)-**3**, Conf. O

C	4.321136	-1.412133	0.377282
C	4.511001	0.082220	0.706734
C	3.202245	0.763515	1.046057
C	2.148700	0.549108	-0.023778
C	1.842586	-0.996392	-0.210236
C	3.195200	-1.651046	-0.635914
C	0.821713	1.277115	0.121336
C	-0.034186	1.097693	-1.139583
C	-0.456551	-0.348453	-1.340606
C	0.812073	-1.225858	-1.356587

O	0.915319	2.722501	0.262100
C	-0.225270	3.296634	-0.244461
C	-0.985137	2.255220	-1.019413
O	3.057744	-3.041767	-0.939810
C	3.027865	1.472695	2.166898
C	-2.221314	2.446797	-1.482104
O	-0.495981	4.460114	-0.073023
H	2.595090	0.891068	-0.972977
C	1.326829	-1.639193	1.097565
O	-1.358976	-0.724396	-0.265015
C	-2.383143	-1.569983	-0.561869
O	-2.591184	-2.001996	-1.677674
C	-3.209882	-1.880375	0.666325
C	-4.092590	-0.700362	1.147128
C	-5.127853	-0.294008	0.088138
C	-4.774491	-1.070261	2.472565
H	4.101713	-1.963388	1.301828
H	5.248049	-1.832268	-0.028531
H	4.946217	0.580163	-0.173356
H	5.230077	0.202292	1.523225
H	3.484523	-1.198163	-1.593681
H	0.280928	0.915114	1.002205
H	0.625804	1.326838	-1.992469
H	-0.993914	-0.483724	-2.282245
H	1.306340	-1.027426	-2.317591
H	0.524837	-2.280147	-1.382975
H	3.077993	-3.548035	-0.116783
H	3.833556	1.574710	2.889387
H	2.104746	1.996159	2.389716
H	-2.748535	1.684765	-2.048023
H	-2.730271	3.388057	-1.296724
H	1.213143	-2.720576	0.961575
H	0.343698	-1.256483	1.374572
H	2.000175	-1.464220	1.939785
H	-3.838631	-2.741775	0.421715
H	-2.527483	-2.168080	1.474365
H	-3.430500	0.155580	1.331066
H	-5.813522	-1.122539	-0.127469
H	-5.725683	0.554050	0.439021
H	-4.657736	-0.002912	-0.856729
H	-4.040078	-1.317798	3.247215
H	-5.435989	-1.936405	2.346533
H	-5.383514	-0.238114	2.841858

B3LYP energy = -1155.17962269 a.u.

(1*R*,5*S*,6*R*,7*R*,8*R*,10*R*)-**3**, Conf. Q

C	4.279435	-1.504719	0.371580
C	4.523856	-0.012104	0.666681
C	3.246064	0.724362	1.008039
C	2.168344	0.523604	-0.040602
C	1.814911	-1.016018	-0.189589
C	3.141203	-1.717319	-0.626863
C	0.865135	1.290186	0.117292
C	-0.018868	1.118052	-1.125367
C	-0.489346	-0.316068	-1.295974
C	0.752465	-1.235302	-1.309345

O	1.002257	2.734624	0.233050
C	-0.129226	3.334421	-0.261715
C	-0.934908	2.303464	-1.003960
O	2.995772	-3.132926	-0.781408
C	3.112523	1.463677	2.114803
C	-2.177495	2.521457	-1.436284
O	-0.362770	4.508101	-0.105973
H	2.607098	0.838337	-1.003026
C	1.310205	-1.625986	1.137511
O	-1.398211	-0.631523	-0.209447
C	-2.338153	-1.588782	-0.436066
O	-2.442027	-2.179913	-1.492400
C	-3.215079	-1.793881	0.778458
C	-4.200139	-0.626281	1.043024
C	-5.197606	-0.449574	-0.111381
C	-4.932068	-0.856520	2.373478
H	4.042628	-2.041474	1.296519
H	5.185576	-1.965905	-0.035214
H	4.966520	0.455123	-0.226938
H	5.255892	0.098253	1.472919
H	3.438367	-1.278088	-1.594442
H	0.331459	0.955896	1.013372
H	0.631692	1.315563	-1.993319
H	-1.036698	-0.452209	-2.232490
H	1.228832	-1.073269	-2.287992
H	0.408157	-2.274271	-1.296645
H	2.492050	-3.322938	-1.582772
H	3.931710	1.551495	2.823789
H	2.211676	2.023650	2.339617
H	-2.741856	1.765454	-1.973620
H	-2.654950	3.479361	-1.252424
H	1.261060	-2.714444	1.039995
H	0.312288	-1.265322	1.391825
H	1.971898	-1.389939	1.973451
H	-3.769101	-2.724642	0.623772
H	-2.565688	-1.919007	1.652472
H	-3.606985	0.292710	1.134309
H	-4.695513	-0.263247	-1.066465
H	-5.814755	-1.347741	-0.234933
H	-5.867971	0.394099	0.084769
H	-4.229141	-0.939893	3.209820
H	-5.527861	-1.777190	2.340659
H	-5.614090	-0.027290	2.590108

B3LYP energy = -1155.17958170 a.u.

(1*R*,5*S*,6*R*,7*R*,8*R*,10*R*)-**3**, Conf. R

C	4.119841	-2.074038	0.119028
C	4.629647	-0.644932	0.394541
C	3.518122	0.274486	0.855551
C	2.333747	0.268542	-0.092601
C	1.728693	-1.192557	-0.227245
C	2.879128	-2.076626	-0.782270
C	1.184343	1.222523	0.189761
C	0.181149	1.223791	-0.971570
C	-0.517179	-0.120248	-1.123230
C	0.557329	-1.216685	-1.256357

O	1.555232	2.624317	0.314291
C	0.490217	3.404450	-0.066983
C	-0.527208	2.530784	-0.746931
O	2.366667	-3.401545	-0.967754
C	3.605669	1.002643	1.974366
C	-1.757713	2.944061	-1.057286
O	0.458615	4.595196	0.127590
H	2.730280	0.535071	-1.086996
C	1.246026	-1.745271	1.134352
O	-1.363225	-0.376003	0.030842
C	-2.709760	-0.439827	-0.152010
O	-3.246025	-0.289731	-1.231535
C	-3.400891	-0.696571	1.173598
C	-4.877902	-1.137894	1.114854
C	-5.814182	-0.038724	0.586632
C	-5.060352	-2.460251	0.352274
H	3.877374	-2.578332	1.060571
H	4.913645	-2.662608	-0.361086
H	5.057519	-0.238034	-0.534818
H	5.439618	-0.666704	1.130265
H	3.160218	-1.663956	-1.766783
H	0.683005	0.950039	1.124842
H	0.777268	1.346789	-1.891219
H	-1.159492	-0.130590	-2.006447
H	0.976484	-1.107751	-2.266215
H	0.078285	-2.198935	-1.231430
H	3.077351	-3.968831	-1.293249
H	4.489540	0.946386	2.604683
H	2.829240	1.692758	2.285067
H	-2.475411	2.293503	-1.547724
H	-2.063575	3.957201	-0.812684
H	0.342606	-1.237576	1.473808
H	2.004620	-1.635739	1.912660
H	1.011817	-2.808038	1.029862
H	-2.802097	-1.438552	1.714368
H	-3.309893	0.233647	1.751853
H	-5.153778	-1.324242	2.162784
H	-6.859828	-0.352927	0.678715
H	-5.696407	0.890658	1.156721
H	-5.614124	0.178749	-0.465728
H	-4.828043	-2.339156	-0.710145
H	-6.095610	-2.809393	0.432333
H	-4.413071	-3.247247	0.757680

B3LYP energy = -1155.17950465 a.u.

(1*R*,5*S*,6*R*,7*R*,8*R*,10*R*)-**3**, Conf. S

C	3.826909	-2.361561	0.003625
C	4.478511	-0.998843	0.314910
C	3.469168	0.009794	0.822650
C	2.278835	0.152331	-0.107670
C	1.530990	-1.235569	-0.277213
C	2.579869	-2.212371	-0.876464
C	1.232274	1.205261	0.220425
C	0.224594	1.347489	-0.928242
C	-0.606020	0.083564	-1.113644
C	0.351814	-1.111115	-1.290002

O	1.745195	2.555715	0.390409
C	0.763582	3.453348	0.046582
C	-0.342401	2.714104	-0.654071
O	1.935101	-3.473134	-1.093843
C	3.639607	0.685572	1.964327
C	-1.518837	3.274624	-0.943488
O	0.855572	4.633337	0.283428
H	2.687985	0.410688	-1.099149
C	1.012431	-1.780407	1.074124
O	-1.464634	-0.131235	0.040459
C	-2.803712	0.049203	-0.108664
O	-3.321976	0.381686	-1.157317
C	-3.540837	-0.203803	1.190570
C	-4.678010	-1.255575	1.099610
C	-5.887047	-0.769803	0.285809
C	-4.160334	-2.610500	0.592492
H	3.548615	-2.869632	0.933262
H	4.552158	-3.009269	-0.507760
H	4.932519	-0.605782	-0.607932
H	5.291986	-1.124412	1.036509
H	2.888123	-1.798465	-1.852254
H	0.712638	0.950139	1.150477
H	0.820899	1.439019	-1.851381
H	-1.252662	0.167239	-1.989751
H	0.768905	-1.015664	-2.302113
H	-0.223891	-2.040291	-1.286138
H	2.581713	-4.098192	-1.446019
H	4.519965	0.520217	2.580155
H	2.937488	1.436621	2.308401
H	-2.309381	2.729466	-1.449464
H	-1.702231	4.307156	-0.660106
H	0.169916	-1.195459	1.444789
H	1.789603	-1.772448	1.841596
H	0.669557	-2.809919	0.941379
H	-2.814894	-0.505481	1.950178
H	-3.965697	0.758805	1.502120
H	-5.012347	-1.395607	2.137176
H	-6.695033	-1.508748	0.330415
H	-6.276514	0.175940	0.679992
H	-5.623351	-0.609466	-0.763080
H	-3.314180	-2.968399	1.189879
H	-3.834444	-2.545508	-0.452490
H	-4.949821	-3.367941	0.642227

B3LYP energy = -1155.17927986 a.u.

(1*R*,5*S*,6*R*,7*R*,8*S*,10*R*)-**3**, Conf. A

C	3.998905	-2.425444	-0.262970
C	4.665500	-1.072247	-0.581072
C	3.915012	0.086320	0.041002
C	2.441940	0.084853	-0.324720
C	1.745885	-1.259309	0.147804
C	2.497536	-2.408840	-0.575955
C	1.588412	1.241077	0.168129
C	0.167527	1.188712	-0.417894
C	-0.555711	-0.040486	0.101425

C	0.254491	-1.287473	-0.296417
O	2.058360	2.564813	-0.204369
C	0.983575	3.421027	-0.233198
C	-0.278744	2.605002	-0.172910
O	1.870613	-3.643842	-0.213357
C	4.514098	0.993007	0.821255
C	-1.477871	3.146188	0.050469
O	1.111328	4.618037	-0.314424
H	2.389889	0.094495	-1.426023
C	1.826444	-1.449779	1.680892
O	-1.862071	-0.096393	-0.534256
C	-2.861792	-0.722692	0.141019
O	-2.730584	-1.179422	1.258200
C	-4.122654	-0.796640	-0.696345
C	-5.419697	-0.881384	0.127267
C	-5.687893	0.424599	0.890868
C	-6.602778	-1.244141	-0.782212
H	4.140756	-2.680353	0.792975
H	4.479060	-3.220756	-0.849335
H	4.681094	-0.934593	-1.673337
H	5.708515	-1.075667	-0.249414
H	2.367230	-2.247539	-1.659930
H	1.541379	1.228983	1.264389
H	0.275983	1.070341	-1.508550
H	-0.714333	0.011200	1.181418
H	0.202781	-1.370740	-1.390456
H	-0.220832	-2.180689	0.115905
H	2.343110	-4.371233	-0.638076
H	5.575622	0.918241	1.042969
H	3.989213	1.845769	1.236680
H	-2.380766	2.546779	0.082175
H	-1.565135	4.217902	0.203348
H	1.148105	-0.777328	2.212635
H	2.831947	-1.265268	2.065687
H	1.538280	-2.472648	1.935251
H	-4.144032	0.053013	-1.388441
H	-4.016192	-1.695369	-1.320649
H	-5.286765	-1.683867	0.863191
H	-6.601446	0.344109	1.489843
H	-4.867150	0.666767	1.573505
H	-5.820059	1.264850	0.196755
H	-7.527480	-1.331537	-0.201722
H	-6.438384	-2.198860	-1.294907
H	-6.764662	-0.475634	-1.548848

B3LYP energy = -1155.18326127 a.u.

(1*R*,5*S*,6*R*,7*R*,8*S*,10*R*)-**3**, Conf. B

C	3.440490	-2.902526	-0.353048
C	4.323653	-1.697977	-0.734002
C	3.841472	-0.417645	-0.085346
C	2.373324	-0.147518	-0.359760
C	1.471597	-1.337087	0.175553
C	1.951365	-2.609862	-0.573319
C	1.780229	1.149087	0.165071
C	0.340275	1.358266	-0.332737
C	-0.566166	0.285784	0.243956

C	-0.023259	-1.090877	-0.179251
O	2.461631	2.361503	-0.256880
C	1.563866	3.401630	-0.231864
C	0.179018	2.833461	-0.081888
O	1.131718	-3.705269	-0.151732
C	4.646718	0.366873	0.639796
C	-0.883416	3.587673	0.206114
O	1.904735	4.554149	-0.338086
H	2.254450	-0.133978	-1.455821
C	1.610861	-1.533228	1.703789
O	-1.892652	0.469387	-0.322808
C	-2.954348	0.043776	0.411114
O	-2.847642	-0.467644	1.506981
C	-4.260720	0.338598	-0.299017
C	-5.414504	-0.601889	0.091935
C	-6.744107	-0.063169	-0.456302
C	-5.157074	-2.037788	-0.390179
H	3.600230	-3.173272	0.696248
H	3.727568	-3.776120	-0.954109
H	4.294380	-1.570564	-1.827257
H	5.367273	-1.892949	-0.467997
H	1.784057	-2.432897	-1.649860
H	1.801520	1.152104	1.262100
H	0.357291	1.218963	-1.426143
H	-0.653410	0.369810	1.330110
H	-0.154233	-1.168208	-1.267057
H	-0.629976	-1.878386	0.273411
H	1.427022	-4.508039	-0.600129
H	5.688232	0.097863	0.796261
H	4.316284	1.304966	1.071472
H	-1.877495	3.165037	0.299794
H	-0.762567	4.657491	0.349049
H	1.116809	-0.736783	2.266885
H	2.655488	-1.553055	2.022880
H	1.141689	-2.477057	1.992037
H	-4.521208	1.375303	-0.041009
H	-4.088189	0.327770	-1.381270
H	-5.468314	-0.615171	1.187429
H	-6.730758	-0.016842	-1.552717
H	-7.577483	-0.710529	-0.163028
H	-6.955662	0.944759	-0.081071
H	-5.084432	-2.076567	-1.484813
H	-5.975168	-2.700891	-0.088579
H	-4.231350	-2.443266	0.029691

B3LYP energy = -1155.18311149 a.u.

(1*R*,5*S*,6*R*,7*R*,8*S*,10*R*)-**3**, Conf. C

C	3.984642	-2.440337	-0.291403
C	4.657397	-1.082558	-0.576375
C	3.909837	0.069686	0.060133
C	2.440024	0.080863	-0.316633
C	1.733025	-1.261424	0.142529
C	2.480798	-2.418383	-0.591151
C	1.591846	1.244004	0.169984
C	0.172148	1.200330	-0.419729
C	-0.558412	-0.025722	0.097421

C	0.244805	-1.274428	-0.308532
O	2.072003	2.563656	-0.202789
C	1.002614	3.427233	-0.233729
C	-0.265310	2.619516	-0.175565
O	1.882682	-3.695137	-0.355697
C	4.506943	0.961805	0.858513
C	-1.461536	3.168311	0.044839
O	1.138475	4.623219	-0.314467
H	2.396630	0.092201	-1.418365
C	1.804811	-1.457909	1.674058
O	-1.867234	-0.070388	-0.533098
C	-2.858961	-0.720618	0.131275
O	-2.716444	-1.205935	1.235237
C	-4.124826	-0.780956	-0.699075
C	-5.416457	-0.881328	0.131445
C	-5.680051	0.410748	0.919920
C	-6.605291	-1.228496	-0.776553
H	4.143552	-2.712716	0.761073
H	4.447611	-3.228234	-0.895680
H	4.679423	-0.926540	-1.665854
H	5.698782	-1.097139	-0.239749
H	2.342120	-2.256711	-1.668415
H	1.540296	1.236012	1.266153
H	0.282344	1.081668	-1.510134
H	-0.712984	0.022958	1.178390
H	0.199208	-1.345851	-1.403506
H	-0.240337	-2.169603	0.088119
H	2.185846	-4.036219	0.496364
H	5.566412	0.879406	1.087191
H	3.982740	1.811327	1.281661
H	-2.368089	2.574302	0.074053
H	-1.542477	4.240613	0.197088
H	2.823021	-1.362681	2.057526
H	1.417801	-2.446394	1.943257
H	1.189344	-0.733419	2.213221
H	-4.151420	0.080658	-1.375924
H	-4.021552	-1.668712	-1.339384
H	-5.278330	-1.696839	0.851933
H	-4.854570	0.641908	1.600752
H	-5.817835	1.262944	0.241665
H	-6.589174	0.318927	1.523950
H	-6.444037	-2.174109	-1.306735
H	-6.772292	-0.446914	-1.528711
H	-7.526168	-1.326174	-0.191657

B3LYP energy = -1155.18281616 a.u.

(1*R*,5*S*,6*R*,7*R*,8*S*,10*R*)-**3**, Conf. D

C	3.976489	-2.447964	-0.269531
C	4.651870	-1.097135	-0.573171
C	3.910380	0.067358	0.047741
C	2.438325	0.078919	-0.321988
C	1.734329	-1.260512	0.149872
C	2.479806	-2.421860	-0.581321
C	1.594274	1.242135	0.170595
C	0.173653	1.202971	-0.417241
C	-0.561950	-0.017815	0.102107

C	0.240829	-1.272785	-0.289377
O	2.074531	2.562740	-0.199538
C	1.007330	3.427874	-0.228255
C	-0.262104	2.622327	-0.171554
O	1.968789	-3.713352	-0.238022
C	4.512916	0.969276	0.830906
C	-1.457508	3.172382	0.049777
O	1.143999	4.623882	-0.307047
H	2.389056	0.089457	-1.423785
C	1.817118	-1.457673	1.680592
O	-1.868241	-0.057529	-0.534789
C	-2.855688	-0.737773	0.106055
O	-2.702085	-1.276897	1.183131
C	-4.130374	-0.755024	-0.712925
C	-5.413695	-0.876146	0.128354
C	-5.651500	0.383563	0.975207
C	-6.617154	-1.168993	-0.779445
H	4.114130	-2.714225	0.784235
H	4.438232	-3.246875	-0.859179
H	4.676736	-0.953174	-1.664653
H	5.692723	-1.110556	-0.235058
H	2.351912	-2.259423	-1.664993
H	1.545909	1.228300	1.266904
H	0.281898	1.083592	-1.507780
H	-0.720283	0.034669	1.182246
H	0.181441	-1.359165	-1.383993
H	-0.256068	-2.147578	0.142408
H	1.105509	-3.841931	-0.650571
H	5.572883	0.885617	1.056644
H	3.993668	1.826036	1.245289
H	-2.365184	2.580017	0.077802
H	-1.536522	4.244501	0.204208
H	1.127005	-0.802420	2.219208
H	2.820041	-1.259099	2.064372
H	1.551445	-2.488951	1.928232
H	-4.156721	0.133070	-1.354472
H	-4.043972	-1.617913	-1.388802
H	-5.277607	-1.723878	0.811008
H	-4.815853	0.574509	1.656067
H	-5.785508	1.266200	0.336307
H	-6.555062	0.276746	1.584912
H	-7.532147	-1.280842	-0.188004
H	-6.474371	-2.092672	-1.351967
H	-6.783319	-0.353513	-1.494848

B3LYP energy = -1155.18265837 a.u.

(1*R*,5*S*,6*R*,7*R*,8*S*,10*R*)-**3**, Conf. E

C	3.432237	-2.908050	-0.383052
C	4.321470	-1.696799	-0.727899
C	3.836703	-0.425472	-0.064284
C	2.373024	-0.148077	-0.352297
C	1.463266	-1.337045	0.168648
C	1.941572	-2.614539	-0.590178
C	1.781464	1.152305	0.165672
C	0.343296	1.364930	-0.335933
C	-0.565737	0.293687	0.239780

C	-0.026721	-1.080976	-0.193889
O	2.468249	2.360871	-0.257401
C	1.573659	3.404363	-0.234926
C	0.186459	2.840947	-0.087019
O	1.139280	-3.760216	-0.294592
C	4.634469	0.346070	0.682671
C	-0.874347	3.599206	0.196404
O	1.918608	4.555483	-0.341312
H	2.264252	-0.134033	-1.449493
C	1.592308	-1.537498	1.696004
O	-1.894517	0.485179	-0.317451
C	-2.950291	0.038311	0.412641
O	-2.833578	-0.498160	1.495680
C	-4.262033	0.344561	-0.281969
C	-5.412339	-0.604091	0.099380
C	-6.746275	-0.054867	-0.427569
C	-5.158384	-2.029983	-0.413262
H	3.604516	-3.200363	0.661911
H	3.706185	-3.769400	-1.002224
H	4.304258	-1.551132	-1.818905
H	5.361742	-1.899732	-0.454572
H	1.765188	-2.436522	-1.659470
H	1.798788	1.160673	1.262852
H	0.362307	1.224704	-1.429145
H	-0.646547	0.372397	1.327008
H	-0.148987	-1.146318	-1.283331
H	-0.641271	-1.872027	0.242099
H	1.438833	-4.150826	0.537187
H	5.673366	0.072513	0.848266
H	4.301226	1.279360	1.122832
H	-1.870099	3.179893	0.287186
H	-0.750346	4.668893	0.337602
H	1.173345	-0.701157	2.261421
H	2.631303	-1.649771	2.014351
H	1.032006	-2.425974	2.005779
H	-4.521338	1.376139	-0.002909
H	-4.097045	0.354164	-1.365401
H	-5.457672	-0.638980	1.194815
H	-6.741538	0.013155	-1.522887
H	-7.577197	-0.708192	-0.140700
H	-6.955068	0.945346	-0.030717
H	-4.229213	-2.443517	-0.009192
H	-5.094232	-2.047199	-1.508895
H	-5.973887	-2.699242	-0.118400

B3LYP energy = -1155.18262340 a.u.

(1*R*,5*S*,6*R*,7*R*,8*S*,10*R*)-**3**, Conf. F

C	3.416672	-2.920779	-0.360953
C	4.304952	-1.720384	-0.738390
C	3.835762	-0.433910	-0.093063
C	2.368241	-0.154046	-0.361148
C	1.465570	-1.337070	0.185063
C	1.932189	-2.621947	-0.570480
C	1.787903	1.148508	0.163074
C	0.346246	1.366810	-0.326214
C	-0.564176	0.305645	0.262805

C	-0.031245	-1.078038	-0.153658
O	2.473865	2.355645	-0.266290
C	1.583777	3.401888	-0.238598
C	0.195632	2.843771	-0.079870
O	1.218506	-3.793780	-0.165295
C	4.648692	0.349487	0.624779
C	-0.860799	3.605827	0.209420
O	1.930825	4.551938	-0.349446
H	2.243497	-0.143909	-1.456952
C	1.618419	-1.534758	1.710482
O	-1.893350	0.500078	-0.293489
C	-2.947614	0.024906	0.421369
O	-2.826087	-0.558256	1.479327
C	-4.261538	0.362361	-0.253956
C	-5.410831	-0.602835	0.086988
C	-6.746535	-0.029509	-0.408830
C	-5.159222	-2.003394	-0.492273
H	3.577840	-3.198390	0.686421
H	3.681328	-3.796255	-0.963182
H	4.278347	-1.592520	-1.831859
H	5.347128	-1.922533	-0.472119
H	1.759092	-2.447690	-1.646139
H	1.816126	1.153559	1.260090
H	0.354352	1.222332	-1.419034
H	-0.640830	0.394148	1.349532
H	-0.178689	-1.161018	-1.240097
H	-0.649970	-1.842746	0.326384
H	0.320807	-3.766276	-0.519300
H	5.688896	0.073948	0.778272
H	4.326800	1.291914	1.053521
H	-1.857454	3.190303	0.307722
H	-0.732064	4.675296	0.347945
H	2.665604	-1.531831	2.020843
H	1.177488	-2.493359	1.997157
H	1.108762	-0.754371	2.282636
H	-4.519696	1.379647	0.074082
H	-4.100636	0.423146	-1.336358
H	-5.452144	-0.688941	1.179690
H	-6.745930	0.090389	-1.499682
H	-7.576540	-0.695474	-0.149898
H	-6.953605	0.950756	0.035655
H	-5.974694	-2.685244	-0.228164
H	-4.230315	-2.435804	-0.107243
H	-5.096152	-1.969277	-1.587664

B3LYP energy = -1155.18256710 a.u.

(1*R*,5*S*,6*R*,7*R*,8*S*,10*R*)-**3**, Conf. G

C	3.258038	-2.928135	-0.430844
C	4.107763	-1.748740	-0.944568
C	3.709241	-0.440527	-0.294353
C	2.222767	-0.161428	-0.419141
C	1.374162	-1.319246	0.253835
C	1.756768	-2.622253	-0.498725
C	1.701848	1.161098	0.117497
C	0.218622	1.370470	-0.231269
C	-0.628284	0.333260	0.483798

C	-0.148728	-1.065525	0.056729
O	2.345288	2.347997	-0.420535
C	1.464165	3.399860	-0.342479
C	0.098397	2.856020	-0.022792
O	0.976215	-3.692112	0.045127
C	4.594027	0.357696	0.313695
C	-0.919268	3.634269	0.350407
O	1.801736	4.542709	-0.531416
H	1.986074	-0.185717	-1.495712
C	1.678057	-1.465736	1.763563
O	-2.008521	0.514076	0.066838
C	-2.983797	0.134220	0.936600
O	-2.755496	-0.311372	2.042813
C	-4.358407	0.341078	0.339684
C	-4.697171	-0.647298	-0.805212
C	-4.691900	-2.105024	-0.321567
C	-6.051001	-0.275555	-1.427596
H	3.527691	-3.168289	0.603390
H	3.469358	-3.824155	-1.030406
H	3.964515	-1.658178	-2.032470
H	5.171596	-1.948760	-0.782949
H	1.475980	-2.478627	-1.556432
H	1.840406	1.203654	1.205170
H	0.117049	1.188791	-1.313862
H	-0.590316	0.460384	1.568630
H	-0.400723	-1.181686	-1.006048
H	-0.708113	-1.827729	0.604177
H	1.224150	-4.515404	-0.394726
H	5.643931	0.081424	0.367668
H	4.319432	1.313506	0.745434
H	-1.900287	3.228387	0.570418
H	-0.773734	4.706995	0.437637
H	2.751032	-1.478561	1.968335
H	1.242878	-2.397855	2.132234
H	1.246348	-0.650016	2.349237
H	-5.082362	0.235913	1.153219
H	-4.416232	1.365601	-0.045970
H	-3.923950	-0.534314	-1.576328
H	-4.919119	-2.785908	-1.149094
H	-3.722874	-2.398205	0.095026
H	-5.447132	-2.261095	0.458354
H	-6.047736	0.749383	-1.815143
H	-6.858083	-0.353449	-0.688496
H	-6.295348	-0.947492	-2.257484

B3LYP energy = -1155.18220855 a.u.

(1*R*,5*S*,6*R*,7*R*,8*S*,10*R*)-**3**, Conf. H

C	4.272346	-1.926731	-0.418294
C	4.687012	-0.487535	-0.782226
C	3.806216	0.538896	-0.102204
C	2.331146	0.297818	-0.366233
C	1.892458	-1.134667	0.150445
C	2.769598	-2.157450	-0.621758
C	1.338958	1.307328	0.184798
C	-0.092764	1.025768	-0.303151
C	-0.577677	-0.302580	0.249736

C	0.400274	-1.404596	-0.198948
O	1.567024	2.684879	-0.218215
C	0.371552	3.360155	-0.169683
C	-0.738056	2.355474	-0.017759
O	2.379640	-3.472262	-0.211537
C	4.304995	1.532725	0.641367
C	-1.984566	2.703622	0.306791
O	0.301643	4.561375	-0.259272
H	2.204384	0.288042	-1.461437
C	2.100747	-1.295351	1.674578
O	-1.888973	-0.570269	-0.318165
C	-2.716707	-1.398337	0.374912
O	-2.430521	-1.877389	1.453412
C	-4.016153	-1.622381	-0.368106
C	-4.932523	-0.375078	-0.430554
C	-6.151962	-0.668258	-1.317137
C	-5.367099	0.084153	0.969451
H	4.529486	-2.142565	0.624495
H	4.831929	-2.640821	-1.037879
H	4.599965	-0.359187	-1.872295
H	5.738392	-0.319951	-0.528392
H	2.538285	-2.035316	-1.694141
H	1.369132	1.299762	1.281584
H	-0.040436	0.923247	-1.399499
H	-0.684682	-0.274682	1.337155
H	0.296711	-1.500474	-1.288367
H	0.099837	-2.359472	0.237918
H	2.926639	-4.119749	-0.674395
H	5.377635	1.629646	0.788370
H	3.680905	2.291895	1.099270
H	-2.776644	1.970836	0.409208
H	-2.225182	3.749384	0.474449
H	3.093509	-0.967766	1.991596
H	1.977167	-2.345845	1.948782
H	1.371777	-0.719247	2.250639
H	-3.773395	-1.938834	-1.389169
H	-4.535067	-2.444549	0.133760
H	-4.358248	0.433199	-0.902272
H	-6.798180	0.212918	-1.393660
H	-5.852016	-0.953653	-2.331627
H	-6.752861	-1.486118	-0.900606
H	-4.512433	0.316286	1.613800
H	-5.951130	-0.696539	1.471630
H	-5.992753	0.981026	0.905157

B3LYP energy = -1155.18193308 a.u.

(1*R*,5*S*,6*R*,7*R*,8*S*,10*R*)-**3**, Conf. I

C	3.252135	-2.929074	-0.462177
C	4.103959	-1.740971	-0.952456
C	3.705783	-0.440070	-0.288212
C	2.220507	-0.158590	-0.416478
C	1.370114	-1.317195	0.251375
C	1.749747	-2.626410	-0.508806
C	1.700255	1.166163	0.116117
C	0.216098	1.375669	-0.228609
C	-0.628316	0.337528	0.488580

C	-0.150507	-1.059102	0.053027
O	2.342984	2.351343	-0.425701
C	1.462749	3.404244	-0.343990
C	0.097678	2.861269	-0.019206
O	0.977093	-3.751401	-0.083569
C	4.588296	0.350992	0.332447
C	-0.918175	3.639758	0.358410
O	1.800618	4.546769	-0.533340
H	1.985873	-0.184657	-1.493538
C	1.673543	-1.461816	1.760299
O	-2.010652	0.521771	0.081833
C	-2.978989	0.112706	0.946173
O	-2.740485	-0.360229	2.039084
C	-4.357923	0.325526	0.362143
C	-4.695675	-0.634851	-0.806719
C	-4.681389	-2.104470	-0.360901
C	-6.053473	-0.253783	-1.414676
H	3.541364	-3.185370	0.566290
H	3.447218	-3.815329	-1.076039
H	3.963422	-1.636816	-2.039282
H	5.167248	-1.944499	-0.791254
H	1.454675	-2.484884	-1.557159
H	1.840576	1.213183	1.203426
H	0.111249	1.194325	-1.310893
H	-0.583380	0.461225	1.573747
H	-0.398636	-1.166715	-1.011395
H	-0.715189	-1.825341	0.589582
H	1.363443	-4.116132	0.723843
H	5.637868	0.073845	0.388215
H	4.312504	1.303049	0.771885
H	-1.898543	3.233933	0.581452
H	-0.771896	4.712377	0.445685
H	1.307287	-0.610609	2.339560
H	2.743353	-1.551468	1.962264
H	1.165183	-2.344865	2.161645
H	-5.076938	0.194298	1.176280
H	-4.424260	1.359449	0.003887
H	-3.925879	-0.498158	-1.577383
H	-5.432876	-2.284299	0.417560
H	-4.908049	-2.764957	-1.204800
H	-3.709167	-2.403666	0.043741
H	-6.056556	0.780967	-1.775243
H	-6.857799	-0.355140	-0.675417
H	-6.297059	-0.904910	-2.261163

B3LYP energy = -1155.18175922 a.u.

(1*R*,5*S*,6*R*,7*R*,8*S*,10*R*)-**3**, Conf. J

C	3.251187	-2.933751	-0.452412
C	4.099908	-1.751372	-0.957305
C	3.708149	-0.443166	-0.303885
C	2.221606	-0.160921	-0.420588
C	1.379007	-1.319277	0.257797
C	1.754002	-2.628895	-0.506204
C	1.707281	1.162521	0.120065
C	0.222637	1.375901	-0.219977
C	-0.622695	0.343261	0.501708

C	-0.145188	-1.058617	0.078434
O	2.349076	2.349241	-0.420178
C	1.471001	3.402744	-0.335727
C	0.105470	2.861387	-0.009939
O	1.084239	-3.783814	0.008218
C	4.595916	0.353932	0.301319
C	-0.909412	3.640718	0.368346
O	1.808880	4.545379	-0.524277
H	1.978787	-0.184550	-1.496214
C	1.697584	-1.473335	1.762267
O	-2.004071	0.529611	0.091634
C	-2.975581	0.094263	0.939819
O	-2.739148	-0.422916	2.012829
C	-4.352807	0.333912	0.362339
C	-4.709446	-0.619319	-0.807229
C	-4.729733	-2.088814	-0.360943
C	-6.057069	-0.208926	-1.418667
H	3.524423	-3.185549	0.578198
H	3.443668	-3.826927	-1.056026
H	3.957579	-1.654720	-2.045003
H	5.163459	-1.954271	-0.797420
H	1.464327	-2.484097	-1.560918
H	1.852268	1.202879	1.207121
H	0.113773	1.193871	-1.301768
H	-0.577541	0.470774	1.586352
H	-0.413875	-1.178225	-0.981092
H	-0.709262	-1.801111	0.651992
H	0.152775	-3.759206	-0.244143
H	5.645511	0.075813	0.350764
H	4.324986	1.309770	0.735401
H	-1.890325	3.236348	0.591699
H	-0.761553	4.713080	0.456026
H	1.251753	-0.675961	2.362984
H	2.772377	-1.464368	1.955789
H	1.291420	-2.422010	2.123630
H	-5.071594	0.211622	1.178082
H	-4.403336	1.368979	0.005889
H	-3.935258	-0.499429	-1.576584
H	-4.958823	-2.745502	-1.207224
H	-3.769682	-2.405634	0.059558
H	-5.494250	-2.253542	0.407890
H	-6.314787	-0.856825	-2.263448
H	-6.035218	0.824308	-1.782514
H	-6.864322	-0.289002	-0.680121

B3LYP energy = -1155.18155914 a.u.

(1*R*,5*S*,6*R*,7*R*,8*S*,10*R*)-**3**, Conf. K

C	4.267677	-1.931518	-0.441415
C	4.688231	-0.484011	-0.763981
C	3.804196	0.532724	-0.074112
C	2.331675	0.299374	-0.356716
C	1.884683	-1.135890	0.142475
C	2.764560	-2.160336	-0.640669
C	1.337119	1.311972	0.184925
C	-0.092563	1.033209	-0.311067
C	-0.580356	-0.294950	0.240394

C	0.394681	-1.395439	-0.217465
O	1.571615	2.687989	-0.218440
C	0.377291	3.366591	-0.177005
C	-0.735921	2.364875	-0.030501
O	2.403318	-3.516072	-0.367492
C	4.297068	1.513677	0.690260
C	-1.983849	2.716787	0.284460
O	0.311106	4.567781	-0.267695
H	2.217883	0.294418	-1.453452
C	2.082133	-1.307074	1.665992
O	-1.895502	-0.558639	-0.319037
C	-2.710906	-1.401458	0.371065
O	-2.408688	-1.893736	1.439479
C	-4.016592	-1.625107	-0.360485
C	-4.933531	-0.378197	-0.417977
C	-6.163019	-0.675764	-1.289068
C	-5.351982	0.086946	0.985012
H	4.537513	-2.166469	0.597464
H	4.813661	-2.636923	-1.077542
H	4.613503	-0.333254	-1.851858
H	5.737560	-0.325384	-0.496058
H	2.529639	-2.034399	-1.706182
H	1.358996	1.307713	1.282077
H	-0.034705	0.929911	-1.407037
H	-0.680953	-0.269399	1.328630
H	0.297241	-1.476233	-1.308518
H	0.087327	-2.356348	0.201400
H	2.823553	-3.797084	0.456259
H	5.368258	1.606668	0.849774
H	3.669664	2.267535	1.152675
H	-2.778566	1.986154	0.381627
H	-2.222859	3.763472	0.448609
H	1.396819	-0.682745	2.244872
H	3.094992	-1.051965	1.986205
H	1.871732	-2.341555	1.957412
H	-3.781708	-1.943531	-1.382859
H	-4.531097	-2.446913	0.146437
H	-4.364758	0.428048	-0.899632
H	-6.809985	0.205075	-1.362900
H	-5.874718	-0.966644	-2.305346
H	-6.759082	-1.491317	-0.861283
H	-4.489859	0.323375	1.617887
H	-5.929007	-0.692042	1.497826
H	-5.979410	0.982801	0.924265

B3LYP energy = -1155.18150631 a.u.

(1*R*,5*S*,6*R*,7*R*,8*S*,10*R*)-**3**, Conf. L

C	4.264481	-1.936812	-0.417908
C	4.683024	-0.496472	-0.767746
C	3.803482	0.533922	-0.092799
C	2.328681	0.298337	-0.363139
C	1.887730	-1.132691	0.153839
C	2.766473	-2.164580	-0.622922
C	1.338600	1.311459	0.185022
C	-0.093008	1.035593	-0.306750
C	-0.585657	-0.289219	0.245751

C	0.393585	-1.395065	-0.193904
O	1.570990	2.688654	-0.216626
C	0.378026	3.367857	-0.170157
C	-0.735492	2.366664	-0.021932
O	2.504313	-3.517677	-0.239616
C	4.300474	1.527384	0.652526
C	-1.981701	2.717983	0.300129
O	0.311142	4.569142	-0.258681
H	2.206081	0.288460	-1.459180
C	2.097010	-1.296589	1.676117
O	-1.896371	-0.549331	-0.326774
C	-2.706370	-1.422363	0.331584
O	-2.392495	-1.963818	1.372449
C	-4.018772	-1.612863	-0.397411
C	-4.929575	-0.360203	-0.402759
C	-6.170338	-0.622009	-1.269282
C	-5.328879	0.059673	1.019940
H	4.518316	-2.163401	0.623484
H	4.810770	-2.655005	-1.038366
H	4.606598	-0.361530	-1.858001
H	5.732892	-0.333814	-0.504643
H	2.538199	-2.043037	-1.695616
H	1.366245	1.302613	1.281999
H	-0.038413	0.932678	-1.402939
H	-0.695268	-0.260824	1.332999
H	0.286509	-1.496088	-1.283651
H	0.073536	-2.336874	0.262486
H	1.647951	-3.795191	-0.588363
H	5.372561	1.620612	0.805499
H	3.676719	2.289302	1.106394
H	-2.776492	1.987700	0.399615
H	-2.219266	3.764316	0.468499
H	3.082052	-0.947858	1.993732
H	2.003336	-2.352559	1.943614
H	1.354202	-0.743496	2.257608
H	-3.793715	-1.894512	-1.432843
H	-4.533395	-2.450332	0.083076
H	-4.361776	0.458520	-0.863890
H	-5.895175	-0.880672	-2.297878
H	-6.767135	-1.447550	-0.862175
H	-6.812537	0.264543	-1.307053
H	-5.902988	-0.733519	1.513795
H	-4.458194	0.272128	1.649647
H	-5.952886	0.959581	0.996931

B3LYP energy = -1155.18148076 a.u.

(1*R*,5*S*,6*R*,7*R*,8*S*,10*R*)-**3**, Conf. M

C	3.672388	-2.717038	-0.230523
C	4.497064	-1.441110	-0.493497
C	3.857734	-0.218012	0.129441
C	2.408853	-0.046308	-0.288764
C	1.549489	-1.312324	0.128011
C	2.195079	-2.526085	-0.593746
C	1.671662	1.189193	0.200418
C	0.276234	1.306929	-0.435184
C	-0.599549	0.155191	0.024625

C	0.084048	-1.165950	-0.372381
O	2.297793	2.459421	-0.126198
C	1.325639	3.430200	-0.168098
C	-0.020246	2.757847	-0.167608
O	1.421386	-3.689081	-0.279432
C	4.523021	0.599722	0.953080
C	-1.159892	3.420774	0.037792
O	1.587356	4.607042	-0.215420
H	2.399036	-0.010353	-1.390730
C	1.549514	-1.539543	1.657920
O	-1.875520	0.259307	-0.663010
C	-2.966507	-0.275962	-0.049409
O	-2.924990	-0.783103	1.052521
C	-4.185003	-0.140103	-0.944007
C	-5.544161	-0.541313	-0.336011
C	-5.631339	-2.041623	-0.010969
C	-5.927090	0.328819	0.872155
H	3.747023	-3.006438	0.823355
H	4.082185	-3.549471	-0.818735
H	4.568124	-1.284635	-1.581029
H	5.520259	-1.567994	-0.126247
H	2.122478	-2.332471	-1.677880
H	1.583982	1.162787	1.293774
H	0.411886	1.202200	-1.524170
H	-0.795710	0.202019	1.098781
H	0.068496	-1.219583	-1.469449
H	-0.503863	-2.008758	-0.001536
H	1.822995	-4.456858	-0.706038
H	5.560247	0.402374	1.211442
H	4.080141	1.497368	1.369665
H	-2.121939	2.920822	0.028051
H	-1.136592	4.491376	0.218818
H	0.940872	-0.798290	2.182560
H	2.555339	-1.489208	2.080958
H	1.128327	-2.523439	1.878309
H	-4.215079	0.899306	-1.294202
H	-3.979701	-0.740999	-1.840448
H	-6.276377	-0.336911	-1.130487
H	-4.938950	-2.312825	0.790349
H	-6.645528	-2.303523	0.310471
H	-5.394563	-2.652855	-0.890219
H	-6.946213	0.098542	1.201475
H	-5.894352	1.396004	0.621087
H	-5.252343	0.153299	1.714923

B3LYP energy = -1155.18076818 a.u.

(1*R*,5*S*,6*R*,7*R*,8*S*,10*R*)-**3**, Conf. N

C	3.039902	-3.115305	-0.288611
C	4.024963	-2.007552	-0.712206
C	3.675392	-0.672869	-0.088244
C	2.233630	-0.273920	-0.344201
C	1.235771	-1.361396	0.235448
C	1.580624	-2.689941	-0.490312
C	1.774469	1.083494	0.160401
C	0.351179	1.414666	-0.318947
C	-0.641475	0.444278	0.296225
C	-0.236355	-0.986767	-0.100443
O	2.556899	2.218194	-0.300459
C	1.759163	3.337461	-0.284791
C	0.330797	2.903249	-0.099083
O	0.671280	-3.694765	-0.028225
C	4.562983	0.049758	0.604425
C	-0.651779	3.758450	0.190568
O	2.202970	4.450847	-0.423327
H	2.094566	-0.273460	-1.437896
C	1.386347	-1.535115	1.765274
O	-1.953600	0.742552	-0.253771
C	-3.038865	0.429927	0.504764
O	-2.956258	-0.067345	1.610369
C	-4.331380	0.791679	-0.197558
C	-5.212412	-0.426980	-0.589034
C	-4.477139	-1.383407	-1.540421
C	-5.782926	-1.167841	0.629122
H	3.193633	-3.377468	0.763852
H	3.233138	-4.024762	-0.873884
H	3.989175	-1.903256	-1.807743
H	5.050143	-2.293538	-0.456907
H	1.409013	-2.522913	-1.567765
H	1.815924	1.107757	1.256533
H	0.336089	1.251284	-1.409122
H	-0.701973	0.560712	1.381362
H	-0.394841	-1.078120	-1.183380
H	-0.903308	-1.703766	0.383410
H	0.888266	-4.533223	-0.455604
H	5.577858	-0.311195	0.750403
H	4.328806	1.024080	1.018261
H	-1.678663	3.432249	0.311701
H	-0.429322	4.815018	0.307626
H	2.431441	-1.637620	2.066559
H	0.843686	-2.428487	2.083760
H	0.972435	-0.687210	2.317302
H	-4.896881	1.430366	0.490896
H	-4.096770	1.375472	-1.091782
H	-6.057834	0.005954	-1.141867
H	-4.077616	-0.855847	-2.413798
H	-3.641202	-1.884340	-1.037777
H	-5.155880	-2.163980	-1.900681
H	-4.988503	-1.613808	1.234371
H	-6.458817	-1.966893	0.304432
H	-6.350229	-0.490097	1.276778

B3LYP energy = -1155.18060887 a.u.

Table S32. Cartesian coordinates and energies of the low-energy conformers calculated at the B3LYP/6-311+G(2d,p) *in vacuo* level.

(1*R*,5*S*,6*S*,7*S*,10*R*)-**2**, Conf. A

C	3.464632	0.018681	-0.246373
C	2.934828	1.404791	-0.654303
C	1.583491	1.667843	-0.037682
C	0.570577	0.588656	-0.375343
C	1.056845	-0.787044	0.192978
C	2.431507	-1.078590	-0.480026
C	-0.890328	0.928837	-0.036882
C	-1.853504	-0.191849	-0.455864
C	-1.403509	-1.535667	0.133621
C	0.044633	-1.870188	-0.235233
C	-3.338203	0.160780	-0.172543
C	-4.293334	-0.783320	-0.915487
C	-3.698372	0.224332	1.318214
O	-1.302917	2.106721	-0.742051
C	1.365427	2.710897	0.763455
O	3.027136	-2.299280	-0.031344
H	0.591217	0.485260	-1.470766
C	1.199677	-0.775553	1.725235
H	3.747950	0.021415	0.809117
H	4.366550	-0.224970	-0.812590
H	3.648043	2.180382	-0.368265
H	2.840601	1.442549	-1.746909
H	2.246384	-1.151105	-1.562265
H	-0.985610	1.104089	1.042381
H	-1.760645	-0.261242	-1.548243
H	-2.055014	-2.333548	-0.230702
H	-1.516668	-1.527046	1.220864
H	0.106550	-2.001141	-1.323267
H	0.319026	-2.829319	0.217126
H	-3.487657	1.160814	-0.586636
H	-4.062156	-0.823523	-1.983480
H	-4.250994	-1.803698	-0.524353
H	-5.326614	-0.441777	-0.813784
H	-4.727154	0.572366	1.440965
H	-3.056674	0.915353	1.869507
H	-3.630145	-0.755451	1.799293
H	-0.618971	2.778519	-0.635174
H	0.417369	2.886734	1.258571
H	2.153316	3.425572	0.973306
H	2.489401	-3.039186	-0.332619
H	1.822180	0.048418	2.072370
H	0.232748	-0.676925	2.219718
H	1.657003	-1.707705	2.060397

B3LYP energy = -737.915663422 a.u.

(1*R*,5*S*,6*S*,7*S*,10*R*)-**2**, Conf. B

C	3.460101	0.025221	-0.236205
C	2.933115	1.410078	-0.655218
C	1.582482	1.668414	-0.035046
C	0.570961	0.589930	-0.378009
C	1.052220	-0.791632	0.182029
C	2.426557	-1.078298	-0.475150
C	-0.890130	0.927570	-0.036798
C	-1.854879	-0.190061	-0.460908
C	-1.401284	-1.541578	0.109114
C	0.045501	-1.871358	-0.266067
C	-3.337631	0.161448	-0.165154
C	-4.297842	-0.773463	-0.913148
C	-3.689421	0.206935	1.328382
O	-1.302011	2.110147	-0.736024
C	1.367569	2.705844	0.774137
O	2.897637	-2.341412	0.006797
H	0.591049	0.492267	-1.473632
C	1.178955	-0.790598	1.717213
H	3.734313	0.034325	0.821505
H	4.371919	-0.207953	-0.797544
H	3.645634	2.188082	-0.373767
H	2.837221	1.440760	-1.747740
H	2.248277	-1.156703	-1.558184
H	-0.984820	1.098499	1.043033
H	-1.768221	-0.247072	-1.554509
H	-2.052618	-2.334262	-0.266904
H	-1.515896	-1.549872	1.196397
H	0.110569	-1.978400	-1.355790
H	0.339515	-2.833893	0.157089
H	-3.489431	1.166474	-0.566327
H	-4.252929	-1.798124	-0.533910
H	-5.330445	-0.432711	-0.801433
H	-4.072732	-0.801749	-1.982819
H	-4.717341	0.553982	1.461507
H	-3.044129	0.890641	1.884760
H	-3.618366	-0.778785	1.796518
H	-0.620619	2.782967	-0.620867
H	0.420956	2.877620	1.273283
H	2.155764	3.419554	0.986388
H	3.740336	-2.538173	-0.416150
H	1.826864	0.008918	2.076057
H	0.209747	-0.655494	2.197884
H	1.592734	-1.741504	2.053940

B3LYP energy = -737.915317555 a.u.

(1*R*,5*S*,6*S*,7*S*,10*R*)-**2**, Conf. C

C	3.463270	0.026395	-0.263342
C	2.927924	1.414629	-0.660098
C	1.582054	1.666017	-0.028221
C	0.569975	0.590221	-0.376216
C	1.051300	-0.794157	0.175025
C	2.432273	-1.083311	-0.484467
C	-0.891597	0.928402	-0.035988
C	-1.857142	-0.188422	-0.460553
C	-1.402133	-1.541667	0.104993
C	0.043314	-1.868852	-0.277790
C	-3.339250	0.162485	-0.161143

C	-4.300936	-0.769182	-0.911249
C	-3.688733	0.202380	1.333108
O	-1.302052	2.111795	-0.733634
C	1.369653	2.695466	0.791845
O	2.966169	-2.356767	-0.114293
H	0.591522	0.496169	-1.472124
C	1.172741	-0.797470	1.710270
H	3.759175	0.043550	0.791665
H	4.361520	-0.213321	-0.837634
H	3.641753	2.191239	-0.377861
H	2.823585	1.454418	-1.751377
H	2.254499	-1.178141	-1.560850
H	-0.987254	1.098908	1.043961
H	-1.772499	-0.243172	-1.554324
H	-2.055002	-2.333084	-0.270845
H	-1.513289	-1.552950	1.192883
H	0.104623	-1.965964	-1.368339
H	0.339628	-2.836595	0.132717
H	-3.491206	1.169088	-0.558265
H	-4.255666	-1.795408	-0.536325
H	-5.333237	-0.428658	-0.796347
H	-4.077719	-0.793090	-1.981368
H	-4.715923	0.550307	1.469185
H	-3.041888	0.883065	1.891505
H	-3.618559	-0.785444	1.796999
H	-0.622326	2.785562	-0.613901
H	0.424426	2.862601	1.295256
H	2.157757	3.408567	1.006501
H	3.324961	-2.295635	0.778258
H	1.872447	-0.044424	2.073475
H	0.215369	-0.597570	2.191399
H	1.504084	-1.777850	2.062123

B3LYP energy = -737.915222647 a.u.

(1*R*,5*S*,6*S*,7*S*,10*R*)-**2**, Conf. D

C	3.415985	0.426499	-0.262068
C	2.671042	1.730552	-0.596696
C	1.307477	1.748836	0.048989
C	0.466492	0.545361	-0.338900
C	1.170231	-0.765489	0.150904
C	2.562847	-0.805280	-0.545311
C	-1.024489	0.638316	0.031395
C	-1.815537	-0.589552	-0.442527
C	-1.144594	-1.878205	0.059168
C	0.331848	-1.967260	-0.331013
C	-3.331623	-0.579351	-0.084503
C	-3.629624	-0.122387	1.350177
C	-4.187613	0.203963	-1.089296
O	-1.615382	1.787392	-0.589029
C	0.951950	2.697027	0.916215
O	3.346333	-1.939603	-0.164090
H	0.485441	0.504079	-1.438460
C	1.331451	-0.814570	1.680502
H	3.708146	0.422608	0.791016
H	4.337435	0.355045	-0.844444
H	3.260716	2.593265	-0.280359
H	2.551664	1.804205	-1.684966
H	2.375348	-0.851053	-1.628460
H	-1.124021	0.726634	1.120369
H	-1.745867	-0.589394	-1.538137
H	-1.677993	-2.737204	-0.357931
H	-1.251418	-1.958032	1.145322
H	0.400023	-2.028669	-1.424955
H	0.754513	-2.896227	0.066785
H	-3.648236	-1.627592	-0.156757
H	-3.038394	-0.666748	2.091437
H	-3.433132	0.945040	1.474188
H	-4.682878	-0.291282	1.587910
H	-3.950167	1.267081	-1.069002
H	-5.250212	0.080959	-0.860174
H	-4.025195	-0.156137	-2.108943
H	-1.031540	2.541665	-0.442958
H	0.001128	2.694631	1.436601
H	1.626730	3.510635	1.157304
H	2.931306	-2.737077	-0.509076
H	1.931882	-1.681244	1.960936
H	1.823904	0.075686	2.070302
H	0.368158	-0.893939	2.185178

B3LYP energy = -737.914007817 a.u.

(1*R*,5*S*,6*S*,7*S*,10*R*)-**2**, Conf. E

C	3.410399	0.430588	-0.253417
C	2.668433	1.734474	-0.599060
C	1.306780	1.748833	0.050693
C	0.466776	0.546198	-0.340879
C	1.166387	-0.770427	0.141397
C	2.557135	-0.807056	-0.540347
C	-1.023974	0.636573	0.031864
C	-1.816122	-0.588267	-0.448251
C	-1.142275	-1.881983	0.037611
C	0.332257	-1.967872	-0.357283
C	-3.330883	-0.579443	-0.084395
C	-3.623703	-0.131755	1.354249
C	-4.191092	0.210189	-1.080678
O	-1.614938	1.790366	-0.581405
C	0.955666	2.692550	0.924544
O	3.225899	-2.003967	-0.127688
H	0.484238	0.509575	-1.440331
C	1.315693	-0.830425	1.673090
H	3.694101	0.431970	0.801848
H	4.339267	0.370205	-0.831634
H	3.257531	2.599561	-0.287936
H	2.547813	1.800570	-1.687516
H	2.375675	-0.858603	-1.624324
H	-1.122635	0.719613	1.121166
H	-1.750773	-0.578272	-1.544085
H	-1.676665	-2.735794	-0.388983
H	-1.249304	-1.975496	1.122947
H	0.400505	-2.008492	-1.451442
H	0.774164	-2.894619	0.014206
H	-3.647550	-1.627210	-0.161843
H	-4.675968	-0.302743	1.595252

H	-3.029027	-0.680617	2.089393
H	-3.427524	0.935094	1.484442
H	-5.252884	0.086471	-0.847859
H	-4.033127	-0.143944	-2.103131
H	-3.953046	1.273148	-1.055245
H	-1.031792	2.543938	-0.429911
H	0.007079	2.686823	1.448828
H	1.631072	3.505418	1.166639
H	4.082552	-2.045327	-0.566011
H	0.345110	-0.869019	2.168108
H	1.873240	-1.724103	1.954745
H	1.841404	0.037605	2.070595

B3LYP energy = -737.913699202 a.u.

(1*R*,5*S*,6*S*,7*S*,10*R*)-**2**, Conf. F

C	3.413617	0.431181	-0.278348
C	2.664787	1.736820	-0.601386
C	1.307538	1.745936	0.056946
C	0.466046	0.546544	-0.340166
C	1.165363	-0.772461	0.133349
C	2.563350	-0.811874	-0.550027
C	-1.025311	0.637245	0.031841
C	-1.818695	-0.586690	-0.448888
C	-1.143634	-1.881717	0.032742
C	0.329070	-1.965084	-0.369329
C	-3.332715	-0.578316	-0.082158
C	-3.622862	-0.135536	1.358577
C	-4.194253	0.215234	-1.074148
O	-1.615080	1.792458	-0.578934
C	0.959411	2.683080	0.939161
O	3.291508	-2.004583	-0.248752
H	0.484699	0.514010	-1.439764
C	1.310061	-0.836900	1.665020
H	3.715695	0.441883	0.775128
H	4.330752	0.362689	-0.868465
H	3.255598	2.599975	-0.287912
H	2.536362	1.811686	-1.688221
H	2.386046	-0.879112	-1.628472
H	-1.124639	0.718879	1.121353
H	-1.755452	-0.575076	-1.544742
H	-1.679833	-2.734539	-0.393343
H	-1.246871	-1.977321	1.118518
H	0.392287	-1.997038	-1.463764
H	0.773997	-2.895590	-0.010030
H	-3.649964	-1.625642	-0.162742
H	-4.674762	-0.306923	1.600701
H	-3.027473	-0.687409	2.090992
H	-3.426192	0.930791	1.492332
H	-5.255646	0.091191	-0.839854
H	-4.038292	-0.135444	-2.098041
H	-3.955718	1.277985	-1.045504
H	-1.032427	2.545795	-0.424197
H	0.011779	2.674733	1.465234
H	1.635288	3.494729	1.184064
H	3.656412	-1.929304	0.640205
H	0.341406	-0.810926	2.164075
H	1.792491	-1.772006	1.961107
H	1.892385	-0.005788	2.063719

B3LYP energy = -737.913591030 a.u.

(1*R*,5*S*,6*S*,7*S*,10*R*)-**2**, Conf. G

C	3.472361	-0.006395	-0.180788
C	2.974249	1.381937	-0.609958
C	1.606683	1.679893	-0.040564
C	0.579579	0.612378	-0.382635
C	1.044965	-0.784476	0.163262
C	2.428638	-1.084005	-0.467084
C	-0.882883	0.938143	-0.018310
C	-1.855441	-0.176179	-0.461309
C	-1.401996	-1.546543	0.060827
C	0.046245	-1.858084	-0.315628
C	-3.333892	0.164390	-0.133879
C	-4.306152	-0.711801	-0.935378
C	-3.673459	0.108694	1.362484
O	-1.286739	2.198125	-0.569938
C	1.384316	2.757975	0.706371
O	2.861417	-2.368033	-0.003130
H	0.595113	0.511514	-1.481852
C	1.144733	-0.795330	1.700770
H	3.709797	-0.009002	0.885858
H	4.398978	-0.250499	-0.713267
H	3.687678	2.152103	-0.310096
H	2.916924	1.412822	-1.706389
H	2.275965	-1.136464	-1.556155
H	-0.962906	1.085420	1.061196
H	-1.784552	-0.209088	-1.559779
H	-2.051234	-2.328020	-0.342527
H	-1.520132	-1.590070	1.146413
H	0.119456	-1.938746	-1.407470
H	0.343726	-2.828832	0.085210
H	-3.481036	1.196611	-0.461500
H	-4.099159	-0.659203	-2.007991
H	-4.253435	-1.762361	-0.636474
H	-5.337224	-0.383286	-0.781179
H	-4.691193	0.470219	1.529955
H	-3.007056	0.733068	1.962035
H	-3.623411	-0.910530	1.754487
H	-1.040236	2.220475	-1.502087
H	0.407851	3.003153	1.099730
H	2.188684	3.452353	0.925004
H	3.735158	-2.546833	-0.366918
H	1.767860	0.016698	2.075026
H	0.165821	-0.685814	2.167878
H	1.573458	-1.740011	2.036352

B3LYP energy = -737.912271707 a.u.

Table S33. Cartesian coordinates and energies of the low-energy conformers calculated at the M06-2X/6-31G(d) *in vacuo* level.

(2*S*,6*R*,7*S*,8*R*)-**1**, Conf. A

C	-4.306756	-0.572748	-0.651391
C	-3.273758	0.444273	-1.050281
C	-2.851567	1.507724	-0.357286
C	-3.525451	2.073441	0.866223
C	-2.402178	-1.698429	0.532692
C	-3.595075	-1.919148	-0.361999
C	-0.363448	1.214735	-0.672571
C	-0.146554	0.565559	0.723706
C	0.013730	-0.971558	0.650576
C	-1.184401	-1.598676	-0.006622
O	0.837605	1.937727	-0.987150
C	1.672140	2.034809	0.074492
C	1.102447	1.246613	1.206203
O	2.699549	2.654855	0.041699
C	1.717233	1.178366	2.382455
C	-1.548563	2.187841	-0.744268
C	-2.703388	-1.378293	1.971942
O	-5.230236	-0.849385	-1.691087
O	1.157745	-1.246459	-0.167065
C	2.278286	-1.719947	0.436467
O	2.359734	-1.936354	1.618598
C	3.377733	-1.886539	-0.579757
C	3.953956	-0.523293	-1.010734
C	4.654489	0.165648	0.159411
C	4.914878	-0.714848	-2.182515
H	-4.839138	-0.251462	0.255108
H	-2.716166	0.155431	-1.944894
H	-4.457993	1.567341	1.122596
H	-3.751316	3.133683	0.699254
H	-3.280159	-2.321794	-1.330880
H	-4.327146	-2.611544	0.068874
H	-2.861439	2.035784	1.739489
H	-1.001344	0.772221	1.377464
H	0.203001	-1.359901	1.656128
H	-1.052926	-1.826355	-1.063513
H	1.338104	0.576620	3.203044
H	2.643088	1.727400	2.532718
H	-1.340858	3.026331	-0.067670
H	-0.467941	0.446606	-1.442613
H	-1.590969	2.601116	-1.758104
H	-3.388890	-2.121683	2.393480
H	-3.201824	-0.403175	2.057405
H	-1.806018	-1.352350	2.595484
H	-5.647705	-0.013640	-1.942534
H	4.161682	-2.501882	-0.128151
H	2.969250	-2.409051	-1.451292
H	3.117411	0.104489	-1.346018
H	5.008170	1.157770	-0.132813
H	3.984353	0.292372	1.015206
H	5.513791	-0.431831	0.487684
H	5.325616	0.246609	-2.505144
H	4.415767	-1.177507	-3.040125
H	5.753913	-1.357861	-1.890611

M062X energy = -1154.59150837 a.u.

(2*S*,6*R*,7*S*,8*R*)-**1**, Conf. B

C	-4.469044	-0.215458	0.025184
C	-3.458084	0.854779	0.389648
C	-2.671674	1.541320	-0.446699
C	-2.815082	1.527173	-1.946581
C	-2.471835	-1.745432	0.150268
C	-3.770611	-1.475269	-0.561951
C	-0.200332	1.434977	-0.056054
C	-0.070322	0.267875	0.965655
C	-0.035636	-1.126254	0.299153
C	-1.324887	-1.372675	-0.424473
O	0.938002	2.292077	0.137932
C	1.771906	1.848276	1.106651
C	1.208370	0.595442	1.685619
O	2.791022	2.411766	1.397672
C	1.841788	-0.067497	2.647695
C	-1.470332	2.289600	0.083903
C	-2.585041	-2.202277	1.580307
O	-5.426983	0.174405	-0.946188
O	1.038608	-1.119694	-0.647724
C	2.161666	-1.823114	-0.355239
O	2.276325	-2.528770	0.614195
C	3.222482	-1.536799	-1.385869
C	3.801422	-0.119794	-1.194263
C	4.593554	-0.024851	0.108926
C	4.675918	0.252776	-2.390002
H	-4.986584	-0.511160	0.950870
H	-3.257068	0.949253	1.458107
H	-2.915235	2.554304	-2.318247
H	-1.918276	1.113192	-2.425476
H	-3.583548	-1.269101	-1.620687
H	-4.470901	-2.316337	-0.500924
H	-3.686251	0.957212	-2.269244
H	-0.926730	0.275761	1.651824
H	0.174059	-1.883197	1.059920
H	-1.341337	-1.023920	-1.456848
H	1.467631	-1.001283	3.056470
H	2.780199	0.322397	3.032904
H	-1.597817	2.556494	1.139705
H	-0.116878	1.052154	-1.078357
H	-1.301956	3.216247	-0.477118
H	-2.951084	-1.388678	2.222866
H	-1.634253	-2.548768	1.991951
H	-3.309093	-3.019864	1.664858
H	-5.817092	1.009986	-0.651865
H	4.011024	-2.287616	-1.280545
H	2.774220	-1.623633	-2.381305
H	2.956091	0.581374	-1.149955

H	4.910992	1.004632	0.295224
H	4.000543	-0.352102	0.968328
H	5.484980	-0.661666	0.055194
H	5.099396	1.253334	-2.261381
H	4.105669	0.240143	-3.324426
H	5.508179	-0.454222	-2.490214

M062X energy = -1154.59081141 a.u.

(2*S*,6*R*,7*S*,8*R*)-**1**, Conf. C

C	-4.419495	-0.792460	-0.478795
C	-3.509182	0.322760	-0.912763
C	-3.111958	1.388331	-0.207941
C	-3.719230	1.846395	1.092825
C	-2.339794	-1.804008	0.488729
C	-3.580033	-2.085903	-0.320056
C	-0.654449	1.293490	-0.741760
C	-0.256526	0.633184	0.608155
C	0.007215	-0.888039	0.469618
C	-1.176727	-1.589640	-0.132370
O	0.462952	2.091285	-1.164205
C	1.427247	2.168741	-0.219366
C	1.000275	1.374099	0.970065
O	2.446723	2.783867	-0.374774
C	1.731033	1.348636	2.079438
C	-1.901467	2.183711	-0.670702
C	-2.553749	-1.557114	1.958160
O	-5.407947	-1.099790	-1.447478
O	1.121524	-1.020911	-0.422410
C	2.322834	-1.350332	0.113304
O	2.468881	-1.713367	1.252786
C	3.414987	-1.201960	-0.918077
C	4.812997	-1.091541	-0.308476
C	5.855518	-1.061721	-1.425511
C	4.926062	0.151020	0.576691
H	-4.891343	-0.557008	0.485934
H	-3.014167	0.118548	-1.865693
H	-2.983513	1.822652	1.907178
H	-4.586798	1.258029	1.397754
H	-3.316362	-2.416088	-1.330736
H	-4.215555	-2.856086	0.131704
H	-4.037071	2.891924	0.998367
H	-1.046634	0.769335	1.354645
H	0.290537	-1.294417	1.445037
H	-1.105701	-1.769572	-1.204184
H	1.459059	0.749455	2.943291
H	2.644981	1.934712	2.125884
H	-1.696433	3.008464	0.023248
H	-0.792635	0.533525	-1.514206
H	-2.060498	2.629731	-1.658621
H	-3.158366	-2.358966	2.396170
H	-3.103736	-0.620069	2.118949
H	-1.616465	-1.495820	2.517127
H	-5.914152	-0.292692	-1.615959
H	3.347076	-2.068410	-1.589946
H	3.187020	-0.318846	-1.527541
H	4.979473	-1.979369	0.314163

H	6.867252	-1.012461	-1.011390
H	5.792310	-1.950848	-2.062055
H	5.712049	-0.179877	-2.061264
H	5.956000	0.286060	0.922496
H	4.283222	0.061484	1.456334
H	4.630031	1.052046	0.024962

M062X energy = -1154.59051979 a.u.

(2*S*,6*R*,7*S*,8*R*)-**1**, Conf. D

C	-4.284837	-0.510830	-0.715671
C	-3.276331	0.576335	-0.959205
C	-2.857783	1.517396	-0.105890
C	-3.519819	1.862591	1.203034
C	-2.359559	-1.762567	0.295795
C	-3.542297	-1.868160	-0.633295
C	-0.362520	1.339991	-0.479365
C	-0.091994	0.504909	0.805272
C	0.037220	-1.019404	0.553796
C	-1.141940	-1.547560	-0.209455
O	0.803478	2.147738	-0.710807
C	1.707844	2.052219	0.289918
C	1.193568	1.097086	1.314290
O	2.747590	2.653188	0.288924
C	1.880649	0.839826	2.422576
C	-1.575052	2.276974	-0.397188
C	-2.680234	-1.693192	1.764792
O	-5.208933	-0.646856	-1.782251
O	1.218566	-1.238980	-0.229749
C	2.292030	-1.779310	0.400981
O	2.271269	-2.144470	1.549953
C	3.502590	-1.818538	-0.494360
C	4.070396	-0.405713	-0.772897
C	5.567925	-0.506563	-1.062253
C	3.351771	0.279663	-1.934816
H	-4.817074	-0.341231	0.230863
H	-2.731784	0.448666	-1.898508
H	-3.753548	2.934053	1.220536
H	-2.846455	1.682472	2.050820
H	-3.215454	-2.110483	-1.650325
H	-4.259729	-2.634723	-0.318629
H	-4.446853	1.314156	1.380850
H	-0.912721	0.637005	1.520023
H	0.169973	-1.518244	1.517259
H	-1.000849	-1.596221	-1.288556
H	1.544969	0.119281	3.161848
H	2.824680	1.350581	2.593222
H	-1.378774	3.014475	0.391188
H	-0.457447	0.686787	-1.351420
H	-1.638123	2.830644	-1.340564
H	-3.237963	-0.777260	2.001042
H	-1.788594	-1.714342	2.395965
H	-3.319667	-2.534682	2.053874
H	-5.652698	0.205329	-1.896419
H	4.242011	-2.433480	0.024477
H	3.245909	-2.304305	-1.443288
H	3.934634	0.206115	0.129664

H	5.985227	0.480260	-1.284505
H	6.114134	-0.925753	-0.211356
H	5.746585	-1.151648	-1.931287
H	3.693547	1.313463	-2.039821
H	2.269189	0.296634	-1.790984
H	3.561365	-0.253425	-2.870875

M062X energy = -1154.58992336 a.u.

(2*S*,6*R*,7*S*,8*R*)-**1**, Conf. E

C	-4.405969	-0.285359	0.123806
C	-3.412351	0.821843	0.415702
C	-2.650050	1.478073	-0.465894
C	-2.807272	1.381539	-1.961176
C	-2.367596	-1.756476	0.300701
C	-3.688628	-1.564712	-0.395877
C	-0.163233	1.482730	-0.106230
C	0.027304	0.352959	0.948816
C	0.057337	-1.071506	0.351966
C	-1.246591	-1.378948	-0.319739
O	0.928614	2.403558	0.065538
C	1.810465	2.004733	1.012560
C	1.321981	0.732076	1.614038
O	2.813052	2.612777	1.267273
C	2.023629	0.096194	2.546729
C	-1.467512	2.287434	0.011802
C	-2.438768	-2.142507	1.753847
O	-5.378866	0.031076	-0.859626
O	1.101656	-1.131134	-0.630702
C	2.215773	-1.844404	-0.332432
O	2.387422	-2.403866	0.721714
C	3.200564	-1.832051	-1.476140
C	4.040333	-0.536661	-1.546378
C	3.229591	0.661180	-2.038321
C	4.715246	-0.234664	-0.208652
H	-4.910818	-0.535679	1.069668
H	-3.199997	0.977519	1.474817
H	-3.666439	0.773996	-2.244864
H	-1.904320	0.966061	-2.426891
H	-3.531257	-1.424378	-1.470186
H	-4.365068	-2.417079	-0.263926
H	-2.936962	2.385041	-2.384716
H	-0.803975	0.376714	1.666255
H	0.295456	-1.780462	1.149462
H	-1.297571	-1.085576	-1.368158
H	1.709490	-0.853698	2.968100
H	2.961473	0.525061	2.889889
H	-1.589989	2.595428	1.056912
H	-0.060248	1.072881	-1.117573
H	-1.341523	3.194992	-0.590212
H	-3.101820	-3.004289	1.887561
H	-2.861061	-1.323657	2.353643
H	-1.463728	-2.395500	2.176564
H	-5.776360	0.878475	-0.613103
H	3.861868	-2.687463	-1.316470
H	2.657022	-1.971466	-2.416225
H	4.823307	-0.739638	-2.288668
H	3.870109	1.543593	-2.131171
H	2.773938	0.461604	-3.014221
H	2.426871	0.911681	-1.339651
H	5.416342	0.598687	-0.311750
H	5.260441	-1.104836	0.171497
H	3.974294	0.053851	0.545056

M062X energy = -1154.58979928 a.u.

(2*S*,6*R*,7*S*,8*R*)-**1**, Conf. F

C	-4.260081	-0.561609	-0.570604
C	-3.253203	0.491598	-0.939813
C	-2.819100	1.512912	-0.192905
C	-3.455927	1.986578	1.088342
C	-2.305013	-1.727313	0.480820
C	-3.523334	-1.912334	-0.387441
C	-0.331023	1.296832	-0.612765
C	-0.046916	0.554789	0.724614
C	0.104615	-0.977331	0.569708
C	-1.107775	-1.571182	-0.089896
O	0.833330	2.086344	-0.908203
C	1.719239	2.104946	0.116472
C	1.218283	1.210611	1.199655
O	2.738351	2.738160	0.085232
C	1.897831	1.045213	2.329815
C	-1.542515	2.239562	-0.579592
C	-2.564681	-1.500644	1.946000
O	-5.215661	-0.786174	-1.593753
O	1.234164	-1.247450	-0.273474
C	2.340188	-1.781561	0.304802
O	2.447982	-1.959469	1.492243
C	3.400042	-2.113487	-0.717816
C	4.218707	-0.885977	-1.178551
C	3.437258	0.002191	-2.145383
C	4.746590	-0.092666	0.016104
H	-4.764148	-0.303403	0.371515
H	-2.727017	0.270144	-1.871928
H	-4.370277	1.448580	1.345654
H	-3.704185	3.051297	0.999102
H	-3.235757	-2.251875	-1.388384
H	-4.231834	-2.639607	0.024871
H	-2.759463	1.905144	1.932866
H	-0.872623	0.716367	1.427377
H	0.303265	-1.405807	1.556539
H	-1.007761	-1.727252	-1.163274
H	1.573713	0.362580	3.109317
H	2.825450	1.591848	2.478161
H	-1.326489	3.037583	0.141786
H	-0.433731	0.584306	-1.435851
H	-1.628768	2.716192	-1.562338
H	-3.233173	-2.273462	2.340974
H	-3.064829	-0.535873	2.106569
H	-1.649252	-1.505931	2.543048
H	-5.651836	0.057481	-1.778260
H	4.064086	-2.835363	-0.235693
H	2.924578	-2.588569	-1.582119
H	5.078706	-1.299027	-1.721901

H	4.038948	0.865707	-2.444785
H	3.156509	-0.551935	-3.047675
H	2.519170	0.382296	-1.690126
H	5.438010	0.687090	-0.316089
H	5.266070	-0.741817	0.728924
H	3.928212	0.405249	0.545960

M062X energy = -1154.58978425 a.u.

(2*S*,6*R*,7*S*,8*R*)-**1**, Conf. G

C	-4.313614	-0.567348	-0.642107
C	-3.277076	0.446158	-1.046561
C	-2.853063	1.512836	-0.361600
C	-3.533570	2.087208	0.853248
C	-2.397958	-1.705527	0.523095
C	-3.594129	-1.920964	-0.368236
C	-0.365229	1.210676	-0.678273
C	-0.149989	0.561940	0.718162
C	0.015809	-0.974667	0.645020
C	-1.178995	-1.606486	-0.013970
O	0.838651	1.928283	-0.994043
C	1.670383	2.028822	0.069586
C	1.096417	1.246107	1.203013
O	2.698900	2.646941	0.036878
C	1.705783	1.184473	2.382445
C	-1.547048	2.187432	-0.748605
C	-2.701167	-1.378321	1.960459
O	-5.345482	-0.742195	-1.594812
O	1.162322	-1.246357	-0.170104
C	2.282349	-1.717520	0.437020
O	2.360118	-1.933763	1.619313
C	3.385546	-1.882212	-0.575300
C	3.963799	-0.518546	-1.002117
C	4.656305	0.170555	0.172696
C	4.932737	-0.709910	-2.167374
H	-4.826570	-0.250375	0.269719
H	-2.703320	0.152941	-1.932182
H	-2.875446	2.056100	1.731420
H	-4.470127	1.585497	1.102815
H	-3.269819	-2.325362	-1.336440
H	-4.324448	-2.617851	0.057875
H	-3.758743	3.145585	0.675174
H	-1.007069	0.766419	1.369625
H	0.204175	-1.362449	1.650976
H	-1.043095	-1.837416	-1.069779
H	1.322746	0.587356	3.204614
H	2.630488	1.734989	2.534339
H	-1.336197	3.023976	-0.070663
H	-0.472075	0.442203	-1.447763
H	-1.588821	2.603567	-1.761337
H	-3.397572	-2.111603	2.381566
H	-3.186469	-0.396197	2.041471
H	-1.805559	-1.362322	2.586687
H	-4.930028	-0.954265	-2.443732
H	4.167742	-2.498104	-0.121409
H	2.980750	-2.403610	-1.449239
H	3.129396	0.109124	-1.342877

H	5.015337	1.160992	-0.118723
H	3.978982	0.301779	1.022106
H	5.510930	-0.428793	0.509600
H	5.344204	0.251832	-2.488147
H	4.440075	-1.174111	-3.027901
H	5.770722	-1.351410	-1.869239

M062X energy = -1154.58971854 a.u.

(2*S*,6*R*,7*S*,8*R*)-**1**, Conf. H

C	-4.388159	-0.888824	-0.370292
C	-3.543205	0.280447	-0.793824
C	-3.151095	1.327524	-0.059029
C	-3.709768	1.702345	1.289113
C	-2.223350	-1.861072	0.441016
C	-3.493326	-2.153600	-0.316876
C	-0.719648	1.357753	-0.716727
C	-0.226887	0.647811	0.575456
C	0.084126	-0.853243	0.345283
C	-1.104785	-1.569417	-0.228505
O	0.341209	2.224395	-1.149968
C	1.348474	2.295394	-0.251244
C	1.018551	1.420467	0.911946
O	2.331842	2.963172	-0.424086
C	1.809631	1.364051	1.978089
C	-1.995679	2.190372	-0.541089
C	-2.368522	-1.692765	1.929613
O	-5.412642	-1.189314	-1.303247
O	1.151296	-0.900406	-0.610638
C	2.395685	-1.189061	-0.152691
O	2.613386	-1.576747	0.967807
C	3.413327	-0.951331	-1.244540
C	4.850540	-0.746553	-0.749562
C	4.936086	0.400382	0.260794
C	5.466624	-2.030755	-0.191775
H	-4.819182	-0.717409	0.626257
H	-3.088520	0.138455	-1.777514
H	-4.066998	2.738923	1.259592
H	-2.934857	1.668116	2.065878
H	-3.270544	-2.424362	-1.354615
H	-4.074353	-2.969443	0.128034
H	-4.541054	1.069712	1.606192
H	-0.981547	0.713794	1.366793
H	0.435003	-1.295370	1.282265
H	-1.084793	-1.696113	-1.309995
H	1.607009	0.707460	2.818999
H	2.703614	1.981028	2.010216
H	-1.787431	2.989852	0.180998
H	-0.862996	0.631571	-1.520371
H	-2.221381	2.675423	-1.497270
H	-2.918410	-2.538023	2.357987
H	-2.944320	-0.787188	2.164367
H	-1.406080	-1.620066	2.442668
H	-5.953237	-0.394219	-1.411227
H	3.356726	-1.807047	-1.931035
H	3.075654	-0.077166	-1.812076
H	5.424567	-0.455650	-1.639143

H	5.981094	0.665642	0.449787
H	4.414178	1.295473	-0.098393
H	4.487863	0.099107	1.212788
H	6.513516	-1.862929	0.081351
H	5.437695	-2.838348	-0.931696
H	4.926327	-2.359570	0.699278

M062X energy = -1154.58965996 a.u.

(2*S*,6*R*,7*S*,8*R*)-**1**, Conf. I

C	-4.505450	-0.560702	0.101131
C	-3.625271	0.669961	0.199992
C	-2.917253	1.239195	-0.781617
C	-3.048622	0.881148	-2.239020
C	-2.347072	-1.788141	0.525948
C	-3.663053	-1.832246	-0.204524
C	-0.448444	1.517085	-0.420043
C	-0.184931	0.624123	0.826903
C	-0.001392	-0.872644	0.492764
C	-1.248986	-1.413884	-0.136570
O	0.567043	2.534110	-0.430364
C	1.458951	2.397179	0.577024
C	1.051535	1.238691	1.422833
O	2.408381	3.118519	0.711055
C	1.766568	0.872167	2.481376
C	-1.815859	2.219294	-0.449070
C	-2.418126	-1.941707	2.021744
O	-5.494357	-0.507849	-0.914852
O	1.079904	-0.981000	-0.444608
C	2.240562	-1.526788	-0.014114
O	2.418654	-1.927855	1.108949
C	3.273890	-1.569855	-1.114142
C	4.625750	-1.027105	-0.632660
C	5.687645	-1.233926	-1.710859
C	4.508457	0.448749	-0.253107
H	-4.992122	-0.699744	1.078746
H	-3.440213	1.018090	1.217706
H	-3.839625	0.151606	-2.412410
H	-2.103658	0.487569	-2.635019
H	-3.493515	-1.847128	-1.285954
H	-4.262193	-2.712603	0.054993
H	-3.274490	1.782451	-2.821999
H	-1.034234	0.693143	1.518895
H	0.276648	-1.408260	1.404038
H	-1.300932	-1.298393	-1.219111
H	1.510419	0.010842	3.090556
H	2.655006	1.440738	2.743356
H	-1.983036	2.693640	0.525110
H	-0.303888	0.936305	-1.337231
H	-1.754291	3.014390	-1.201291
H	-1.435591	-2.051999	2.486069
H	-2.909695	-1.072271	2.480904
H	-3.018564	-2.818540	2.287272
H	-5.980851	0.321680	-0.805033
H	3.376818	-2.619798	-1.416028
H	2.913210	-1.005761	-1.979891
H	4.903753	-1.599441	0.260599

H	6.661104	-0.866509	-1.371958
H	5.795692	-2.291694	-1.972671
H	5.422887	-0.684590	-2.622108
H	5.469069	0.842062	0.093332
H	3.779209	0.603560	0.548270
H	4.192711	1.049688	-1.114512

M062X energy = -1154.58957113 a.u.

(2*S*,6*R*,7*S*,8*R*)-**1**, Conf. J

C	-4.314485	-0.553786	-0.657854
C	-3.281159	0.456313	-1.058624
C	-2.849980	1.510154	-0.358925
C	-3.519298	2.077261	0.866053
C	-2.402958	-1.689204	0.536783
C	-3.593000	-1.903511	-0.362081
C	-0.362327	1.213641	-0.675985
C	-0.144217	0.570129	0.722720
C	0.014414	-0.967155	0.655248
C	-1.184284	-1.593742	-0.001149
O	0.840815	1.931990	-0.995294
C	1.676796	2.031749	0.064210
C	1.106429	1.250940	1.200931
O	2.706671	2.647712	0.027576
C	1.721477	1.188398	2.377333
C	-1.546183	2.187138	-0.749598
C	-2.707853	-1.364772	1.974083
O	-5.225179	-0.716698	-1.730636
O	1.157646	-1.247334	-0.161428
C	2.277264	-1.721268	0.443613
O	2.357474	-1.935704	1.626186
C	3.376579	-1.892380	-0.571972
C	3.954779	-0.531607	-1.008165
C	4.655502	0.161181	0.159574
C	4.916224	-0.729459	-2.178489
H	-4.844206	-0.226450	0.247572
H	-2.733304	0.172130	-1.960009
H	-4.455206	1.576452	1.120044
H	-3.742500	3.137612	0.697088
H	-3.273669	-2.305424	-1.330092
H	-4.317996	-2.604567	0.074872
H	-2.855662	2.038477	1.739779
H	-0.997999	0.780810	1.376407
H	0.202422	-1.353255	1.661950
H	-1.052267	-1.823548	-1.057463
H	1.341494	0.592400	3.201739
H	2.648285	1.736781	2.524060
H	-1.336617	3.027705	-0.076143
H	-0.469187	0.442593	-1.442780
H	-1.590433	2.596732	-1.764721
H	-3.393053	-2.107473	2.397871
H	-3.206428	-0.389486	2.054997
H	-1.811445	-1.336606	2.598772
H	-5.829166	-1.437422	-1.500430
H	4.159719	-2.507191	-0.118238
H	2.967351	-2.417456	-1.441651
H	3.119382	0.095966	-1.346589

H	5.013783	1.150093	-0.137840
H	3.983703	0.296301	1.012764
H	5.511597	-0.437890	0.493313
H	5.327697	0.230221	-2.505365
H	4.417270	-1.195706	-3.034248
H	5.754693	-1.371641	-1.883119

M062X energy = -1154.58951636 a.u.

(2*S*,6*R*,7*S*,8*R*)-**1**, Conf. K

C	-4.249720	-1.247923	-0.511647
C	-3.506391	-0.022074	-0.963861
C	-3.294273	1.113604	-0.289381
C	-3.999351	1.518698	0.979145
C	-2.068390	-1.894600	0.543639
C	-3.228312	-2.390825	-0.281566
C	-0.831622	1.391832	-0.763355
C	-0.376495	0.833937	0.615498
C	0.113148	-0.633460	0.540631
C	-0.936238	-1.523975	-0.060700
O	0.148898	2.358947	-1.169395
C	1.058127	2.612118	-0.201017
C	0.743433	1.765117	0.987394
O	1.958664	3.393587	-0.338265
C	1.457594	1.850209	2.104615
C	-2.208051	2.069571	-0.754917
C	-2.357470	-1.634878	1.997889
O	-5.148544	-1.734308	-1.494472
O	1.263772	-0.642021	-0.316067
C	2.480025	-0.786463	0.263703
O	2.639688	-1.051330	1.428594
C	3.575471	-0.523663	-0.740096
C	4.958477	-0.954337	-0.253910
C	5.044856	-2.474830	-0.121640
C	6.032682	-0.427881	-1.204624
H	-4.783263	-1.054610	0.429834
H	-2.957922	-0.179957	-1.896163
H	-4.471728	2.498060	0.835648
H	-3.289646	1.640131	1.807665
H	-2.888291	-2.713182	-1.271648
H	-3.751385	-3.232722	0.186209
H	-4.773193	0.814993	1.291571
H	-1.202214	0.867391	1.334890
H	0.421098	-0.957246	1.538948
H	-0.809683	-1.726363	-1.123309
H	1.268196	1.216078	2.965398
H	2.273101	2.565868	2.162432
H	-2.154393	2.941160	-0.090509
H	-0.811662	0.604776	-1.521798
H	-2.406705	2.450504	-1.762881
H	-1.457193	-1.409130	2.575028
H	-2.842512	-2.505558	2.452675
H	-3.050185	-0.789901	2.111365
H	-5.768053	-1.021434	-1.704535
H	3.312201	-1.015078	-1.684815
H	3.548524	0.557322	-0.940075
H	5.109491	-0.512168	0.738179

H	6.032646	-2.777337	0.239927
H	4.296648	-2.854668	0.579083
H	4.885608	-2.952917	-1.096170
H	7.031402	-0.713206	-0.860002
H	5.998871	0.663431	-1.285091
H	5.895419	-0.844294	-2.209829

M062X energy = -1154.58951310 a.u.

(2*S*,6*R*,7*S*,8*R*)-**1**, Conf. L

C	-4.329526	-0.804195	-0.455451
C	-3.437053	0.333446	-0.866827
C	-3.037729	1.374665	-0.128173
C	-3.628871	1.777293	1.198420
C	-2.227472	-1.815246	0.463576
C	-3.473269	-2.090576	-0.339144
C	-0.565307	1.375975	-0.693032
C	-0.145642	0.646392	0.614503
C	0.113398	-0.866216	0.431272
C	-1.082169	-1.549943	-0.169882
O	0.505565	2.269403	-1.037242
C	1.434853	2.362580	-0.057098
C	1.093457	1.390708	1.023093
O	2.370849	3.111201	-0.113693
C	1.873434	1.241964	2.089119
C	-1.850869	2.208767	-0.579147
C	-2.422470	-1.631514	1.944487
O	-5.323583	-1.097270	-1.422870
O	1.212667	-1.032230	-0.475654
C	2.358157	-1.569415	0.010732
O	2.535636	-1.811015	1.179605
C	3.358513	-1.816929	-1.093334
C	4.596561	-0.902966	-0.987939
C	4.183931	0.566941	-0.943174
C	5.476499	-1.262607	0.208785
H	-4.794855	-0.601034	0.519532
H	-2.955151	0.168486	-1.834027
H	-4.488460	1.172707	1.493638
H	-3.953757	2.823749	1.149031
H	-3.216647	-2.390499	-1.360925
H	-4.093942	-2.880592	0.098683
H	-2.881093	1.727050	2.000418
H	-0.936735	0.739573	1.367844
H	0.399336	-1.288175	1.399235
H	-1.031865	-1.682245	-1.249920
H	1.673605	0.506362	2.862235
H	2.759764	1.863862	2.186614
H	-1.661744	3.017772	0.137586
H	-0.650657	0.665944	-1.520067
H	-2.033772	2.683034	-1.549705
H	-2.998969	-2.464844	2.361136
H	-2.993572	-0.715813	2.149740
H	-1.477984	-1.566362	2.490859
H	-5.841627	-0.292443	-1.563950
H	3.666416	-2.865683	-1.017055
H	2.863236	-1.667072	-2.055629
H	5.173374	-1.079081	-1.905150

H	5.053830	1.225376	-1.023961
H	3.488348	0.824072	-1.749706
H	3.691708	0.796302	0.008251
H	6.366677	-0.625827	0.230134
H	5.804658	-2.306375	0.163073
H	4.930256	-1.123724	1.146210

M062X energy = -1154.58919227 a.u.

(2*S*,6*R*,7*S*,8*R*)-**1**, Conf. M

C	-4.427470	-0.772210	-0.471897
C	-3.507256	0.336041	-0.908066
C	-3.104921	1.402400	-0.209235
C	-3.718557	1.869600	1.084350
C	-2.343774	-1.806603	0.476269
C	-3.588225	-2.076349	-0.329929
C	-0.648145	1.287030	-0.743213
C	-0.256574	0.622856	0.606368
C	0.005790	-0.898403	0.463430
C	-1.178012	-1.597670	-0.141863
O	0.477491	2.073103	-1.165849
C	1.439832	2.144665	-0.218568
C	1.001254	1.359547	0.972963
O	2.466334	2.748176	-0.373985
C	1.723802	1.337931	2.087765
C	-1.887089	2.187647	-0.670616
C	-2.559803	-1.553998	1.944575
O	-5.522602	-0.983259	-1.342901
O	1.120652	-1.028805	-0.428080
C	2.322143	-1.358792	0.108382
O	2.465458	-1.731588	1.244876
C	3.416672	-1.196940	-0.918365
C	4.812048	-1.078927	-0.303774
C	5.856250	-1.027222	-1.418609
C	4.910950	0.155804	0.594066
H	-4.880703	-0.539514	0.495460
H	-2.994107	0.127131	-1.852916
H	-4.590810	1.286063	1.384462
H	-4.035843	2.914077	0.979012
H	-3.315816	-2.408269	-1.340744
H	-4.226674	-2.847468	0.115648
H	-2.987462	1.851324	1.903238
H	-1.048842	0.759497	1.350406
H	0.287751	-1.308405	1.437790
H	-1.102890	-1.779195	-1.213295
H	1.442492	0.746108	2.953714
H	2.640052	1.920122	2.137302
H	-1.675228	3.008693	0.025568
H	-0.792439	0.528240	-1.515793
H	-2.042869	2.638450	-1.656899
H	-3.090890	-0.605460	2.101545
H	-1.623504	-1.510472	2.506670
H	-3.181989	-2.342841	2.381388
H	-5.168555	-1.117906	-2.234520
H	3.357908	-2.060436	-1.594903
H	3.184374	-0.312269	-1.523870
H	4.986267	-1.971487	0.309885

H	6.866769	-0.973540	-1.002134
H	5.802092	-1.909600	-2.065320
H	5.705658	-0.139433	-2.044317
H	5.939899	0.300252	0.939032
H	4.271531	0.048106	1.474196
H	4.601671	1.058769	0.052683

M062X energy = -1154.58875155 a.u.

(2*S*,6*R*,7*S*,8*R*)-**1**, Conf. N

C	-4.317746	-1.216131	0.181424
C	-3.728308	0.180052	0.150286
C	-3.147004	0.790356	-0.887891
C	-3.170641	0.269596	-2.301236
C	-1.952420	-1.884846	0.732668
C	-3.210476	-2.295623	0.014017
C	-0.803720	1.656801	-0.581056
C	-0.346804	0.985620	0.747570
C	0.131887	-0.474474	0.592026
C	-0.960113	-1.330856	0.030387
O	-0.062009	2.881581	-0.709298
C	0.846320	3.052397	0.278001
C	0.751691	1.899820	1.220043
O	1.585257	3.995990	0.328521
C	1.595024	1.778335	2.240162
C	-2.296365	2.018162	-0.663563
C	-2.014406	-1.911520	2.236310
O	-5.268952	-1.486129	-0.836187
O	1.242482	-0.470398	-0.317178
C	2.467789	-0.745234	0.184875
O	2.668913	-1.064202	1.330433
C	3.528363	-0.560381	-0.873362
C	4.871639	-1.184130	-0.497694
C	4.770317	-2.708423	-0.440385
C	5.947321	-0.746434	-1.490517
H	-4.787356	-1.356517	1.167147
H	-3.637118	0.658140	1.127136
H	-3.583451	1.033978	-2.970940
H	-3.773214	-0.633595	-2.395796
H	-3.018186	-2.387190	-1.059880
H	-3.606326	-3.254288	0.368318
H	-2.154416	0.062990	-2.661044
H	-1.183516	0.972785	1.458931
H	0.483789	-0.834871	1.561453
H	-1.023882	-1.330504	-1.057684
H	1.579045	0.929696	2.916910
H	2.346598	2.547205	2.396909
H	-2.577033	2.536224	0.261007
H	-0.516084	1.034238	-1.435308
H	-2.407829	2.729975	-1.489927
H	-2.707213	-1.144079	2.609626
H	-1.043786	-1.741817	2.707380
H	-2.396818	-2.876633	2.585630
H	-5.925125	-0.774835	-0.817216
H	3.151815	-0.963507	-1.821228
H	3.626775	0.524343	-1.020370
H	5.136409	-0.818413	0.501679

H	5.730593	-3.150094	-0.156203
H	4.021331	-3.028291	0.289078
H	4.494903	-3.111427	-1.423008
H	6.918680	-1.175684	-1.226044
H	6.053322	0.343017	-1.515432
H	5.698472	-1.084092	-2.503825

M062X energy = -1154.58873823 a.u.

(2*S*,6*R*,7*S*,8*R*)-**1**, Conf. O

C	-4.166199	-1.013036	-0.378502
C	-3.357202	0.148863	-0.885777
C	-3.060517	1.289816	-0.252956
C	-3.707480	1.771674	1.019877
C	-2.002524	-1.780087	0.637424
C	-3.219319	-2.216977	-0.137864
C	-0.600251	1.391273	-0.783256
C	-0.176528	0.819468	0.597284
C	0.259764	-0.657903	0.510210
C	-0.867845	-1.508768	-0.012142
O	0.450972	2.274807	-1.202864
C	1.347182	2.514377	-0.217231
C	0.954752	1.723824	0.988384
O	2.281488	3.255397	-0.347359
C	1.581513	1.867219	2.151020
C	-1.926666	2.163351	-0.766612
C	-2.222046	-1.463998	2.092276
O	-5.120699	-1.465053	-1.324642
O	1.353798	-0.681024	-0.415588
C	2.235872	-1.701168	-0.278660
O	2.137193	-2.537358	0.584092
C	3.342916	-1.617589	-1.299108
C	4.592545	-0.902762	-0.738279
C	4.281627	0.537686	-0.333978
C	5.213971	-1.681680	0.420670
H	-4.661644	-0.757320	0.568856
H	-2.843336	-0.071286	-1.825095
H	-4.510817	1.122074	1.372394
H	-4.128138	2.771748	0.859009
H	-2.931667	-2.593252	-1.125635
H	-3.785748	-3.005670	0.370279
H	-2.969802	1.876374	1.826132
H	-1.006865	0.866830	1.308894
H	0.620400	-0.993115	1.488482
H	-0.796009	-1.750677	-1.072127
H	1.301189	1.302226	3.035541
H	2.408017	2.568451	2.228302
H	-1.802231	3.043605	-0.123409
H	-0.646352	0.595205	-1.530637
H	-2.122034	2.534033	-1.778930
H	-2.874007	-0.587042	2.207635
H	-1.289312	-1.264056	2.625943
H	-2.724625	-2.299480	2.591828
H	-5.699175	-0.719172	-1.537353
H	3.600778	-2.643255	-1.578589
H	2.976611	-1.084451	-2.180673
H	5.314634	-0.880437	-1.564689

H	5.198114	1.065275	-0.052359
H	3.805557	1.097571	-1.144780
H	3.606022	0.566210	0.529134
H	6.132456	-1.191851	0.759133
H	5.460320	-2.707384	0.127967
H	4.524270	-1.736817	1.268971

M062X energy = -1154.58873170 a.u.

(2*S*,6*R*,7*S*,8*R*)-**1**, Conf. P

C	-4.429338	-0.770738	-0.483387
C	-3.517177	0.338173	-0.916042
C	-3.110052	1.393326	-0.203611
C	-3.713233	1.850648	1.098909
C	-2.341444	-1.797062	0.487028
C	-3.580823	-2.068724	-0.325366
C	-0.653668	1.294507	-0.742106
C	-0.254109	0.634559	0.607497
C	0.007534	-0.886893	0.469526
C	-1.177433	-1.585790	-0.133193
O	0.466235	2.087890	-1.167879
C	1.432772	2.162751	-0.226005
C	1.004998	1.372819	0.966499
O	2.455564	2.771821	-0.384750
C	1.736335	1.348822	2.075480
C	-1.898480	2.186442	-0.668278
C	-2.558030	-1.550082	1.955875
O	-5.418335	-0.955161	-1.480347
O	1.121247	-1.022577	-0.422468
C	2.322047	-1.354098	0.113443
O	2.466376	-1.721494	1.251739
C	3.415073	-1.202145	-0.916385
C	4.812918	-1.095917	-0.305695
C	5.855514	-1.060292	-1.422534
C	4.926293	0.141768	0.586298
H	-4.897728	-0.532876	0.482015
H	-3.032367	0.140106	-1.874851
H	-2.978109	1.823438	1.913938
H	-4.585244	1.267247	1.400265
H	-3.314205	-2.394796	-1.336900
H	-4.207781	-2.848481	0.129825
H	-4.027846	2.897009	1.004380
H	-1.042707	0.772856	1.355088
H	0.289660	-1.294333	1.444896
H	-1.106412	-1.764331	-1.205200
H	1.463152	0.753228	2.941452
H	2.651727	1.932795	2.119755
H	-1.690518	3.010421	0.025736
H	-0.795608	0.534598	-1.513933
H	-2.059719	2.633029	-1.655468
H	-3.162684	-2.351908	2.394405
H	-3.107456	-0.612613	2.115188
H	-1.621245	-1.489142	2.515546
H	-5.937386	-1.737646	-1.244079
H	3.346594	-2.065210	-1.592560
H	3.188371	-0.315847	-1.521686
H	4.979001	-1.987329	0.311942

H	6.867292	-1.014679	-1.008102
H	5.791367	-1.945372	-2.064633
H	5.712915	-0.174349	-2.052728
H	5.956801	0.276082	0.930695
H	4.285388	0.046339	1.466736
H	4.627864	1.045471	0.040199

M062X energy = -1154.58853793 a.u.

(2*S*,6*R*,7*S*,8*R*)-**1**, Conf. Q

C	-3.957686	-1.350611	-0.442805
C	-3.164840	-0.261789	-1.111096
C	-3.043116	1.018355	-0.741239
C	-3.908369	1.716071	0.276155
C	-1.931690	-1.615538	1.011753
C	-2.972620	-2.354177	0.209592
C	-0.540082	1.248208	-0.964328
C	-0.258872	1.097876	0.557806
C	0.229312	-0.319842	0.943302
C	-0.732882	-1.373185	0.474760
O	0.484453	2.100112	-1.499140
C	1.270388	2.632390	-0.535030
C	0.814028	2.124421	0.792585
O	2.180973	3.377128	-0.771969
C	1.395627	2.520522	1.919742
C	-1.906829	1.856791	-1.304184
C	-2.407575	-1.000727	2.300284
O	-4.721900	-2.112271	-1.362779
O	1.481881	-0.517761	0.274512
C	2.608062	-0.511462	1.031159
O	2.602719	-0.456920	2.234187
C	3.838181	-0.543427	0.158278
C	3.778080	-1.562455	-0.987375
C	5.066910	-1.496049	-1.804961
C	3.542327	-2.973884	-0.450046
H	-4.608137	-0.933684	0.339166
H	-2.499821	-0.639323	-1.892113
H	-3.315997	2.065737	1.131474
H	-4.351009	2.612160	-0.175660
H	-2.505289	-2.913051	-0.608546
H	-3.551072	-3.061559	0.814681
H	-4.721919	1.094212	0.654529
H	-1.165217	1.303417	1.138098
H	0.404295	-0.356866	2.022320
H	-0.472557	-1.840516	-0.474220
H	1.108024	2.131268	2.891718
H	2.203250	3.246047	1.874005
H	-1.939683	2.872894	-0.891181
H	-0.428778	0.286376	-1.471659
H	-1.975419	1.954206	-2.393405
H	-1.592702	-0.584365	2.897909
H	-2.928297	-1.747934	2.909380
H	-3.127000	-0.194565	2.103572
H	-5.308765	-1.502434	-1.831459
H	3.960093	0.469696	-0.249268
H	4.690354	-0.743549	0.815210
H	2.936022	-1.288794	-1.633514

H	5.031288	-2.195945	-2.645596
H	5.234810	-0.491491	-2.206020
H	5.930869	-1.761962	-1.184524
H	3.515151	-3.702299	-1.266605
H	2.593870	-3.043766	0.093259
H	4.348501	-3.265127	0.233873

M062X energy = -1154.58844891 a.u.

(2*S*,6*R*,7*S*,8*R*)-**1**, Conf. R

C	-4.125882	-0.869424	-0.601494
C	-3.116453	0.075324	-1.192745
C	-2.807116	1.314999	-0.795197
C	-3.605795	2.143481	0.177229
C	-2.264108	-1.502151	0.957132
C	-3.371339	-2.037191	0.085962
C	-0.309833	1.088763	-0.845314
C	-0.173288	0.880492	0.689371
C	0.085151	-0.607296	1.044129
C	-1.007360	-1.485780	0.503485
O	0.906621	1.711674	-1.291264
C	1.712535	2.060402	-0.264250
C	1.032525	1.713846	1.019978
O	2.798605	2.550067	-0.421433
C	1.551150	2.057682	2.194068
C	-1.506755	1.948485	-1.266382
C	-2.696707	-0.806145	2.219805
O	-4.946285	-1.476328	-1.585541
O	1.320137	-0.931087	0.393376
C	2.433285	-1.037611	1.167187
O	2.395418	-1.216956	2.356581
C	3.692475	-0.804525	0.365534
C	3.607443	-1.052482	-1.142322
C	4.883788	-0.547900	-1.813718
C	3.367866	-2.531356	-1.443397
H	-4.745819	-0.356993	0.147887
H	-2.474478	-0.404386	-1.935783
H	-3.874106	3.097941	-0.291889
H	-3.013065	2.395386	1.066102
H	-2.963604	-2.662365	-0.715921
H	-4.098920	-2.637010	0.644579
H	-4.527608	1.659994	0.506176
H	-1.075513	1.211396	1.213696
H	0.220254	-0.711321	2.124981
H	-0.768714	-1.997255	-0.427981
H	1.087296	1.783802	3.137043
H	2.479004	2.622459	2.222764
H	-1.386354	2.949023	-0.832570
H	-0.355225	0.126188	-1.359891
H	-1.480832	2.066316	-2.355319
H	-3.236273	0.122504	1.989083
H	-1.856244	-0.552557	2.870820
H	-3.386173	-1.440613	2.787666
H	-5.384080	-0.768937	-2.079708
H	3.938896	0.252298	0.547398
H	4.489700	-1.398330	0.826245
H	2.763079	-0.470980	-1.531736

H	4.848370	-0.718404	-2.894214
H	5.015828	0.525239	-1.642843
H	5.764151	-1.070797	-1.420435
H	3.290776	-2.703230	-2.521679
H	2.444627	-2.889144	-0.976525
H	4.199576	-3.138849	-1.066076

M062X energy = -1154.58844054 a.u.

(2*R*,6*R*,7*S*,8*R*)-**1**, Conf. A

C	4.460132	-0.043910	0.425676
C	3.451893	0.977518	-0.048068
C	2.591584	1.662878	0.708918
C	2.633812	1.667328	2.214516
C	2.527098	-1.640106	0.090836
C	3.759796	-1.348156	0.907095
C	0.155300	1.470789	0.147543
C	0.125716	0.304407	-0.881544
C	0.087390	-1.091816	-0.221729
C	1.331913	-1.304442	0.586269
O	-0.994662	2.294409	-0.114122
C	-1.748110	1.831897	-1.139617
C	-1.109812	0.599926	-1.684555
O	-2.762148	2.366483	-1.495541
C	-1.660195	-0.074381	-2.689053
C	1.406257	2.361927	0.085636
C	2.763073	-2.092263	-1.324449
O	5.330986	-0.417730	-0.632402
O	-1.047302	-1.121713	0.653276
C	-2.131327	-1.845739	0.277340
O	-2.167873	-2.534453	-0.710473
C	-3.261133	-1.609081	1.245682
C	-3.863448	-0.202194	1.054275
C	-4.569663	-0.087938	-0.295854
C	-4.825473	0.112669	2.198205
H	5.050292	0.345721	1.268989
H	3.334150	1.012514	-1.133281
H	3.534883	1.193524	2.610578
H	1.768216	1.142982	2.639720
H	3.480062	-1.217269	1.957980
H	4.505830	-2.148679	0.845307
H	2.594214	2.695228	2.592716
H	1.025883	0.336757	-1.508241
H	-0.051979	-1.851305	-0.995151
H	1.264741	-0.965202	1.620040
H	-1.236424	-0.995690	-3.076989
H	-2.581774	0.293956	-3.132268
H	1.601833	2.618881	-0.962149
H	0.021210	1.082799	1.162782
H	1.172100	3.290505	0.619170
H	3.303641	-1.323068	-1.888672
H	1.838925	-2.335657	-1.853617
H	3.406038	-2.979179	-1.332478
H	5.767489	0.384261	-0.953757
H	-4.022707	-2.375309	1.073969
H	-2.873176	-1.710909	2.264798

H	-3.036173	0.520745	1.087279
H	-4.915065	0.935143	-0.467311
H	-3.908099	-0.358436	-1.124226
H	-5.436381	-0.759556	-0.324846
H	-5.262186	1.107873	2.072186
H	-4.319698	0.081477	3.168757
H	-5.646653	-0.613942	2.220081

M062X energy = -1154.59276196 a.u.

(2*R*,6*R*,7*S*,8*R*)-**1**, Conf. B

C	4.465827	-0.025417	0.401015
C	3.456723	0.997094	-0.053937
C	2.589648	1.656582	0.716845
C	2.630027	1.632648	2.222619
C	2.529143	-1.630066	0.099878
C	3.758856	-1.323269	0.913972
C	0.153558	1.473951	0.160718
C	0.128849	0.310864	-0.872233
C	0.089674	-1.087847	-0.217633
C	1.331778	-1.300408	0.593953
O	-0.997542	2.296171	-0.101582
C	-1.746870	1.835810	-1.130537
C	-1.104598	0.606842	-1.678121
O	-2.761550	2.368690	-1.487489
C	-1.650612	-0.064595	-2.686895
C	1.404444	2.365001	0.104416
C	2.767521	-2.077716	-1.316646
O	5.284444	-0.291660	-0.726988
O	-1.047496	-1.123377	0.653602
C	-2.128395	-1.849194	0.271971
O	-2.158716	-2.537279	-0.716507
C	-3.262961	-1.615951	1.235551
C	-3.867602	-0.210184	1.043165
C	-4.568607	-0.096207	-0.309682
C	-4.835127	0.101231	2.183410
H	5.081345	0.368535	1.224583
H	3.368929	1.071239	-1.138360
H	2.563179	2.652429	2.618458
H	3.546557	1.180654	2.609249
H	3.477793	-1.171105	1.962049
H	4.491906	-2.141966	0.883927
H	1.781012	1.077305	2.642154
H	1.031501	0.347594	-1.495234
H	-0.045484	-1.845050	-0.994125
H	1.260968	-0.962875	1.627769
H	-1.224025	-0.983625	-3.077170
H	-2.571142	0.304202	-3.131886
H	1.598253	2.631764	-0.941149
H	0.016870	1.082963	1.174568
H	1.172572	3.288674	0.647417
H	3.286931	-1.297285	-1.885215
H	1.845400	-2.342213	-1.838929
H	3.425685	-2.954204	-1.328810
H	5.948287	-0.946025	-0.468759
H	-4.022090	-2.383695	1.059830
H	-2.879113	-1.717889	2.256232

H	-3.042132	0.514527	1.080462
H	-4.914763	0.926463	-0.481924
H	-3.903327	-0.365247	-1.135535
H	-5.434197	-0.769134	-0.342401
H	-5.273121	1.095776	2.056747
H	-4.333528	0.069770	3.156126
H	-5.655033	-0.626957	2.200677

M062X energy = -1154.59191884 a.u.

(2*R*,6*R*,7*S*,8*R*)-**1**, Conf. C

C	-4.407633	-0.132968	-0.333294
C	-3.416533	0.925694	0.090112
C	-2.585484	1.607718	-0.701513
C	-2.651367	1.569951	-2.205591
C	-2.428922	-1.668561	0.012051
C	-3.683123	-1.432729	-0.789274
C	-0.125633	1.523917	-0.173436
C	-0.026130	0.376460	0.873534
C	0.002642	-1.039893	0.259506
C	-1.256714	-1.296359	-0.510848
O	0.977064	2.413939	0.073632
C	1.790460	1.981239	1.066502
C	1.234210	0.715207	1.621238
O	2.790872	2.560932	1.387256
C	1.860381	0.053040	2.588691
C	-1.414362	2.360001	-0.116513
C	-2.627885	-2.105680	1.437496
O	-5.247836	-0.496460	0.752956
O	1.114287	-1.122151	-0.646192
C	2.181334	-1.870255	-0.274027
O	2.261725	-2.439885	0.785655
C	3.244091	-1.887447	-1.345816
C	4.122779	-0.616753	-1.360683
C	3.383825	0.603138	-1.908395
C	4.715307	-0.329695	0.018535
H	-5.023884	0.215844	-1.175771
H	-3.279428	0.994943	1.171515
H	-3.527472	1.032113	-2.575129
H	-1.760942	1.089944	-2.631651
H	-3.425232	-1.325471	-1.848314
H	-4.406928	-2.249749	-0.691264
H	-2.681026	2.588448	-2.609700
H	-0.898932	0.410154	1.539174
H	0.159597	-1.766498	1.060359
H	-1.222837	-0.972350	-1.551366
H	1.497416	-0.892306	2.979518
H	2.786019	0.454638	2.992749
H	-1.602000	2.634969	0.928130
H	0.024589	1.125760	-1.183891
H	-1.223046	3.284450	-0.674120
H	-3.192579	-1.348434	1.994241
H	-1.688318	-2.301928	1.959002
H	-3.234210	-3.017637	1.468574
H	-5.695188	0.304462	1.061825
H	3.868390	-2.760113	-1.138309
H	2.763457	-2.015894	-2.321045

H	4.947653	-0.845723	-2.048222
H	2.552011	0.890778	-1.259171
H	4.059989	1.461075	-1.973267
H	2.977473	0.408770	-2.906915
H	5.200568	-1.216190	0.439753
H	3.936166	-0.007711	0.718099
H	5.452346	0.476521	-0.040903

M062X energy = -1154.59173822 a.u.

(2*R*,6*R*,7*S*,8*R*)-**1**, Conf. D

C	-4.509506	-0.485332	-0.368708
C	-3.627166	0.717279	-0.126784
C	-2.842471	1.327981	-1.017821
C	-2.873624	1.028549	-2.493584
C	-2.406778	-1.751119	0.239615
C	-3.662811	-1.772143	-0.592826
C	-0.400058	1.546172	-0.471352
C	-0.230851	0.605431	0.756935
C	-0.046115	-0.880918	0.381053
C	-1.259181	-1.385514	-0.339825
O	0.627161	2.548260	-0.379793
C	1.447541	2.362380	0.680584
C	0.971632	1.179378	1.452802
O	2.393657	3.065459	0.905251
C	1.615643	0.764082	2.538814
C	-1.753062	2.269520	-0.562831
C	-2.589479	-1.929271	1.722046
O	-5.334619	-0.735526	0.760137
O	1.086726	-0.972463	-0.496332
C	2.215699	-1.544729	-0.019392
O	2.327787	-1.984532	1.097625
C	3.309085	-1.561522	-1.060508
C	4.633795	-1.036844	-0.491165
C	5.748363	-1.207632	-1.521385
C	4.496513	0.424828	-0.066274
H	-5.137101	-0.341584	-1.261451
H	-3.523880	0.983182	0.927426
H	-1.946413	0.541808	-2.822509
H	-2.959081	1.958647	-3.067225
H	-3.396135	-1.825009	-1.653845
H	-4.311712	-2.623105	-0.356832
H	-3.706441	0.379011	-2.772803
H	-1.123044	0.661321	1.393874
H	0.173464	-1.451351	1.287024
H	-1.232313	-1.254438	-1.421661
H	1.313806	-0.114812	3.099971
H	2.493071	1.311539	2.873465
H	-1.984495	2.700555	0.418454
H	-0.203162	0.995860	-1.397603
H	-1.629660	3.098077	-1.270033
H	-3.177599	-1.102715	2.139023
H	-1.641500	-1.993652	2.261256
H	-3.163475	-2.839773	1.924455
H	-5.858546	0.061407	0.925616
H	3.427401	-2.602692	-1.385875
H	2.998656	-0.971513	-1.928432

H	4.867085	-1.640033	0.394652
H	6.704382	-0.855991	-1.121689
H	5.867577	-2.255561	-1.816111
H	5.529986	-0.624885	-2.424195
H	5.441466	0.808193	0.330664
H	3.736347	0.549923	0.711118
H	4.212618	1.054547	-0.918224

M062X energy = -1154.59146367 a.u.

(2*R*,6*R*,7*S*,8*R*)-**1**, Conf. E

C	-4.464655	-0.429734	-0.237762
C	-3.554667	0.737428	0.064907
C	-2.814184	1.424191	-0.807967
C	-2.924376	1.260932	-2.301046
C	-2.351660	-1.760450	0.145680
C	-3.648426	-1.698291	-0.620305
C	-0.342167	1.603161	-0.366268
C	-0.115860	0.551521	0.759273
C	0.018158	-0.899805	0.252422
C	-1.236165	-1.326924	-0.448663
O	0.678003	2.605626	-0.216363
C	1.541103	2.329582	0.788286
C	1.131719	1.049443	1.434117
O	2.476583	3.031646	1.056255
C	1.865109	0.501715	2.397998
C	-1.699690	2.322601	-0.329311
C	-2.463952	-2.097876	1.607242
O	-5.235935	-0.773288	0.904739
O	1.106390	-0.947982	-0.683311
C	2.212495	-1.648008	-0.336009
O	2.355287	-2.184315	0.735586
C	3.226197	-1.657250	-1.454990
C	4.447282	-0.763217	-1.153548
C	4.023884	0.673391	-0.849686
C	5.303690	-1.327180	-0.020228
H	-5.134757	-0.199064	-1.079718
H	-3.388680	0.902851	1.131697
H	-3.050244	2.238138	-2.781240
H	-3.764194	0.626852	-2.594015
H	-3.438157	-1.659659	-1.694583
H	-4.295039	-2.561538	-0.426181
H	-2.011093	0.820355	-2.721437
H	-0.968221	0.566766	1.451924
H	0.264855	-1.546231	1.098328
H	-1.264029	-1.084717	-1.511312
H	1.618461	-0.452260	2.853526
H	2.762034	1.016106	2.733873
H	-1.879969	2.665304	0.696491
H	-0.179941	1.145599	-1.348498
H	-1.612390	3.211339	-0.965343
H	-2.989261	-3.051027	1.732149
H	-3.071091	-1.347399	2.127428
H	-1.493520	-2.174174	2.102877
H	-5.728253	0.014484	1.177176
H	3.554040	-2.694215	-1.583485
H	2.738399	-1.324875	-2.374668

H	5.047356	-0.765221	-2.072628
H	3.554811	0.731304	0.137753
H	4.889269	1.342295	-0.832773
H	3.309401	1.058901	-1.585877
H	5.639580	-2.345467	-0.241837
H	4.736589	-1.357912	0.915177
H	6.188409	-0.701400	0.135094

M062X energy = -1154.59115685 a.u.

(2*R*,6*R*,7*S*,8*R*)-**1**, Conf. F

C	4.412875	-0.112452	0.312959
C	3.421649	0.945148	-0.096870
C	2.582894	1.604247	0.704757
C	2.645370	1.543686	2.208528
C	2.431616	-1.658133	-0.000705
C	3.681891	-1.406434	0.800596
C	0.124132	1.525676	0.181733
C	0.028941	0.379897	-0.867506
C	-0.000709	-1.037627	-0.256189
C	1.256031	-1.292295	0.518941
O	-0.981057	2.413016	-0.065186
C	-1.790887	1.980831	-1.060584
C	-1.229938	0.717712	-1.617914
O	-2.792774	2.557808	-1.381949
C	-1.851955	0.057185	-2.589116
C	1.412052	2.362671	0.127654
C	2.635597	-2.091562	-1.426746
O	5.206952	-0.366955	-0.835164
O	-1.115099	-1.123547	0.645539
C	-2.179095	-1.873908	0.269024
O	-2.253385	-2.444496	-0.790584
C	-3.246137	-1.893347	1.336496
C	-4.125922	-0.623364	1.350662
C	-3.390773	0.595939	1.904804
C	-4.712577	-0.333713	-0.030493
H	5.050474	0.243265	1.137092
H	3.316462	1.048636	-1.177395
H	2.635931	2.556564	2.627397
H	3.543886	1.038195	2.570524
H	3.421622	-1.277924	1.857109
H	4.392957	-2.242145	0.735049
H	1.775501	1.022843	2.629413
H	0.904055	0.416381	-1.529978
H	-0.153388	-1.763320	-1.058761
H	1.217116	-0.968945	1.559192
H	-1.485570	-0.885787	-2.982498
H	-2.777230	0.458204	-2.994569
H	1.598451	2.644193	-0.915364
H	-0.027008	1.125810	1.191462
H	1.221976	3.283582	0.691519
H	3.254503	-2.995762	-1.460265
H	3.183424	-1.323837	-1.985921
H	1.698034	-2.306019	-1.944424
H	5.862961	-1.038669	-0.602960
H	-3.868954	-2.766107	1.124981
H	-2.769100	-2.023300	2.313330

H	-4.953600	-0.854973	2.033950
H	-4.068201	1.452952	1.968569
H	-2.989115	0.399552	2.904843
H	-2.556189	0.886291	1.260423
H	-5.450695	0.471611	0.027497
H	-5.195060	-1.219714	-0.455960
H	-3.930668	-0.009112	-0.725683

M062X energy = -1154.59086087 a.u.

(2*R*,6*R*,7*S*,8*R*)-**1**, Conf. G

C	4.318271	-1.222018	0.297266
C	3.736289	0.165635	0.162254
C	3.106046	0.864708	1.108661
C	3.055947	0.448715	2.555326
C	1.989964	-1.918389	-0.387810
C	3.196265	-2.291278	0.434655
C	0.782957	1.709581	0.606649
C	0.395767	0.942329	-0.692425
C	-0.093530	-0.503122	-0.462066
C	0.960753	-1.319360	0.218854
O	0.049066	2.946163	0.598114
C	-0.802303	3.044697	-0.449189
C	-0.669850	1.820793	-1.290314
O	-1.526334	3.986666	-0.614052
C	-1.462116	1.623171	-2.339255
C	2.271222	2.068739	0.744874
C	2.140860	-2.047231	-1.879145
O	5.065899	-1.567053	-0.860187
O	-1.256260	-0.439180	0.378322
C	-2.448988	-0.756220	-0.174091
O	-2.582774	-1.147778	-1.306895
C	-3.569687	-0.519018	0.809379
C	-4.894663	-1.144869	0.376104
C	-4.811373	-2.671284	0.392807
C	-6.026208	-0.651534	1.276568
H	4.959993	-1.296632	1.188288
H	3.700988	0.531154	-0.866231
H	3.349320	1.284765	3.200506
H	3.713122	-0.396202	2.773462
H	2.914706	-2.358988	1.490971
H	3.632833	-3.249703	0.131911
H	2.038492	0.164738	2.853744
H	1.269580	0.877727	-1.354584
H	-0.388120	-0.930090	-1.423666
H	0.953725	-1.253157	1.307026
H	-1.422970	0.722455	-2.943888
H	-2.196550	2.382721	-2.593785
H	2.607119	2.511816	-0.200068
H	0.440654	1.155100	1.487765
H	2.344311	2.842769	1.517878
H	2.902019	-1.350099	-2.249743
H	1.207533	-1.867575	-2.417713
H	2.499555	-3.049768	-2.135287
H	5.767000	-0.908216	-0.967821
H	-3.255341	-0.889163	1.792921
H	-3.664086	0.571180	0.909881

H	-5.088641	-0.821949	-0.653837
H	-5.756985	-3.114194	0.064727
H	-4.020553	-3.031569	-0.270369
H	-4.607630	-3.033011	1.408379
H	-6.984078	-1.082166	0.968696
H	-6.119098	0.439000	1.245931
H	-5.847413	-0.945760	2.317890

M062X energy = -1154.59061929 a.u.

(2*R*,6*R*,7*S*,8*R*)-**1**, Conf. H

C	-4.517002	-0.463123	-0.343013
C	-3.634079	0.736288	-0.115478
C	-2.843825	1.326274	-1.014461
C	-2.875041	1.007350	-2.486516
C	-2.407758	-1.745763	0.218848
C	-3.663562	-1.745842	-0.613250
C	-0.401142	1.549268	-0.480475
C	-0.234341	0.607299	0.747241
C	-0.047626	-0.878756	0.370662
C	-1.258023	-1.380448	-0.356405
O	0.628305	2.549220	-0.387425
C	1.446217	2.362083	0.674099
C	0.966551	1.180340	1.446334
O	2.393967	3.062592	0.900428
C	1.606310	0.766014	2.535189
C	-1.753992	2.272500	-0.570748
C	-2.591144	-1.930898	1.700609
O	-5.296739	-0.599798	0.834535
O	1.088666	-0.970718	-0.501809
C	2.214284	-1.546207	-0.020587
O	2.319429	-1.989996	1.095532
C	3.313150	-1.561570	-1.055884
C	4.635559	-1.040030	-0.478337
C	5.755332	-1.210759	-1.502888
C	4.498202	0.421024	-0.051426
H	-5.174970	-0.308353	-1.212288
H	-3.561182	1.030237	0.932050
H	-3.730399	0.385399	-2.761295
H	-1.964641	0.485029	-2.808180
H	-3.399451	-1.767454	-1.676432
H	-4.295800	-2.622253	-0.412007
H	-2.928179	1.932550	-3.071644
H	-1.128618	0.663037	1.381294
H	0.167209	-1.450505	1.277003
H	-1.227935	-1.243596	-1.437150
H	1.301356	-0.111384	3.097019
H	2.483002	1.313243	2.872126
H	-1.982032	2.708974	0.408810
H	-0.204356	1.000102	-1.407527
H	-1.633815	3.097055	-1.283172
H	-3.181856	-2.832284	1.899231
H	-3.157288	-1.094110	2.127154
H	-1.642641	-2.023236	2.234605
H	-5.881302	-1.361897	0.721098
H	3.431969	-2.602064	-1.383281
H	3.007900	-0.968921	-1.923828

H	4.863191	-1.645319	0.407562
H	6.709743	-0.861379	-1.097417
H	5.874461	-2.258372	-1.798837
H	5.542769	-0.626032	-2.405805
H	5.441819	0.802402	0.350560
H	3.734586	0.545724	0.722606
H	4.219013	1.052734	-0.903432

M062X energy = -1154.59060660 a.u.

(2*R*,6*R*,7*S*,8*R*)-**1**, Conf. I

C	-4.502169	-0.683974	-0.134140
C	-3.679929	0.550528	0.153725
C	-3.046102	1.315487	-0.738323
C	-3.223163	1.173358	-2.227369
C	-2.273613	-1.846516	0.122839
C	-3.600269	-1.866099	-0.591992
C	-0.578910	1.645805	-0.417714
C	-0.213930	0.626598	0.700659
C	0.019468	-0.807566	0.172979
C	-1.214647	-1.321483	-0.501745
O	0.396429	2.700606	-0.363037
C	1.353066	2.481872	0.566902
C	1.033585	1.221235	1.295682
O	2.288937	3.216949	0.724847
C	1.830944	0.765121	2.256254
C	-1.972719	2.282216	-0.296566
C	-2.302061	-2.221901	1.579458
O	-5.179890	-1.120607	1.035177
O	1.089846	-0.722967	-0.778612
C	2.299874	-1.211558	-0.415373
O	2.484646	-1.846297	0.593365
C	3.341083	-0.842241	-1.447324
C	4.769365	-0.728829	-0.894880
C	4.835794	0.221687	0.303449
C	5.375674	-2.091655	-0.557299
H	-5.232625	-0.494878	-0.935215
H	-3.471351	0.705162	1.214686
H	-2.314418	0.779203	-2.700948
H	-3.413964	2.151805	-2.682647
H	-3.436691	-1.771677	-1.670920
H	-4.165078	-2.786876	-0.406751
H	-4.048985	0.508513	-2.490824
H	-1.025140	0.573878	1.437919
H	0.340189	-1.444675	1.000750
H	-1.299402	-1.060297	-1.556635
H	1.646449	-0.171111	2.774463
H	2.714976	1.337000	2.525323
H	-2.125569	2.599177	0.741856
H	-0.463152	1.179844	-1.402105
H	-1.973915	3.181990	-0.922698
H	-2.785010	-3.196798	1.705853
H	-2.909701	-1.508539	2.148914
H	-1.306121	-2.273013	2.025248
H	-5.730544	-0.388942	1.348853
H	3.295129	-1.600115	-2.241053
H	3.027408	0.104159	-1.899052

H	5.361448	-0.288552	-1.708181
H	4.360652	-0.236157	1.176844
H	5.877176	0.438250	0.561184
H	4.331674	1.174082	0.100148
H	5.375625	-2.752533	-1.431102
H	4.810674	-2.577633	0.241917
H	6.412487	-1.975258	-0.225088

M062X energy = -1154.59036081 a.u.

(2*R*,6*R*,7*S*,8*R*)-**1**, Conf. J

C	-4.256744	-0.647122	-1.099443
C	-3.229290	0.443503	-1.291549
C	-2.854417	1.463800	-0.511322
C	-3.555700	1.976644	0.722405
C	-2.399368	-1.762207	0.206234
C	-3.519054	-1.978914	-0.781198
C	-0.344934	1.218784	-0.720586
C	-0.194969	0.504936	0.651939
C	-0.004432	-1.023595	0.511888
C	-1.149051	-1.635806	-0.246786
O	0.857263	1.978544	-0.929041
C	1.630211	2.033227	0.180715
C	1.013507	1.179059	1.237557
O	2.646618	2.670179	0.235903
C	1.564381	1.058347	2.440977
C	-1.543056	2.173257	-0.820175
C	-2.801941	-1.492660	1.629922
O	-5.196005	-0.432001	-0.060162
O	1.190517	-1.241679	-0.249332
C	2.281420	-1.730433	0.393393
O	2.298629	-2.008793	1.565439
C	3.442127	-1.822803	-0.562780
C	4.022832	-0.428741	-0.872193
C	4.645954	0.191786	0.377061
C	5.051313	-0.534781	-1.996708
H	-4.794359	-0.799571	-2.048494
H	-2.600713	0.239185	-2.162243
H	-2.862941	2.001078	1.573368
H	-4.422157	1.383950	1.007209
H	-3.112818	-2.374968	-1.717746
H	-4.274086	-2.681711	-0.411489
H	-3.867291	3.016323	0.558244
H	-1.088293	0.663430	1.265891
H	0.131366	-1.460038	1.506010
H	-0.940435	-1.830058	-1.298315
H	1.150576	0.409302	3.206824
H	2.470116	1.613104	2.670918
H	-1.381396	2.993305	-0.109578
H	-0.384752	0.487269	-1.531594
H	-1.542923	2.617872	-1.821463
H	-3.309003	-0.523726	1.708018
H	-1.948683	-1.499164	2.313792
H	-3.521973	-2.244293	1.970335
H	-5.746374	0.323136	-0.313678
H	4.206568	-2.457131	-0.103987
H	3.094584	-2.292979	-1.488876

H	3.197908	0.209814	-1.215415
H	3.933700	0.232861	1.206710
H	5.507914	-0.403673	0.702194
H	4.980719	1.212626	0.175334
H	5.471898	0.448498	-2.228034
H	4.606562	-0.939437	-2.911667
H	5.877386	-1.192687	-1.700835

M062X energy = -1154.58998272 a.u.

(2*R*,6*R*,7*S*,8*R*)-**1**, Conf. K

C	4.033736	-1.250532	0.268596
C	3.465031	0.136228	0.455932
C	2.706899	0.562044	1.468746
C	2.459503	-0.241996	2.718123
C	1.830133	-1.644847	-0.903531
C	2.914277	-2.277682	-0.070513
C	0.471545	1.596999	0.920463
C	0.267779	1.238775	-0.581372
C	-0.236281	-0.196834	-0.833710
C	0.726807	-1.206804	-0.289501
O	-0.256986	2.812051	1.163279
C	-0.958633	3.230041	0.083956
C	-0.717839	2.280265	-1.039388
O	-1.649869	4.210311	0.096750
C	-1.372755	2.395933	-2.190106
C	1.925511	1.848288	1.353023
C	2.182699	-1.356276	-2.336859
O	4.949850	-1.275510	-0.816710
O	-1.491485	-0.342018	-0.153719
C	-2.602587	-0.524848	-0.906478
O	-2.599825	-0.553761	-2.110822
C	-3.813637	-0.699270	-0.024321
C	-3.630434	-1.806754	1.024955
C	-4.907548	-1.956453	1.849125
C	-3.245299	-3.128229	0.359824
H	4.536321	-1.597877	1.183875
H	3.568369	0.777657	-0.422120
H	3.065101	-1.150226	2.761351
H	1.405888	-0.539566	2.798065
H	2.488335	-2.641904	0.870518
H	3.399535	-3.119372	-0.577389
H	2.682975	0.359472	3.606836
H	1.222725	1.338912	-1.114681
H	-0.410078	-0.325223	-1.904749
H	0.575979	-1.450821	0.762728
H	-1.252124	1.689933	-3.005846
H	-2.075796	3.214377	-2.317934
H	2.387336	2.527972	0.627262
H	0.012356	0.830437	1.555176
H	1.886668	2.370043	2.316644
H	3.035740	-0.669457	-2.388555
H	1.351573	-0.931952	-2.904584
H	2.505213	-2.276813	-2.835724
H	5.649640	-0.633824	-0.628048
H	-4.010269	0.258224	0.473628
H	-4.659573	-0.923397	-0.680955

H	-2.813632	-1.501094	1.689693
H	-4.783789	-2.717876	2.625432
H	-5.182283	-1.015596	2.337039
H	-5.744510	-2.262295	1.210517
H	-3.142909	-3.923250	1.104971
H	-2.292713	-3.046873	-0.175326
H	-4.013012	-3.436492	-0.359838

M062X energy = -1154.58976402 a.u.

(2*R*,6*R*,7*S*,8*R*)-**1**, Conf. L

C	4.333139	-1.193566	0.282468
C	3.745786	0.188337	0.161251
C	3.099362	0.865868	1.111220
C	3.041495	0.433920	2.553148
C	1.998547	-1.912039	-0.372290
C	3.208397	-2.266600	0.451770
C	0.777782	1.709198	0.612578
C	0.398602	0.938241	-0.686695
C	-0.089854	-0.507419	-0.455087
C	0.963633	-1.319883	0.231393
O	0.038288	2.942656	0.599017
C	-0.809043	3.035949	-0.451394
C	-0.667139	1.811863	-1.291183
O	-1.537983	3.973510	-0.620374
C	-1.453344	1.610119	-2.343868
C	2.264286	2.071994	0.755612
C	2.152592	-2.036017	-1.864023
O	5.074231	-1.404834	-0.908690
O	-1.255640	-0.443096	0.380507
C	-2.445437	-0.764924	-0.175691
O	-2.572355	-1.170864	-1.304217
C	-3.572019	-0.512072	0.796888
C	-4.893920	-1.146713	0.367137
C	-4.808649	-2.672534	0.409347
C	-6.030582	-0.640336	1.253778
H	4.999691	-1.263305	1.156028
H	3.750307	0.574511	-0.858499
H	3.322390	1.267816	3.206713
H	3.710699	-0.402561	2.768133
H	2.932484	-2.316109	1.511001
H	3.628073	-3.243805	0.174233
H	2.026802	0.135010	2.846307
H	1.276205	0.874159	-1.343889
H	-0.380104	-0.937365	-1.416780
H	0.951752	-1.253625	1.319271
H	-1.407136	0.709338	-2.947991
H	-2.189307	2.366619	-2.602994
H	2.601146	2.521955	-0.185607
H	0.433627	1.155133	1.493313
H	2.334443	2.840910	1.534036
H	2.889948	-1.315072	-2.237170
H	1.212468	-1.886597	-2.399893
H	2.536841	-3.029120	-2.122704
H	5.472810	-2.285026	-0.863994
H	-3.263278	-0.864066	1.788819
H	-3.667859	0.579718	0.877011

H	-5.083164	-0.841023	-0.668920
H	-5.751936	-3.122235	0.083835
H	-4.013972	-3.042406	-0.243909
H	-4.609507	-3.017128	1.431773
H	-6.986384	-1.077109	0.948146
H	-6.124560	0.449441	1.205026
H	-5.856611	-0.917351	2.300620

M062X energy = -1154.58975417 a.u.

(2*R*,6*R*,7*S*,8*R*)-**1**, Conf. M

C	-4.508730	-0.664595	-0.111135
C	-3.687120	0.567986	0.162425
C	-3.043108	1.311648	-0.739067
C	-3.214044	1.149248	-2.227132
C	-2.277384	-1.838069	0.109140
C	-3.604427	-1.840927	-0.603948
C	-0.578017	1.649846	-0.423670
C	-0.216296	0.629284	0.694533
C	0.017215	-0.804685	0.166164
C	-1.215780	-1.315427	-0.512802
O	0.398605	2.703719	-0.365596
C	1.353876	2.481998	0.564454
C	1.031280	1.221191	1.292024
O	2.291936	3.214122	0.723903
C	1.826006	0.763819	2.254134
C	-1.972364	2.284659	-0.305100
C	-2.307656	-2.214277	1.565812
O	-5.151323	-0.983326	1.113214
O	1.089943	-0.721438	-0.782587
C	2.298069	-1.213064	-0.416714
O	2.477900	-1.852405	0.590001
C	3.343520	-0.840909	-1.443307
C	4.770107	-0.732549	-0.885447
C	4.834492	0.212664	0.317129
C	5.372495	-2.098073	-0.551608
H	-5.262253	-0.471770	-0.890166
H	-3.514435	0.749378	1.223763
H	-2.317178	0.720613	-2.693571
H	-3.375288	2.125764	-2.697543
H	-3.444054	-1.724096	-1.681546
H	-4.155312	-2.780044	-0.451982
H	-4.060812	0.507929	-2.483410
H	-1.029322	0.577533	1.429867
H	0.334530	-1.443366	0.994103
H	-1.297329	-1.051723	-1.567056
H	1.639069	-0.171992	2.772260
H	2.710147	1.334616	2.525154
H	-2.125447	2.607421	0.731377
H	-0.459436	1.185265	-1.408418
H	-1.976031	3.180681	-0.936629
H	-2.802959	-3.183535	1.693866
H	-2.898475	-1.490160	2.139368
H	-1.310584	-2.285090	2.006056
H	-5.692542	-1.772523	0.972432
H	3.299259	-1.595235	-2.240535
H	3.033041	0.107993	-1.891906
H	5.365744	-0.290048	-1.694930
H	5.875499	0.426876	0.578304
H	4.331965	1.166520	0.116729
H	4.356660	-0.248356	1.187368
H	6.408485	-1.985251	-0.215649
H	5.373794	-2.755450	-1.428061
H	4.803959	-2.585932	0.243961

M062X energy = -1154.58953553 a.u.

(2*R*,6*R*,7*S*,8*S*)-**1**, Conf. A

C	1.870066	-3.307426	-0.336302
C	2.474407	-1.975424	-0.712575
C	3.130030	-1.141151	0.097367
C	3.560521	-1.510485	1.492938
C	-0.096238	-1.822356	0.234716
C	0.601171	-3.121032	0.553711
C	2.295035	1.215928	0.356015
C	0.869881	1.180368	-0.278465
C	-0.182602	0.671207	0.730278
C	0.066860	-0.780400	1.059592
O	2.770815	2.566269	0.250843
C	1.879767	3.393152	-0.346134
C	0.666195	2.605897	-0.713556
O	2.094460	4.558851	-0.525836
C	-0.353114	3.191016	-1.335363
C	3.342840	0.299944	-0.296805
C	-0.771791	-1.776146	-1.111111
O	1.458989	-4.018893	-1.495945
O	-1.477812	0.889719	0.154715
C	-2.503852	0.898787	1.040101
O	-2.348109	0.788138	2.228789
C	-3.829448	1.001062	0.331326
C	-4.140918	-0.289742	-0.448025
C	-4.045403	-1.512858	0.464802
C	-5.523539	-0.186938	-1.088864
H	2.585529	-3.919030	0.233053
H	2.174938	-1.615492	-1.699069
H	3.450051	-2.578262	1.695725
H	2.976543	-0.969837	2.248782
H	0.911962	-3.125165	1.603556
H	-0.034534	-3.996772	0.379463
H	4.610234	-1.239594	1.653473
H	0.867088	0.502629	-1.140912
H	-0.121284	1.272716	1.643135
H	0.568627	-0.952351	2.010798
H	-1.243439	2.645417	-1.625925
H	-0.290736	4.252670	-1.558928
H	3.287977	0.426969	-1.384328
H	2.236131	0.996720	1.429047
H	4.325919	0.657209	0.031856
H	-1.052737	-0.767472	-1.414927
H	-1.684387	-2.386261	-1.082496
H	-0.128439	-2.230777	-1.871730
H	2.233643	-4.122158	-2.067278
H	-4.593046	1.173901	1.095352

H	-3.812998	1.856787	-0.352860
H	-3.391443	-0.388054	-1.243560
H	-3.041800	-1.627902	0.890088
H	-4.751161	-1.423394	1.299065
H	-4.288214	-2.426580	-0.087527
H	-6.296936	-0.090210	-0.318030
H	-5.748085	-1.081472	-1.677942
H	-5.595353	0.682658	-1.750280

M062X energy = -1154.58726526 a.u.

(2*R*,6*R*,7*S*,8*S*)-**1**, Conf. B

C	1.935070	-3.317572	-0.329897
C	2.573970	-1.986543	-0.644228
C	3.186308	-1.172037	0.217932
C	3.526085	-1.563621	1.631895
C	-0.062378	-1.828642	0.118713
C	0.612041	-3.130336	0.475853
C	2.362797	1.194577	0.446658
C	0.987425	1.193775	-0.290011
C	-0.144736	0.670322	0.618645
C	0.079730	-0.785486	0.945073
O	2.867736	2.537299	0.398031
C	2.032202	3.390983	-0.240005
C	0.832058	2.633102	-0.701203
O	2.277430	4.555935	-0.381271
C	-0.139085	3.253714	-1.364408
C	3.440958	0.271070	-0.143162
C	-0.688638	-1.794356	-1.251442
O	1.596284	-4.008185	-1.524967
O	-1.379280	0.894261	-0.075666
C	-2.491669	0.777915	0.681689
O	-2.462178	0.580402	1.869845
C	-3.752679	0.911548	-0.139217
C	-4.806187	-0.105813	0.312906
C	-6.122699	0.126747	-0.424344
C	-4.297571	-1.531551	0.096434
H	2.605109	-3.944312	0.276902
H	2.338714	-1.607901	-1.641241
H	3.378610	-2.629780	1.818217
H	2.912713	-1.014191	2.357664
H	0.855150	-3.140640	1.543412
H	-0.013769	-4.003304	0.257728
H	4.570875	-1.319493	1.855406
H	1.037796	0.537345	-1.167754
H	-0.168890	1.259148	1.542137
H	0.546525	-0.961353	1.913340
H	-1.021836	2.735399	-1.720156
H	-0.042671	4.319641	-1.553219
H	3.462782	0.410553	-1.230301
H	2.221675	0.960610	1.509076
H	4.403502	0.612756	0.255391
H	-0.010074	-2.245296	-1.983370
H	-0.970187	-0.791940	-1.573731
H	-1.592871	-2.416611	-1.252015
H	2.405670	-4.109441	-2.046341
H	-4.129587	1.933468	-0.004644

H	-3.514346	0.783994	-1.200993
H	-4.960925	0.047796	1.387323
H	-6.505077	1.138645	-0.255016
H	-5.990083	-0.008072	-1.504562
H	-6.885703	-0.583570	-0.091172
H	-5.028203	-2.265214	0.450714
H	-3.357937	-1.705160	0.633363
H	-4.124902	-1.717551	-0.971217

M062X energy = -1154.58689725 a.u.

(2*R*,6*R*,7*S*,8*S*)-**1**, Conf. C

C	3.148808	-2.661472	-0.224101
C	3.283250	-1.192550	-0.545228
C	3.533842	-0.207970	0.320386
C	3.932346	-0.444408	1.753601
C	0.733667	-1.983903	0.107822
C	1.813421	-2.950651	0.527746
C	1.907009	1.704261	0.466625
C	0.664239	1.211909	-0.337712
C	-0.259500	0.326420	0.526866
C	0.455303	-0.947079	0.905556
O	1.897498	3.139201	0.423873
C	0.851378	3.637295	-0.277073
C	0.035155	2.499880	-0.794399
O	0.669124	4.812852	-0.425913
C	-1.042228	2.730539	-1.538745
C	3.272572	1.229995	-0.057403
C	0.195516	-2.197596	-1.283724
O	3.130472	-3.438642	-1.413831
O	-1.450638	0.096418	-0.236029
C	-2.506540	-0.377926	0.463200
O	-2.486379	-0.543456	1.655807
C	-3.664932	-0.685503	-0.456503
C	-4.970654	-0.961984	0.287774
C	-5.482230	0.299690	0.983165
C	-6.017598	-1.511592	-0.680383
H	3.972242	-3.001554	0.421164
H	2.967193	-0.930358	-1.557176
H	4.163394	-1.492368	1.957575
H	3.134296	-0.142960	2.444554
H	1.996981	-2.855844	1.603040
H	1.552020	-3.993642	0.315092
H	4.812716	0.155909	2.009793
H	0.992303	0.608779	-1.193502
H	-0.544937	0.875718	1.430995
H	0.915341	-0.924765	1.892775
H	-1.650281	1.928743	-1.941080
H	-1.320428	3.759497	-1.750561
H	3.295660	1.366341	-1.144919
H	1.805547	1.431355	1.524219
H	4.028348	1.894343	0.377787
H	1.018519	-2.412326	-1.973657
H	-0.389041	-1.355503	-1.653908
H	-0.447164	-3.087558	-1.291592
H	3.946048	-3.251831	-1.900817
H	-3.782213	0.146847	-1.162485

H	-3.363766	-1.554096	-1.058405
H	-4.759272	-1.716817	1.054750
H	-4.749770	0.682782	1.698142
H	-5.694012	1.083738	0.245442
H	-6.410183	0.093212	1.525428
H	-6.956438	-1.723305	-0.159621
H	-5.678653	-2.436900	-1.157955
H	-6.232001	-0.782552	-1.471149

M062X energy = -1154.58651366 a.u.

(2*R*,6*R*,7*S*,8*S*)-**1**, Conf. D

C	1.848128	-3.319969	-0.363745
C	2.468454	-1.997343	-0.731081
C	3.109376	-1.168311	0.094509
C	3.516498	-1.542732	1.495988
C	-0.099612	-1.816039	0.247037
C	0.601917	-3.111895	0.566484
C	2.307878	1.199356	0.355172
C	0.880979	1.176206	-0.276322
C	-0.174055	0.679231	0.735537
C	0.066287	-0.772837	1.069688
O	2.796109	2.545148	0.245492
C	1.910893	3.379500	-0.348680
C	0.688958	2.603082	-0.712290
O	2.134399	4.543548	-0.528893
C	-0.326613	3.197364	-1.331413
C	3.344504	0.270157	-0.296258
C	-0.774557	-1.768455	-1.099061
O	1.492299	-3.940320	-1.589185
O	-1.468801	0.906205	0.161617
C	-2.494707	0.909047	1.046745
O	-2.339596	0.783746	2.234150
C	-3.820113	1.024190	0.339271
C	-4.138445	-0.257686	-0.451996
C	-4.049675	-1.489765	0.449439
C	-5.520406	-0.141435	-1.092059
H	2.565772	-3.954395	0.178152
H	2.212055	-1.656209	-1.734407
H	3.402910	-2.611728	1.691853
H	2.927141	-1.003931	2.248853
H	0.944131	-3.099649	1.606947
H	-0.058021	-3.982757	0.446373
H	4.565613	-1.277463	1.669692
H	0.870736	0.496687	-1.137482
H	-0.106921	1.282814	1.646644
H	0.566807	-0.943831	2.021415
H	-1.222787	2.660106	-1.619511
H	-0.254683	4.258258	-1.555754
H	3.294866	0.399217	-1.383680
H	2.248737	0.984870	1.429251
H	4.331536	0.613518	0.035619
H	-0.115800	-2.184853	-1.868287
H	-1.085442	-0.763232	-1.383280
H	-1.669891	-2.404705	-1.083143
H	1.096045	-4.799169	-1.387159
H	-4.582750	1.193527	1.105047

H	-3.799684	1.886078	-0.336934
H	-3.389808	-0.353026	-1.248664
H	-3.047261	-1.612838	0.875349
H	-4.756242	-1.405046	1.283499
H	-4.296140	-2.396978	-0.112053
H	-6.293472	-0.047287	-0.320582
H	-5.749924	-1.029143	-1.689520
H	-5.586948	0.734551	-1.745475

M062X energy = -1154.58629771 a.u.

(2*R*,6*R*,7*S*,8*S*)-**1**, Conf. E

C	3.229139	-2.599348	0.382675
C	3.450827	-1.103924	0.310959
C	3.276393	-0.302399	-0.745254
C	3.074781	-0.777877	-2.158044
C	0.808740	-1.900779	0.725728
C	1.727521	-2.964494	0.182232
C	1.911797	1.546395	0.300145
C	0.541367	1.425800	-0.434817
C	-0.328938	0.307355	0.172794
C	0.281484	-1.024042	-0.133193
O	2.040617	2.910896	0.727122
C	0.948910	3.657957	0.442205
C	-0.038448	2.807399	-0.286471
O	0.853407	4.813953	0.745377
C	-1.192642	3.322522	-0.699728
C	3.168045	1.193451	-0.508606
C	0.695891	-1.792966	2.222041
O	3.930235	-3.352055	-0.596599
O	-1.623735	0.412771	-0.445529
C	-2.633199	-0.174150	0.230130
O	-2.483810	-0.737671	1.286188
C	-3.949591	0.011233	-0.488401
C	-5.045059	-0.928406	0.015060
C	-6.396107	-0.519132	-0.569349
C	-4.717509	-2.380679	-0.331941
H	3.533313	-2.931352	1.386798
H	3.592348	-0.625218	1.282035
H	2.120840	-0.412020	-2.559347
H	3.107936	-1.865177	-2.236711
H	1.546657	-3.943825	0.639201
H	1.579373	-3.068076	-0.897527
H	3.857908	-0.360644	-2.803765
H	0.699590	1.193303	-1.496000
H	-0.444011	0.476469	1.247353
H	0.416305	-1.199885	-1.201493
H	-1.924865	2.741064	-1.247634
H	-1.405346	4.365481	-0.480527
H	3.150207	1.749093	-1.453922
H	1.898193	0.931928	1.209014
H	4.025272	1.561271	0.065752
H	1.598807	-1.340420	2.655847
H	0.599728	-2.790848	2.662860
H	-0.174934	-1.207452	2.524224
H	4.859976	-3.085976	-0.554121
H	-4.240688	1.061220	-0.344256

H	-3.781604	-0.116087	-1.564770
H	-5.082372	-0.834081	1.106837
H	-7.191488	-1.179212	-0.210009
H	-6.658660	0.508337	-0.296447
H	-6.379071	-0.583142	-1.664096
H	-5.491431	-3.054752	0.048009
H	-3.760869	-2.685249	0.101267
H	-4.665226	-2.511410	-1.420032

M062X energy = -1154.58625349 a.u.

(2*R*,6*R*,7*S*,8*S*)-**1**, Conf. F

C	3.785519	-1.939076	-0.296911
C	3.506720	-0.490728	-0.620701
C	3.489604	0.526439	0.243204
C	3.969930	0.412528	1.666577
C	1.288409	-1.940279	0.116814
C	2.603512	-2.573841	0.498413
C	1.407310	1.923435	0.445514
C	0.318056	1.103068	-0.311975
C	-0.291345	0.003706	0.584602
C	0.755504	-1.024347	0.933173
O	1.006746	3.301396	0.400208
C	-0.165940	3.487165	-0.250533
C	-0.662743	2.164398	-0.731096
O	-0.668902	4.566846	-0.388618
C	-1.799988	2.080241	-1.415683
C	2.832720	1.834409	-0.124556
C	0.789567	-2.288774	-1.261098
O	3.940028	-2.702176	-1.485646
O	-1.408777	-0.545158	-0.126459
C	-2.311343	-1.202795	0.634002
O	-2.203940	-1.327160	1.827571
C	-3.481936	-1.703142	-0.176433
C	-4.784800	-1.067176	0.333082
C	-4.757355	0.449019	0.139758
C	-5.988938	-1.688382	-0.370517
H	4.690579	-2.038010	0.320929
H	3.111687	-0.329237	-1.625870
H	3.135267	0.469037	2.377111
H	4.644931	1.241355	1.908721
H	2.788655	-2.424171	1.567189
H	2.628825	-3.650142	0.292686
H	4.498928	-0.524032	1.856879
H	0.764254	0.612026	-1.186085
H	-0.678564	0.461933	1.501537
H	1.215235	-0.881006	1.910127
H	-2.182480	1.136812	-1.787912
H	-2.364453	2.988923	-1.608157
H	2.781936	1.962141	-1.212137
H	1.419050	1.644792	1.506233
H	3.391559	2.684029	0.285473
H	1.612697	-2.238279	-1.981643
H	-0.035153	-1.658541	-1.592717
H	0.444193	-3.330543	-1.268572
H	4.656916	-2.304138	-2.000229
H	-3.330132	-1.479797	-1.237703

H	-3.527271	-2.791186	-0.055191
H	-4.845481	-1.283324	1.406785
H	-3.899119	0.909989	0.641805
H	-4.697626	0.698693	-0.927119
H	-5.665741	0.909631	0.539829
H	-6.923286	-1.258593	0.003565
H	-6.027697	-2.771409	-0.215623
H	-5.943718	-1.502120	-1.450173

M062X energy = -1154.58621433 a.u.

(2*R*,6*R*,7*S*,8*S*)-**1**, Conf. G

C	3.618107	-2.244917	0.439016
C	3.610402	-0.731747	0.417933
C	3.394306	0.072266	-0.628592
C	3.374259	-0.377815	-2.063754
C	1.101561	-1.932138	0.635627
C	2.205184	-2.826839	0.131395
C	1.696439	1.659195	0.359033
C	0.412302	1.354116	-0.473012
C	-0.314503	0.102025	0.058499
C	0.507456	-1.113052	-0.236724
O	1.585426	3.013563	0.822226
C	0.415913	3.596514	0.470081
C	-0.377404	2.629136	-0.344523
O	0.124689	4.715791	0.786400
C	-1.562685	2.979234	-0.835069
C	3.042863	1.525987	-0.365053
C	0.874018	-1.898023	2.122076
O	4.484002	-2.850711	-0.509436
O	-1.575551	0.032229	-0.629723
C	-2.524366	-0.710384	-0.021617
O	-2.359162	-1.254296	1.042405
C	-3.781717	-0.773727	-0.857513
C	-4.978218	-1.357741	-0.106984
C	-5.425080	-0.428408	1.021675
C	-6.127063	-1.621035	-1.079339
H	3.907097	-2.560388	1.452896
H	3.603727	-0.272141	1.408554
H	3.590817	-1.441893	-2.164386
H	4.121593	0.183905	-2.638405
H	2.147465	-3.836590	0.552921
H	2.143126	-2.916232	-0.958069
H	2.403799	-0.158767	-2.527499
H	0.672740	1.174037	-1.524067
H	-0.511806	0.221822	1.127567
H	0.742056	-1.228137	-1.295956
H	-2.157594	2.311967	-1.447516
H	-1.944966	3.972425	-0.614849
H	3.004620	2.103958	-1.296240
H	1.714280	1.022128	1.252179
H	3.793449	1.999265	0.277392
H	0.874653	-2.917070	2.523475
H	-0.081765	-1.436596	2.379162
H	1.682228	-1.356155	2.633012
H	5.361348	-2.458645	-0.392713
H	-4.003938	0.235527	-1.227748

H	-3.540483	-1.375511	-1.743960
H	-4.656469	-2.308144	0.335598
H	-6.275847	-0.857191	1.560324
H	-4.618885	-0.260055	1.739928
H	-5.741090	0.541079	0.616732
H	-6.986307	-2.053993	-0.558035
H	-5.831393	-2.310924	-1.876671
H	-6.458338	-0.686171	-1.547527

M062X energy = -1154.58617978 a.u.

(2*R*,6*R*,7*S*,8*S*)-**1**, Conf. H

C	2.991253	-2.625664	0.363792
C	3.248584	-1.154603	0.117160
C	2.941272	-0.442334	-0.971943
C	2.506842	-1.033193	-2.285263
C	0.685128	-1.784136	1.020395
C	1.465203	-2.933358	0.435689
C	1.836302	1.568622	0.089880
C	0.356537	1.452534	-0.392753
C	-0.433758	0.434022	0.451459
C	0.073622	-0.946959	0.176719
O	2.086754	2.959378	0.342771
C	0.988273	3.731951	0.174287
C	-0.142991	2.868076	-0.275829
O	0.989486	4.915388	0.365931
C	-1.339202	3.398354	-0.512653
C	2.927057	1.072473	-0.869642
C	0.807614	-1.554455	2.501901
O	3.499754	-3.494233	-0.638086
O	-1.808183	0.531539	0.040506
C	-2.704259	0.011738	0.910768
O	-2.403807	-0.393512	2.005390
C	-4.079782	-0.038984	0.297794
C	-4.112003	-1.009233	-0.896977
C	-3.620574	-2.396704	-0.482747
C	-5.522882	-1.074989	-1.477569
H	3.440746	-2.882797	1.334727
H	3.556394	-0.599343	1.005499
H	2.487029	-2.123263	-2.260369
H	3.195999	-0.717419	-3.078768
H	1.325354	-3.861994	1.000158
H	1.142754	-3.116826	-0.594372
H	1.516699	-0.656948	-2.572884
H	0.323798	1.124186	-1.439926
H	-0.366488	0.703220	1.509330
H	0.037447	-1.213343	-0.881049
H	-2.180151	2.805101	-0.851906
H	-1.475512	4.465478	-0.358662
H	2.775506	1.538390	-1.850688
H	1.950292	1.047710	1.048767
H	3.880025	1.446256	-0.479796
H	1.786408	-1.123172	2.755911
H	0.734814	-2.508199	3.035162
H	0.020975	-0.897271	2.878458
H	4.433051	-3.272130	-0.766632
H	-4.773895	-0.367178	1.077080

H	-4.368156	0.966455	-0.030078
H	-3.432490	-0.611525	-1.661022
H	-3.661307	-3.090055	-1.328631
H	-2.588141	-2.371356	-0.115881
H	-4.249479	-2.802297	0.318591
H	-5.553202	-1.730105	-2.353736
H	-5.878632	-0.085152	-1.781633
H	-6.225297	-1.473849	-0.736544

M062X energy = -1154.58610302 a.u.

(2*R*,6*R*,7*S*,8*S*)-**1**, Conf. I

C	1.948937	-3.311139	-0.344194
C	2.590989	-1.984619	-0.654115
C	3.167722	-1.160705	0.222249
C	3.472087	-1.543782	1.647027
C	-0.050585	-1.830164	0.147466
C	0.649376	-3.115854	0.510814
C	2.344257	1.203448	0.438298
C	0.965829	1.184007	-0.293505
C	-0.157747	0.671839	0.632710
C	0.070674	-0.781294	0.970449
O	2.837896	2.549908	0.373552
C	1.995906	3.388763	-0.274629
C	0.801560	2.615873	-0.726230
O	2.231308	4.553973	-0.430544
C	-0.172120	3.219018	-1.401657
C	3.427089	0.280268	-0.142349
C	-0.664377	-1.807208	-1.227978
O	1.666625	-3.911564	-1.598302
O	-1.402894	0.895628	-0.042196
C	-2.500334	0.825475	0.742200
O	-2.450486	0.644927	1.932493
C	-3.777355	0.965637	-0.050812
C	-4.744412	-0.175270	0.294007
C	-6.074988	0.019481	-0.428309
C	-4.119909	-1.527237	-0.052371
H	2.631945	-3.954817	0.230965
H	2.411659	-1.637376	-1.671817
H	3.352871	-2.615382	1.823884
H	2.824175	-1.016049	2.358721
H	0.930385	-3.099134	1.569566
H	0.008441	-3.995280	0.354778
H	4.503355	-1.272550	1.900279
H	1.015500	0.513349	-1.160632
H	-0.167235	1.267667	1.551615
H	0.532764	-0.947054	1.942373
H	-1.049935	2.688609	-1.751860
H	-0.083178	4.282421	-1.607468
H	3.455466	0.415301	-1.229817
H	2.207841	0.981017	1.503896
H	4.386847	0.624311	0.261039
H	0.079410	-2.096412	-1.978912
H	-1.094969	-0.840922	-1.489577
H	-1.460429	-2.560957	-1.284225
H	1.243523	-4.765560	-1.433655
H	-4.228127	1.931172	0.208160

H	-3.550889	0.973518	-1.122298
H	-4.911400	-0.136694	1.377034
H	-6.535161	0.978755	-0.169939
H	-5.931603	-0.004757	-1.515161
H	-6.780011	-0.775825	-0.167245
H	-4.798060	-2.347592	0.202608
H	-3.180456	-1.685378	0.490735
H	-3.907917	-1.586579	-1.127505

M062X energy = -1154.58597757 a.u.

(2*R*,6*R*,7*S*,8*S*)-**1**, Conf. J

C	2.880691	-2.769859	0.435171
C	3.226990	-1.309664	0.241734
C	3.048790	-0.563139	-0.853505
C	2.698541	-1.106671	-2.211551
C	0.574461	-1.826788	0.928130
C	1.340577	-3.003318	0.380196
C	1.957345	1.471126	0.168219
C	0.519722	1.437959	-0.437526
C	-0.387590	0.453298	0.326821
C	0.061094	-0.949849	0.060178
O	2.246810	2.840184	0.488350
C	1.207817	3.670680	0.239048
C	0.085379	2.875927	-0.341262
O	1.247108	4.847852	0.462983
C	-1.049475	3.473552	-0.692064
C	3.098153	0.948512	-0.715852
C	0.603039	-1.626668	2.418795
O	3.425514	-3.645365	-0.541083
O	-1.722635	0.641606	-0.174304
C	-2.705914	0.202402	0.638401
O	-2.506699	-0.252613	1.738103
C	-4.062737	0.293568	-0.012951
C	-4.646069	-1.118074	-0.195156
C	-6.037214	-1.033364	-0.817943
C	-3.711434	-1.984207	-1.040784
H	3.237409	-3.065871	1.433228
H	3.485022	-0.785805	1.164286
H	3.462074	-0.803580	-2.939262
H	1.751023	-0.683188	-2.569243
H	1.112856	-3.933312	0.913004
H	1.090964	-3.152476	-0.675402
H	2.632672	-2.195110	-2.215273
H	0.556612	1.121463	-1.487968
H	-0.379760	0.699630	1.392191
H	0.086306	-1.198317	-1.002172
H	-1.878901	2.932342	-1.131876
H	-1.145875	4.544065	-0.531346
H	3.045365	1.441481	-1.694087
H	1.968209	0.917440	1.115277
H	4.033645	1.268098	-0.243891
H	-0.172821	-0.934580	2.752612
H	1.582292	-1.252770	2.749360
H	0.443171	-2.583796	2.926435
H	4.375948	-3.468562	-0.590293
H	-4.713951	0.884838	0.639406

H	-3.980055	0.799927	-0.980051
H	-4.724860	-1.562716	0.804468
H	-6.480765	-2.028500	-0.920312
H	-6.711293	-0.421529	-0.209810
H	-5.983886	-0.586147	-1.817752
H	-4.133440	-2.983692	-1.184135
H	-2.729522	-2.099322	-0.567739
H	-3.562228	-1.535854	-2.030627

M062X energy = -1154.58594655 a.u.

(2*R*,6*R*,7*S*,8*S*)-**1**, Conf. K

C	2.106843	-3.233682	-0.238964
C	2.702219	-1.870946	-0.505161
C	3.154985	-1.010770	0.409172
C	3.352266	-1.370632	1.858409
C	0.003760	-1.851792	0.029193
C	0.710361	-3.117451	0.446815
C	2.169965	1.298387	0.534001
C	0.863953	1.192296	-0.314617
C	-0.299623	0.625447	0.528343
C	-0.032950	-0.818515	0.880040
O	2.585069	2.672128	0.501193
C	1.758061	3.454196	-0.232226
C	0.658009	2.607646	-0.780352
O	1.939094	4.629747	-0.382717
C	-0.280969	3.143915	-1.554233
C	3.355294	0.443055	0.057699
C	-0.439021	-1.815608	-1.409448
O	1.907606	-3.942508	-1.453890
O	-1.513229	0.802714	-0.216166
C	-2.642920	0.825360	0.528406
O	-2.637809	0.718910	1.729633
C	-3.879189	0.957783	-0.326769
C	-4.714083	-0.340933	-0.306232
C	-3.858744	-1.536569	-0.726467
C	-5.372884	-0.582556	1.051608
H	2.753778	-3.823131	0.427896
H	2.574537	-1.519754	-1.530720
H	4.330163	-1.020922	2.208190
H	3.294674	-2.447734	2.031916
H	0.854885	-3.119226	1.532250
H	0.153294	-4.020885	0.173662
H	2.599423	-0.892883	2.497983
H	1.029155	0.518275	-1.164114
H	-0.391306	1.214922	1.446307
H	0.314916	-0.980981	1.899029
H	-1.088141	2.557379	-1.977695
H	-0.236823	4.208386	-1.768528
H	3.470307	0.577992	-1.024187
H	1.955355	1.070566	1.585298
H	4.252644	0.846970	0.541295
H	-0.863246	-0.854703	-1.701803
H	-1.198789	-2.589375	-1.574610
H	0.396363	-2.072722	-2.069873
H	2.763818	-4.008173	-1.900853
H	-4.472679	1.786425	0.073387

H	-3.583786	1.199510	-1.351433
H	-5.506405	-0.202738	-1.052910
H	-3.401057	-1.374798	-1.708177
H	-3.051486	-1.708867	-0.002151
H	-4.462269	-2.448212	-0.776071
H	-6.005236	-1.475316	1.011826
H	-6.000047	0.266054	1.343376
H	-4.619894	-0.725750	1.830976

M062X energy = -1154.58591732 a.u.

(2*R*,6*R*,7*S*,8*S*)-**1**, Conf. L

C	-4.083995	-1.458867	-0.249511
C	-3.687212	0.000060	-0.188195
C	-3.157324	0.663347	0.844893
C	-3.092061	0.141431	2.254116
C	-1.606741	-1.774478	-0.732523
C	-2.841934	-2.392405	-0.127847
C	-1.225513	1.806314	-0.300877
C	0.020443	1.154155	0.376684
C	0.348778	-0.211169	-0.262650
C	-0.728049	-1.190874	0.087082
O	-0.821386	3.102352	-0.767813
C	0.485258	3.362686	-0.533856
C	1.083907	2.202572	0.190635
O	1.017406	4.383447	-0.869177
C	2.354882	2.239609	0.579143
C	-2.472253	1.991364	0.574842
C	-1.544320	-1.704136	-2.233669
O	-4.969454	-1.879309	0.778039
O	1.605975	-0.650312	0.284166
C	2.219411	-1.623079	-0.429617
O	1.799385	-2.031169	-1.483236
C	3.467271	-2.121080	0.259463
C	4.417911	-1.003878	0.708287
C	5.662234	-1.608959	1.356503
C	4.794983	-0.102661	-0.466939
H	-4.551381	-1.633439	-1.230226
H	-3.673572	0.499895	-1.159034
H	-2.052706	0.090468	2.603586
H	-3.554336	-0.841550	2.350409
H	-3.088484	-3.358024	-0.583380
H	-2.688350	-2.558344	0.943410
H	-3.608322	0.834746	2.930264
H	-0.170195	0.991431	1.445174
H	0.466833	-0.088851	-1.342986
H	-0.867666	-1.308269	1.162940
H	2.817786	1.429767	1.130714
H	2.949971	3.115991	0.336180
H	-2.184329	2.492696	1.506799
H	-1.508056	1.228579	-1.189227
H	-3.138457	2.672195	0.033923
H	-1.852512	-2.662591	-2.665131
H	-0.537148	-1.482666	-2.592662
H	-2.237920	-0.945084	-2.622205
H	-5.722793	-1.271455	0.778043
H	3.154564	-2.714273	1.128127

H	3.966383	-2.794345	-0.444188
H	3.891928	-0.404989	1.462526
H	6.339911	-0.824391	1.706962
H	5.401864	-2.240569	2.211974
H	6.209608	-2.226203	0.634644
H	5.468150	0.698588	-0.145189
H	3.912334	0.363086	-0.919199
H	5.307445	-0.682175	-1.243860

M062X energy = -1154.58585902 a.u.

(2*R*,6*R*,7*S*,8*S*)-**1**, Conf. M

C	-1.990560	-3.258219	-0.585764
C	-2.693745	-1.925154	-0.486296
C	-2.926607	-1.115732	0.553841
C	-2.758722	-1.435583	2.018236
C	0.079912	-1.800320	-0.611357
C	-0.567522	-3.039648	-1.182066
C	-2.263041	1.075872	-0.520859
C	-0.869453	1.142805	0.165303
C	0.257036	0.735167	-0.807117
C	0.045814	-0.668407	-1.324506
O	-2.698774	2.430603	-0.711566
C	-1.924301	3.319094	-0.040765
C	-0.784531	2.582388	0.584820
O	-2.164729	4.492755	0.000117
C	0.106124	3.212185	1.344287
C	-3.336892	0.324207	0.275336
C	0.565645	-1.918926	0.807223
O	-1.834766	-3.963345	0.634016
O	1.490069	0.893827	-0.090872
C	2.598145	0.958093	-0.867286
O	2.559198	0.936296	-2.070337
C	3.847847	0.997040	-0.025828
C	4.058493	-0.333560	0.719140
C	4.025667	-1.512524	-0.253643
C	5.375342	-0.292796	1.491902
H	-2.538560	-3.892758	-1.299621
H	-2.892359	-1.511156	-1.478416
H	-3.719723	-1.304870	2.531926
H	-2.065132	-0.724702	2.485815
H	-0.663890	-2.941343	-2.268093
H	0.006483	-3.949015	-0.971118
H	-2.388344	-2.441138	2.203462
H	-0.832195	0.463785	1.022892
H	0.269861	1.431353	-1.652874
H	-0.319073	-0.720883	-2.349101
H	0.941043	2.692426	1.801916
H	-0.002027	4.280521	1.510902
H	-3.498687	0.862249	1.217774
H	-2.180711	0.639007	-1.520105
H	-4.275613	0.380740	-0.286532
H	0.925504	-0.972184	1.211036
H	1.390700	-2.642102	0.850723
H	-0.227793	-2.321579	1.444990
H	-2.719667	-4.190397	0.954646
H	4.686467	1.189888	-0.701401

H	3.777317	1.820950	0.693184
H	3.235289	-0.448412	1.436038
H	4.190218	-2.456385	0.276263
H	3.066582	-1.580283	-0.779995
H	4.812223	-1.406230	-1.010120
H	5.522627	-1.216452	2.060218
H	5.402467	0.546671	2.194280
H	6.221104	-0.184680	0.802827

M062X energy = -1154.58571760 a.u.

(2*R*,6*R*,7*S*,8*S*)-**1**, Conf. N

C	2.697059	-2.866060	-0.374965
C	2.898173	-1.508198	-0.978696
C	3.145820	-0.396616	-0.279964
C	3.572716	-0.402373	1.169913
C	0.547849	-1.959854	0.636058
C	1.175967	-3.091703	-0.142791
C	1.846677	1.763732	-0.061858
C	0.335233	1.568411	-0.378317
C	-0.343895	0.444063	0.417382
C	0.116139	-0.900392	-0.057554
O	2.108564	3.156643	-0.309062
C	0.976647	3.894846	-0.220331
C	-0.190514	2.964490	-0.134424
O	0.977244	5.093460	-0.227726
C	-1.403899	3.455987	0.099286
C	2.896576	0.974432	-0.859262
C	0.573929	-2.080978	2.133669
O	3.100800	-3.921318	-1.230216
O	-1.757062	0.562106	0.139380
C	-2.575761	-0.099824	0.987794
O	-2.187705	-0.655491	1.984363
C	-3.998157	-0.075529	0.488661
C	-4.133741	-0.819554	-0.851789
C	-3.599272	-2.247356	-0.736166
C	-5.592348	-0.811898	-1.304388
H	3.208932	-2.941592	0.595488
H	2.579967	-1.427475	-2.019904
H	4.139436	-1.298122	1.432520
H	2.710057	-0.350665	1.846427
H	1.056018	-4.056402	0.362405
H	0.722415	-3.178925	-1.136163
H	4.202751	0.467985	1.385193
H	0.228776	1.338505	-1.449295
H	-0.190282	0.590990	1.491129
H	0.137157	-0.973435	-1.146465
H	-2.289890	2.837608	0.152770
H	-1.503238	4.529940	0.233862
H	2.593665	0.916216	-1.910674
H	2.000215	1.601923	1.013522
H	3.812785	1.577148	-0.810619
H	0.277679	-1.163321	2.642950
H	1.570878	-2.383278	2.478001
H	-0.125472	-2.864582	2.447208
H	4.036277	-3.788641	-1.437819
H	-4.619944	-0.548176	1.254847

H	-4.321581	0.965529	0.369529
H	-3.529816	-0.278314	-1.590605
H	-3.718804	-2.782326	-1.683414
H	-2.536129	-2.263087	-0.470243
H	-4.145824	-2.799921	0.037202
H	-5.701038	-1.297412	-2.279075
H	-5.981365	0.208097	-1.388356
H	-6.219541	-1.355214	-0.587880

M062X energy = -1154.58565988 a.u.

(2*R*,6*R*,7*S*,8*S*)-**1**, Conf. O

C	2.578757	-2.924079	0.485399
C	3.033105	-1.489992	0.322291
C	2.949743	-0.722700	-0.769545
C	2.616048	-1.226300	-2.147011
C	0.325816	-1.825830	0.903472
C	1.028956	-3.048830	0.372915
C	1.971057	1.378422	0.236430
C	0.558084	1.454475	-0.420032
C	-0.443071	0.524873	0.293933
C	-0.075805	-0.901313	0.026028
O	2.348834	2.720519	0.577573
C	1.381040	3.626169	0.303072
C	0.223153	2.918737	-0.319504
O	1.497933	4.795657	0.539581
C	-0.855630	3.599078	-0.695209
C	3.101754	0.780334	-0.612339
C	0.297119	-1.646913	2.396853
O	3.096287	-3.825977	-0.481710
O	-1.741068	0.808172	-0.256987
C	-2.785015	0.406453	0.501067
O	-2.656462	-0.075666	1.600531
C	-4.096570	0.585414	-0.220491
C	-4.583254	-0.755239	-0.816457
C	-3.518804	-1.380942	-1.719655
C	-5.017534	-1.733900	0.273931
H	2.877741	-3.253875	1.491766
H	3.291057	-0.994080	1.260234
H	1.723201	-0.723018	-2.539712
H	2.461414	-2.305529	-2.165000
H	0.717653	-3.964067	0.888590
H	0.808374	-3.173955	-0.692181
H	3.432950	-0.982242	-2.837951
H	0.612028	1.148830	-1.473074
H	-0.462663	0.757634	1.362361
H	-0.009195	-1.134845	-1.037853
H	-1.707490	3.122256	-1.165261
H	-0.880607	4.671965	-0.524204
H	3.119978	1.284352	-1.586275
H	1.908282	0.819984	1.178902
H	4.040349	1.027651	-0.104443
H	0.052958	-2.597320	2.883075
H	-0.446979	-0.909547	2.705367
H	1.282489	-1.341118	2.776385
H	4.058292	-3.719241	-0.491348
H	-4.829080	0.957883	0.501590

H	-3.971026	1.324374	-1.016673
H	-5.459199	-0.510808	-1.430687
H	-3.923671	-2.253807	-2.240813
H	-3.154524	-0.670267	-2.468888
H	-2.657591	-1.716955	-1.128519
H	-5.401285	-2.656514	-0.173059
H	-5.805664	-1.305557	0.901360
H	-4.175677	-1.991324	0.923519

M062X energy = -1154.58559931 a.u.

(2*R*,6*R*,7*S*,8*S*)-**1**, Conf. P

C	3.169281	-2.646326	-0.248824
C	3.303175	-1.178426	-0.555428
C	3.526120	-0.203090	0.326602
C	3.899134	-0.451232	1.764971
C	0.749448	-1.980834	0.116274
C	1.843057	-2.930691	0.537080
C	1.902454	1.709461	0.471401
C	0.664653	1.209861	-0.336460
C	-0.259053	0.324352	0.528254
C	0.459979	-0.945408	0.911893
O	1.886849	3.144429	0.424745
C	0.840999	3.636188	-0.280285
C	0.031928	2.493955	-0.798841
O	0.652656	4.810547	-0.431520
C	-1.042691	2.718495	-1.548983
C	3.271335	1.237489	-0.044674
C	0.216964	-2.199124	-1.276731
O	3.193471	-3.312773	-1.501466
O	-1.448906	0.089439	-0.235217
C	-2.503994	-0.387265	0.463163
O	-2.482740	-0.557783	1.655143
C	-3.663524	-0.690512	-0.456639
C	-4.969677	-0.965282	0.287548
C	-5.477446	0.296135	0.986222
C	-6.018571	-1.509310	-0.681630
H	4.007370	-2.996435	0.372541
H	3.036105	-0.919828	-1.580489
H	4.128577	-1.500937	1.962527
H	3.093931	-0.154174	2.449348
H	2.044071	-2.813862	1.607651
H	1.568388	-3.981348	0.366416
H	4.777831	0.144603	2.037216
H	0.998734	0.604859	-1.188732
H	-0.547389	0.874673	1.430788
H	0.917127	-0.917538	1.900071
H	-1.645210	1.913484	-1.953253
H	-1.323909	3.745866	-1.764512
H	3.303442	1.379426	-1.131143
H	1.797041	1.439934	1.529565
H	4.024480	1.898629	0.400075
H	1.045703	-2.363222	-1.973875
H	-0.404985	-1.377911	-1.631760
H	-0.388182	-3.115324	-1.295974
H	3.107184	-4.262202	-1.337931
H	-3.779060	0.143488	-1.160903

H	-3.364809	-1.558671	-1.060459
H	-4.760175	-1.722610	1.052600
H	-4.743937	0.675238	1.702238
H	-5.686757	1.082752	0.250565
H	-6.406077	0.091041	1.527835
H	-6.957795	-1.719769	-0.161069
H	-5.682531	-2.434211	-1.162089
H	-6.231218	-0.777433	-1.470214

M062X energy = -1154.58552565 a.u.

(2*R*,6*R*,7*S*,8*S*)-**1**, Conf. Q

C	3.770605	-1.836011	-0.264721
C	3.472101	-0.379143	-0.526297
C	3.405370	0.595619	0.383192
C	3.830091	0.420015	1.817885
C	1.257275	-1.911554	0.025687
C	2.566848	-2.538667	0.435747
C	1.281586	1.924809	0.556082
C	0.263323	1.115339	-0.304794
C	-0.383223	-0.025653	0.510760
C	0.666560	-1.051002	0.861851
O	0.833092	3.288597	0.570511
C	-0.289970	3.480239	-0.162317
C	-0.697074	2.176882	-0.763314
O	-0.817307	4.550985	-0.276131
C	-1.742512	2.102513	-1.581354
C	2.735182	1.907715	0.054128
C	0.829605	-2.196157	-1.390944
O	4.004118	-2.530083	-1.482788
O	-1.454821	-0.561182	-0.277254
C	-2.307167	-1.372118	0.392357
O	-2.201404	-1.600919	1.571138
C	-3.402350	-1.900883	-0.501836
C	-4.748567	-1.191549	-0.240082
C	-5.277725	-1.463319	1.168071
C	-4.643593	0.309106	-0.511568
H	4.645997	-1.947659	0.392271
H	3.111776	-0.176347	-1.537301
H	2.965027	0.416956	2.493669
H	4.471072	1.251751	2.131374
H	2.697268	-2.446075	1.519122
H	2.626759	-3.601089	0.172779
H	4.376106	-0.511729	1.981715
H	0.779962	0.658506	-1.157237
H	-0.820709	0.385953	1.427532
H	1.077263	-0.948755	1.865324
H	-2.042895	1.170558	-2.046668
H	-2.314154	3.002678	-1.791586
H	2.734998	2.095510	-1.025972
H	1.252812	1.587208	1.598941
H	3.250288	2.745324	0.538782
H	1.694464	-2.144873	-2.060453
H	0.044480	-1.529070	-1.746847
H	0.452013	-3.224804	-1.456928
H	4.736534	-2.089350	-1.937441
H	-3.108385	-1.774721	-1.547809

H	-3.505419	-2.969838	-0.290177
H	-5.457264	-1.620228	-0.960268
H	-5.360684	-2.537709	1.360732
H	-4.612173	-1.041645	1.926323
H	-6.269149	-1.016342	1.292034
H	-5.611468	0.797527	-0.363101
H	-4.319115	0.506701	-1.538754
H	-3.923042	0.785293	0.164937

M062X energy = -1154.58547181 a.u.

(2*R*,6*R*,7*S*,8*S*)-**1**, Conf. R

C	3.502540	-2.410770	-0.254717
C	3.554755	-0.979671	-0.698740
C	3.397840	0.070211	0.112564
C	3.464091	-0.025922	1.619084
C	1.038930	-2.031817	0.264101
C	2.045776	-2.932326	-0.412989
C	1.680226	1.930491	0.159073
C	0.339693	1.500376	-0.502278
C	-0.247855	0.178456	0.010361
C	0.574415	-0.990872	-0.435369
O	1.705500	3.365445	0.049192
C	0.453129	3.870860	-0.055367
C	-0.498465	2.734819	-0.256581
O	0.215424	5.044146	0.004962
C	-1.807806	2.957485	-0.188489
C	3.013233	1.429945	-0.417135
C	0.734980	-2.331716	1.705156
O	4.291630	-3.272383	-1.056856
O	-1.554452	0.080520	-0.603215
C	-2.413041	-0.780997	-0.023202
O	-2.167774	-1.383136	0.993177
C	-3.693865	-0.895680	-0.817584
C	-4.870978	-1.384480	0.026336
C	-5.230766	-0.356727	1.099408
C	-6.072301	-1.682825	-0.868722
H	3.790165	-2.503653	0.802854
H	3.475834	-0.840089	-1.778562
H	4.123806	-0.828364	1.955767
H	2.473488	-0.211463	2.054072
H	2.014122	-3.954193	-0.019074
H	1.855399	-2.986256	-1.490388
H	3.833771	0.913287	2.045765
H	0.503780	1.401164	-1.586233
H	-0.379119	0.215527	1.096629
H	0.861973	-0.925737	-1.486017
H	-2.549676	2.182279	-0.328225
H	-2.143024	3.971502	0.013125
H	2.966426	1.435019	-1.511700
H	1.628353	1.697163	1.231290
H	3.758201	2.176997	-0.113122
H	0.171568	-3.269830	1.769210
H	0.131486	-1.561364	2.186558
H	1.662772	-2.476168	2.273168
H	5.203165	-2.950160	-1.022467
H	-3.914383	0.072079	-1.284437

H	-3.489493	-1.593992	-1.640700
H	-4.554681	-2.308170	0.525280
H	-6.066760	-0.708237	1.711928
H	-4.383737	-0.164704	1.764381
H	-5.532457	0.591443	0.636445
H	-6.918060	-2.046892	-0.277353
H	-5.834689	-2.440568	-1.622636
H	-6.398044	-0.776384	-1.393109

M062X energy = -1154.58541701 a.u.

(2*R*,6*R*,7*S*,8*S*)-**1**, Conf. S

C	2.813497	-2.958320	-0.337518
C	3.162022	-1.571734	-0.790206
C	3.304244	-0.521470	0.023426
C	3.443865	-0.641714	1.522996
C	0.537882	-2.034693	0.321029
C	1.270369	-3.137747	-0.406646
C	2.047932	1.671822	0.147073
C	0.622643	1.553783	-0.469238
C	-0.225935	0.412547	0.113187
C	0.269491	-0.918434	-0.364667
O	2.389840	3.066305	0.054713
C	1.283389	3.841133	-0.036056
C	0.098360	2.954612	-0.249448
O	1.316777	5.037201	0.037270
C	-1.121541	3.484441	-0.247455
C	3.211996	0.895275	-0.487895
C	0.283027	-2.253414	1.785895
O	3.341118	-3.968469	-1.180044
O	-1.564772	0.615095	-0.395339
C	-2.547641	-0.016239	0.277189
O	-2.358411	-0.665400	1.276224
C	-3.894253	0.234164	-0.362900
C	-4.976431	-0.727113	0.129163
C	-6.348667	-0.270351	-0.363268
C	-4.681855	-2.156488	-0.326376
H	3.134544	-3.121596	0.701575
H	3.050790	-1.406774	-1.863680
H	4.055020	0.178851	1.915581
H	3.912377	-1.581205	1.823776
H	1.032753	-4.127935	-0.002275
H	1.009228	-3.141606	-1.470341
H	2.469242	-0.586186	2.024327
H	0.722984	1.385486	-1.551943
H	-0.259967	0.483539	1.204926
H	0.487623	-0.924477	-1.433814
H	-2.017103	2.903672	-0.422814
H	-1.214336	4.552876	-0.070841
H	3.120411	0.922845	-1.579333
H	1.984467	1.436643	1.218033
H	4.116957	1.454000	-0.216161
H	1.192152	-2.609464	2.286555
H	-0.477170	-3.033379	1.910182
H	-0.085315	-1.362071	2.294842
H	4.302041	-3.859066	-1.201565
H	-4.167089	1.274321	-0.135049

H	-3.778309	0.179213	-1.452072
H	-4.961122	-0.704198	1.225395
H	-7.133715	-0.943624	-0.005748
H	-6.585433	0.740943	-0.016361
H	-6.385111	-0.268253	-1.459308
H	-3.709236	-2.496563	0.039296
H	-4.682868	-2.217011	-1.421779
H	-5.444584	-2.847205	0.046293

M062X energy = -1154.58538037 a.u.

(2*R*,6*R*,7*S*,8*S*)-**1**, Conf. T

C	-2.108404	-3.219789	-0.598231
C	-2.778369	-1.866673	-0.559610
C	-3.059197	-1.039587	0.454275
C	-3.006454	-1.347529	1.929856
C	0.000622	-1.822356	-0.515050
C	-0.649297	-3.046497	-1.115101
C	-2.263328	1.119539	-0.597036
C	-0.912867	1.147475	0.174192
C	0.261036	0.705322	-0.724718
C	0.048358	-0.698699	-1.240494
O	-2.644502	2.486103	-0.818685
C	-1.889829	3.353075	-0.099258
C	-0.814275	2.585161	0.598212
O	-2.098546	4.533271	-0.074132
C	0.043147	3.191163	1.413363
C	-3.407301	0.408108	0.136009
C	0.386965	-1.940325	0.934029
O	-2.041030	-3.906874	0.639534
O	1.449158	0.843958	0.068038
C	2.605449	0.845645	-0.630146
O	2.650037	0.773462	-1.832201
C	3.814879	0.899283	0.270350
C	4.713597	-0.320642	0.015410
C	5.973493	-0.237180	0.873292
C	3.945566	-1.615620	0.281502
H	-2.631461	-3.851824	-1.332807
H	-2.898090	-1.459552	-1.566874
H	-3.997304	-1.181986	2.371759
H	-2.326972	-0.652307	2.439786
H	-0.683221	-2.951931	-2.205224
H	-0.113862	-3.970380	-0.868862
H	-2.682094	-2.361694	2.151047
H	-0.946149	0.468265	1.032322
H	0.344037	1.392515	-1.574057
H	-0.249199	-0.752314	-2.286567
H	0.834293	2.650201	1.921257
H	-0.046277	4.262281	1.573342
H	-3.611447	0.961401	1.061272
H	-2.135838	0.675533	-1.588376
H	-4.307096	0.486343	-0.484074
H	-0.484808	-2.219080	1.535134
H	0.835798	-1.027801	1.327844
H	1.110991	-2.756049	1.052677
H	-2.948032	-4.113017	0.908435
H	4.362450	1.820724	0.042630

H	3.499448	0.934274	1.318220
H	4.995774	-0.294476	-1.044111
H	6.635597	-1.086233	0.677102
H	6.533069	0.682582	0.674322
H	5.715788	-0.252509	1.938997
H	4.579690	-2.489437	0.101647
H	3.064997	-1.698971	-0.366764
H	3.606238	-1.653171	1.324414

M062X energy = -1154.58534057 a.u.

(2*R*,6*R*,7*S*,8*S*)-**1**, Conf. U

C	1.579529	-3.511850	-0.271713
C	2.283805	-2.308099	-0.823178
C	2.822250	-1.338064	-0.078508
C	3.073398	-1.471433	1.405746
C	-0.234292	-1.924813	0.543534
C	0.055277	-3.212848	-0.191391
C	2.312977	1.137431	0.055272
C	0.864359	1.455671	-0.415548
C	-0.230949	0.636736	0.282542
C	-0.199941	-0.792099	-0.167221
O	3.050905	2.356987	-0.139944
C	2.229720	3.433825	-0.154020
C	0.815522	2.950507	-0.194917
O	2.633858	4.562267	-0.144216
C	-0.179139	3.822344	-0.057168
C	3.111705	0.029056	-0.648125
C	-0.416985	-2.026682	2.031546
O	1.688523	-4.651537	-1.107001
O	-1.485803	1.213888	-0.144162
C	-2.557511	0.907918	0.618566
O	-2.478114	0.305388	1.661497
C	-3.843523	1.365814	-0.021057
C	-4.552690	0.191538	-0.734348
C	-3.647327	-0.454073	-1.784568
C	-5.062413	-0.851312	0.260316
H	1.940053	-3.744346	0.741041
H	2.116773	-2.136474	-1.888250
H	3.930040	-0.857146	1.704700
H	3.282901	-2.502186	1.699672
H	-0.428088	-4.074771	0.281537
H	-0.302152	-3.158489	-1.225652
H	2.211215	-1.132159	1.993714
H	0.797701	1.259573	-1.496382
H	-0.152347	0.742409	1.368982
H	-0.083563	-0.885377	-1.248564
H	-1.222516	3.538146	-0.090866
H	0.075235	4.868521	0.091424
H	2.918018	0.063662	-1.725940
H	2.294238	0.950971	1.137653
H	4.167154	0.286866	-0.491227
H	0.388689	-2.622835	2.478526
H	-1.357210	-2.547134	2.248458
H	-0.465640	-1.054882	2.523930
H	2.629854	-4.845486	-1.216689
H	-4.488997	1.759263	0.769669

H	-3.628893	2.161051	-0.740586
H	-5.418104	0.632041	-1.245799
H	-4.204155	-1.196965	-2.364079
H	-3.238551	0.287559	-2.478734
H	-2.804264	-0.968520	-1.306780
H	-5.618556	-1.635298	-0.263415
H	-5.726126	-0.400995	1.005267
H	-4.230291	-1.320337	0.794432

M062X energy = -1154.58533480 a.u.

(2*R*,6*R*,7*S*,8*S*)-**1**, Conf. V

C	2.577309	-3.021758	-0.245558
C	2.979172	-1.594043	-0.527791
C	3.371367	-0.688450	0.371112
C	3.679490	-1.026835	1.806503
C	0.310687	-1.934099	0.038683
C	1.192307	-3.080565	0.468667
C	2.110013	1.483874	0.537030
C	0.821792	1.252559	-0.311667
C	-0.274030	0.508266	0.481322
C	0.201678	-0.874434	0.847315
O	2.366880	2.896765	0.537982
C	1.441300	3.600544	-0.155937
C	0.441257	2.649221	-0.723461
O	1.478538	4.794021	-0.263602
C	-0.567091	3.097529	-1.465582
C	3.380366	0.781758	0.029359
C	-0.235920	-2.038029	-1.362814
O	2.454444	-3.762239	-1.452447
O	-1.439279	0.497286	-0.352622
C	-2.595224	0.142731	0.254296
O	-2.666353	-0.096520	1.432771
C	-3.720730	0.113999	-0.755960
C	-5.046793	-0.458641	-0.245071
C	-4.896561	-1.912836	0.206477
C	-5.668789	0.408106	0.851694
H	3.309023	-3.515031	0.411392
H	2.743740	-1.259051	-1.540444
H	3.739767	-2.103702	1.979578
H	2.915417	-0.626958	2.485618
H	1.362194	-3.031594	1.549313
H	0.757075	-4.058847	0.233940
H	4.632983	-0.578813	2.108526
H	1.063029	0.639268	-1.189112
H	-0.514758	1.073291	1.389093
H	0.636437	-0.946865	1.843551
H	-1.303669	2.433401	-1.902544
H	-0.653861	4.166968	-1.638377
H	3.456970	0.938593	-1.052894
H	1.930951	1.207182	1.583167
H	4.231268	1.287755	0.500436
H	-0.553511	-1.079667	-1.773915
H	-1.106939	-2.707353	-1.364325
H	0.508814	-2.495576	-2.021267
H	3.300272	-3.705362	-1.919772
H	-3.855531	1.144883	-1.110667

H	-3.359798	-0.457370	-1.620283
H	-5.724679	-0.443463	-1.109156
H	-5.873010	-2.336547	0.461498
H	-4.459721	-2.530806	-0.586329
H	-4.254565	-1.978787	1.089178
H	-6.658563	0.026533	1.121970
H	-5.789492	1.444028	0.515918
H	-5.043282	0.406006	1.747557

M062X energy = -1154.58526135 a.u.

(2*R*,6*R*,7*S*,8*S*)-**1**, Conf. W

C	3.801225	-1.834490	-0.333680
C	3.480686	-0.392152	-0.647611
C	3.450942	0.621519	0.220318
C	3.952723	0.512661	1.636672
C	1.313390	-1.906592	0.128652
C	2.652716	-2.503769	0.482493
C	1.332997	1.945957	0.455364
C	0.275221	1.081462	-0.296771
C	-0.311486	-0.010304	0.625895
C	0.763931	-1.015313	0.960826
O	0.868845	3.304359	0.423219
C	-0.292517	3.446582	-0.259131
C	-0.717202	2.108472	-0.765173
O	-0.836388	4.505159	-0.404466
C	-1.797939	1.983900	-1.529649
C	2.753844	1.913214	-0.131511
C	0.799842	-2.256868	-1.243563
O	3.951363	-2.589370	-1.528292
O	-1.440344	-0.580841	-0.047597
C	-2.247687	-1.342367	0.728697
O	-2.046783	-1.528387	1.901557
C	-3.435649	-1.840298	-0.052821
C	-4.494764	-0.732657	-0.215932
C	-5.652494	-1.248480	-1.068112
C	-4.992175	-0.241828	1.143607
H	4.720879	-1.912027	0.265431
H	3.070442	-0.236075	-1.647481
H	4.514431	-0.407650	1.812403
H	3.125541	0.535145	2.357881
H	2.853731	-2.352238	1.548185
H	2.704850	-3.578372	0.272829
H	4.603021	1.361233	1.877666
H	0.746635	0.577363	-1.148876
H	-0.679553	0.454819	1.547700
H	1.228059	-0.868525	1.935036
H	-2.110914	1.026849	-1.931920
H	-2.383126	2.869173	-1.763517
H	2.686656	2.046919	-1.217581
H	1.369858	1.664415	1.514355
H	3.290220	2.776595	0.279506
H	1.603480	-2.166741	-1.982125
H	-0.055674	-1.654332	-1.548960
H	0.493371	-3.310751	-1.256576
H	4.647822	-2.171881	-2.055367
H	-3.101925	-2.186203	-1.036870

H	-3.863372	-2.684571	0.496327
H	-4.019555	0.108648	-0.737605
H	-5.308983	-1.584592	-2.051782
H	-6.142905	-2.095217	-0.573583
H	-6.404640	-0.467910	-1.218068
H	-5.448497	-1.066913	1.703157
H	-5.746780	0.540615	1.018106
H	-4.180233	0.162961	1.754947

M062X energy = -1154.58521966 a.u.

(2*R*,6*R*,7*S*,8*S*)-**1**, Conf. X

C	-3.955418	-1.630295	-0.446734
C	-3.617637	-0.156968	-0.521014
C	-3.288708	0.661372	0.484104
C	-3.441880	0.328055	1.943094
C	-1.425540	-1.881470	-0.495263
C	-2.722238	-2.476490	-0.008527
C	-1.229587	1.745994	-0.482984
C	-0.098726	1.239468	0.463729
C	0.377725	-0.167662	0.046712
C	-0.701913	-1.155993	0.361972
O	-0.784257	2.990458	-1.042805
C	0.453643	3.343676	-0.623686
C	0.945316	2.314571	0.339098
O	1.007160	4.340528	-0.994228
C	2.115952	2.462713	0.951498
C	-2.608241	1.977886	0.150815
C	-1.129063	-2.004681	-1.964865
O	-4.982182	-1.961098	0.476708
O	1.566078	-0.454665	0.805508
C	2.323013	-1.459784	0.313611
O	2.050138	-2.051764	-0.702783
C	3.549836	-1.713472	1.155889
C	4.838900	-1.237093	0.452427
C	5.128517	-2.025772	-0.824574
C	4.791822	0.265402	0.174687
H	-4.252891	-1.952802	-1.455905
H	-3.457812	0.213391	-1.535823
H	-3.891497	-0.653714	2.094833
H	-4.076284	1.079209	2.430477
H	-2.865301	-3.503977	-0.361617
H	-2.736829	-2.495025	1.086118
H	-2.471986	0.367206	2.455966
H	-0.462070	1.181519	1.497479
H	0.641089	-0.165219	-1.014950
H	-1.003033	-1.143965	1.410709
H	2.481691	1.744206	1.675818
H	2.725336	3.332039	0.718467
H	-2.491298	2.606031	1.042227
H	-1.345319	1.048868	-1.321789
H	-3.197064	2.551163	-0.573502
H	-0.082951	-1.788967	-2.190181
H	-1.768901	-1.335067	-2.556648
H	-1.343014	-3.024401	-2.302728
H	-5.743113	-1.396302	0.278932
H	3.436345	-1.208204	2.119054

H	3.606157	-2.792979	1.329792
H	5.653435	-1.427729	1.163151
H	6.095993	-1.726291	-1.239784
H	5.158588	-3.102193	-0.628140
H	4.358832	-1.848179	-1.580217
H	5.719568	0.600538	-0.299166
H	4.658107	0.838573	1.098266
H	3.964097	0.517857	-0.499988

M062X energy = -1154.58518106 a.u.

(2*R*,6*R*,7*S*,8*S*)-**1**, Conf. Y

C	2.092844	-3.242070	-0.263599
C	2.698761	-1.886446	-0.517210
C	3.140319	-1.033551	0.408103
C	3.318598	-1.400026	1.858288
C	-0.003344	-1.850052	0.045024
C	0.710032	-3.110967	0.464079
C	2.182064	1.283837	0.537124
C	0.876318	1.185033	-0.312804
C	-0.292611	0.631228	0.531456
C	-0.035597	-0.812781	0.891132
O	2.605030	2.655361	0.503924
C	1.784952	3.441117	-0.232481
C	0.681448	2.600324	-0.783150
O	1.972325	4.615661	-0.383746
C	-0.250127	3.140663	-1.563039
C	3.360766	0.418856	0.063418
C	-0.447384	-1.816426	-1.393429
O	1.958973	-3.853934	-1.536522
O	-1.504146	0.813234	-0.215420
C	-2.634952	0.840850	0.526773
O	-2.632879	0.732407	1.727889
C	-3.868801	0.980340	-0.330925
C	-4.713415	-0.311892	-0.308230
C	-3.866079	-1.515466	-0.722549
C	-5.377417	-0.544243	1.048745
H	2.748619	-3.852740	0.375552
H	2.609631	-1.550631	-1.550296
H	4.299709	-1.066211	2.214834
H	3.247539	-2.477317	2.026539
H	0.884184	-3.097129	1.545655
H	0.125282	-4.013569	0.237111
H	2.569906	-0.914386	2.496738
H	1.037438	0.506613	-1.159622
H	-0.381460	1.225639	1.446477
H	0.314325	-0.970733	1.910012
H	-1.058936	2.558601	-1.989583
H	-0.197489	4.204180	-1.780144
H	3.483338	0.556124	-1.017221
H	1.964728	1.058815	1.588532
H	4.259979	0.810862	0.553397
H	-0.888362	-0.861335	-1.679110
H	-1.197564	-2.599597	-1.562022
H	0.392143	-2.053411	-2.056154
H	1.570087	-4.730468	-1.409882
H	-4.456831	1.814532	0.065785

H	-3.569728	1.217396	-1.355600
H	-5.502850	-0.170484	-1.057325
H	-3.406037	-1.360983	-1.704356
H	-3.060933	-1.690254	0.003586
H	-4.476441	-2.422762	-0.769140
H	-6.016705	-1.432138	1.010231
H	-5.998769	0.310073	1.336175
H	-4.627467	-0.690495	1.830478

M062X energy = -1154.58503093 a.u.

(2*R*,6*R*,7*S*,8*S*)-**1**, Conf. Z

C	-2.066557	-3.208732	-0.562047
C	-2.727652	-1.859315	-0.475182
C	-2.979583	-1.051191	0.560426
C	-2.905934	-1.396543	2.025192
C	0.035204	-1.806246	-0.652964
C	-0.661438	-3.017665	-1.224172
C	-2.229478	1.113619	-0.529955
C	-0.833922	1.134639	0.155083
C	0.280893	0.722544	-0.829074
C	0.040687	-0.671103	-1.362041
O	-2.615825	2.483654	-0.723590
C	-1.823883	3.341812	-0.034876
C	-0.715593	2.564511	0.598750
O	-2.027823	4.522155	0.013721
C	0.178896	3.157101	1.383085
C	-3.323919	0.405762	0.276204
C	0.498404	-1.933680	0.773291
O	-1.963301	-3.823899	0.707549
O	1.520659	0.853578	-0.118958
C	2.626688	0.898662	-0.898984
O	2.583544	0.875028	-2.101789
C	3.878600	0.926372	-0.059343
C	4.037479	-0.372037	0.750752
C	3.963857	-1.593910	-0.165484
C	5.350892	-0.344052	1.529678
H	-2.659332	-3.850984	-1.232831
H	-2.864753	-1.435204	-1.472296
H	-2.144228	-0.782458	2.525151
H	-2.690787	-2.446936	2.203445
H	-0.801765	-2.895530	-2.303641
H	-0.084916	-3.941071	-1.071384
H	-3.861595	-1.142018	2.500451
H	-0.811976	0.438122	0.999843
H	0.299817	1.428301	-1.666812
H	-0.318148	-0.706246	-2.389344
H	0.991107	2.607324	1.846697
H	0.097483	4.225271	1.565186
H	-3.450331	0.950954	1.220092
H	-2.165077	0.673645	-1.529004
H	-4.267569	0.503734	-0.272137
H	-0.349402	-2.173079	1.424497
H	0.997496	-1.032627	1.130713
H	1.203501	-2.770658	0.859976
H	-1.531105	-4.680463	0.583058
H	4.722970	1.055163	-0.742987

H	3.840219	1.787687	0.617295
H	3.207082	-0.421511	1.466789
H	4.077968	-2.518009	0.410652
H	3.009085	-1.643023	-0.702351
H	4.764861	-1.559311	-0.913535
H	5.460263	-1.244630	2.142058
H	5.405211	0.525958	2.192133
H	6.204037	-0.299820	0.842790

M062X energy = -1154.58492330 a.u.

(2*S*,6*R*,7*S*,8*S*)-**1**, Conf. A

C	-3.190833	-2.678074	0.105418
C	-3.437235	-1.189372	0.047449
C	-3.247208	-0.292834	1.017556
C	-2.994720	-0.651302	2.455787
C	-0.809398	-1.968416	-0.418866
C	-1.674382	-2.999512	0.260349
C	-1.961574	1.486432	-0.237218
C	-0.557115	1.452848	0.440624
C	0.305041	0.298437	-0.107215
C	-0.263456	-1.011852	0.338178
O	-2.141507	2.809150	-0.765940
C	-1.052854	3.595434	-0.599230
C	-0.012030	2.824639	0.143568
O	-0.996979	4.723781	-1.000798
C	1.154076	3.389859	0.442264
C	-3.172570	1.178585	0.653776
C	-0.778423	-1.989041	-1.922092
O	-3.603675	-3.295158	-1.104945
O	1.625049	0.475748	0.439178
C	2.614736	-0.143091	-0.237707
O	2.430868	-0.778530	-1.246092
C	3.956737	0.103012	0.412803
C	5.045208	-0.848296	-0.084128
C	6.412715	-0.392934	0.422529
C	4.753019	-2.284222	0.351728
H	-3.715472	-3.137825	0.956904
H	-3.604021	-0.822238	-0.968164
H	-3.018537	-1.729580	2.631165
H	-2.021488	-0.272907	2.793001
H	-1.526768	-4.003903	-0.151389
H	-1.436620	-3.028915	1.328904
H	-3.750124	-0.184689	3.099596
H	-0.662824	1.313089	1.524617
H	0.368716	0.374586	-1.196351
H	-0.341501	-1.099764	1.423394
H	1.928171	2.868609	0.992939
H	1.333414	4.412821	0.121946
H	-3.124662	1.810275	1.549152
H	-1.973000	0.800450	-1.093441
H	-4.061421	1.485507	0.091829
H	0.049623	-1.398805	-2.320262
H	-1.726033	-1.623901	-2.338445
H	-0.668348	-3.018578	-2.276772
H	-4.538877	-3.085930	-1.241118

H	4.224329	1.147275	0.199181
H	3.835207	0.033531	1.500738
H	5.036589	-0.812005	-1.180033
H	7.202506	-1.058994	0.061885
H	6.648094	0.623451	0.089885
H	6.442154	-0.404463	1.518816
H	5.521667	-2.967362	-0.022785
H	3.785078	-2.623793	-0.026307
H	4.745710	-2.357678	1.446459

M062X energy = -1154.58742432 a.u.

(2*S*,6*R*,7*S*,8*S*)-**1**, Conf. B

C	-4.049474	-1.680942	0.039216
C	-3.733819	-0.210114	-0.092952
C	-3.317943	0.625960	0.860672
C	-3.327107	0.297637	2.328090
C	-1.542124	-1.906124	-0.293384
C	-2.768380	-2.515194	0.338713
C	-1.376931	1.730334	-0.319066
C	-0.141669	1.239307	0.496465
C	0.289822	-0.172822	0.051680
C	-0.739126	-1.169928	0.480691
O	-1.015031	2.985595	-0.914266
C	0.264965	3.342331	-0.656198
C	0.883199	2.307655	0.224327
O	0.761403	4.344687	-1.087918
C	2.137873	2.440146	0.645123
C	-2.683858	1.944181	0.456129
C	-1.418730	-2.028029	-1.786418
O	-4.574737	-2.185494	-1.179973
O	1.546757	-0.444810	0.696773
C	2.317374	-1.366968	0.084779
O	2.003108	-1.922815	-0.938762
C	3.630155	-1.581828	0.798626
C	4.803730	-1.298381	-0.150769
C	4.800118	0.165975	-0.589858
C	6.126984	-1.667424	0.515208
H	-4.763268	-1.864649	0.856675
H	-3.673505	0.122878	-1.132002
H	-3.889456	1.058333	2.882744
H	-2.309617	0.300304	2.739494
H	-2.965643	-3.529556	-0.025250
H	-2.634185	-2.560731	1.424713
H	-3.774893	-0.676795	2.538496
H	-0.379176	1.202388	1.567562
H	0.450444	-0.179916	-1.029919
H	-0.917776	-1.165757	1.557624
H	2.606178	1.715487	1.301172
H	2.710764	3.305543	0.322434
H	-2.480159	2.575218	1.329812
H	-1.569860	1.033159	-1.143798
H	-3.350644	2.512195	-0.201845
H	-0.428960	-1.736464	-2.143629
H	-2.188150	-1.430121	-2.291020
H	-1.598080	-3.065854	-2.086100
H	-5.361955	-1.667540	-1.401804

H	3.683638	-0.943247	1.686604
H	3.660074	-2.627471	1.125785
H	4.657604	-1.930104	-1.035455
H	5.609066	0.362939	-1.299938
H	3.856375	0.444441	-1.072554
H	4.945812	0.825611	0.274984
H	6.967468	-1.487048	-0.162160
H	6.147322	-2.722088	0.808068
H	6.288229	-1.063095	1.416014

M062X energy = -1154.58732939 a.u.

(2*S*,6*R*,7*S*,8*S*)-**1**, Conf. C

C	-3.031127	-2.623045	0.183368
C	-3.254412	-1.132132	0.274069
C	-2.908214	-0.312030	1.268638
C	-2.436583	-0.779251	2.617877
C	-0.764888	-1.893174	-0.696708
C	-1.517583	-2.966413	0.048585
C	-1.817909	1.554725	-0.043801
C	-0.320179	1.454533	0.380703
C	0.411252	0.351647	-0.409346
C	-0.091686	-0.989719	0.023218
O	-2.055150	2.917556	-0.427703
C	-0.941421	3.683948	-0.367570
C	0.192927	2.846106	0.122247
O	-0.933277	4.844981	-0.667038
C	1.402528	3.376044	0.276963
C	-2.868512	1.183399	1.011244
C	-0.986752	-1.814385	-2.180670
O	-3.662361	-3.147251	-0.975765
O	1.809658	0.470190	-0.096087
C	2.645089	-0.125278	-0.978619
O	2.269685	-0.633070	-2.004868
C	4.064722	-0.102510	-0.472598
C	4.207452	-0.918788	0.824364
C	3.682838	-2.341737	0.630444
C	5.666227	-0.926407	1.276375
H	-3.408963	-3.138953	1.079269
H	-3.569183	-0.688888	-0.673919
H	-1.408220	-0.447765	2.811348
H	-2.467863	-1.866548	2.722312
H	-1.461302	-3.940239	-0.450097
H	-1.100243	-3.071366	1.055640
H	-3.058269	-0.342667	3.408756
H	-0.241814	1.216094	1.449788
H	0.280991	0.521903	-1.481457
H	0.006651	-1.155323	1.098164
H	2.245168	2.800688	0.642368
H	1.549353	4.423277	0.026448
H	-2.658699	1.741000	1.932089
H	-1.986967	0.941257	-0.937491
H	-3.832944	1.539924	0.633351
H	-2.016183	-1.505922	-2.402238
H	-0.866004	-2.808032	-2.624402
H	-0.281980	-1.136993	-2.666535
H	-4.602302	-2.920382	-0.929740

H	4.698523	-0.520623	-1.260249
H	4.366289	0.936440	-0.294443
H	3.600212	-0.424667	1.593128
H	3.808063	-2.927914	1.546275
H	2.619712	-2.349966	0.364992
H	4.230977	-2.846937	-0.173457
H	5.778858	-1.466142	2.221748
H	6.048677	0.089742	1.417751
H	6.296412	-1.423447	0.529577

M062X energy = -1154.58725766 a.u.

(2*S*,6*R*,7*S*,8*S*)-**1**, Conf. D

C	-2.396007	-2.954325	-0.077276
C	-2.923764	-1.549818	-0.175831
C	-3.018037	-0.633809	0.794504
C	-2.876318	-0.907029	2.269305
C	-0.163496	-1.848533	-0.378900
C	-1.018237	-3.022391	-0.789441
C	-2.068289	1.338399	-0.464637
C	-0.649812	1.243910	0.167159
C	0.360377	0.607385	-0.812816
C	-0.066421	-0.791830	-1.194511
O	-2.300570	2.724328	-0.756702
C	-1.378435	3.533030	-0.178140
C	-0.337725	2.680971	0.472788
O	-1.440383	4.729158	-0.222153
C	0.663083	3.224577	1.158018
C	-3.205086	0.830226	0.430899
C	0.364945	-1.899780	1.030017
O	-3.215554	-3.893947	-0.751788
O	1.640901	0.652957	-0.168151
C	2.705108	0.521401	-0.997502
O	2.596474	0.410443	-2.190877
C	3.995530	0.488652	-0.218957
C	4.056708	-0.728634	0.719834
C	3.789238	-2.020589	-0.052511
C	5.411251	-0.776409	1.423669
H	-2.267780	-3.248616	0.973747
H	-3.081631	-1.228716	-1.208660
H	-2.757371	-1.966021	2.505995
H	-2.022798	-0.361794	2.692638
H	-1.220309	-2.997394	-1.865475
H	-0.555354	-3.988094	-0.556017
H	-3.766807	-0.541281	2.794794
H	-0.673334	0.629971	1.073756
H	0.419107	1.223392	-1.716651
H	-0.482664	-0.881979	-2.196146
H	1.430015	2.622301	1.633235
H	0.718342	4.306401	1.243991
H	-3.227873	1.449629	1.336258
H	-2.101243	0.813329	-1.423189
H	-4.151508	1.001705	-0.093889
H	0.999583	-2.786072	1.155799
H	-0.458986	-2.000888	1.747538
H	0.953737	-1.020535	1.293165
H	-4.099208	-3.847723	-0.360347

H	4.810542	0.449474	-0.947726
H	4.088395	1.414386	0.360833
H	3.272646	-0.604159	1.477734
H	3.828166	-2.887618	0.614914
H	2.806123	-2.008350	-0.538058
H	4.544066	-2.161117	-0.835114
H	5.456410	-1.613714	2.126945
H	5.607285	0.145986	1.980018
H	6.217838	-0.908420	0.693249

M062X energy = -1154.58723499 a.u.

(2*S*,6*R*,7*S*,8*S*)-**1**, Conf. E

C	-2.801805	-2.877270	0.091434
C	-3.185474	-1.418651	0.162159
C	-2.973025	-0.565651	1.166351
C	-2.521379	-0.977711	2.540152
C	-0.571428	-1.913923	-0.646242
C	-1.255190	-3.062171	0.051842
C	-2.002734	1.395765	-0.094545
C	-0.535402	1.455155	0.429528
C	0.361433	0.431772	-0.295016
C	-0.027915	-0.955375	0.111070
O	-2.353135	2.722682	-0.515115
C	-1.329386	3.600197	-0.398288
C	-0.152889	2.890852	0.186250
O	-1.418336	4.750966	-0.722689
C	0.978731	3.548813	0.419534
C	-3.075588	0.924157	0.896259
C	-0.718003	-1.839986	-2.140443
O	-3.299804	-3.461802	-1.103151
O	1.712463	0.695457	0.122908
C	2.668032	0.206978	-0.695410
O	2.429360	-0.341936	-1.742683
C	4.050237	0.373587	-0.115670
C	4.673646	-1.008638	0.141976
C	6.086519	-0.852308	0.698540
C	3.794742	-1.832151	1.084451
H	-3.178788	-3.432100	0.964315
H	-3.500919	-1.011344	-0.801572
H	-1.541827	-0.543933	2.778693
H	-2.448183	-2.062334	2.652106
H	-1.069944	-4.022085	-0.442532
H	-0.890027	-3.131903	1.082091
H	-3.221443	-0.603249	3.296501
H	-0.505583	1.231189	1.503995
H	0.295487	0.585555	-1.375688
H	0.033893	-1.117755	1.189084
H	1.848092	3.072861	0.857312
H	1.030133	4.603007	0.160545
H	-2.986073	1.505853	1.821831
H	-2.048456	0.759572	-0.987349
H	-4.045760	1.173231	0.452454
H	-0.537415	-2.826379	-2.579156
H	-0.014347	-1.132938	-2.584696
H	-1.744026	-1.567480	-2.418626
H	-4.258530	-3.329669	-1.121274

H	4.658513	0.926956	-0.839070
H	3.999729	0.949697	0.813898
H	4.722281	-1.522316	-0.826078
H	6.557333	-1.829012	0.846907
H	6.720827	-0.267835	0.024316
H	6.063312	-0.341038	1.668430
H	4.244842	-2.811031	1.276815
H	2.797385	-1.999141	0.661764
H	3.676386	-1.319973	2.047236

M062X energy = -1154.58707160 a.u.

(2*S*,6*R*,7*S*,8*S*)-**1**, Conf. F

C	-4.082896	-1.479516	0.228111
C	-3.692568	-0.022934	0.152595
C	-3.108992	0.716140	1.098454
C	-2.960733	0.284353	2.531201
C	-1.649286	-1.819756	-0.415230
C	-2.830185	-2.400528	0.320579
C	-1.256637	1.783032	-0.240837
C	0.032905	1.175486	0.394070
C	0.326785	-0.225370	-0.181636
C	-0.716534	-1.184782	0.300780
O	-0.889601	3.047882	-0.811638
C	0.427849	3.324990	-0.675932
C	1.076309	2.213402	0.080587
O	0.931790	4.323958	-1.106830
C	2.367096	2.275159	0.393051
C	-2.444430	2.021479	0.701316
C	-1.706694	-1.844736	-1.917101
O	-4.773742	-1.867965	-0.950246
O	1.621404	-0.626067	0.305220
C	2.197127	-1.632598	-0.393466
O	1.716897	-2.097474	-1.396112
C	3.485763	-2.092269	0.246014
C	4.450498	-0.952932	0.595586
C	5.726310	-1.521488	1.215083
C	4.769175	-0.109954	-0.638982
H	-4.708028	-1.681381	1.111321
H	-3.736832	0.380239	-0.862061
H	-1.902506	0.207844	2.812432
H	-3.434342	-0.680026	2.731428
H	-3.131856	-3.375715	-0.077351
H	-2.574864	-2.525831	1.378164
H	-3.407973	1.029538	3.199937
H	-0.088764	1.077168	1.480637
H	0.371462	-0.169458	-1.272697
H	-0.773259	-1.240434	1.389612
H	2.861308	1.497746	0.963997
H	2.945769	3.135522	0.067157
H	-2.098088	2.578953	1.580104
H	-1.593319	1.151091	-1.071485
H	-3.146779	2.669155	0.165191
H	-2.015430	-2.838039	-2.258212
H	-0.739215	-1.614558	-2.367592
H	-2.466304	-1.146363	-2.290887
H	-5.544196	-1.290054	-1.047066

H	3.224004	-2.649922	1.154285
H	3.950466	-2.792990	-0.454417
H	3.956937	-0.318460	1.342664
H	6.414965	-0.718780	1.496595
H	5.508184	-2.111636	2.111137
H	6.243748	-2.171527	0.499942
H	5.445447	0.713525	-0.387299
H	3.863799	0.323121	-1.078945
H	5.256503	-0.724469	-1.405210

M062X energy = -1154.58693527 a.u.

(2*S*,6*R*,7*S*,8*S*)-**1**, Conf. G

C	-2.285774	-3.057573	-0.105571
C	-2.874776	-1.683747	-0.264432
C	-3.096572	-0.765392	0.682645
C	-3.060267	-1.017304	2.167657
C	-0.098645	-1.840142	-0.272207
C	-0.861109	-3.062691	-0.721590
C	-2.160616	1.238482	-0.539900
C	-0.784785	1.227362	0.186123
C	0.323195	0.634267	-0.710729
C	-0.001261	-0.790455	-1.096669
O	-2.445855	2.606916	-0.866859
C	-1.610974	3.470386	-0.237025
C	-0.571446	2.682676	0.492019
O	-1.735441	4.660847	-0.299635
C	0.352423	3.286163	1.233012
C	-3.327630	0.683421	0.285841
C	0.328052	-1.845851	1.171955
O	-3.009985	-4.047564	-0.816682
O	1.548480	0.755608	0.026386
C	2.674861	0.638941	-0.712289
O	2.666063	0.479310	-1.906211
C	3.919533	0.677942	0.139144
C	4.698518	-0.638653	-0.011944
C	5.991210	-0.582704	0.797967
C	3.831070	-1.825843	0.407512
H	-2.214751	-3.328688	0.956911
H	-2.964634	-1.381104	-1.310830
H	-2.265039	-0.433064	2.648602
H	-2.917505	-2.068112	2.427078
H	-0.990062	-3.061610	-1.809086
H	-0.365947	-3.999508	-0.441573
H	-4.003180	-0.683011	2.617112
H	-0.834936	0.622678	1.097983
H	0.416665	1.241289	-1.617879
H	-0.341306	-0.915476	-2.123010
H	1.120022	2.730802	1.761077
H	0.343623	4.370071	1.309295
H	-3.439236	1.310940	1.179263
H	-2.102610	0.703979	-1.492230
H	-4.244715	0.803486	-0.301773
H	-0.544323	-1.943888	1.830970
H	0.883453	-0.950881	1.453688
H	0.965501	-2.717391	1.366031
H	-3.920526	-4.035483	-0.489740

H	4.535374	1.516995	-0.202822
H	3.651521	0.848683	1.186923
H	4.943512	-0.745213	-1.075782
H	6.569594	-1.503209	0.672071
H	6.620234	0.258931	0.490641
H	5.772279	-0.468305	1.866395
H	4.382110	-2.766341	0.309173
H	2.927099	-1.899421	-0.209128
H	3.521645	-1.723798	1.455527

M062X energy = -1154.58689820 a.u.

(2*S*,6*R*,7*S*,8*S*)-**1**, Conf. H

C	-2.491814	-3.039932	0.038688
C	-2.985543	-1.612645	0.057051
C	-2.915058	-0.735578	1.060645
C	-2.540687	-1.099120	2.470723
C	-0.301195	-1.924106	-0.588283
C	-0.937118	-3.104538	0.101357
C	-2.027707	1.286856	-0.176310
C	-0.596203	1.465827	0.417973
C	0.408037	0.502220	-0.244440
C	0.101059	-0.901556	0.173269
O	-2.459960	2.579884	-0.625983
C	-1.512306	3.534347	-0.476332
C	-0.312502	2.923028	0.168475
O	-1.673502	4.671688	-0.819611
C	0.756266	3.665858	0.440173
C	-3.110259	0.740619	0.764536
C	-0.341943	-1.911942	-2.090359
O	-2.866918	-3.677607	-1.173437
O	1.716618	0.872565	0.224489
C	2.741683	0.456194	-0.552062
O	2.584341	-0.102965	-1.610040
C	4.074977	0.723709	0.099289
C	4.641595	-0.568069	0.731161
C	3.633852	-1.202444	1.691809
C	5.084447	-1.570254	-0.334329
H	-2.881860	-3.609274	0.896200
H	-3.251567	-1.240122	-0.935221
H	-1.619556	-0.587035	2.777509
H	-2.392523	-2.173454	2.604657
H	-0.645602	-4.059742	-0.349118
H	-0.638424	-3.115684	1.154967
H	-3.323645	-0.773799	3.166292
H	-0.603571	1.260311	1.496756
H	0.379157	0.631773	-1.329693
H	0.091275	-1.029963	1.257629
H	1.638490	3.259507	0.920680
H	0.742231	4.717925	0.168413
H	-3.111671	1.334999	1.686213
H	-1.978267	0.644415	-1.064057
H	-4.072707	0.913965	0.270755
H	0.014774	-2.872848	-2.475546
H	0.283317	-1.123148	-2.513059
H	-1.373339	-1.805613	-2.449246
H	-3.830252	-3.623692	-1.252034

H	4.759833	1.090905	-0.670771
H	3.953055	1.493152	0.866555
H	5.524337	-0.261735	1.306665
H	4.094002	-2.032745	2.236185
H	3.257702	-0.477176	2.421002
H	2.773029	-1.601988	1.140863
H	5.516633	-2.459947	0.134761
H	5.837559	-1.135737	-0.999433
H	4.235464	-1.882635	-0.949482

M062X energy = -1154.58683757 a.u.

(2*S*,6*R*,7*S*,8*S*)-**1**, Conf. I

C	-3.180732	-2.688069	0.092158
C	-3.436617	-1.205027	0.046484
C	-3.236079	-0.314884	1.019288
C	-2.965958	-0.676141	2.453827
C	-0.792381	-1.972648	-0.405798
C	-1.657690	-2.996114	0.283454
C	-1.975791	1.482211	-0.233092
C	-0.567296	1.458025	0.437851
C	0.301017	0.308644	-0.110491
C	-0.255358	-1.004285	0.342089
O	-2.168925	2.806467	-0.754106
C	-1.083863	3.598293	-0.596381
C	-0.031539	2.832534	0.135777
O	-1.037144	4.727746	-0.996356
C	1.134141	3.404385	0.423191
C	-3.180281	1.158664	0.661020
C	-0.764418	-2.015346	-1.907605
O	-3.651501	-3.193486	-1.147134
O	1.622015	0.495267	0.430888
C	2.610883	-0.130331	-0.240755
O	2.425358	-0.777258	-1.241609
C	3.953764	0.122839	0.405199
C	5.042327	-0.831781	-0.085043
C	6.410033	-0.371687	0.416806
C	4.751446	-2.264412	0.362492
H	-3.724318	-3.162073	0.924165
H	-3.649288	-0.844320	-0.961123
H	-2.983729	-1.755177	2.626568
H	-3.720610	-0.217727	3.104384
H	-1.472523	-4.011009	-0.094812
H	-1.436322	-3.001911	1.356291
H	-1.993287	-0.292920	2.787309
H	-0.667382	1.320505	1.522701
H	0.360758	0.382785	-1.199813
H	-0.333909	-1.084138	1.427657
H	1.916633	2.888068	0.966505
H	1.303977	4.428392	0.101014
H	-3.136037	1.788202	1.558150
H	-1.986401	0.800365	-1.092694
H	-4.074227	1.457030	0.102807
H	0.001834	-1.359360	-2.324499
H	-1.746922	-1.755696	-2.321305
H	-0.550866	-3.036421	-2.243154
H	-3.499896	-4.148549	-1.159689

H	4.220122	1.165463	0.182406
H	3.833988	0.062617	1.493897
H	5.032819	-0.804372	-1.181207
H	7.199976	-1.039964	0.060606
H	6.644357	0.642206	0.075984
H	6.440457	-0.374592	1.513115
H	5.520360	-2.949958	-0.007109
H	3.783409	-2.607367	-0.012213
H	4.744939	-2.329191	1.457780

M062X energy = -1154.58652371 a.u.

H	3.684478	-0.938570	1.685306
H	3.660447	-2.625840	1.133084
H	4.648975	-1.939117	-1.035083
H	5.595638	0.353691	-1.316739
H	3.843893	0.434766	-1.082063
H	4.938680	0.824517	0.258395
H	6.961337	-1.487366	-0.173048
H	6.146840	-2.718094	0.807396
H	6.287302	-1.055346	1.405150

M062X energy = -1154.58643900 a.u.

(2*S*,6*R*,7*S*,8*S*)-**1**, Conf. J

C	-4.055130	-1.668178	0.021369
C	-3.739521	-0.200768	-0.096855
C	-3.312295	0.622083	0.861990
C	-3.311775	0.284369	2.327441
C	-1.541720	-1.906623	-0.279833
C	-2.772360	-2.500235	0.357321
C	-1.379215	1.735749	-0.316728
C	-0.141509	1.243817	0.495389
C	0.289609	-0.169038	0.052539
C	-0.737261	-1.165691	0.487873
O	-1.018629	2.992993	-0.909251
C	0.262072	3.348037	-0.656200
C	0.883476	2.311407	0.220179
O	0.758541	4.350567	-1.087803
C	2.139590	2.443549	0.636660
C	-2.684844	1.944643	0.461495
C	-1.423438	-2.039961	-1.771962
O	-4.593045	-2.042364	-1.237025
O	1.547975	-0.439485	0.695727
C	2.312122	-1.371658	0.091242
O	1.990665	-1.938810	-0.923969
C	3.627887	-1.581878	0.800951
C	4.797408	-1.301961	-0.154664
C	4.790028	0.159861	-0.602038
C	6.123696	-1.665173	0.508503
H	-4.792763	-1.854402	0.817296
H	-3.719047	0.143295	-1.132144
H	-2.293488	0.280793	2.736990
H	-3.765061	-0.688371	2.534924
H	-2.947975	-3.533644	0.027738
H	-2.647662	-2.520735	1.445659
H	-3.870246	1.043520	2.888141
H	-0.376360	1.208742	1.567144
H	0.447877	-0.178901	-1.029328
H	-0.914457	-1.154658	1.564764
H	2.610530	1.718614	1.290454
H	2.710789	3.309633	0.312767
H	-2.481008	2.573663	1.336656
H	-1.574226	1.041248	-1.143193
H	-3.354935	2.511813	-0.193626
H	-1.565412	-3.087856	-2.059655
H	-0.445568	-1.722680	-2.139056
H	-2.215961	-1.473618	-2.276479
H	-4.817580	-2.982419	-1.200916

(2*S*,6*R*,7*S*,8*S*)-**1**, Conf. K

C	-3.992795	-1.639886	0.008422
C	-3.664252	-0.179872	-0.193192
C	-3.284171	0.707778	0.728500
C	-3.359027	0.464153	2.210466
C	-1.474460	-1.890816	-0.197833
C	-2.730446	-2.455686	0.416314
C	-1.284085	1.725081	-0.425494
C	-0.097627	1.277962	0.482130
C	0.358683	-0.151858	0.125016
C	-0.694464	-1.120269	0.565973
O	-0.877458	2.934856	-1.082610
C	0.382622	3.312130	-0.761762
C	0.933205	2.344072	0.231631
O	0.909484	4.284329	-1.224983
C	2.135060	2.527457	0.769305
C	-2.622872	1.996464	0.273737
C	-1.288868	-2.091907	-1.676191
O	-4.461001	-2.213519	-1.203298
O	1.589978	-0.391700	0.830174
C	2.326654	-1.418592	0.352019
O	2.006210	-2.063485	-0.616945
C	3.595112	-1.624107	1.144364
C	4.847200	-1.197721	0.348903
C	5.085279	-2.078533	-0.877539
C	4.776025	0.279518	-0.038107
H	-4.745254	-1.774782	0.800255
H	-3.554205	0.091910	-1.245859
H	-3.930207	1.263069	2.698171
H	-2.359238	0.473901	2.663480
H	-2.916096	-3.491062	0.109924
H	-2.644495	-2.430704	1.507917
H	-3.831295	-0.490398	2.455946
H	-0.399208	1.283180	1.537276
H	0.559223	-0.215045	-0.948464
H	-0.915823	-1.054096	1.632912
H	2.538124	1.850947	1.514059
H	2.731016	3.380167	0.454803
H	-2.452868	2.679297	1.115139
H	-1.445156	0.979009	-1.213162
H	-3.255200	2.525956	-0.447382
H	-1.987997	-1.469410	-2.248988
H	-1.520352	-3.128670	-1.940103
H	-0.264751	-1.878771	-1.989525
H	-5.229739	-1.703383	-1.496412

H	3.531435	-1.057695	2.077612
H	3.659518	-2.690466	1.384250
H	5.694640	-1.331174	1.033878
H	6.033291	-1.810977	-1.355039
H	5.127919	-3.137559	-0.603550
H	4.283921	-1.957823	-1.611261
H	5.678668	0.582567	-0.577444
H	4.678927	0.920078	0.844855
H	3.916858	0.475885	-0.691954

M062X energy = -1154.58639985 a.u.

(2*S*,6*R*,7*S*,8*S*)-**1**, Conf. L

C	-3.079081	-2.586137	0.175108
C	-3.281427	-1.098017	0.284485
C	-2.903542	-0.293499	1.279152
C	-2.420434	-0.774214	2.619678
C	-0.789341	-1.894000	-0.680434
C	-1.559033	-2.945426	0.076927
C	-1.806657	1.566349	-0.034007
C	-0.305735	1.452740	0.377224
C	0.410014	0.340056	-0.413610
C	-0.100918	-0.994650	0.029831
O	-2.033934	2.932000	-0.415156
C	-0.912153	3.686279	-0.370783
C	0.219178	2.838126	0.108893
O	-0.894011	4.846333	-0.674076
C	1.435132	3.357094	0.250275
C	-2.851429	1.202870	1.029323
C	-1.018788	-1.821029	-2.163586
O	-3.773247	-2.978041	-0.998347
O	1.811832	0.448215	-0.111799
C	2.635124	-0.167887	-0.991004
O	2.247065	-0.691616	-2.004582
C	4.060220	-0.142386	-0.500133
C	4.211662	-0.916487	0.821164
C	3.676199	-2.341512	0.679313
C	5.675232	-0.919134	1.257673
H	-3.491163	-3.110843	1.051110
H	-3.635172	-0.652283	-0.646538
H	-2.485976	-1.860032	2.726405
H	-3.017579	-0.320087	3.419512
H	-1.476674	-3.934706	-0.394025
H	-1.158083	-3.031873	1.092826
H	-1.379707	-0.474886	2.799748
H	-0.220776	1.218110	1.446712
H	0.272549	0.506140	-1.485466
H	0.002591	-1.154561	1.104867
H	2.276100	2.775264	0.609156
H	1.588823	4.402303	-0.004550
H	-2.627534	1.754816	1.950294
H	-1.990394	0.955264	-0.926365
H	-3.815378	1.570173	0.660964
H	-2.045020	-1.500338	-2.381645
H	-0.908834	-2.818210	-2.603989
H	-0.306044	-1.157262	-2.656349
H	-3.664000	-3.932696	-1.108464

H	4.681606	-0.590754	-1.281057
H	4.372304	0.899316	-0.359907
H	3.616067	-0.393518	1.579802
H	3.804881	-2.897605	1.613235
H	2.610895	-2.351300	0.422445
H	4.214886	-2.876620	-0.111512
H	5.794900	-1.425551	2.220437
H	6.065984	0.098619	1.358846
H	6.293763	-1.446824	0.522265

M062X energy = -1154.58635256 a.u.

(2*S*,6*R*,7*S*,8*S*)-**1**, Conf. M

C	-3.346176	-2.417209	-0.116285
C	-3.460534	-0.933790	-0.329464
C	-3.407073	0.038118	0.588281
C	-3.494806	-0.164750	2.078705
C	-0.874869	-1.972383	-0.154320
C	-1.965866	-2.893927	-0.641691
C	-1.820729	1.599568	-0.605224
C	-0.564018	1.186009	0.212005
C	0.346233	0.224302	-0.583194
C	-0.405135	-1.026327	-0.974257
O	-1.640635	2.974353	-0.977177
C	-0.606365	3.554152	-0.319075
C	0.089745	2.509333	0.491340
O	-0.340823	4.718622	-0.418953
C	1.122774	2.817598	1.268913
C	-3.149224	1.470616	0.150504
C	-0.534494	-2.097098	1.307120
O	-4.305648	-3.149932	-0.859802
O	1.483393	-0.026436	0.254850
C	2.561559	-0.551063	-0.371396
O	2.594430	-0.764335	-1.555979
C	3.668531	-0.844653	0.614052
C	5.007003	-1.150446	-0.057348
C	5.560844	0.088170	-0.761769
C	6.001084	-1.678990	0.975871
H	-3.425861	-2.663837	0.951716
H	-3.416659	-0.653872	-1.385193
H	-3.708484	-1.196299	2.365003
H	-4.289433	0.471094	2.487482
H	-2.025772	-2.879527	-1.734987
H	-1.817052	-3.933080	-0.326283
H	-2.566233	0.144452	2.576020
H	-0.858626	0.674035	1.134437
H	0.702696	0.729019	-1.488043
H	-0.718274	-1.054449	-2.016405
H	1.641524	2.068433	1.857352
H	1.461586	3.848985	1.315179
H	-3.110633	2.136427	1.021893
H	-1.880346	1.035110	-1.539526
H	-3.945534	1.846557	-0.501455
H	-0.150988	-3.104622	1.510808
H	-1.433743	-1.977571	1.924629
H	0.213827	-1.375005	1.634179
H	-5.182570	-2.839916	-0.593279

H	3.757643	0.004382	1.303778
H	3.330750	-1.696065	1.221067
H	4.827422	-1.925159	-0.812313
H	6.512653	-0.138331	-1.252122
H	4.866769	0.454877	-1.522313
H	5.741667	0.891570	-0.036757
H	6.963159	-1.909191	0.508064
H	5.633070	-2.589511	1.460323
H	6.180805	-0.930050	1.756614

M062X energy = -1154.58632374 a.u.

(2*S*,6*R*,7*S*,8*S*)-**1**, Conf. N

C	-2.353597	-2.997384	-0.159377
C	-2.877580	-1.607697	-0.396058
C	-3.144268	-0.660737	0.510892
C	-3.243206	-0.879750	1.998056
C	-0.116996	-1.860392	-0.148021
C	-0.879284	-3.065890	-0.640192
C	-2.024208	1.283293	-0.651144
C	-0.723905	1.227562	0.201745
C	0.435107	0.585739	-0.591109
C	0.098666	-0.835161	-0.980466
O	-2.220260	2.656985	-1.018649
C	-1.413759	3.495235	-0.320967
C	-0.483715	2.676739	0.514295
O	-1.480871	4.688634	-0.409195
C	0.385579	3.250386	1.340224
C	-3.284815	0.786499	0.067776
C	0.175907	-1.844875	1.328570
O	-3.043780	-3.977388	-0.916629
O	1.608227	0.687838	0.229514
C	2.783231	0.637519	-0.441561
O	2.847866	0.526214	-1.640234
C	3.966915	0.689856	0.492023
C	4.657797	-0.687831	0.589793
C	3.660988	-1.766165	1.015462
C	5.360304	-1.070598	-0.712376
H	-2.390564	-3.248438	0.909800
H	-2.869390	-1.326160	-1.452072
H	-2.465566	-0.319855	2.533650
H	-3.171149	-1.929452	2.289372
H	-0.907167	-3.087570	-1.734659
H	-0.447695	-4.012240	-0.294798
H	-4.204188	-0.494834	2.360049
H	-0.886977	0.634097	1.107283
H	0.612494	1.173330	-1.498068
H	-0.147013	-0.971704	-2.032009
H	1.070212	2.668127	1.947755
H	0.416896	4.334423	1.408822
H	-3.458836	1.435795	0.935274
H	-1.899037	0.732078	-1.587634
H	-4.136313	0.925209	-0.607795
H	0.710244	-0.948477	1.644967
H	0.788790	-2.715596	1.592790
H	-0.752847	-1.929488	1.906568
H	-3.979549	-3.928449	-0.675461

H	4.670262	1.428583	0.095174
H	3.632020	1.015175	1.480649
H	5.418221	-0.589332	1.375033
H	4.166703	-2.725721	1.161212
H	3.156900	-1.499577	1.950805
H	2.890251	-1.908925	0.246189
H	5.884390	-2.024274	-0.593526
H	6.094119	-0.312287	-1.003987
H	4.640359	-1.170795	-1.529221

M062X energy = -1154.58625000 a.u.

(2*S*,6*R*,7*S*,8*S*)-**1**, Conf. O

C	-2.799001	-2.884908	0.075264
C	-3.187969	-1.433079	0.163076
C	-2.963863	-0.586962	1.169505
C	-2.500936	-1.000840	2.539169
C	-0.555354	-1.921229	-0.625599
C	-1.243452	-3.059430	0.083058
C	-2.016983	1.386252	-0.088275
C	-0.546618	1.457492	0.427743
C	0.354672	0.436203	-0.293999
C	-0.022792	-0.950666	0.123818
O	-2.379885	2.711544	-0.504398
C	-1.360745	3.594895	-0.400337
C	-0.174097	2.894207	0.174731
O	-1.458525	4.744492	-0.726726
C	0.955807	3.559826	0.394166
C	-3.080559	0.903208	0.906751
C	-0.696707	-1.865549	-2.120930
O	-3.357080	-3.352672	-1.142331
O	1.706121	0.711231	0.116287
C	2.660465	0.212771	-0.696829
O	2.420143	-0.354434	-1.734144
C	4.044549	0.393634	-0.125730
C	4.677627	-0.981970	0.142780
C	6.093736	-0.812352	0.687035
C	3.810059	-1.799364	1.100904
H	-3.204219	-3.459447	0.922888
H	-3.543776	-1.034926	-0.788396
H	-3.197800	-0.627543	3.299080
H	-1.519942	-0.569113	2.775891
H	-1.013870	-4.029966	-0.378199
H	-0.903138	-3.103566	1.123574
H	-2.431820	-2.085944	2.650532
H	-0.510953	1.240118	1.503507
H	0.283118	0.581881	-1.375376
H	0.035682	-1.101522	1.203402
H	1.832650	3.091295	0.824950
H	0.997618	4.613310	0.130614
H	-2.990418	1.482354	1.833913
H	-2.063424	0.751204	-0.981841
H	-4.054991	1.145674	0.468946
H	-1.727421	-1.620381	-2.405971
H	-0.487500	-2.851599	-2.549725
H	-0.004920	-1.149658	-2.569170
H	-3.130366	-4.287911	-1.238407

H	4.645614	0.943092	-0.858138
H	3.995494	0.979378	0.797840
H	4.721915	-1.506259	-0.819792
H	6.570370	-1.785086	0.842909
H	6.720174	-0.232673	0.001425
H	6.075461	-0.290197	1.651176
H	4.266456	-2.773868	1.300673
H	2.810346	-1.975609	0.687541
H	3.696426	-1.277155	2.058833

M062X energy = -1154.58618102 a.u.

(2*S*,6*R*,7*S*,8*S*)-**1**, Conf. P

C	-3.757691	-1.736441	0.119798
C	-3.537457	-0.261688	-0.070713
C	-3.128384	0.634944	0.833891
C	-3.068580	0.407866	2.322131
C	-1.283191	-1.894064	-0.269093
C	-2.620807	-2.507609	-0.601893
C	-1.374371	1.810291	-0.553161
C	-0.168622	1.064017	0.087182
C	0.368511	-0.042582	-0.846830
C	-0.711252	-1.057878	-1.141819
O	-0.903523	3.113574	-0.928936
C	0.314392	3.391944	-0.401867
C	0.815858	2.176489	0.308066
O	0.850015	4.457397	-0.523177
C	1.964580	2.196012	0.976800
C	-2.588789	1.978051	0.369125
C	-0.791142	-2.149470	1.131260
O	-4.960650	-2.190212	-0.477635
O	1.516010	-0.605655	-0.195349
C	2.313800	-1.358714	-0.991474
O	2.113412	-1.503436	-2.169000
C	3.440779	-1.987831	-0.207787
C	4.170915	-1.021509	0.733043
C	5.292059	-1.757879	1.464981
C	4.715628	0.182333	-0.034696
H	-3.742056	-1.997179	1.187179
H	-3.566337	0.037970	-1.121485
H	-3.484930	-0.552212	2.632882
H	-3.627719	1.200097	2.834451
H	-2.825071	-2.435307	-1.675343
H	-2.685772	-3.564019	-0.317012
H	-2.036899	0.471102	2.691700
H	-0.467021	0.588602	1.027487
H	0.705753	0.410236	-1.785885
H	-1.157019	-0.968992	-2.130719
H	2.339646	1.322751	1.500642
H	2.549946	3.111354	1.000975
H	-2.280570	2.585250	1.229552
H	-1.686635	1.320485	-1.479540
H	-3.346527	2.555617	-0.171843
H	-0.683974	-3.229013	1.293454
H	-1.523602	-1.795936	1.868190
H	0.169082	-1.676329	1.337938
H	-5.689870	-1.687453	-0.088235

H	3.013366	-2.818111	0.370066
H	4.133047	-2.415005	-0.939822
H	3.444053	-0.667600	1.475241
H	5.808502	-1.090054	2.161361
H	4.907191	-2.610886	2.033408
H	6.033166	-2.135583	0.750989
H	5.227205	0.876896	0.639775
H	3.916308	0.734861	-0.540653
H	5.437221	-0.142584	-0.793663

M062X energy = -1154.58618071 a.u.

(2*S*,6*R*,7*S*,8*S*)-**1**, Conf. Q

C	-3.844282	-1.779973	-0.007365
C	-3.610217	-0.315679	-0.254854
C	-3.293499	0.629341	0.637609
C	-3.377028	0.478550	2.134204
C	-1.341601	-1.937851	-0.129308
C	-2.636222	-2.581145	-0.561721
C	-1.414544	1.751294	-0.628098
C	-0.273153	1.051180	0.163915
C	0.353355	-0.106293	-0.642742
C	-0.686367	-1.146619	-0.985229
O	-0.918064	3.040230	-1.020129
C	0.248021	3.353323	-0.403214
C	0.690074	2.177954	0.406183
O	0.788625	4.415602	-0.530734
C	1.785994	2.231588	1.156959
C	-2.715842	1.951742	0.159563
C	-0.994634	-2.113206	1.325223
O	-4.976391	-2.276959	-0.701286
O	1.432078	-0.609922	0.158703
C	2.361565	-1.328671	-0.509537
O	2.300172	-1.548079	-1.692284
C	3.498742	-1.759990	0.383697
C	4.812707	-1.119865	-0.093035
C	4.738024	0.404224	-0.000465
C	5.988594	-1.660456	0.716762
H	-3.941546	-1.980618	1.068757
H	-3.536722	-0.071820	-1.317818
H	-3.819727	-0.467151	2.452461
H	-3.984190	1.292097	2.549444
H	-2.728915	-2.573335	-1.652820
H	-2.727692	-3.619774	-0.223517
H	-2.385688	0.566396	2.597545
H	-0.656116	0.631480	1.099970
H	0.784894	0.291258	-1.568140
H	-1.030883	-1.116818	-2.017275
H	2.117807	1.384612	1.748032
H	2.370386	3.147575	1.179270
H	-2.496872	2.605206	1.013478
H	-1.631019	1.213473	-1.555366
H	-3.418317	2.494068	-0.483048
H	-0.854576	-3.178985	1.543777
H	-1.821129	-1.771966	1.961015
H	-0.086089	-1.585451	1.615177
H	-5.746950	-1.772510	-0.404706

H	3.289743	-1.484137	1.422583
H	3.574364	-2.851115	0.322067
H	4.941154	-1.401663	-1.145425
H	5.657599	0.862807	-0.376515
H	3.900424	0.809015	-0.579806
H	4.609098	0.718574	1.042940
H	6.932007	-1.228065	0.369314
H	6.064248	-2.749540	0.635449
H	5.875219	-1.407278	1.777719

M062X energy = -1154.58614918 a.u.

(2*S*,6*R*,7*S*,8*S*)-**1**, Conf. R

C	-3.354314	-2.507874	-0.001036
C	-3.483785	-1.010447	-0.145764
C	-3.352161	-0.082782	0.804514
C	-3.292316	-0.388357	2.275796
C	-0.885651	-1.986006	-0.280905
C	-1.891781	-2.924251	0.336355
C	-1.805728	1.542057	-0.355047
C	-0.496726	1.442875	0.486993
C	0.341641	0.217075	0.068844
C	-0.357157	-1.035274	0.495775
O	-1.820758	2.845460	-0.956196
C	-0.711630	3.568576	-0.676179
C	0.169708	2.766696	0.223435
O	-0.528558	4.672982	-1.105540
C	1.318036	3.270561	0.665335
C	-3.132157	1.361680	0.394777
C	-0.692642	-2.082464	-1.768578
O	-3.676082	-3.151664	-1.225384
O	1.610307	0.334555	0.736744
C	2.626808	-0.353372	0.171694
O	2.498100	-1.002911	-0.836667
C	3.894633	-0.169310	0.976602
C	5.141138	-0.857695	0.410900
C	4.998359	-2.380960	0.390373
C	5.520907	-0.311834	-0.967090
H	-4.002225	-2.889581	0.802761
H	-3.502229	-0.681620	-1.187736
H	-3.422244	-1.451911	2.491128
H	-4.074256	0.165000	2.809534
H	-1.772696	-3.954677	-0.016379
H	-1.775176	-2.918679	1.425219
H	-2.334530	-0.068827	2.706378
H	-0.735601	1.344701	1.554135
H	0.515262	0.246623	-1.010440
H	-0.558379	-1.070120	1.568087
H	1.970699	2.721835	1.334292
H	1.608624	4.267552	0.345328
H	-3.143775	2.030745	1.263804
H	-1.767386	0.816661	-1.177083
H	-3.924638	1.700012	-0.281689
H	-0.569718	-3.131667	-2.056306
H	0.187770	-1.530942	-2.104698
H	-1.582373	-1.724462	-2.301833
H	-4.570085	-2.880040	-1.478414

H	4.059369	0.912145	1.067645
H	3.680350	-0.526203	1.992030
H	5.954260	-0.607807	1.105844
H	5.937731	-2.846027	0.074717
H	4.749496	-2.769393	1.384193
H	4.214211	-2.686998	-0.306407
H	6.466918	-0.748224	-1.302753
H	5.644387	0.776582	-0.942063
H	4.751060	-0.555421	-1.703712

M062X energy = -1154.58614848 a.u.

(2*S*,6*R*,7*S*,8*S*)-**1**, Conf. S

C	-4.095144	-1.458869	0.207698
C	-3.700872	-0.007389	0.142355
C	-3.110696	0.720930	1.091368
C	-2.964672	0.286887	2.523800
C	-1.651496	-1.822982	-0.388195
C	-2.838580	-2.379645	0.355119
C	-1.255840	1.786631	-0.241913
C	0.032371	1.177853	0.395891
C	0.324455	-0.225857	-0.174113
C	-0.717146	-1.181447	0.319588
O	-0.884755	3.050105	-0.813875
C	0.432708	3.324022	-0.678702
C	1.078489	2.212432	0.080490
O	0.940286	4.320629	-1.111085
C	2.368742	2.273186	0.395222
C	-2.445376	2.026861	0.697311
C	-1.709503	-1.865765	-1.889618
O	-4.779609	-1.721289	-1.006919
O	1.621402	-0.625410	0.307994
C	2.190713	-1.638263	-0.386103
O	1.701333	-2.113103	-1.379849
C	3.484486	-2.093773	0.246039
C	4.450329	-0.953117	0.587815
C	5.730454	-1.520094	1.199864
C	4.760570	-0.111438	-0.649739
H	-4.757418	-1.655719	1.065128
H	-3.776941	0.404519	-0.865047
H	-1.908401	0.195618	2.808309
H	-3.454760	-0.669034	2.725643
H	-3.114013	-3.381822	-0.001666
H	-2.596231	-2.466709	1.420068
H	-3.403253	1.038513	3.191061
H	-0.090797	1.083997	1.482693
H	0.363422	-0.176027	-1.265667
H	-0.773861	-1.223483	1.408757
H	2.861099	1.496689	0.969013
H	2.948923	3.132177	0.068375
H	-2.100600	2.583932	1.577027
H	-1.592950	1.154461	-1.072205
H	-3.146145	2.674537	0.159366
H	-0.740087	-1.647439	-2.341415
H	-2.465609	-1.168821	-2.272068
H	-2.018564	-2.863259	-2.220289
H	-5.058864	-2.647253	-1.000853

H	3.229052	-2.650292	1.156865
H	3.945562	-2.795506	-0.455814
H	3.961075	-0.317992	1.337103
H	6.420144	-0.716602	1.476470
H	5.518442	-2.109748	2.097734
H	6.243978	-2.170233	0.481980
H	5.437845	0.712779	-0.403237
H	3.852145	0.320703	-1.084185
H	5.243608	-0.726427	-1.418335

M062X energy = -1154.58605342 a.u.

(2*S*,6*R*,7*S*,8*S*)-**1**, Conf. T

C	-4.107635	-1.523787	0.100706
C	-3.725002	-0.075404	-0.087446
C	-3.272666	0.777591	0.833847
C	-3.298749	0.507265	2.312917
C	-1.614660	-1.876453	-0.231503
C	-2.864167	-2.402581	0.428852
C	-1.283450	1.739120	-0.385304
C	-0.081256	1.217351	0.458919
C	0.298742	-0.218404	0.039898
C	-0.769356	-1.155418	0.511257
O	-0.851256	2.947057	-1.029692
C	0.432885	3.266985	-0.744667
C	0.981345	2.249706	0.200337
O	0.980649	4.231552	-1.199501
C	2.207423	2.376686	0.698682
C	-2.578408	2.048623	0.377825
C	-1.504074	-2.054139	-1.720446
O	-4.660255	-2.049517	-1.097083
O	1.557064	-0.520389	0.665610
C	2.242901	-1.543567	0.108547
O	1.844499	-2.166606	-0.844371
C	3.578967	-1.734536	0.778641
C	4.631570	-0.771540	0.192485
C	5.938276	-0.901216	0.972975
C	4.856938	-1.033620	-1.296291
H	-4.825871	-1.643332	0.926163
H	-3.646255	0.212520	-1.138623
H	-3.818123	1.317927	2.837659
H	-2.282530	0.468553	2.725814
H	-3.109033	-3.421070	0.107911
H	-2.726345	-2.409413	1.515380
H	-3.798911	-0.432626	2.559231
H	-0.339779	1.201577	1.525276
H	0.435603	-0.259576	-1.044701
H	-0.939059	-1.104658	1.588494
H	2.614008	1.669480	1.412802
H	2.818251	3.219242	0.385649
H	-2.343887	2.703084	1.226295
H	-1.509501	1.021650	-1.183547
H	-3.218660	2.621041	-0.302314
H	-2.203539	-1.394154	-2.249168
H	-1.787739	-3.075853	-1.991948
H	-0.487631	-1.875177	-2.077162
H	-5.418490	-1.499538	-1.341518

H	3.473069	-1.560973	1.854049
H	3.892798	-2.769495	0.610850
H	4.249852	0.251344	0.313242
H	6.691424	-0.206742	0.588221
H	5.793573	-0.691838	2.037761
H	6.340363	-1.916894	0.878936
H	5.594434	-0.334166	-1.701536
H	3.934153	-0.932348	-1.873786
H	5.234464	-2.051321	-1.450529

M062X energy = -1154.58604371 a.u.

(2*S*,6*R*,7*S*,8*S*)-**1**, Conf. U

C	-4.110508	-1.539402	-0.005245
C	-3.731862	-0.095660	-0.187764
C	-3.279821	0.761349	0.734640
C	-3.316529	0.535724	2.223977
C	-1.649202	-1.962915	-0.276467
C	-3.028634	-2.431988	-0.669112
C	-1.355967	1.745308	-0.567413
C	-0.275704	0.871516	0.127441
C	0.199836	-0.283275	-0.790266
C	-0.957363	-1.193303	-1.123461
O	-0.722966	2.985723	-0.914504
C	0.487351	3.129129	-0.318125
C	0.807394	1.867547	0.418137
O	1.141416	4.129529	-0.400493
C	1.900979	1.765210	1.168112
C	-2.587498	2.042530	0.297844
C	-1.241361	-2.260639	1.141182
O	-5.324213	-1.871348	-0.658843
O	1.256119	-0.966836	-0.102299
C	2.495817	-0.909998	-0.645729
O	2.749383	-0.368902	-1.690450
C	3.494872	-1.638897	0.222915
C	4.935281	-1.202330	-0.044750
C	5.140426	0.259820	0.351954
C	5.907950	-2.114230	0.700702
H	-4.173680	-1.792563	1.061987
H	-3.681310	0.199240	-1.239175
H	-2.303599	0.499549	2.645525
H	-3.839340	-0.378177	2.512235
H	-3.173794	-2.345829	-1.751299
H	-3.220991	-3.473209	-0.386265
H	-3.821374	1.380243	2.708325
H	-0.673491	0.419853	1.042155
H	0.616987	0.140199	-1.707965
H	-1.362765	-1.056464	-2.124812
H	2.138245	0.863061	1.724427
H	2.582977	2.608719	1.233667
H	-2.258060	2.616219	1.173176
H	-1.676759	1.290971	-1.509035
H	-3.256882	2.693339	-0.275542
H	-1.256912	-3.344050	1.310216
H	-1.958759	-1.825091	1.848686
H	-0.243142	-1.893814	1.378984
H	-6.017816	-1.301169	-0.298318

H	3.225716	-1.490052	1.276007
H	3.367702	-2.711241	0.021453
H	5.109634	-1.294166	-1.123261
H	6.167779	0.577747	0.149058
H	4.466810	0.919309	-0.203706
H	4.956086	0.394863	1.425877
H	6.944320	-1.828308	0.496832
H	5.781767	-3.161956	0.408170
H	5.749995	-2.045526	1.783868

M062X energy = -1154.58601644 a.u.

(2*S*,6*R*,7*S*,8*S*)-**1**, Conf. V

C	-2.489899	-3.047466	0.028834
C	-2.989760	-1.627547	0.070442
C	-2.893328	-0.756548	1.076088
C	-2.486163	-1.121233	2.476967
C	-0.294818	-1.924675	-0.583742
C	-0.928506	-3.100177	0.114612
C	-2.039208	1.275326	-0.164067
C	-0.602039	1.464976	0.414365
C	0.403449	0.506052	-0.252688
C	0.109055	-0.898453	0.171570
O	-2.485707	2.566290	-0.606769
C	-1.541836	3.525808	-0.473028
C	-0.329445	2.922946	0.156586
O	-1.713119	4.661798	-0.816314
C	0.738289	3.673142	0.411716
C	-3.106451	0.718664	0.787935
C	-0.348962	-1.915221	-2.085513
O	-2.951899	-3.576150	-1.203878
O	1.712687	0.885408	0.207661
C	2.736667	0.461259	-0.565485
O	2.578170	-0.114289	-1.614568
C	4.070684	0.742650	0.078641
C	4.640845	-0.537007	0.731736
C	3.634252	-1.159358	1.701404
C	5.087509	-1.554470	-0.317597
H	-2.891054	-3.637528	0.867588
H	-3.311720	-1.266979	-0.907713
H	-3.254642	-0.795095	3.188145
H	-1.557483	-0.613298	2.767884
H	-0.600195	-4.058131	-0.311911
H	-0.641027	-3.096819	1.171814
H	-2.342117	-2.196678	2.607964
H	-0.598037	1.263873	1.494036
H	0.366879	0.632230	-1.338133
H	0.108884	-1.023201	1.256098
H	1.629586	3.274251	0.881548
H	0.713648	4.724275	0.137043
H	-3.102254	1.311103	1.710901
H	-1.996093	0.634337	-1.053213
H	-4.075687	0.883970	0.305122
H	0.001432	-2.877967	-2.473822
H	0.280467	-1.132566	-2.513313
H	-1.382006	-1.800223	-2.436554
H	-2.643856	-4.490213	-1.274166

H	4.753541	1.098119	-0.698651
H	3.948414	1.524708	0.832963
H	5.522149	-0.219059	1.303082
H	3.253658	-0.423610	2.417634
H	2.776144	-1.571418	1.155502
H	4.096939	-1.978592	2.260231
H	4.240213	-1.875923	-0.930510
H	5.519051	-2.436913	0.165614
H	5.842103	-1.129120	-0.986896

M062X energy = -1154.58594617 a.u.

(2*S*,6*R*,7*S*,8*S*)-**1**, Conf. W

C	-3.920350	-1.760291	0.418562
C	-3.561761	-0.321589	0.702941
C	-2.634805	0.135909	1.547419
C	-1.935264	-0.710911	2.574501
C	-1.879375	-1.593452	-1.096653
C	-2.753317	-2.519010	-0.284747
C	-1.358880	1.739355	0.085194
C	0.060819	1.098438	0.060361
C	0.145940	-0.052686	-0.975918
C	-0.718802	-1.203477	-0.557613
O	-1.193202	3.146935	-0.142453
C	0.095351	3.495365	-0.358546
C	0.949539	2.273672	-0.249503
O	0.435755	4.621515	-0.590611
C	2.266858	2.377401	-0.399759
C	-2.155172	1.565777	1.386186
C	-2.493557	-1.071828	-2.365334
O	-5.028861	-1.826931	-0.468638
O	1.514732	-0.454450	-1.207459
C	2.231012	-0.971304	-0.196864
O	1.826464	-1.094751	0.936652
C	3.605817	-1.385240	-0.672359
C	4.604952	-1.578998	0.467938
C	5.891406	-2.206005	-0.067488
C	4.896366	-0.251795	1.168793
H	-4.155173	-2.303494	1.346393
H	-3.994501	0.381023	-0.013611
H	-0.854656	-0.755545	2.387538
H	-2.315936	-1.735307	2.600959
H	-3.218762	-3.297564	-0.899807
H	-2.145547	-3.008965	0.482507
H	-2.064394	-0.274520	3.572223
H	0.305880	0.687587	1.043931
H	-0.153631	0.344531	-1.949249
H	-0.407158	-1.670547	0.373307
H	2.946999	1.537259	-0.313142
H	2.689396	3.355320	-0.615293
H	-1.529970	1.880696	2.230309
H	-1.962973	1.356247	-0.747189
H	-3.003820	2.256377	1.334172
H	-2.724856	-1.909855	-3.032819
H	-1.851422	-0.373849	-2.906660
H	-3.451294	-0.582730	-2.151871
H	-5.759421	-1.334622	-0.067111

H	3.473431	-2.316792	-1.239329
H	3.966057	-0.640997	-1.393868
H	4.147995	-2.260126	1.195620
H	6.613911	-2.364762	0.738770
H	5.699297	-3.172126	-0.545639
H	6.360047	-1.549726	-0.810689
H	5.612906	-0.390594	1.984250
H	3.985315	0.182004	1.591180
H	5.332514	0.466554	0.462526

M062X energy = -1154.58580687 a.u.

(2*S*,6*R*,7*S*,8*S*)-**1**, Conf. X

C	-2.421030	-2.938611	-0.069339
C	-2.934339	-1.527871	-0.175936
C	-3.032857	-0.609052	0.790098
C	-2.918965	-0.886698	2.265780
C	-0.175838	-1.847620	-0.385003
C	-1.040693	-3.015035	-0.791457
C	-2.056156	1.351689	-0.469565
C	-0.640746	1.241991	0.166105
C	0.366036	0.604001	-0.816253
C	-0.066764	-0.792431	-1.201281
O	-2.271207	2.739688	-0.764510
C	-1.345047	3.538551	-0.178073
C	-0.318091	2.674972	0.479586
O	-1.394101	4.735187	-0.221881
C	0.681881	3.206721	1.175245
C	-3.200166	0.856923	0.423851
C	0.340526	-1.897428	1.028563
O	-3.304293	-3.909875	-0.597301
O	1.647721	0.642575	-0.174055
C	2.708404	0.496209	-1.005321
O	2.594445	0.374446	-2.197280
C	4.001264	0.463905	-0.231037
C	4.056840	-0.739650	0.725444
C	3.778190	-2.040907	-0.027211
C	5.413244	-0.786394	1.425737
H	-2.289108	-3.222375	0.977696
H	-3.067405	-1.191836	-1.209855
H	-2.070892	-0.347013	2.707019
H	-2.815024	-1.947586	2.500334
H	-1.231167	-2.982388	-1.872134
H	-0.586359	-3.985551	-0.562086
H	-3.818080	-0.518396	2.774222
H	-0.672355	0.622433	1.068609
H	0.425919	1.221941	-1.718829
H	-0.475148	-0.879865	-2.206563
H	1.438908	2.595421	1.654837
H	0.746769	4.287641	1.265876
H	-3.216375	1.476602	1.329092
H	-2.091423	0.826791	-1.428219
H	-4.144430	1.040125	-0.100946
H	0.946268	-2.800951	1.171520
H	-0.491760	-1.961329	1.741087
H	0.953809	-1.032627	1.283530
H	-3.514892	-3.652965	-1.507279

H	4.813056	0.407702	-0.962291
H	4.103542	1.397516	0.334337
H	3.276087	-0.598806	1.483943
H	3.812340	-2.898256	0.652792
H	2.794036	-2.028566	-0.510778
H	4.530321	-2.198193	-0.809222
H	5.455393	-1.614150	2.140379
H	5.616550	0.142329	1.968744
H	6.216757	-0.933391	0.694804

M062X energy = -1154.58551595 a.u.

(2*S*,6*R*,7*S*,8*S*)-**1**, Conf. Y

C	-4.000379	-1.622090	-0.013121
C	-3.669978	-0.165671	-0.204798
C	-3.279379	0.710905	0.721879
C	-3.349390	0.461431	2.203288
C	-1.478563	-1.889529	-0.176199
C	-2.741528	-2.433233	0.441016
C	-1.280689	1.731829	-0.427054
C	-0.095867	1.282198	0.482361
C	0.357098	-0.149859	0.129838
C	-0.696618	-1.113600	0.579722
O	-0.870652	2.942399	-1.081242
C	0.390037	3.315466	-0.760799
C	0.938517	2.344872	0.231614
O	0.920492	4.286490	-1.222671
C	2.140473	2.525580	0.769906
C	-2.620810	2.001828	0.269706
C	-1.296145	-2.107178	-1.652263
O	-4.470778	-2.073678	-1.273038
O	1.590288	-0.390581	0.831592
C	2.318756	-1.425602	0.359042
O	1.988498	-2.079295	-0.600886
C	3.592286	-1.628486	1.143750
C	4.838924	-1.203859	0.338573
C	5.066182	-2.085564	-0.889252
C	4.767112	0.273347	-0.048364
H	-4.780090	-1.757041	0.752504
H	-3.598770	0.115055	-1.256806
H	-3.909398	1.265885	2.694745
H	-2.349594	0.454953	2.656480
H	-2.908004	-3.486475	0.175510
H	-2.668527	-2.378333	1.532951
H	-3.837666	-0.485850	2.446212
H	-0.398768	1.291172	1.537113
H	0.553862	-0.218425	-0.943957
H	-0.918514	-1.035664	1.645500
H	2.542065	1.848338	1.514779
H	2.737975	3.377373	0.455825
H	-2.452931	2.684534	1.111702
H	-1.441681	0.987719	-1.216613
H	-3.252896	2.530009	-0.452396
H	-0.277925	-1.880150	-1.974089
H	-2.012837	-1.508888	-2.228569
H	-1.503317	-3.153888	-1.900149
H	-4.697880	-3.010171	-1.191160

H	3.534866	-1.059173	2.075633
H	3.658806	-2.694151	1.385964
H	5.691309	-1.338427	1.017171
H	6.010668	-1.819467	-1.374449
H	5.109593	-3.144559	-0.615222
H	4.259283	-1.964105	-1.616835
H	5.665881	0.574486	-0.595151
H	4.678525	0.914275	0.835166
H	3.902921	0.471555	-0.694955

M062X energy = -1154.58550445 a.u.

(1*R*,5*S*,6*S*,7*S*,10*R*)-**2**, Conf. A

C	3.448252	0.033245	-0.191323
C	2.923867	1.396321	-0.669328
C	1.554551	1.662289	-0.093930
C	0.557594	0.568833	-0.425924
C	1.050250	-0.774097	0.185422
C	2.432533	-1.073804	-0.446661
C	-0.895856	0.889547	-0.070044
C	-1.836248	-0.239096	-0.499447
C	-1.394348	-1.563414	0.131111
C	0.063684	-1.887042	-0.208160
C	-3.316520	0.125518	-0.252617
C	-4.252852	-1.007889	-0.676912
C	-3.606128	0.548867	1.189573
O	-1.340297	2.062202	-0.732423
C	1.306442	2.710883	0.694833
O	3.003987	-2.273503	0.042356
H	0.577260	0.443268	-1.523179
C	1.176756	-0.708611	1.713487
H	3.670132	0.067712	0.881127
H	4.380926	-0.223096	-0.703273
H	3.622327	2.192678	-0.395267
H	2.855220	1.383902	-1.766476
H	2.277189	-1.152154	-1.538474
H	-0.970410	1.019674	1.023243
H	-1.714147	-0.335989	-1.590818
H	-2.029324	-2.377771	-0.232570
H	-1.530812	-1.529533	1.219369
H	0.137781	-2.056197	-1.293749
H	0.352760	-2.823841	0.287037
H	-3.520571	0.990745	-0.892888
H	-4.205939	-1.850631	0.022010
H	-5.290299	-0.659711	-0.698795
H	-4.002264	-1.383277	-1.675770
H	-4.677651	0.732265	1.320662
H	-3.079033	1.471794	1.447506
H	-3.317539	-0.228435	1.907364
H	-0.623893	2.712862	-0.696186
H	0.339013	2.878076	1.162276
H	2.083281	3.436826	0.918087
H	2.450049	-3.010508	-0.250629
H	1.723389	-1.587817	2.067249
H	1.713533	0.185003	2.041727
H	0.197646	-0.694726	2.200480

M062X energy = -737.357403973 a.u.

(1*R*,5*S*,6*S*,7*S*,10*R*)-**2**, Conf. B

C	3.447075	0.040556	-0.221262
C	2.914713	1.406515	-0.682852
C	1.554227	1.659249	-0.082583
C	0.557541	0.570723	-0.426769
C	1.043603	-0.782749	0.165299
C	2.432897	-1.082649	-0.449283
C	-0.897331	0.890602	-0.071598
C	-1.840820	-0.234423	-0.504463
C	-1.392616	-1.569567	0.101657
C	0.061152	-1.885341	-0.259114
C	-3.318567	0.129191	-0.241344
C	-4.259079	-0.997498	-0.674132
C	-3.597406	0.531636	1.209041
O	-1.337066	2.067799	-0.729337
C	1.314998	2.689205	0.732859
O	2.951940	-2.328304	-0.018590
H	0.579182	0.455807	-1.524814
C	1.142158	-0.733183	1.696819
H	3.692107	0.093762	0.848681
H	4.372687	-0.212979	-0.747903
H	3.616198	2.202331	-0.414612
H	2.828828	1.399462	-1.778444
H	2.286641	-1.201463	-1.532090
H	-0.974970	1.019283	1.021162
H	-1.727927	-0.317329	-1.597901
H	-2.031374	-2.376275	-0.272345
H	-1.523007	-1.556744	1.191690
H	0.129638	-2.007668	-1.350057
H	0.378629	-2.836558	0.180842
H	-3.527330	1.003575	-0.867686
H	-4.206497	-1.850089	0.012215
H	-5.296516	-0.648528	-0.682868
H	-4.016269	-1.358841	-1.679981
H	-4.668402	0.709251	1.352114
H	-3.071887	1.452998	1.476098
H	-3.299710	-0.254859	1.913035
H	-0.627455	2.724051	-0.667755
H	0.352005	2.844471	1.213433
H	2.092521	3.412613	0.962167
H	3.266699	-2.215627	0.890188
H	1.806541	0.062348	2.045570
H	0.167434	-0.559492	2.159914
H	1.507975	-1.693066	2.079122

M062X energy = -737.356863028 a.u.

(1*R*,5*S*,6*S*,7*S*,10*R*)-**2**, Conf. C

C	3.442432	0.040039	-0.188125
C	2.920300	1.401880	-0.673068
C	1.552986	1.662674	-0.090976
C	0.557737	0.569820	-0.428100
C	1.044814	-0.779436	0.175558
C	2.426702	-1.075603	-0.440851
C	-0.896381	0.888538	-0.071112

C	-1.838103	-0.237974	-0.503889
C	-1.391930	-1.570222	0.108604
C	0.064368	-1.889053	-0.239716
C	-3.316847	0.125984	-0.246427
C	-4.255708	-1.003512	-0.675476
C	-3.599171	0.536013	1.201136
O	-1.339378	2.064482	-0.730097
C	1.308934	2.704708	0.707513
O	2.878647	-2.310852	0.086333
H	0.577222	0.449618	-1.525623
C	1.153967	-0.721797	1.706410
H	3.662086	0.080363	0.884429
H	4.383138	-0.202629	-0.699873
H	3.619424	2.199417	-0.404083
H	2.847387	1.384358	-1.769765
H	2.277587	-1.167276	-1.532332
H	-0.971620	1.017030	1.021915
H	-1.721721	-0.323813	-1.596825
H	-2.027475	-2.379079	-0.266480
H	-1.529625	-1.553255	1.197377
H	0.139368	-2.027995	-1.328559
H	0.377763	-2.831786	0.220136
H	-3.524800	0.996966	-0.877760
H	-4.203743	-1.852610	0.015216
H	-5.293434	-0.655400	-0.688058
H	-4.010530	-1.369730	-1.679038
H	-4.670534	0.714435	1.340763
H	-3.073873	1.458638	1.464101
H	-3.302799	-0.246492	1.910007
H	-0.625989	2.717577	-0.682585
H	0.343354	2.867054	1.180329
H	2.086160	3.429577	0.933163
H	3.741663	-2.505338	-0.304731
H	1.737447	0.138680	2.044530
H	0.169129	-0.646226	2.175226
H	1.635651	-1.633480	2.069750

M062X energy = -737.356844470 a.u.

(1*R*,5*S*,6*S*,7*S*,10*R*)-**2**, Conf. D

C	3.397059	0.439211	-0.241887
C	2.652766	1.729605	-0.618983
C	1.280904	1.743737	0.009157
C	0.450680	0.531999	-0.370122
C	1.165003	-0.752082	0.140262
C	2.555100	-0.794653	-0.542520
C	-1.023947	0.610362	0.037104
C	-1.800245	-0.621292	-0.436963
C	-1.128301	-1.895446	0.087840
C	0.348072	-1.977316	-0.303562
C	-3.306022	-0.592023	-0.074689
C	-3.570039	-0.051753	1.333164
C	-4.147562	0.170710	-1.101176
O	-1.644510	1.748130	-0.541281
C	0.907842	2.687501	0.877313
O	3.319425	-1.918293	-0.144941
H	0.453378	0.477848	-1.473329
C	1.330249	-0.761485	1.665837
H	3.647472	0.444360	0.824813
H	4.339956	0.361779	-0.791995
H	3.229597	2.607603	-0.313262
H	2.547453	1.770706	-1.712464
H	2.376920	-0.831051	-1.633037
H	-1.083720	0.666849	1.137207
H	-1.719742	-0.628106	-1.535701
H	-1.657746	-2.767836	-0.311751
H	-1.236932	-1.947780	1.179157
H	0.412870	-2.066838	-1.399157
H	0.789414	-2.887659	0.124042
H	-3.641404	-1.639637	-0.094652
H	-2.967325	-0.562487	2.093095
H	-3.345943	1.019384	1.377193
H	-4.623143	-0.185981	1.599633
H	-3.868082	1.225981	-1.120511
H	-5.212349	0.092838	-0.855140
H	-4.004376	-0.238388	-2.107096
H	-1.027514	2.491452	-0.467481
H	-0.054499	2.673126	1.383368
H	1.574247	3.507730	1.129152
H	2.874968	-2.713600	-0.470307
H	2.008857	-1.571352	1.949545
H	1.743381	0.179233	2.038179
H	0.375597	-0.919303	2.175630

M062X energy = -737.356769754 a.u.

(1*R*,5*S*,6*S*,7*S*,10*R*)-**2**, Conf. E

C	3.394584	0.446649	-0.266897
C	2.643827	1.738332	-0.627246
C	1.282325	1.739242	0.022028
C	0.451257	0.534044	-0.372003
C	1.158810	-0.761280	0.117872
C	2.557631	-0.803750	-0.544990
C	-1.025155	0.611289	0.033012
C	-1.804904	-0.616385	-0.446498
C	-1.126861	-1.899309	0.052444
C	0.343521	-1.972577	-0.359215
C	-3.307316	-0.589589	-0.070717
C	-3.558862	-0.069480	1.346949
C	-4.157574	0.188426	-1.078418
O	-1.640651	1.755002	-0.539479
C	0.920696	2.663335	0.915639
O	3.273865	-1.978299	-0.207515
H	0.455677	0.492409	-1.475402
C	1.296159	-0.793096	1.646810
H	3.662532	0.472658	0.798547
H	4.330796	0.372126	-0.829316
H	3.223289	2.616045	-0.325339
H	2.522831	1.783303	-1.718560
H	2.397223	-0.877571	-1.629746
H	-1.088897	0.664455	1.132583
H	-1.734526	-0.609893	-1.545869
H	-1.661815	-2.763507	-0.357557
H	-1.226868	-1.972249	1.144018

H	0.396222	-2.014204	-1.457065
H	0.812869	-2.890396	0.010190
H	-3.643572	-1.636509	-0.102369
H	-4.608795	-0.210335	1.622440
H	-2.946881	-0.589087	2.093499
H	-3.337875	1.001816	1.404081
H	-5.220265	0.108160	-0.823968
H	-4.023829	-0.206507	-2.091215
H	-3.877280	1.243694	-1.085645
H	-1.029970	2.500502	-0.439096
H	-0.035995	2.636908	1.431868
H	1.588304	3.480148	1.175519
H	3.593657	-1.875295	0.700668
H	0.321168	-0.791247	2.141097
H	1.807416	-1.711457	1.957475
H	1.850035	0.067299	2.032503

M062X energy = -737.356251442 a.u.

(1*R*,5*S*,6*S*,7*S*,10*R*)-**2**, Conf. F

C	3.391248	0.443978	-0.235385
C	2.649811	1.734160	-0.620043
C	1.280315	1.743222	0.013021
C	0.451413	0.533318	-0.373194
C	1.159828	-0.758122	0.128942
C	2.550770	-0.798262	-0.535342
C	-1.023900	0.609678	0.033952
C	-1.801775	-0.619304	-0.444858
C	-1.125763	-1.900176	0.060347
C	0.347929	-1.976826	-0.339119
C	-3.305346	-0.592121	-0.073344
C	-3.560704	-0.066073	1.341402
C	-4.153789	0.180801	-1.086456
O	-1.642820	1.751213	-0.540537
C	0.912153	2.679885	0.890746
O	3.202634	-1.977977	-0.097781
H	0.453999	0.485511	-1.476368
C	1.305967	-0.779774	1.657527
H	3.637350	0.454258	0.832121
H	4.340618	0.381141	-0.783448
H	3.226788	2.613564	-0.318570
H	2.542196	1.769794	-1.713390
H	2.382857	-0.846890	-1.626830
H	-1.084903	0.663865	1.133685
H	-1.728143	-0.615053	-1.544113
H	-1.657397	-2.765773	-0.351310
H	-1.233637	-1.970420	1.151026
H	0.409642	-2.034263	-1.436102
H	0.812696	-2.887949	0.051048
H	-3.640760	-1.639459	-0.101315
H	-4.611462	-0.205567	1.614669
H	-2.950570	-0.582437	2.091562
H	-3.339449	1.005356	1.394407
H	-5.217155	0.100026	-0.834827
H	-4.016502	-0.217728	-2.097422
H	-3.874868	1.236399	-1.096767
H	-1.029073	2.495701	-0.453699
H	-0.047985	2.660647	1.400699
H	1.579237	3.498503	1.146245
H	4.070045	-2.016543	-0.524016
H	0.334690	-0.873466	2.150966
H	1.921859	-1.634014	1.950940
H	1.772481	0.132608	2.038190

M062X energy = -737.356232023 a.u.

(1*R*,5*S*,6*S*,7*S*,10*R*)-**2**, Conf. G

C	3.458502	0.005252	-0.141182
C	2.958788	1.368076	-0.635499
C	1.573909	1.674790	-0.114297
C	0.564719	0.585792	-0.428384
C	1.046111	-0.767950	0.177167
C	2.435039	-1.081718	-0.432850
C	-0.888247	0.891768	-0.044012
C	-1.833460	-0.230953	-0.500704
C	-1.394707	-1.570969	0.097527
C	0.066591	-1.881104	-0.232515
C	-3.313501	0.124837	-0.243047
C	-4.250418	-0.993296	-0.704693
C	-3.602303	0.500436	1.212551
O	-1.326405	2.143353	-0.551235
C	1.314432	2.776696	0.588774
O	2.974947	-2.299172	0.049335
H	0.574238	0.446064	-1.527933
C	1.154665	-0.701512	1.707104
H	3.647197	0.034768	0.937770
H	4.402727	-0.263233	-0.625313
H	3.657122	2.160481	-0.349809
H	2.927142	1.348810	-1.735473
H	2.300489	-1.142066	-1.528932
H	-0.948987	0.994421	1.047133
H	-1.715042	-0.312044	-1.596569
H	-2.024676	-2.378703	-0.289450
H	-1.541536	-1.560602	1.184765
H	0.151840	-2.042363	-1.318817
H	0.356189	-2.819845	0.258181
H	-3.520113	1.012776	-0.850876
H	-4.001795	-1.335131	-1.716166
H	-4.201697	-1.859355	-0.035248
H	-5.288013	-0.645041	-0.712348
H	-4.673707	0.679759	1.349549
H	-3.075268	1.413708	1.502553
H	-3.314823	-0.301058	1.903384
H	-1.044171	2.202571	-1.476207
H	0.319115	3.018337	0.943191
H	2.106282	3.489919	0.803845
H	2.423061	-3.023288	-0.277061
H	1.646300	0.217018	2.038219
H	0.172279	-0.734026	2.186666
H	1.736799	-1.556194	2.064509

M062X energy = -737.354807713 a.u.

(1*R*,5*S*,6*S*,7*S*,10*R*)-**2**, Conf. H

C	3.452916	0.009980	-0.129820

C	2.958851	1.371877	-0.632931
C	1.573508	1.675805	-0.111562
C	0.566000	0.587928	-0.432840
C	1.040036	-0.772577	0.165028
C	2.429281	-1.082790	-0.426641
C	-0.887649	0.892090	-0.047334
C	-1.835239	-0.228394	-0.505282
C	-1.391660	-1.578332	0.068850
C	0.068173	-1.881947	-0.270345
C	-3.313328	0.125455	-0.232308
C	-4.253696	-0.988646	-0.696500
C	-3.589760	0.486199	1.229468
O	-1.323037	2.146086	-0.551926
C	1.316178	2.774332	0.597228
O	2.845101	-2.337980	0.084179
H	0.576650	0.454759	-1.532737
C	1.127679	-0.714793	1.697861
H	3.634049	0.043995	0.950219
H	4.407716	-0.244886	-0.609297
H	3.657575	2.164804	-0.349642
H	2.927735	1.347050	-1.732712
H	2.303820	-1.150645	-1.523402
H	-0.948169	0.996499	1.043125
H	-1.725446	-0.296599	-1.603324
H	-2.021863	-2.378282	-0.333837
H	-1.540942	-1.590106	1.155930
H	0.155422	-2.009164	-1.359971
H	0.382674	-2.828034	0.181379
H	-3.527115	1.019345	-0.829213
H	-4.013855	-1.320170	-1.713490
H	-4.197073	-1.861298	-0.036478
H	-5.291716	-0.641362	-0.690930
H	-4.660625	0.658852	1.379153
H	-3.064300	1.399367	1.522752
H	-3.290772	-0.320383	1.909337
H	-1.118708	2.167313	-1.499022
H	0.320908	3.015678	0.951731
H	2.108613	3.485813	0.816244
H	3.733273	-2.517075	-0.254433
H	1.650212	0.182529	2.040820
H	0.135783	-0.704663	2.157723
H	1.661894	-1.595308	2.064817

M062X energy = -737.354552551 a.u.

(1*R*,5*S*,6*S*,7*S*,10*R*)-**2**, Conf. I

C	3.458578	0.012492	-0.167257
C	2.951367	1.379108	-0.644298
C	1.573105	1.673294	-0.100655
C	0.565588	0.589034	-0.430035
C	1.039605	-0.775168	0.156814
C	2.435500	-1.088977	-0.435447
C	-0.889567	0.894604	-0.049763
C	-1.837830	-0.226422	-0.505166
C	-1.391718	-1.577474	0.065870
C	0.065679	-1.877950	-0.285907
C	-3.315173	0.127086	-0.228362
C	-4.256681	-0.985431	-0.694072
C	-3.589080	0.483690	1.234903
O	-1.320924	2.148188	-0.557404
C	1.319271	2.758075	0.629963
O	2.921653	-2.352641	-0.016537
H	0.580460	0.460818	-1.530509
C	1.117980	-0.726320	1.689538
H	3.668595	0.057814	0.910572
H	4.397048	-0.251935	-0.665615
H	3.652806	2.170212	-0.362274
H	2.904720	1.366435	-1.743436
H	2.308948	-1.186063	-1.523371
H	-0.954329	1.003253	1.039840
H	-1.730963	-0.294489	-1.603439
H	-2.024997	-2.376430	-0.333757
H	-1.533392	-1.591050	1.154220
H	0.145430	-1.989843	-1.377529
H	0.385338	-2.831541	0.146776
H	-3.529605	1.022746	-0.822406
H	-5.294460	-0.637593	-0.686257
H	-4.018398	-1.314502	-1.712177
H	-4.199737	-1.859759	-0.036289
H	-3.063847	1.396390	1.530154
H	-3.288900	-0.324919	1.911952
H	-4.659718	0.655461	1.386895
H	-1.123632	2.163917	-1.506131
H	0.326062	2.990620	0.996549
H	2.110686	3.469216	0.853521
H	3.259502	-2.251307	0.885071
H	1.532991	-1.665010	2.074038
H	1.732950	0.105491	2.044056
H	0.130864	-0.610144	2.144315

M062X energy = -737.354470376 a.u.

(1*R*,5*S*,6*S*,7*S*,10*R*)-**2**, Conf. J

C	3.413775	0.418682	-0.185520
C	2.691078	1.712797	-0.578247
C	1.293550	1.763360	-0.005881
C	0.455847	0.552038	-0.372889
C	1.162014	-0.743992	0.130607
C	2.564003	-0.795894	-0.526312
C	-1.016735	0.607647	0.057507
C	-1.792898	-0.617512	-0.449770
C	-1.122933	-1.905751	0.041507
C	0.356661	-1.968815	-0.334872
C	-3.298955	-0.600297	-0.091322
C	-3.565245	-0.125130	1.339574
C	-4.143347	0.207411	-1.080765
O	-1.642460	1.812828	-0.358998
C	0.893291	2.761217	0.781265
O	3.301227	-1.940612	-0.135881
H	0.454683	0.485552	-1.479230
C	1.303819	-0.759044	1.659041
H	3.629465	0.411184	0.888744
H	4.372595	0.332908	-0.706463
H	3.265767	2.586709	-0.257069

H	2.626735	1.756012	-1.676092
H	2.407348	-0.809160	-1.621040
H	-1.062767	0.637236	1.153557
H	-1.711848	-0.606753	-1.551339
H	-1.645407	-2.769552	-0.385494
H	-1.242207	-1.986121	1.129802
H	0.435219	-2.046272	-1.430805
H	0.799892	-2.880384	0.087561
H	-3.634981	-1.645429	-0.161283
H	-4.616852	-0.279808	1.600830
H	-2.958277	-0.666156	2.074546
H	-3.349596	0.944124	1.434614
H	-5.209540	0.088674	-0.859596
H	-3.977133	-0.130299	-2.109961
H	-3.897621	1.269989	-1.019262
H	-1.469555	1.922011	-1.306503
H	-0.113848	2.822605	1.176603
H	1.574124	3.572118	1.028013
H	2.859245	-2.721160	-0.497678
H	1.663118	0.199995	2.041196
H	0.351349	-0.968464	2.154324
H	2.016394	-1.537579	1.947513

M062X energy = -737.354251269 a.u.

(1*R*,5*S*,6*R*,7*R*,8*R*,10*R*)-**3**, Conf. A

C	4.441343	-0.712262	0.435056
C	4.334926	0.811203	0.600119
C	2.911989	1.246358	0.859625
C	1.946819	0.722156	-0.177876
C	1.976793	-0.846617	-0.207152
C	3.422007	-1.242935	-0.572772
C	0.499168	1.146969	-0.059316
C	-0.291464	0.684000	-1.286284
C	-0.389545	-0.830390	-1.335869
C	1.034543	-1.405674	-1.302934
O	0.282689	2.567248	-0.024125
C	-0.955962	2.837686	-0.532244
C	-1.472587	1.604103	-1.221369
O	3.468421	-2.655555	-0.666281
C	2.563508	1.998750	1.901094
C	-2.725109	1.506357	-1.654709
O	-1.482164	3.908301	-0.428243
H	2.307840	1.071422	-1.160924
C	1.611705	-1.447600	1.162487
O	-1.123767	-1.318716	-0.201811
C	-2.402041	-1.713340	-0.388526
O	-2.975673	-1.650915	-1.449060
C	-3.009552	-2.215878	0.897983
C	-3.707359	-1.092090	1.695020
C	-2.725664	-0.000435	2.123236
C	-4.879286	-0.496030	0.915505
H	4.280795	-1.214882	1.394726
H	5.451941	-0.979673	0.099112
H	4.680025	1.287952	-0.329352
H	4.994706	1.157725	1.400878

H	3.642104	-0.797045	-1.560327
H	0.055537	0.726024	0.852824
H	0.300107	0.982885	-2.166841
H	-0.908098	-1.160019	-2.239875
H	1.477978	-1.196119	-2.286591
H	0.985728	-2.494188	-1.208507
H	4.382218	-2.914067	-0.850586
H	3.308151	2.329567	2.620122
H	1.544145	2.335580	2.057504
H	-3.096359	0.614283	-2.149301
H	-3.402858	2.341701	-1.498784
H	1.876979	-2.508553	1.166070
H	0.542773	-1.368145	1.365528
H	2.147268	-0.950495	1.975095
H	-3.741640	-2.981902	0.628157
H	-2.223271	-2.668628	1.509027
H	-4.102223	-1.573989	2.598490
H	-2.380720	0.569584	1.253292
H	-3.208861	0.702234	2.808757
H	-1.846888	-0.420701	2.625038
H	-4.527179	0.003198	0.006437
H	-5.406449	0.244995	1.524112
H	-5.595186	-1.267648	0.615634

M062X energy = -1154.62605861 a.u.

(1*R*,5*S*,6*R*,7*R*,8*R*,10*R*)-**3**, Conf. B

C	4.454104	-0.690901	0.409558
C	4.324685	0.827202	0.605400
C	2.897656	1.238934	0.877759
C	1.939685	0.725410	-0.170949
C	1.976258	-0.841692	-0.219696
C	3.429757	-1.235620	-0.586183
C	0.491445	1.150138	-0.058072
C	-0.296031	0.692390	-1.288785
C	-0.387301	-0.822427	-1.348746
C	1.040325	-1.387692	-1.325632
O	0.275435	2.569962	-0.017002
C	-0.964135	2.840988	-0.523619
C	-1.479317	1.609553	-1.218295
O	3.574689	-2.633053	-0.760700
C	2.538755	1.962380	1.936018
C	-2.732227	1.511667	-1.650471
O	-1.492104	3.909999	-0.414047
H	2.307338	1.084307	-1.148220
C	1.602105	-1.456378	1.140112
O	-1.110968	-1.324049	-0.213085
C	-2.391858	-1.714942	-0.393308
O	-2.972717	-1.643463	-1.449032
C	-2.991796	-2.225068	0.893727
C	-3.693784	-1.107150	1.695431
C	-2.716680	-0.010655	2.121711
C	-4.871957	-0.516164	0.921497
H	4.320184	-1.197755	1.374964
H	5.455891	-0.949266	0.051615
H	4.659577	1.324681	-0.316571
H	4.983827	1.167691	1.409362

H	3.643100	-0.806901	-1.575787
H	0.042486	0.726924	0.850202
H	0.295349	0.999354	-2.166675
H	-0.908788	-1.148703	-2.252219
H	1.483578	-1.155474	-2.304033
H	1.002043	-2.478659	-1.256561
H	3.644175	-3.034840	0.117440
H	3.278267	2.287084	2.663048
H	1.515099	2.283583	2.098233
H	-3.102586	0.621532	-2.149141
H	-3.411118	2.345182	-1.489832
H	0.549472	-1.303636	1.382157
H	2.198426	-1.037371	1.954369
H	1.759252	-2.540722	1.108126
H	-3.720151	-2.994867	0.624500
H	-2.201015	-2.674646	1.501337
H	-4.082432	-1.593003	2.599458
H	-3.201665	0.689872	2.808107
H	-1.835070	-0.426567	2.622417
H	-2.376291	0.561049	1.251009
H	-5.583704	-1.291449	0.621314
H	-4.526085	-0.011555	0.013062
H	-5.401963	0.219139	1.534505

M062X energy = -1154.62581074 a.u.

(1*R*,5*S*,6*R*,7*R*,8*R*,10*R*)-**3**, Conf. C

C	4.357435	-1.006686	0.565304
C	4.339986	0.495993	0.882085
C	2.933855	1.005220	1.095857
C	2.010427	0.659610	-0.049269
C	1.941300	-0.895877	-0.242902
C	3.379915	-1.356488	-0.556181
C	0.588291	1.172104	0.024264
C	-0.146265	0.893370	-1.290492
C	-0.333579	-0.597477	-1.510830
C	1.045408	-1.270881	-1.449994
O	0.463058	2.593477	0.194820
C	-0.718880	3.001311	-0.355253
C	-1.268857	1.882336	-1.197238
O	3.340107	-2.751690	-0.797000
C	2.564185	1.672843	2.186645
C	-2.498016	1.915061	-1.701487
O	-1.179651	4.091168	-0.171273
H	2.459747	1.080761	-0.965494
C	1.442017	-1.605044	1.029148
O	-1.176575	-1.152161	-0.487645
C	-2.460528	-1.425751	-0.801364
O	-2.951056	-1.204325	-1.881888
C	-3.206451	-2.007328	0.372643
C	-3.997749	-0.922228	1.128214
C	-4.873931	-1.578787	2.193903
C	-3.065307	0.115748	1.753031
H	4.095397	-1.589762	1.454541
H	5.369673	-1.311986	0.268890
H	4.780067	1.037138	0.031316
H	4.963211	0.711901	1.754773

H	3.698232	-0.829057	-1.474375
H	0.057291	0.694611	0.858778
H	0.520809	1.240628	-2.096249
H	-0.805357	-0.793134	-2.477106
H	1.569533	-0.991641	-2.374889
H	0.921594	-2.357211	-1.477499
H	4.246724	-3.057661	-0.939353
H	3.277155	1.875010	2.981462
H	1.560881	2.065589	2.313622
H	-2.897861	1.104349	-2.301753
H	-3.127470	2.776441	-1.494157
H	0.369098	-1.469508	1.172150
H	1.950591	-1.233393	1.922234
H	1.636137	-2.677305	0.936717
H	-3.897720	-2.756073	-0.024632
H	-2.502944	-2.495128	1.055325
H	-4.645461	-0.420988	0.396777
H	-5.560873	-2.308804	1.754395
H	-4.253145	-2.099052	2.932878
H	-5.467797	-0.828590	2.724713
H	-2.495909	0.657577	0.992177
H	-2.352409	-0.368717	2.432091
H	-3.635771	0.849946	2.330054

M062X energy = -1154.62534577 a.u.

(1*R*,5*S*,6*R*,7*R*,8*R*,10*R*)-**3**, Conf. D

C	4.448593	-0.708132	0.437738
C	4.332503	0.813638	0.607371
C	2.909029	1.250711	0.860393
C	1.946341	0.723607	-0.178137
C	1.982791	-0.844372	-0.199021
C	3.435818	-1.239569	-0.568732
C	0.497916	1.146456	-0.062738
C	-0.290697	0.676956	-1.288691
C	-0.390882	-0.836966	-1.327491
C	1.034802	-1.411188	-1.286089
O	0.277249	2.566036	-0.034062
C	-0.960984	2.831976	-0.544977
C	-1.473735	1.594782	-1.230854
O	3.612958	-2.643167	-0.629909
C	2.555364	2.007381	1.897081
C	-2.724836	1.492338	-1.667021
O	-1.490649	3.901153	-0.446080
H	2.309316	1.070705	-1.161636
C	1.626333	-1.444627	1.171781
O	-1.122874	-1.318785	-0.191330
C	-2.401365	-1.718505	-0.377218
O	-2.969998	-1.668109	-1.440698
C	-3.010339	-2.209607	0.912475
C	-3.708645	-1.079086	1.699913
C	-2.727141	0.015016	2.122090
C	-4.879088	-0.488609	0.914027
H	4.289120	-1.215508	1.395182
H	5.451402	-0.982340	0.096193
H	4.682453	1.294780	-0.318173
H	4.986910	1.159660	1.412776

H	3.656982	-0.791222	-1.554625
H	0.054924	0.727248	0.850743
H	0.301972	0.970596	-2.170334
H	-0.907573	-1.173924	-2.229958
H	1.472583	-1.215306	-2.276916
H	0.962270	-2.499038	-1.170529
H	3.155069	-2.975477	-1.414252
H	3.297076	2.340372	2.618062
H	1.535586	2.345476	2.048170
H	-3.093363	0.597892	-2.159402
H	-3.404806	2.326760	-1.516021
H	1.931529	-2.495466	1.185922
H	0.554050	-1.400496	1.369004
H	2.141477	-0.924232	1.982611
H	-3.742429	-2.977437	0.647965
H	-2.224932	-2.657562	1.528188
H	-4.105055	-1.554640	2.605979
H	-3.210993	0.721787	2.802822
H	-1.848797	-0.401935	2.627335
H	-2.381577	0.579982	1.249113
H	-5.406903	0.257052	1.516278
H	-5.594895	-1.262081	0.618789
H	-4.525534	0.004140	0.001935

M062X energy = -1154.62534038 a.u.

(1*R*,5*S*,6*R*,7*R*,8*R*,10*R*)-**3**, Conf. E

C	4.370590	-0.989464	0.539153
C	4.328862	0.504783	0.892065
C	2.916513	0.989674	1.117793
C	2.002065	0.661921	-0.039025
C	1.939408	-0.889339	-0.259187
C	3.386165	-1.347919	-0.574118
C	0.580275	1.176335	0.030330
C	-0.150003	0.910005	-1.289189
C	-0.331586	-0.579379	-1.526291
C	1.051592	-1.243716	-1.476845
O	0.456658	2.595908	0.213379
C	-0.725130	3.008484	-0.334446
C	-1.273890	1.896863	-1.187341
O	3.449911	-2.725768	-0.893164
C	2.533230	1.622108	2.224714
C	-2.502730	1.933469	-1.692054
O	-1.186632	4.096183	-0.140762
H	2.459313	1.095431	-0.945694
C	1.429813	-1.616550	0.997141
O	-1.165042	-1.152377	-0.504773
C	-2.452225	-1.420549	-0.811958
O	-2.951147	-1.182646	-1.884793
C	-3.189151	-2.018956	0.359329
C	-3.985467	-0.946370	1.127146
C	-4.848048	-1.617278	2.194897
C	-3.057989	0.096498	1.751154
H	4.133724	-1.583614	1.432313
H	5.375995	-1.282298	0.219999
H	4.760286	1.070596	0.053375
H	4.950667	0.709448	1.768559

H	3.697537	-0.833076	-1.494232
H	0.042922	0.694097	0.857806
H	0.518271	1.267600	-2.089385
H	-0.806506	-0.766698	-2.492652
H	1.577217	-0.938445	-2.392371
H	0.938916	-2.330204	-1.534595
H	3.430538	-3.222512	-0.062332
H	3.239801	1.813024	3.027952
H	1.524367	1.998991	2.356944
H	-2.901677	1.128139	-2.300057
H	-3.132848	2.792543	-1.477333
H	0.374923	-1.414824	1.187554
H	1.992758	-1.330922	1.889172
H	1.518007	-2.699832	0.854989
H	-3.876254	-2.769518	-0.041803
H	-2.479480	-2.507531	1.035109
H	-4.642578	-0.446762	0.403124
H	-4.217453	-2.135491	2.927007
H	-5.445640	-0.875815	2.733676
H	-5.530805	-2.351482	1.755901
H	-2.335833	-0.385263	2.422495
H	-3.630532	0.822268	2.336634
H	-2.499382	0.648027	0.989139

M062X energy = -1154.62511660 a.u.

(1*R*,5*S*,6*R*,7*R*,8*R*,10*R*)-**3**, Conf. F

C	3.818744	-1.892340	0.073161
C	4.246208	-0.536014	0.653401
C	3.054439	0.289691	1.077028
C	2.032311	0.443987	-0.025017
C	1.511751	-0.964129	-0.486434
C	2.746177	-1.731008	-1.003503
C	0.820107	1.301600	0.265682
C	-0.006047	1.503164	-1.006858
C	-0.620659	0.198598	-1.474953
C	0.500634	-0.835552	-1.652573
O	1.102088	2.641300	0.704713
C	0.050577	3.444083	0.358596
C	-0.819417	2.697261	-0.614642
O	2.299496	-2.985095	-1.487055
C	2.929176	0.806294	2.297598
C	-2.032045	3.113374	-0.961573
O	-0.088127	4.554025	0.785468
H	2.551549	0.895390	-0.888232
C	0.872499	-1.745126	0.675636
O	-1.564538	-0.250748	-0.488697
C	-2.877755	-0.199416	-0.799745
O	-3.300892	0.185195	-1.861881
C	-3.743372	-0.637851	0.359331
C	-3.152216	-1.723347	1.264496
C	-4.130511	-2.035335	2.396988
C	-2.816299	-2.986586	0.472113
H	3.427781	-2.541986	0.863560
H	4.689558	-2.402547	-0.359312
H	4.795335	0.018035	-0.122353
H	4.933689	-0.676693	1.492544

H	3.171495	-1.140831	-1.836111
H	0.201019	0.828344	1.039248
H	0.699070	1.823543	-1.790488
H	-1.157071	0.330128	-2.417455
H	1.047461	-0.544232	-2.560547
H	0.064128	-1.818239	-1.856191
H	3.074208	-3.491718	-1.768074
H	3.696497	0.644206	3.049842
H	2.086393	1.428379	2.579849
H	-2.654324	2.558385	-1.657611
H	-2.419497	4.033738	-0.533440
H	-0.119587	-1.360581	0.917298
H	1.483454	-1.697086	1.580658
H	0.762377	-2.793156	0.381575
H	-3.948457	0.267538	0.946906
H	-4.698151	-0.960153	-0.069134
H	-2.227275	-1.329916	1.703299
H	-4.363675	-1.141159	2.984045
H	-5.071196	-2.430118	1.995471
H	-3.714692	-2.787111	3.074554
H	-2.396984	-3.755962	1.127965
H	-2.083896	-2.783707	-0.315748
H	-3.719224	-3.398003	0.005111

M062X energy = -1154.62468123 a.u.

(1*R*,5*S*,6*R*,7*R*,8*R*,10*R*)-**3**, Conf. G

C	4.367915	-0.994585	0.571452
C	4.336601	0.507098	0.889569
C	2.929169	1.015515	1.096263
C	2.009163	0.662846	-0.049641
C	1.949640	-0.893702	-0.231744
C	3.397496	-1.350288	-0.547591
C	0.585164	1.170521	0.019072
C	-0.146324	0.881597	-1.295273
C	-0.333479	-0.610528	-1.502187
C	1.047793	-1.281203	-1.430670
O	0.452789	2.592019	0.181000
C	-0.729330	2.992303	-0.373650
C	-1.272594	1.867070	-1.212020
O	3.490150	-2.749515	-0.744015
C	2.553017	1.688676	2.181481
C	-2.499650	1.893004	-1.721625
O	-1.195976	4.080603	-0.196656
H	2.459683	1.081170	-0.966995
C	1.460308	-1.601756	1.043233
O	-1.173876	-1.158348	-0.475495
C	-2.458117	-1.438250	-0.789521
O	-2.942891	-1.232496	-1.875367
C	-3.206568	-2.004616	0.389652
C	-3.998058	-0.909926	1.131789
C	-4.878919	-1.554913	2.200705
C	-3.065786	0.132174	1.749876
H	4.108321	-1.581529	1.458961
H	5.373026	-1.303631	0.268640
H	4.780074	1.051747	0.042636
H	4.953548	0.725416	1.766142

H	3.715343	-0.821686	-1.464946
H	0.055624	0.694302	0.855498
H	0.521448	1.223512	-2.102787
H	-0.802920	-0.816565	-2.467519
H	1.565642	-1.015634	-2.365025
H	0.900634	-2.367676	-1.436267
H	3.064550	-2.969542	-1.584087
H	3.262427	1.895948	2.978100
H	1.548678	2.080688	2.302664
H	-2.894968	1.078575	-2.319865
H	-3.132614	2.753409	-1.521113
H	0.381828	-1.502764	1.175858
H	1.948935	-1.202301	1.935081
H	1.696931	-2.667494	0.967051
H	-3.898167	-2.756833	-0.000345
H	-2.505036	-2.485345	1.079336
H	-4.642494	-0.414801	0.393335
H	-5.566074	-2.287500	1.765888
H	-4.261630	-2.069353	2.946606
H	-5.472813	-0.798443	2.722382
H	-2.493720	0.666909	0.986048
H	-2.355466	-0.347001	2.435291
H	-3.637007	0.872092	2.318690

M062X energy = -1154.62462262 a.u.

(1*R*,5*S*,6*R*,7*R*,8*R*,10*R*)-**3**, Conf. H

C	3.520803	-2.464823	-0.047584
C	4.255569	-1.158026	0.285984
C	3.304361	-0.092771	0.778035
C	2.147956	0.136039	-0.167252
C	1.334220	-1.192132	-0.364120
C	2.319937	-2.218245	-0.959991
C	1.159596	1.223453	0.193850
C	0.168194	1.444988	-0.951093
C	-0.719572	0.232074	-1.154858
C	0.177011	-0.995385	-1.374478
O	1.730045	2.525437	0.407860
C	0.781887	3.469529	0.125963
C	-0.355132	2.803007	-0.598080
O	1.598333	-3.411758	-1.207071
C	3.475716	0.554678	1.928576
C	-1.519477	3.404631	-0.812577
O	0.905727	4.623140	0.421586
H	2.578685	0.400020	-1.148773
C	0.782439	-1.725226	0.970288
O	-1.540093	0.039386	0.009143
C	-2.859736	0.309033	-0.091299
O	-3.386111	0.721933	-1.096809
C	-3.584946	-0.025684	1.187736
C	-4.145646	-1.465630	1.164500
C	-5.181260	-1.648982	0.055098
C	-3.030827	-2.506861	1.057331
H	3.171974	-2.953979	0.868094
H	4.210227	-3.162910	-0.540676
H	4.746487	-0.790757	-0.627484
H	5.044406	-1.338041	1.022134

H	2.686885	-1.800460	-1.915691
H	0.616976	0.950922	1.108443
H	0.770183	1.551832	-1.867857
H	-1.381463	0.369499	-2.013294
H	0.605364	-0.889208	-2.381251
H	-0.437499	-1.900614	-1.392911
H	2.221706	-4.078676	-1.527180
H	4.323457	0.330167	2.570355
H	2.806641	1.343957	2.254665
H	-2.337479	2.910129	-1.327667
H	-1.664271	4.420156	-0.454417
H	-0.064859	-1.133507	1.319700
H	1.544886	-1.719012	1.753398
H	0.435590	-2.752822	0.826019
H	-2.899392	0.093178	2.031471
H	-4.408039	0.686780	1.289362
H	-4.648354	-1.602402	2.130358
H	-5.989127	-0.915411	0.138544
H	-4.724844	-1.526178	-0.932161
H	-5.620331	-2.650146	0.105176
H	-2.518218	-2.439662	0.090893
H	-3.441773	-3.517133	1.146356
H	-2.277600	-2.372831	1.840538

M062X energy = -1154.62435534 a.u.

(1*R*,5*S*,6*R*,7*R*,8*R*,10*R*)-**3**, Conf. I

C	3.819969	-1.909390	0.044812
C	4.241791	-0.560356	0.646064
C	3.049433	0.257545	1.081592
C	2.031973	0.428938	-0.021566
C	1.500623	-0.970859	-0.492842
C	2.731680	-1.752927	-1.017045
C	0.830971	1.302359	0.269153
C	0.004335	1.513782	-1.001306
C	-0.621780	0.214852	-1.471202
C	0.493271	-0.823673	-1.658361
O	1.130708	2.637730	0.708058
C	0.086411	3.452359	0.366394
C	-0.795137	2.716110	-0.604836
O	2.369655	-3.003733	-1.572535
C	2.918223	0.753532	2.310044
C	-2.003580	3.147521	-0.947696
O	-0.039061	4.562931	0.795314
H	2.559113	0.876289	-0.882294
C	0.849158	-1.747179	0.663763
O	-1.561681	-0.236510	-0.481168
C	-2.877398	-0.163341	-0.779512
O	-3.304531	0.241697	-1.832103
C	-3.738191	-0.609229	0.380214
C	-3.161793	-1.733517	1.246856
C	-4.135500	-2.059106	2.379416
C	-2.860022	-2.978790	0.413380
H	3.449237	-2.566485	0.843536
H	4.678475	-2.414015	-0.410006
H	4.789083	0.004682	-0.122612
H	4.930771	-0.711760	1.482196

H	3.145485	-1.181343	-1.860071
H	0.206049	0.839080	1.043858
H	0.711005	1.828726	-1.785735
H	-1.162983	0.353645	-2.409865
H	1.044316	-0.524991	-2.561152
H	0.053370	-1.801417	-1.878686
H	2.249733	-3.628457	-0.842708
H	3.682072	0.579839	3.063196
H	2.074592	1.372006	2.598160
H	-2.634758	2.601596	-1.642739
H	-2.378047	4.072059	-0.516978
H	-0.103068	-1.305936	0.961603
H	1.493366	-1.786493	1.545709
H	0.627840	-2.771305	0.340492
H	-3.911793	0.284328	0.995225
H	-4.706366	-0.896456	-0.042875
H	-2.224583	-1.372923	1.687779
H	-4.344396	-1.178131	2.994795
H	-5.087903	-2.422274	1.975840
H	-3.729257	-2.839056	3.030464
H	-2.451680	-3.776741	1.041662
H	-2.131733	-2.766034	-0.375927
H	-3.775278	-3.357138	-0.057321

M062X energy = -1154.62434916 a.u.

(1*R*,5*S*,6*R*,7*R*,8*R*,10*R*)-**3**, Conf. J

C	3.532308	-2.455506	-0.077364
C	4.250781	-1.147090	0.284489
C	3.288189	-0.099023	0.789811
C	2.139754	0.137708	-0.162366
C	1.328100	-1.188193	-0.375273
C	2.318215	-2.218634	-0.975351
C	1.153337	1.228222	0.195218
C	0.165164	1.454067	-0.951485
C	-0.721209	0.240982	-1.162313
C	0.179220	-0.981257	-1.391237
O	1.726826	2.527721	0.413200
C	0.779870	3.474187	0.133297
C	-0.357474	2.811296	-0.594044
O	1.679492	-3.437029	-1.310045
C	3.441938	0.527946	1.954074
C	-1.521009	3.414930	-0.807266
O	0.904625	4.626459	0.433096
H	2.578753	0.403616	-1.139863
C	0.764751	-1.725816	0.951161
O	-1.537675	0.035587	0.002982
C	-2.857254	0.310679	-0.089282
O	-3.386187	0.734982	-1.088307
C	-3.578695	-0.034586	1.189182
C	-4.126841	-1.479224	1.162682
C	-5.150973	-1.672533	0.044352
C	-3.002647	-2.511557	1.066726
H	3.205184	-2.960130	0.842030
H	4.214589	-3.140750	-0.590642
H	4.741863	-0.760357	-0.620468
H	5.038508	-1.332925	1.020485

H	2.667868	-1.813074	-1.935512
H	0.606272	0.958113	1.107760
H	0.769522	1.564339	-1.866251
H	-1.385611	0.383017	-2.018006
H	0.616273	-0.860281	-2.392373
H	-0.431679	-1.888445	-1.431771
H	1.580329	-3.952077	-0.496233
H	4.284763	0.298998	2.600695
H	2.763798	1.306869	2.286757
H	-2.339159	2.923273	-1.324777
H	-1.664883	4.429398	-0.445761
H	1.537044	-1.806978	1.720247
H	0.321899	-2.715515	0.786960
H	-0.034147	-1.093643	1.341166
H	-2.894404	0.088675	2.033439
H	-4.408096	0.670120	1.292535
H	-4.636601	-1.619551	2.124272
H	-5.584354	-2.676191	0.093206
H	-5.963954	-0.943708	0.118186
H	-4.686576	-1.549781	-0.939250
H	-2.480631	-2.438464	0.105671
H	-3.406664	-3.525056	1.151170
H	-2.259700	-2.370834	1.858850

M062X energy = -1154.62413421 a.u.

(1*R*,5*S*,6*R*,7*R*,8*R*,10*R*)-**3**, Conf. K

C	2.461539	-3.304479	0.074692
C	3.550537	-2.348917	0.583986
C	2.969034	-1.026997	1.026223
C	2.108886	-0.385600	-0.037431
C	0.919290	-1.334499	-0.425455
C	1.559856	-2.632184	-0.960474
C	1.521870	0.973892	0.272234
C	0.832787	1.552010	-0.966501
C	-0.379591	0.738126	-1.360565
C	0.057275	-0.723376	-1.557723
O	2.476761	1.988191	0.631622
C	1.963154	3.210352	0.299901
C	0.766388	2.997693	-0.585826
O	0.509991	-3.489367	-1.370367
C	3.190709	-0.505180	2.230756
C	-0.099442	3.962058	-0.868730
O	2.440936	4.242132	0.674788
H	2.737067	-0.271697	-0.937997
C	0.033963	-1.666992	0.789885
O	-1.346705	0.886161	-0.308166
C	-2.612017	0.525617	-0.603664
O	-2.954143	0.146467	-1.696799
C	-3.526405	0.667351	0.590873
C	-4.611150	-0.413231	0.607884
C	-3.985113	-1.800919	0.751327
C	-5.607176	-0.141760	1.733656
H	1.840578	-3.656769	0.905214
H	2.926374	-4.191540	-0.375523
H	4.260305	-2.159959	-0.235042
H	4.117264	-2.810716	1.397722

H	2.177340	-2.353757	-1.834313
H	0.802228	0.896385	1.097262
H	1.555408	1.460988	-1.793120
H	-0.830681	1.105783	-2.287548
H	0.634348	-0.749412	-2.493075
H	-0.823632	-1.349409	-1.723537
H	0.900976	-4.321316	-1.671640
H	3.808751	-1.027164	2.956311
H	2.797721	0.462420	2.524705
H	-0.970140	3.789188	-1.494207
H	0.055047	4.958325	-0.464839
H	-0.592393	-0.820436	1.077451
H	0.631344	-1.948784	1.660796
H	-0.623565	-2.502482	0.532167
H	-2.928170	0.640786	1.508367
H	-3.981432	1.664994	0.534169
H	-5.133102	-0.366352	-0.355035
H	-3.421238	-1.873778	1.689787
H	-4.756178	-2.577332	0.762179
H	-3.302961	-2.017735	-0.077107
H	-6.078279	0.840618	1.625323
H	-5.105744	-0.169700	2.708398
H	-6.398316	-0.897614	1.744222

M062X energy = -1154.62408929 a.u.

(1*R*,5*S*,6*R*,7*R*,8*R*,10*R*)-**3**, Conf. L

C	2.462423	-3.308519	0.079102
C	3.540525	-2.350092	0.604586
C	2.959545	-1.025664	1.040112
C	2.108765	-0.383441	-0.030701
C	0.924826	-1.335679	-0.423224
C	1.573547	-2.637808	-0.960729
C	1.519391	0.976176	0.273968
C	0.836498	1.553171	-0.969203
C	-0.375491	0.740669	-1.365669
C	0.064492	-0.722364	-1.554988
O	2.470845	1.992099	0.636820
C	1.958943	3.213537	0.300869
C	0.767560	2.999638	-0.592088
O	0.609774	-3.595770	-1.356129
C	3.172861	-0.500142	2.244596
C	-0.095768	3.963832	-0.882856
O	2.433202	4.246302	0.676864
H	2.744187	-0.270825	-0.926765
C	0.034000	-1.676109	0.784192
O	-1.346823	0.891679	-0.319526
C	-2.604942	0.501622	-0.610133
O	-2.931405	0.080071	-1.693023
C	-3.528938	0.669611	0.572741
C	-4.611552	-0.412473	0.610269
C	-3.984465	-1.794210	0.799037
C	-5.617878	-0.111151	1.719228
H	1.831575	-3.665635	0.900174
H	2.920658	-4.191743	-0.376366
H	4.263007	-2.161729	-0.203546
H	4.095574	-2.811974	1.426315

H	2.201074	-2.357251	-1.826503
H	0.795089	0.898057	1.095123
H	1.562460	1.460040	-1.792698
H	-0.822340	1.103924	-2.296635
H	0.639755	-0.745671	-2.493346
H	-0.832038	-1.331867	-1.712216
H	0.163318	-3.272398	-2.151023
H	3.783470	-1.022037	2.976410
H	2.780662	0.469457	2.533117
H	-0.962614	3.790973	-1.513667
H	0.056993	4.960694	-0.479811
H	-0.619598	-0.845075	1.057211
H	0.629074	-1.933860	1.663714
H	-0.594377	-2.535173	0.528221
H	-2.936839	0.668960	1.494609
H	-3.985008	1.664801	0.487198
H	-5.125425	-0.394008	-0.357966
H	-3.429895	-1.840145	1.744575
H	-4.753857	-2.571834	0.823382
H	-3.293234	-2.032555	-0.015853
H	-6.090104	0.866535	1.578539
H	-5.125000	-0.110069	2.698658
H	-6.407376	-0.868268	1.744446

M062X energy = -1154.62404411 a.u.

(1*R*,5*S*,6*R*,7*R*,8*R*,10*R*)-**3**, Conf. M

C	3.538416	-2.593436	0.175987
C	4.305115	-1.317300	0.552368
C	3.368551	-0.206327	0.964131
C	2.298637	0.064839	-0.067809
C	1.441244	-1.226549	-0.319637
C	2.421125	-2.305237	-0.826417
C	1.337398	1.198905	0.213152
C	0.438142	1.448916	-0.999602
C	-0.479142	0.277146	-1.269566
C	0.377263	-0.990643	-1.420187
O	1.948674	2.479925	0.448605
C	1.061702	3.458453	0.096548
C	-0.056444	2.825771	-0.685635
O	1.665345	-3.468358	-1.111124
C	3.480675	0.443738	2.120484
C	-1.196843	3.451156	-0.946915
O	1.213918	4.610699	0.383668
H	2.813180	0.300537	-1.015591
C	0.762449	-1.718416	0.971606
O	-1.398203	0.194030	-0.168485
C	-2.530622	-0.508319	-0.384346
O	-2.807498	-1.006721	-1.447234
C	-3.359281	-0.596160	0.875384
C	-4.788577	-1.075737	0.623662
C	-5.481721	-1.374498	1.952348
C	-5.577246	-0.043318	-0.182360
H	3.100387	-3.055282	1.067205
H	4.229625	-3.328095	-0.257729
H	4.877895	-0.982256	-0.325088
H	5.028643	-1.523686	1.346416

H	2.876911	-1.918827	-1.756666
H	0.722835	0.965319	1.091859
H	1.109273	1.529342	-1.869699
H	-1.063269	0.425390	-2.183016
H	0.887074	-0.906502	-2.390509
H	-0.274785	-1.865529	-1.491101
H	2.276426	-4.161530	-1.397236
H	4.266126	0.188203	2.826628
H	2.824617	1.264658	2.390181
H	-2.003278	2.973147	-1.495148
H	-1.341362	4.471370	-0.603676
H	-0.065298	-1.069473	1.262819
H	1.464506	-1.756259	1.808393
H	0.365067	-2.723433	0.803007
H	-2.829131	-1.283822	1.549237
H	-3.348038	0.383016	1.369484
H	-4.727348	-1.998802	0.035007
H	-4.944134	-2.137940	2.524207
H	-5.540330	-0.470101	2.569695
H	-6.502805	-1.731398	1.787119
H	-6.596118	-0.395180	-0.371089
H	-5.102380	0.150872	-1.147658
H	-5.647232	0.901757	0.370338

M062X energy = -1154.62393801 a.u.

(1*R*,5*S*,6*R*,7*R*,8*R*,10*R*)-**3**, Conf. N

C	3.505322	-2.630056	0.174036
C	4.280380	-1.362371	0.560194
C	3.360025	-0.236271	0.967735
C	2.296236	0.049676	-0.066687
C	1.427169	-1.232964	-0.315800
C	2.399032	-2.327464	-0.828582
C	1.348337	1.195236	0.212979
C	0.451896	1.455287	-1.000246
C	-0.481828	0.296178	-1.265842
C	0.360843	-0.983204	-1.410452
O	1.973480	2.469468	0.447092
C	1.099015	3.458272	0.093531
C	-0.026020	2.838776	-0.689630
O	1.738937	-3.551075	-1.093644
C	3.477849	0.415555	2.122591
C	-1.157244	3.478947	-0.954651
O	1.263886	4.608889	0.379356
H	2.815877	0.278456	-1.013776
C	0.746435	-1.722881	0.973559
O	-1.402164	0.228400	-0.166551
C	-2.522776	-0.497513	-0.368076
O	-2.776743	-1.040250	-1.415447
C	-3.365457	-0.550750	0.883493
C	-4.785852	-1.056927	0.632532
C	-5.490705	-1.316041	1.963403
C	-5.577294	-0.063998	-0.219126
H	3.057477	-3.092345	1.060252
H	4.176844	-3.373281	-0.266582
H	4.865601	-1.032799	-0.311254
H	4.994877	-1.579311	1.359615

H	2.864326	-1.943386	-1.754883
H	0.730948	0.968250	1.091714
H	1.123313	1.524682	-1.871116
H	-1.063113	0.446863	-2.180961
H	0.863162	-0.906792	-2.387031
H	-0.322895	-1.837426	-1.466492
H	1.173975	-3.433357	-1.869962
H	4.257005	0.149471	2.831740
H	2.833868	1.247190	2.388702
H	-1.969306	3.011723	-1.503810
H	-1.288235	4.501587	-0.613228
H	-0.094254	-1.086346	1.256115
H	1.444381	-1.743707	1.813992
H	0.370893	-2.737894	0.810719
H	-2.835233	-1.207413	1.587616
H	-3.371555	0.445326	1.342507
H	-4.706733	-2.000046	0.078766
H	-5.567400	-0.390735	2.546767
H	-6.505363	-1.691194	1.799462
H	-4.950906	-2.051701	2.568521
H	-5.094308	0.101535	-1.185782
H	-5.665007	0.899324	0.298301
H	-6.589590	-0.435049	-0.406138

M062X energy = -1154.62384650 a.u.

(1*R*,5*S*,6*R*,7*R*,8*R*,10*R*)-**3**, Conf. O

C	2.478213	-3.312240	0.065885
C	3.551135	-2.351156	0.598822
C	2.956026	-1.036876	1.044456
C	2.110578	-0.392648	-0.028529
C	0.922251	-1.337326	-0.426804
C	1.561377	-2.645331	-0.959952
C	1.533277	0.973556	0.270558
C	0.847676	1.550062	-0.970796
C	-0.366269	0.737131	-1.363490
C	0.074248	-0.721944	-1.565460
O	2.494796	1.982637	0.625262
C	1.985959	3.206556	0.291100
C	0.787213	2.996918	-0.593216
O	0.590523	-3.552189	-1.447430
C	3.153628	-0.522756	2.256433
C	-0.074728	3.964313	-0.877612
O	2.468070	4.237076	0.663376
H	2.747229	-0.287639	-0.924373
C	0.025071	-1.658713	0.781384
O	-1.328594	0.878715	-0.304953
C	-2.592466	0.505593	-0.590604
O	-2.938246	0.117208	-1.679217
C	-3.499136	0.652242	0.609495
C	-4.645648	-0.361193	0.592792
C	-4.104450	-1.787846	0.693626
C	-5.625465	-0.065912	1.726930
H	1.870543	-3.683395	0.902434
H	2.941946	-4.186651	-0.401897
H	4.268680	-2.150717	-0.210244
H	4.111852	-2.816287	1.414914

H	2.161074	-2.377218	-1.841407
H	0.812665	0.908743	1.095543
H	1.570765	1.454336	-1.796408
H	-0.822222	1.107522	-2.287012
H	0.664443	-0.740842	-2.492527
H	-0.801728	-1.349271	-1.750981
H	0.197059	-4.003140	-0.686342
H	3.761745	-1.046216	2.989233
H	2.751901	0.441637	2.549680
H	-0.946303	3.794010	-1.502573
H	0.084258	4.960796	-0.476012
H	-0.524336	-0.781975	1.128654
H	0.599531	-2.044641	1.627332
H	-0.725062	-2.404968	0.493540
H	-2.901874	0.558160	1.523639
H	-3.891687	1.677710	0.593816
H	-5.162734	-0.255053	-0.367969
H	-3.438770	-2.018459	-0.144165
H	-3.546046	-1.921301	1.628903
H	-4.920882	-2.516366	0.683880
H	-6.036306	0.945963	1.649717
H	-5.128272	-0.154381	2.700264
H	-6.460804	-0.772512	1.713964

M062X energy = -1154.62377681 a.u.

(1*R*,5*S*,6*R*,7*R*,8*R*,10*R*)-**3**, Conf. P

C	4.243543	-1.415938	0.411627
C	4.486736	0.084832	0.623608
C	3.206540	0.830226	0.918251
C	2.133167	0.576266	-0.114691
C	1.806858	-0.957112	-0.188936
C	3.125765	-1.665756	-0.592746
C	0.821384	1.310495	0.054602
C	-0.082318	1.078481	-1.159040
C	-0.510884	-0.367412	-1.269904
C	0.747530	-1.252161	-1.278956
O	0.918604	2.744138	0.129749
C	-0.257991	3.284990	-0.305837
C	-1.053344	2.203796	-0.985543
O	2.973040	-3.068834	-0.695640
C	3.051339	1.611818	1.984863
C	-2.341058	2.332616	-1.278914
O	-0.544743	4.436965	-0.152258
H	2.548763	0.867724	-1.095310
C	1.328223	-1.507632	1.165272
O	-1.373777	-0.635102	-0.156854
C	-2.173120	-1.721248	-0.259146
O	-2.149916	-2.468738	-1.204960
C	-3.100814	-1.807432	0.925046
C	-4.064607	-0.607453	0.965074
C	-4.906376	-0.547724	-0.309782
C	-4.954679	-0.692349	2.203171
H	3.978322	-1.900122	1.357643
H	5.151427	-1.906317	0.047320
H	4.925711	0.500196	-0.295798
H	5.214417	0.245529	1.424428

H	3.435190	-1.248973	-1.568840
H	0.314883	0.970109	0.967079
H	0.529916	1.299428	-2.047928
H	-1.081617	-0.550908	-2.186270
H	1.207346	-1.113427	-2.269614
H	0.427200	-2.297846	-1.215486
H	2.413645	-3.262003	-1.460848
H	3.859507	1.739603	2.699925
H	2.139823	2.171075	2.167861
H	-2.907400	1.535279	-1.751436
H	-2.855941	3.259543	-1.043452
H	1.377697	-2.600504	1.139314
H	0.298130	-1.217160	1.380584
H	1.953732	-1.152519	1.987942
H	-3.656516	-2.746033	0.842502
H	-2.500663	-1.832510	1.842052
H	-3.454926	0.302884	1.026275
H	-4.283239	-0.494938	-1.209575
H	-5.533453	-1.442529	-0.398376
H	-5.563781	0.327110	-0.301322
H	-4.360690	-0.711201	3.122311
H	-5.566009	-1.601895	2.175822
H	-5.632482	0.165147	2.254788

M062X energy = -1154.62371826 a.u.

(1*R*,5*S*,6*R*,7*R*,8*R*,10*R*)-**3**, Conf. Q

C	4.250696	-1.393380	0.419588
C	4.490511	0.109698	0.622143
C	3.203626	0.843236	0.917402
C	2.132668	0.579379	-0.115413
C	1.812572	-0.956304	-0.192164
C	3.130627	-1.653935	-0.587214
C	0.815994	1.305357	0.051433
C	-0.083078	1.067282	-1.163983
C	-0.499611	-0.382500	-1.278144
C	0.762647	-1.259270	-1.289625
O	0.905328	2.739708	0.127410
C	-0.274086	3.273016	-0.310627
C	-1.061132	2.186674	-0.991448
O	2.859198	-3.037524	-0.716488
C	3.043787	1.622646	1.984854
C	-2.349221	2.307146	-1.286979
O	-0.567452	4.423562	-0.157300
H	2.546396	0.872754	-1.095870
C	1.329063	-1.504347	1.162807
O	-1.361712	-0.657436	-0.164422
C	-2.188620	-1.720270	-0.285860
O	-2.202913	-2.439416	-1.252955
C	-3.100402	-1.816820	0.910604
C	-4.053213	-0.609357	0.979818
C	-4.922418	-0.533840	-0.275678
C	-4.917088	-0.694833	2.236324
H	3.987888	-1.871830	1.369062
H	5.172458	-1.872652	0.064346
H	4.922389	0.522264	-0.301739
H	5.220256	0.278889	1.419300

H	3.436742	-1.240150	-1.565789
H	0.309173	0.962917	0.962708
H	0.529604	1.293647	-2.051155
H	-1.069709	-0.566809	-2.194438
H	1.230514	-1.113461	-2.273776
H	0.474182	-2.312627	-1.239107
H	3.687349	-3.487518	-0.934228
H	3.851726	1.757675	2.698915
H	2.127349	2.173391	2.168863
H	-2.908631	1.505754	-1.760876
H	-2.871144	3.230375	-1.052427
H	0.307263	-1.189540	1.381811
H	1.964823	-1.167403	1.985634
H	1.347753	-2.597340	1.130665
H	-3.666151	-2.748919	0.822423
H	-2.488509	-1.859446	1.819013
H	-3.434598	0.295496	1.033852
H	-5.567678	0.349712	-0.249716
H	-4.319125	-0.487626	-1.188969
H	-5.563322	-1.419954	-0.352298
H	-5.588025	0.166766	2.307167
H	-4.303607	-0.722739	3.142377
H	-5.534897	-1.600253	2.217263

M062X energy = -1154.62370475 a.u.

(1*R*,5*S*,6*R*,7*R*,8*R*,10*R*)-**3**, Conf. R

C	3.544793	-2.466938	-0.034404
C	4.270000	-1.155574	0.299921
C	3.318324	-0.086969	0.783546
C	2.161502	0.134436	-0.163295
C	1.354841	-1.198754	-0.347603
C	2.347580	-2.228766	-0.945654
C	1.170936	1.221759	0.191629
C	0.176760	1.432411	-0.953165
C	-0.711195	0.217857	-1.141528
C	0.187958	-1.011505	-1.348940
O	1.736997	2.527079	0.394861
C	0.786314	3.466536	0.107401
C	-0.349702	2.792042	-0.611199
O	1.743926	-3.489623	-1.171524
C	3.487206	0.570663	1.928724
C	-1.514902	3.390019	-0.830907
O	0.906326	4.622565	0.394273
H	2.591800	0.392597	-1.146953
C	0.812382	-1.731814	0.989278
O	-1.524757	0.030700	0.025897
C	-2.846620	0.296811	-0.070790
O	-3.376134	0.702150	-1.077479
C	-3.563467	-0.037654	1.212413
C	-4.182318	-1.452916	1.157633
C	-5.300511	-1.540086	0.119084
C	-3.117986	-2.523661	0.912857
H	3.195718	-2.957807	0.880359
H	4.223541	-3.166831	-0.531356
H	4.766298	-0.790046	-0.611494
H	5.055239	-1.330876	1.041081

H	2.715130	-1.813101	-1.901705
H	0.630748	0.953038	1.109040
H	0.776006	1.531691	-1.872638
H	-1.376230	0.344717	-1.999349
H	0.602310	-0.917738	-2.364532
H	-0.445413	-1.906909	-1.336902
H	1.132531	-3.407259	-1.916294
H	4.334315	0.351746	2.573184
H	2.817469	1.362164	2.248098
H	-2.332448	2.891024	-1.342313
H	-1.660915	4.408115	-0.480651
H	-0.056380	-1.165580	1.328908
H	1.570910	-1.692662	1.774781
H	0.505359	-2.773998	0.854443
H	-2.857594	0.029024	2.044659
H	-4.354348	0.704744	1.350653
H	-4.618734	-1.624796	2.149553
H	-5.761775	-2.532386	0.137777
H	-6.080540	-0.796875	0.311214
H	-4.913726	-1.361323	-0.888938
H	-2.293885	-2.447698	1.629534
H	-2.693044	-2.429852	-0.093770
H	-3.554585	-3.523473	0.995371

M062X energy = -1154.62359280 a.u.

(1*R*,5*S*,6*R*,7*R*,8*R*,10*R*)-**3**, Conf. S

C	3.134223	-2.688522	0.095036
C	3.934926	-1.490842	0.626228
C	3.027109	-0.359640	1.047237
C	2.060819	0.048057	-0.040557
C	1.162974	-1.170184	-0.461662
C	2.126019	-2.262023	-0.971354
C	1.134783	1.207899	0.253398
C	0.367093	1.602656	-1.009263
C	-0.583643	0.515052	-1.456948
C	0.203694	-0.795599	-1.619256
O	1.782103	2.429755	0.650774
C	0.983726	3.482440	0.300328
C	-0.082983	2.978678	-0.632400
O	1.338778	-3.354296	-1.409947
C	3.078070	0.196702	2.255730
C	-1.157121	3.689351	-0.950562
O	1.165069	4.597947	0.695324
H	2.663481	0.326422	-0.922488
C	0.356280	-1.714142	0.730423
O	-1.628123	0.422205	-0.474292
C	-2.820244	-0.043979	-0.913418
O	-3.007793	-0.412682	-2.045528
C	-3.843366	-0.046150	0.194880
C	-3.461615	-1.002350	1.336627
C	-4.535800	-0.971579	2.422442
C	-3.254649	-2.421727	0.809002
H	2.594986	-3.181979	0.910811
H	3.818153	-3.433141	-0.333179
H	4.594807	-1.128282	-0.175911
H	4.578582	-1.795905	1.456411

H	2.680380	-1.833988	-1.826846
H	0.430517	0.937683	1.051084
H	1.120455	1.708283	-1.806400
H	-1.051181	0.765583	-2.413569
H	0.791637	-0.689729	-2.542092
H	-0.495318	-1.618928	-1.795385
H	1.935724	-4.058016	-1.700094
H	3.789447	-0.154715	2.998273
H	2.445589	1.032167	2.536691
H	-1.927239	3.303616	-1.612251
H	-1.281861	4.687932	-0.542131
H	-0.496568	-1.072063	0.958766
H	0.967735	-1.793841	1.633128
H	-0.027087	-2.707138	0.478190
H	-3.939258	0.974486	0.582669
H	-4.797696	-0.344965	-0.248664
H	-2.519288	-0.643774	1.769739
H	-4.677134	0.039413	2.817626
H	-5.496272	-1.316291	2.022012
H	-4.265710	-1.627439	3.255710
H	-2.951571	-3.096627	1.615566
H	-2.482983	-2.460315	0.032478
H	-4.184392	-2.806463	0.373730

M062X energy = -1154.62356166 a.u.

(1*R*,5*S*,6*R*,7*R*,8*R*,10*R*)-**3**, Conf. T

C	3.536733	-2.602175	0.149504
C	4.290589	-1.328577	0.559443
C	3.345832	-0.229114	0.981858
C	2.290169	0.057514	-0.059793
C	1.429708	-1.226536	-0.331841
C	2.409988	-2.313592	-0.842629
C	1.337620	1.200326	0.215804
C	0.446175	1.460411	-1.000520
C	-0.476170	0.293835	-1.277750
C	0.378474	-0.973206	-1.438998
O	1.959118	2.474753	0.457453
C	1.080327	3.461284	0.105786
C	-0.039698	2.839686	-0.683066
O	1.738242	-3.501574	-1.217259
C	3.438102	0.399181	2.151892
C	-1.174141	3.474871	-0.946572
O	1.239508	4.611100	0.398177
H	2.817310	0.292798	-1.000867
C	0.733590	-1.720498	0.948191
O	-1.391404	0.204638	-0.173151
C	-2.521816	-0.501902	-0.385741
O	-2.796772	-1.006905	-1.445902
C	-3.350950	-0.585448	0.874238
C	-4.778317	-1.072038	0.624632
C	-5.470851	-1.365971	1.954724
C	-5.570532	-0.047041	-0.187376
H	3.114124	-3.081349	1.043226
H	4.220956	-3.325759	-0.305180
H	4.869862	-0.975460	-0.306213
H	5.007895	-1.546822	1.356057

H	2.854181	-1.937207	-1.775112
H	0.715166	0.973259	1.090504
H	1.121635	1.539500	-1.867309
H	-1.063020	0.450330	-2.188093
H	0.899425	-0.874784	-2.401827
H	-0.272679	-1.846417	-1.534698
H	1.545625	-4.000641	-0.410283
H	4.213702	0.133496	2.865109
H	2.776193	1.214074	2.426146
H	-1.981844	3.005596	-1.500421
H	-1.312104	4.494866	-0.600008
H	-0.037356	-1.026371	1.286799
H	1.438978	-1.858468	1.771505
H	0.233048	-2.675183	0.747580
H	-2.818999	-1.266589	1.553428
H	-3.344010	0.396856	1.362149
H	-4.713517	-1.998085	0.041020
H	-4.930936	-2.124354	2.531190
H	-5.533094	-0.458428	2.567032
H	-6.490508	-1.727474	1.790978
H	-6.587911	-0.403936	-0.374428
H	-5.096274	0.143633	-1.153649
H	-5.644361	0.900753	0.360091

M062X energy = -1154.62355728 a.u.

(1*R*,5*S*,6*R*,7*R*,8*R*,10*R*)-**3**, Conf. U

C	3.094320	-2.728875	0.109833
C	3.898393	-1.541525	0.657685
C	3.005434	-0.394452	1.067564
C	2.057664	0.028308	-0.030883
C	1.150117	-1.179437	-0.456472
C	2.107555	-2.288512	-0.963773
C	1.145488	1.201521	0.253032
C	0.392799	1.605278	-1.016384
C	-0.571668	0.531813	-1.467134
C	0.200198	-0.791216	-1.616687
O	1.806174	2.415158	0.652546
C	1.026651	3.478637	0.293668
C	-0.040011	2.988916	-0.646845
O	1.412566	-3.447622	-1.383818
C	3.051033	0.164264	2.275220
C	-1.099959	3.715803	-0.975456
O	1.220314	4.592357	0.687286
H	2.674078	0.296772	-0.906784
C	0.329141	-1.718381	0.726243
O	-1.625006	0.458786	-0.494528
C	-2.796300	-0.063971	-0.926943
O	-2.946763	-0.506992	-2.038385
C	-3.841785	-0.027327	0.158605
C	-3.481540	-0.943992	1.339983
C	-4.565882	-0.859246	2.412758
C	-3.287357	-2.385118	0.869532
H	2.538381	-3.221145	0.915145
H	3.761882	-3.481016	-0.321562
H	4.578021	-1.186950	-0.131569
H	4.523540	-1.857379	1.497951

H	2.679004	-1.865950	-1.810505
H	0.431292	0.941812	1.045617
H	1.153132	1.697065	-1.808651
H	-1.027242	0.783440	-2.429653
H	0.789435	-0.693239	-2.541544
H	-0.529486	-1.592067	-1.784787
H	0.927855	-3.238078	-2.194363
H	3.747253	-0.197929	3.026819
H	2.429916	1.011126	2.547454
H	-1.871435	3.341529	-1.641989
H	-1.211513	4.717135	-0.569978
H	-0.530272	-1.080441	0.942781
H	0.929875	-1.792831	1.636169
H	-0.042588	-2.716472	0.473527
H	-3.944756	1.005647	0.509483
H	-4.787012	-0.343367	-0.292346
H	-2.539374	-0.579393	1.768786
H	-4.313056	-1.488400	3.271542
H	-4.696526	0.167107	2.769983
H	-5.527067	-1.205041	2.015045
H	-2.994405	-3.030678	1.703208
H	-2.513476	-2.462836	0.098308
H	-4.219478	-2.776844	0.445839

M062X energy = -1154.62350181 a.u.

(1*R*,5*S*,6*R*,7*R*,8*R*,10*R*)-**3**, Conf. V

C	4.260754	-1.382617	0.391942
C	4.480358	0.118492	0.630287
C	3.185906	0.832472	0.938345
C	2.124137	0.582074	-0.106521
C	1.807153	-0.952158	-0.204892
C	3.132473	-1.650060	-0.604334
C	0.808659	1.311830	0.054058
C	-0.086548	1.080420	-1.165461
C	-0.501760	-0.369476	-1.286798
C	0.763792	-1.240206	-1.310741
O	0.902135	2.745170	0.136073
C	-0.276098	3.282337	-0.301641
C	-1.063684	2.200087	-0.988953
O	2.958711	-3.037114	-0.824107
C	3.011054	1.584324	2.023019
C	-2.351189	2.323581	-1.285759
O	-0.568324	4.432310	-0.143170
H	2.548154	0.883058	-1.080372
C	1.312403	-1.513182	1.139437
O	-1.355052	-0.652493	-0.167637
C	-2.170489	-1.724942	-0.282177
O	-2.175515	-2.451460	-1.243735
C	-3.082834	-1.822633	0.913848
C	-4.044473	-0.621747	0.976558
C	-4.906264	-0.552880	-0.284390
C	-4.915285	-0.712846	2.227859
H	4.022168	-1.874030	1.345100
H	5.174927	-1.850842	0.013180
H	4.909178	0.554961	-0.283631
H	5.207634	0.277810	1.431822

H	3.428855	-1.246896	-1.583202
H	0.294734	0.969789	0.961282
H	0.528563	1.310906	-2.049821
H	-1.077815	-0.549967	-2.200198
H	1.234944	-1.072647	-2.289640
H	0.482214	-2.296324	-1.285784
H	2.939080	-3.474388	0.039476
H	3.812577	1.710983	2.745751
H	2.088301	2.122747	2.212757
H	-2.910846	1.525476	-1.764889
H	-2.872264	3.246335	-1.047519
H	1.216480	-2.602919	1.068886
H	0.325528	-1.129035	1.402679
H	1.992279	-1.271241	1.960264
H	-3.641657	-2.759223	0.829159
H	-2.472244	-1.856673	1.823622
H	-3.432537	0.287424	1.033699
H	-5.537147	-1.445557	-0.367372
H	-5.561021	0.323677	-0.260541
H	-4.297566	-0.497561	-1.193611
H	-4.307228	-0.737642	3.137687
H	-5.527896	-1.621670	2.204666
H	-5.591432	0.144912	2.295030

M062X energy = -1154.62338172 a.u.

H	5.613146	-1.293880	-0.184353
H	2.272626	-2.282877	-1.666661
H	1.571200	1.182918	1.253198
H	0.334802	1.118959	-1.532136
H	-0.717123	0.108290	1.148082
H	0.129052	-1.306319	-1.427207
H	-0.337444	-2.103926	0.080417
H	2.112138	-4.382527	-0.609593
H	5.536595	0.717110	1.091498
H	3.985268	1.718200	1.221688
H	-2.260099	2.672992	0.084594
H	-1.369265	4.305610	0.256555
H	1.004753	-0.804785	2.164292
H	2.714350	-1.216689	2.041732
H	1.472440	-2.481487	1.890061
H	-4.225039	0.490654	-1.155256
H	-4.018385	-1.192608	-1.605377
H	-5.154948	-1.874971	0.545130
H	-6.468048	-0.160008	1.800063
H	-4.732745	0.178820	1.871021
H	-5.782451	1.112359	0.778982
H	-7.467834	-1.299863	-0.239393
H	-6.436675	-1.765903	-1.602248
H	-6.797178	-0.056918	-1.304665

M062X energy = -1154.62459410 a.u.

(1*R*,5*S*,6*R*,7*R*,8*S*,10*R*)-**3**, Conf. A

C	3.833741	-2.546348	-0.208990
C	4.579199	-1.245377	-0.538008
C	3.876148	-0.042908	0.045359
C	2.418900	0.030622	-0.348433
C	1.658297	-1.262696	0.117081
C	2.353584	-2.450784	-0.577008
C	1.631311	1.219834	0.155957
C	0.221901	1.232588	-0.441742
C	-0.565433	0.048450	0.065554
C	0.178789	-1.232550	-0.331477
O	2.161026	2.508790	-0.192525
C	1.133610	3.412201	-0.200646
C	-0.164834	2.652797	-0.170522
O	1.650593	-3.626624	-0.220592
C	4.487748	0.838151	0.834393
C	-1.332622	3.236060	0.070673
O	1.307844	4.595656	-0.239831
H	2.376021	0.051895	-1.450663
C	1.721924	-1.445959	1.644436
O	-1.854492	0.087161	-0.567018
C	-2.839832	-0.606877	0.040772
O	-2.683174	-1.220482	1.067193
C	-4.129242	-0.521212	-0.743170
C	-5.356706	-0.900252	0.085493
C	-5.594981	0.117240	1.201484
C	-6.583528	-1.016105	-0.817976
H	3.916754	-2.776622	0.858872
H	4.287805	-3.384194	-0.754324
H	4.618066	-1.131490	-1.631515

(1*R*,5*S*,6*R*,7*R*,8*S*,10*R*)-**3**, Conf. B

C	3.815496	-2.569178	-0.215486
C	4.568062	-1.270010	-0.532584
C	3.873952	-0.061980	0.049783
C	2.418015	0.023948	-0.346704
C	1.652294	-1.265174	0.118428
C	2.341197	-2.462960	-0.583619
C	1.638959	1.218384	0.158303
C	0.229962	1.242111	-0.440372
C	-0.567258	0.065960	0.067873
C	0.170580	-1.221488	-0.323122
O	2.176780	2.504189	-0.188356
C	1.156111	3.414797	-0.195955
C	-0.147923	2.664541	-0.168298
O	1.744493	-3.700824	-0.248098
C	4.488976	0.814967	0.840809
C	-1.311861	3.255653	0.072092
O	1.337495	4.597033	-0.233235
H	2.377227	0.046160	-1.449339
C	1.717185	-1.457120	1.643341
O	-1.855827	0.115297	-0.564131
C	-2.837708	-0.602345	0.022084
O	-2.669303	-1.262960	1.017397
C	-4.135354	-0.478565	-0.742309
C	-5.354773	-0.893224	0.081544
C	-5.579008	0.072717	1.245240
C	-6.591057	-0.965076	-0.813559
H	3.893669	-2.807605	0.851036
H	4.249147	-3.411338	-0.763281
H	4.615432	-1.151380	-1.625357

H	5.599590	-1.326891	-0.173090
H	2.264037	-2.291151	-1.672802
H	1.577866	1.179571	1.255627
H	0.342099	1.127394	-1.530722
H	-0.717189	0.125812	1.150729
H	0.108899	-1.301662	-1.419078
H	-0.365844	-2.071713	0.112586
H	0.859114	-3.730558	-0.636487
H	5.536036	0.685775	1.100984
H	3.992376	1.698811	1.227161
H	-2.243644	2.699756	0.084123
H	-1.340638	4.325215	0.259210
H	0.988512	-0.834663	2.170622
H	2.706018	-1.215270	2.041317
H	1.490517	-2.501018	1.878653
H	-4.233204	0.551529	-1.105806
H	-4.036149	-1.108785	-1.636516
H	-5.150836	-1.888474	0.493926
H	-4.710228	0.102770	1.908444
H	-5.768937	1.086260	0.870535
H	-6.446406	-0.230323	1.839396
H	-7.469990	-1.272430	-0.239019
H	-6.454766	-1.679080	-1.632368
H	-6.806895	0.015653	-1.254043

M062X energy = -1154.62456863 a.u.

(1*R*,5*S*,6*R*,7*R*,8*S*,10*R*)-**3**, Conf. C

C	3.387173	-2.913973	-0.308731
C	4.294019	-1.733437	-0.681319
C	3.811981	-0.439560	-0.069996
C	2.361297	-0.150926	-0.380587
C	1.452064	-1.319290	0.141154
C	1.922843	-2.602355	-0.589764
C	1.788043	1.141621	0.157346
C	0.362832	1.361053	-0.357951
C	-0.558628	0.309954	0.211783
C	-0.032946	-1.068664	-0.211205
O	2.479365	2.338257	-0.233358
C	1.597913	3.383122	-0.192884
C	0.205039	2.824173	-0.084937
O	1.179969	-3.743984	-0.208078
C	4.589762	0.343068	0.675341
C	-0.849729	3.576769	0.202996
O	1.940710	4.528061	-0.253053
H	2.258215	-0.124662	-1.479066
C	1.581487	-1.517790	1.661150
O	-1.865204	0.538292	-0.339086
C	-2.897672	-0.043317	0.308425
O	-2.757321	-0.745849	1.278953
C	-4.217010	0.335571	-0.323337
C	-5.385597	-0.522683	0.160937
C	-6.706967	0.073220	-0.322924
C	-5.235068	-1.968930	-0.311569
H	3.494754	-3.160337	0.753383
H	3.665184	-3.808970	-0.873582
H	4.292306	-1.623324	-1.776009

H	5.327005	-1.934323	-0.382800
H	1.805209	-2.422171	-1.673957
H	1.787583	1.113999	1.256691
H	0.391867	1.225880	-1.451302
H	-0.629192	0.391740	1.301369
H	-0.171901	-1.139623	-1.300698
H	-0.655253	-1.835205	0.263266
H	0.277556	-3.649539	-0.543320
H	5.622072	0.068536	0.874556
H	4.245669	1.288105	1.082273
H	-1.848754	3.158638	0.267326
H	-0.717530	4.641133	0.374666
H	2.617041	-1.419868	1.996944
H	1.222845	-2.518425	1.919175
H	0.982664	-0.797140	2.225522
H	-4.387286	1.395261	-0.088433
H	-4.108619	0.281744	-1.413519
H	-5.368371	-0.514873	1.257253
H	-6.743135	0.094616	-1.418688
H	-7.554210	-0.524454	0.026451
H	-6.843259	1.097773	0.038349
H	-6.067052	-2.582224	0.047598
H	-4.305859	-2.410815	0.057600
H	-5.234726	-2.015450	-1.407640

M062X energy = -1154.62454529 a.u.

(1*R*,5*S*,6*R*,7*R*,8*S*,10*R*)-**3**, Conf. D

C	3.399424	-2.899986	-0.305191
C	4.304741	-1.718508	-0.680726
C	3.814449	-0.429535	-0.065446
C	2.363509	-0.146903	-0.379721
C	1.451843	-1.318561	0.133486
C	1.930125	-2.592227	-0.591658
C	1.782994	1.142033	0.159349
C	0.359418	1.357604	-0.361561
C	-0.560221	0.298701	0.198529
C	-0.030575	-1.074939	-0.232916
O	2.473178	2.341613	-0.225193
C	1.587714	3.383464	-0.186031
C	0.196562	2.819506	-0.084860
O	1.086992	-3.656234	-0.190659
C	4.588080	0.352074	0.685173
C	-0.861676	3.567873	0.201518
O	1.928078	4.529546	-0.242197
H	2.264088	-0.117296	-1.478117
C	1.572577	-1.513693	1.655945
O	-1.865400	0.523972	-0.357819
C	-2.900904	-0.038043	0.301558
O	-2.768882	-0.702999	1.298919
C	-4.215723	0.313226	-0.356190
C	-5.388909	-0.518748	0.161338
C	-6.706831	0.059439	-0.352534
C	-5.241536	-1.984928	-0.246287
H	3.507023	-3.142971	0.757622
H	3.697997	-3.792694	-0.870408
H	4.299571	-1.607891	-1.775251

H	5.338848	-1.916061	-0.383957
H	1.813826	-2.411562	-1.676054
H	1.777932	1.112552	1.258429
H	0.394602	1.226894	-1.455272
H	-0.637312	0.377194	1.287860
H	-0.151297	-1.136930	-1.323852
H	-0.642728	-1.864481	0.210750
H	1.413053	-4.469275	-0.600996
H	5.621156	0.081038	0.885476
H	4.238776	1.293785	1.095262
H	-1.859192	3.145460	0.261151
H	-0.734181	4.632255	0.376655
H	0.987972	-0.775110	2.211304
H	2.608463	-1.434748	1.996582
H	1.187053	-2.501771	1.920401
H	-4.385290	1.383513	-0.174939
H	-4.100409	0.205321	-1.441667
H	-5.375080	-0.462642	1.256335
H	-7.557251	-0.520321	0.018864
H	-6.841465	1.099107	-0.036385
H	-6.738843	0.033783	-1.448374
H	-4.313360	-2.412140	0.141857
H	-5.240432	-2.079798	-1.339237
H	-6.075607	-2.579508	0.138913

M062X energy = -1154.62452275 a.u.

(1*R*,5*S*,6*R*,7*R*,8*S*,10*R*)-**3**, Conf. E

C	4.258064	-1.864564	-0.327588
C	4.669579	-0.430248	-0.685438
C	3.744104	0.590320	-0.067227
C	2.289704	0.329606	-0.385307
C	1.870637	-1.095891	0.119939
C	2.782652	-2.109673	-0.616690
C	1.281334	1.315552	0.161502
C	-0.124951	1.004480	-0.359309
C	-0.599991	-0.320327	0.186405
C	0.399381	-1.403272	-0.243922
O	1.487028	2.686571	-0.214702
C	0.284454	3.335645	-0.167665
C	-0.806533	2.304310	-0.066003
O	2.509952	-3.447773	-0.248287
C	4.178002	1.595896	0.690134
C	-2.059605	2.615173	0.242634
O	0.183822	4.527110	-0.216871
H	2.187116	0.328344	-1.484171
C	2.055923	-1.248321	1.639406
O	-1.892753	-0.580300	-0.381589
C	-2.637869	-1.526436	0.234371
O	-2.257381	-2.140616	1.199432
C	-3.987031	-1.664565	-0.425969
C	-4.797809	-0.359716	-0.357608
C	-6.150641	-0.547980	-1.041416
C	-4.976901	0.091490	1.092463
H	4.444222	-2.063738	0.733596
H	4.847439	-2.589970	-0.896605
H	4.631254	-0.318116	-1.779245

H	5.703156	-0.240719	-0.381581
H	2.611195	-1.975228	-1.700396
H	1.289694	1.278113	1.260517
H	-0.046690	0.907299	-1.454345
H	-0.707301	-0.287172	1.275815
H	0.302730	-1.506107	-1.335491
H	0.098890	-2.350556	0.216603
H	1.635967	-3.687993	-0.586109
H	5.238358	1.716862	0.894396
H	3.509835	2.344547	1.102597
H	-2.836010	1.860057	0.311787
H	-2.322936	3.651994	0.431248
H	2.979790	-0.777466	1.985083
H	2.091613	-2.313260	1.886518
H	1.229587	-0.806665	2.203811
H	-3.836145	-1.951523	-1.472947
H	-4.516998	-2.473529	0.085313
H	-4.233544	0.407479	-0.903263
H	-6.031896	-0.853848	-2.085652
H	-6.737010	-1.318188	-0.527125
H	-6.728935	0.380764	-1.023426
H	-5.538298	-0.659876	1.659746
H	-4.016224	0.234609	1.600581
H	-5.529217	1.035087	1.142611

M062X energy = -1154.62437551 a.u.

(1*R*,5*S*,6*R*,7*R*,8*S*,10*R*)-**3**, Conf. F

C	4.260473	-1.855664	-0.332154
C	4.670951	-0.422763	-0.699100
C	3.745026	0.593931	-0.075034
C	2.290236	0.329234	-0.387492
C	1.870656	-1.097800	0.116358
C	2.779642	-2.103806	-0.617441
C	1.281452	1.312943	0.162824
C	-0.125481	0.999232	-0.354044
C	-0.594752	-0.328778	0.190800
C	0.401299	-1.409231	-0.250574
O	1.485181	2.684424	-0.213652
C	0.281319	3.331480	-0.163840
C	-0.807526	2.298467	-0.059163
O	2.384214	-3.404880	-0.224605
C	4.181631	1.598927	0.681450
C	-2.060518	2.607662	0.251665
O	0.179798	4.523068	-0.213238
H	2.183455	0.328989	-1.485610
C	2.056393	-1.245981	1.637477
O	-1.889338	-0.590976	-0.372733
C	-2.643832	-1.513547	0.266734
O	-2.281639	-2.090796	1.260699
C	-3.983451	-1.672994	-0.408863
C	-4.800121	-0.370371	-0.378504
C	-6.137216	-0.573572	-1.088539
C	-5.012811	0.102273	1.060176
H	4.451346	-2.049490	0.729103
H	4.863316	-2.573987	-0.903240
H	4.623507	-0.314428	-1.792829

H	5.706362	-0.230883	-0.403146
H	2.603247	-1.970557	-1.700633
H	1.292697	1.276229	1.261641
H	-0.049843	0.903332	-1.449385
H	-0.699235	-0.296496	1.280404
H	0.310699	-1.500896	-1.342389
H	0.120140	-2.371693	0.184650
H	2.983353	-4.039946	-0.641047
H	5.242804	1.722221	0.880287
H	3.513581	2.345258	1.098191
H	-2.835022	1.850750	0.322604
H	-2.325665	3.644047	0.440187
H	2.991035	-0.793992	1.980271
H	2.061313	-2.308463	1.894265
H	1.241599	-0.778356	2.197070
H	-3.816962	-1.981223	-1.447245
H	-4.517483	-2.472701	0.112685
H	-4.226689	0.390624	-0.923403
H	-6.719921	0.352662	-1.095758
H	-5.994561	-0.893265	-2.125618
H	-6.731190	-1.339494	-0.576488
H	-4.064340	0.250136	1.589138
H	-5.588921	-0.640256	1.624403
H	-5.564566	1.047302	1.083705

M062X energy = -1154.62427202 a.u.

(1*R*,5*S*,6*R*,7*R*,8*S*,10*R*)-**3**, Conf. G

C	3.825914	-2.557344	-0.242769
C	4.573852	-1.249432	-0.538220
C	3.871170	-0.055929	0.063143
C	2.417847	0.027778	-0.341205
C	1.648876	-1.264382	0.110500
C	2.341238	-2.459307	-0.591574
C	1.633817	1.222243	0.156970
C	0.225634	1.241219	-0.443595
C	-0.566593	0.058723	0.061150
C	0.173308	-1.221663	-0.345188
O	2.171497	2.507260	-0.191913
C	1.148407	3.416192	-0.200888
C	-0.154143	2.663364	-0.172292
O	1.671758	-3.681973	-0.350022
C	4.477591	0.809652	0.872993
C	-1.319224	3.252676	0.067262
O	1.328730	4.598605	-0.239172
H	2.383660	0.050259	-1.443849
C	1.701347	-1.451728	1.636177
O	-1.857546	0.104935	-0.565896
C	-2.838697	-0.599873	0.036652
O	-2.675141	-1.225629	1.054893
C	-4.131131	-0.509205	-0.741145
C	-5.354616	-0.900814	0.087630
C	-5.591654	0.103376	1.215871
C	-6.584219	-1.009572	-0.812932
H	3.928408	-2.805946	0.822794
H	4.261648	-3.387309	-0.808271
H	4.618465	-1.115272	-1.628914

H	5.606067	-1.307595	-0.180755
H	2.245528	-2.296551	-1.674554
H	1.569378	1.190034	1.254024
H	0.340164	1.127931	-1.533823
H	-0.714432	0.114095	1.144651
H	0.130655	-1.282566	-1.441854
H	-0.350182	-2.097028	0.048788
H	1.951548	-4.010004	0.516725
H	5.523940	0.682455	1.137123
H	3.973809	1.684945	1.269682
H	-2.249583	2.694404	0.079516
H	-1.350523	4.322431	0.252937
H	2.711106	-1.320537	2.033684
H	1.343251	-2.453282	1.897735
H	1.050129	-0.749582	2.163386
H	-4.231187	0.506797	-1.141851
H	-4.021319	-1.170850	-1.610981
H	-5.148857	-1.880249	0.535334
H	-4.727308	0.160017	1.883151
H	-5.783387	1.102636	0.805283
H	-6.461859	-0.183023	1.814295
H	-6.438276	-1.750320	-1.605873
H	-6.801728	-0.045574	-1.288245
H	-7.465855	-1.301759	-0.234496

M062X energy = -1154.62426701 a.u.

(1*R*,5*S*,6*R*,7*R*,8*S*,10*R*)-**3**, Conf. H

C	3.387719	-2.910391	-0.337699
C	4.297091	-1.722895	-0.684417
C	3.808202	-0.441933	-0.052083
C	2.361227	-0.150343	-0.374181
C	1.442005	-1.319003	0.129555
C	1.914658	-2.600342	-0.601757
C	1.785609	1.143850	0.158244
C	0.362595	1.364462	-0.362330
C	-0.560337	0.308099	0.198280
C	-0.035842	-1.064497	-0.241974
O	2.481643	2.338223	-0.230307
C	1.600943	3.384672	-0.190803
C	0.207089	2.827398	-0.086660
O	1.092331	-3.714660	-0.312901
C	4.577480	0.326377	0.716499
C	-0.847224	3.581041	0.200408
O	1.946684	4.528909	-0.248359
H	2.267900	-0.122330	-1.473282
C	1.554056	-1.513377	1.651192
O	-1.867039	0.540361	-0.350292
C	-2.899136	-0.031662	0.306088
O	-2.760877	-0.708768	1.294677
C	-4.217322	0.325153	-0.341222
C	-5.384708	-0.522829	0.163444
C	-6.706869	0.057428	-0.337004
C	-5.229971	-1.980308	-0.271708
H	3.514822	-3.172440	0.721966
H	3.666894	-3.793570	-0.921261
H	4.297736	-1.594951	-1.776771

H	5.329150	-1.928985	-0.386058
H	1.780989	-2.424908	-1.678736
H	1.779777	1.121095	1.257393
H	0.396912	1.233068	-1.455961
H	-0.632174	0.382552	1.288455
H	-0.149561	-1.115774	-1.334061
H	-0.655570	-1.856848	0.185950
H	1.371672	-4.079655	0.539083
H	5.608109	0.049966	0.921900
H	4.227712	1.264146	1.135491
H	-1.846618	3.163361	0.261708
H	-0.714463	4.645037	0.373976
H	1.042949	-0.724466	2.209253
H	2.593202	-1.530539	1.989926
H	1.069955	-2.452445	1.940203
H	-4.391917	1.390788	-0.138422
H	-4.104378	0.238938	-1.428817
H	-5.368826	-0.486648	1.259273
H	-6.741087	0.051636	-1.433035
H	-7.553212	-0.533852	0.025395
H	-6.846623	1.090319	-0.001473
H	-5.231085	-2.055079	-1.366138
H	-6.059521	-2.586782	0.104560
H	-4.298285	-2.409569	0.105722

M062X energy = -1154.62418740 a.u.

(1*R*,5*S*,6*R*,7*R*,8*S*,10*R*)-**3**, Conf. I

C	3.266066	-2.837859	-0.404467
C	4.061970	-1.659548	-0.981466
C	3.639744	-0.343774	-0.372527
C	2.149182	-0.109572	-0.460788
C	1.374725	-1.267113	0.263222
C	1.765951	-2.579206	-0.462842
C	1.626477	1.198046	0.092008
C	0.130420	1.352723	-0.196336
C	-0.652974	0.314003	0.568849
C	-0.154597	-1.073349	0.140845
O	2.205559	2.386171	-0.469920
C	1.307639	3.410021	-0.343060
C	-0.029541	2.824755	0.021934
O	1.132188	-3.714083	0.095005
C	4.499543	0.500862	0.193433
C	-1.046845	3.565640	0.443819
O	1.597654	4.557338	-0.520804
H	1.871358	-0.150671	-1.528068
C	1.750931	-1.371861	1.751108
O	-2.036488	0.473381	0.221611
C	-2.931518	-0.122423	1.043307
O	-2.612649	-0.744589	2.025042
C	-4.339801	0.081035	0.543263
C	-4.540941	-0.494192	-0.868759
C	-4.154541	-1.972658	-0.912600
C	-5.989399	-0.293119	-1.309948
H	3.549613	-3.018690	0.638257
H	3.480498	-3.757436	-0.957420
H	3.883063	-1.614648	-2.066114

H	5.135425	-1.816858	-0.841820
H	1.471279	-2.466442	-1.522249
H	1.803201	1.235100	1.176767
H	-0.011375	1.156309	-1.271543
H	-0.553743	0.457081	1.649754
H	-0.461720	-1.209379	-0.907843
H	-0.668348	-1.826678	0.748635
H	0.185199	-3.662381	-0.095427
H	5.559031	0.263882	0.238685
H	4.193027	1.459847	0.597727
H	-2.006179	3.126524	0.696495
H	-0.924129	4.640854	0.536867
H	1.225438	-0.634116	2.364268
H	2.822810	-1.228250	1.909552
H	1.472265	-2.363988	2.117787
H	-5.009514	-0.407222	1.257258
H	-4.559664	1.155217	0.539210
H	-3.882747	0.059852	-1.548659
H	-3.100350	-2.121807	-0.652564
H	-4.758979	-2.550687	-0.203979
H	-4.315211	-2.387451	-1.912313
H	-6.142328	-0.665481	-2.327438
H	-6.270596	0.764600	-1.289890
H	-6.672399	-0.837294	-0.647385

M062X energy = -1154.62406363 a.u.

(1*R*,5*S*,6*R*,7*R*,8*S*,10*R*)-**3**, Conf. J

C	3.227157	-2.866647	-0.389473
C	4.042163	-1.703983	-0.973061
C	3.634833	-0.383003	-0.365027
C	2.147865	-0.129578	-0.458917
C	1.349372	-1.274301	0.261540
C	1.726266	-2.586042	-0.455145
C	1.640190	1.185653	0.090095
C	0.148246	1.360396	-0.206676
C	-0.653647	0.330888	0.553106
C	-0.176084	-1.062723	0.124379
O	2.240205	2.364387	-0.470175
C	1.355646	3.400987	-0.349905
C	0.008675	2.834984	0.008586
O	0.974227	-3.629129	0.136585
C	4.505362	0.448665	0.203620
C	-1.000493	3.590814	0.423588
O	1.663934	4.543686	-0.527977
H	1.873862	-0.167175	-1.526957
C	1.714654	-1.376025	1.753670
O	-2.032756	0.508623	0.196249
C	-2.941837	-0.045960	1.031230
O	-2.641691	-0.628945	2.042068
C	-4.341751	0.147465	0.503927
C	-4.535610	-0.517761	-0.869336
C	-4.179995	-2.003286	-0.807742
C	-5.973100	-0.316708	-1.344998
H	3.506381	-3.044845	0.654790
H	3.446294	-3.789615	-0.942339
H	3.862055	-1.660180	-2.057451

H	5.113301	-1.876184	-0.833407
H	1.436257	-2.471594	-1.515794
H	1.811616	1.222522	1.175520
H	0.010367	1.165588	-1.282690
H	-0.560733	0.474667	1.634440
H	-0.468833	-1.191368	-0.928015
H	-0.696395	-1.827990	0.707176
H	1.243953	-4.463528	-0.271776
H	5.561463	0.197313	0.251700
H	4.210402	1.411574	0.607073
H	-1.966918	3.164760	0.671573
H	-0.863862	4.664450	0.515625
H	2.790112	-1.268657	1.918324
H	1.393182	-2.349082	2.134054
H	1.213518	-0.609538	2.351166
H	-5.027091	-0.283453	1.239702
H	-4.544162	1.222197	0.425867
H	-3.856305	-0.023660	-1.574440
H	-4.337034	-2.480737	-1.779788
H	-3.133802	-2.159167	-0.522372
H	-4.807567	-2.518831	-0.071451
H	-6.232105	0.745526	-1.400266
H	-6.676558	-0.801017	-0.657711
H	-6.120235	-0.753948	-2.337307

M062X energy = -1154.62405963 a.u.

(1*R*,5*S*,6*R*,7*R*,8*S*,10*R*)-**3**, Conf. K

C	3.032557	-3.071652	-0.288856
C	3.977261	-1.985888	-0.823126
C	3.649834	-0.631839	-0.239930
C	2.198116	-0.252547	-0.420902
C	1.263721	-1.321158	0.251291
C	1.567830	-2.663640	-0.442642
C	1.773547	1.103623	0.097986
C	0.321843	1.404228	-0.286361
C	-0.608292	0.450430	0.424631
C	-0.227350	-0.981006	0.024659
O	2.504313	2.224139	-0.425008
C	1.706245	3.333207	-0.356516
C	0.296754	2.886419	-0.079493
O	0.695593	-3.635942	0.103025
C	4.553626	0.125115	0.379007
C	-0.666817	3.728674	0.272880
O	2.121523	4.444513	-0.514812
H	1.984746	-0.271531	-1.503196
C	1.530738	-1.446848	1.762315
O	-1.944866	0.741606	-0.013933
C	-2.940672	0.269289	0.765759
O	-2.751290	-0.340159	1.789357
C	-4.301545	0.552484	0.179501
C	-5.007108	-0.767065	-0.175639
C	-6.415123	-0.485942	-0.695105
C	-4.190964	-1.559549	-1.197277
H	3.233797	-3.268164	0.769985
H	3.203651	-4.012633	-0.828150
H	3.865506	-1.932098	-1.916220

H	5.019613	-2.248486	-0.620568
H	1.351435	-2.529301	-1.518374
H	1.883303	1.130324	1.191663
H	0.231186	1.216655	-1.368579
H	-0.568169	0.591011	1.509604
H	-0.468256	-1.089775	-1.042844
H	-0.843885	-1.696075	0.576392
H	0.916182	-4.491893	-0.289918
H	5.579685	-0.215345	0.489108
H	4.319039	1.111766	0.764356
H	-1.679939	3.389668	0.461396
H	-0.443392	4.787005	0.372271
H	1.060150	-0.639398	2.329953
H	2.599743	-1.428164	1.990589
H	1.107888	-2.388513	2.121979
H	-4.884740	1.091849	0.933562
H	-4.202765	1.185324	-0.707760
H	-5.075327	-1.353409	0.748873
H	-6.374436	0.105056	-1.617684
H	-6.941338	-1.418986	-0.918793
H	-7.008130	0.071199	0.037184
H	-4.048973	-0.974291	-2.113734
H	-4.701991	-2.489092	-1.465911
H	-3.201974	-1.823983	-0.807320

M062X energy = -1154.62398391 a.u.

(1*R*,5*S*,6*R*,7*R*,8*S*,10*R*)-**3**, Conf. L

C	4.261566	-1.856199	-0.364088
C	4.669499	-0.413947	-0.696921
C	3.742652	0.590840	-0.055720
C	2.290009	0.331407	-0.380125
C	1.866370	-1.098021	0.110550
C	2.777815	-2.108063	-0.630583
C	1.278893	1.317475	0.162364
C	-0.126784	1.004255	-0.358341
C	-0.596446	-0.324567	0.185264
C	0.398870	-1.402087	-0.264075
O	1.486155	2.687507	-0.215586
C	0.282599	3.336234	-0.168156
C	-0.807916	2.304473	-0.065351
O	2.424069	-3.449831	-0.355736
C	4.174489	1.581733	0.721773
C	-2.061504	2.615409	0.241333
O	0.182678	4.527797	-0.217533
H	2.192337	0.333455	-1.479221
C	2.042226	-1.251185	1.630738
O	-1.893193	-0.585290	-0.372552
C	-2.639859	-1.516164	0.264327
O	-2.267479	-2.099749	1.251068
C	-3.982898	-1.676669	-0.403706
C	-4.800289	-0.374614	-0.371723
C	-6.141764	-0.580866	-1.072534
C	-5.003840	0.101828	1.067030
H	4.476297	-2.063491	0.693534
H	4.846351	-2.568783	-0.954601
H	4.623604	-0.284468	-1.788130

H	5.704687	-0.227969	-0.396187
H	2.588790	-1.983586	-1.706352
H	1.283983	1.285069	1.261265
H	-0.048469	0.908397	-1.453458
H	-0.696445	-0.295103	1.275564
H	0.312973	-1.480689	-1.357128
H	0.113951	-2.371059	0.154007
H	2.810086	-3.692184	0.498340
H	5.234389	1.702638	0.928576
H	3.503607	2.320793	1.147219
H	-2.837232	1.859528	0.309874
H	-2.325915	3.652273	0.428249
H	1.278701	-0.707805	2.193614
H	3.016188	-0.890912	1.972053
H	1.934003	-2.304508	1.911059
H	-3.821105	-1.986893	-1.442273
H	-4.513593	-2.476158	0.121555
H	-4.231035	0.385371	-0.922335
H	-6.725056	0.344988	-1.078780
H	-6.005512	-0.903533	-2.109525
H	-6.731967	-1.345632	-0.554455
H	-4.051819	0.253210	1.588701
H	-5.574684	-0.640010	1.637475
H	-5.557083	1.045946	1.091737

M062X energy = -1154.62396566 a.u.

(1*R*,5*S*,6*R*,7*R*,8*S*,10*R*)-**3**, Conf. M

C	2.821778	-3.170609	-0.257677
C	3.833733	-2.125929	-0.748915
C	3.551696	-0.763712	-0.161220
C	2.126495	-0.313715	-0.385815
C	1.120435	-1.343084	0.242906
C	1.383909	-2.692064	-0.455213
C	1.749622	1.056550	0.133430
C	0.327296	1.429967	-0.294006
C	-0.668969	0.514130	0.376248
C	-0.343581	-0.928874	-0.030986
O	2.549770	2.145427	-0.353688
C	1.803678	3.290812	-0.298518
C	0.366371	2.909473	-0.070476
O	0.448828	-3.626711	0.050847
C	4.469073	-0.057285	0.496212
C	-0.565861	3.793598	0.263111
O	2.276167	4.382374	-0.431805
H	1.949045	-0.312235	-1.474758
C	1.329591	-1.495777	1.760497
O	-1.976212	0.875921	-0.094984
C	-3.017299	0.463196	0.662480
O	-2.882027	-0.151155	1.692436
C	-4.338212	0.828689	0.032430
C	-4.964492	-0.384089	-0.691903
C	-4.008018	-0.960710	-1.736953
C	-5.414102	-1.462054	0.293950
H	2.978031	-3.386098	0.805037
H	2.966173	-4.113666	-0.801134
H	3.760486	-2.056226	-1.844363

H	4.855263	-2.439954	-0.515795
H	1.210316	-2.537813	-1.536041
H	1.824554	1.068022	1.230335
H	0.263243	1.258162	-1.380763
H	-0.653780	0.639772	1.463640
H	-0.549387	-1.011924	-1.108084
H	-1.014022	-1.620441	0.487169
H	0.642202	-4.488698	-0.343241
H	5.473519	-0.447552	0.636212
H	4.268851	0.935508	0.885163
H	-1.599397	3.502362	0.417667
H	-0.294687	4.838670	0.382120
H	0.881020	-0.671064	2.321037
H	2.389925	-1.532542	2.024318
H	0.848808	-2.418483	2.095670
H	-5.005714	1.165287	0.831196
H	-4.183528	1.647034	-0.675708
H	-5.851162	0.001259	-1.210981
H	-3.631922	-0.185533	-2.412393
H	-3.144041	-1.432434	-1.253388
H	-4.509794	-1.726900	-2.335514
H	-4.561917	-1.857961	0.854390
H	-5.890592	-2.290974	-0.238911
H	-6.132543	-1.063513	1.017183

M062X energy = -1154.62378129 a.u.

(1*R*,5*S*,6*R*,7*R*,8*S*,10*R*)-**3**, Conf. N

C	3.226288	-2.867584	-0.424804
C	4.045345	-1.692135	-0.976990
C	3.635363	-0.381317	-0.349595
C	2.149817	-0.126125	-0.453345
C	1.346879	-1.273934	0.255244
C	1.724068	-2.589909	-0.469834
C	1.639215	1.190503	0.090236
C	0.147295	1.363907	-0.207808
C	-0.653452	0.331872	0.550613
C	-0.175954	-1.058349	0.111639
O	2.239650	2.367652	-0.471705
C	1.354814	3.404581	-0.351326
C	0.007701	2.838552	0.007503
O	0.979368	-3.694807	0.005495
C	4.500150	0.441109	0.240804
C	-1.001675	3.594496	0.421806
O	1.663126	4.547147	-0.529154
H	1.883094	-0.164074	-1.523344
C	1.703250	-1.377572	1.747847
O	-2.033780	0.511288	0.201493
C	-2.937180	-0.057717	1.033198
O	-2.628807	-0.653800	2.034200
C	-4.340230	0.138172	0.516210
C	-4.540292	-0.513424	-0.862905
C	-4.182289	-1.998943	-0.818668
C	-5.980692	-0.309436	-1.328435
H	3.523480	-3.065850	0.614418
H	3.428288	-3.780084	-0.994897
H	3.872386	-1.629178	-2.061302

H	5.115452	-1.868215	-0.833749
H	1.419588	-2.478204	-1.520243
H	1.808471	1.232702	1.175743
H	0.010468	1.169562	-1.284004
H	-0.555937	0.470219	1.632458
H	-0.461582	-1.176025	-0.943813
H	-0.702971	-1.830567	0.679059
H	1.392024	-4.003623	0.824958
H	5.555898	0.189655	0.295196
H	4.201142	1.398376	0.654956
H	-1.968207	3.168486	0.669389
H	-0.865137	4.668197	0.513231
H	1.270835	-0.563324	2.335188
H	2.783192	-1.362767	1.916213
H	1.290973	-2.303520	2.163029
H	-5.020481	-0.302030	1.251193
H	-4.545303	1.213203	0.450455
H	-3.865709	-0.010898	-1.566592
H	-4.804455	-2.522848	-0.083680
H	-4.344763	-2.466791	-1.794367
H	-3.134029	-2.157002	-0.542343
H	-6.241488	0.752934	-1.371542
H	-6.679676	-0.801811	-0.642317
H	-6.132523	-0.736860	-2.324261

M062X energy = -1154.62376940 a.u.

(1*R*,5*S*,6*R*,7*R*,8*S*,10*R*)-**3**, Conf. O

C	2.822357	-3.176760	-0.267504
C	3.832231	-2.128334	-0.753261
C	3.553478	-0.764932	-0.166984
C	2.128005	-0.313472	-0.387700
C	1.128704	-1.344636	0.246351
C	1.388535	-2.699684	-0.459448
C	1.753769	1.056904	0.132887
C	0.329526	1.430664	-0.288343
C	-0.663707	0.517717	0.388682
C	-0.337273	-0.927185	-0.013661
O	2.550500	2.146551	-0.357410
C	1.804859	3.291701	-0.298451
C	0.368280	2.910154	-0.064805
O	0.545403	-3.728612	0.021744
C	4.471811	-0.056661	0.487223
C	-0.562856	3.793949	0.272542
O	2.275499	4.383695	-0.432902
H	1.946926	-0.312924	-1.476392
C	1.345622	-1.504471	1.760801
O	-1.972838	0.879254	-0.076445
C	-3.012419	0.442948	0.669745
O	-2.873062	-0.208045	1.676764
C	-4.334748	0.830548	0.056493
C	-4.971905	-0.362203	-0.691384
C	-4.017924	-0.929173	-1.743907
C	-5.436511	-1.453071	0.273011
H	2.978714	-3.397287	0.794243
H	2.949741	-4.117908	-0.811004
H	3.762225	-2.057203	-1.848933

H	4.853501	-2.442273	-0.518770
H	1.213229	-2.544082	-1.539644
H	1.833138	1.067253	1.229684
H	0.259759	1.257890	-1.374590
H	-0.642306	0.643042	1.476024
H	-0.561094	-1.014372	-1.088086
H	-1.011190	-1.597626	0.531895
H	-0.360618	-3.540348	-0.259915
H	5.476318	-0.447126	0.625833
H	4.273139	0.936888	0.875144
H	-1.595962	3.503331	0.431139
H	-0.290886	4.838897	0.390713
H	2.407467	-1.519572	2.019498
H	0.892337	-2.445384	2.085883
H	0.878488	-0.698271	2.333682
H	-4.995773	1.152871	0.866428
H	-4.178964	1.664020	-0.633355
H	-5.852329	0.041454	-1.206786
H	-4.523011	-1.682178	-2.356042
H	-3.632521	-0.146732	-2.405407
H	-3.158957	-1.414497	-1.263914
H	-6.156089	-1.060763	0.998317
H	-4.591484	-1.864956	0.832925
H	-5.916820	-2.268905	-0.276305

M062X energy = -1154.62371086 a.u.

(1*R*,5*S*,6*R*,7*R*,8*S*,10*R*)-**3**, Conf. P

C	3.031609	-3.072585	-0.323942
C	3.978271	-1.974572	-0.829811
C	3.647293	-0.629733	-0.228124
C	2.197241	-0.249241	-0.416139
C	1.260589	-1.320218	0.246991
C	1.563790	-2.667703	-0.454476
C	1.770990	1.108830	0.097128
C	0.318241	1.407464	-0.285122
C	-0.609192	0.451217	0.427036
C	-0.227702	-0.976937	0.016809
O	2.500490	2.227614	-0.430058
C	1.702028	3.336853	-0.360149
C	0.293237	2.889811	-0.078833
O	0.701487	-3.702201	-0.021334
C	4.544564	0.119213	0.409719
C	-0.669664	3.731981	0.275578
O	2.116595	4.448033	-0.519984
H	1.988815	-0.270162	-1.499493
C	1.522006	-1.445373	1.757475
O	-1.948193	0.743943	-0.001320
C	-2.937972	0.256861	0.777384
O	-2.739043	-0.366211	1.791217
C	-4.303148	0.542470	0.203413
C	-4.998778	-0.774102	-0.182332
C	-6.411182	-0.491461	-0.688947
C	-4.180519	-1.533754	-1.226889
H	3.251512	-3.289439	0.730697
H	3.186642	-4.002325	-0.880737
H	3.872859	-1.903467	-1.922260

H	5.019558	-2.240496	-0.625746
H	1.329713	-2.536125	-1.520457
H	1.881685	1.141801	1.190452
H	0.226252	1.219735	-1.367171
H	-0.562924	0.586591	1.512628
H	-0.462115	-1.075802	-1.052942
H	-0.849355	-1.698583	0.554066
H	1.038633	-4.039814	0.821016
H	5.570135	-0.221156	0.524342
H	4.305658	1.101009	0.805092
H	-1.682208	3.392761	0.466759
H	-0.446315	4.790465	0.373498
H	1.127153	-0.594574	2.318869
H	2.588024	-1.520713	1.987111
H	1.010265	-2.331105	2.149129
H	-4.888043	1.057675	0.972807
H	-4.213195	1.197614	-0.668454
H	-5.058753	-1.383998	0.727448
H	-6.379003	0.122855	-1.596482
H	-6.930423	-1.422956	-0.934192
H	-7.005822	0.042054	0.059455
H	-3.188030	-1.801257	-0.848215
H	-4.046322	-0.924162	-2.128484
H	-4.685310	-2.459972	-1.517542

M062X energy = -1154.62368704 a.u.

(1*R*,5*S*,6*R*,7*R*,8*S*,10*R*)-**3**, Conf. Q

C	2.817949	-3.173876	-0.295314
C	3.832160	-2.118555	-0.759138
C	3.548126	-0.765629	-0.151886
C	2.125220	-0.312429	-0.381497
C	1.116152	-1.342755	0.238543
C	1.376335	-2.696912	-0.467570
C	1.749430	1.060180	0.133041
C	0.326266	1.433906	-0.291422
C	-0.668655	0.516682	0.379898
C	-0.344456	-0.923456	-0.037718
O	2.549653	2.146476	-0.358383
C	1.804845	3.293112	-0.300852
C	0.367642	2.913438	-0.067963
O	0.451665	-3.692198	-0.072669
C	4.459748	-0.068769	0.523474
C	-0.562698	3.798651	0.267997
O	2.278194	4.383955	-0.435441
H	1.951485	-0.312384	-1.471174
C	1.320482	-1.495312	1.755265
O	-1.977806	0.882003	-0.081595
C	-3.014641	0.453736	0.673344
O	-2.871876	-0.176515	1.692905
C	-4.339274	0.824622	0.055028
C	-4.963361	-0.377427	-0.689049
C	-4.009711	-0.930041	-1.749495
C	-5.403347	-1.475647	0.278593
H	2.993338	-3.410743	0.763343
H	2.945509	-4.104926	-0.856878
H	3.764523	-2.031154	-1.853386

H	4.852341	-2.437356	-0.526264
H	1.183068	-2.544453	-1.538970
H	1.826272	1.077328	1.229670
H	0.260112	1.262581	-1.378104
H	-0.647679	0.636544	1.468053
H	-0.544202	-0.996575	-1.116521
H	-1.020466	-1.620159	0.466183
H	0.746010	-4.054276	0.775541
H	5.463224	-0.460415	0.666393
H	4.256483	0.919218	0.923310
H	-1.596098	3.508498	0.425410
H	-0.290115	4.843514	0.385631
H	2.373284	-1.625680	2.018835
H	0.752894	-2.358275	2.119843
H	0.949747	-0.631470	2.313097
H	-5.005029	1.143911	0.862305
H	-4.190569	1.656224	-0.638788
H	-5.854317	0.013870	-1.196159
H	-3.640616	-0.140708	-2.412280
H	-3.141309	-1.407514	-1.279764
H	-4.510948	-1.687165	-2.359742
H	-5.879830	-2.295901	-0.267382
H	-6.118720	-1.093837	1.013806
H	-4.546304	-1.879597	0.825790

M062X energy = -1154.62347176 a.u.

(1*R*,5*S*,6*R*,7*R*,8*S*,10*R*)-**3**, Conf. R

C	3.410925	-2.886684	-0.196640
C	4.327898	-1.692156	-0.491533
C	3.784902	-0.409500	0.091689
C	2.357939	-0.135734	-0.324505
C	1.427118	-1.319454	0.118039
C	1.967977	-2.590616	-0.584671
C	1.729911	1.144652	0.180106
C	0.344315	1.352469	-0.438070
C	-0.604139	0.284221	0.049855
C	-0.029749	-1.083700	-0.344137
O	2.433307	2.353126	-0.147567
C	1.538239	3.387033	-0.163101
C	0.148395	2.810463	-0.162085
O	1.213136	-3.744264	-0.268079
C	4.494710	0.376349	0.898924
C	-0.933505	3.546666	0.059850
O	1.870174	4.536479	-0.187994
H	2.337148	-0.100590	-1.427389
C	1.443752	-1.529175	1.641981
O	-1.867355	0.502128	-0.597196
C	-2.940272	-0.101665	-0.038927
O	-2.859141	-0.804302	0.938745
C	-4.194505	0.245595	-0.810400
C	-5.485957	-0.399046	-0.296084
C	-5.441509	-1.925885	-0.387145
C	-5.839378	0.068394	1.117421
H	3.441737	-3.140144	0.868813
H	3.741943	-3.773651	-0.745330
H	4.406578	-1.572822	-1.582423

H	5.338165	-1.882886	-0.117999
H	1.929804	-2.402949	-1.673289
H	1.647866	1.106423	1.276077
H	0.457047	1.230119	-1.527514
H	-0.758686	0.354548	1.131492
H	-0.084053	-1.145260	-1.441709
H	-0.676242	-1.862859	0.074094
H	0.339080	-3.660780	-0.673876
H	5.512680	0.112840	1.172813
H	4.108870	1.313688	1.285762
H	-1.929175	3.115845	0.050131
H	-0.827342	4.610309	0.252708
H	0.792764	-0.822326	2.164452
H	2.449121	-1.418747	2.056373
H	1.081867	-2.537138	1.864504
H	-4.279140	1.339948	-0.804581
H	-4.010400	-0.031081	-1.855907
H	-6.276526	-0.046924	-0.972069
H	-5.204009	-2.255505	-1.404691
H	-4.689258	-2.332242	0.293572
H	-6.412564	-2.351528	-0.116121
H	-5.095506	-0.280362	1.837899
H	-6.815721	-0.326080	1.415434
H	-5.889440	1.161459	1.172107

M062X energy = -1154.62337936 a.u.

(1*R*,5*S*,6*R*,7*R*,8*S*,10*R*)-**3**, Conf. S

C	3.441642	-2.856674	-0.190187
C	4.349364	-1.655661	-0.490133
C	3.790308	-0.380829	0.095283
C	2.361282	-0.122749	-0.323328
C	1.436988	-1.315256	0.112949
C	1.992039	-2.574055	-0.583000
C	1.718306	1.150300	0.181195
C	0.332925	1.344787	-0.441485
C	-0.605683	0.263436	0.038715
C	-0.018198	-1.096305	-0.361366
O	2.411872	2.365863	-0.142358
C	1.506221	3.390848	-0.159907
C	0.122479	2.800405	-0.163700
O	1.141960	-3.656681	-0.252821
C	4.491507	0.409671	0.905417
C	-0.967367	3.525372	0.056603
O	1.827941	4.543451	-0.182625
H	2.342592	-0.085526	-1.425830
C	1.448123	-1.520123	1.638892
O	-1.868167	0.468439	-0.614751
C	-2.941491	-0.123649	-0.045417
O	-2.868620	-0.782816	0.962298
C	-4.187961	0.175799	-0.849630
C	-5.485915	-0.430674	-0.306457
C	-5.449459	-1.960459	-0.305795
C	-5.844622	0.122112	1.074563
H	3.474950	-3.105616	0.876184
H	3.798371	-3.739283	-0.737266
H	4.423009	-1.536642	-1.581306

H	5.362291	-1.836136	-0.118793
H	1.953084	-2.386817	-1.671821
H	1.633072	1.110714	1.276659
H	0.451622	1.226513	-1.530756
H	-0.767404	0.331218	1.119405
H	-0.055466	-1.150981	-1.458747
H	-0.646331	-1.901216	0.029267
H	1.514256	-4.460597	-0.641182
H	5.512115	0.156652	1.179577
H	4.094722	1.341643	1.294034
H	-1.958259	3.083731	0.043714
H	-0.873034	4.589796	0.251328
H	2.453649	-1.421471	2.056638
H	1.065899	-2.518411	1.867424
H	0.807372	-0.798963	2.153687
H	-4.269436	1.268112	-0.918551
H	-3.994696	-0.171829	-1.872146
H	-6.270843	-0.115314	-1.006908
H	-5.210221	-2.351244	-1.300944
H	-4.700650	-2.329040	0.399511
H	-6.423857	-2.363909	-0.013132
H	-5.106364	-0.187457	1.818392
H	-6.824778	-0.248605	1.390305
H	-5.888502	1.216849	1.064043

M062X energy = -1154.62337150 a.u.

(1*R*,5*S*,6*R*,7*R*,8*S*,10*R*)-**3**, Conf. T

C	3.998811	-2.252054	-0.163840
C	4.621099	-0.872623	-0.418163
C	3.776676	0.239388	0.156845
C	2.338588	0.179881	-0.304489
C	1.696109	-1.193789	0.097287
C	2.538927	-2.293712	-0.596937
C	1.414652	1.270647	0.189457
C	0.036301	1.162183	-0.470727
C	-0.652555	-0.105911	-0.025993
C	0.239818	-1.294870	-0.412308
O	1.829300	2.613312	-0.106864
C	0.720720	3.413107	-0.142618
C	-0.497412	2.531690	-0.186724
O	2.064798	-3.596057	-0.314281
C	4.258870	1.155762	0.993962
C	-1.724766	2.994514	0.015242
O	0.778941	4.608408	-0.149161
H	2.343402	0.226462	-1.407144
C	1.713393	-1.417821	1.619059
O	-1.909540	-0.183142	-0.718938
C	-2.801193	-1.075412	-0.235124
O	-2.584019	-1.759715	0.735946
C	-4.081572	-1.082346	-1.036564
C	-5.283489	-0.575011	-0.212810
C	-5.671009	-1.539440	0.908147
C	-5.014994	0.827312	0.333334
H	4.055523	-2.508369	0.899859
H	4.543775	-3.028922	-0.708723
H	4.703378	-0.721319	-1.504802

H	5.636047	-0.829241	-0.012504
H	2.491048	-2.103645	-1.684778
H	1.311758	1.198414	1.281956
H	0.206231	1.087780	-1.557091
H	-0.857180	-0.092917	1.049737
H	0.236433	-1.347163	-1.511737
H	-0.218867	-2.210662	-0.024941
H	1.205233	-3.710183	-0.742959
H	5.301299	1.131589	1.299696
H	3.653786	1.971623	1.375203
H	-2.592460	2.344864	-0.023078
H	-1.871098	4.050778	0.222222
H	2.653242	-1.086218	2.067923
H	1.586161	-2.485253	1.821099
H	0.901413	-0.887396	2.124815
H	-3.945352	-0.464715	-1.927747
H	-4.263876	-2.115403	-1.351561
H	-6.123147	-0.513251	-0.916630
H	-6.569095	-1.181212	1.421175
H	-5.880795	-2.538949	0.513997
H	-4.866072	-1.631889	1.641779
H	-4.189049	0.808414	1.056193
H	-5.894779	1.219703	0.851850
H	-4.754655	1.525659	-0.469649

M062X energy = -1154.62324597 a.u.

(1*R*,5*S*,6*R*,7*R*,8*S*,10*R*)-**3**, Conf. U

C	4.007396	-2.239951	-0.165000
C	4.628500	-0.860951	-0.427742
C	3.780791	0.246307	0.151808
C	2.341945	0.181434	-0.306037
C	1.699658	-1.194346	0.093472
C	2.542112	-2.285342	-0.597053
C	1.415298	1.269119	0.189989
C	0.036808	1.156954	-0.468703
C	-0.646115	-0.115649	-0.025945
C	0.245831	-1.300882	-0.421129
O	1.827076	2.613100	-0.105668
C	0.715801	3.409794	-0.139719
C	-0.499625	2.524899	-0.182411
O	1.950013	-3.536514	-0.301131
C	4.263757	1.162105	0.989034
C	-1.728084	2.983596	0.022616
O	0.771847	4.605399	-0.145712
H	2.344104	0.229430	-1.408293
C	1.715972	-1.414402	1.617122
O	-1.903668	-0.196910	-0.717781
C	-2.804928	-1.065372	-0.209621
O	-2.606101	-1.709052	0.792189
C	-4.073469	-1.100216	-1.030052
C	-5.293884	-0.590426	-0.236649
C	-5.697674	-1.542807	0.888975
C	-5.043988	0.820262	0.296998
H	4.068097	-2.491323	0.899591
H	4.568224	-3.011046	-0.709537
H	4.702582	-0.712184	-1.515209

H	5.645738	-0.814329	-0.028296
H	2.490484	-2.095455	-1.684898
H	1.313660	1.196805	1.282406
H	0.206091	1.085179	-1.555339
H	-0.850546	-0.103616	1.049728
H	0.251355	-1.343602	-1.519699
H	-0.193585	-2.233272	-0.057815
H	2.506373	-4.230099	-0.682064
H	5.307436	1.141185	1.290906
H	3.656858	1.974938	1.373734
H	-2.593250	2.330396	-0.014492
H	-1.878146	4.039109	0.230794
H	0.917001	-0.861786	2.119083
H	2.662791	-1.100633	2.064865
H	1.559906	-2.476001	1.825643
H	-3.928517	-0.498750	-1.930862
H	-4.239060	-2.140836	-1.329491
H	-6.120625	-0.542206	-0.956786
H	-5.897528	-2.547408	0.502549
H	-4.904327	-1.624046	1.636282
H	-6.605639	-1.181844	1.382560
H	-5.936288	1.215017	0.791958
H	-4.769865	1.509686	-0.509208
H	-4.233281	0.813755	1.037000

M062X energy = -1154.62316939 a.u.

(1*R*,5*S*,6*R*,7*R*,8*S*,10*R*)-**3**, Conf. V

C	3.436451	-2.864412	-0.223917
C	4.345476	-1.656076	-0.490174
C	3.783890	-0.390864	0.112881
C	2.359942	-0.124654	-0.316219
C	1.429202	-1.315940	0.106322
C	1.982067	-2.580480	-0.597669
C	1.719785	1.152669	0.181836
C	0.335948	1.351179	-0.443192
C	-0.605701	0.270963	0.034908
C	-0.020688	-1.086804	-0.374665
O	2.419027	2.364087	-0.142951
C	1.517026	3.392853	-0.161169
C	0.130818	2.807646	-0.165497
O	1.162604	-3.713431	-0.382778
C	4.476811	0.385612	0.943409
C	-0.956420	3.536910	0.053522
O	1.843146	4.544059	-0.183508
H	2.349953	-0.087560	-1.418969
C	1.429366	-1.523100	1.630359
O	-1.869700	0.481796	-0.612598
C	-2.939616	-0.120353	-0.046962
O	-2.860560	-0.790233	0.953455
C	-4.189207	0.182513	-0.844493
C	-5.484933	-0.426698	-0.299029
C	-5.449808	-1.956469	-0.308049
C	-5.836848	0.118033	1.086916
H	3.487200	-3.133950	0.840229
H	3.777381	-3.736301	-0.791395
H	4.427283	-1.517737	-1.578183

H	5.355576	-1.844756	-0.115010
H	1.927684	-2.396597	-1.680112
H	1.631323	1.118857	1.277159
H	0.456085	1.232971	-1.532281
H	-0.763869	0.333735	1.116631
H	-0.049459	-1.129670	-1.472670
H	-0.655111	-1.895395	-0.002076
H	1.380413	-4.079855	0.486391
H	5.494295	0.127806	1.224557
H	4.077077	1.312543	1.341317
H	-1.949002	3.099135	0.039712
H	-0.858087	4.601075	0.247696
H	0.862632	-0.749068	2.154459
H	2.440038	-1.524765	2.046829
H	0.942649	-2.473617	1.874117
H	-4.271029	1.275061	-0.908384
H	-3.999707	-0.160810	-1.869190
H	-6.272756	-0.106432	-0.993923
H	-6.423359	-2.360775	-0.013798
H	-5.215016	-2.341427	-1.306450
H	-4.698447	-2.330213	0.391791
H	-5.879966	1.212845	1.083318
H	-5.095515	-0.196595	1.825613
H	-6.815780	-0.253894	1.404959

M062X energy = -1154.62302830 a.u.

(1*R*,5*S*,6*R*,7*R*,8*S*,10*R*)-**3**, Conf. W

C	4.010539	-2.238708	-0.196502
C	4.626391	-0.851350	-0.427412
C	3.774909	0.243869	0.168674
C	2.339611	0.183906	-0.299313
C	1.694853	-1.193400	0.087210
C	2.540003	-2.288819	-0.609873
C	1.411736	1.273907	0.190105
C	0.034281	1.162183	-0.471148
C	-0.648389	-0.111941	-0.030804
C	0.244752	-1.293090	-0.434690
O	1.826237	2.616355	-0.106425
C	0.715448	3.414390	-0.141104
C	-0.501040	2.530640	-0.184809
O	1.995623	-3.581707	-0.427198
C	4.249959	1.145672	1.025377
C	-1.729197	2.990741	0.018911
O	0.772688	4.609785	-0.146411
H	2.350037	0.233157	-1.401594
C	1.700723	-1.416613	1.608915
O	-1.908217	-0.192144	-0.717618
C	-2.801178	-1.072093	-0.214127
O	-2.592321	-1.723942	0.780605
C	-4.073618	-1.107602	-1.027788
C	-5.288860	-0.594325	-0.228405
C	-5.685196	-1.542430	0.903410
C	-5.035351	0.818323	0.298273
H	4.093481	-2.504374	0.866620
H	4.556335	-3.001987	-0.760454
H	4.703137	-0.683029	-1.511552

H	5.642643	-0.809991	-0.024686
H	2.473660	-2.107303	-1.692061
H	1.305724	1.206035	1.282301
H	0.205436	1.091318	-1.557517
H	-0.849100	-0.104279	1.045812
H	0.256754	-1.322407	-1.533495
H	-0.196722	-2.231539	-0.089877
H	2.268747	-3.900804	0.444994
H	5.291568	1.122351	1.334061
H	3.638815	1.951297	1.418794
H	-2.595125	2.338646	-0.019437
H	-1.878167	4.046459	0.226835
H	0.957349	-0.800370	2.121393
H	2.672997	-1.192746	2.055535
H	1.437968	-2.456576	1.830654
H	-3.933040	-0.508117	-1.930576
H	-4.241950	-2.148709	-1.323593
H	-6.120153	-0.548812	-0.943378
H	-5.887317	-2.548560	0.522287
H	-4.887041	-1.620661	1.646001
H	-6.589874	-1.179666	1.401644
H	-4.220569	0.814518	1.033868
H	-5.924728	1.215463	0.796545
H	-4.765197	1.504347	-0.512134

M062X energy = -1154.62286370 a.u.

(1*R*,5*S*,6*R*,7*R*,8*S*,10*R*)-**3**, Conf. X

C	4.229195	-1.807209	-0.617651
C	4.420369	-0.431439	-1.271301
C	3.620553	0.634182	-0.560568
C	2.158553	0.276235	-0.426137
C	1.994195	-1.073551	0.362531
C	2.748634	-2.146300	-0.448137
C	1.258604	1.298445	0.234831
C	-0.204113	0.854317	0.150133
C	-0.399156	-0.385051	0.999351
C	0.505915	-1.495468	0.452013
O	1.251124	2.602307	-0.368320
C	0.048200	3.198259	-0.104462
C	-0.892713	2.151067	0.428527
O	2.572422	-3.385833	0.214544
C	4.165461	1.757125	-0.096797
C	-2.048417	2.452129	1.010007
O	-0.157580	4.361667	-0.295456
H	1.765751	0.105131	-1.442369
C	2.593614	-0.984085	1.777695
O	-1.774433	-0.793367	1.124730
C	-2.505196	-1.041321	0.022491
O	-2.079647	-0.985252	-1.106606
C	-3.928476	-1.407385	0.381632
C	-4.922328	-0.974798	-0.698853
C	-6.311996	-1.528238	-0.389140
C	-4.953902	0.548976	-0.822106
H	4.711405	-1.835085	0.365697
H	4.708209	-2.581319	-1.231430
H	4.076132	-0.488869	-2.314492

H	5.480138	-0.161795	-1.298248
H	2.279089	-2.186835	-1.447388
H	1.557428	1.432245	1.284798
H	-0.396598	0.592692	-0.898532
H	-0.137131	-0.150080	2.035889
H	0.145798	-1.765507	-0.546778
H	0.428116	-2.385189	1.083903
H	3.064115	-4.059746	-0.275123
H	5.228774	1.947338	-0.215285
H	3.577270	2.534146	0.380090
H	-2.709636	1.684834	1.400906
H	-2.346163	3.492525	1.104434
H	2.744354	-1.994711	2.166520
H	1.934277	-0.456991	2.472878
H	3.552140	-0.458438	1.781402
H	-3.954356	-2.497370	0.513378
H	-4.180533	-0.965915	1.352219
H	-4.571755	-1.391591	-1.650168
H	-6.678776	-1.139269	0.568285
H	-7.028533	-1.237002	-1.163147
H	-6.304870	-2.621388	-0.327773
H	-5.648674	0.862872	-1.606997
H	-3.965759	0.950779	-1.067977
H	-5.287297	1.003040	0.119820

M062X energy = -1154.62280309 a.u.

Table S34. Cartesian coordinates and energies of the low-energy conformers calculated at the M06-2X/6-31+G(d,p) *in vacuo* level.

(1*R*,5*S*,6*S*,7*S*,10*R*)-**2**, Conf. A

C	3.449478	0.031552	-0.206769
C	2.922906	1.396767	-0.676516
C	1.560507	1.660512	-0.085433
C	0.560399	0.570881	-0.419037
C	1.049755	-0.776855	0.189157
C	2.430340	-1.075230	-0.449187
C	-0.894122	0.896944	-0.069596
C	-1.838648	-0.230003	-0.494620
C	-1.397273	-1.555721	0.133610
C	0.058354	-1.883687	-0.210490
C	-3.319136	0.132753	-0.244003
C	-4.256729	-0.984313	-0.710164
C	-3.619097	0.508901	1.209872
O	-1.332027	2.068260	-0.744903
C	1.321757	2.703027	0.717004
O	3.009749	-2.274761	0.039986
H	0.582673	0.446822	-1.516604
C	1.173655	-0.715155	1.718083
H	3.686853	0.064896	0.862442
H	4.374120	-0.226341	-0.732082
H	3.624395	2.190666	-0.404281
H	2.842235	1.390347	-1.772744
H	2.269289	-1.163381	-1.538592
H	-0.977428	1.043468	1.020412
H	-1.720935	-0.325363	-1.586993
H	-2.036092	-2.367052	-0.229274
H	-1.528949	-1.523437	1.222322
H	0.130555	-2.045702	-1.297288
H	0.342463	-2.824734	0.278945
H	-3.517731	1.017604	-0.858792
H	-4.220831	-1.847928	-0.036711
H	-5.291896	-0.630118	-0.730859
H	-3.999341	-1.329746	-1.717848
H	-4.688780	0.706038	1.333460
H	-3.082259	1.412257	1.512850
H	-3.354543	-0.299930	1.901212
H	-0.640895	2.738846	-0.684221
H	0.361438	2.862606	1.201385
H	2.103929	3.423778	0.937807
H	2.488279	-3.026582	-0.262117
H	1.657983	-1.626105	2.081602
H	1.766461	0.141446	2.046704
H	0.194355	-0.632573	2.197135

M062X energy = -737.415546252 a.u.

(1*R*,5*S*,6*S*,7*S*,10*R*)-**2**, Conf. B

C	3.444899	0.036136	-0.198893
C	2.921643	1.399814	-0.678989
C	1.560650	1.661036	-0.083267
C	0.560602	0.573705	-0.422436
C	1.043791	-0.779924	0.177702
C	2.424653	-1.076651	-0.443220
C	-0.894111	0.897548	-0.070622
C	-1.839965	-0.227295	-0.499527
C	-1.394131	-1.561605	0.108960
C	0.059738	-1.884297	-0.243604
C	-3.318741	0.134079	-0.236407
C	-4.259282	-0.978176	-0.708110
C	-3.610168	0.494201	1.223349
O	-1.331353	2.072820	-0.741096
C	1.326892	2.697790	0.727931
O	2.877937	-2.315782	0.082525
H	0.582230	0.455426	-1.520363
C	1.150913	-0.729880	1.709556
H	3.675058	0.076978	0.871543
H	4.379343	-0.209000	-0.719938
H	3.623967	2.194902	-0.412249
H	2.838233	1.386340	-1.774823
H	2.270480	-1.175753	-1.532594
H	-0.977099	1.041296	1.019416
H	-1.728843	-0.310357	-1.593610
H	-2.033317	-2.367217	-0.266021
H	-1.526926	-1.547328	1.198173
H	0.133747	-2.018943	-1.333129
H	0.369653	-2.829885	0.212571
H	-3.521815	1.025411	-0.840471
H	-4.217788	-1.848865	-0.044288
H	-5.294777	-0.624190	-0.718144
H	-4.008032	-1.312888	-1.720922
H	-4.679693	0.686364	1.356496
H	-3.074422	1.396222	1.532455
H	-3.337603	-0.321000	1.903967
H	-0.642399	2.744685	-0.672261
H	0.368121	2.854340	1.216233
H	2.110407	3.416289	0.951587
H	3.725341	-2.537651	-0.318305
H	1.777743	0.097201	2.051726
H	0.169320	-0.601560	2.173011
H	1.582108	-1.666012	2.073959

M062X energy = -737.415322472 a.u.

(1*R*,5*S*,6*S*,7*S*,10*R*)-**2**, Conf. C

C	3.448808	0.035836	-0.229759
C	2.916785	1.404485	-0.684723
C	1.561338	1.657017	-0.074018
C	0.560612	0.573488	-0.421164
C	1.042993	-0.784135	0.168680
C	2.429865	-1.083146	-0.453360
C	-0.895031	0.899159	-0.072399
C	-1.842687	-0.224832	-0.499220
C	-1.394608	-1.560304	0.106190
C	0.056133	-1.881550	-0.259883
C	-3.320466	0.136822	-0.231436

C	-4.262927	-0.972191	-0.706974
C	-3.609240	0.489867	1.230602
O	-1.328557	2.074554	-0.744097
C	1.330308	2.682622	0.752102
O	2.949588	-2.333495	-0.029605
H	0.585153	0.460191	-1.519505
C	1.140931	-0.738197	1.701323
H	3.705045	0.086959	0.837482
H	4.367918	-0.218876	-0.766973
H	3.620925	2.197398	-0.416051
H	2.822990	1.403200	-1.779544
H	2.276953	-1.201645	-1.535442
H	-0.980903	1.045426	1.016980
H	-1.734960	-0.307486	-1.593560
H	-2.037892	-2.364737	-0.264038
H	-1.518288	-1.546441	1.196829
H	0.122911	-2.000966	-1.351236
H	0.368409	-2.835492	0.177740
H	-3.523571	1.031262	-0.830889
H	-4.221415	-1.846174	-0.047493
H	-5.298019	-0.617071	-0.713547
H	-4.013791	-1.302171	-1.721793
H	-4.678193	0.683276	1.366139
H	-3.071802	1.389329	1.544320
H	-3.337741	-0.329737	1.906428
H	-0.642259	2.748294	-0.666089
H	0.372871	2.832781	1.245105
H	2.113545	3.400036	0.980230
H	3.283629	-2.242276	0.870888
H	1.848850	0.018062	2.050464
H	0.176594	-0.504774	2.159447
H	1.447900	-1.716488	2.087968

M062X energy = -737.414986247 a.u.

(1*R*,5*S*,6*S*,7*S*,10*R*)-**2**, Conf. D

C	3.398438	0.440868	-0.248543
C	2.652209	1.731935	-0.619692
C	1.285156	1.742322	0.017566
C	0.454111	0.533278	-0.367072
C	1.165092	-0.755408	0.141784
C	2.556179	-0.794712	-0.540957
C	-1.023391	0.614504	0.030796
C	-1.802310	-0.615731	-0.441821
C	-1.130966	-1.892163	0.079038
C	0.345211	-1.975172	-0.312130
C	-3.307581	-0.589037	-0.076018
C	-3.571448	-0.066247	1.339275
C	-4.156863	0.179912	-1.092648
O	-1.640253	1.751845	-0.559276
C	0.917662	2.679943	0.897209
O	3.327736	-1.916562	-0.140881
H	0.462857	0.481796	-1.470585
C	1.321383	-0.770018	1.668800
H	3.659263	0.446932	0.815707
H	4.335880	0.362943	-0.807593
H	3.230074	2.608540	-0.312991
H	2.539019	1.777556	-1.712065
H	2.378953	-0.841139	-1.630392
H	-1.095869	0.683410	1.128963
H	-1.726038	-0.620984	-1.541181
H	-1.661608	-2.761248	-0.325586
H	-1.239687	-1.950050	1.170023
H	0.410794	-2.055832	-1.408385
H	0.781999	-2.890276	0.109293
H	-3.639632	-1.637435	-0.106050
H	-4.621543	-0.216636	1.607883
H	-2.960535	-0.578092	2.091544
H	-3.362341	1.007412	1.396045
H	-5.220328	0.086064	-0.847393
H	-4.009391	-0.213017	-2.104148
H	-3.889514	1.238584	-1.097897
H	-1.050037	2.509486	-0.463275
H	-0.038484	2.656922	1.414499
H	1.587961	3.496651	1.149590
H	2.921912	-2.720710	-0.483109
H	1.791646	0.142996	2.040725
H	0.354086	-0.861779	2.170248
H	1.943396	-1.620004	1.963864

M062X energy = -737.414579931 a.u.

(1*R*,5*S*,6*S*,7*S*,10*R*)-**2**, Conf. E

C	3.393999	0.443994	-0.239054
C	2.651203	1.734770	-0.621026
C	1.285804	1.742571	0.020114
C	0.454430	0.536414	-0.370814
C	1.159478	-0.758874	0.129843
C	2.551642	-0.797623	-0.534090
C	-1.023138	0.615138	0.028378
C	-1.803167	-0.612725	-0.449520
C	-1.127477	-1.895745	0.051520
C	0.346033	-1.973341	-0.346918
C	-3.306209	-0.589134	-0.074383
C	-3.561856	-0.081041	1.347760
C	-4.162792	0.189295	-1.077646
O	-1.639487	1.756491	-0.556292
C	0.924191	2.674231	0.908368
O	3.205280	-1.980319	-0.096525
H	0.462383	0.490993	-1.474307
C	1.299757	-0.788654	1.659460
H	3.646183	0.456429	0.827122
H	4.339496	0.379705	-0.792776
H	3.229446	2.612962	-0.319441
H	2.536928	1.773031	-1.713437
H	2.383907	-0.854110	-1.624465
H	-1.095674	0.680691	1.126373
H	-1.733739	-0.606608	-1.549347
H	-1.659981	-2.758264	-0.364814
H	-1.235308	-1.971219	1.141801
H	0.409764	-2.025826	-1.444123
H	0.807748	-2.887237	0.040248
H	-3.637611	-1.637375	-0.112562
H	-4.609512	-0.237411	1.622708

H	-2.943485	-0.598361	2.090151
H	-3.356016	0.992831	1.413883
H	-5.224724	0.092470	-0.826563
H	-4.021529	-0.193506	-2.093920
H	-3.896065	1.248201	-1.074322
H	-1.052339	2.514923	-0.449326
H	-0.030064	2.648047	1.428875
H	1.596238	3.488538	1.164107
H	4.062623	-2.046524	-0.530778
H	0.322644	-0.832978	2.147591
H	1.871081	-1.672605	1.954785
H	1.809883	0.098168	2.043127

M062X energy = -737.414383358 a.u.

(1*R*,5*S*,6*S*,7*S*,10*R*)-**2**, Conf. F

C	3.397064	0.445285	-0.268125
C	2.646306	1.738402	-0.624509
C	1.287447	1.737824	0.029479
C	0.454615	0.535945	-0.369821
C	1.158910	-0.763047	0.120170
C	2.557418	-0.802856	-0.544955
C	-1.024331	0.616324	0.026168
C	-1.806212	-0.610980	-0.450111
C	-1.128516	-1.894595	0.048007
C	0.341361	-1.970453	-0.363643
C	-3.308218	-0.586741	-0.071141
C	-3.560262	-0.081489	1.352672
C	-4.166130	0.194863	-1.070784
O	-1.636896	1.758927	-0.558983
C	0.929908	2.658339	0.930997
O	3.275071	-1.980844	-0.212811
H	0.465139	0.496245	-1.473456
C	1.290528	-0.798836	1.650509
H	3.672090	0.471265	0.795342
H	4.329040	0.370398	-0.837367
H	3.226650	2.614305	-0.320109
H	2.521031	1.787702	-1.715017
H	2.394411	-0.877161	-1.629452
H	-1.100287	0.683211	1.123776
H	-1.739758	-0.605265	-1.550024
H	-1.664918	-2.756917	-0.363432
H	-1.227468	-1.969203	1.139561
H	0.395692	-2.008787	-1.461526
H	0.805968	-2.891201	0.004329
H	-3.640699	-1.634537	-0.110920
H	-4.607284	-0.238024	1.629740
H	-2.940637	-0.600809	2.092746
H	-3.354091	0.992222	1.420791
H	-5.227519	0.099090	-0.817201
H	-4.027995	-0.186252	-2.088058
H	-3.897955	1.253399	-1.066227
H	-1.051530	2.517641	-0.443580
H	-0.022551	2.626209	1.454544
H	1.601918	3.471164	1.191558
H	3.613994	-1.895491	0.686439
H	0.314797	-0.742055	2.139670
H	1.750243	-1.741896	1.966770
H	1.886990	0.032169	2.035990

M062X energy = -737.414038468 a.u.

(1*R*,5*S*,6*S*,7*S*,10*R*)-**2**, Conf. G

C	3.454482	0.009433	-0.133890
C	2.958711	1.372836	-0.633024
C	1.576635	1.674236	-0.102400
C	0.567548	0.589226	-0.429439
C	1.038727	-0.773974	0.167199
C	2.428639	-1.081822	-0.426800
C	-0.887854	0.897655	-0.054355
C	-1.837747	-0.222425	-0.508114
C	-1.393659	-1.573135	0.064245
C	0.065244	-1.879259	-0.275167
C	-3.315792	0.128869	-0.227077
C	-4.257144	-0.982219	-0.699739
C	-3.591299	0.474935	1.239377
O	-1.318868	2.148888	-0.575842
C	1.321941	2.767300	0.619834
O	2.846799	-2.341243	0.080416
H	0.585199	0.457770	-1.529660
C	1.121817	-0.724188	1.701093
H	3.642207	0.043410	0.945094
H	4.405424	-0.245382	-0.619966
H	3.658154	2.164520	-0.349088
H	2.920774	1.352638	-1.732487
H	2.300402	-1.155085	-1.522112
H	-0.955616	1.012267	1.035314
H	-1.734617	-0.288389	-1.606865
H	-2.024505	-2.371243	-0.340120
H	-1.542037	-1.587931	1.151271
H	0.154180	-2.003003	-1.365155
H	0.376333	-2.827521	0.174275
H	-3.533068	1.027126	-0.816059
H	-4.016336	-1.306925	-1.718542
H	-4.204264	-1.859159	-0.045227
H	-5.294044	-0.632408	-0.694285
H	-4.662054	0.645905	1.389926
H	-3.067790	1.385102	1.544729
H	-3.293239	-0.338921	1.910713
H	-1.032693	2.221003	-1.494694
H	0.327650	2.999582	0.984151
H	2.116060	3.475774	0.841665
H	3.724134	-2.541840	-0.263191
H	1.681476	0.146787	2.051531
H	0.128719	-0.671259	2.154325
H	1.614612	-1.628785	2.067408

M062X energy = -737.412562968 a.u.

Table S35. Cartesian coordinates and energies of the low-energy conformers calculated at the M06-2X/6-311+G(2d,p) *in vacuo* level.

(1*R*,5*S*,6*S*,7*S*,10*R*)-**2**, Conf. A

C	3.443735	0.030398	-0.199487
C	2.919660	1.394207	-0.668516
C	1.557040	1.658773	-0.085097
C	0.559397	0.570952	-0.419493
C	1.047387	-0.774479	0.189177
C	2.426052	-1.072347	-0.446448
C	-0.892581	0.895732	-0.071786
C	-1.834383	-0.229857	-0.497379
C	-1.394423	-1.554129	0.128852
C	0.059827	-1.880344	-0.212310
C	-3.312954	0.130482	-0.244923
C	-4.247918	-0.991611	-0.696499
C	-3.608486	0.517869	1.204507
O	-1.330091	2.067575	-0.745846
C	1.316731	2.697985	0.709378
O	3.003059	-2.275068	0.036642
H	0.584338	0.446944	-1.513346
C	1.167693	-0.712976	1.716226
H	3.677672	0.061940	0.867334
H	4.366983	-0.225983	-0.721713
H	3.617642	2.186008	-0.394164
H	2.842146	1.387461	-1.761824
H	2.267263	-1.155955	-1.532482
H	-0.976390	1.040406	1.014209
H	-1.717919	-0.324463	-1.586298
H	-2.029855	-2.362419	-0.237097
H	-1.529138	-1.524098	1.213721
H	0.133631	-2.040930	-1.295735
H	0.343175	-2.818263	0.276116
H	-3.517563	1.006463	-0.864650
H	-4.207881	-1.844820	-0.015095
H	-5.280938	-0.640109	-0.718052
H	-3.992259	-1.346205	-1.698244
H	-4.677281	0.701459	1.331528
H	-3.083044	1.429106	1.492807
H	-3.330109	-0.278193	1.900370
H	-0.634444	2.730609	-0.691113
H	0.355083	2.860363	1.183679
H	2.096622	3.417023	0.930832
H	2.470525	-3.018103	-0.260666
H	1.650477	-1.621419	2.079448
H	1.757106	0.141913	2.045494
H	0.189746	-0.632713	2.191349

M062X energy = -737.598954464 a.u.

(1*R*,5*S*,6*S*,7*S*,10*R*)-**2**, Conf. B

C	3.438791	0.035977	-0.192688
C	2.917151	1.398006	-0.671706
C	1.556174	1.658915	-0.082745
C	0.559137	0.572912	-0.422587
C	1.041585	-0.778340	0.177914
C	2.420847	-1.073254	-0.441163
C	-0.893072	0.895516	-0.072481
C	-1.836311	-0.227644	-0.502228
C	-1.391925	-1.560399	0.104402
C	0.060572	-1.880977	-0.245492
C	-3.313204	0.131333	-0.237568
C	-4.250903	-0.986282	-0.694594
C	-3.600469	0.503308	1.217672
O	-1.329793	2.071366	-0.741623
C	1.320516	2.691904	0.721045
O	2.875491	-2.314614	0.077182
H	0.583541	0.454598	-1.516789
C	1.145208	-0.728164	1.707693
H	3.666363	0.075245	0.875144
H	4.370909	-0.208265	-0.711878
H	3.615451	2.191529	-0.403011
H	2.836380	1.384419	-1.764576
H	2.268245	-1.167523	-1.527181
H	-0.976582	1.037240	1.013545
H	-1.726241	-0.310148	-1.592787
H	-2.027383	-2.363178	-0.273585
H	-1.527361	-1.548325	1.189737
H	0.135991	-2.013395	-1.331686
H	0.368127	-2.824540	0.208997
H	-3.522224	1.013624	-0.846943
H	-4.001208	-1.330512	-1.701410
H	-4.205284	-1.846458	-0.022479
H	-5.284258	-0.635032	-0.705772
H	-4.669094	0.681536	1.353997
H	-3.076642	1.413683	1.511816
H	-3.313861	-0.298672	1.903274
H	-0.636935	2.736259	-0.677247
H	0.360599	2.850414	1.200040
H	2.101313	3.409229	0.945093
H	3.741180	-2.506520	-0.293749
H	1.767124	0.098505	2.050184
H	0.164736	-0.604044	2.167357
H	1.576940	-1.660546	2.072580

M062X energy = -737.598564608 a.u.

(1*R*,5*S*,6*S*,7*S*,10*R*)-**2**, Conf. C

C	3.443030	0.036375	-0.225105
C	2.911867	1.403165	-0.678642
C	1.556857	1.655253	-0.073595
C	0.558965	0.573201	-0.421298
C	1.040811	-0.782308	0.167993
C	2.426362	-1.079561	-0.450902
C	-0.893998	0.897130	-0.073209
C	-1.839094	-0.224724	-0.502150
C	-1.392406	-1.559239	0.100090
C	0.057100	-1.877894	-0.263272
C	-3.314965	0.134287	-0.232497

C	-4.254611	-0.979895	-0.693843
C	-3.599304	0.498441	1.225283
O	-1.327380	2.074060	-0.741678
C	1.324420	2.676905	0.745593
O	2.946872	-2.329476	-0.028460
H	0.585845	0.460333	-1.516039
C	1.137078	-0.739404	1.698403
H	3.697253	0.084289	0.839354
H	4.359751	-0.216840	-0.760968
H	3.612157	2.194614	-0.408683
H	2.819552	1.401748	-1.770487
H	2.275559	-1.195340	-1.530095
H	-0.979948	1.039895	1.012366
H	-1.732380	-0.305642	-1.593078
H	-2.031824	-2.360604	-0.273962
H	-1.518691	-1.548527	1.186879
H	0.125265	-1.994689	-1.351409
H	0.367710	-2.829358	0.172907
H	-3.524086	1.020053	-0.836785
H	-4.209099	-1.843647	-0.026322
H	-5.287549	-0.627434	-0.701530
H	-4.006943	-1.319074	-1.702817
H	-4.667413	0.677494	1.364254
H	-3.074014	1.406414	1.524304
H	-3.313239	-0.308184	1.905731
H	-0.636884	2.740585	-0.667846
H	0.366185	2.828842	1.230140
H	2.105149	3.392993	0.973863
H	3.277627	-2.234740	0.870290
H	1.836074	0.020348	2.048510
H	0.172667	-0.517285	2.154758
H	1.452673	-1.713306	2.080668

M062X energy = -737.598552711 a.u.

(1*R*,5*S*,6*S*,7*S*,10*R*)-**2**, Conf. D

C	3.394129	0.436770	-0.243515
C	2.651205	1.726963	-0.614904
C	1.284185	1.740297	0.016121
C	0.453935	0.533680	-0.367097
C	1.162814	-0.753012	0.142751
C	2.551313	-0.793881	-0.538472
C	-1.020672	0.615890	0.029832
C	-1.798423	-0.612564	-0.441511
C	-1.128796	-1.887373	0.078210
C	0.345260	-1.970956	-0.311249
C	-3.301268	-0.588953	-0.072503
C	-3.563452	-0.060053	1.338237
C	-4.155838	0.169908	-1.088844
O	-1.635703	1.753502	-0.560858
C	0.916912	2.676325	0.886772
O	3.320082	-1.919280	-0.144334
H	0.464179	0.481445	-1.466925
C	1.316945	-0.767154	1.667837
H	3.652502	0.441646	0.818130
H	4.329604	0.358674	-0.799930
H	3.226913	2.600790	-0.307777
H	2.540267	1.771256	-1.704406
H	2.374663	-0.836430	-1.624105
H	-1.092942	0.685184	1.123802
H	-1.724282	-0.617961	-1.537242
H	-1.657941	-2.753084	-0.327170
H	-1.239313	-1.945974	1.165465
H	0.411334	-2.051207	-1.404063
H	0.780350	-2.883027	0.109871
H	-3.628897	-1.635041	-0.096076
H	-2.950460	-0.564369	2.089451
H	-3.358425	1.011755	1.387893
H	-4.609820	-0.212270	1.608823
H	-3.898753	1.228113	-1.097486
H	-5.215290	0.069603	-0.841638
H	-4.007763	-0.223683	-2.096797
H	-1.039982	2.504601	-0.469851
H	-0.040841	2.657908	1.395194
H	1.586027	3.490883	1.137888
H	2.900833	-2.716711	-0.479702
H	1.937780	-1.614356	1.962812
H	1.783986	0.144149	2.039257
H	0.351738	-0.860983	2.166122

M062X energy = -737.597842623 a.u.

(1*R*,5*S*,6*S*,7*S*,10*R*)-**2**, Conf. E

C	3.389029	0.440395	-0.235113
C	2.648936	1.730100	-0.616412
C	1.283933	1.739792	0.019089
C	0.453873	0.535761	-0.370490
C	1.157390	-0.757479	0.130988
C	2.546881	-0.796471	-0.532699
C	-1.020829	0.615512	0.027742
C	-1.800039	-0.610280	-0.449077
C	-1.126203	-1.891455	0.051579
C	0.345077	-1.969476	-0.345674
C	-3.300528	-0.589131	-0.071152
C	-3.554423	-0.073958	1.346249
C	-4.161958	0.179228	-1.074560
O	-1.635212	1.757414	-0.556960
C	0.922096	2.669361	0.898773
O	3.201356	-1.981122	-0.103003
H	0.463593	0.489435	-1.470277
C	1.296218	-0.786236	1.658393
H	3.639642	0.451512	0.828225
H	4.331637	0.375398	-0.787643
H	3.224550	2.605695	-0.313989
H	2.536430	1.767805	-1.705859
H	2.378684	-0.848397	-1.619236
H	-1.093101	0.680722	1.121614
H	-1.732474	-0.604786	-1.545226
H	-1.656891	-2.751047	-0.364842
H	-1.235273	-1.966791	1.138177
H	0.408648	-2.020991	-1.439445
H	0.803761	-2.881597	0.040495
H	-3.628013	-1.634988	-0.102472
H	-4.598512	-0.231148	1.622895

H	-2.934669	-0.584068	2.087957
H	-3.351649	0.997934	1.404715
H	-5.219923	0.076240	-0.821789
H	-4.020006	-0.204616	-2.087158
H	-3.905323	1.237636	-1.074862
H	-1.041635	2.508859	-0.456645
H	-0.033592	2.646884	1.410811
H	1.592458	3.481900	1.153285
H	4.074183	-2.015621	-0.504043
H	0.321467	-0.834390	2.143896
H	1.868443	-1.665911	1.954262
H	1.801689	0.100016	2.041024

M062X energy = -737.597479768 a.u.

(1*R*,5*S*,6*S*,7*S*,10*R*)-**2**, Conf. F

C	3.392303	0.442884	-0.265531
C	2.643378	1.734545	-0.620811
C	1.285423	1.735449	0.028687
C	0.453895	0.535833	-0.369555
C	1.156814	-0.761253	0.120238
C	2.553176	-0.801293	-0.542866
C	-1.022045	0.616742	0.026049
C	-1.803103	-0.607997	-0.450254
C	-1.127093	-1.890360	0.045626
C	0.340672	-1.965961	-0.364525
C	-3.302471	-0.586850	-0.067853
C	-3.552227	-0.075878	1.351741
C	-4.165700	0.185552	-1.066566
O	-1.632928	1.760271	-0.558224
C	0.928235	2.653404	0.922460
O	3.270828	-1.979023	-0.211877
H	0.465812	0.496009	-1.469594
C	1.288625	-0.799082	1.648078
H	3.666733	0.466030	0.794729
H	4.321194	0.367984	-0.834028
H	3.221096	2.608113	-0.316257
H	2.518717	1.783116	-1.708309
H	2.390906	-0.873347	-1.624146
H	-1.097440	0.682582	1.119538
H	-1.738895	-0.601470	-1.546526
H	-1.661542	-2.749248	-0.367196
H	-1.227261	-1.966336	1.133391
H	0.395014	-2.002657	-1.459073
H	0.802720	-2.884547	0.002549
H	-3.630854	-1.632312	-0.101640
H	-4.595541	-0.233665	1.630845
H	-2.930744	-0.588392	2.090455
H	-3.349409	0.995878	1.413016
H	-5.223039	0.083299	-0.811034
H	-4.027183	-0.195566	-2.080606
H	-3.907647	1.243619	-1.064591
H	-1.042522	2.512691	-0.446116
H	-0.025121	2.624561	1.438612
H	1.598683	3.464385	1.181732
H	3.606351	-1.890393	0.685739
H	0.315087	-0.750571	2.135677
H	1.754199	-1.737038	1.960559
H	1.878180	0.032875	2.033574

M062X energy = -737.597466797 a.u.

Table S36. Cartesian coordinates and energies of the low-energy conformers calculated at the B3LYP/6-31+G(d,p) SMD (solvent: $CHCl_3$) level.

(1*R*,5*S*,6*S*,7*S*,10*R*)-**2**, Conf. A

C	3.474215	0.016251	-0.260844
C	2.941909	1.406191	-0.666975
C	1.593301	1.668493	-0.035364
C	0.574160	0.590462	-0.377132
C	1.059575	-0.790474	0.194366
C	2.435487	-1.083698	-0.484077
C	-0.891359	0.932821	-0.038446
C	-1.859511	-0.190239	-0.454378
C	-1.408219	-1.534108	0.144695
C	0.040457	-1.873939	-0.231216
C	-3.349631	0.162266	-0.174846
C	-4.304155	-0.784173	-0.922526
C	-3.716214	0.225985	1.317758
O	-1.301125	2.114061	-0.756543
C	1.383463	2.708594	0.786049
O	3.034447	-2.307456	-0.027494
H	0.597927	0.483832	-1.474347
C	1.201391	-0.773004	1.729401
H	3.768063	0.024211	0.795320
H	4.372687	-0.225642	-0.839741
H	3.661182	2.181824	-0.384710
H	2.837687	1.440970	-1.761395
H	2.245896	-1.168026	-1.566373
H	-0.988815	1.122080	1.039918
H	-1.763741	-0.270175	-1.548825
H	-2.064161	-2.335051	-0.213991
H	-1.515776	-1.517061	1.235531
H	0.099320	-2.004260	-1.321975
H	0.313898	-2.836852	0.220424
H	-3.503057	1.164562	-0.591006
H	-4.260600	-1.808550	-0.533325
H	-5.341579	-0.443007	-0.823145
H	-4.070703	-0.822106	-1.993764
H	-4.743537	0.589672	1.439013
H	-3.064658	0.906894	1.877085
H	-3.662493	-0.758891	1.797433
H	-0.644916	2.809023	-0.601283
H	0.438733	2.881789	1.295622
H	2.177927	3.420643	0.998411
H	2.481700	-3.050623	-0.308196
H	1.587646	-1.734965	2.080890
H	1.884646	0.007367	2.073355
H	0.240935	-0.599073	2.222584

B3LYP energy = -737.769717432 a.u.

(1*R*,5*S*,6*S*,7*S*,10*R*)-**2**, Conf. B

C	3.471616	0.022132	-0.278268
C	2.935183	1.414969	-0.671684
C	1.590945	1.667229	-0.027814
C	0.572581	0.591569	-0.377929
C	1.055509	-0.796470	0.178767
C	2.433918	-1.087463	-0.493998
C	-0.893353	0.931391	-0.036390
C	-1.862665	-0.189060	-0.457058
C	-1.407386	-1.538512	0.127480
C	0.038433	-1.873749	-0.260909
C	-3.351504	0.161487	-0.168358
C	-4.309024	-0.779293	-0.919338
C	-3.711836	0.212438	1.326288
O	-1.302928	2.117670	-0.746594
C	1.384706	2.696123	0.808617
O	2.974163	-2.364639	-0.118261
H	0.596431	0.493441	-1.475745
C	1.187187	-0.790155	1.716318
H	3.776040	0.035983	0.776980
H	4.366637	-0.216238	-0.864174
H	3.655149	2.190421	-0.390273
H	2.822791	1.454659	-1.764914
H	2.248369	-1.186838	-1.570984
H	-0.990464	1.115100	1.042939
H	-1.771490	-0.259666	-1.552499
H	-2.065753	-2.335702	-0.235352
H	-1.510450	-1.531830	1.219286
H	0.094118	-1.981888	-1.353661
H	0.329766	-2.841180	0.162935
H	-3.507415	1.167133	-0.575541
H	-4.264264	-1.806330	-0.537460
H	-5.346027	-0.438487	-0.814061
H	-4.079051	-0.810061	-1.991529
H	-4.739240	0.573336	1.455174
H	-3.059019	0.889737	1.888671
H	-3.654389	-0.776418	1.797223
H	-0.647143	2.811780	-0.585910
H	0.442161	2.861431	1.324764
H	2.178810	3.407507	1.024666
H	3.303031	-2.307217	0.791192
H	1.921909	-0.062117	2.070130
H	0.239081	-0.545970	2.202563
H	1.484655	-1.780653	2.080074

B3LYP energy = -737.769534039 a.u.

(1*R*,5*S*,6*S*,7*S*,10*R*)-**2**, Conf. C

C	3.469790	0.023247	-0.248370
C	2.939993	1.411446	-0.666184
C	1.591067	1.669684	-0.033554
C	0.573684	0.591342	-0.379754
C	1.055452	-0.794350	0.185164
C	2.431147	-1.082607	-0.477909
C	-0.892322	0.931044	-0.039602
C	-1.861367	-0.190014	-0.459591
C	-1.406821	-1.539749	0.124386
C	0.041061	-1.874929	-0.256127
C	-3.350093	0.160561	-0.169568

C	-4.308486	-0.780575	-0.919032
C	-3.708956	0.212066	1.325474
O	-1.302021	2.115488	-0.753298
C	1.381433	2.705309	0.793634
O	2.913615	-2.346513	0.009353
H	0.597615	0.489348	-1.477145
C	1.182532	-0.785521	1.722846
H	3.753313	0.036666	0.810371
H	4.377269	-0.213953	-0.819342
H	3.657763	2.189802	-0.387429
H	2.835955	1.439496	-1.760727
H	2.250747	-1.169967	-1.561645
H	-0.990135	1.117039	1.039155
H	-1.770885	-0.259904	-1.555211
H	-2.062791	-2.336964	-0.242812
H	-1.515071	-1.535212	1.215466
H	0.102493	-1.987468	-1.348323
H	0.330678	-2.839331	0.174855
H	-3.506572	1.166043	-0.576949
H	-4.263376	-1.807367	-0.536563
H	-5.345370	-0.439674	-0.812737
H	-4.079794	-0.811925	-1.991512
H	-4.735979	0.573787	1.455226
H	-3.055061	0.888825	1.887181
H	-3.651758	-0.776663	1.796630
H	-0.647221	2.810591	-0.593112
H	0.437226	2.874329	1.305539
H	2.174999	3.417735	1.008135
H	3.744018	-2.549243	-0.445163
H	1.889323	-0.030740	2.076654
H	0.223493	-0.576806	2.204737
H	1.527701	-1.761970	2.075406

B3LYP energy = -737.769283236 a.u.

(1*R*,5*S*,6*S*,7*S*,10*R*)-**2**, Conf. D

C	3.428331	0.425209	-0.256976
C	2.684169	1.733873	-0.590875
C	1.319190	1.750217	0.060652
C	0.472691	0.549677	-0.342221
C	1.170424	-0.772093	0.142835
C	2.572543	-0.808847	-0.543463
C	-1.025158	0.645818	0.021261
C	-1.823909	-0.579695	-0.458415
C	-1.151790	-1.878606	0.029745
C	0.327433	-1.966629	-0.362102
C	-3.341546	-0.574039	-0.090243
C	-3.632284	-0.170897	1.365626
C	-4.209438	0.246387	-1.059449
O	-1.606184	1.803405	-0.610937
C	0.967994	2.695788	0.945946
O	3.356732	-1.946968	-0.150474
H	0.500663	0.512837	-1.443952
C	1.310709	-0.832633	1.676752
H	3.720897	0.423312	0.799461
H	4.351395	0.357204	-0.843602
H	3.278856	2.596155	-0.272324
H	2.562659	1.806669	-1.681720
H	2.394369	-0.862368	-1.629719
H	-1.133946	0.743662	1.110333
H	-1.758964	-0.573023	-1.557206
H	-1.687435	-2.733370	-0.401039
H	-1.258253	-1.970253	1.117790
H	0.399369	-2.008181	-1.459098
H	0.745602	-2.907471	0.020607
H	-3.662853	-1.620649	-0.198320
H	-3.033426	-0.743334	2.083646
H	-3.438251	0.894387	1.535132
H	-4.687040	-0.352236	1.604552
H	-3.995307	1.315917	-0.983661
H	-5.273911	0.091718	-0.842076
H	-4.037260	-0.055819	-2.099782
H	-1.040265	2.566213	-0.420202
H	0.017434	2.695217	1.473384
H	1.650360	3.505892	1.193267
H	2.921748	-2.751304	-0.467143
H	1.820712	-1.754931	1.972203
H	1.886094	0.007602	2.073406
H	0.336220	-0.816519	2.172912

B3LYP energy = -737.767695034 a.u.

(1*R*,5*S*,6*S*,7*S*,10*R*)-**2**, Conf. E

C	3.425720	0.427068	-0.276543
C	2.678357	1.738439	-0.596846
C	1.319482	1.747830	0.067105
C	0.470904	0.551542	-0.342050
C	1.166677	-0.776614	0.127878
C	2.569603	-0.815111	-0.554493
C	-1.026288	0.644676	0.026714
C	-1.826242	-0.577011	-0.461610
C	-1.151683	-1.880339	0.012923
C	0.324713	-1.964696	-0.387399
C	-3.343307	-0.573930	-0.091746
C	-3.632989	-0.183596	1.367834
C	-4.212232	0.254633	-1.053150
O	-1.608576	1.808923	-0.592037
C	0.975915	2.683250	0.966224
O	3.299433	-2.013423	-0.244783
H	0.495694	0.522900	-1.443882
C	1.302453	-0.849771	1.663321
H	3.729950	0.432754	0.778758
H	4.343677	0.361050	-0.871540
H	3.275136	2.599991	-0.279847
H	2.547640	1.814786	-1.686135
H	2.396822	-0.884065	-1.635933
H	-1.132434	0.732225	1.117003
H	-1.763186	-0.560810	-1.560377
H	-1.689968	-2.730918	-0.423067
H	-1.255444	-1.981782	1.100762
H	0.392083	-1.989874	-1.484649
H	0.761796	-2.902426	-0.026941
H	-3.664514	-1.619575	-0.208505
H	-4.687743	-0.366338	1.605820

H	-3.034221	-0.763499	2.079940
H	-3.438024	0.879982	1.546933
H	-5.276666	0.096305	-0.837987
H	-4.038958	-0.037197	-2.096231
H	-3.999885	1.323748	-0.966798
H	-1.040896	2.569370	-0.397314
H	0.028682	2.677200	1.499634
H	1.659478	3.491457	1.216521
H	3.618339	-1.955004	0.668174
H	0.330815	-0.781826	2.159982
H	1.746617	-1.806116	1.963454
H	1.921845	-0.044574	2.067809

B3LYP energy = -737.767553923 a.u.

(1*R*,5*S*,6*S*,7*S*,10*R*)-**2**, Conf. F

C	3.487192	-0.007109	-0.202407
C	2.979960	1.386747	-0.614938
C	1.611340	1.679965	-0.036430
C	0.582454	0.610698	-0.385162
C	1.053976	-0.783738	0.175624
C	2.441597	-1.086134	-0.473688
C	-0.885994	0.944288	-0.033810
C	-1.860441	-0.179807	-0.458786
C	-1.407283	-1.540711	0.096927
C	0.043161	-1.862126	-0.280547
C	-3.345645	0.163781	-0.144858
C	-4.314601	-0.750353	-0.913603
C	-3.689695	0.167057	1.354748
O	-1.280566	2.193147	-0.630276
C	1.389326	2.755146	0.731160
O	3.009987	-2.327972	-0.026111
H	0.607509	0.505008	-1.485376
C	1.166046	-0.780101	1.713511
H	3.744512	-0.013295	0.863245
H	4.401657	-0.255343	-0.753112
H	3.697588	2.155430	-0.309829
H	2.916162	1.426164	-1.713223
H	2.279163	-1.147273	-1.562327
H	-0.974659	1.123288	1.042644
H	-1.783853	-0.237662	-1.558545
H	-2.059906	-2.332777	-0.287602
H	-1.518751	-1.556758	1.186944
H	0.110158	-1.966717	-1.373846
H	0.318330	-2.834082	0.150149
H	-3.502574	1.183902	-0.514435
H	-4.097959	-0.751070	-1.989216
H	-4.268869	-1.788470	-0.562825
H	-5.349194	-0.410191	-0.785874
H	-4.713773	0.528582	1.505554
H	-3.028078	0.821931	1.932854
H	-3.633549	-0.837095	1.791717
H	-1.136355	2.132483	-1.586962
H	0.412859	2.992883	1.139029
H	2.195853	3.449667	0.958490
H	2.454114	-3.057771	-0.333925
H	1.820923	0.015262	2.077578
H	0.193164	-0.637740	2.192058
H	1.571795	-1.735367	2.061479

B3LYP energy = -737.767356153 a.u.

(1*R*,5*S*,6*S*,7*S*,10*R*)-**2**, Conf. G

C	3.423002	0.426010	-0.250920
C	2.682051	1.735699	-0.592784
C	1.318147	1.750592	0.061290
C	0.471680	0.550180	-0.341703
C	1.167595	-0.774824	0.139345
C	2.563822	-0.811093	-0.540509
C	-1.025765	0.643947	0.024446
C	-1.824069	-0.580346	-0.459885
C	-1.150742	-1.880938	0.023073
C	0.327454	-1.968223	-0.369967
C	-3.342018	-0.575791	-0.093631
C	-3.635031	-0.176159	1.362748
C	-4.209242	0.246005	-1.062380
O	-1.607678	1.804528	-0.602076
C	0.969572	2.693853	0.950182
O	3.238089	-2.012397	-0.127933
H	0.497673	0.515017	-1.443244
C	1.304809	-0.839122	1.674640
H	3.710131	0.426741	0.806847
H	4.350997	0.363133	-0.834533
H	3.276806	2.599379	-0.277971
H	2.559835	1.803228	-1.683779
H	2.387311	-0.863619	-1.627082
H	-1.133870	0.737207	1.113961
H	-1.758265	-0.569335	-1.558622
H	-1.687010	-2.733912	-0.410830
H	-1.258741	-1.977107	1.110806
H	0.399755	-2.002936	-1.466828
H	0.762140	-2.902127	0.002235
H	-3.662461	-1.622348	-0.204333
H	-4.690042	-0.358556	1.599901
H	-3.036816	-0.750431	2.079796
H	-3.441552	0.888787	1.535150
H	-5.274080	0.089304	-0.847982
H	-4.034261	-0.053325	-2.103090
H	-3.996924	1.315689	-0.983310
H	-1.041034	2.566346	-0.409738
H	0.020508	2.691004	1.480318
H	1.651870	3.504044	1.197439
H	4.088274	-2.055779	-0.588872
H	0.329044	-0.809520	2.167520
H	1.799830	-1.769640	1.967891
H	1.889997	-0.007035	2.074753

B3LYP energy = -737.767345691 a.u.

(1*R*,5*S*,6*S*,7*S*,10*R*)-**2**, Conf. H

C	3.485288	-0.001219	-0.221568
C	2.973136	1.395897	-0.620272
C	1.609524	1.678286	-0.026164
C	0.581335	0.612712	-0.385526
C	1.049612	-0.788763	0.159331

C	2.439628	-1.089467	-0.483897
C	-0.887978	0.944467	-0.034168
C	-1.863597	-0.178064	-0.461164
C	-1.405819	-1.544321	0.079182
C	0.041360	-1.859879	-0.313005
C	-3.347170	0.163246	-0.137070
C	-4.319907	-0.745586	-0.907390
C	-3.683434	0.153857	1.364289
O	-1.280443	2.195549	-0.628124
C	1.391883	2.739314	0.762066
O	2.948387	-2.382846	-0.117989
H	0.608206	0.516615	-1.486304
C	1.149315	-0.798265	1.699544
H	3.755031	-0.001399	0.843249
H	4.395709	-0.245672	-0.780916
H	3.692097	2.164086	-0.316719
H	2.899043	1.441092	-1.717455
H	2.280569	-1.166604	-1.567297
H	-0.977977	1.122418	1.042253
H	-1.792588	-0.227536	-1.561730
H	-2.061362	-2.332080	-0.309425
H	-1.511670	-1.571822	1.169964
H	0.103566	-1.938395	-1.408251
H	0.337880	-2.836144	0.085828
H	-3.507144	1.186266	-0.497533
H	-5.353839	-0.405858	-0.773074
H	-4.107720	-0.740093	-1.983850
H	-4.272824	-1.785825	-0.563254
H	-3.020907	0.806272	1.944248
H	-3.621725	-0.853692	1.792453
H	-4.707839	0.510951	1.523398
H	-1.154193	2.129121	-1.587020
H	0.418027	2.967289	1.181768
H	2.197925	3.432794	0.994271
H	3.271270	-2.341668	0.794440
H	1.450173	-1.789267	2.059391
H	1.868174	-0.065651	2.075297
H	0.189504	-0.570113	2.170050

B3LYP energy = -737.767341767 a.u.

(1*R*,5*S*,6*S*,7*S*,10*R*)-**2**, Conf. I

C	3.482803	-0.002498	-0.183489
C	2.980933	1.389931	-0.609484
C	1.610352	1.681323	-0.034413
C	0.582838	0.612569	-0.389152
C	1.048705	-0.786934	0.164374
C	2.436981	-1.084830	-0.466231
C	-0.886087	0.943731	-0.036747
C	-1.862307	-0.178022	-0.465142
C	-1.405847	-1.546908	0.069130
C	0.044314	-1.861335	-0.312432
C	-3.345147	0.162618	-0.136069
C	-4.320394	-0.745620	-0.903950
C	-3.676684	0.151453	1.366420
O	-1.280206	2.194784	-0.630020
C	1.387292	2.753973	0.736476
O	2.883348	-2.368620	0.004628
H	0.608882	0.512430	-1.489579
C	1.141050	-0.793799	1.704945
H	3.726098	-0.003852	0.885268
H	4.407633	-0.246583	-0.723171
H	3.697095	2.160858	-0.306490
H	2.920528	1.421840	-1.708128
H	2.286557	-1.145529	-1.556849
H	-0.974605	1.120886	1.039885
H	-1.793745	-0.222909	-1.566167
H	-2.057852	-2.332796	-0.329161
H	-1.519616	-1.581643	1.158595
H	0.114979	-1.943146	-1.407093
H	0.337985	-2.835150	0.093439
H	-3.506617	1.186010	-0.494781
H	-4.111172	-0.740211	-1.981028
H	-4.273033	-1.785856	-0.559867
H	-5.353774	-0.405332	-0.766752
H	-4.700701	0.508139	1.529011
H	-3.012492	0.803223	1.945102
H	-3.613405	-0.856462	1.793376
H	-1.153987	2.128897	-1.588924
H	0.410156	2.989966	1.143685
H	2.193079	3.448383	0.966834
H	3.739931	-2.558434	-0.404667
H	1.813846	-0.019829	2.082694
H	0.166005	-0.623159	2.169212
H	1.510283	-1.762966	2.053270

B3LYP energy = -737.767051037 a.u.

(1*R*,5*S*,6*S*,7*S*,10*R*)-**2**, Conf. J

C	3.485776	-0.004588	-0.223235
C	2.972620	1.389186	-0.631181
C	1.608320	1.677665	-0.041402
C	0.582614	0.606526	-0.391598
C	1.056255	-0.779698	0.184583
C	2.438058	-1.086960	-0.474928
C	-0.882365	0.942158	-0.053217
C	-1.855257	-0.190749	-0.460031
C	-1.404876	-1.536299	0.134000
C	0.043175	-1.866285	-0.245474
C	-3.346680	0.150131	-0.169469
C	-4.301817	-0.813930	-0.894109
C	-3.700388	0.232709	1.325903
O	-1.191801	2.148354	-0.780231
C	1.391033	2.744182	0.738828
O	3.011606	-2.324211	-0.020224
H	0.609401	0.500490	-1.489197
C	1.183455	-0.759161	1.721401
H	3.758896	-0.008000	0.838570
H	4.392460	-0.253575	-0.786407
H	3.691707	2.158467	-0.330921
H	2.897803	1.430232	-1.728400
H	2.265115	-1.158797	-1.560966
H	-0.972502	1.141531	1.023385
H	-1.766920	-0.269055	-1.554989

H	-2.058400	-2.337935	-0.227863
H	-1.511346	-1.524778	1.224942
H	0.101757	-1.991695	-1.336890
H	0.321560	-2.829610	0.202156
H	-3.534172	1.141535	-0.605690
H	-4.248477	-1.829679	-0.485424
H	-5.340180	-0.476760	-0.792889
H	-4.074470	-0.870210	-1.965552
H	-4.730577	0.585714	1.452871
H	-3.050399	0.924731	1.873659
H	-3.630573	-0.745721	1.815443
H	-2.018327	2.515678	-0.438555
H	0.417572	2.974426	1.159066
H	2.196216	3.439955	0.966604
H	2.457747	-3.057336	-0.323666
H	1.575122	-1.717335	2.077554
H	1.855561	0.028610	2.069858
H	0.217455	-0.593359	2.206507

B3LYP energy = -737.766744927 a.u.

(1*R*,5*S*,6*S*,7*S*,10*R*)-**2**, Conf. K

C	3.483343	0.000885	-0.242839
C	2.965573	1.397498	-0.638019
C	1.606453	1.676239	-0.033059
C	0.581133	0.607945	-0.391209
C	1.051881	-0.785339	0.169271
C	2.435831	-1.090920	-0.484874
C	-0.884210	0.941203	-0.050365
C	-1.858306	-0.188901	-0.462694
C	-1.404036	-1.540482	0.116051
C	0.041124	-1.865281	-0.276366
C	-3.348695	0.149472	-0.163329
C	-4.306227	-0.809934	-0.891060
C	-3.696344	0.220482	1.334045
O	-1.191478	2.152981	-0.769054
C	1.393603	2.731377	0.763767
O	2.950386	-2.379812	-0.110534
H	0.607961	0.510753	-1.489424
C	1.168062	-0.777333	1.708555
H	3.768544	0.004258	0.818019
H	4.386031	-0.244855	-0.814098
H	3.686079	2.166461	-0.340015
H	2.880494	1.443334	-1.734077
H	2.265610	-1.178961	-1.565516
H	-0.974696	1.134860	1.027301
H	-1.774293	-0.257404	-1.558621
H	-2.060044	-2.337889	-0.250813
H	-1.506236	-1.540176	1.207906
H	0.096236	-1.966479	-1.369893
H	0.339355	-2.833182	0.141211
H	-3.539945	1.143956	-0.591252
H	-4.081661	-0.860015	-1.963368
H	-4.251756	-1.828050	-0.488607
H	-5.344231	-0.473084	-0.784921
H	-3.046420	0.910956	1.883918
H	-3.620995	-0.761238	1.816078
H	-4.727193	0.568987	1.468082
H	-2.028486	2.507572	-0.439666
H	0.422722	2.953408	1.194365
H	2.198618	3.426551	0.994051
H	3.281787	-2.328477	0.798281
H	1.895438	-0.045194	2.068288
H	0.214498	-0.536684	2.185750
H	1.466205	-1.766226	2.076469

B3LYP energy = -737.766644706 a.u.

(1*R*,5*S*,6*S*,7*S*,10*R*)-**2**, Conf. L

C	3.481173	0.000614	-0.209369
C	2.971844	1.392672	-0.630111
C	1.606871	1.678823	-0.040495
C	0.582319	0.607612	-0.394322
C	1.051623	-0.783057	0.175800
C	2.432718	-1.086017	-0.468265
C	-0.882979	0.940611	-0.054092
C	-1.856866	-0.190095	-0.465731
C	-1.403650	-1.542056	0.112440
C	0.043884	-1.867021	-0.270586
C	-3.347132	0.148227	-0.164918
C	-4.305443	-0.813742	-0.888248
C	-3.692444	0.222837	1.332881
O	-1.191793	2.151009	-0.775083
C	1.389786	2.742516	0.743707
O	2.887493	-2.363647	0.012082
H	0.609049	0.505648	-1.492050
C	1.163091	-0.770528	1.715232
H	3.743284	0.002889	0.855001
H	4.396878	-0.244526	-0.763985
H	3.689856	2.164478	-0.333647
H	2.897863	1.426132	-1.727557
H	2.268669	-1.159275	-1.555802
H	-0.973212	1.135832	1.023197
H	-1.773672	-0.257939	-1.561839
H	-2.056852	-2.339394	-0.259626
H	-1.512086	-1.544201	1.203432
H	0.105735	-1.974138	-1.363354
H	0.339801	-2.831370	0.155830
H	-3.539651	1.141513	-0.595074
H	-4.250622	-1.830352	-0.482076
H	-5.343333	-0.476489	-0.782164
H	-4.082125	-0.867778	-1.960657
H	-4.723545	0.570384	1.467595
H	-3.042525	0.915519	1.879886
H	-3.614995	-0.757388	1.817550
H	-2.027326	2.506854	-0.443348
H	0.416420	2.970291	1.165419
H	2.194466	3.438434	0.972916
H	3.733873	-2.561500	-0.414200
H	1.517807	-1.741860	2.072702
H	1.854977	-0.004759	2.074339
H	0.196897	-0.574271	2.187980

B3LYP energy   -737.766422713 a.u.

Table S37. Cartesian coordinates and energies of the low-energy conformers calculated at the mPW1PW91/6-311+G(2d,p) SMD (solvent: $CHCl_3$) level.

(2*S*,6*R*,7*S*,8*R*)-**1**, Conf. A

C	-4.049193	-1.457825	-0.434979
C	-3.415881	-0.209487	-0.961078
C	-3.325926	0.977958	-0.357567
C	-4.076075	1.377417	0.876341
C	-1.844905	-1.922834	0.670691
C	-2.956438	-2.513575	-0.153100
C	-0.899039	1.479376	-0.834246
C	-0.389150	1.021436	0.553243
C	0.185374	-0.411041	0.581926
C	-0.799225	-1.393080	0.035283
O	-0.015118	2.542447	-1.261187
C	0.835489	2.920220	-0.294944
C	0.623917	2.070133	0.900464
O	1.620287	3.818437	-0.451078
C	1.271134	2.281022	2.037671
C	-2.334958	2.005454	-0.867702
C	-2.088957	-1.810181	2.142024
O	-4.939403	-2.060313	-1.372850
O	1.363758	-0.407670	-0.245707
C	2.398885	-1.172145	0.139296
O	2.403637	-1.819920	1.155828
C	3.504745	-1.136576	-0.876802
C	4.895568	-1.387789	-0.300805
C	5.896310	-1.604759	-1.429039
C	5.334124	-0.244012	0.605794
H	-4.587091	-1.254176	0.494880
H	-2.845084	-0.361777	-1.875594
H	-4.796104	0.631705	1.209317
H	-4.621965	2.307407	0.686476
H	-2.573028	-2.848389	-1.119887
H	-3.424429	-3.370456	0.338529
H	-3.395227	1.593166	1.705752
H	-1.210178	1.026369	1.270377
H	0.484091	-0.642165	1.603454
H	-0.728925	-1.537137	-1.038976
H	1.111354	1.666669	2.916008
H	1.989164	3.089830	2.110307
H	-2.387160	2.911084	-0.257797
H	-0.781860	0.691177	-1.575261
H	-2.562220	2.303441	-1.894551
H	-2.946857	-1.163542	2.353007
H	-1.229755	-1.421987	2.688190
H	-2.334801	-2.792208	2.556758
H	-5.614546	-1.411895	-1.596913
H	3.254767	-1.913711	-1.608712
H	3.464768	-0.185625	-1.412671
H	4.845460	-2.302117	0.298132
H	6.894492	-1.802470	-1.031445
H	5.614363	-2.452140	-2.059278
H	5.965460	-0.720690	-2.069996
H	6.318656	-0.442910	1.035706
H	4.637450	-0.093639	1.433244
H	5.399125	0.694077	0.046082

mPW1PW91 energy = -1155.17572935 a.u.

(2*S*,6*R*,7*S*,8*R*)-**1**, Conf. B

C	-3.893497	-1.705609	-0.461938
C	-3.367745	-0.410182	-0.992338
C	-3.375269	0.781367	-0.390010
C	-4.155726	1.119242	0.843291
C	-1.672485	-1.961382	0.679990
C	-2.713356	-2.655569	-0.155272
C	-0.997698	1.490258	-0.848636
C	-0.462356	1.100823	0.549792
C	0.220074	-0.284000	0.618830
C	-0.665359	-1.346960	0.058374
O	-0.210407	2.622749	-1.286317
C	0.601179	3.084849	-0.323423
C	0.467243	2.229647	0.879651
O	1.302057	4.048737	-0.487650
C	1.116344	2.492329	2.004997
C	-2.473972	1.886345	-0.902844
C	-1.953828	-1.859815	2.145464
O	-4.711063	-2.394409	-1.406142
O	1.424669	-0.221447	-0.168834
C	2.580473	-0.585332	0.412032
O	2.658604	-1.003113	1.540004
C	3.743291	-0.356834	-0.511375
C	4.948168	-1.250064	-0.230791
C	4.646911	-2.704928	-0.569994
C	6.165414	-0.748003	-0.997015
H	-4.462033	-1.543626	0.458234
H	-2.786856	-0.517586	-1.907201
H	-4.814300	0.317754	1.173795
H	-4.774071	2.002238	0.651532
H	-2.287295	-2.962260	-1.113436
H	-3.109405	-3.547191	0.337637
H	-3.496528	1.388830	1.674595
H	-1.287642	1.058123	1.261286
H	0.499496	-0.477349	1.652943
H	-0.561703	-1.492339	-1.012880
H	1.021222	1.869377	2.886436
H	1.769863	3.355166	2.064329
H	-2.612390	2.790614	-0.304537
H	-0.804254	0.703587	-1.574924
H	-2.715271	2.150177	-1.935846
H	-2.095282	-2.858475	2.569701
H	-2.883301	-1.312379	2.330877
H	-1.156158	-1.369326	2.702575
H	-5.446206	-1.817186	-1.636118
H	3.403662	-0.460378	-1.544640
H	4.014768	0.697716	-0.383399
H	5.162844	-1.185072	0.840007

H	5.497825	-3.346775	-0.329630
H	3.784417	-3.078694	-0.013850
H	4.435438	-2.820759	-1.637508
H	7.039448	-1.370340	-0.791318
H	6.415544	0.280005	-0.722993
H	5.990443	-0.771079	-2.076877

mPW1PW91 energy = -1155.17567708 a.u.

(2*S*,6*R*,7*S*,8*R*)-**1**, Conf. C

C	-4.152907	-1.293114	-0.447172
C	-3.453954	-0.076188	-0.962402
C	-3.278400	1.090632	-0.337800
C	-3.977833	1.509531	0.919047
C	-1.968528	-1.915932	0.625547
C	-3.122528	-2.417947	-0.199509
C	-0.831208	1.462518	-0.825322
C	-0.346509	0.948068	0.551323
C	0.140941	-0.516444	0.552081
C	-0.904385	-1.423111	-0.009383
O	0.108846	2.485336	-1.231084
C	0.982740	2.791842	-0.260501
C	0.727396	1.927897	0.915893
O	1.816641	3.647600	-0.399965
C	1.390920	2.074015	2.053780
C	-2.236812	2.066173	-0.848019
C	-2.185880	-1.838870	2.103260
O	-5.089952	-1.825084	-1.382179
O	1.311911	-0.584396	-0.283696
C	2.388303	-1.238564	0.183616
O	2.443878	-1.734563	1.280950
C	3.473696	-1.303509	-0.852894
C	4.887890	-1.327830	-0.278231
C	5.890446	-1.656126	-1.377332
C	5.232566	-0.006859	0.398783
H	-4.665855	-1.073641	0.493272
H	-2.915005	-0.244286	-1.893496
H	-3.268497	1.687430	1.733290
H	-4.720946	0.792314	1.263574
H	-2.769665	-2.753407	-1.177677
H	-3.636916	-3.255880	0.278154
H	-4.487014	2.464466	0.751451
H	-1.164497	0.983702	1.270951
H	0.428967	-0.781940	1.567879
H	-0.857416	-1.535976	-1.088611
H	1.200200	1.448349	2.917767
H	2.155047	2.837887	2.140947
H	-2.236679	2.969686	-0.233089
H	-0.754994	0.684899	-1.582830
H	-2.453137	2.380923	-1.872103
H	-3.004533	-1.154297	2.347859
H	-1.299861	-1.515898	2.648995
H	-2.479999	-2.819244	2.489544
H	-5.726046	-1.132889	-1.589032
H	3.282649	-2.224203	-1.417092
H	3.348330	-0.477286	-1.555937
H	4.925911	-2.120738	0.474754

H	6.907444	-1.691906	-0.980034
H	5.678537	-2.623995	-1.838797
H	5.870935	-0.898266	-2.166204
H	6.233712	-0.042492	0.834790
H	4.531000	0.231134	1.201374
H	5.212025	0.817313	-0.320489

mPW1PW91 energy = -1155.17561540 a.u.

(2*S*,6*R*,7*S*,8*R*)-**1**, Conf. D

C	-3.708836	-1.611708	-0.473086
C	-3.052314	-0.435648	-1.121592
C	-3.093207	0.840443	-0.731916
C	-4.030898	1.399763	0.294084
C	-1.676535	-1.734815	0.995335
C	-2.630098	-2.529722	0.145624
C	-0.642838	1.404823	-0.952743
C	-0.322277	1.232590	0.551139
C	0.285242	-0.136990	0.926323
C	-0.567898	-1.257237	0.428552
O	0.250479	2.431846	-1.444140
C	0.939253	3.026052	-0.458077
C	0.600708	2.379431	0.831648
O	1.698595	3.935241	-0.667983
C	1.093539	2.807314	1.985156
C	-2.077542	1.823685	-1.278398
C	-2.142651	-1.387292	2.373166
O	-4.423511	-2.424250	-1.402553
O	1.579768	-0.216488	0.300617
C	2.612376	-0.654937	1.038402
O	2.511649	-0.987751	2.193607
C	3.890343	-0.651457	0.249833
C	3.851791	-1.494073	-1.031114
C	5.164501	-1.340126	-1.788504
C	3.561803	-2.958917	-0.727559
H	-4.388129	-1.285220	0.319035
H	-2.350788	-0.708121	-1.908397
H	-4.581902	2.241299	-0.138566
H	-3.489927	1.805823	1.154493
H	-2.095933	-3.002486	-0.681872
H	-3.132684	-3.315654	0.715202
H	-4.759482	0.677089	0.658057
H	-1.237348	1.321397	1.136763
H	0.426604	-0.167519	2.005495
H	-0.332038	-1.577802	-0.581923
H	0.846178	2.336143	2.929176
H	1.770982	3.653316	2.004538
H	-2.252581	2.816526	-0.856247
H	-0.388884	0.507303	-1.513047
H	-2.162979	1.922356	-2.363670
H	-3.067096	-0.802074	2.341314
H	-1.405455	-0.825790	2.945976
H	-2.376672	-2.299828	2.929843
H	-5.103502	-1.877691	-1.809349
H	4.107830	0.389650	-0.007428
H	4.679639	-1.004181	0.915033
H	3.046407	-1.104865	-1.661001

H	5.144563	-1.906078	-2.722905
H	5.364872	-0.294634	-2.035502
H	6.005843	-1.710740	-1.195415
H	3.518757	-3.544835	-1.648621
H	2.607845	-3.089538	-0.210467
H	4.343272	-3.389698	-0.094537

mPW1PW91 energy = -1155.17532997 a.u.

(2*S*,6*R*,7*S*,8*R*)-**1**, Conf. E

C	-4.151926	-0.969664	-0.534670
C	-3.318892	0.178817	-1.006498
C	-3.066190	1.321870	-0.364451
C	-3.788643	1.802854	0.856583
C	-2.073679	-1.803522	0.604561
C	-3.242279	-2.188861	-0.261010
C	-0.575149	1.454689	-0.752104
C	-0.192951	0.878236	0.631243
C	0.166818	-0.621964	0.617211
C	-0.942269	-1.421507	0.012349
O	0.475735	2.383705	-1.108282
C	1.328986	2.602956	-0.096238
C	0.940196	1.760449	1.059538
O	2.241868	3.380839	-0.188184
C	1.542589	1.855132	2.236135
C	-1.911923	2.195781	-0.813019
C	-2.332110	-1.711059	2.074543
O	-5.097752	-1.402383	-1.511173
O	1.356616	-0.759134	-0.181798
C	2.163616	-1.797434	0.090547
O	1.946249	-2.586504	0.976657
C	3.342720	-1.838956	-0.837170
C	4.316862	-0.663968	-0.663526
C	4.875066	-0.599017	0.752448
C	5.438957	-0.779059	-1.687369
H	-4.678490	-0.709899	0.387956
H	-2.751119	-0.036977	-1.910463
H	-3.115366	1.886449	1.715556
H	-4.630219	1.173101	1.140811
H	-2.891324	-2.552921	-1.229556
H	-3.849060	-2.975602	0.194418
H	-4.173089	2.812415	0.677366
H	-1.033969	0.968472	1.318570
H	0.397920	-0.932526	1.636039
H	-0.865667	-1.535486	-1.065143
H	1.247301	1.250259	3.085669
H	2.360740	2.552939	2.373039
H	-1.845956	3.082904	-0.178092
H	-0.550446	0.681981	-1.517780
H	-2.055710	2.550793	-1.836846
H	-3.101766	-0.965009	2.296715
H	-1.441026	-1.460280	2.649045
H	-2.713501	-2.666632	2.446906
H	-5.668597	-0.655975	-1.719939
H	3.859032	-2.783432	-0.659908
H	2.962912	-1.843507	-1.862464
H	3.766311	0.260671	-0.861367

H	5.562377	0.243602	0.856820
H	4.087630	-0.473008	1.499409
H	5.425119	-1.511843	0.999890
H	6.133341	0.059778	-1.600824
H	5.051344	-0.787100	-2.709003
H	6.010752	-1.699997	-1.539201

mPW1PW91 energy = -1155.17503993 a.u.

(2*S*,6*R*,7*S*,8*R*)-**1**, Conf. F

C	-4.056599	-1.456405	-0.431163
C	-3.421255	-0.208370	-0.959273
C	-3.327680	0.979914	-0.359979
C	-4.080027	1.383092	0.870845
C	-1.842091	-1.920081	0.671976
C	-2.954046	-2.512178	-0.149641
C	-0.899768	1.478507	-0.837544
C	-0.390655	1.024417	0.551274
C	0.187179	-0.406708	0.583426
C	-0.793988	-1.392921	0.038049
O	-0.014555	2.539338	-1.267532
C	0.834056	2.921117	-0.300973
C	0.619993	2.075754	0.897363
O	1.619394	3.818450	-0.459260
C	1.263630	2.291849	2.035617
C	-2.335285	2.005016	-0.872506
C	-2.092457	-1.799239	2.141650
O	-5.044811	-1.998497	-1.300986
O	1.366600	-0.402349	-0.242887
C	2.398982	-1.171742	0.140088
O	2.399787	-1.824796	1.153191
C	3.506877	-1.134423	-0.873760
C	4.895949	-1.391476	-0.296136
C	5.898445	-1.605875	-1.423290
C	5.335427	-0.252738	0.616347
H	-4.590838	-1.251773	0.495493
H	-2.842085	-0.360105	-1.870067
H	-3.401085	1.609194	1.699134
H	-4.632201	2.307668	0.673205
H	-2.562896	-2.849141	-1.114559
H	-3.420819	-3.369823	0.341478
H	-4.797399	0.636124	1.206312
H	-1.212773	1.028950	1.267156
H	0.485273	-0.634920	1.605826
H	-0.718860	-1.541872	-1.035355
H	1.101694	1.680928	2.915960
H	1.980494	3.101769	2.107297
H	-2.386844	2.911932	-0.264528
H	-0.782464	0.687975	-1.576129
H	-2.562832	2.301570	-1.899713
H	-1.235107	-1.408923	2.689063
H	-2.341203	-2.778643	2.560757
H	-2.950373	-1.150402	2.345394
H	-4.632663	-2.175927	-2.153555
H	3.256674	-1.908017	-1.609380
H	3.470242	-0.181140	-1.405700
H	4.842437	-2.308269	0.298722

H	6.895143	-1.808477	-1.024479
H	5.615471	-2.449356	-2.058306
H	5.971640	-0.718837	-2.059661
H	6.318793	-0.455802	1.046930
H	4.637721	-0.104607	1.443316
H	5.403410	0.687682	0.060948

mPW1PW91 energy = -1155.17465551 a.u.

(2*S*,6*R*,7*S*,8*R*)-**1**, Conf. G

C	-3.906026	-1.699617	-0.460046
C	-3.375614	-0.404028	-0.988869
C	-3.377411	0.787682	-0.388955
C	-4.158519	1.129366	0.842320
C	-1.675162	-1.957986	0.679838
C	-2.717879	-2.650621	-0.153866
C	-0.997606	1.488333	-0.850532
C	-0.462498	1.101187	0.548382
C	0.220222	-0.283460	0.619867
C	-0.663708	-1.348213	0.060279
O	-0.206069	2.616170	-1.292584
C	0.605545	3.080084	-0.330483
C	0.467701	2.230139	0.875912
O	1.309454	4.041173	-0.497939
C	1.114822	2.496392	2.001549
C	-2.472452	1.889102	-0.903535
C	-1.963792	-1.848431	2.143367
O	-4.825288	-2.334107	-1.342599
O	1.425520	-0.221856	-0.166901
C	2.580650	-0.586350	0.415246
O	2.657150	-1.004616	1.543089
C	3.744679	-0.358111	-0.506670
C	4.948656	-1.252218	-0.225127
C	4.646813	-2.706902	-0.564616
C	6.166882	-0.750942	-0.990290
H	-4.473312	-1.537815	0.455805
H	-2.787199	-0.509253	-1.900822
H	-4.782547	2.006814	0.643818
H	-3.500323	1.408748	1.671249
H	-2.285155	-2.957327	-1.111082
H	-3.113851	-3.543224	0.337037
H	-4.814161	0.327291	1.176754
H	-1.288153	1.059799	1.259539
H	0.498832	-0.475007	1.654552
H	-0.554832	-1.498045	-1.009981
H	1.016623	1.877340	2.885393
H	1.769492	3.358502	2.058787
H	-2.607187	2.793978	-0.305342
H	-0.807399	0.698739	-1.574615
H	-2.714783	2.153957	-1.936035
H	-2.111023	-2.844439	2.571768
H	-2.892083	-1.296642	2.321319
H	-1.167157	-1.358059	2.701961
H	-4.376964	-2.496940	-2.179684
H	3.406265	-0.460844	-1.540434
H	4.016670	0.696200	-0.377877
H	5.162469	-1.187460	0.845842

H	5.497265	-3.349165	-0.323784
H	3.783903	-3.080281	-0.008836
H	4.436013	-2.822601	-1.632304
H	7.040415	-1.373620	-0.783549
H	6.417230	0.277003	-0.716292
H	5.992991	-0.774192	-2.070332

mPW1PW91 energy = -1155.17463629 a.u.

(2*S*,6*R*,7*S*,8*R*)-**1**, Conf. H

C	-3.755989	-1.846235	-0.450324
C	-3.265373	-0.558531	-1.033279
C	-3.349597	0.663421	-0.504466
C	-4.193815	1.032541	0.676276
C	-1.570021	-1.919997	0.800338
C	-2.540119	-2.714861	-0.030191
C	-0.991011	1.460717	-0.899173
C	-0.507730	1.175195	0.542182
C	0.248851	-0.159528	0.709997
C	-0.560965	-1.301061	0.186510
O	-0.233163	2.602733	-1.363590
C	0.497463	3.162748	-0.387550
C	0.333827	2.374862	0.857130
O	1.157537	4.151396	-0.571610
C	0.877173	2.752113	2.005594
C	-2.479464	1.779219	-1.046664
C	-1.929114	-1.733550	2.240111
O	-4.598203	-2.579069	-1.333579
O	1.466954	-0.060413	-0.052211
C	2.595898	-0.531563	0.502076
O	2.638890	-1.041940	1.593344
C	3.779820	-0.295046	-0.392245
C	4.892287	-1.329255	-0.239265
C	4.443903	-2.701214	-0.728272
C	6.142400	-0.865947	-0.976206
H	-4.376172	-1.660047	0.425441
H	-2.638129	-0.685211	-1.916252
H	-3.582973	1.402795	1.505863
H	-4.811820	0.214465	1.041815
H	-2.050106	-3.061388	-0.945198
H	-2.916726	-3.592392	0.501288
H	-4.860870	1.856566	0.402171
H	-1.361621	1.114819	1.217192
H	0.510815	-0.278727	1.760103
H	-0.396005	-1.510937	-0.866539
H	0.750989	2.183585	2.919521
H	1.472187	3.657033	2.051597
H	-2.689305	2.710325	-0.514123
H	-0.724504	0.644906	-1.568106
H	-2.683081	1.966320	-2.104254
H	-2.048220	-2.706962	2.725391
H	-2.889501	-1.218803	2.343306
H	-1.181908	-1.170321	2.798300
H	-4.106070	-2.758168	-2.142206
H	3.439037	-0.231407	-1.428041
H	4.153702	0.701255	-0.128954
H	5.126643	-1.405917	0.826599

H	5.234451	-3.442295	-0.588748
H	3.562784	-3.054307	-0.187959
H	4.199052	-2.675696	-1.794629
H	6.953409	-1.588827	-0.860809
H	6.496935	0.096838	-0.599542
H	5.950331	-0.753313	-2.047544

mPW1PW91 energy = -1155.17462432 a.u.

(2*S*,6*R*,7*S*,8*R*)-**1**, Conf. I

C	-4.177104	-1.265119	-0.439349
C	-3.468361	-0.052875	-0.956000
C	-3.274081	1.109470	-0.330601
C	-3.962875	1.530750	0.930797
C	-1.982691	-1.917475	0.609894
C	-3.146040	-2.402443	-0.211874
C	-0.825165	1.460373	-0.827288
C	-0.342671	0.936973	0.546398
C	0.135027	-0.530956	0.541302
C	-0.915824	-1.428570	-0.023957
O	0.123237	2.476275	-1.231362
C	1.000182	2.772943	-0.260359
C	0.738232	1.907963	0.913809
O	1.841161	3.622002	-0.398082
C	1.402497	2.046113	2.052244
C	-2.225699	2.075613	-0.844802
C	-2.197940	-1.845677	2.088266
O	-5.221958	-1.714717	-1.295819
O	1.306436	-0.606310	-0.293462
C	2.389240	-1.240268	0.187285
O	2.448630	-1.716037	1.293270
C	3.476578	-1.312926	-0.846781
C	4.889910	-1.320738	-0.269563
C	5.896336	-1.663066	-1.360748
C	5.227002	0.013169	0.385577
H	-4.673654	-1.045605	0.504819
H	-2.930446	-0.221532	-1.889099
H	-3.248234	1.703975	1.741550
H	-4.709950	0.818300	1.276344
H	-2.795162	-2.731670	-1.194879
H	-3.663447	-3.242106	0.259044
H	-4.468390	2.488044	0.765992
H	-1.160064	0.974903	1.266543
H	0.420147	-0.801885	1.556386
H	-0.870673	-1.535618	-1.103940
H	1.206683	1.419678	2.914517
H	2.172157	2.804134	2.141810
H	-2.215494	2.978765	-0.229542
H	-0.756041	0.684762	-1.587607
H	-2.443860	2.393129	-1.867622
H	-2.497634	-2.825868	2.470666
H	-3.012118	-1.157252	2.336604
H	-1.309515	-1.530216	2.634342
H	-4.845096	-1.893200	-2.164416
H	3.291897	-2.242416	-1.398600
H	3.347129	-0.497280	-1.561378
H	4.930104	-2.100636	0.496790

H	6.912518	-1.687408	-0.960550
H	5.689895	-2.639613	-1.806156
H	5.875502	-0.918804	-2.162420
H	6.227542	-0.010291	0.823737
H	4.522854	0.261458	1.182743
H	5.203978	0.825011	-0.347538

mPW1PW91 energy = -1155.17454429 a.u.

(2*S*,6*R*,7*S*,8*R*)-**1**, Conf. J

C	-4.063892	-1.440309	-0.440590
C	-3.432043	-0.195467	-0.968310
C	-3.324399	0.984008	-0.354718
C	-4.064097	1.381804	0.885753
C	-1.848100	-1.912694	0.666346
C	-2.959320	-2.496228	-0.161856
C	-0.896773	1.482697	-0.834544
C	-0.384547	1.026607	0.552640
C	0.186831	-0.406824	0.581770
C	-0.799722	-1.385468	0.033045
O	-0.012066	2.544303	-1.264601
C	0.841214	2.921939	-0.301047
C	0.630993	2.073981	0.896225
O	1.627505	3.818463	-0.459945
C	1.280666	2.285716	2.031856
C	-2.332097	2.009213	-0.867063
C	-2.095271	-1.800445	2.137233
O	-4.981220	-1.937146	-1.410541
O	1.366170	-0.406265	-0.244513
C	2.396727	-1.177599	0.138497
O	2.396212	-1.830916	1.151596
C	3.504968	-1.142078	-0.875036
C	4.893664	-1.399430	-0.296592
C	5.896674	-1.614515	-1.423156
C	5.332966	-0.260411	0.615636
H	-4.596997	-1.233501	0.491023
H	-2.872823	-0.345187	-1.890093
H	-3.378360	1.590910	1.712845
H	-4.606583	2.314864	0.701530
H	-2.575640	-2.823544	-1.131070
H	-3.421283	-3.359636	0.328657
H	-4.787612	0.638972	1.217267
H	-1.203673	1.034044	1.271859
H	0.483674	-0.639529	1.603505
H	-0.728845	-1.528089	-1.041390
H	1.121598	1.673074	2.911532
H	1.999984	3.093614	2.101844
H	-2.382948	2.916098	-0.258945
H	-0.781498	0.693307	-1.574613
H	-2.560801	2.304863	-1.894164
H	-1.234312	-1.419460	2.685667
H	-2.349015	-2.781018	2.550833
H	-2.948177	-1.146955	2.347203
H	-5.328753	-2.777600	-1.093850
H	3.254389	-1.916059	-1.610087
H	3.469183	-0.189257	-1.407825
H	4.839584	-2.315995	0.298577

H	6.893144	-1.817264	-1.023812
H	5.613675	-2.458064	-2.058046
H	5.970354	-0.727723	-2.059799
H	6.315997	-0.463487	1.047021
H	4.634710	-0.111567	1.442034
H	5.401568	0.679696	0.059785

mPW1PW91 energy = -1155.17445747 a.u.

(2*S*,6*R*,7*S*,8*R*)-**1**, Conf. K

C	-3.754735	-1.841903	-0.458388
C	-3.271926	-0.556075	-1.041334
C	-3.349180	0.660539	-0.499678
C	-4.186053	1.024961	0.687987
C	-1.566277	-1.915919	0.794747
C	-2.532696	-2.706786	-0.043255
C	-0.992972	1.466549	-0.897351
C	-0.504748	1.181235	0.542544
C	0.251024	-0.153853	0.708771
C	-0.559862	-1.292432	0.181468
O	-0.240912	2.612744	-1.361730
C	0.492592	3.172296	-0.388049
C	0.337458	2.380870	0.855539
O	1.148949	4.163480	-0.572335
C	0.887562	2.755324	2.001685
C	-2.482711	1.779659	-1.040982
C	-1.921995	-1.739387	2.236592
O	-4.528957	-2.528003	-1.437924
O	1.470138	-0.054975	-0.051736
C	2.596580	-0.533005	0.501214
O	2.636960	-1.047121	1.590972
C	3.781772	-0.299243	-0.392137
C	4.890595	-1.337495	-0.240235
C	4.438019	-2.706907	-0.732441
C	6.142829	-0.876904	-0.975311
H	-4.371847	-1.655370	0.424411
H	-2.660347	-0.682278	-1.933239
H	-3.570199	1.379756	1.520610
H	-4.811057	0.208791	1.045912
H	-2.048531	-3.042747	-0.963279
H	-2.899357	-3.590837	0.488582
H	-4.845278	1.858582	0.424242
H	-1.356557	1.121256	1.220221
H	0.511464	-0.275766	1.758937
H	-0.399417	-1.496059	-0.873355
H	0.767839	2.183849	2.914657
H	1.481935	3.660715	2.046697
H	-2.694644	2.709362	-0.506755
H	-0.725734	0.653001	-1.568678
H	-2.688805	1.966742	-2.098037
H	-2.041006	-2.715876	2.715927
H	-2.881033	-1.223295	2.345614
H	-1.172379	-1.181382	2.796870
H	-4.759192	-3.394317	-1.085693
H	3.441511	-0.233062	-1.427935
H	4.158964	0.695429	-0.127415
H	5.123873	-1.417049	0.825673

H	5.225851	-3.450988	-0.593389
H	3.554912	-3.057901	-0.194023
H	4.194448	-2.678450	-1.798987
H	6.951019	-1.603239	-0.861703
H	6.500907	0.083448	-0.595769
H	5.951589	-0.760456	-2.046376

mPW1PW91 energy = -1155.17441707 a.u.

(2*S*,6*R*,7*S*,8*R*)-**1**, Conf. L

C	-4.181153	-1.245225	-0.450335
C	-3.472097	-0.038571	-0.967408
C	-3.265419	1.115967	-0.332126
C	-3.944321	1.536578	0.935108
C	-1.989832	-1.908072	0.608762
C	-3.151520	-2.385103	-0.219555
C	-0.815409	1.462462	-0.826225
C	-0.336588	0.937679	0.548293
C	0.134798	-0.531984	0.542592
C	-0.920980	-1.422705	-0.024319
O	0.135570	2.477549	-1.227254
C	1.012134	2.770644	-0.255304
C	0.747106	1.904744	0.917609
O	1.855344	3.618018	-0.390560
C	1.410724	2.039614	2.056794
C	-2.214987	2.079259	-0.847215
C	-2.204597	-1.840138	2.087388
O	-5.153716	-1.650737	-1.409036
O	1.305579	-0.610412	-0.292589
C	2.382622	-1.258202	0.181771
O	2.437227	-1.746824	1.282553
C	3.471343	-1.326666	-0.851155
C	4.883981	-1.325572	-0.271576
C	5.894614	-1.654795	-1.362938
C	5.209132	0.008372	0.389550
H	-4.677820	-1.020414	0.497230
H	-2.951541	-0.206466	-1.908718
H	-3.225149	1.697627	1.744423
H	-4.697182	0.829593	1.279413
H	-2.805568	-2.710228	-1.203680
H	-3.667459	-3.227343	0.253350
H	-4.440135	2.500154	0.777516
H	-1.154747	0.978249	1.267352
H	0.418826	-0.805827	1.557261
H	-0.878177	-1.526309	-1.104611
H	1.212410	1.412512	2.918036
H	2.182458	2.795317	2.148076
H	-2.204522	2.984429	-0.234874
H	-0.746012	0.687320	-1.586940
H	-2.431576	2.392669	-1.871541
H	-2.511369	-2.819400	2.466888
H	-3.012918	-1.145698	2.338226
H	-1.313573	-1.533791	2.634496
H	-5.562055	-2.465571	-1.097980
H	3.293081	-2.258075	-1.401705
H	3.338856	-0.512521	-1.566809
H	4.929562	-2.108297	0.491613

H	6.910379	-1.673213	-0.961332
H	5.696462	-2.630600	-1.813684
H	5.869122	-0.906657	-2.160843
H	6.209842	-0.008021	0.827644
H	4.502787	0.246748	1.187830
H	5.178686	0.823331	-0.339801

mPW1PW91energy = -1155.17435292 a.u.

(2*S*,6*R*,7*S*,8*R*)-**1**, Conf. M

C	-3.719231	-1.605504	-0.473093
C	-3.056123	-0.432175	-1.122588
C	-3.092957	0.844795	-0.738480
C	-4.035181	1.409050	0.280002
C	-1.678964	-1.731224	0.996334
C	-2.633804	-2.525521	0.148001
C	-0.639915	1.401909	-0.955424
C	-0.323372	1.232482	0.549441
C	0.284692	-0.135768	0.928649
C	-0.566235	-1.258629	0.432938
O	0.257712	2.424959	-1.447295
C	0.943045	3.021985	-0.460418
C	0.598605	2.380069	0.830141
O	1.704529	3.929304	-0.670480
C	1.087451	2.811031	1.984195
C	-2.072760	1.823761	-1.284497
C	-2.152539	-1.373284	2.369014
O	-4.545160	-2.351171	-1.361117
O	1.580159	-0.215382	0.304824
C	2.610645	-0.658653	1.042892
O	2.506778	-0.996655	2.196251
C	3.890325	-0.653392	0.257059
C	3.853899	-1.492615	-1.026179
C	5.168516	-1.338127	-1.780127
C	3.561792	-2.957970	-0.727085
H	-4.393947	-1.275648	0.315718
H	-2.345245	-0.704852	-1.902502
H	-3.498481	1.824596	1.138643
H	-4.588288	2.244064	-0.162361
H	-2.091954	-3.000287	-0.675800
H	-3.137319	-3.311405	0.716599
H	-4.763897	0.687055	0.644764
H	-1.240355	1.321580	1.132058
H	0.424411	-0.163540	2.008144
H	-0.324839	-1.584714	-0.574603
H	0.835968	2.343054	2.928710
H	1.765307	3.656704	2.003641
H	-2.245807	2.817577	-0.863930
H	-0.386721	0.502365	-1.512961
H	-2.156601	1.922005	-2.369931
H	-1.417101	-0.809931	2.942145
H	-2.392675	-2.281248	2.930469
H	-3.074849	-0.785448	2.327514
H	-4.001851	-2.656010	-2.096018
H	4.108758	0.388258	0.002986
H	4.678180	-1.008417	0.922725
H	3.050432	-1.100730	-1.656906

H	5.150410	-1.902195	-2.715714
H	5.370270	-0.292286	-2.024423
H	6.008091	-1.710617	-1.185760
H	3.520771	-3.541569	-1.649753
H	2.606590	-3.089077	-0.212348
H	4.341282	-3.391038	-0.093225

mPW1PW91 energy = -1155.17429010 a.u.

(2*S*,6*R*,7*S*,8*R*)-**1**, Conf. N

C	-3.720064	-1.594177	-0.484368
C	-3.060236	-0.424828	-1.134654
C	-3.088634	0.847875	-0.736697
C	-4.023226	1.410258	0.290147
C	-1.681279	-1.722682	0.993877
C	-2.632708	-2.512886	0.138433
C	-0.635827	1.407623	-0.953828
C	-0.317638	1.238779	0.550831
C	0.286002	-0.131127	0.929848
C	-0.568411	-1.249806	0.431202
O	0.260512	2.431918	-1.446251
C	0.949686	3.026390	-0.460954
C	0.607872	2.383771	0.830015
O	1.712133	3.932851	-0.671752
C	1.100205	2.812811	1.983276
C	-2.069006	1.827577	-1.282479
C	-2.153677	-1.370605	2.368447
O	-4.461280	-2.310533	-1.467614
O	1.581664	-0.214192	0.306964
C	2.608586	-0.667801	1.043262
O	2.501735	-1.011020	2.194933
C	3.888562	-0.667208	0.258018
C	3.847453	-1.502637	-1.027649
C	5.163608	-1.354786	-1.780280
C	3.545192	-2.966884	-0.733334
H	-4.395514	-1.260048	0.307469
H	-2.363982	-0.699407	-1.925068
H	-4.759410	0.691720	0.646667
H	-4.566812	2.256991	-0.141541
H	-2.096087	-2.982057	-0.689492
H	-3.131759	-3.302432	0.710011
H	-3.481943	1.808884	1.153910
H	-1.233326	1.331319	1.134863
H	0.424823	-0.160549	2.009442
H	-0.329804	-1.572502	-0.577934
H	0.850024	2.344443	2.927969
H	1.779873	3.657041	2.001921
H	-2.243107	2.821608	-0.862779
H	-0.382701	0.508417	-1.511815
H	-2.152988	1.923810	-2.368010
H	-1.416171	-0.812761	2.944406
H	-2.396141	-2.280572	2.925847
H	-3.073890	-0.779195	2.330303
H	-4.827550	-3.098801	-1.052939
H	4.112827	0.373850	0.006683
H	4.674158	-1.028585	0.922934
H	3.047151	-1.103647	-1.657864

H	5.142285	-1.916349	-2.717294
H	5.372197	-0.309673	-2.021966
H	6.000426	-1.734235	-1.186387
H	3.500765	-3.547009	-1.657993
H	2.588574	-3.093122	-0.220065
H	4.321205	-3.407289	-0.100208

mPW1PW91 energy = -1155.17410107 a.u.

(2*S*,6*R*,7*S*,8*R*)-**1**, Conf. O

C	-4.162491	-0.959616	-0.530752
C	-3.323473	0.185191	-1.004754
C	-3.065298	1.328899	-0.367959
C	-3.788707	1.816879	0.849070
C	-2.076322	-1.799763	0.606918
C	-3.247850	-2.182913	-0.255126
C	-0.573364	1.451329	-0.755381
C	-0.193315	0.877704	0.629539
C	0.166350	-0.622639	0.618152
C	-0.942397	-1.424087	0.015032
O	0.481397	2.374856	-1.114546
C	1.333061	2.596769	-0.101621
C	0.940086	1.759989	1.056934
O	2.247818	3.372226	-0.195140
C	1.539401	1.859044	2.234758
C	-1.907272	2.196855	-0.818614
C	-2.337660	-1.697352	2.075760
O	-5.196154	-1.318259	-1.441910
O	1.356200	-0.760986	-0.180701
C	2.162760	-1.799543	0.092133
O	1.943670	-2.589588	0.976927
C	3.343531	-1.840243	-0.833578
C	4.317038	-0.665014	-0.657941
C	4.872982	-0.600483	0.758917
C	5.440713	-0.779290	-1.680125
H	-4.686879	-0.696521	0.386988
H	-2.747221	-0.033936	-1.904016
H	-3.114636	1.912989	1.706163
H	-4.627690	1.186109	1.138034
H	-2.892818	-2.552278	-1.222213
H	-3.856215	-2.967814	0.201015
H	-4.179901	2.821954	0.659706
H	-1.035095	0.969731	1.315725
H	0.397509	-0.931471	1.637499
H	-0.862896	-1.544171	-1.061694
H	1.240647	1.258621	3.086232
H	2.358124	2.556321	2.370915
H	-1.838205	3.085151	-0.185735
H	-0.551111	0.675835	-1.518431
H	-2.049907	2.550687	-1.843015
H	-2.722897	-2.649330	2.453260
H	-3.105237	-0.947463	2.291777
H	-1.446923	-1.445836	2.650411
H	-4.789789	-1.542268	-2.286324
H	3.859860	-2.784630	-0.655874
H	2.965564	-1.844441	-1.859586
H	3.766538	0.259541	-0.856256

H	5.559821	0.242327	0.864663
H	4.084348	-0.474948	1.504684
H	5.422965	-1.513208	1.006874
H	6.134718	0.059673	-1.591989
H	5.054681	-0.786747	-2.702378
H	6.012546	-1.700152	-1.531644

mPW1PW91 energy = -1155.17403553 a.u.

(2*S*,6*R*,7*S*,8*R*)-**1**, Conf. P

C	-4.164484	-0.950180	-0.537730
C	-3.333070	0.194886	-1.011207
C	-3.064856	1.327833	-0.360075
C	-3.776730	1.805898	0.868005
C	-2.075850	-1.794402	0.598971
C	-3.243367	-2.172322	-0.270442
C	-0.574146	1.456322	-0.754414
C	-0.188939	0.881527	0.628669
C	0.167932	-0.619251	0.616033
C	-0.941934	-1.415887	0.009011
O	0.477860	2.382926	-1.114817
C	1.333514	2.602769	-0.105322
C	0.946334	1.762820	1.053012
O	2.247465	3.379148	-0.200387
C	1.551309	1.859138	2.228139
C	-1.909831	2.198819	-0.812572
C	-2.337416	-1.702005	2.068479
O	-5.122560	-1.269482	-1.542421
O	1.358793	-0.760133	-0.180648
C	2.161678	-1.801165	0.092754
O	1.939669	-2.590370	0.977733
C	3.342731	-1.846067	-0.832291
C	4.319485	-0.673416	-0.657276
C	4.874880	-0.609056	0.759787
C	5.443245	-0.791931	-1.678861
H	-4.683650	-0.689059	0.388419
H	-2.777030	-0.016588	-1.923188
H	-4.620497	1.179106	1.151867
H	-4.158438	2.817366	0.694127
H	-2.891271	-2.527948	-1.241719
H	-3.844410	-2.966883	0.183583
H	-3.098876	1.883366	1.724010
H	-1.028222	0.974033	1.317703
H	0.396226	-0.930167	1.635399
H	-0.863745	-1.529245	-1.068446
H	1.257002	1.256476	3.079586
H	2.370445	2.556390	2.361928
H	-1.840910	3.086887	-0.179296
H	-0.553116	0.682720	-1.519257
H	-2.056605	2.551952	-1.836525
H	-2.722384	-2.656576	2.440023
H	-3.104472	-0.953118	2.289825
H	-1.446500	-1.454807	2.644754
H	-5.601252	-2.057162	-1.263153
H	3.856325	-2.791727	-0.653422
H	2.965113	-1.850090	-1.858397
H	3.771637	0.252443	-0.856787

H	5.564632	0.231471	0.864876
H	4.086270	-0.479709	1.504962
H	5.421502	-1.523386	1.009292
H	6.139376	0.045382	-1.591693
H	5.057440	-0.799753	-2.701175
H	6.012689	-1.714069	-1.529002

mPW1PW91 energy = -1155.17385397 a.u.

H	5.063059	-1.478921	0.686370
H	5.195356	-3.493356	-0.756134
H	3.518613	-3.135958	-0.347028
H	4.145896	-2.725879	-1.949318
H	6.882772	-1.606726	-1.014566
H	6.400799	0.066625	-0.722615
H	5.859527	-0.768044	-2.181681

mPW1PW91 energy = -1155.17539035 a.u.

(2*R*,6*R*,7*S*,8*R*)-**1**, Conf. A

C	-4.011861	-1.585792	-0.307197
C	-3.592098	-0.144955	-0.286979
C	-3.051266	0.560570	-1.279807
C	-2.927484	0.069803	-2.688870
C	-1.690864	-1.940944	0.666153
C	-2.788426	-2.534462	-0.172303
C	-0.880614	1.701801	-0.741222
C	-0.482028	1.152281	0.650500
C	0.176274	-0.240028	0.648778
C	-0.735967	-1.249528	0.040637
O	-0.257944	3.004094	-0.858027
C	0.476316	3.328157	0.215638
C	0.398352	2.232330	1.207498
O	1.075123	4.369235	0.287421
C	1.042416	2.287532	2.364438
C	-2.382484	1.878246	-0.984589
C	-1.863249	-2.004398	2.150030
O	-4.894012	-1.871992	0.775731
O	1.384581	-0.148609	-0.131858
C	2.519525	-0.634957	0.395999
O	2.578990	-1.173285	1.472915
C	3.690729	-0.374107	-0.508695
C	4.821402	-1.390673	-0.376981
C	4.393381	-2.762492	-0.883871
C	6.059552	-0.895349	-1.113405
H	-4.511860	-1.836677	-1.248730
H	-3.613581	0.301780	0.704891
H	-3.359908	0.796804	-3.383298
H	-3.421419	-0.887800	-2.848904
H	-2.410223	-2.723219	-1.178631
H	-3.155127	-3.481898	0.230939
H	-1.877719	-0.044024	-2.979244
H	-1.380806	1.052049	1.263312
H	0.447775	-0.493936	1.671744
H	-0.719012	-1.266985	-1.045813
H	0.993179	1.487469	3.093648
H	1.646940	3.154472	2.605123
H	-2.827232	2.356338	-0.108424
H	-0.442278	1.100391	-1.537988
H	-2.496750	2.561655	-1.830461
H	-2.750030	-1.444684	2.463098
H	-1.001762	-1.621939	2.696763
H	-2.032051	-3.038109	2.464519
H	-5.665582	-1.302491	0.689712
H	3.337577	-0.307075	-1.540286
H	4.050312	0.626393	-0.240975

(2*R*,6*R*,7*S*,8*R*)-**1**, Conf. B

C	-4.282030	-1.159320	-0.281160
C	-3.664940	0.203586	-0.160014
C	-3.078634	0.919806	-1.118946
C	-3.094138	0.549522	-2.569808
C	-1.982510	-1.914930	0.494778
C	-3.198274	-2.272839	-0.313347
C	-0.746675	1.719073	-0.623862
C	-0.350718	0.978233	0.677109
C	0.098163	-0.482846	0.499211
C	-0.981604	-1.293326	-0.131840
O	0.031134	2.941127	-0.648563
C	0.872078	3.043114	0.391194
C	0.708882	1.861329	1.266766
O	1.612805	3.982135	0.521510
C	1.435063	1.694908	2.362922
C	-2.222848	2.105246	-0.752522
C	-2.074482	-2.108055	1.974506
O	-5.137714	-1.429633	0.826777
O	1.262899	-0.477209	-0.350103
C	2.308020	-1.240223	0.009556
O	2.336085	-1.902485	1.016413
C	3.396094	-1.188157	-1.025453
C	4.797449	-1.438451	-0.475091
C	5.780304	-1.644617	-1.620964
C	5.247471	-0.300136	0.432630
H	-4.860821	-1.246924	-1.206664
H	-3.576419	0.554270	0.866292
H	-3.742650	-0.299603	-2.782442
H	-2.090040	0.298704	-2.928023
H	-2.909009	-2.414628	-1.356159
H	-3.670890	-3.195743	0.031779
H	-3.433034	1.396306	-3.174434
H	-1.216536	0.941788	1.342609
H	0.388648	-0.874042	1.472023
H	-1.033402	-1.201894	-1.213334
H	1.322338	0.829248	3.004868
H	2.172633	2.438794	2.641094
H	-2.551885	2.549490	0.190021
H	-0.429075	1.157063	-1.502265
H	-2.290481	2.875768	-1.525383
H	-2.878974	-1.497825	2.396261
H	-1.149020	-1.867847	2.497250
H	-2.331680	-3.146733	2.201578
H	-5.818682	-0.749211	0.850298
H	3.137603	-1.959468	-1.760544
H	3.342661	-0.232060	-1.550893

H	4.759644	-2.357099	0.118012
H	6.785868	-1.839704	-1.241078
H	5.492002	-2.489942	-2.251113
H	5.835296	-0.756813	-2.258259
H	6.237714	-0.501384	0.847966
H	4.561596	-0.155134	1.269976
H	5.304901	0.641637	-0.121714

mPW1PW91 energy = -1155.17535609 a.u.

(2*R*,6*R*,7*S*,8*R*)-**1**, Conf. C

C	4.297685	-1.128489	0.257893
C	3.676677	0.229234	0.149228
C	3.077921	0.927554	1.112184
C	3.090654	0.543045	2.559443
C	1.987389	-1.914644	-0.462450
C	3.209880	-2.245131	0.345149
C	0.743795	1.724700	0.626744
C	0.350581	0.977239	-0.671071
C	-0.094048	-0.484210	-0.485953
C	0.987009	-1.283759	0.156166
O	-0.037005	2.945102	0.645416
C	-0.877541	3.040099	-0.395054
C	-0.710327	1.855134	-1.265823
O	-1.621357	3.976129	-0.529896
C	-1.433867	1.683349	-2.362886
C	2.219107	2.113166	0.753870
C	2.072871	-2.134568	-1.939097
O	5.127198	-1.283081	-0.888933
O	-1.263151	-0.477709	0.357727
C	-2.303011	-1.247388	-0.001935
O	-2.322523	-1.918191	-1.003499
C	-3.398103	-1.191427	1.025511
C	-4.796112	-1.438763	0.464985
C	-5.788432	-1.639544	1.603594
C	-5.235433	-0.300725	-0.448365
H	4.913717	-1.206584	1.160506
H	3.605798	0.593655	-0.872679
H	2.090364	0.267970	2.910601
H	3.410133	1.390535	3.173559
H	2.934538	-2.349119	1.396812
H	3.670036	-3.188247	0.033154
H	3.757001	-0.293522	2.767185
H	1.217270	0.939518	-1.335341
H	-0.377759	-0.883044	-1.457677
H	1.043064	-1.170947	1.235313
H	-1.317665	0.815893	-3.001761
H	-2.172383	2.424746	-2.645144
H	2.545402	2.561815	-0.187480
H	0.426639	1.166185	1.507595
H	2.287382	2.880601	1.529790
H	2.352566	-3.171080	-2.149661
H	2.857194	-1.510973	-2.379158
H	1.136654	-1.928345	-2.457155
H	5.549181	-2.146394	-0.839376
H	-3.147042	-1.962846	1.763059
H	-3.346368	-0.234885	1.550201

H	-4.756559	-2.358691	-0.126030
H	-6.791403	-1.833182	1.216196
H	-5.507166	-2.483928	2.238159
H	-5.846269	-0.749918	2.238053
H	-6.223246	-0.499187	-0.870798
H	-4.542922	-0.159581	-1.280912
H	-5.293612	0.642335	0.103684

mPW1PW91 energy = -1155.17510394 a.u.

(2*R*,6*R*,7*S*,8*R*)-**1**, Conf. D

C	-4.026202	-1.569969	-0.293126
C	-3.609261	-0.132116	-0.287753
C	-3.045203	0.552837	-1.280727
C	-2.899759	0.039797	-2.679946
C	-1.689785	-1.938606	0.646115
C	-2.790072	-2.518629	-0.195845
C	-0.880743	1.707662	-0.748002
C	-0.484699	1.158091	0.644170
C	0.173863	-0.233953	0.642315
C	-0.736192	-1.240191	0.026134
O	-0.264488	3.013552	-0.860236
C	0.468158	3.337190	0.214449
C	0.393460	2.238545	1.203651
O	1.063753	4.379956	0.289337
C	1.037727	2.292643	2.360507
C	-2.382331	1.875752	-0.996839
C	-1.859906	-2.014331	2.129961
O	-4.910268	-1.737017	0.810246
O	1.386112	-0.141323	-0.132311
C	2.516618	-0.635422	0.397444
O	2.569487	-1.179255	1.472026
C	3.692356	-0.376297	-0.501857
C	4.820432	-1.395160	-0.366069
C	4.391516	-2.766293	-0.874088
C	6.062036	-0.902543	-1.098465
H	-4.555704	-1.821949	-1.218567
H	-3.663272	0.332322	0.693854
H	-1.846567	-0.068088	-2.959614
H	-3.333572	0.751055	-3.389774
H	-2.423089	-2.681220	-1.211574
H	-3.137229	-3.484366	0.185789
H	-3.384447	-0.924228	-2.829503
H	-1.385121	1.056710	1.254520
H	0.440007	-0.490962	1.665922
H	-0.721032	-1.246725	-1.060326
H	0.990519	1.490886	3.087990
H	1.639919	3.160669	2.603132
H	-2.831283	2.359844	-0.126205
H	-0.435180	1.109973	-1.543646
H	-2.497156	2.551151	-1.849171
H	-2.044168	-3.048187	2.435766
H	-2.734936	-1.440989	2.451452
H	-0.989878	-1.652618	2.677191
H	-5.218337	-2.648528	0.812019
H	3.343640	-0.307967	-1.534896
H	4.052765	0.623388	-0.232302

H	5.058344	-1.483674	0.698105
H	5.191821	-3.498541	-0.743723
H	3.514440	-3.138020	-0.339830
H	4.147483	-2.729467	-1.940325
H	6.883425	-1.615646	-0.996822
H	6.403992	0.058782	-0.706727
H	5.865757	-0.774972	-2.167400

mPW1PW91 energy = -1155.17508970 a.u.

(2*R*,6*R*,7*S*,8*R*)-**1**, Conf. E

C	3.912115	-1.362710	0.363879
C	3.384750	0.035828	0.498124
C	2.685539	0.551257	1.508700
C	2.453374	-0.144063	2.814103
C	1.753674	-1.698600	-0.936039
C	2.792100	-2.350191	-0.066256
C	0.510134	1.638854	0.876252
C	0.311818	1.281660	-0.617527
C	-0.226448	-0.132690	-0.896590
C	0.687448	-1.166839	-0.334657
O	-0.211784	2.874596	1.097969
C	-0.848136	3.309535	0.001047
C	-0.591307	2.371960	-1.114808
O	-1.508243	4.315439	0.004325
C	-1.136191	2.539900	-2.311238
C	1.957717	1.858031	1.325405
C	2.093999	-1.520964	-2.381360
O	4.938367	-1.426426	-0.623574
O	-1.521543	-0.232557	-0.272626
C	-2.525067	-0.768768	-0.985114
O	-2.401745	-1.164822	-2.117900
C	-3.803154	-0.793246	-0.197196
C	-3.735018	-1.627607	1.088958
C	-5.052971	-1.516486	1.844939
C	-3.392109	-3.083067	0.795720
H	4.312575	-1.725539	1.316045
H	3.476796	0.625326	-0.412119
H	2.983824	-1.092241	2.890510
H	1.388814	-0.342375	2.976952
H	2.316969	-2.719736	0.844314
H	3.277001	-3.196691	-0.558995
H	2.775346	0.493283	3.643716
H	1.276898	1.335965	-1.127157
H	-0.357597	-0.242955	-1.971143
H	0.553748	-1.351598	0.727807
H	-0.961825	1.851221	-3.129450
H	-1.783416	3.389621	-2.495435
H	2.462874	2.488407	0.589683
H	0.027897	0.901135	1.518432
H	1.924702	2.410104	2.268629
H	1.269196	-1.118549	-2.969006
H	2.386233	-2.479236	-2.819580
H	2.957613	-0.858625	-2.498864
H	5.641310	-0.822837	-0.361733
H	-4.052389	0.241447	0.055267
H	-4.581906	-1.176820	-0.857646

H	-2.944582	-1.205827	1.717141
H	-5.013195	-2.075995	2.782603
H	-5.291027	-0.477557	2.086102
H	-5.880011	-1.920686	1.253822
H	-3.335648	-3.661758	1.720665
H	-2.430674	-3.184006	0.286113
H	-4.153552	-3.544085	0.159718

mPW1PW91 energy = -1155.17488573 a.u.

(2*R*,6*R*,7*S*,8*R*)-**1**, Conf. F

C	-4.326273	-0.615996	-0.404236
C	-3.504707	0.616715	-0.166193
C	-2.804985	1.318618	-1.057250
C	-2.859646	1.095403	-2.536481
C	-2.202842	-1.792625	0.351328
C	-3.432303	-1.881823	-0.509748
C	-0.392072	1.676074	-0.462914
C	-0.164983	0.800709	0.792931
C	0.070409	-0.694996	0.518471
C	-1.099654	-1.279688	-0.197088
O	0.586449	2.741423	-0.399424
C	1.379040	2.656120	0.678762
C	0.982162	1.478024	1.482364
O	2.254001	3.454104	0.891124
C	1.601001	1.157218	2.609328
C	-1.778356	2.315886	-0.583796
C	-2.369563	-2.102765	1.804244
O	-5.241297	-0.829869	0.668056
O	1.251170	-0.798969	-0.300842
C	2.058542	-1.852841	-0.099589
O	1.858167	-2.691068	0.744512
C	3.215770	-1.845431	-1.055924
C	4.188405	-0.674298	-0.851735
C	4.825910	-0.709029	0.531190
C	5.248355	-0.693080	-1.945393
H	-4.887821	-0.539436	-1.341314
H	-3.373540	0.855416	0.887292
H	-3.057049	2.039067	-3.054360
H	-3.627612	0.380580	-2.829537
H	-3.133303	-1.982019	-1.554643
H	-4.056189	-2.742590	-0.255830
H	-1.899963	0.731504	-2.918519
H	-1.056238	0.846110	1.423078
H	0.266641	-1.193437	1.465911
H	-1.104795	-1.085369	-1.266396
H	1.315175	0.296706	3.202954
H	2.425903	1.764170	2.964258
H	-2.057560	2.737345	0.385023
H	-0.157075	1.121544	-1.371793
H	-1.695597	3.142133	-1.294962
H	-3.057905	-1.395422	2.277428
H	-1.429394	-2.091836	2.354979
H	-2.820978	-3.091343	1.926578
H	-5.793685	-0.045414	0.748900
H	3.739926	-2.794973	-0.938796
H	2.807536	-1.802906	-2.069452

H	3.618072	0.254872	-0.944682
H	5.504036	0.136878	0.664743
H	4.078895	-0.658464	1.327011
H	5.403350	-1.627270	0.673833
H	5.942336	0.142585	-1.829594
H	4.800256	-0.619580	-2.939361
H	5.833408	-1.616982	-1.909792

mPW1PW91 energy = -1155.17483341 a.u.

H	-2.956700	-1.189406	1.681163
H	-5.030826	-2.066796	2.730848
H	-5.309916	-0.473880	2.022041
H	-5.882653	-1.925250	1.192299
H	-3.334670	-3.647564	1.702478
H	-2.414241	-3.177301	0.274989
H	-4.132942	-3.548561	0.130014

mPW1PW91 energy = -1155.17459403 a.u.

(2*R*,6*R*,7*S*,8*R*)-**1**, Conf. G

C	3.894041	-1.369571	0.370976
C	3.377942	0.024249	0.550396
C	2.638478	0.496937	1.551929
C	2.340475	-0.259618	2.809533
C	1.733722	-1.689003	-0.935454
C	2.754496	-2.348661	-0.053128
C	0.495013	1.627130	0.908080
C	0.338409	1.302413	-0.597514
C	-0.224709	-0.094613	-0.913215
C	0.662199	-1.151326	-0.348656
O	-0.225067	2.862648	1.135277
C	-0.814570	3.332491	0.026640
C	-0.517078	2.425550	-1.104820
O	-1.467545	4.343021	0.031619
C	-0.979824	2.650924	-2.326059
C	1.931309	1.819017	1.400900
C	2.097902	-1.504482	-2.374235
O	4.920431	-1.292558	-0.612668
O	-1.532484	-0.175571	-0.313929
C	-2.507432	-0.776879	-1.013938
O	-2.349974	-1.230042	-2.120802
C	-3.797362	-0.797517	-0.245735
C	-3.737710	-1.621783	1.048210
C	-5.064100	-1.512931	1.789563
C	-3.382461	-3.077565	0.771670
H	4.319146	-1.755201	1.303971
H	3.532646	0.658188	-0.319418
H	2.613181	0.339351	3.684071
H	2.875059	-1.206768	2.870339
H	2.271035	-2.701157	0.860611
H	3.218915	-3.216248	-0.533416
H	1.270202	-0.472037	2.905326
H	1.321253	1.337349	-1.073647
H	-0.338493	-0.185457	-1.991751
H	0.507705	-1.342947	0.709748
H	-0.765270	1.989858	-3.157429
H	-1.597222	3.520804	-2.518566
H	2.460909	2.469686	0.700889
H	-0.010876	0.880964	1.521061
H	1.879986	2.337074	2.362470
H	2.967422	-0.847195	-2.474282
H	1.284118	-1.093663	-2.971192
H	2.388473	-2.462414	-2.815041
H	5.289754	-2.173330	-0.729003
H	-4.056197	0.236978	-0.003733
H	-4.565117	-1.193629	-0.911672

(2*R*,6*R*,7*S*,8*R*)-**1**, Conf. H

C	4.339014	-0.596883	0.371086
C	3.523685	0.642874	0.173552
C	2.795838	1.289339	1.082422
C	2.819471	0.987124	2.548790
C	2.201016	-1.777077	-0.351754
C	3.431522	-1.859147	0.506045
C	0.387191	1.685140	0.494324
C	0.166728	0.824354	-0.772512
C	-0.069447	-0.674438	-0.517084
C	1.098121	-1.264232	0.197701
O	-0.578944	2.761765	0.428350
C	-1.366680	2.687949	-0.654058
C	-0.975392	1.510473	-1.461517
O	-2.234423	3.493728	-0.866923
C	-1.593469	1.198045	-2.591226
C	1.778353	2.308582	0.638584
C	2.363944	-2.087781	-1.805263
O	5.211261	-0.687676	-0.750098
O	-1.255209	-0.788901	0.294061
C	-2.046681	-1.853995	0.090859
O	-1.828226	-2.692628	-0.748542
C	-3.212019	-1.860139	1.037433
C	-4.196658	-0.700274	0.827529
C	-4.809970	-0.729031	-0.566389
C	-5.276868	-0.747231	1.900593
H	4.936718	-0.531010	1.287044
H	3.431465	0.941466	-0.867771
H	3.590600	0.266938	2.820164
H	1.857281	0.591625	2.890966
H	3.137930	-1.935820	1.555180
H	4.041364	-2.737698	0.271103
H	2.995741	1.903429	3.120554
H	1.062324	0.877100	-1.396059
H	-0.259418	-1.161897	-1.471505
H	1.103673	-1.070850	1.266940
H	-1.311426	0.338240	-3.187713
H	-2.413587	1.811570	-2.945915
H	2.064109	2.755705	-0.316693
H	0.134056	1.125015	1.394986
H	1.700139	3.114593	1.373407
H	1.419119	-2.095616	-2.347903
H	2.830335	-3.069410	-1.929105
H	3.034412	-1.368549	-2.286030
H	5.761833	-1.468757	-0.638110
H	-3.724390	-2.815285	0.913910
H	-2.813310	-1.815214	2.054717

H	-3.641560	0.235737	0.940829
H	-5.497891	0.108618	-0.701935
H	-4.051554	-0.659369	-1.349837
H	-5.372244	-1.653535	-0.727962
H	-5.979928	0.080422	1.782200
H	-4.849564	-0.681198	2.904184
H	-5.848095	-1.678481	1.840885

mPW1PW91 energy = -1155.17451750 a.u.

(2*R*,6*R*,7*S*,8*R*)-**1**, Conf. I

C	-4.283300	-1.187964	-0.272632
C	-3.681796	0.183000	-0.169444
C	-3.076467	0.878764	-1.130801
C	-3.066649	0.476753	-2.573197
C	-1.960333	-1.909592	0.512344
C	-3.173544	-2.290183	-0.288040
C	-0.758263	1.725200	-0.640205
C	-0.354740	0.998094	0.665884
C	0.105943	-0.460735	0.503037
C	-0.964920	-1.286986	-0.122013
O	-0.005768	2.962949	-0.664757
C	0.835274	3.080639	0.373052
C	0.696690	1.895509	1.248673
O	1.558286	4.033348	0.502587
C	1.435472	1.738036	2.337582
C	-2.240755	2.081934	-0.777831
C	-2.036386	-2.070069	1.999489
O	-5.245759	-1.409555	0.750808
O	1.273419	-0.454980	-0.341150
C	2.309072	-1.233106	0.014264
O	2.326617	-1.903267	1.016147
C	3.399374	-1.186151	-1.018309
C	4.797418	-1.452826	-0.467268
C	5.779508	-1.662075	-1.613223
C	5.257270	-0.323897	0.447162
H	-4.851204	-1.296163	-1.197755
H	-3.622398	0.568626	0.847631
H	-2.056112	0.225817	-2.912207
H	-3.404060	1.308226	-3.199468
H	-2.880750	-2.439392	-1.329459
H	-3.632820	-3.216918	0.065106
H	-3.707545	-0.380117	-2.777165
H	-1.220762	0.961460	1.331439
H	0.393946	-0.838874	1.482279
H	-1.013849	-1.212740	-1.204803
H	1.342952	0.869432	2.978841
H	2.163721	2.493011	2.610383
H	-2.580448	2.532622	0.157872
H	-0.423927	1.166569	-1.514612
H	-2.319963	2.841627	-1.560313
H	-2.452863	-3.046766	2.257727
H	-2.694629	-1.319230	2.453975
H	-1.063444	-1.979349	2.482990
H	-4.835430	-1.243880	1.605651
H	3.134654	-1.951479	-1.757455
H	3.355929	-0.227137	-1.539265

H	4.750052	-2.374371	0.120620
H	6.782547	-1.869790	-1.233395
H	5.483311	-2.500510	-2.248871
H	5.844229	-0.770938	-2.244869
H	6.245271	-0.536865	0.861922
H	4.572120	-0.177273	1.284847
H	5.323922	0.620354	-0.101874

mPW1PW91 energy = -1155.17348124 a.u.

(2*R*,6*R*,7*S*,8*R*)-**1**, Conf. J

C	-4.072264	-1.542155	-0.300758
C	-3.633573	-0.107134	-0.267679
C	-3.069502	0.592449	-1.250956
C	-2.950538	0.106111	-2.662151
C	-1.723391	-1.950364	0.609844
C	-2.845665	-2.507447	-0.219058
C	-0.883596	1.709158	-0.725302
C	-0.476482	1.122868	0.648463
C	0.164582	-0.277134	0.607001
C	-0.767368	-1.259620	-0.014896
O	-0.253589	3.009947	-0.815623
C	0.495956	3.300646	0.256857
C	0.421227	2.180069	1.221336
O	1.103629	4.334880	0.348708
C	1.077692	2.202172	2.372356
C	-2.386160	1.900610	-0.949382
C	-1.862607	-2.029306	2.099113
O	-5.034721	-1.811750	0.711317
O	1.366752	-0.185324	-0.181732
C	2.506748	-0.668401	0.340061
O	2.573607	-1.205068	1.417126
C	3.668510	-0.403588	-0.575309
C	4.861815	-1.329399	-0.361723
C	4.540547	-2.757021	-0.787210
C	6.079396	-0.796423	-1.106396
H	-4.595265	-1.769398	-1.230968
H	-3.666019	0.346904	0.721674
H	-1.903029	-0.009292	-2.959400
H	-3.385471	0.837281	-3.350666
H	-2.490185	-2.667076	-1.239184
H	-3.211441	-3.465022	0.160506
H	-3.449578	-0.848400	-2.824253
H	-1.370521	1.019956	1.268010
H	0.440863	-0.558551	1.621795
H	-0.763754	-1.255275	-1.101309
H	1.029384	1.385880	3.083413
H	1.692090	3.058156	2.626991
H	-2.816807	2.372717	-0.062957
H	-0.456089	1.125075	-1.540544
H	-2.504696	2.594531	-1.786109
H	-2.612304	-1.321747	2.474360
H	-0.929733	-1.814546	2.620585
H	-2.195018	-3.025714	2.400237
H	-4.664736	-1.566204	1.565207
H	3.318330	-0.438206	-1.609991
H	3.957536	0.637288	-0.387754

H	5.086526	-1.333456	0.709020
H	5.385450	-3.421758	-0.592443
H	3.677608	-3.153998	-0.248457
H	4.321557	-2.804618	-1.858434
H	6.948558	-1.437533	-0.942184
H	6.341645	0.211840	-0.776065
H	5.897722	-0.756848	-2.184711

mPW1PW91energy = -1155.17347120 a.u.

(2*R*,6*R*,7*S*,8*R*)-**1**, Conf. K

C	3.934734	-1.364452	0.355619
C	3.411821	0.034236	0.505751
C	2.687806	0.532924	1.506468
C	2.431833	-0.182275	2.796624
C	1.746270	-1.707390	-0.914936
C	2.792365	-2.351522	-0.050860
C	0.518777	1.640561	0.884321
C	0.316454	1.278057	-0.607448
C	-0.225522	-0.135840	-0.881795
C	0.684438	-1.168539	-0.311876
O	-0.187282	2.886566	1.098758
C	-0.821253	3.321282	0.000375
C	-0.582328	2.370315	-1.108219
O	-1.466926	4.336362	-0.003142
C	-1.138292	2.529690	-2.300651
C	1.968500	1.845406	1.332848
C	2.064999	-1.531540	-2.367700
O	5.031391	-1.411601	-0.549201
O	-1.523936	-0.229714	-0.265475
C	-2.525061	-0.762821	-0.984283
O	-2.394621	-1.162314	-2.115076
C	-3.809042	-0.779458	-0.206216
C	-3.755558	-1.614671	1.080035
C	-5.079831	-1.498122	1.824018
C	-3.416482	-3.071449	0.788876
H	4.349996	-1.731062	1.295293
H	3.532991	0.651174	-0.383791
H	1.363486	-0.369241	2.946914
H	2.754825	0.437090	3.639347
H	2.324915	-2.702807	0.871531
H	3.264136	-3.210829	-0.534300
H	2.950941	-1.137357	2.863309
H	1.281576	1.328372	-1.117475
H	-0.349652	-0.251246	-1.957027
H	0.553534	-1.346462	0.751859
H	-0.978976	1.830255	-3.112791
H	-1.779897	3.383076	-2.487405
H	2.477219	2.477330	0.600816
H	0.027362	0.911763	1.529876
H	1.940494	2.391739	2.279614
H	2.487047	-2.450606	-2.781243
H	2.813429	-0.745425	-2.524605
H	1.190295	-1.265959	-2.961641
H	4.773290	-0.991341	-1.375850
H	-4.054106	0.256633	0.044535
H	-4.584953	-1.158415	-0.872650

H	-2.968906	-1.196643	1.715414
H	-5.051098	-2.058501	2.761538
H	-5.315451	-0.458277	2.063628
H	-5.903122	-1.898201	1.224927
H	-3.372081	-3.651082	1.713843
H	-2.450408	-3.176408	0.288884
H	-4.173668	-3.528321	0.144840

mPW1PW91 energy = -1155.17301201 a.u.

(2*R*,6*R*,7*S*,8*R*)-**1**, Conf. L

C	4.343034	-0.614901	0.383650
C	3.527630	0.625729	0.166068
C	2.807223	1.294269	1.065537
C	2.842400	1.021088	2.537153
C	2.193127	-1.791386	-0.351460
C	3.430210	-1.878679	0.497761
C	0.395998	1.686688	0.482305
C	0.165180	0.818324	-0.777565
C	-0.071370	-0.678933	-0.513055
C	1.097970	-1.265236	0.200914
O	-0.568299	2.764559	0.416137
C	-1.363103	2.686172	-0.660813
C	-0.979972	1.502666	-1.463498
O	-2.229920	3.492761	-0.873462
C	-1.606832	1.184468	-2.586673
C	1.789464	2.308669	0.611141
C	2.327014	-2.119659	-1.806332
O	5.340297	-0.760494	-0.619867
O	-1.254976	-0.789239	0.300626
C	-2.053325	-1.849478	0.095561
O	-1.841589	-2.685758	-0.747782
C	-3.215731	-1.852391	1.045389
C	-4.196845	-0.688808	0.838976
C	-4.818798	-0.718033	-0.551109
C	-5.270115	-0.728489	1.919206
H	4.909824	-0.550464	1.313739
H	3.419654	0.911017	-0.879642
H	3.621345	0.313083	2.817698
H	1.885439	0.624314	2.892246
H	3.135850	-1.967837	1.545648
H	4.046631	-2.746397	0.249412
H	3.014120	1.949884	3.089573
H	1.056720	0.867182	-1.407229
H	-0.262214	-1.171124	-1.465198
H	1.112053	-1.062644	1.268280
H	-1.332013	0.319800	-3.179486
H	-2.427958	1.797902	-2.939186
H	2.071402	2.742599	-0.351411
H	0.148956	1.132828	1.388523
H	1.716980	3.124751	1.335146
H	1.362642	-2.248844	-2.297779
H	2.898032	-3.042309	-1.935610
H	2.868591	-1.338114	-2.352878
H	4.919616	-0.723964	-1.484866
H	-3.731773	-2.805663	0.922804
H	-2.813726	-1.809379	2.061406

H	-3.637026	0.245074	0.946654
H	-5.503593	0.122497	-0.684605
H	-4.064554	-0.653790	-1.339054
H	-5.386219	-1.640308	-0.707234
H	-5.970712	0.101554	1.803018
H	-4.835717	-0.661117	2.919640
H	-5.845415	-1.657634	1.866233

mPW1PW91 energy = -1155.17291002 a.u.

(2*R*,6*R*,7*S*,8*R*)-**1**, Conf. M

C	3.769324	-1.704685	0.160468
C	3.415075	-0.249279	0.248303
C	2.899473	0.408110	1.286425
C	2.742271	-0.167641	2.659467
C	1.453440	-1.863739	-0.877706
C	2.508920	-2.581145	-0.082941
C	0.795784	1.694157	0.808501
C	0.390283	1.272453	-0.625125
C	-0.332023	-0.082898	-0.734888
C	0.525784	-1.180119	-0.204636
O	0.236515	3.013756	1.018192
C	-0.469739	3.452694	-0.033507
C	-0.436282	2.430194	-1.103060
O	-1.016342	4.524518	-0.031321
C	-1.079994	2.593464	-2.250000
C	2.300349	1.776097	1.082574
C	1.643783	-1.811946	-2.359782
O	4.667100	-1.942598	-0.921460
O	-1.543108	0.013497	0.040358
C	-2.683757	-0.430321	-0.514666
O	-2.738202	-0.933144	-1.610050
C	-3.863618	-0.212995	0.390554
C	-4.373312	-1.490069	1.087020
C	-4.940399	-2.509885	0.108187
C	-3.315569	-2.115992	1.987833
H	4.231505	-2.055406	1.088882
H	3.464025	0.268613	-0.707484
H	3.151404	-1.172990	2.750627
H	1.688775	-0.206647	2.955558
H	2.104752	-2.850077	0.894522
H	2.843078	-3.500384	-0.570655
H	3.241578	0.470580	3.395355
H	1.290985	1.178764	-1.236248
H	-0.606759	-0.243516	-1.775725
H	0.494117	-1.287448	0.875927
H	-1.067338	1.843241	-3.031918
H	-1.646359	3.500371	-2.427655
H	2.782338	2.295860	0.251000
H	0.315850	1.054783	1.550186
H	2.434798	2.386979	1.979420
H	1.741263	-2.824824	-2.761117
H	2.574365	-1.295040	-2.614154
H	0.822581	-1.320554	-2.880797
H	5.452837	-1.404376	-0.780170
H	-3.599873	0.531006	1.142450
H	-4.662448	0.196294	-0.231494
H	-5.195343	-1.149684	1.725694
H	-5.378995	-3.351186	0.650500
H	-5.720934	-2.072161	-0.518532
H	-4.167518	-2.905365	-0.553894
H	-3.740691	-2.947727	2.554422
H	-2.916450	-1.393959	2.704327
H	-2.478001	-2.512112	1.407094

mPW1PW91 energy = -1155.17279154 a.u.

(2*R*,6*R*,7*S*,8*S*)-**1**, Conf. A

C	3.117437	-2.637029	-0.345738
C	3.154463	-1.242940	-0.878105
C	3.552401	-0.146571	-0.230123
C	4.348787	-0.127710	1.039094
C	0.859951	-1.949176	0.552076
C	1.640156	-3.044028	-0.128169
C	1.811860	1.599181	0.109657
C	0.430267	1.370196	-0.563744
C	-0.370660	0.251223	0.142058
C	0.205914	-1.076625	-0.215978
O	1.888247	3.011640	0.417538
C	0.762398	3.670926	0.115316
C	-0.193257	2.737452	-0.522410
O	0.631491	4.845171	0.342720
C	-1.347013	3.179363	-1.005437
C	3.069282	1.206113	-0.686409
C	1.007367	-1.855332	2.036059
O	3.657538	-3.601450	-1.246866
O	-1.723242	0.323916	-0.360903
C	-2.702478	-0.086167	0.459312
O	-2.506772	-0.491196	1.577960
C	-4.058417	0.067954	-0.171936
C	-5.014115	-1.082568	0.144751
C	-6.429471	-0.725908	-0.290452
C	-4.550196	-2.379763	-0.506775
H	3.645096	-2.698669	0.609592
H	2.612158	-1.109477	-1.811910
H	5.267374	0.446116	0.878300
H	4.633888	-1.118802	1.387689
H	1.617365	-3.975971	0.442339
H	1.218712	-3.245000	-1.115598
H	3.814288	0.378733	1.848422
H	0.573026	1.068297	-1.604014
H	-0.395551	0.429780	1.215544
H	0.173053	-1.273192	-1.285471
H	-2.046146	2.535732	-1.520197
H	-1.602105	4.226949	-0.890161
H	2.854499	1.214739	-1.756538
H	1.831259	1.097286	1.078316
H	3.826774	1.969037	-0.490774
H	2.059288	-1.752177	2.319275
H	0.654084	-2.781094	2.500502
H	0.449851	-1.029611	2.474442
H	4.581412	-3.377100	-1.397342
H	-4.464108	1.006276	0.222758

H	-3.946686	0.196863	-1.250179
H	-5.009732	-1.222262	1.229732
H	-7.123999	-1.536785	-0.058854
H	-6.788746	0.174887	0.213313
H	-6.478226	-0.547300	-1.368900
H	-3.543498	-2.658442	-0.186224
H	-4.542319	-2.289453	-1.597451
H	-5.217171	-3.205549	-0.248483

mPW1PW91 energy = -1155.17247321 a.u.

(2*R*,6*R*,7*S*,8*S*)-**1**, Conf. B

C	3.742898	-2.103268	-0.285609
C	3.513473	-0.717489	-0.790077
C	3.609123	0.416994	-0.094330
C	4.281141	0.566066	1.237072
C	1.330534	-1.958237	0.450288
C	2.378665	-2.829172	-0.192474
C	1.511380	1.732862	0.145163
C	0.258367	1.232787	-0.626642
C	-0.317467	-0.059601	-0.003283
C	0.560370	-1.215199	-0.345972
O	1.258702	3.117513	0.483353
C	0.032369	3.522683	0.128452
C	-0.653494	2.427303	-0.592691
O	-0.369330	4.629293	0.376667
C	-1.845133	2.623383	-1.141558
C	2.874073	1.645420	-0.565278
C	1.342622	-1.897354	1.943410
O	4.545258	-2.896675	-1.157612
O	-1.616755	-0.271911	-0.598765
C	-2.530685	-0.914997	0.145506
O	-2.344172	-1.243712	1.289986
C	-3.775814	-1.201296	-0.645965
C	-5.038087	-1.332276	0.201129
C	-5.421366	-0.001825	0.838132
C	-6.180989	-1.883847	-0.641836
H	4.199224	-2.081621	0.707256
H	3.030770	-0.675213	-1.764440
H	5.066523	1.325424	1.162159
H	4.741613	-0.351869	1.597828
H	2.521888	-3.765479	0.352764
H	2.082539	-3.078320	-1.214030
H	3.588745	0.924665	2.004613
H	0.526526	1.010221	-1.662179
H	-0.451892	0.071591	1.068565
H	0.648765	-1.370113	-1.418987
H	-2.349736	1.862604	-1.720043
H	-2.334775	3.582988	-1.017831
H	2.733862	1.637784	-1.647726
H	1.573723	1.214982	1.103841
H	3.431498	2.549184	-0.306762
H	0.577064	-1.241556	2.354353
H	2.317427	-1.566686	2.314979
H	1.181132	-2.898456	2.354731
H	5.404623	-2.470167	-1.235514
H	-3.894977	-0.438182	-1.418774

H	-3.576380	-2.141976	-1.173230
H	-4.823000	-2.046222	1.001555
H	-6.300928	-0.115600	1.475938
H	-4.615168	0.398117	1.456691
H	-5.661673	0.743421	0.073462
H	-7.086330	-2.000899	-0.041653
H	-5.932367	-2.860207	-1.065321
H	-6.420178	-1.212096	-1.471891

mPW1PW91 energy = -1155.17240079 a.u.

(2*R*,6*R*,7*S*,8*S*)-**1**, Conf. C

C	3.625207	-2.216531	-0.367897
C	3.447803	-0.797996	-0.799455
C	3.611425	0.295841	-0.052662
C	4.304438	0.346959	1.275129
C	1.225992	-2.006946	0.384590
C	2.236476	-2.897143	-0.290311
C	1.564911	1.677397	0.224424
C	0.330798	1.231495	-0.606117
C	-0.337399	-0.031743	-0.001525
C	0.463465	-1.228641	-0.385119
O	1.324790	3.052237	0.608571
C	0.142738	3.513515	0.178140
C	-0.521702	2.466505	-0.630286
O	-0.238454	4.625618	0.432958
C	-1.639666	2.724033	-1.296153
C	2.941254	1.584732	-0.455682
C	1.271535	-1.967132	1.878107
O	4.386361	-2.994458	-1.289733
O	-1.655024	-0.172819	-0.572353
C	-2.704817	-0.205153	0.264953
O	-2.610268	-0.117396	1.463714
C	-4.001583	-0.329652	-0.485343
C	-5.058712	-1.153555	0.248177
C	-6.405845	-1.017748	-0.449109
C	-4.642619	-2.615136	0.360144
H	4.095502	-2.262180	0.617683
H	2.958687	-0.684442	-1.764923
H	5.124052	1.071222	1.225220
H	4.723312	-0.608270	1.586388
H	2.348838	-3.854481	0.224818
H	1.925996	-3.103039	-1.317169
H	3.637821	0.701424	2.067086
H	0.640164	0.983608	-1.623581
H	-0.446536	0.086053	1.073652
H	0.523289	-1.371998	-1.461709
H	-2.107505	1.995650	-1.944469
H	-2.103192	3.699889	-1.203698
H	2.829916	1.658978	-1.539058
H	1.592031	1.123971	1.164451
H	3.529203	2.442397	-0.120098
H	0.529851	-1.300199	2.315161
H	2.260570	-1.662407	2.233259
H	1.097727	-2.970505	2.278905
H	5.264025	-2.603690	-1.349631
H	-4.363777	0.695339	-0.624982

H	-3.806765	-0.734737	-1.480543
H	-5.149614	-0.743488	1.258241
H	-7.175707	-1.586945	0.077096
H	-6.731699	0.024624	-0.490680
H	-6.360243	-1.394539	-1.475400
H	-5.390310	-3.191173	0.910291
H	-3.690700	-2.726170	0.884326
H	-4.536369	-3.068950	-0.629922

mPW1PW91 energy = -1155.17221838 a.u.

(2*R*,6*R*,7*S*,8*S*)-**1**, Conf. D

C	3.128855	-2.533739	-0.387412
C	3.069368	-1.156312	-0.959966
C	3.457271	-0.024728	-0.368646
C	4.323697	0.063692	0.851017
C	0.910585	-1.922254	0.650237
C	1.689747	-2.999209	-0.058980
C	1.672764	1.658216	0.036727
C	0.263370	1.335937	-0.531451
C	-0.434226	0.215462	0.276625
C	0.164226	-1.100265	-0.089252
O	1.697085	3.083407	0.288750
C	0.523154	3.675887	0.033517
C	-0.421011	2.673040	-0.508135
O	0.347554	4.850854	0.223478
C	-1.619360	3.038061	-0.944194
C	2.890483	1.293202	-0.830232
C	1.157346	-1.782044	2.117493
O	3.652050	-3.501169	-1.295128
O	-1.827390	0.208953	-0.102451
C	-2.733588	-0.012206	0.863168
O	-2.448807	-0.154109	2.026755
C	-4.127591	-0.051797	0.306414
C	-4.386895	-1.239053	-0.632810
C	-4.172544	-2.570457	0.076075
C	-5.795167	-1.141814	-1.205159
H	3.719711	-2.542820	0.532080
H	2.466972	-1.075841	-1.862562
H	4.674048	-0.904184	1.204970
H	3.811586	0.564142	1.678396
H	1.749539	-3.916730	0.531750
H	1.209717	-3.243002	-1.009575
H	5.203421	0.675335	0.625463
H	0.353232	0.992727	-1.564748
H	-0.372706	0.431090	1.341170
H	0.061783	-1.329447	-1.147593
H	-2.310227	2.339719	-1.395315
H	-1.921539	4.075828	-0.858218
H	2.604334	1.260266	-1.883112
H	1.782765	1.196227	1.019060
H	3.626800	2.091339	-0.707491
H	0.582438	-0.979164	2.575901
H	2.218152	-1.609655	2.322842
H	0.893921	-2.714540	2.626043
H	4.554633	-3.244451	-1.509536
H	-4.812964	-0.086391	1.154331

H	-4.301435	0.883261	-0.233099
H	-3.674536	-1.170891	-1.460944
H	-4.361050	-3.404833	-0.603439
H	-3.151642	-2.675694	0.450989
H	-4.851622	-2.674201	0.927399
H	-5.994560	-1.968196	-1.891332
H	-5.941785	-0.208540	-1.754553
H	-6.545848	-1.182550	-0.410340

mPW1PW91 energy = -1155.17187674 a.u.

(2*R*,6*R*,7*S*,8*S*)-**1**, Conf. E

C	3.128145	-2.638479	-0.338769
C	3.162650	-1.246246	-0.879589
C	3.554646	-0.145511	-0.236916
C	4.353362	-0.120667	1.030194
C	0.860198	-1.946216	0.551622
C	1.641645	-3.040860	-0.127012
C	1.811767	1.599045	0.103708
C	0.427253	1.372601	-0.564687
C	-0.372313	0.253799	0.142569
C	0.203133	-1.074572	-0.215186
O	1.891269	3.011392	0.411568
C	0.765119	3.672318	0.114692
C	-0.194716	2.740441	-0.519261
O	0.636542	4.846604	0.343045
C	-1.350523	3.183903	-0.995868
C	3.065785	1.203916	-0.696786
C	1.014054	-1.850201	2.034786
O	3.784955	-3.600286	-1.156013
O	-1.725528	0.326274	-0.358920
C	-2.703627	-0.086131	0.461747
O	-2.506120	-0.493669	1.579086
C	-4.060462	0.068276	-0.167477
C	-5.013777	-1.085199	0.145728
C	-6.430027	-0.729902	-0.287608
C	-4.547603	-2.379295	-0.510350
H	3.648542	-2.690549	0.616360
H	2.613448	-1.111263	-1.810640
H	5.273981	0.447684	0.862101
H	4.637651	-1.110385	1.382918
H	1.616043	-3.974601	0.440290
H	1.214149	-3.238040	-1.114692
H	3.823152	0.393796	1.837325
H	0.565742	1.072077	-1.605987
H	-0.395757	0.432674	1.216041
H	0.166438	-1.272096	-1.284553
H	-2.053103	2.541474	-1.507419
H	-1.604093	4.231588	-0.878187
H	2.845858	1.207596	-1.765958
H	1.833479	1.096982	1.072201
H	3.823117	1.968894	-0.508902
H	0.657433	-2.772933	2.502661
H	0.463288	-1.020219	2.473500
H	2.067630	-1.752527	2.313478
H	3.379030	-3.584456	-2.029531
H	-4.467427	1.004248	0.231399

H	-3.950215	0.201705	-1.245341
H	-5.008606	-1.228570	1.230205
H	-7.122954	-1.542682	-0.057921
H	-6.790624	0.168743	0.219006
H	-6.479811	-0.548212	-1.365501
H	-5.212803	-3.207269	-0.254511
H	-3.540255	-2.657052	-0.190961
H	-4.540530	-2.285243	-1.600734

mPW1PW91 energy = -1155.17134766 a.u.

(2*R*,6*R*,7*S*,8*S*)-**1**, Conf. F

C	3.126808	-2.637248	-0.350917
C	3.162410	-1.248235	-0.887437
C	3.550119	-0.150919	-0.236953
C	4.345990	-0.128109	1.032260
C	0.861646	-1.940504	0.555956
C	1.638745	-3.034674	-0.127605
C	1.812402	1.600438	0.104444
C	0.427312	1.375688	-0.562781
C	-0.372315	0.258186	0.145930
C	0.203296	-1.070209	-0.211243
O	1.895298	3.013563	0.408727
C	0.769924	3.675833	0.112314
C	-0.192739	2.744368	-0.517959
O	0.643782	4.850887	0.338557
C	-1.348791	3.188737	-0.993181
C	3.064591	1.200119	-0.695650
C	1.018753	-1.841553	2.038633
O	3.735151	-3.524303	-1.283656
O	-1.725526	0.330092	-0.355730
C	-2.703263	-0.086077	0.462923
O	-2.506054	-0.496364	1.579470
C	-4.059805	0.067996	-0.167117
C	-5.012667	-1.086410	0.143787
C	-6.428631	-0.731611	-0.290985
C	-4.544871	-2.379475	-0.513197
H	3.656933	-2.693257	0.603108
H	2.623023	-1.118173	-1.823106
H	3.808794	0.372715	1.843356
H	5.260452	0.451939	0.870845
H	1.609401	-3.967218	0.445107
H	1.214509	-3.231830	-1.114665
H	4.639858	-1.117989	1.376750
H	0.564725	1.074147	-1.603883
H	-0.395840	0.438356	1.219225
H	0.165586	-1.269237	-1.280112
H	-2.053051	2.546338	-1.502441
H	-1.600532	4.237044	-0.877119
H	2.844914	1.204395	-1.764794
H	1.833266	1.101175	1.074428
H	3.824842	1.962225	-0.507585
H	2.071955	-1.731021	2.314062
H	0.672953	-2.767041	2.509332
H	0.460179	-1.016850	2.477576
H	3.641300	-4.423076	-0.950652
H	-4.467749	1.003311	0.232336

H	-3.948577	0.202679	-1.244722
H	-5.008759	-1.230924	1.228136
H	-7.121380	-1.545044	-0.063025
H	-6.790458	0.166345	0.215990
H	-6.477083	-0.548932	-1.368765
H	-5.209664	-3.208251	-0.258813
H	-3.537487	-2.656634	-0.193412
H	-4.536810	-2.284246	-1.603441

mPW1PW91 energy = -1155.17134598 a.u.

(2*R*,6*R*,7*S*,8*S*)-**1**, Conf. G

C	3.748913	-2.108737	-0.276874
C	3.520374	-0.725871	-0.792914
C	3.612733	0.413220	-0.105762
C	4.289093	0.570951	1.221968
C	1.326359	-1.953275	0.454711
C	2.373060	-2.827523	-0.185241
C	1.515944	1.732090	0.136567
C	0.255487	1.236753	-0.626372
C	-0.320899	-0.053908	-0.000504
C	0.554089	-1.212028	-0.341523
O	1.271518	3.118455	0.474083
C	0.043983	3.527907	0.128975
C	-0.652224	2.434264	-0.585106
O	-0.351645	4.636251	0.379055
C	-1.848524	2.634024	-1.122379
C	2.873992	1.637310	-0.582198
C	1.344988	-1.885629	1.947525
O	4.652605	-2.881936	-1.057765
O	-1.620643	-0.264544	-0.595855
C	-2.532737	-0.914132	0.145251
O	-2.344352	-1.249767	1.287310
C	-3.777849	-1.197920	-0.647199
C	-5.040060	-1.334102	0.199180
C	-5.424268	-0.007397	0.843297
C	-6.182439	-1.881770	-0.647072
H	4.199317	-2.078573	0.713770
H	3.028650	-0.685784	-1.764371
H	5.079492	1.323815	1.136025
H	4.747213	-0.345735	1.588530
H	2.512515	-3.765129	0.358542
H	2.068892	-3.075445	-1.206528
H	3.601538	0.941692	1.988140
H	0.515495	1.014162	-1.664068
H	-0.454633	0.079036	1.071241
H	0.637333	-1.371097	-1.414510
H	-2.361512	1.874581	-1.695225
H	-2.333649	3.595366	-0.994491
H	2.726839	1.620812	-1.663701
H	1.581916	1.215502	1.095674
H	3.433630	2.542975	-0.335587
H	0.581196	-1.227837	2.358445
H	2.321197	-1.552864	2.313370
H	1.185437	-2.884740	2.364303
H	4.319553	-2.916358	-1.961027
H	-3.897847	-0.431403	-1.416506

H	-3.577742	-2.136154	-1.178607
H	-4.824706	-2.052198	0.995807
H	-6.304433	-0.125155	1.479517
H	-4.618856	0.389210	1.465022
H	-5.664022	0.742143	0.082662
H	-7.087902	-2.002366	-0.047822
H	-5.933273	-2.855795	-1.075613
H	-6.421590	-1.205687	-1.473604

mPW1PW91 energy = -1155.17127211 a.u.

(2*R*,6*R*,7*S*,8*S*)-**1**, Conf. H

C	3.749526	-2.102892	-0.284889
C	3.525032	-0.722586	-0.796601
C	3.608020	0.410988	-0.099624
C	4.275067	0.564776	1.233505
C	1.324338	-1.950000	0.446690
C	2.370518	-2.819416	-0.199935
C	1.513766	1.737876	0.137868
C	0.253524	1.244119	-0.626262
C	-0.321141	-0.048155	-0.002974
C	0.555375	-1.203826	-0.348051
O	1.272550	3.126187	0.470572
C	0.044188	3.535524	0.128619
C	-0.654469	2.441334	-0.582232
O	-0.350387	4.644388	0.378491
C	-1.852410	2.640903	-1.115944
C	2.873230	1.637218	-0.576683
C	1.338255	-1.891615	1.939936
O	4.612569	-2.806420	-1.171959
O	-1.621638	-0.259069	-0.596916
C	-2.528589	-0.918708	0.141080
O	-2.335280	-1.263737	1.279674
C	-3.775351	-1.200647	-0.649424
C	-5.035240	-1.340777	0.199819
C	-5.418712	-0.016593	0.849559
C	-6.179479	-1.886390	-0.645205
H	4.196972	-2.074624	0.711575
H	3.049577	-0.683445	-1.774324
H	4.740648	-0.350406	1.594453
H	3.580085	0.921422	1.999653
H	2.500679	-3.761710	0.341839
H	2.075917	-3.057535	-1.224595
H	5.057786	1.326755	1.158108
H	0.513837	1.024236	-1.664475
H	-0.453871	0.082198	1.069258
H	0.643414	-1.356544	-1.421414
H	-2.367086	1.881187	-1.686898
H	-2.336946	3.602443	-0.987272
H	2.729465	1.621336	-1.658574
H	1.575237	1.224166	1.098911
H	3.435409	2.540837	-0.328052
H	0.574004	-1.235137	2.352097
H	2.313634	-1.562175	2.310984
H	1.175141	-2.892773	2.350645
H	4.677076	-3.718482	-0.869458
H	-3.897950	-0.431629	-1.415768

H	-3.575787	-2.136939	-1.184442
H	-4.817262	-2.061406	0.993451
H	-6.297487	-0.137024	1.487212
H	-4.612157	0.378174	1.471009
H	-5.660296	0.735595	0.092132
H	-7.083308	-2.009485	-0.043985
H	-5.930803	-2.858842	-1.077587
H	-6.421132	-1.207791	-1.468917

mPW1PW91 energy = -1155.17125786 a.u.

(2*R*,6*R*,7*S*,8*S*)-**1**, Conf. I

C	3.969357	-1.639816	-0.421034
C	3.474980	-0.322469	-0.921068
C	3.464432	0.835255	-0.257658
C	4.232484	1.124643	0.995996
C	1.636051	-1.852560	0.523780
C	2.755351	-2.570594	-0.184673
C	1.201171	1.764837	0.141879
C	0.009912	1.052903	-0.557275
C	-0.340508	-0.275963	0.155387
C	0.676032	-1.301797	-0.220359
O	0.724945	3.075664	0.530025
C	-0.555828	3.283416	0.195715
C	-1.060675	2.104740	-0.542752
O	-1.128656	4.305512	0.469049
C	-2.242467	2.131584	-1.143325
C	2.502344	1.921994	-0.665684
C	1.776796	-1.698794	2.003553
O	4.803603	-2.321746	-1.355440
O	-1.636618	-0.701438	-0.320630
C	-2.339854	-1.503246	0.496786
O	-1.975132	-1.798281	1.608060
C	-3.610375	-1.987753	-0.141740
C	-4.638577	-0.887111	-0.435943
C	-5.873476	-1.499641	-1.084391
C	-5.007981	-0.109209	0.820541
H	4.508578	-1.516165	0.521741
H	2.896277	-0.385699	-1.840493
H	4.879882	0.306653	1.306942
H	3.570063	1.372917	1.830977
H	3.094865	-3.447899	0.371561
H	2.418149	-2.906089	-1.168059
H	4.861931	2.006180	0.837002
H	0.273752	0.815532	-1.589898
H	-0.408303	-0.115567	1.229570
H	0.689010	-1.504188	-1.289221
H	-2.600200	1.309302	-1.747190
H	-2.874147	3.006745	-1.041179
H	2.288724	1.890795	-1.735767
H	1.428579	1.261347	1.082318
H	2.914620	2.907202	-0.435000
H	1.843270	-2.685591	2.472128
H	0.944864	-1.167821	2.463109
H	2.703834	-1.175654	2.257285
H	5.573346	-1.768094	-1.521215
H	-3.343211	-2.494001	-1.073613

H	-4.039802	-2.728391	0.534488
H	-4.189469	-0.192101	-1.150980
H	-6.602947	-0.726783	-1.337917
H	-5.622449	-2.036191	-2.002769
H	-6.361379	-2.206663	-0.407249
H	-5.740252	0.668654	0.592383
H	-4.140947	0.379965	1.271003
H	-5.446799	-0.768594	1.575141

mPW1PW91 energy = -1155.17082270 a.u.

(2*R*,6*R*,7*S*,8*S*)-**1**, Conf. J

C	3.998781	-1.630381	-0.396524
C	3.524476	-0.288152	-0.847784
C	3.487156	0.834418	-0.127259
C	4.193257	1.054356	1.176143
C	1.632962	-1.880093	0.450874
C	2.778611	-2.570510	-0.242264
C	1.212457	1.766111	0.201939
C	0.067160	1.070088	-0.583296
C	-0.321423	-0.280096	0.067416
C	0.702815	-1.298324	-0.307254
O	0.708884	3.065033	0.595532
C	-0.548120	3.281737	0.184785
C	-1.006803	2.118500	-0.606143
O	-1.136615	4.298791	0.443293
C	-2.157649	2.150708	-1.264420
C	2.555023	1.950163	-0.526809
C	1.717360	-1.787129	1.939862
O	4.861381	-2.267569	-1.336858
O	-1.602326	-0.668582	-0.472188
C	-2.348796	-1.491830	0.282857
O	-2.013266	-1.872044	1.377305
C	-3.637741	-1.851117	-0.396719
C	-4.734468	-0.784846	-0.227168
C	-5.955918	-1.182199	-1.046094
C	-5.102599	-0.568809	1.234879
H	4.509442	-1.552961	0.566487
H	2.989940	-0.300482	-1.795525
H	4.857434	1.920195	1.085571
H	4.795866	0.205285	1.493901
H	3.098495	-3.468388	0.292365
H	2.479351	-2.868382	-1.249745
H	3.491980	1.294100	1.981252
H	0.393049	0.861913	-1.604509
H	-0.429718	-0.159112	1.143513
H	0.757451	-1.458021	-1.382185
H	-2.480647	1.337676	-1.899673
H	-2.797941	3.021464	-1.179015
H	2.396294	1.977184	-1.606581
H	1.389920	1.237892	1.139600
H	2.962384	2.917253	-0.222579
H	0.870992	-1.270227	2.388963
H	2.636153	-1.280632	2.250788
H	1.760675	-2.793062	2.368668
H	5.638595	-1.710752	-1.448375
H	-3.442019	-2.001639	-1.460129

H	-3.977991	-2.795919	0.030711
H	-4.348864	0.159273	-0.625644
H	-6.736914	-0.421606	-0.973772
H	-5.708027	-1.307097	-2.103033
H	-6.377244	-2.126008	-0.687792
H	-5.883846	0.189587	1.324713
H	-4.248793	-0.236343	1.828408
H	-5.479731	-1.491791	1.684819

mPW1PW91 energy = -1155.17081948 a.u.

(2*R*,6*R*,7*S*,8*S*)-**1**, Conf. K

C	3.146606	-2.530972	-0.377142
C	3.084761	-1.156062	-0.958470
C	3.461036	-0.019363	-0.371064
C	4.325201	0.077577	0.848972
C	0.910471	-1.922082	0.639696
C	1.695275	-2.994951	-0.068803
C	1.670390	1.659670	0.030582
C	0.259042	1.339377	-0.534145
C	-0.435676	0.215803	0.271753
C	0.163155	-1.098690	-0.097674
O	1.696801	3.084884	0.282680
C	0.521739	3.677952	0.034847
C	-0.425661	2.676192	-0.503502
O	0.347090	4.852605	0.227464
C	-1.626835	3.042194	-0.930894
C	2.886263	1.293086	-0.838515
C	1.158906	-1.784127	2.106920
O	3.801515	-3.486591	-1.202716
O	-1.829736	0.208777	-0.104555
C	-2.733397	-0.016472	0.862745
O	-2.445353	-0.162272	2.024983
C	-4.128827	-0.055600	0.309609
C	-4.390645	-1.242529	-0.629435
C	-4.174354	-2.574205	0.078363
C	-5.800470	-1.145006	-1.197871
H	3.720566	-2.529543	0.548148
H	2.478108	-1.076221	-1.859909
H	5.206590	0.684853	0.618427
H	4.675218	-0.888082	1.208858
H	1.747508	-3.917990	0.513857
H	1.217251	-3.228823	-1.025115
H	3.813408	0.585915	1.671776
H	0.345717	1.000785	-1.569314
H	-0.372306	0.428709	1.336717
H	0.060121	-1.325025	-1.156719
H	-2.320801	2.344977	-1.378963
H	-1.928446	4.079731	-0.840247
H	2.596627	1.252401	-1.890239
H	1.781738	1.197574	1.012664
H	3.619962	2.094812	-0.724227
H	0.899360	-2.718322	2.614222
H	0.582359	-0.983719	2.567420
H	2.219468	-1.608660	2.310664
H	3.357822	-3.503696	-2.057636
H	-4.811950	-0.090783	1.159294

H	-4.304232	0.879604	-0.229071
H	-3.680557	-1.173840	-1.459530
H	-4.364855	-3.408455	-0.600798
H	-3.152525	-2.679480	0.450785
H	-4.851151	-2.678168	0.931438
H	-6.001901	-1.971002	-1.883927
H	-5.948567	-0.211390	-1.746265
H	-6.548933	-1.186139	-0.401011

mPW1PW91 energy = -1155.17072109 a.u.

(2*R*,6*R*,7*S*,8*S*)-**1**, Conf. L

C	3.120324	-2.523954	-0.408980
C	3.050601	-1.154890	-0.992024
C	3.435120	-0.019456	-0.408197
C	4.317746	0.078841	0.798857
C	0.914608	-1.898583	0.679001
C	1.676157	-2.976772	-0.045858
C	1.664972	1.675649	0.020205
C	0.240754	1.352756	-0.509016
C	-0.434807	0.240861	0.326905
C	0.154007	-1.077138	-0.046491
O	1.700408	3.103739	0.255484
C	0.522008	3.697233	0.025231
C	-0.438879	2.692319	-0.482354
O	0.354313	4.874426	0.209080
C	-1.645200	3.057114	-0.895965
C	2.859687	1.294448	-0.870833
C	1.190932	-1.754349	2.140533
O	3.688157	-3.417864	-1.360498
O	-1.839040	0.241735	-0.010672
C	-2.695973	-0.143216	0.947857
O	-2.353251	-0.435655	2.067040
C	-4.114337	-0.145465	0.453589
C	-4.385726	-1.146544	-0.678379
C	-4.075454	-2.575329	-0.249761
C	-5.831364	-1.016649	-1.140047
H	3.729932	-2.523126	0.497891
H	2.440143	-1.083331	-1.889708
H	3.816014	0.578810	1.632823
H	5.190737	0.693942	0.557431
H	1.738278	-3.893723	0.548830
H	1.175125	-3.218127	-0.986228
H	4.680107	-0.885891	1.148854
H	0.304111	1.000322	-1.541143
H	-0.341301	0.463706	1.388035
H	0.029254	-1.307936	-1.102112
H	-2.348912	2.355947	-1.322212
H	-1.941181	4.097206	-0.816400
H	2.549086	1.249892	-1.916346
H	1.797273	1.225098	1.005055
H	3.601691	2.091107	-0.775431
H	2.255346	-1.580395	2.325187
H	0.936738	-2.684741	2.657702
H	0.624171	-0.950188	2.606436
H	3.661400	-4.307358	-0.992265
H	-4.752446	-0.365619	1.310504

H	-4.349755	0.865554	0.108777
H	-3.733191	-0.887563	-1.517979
H	-4.286605	-3.275871	-1.061064
H	-3.026936	-2.702502	0.029926
H	-4.685947	-2.867087	0.609775
H	-6.036727	-1.695082	-1.971382
H	-6.057499	-0.000773	-1.473125
H	-6.524658	-1.264587	-0.330942

mPW1PW91 energy = -1155.17070672 a.u.

(2*R*,6*R*,7*S*,8*S*)-**1**, Conf. M

C	3.008943	-2.642105	-0.283658
C	3.062199	-1.251317	-0.823647
C	3.461274	-0.153965	-0.177918
C	4.240888	-0.132469	1.101516
C	0.739883	-1.934914	0.570522
C	1.526533	-3.040357	-0.084896
C	1.721433	1.597418	0.120878
C	0.354573	1.371158	-0.582437
C	-0.473235	0.265101	0.115391
C	0.100077	-1.070300	-0.218586
O	1.793979	3.009662	0.430832
C	0.678071	3.672141	0.099480
C	-0.261394	2.741629	-0.565975
O	0.543849	4.846408	0.324357
C	-1.397263	3.186147	-1.087343
C	2.993886	1.199654	-0.648395
C	0.866875	-1.821420	2.055204
O	3.556323	-3.614899	-1.171562
O	-1.813594	0.336362	-0.417156
C	-2.834716	0.165240	0.438488
O	-2.688002	-0.022119	1.621306
C	-4.164096	0.242912	-0.258194
C	-4.849595	-1.124032	-0.455390
C	-4.006374	-2.071533	-1.299875
C	-5.258475	-1.771861	0.860557
H	3.523267	-2.701599	0.679092
H	2.533063	-1.119079	-1.765133
H	5.167395	0.430626	0.948394
H	4.511170	-1.123231	1.462582
H	1.490615	-3.966148	0.494909
H	1.120101	-3.249982	-1.076804
H	3.700490	0.386682	1.898795
H	0.518783	1.058289	-1.616202
H	-0.521533	0.453707	1.185825
H	0.082799	-1.280768	-1.285691
H	-2.080039	2.543478	-1.625162
H	-1.653479	4.234595	-0.983095
H	2.801928	1.206781	-1.722868
H	1.720157	1.095614	1.089703
H	3.750214	1.959862	-0.438482
H	0.523871	-2.747425	2.526709
H	0.289774	-0.999648	2.475945
H	1.912998	-1.696055	2.350515
H	4.483215	-3.395947	-1.311079
H	-4.804913	0.878861	0.356105

H	-4.030640	0.723058	-1.227889
H	-5.763888	-0.900484	-1.015401
H	-4.565072	-2.982884	-1.525245
H	-3.714413	-1.616597	-2.249439
H	-3.093629	-2.369464	-0.776568
H	-5.825359	-2.687006	0.672272
H	-5.886263	-1.105747	1.457334
H	-4.387983	-2.035579	1.464329

mPW1PW91 energy = -1155.16980682 a.u.

(2*S*,6*R*,7*S*,8*S*)-**1**, Conf. A

C	-2.903070	-2.733855	-0.046143
C	-3.244296	-1.297638	-0.280227
C	-3.334878	-0.323932	0.628613
C	-3.380239	-0.528692	2.111476
C	-0.517109	-1.971657	-0.159849
C	-1.492636	-3.025414	-0.612088
C	-2.020138	1.450750	-0.586146
C	-0.694439	1.238318	0.187436
C	0.312447	0.402287	-0.635501
C	-0.271983	-0.940818	-0.971588
O	-2.054360	2.849443	-0.956301
C	-1.086569	3.558268	-0.354811
C	-0.241429	2.642652	0.447891
O	-0.987069	4.748875	-0.493494
C	0.708756	3.102305	1.250655
C	-3.299461	1.123938	0.182849
C	-0.020106	-2.113380	1.245069
O	-3.785589	-3.629070	-0.719534
O	1.519797	0.335284	0.142818
C	2.659969	0.113218	-0.531708
O	2.701879	-0.046491	-1.725152
C	3.851790	0.145096	0.383283
C	5.028218	-0.701402	-0.095600
C	6.268428	-0.389305	0.731238
C	4.697587	-2.188107	-0.047471
H	-2.906505	-2.959864	1.023520
H	-3.254539	-1.019450	-1.332827
H	-2.504359	-0.091330	2.601629
H	-3.449461	-1.575351	2.403692
H	-1.573822	-3.028088	-1.701323
H	-1.194830	-4.028442	-0.294387
H	-4.250030	-0.007637	2.524555
H	-0.880913	0.702072	1.117830
H	0.544944	0.930673	-1.561341
H	-0.681732	-0.993028	-1.975147
H	1.311494	2.446362	1.865051
H	0.896577	4.168752	1.304766
H	-3.364495	1.785579	1.050365
H	-2.016406	0.906181	-1.528064
H	-4.149213	1.363376	-0.461896
H	0.529479	-1.245174	1.598863
H	0.645806	-2.980632	1.312474
H	-0.846155	-2.312434	1.932873
H	-4.675736	-3.470160	-0.389434

H	4.148049	1.198986	0.443443
H	3.540022	-0.145680	1.389108
H	5.227838	-0.427823	-1.135798
H	7.123942	-0.975430	0.387922
H	6.539237	0.667424	0.664033
H	6.108959	-0.625663	1.787742
H	5.530538	-2.784706	-0.426596
H	3.819628	-2.427473	-0.651367
H	4.499368	-2.511143	0.979339

mPW1PW91 energy = -1155.17190060 a.u.

(2*S*,6*R*,7*S*,8*S*)-**1**, Conf. B

C	-3.507714	-2.233559	-0.111290
C	-3.529497	-0.753410	-0.316982
C	-3.411186	0.200432	0.609824
C	-3.504640	-0.016564	2.088888
C	-1.010398	-2.000356	-0.167946
C	-2.181013	-2.807963	-0.662155
C	-1.725602	1.654157	-0.564560
C	-0.484721	1.172594	0.227018
C	0.332754	0.141845	-0.584437
C	-0.524647	-1.036877	-0.953573
O	-1.456448	3.023533	-0.945768
C	-0.368482	3.517538	-0.334985
C	0.248055	2.452994	0.492088
O	-0.015645	4.657656	-0.483685
C	1.249951	2.710807	1.321589
C	-3.055243	1.611599	0.186634
C	-0.595243	-2.270253	1.244352
O	-4.547066	-2.904697	-0.820843
O	1.478197	-0.195445	0.216572
C	2.533257	-0.710928	-0.437311
O	2.566058	-0.847459	-1.633777
C	3.618632	-1.124573	0.515808
C	5.014562	-1.150325	-0.099414
C	5.491456	0.252689	-0.454142
C	5.991442	-1.837044	0.846369
H	-3.582382	-2.476532	0.952155
H	-3.474117	-0.461069	-1.364548
H	-3.805036	-1.027246	2.360662
H	-4.237681	0.676588	2.514474
H	-2.238295	-2.769708	-1.752134
H	-2.112083	-3.858042	-0.365592
H	-2.554629	0.204526	2.585858
H	-0.787973	0.693900	1.157656
H	0.696603	0.614324	-1.497990
H	-0.912861	-0.983099	-1.965723
H	1.682639	1.948980	1.956718
H	1.655578	3.714633	1.380396
H	-2.986575	2.263413	1.061324
H	-1.824371	1.110899	-1.501841
H	-3.822098	2.036802	-0.466176
H	-0.169653	-3.277112	1.315818
H	-1.461043	-2.260032	1.912237
H	0.144475	-1.568005	1.619738
H	-5.388049	-2.549744	-0.515399

H	3.586887	-0.480372	1.397737
H	3.340135	-2.128275	0.858895
H	4.957174	-1.736515	-1.021283
H	6.474203	0.220278	-0.930001
H	4.807120	0.749800	-1.144704
H	5.578326	0.874147	0.442628
H	6.991566	-1.880704	0.409051
H	5.680791	-2.860612	1.070655
H	6.070245	-1.296747	1.794729

mPW1PW91 energy = -1155.17188881 a.u.

(2*S*,6*R*,7*S*,8*S*)-**1**, Conf. C

C	-3.136722	-2.658498	0.165232
C	-3.361425	-1.177195	0.136517
C	-3.168858	-0.291905	1.113478
C	-2.903408	-0.648326	2.540903
C	-0.817778	-1.934004	-0.638460
C	-1.622770	-3.006038	0.045477
C	-1.925599	1.472646	-0.184828
C	-0.498923	1.415806	0.420584
C	0.343751	0.301813	-0.235666
C	-0.221319	-1.025801	0.136245
O	-2.098260	2.809856	-0.709251
C	-1.017362	3.580321	-0.528328
C	0.014682	2.809591	0.202951
O	-0.972507	4.717584	-0.918217
C	1.144460	3.383958	0.594525
C	-3.093734	1.178644	0.754659
C	-0.877132	-1.895889	-2.130926
O	-3.802620	-3.291662	-0.925264
O	1.678261	0.424990	0.307446
C	2.692306	0.012261	-0.467875
O	2.544339	-0.408726	-1.588051
C	4.022892	0.168469	0.214579
C	4.926713	-1.055091	0.046125
C	6.334121	-0.743281	0.537016
C	4.349928	-2.269090	0.764393
H	-3.494694	-3.097403	1.102204
H	-3.536314	-0.795015	-0.867615
H	-1.906086	-0.318877	2.850539
H	-2.977809	-1.718235	2.732697
H	-1.555995	-3.964473	-0.475185
H	-1.237787	-3.149278	1.056323
H	-3.613009	-0.135830	3.198451
H	-0.555923	1.201669	1.490312
H	0.394737	0.455505	-1.311923
H	-0.250860	-1.186650	1.211822
H	1.895295	2.855585	1.165841
H	1.329377	4.422290	0.342618
H	-3.000851	1.795419	1.651980
H	-1.993496	0.811268	-1.048816
H	-4.005742	1.494784	0.241791
H	-0.502503	-2.839838	-2.538275
H	-0.289875	-1.088520	-2.565094
H	-1.910718	-1.806888	-2.478233
H	-4.738122	-3.070014	-0.871374

H	4.497201	1.042826	-0.243707
H	3.869807	0.396793	1.270962
H	4.974940	-1.279055	-1.023649
H	6.993038	-1.604221	0.402378
H	6.770878	0.098282	-0.006410
H	6.334969	-0.490490	1.601616
H	4.977392	-3.149498	0.607735
H	3.346562	-2.514086	0.407413
H	4.288952	-2.092731	1.842690

mPW1PW91 energy = -1155.17177404 a.u.

(2*S*,6*R*,7*S*,8*S*)-**1**, Conf. D

C	-3.494096	-2.384165	0.212515
C	-3.541710	-0.892420	0.080004
C	-3.313874	0.031359	1.012908
C	-3.173748	-0.251529	2.474645
C	-1.071516	-1.978464	-0.502631
C	-2.027834	-2.912621	0.189277
C	-1.797989	1.551828	-0.295572
C	-0.436275	1.354922	0.417213
C	0.319578	0.130366	-0.145046
C	-0.401445	-1.110275	0.258036
O	-1.761333	2.866595	-0.898519
C	-0.616740	3.517640	-0.650152
C	0.247751	2.673663	0.206531
O	-0.403809	4.620446	-1.081115
C	1.387633	3.139940	0.698829
C	-3.051711	1.457465	0.571657
C	-1.066566	-2.008190	-1.996494
O	-4.183069	-3.008951	-0.868816
O	1.631094	0.141140	0.460604
C	2.672312	-0.224788	-0.303327
O	2.568807	-0.563502	-1.455363
C	3.967721	-0.106612	0.451064
C	5.056660	-1.064569	-0.024528
C	6.399208	-0.672439	0.579101
C	4.705074	-2.509602	0.306801
H	-3.940973	-2.714642	1.155439
H	-3.609786	-0.563949	-0.955779
H	-3.395887	-1.286773	2.732228
H	-2.159875	-0.026313	2.822123
H	-2.052354	-3.898645	-0.281118
H	-1.709217	-3.045298	1.224387
H	-3.844324	0.393083	3.051704
H	-0.593495	1.180893	1.484048
H	0.438313	0.222654	-1.222659
H	-0.495094	-1.214155	1.336903
H	2.010006	2.558869	1.365658
H	1.712133	4.140764	0.436643
H	-2.944748	2.118995	1.435036
H	-1.891319	0.854001	-1.127451
H	-3.886908	1.836957	-0.022585
H	-0.372809	-1.295061	-2.438955
H	-2.068178	-1.819489	-2.393770
H	-0.787140	-3.007748	-2.343279
H	-5.090441	-2.686585	-0.869501

H	4.296866	0.929919	0.312476
H	3.772336	-0.229765	1.518830
H	5.125816	-0.969182	-1.112095
H	7.191962	-1.341026	0.235722
H	6.679280	0.347171	0.302102
H	6.373103	-0.725091	1.671933
H	5.465671	-3.194203	-0.075905
H	3.748197	-2.802620	-0.130696
H	4.640743	-2.657644	1.389228

mPW1PW91 energy = -1155.17174391 a.u.

H	3.689144	-0.730820	1.600551
H	3.496588	-2.388396	1.062995
H	4.885172	-1.871666	-0.962745
H	6.292647	0.172317	-0.977074
H	4.584856	0.603016	-1.016974
H	5.498819	0.747221	0.489865
H	7.059480	-1.926585	0.251813
H	5.876312	-2.989369	1.017896
H	6.253263	-1.420790	1.737523

mPW1PW91 energy = -1155.17172100 a.u.

(2*S*,6*R*,7*S*,8*S*)-**1**, Conf. E

C	-3.995025	-1.829463	0.168128
C	-3.731685	-0.362680	0.017078
C	-3.346748	0.510561	0.946819
C	-3.310327	0.227524	2.414482
C	-1.516927	-1.938475	-0.458716
C	-2.669629	-2.649280	0.197558
C	-1.505983	1.666395	-0.316120
C	-0.238978	1.210535	0.452443
C	0.261989	-0.156563	-0.063626
C	-0.713247	-1.215027	0.323726
O	-1.180660	2.934532	-0.932016
C	0.067720	3.337496	-0.658048
C	0.711864	2.351807	0.240483
O	0.518918	4.361157	-1.100486
C	1.912543	2.577801	0.756598
C	-2.785326	1.844686	0.498607
C	-1.458264	-1.986482	-1.951085
O	-4.767239	-2.318732	-0.926544
O	1.525294	-0.415526	0.588483
C	2.481325	-1.020876	-0.133644
O	2.368095	-1.283679	-1.304694
C	3.675615	-1.365269	0.711773
C	5.004300	-1.299540	-0.038057
C	5.361344	0.135424	-0.407160
C	6.108200	-1.946227	0.788612
H	-4.526765	-2.042695	1.100981
H	-3.700815	-0.043965	-1.023406
H	-3.844991	1.007777	2.965347
H	-2.281563	0.237574	2.789586
H	-2.880644	-3.611657	-0.275011
H	-2.421614	-2.839412	1.242930
H	-3.753698	-0.733753	2.672953
H	-0.464779	1.102564	1.515542
H	0.434154	-0.109375	-1.136774
H	-0.862181	-1.283183	1.399444
H	2.380811	1.893471	1.450775
H	2.449253	3.480276	0.486140
H	-2.582835	2.492288	1.355393
H	-1.707505	0.985382	-1.143192
H	-3.502822	2.369175	-0.137498
H	-0.605033	-1.452253	-2.366199
H	-2.374626	-1.583174	-2.392195
H	-1.396811	-3.027295	-2.282785
H	-5.585657	-1.812768	-0.962947

(2*S*,6*R*,7*S*,8*S*)-**1**, Conf. F

C	-3.157392	-2.654408	0.154675
C	-3.387026	-1.177075	0.142523
C	-3.159421	-0.300534	1.118355
C	-2.856897	-0.668902	2.535519
C	-0.824194	-1.929798	-0.619003
C	-1.630904	-2.992444	0.074531
C	-1.928920	1.473894	-0.175928
C	-0.498439	1.422095	0.421248
C	0.342020	0.306670	-0.234879
C	-0.220237	-1.019157	0.147070
O	-2.109809	2.811917	-0.696293
C	-1.029058	3.584305	-0.525956
C	0.011511	2.816083	0.196104
O	-0.989583	4.721644	-0.916529
C	1.144131	3.393044	0.575395
C	-3.091886	1.171936	0.767413
C	-0.895673	-1.897975	-2.111215
O	-3.875265	-3.178477	-0.957660
O	1.679307	0.434029	0.300635
C	2.688044	0.003164	-0.471432
O	2.532987	-0.438868	-1.582536
C	4.022456	0.169112	0.201410
C	4.930102	-1.051914	0.037631
C	6.339562	-0.729988	0.516063
C	4.363467	-2.261675	0.771129
H	-3.539062	-3.109441	1.075012
H	-3.605684	-0.792807	-0.851444
H	-3.552628	-0.164441	3.213865
H	-1.853319	-0.341818	2.826584
H	-1.531940	-3.964726	-0.418632
H	-1.266084	-3.108377	1.097075
H	-2.928187	-1.740766	2.718168
H	-0.549387	1.211677	1.492017
H	0.387014	0.455487	-1.312119
H	-0.242450	-1.174218	1.223480
H	1.901656	2.867285	1.140257
H	1.324286	4.431314	0.319799
H	-2.998194	1.785567	1.666843
H	-1.998859	0.813992	-1.040947
H	-4.006698	1.485733	0.258391
H	-1.932498	-1.810520	-2.448912
H	-0.522619	-2.842700	-2.518773
H	-0.312619	-1.091914	-2.553233
H	-3.756889	-4.133208	-0.967558

H	4.490465	1.041921	-0.266353
H	3.874834	0.405871	1.256744
H	4.971913	-1.285641	-1.030318
H	7.001034	-1.589599	0.385481
H	6.769427	0.107966	-0.038295
H	6.346405	-0.466738	1.578095
H	4.992984	-3.141131	0.617332
H	3.358188	-2.513317	0.424294
H	4.310332	-2.076009	1.848288

mPW1PW91 energy = -1155.17129978 a.u.

(2*S*,6*R*,7*S*,8*S*)-**1**, Conf. G

C	-3.520892	-2.371525	0.195264
C	-3.569875	-0.881806	0.082099
C	-3.307481	0.027083	1.018745
C	-3.137940	-0.274008	2.473579
C	-1.082741	-1.971448	-0.482679
C	-2.044111	-2.892635	0.216672
C	-1.798293	1.554768	-0.285469
C	-0.432317	1.359717	0.420224
C	0.319624	0.132225	-0.140802
C	-0.401712	-1.105369	0.270961
O	-1.766280	2.870539	-0.887312
C	-0.619372	3.520594	-0.649614
C	0.251641	2.677131	0.201112
O	-0.408642	4.622752	-1.083623
C	1.395674	3.143448	0.683656
C	-3.048547	1.456136	0.586271
C	-1.087935	-2.003444	-1.976704
O	-4.243957	-2.879524	-0.921102
O	1.633658	0.143810	0.459460
C	2.670285	-0.233950	-0.304715
O	2.560721	-0.585224	-1.452448
C	3.968940	-0.111205	0.443370
C	5.058429	-1.067478	-0.033909
C	6.402096	-0.670234	0.563871
C	4.712253	-2.512620	0.302659
H	-3.997055	-2.717263	1.118741
H	-3.676081	-0.542513	-0.946162
H	-3.351958	-1.313759	2.719911
H	-3.803946	0.357848	3.069919
H	-2.041574	-3.894930	-0.223249
H	-1.746012	-2.993971	1.262269
H	-2.120592	-0.047321	2.808983
H	-0.583304	1.190542	1.488742
H	0.433689	0.220374	-1.219266
H	-0.486807	-1.206080	1.350675
H	2.022635	2.563214	1.346896
H	1.718787	4.143608	0.417245
H	-2.939119	2.113471	1.452600
H	-1.895062	0.857652	-1.117686
H	-3.885790	1.837075	-0.003926
H	-0.382084	-1.304960	-2.423295
H	-2.087599	-1.793362	-2.368071
H	-0.830120	-3.008501	-2.324702
H	-4.249217	-3.839801	-0.862493

H	4.294822	0.925869	0.301230
H	3.778188	-0.231906	1.512310
H	5.123465	-0.974788	-1.121966
H	7.195414	-1.337569	0.219383
H	6.678420	0.349411	0.283294
H	6.379950	-0.720104	1.656921
H	5.473389	-3.196078	-0.081047
H	3.754551	-2.809512	-0.130365
H	4.652650	-2.657744	1.385765

mPW1PW91 energy = -1155.17124314 a.u.

(2*S*,6*R*,7*S*,8*S*)-**1**, Conf. H

C	-4.011440	-1.815780	0.139712
C	-3.751200	-0.348998	0.018480
C	-3.338508	0.498602	0.958263
C	-3.274208	0.183336	2.418592
C	-1.518676	-1.932882	-0.435769
C	-2.675215	-2.627167	0.228255
C	-1.509282	1.674178	-0.300080
C	-0.232650	1.220540	0.454555
C	0.262474	-0.149146	-0.059347
C	-0.712169	-1.203904	0.338874
O	-1.195540	2.947920	-0.911140
C	0.056705	3.349853	-0.656321
C	0.716168	2.360758	0.227382
O	0.500867	4.375570	-1.101447
C	1.926686	2.584877	0.720798
C	-2.783327	1.840263	0.525527
C	-1.461721	-1.992856	-1.927892
O	-4.775801	-2.179281	-1.004928
O	1.529301	-0.406350	0.587469
C	2.473382	-1.034844	-0.129928
O	2.347423	-1.321307	-1.294219
C	3.673179	-1.371392	0.711010
C	4.997506	-1.301243	-0.046563
C	5.344989	0.134635	-0.421010
C	6.109166	-1.940430	0.775459
H	-4.586314	-2.044095	1.043579
H	-3.757182	-0.004788	-1.013476
H	-2.241821	0.202957	2.782680
H	-3.700883	-0.789776	2.661093
H	-2.859321	-3.616791	-0.202009
H	-2.446735	-2.769399	1.286325
H	-3.815692	0.941565	2.993323
H	-0.446188	1.117919	1.520767
H	0.428441	-0.107120	-1.133716
H	-0.858946	-1.262476	1.415198
H	2.408299	1.899274	1.404420
H	2.457860	3.487932	0.441507
H	-2.576950	2.477593	1.389096
H	-1.715413	0.997228	-1.129563
H	-3.505859	2.370224	-0.100090
H	-1.411186	-3.036460	-2.253109
H	-0.601886	-1.471619	-2.345772
H	-2.372942	-1.581038	-2.371543
H	-4.981981	-3.116938	-0.942874

H	3.688948	-0.734071	1.597612
H	3.501357	-2.394585	1.065463
H	4.875730	-1.876235	-0.969150
H	6.271947	0.175022	-0.997711
H	4.561806	0.597541	-1.025924
H	5.485898	0.748843	0.473814
H	7.057755	-1.916010	0.234102
H	5.884317	-2.984583	1.007134
H	6.255777	-1.412925	1.722982

mPW1PW91 energy = -1155.17118952 a.u.

(2*S*,6*R*,7*S*,8*S*)-**1**, Conf. I

C	-3.275665	-2.404634	0.269573
C	-3.357051	-0.910475	0.335617
C	-2.954084	-0.102951	1.315628
C	-2.535936	-0.563008	2.675494
C	-1.023930	-1.841833	-0.807524
C	-1.832778	-2.879770	-0.077062
C	-1.713004	1.601404	-0.058649
C	-0.236870	1.378261	0.358777
C	0.412043	0.236899	-0.453772
C	-0.227523	-1.056454	-0.079550
O	-1.813229	2.974979	-0.502067
C	-0.649803	3.633382	-0.414591
C	0.379110	2.731627	0.152832
O	-0.540650	4.782698	-0.752975
C	1.592836	3.180416	0.443872
C	-2.780563	1.370963	1.009703
C	-1.292372	-1.683875	-2.268822
O	-4.140872	-2.913619	-0.743175
O	1.806300	0.215438	-0.076120
C	2.680655	-0.257173	-0.977890
O	2.365435	-0.608491	-2.087670
C	4.081863	-0.267040	-0.435174
C	4.268387	-1.127685	0.821379
C	3.936255	-2.589583	0.550493
C	5.690520	-0.976090	1.345396
H	-3.543405	-2.860305	1.228350
H	-3.622893	-0.458417	-0.618281
H	-2.705045	-1.628092	2.832197
H	-3.085268	-0.015504	3.448068
H	-1.930583	-3.803596	-0.652758
H	-1.331998	-3.124644	0.861058
H	-1.474504	-0.359581	2.852398
H	-0.185312	1.101672	1.414245
H	0.346844	0.450781	-1.519339
H	-0.119315	-1.290934	0.977338
H	2.346518	2.553899	0.900649
H	1.846146	4.212110	0.226758
H	-2.517759	1.930931	1.910674
H	-1.951442	0.994939	-0.932421
H	-3.712648	1.795752	0.628560
H	-1.103478	-2.630518	-2.783810
H	-0.676705	-0.918269	-2.738252
H	-2.345327	-1.447432	-2.448437
H	-5.033944	-2.606849	-0.554534

H	4.734348	-0.617752	-1.235892
H	4.354877	0.768506	-0.210914
H	3.580774	-0.754205	1.586471
H	4.062097	-3.188381	1.455550
H	2.906692	-2.720221	0.208216
H	4.595377	-3.005036	-0.217438
H	5.834615	-1.565068	2.254181
H	5.922100	0.065565	1.581116
H	6.420820	-1.321675	0.607911

mPW1PW91 energy = -1155.17118681 a.u.

(2*S*,6*R*,7*S*,8*S*)-**1**, Conf. J

C	-2.908977	-2.736642	-0.035345
C	-3.251049	-1.299779	-0.271912
C	-3.335436	-0.322554	0.632474
C	-3.378152	-0.525098	2.115298
C	-0.517394	-1.969407	-0.164395
C	-1.495016	-3.021910	-0.613907
C	-2.020667	1.450106	-0.586087
C	-0.695128	1.238566	0.187685
C	0.312829	0.404925	-0.636385
C	-0.269752	-0.938236	-0.975286
O	-2.054011	2.848147	-0.958991
C	-1.087377	3.557923	-0.356456
C	-0.243819	2.643311	0.449086
O	-0.987506	4.748249	-0.496890
C	0.703611	3.103658	1.254672
C	-3.299956	1.124299	0.183107
C	-0.022911	-2.111536	1.241445
O	-3.856807	-3.645353	-0.584358
O	1.520124	0.337838	0.141893
C	2.659988	0.113815	-0.532350
O	2.701508	-0.047716	-1.725645
C	3.851822	0.145969	0.382544
C	5.027908	-0.701603	-0.095255
C	6.268104	-0.389181	0.731490
C	4.696612	-2.188111	-0.045412
H	-2.905355	-2.957775	1.031040
H	-3.259734	-1.018074	-1.325004
H	-2.505113	-0.081335	2.604818
H	-3.443675	-1.571611	2.408317
H	-1.575309	-3.017235	-1.705014
H	-1.195788	-4.026676	-0.303601
H	-4.251722	-0.009396	2.527017
H	-0.881320	0.701314	1.117493
H	0.545015	0.935270	-1.561225
H	-0.676031	-0.989488	-1.980411
H	1.304717	2.448106	1.871105
H	0.890587	4.170215	1.309445
H	-3.365273	1.787742	1.049169
H	-2.016479	0.904288	-1.527325
H	-4.150059	1.362218	-0.461714
H	0.526348	-1.243458	1.595957
H	0.642783	-2.978855	1.309595
H	-0.849463	-2.310231	1.928561
H	-3.925362	-3.476217	-1.530316

H	4.148564	1.199775	0.441682
H	3.539800	-0.143511	1.388658
H	5.227861	-0.429275	-1.135727
H	7.123409	-0.976054	0.388944
H	6.539368	0.667355	0.663254
H	6.108302	-0.624386	1.788186
H	5.529465	-2.785544	-0.423433
H	3.818744	-2.427808	-0.649326
H	4.497838	-2.509745	0.981716

mPW1PW91 energy = -1155.17095101 a.u.

(2*S*,6*R*,7*S*,8*S*)-**1**, Conf. K

C	-3.514453	-2.234900	-0.102765
C	-3.536347	-0.753575	-0.308156
C	-3.411823	0.201386	0.615411
C	-3.502532	-0.014927	2.094355
C	-1.009996	-1.996415	-0.171809
C	-2.181505	-2.803543	-0.663530
C	-1.727014	1.653062	-0.563158
C	-0.485209	1.173498	0.227815
C	0.334155	0.145312	-0.584997
C	-0.520630	-1.034316	-0.957238
O	-1.457683	3.021256	-0.948720
C	-0.370213	3.517210	-0.338366
C	0.245454	2.455116	0.492502
O	-0.017113	4.656765	-0.490507
C	1.244860	2.715490	1.324188
C	-3.055608	1.611885	0.189683
C	-0.599884	-2.263715	1.242533
O	-4.620974	-2.906272	-0.694380
O	1.479451	-0.191922	0.216263
C	2.533647	-0.709628	-0.437033
O	2.565947	-0.847537	-1.633466
C	3.618344	-1.123936	0.516502
C	5.014301	-1.152891	-0.098456
C	5.493689	0.248883	-0.454696
C	5.989744	-1.840164	0.848421
H	-3.583749	-2.476655	0.956898
H	-3.478347	-0.456061	-1.355632
H	-2.554123	0.213521	2.591107
H	-3.798497	-1.026645	2.366608
H	-2.234892	-2.760478	-1.755289
H	-2.111661	-3.854954	-0.372412
H	-4.241022	0.673154	2.518667
H	-0.787293	0.693752	1.158224
H	0.698163	0.620085	-1.497329
H	-0.903503	-0.982094	-1.971649
H	1.676361	1.955358	1.962180
H	1.649398	3.719794	1.382269
H	-2.985567	2.264673	1.063475
H	-1.826574	1.107375	-1.499009
H	-3.823575	2.036764	-0.462020
H	-0.173986	-3.270193	1.317051
H	-1.467583	-2.252537	1.907744
H	0.138237	-1.560465	1.619095
H	-4.629034	-2.704300	-1.636308

H	3.587579	-0.478586	1.397613
H	3.338043	-2.126687	0.860932
H	4.956142	-1.740084	-1.019659
H	6.476462	0.214207	-0.930345
H	4.810289	0.746437	-1.145883
H	5.581482	0.871159	0.441399
H	6.989954	-1.885946	0.411527
H	5.677344	-2.862965	1.073782
H	6.069055	-1.298946	1.796203

mPW1PW91 energy = -1155.17094239 a.u.

(2*S*,6*R*,7*S*,8*S*)-**1**, Conf. L

C	-2.745405	-2.759022	-0.070465
C	-3.130442	-1.320190	-0.197848
C	-3.160289	-0.395298	0.764696
C	-3.078746	-0.672284	2.234238
C	-0.395427	-1.927485	-0.333025
C	-1.377713	-2.985510	-0.758118
C	-1.974292	1.452310	-0.465086
C	-0.586933	1.236669	0.190210
C	0.361757	0.476144	-0.764242
C	-0.217034	-0.864834	-1.121317
O	-2.057822	2.861973	-0.780371
C	-1.059611	3.566499	-0.225079
C	-0.147169	2.638049	0.483996
O	-0.987520	4.762527	-0.328692
C	0.838019	3.084508	1.251101
C	-3.182988	1.072827	0.388679
C	0.191708	-2.102693	1.032388
O	-3.659856	-3.636403	-0.724153
O	1.644547	0.402177	-0.116594
C	2.725174	0.395488	-0.916526
O	2.658125	0.444078	-2.119471
C	4.000643	0.330916	-0.126645
C	4.200559	-0.989223	0.631945
C	4.220015	-2.183164	-0.314252
C	5.480143	-0.918610	1.455366
H	-2.662804	-3.043416	0.981800
H	-3.233101	-0.989034	-1.229993
H	-3.092861	-1.733286	2.478752
H	-2.179830	-0.229417	2.675061
H	-1.544732	-2.939715	-1.836397
H	-1.032222	-3.993513	-0.513332
H	-3.927015	-0.201182	2.741778
H	-0.684151	0.648689	1.102172
H	0.489242	1.059365	-1.676702
H	-0.686158	-0.888561	-2.099620
H	1.484798	2.416736	1.805144
H	1.008568	4.151434	1.341480
H	-3.187525	1.692500	1.288936
H	-2.036496	0.940446	-1.422965
H	-4.084680	1.326153	-0.175186
H	0.875837	-2.958080	1.030130
H	-0.585077	-2.338742	1.764552
H	0.746543	-1.234051	1.377085
H	-4.525339	-3.513601	-0.321138

H	4.820416	0.486468	-0.829067
H	4.000115	1.161344	0.584298
H	3.358560	-1.108870	1.320586
H	4.353960	-3.114434	0.241067
H	3.291931	-2.269631	-0.884258
H	5.042124	-2.102136	-1.031454
H	5.629796	-1.841335	2.020857
H	5.454573	-0.090784	2.168394
H	6.353909	-0.778754	0.812176

mPW1PW91 energy = -1155.17080258 a.u.

(2*S*,6*R*,7*S*,8*S*)-**1**, Conf. M

C	-2.919994	-2.728558	-0.040915
C	-3.262308	-1.295484	-0.273267
C	-3.332193	-0.321735	0.635740
C	-3.365850	-0.525062	2.118874
C	-0.522259	-1.966550	-0.165618
C	-1.504327	-3.012257	-0.620113
C	-2.020215	1.452618	-0.583968
C	-0.692618	1.241948	0.186756
C	0.313033	0.406213	-0.637449
C	-0.274366	-0.934820	-0.975613
O	-2.055519	2.851217	-0.955557
C	-1.087019	3.560929	-0.356864
C	-0.240023	2.646594	0.445531
O	-0.987631	4.751433	-0.497171
C	0.710927	3.107433	1.246698
C	-3.297089	1.125705	0.188311
C	-0.024175	-2.113956	1.238431
O	-3.888474	-3.546931	-0.689272
O	1.519995	0.335852	0.141412
C	2.659931	0.110760	-0.532282
O	2.702065	-0.051228	-1.725472
C	3.851403	0.142519	0.383195
C	5.027915	-0.704426	-0.094523
C	6.267762	-0.391961	0.732714
C	4.697152	-2.191080	-0.045495
H	-2.915679	-2.951344	1.029222
H	-3.286933	-1.018037	-1.325526
H	-2.486019	-0.089427	2.603495
H	-3.437937	-1.571392	2.411208
H	-1.592678	-3.003959	-1.708747
H	-1.199537	-4.018425	-0.312443
H	-4.232245	-0.002252	2.536768
H	-0.876475	0.705672	1.117585
H	0.546916	0.935430	-1.562441
H	-0.685574	-0.983692	-1.978734
H	1.314742	2.452332	1.860962
H	0.898132	4.174035	1.299828
H	-3.360473	1.788356	1.055158
H	-2.018849	0.907204	-1.525449
H	-4.148636	1.363344	-0.454610
H	0.639234	-2.983420	1.303237
H	-0.849821	-2.310780	1.927253
H	0.528862	-1.248424	1.593367
H	-3.607575	-4.464903	-0.612272

H	4.147830	1.196379	0.443073
H	3.539026	-0.147512	1.389062
H	5.228103	-0.431637	-1.134814
H	7.123412	-0.978364	0.390208
H	6.538683	0.664691	0.664959
H	6.107735	-0.627566	1.789301
H	5.530228	-2.788026	-0.423818
H	3.819439	-2.430673	-0.649687
H	4.498481	-2.513338	0.981486

mPW1PW91 energy = -1155.17074217 a.u.

(2*S*,6*R*,7*S*,8*S*)-**1**, Conf. N

C	-3.292559	-2.403016	0.259732
C	-3.378604	-0.913615	0.349573
C	-2.939229	-0.120910	1.324525
C	-2.480639	-0.597469	2.665615
C	-1.024252	-1.836792	-0.789328
C	-1.830594	-2.868477	-0.050213
C	-1.717523	1.600177	-0.043012
C	-0.235837	1.386290	0.360436
C	0.411443	0.244325	-0.452154
C	-0.221905	-1.049575	-0.069691
O	-1.831250	2.975402	-0.478953
C	-0.670039	3.638914	-0.408565
C	0.371694	2.741741	0.142798
O	-0.570783	4.789108	-0.747385
C	1.588043	3.196131	0.413438
C	-2.776063	1.356453	1.031413
C	-1.305590	-1.680539	-2.248539
O	-4.199949	-2.794491	-0.764935
O	1.807853	0.227856	-0.081606
C	2.677719	-0.254751	-0.982194
O	2.357515	-0.616632	-2.087210
C	4.080626	-0.262650	-0.443917
C	4.270200	-1.123651	0.812141
C	3.935549	-2.585225	0.542598
C	5.694114	-0.973671	1.331702
H	-3.587535	-2.876857	1.202089
H	-3.690113	-0.452262	-0.585088
H	-1.417960	-0.384526	2.821644
H	-2.636074	-1.666593	2.809588
H	-1.893723	-3.809479	-0.605998
H	-1.345170	-3.086071	0.903383
H	-3.018742	-0.068916	3.459124
H	-0.172334	1.115430	1.416753
H	0.339918	0.454818	-1.517974
H	-0.103870	-1.281444	0.986548
H	2.352001	2.573670	0.858521
H	1.832858	4.228776	0.191131
H	-2.510055	1.910854	1.934941
H	-1.959234	0.995923	-0.917541
H	-3.712823	1.778433	0.658999
H	-0.690815	-0.918224	-2.724290
H	-2.359125	-1.439516	-2.418310
H	-1.124143	-2.628241	-2.764678
H	-4.169457	-3.752980	-0.842442

H	4.731372	-0.613192	-1.246122
H	4.353576	0.772813	-0.219590
H	3.585397	-0.749251	1.579314
H	4.064195	-3.184024	1.447272
H	2.904516	-2.714683	0.204282
H	4.591321	-3.001447	-0.227780
H	5.840216	-1.562440	2.240307
H	5.927639	0.067767	1.566353
H	6.421745	-1.320417	0.592129

mPW1PW91 energy = -1155.17072828 a.u.

(2*S*,6*R*,7*S*,8*S*)-**1**, Conf. O

C	-3.520162	-2.226826	-0.105908
C	-3.548050	-0.749532	-0.307519
C	-3.405288	0.198318	0.620431
C	-3.476405	-0.023227	2.099890
C	-1.011209	-1.992385	-0.177506
C	-2.186488	-2.790739	-0.673678
C	-1.727957	1.657610	-0.559369
C	-0.482152	1.179929	0.226742
C	0.333953	0.150175	-0.586850
C	-0.525510	-1.025639	-0.959329
O	-1.464026	3.027939	-0.941828
C	-0.374522	3.524768	-0.336791
C	0.249326	2.461968	0.487269
O	-0.024954	4.665723	-0.487424
C	1.255938	2.722315	1.310225
C	-3.054375	1.610539	0.196545
C	-0.593217	-2.271931	1.232170
O	-4.626983	-2.807226	-0.788843
O	1.478046	-0.191213	0.214630
C	2.531195	-0.711501	-0.438215
O	2.564019	-0.849579	-1.634564
C	3.614495	-1.128677	0.515815
C	5.011020	-1.157702	-0.097910
C	5.491666	0.244288	-0.451551
C	5.985203	-1.847095	0.848738
H	-3.583034	-2.470438	0.957878
H	-3.514576	-0.453324	-1.354730
H	-2.519854	0.195708	2.585208
H	-3.776235	-1.033888	2.372015
H	-2.250512	-2.738035	-1.762725
H	-2.109756	-3.845732	-0.389488
H	-4.203606	0.668959	2.536773
H	-0.780122	0.701437	1.159200
H	0.699584	0.623919	-1.499027
H	-0.916608	-0.965766	-1.970024
H	1.694394	1.961726	1.942871
H	1.659607	3.727097	1.366056
H	-2.984995	2.262635	1.070931
H	-1.829640	1.113950	-1.496032
H	-3.825033	2.032461	-0.453787
H	-0.168165	-3.279552	1.296834
H	-1.457031	-2.263960	1.902460
H	0.148082	-1.572731	1.610002
H	-4.551001	-3.765083	-0.724259

H	3.583591	-0.485000	1.398132
H	3.333088	-2.131919	0.857908
H	4.953230	-1.743397	-1.020079
H	6.474804	0.209647	-0.926428
H	4.809204	0.743396	-1.142537
H	5.579144	0.865110	0.445584
H	6.985689	-1.893101	0.412502
H	5.671845	-2.869970	1.072493
H	6.064316	-1.307229	1.797306

mPW1PW91 energy = -1155.17072085 a.u.

(2*S*,6*R*,7*S*,8*S*)-**1**, Conf. P

C	-2.769492	-2.703094	0.326456
C	-3.254947	-1.294720	0.200983
C	-3.136684	-0.327335	1.113774
C	-2.811649	-0.548532	2.558317
C	-0.800485	-1.837938	-1.107556
C	-1.722038	-2.989988	-0.769918
C	-2.010621	1.427713	-0.256953
C	-0.597744	1.361094	0.378597
C	0.294823	0.299261	-0.302510
C	-0.314492	-1.052480	-0.145147
O	-2.172658	2.771211	-0.766272
C	-1.110034	3.549076	-0.517341
C	-0.099782	2.769548	0.235664
O	-1.062432	4.696957	-0.873957
C	1.002768	3.343446	0.698804
C	-3.197156	1.113532	0.656135
C	-0.588047	-1.612239	-2.571553
O	-3.793889	-3.680768	0.135883
O	1.567075	0.357741	0.382543
C	2.658870	0.036109	-0.328286
O	2.626874	-0.268154	-1.494288
C	3.910780	0.146704	0.497551
C	4.972901	-0.893023	0.143321
C	6.286523	-0.557657	0.838061
C	4.507525	-2.300516	0.496779
H	-2.313312	-2.853068	1.308369
H	-3.547054	-1.009491	-0.809114
H	-1.858054	-0.091678	2.840538
H	-2.780262	-1.601096	2.835726
H	-2.283394	-3.252253	-1.670774
H	-1.155214	-3.885216	-0.496089
H	-3.572823	-0.064404	3.178859
H	-0.669406	1.097289	1.434587
H	0.453982	0.563679	-1.346028
H	-0.505478	-1.320491	0.890382
H	1.732645	2.802820	1.285965
H	1.186417	4.392001	0.492782
H	-3.193098	1.799220	1.506928
H	-2.059867	0.774727	-1.128025
H	-4.109076	1.308743	0.085987
H	-0.272824	-2.547092	-3.045205
H	0.169721	-0.861170	-2.786344
H	-1.522667	-1.320925	-3.062798
H	-4.486931	-3.506792	0.780712

H	4.302530	1.153712	0.313701
H	3.651354	0.097098	1.557176
H	5.130756	-0.845114	-0.938139
H	7.061625	-1.282017	0.577542
H	6.648427	0.433744	0.554132
H	6.173572	-0.572493	1.926408
H	5.258985	-3.041720	0.215100
H	3.579796	-2.561167	-0.017722
H	4.333780	-2.396509	1.572989

mPW1PW91 energy = -1155.17071723 a.u.

(2*S*,6*R*,7*S*,8*S*)-**1**, Conf. Q

C	-3.510123	-2.107799	0.344700
C	-3.605889	-0.628169	0.151825
C	-3.271075	0.315205	1.035284
C	-3.071321	0.084732	2.500495
C	-1.322882	-1.851814	-1.012276
C	-2.531395	-2.703443	-0.689001
C	-1.667951	1.652020	-0.331787
C	-0.352480	1.247734	0.382472
C	0.257947	-0.038585	-0.218566
C	-0.692087	-1.175470	-0.050912
O	-1.447649	2.966551	-0.891754
C	-0.230777	3.451625	-0.607354
C	0.503141	2.470366	0.226018
O	0.132901	4.530142	-0.996641
C	1.695195	2.756415	0.732160
C	-2.933101	1.699868	0.527453
C	-0.990347	-1.760707	-2.468190
O	-4.745001	-2.793206	0.129447
O	1.469916	-0.289931	0.528640
C	2.474218	-0.897408	-0.123283
O	2.442717	-1.156875	-1.299953
C	3.601277	-1.245966	0.807983
C	4.974428	-1.279202	0.142458
C	5.417671	0.115630	-0.281689
C	5.993927	-1.919562	1.075819
H	-3.150149	-2.330662	1.352581
H	-3.778995	-0.321456	-0.879120
H	-3.318861	-0.928251	2.814875
H	-3.710034	0.772180	3.064732
H	-3.104484	-2.843846	-1.609541
H	-2.236306	-3.705348	-0.362324
H	-2.045726	0.301107	2.815394
H	-0.539885	1.055208	1.439807
H	0.525188	0.129139	-1.259858
H	-0.994295	-1.334241	0.980258
H	2.229486	2.070304	1.375266
H	2.156862	3.710545	0.504339
H	-2.782734	2.397834	1.354892
H	-1.848500	0.995621	-1.182634
H	-3.737583	2.099552	-0.094975
H	-1.794246	-1.265892	-3.023548
H	-0.905403	-2.767869	-2.888077
H	-0.055043	-1.240085	-2.664475
H	-5.389810	-2.438107	0.749578

H	3.590805	-0.562716	1.660172
H	3.352912	-2.237282	1.205740
H	4.890828	-1.899383	-0.754827
H	6.382987	0.079413	-0.791763
H	4.701605	0.577031	-0.965397
H	5.526658	0.772133	0.587236
H	6.979136	-1.963380	0.605966
H	5.706338	-2.939669	1.342609
H	6.095924	-1.348138	2.003525

mPW1PW91 energy = -1155.17066748 a.u.

(2*S*,6*R*,7*S*,8*S*)-**1**, Conf. R

C	-3.806287	-1.705737	0.058637
C	-3.574878	-0.242364	-0.136856
C	-3.200537	0.651657	0.781359
C	-3.203600	0.422504	2.261390
C	-1.327144	-1.935595	-0.244678
C	-2.669498	-2.500191	-0.626575
C	-1.382884	1.798725	-0.530442
C	-0.192267	1.066303	0.138638
C	0.342134	-0.064947	-0.769527
C	-0.750293	-1.055572	-1.065930
O	-0.892411	3.105869	-0.910897
C	0.316965	3.366160	-0.391298
C	0.784819	2.179688	0.363715
O	0.866947	4.423443	-0.553280
C	1.872569	2.223832	1.120410
C	-2.627148	1.982328	0.336968
C	-0.828667	-2.321467	1.112961
O	-5.016759	-2.159907	-0.543210
O	1.481900	-0.638920	-0.105802
C	2.339796	-1.318901	-0.885571
O	2.206764	-1.419133	-2.079819
C	3.453593	-1.937789	-0.090404
C	4.348076	-0.933260	0.647323
C	5.412020	-1.680533	1.440970
C	4.982012	0.065230	-0.313078
H	-3.817281	-1.953672	1.123371
H	-3.560474	0.059544	-1.182927
H	-3.658314	-0.522883	2.552809
H	-3.758923	1.227287	2.754126
H	-2.823032	-2.422063	-1.704976
H	-2.769553	-3.552732	-0.347936
H	-2.190004	0.458073	2.673258
H	-0.502642	0.616530	1.081195
H	0.691158	0.368382	-1.707909
H	-1.217854	-0.904817	-2.033691
H	2.195926	1.376948	1.710970
H	2.462170	3.132609	1.164829
H	-2.362334	2.588792	1.206980
H	-1.661104	1.315589	-1.464673
H	-3.356028	2.557321	-0.240285
H	-0.553101	-3.381869	1.112717
H	-1.616755	-2.214322	1.863401
H	0.040600	-1.752542	1.432821
H	-5.744374	-1.668061	-0.149181

H	3.001309	-2.622352	0.633257
H	4.048599	-2.530277	-0.786829
H	3.719653	-0.382866	1.353806
H	6.041446	-0.983855	1.999601
H	4.966954	-2.376162	2.156661
H	6.063738	-2.256178	0.777230
H	5.616411	0.772205	0.226505
H	4.233133	0.645743	-0.857271
H	5.606459	-0.445593	-1.051867

mPW1PW91 energy = -1155.17063822 a.u.

(2*S*,6*R*,7*S*,8*S*)-**1**, Conf. S

C	-3.838819	-1.709650	0.009910
C	-3.593821	-0.255964	-0.236681
C	-3.275192	0.681642	0.658643
C	-3.367936	0.525811	2.144874
C	-1.347364	-1.949890	-0.154947
C	-2.668953	-2.533019	-0.578599
C	-1.385293	1.769484	-0.596292
C	-0.243333	1.056712	0.170053
C	0.344501	-0.098943	-0.671262
C	-0.727476	-1.107043	-0.983500
O	-0.859109	3.056780	-0.997414
C	0.313208	3.332918	-0.405043
C	0.717674	2.176186	0.428783
O	0.881341	4.379751	-0.571709
C	1.738629	2.247246	1.271679
C	-2.678353	1.992352	0.186383
C	-0.916363	-2.274320	1.241481
O	-5.016193	-2.188753	-0.637155
O	1.458872	-0.628713	0.067415
C	2.337631	-1.365335	-0.633428
O	2.214824	-1.591556	-1.811368
C	3.474572	-1.839786	0.223508
C	4.549924	-0.764834	0.458637
C	5.615391	-1.313270	1.399368
C	5.169291	-0.279988	-0.845653
H	-3.908753	-1.912431	1.081921
H	-3.517577	-0.002393	-1.292847
H	-2.385032	0.613625	2.618624
H	-3.816269	-0.416192	2.455879
H	-2.766648	-2.504813	-1.665961
H	-2.784086	-3.571901	-0.257632
H	-3.975209	1.337938	2.558183
H	-0.615489	0.632307	1.101941
H	0.734517	0.303483	-1.607747
H	-1.141121	-1.000477	-1.981214
H	2.006901	1.421337	1.917505
H	2.326224	3.156285	1.333923
H	-2.466084	2.640091	1.040705
H	-1.612866	1.251222	-1.525394
H	-3.372116	2.536853	-0.459623
H	-0.653885	-3.336036	1.304782
H	-1.735287	-2.122799	1.949992
H	-0.054924	-1.700241	1.573287
H	-5.763000	-1.682058	-0.302320

H	3.075440	-2.166659	1.185927
H	3.920806	-2.700729	-0.276780
H	4.069125	0.088714	0.947259
H	6.374661	-0.556675	1.610653
H	5.186028	-1.631404	2.352663
H	6.121646	-2.175851	0.956313
H	5.935898	0.473063	-0.649076
H	4.429107	0.169022	-1.511006
H	5.642606	-1.105211	-1.385569

mPW1PW91 energy = -1155.17050890 a.u.

(2*S*,6*R*,7*S*,8*S*)-**1**, Conf. T

C	-4.089753	-1.506901	0.261409
C	-3.690308	-0.077099	0.060684
C	-3.218853	0.787376	0.958271
C	-3.206138	0.557063	2.435514
C	-1.648930	-1.872593	-0.409102
C	-2.845617	-2.444262	0.301725
C	-1.293057	1.707166	-0.366905
C	-0.066718	1.148936	0.399376
C	0.303755	-0.257612	-0.120387
C	-0.751261	-1.212982	0.325562
O	-0.848140	2.911657	-1.033595
C	0.425956	3.217995	-0.751780
C	0.970020	2.212110	0.189228
O	0.967745	4.184837	-1.219677
C	2.157749	2.370694	0.757075
C	-2.531939	2.043689	0.460785
C	-1.639145	-1.970118	-1.899966
O	-4.918734	-1.954524	-0.809352
O	1.575232	-0.608370	0.468841
C	2.290683	-1.546166	-0.174313
O	1.938429	-2.048239	-1.212892
C	3.562715	-1.877194	0.551044
C	4.646446	-0.793018	0.437222
C	5.870291	-1.216104	1.239191
C	5.014609	-0.502275	-1.012138
H	-4.625816	-1.640915	1.206284
H	-3.635456	0.200853	-0.990262
H	-2.180704	0.509006	2.816779
H	-3.718955	-0.359023	2.727732
H	-3.155026	-3.403300	-0.121123
H	-2.591437	-2.609238	1.350054
H	-3.685449	1.394124	2.953436
H	-0.296953	1.057497	1.462834
H	0.424160	-0.236244	-1.202266
H	-0.872576	-1.235415	1.406748
H	2.541133	1.673491	1.489845
H	2.769541	3.226712	0.494993
H	-2.247573	2.690945	1.294168
H	-1.580191	1.024115	-1.166083
H	-3.202387	2.622300	-0.179880
H	-1.696189	-3.021148	-2.198766
H	-0.744584	-1.543950	-2.350776
H	-2.519685	-1.484574	-2.331045
H	-5.687948	-1.376854	-0.852587

H	3.326088	-2.040772	1.605157
H	3.935009	-2.813681	0.132883
H	4.249434	0.125342	0.880604
H	6.640966	-0.442292	1.210176
H	5.620357	-1.400430	2.286952
H	6.306223	-2.133845	0.833650
H	5.801187	0.254163	-1.063118
H	4.163256	-0.129651	-1.586093
H	5.384283	-1.402346	-1.511861

mPW1PW91 energy = -1155.17023438 a.u.

(2*S*,6*R*,7*S*,8*S*)-**1**, Conf. U

C	-3.142946	-2.672167	0.171499
C	-3.383502	-1.192556	0.153064
C	-3.146060	-0.310133	1.122205
C	-2.821423	-0.673890	2.535791
C	-0.828087	-1.910119	-0.653695
C	-1.615655	-2.997005	0.025249
C	-1.924695	1.470364	-0.170842
C	-0.494583	1.419338	0.426673
C	0.349300	0.313644	-0.241566
C	-0.208017	-1.019426	0.122465
O	-2.107825	2.808750	-0.688273
C	-1.029801	3.584409	-0.510962
C	0.010751	2.816450	0.211078
O	-0.993113	4.723098	-0.897271
C	1.140766	3.394412	0.596533
C	-3.088417	1.162166	0.769581
C	-0.926392	-1.824626	-2.144102
O	-3.924976	-3.337444	-0.813047
O	1.685179	0.436668	0.295792
C	2.694227	0.012121	-0.479996
O	2.538883	-0.415519	-1.596695
C	4.027795	0.164249	0.197148
C	4.921516	-1.068213	0.038711
C	6.331416	-0.763560	0.526954
C	4.335650	-2.271679	0.767343
H	-3.472917	-3.113290	1.112591
H	-3.616545	-0.801351	-0.837221
H	-3.513688	-0.175000	3.221701
H	-1.817966	-0.335143	2.812888
H	-1.542241	-3.950355	-0.504296
H	-1.215268	-3.146415	1.029722
H	-2.878316	-1.745711	2.722880
H	-0.544006	1.200008	1.495590
H	0.393709	0.476778	-1.317012
H	-0.206979	-1.200527	1.194955
H	1.898137	2.867327	1.160396
H	1.319161	4.434465	0.347070
H	-3.001415	1.777706	1.668334
H	-1.991335	0.812254	-1.037495
H	-4.003949	1.468919	0.257571
H	-0.246794	-1.090168	-2.573724
H	-1.943273	-1.567358	-2.463239
H	-0.697346	-2.795931	-2.591002
H	-3.726124	-2.956458	-1.674567

H	4.508084	1.030063	-0.270941
H	3.879261	0.404046	1.251606
H	4.967809	-1.301516	-1.029185
H	6.983137	-1.631060	0.399776
H	6.775121	0.069727	-0.023493
H	6.334217	-0.501773	1.589358
H	4.957054	-3.157779	0.618884
H	3.330831	-2.513044	0.411986
H	4.275391	-2.085500	1.844025

mPW1PW91 energy = -1155.17002895 a.u.

(2*S*,6*R*,7*S*,8*S*)-**1**, Conf. V

C	-4.014596	-1.819689	0.165853
C	-3.750348	-0.352001	0.024973
C	-3.336154	0.505449	0.955997
C	-3.269424	0.200252	2.418362
C	-1.521353	-1.931432	-0.463813
C	-2.677251	-2.640317	0.187906
C	-1.505230	1.670533	-0.307247
C	-0.234290	1.212239	0.453279
C	0.262416	-0.155010	-0.065790
C	-0.716097	-1.211199	0.320002
O	-1.182915	2.940285	-0.921104
C	0.068023	3.340932	-0.654850
C	0.716926	2.352382	0.237071
O	0.517731	4.364732	-1.098302
C	1.921163	2.575733	0.745976
C	-2.780977	1.843913	0.513915
C	-1.450511	-1.974026	-1.957339
O	-4.906757	-2.282027	-0.841304
O	1.524670	-0.418312	0.585896
C	2.477457	-1.030000	-0.135260
O	2.361150	-1.296890	-1.305173
C	3.672085	-1.374807	0.709323
C	5.000843	-1.302818	-0.040057
C	5.354108	0.134489	-0.403607
C	6.106172	-1.949535	0.784621
H	-4.535427	-2.035843	1.099238
H	-3.752464	-0.005712	-1.008481
H	-2.235731	0.217936	2.778668
H	-3.698621	-0.769382	2.669582
H	-2.887768	-3.603842	-0.282887
H	-2.428315	-2.832367	1.233276
H	-3.805863	0.964908	2.989057
H	-0.453999	1.103909	1.517483
H	0.434480	-0.106334	-1.139014
H	-0.865562	-1.281443	1.395265
H	2.392954	1.889522	1.435898
H	2.457082	3.478255	0.474137
H	-2.575978	2.487495	1.373075
H	-1.711195	0.991778	-1.135321
H	-3.502518	2.369563	-0.116507
H	-1.452571	-3.013162	-2.298613
H	-0.559519	-1.492499	-2.357315
H	-2.325183	-1.498260	-2.414885
H	-4.541318	-2.057155	-1.703279

H	3.683935	-0.743773	1.600501
H	3.495613	-2.399841	1.056222
H	4.883342	-1.871925	-0.966828
H	6.285021	0.176011	-0.973822
H	4.576178	0.602828	-1.011074
H	5.490547	0.743002	0.495790
H	7.057658	-1.925045	0.248409
H	5.877188	-2.994184	1.009859
H	6.249235	-1.427275	1.735571

mPW1PW91 energy = -1155.16997316 a.u.

(2*S*,6*R*,7*S*,8*S*)-**1**, Conf. W

C	-3.534287	-2.367884	0.214181
C	-3.578657	-0.875792	0.084971
C	-3.307662	0.039581	1.013723
C	-3.133955	-0.255082	2.469507
C	-1.096516	-1.962117	-0.501581
C	-2.056382	-2.895874	0.183951
C	-1.787236	1.555288	-0.290065
C	-0.426495	1.352770	0.422969
C	0.323286	0.124883	-0.140359
C	-0.404533	-1.112679	0.260548
O	-1.744019	2.869102	-0.894483
C	-0.596137	3.514915	-0.646895
C	0.263839	2.667980	0.211349
O	-0.377486	4.615754	-1.079842
C	1.405080	3.129420	0.705015
C	-3.043119	1.465280	0.574502
C	-1.108299	-1.959498	-1.997178
O	-4.344637	-2.996111	-0.771609
O	1.634305	0.127785	0.464628
C	2.672551	-0.246992	-0.299435
O	2.564645	-0.590942	-1.449534
C	3.969271	-0.129935	0.452510
C	5.063440	-1.075753	-0.034803
C	6.404575	-0.679623	0.569504
C	4.723120	-2.526465	0.283366
H	-3.967934	-2.694991	1.159465
H	-3.686149	-0.532079	-0.944046
H	-3.797777	0.380639	3.064065
H	-2.115637	-0.027199	2.800746
H	-2.080744	-3.881203	-0.288451
H	-1.736530	-3.033758	1.218617
H	-3.347244	-1.293035	2.723397
H	-0.583217	1.180157	1.489903
H	0.442231	0.218903	-1.217871
H	-0.481413	-1.229942	1.339140
H	2.023570	2.546134	1.373549
H	1.734570	4.128557	0.442649
H	-2.937001	2.127221	1.437650
H	-1.882840	0.856832	-1.121382
H	-3.875953	1.845062	-0.022631
H	-0.350765	-1.308525	-2.430850
H	-2.084199	-1.646281	-2.384672
H	-0.937540	-2.972284	-2.373138
H	-4.083560	-2.672425	-1.640073

H	4.291428	0.910255	0.324936
H	3.776476	-0.264909	1.519372
H	5.129456	-0.969513	-1.121569
H	7.201425	-1.338862	0.217695
H	6.676359	0.344713	0.301861
H	6.381498	-0.743141	1.661794
H	5.488486	-3.201704	-0.106400
H	3.768170	-2.822608	-0.156119
H	4.661059	-2.685256	1.364414

mPW1PW91 energy = -1155.16996773 a.u.

(2*S*,6*R*,7*S*,8*S*)-**1**, Conf. X

C	2.679065	-2.648227	-0.322363
C	3.132661	-1.224962	-0.377841
C	2.840502	-0.331124	-1.325573
C	2.298620	-0.661808	-2.680973
C	0.910679	-1.708605	1.314896
C	1.806035	-2.867170	0.932018
C	1.880616	1.504994	0.067811
C	0.392236	1.366480	-0.346658
C	-0.362730	0.332144	0.518756
C	0.266902	-1.011606	0.377312
O	2.080183	2.887271	0.441817
C	0.968638	3.623713	0.307882
C	-0.122790	2.770488	-0.218230
O	0.942186	4.794284	0.583748
C	-1.304356	3.287932	-0.527251
C	2.927542	1.140568	-0.986560
C	0.910293	-1.371570	2.772442
O	3.751167	-3.585159	-0.202768
O	-1.714211	0.310136	0.004645
C	-2.695526	0.006769	0.868538
O	-2.506673	-0.202943	2.041085
C	-4.037408	-0.014535	0.192390
C	-4.168909	-1.065686	-0.917873
C	-3.970416	-2.476193	-0.377745
C	-5.523422	-0.925785	-1.600326
H	2.095151	-2.888546	-1.214623
H	3.572014	-0.857748	0.548996
H	2.935872	-0.207245	-3.446412
H	1.298412	-0.244971	-2.835645
H	2.496881	-3.052681	1.758974
H	1.230702	-3.789637	0.807013
H	2.256460	-1.731522	-2.880105
H	0.318872	1.034544	-1.383792
H	-0.396496	0.667211	1.553465
H	0.315692	-1.359162	-0.650767
H	-2.103279	2.693976	-0.949788
H	-1.483562	4.344192	-0.360439
H	2.776414	1.760490	-1.873640
H	2.075271	0.925482	0.970125
H	3.907993	1.393553	-0.575449
H	0.179177	-0.611312	3.040378
H	1.901842	-1.037011	3.095968
H	0.686322	-2.269525	3.356649
H	4.327372	-3.468423	-0.964915

H	-4.786096	-0.186356	0.967062
H	-4.211914	0.980113	-0.228153
H	-3.389092	-0.867328	-1.659661
H	-4.047480	-3.213427	-1.180416
H	-2.991280	-2.601358	0.090943
H	-4.729766	-2.717849	0.371646
H	-5.627686	-1.649317	-2.412267
H	-5.658966	0.072586	-2.023782
H	-6.339437	-1.102342	-0.893474

mPW1PW91 energy = -1155.16994486 a.u.

(2*S*,6*R*,7*S*,8*S*)-**1**, Conf. Y

C	-2.753639	-2.762941	-0.060562
C	-3.137454	-1.322496	-0.187020
C	-3.159511	-0.395225	0.772237
C	-3.072573	-0.671532	2.241182
C	-0.397432	-1.925062	-0.332033
C	-1.380227	-2.983405	-0.753995
C	-1.975733	1.451422	-0.461799
C	-0.587292	1.238604	0.191734
C	0.360825	0.478345	-0.763322
C	-0.216431	-0.863082	-1.121005
O	-2.060633	2.860138	-0.781134
C	-1.062146	3.566913	-0.229058
C	-0.148629	2.641096	0.482034
O	-0.990557	4.762578	-0.336879
C	0.836543	3.090308	1.247518
C	-3.182778	1.072506	0.394193
C	0.185488	-2.098683	1.035393
O	-3.721880	-3.657809	-0.595952
O	1.644313	0.405663	-0.116874
C	2.723329	0.391061	-0.918754
O	2.653590	0.429615	-2.122006
C	4.000293	0.331190	-0.131117
C	4.203580	-0.986414	0.631204
C	4.223573	-2.183296	-0.311269
C	5.484378	-0.911050	1.452303
H	-2.671580	-3.046491	0.987601
H	-3.239997	-0.985313	-1.218723
H	-2.177548	-0.219998	2.681126
H	-3.078759	-1.732629	2.484889
H	-1.542518	-2.934208	-1.834642
H	-1.034443	-3.991957	-0.512493
H	-3.925369	-0.209338	2.749270
H	-0.682522	0.652249	1.104856
H	0.487172	1.061830	-1.675840
H	-0.681596	-0.887453	-2.101298
H	1.484056	2.424516	1.803080
H	1.006178	4.157608	1.335103
H	-3.186028	1.693306	1.293633
H	-2.038339	0.937337	-1.418517
H	-4.085758	1.324924	-0.167985
H	0.867684	-2.955561	1.036735
H	-0.593460	-2.331035	1.766295
H	0.741335	-1.230491	1.379509
H	-3.844304	-3.448834	-1.528407

H	4.818568	0.485506	-0.835552
H	3.999843	1.163657	0.577348
H	3.362954	-1.105366	1.321640
H	4.360650	-3.112462	0.246793
H	3.294327	-2.273480	-0.878842
H	5.043954	-2.102843	-1.030508
H	5.636566	-1.831810	2.020290
H	5.458445	-0.081189	2.162929
H	6.356837	-0.771504	0.807274

mPW1PW91energy = -1155.16986272 a.u.

(1*R*,5*S*,6*S*,7*S*,10*R*)-**2**, Conf. A

C	3.442704	0.014453	-0.243782
C	2.919867	1.391063	-0.659693
C	1.576665	1.652446	-0.043458
C	0.567980	0.580973	-0.380021
C	1.048466	-0.782142	0.191534
C	2.413742	-1.076480	-0.476247
C	-0.882091	0.924883	-0.038548
C	-1.841076	-0.188004	-0.455241
C	-1.396006	-1.520479	0.141487
C	0.041050	-1.858875	-0.231534
C	-3.317037	0.163052	-0.174686
C	-4.265175	-0.776766	-0.913850
C	-3.672924	0.222059	1.308104
O	-1.283327	2.101941	-0.735982
C	1.358116	2.687694	0.765027
O	2.998430	-2.292476	-0.028332
H	0.591580	0.471738	-1.474880
C	1.188159	-0.758786	1.713668
H	3.719827	0.024406	0.813811
H	4.346936	-0.230329	-0.805862
H	3.632636	2.168201	-0.378586
H	2.820452	1.419217	-1.751419
H	2.225814	-1.148290	-1.557668
H	-0.973860	1.096970	1.041585
H	-1.744786	-0.266161	-1.547027
H	-2.052888	-2.318946	-0.211420
H	-1.502200	-1.498680	1.229370
H	0.102132	-1.986811	-1.319471
H	0.315145	-2.818804	0.218619
H	-3.469901	1.163681	-0.587646
H	-4.217328	-1.797939	-0.526285
H	-5.300156	-0.440632	-0.810271
H	-4.036589	-0.813630	-1.982552
H	-4.697036	0.581807	1.436496
H	-3.021613	0.900012	1.864633
H	-3.615346	-0.761387	1.782417
H	-0.605063	2.771391	-0.596193
H	0.409255	2.856072	1.263602
H	2.146387	3.402086	0.978209
H	2.435998	-3.021952	-0.304721
H	1.616291	-1.700421	2.062260
H	1.834742	0.050178	2.054158
H	0.224111	-0.628050	2.206786

mPW1PW91 energy = -737.762624271 a.u.

(1*R*,5*S*,6*S*,7*S*,10*R*)-**2**, Conf. B

C	3.440520	0.020947	-0.264638
C	2.912494	1.401301	-0.664004
C	1.574113	1.650677	-0.033724
C	0.566367	0.582475	-0.381525
C	1.043986	-0.788633	0.173096
C	2.412965	-1.080426	-0.486664
C	-0.884280	0.923612	-0.037317
C	-1.845117	-0.186002	-0.458853
C	-1.395617	-1.525471	0.119949
C	0.038495	-1.857717	-0.267327
C	-3.319199	0.162873	-0.165506
C	-4.272023	-0.767689	-0.910321
C	-3.666524	0.202933	1.320006
O	-1.285077	2.105917	-0.726519
C	1.359318	2.672211	0.793016
O	2.942165	-2.345024	-0.110546
H	0.590679	0.483751	-1.477167
C	1.171421	-0.779045	1.697705
H	3.731544	0.035203	0.791373
H	4.339733	-0.219491	-0.836887
H	3.625929	2.177872	-0.382507
H	2.803678	1.437270	-1.754400
H	2.229919	-1.172086	-1.562618
H	-0.976043	1.090047	1.043548
H	-1.755427	-0.252830	-1.551904
H	-2.054874	-2.319293	-0.239220
H	-1.496715	-1.517302	1.208889
H	0.095626	-1.958642	-1.357683
H	0.332645	-2.823455	0.150229
H	-3.474674	1.168617	-0.564904
H	-4.222433	-1.793200	-0.534719
H	-5.306257	-0.432181	-0.797305
H	-4.049018	-0.792677	-1.980514
H	-4.690019	0.560489	1.459137
H	-3.012276	0.873904	1.881681
H	-3.605768	-0.786632	1.780917
H	-0.608886	2.775625	-0.578549
H	0.412681	2.831521	1.298681
H	2.147316	3.385204	1.011956
H	3.267914	-2.279246	0.792866
H	1.889183	-0.036983	2.049023
H	0.220567	-0.554426	2.181540
H	1.486750	-1.760491	2.061708

mPW1PW91 energy = -737.762492816 a.u.

(1*R*,5*S*,6*S*,7*S*,10*R*)-**2**, Conf. C

C	3.437942	0.021327	-0.231255
C	2.917790	1.396101	-0.658700
C	1.574134	1.653300	-0.041608
C	0.567239	0.581516	-0.382771
C	1.043962	-0.786316	0.182323
C	2.409475	-1.075163	-0.470708
C	-0.883254	0.922717	-0.039540
C	-1.843419	-0.187755	-0.460604
C	-1.395365	-1.526781	0.119805
C	0.041248	-1.860142	-0.257027
C	-3.317809	0.161397	-0.168552
C	-4.270354	-0.771236	-0.911129
C	-3.665618	0.205067	1.316782
O	-1.284437	2.103180	-0.732144
C	1.355715	2.683968	0.772779
O	2.880409	-2.330156	0.005528
H	0.590950	0.476880	-1.477805
C	1.168204	-0.770537	1.707099
H	3.704431	0.036397	0.828826
H	4.350977	-0.218335	-0.785658
H	3.628856	2.176136	-0.381111
H	2.818559	1.417519	-1.750517
H	2.228805	-1.149365	-1.553398
H	-0.975169	1.091188	1.041009
H	-1.752896	-0.255080	-1.553637
H	-2.051913	-2.321125	-0.243243
H	-1.503100	-1.519027	1.207859
H	0.105698	-1.969124	-1.346347
H	0.332205	-2.821807	0.171214
H	-3.473395	1.166146	-0.570421
H	-4.220809	-1.795663	-0.532564
H	-5.304658	-0.435503	-0.799339
H	-4.047097	-0.799276	-1.981227
H	-4.689157	0.563065	1.454648
H	-3.011478	0.877196	1.877125
H	-3.605076	-0.783305	1.780232
H	-0.608176	2.773237	-0.586135
H	0.407438	2.848191	1.273799
H	2.143124	3.398658	0.988228
H	3.727720	-2.508119	-0.413834
H	1.835005	0.017733	2.057515
H	0.202468	-0.609418	2.187432
H	1.559571	-1.726174	2.058901

mPW1PW91 energy = -737.762028762 a.u.

(1*R*,5*S*,6*S*,7*S*,10*R*)-**2**, Conf. D

C	3.396062	0.422836	-0.243759
C	2.660631	1.717395	-0.594251
C	1.302349	1.734596	0.044691
C	0.465578	0.539882	-0.346549
C	1.159890	-0.761075	0.144437
C	2.548723	-0.800932	-0.536114
C	-1.015862	0.636258	0.023935
C	-1.803550	-0.580861	-0.454769
C	-1.137628	-1.865349	0.039360
C	0.330666	-1.953295	-0.348215
C	-3.307870	-0.573715	-0.089416
C	-3.588526	-0.150749	1.349701
C	-4.168838	0.229660	-1.060157
O	-1.594429	1.787874	-0.584581
C	0.945189	2.674162	0.918130
O	3.317044	-1.934546	-0.155693
H	0.491361	0.495364	-1.445885

C	1.301724	-0.808221	1.665713
H	3.670092	0.425248	0.814609
H	4.325997	0.351277	-0.812694
H	3.247244	2.583108	-0.282021
H	2.543269	1.777753	-1.682943
H	2.365626	-0.840513	-1.620131
H	-1.113741	0.715665	1.114128
H	-1.735567	-0.575658	-1.550568
H	-1.672760	-2.721397	-0.381036
H	-1.245288	-1.945744	1.125282
H	0.404409	-2.001057	-1.441880
H	0.753136	-2.886120	0.040915
H	-3.627612	-1.618977	-0.182180
H	-2.990251	-0.712105	2.071943
H	-3.388511	0.913039	1.498798
H	-4.639421	-0.322267	1.596795
H	-3.951448	1.296154	-0.999858
H	-5.230127	0.083899	-0.838708
H	-4.001724	-0.086663	-2.093384
H	-1.008679	2.533533	-0.412291
H	-0.006724	2.665788	1.438274
H	1.620432	3.486858	1.164102
H	2.859786	-2.726152	-0.454408
H	1.887533	-1.681143	1.959314
H	1.799203	0.077922	2.060365
H	0.331615	-0.877975	2.159231

mPW1PW91 energy = -737.760619359 a.u.

(1*R*,5*S*,6*S*,7*S*,10*R*)-**2**, Conf. E

C	3.392499	0.427241	-0.269760
C	2.651608	1.724991	-0.599583
C	1.300769	1.731922	0.054611
C	0.463697	0.540785	-0.344976
C	1.156831	-0.767527	0.127955
C	2.546870	-0.807152	-0.548762
C	-1.017829	0.634042	0.028887
C	-1.806992	-0.579079	-0.457594
C	-1.138133	-1.867427	0.023518
C	0.326323	-1.951642	-0.376078
C	-3.310719	-0.573979	-0.090451
C	-3.590331	-0.162296	1.352117
C	-4.172621	0.236671	-1.054320
O	-1.596136	1.792029	-0.567880
C	0.950229	2.660097	0.942957
O	3.267763	-1.993174	-0.241890
H	0.487049	0.506509	-1.444554
C	1.295610	-0.830087	1.650325
H	3.684417	0.434775	0.785960
H	4.314299	0.359465	-0.852382
H	3.240416	2.589345	-0.287213
H	2.521947	1.793271	-1.686221
H	2.369183	-0.868498	-1.627694
H	-1.114651	0.704178	1.119800
H	-1.740970	-0.565414	-1.553443
H	-1.676586	-2.719810	-0.400318
H	-1.240050	-1.957012	1.109677
H	0.392764	-1.979505	-1.470301
H	0.767970	-2.882788	-0.013758
H	-3.630398	-1.618496	-0.190893
H	-4.641278	-0.334828	1.598445
H	-2.992288	-0.730497	2.069223
H	-3.389242	0.900100	1.509720
H	-5.233859	0.088536	-0.834005
H	-4.005355	-0.071314	-2.090019
H	-3.955906	1.302833	-0.985576
H	-1.010983	2.535996	-0.386641
H	0.002081	2.644534	1.469820
H	1.625789	3.471506	1.192383
H	3.583682	-1.927790	0.665092
H	0.325298	-0.795604	2.146585
H	1.772298	-1.766516	1.952440
H	1.884496	-0.003180	2.048955

mPW1PW91 energy = -737.760531328 a.u.

(1*R*,5*S*,6*S*,7*S*,10*R*)-**2**, Conf. F

C	3.389704	0.426058	-0.240255
C	2.655826	1.720735	-0.596803
C	1.300024	1.733943	0.047345
C	0.464373	0.538998	-0.345307
C	1.156961	-0.766088	0.139805
C	2.541578	-0.802902	-0.533743
C	-1.017016	0.633327	0.027134
C	-1.804942	-0.581406	-0.457268
C	-1.138007	-1.868201	0.029820
C	0.329125	-1.955563	-0.359764
C	-3.309482	-0.575212	-0.093275
C	-3.591677	-0.158185	1.347257
C	-4.169936	0.231672	-1.061608
O	-1.595375	1.788645	-0.575319
C	0.945829	2.669386	0.926474
O	3.207098	-1.993959	-0.131368
H	0.488328	0.497590	-1.444534
C	1.293745	-0.818875	1.662465
H	3.660640	0.431402	0.818743
H	4.323189	0.360812	-0.808561
H	3.242023	2.588402	-0.288997
H	2.535447	1.776283	-1.685336
H	2.359413	-0.844616	-1.617921
H	-1.114724	0.707552	1.117642
H	-1.736383	-0.570705	-1.553049
H	-1.673934	-2.721846	-0.394784
H	-1.246386	-1.954940	1.115425
H	0.403118	-1.993138	-1.453357
H	0.766384	-2.883480	0.015314
H	-3.628827	-1.620171	-0.190391
H	-4.642936	-0.330303	1.592606
H	-2.994335	-0.723070	2.067499
H	-3.391303	0.904903	1.501055
H	-5.231503	0.084017	-0.842530
H	-4.000883	-0.079938	-2.095968
H	-3.953625	1.298140	-0.996348
H	-1.009605	2.533227	-0.398762

H	-0.003618	2.656945	1.451014
H	1.620583	3.482500	1.172477
H	4.073428	-2.006124	-0.549402
H	0.319532	-0.855402	2.151319
H	1.847111	-1.711891	1.956497
H	1.818452	0.050244	2.060188

mPW1PW91 energy = -737.760100789 a.u.

(1*R*,5*S*,6*S*,7*S*,10*R*)-**2**, Conf. G

C	3.454348	-0.004267	-0.212905
C	2.951311	1.381997	-0.612780
C	1.596014	1.660689	-0.027155
C	0.575603	0.606475	-0.388899
C	1.037189	-0.779299	0.152633
C	2.416672	-1.082519	-0.479341
C	-0.879575	0.940469	-0.038980
C	-1.846626	-0.173449	-0.461511
C	-1.394415	-1.529098	0.073962
C	0.040047	-1.842068	-0.319611
C	-3.314745	0.166027	-0.130349
C	-4.284295	-0.727489	-0.898306
C	-3.636722	0.137302	1.361432
O	-1.267263	2.179684	-0.624150
C	1.376141	2.706977	0.762242
O	2.913298	-2.363665	-0.113230
H	0.604915	0.511446	-1.487503
C	1.133420	-0.786825	1.679970
H	3.714364	-0.006179	0.851308
H	4.366250	-0.251472	-0.762034
H	3.665128	2.149865	-0.308726
H	2.878056	1.426630	-1.706677
H	2.258689	-1.152067	-1.561295
H	-0.964995	1.109546	1.037312
H	-1.778977	-0.220845	-1.559557
H	-2.052303	-2.313723	-0.308223
H	-1.495294	-1.553802	1.162216
H	0.102246	-1.913127	-1.412359
H	0.340247	-2.816446	0.072457
H	-3.472438	1.190911	-0.477355
H	-5.315407	-0.396676	-0.749145
H	-4.083441	-0.706397	-1.973114
H	-4.229254	-1.768939	-0.570045
H	-2.967182	0.774000	1.944506
H	-3.578803	-0.874181	1.771626
H	-4.654226	0.496992	1.534408
H	-1.126376	2.114947	-1.574786
H	0.401792	2.926500	1.179036
H	2.179329	3.397312	1.000660
H	3.235180	-2.313848	0.792549
H	1.449399	-1.769762	2.039525
H	1.837050	-0.042532	2.053920
H	0.171575	-0.575832	2.147927

mPW1PW91 energy = -737.760032458 a.u.

(1*R*,5*S*,6*S*,7*S*,10*R*)-**2**, Conf. H

C	3.455995	-0.011253	-0.189766
C	2.959136	1.370934	-0.607752
C	1.598175	1.662574	-0.040298
C	0.576838	0.604011	-0.389003
C	1.042058	-0.773422	0.171506
C	2.417960	-1.079322	-0.468279
C	-0.877445	0.940139	-0.039227
C	-1.842626	-0.176404	-0.458593
C	-1.395245	-1.524735	0.097103
C	0.042543	-1.845272	-0.280149
C	-3.313102	0.165737	-0.141288
C	-4.276996	-0.739053	-0.903139
C	-3.644670	0.159508	1.348625
O	-1.267770	2.176405	-0.628116
C	1.373757	2.725121	0.725826
O	2.970006	-2.313856	-0.029053
H	0.603936	0.496995	-1.486825
C	1.152836	-0.763661	1.696586
H	3.699944	-0.017185	0.875934
H	4.373573	-0.263231	-0.726764
H	3.671448	2.139745	-0.302976
H	2.898124	1.406697	-1.702936
H	2.256624	-1.128186	-1.555657
H	-0.961298	1.110961	1.037071
H	-1.767646	-0.235316	-1.555558
H	-2.050121	-2.315052	-0.278233
H	-1.502508	-1.533614	1.184616
H	0.109801	-1.948352	-1.370595
H	0.319694	-2.813656	0.149506
H	-3.468493	1.185258	-0.504474
H	-4.068723	-0.732640	-1.976758
H	-4.224540	-1.775806	-0.559850
H	-5.309112	-0.406231	-0.766054
H	-4.663271	0.521660	1.509426
H	-2.978895	0.805100	1.926089
H	-3.589607	-0.845362	1.775384
H	-1.107281	2.117426	-1.575954
H	0.396670	2.955423	1.129975
H	2.177431	3.416938	0.958209
H	2.403732	-3.028923	-0.333314
H	1.783132	0.049814	2.056122
H	0.179073	-0.648355	2.174184
H	1.585133	-1.703919	2.043880

mPW1PW91 energy = -737.760001242 a.u.

(1*R*,5*S*,6*S*,7*S*,10*R*)-**2**, Conf. I

C	3.450887	-0.006164	-0.173102
C	2.958863	1.374399	-0.603911
C	1.596672	1.663611	-0.037989
C	0.576556	0.605526	-0.391985
C	1.036586	-0.777217	0.160612
C	2.413124	-1.077525	-0.462720
C	-0.877991	0.939096	-0.039995
C	-1.844981	-0.174571	-0.463894
C	-1.394628	-1.531334	0.069707
C	0.042917	-1.844829	-0.311830
C	-3.313151	0.164544	-0.131960

C	-4.282985	-0.730645	-0.897640
C	-3.633864	0.138058	1.360188
O	-1.267896	2.178223	-0.624152
C	1.372098	2.722984	0.732506
O	2.847571	-2.352429	-0.003572
H	0.603691	0.504196	-1.490010
C	1.128698	-0.777525	1.688193
H	3.681783	-0.007294	0.895354
H	4.378099	-0.253442	-0.700474
H	3.669823	2.145744	-0.302287
H	2.899717	1.402427	-1.699333
H	2.260588	-1.124989	-1.551774
H	-0.961350	1.106824	1.036698
H	-1.777464	-0.219733	-1.562124
H	-2.048925	-2.315305	-0.319959
H	-1.504199	-1.559795	1.156757
H	0.113998	-1.923592	-1.403633
H	0.339093	-2.815711	0.090909
H	-3.471801	1.188820	-0.480279
H	-4.082742	-0.711671	-1.972640
H	-4.227728	-1.771395	-0.567241
H	-5.314058	-0.399612	-0.748598
H	-4.651216	0.498109	1.533426
H	-2.963691	0.775449	1.941704
H	-3.575690	-0.872680	1.772126
H	-1.127564	2.114135	-1.574888
H	0.394893	2.950986	1.137595
H	2.175224	3.414644	0.967321
H	3.725228	-2.512743	-0.363476
H	1.773046	0.019077	2.060889
H	0.151230	-0.639046	2.151499
H	1.529815	-1.730204	2.037151

mPW1PW91 energy = -737.759535593 a.u.

(1*R*,5*S*,6*S*,7*S*,10*R*)-**2**, Conf. J

C	3.455176	-0.007508	-0.205268
C	2.952645	1.373279	-0.623180
C	1.593899	1.661234	-0.049427
C	0.577236	0.599047	-0.398957
C	1.044478	-0.768872	0.180559
C	2.416378	-1.079958	-0.465539
C	-0.874390	0.935774	-0.062642
C	-1.836850	-0.190390	-0.463203
C	-1.391923	-1.522749	0.131385
C	0.044427	-1.850214	-0.245530
C	-3.313718	0.149563	-0.170820
C	-4.262205	-0.823573	-0.865849
C	-3.650602	0.251013	1.314740
O	-1.184076	2.129922	-0.778576
C	1.371761	2.718151	0.724550
O	2.973053	-2.309046	-0.015953
H	0.607782	0.489469	-1.493816
C	1.167043	-0.740383	1.704694
H	3.711533	-0.008483	0.857607
H	4.367084	-0.260628	-0.751386
H	3.665077	2.143773	-0.322875
H	2.884056	1.408165	-1.717664
H	2.247193	-1.141320	-1.550734
H	-0.958462	1.127337	1.014008
H	-1.749971	-0.271436	-1.555425
H	-2.045814	-2.322805	-0.223666
H	-1.496696	-1.505066	1.219406
H	0.104827	-1.974646	-1.333998
H	0.325293	-2.809364	0.201947
H	-3.505772	1.130562	-0.621120
H	-4.204508	-1.826720	-0.436270
H	-5.298192	-0.489379	-0.767990
H	-4.039627	-0.902971	-1.933262
H	-4.680424	0.591906	1.447662
H	-3.005585	0.956657	1.843869
H	-3.564453	-0.716357	1.815944
H	-2.013780	2.480182	-0.443576
H	0.396371	2.943279	1.136557
H	2.173775	3.411675	0.957185
H	2.410288	-3.028239	-0.316925
H	1.591815	-1.680882	2.060723
H	1.808385	0.070692	2.049375
H	0.197852	-0.608889	2.187578

mPW1PW91 energy = -737.759349994 a.u.

(1*R*,5*S*,6*S*,7*S*,10*R*)-**2**, Conf. K

C	3.452944	-0.000967	-0.230430
C	2.944113	1.383556	-0.630835
C	1.591309	1.659347	-0.038888
C	0.575451	0.600398	-0.398405
C	1.040013	-0.775221	0.163494
C	2.414559	-1.083875	-0.476626
C	-0.876590	0.934497	-0.059653
C	-1.840595	-0.188439	-0.465810
C	-1.391498	-1.527638	0.111690
C	0.041796	-1.848975	-0.279931
C	-3.315991	0.149197	-0.162931
C	-4.267832	-0.815500	-0.865163
C	-3.646402	0.233436	1.325129
O	-1.183761	2.134398	-0.767184
C	1.373659	2.702225	0.755122
O	2.917033	-2.359611	-0.098969
H	0.606338	0.501417	-1.494076
C	1.150841	-0.760769	1.690003
H	3.726186	0.002951	0.830549
H	4.358618	-0.249874	-0.789075
H	3.658095	2.153324	-0.331916
H	2.863010	1.426623	-1.723962
H	2.247190	-1.166443	-1.556016
H	-0.961360	1.120368	1.017903
H	-1.758929	-0.258640	-1.559157
H	-2.047968	-2.323013	-0.249273
H	-1.491067	-1.523003	1.200745
H	0.097469	-1.945330	-1.370873
H	0.344573	-2.813743	0.133363
H	-3.511091	1.135358	-0.600782
H	-4.049871	-0.881931	-1.934376

H	-4.207834	-1.823719	-0.448079
H	-5.303372	-0.482513	-0.758520
H	-2.999647	0.933600	1.859475
H	-3.557108	-0.739583	1.814679
H	-4.675911	0.571838	1.466697
H	-2.021877	2.474088	-0.442464
H	0.401004	2.917425	1.178888
H	2.175272	3.394906	0.991709
H	3.246581	-2.297750	0.803251
H	1.861533	-0.014691	2.046402
H	0.194229	-0.537379	2.163022
H	1.465102	-1.740226	2.060516

mPW1PW91 energy = -737.759296796 a.u.

Table S38. Cartesian coordinates and energies of the low-energy conformers calculated at the ωB97XD/6-31+G(d,p) *in vacuo* level.

(2*S*,6*R*,7*S*,8*R*)-**1**, Conf. A

C	-4.304780	-0.627320	-0.621733
C	-3.317387	0.430322	-1.029116
C	-2.907432	1.487862	-0.317513
C	-3.554786	1.990280	0.947169
C	-2.349652	-1.734333	0.500305
C	-3.555168	-1.962547	-0.378761
C	-0.409692	1.303639	-0.687817
C	-0.140739	0.617495	0.679655
C	0.040027	-0.917050	0.585110
C	-1.150982	-1.558620	-0.065463
O	0.757432	2.092162	-0.990151
C	1.615915	2.165263	0.050139
C	1.093966	1.323500	1.164252
O	2.627922	2.817226	0.013031
C	1.701772	1.249033	2.344891
C	-1.639832	2.224006	-0.721604
C	-2.625421	-1.500780	1.961518
O	-5.265673	-0.899238	-1.633764
O	1.192351	-1.170837	-0.230143
C	2.254510	-1.797025	0.328706
O	2.269312	-2.194905	1.470548
C	3.387663	-1.893895	-0.657915
C	4.057630	-0.528696	-0.923507
C	4.698466	0.037275	0.344620
C	5.090971	-0.671471	-2.042580
H	-4.817706	-0.341254	0.305752
H	-2.783341	0.188551	-1.949712
H	-4.468040	1.455301	1.213642
H	-3.808530	3.050079	0.828331
H	-3.250798	-2.345561	-1.358263
H	-4.259494	-2.680280	0.055479
H	-2.863109	1.934355	1.796993
H	-0.980209	0.786110	1.359870
H	0.234422	-1.310078	1.586704
H	-1.044034	-1.727168	-1.135177
H	1.328529	0.615072	3.143229
H	2.609087	1.818838	2.521780
H	-1.457342	3.063306	-0.040615
H	-0.481225	0.562906	-1.486178
H	-1.723874	2.646041	-1.728401
H	-1.717252	-1.501385	2.569010
H	-3.294346	-2.274653	2.351902
H	-3.130123	-0.538112	2.112655
H	-5.714164	-0.078503	-1.857261
H	4.118763	-2.600327	-0.254505
H	2.992447	-2.294933	-1.597260
H	3.281539	0.167775	-1.262928
H	5.115122	1.029794	0.153221
H	3.972501	0.135783	1.156294
H	5.504283	-0.619155	0.694328
H	5.565543	0.291786	-2.252126
H	4.631999	-1.032153	-2.968982
H	5.878306	-1.379024	-1.756089

ωB97XD energy = -1154.82248790 a.u.

(2*S*,6*R*,7*S*,8*R*)-**1**, Conf. B

C	-4.342666	-0.906345	-0.521053
C	-3.465456	0.222327	-0.985254
C	-3.116840	1.324488	-0.309507
C	-3.750928	1.796872	0.973279
C	-2.257660	-1.809255	0.556138
C	-3.470108	-2.163843	-0.269657
C	-0.629060	1.337090	-0.781243
C	-0.239618	0.717631	0.588610
C	0.058568	-0.800859	0.532807
C	-1.097893	-1.553008	-0.058024
O	0.450746	2.210622	-1.161849
C	1.347180	2.388455	-0.166945
C	0.953380	1.537840	0.992760
O	2.294021	3.125291	-0.268000
C	1.623677	1.548493	2.141437
C	-1.931493	2.152737	-0.779290
C	-2.500714	-1.562333	2.020915
O	-5.304444	-1.289741	-1.495251
O	1.202546	-0.998194	-0.313063
C	2.339588	-1.467170	0.246262
O	2.444184	-1.742524	1.419683
C	3.437114	-1.612551	-0.777594
C	4.781810	-1.057905	-0.282388
C	5.887012	-1.392493	-1.285580
C	4.695823	0.450416	-0.039768
H	-4.852084	-0.642065	0.414824
H	-2.949920	0.007272	-1.922926
H	-4.600076	1.189172	1.291026
H	-4.104090	2.826994	0.847254
H	-3.169631	-2.549154	-1.249456
H	-4.092884	-2.925711	0.211323
H	-3.020564	1.820265	1.791544
H	-1.057612	0.838236	1.304591
H	0.313354	-1.141608	1.539562
H	-1.015290	-1.741665	-1.126564
H	1.348123	0.907362	2.972891
H	2.484736	2.200226	2.256641
H	-1.791406	3.019193	-0.122743
H	-0.676969	0.569240	-1.555275
H	-2.089146	2.544052	-1.789626
H	-3.097068	-0.653283	2.168301
H	-1.575613	-1.451231	2.591327
H	-3.068853	-2.390372	2.457319
H	-5.833062	-0.519148	-1.721980
H	3.531632	-2.686134	-0.982967
H	3.134140	-1.122827	-1.707978
H	5.008646	-1.552293	0.669463

H	6.853756	-1.020078	-0.932382
H	5.978371	-2.472752	-1.440923
H	5.686736	-0.925741	-2.257487
H	5.653793	0.843450	0.314657
H	3.940160	0.696319	0.711894
H	4.434084	0.985133	-0.959909

ωB97XD energy = -1154.82143750 a.u.

(2*S*,6*R*,7*S*,8*R*)-**1**, Conf. C

C	-4.463625	-0.284837	0.042448
C	-3.478439	0.841968	0.287923
C	-2.709605	1.476191	-0.605920
C	-2.841626	1.331223	-2.099072
C	-2.427964	-1.752128	0.309529
C	-3.741952	-1.587541	-0.409958
C	-0.233595	1.497311	-0.184390
C	-0.074382	0.414572	0.920046
C	-0.016554	-1.034088	0.382343
C	-1.299877	-1.382076	-0.306118
O	0.868905	2.413864	-0.028686
C	1.704619	2.066858	0.973591
C	1.182891	0.836273	1.629524
O	2.697906	2.694905	1.237003
C	1.806579	0.262242	2.654456
C	-1.537850	2.309668	-0.135963
C	-2.508990	-2.101857	1.772083
O	-5.444455	-0.021512	-0.953113
O	1.063260	-1.102034	-0.559431
C	2.141596	-1.862501	-0.258751
O	2.208546	-2.571588	0.718378
C	3.223052	-1.662164	-1.286968
C	3.899823	-0.279938	-1.154889
C	4.640446	-0.143664	0.176633
C	4.847713	-0.051707	-2.333735
H	-4.969676	-0.488869	0.997163
H	-3.276962	1.034513	1.342159
H	-2.986647	2.317109	-2.556247
H	-1.923675	0.919120	-2.536230
H	-3.574224	-1.503105	-1.487558
H	-4.419112	-2.432041	-0.242324
H	-3.681508	0.696842	-2.381132
H	-0.928836	0.458914	1.605026
H	0.206739	-1.710684	1.210641
H	-1.340558	-1.116522	-1.360643
H	1.439976	-0.650041	3.114444
H	2.721152	0.701253	3.041717
H	-1.690021	2.670070	0.887465
H	-0.106014	1.050650	-1.174438
H	-1.399808	3.186264	-0.777965
H	-2.842886	-1.237545	2.362999
H	-1.553468	-2.435248	2.183193
H	-3.242446	-2.897982	1.934664
H	-5.890723	0.800664	-0.729904
H	3.962341	-2.457382	-1.156238
H	2.775235	-1.756304	-2.281772
H	3.113401	0.484121	-1.197207

H	5.064912	0.858675	0.280355
H	3.977524	-0.311352	1.029653
H	5.455464	-0.874621	0.238307
H	5.326636	0.929031	-2.256963
H	4.317685	-0.096428	-3.290837
H	5.638642	-0.811022	-2.348404

ωB97XD energy = -1154.82087318 a.u.

(2*S*,6*R*,7*S*,8*R*)-**1**, Conf. D

C	-4.273503	-0.565038	-0.712397
C	-3.284607	0.529133	-0.999260
C	-2.873667	1.498371	-0.172146
C	-3.524099	1.855608	1.139313
C	-2.339338	-1.770634	0.348797
C	-3.520429	-1.913648	-0.580532
C	-0.363034	1.367575	-0.521319
C	-0.089075	0.561642	0.779647
C	0.046816	-0.969322	0.575181
C	-1.129686	-1.528223	-0.166827
O	0.787043	2.207671	-0.740115
C	1.665296	2.164795	0.284619
C	1.170755	1.195994	1.302795
O	2.674600	2.821957	0.302325
C	1.828642	0.966761	2.435871
C	-1.605451	2.271775	-0.491378
C	-2.655640	-1.706201	1.818908
O	-5.213611	-0.745222	-1.763308
O	1.228927	-1.218584	-0.201470
C	2.283687	-1.796362	0.417378
O	2.251014	-2.179311	1.565314
C	3.491941	-1.869002	-0.477902
C	4.117398	-0.480777	-0.768939
C	5.614960	-0.640517	-1.042377
C	3.430480	0.228341	-1.937980
H	-4.804863	-0.369364	0.227961
H	-2.747816	0.389793	-1.939452
H	-3.755144	2.927101	1.151523
H	-2.844390	1.678862	1.982065
H	-3.189126	-2.197914	-1.584593
H	-4.231199	-2.672607	-0.236404
H	-4.450916	1.311679	1.329692
H	-0.914266	0.697155	1.485125
H	0.181732	-1.435449	1.554105
H	-0.999859	-1.580904	-1.246103
H	1.487976	0.235438	3.161499
H	2.750156	1.503809	2.640101
H	-1.438027	3.053268	0.258835
H	-0.412811	0.700846	-1.385181
H	-1.679092	2.774598	-1.461146
H	-3.213321	-0.792050	2.057609
H	-1.763276	-1.726967	2.448657
H	-3.291890	-2.549453	2.107577
H	-5.662995	0.090826	-1.917294
H	4.210206	-2.507778	0.040331
H	3.219003	-2.352418	-1.423066
H	4.004131	0.141873	0.128050

H	6.072258	0.329193	-1.261130
H	6.134559	-1.074736	-0.182304
H	5.783727	-1.294447	-1.906697
H	3.828655	1.239491	-2.060310
H	2.352160	0.310702	-1.789862
H	3.601985	-0.324256	-2.870276

ωB97XD energy = -1154.82052521 a.u.

(2*S*,6*R*,7*S*,8*R*)-**1**, Conf. E

C	-4.241983	-0.594495	-0.574869
C	-3.258245	0.474015	-0.960671
C	-2.829501	1.499582	-0.214589
C	-3.449477	1.952436	1.082249
C	-2.275138	-1.737247	0.491048
C	-3.491413	-1.936745	-0.380300
C	-0.327202	1.346485	-0.628564
C	-0.031628	0.604148	0.704640
C	0.120339	-0.930514	0.569739
C	-1.085784	-1.536361	-0.085676
O	0.817494	2.177926	-0.901384
C	1.687209	2.218088	0.132564
C	1.214889	1.286343	1.194584
O	2.675435	2.905929	0.122765
C	1.875747	1.120401	2.336890
C	-1.570775	2.250729	-0.615121
C	-2.532090	-1.559588	1.963496
O	-5.214176	-0.834991	-1.584055
O	1.259577	-1.220591	-0.255884
C	2.319059	-1.840816	0.313613
O	2.376759	-2.106917	1.492755
C	3.392606	-2.161934	-0.696714
C	4.223732	-0.947242	-1.175849
C	3.466769	-0.061421	-2.167275
C	4.775684	-0.139910	0.001019
H	-4.744745	-0.336245	0.366175
H	-2.743060	0.267989	-1.900559
H	-4.358593	1.409204	1.346205
H	-3.702437	3.016726	1.011141
H	-3.199414	-2.290555	-1.374437
H	-4.191581	-2.666452	0.040509
H	-2.740614	1.860903	1.914580
H	-0.855893	0.758004	1.407415
H	0.308793	-1.343654	1.563964
H	-0.995582	-1.663656	-1.162669
H	1.549071	0.412585	3.091740
H	2.784958	1.685003	2.520821
H	-1.382569	3.071332	0.086728
H	-0.388412	0.641062	-1.460091
H	-1.672466	2.701773	-1.607603
H	-3.188235	-2.353351	2.335167
H	-3.043535	-0.607949	2.155657
H	-1.615870	-1.572158	2.558532
H	-5.664911	-0.007733	-1.776785
H	4.052588	-2.883650	-0.210036
H	2.923657	-2.644537	-1.560710
H	5.076214	-1.385721	-1.710613

H	4.111040	0.748567	-2.522010
H	3.132869	-0.638350	-3.036547
H	2.585036	0.395174	-1.712636
H	5.463913	0.632925	-0.352796
H	5.309822	-0.781289	0.709484
H	3.972450	0.367500	0.544518

ωB97XD energy = -1154.82051509 a.u.

(2*S*,6*R*,7*S*,8*R*)-**1**, Conf. F

C	-4.110870	-1.469790	-0.481310
C	-3.464744	-0.213636	-0.994666
C	-3.335379	0.961286	-0.365406
C	-4.057238	1.355670	0.897151
C	-1.903066	-1.914704	0.630816
C	-3.013898	-2.525626	-0.188333
C	-0.891397	1.439050	-0.830169
C	-0.402793	0.966215	0.567314
C	0.169517	-0.473278	0.582147
C	-0.810564	-1.454937	0.012193
O	-0.000191	2.490880	-1.247114
C	0.861408	2.858094	-0.274268
C	0.631516	1.998154	0.922090
O	1.667385	3.741175	-0.418227
C	1.310713	2.161002	2.054008
C	-2.329046	1.980256	-0.875370
C	-2.206648	-1.669636	2.084583
O	-4.981412	-2.066812	-1.433744
O	1.349090	-0.477968	-0.236787
C	2.544947	-0.658419	0.366493
O	2.668888	-0.925818	1.539588
C	3.672642	-0.433121	-0.608097
C	4.969638	-1.157346	-0.236102
C	4.805817	-2.675221	-0.350718
C	6.118161	-0.659792	-1.116200
H	-4.661692	-1.271439	0.447242
H	-2.913101	-0.363404	-1.924486
H	-4.590126	2.299275	0.732124
H	-3.352071	1.541869	1.716692
H	-2.633411	-2.874359	-1.153998
H	-3.481491	-3.379463	0.313731
H	-4.784215	0.614888	1.234254
H	-1.230780	0.976386	1.281939
H	0.462510	-0.720612	1.605701
H	-0.681845	-1.658789	-1.048913
H	1.161693	1.512124	2.911311
H	2.053793	2.949243	2.127826
H	-2.370830	2.893035	-0.269844
H	-0.776689	0.643942	-1.569581
H	-2.546183	2.274625	-1.907335
H	-1.327245	-1.365921	2.657213
H	-2.614805	-2.574624	2.546339
H	-2.966256	-0.885598	2.195369
H	-5.644121	-1.418535	-1.689191
H	3.336405	-0.710926	-1.612972
H	3.831915	0.653784	-0.627811
H	5.196010	-0.915093	0.808685

H	5.732343	-3.187198	-0.072521
H	4.011950	-3.043045	0.305964
H	4.563050	-2.960819	-1.381767
H	7.053721	-1.164764	-0.856211
H	6.273591	0.417898	-1.000992
H	5.915499	-0.859467	-2.175435

ωB97XD energy = -1154.82034764 a.u.

(2*S*,6*R*,7*S*,8*R*)-**1**, Conf. G

C	-3.851378	-1.513333	-0.429089
C	-3.141539	-0.371375	-1.100466
C	-3.093487	0.908763	-0.710713
C	-3.972066	1.529397	0.344507
C	-1.794897	-1.676092	1.004554
C	-2.803281	-2.464592	0.204742
C	-0.610428	1.331013	-0.977822
C	-0.281318	1.160972	0.531761
C	0.277711	-0.234981	0.902163
C	-0.634346	-1.331338	0.437102
O	0.328633	2.287756	-1.502342
C	1.077391	2.863538	-0.536817
C	0.714732	2.260469	0.777817
O	1.895846	3.715265	-0.770475
C	1.271068	2.661154	1.917551
C	-2.029952	1.831873	-1.283782
C	-2.269459	-1.145618	2.330669
O	-4.595966	-2.309149	-1.342284
O	1.540088	-0.385266	0.237153
C	2.657673	-0.473978	0.992106
O	2.648567	-0.476722	2.201478
C	3.887237	-0.550629	0.124265
C	3.815303	-1.619020	-0.980213
C	5.095738	-1.587202	-1.816825
C	3.575900	-3.010352	-0.388685
H	-4.515865	-1.145299	0.363406
H	-2.472686	-0.692449	-1.900869
H	-3.378595	1.894611	1.191892
H	-4.480147	2.405769	-0.074293
H	-2.313076	-2.996624	-0.617086
H	-3.331962	-3.204991	0.814527
H	-4.735768	0.851647	0.729972
H	-1.184014	1.301601	1.133383
H	0.453659	-0.265723	1.980423
H	-0.382601	-1.747561	-0.536442
H	1.030323	2.197720	2.869070
H	2.006775	3.459529	1.905785
H	-2.135852	2.838383	-0.862582
H	-0.423433	0.402686	-1.520897
H	-2.128946	1.931677	-2.369634
H	-1.465572	-0.704950	2.924857
H	-2.724697	-1.948232	2.920163
H	-3.041088	-0.378593	2.188158
H	-5.216641	-1.740102	-1.806663
H	4.024571	0.439439	-0.328306
H	4.736225	-0.736715	0.788190
H	2.970432	-1.368521	-1.631975

H	5.049574	-2.324790	-2.624074
H	5.254785	-0.602293	-2.267610
H	5.971261	-1.822736	-1.199922
H	3.545895	-3.767743	-1.178166
H	2.627507	-3.061921	0.156594
H	4.378974	-3.280918	0.306903

ωB97XD energy = -1154.82026763 a.u.

(2*S*,6*R*,7*S*,8*R*)-**1**, Conf. H

C	-4.471658	-0.590474	0.087456
C	-3.608974	0.655782	0.129366
C	-2.889052	1.193839	-0.862955
C	-2.982696	0.778429	-2.307499
C	-2.303870	-1.775143	0.605408
C	-3.615637	-1.871891	-0.130735
C	-0.427162	1.554949	-0.462182
C	-0.160697	0.696349	0.806089
C	0.025934	-0.813870	0.540059
C	-1.212224	-1.395811	-0.067507
O	0.552328	2.612904	-0.459027
C	1.413974	2.530892	0.576871
C	1.049690	1.352822	1.412687
O	2.313247	3.315304	0.737520
C	1.756870	1.003035	2.483509
C	-1.815802	2.211197	-0.547410
C	-2.372525	-1.886521	2.105239
O	-5.456990	-0.606279	-0.938184
O	1.118098	-0.976926	-0.379924
C	2.222759	-1.626408	0.046542
O	2.350204	-2.069806	1.165392
C	3.264070	-1.725525	-1.038648
C	4.589974	-1.053444	-0.638585
C	5.646337	-1.309884	-1.714850
C	4.400753	0.445644	-0.398231
H	-4.970639	-0.676626	1.063511
H	-3.446339	1.052585	1.131902
H	-3.745276	0.017074	-2.470155
H	-2.019669	0.401225	-2.673287
H	-3.438979	-1.961397	-1.206382
H	-4.206958	-2.739008	0.182993
H	-3.229577	1.649081	-2.926628
H	-1.015129	0.780780	1.487962
H	0.295431	-1.301661	1.479411
H	-1.269282	-1.310854	-1.150942
H	1.515073	0.125764	3.075002
H	2.620392	1.595096	2.772168
H	-2.017959	2.719942	0.401725
H	-0.228869	0.971331	-1.365707
H	-1.763102	2.977829	-1.327979
H	-1.391911	-2.018535	2.567840
H	-2.829446	-0.988065	2.542633
H	-3.000329	-2.734446	2.397280
H	-5.986180	0.193244	-0.863594
H	3.433638	-2.793378	-1.216242
H	2.876220	-1.282807	-1.960969
H	4.923018	-1.518607	0.297052

H	6.604210	-0.863609	-1.430419
H	5.806888	-2.381176	-1.874976
H	5.343715	-0.865555	-2.670691
H	5.351108	0.923035	-0.141870
H	3.704352	0.642547	0.421562
H	4.010111	0.940786	-1.295151

ωB97XD energy = -1154.82004927 a.u.

(2*S*,6*R*,7*S*,8*R*)-**1**, Conf. I

C	-4.297965	-0.909609	-0.433088
C	-3.459608	0.261239	-0.864590
C	-3.095813	1.324400	-0.136550
C	-3.677732	1.702553	1.201003
C	-2.152466	-1.841400	0.486355
C	-3.396907	-2.165164	-0.304076
C	-0.627616	1.429279	-0.717538
C	-0.165122	0.719171	0.584224
C	0.145785	-0.785829	0.410665
C	-1.031637	-1.517166	-0.166127
O	0.410319	2.363572	-1.069826
C	1.343024	2.490640	-0.100739
C	1.034635	1.525996	0.993842
O	2.255762	3.272183	-0.174912
C	1.773331	1.444720	2.096904
C	-1.949747	2.205678	-0.606433
C	-2.325593	-1.699706	1.974613
O	-5.298959	-1.246568	-1.384917
O	1.244733	-0.911866	-0.505097
C	2.349659	-1.571048	-0.087405
O	2.473517	-2.016605	1.031187
C	3.374310	-1.678121	-1.189297
C	4.684338	-0.910919	-0.901317
C	4.419095	0.575045	-0.653435
C	5.495958	-1.532834	0.237415
H	-4.767123	-0.714345	0.539961
H	-2.984338	0.114210	-1.836129
H	-4.498069	1.056787	1.518776
H	-4.057649	2.729903	1.155501
H	-3.137444	-2.480463	-1.319905
H	-3.984491	-2.967721	0.154479
H	-2.910684	1.692748	1.985099
H	-0.949786	0.777371	1.344091
H	0.459099	-1.190102	1.376655
H	-1.003061	-1.631791	-1.248010
H	1.559359	0.723559	2.879668
H	2.627575	2.103561	2.222019
H	-1.799201	3.034099	0.095412
H	-0.684122	0.720172	-1.545572
H	-2.161452	2.653812	-1.582673
H	-2.940305	-0.822550	2.213299
H	-1.375524	-1.596255	2.503881
H	-2.845394	-2.572792	2.382561
H	-5.847795	-0.471952	-1.537990
H	3.595665	-2.743856	-1.312050
H	2.929127	-1.310498	-2.116884
H	5.273723	-1.001486	-1.822946

H	5.359246	1.129256	-0.577428
H	3.828283	1.026128	-1.456522
H	3.875275	0.725571	0.285403
H	6.454721	-1.015092	0.344348
H	5.701191	-2.591684	0.048334
H	4.960627	-1.463887	1.188583

ωB97XD energy = -1154.81987696 a.u.

(2*S*,6*R*,7*S*,8*R*)-**1**, Conf. J

C	-4.477702	-0.704980	0.164036
C	-3.660897	0.572720	0.176018
C	-2.987738	1.130044	-0.838309
C	-3.103183	0.699209	-2.276857
C	-2.253375	-1.802800	0.627915
C	-3.580941	-1.955763	-0.068901
C	-0.533277	1.586477	-0.507503
C	-0.201635	0.748693	0.759989
C	0.031954	-0.755699	0.497986
C	-1.195087	-1.389571	-0.077807
O	0.409698	2.676603	-0.541082
C	1.312300	2.624576	0.462005
C	1.008460	1.445732	1.320294
O	2.195837	3.433398	0.582982
C	1.758756	1.126890	2.371271
C	-1.946495	2.190672	-0.559165
C	-2.276580	-1.901064	2.130396
O	-5.488438	-0.764577	-0.834951
O	1.115548	-0.868142	-0.438835
C	2.244952	-1.488629	-0.036249
O	2.379316	-1.994085	1.054817
C	3.293967	-1.477999	-1.119849
C	4.702101	-1.198215	-0.575944
C	5.740576	-1.389079	-1.683010
C	4.790754	0.206881	0.024979
H	-4.947856	-0.802708	1.153291
H	-3.487975	0.982806	1.171453
H	-3.848936	-0.083283	-2.415200
H	-2.139315	0.343325	-2.661404
H	-3.432037	-2.049116	-1.148416
H	-4.129152	-2.841506	0.269721
H	-3.386508	1.557113	-2.898075
H	-1.036137	0.812250	1.468280
H	0.338327	-1.229255	1.433123
H	-1.282592	-1.317574	-1.159966
H	1.558686	0.250113	2.979175
H	2.612896	1.746654	2.627661
H	-2.142489	2.699083	0.391377
H	-0.340558	1.001228	-1.411219
H	-1.944451	2.951873	-1.346665
H	-1.280938	-2.009247	2.566482
H	-2.739083	-1.007930	2.572966
H	-2.877788	-2.758974	2.447963
H	-6.046644	0.013858	-0.749446
H	3.265503	-2.468475	-1.591288
H	3.017090	-0.745428	-1.884309
H	4.896106	-1.928769	0.218002

H	6.751620	-1.229182	-1.295839
H	5.697007	-2.397828	-2.107174
H	5.579062	-0.672203	-2.497075
H	5.790249	0.394058	0.429302
H	4.072527	0.342867	0.839181
H	4.586259	0.972107	-0.733120

ωB97XD energy = -1154.81979242 a.u.

(2*S*,6*R*,7*S*,8*R*)-**1**, Conf. K

C	-4.396864	-0.320820	0.136132
C	-3.419551	0.820171	0.340938
C	-2.664455	1.436579	-0.576690
C	-2.808164	1.254403	-2.064596
C	-2.334966	-1.753053	0.415199
C	-3.666117	-1.630471	-0.281228
C	-0.171221	1.534442	-0.192562
C	0.038909	0.457596	0.909524
C	0.072155	-1.002266	0.404292
C	-1.230012	-1.372270	-0.234377
O	0.883602	2.504644	-0.029514
C	1.753809	2.177114	0.950408
C	1.317621	0.896433	1.571150
O	2.713297	2.853369	1.218349
C	2.027153	0.300074	2.525202
C	-1.503627	2.300020	-0.138404
C	-2.379819	-2.074497	1.885138
O	-5.385463	-0.096511	-0.861326
O	1.116071	-1.126350	-0.575257
C	2.178881	-1.909570	-0.283866
O	2.300552	-2.505108	0.762749
C	3.175657	-1.938132	-1.415247
C	4.077256	-0.682351	-1.501936
C	3.336507	0.550110	-2.024759
C	4.775305	-0.391277	-0.171605
H	-4.895928	-0.499775	1.099483
H	-3.207546	1.040195	1.387731
H	-3.637270	0.596700	-2.324097
H	-1.885796	0.851769	-2.501245
H	-3.521344	-1.583578	-1.364433
H	-4.327346	-2.477612	-0.069305
H	-2.977567	2.226698	-2.542073
H	-0.788311	0.510886	1.627838
H	0.317837	-1.652882	1.246592
H	-1.302615	-1.128861	-1.292457
H	1.736911	-0.654302	2.952267
H	2.942690	0.763714	2.880294
H	-1.648548	2.670508	0.882466
H	-0.023663	1.094980	-1.184187
H	-1.399785	3.172357	-0.792868
H	-3.051673	-2.918464	2.071654
H	-2.774285	-1.223147	2.456834
H	-1.400150	-2.324663	2.297977
H	-5.838714	0.727421	-0.659802
H	3.798710	-2.819719	-1.247900
H	2.635882	-2.063494	-2.359276
H	4.848048	-0.941679	-2.238961

H	4.031517	1.385564	-2.150966
H	2.861204	0.351015	-2.991068
H	2.557940	0.874697	-1.330614
H	5.510363	0.410014	-0.291018
H	5.288631	-1.277431	0.214693
H	4.055585	-0.064678	0.586167

ωB97XD energy = -1154.81971422 a.u.

(2*S*,6*R*,7*S*,8*R*)-**1**, Conf. L

C	-4.339768	-0.972119	-0.384430
C	-3.535346	0.221373	-0.818022
C	-3.174308	1.281614	-0.084263
C	-3.731156	1.631480	1.271293
C	-2.152879	-1.873917	0.465552
C	-3.410137	-2.210661	-0.298798
C	-0.729124	1.428109	-0.727245
C	-0.212583	0.722142	0.556958
C	0.120722	-0.777343	0.355172
C	-1.051746	-1.524874	-0.207969
O	0.287703	2.369318	-1.120197
C	1.270365	2.485010	-0.201521
C	0.998540	1.537284	0.916940
O	2.195303	3.245574	-0.331491
C	1.780725	1.467649	1.990252
C	-2.054930	2.188217	-0.570374
C	-2.295404	-1.751057	1.958876
O	-5.357203	-1.314793	-1.316596
O	1.202994	-0.847337	-0.584867
C	2.394323	-1.325611	-0.158253
O	2.570002	-1.771537	0.952538
C	3.425463	-1.227718	-1.256594
C	4.839487	-0.834974	-0.787496
C	4.824172	0.462427	0.026252
C	5.552041	-1.962150	-0.034818
H	-4.788748	-0.801142	0.602631
H	-3.080340	0.096106	-1.802209
H	-4.531018	0.965604	1.599620
H	-4.132010	2.651596	1.248354
H	-3.170181	-2.503384	-1.326094
H	-3.969062	-3.032841	0.160914
H	-2.945722	1.626862	2.037010
H	-0.968557	0.767654	1.346003
H	0.466188	-1.191649	1.305978
H	-1.044394	-1.628936	-1.291185
H	1.598733	0.754811	2.788521
H	2.641677	2.124489	2.070883
H	-1.897406	3.008914	0.138966
H	-0.808857	0.717446	-1.551920
H	-2.298590	2.645689	-1.534765
H	-2.789244	-2.638467	2.368271
H	-2.921847	-0.889149	2.221014
H	-1.336516	-1.634442	2.469023
H	-5.925355	-0.549408	-1.443462
H	3.456283	-2.208085	-1.749244
H	3.059674	-0.510230	-1.995863
H	5.400150	-0.644031	-1.711877

H	5.845440	0.803781	0.221911
H	4.294544	1.267326	-0.494516
H	4.335201	0.303985	0.993230
H	6.584684	-1.675422	0.189783
H	5.581742	-2.880841	-0.631156
H	5.042403	-2.185319	0.906000

ωB97XD energy = -1154.81962221 a.u.

(2*S*,6*R*,7*S*,8*R*)-**1**, Conf. M

C	-4.428034	-0.602589	0.223792
C	-3.559829	0.635978	0.330780
C	-2.898666	1.267388	-0.647326
C	-3.077870	0.988420	-2.116342
C	-2.235284	-1.821421	0.510740
C	-3.587731	-1.856736	-0.154440
C	-0.414222	1.608315	-0.361285
C	-0.068413	0.636313	0.801660
C	0.088742	-0.843675	0.391187
C	-1.187953	-1.369618	-0.187062
O	0.555735	2.674103	-0.311310
C	1.480012	2.500718	0.656889
C	1.184599	1.233508	1.382385
O	2.378029	3.280523	0.844673
C	1.980608	0.770633	2.342286
C	-1.808063	2.256045	-0.301719
C	-2.215795	-2.077467	1.993771
O	-5.465955	-0.528068	-0.745865
O	1.119594	-0.930204	-0.606163
C	2.217970	-1.667613	-0.327453
O	2.391147	-2.231175	0.730034
C	3.174450	-1.691000	-1.493106
C	4.446359	-0.839326	-1.271442
C	4.108557	0.631069	-1.016892
C	5.344534	-1.400929	-0.167202
H	-4.876203	-0.775521	1.212924
H	-3.333647	0.932671	1.355470
H	-3.360177	1.911237	-2.636636
H	-3.847585	0.240246	-2.304360
H	-3.471732	-1.847252	-1.242195
H	-4.161023	-2.751202	0.112363
H	-2.137325	0.652430	-2.570215
H	-0.874553	0.660843	1.544623
H	0.412010	-1.412773	1.265589
H	-1.312707	-1.182678	-1.251839
H	1.793512	-0.172887	2.844948
H	2.863455	1.336720	2.624639
H	-1.954141	2.674749	0.700219
H	-0.265492	1.114476	-1.326053
H	-1.803896	3.092021	-1.009748
H	-2.677382	-1.241932	2.537908
H	-1.207842	-2.215948	2.390947
H	-2.798538	-2.972487	2.234618
H	-5.991455	0.257625	-0.568637
H	3.461722	-2.736098	-1.645876
H	2.651319	-1.339436	-2.385707
H	4.996500	-0.903369	-2.219108

H	5.015021	1.243406	-1.018293
H	3.429078	1.031253	-1.776482
H	3.633176	0.761000	-0.039546
H	6.274666	-0.826780	-0.107869
H	5.601180	-2.448369	-0.355326
H	4.849838	-1.351461	0.807362

ωB97XD enerrgy = -1154.81899938 a.u.

(2*S*,6*R*,7*S*,8*R*)-**1**, Conf. N

C	-3.691348	-1.784276	-0.269752
C	-3.199825	-0.516373	-0.909813
C	-3.226398	0.723127	-0.403919
C	-4.004004	1.147861	0.815002
C	-1.460849	-1.831952	0.888772
C	-2.475944	-2.657344	0.136178
C	-0.856870	1.455840	-0.925131
C	-0.321697	1.199229	0.510965
C	0.417127	-0.149863	0.681427
C	-0.430917	-1.299407	0.223000
O	-0.105357	2.563140	-1.455883
C	0.692741	3.140722	-0.531383
C	0.575282	2.382569	0.747485
O	1.368640	4.107907	-0.770096
C	1.227003	2.745721	1.848555
C	-2.350783	1.804147	-1.016106
C	-1.805348	-1.490107	2.313471
O	-4.464372	-2.582927	-1.156015
O	1.597564	-0.106070	-0.133101
C	2.798498	-0.218757	0.476536
O	2.930296	-0.347066	1.672969
C	3.921128	-0.199908	-0.526942
C	4.364576	-1.621591	-0.950023
C	4.999623	-2.396215	0.207140
C	3.221767	-2.415207	-1.589784
H	-4.277371	-1.561600	0.631465
H	-2.622696	-0.689554	-1.819941
H	-4.634644	0.358443	1.227515
H	-4.651334	1.994248	0.557395
H	-2.041155	-3.054922	-0.786718
H	-2.841615	-3.505609	0.724742
H	-3.335104	1.502656	1.608819
H	-1.149444	1.184516	1.225942
H	0.726302	-0.246633	1.725164
H	-0.271408	-1.589113	-0.813973
H	1.160150	2.177178	2.770896
H	1.861983	3.626462	1.831921
H	-2.510487	2.759346	-0.502745
H	-0.637151	0.608427	-1.577493
H	-2.594607	1.967656	-2.070903
H	-2.085777	-2.394226	2.863898
H	-2.667968	-0.813059	2.352724
H	-0.980540	-1.012437	2.847193
H	-5.201689	-2.055946	-1.477514
H	3.600173	0.361815	-1.408075
H	4.763480	0.319477	-0.062658
H	5.135508	-1.465496	-1.715395

H	5.376223	-3.362242	-0.144362
H	5.836846	-1.843831	0.644913
H	4.274289	-2.583911	1.005164
H	3.595818	-3.360737	-1.994664
H	2.751237	-1.856869	-2.405289
H	2.444232	-2.653199	-0.855095

ωB97XD energy = -1154.81894311 a.u.

(2*R*,6*R*,7*S*,8*R*)-**1**, Conf. A

C	4.450234	-0.177111	0.439014
C	3.474599	0.907833	0.051391
C	2.643019	1.575627	0.857170
C	2.676001	1.479482	2.359621
C	2.471948	-1.689155	-0.064737
C	3.718563	-1.512892	0.766453
C	0.201334	1.530160	0.255544
C	0.123550	0.442171	-0.852080
C	0.057109	-1.004966	-0.313767
C	1.296258	-1.328929	0.462804
O	-0.906333	2.426606	0.035748
C	-1.676440	2.064947	-1.012944
C	-1.095775	0.843569	-1.636169
O	-2.663522	2.675926	-1.333557
C	-1.648723	0.260609	-2.696101
C	1.492178	2.364932	0.274961
C	2.677462	-2.053447	-1.509673
O	5.352270	-0.448061	-0.629093
O	-1.081813	-1.091729	0.555580
C	-2.119564	-1.879591	0.191092
O	-2.112130	-2.585290	-0.790681
C	-3.261762	-1.718407	1.159306
C	-3.954096	-0.344966	1.022722
C	-4.614112	-0.184700	-0.348045
C	-4.976629	-0.166108	2.146481
H	5.024427	0.109374	1.331487
H	3.350855	1.021374	-1.026301
H	3.546936	0.934932	2.729908
H	1.782071	0.972396	2.743713
H	3.448788	-1.495845	1.827256
H	4.443063	-2.319707	0.614093
H	2.685411	2.479862	2.805940
H	1.017798	0.495627	-1.483542
H	-0.098183	-1.688601	-1.151228
H	1.253732	-1.059456	1.516875
H	-1.241136	-0.644901	-3.134361
H	-2.546286	0.685420	-3.135611
H	1.714870	2.693273	-0.746389
H	0.027319	1.084332	1.239007
H	1.291860	3.261047	0.872354
H	3.189837	-1.242832	-2.041698
H	1.742992	-2.276999	-2.028923
H	3.330905	-2.927919	-1.588248
H	5.830778	0.358888	-0.840999
H	-3.978147	-2.522119	0.967675
H	-2.869323	-1.830710	2.175557

H	-3.185222	0.430261	1.133098
H	-5.057224	0.809807	-0.448736
H	-3.894789	-0.308406	-1.162037
H	-5.404504	-0.932720	-0.482904
H	-5.465000	0.809994	2.070221
H	-4.505432	-0.234094	3.132585
H	-5.755096	-0.936243	2.088975

ωB97XD energy = -1154.82278394 a.u.

(2*R*,6*R*,7*S*,8*R*)-**1**, Conf. B

C	-4.467888	-0.565907	-0.394324
C	-3.610691	0.657225	-0.175562
C	-2.840088	1.276638	-1.075057
C	-2.847888	0.959508	-2.547138
C	-2.348933	-1.777112	0.317866
C	-3.601577	-1.855003	-0.519623
C	-0.405307	1.595196	-0.493908
C	-0.207524	0.684544	0.750423
C	-0.016012	-0.814330	0.432682
C	-1.218754	-1.365482	-0.267766
O	0.576038	2.646933	-0.399132
C	1.387639	2.514605	0.671755
C	0.976236	1.307282	1.440434
O	2.284050	3.283742	0.905860
C	1.630821	0.908110	2.527409
C	-1.785505	2.260608	-0.623341
C	-2.522599	-1.964257	1.799755
O	-5.351503	-0.764708	0.704671
O	1.124646	-0.946007	-0.433687
C	2.203524	-1.615144	0.025380
O	2.271656	-2.101475	1.131469
C	3.300414	-1.679929	-1.006814
C	4.618391	-1.071727	-0.494891
C	5.733732	-1.316963	-1.512616
C	4.458619	0.421049	-0.199754
H	-5.055912	-0.475284	-1.318362
H	-3.510044	0.938596	0.873403
H	-3.641561	0.264007	-2.827366
H	-1.894815	0.516196	-2.861613
H	-3.327452	-1.972955	-1.572765
H	-4.240927	-2.698848	-0.239875
H	-2.975419	1.877358	-3.131554
H	-1.094213	0.747321	1.392142
H	0.200191	-1.342305	1.363620
H	-1.207441	-1.234969	-1.348712
H	1.357254	0.008673	3.069602
H	2.483537	1.481054	2.879577
H	-2.050434	2.708053	0.341028
H	-0.163842	1.046514	-1.409137
H	-1.682835	3.076594	-1.347172
H	-3.166622	-1.178328	2.211821
H	-1.575627	-1.964406	2.343736
H	-3.032510	-2.911851	2.000934
H	-5.910321	0.012774	0.795812
H	3.451969	-2.740646	-1.237114
H	2.976925	-1.174854	-1.921761

H	4.874870	-1.587439	0.438197
H	6.682431	-0.907985	-1.151275
H	5.877532	-2.385802	-1.702479
H	5.504374	-0.830211	-2.468179
H	5.399386	0.852264	0.155340
H	3.703874	0.605701	0.569973
H	4.158886	0.969431	-1.100731

ωB97XD energy = -1154.82191462 a.u.

(2*R*,6*R*,7*S*,8*R*)-**1**, Conf. C

C	4.478949	-0.685358	0.324674
C	3.665190	0.571362	0.131129
C	2.940942	1.216379	1.050921
C	2.972974	0.892562	2.521243
C	2.298291	-1.811880	-0.334324
C	3.565844	-1.938817	0.473875
C	0.507612	1.626968	0.528915
C	0.244424	0.733709	-0.715976
C	0.005556	-0.758836	-0.400013
C	1.197853	-1.358624	0.276848
O	-0.438695	2.712398	0.468055
C	-1.290872	2.611189	-0.574642
C	-0.939358	1.398908	-1.365779
O	-2.171256	3.408216	-0.772638
C	-1.636674	1.031830	-2.437382
C	1.913601	2.241473	0.628314
C	2.431496	-2.001394	-1.820228
O	5.322020	-0.917319	-0.799439
O	-1.128398	-0.839436	0.480521
C	-2.232250	-1.480048	0.041742
O	-2.311230	-2.016573	-1.039841
C	-3.331088	-1.451268	1.074444
C	-4.716087	-1.205171	0.459992
C	-5.800469	-1.384620	1.524187
C	-4.795327	0.183949	-0.178236
H	5.096974	-0.620542	1.231233
H	3.550044	0.860700	-0.914163
H	2.012513	0.482707	2.857971
H	3.148679	1.802256	3.105920
H	3.312173	-2.044003	1.533462
H	4.164085	-2.808146	0.181329
H	3.747518	0.167431	2.778957
H	1.113840	0.770671	-1.383033
H	-0.246155	-1.276964	-1.327600
H	1.214503	-1.231109	1.358125
H	-1.404979	0.131447	-2.997579
H	-2.479822	1.635754	-2.759638
H	2.172299	2.683991	-0.339946
H	0.271036	1.079161	1.445964
H	1.857857	3.056637	1.358076
H	3.085012	-1.231397	-2.247234
H	1.472249	-1.977016	-2.341601
H	2.913039	-2.960854	-2.034239
H	5.910119	-0.163591	-0.904799
H	-3.312805	-2.428163	1.574135
H	-3.098488	-0.694769	1.830405

H	-4.863872	-1.957162	-0.323875
H	-6.795253	-1.244818	1.089877
H	-5.764613	-2.383653	1.971382
H	-5.682743	-0.648824	2.328879
H	-5.777905	0.347842	-0.631311
H	-4.042724	0.311085	-0.962428
H	-4.635738	0.969161	0.570245

ωB97XD energy = -1154.82174371 a.u.

(2*R*,6*R*,7*S*,8*R*)-**1**, Conf. D

C	4.392587	-0.208397	0.344034
C	3.417932	0.887245	-0.014698
C	2.600688	1.548118	0.811047
C	2.653683	1.435189	2.311706
C	2.389960	-1.697045	-0.130366
C	3.657393	-1.542099	0.672938
C	0.137170	1.569477	0.247249
C	0.012987	0.469181	-0.844518
C	-0.024411	-0.981067	-0.314593
C	1.235221	-1.311140	0.423843
O	-0.926202	2.513450	0.005141
C	-1.731016	2.150876	-1.017694
C	-1.230411	0.871733	-1.591133
O	-2.687930	2.801300	-1.350901
C	-1.864571	0.248713	-2.580604
C	1.455230	2.361384	0.254475
C	2.557577	-2.071902	-1.577121
O	5.271163	-0.476263	-0.744330
O	-1.133290	-1.111207	0.591173
C	-2.152639	-1.928015	0.243786
O	-2.188929	-2.544213	-0.797236
C	-3.220715	-1.967756	1.308096
C	-4.150212	-0.730206	1.327665
C	-3.467631	0.523589	1.878225
C	-4.775327	-0.468967	-0.044427
H	4.986650	0.065664	1.227376
H	3.279194	1.014921	-1.089005
H	3.506836	0.851356	2.663050
H	1.745315	0.962780	2.705728
H	3.413275	-1.536503	1.740022
H	4.370698	-2.353099	0.493042
H	2.712211	2.430274	2.766471
H	0.882026	0.522653	-1.511642
H	-0.194497	-1.652627	-1.158371
H	1.225265	-1.036626	1.477493
H	-1.525057	-0.702801	-2.976755
H	-2.765504	0.687313	-2.999233
H	1.667641	2.689140	-0.769224
H	-0.058651	1.145119	1.237193
H	1.287391	3.260192	0.857877
H	3.110812	-1.291848	-2.113496
H	1.606587	-2.240942	-2.086743
H	3.157560	-2.983628	-1.661912
H	5.751270	0.329080	-0.958754
H	-3.813918	-2.863171	1.108676
H	-2.738925	-2.076344	2.285191

H	-4.957483	-0.998003	2.021578
H	-2.662326	0.862507	1.222382
H	-4.188496	1.342267	1.962092
H	-3.037589	0.342285	2.868978
H	-5.251907	-1.368696	-0.446163
H	-4.019236	-0.140720	-0.765315
H	-5.528612	0.321472	0.021217

ωB97XD energy = -1154.82162298 a.u.

(2*R*,6*R*,7*S*,8*R*)-**1**, Conf. E

C	-4.447648	-0.525184	-0.238961
C	-3.572597	0.673431	0.039152
C	-2.859467	1.376662	-0.846096
C	-2.965315	1.198480	-2.337715
C	-2.293330	-1.794311	0.206136
C	-3.596035	-1.790911	-0.555171
C	-0.388342	1.647954	-0.396629
C	-0.114846	0.621641	0.739188
C	0.045110	-0.841162	0.273392
C	-1.200621	-1.321309	-0.403672
O	0.595016	2.692768	-0.257365
C	1.467024	2.464225	0.747702
C	1.112769	1.177603	1.408743
O	2.368198	3.218841	1.009120
C	1.844671	0.669791	2.396565
C	-1.773626	2.315702	-0.373702
C	-2.374216	-2.130075	1.669728
O	-5.249844	-0.837227	0.895617
O	1.134536	-0.902756	-0.662998
C	2.208403	-1.659346	-0.343649
O	2.313804	-2.259658	0.702477
C	3.234699	-1.654164	-1.448790
C	4.505282	-0.839106	-1.113630
C	4.179699	0.626886	-0.821245
C	5.320669	-1.463206	0.021090
H	-5.101841	-0.344239	-1.103435
H	-3.402295	0.854191	1.101245
H	-2.043716	0.770588	-2.751970
H	-3.109410	2.167856	-2.827336
H	-3.389691	-1.796990	-1.630166
H	-4.217972	-2.662181	-0.324543
H	-3.789789	0.544384	-2.628675
H	-0.957238	0.624517	1.440985
H	0.305896	-1.453710	1.138943
H	-1.254951	-1.086254	-1.465422
H	1.613491	-0.285394	2.856902
H	2.718272	1.211053	2.747790
H	-1.973495	2.668132	0.644280
H	-0.203229	1.191738	-1.373758
H	-1.716900	3.198055	-1.020675
H	-2.891597	-3.084765	1.808024
H	-2.971091	-1.380715	2.203052
H	-1.394115	-2.202153	2.145998
H	-5.800319	-0.076283	1.102852
H	3.511869	-2.698082	-1.626845
H	2.774013	-1.256340	-2.356307

H	5.113951	-0.877038	-2.026501
H	3.649244	0.729168	0.130655
H	5.096092	1.219186	-0.744307
H	3.555501	1.070810	-1.603587
H	5.566758	-2.508498	-0.191871
H	4.767805	-1.440329	0.964911
H	6.257374	-0.914051	0.160332

ωB97XD energy = -1154.82084725 a.u.

(2*R*,6*R*,7*S*,8*R*)-**1**, Conf. F

C	4.000831	-1.311023	0.301625
C	3.448299	0.080273	0.498031
C	2.688193	0.515584	1.507436
C	2.404570	-0.285112	2.750922
C	1.810294	-1.657039	-0.936231
C	2.881828	-2.316130	-0.104263
C	0.485736	1.608365	0.925597
C	0.290836	1.256104	-0.576707
C	-0.228322	-0.171240	-0.850544
C	0.715601	-1.201903	-0.316095
O	-0.221626	2.843100	1.155987
C	-0.893730	3.276692	0.067558
C	-0.653012	2.327495	-1.054552
O	-1.564035	4.276714	0.075932
C	-1.252877	2.465620	-2.233875
C	1.937568	1.821474	1.385630
C	2.158895	-1.363347	-2.369567
O	4.969550	-1.325533	-0.741795
O	-1.496939	-0.308300	-0.190766
C	-2.583792	-0.586597	-0.942439
O	-2.550777	-0.724714	-2.143660
C	-3.814604	-0.700436	-0.080607
C	-3.688586	-1.758404	1.030990
C	-4.971625	-1.789969	1.863531
C	-3.370254	-3.137835	0.449069
H	4.460720	-1.687683	1.226034
H	3.569795	0.726300	-0.372418
H	2.963148	-1.222459	2.789844
H	1.338311	-0.531721	2.828197
H	2.436122	-2.710678	0.814401
H	3.368884	-3.145659	-0.627319
H	2.655930	0.298487	3.643731
H	1.253088	1.338549	-1.096234
H	-0.389236	-0.282840	-1.924620
H	0.567445	-1.446219	0.734687
H	-1.111081	1.754285	-3.041435
H	-1.925901	3.301806	-2.397511
H	2.432615	2.495154	0.677568
H	-0.005210	0.860801	1.556236
H	1.895502	2.334954	2.352486
H	1.304427	-1.012549	-2.952344
H	2.558242	-2.260564	-2.852208
H	2.953706	-0.609676	-2.423496
H	5.681264	-0.721234	-0.510728
H	-4.001276	0.283105	0.366447
H	-4.650943	-0.938779	-0.743578

H	-2.860206	-1.458454	1.683743
H	-4.890963	-2.521845	2.673385
H	-5.182732	-0.813023	2.310310
H	-5.831435	-2.071878	1.244144
H	-3.308830	-3.888732	1.242971
H	-2.415444	-3.142075	-0.087331
H	-4.150416	-3.452950	-0.253597

ωB97XD energy = -1154.82064091 a.u.

(2*R*,6*R*,7*S*,8*R*)-**1**, Conf. G

C	-4.235701	-1.340536	-0.312525
C	-3.700593	0.069071	-0.239023
C	-3.075072	0.748341	-1.205353
C	-2.965590	0.272233	-2.629793
C	-1.906967	-1.922284	0.518685
C	-3.082642	-2.387960	-0.303671
C	-0.802957	1.712670	-0.669867
C	-0.418002	1.022870	0.670069
C	0.119094	-0.418434	0.532987
C	-0.894084	-1.312986	-0.108496
O	-0.115047	2.979399	-0.696969
C	0.699100	3.159159	0.364845
C	0.589268	1.974184	1.260695
O	1.380071	4.142871	0.500847
C	1.329153	1.853347	2.359654
C	-2.299513	2.001509	-0.872185
C	-2.069124	-1.983251	2.012745
O	-5.063139	-1.624840	0.810937
O	1.297585	-0.370870	-0.289763
C	2.478109	-0.712421	0.269005
O	2.590108	-1.125335	1.400537
C	3.614129	-0.466231	-0.692111
C	4.885189	-1.257035	-0.369941
C	4.669207	-2.759459	-0.569690
C	6.047481	-0.751342	-1.227272
H	-4.812731	-1.496272	-1.234767
H	-3.699419	0.485890	0.768918
H	-3.307684	1.052017	-3.319229
H	-3.547939	-0.631188	-2.821572
H	-2.763494	-2.544146	-1.338914
H	-3.506308	-3.327459	0.066172
H	-1.923043	0.055240	-2.893907
H	-1.303780	0.961132	1.313827
H	0.409913	-0.776319	1.522712
H	-0.881878	-1.302350	-1.197254
H	1.279416	0.976742	2.997780
H	2.024181	2.643812	2.626392
H	-2.689646	2.474835	0.035628
H	-0.409536	1.142287	-1.516865
H	-2.378296	2.733882	-1.683183
H	-2.851010	-1.288400	2.342115
H	-1.146621	-1.752731	2.550056
H	-2.402223	-2.980840	2.315441
H	-5.793633	-0.999297	0.822351
H	3.266918	-0.670263	-1.711168
H	3.812221	0.613250	-0.646515
H	5.123812	-1.082258	0.685439
H	5.579167	-3.316534	-0.325216
H	3.865172	-3.136415	0.068599
H	4.415275	-2.977497	-1.614594
H	6.969104	-1.292010	-0.990353
H	6.232060	0.315735	-1.064967
H	5.840450	-0.899308	-2.294172

ωB97XD energy = -1154.82060391 a.u.

(2*R*,6*R*,7*S*,8*R*)-**1**, Conf. H

C	-3.944760	-1.576813	-0.222265
C	-3.517829	-0.137445	-0.379868
C	-2.859977	0.406057	-1.408590
C	-2.593173	-0.309200	-2.706455
C	-1.666708	-1.786494	0.882682
C	-2.729512	-2.502260	0.086816
C	-0.721213	1.651854	-0.900567
C	-0.407928	1.250233	0.568761
C	0.241384	-0.138368	0.742390
C	-0.649897	-1.222034	0.221360
O	-0.129801	2.950023	-1.109154
C	0.568056	3.389822	-0.039360
C	0.469942	2.375617	1.046888
O	1.153362	4.441823	-0.035056
C	1.119277	2.512831	2.199746
C	-2.211369	1.763513	-1.262799
C	-1.953991	-1.577040	2.343826
O	-4.857593	-1.714350	0.862274
O	1.469906	-0.142045	-0.004078
C	2.616874	-0.389751	0.661587
O	2.670618	-0.602991	1.851893
C	3.804840	-0.402550	-0.264066
C	4.402735	-1.816989	-0.393480
C	3.380256	-2.804337	-0.962664
C	5.664797	-1.771132	-1.256117
H	-4.417527	-1.952545	-1.140413
H	-3.633016	0.452993	0.529993
H	-2.914665	0.307653	-3.552999
H	-3.104639	-1.271481	-2.773457
H	-2.307163	-2.831154	-0.867987
H	-3.120977	-3.383286	0.605670
H	-1.520467	-0.494905	-2.843005
H	-1.341116	1.226440	1.144201
H	0.483424	-0.280870	1.797439
H	-0.544882	-1.416302	-0.844873
H	1.079097	1.758705	2.979392
H	1.726221	3.396806	2.370189
H	-2.714542	2.358306	-0.492428
H	-0.211851	0.976769	-1.594951
H	-2.273031	2.323573	-2.202347
H	-1.125161	-1.110012	2.879922
H	-2.174372	-2.535728	2.824233
H	-2.850554	-0.958971	2.472067
H	-5.625391	-1.160984	0.690918
H	3.511583	-0.023501	-1.247624
H	4.553330	0.274698	0.159257

H	4.679320	-2.146892	0.615275
H	3.821058	-3.801036	-1.062974
H	2.497668	-2.893757	-0.320836
H	3.043052	-2.483172	-1.955613
H	6.126157	-2.761296	-1.323897
H	6.407240	-1.080148	-0.843773
H	5.428458	-1.442673	-2.275378

ωB97XD energy = -1154.82044635 a.u.

(2*R*,6*R*,7*S*,8*R*)-**1**, Conf. I

C	3.800217	-1.613354	0.082633
C	3.421113	-0.160128	0.235041
C	2.828460	0.418349	1.284213
C	2.601840	-0.269607	2.604384
C	1.468398	-1.780919	-0.911960
C	2.547000	-2.509828	-0.149508
C	0.702400	1.715714	0.857591
C	0.308260	1.303192	-0.588919
C	-0.382791	-0.069628	-0.711561
C	0.504263	-1.168825	-0.215290
O	0.156121	3.031979	1.075141
C	-0.582551	3.474890	0.034324
C	-0.563645	2.443921	-1.040527
O	-1.140321	4.541687	0.042806
C	-1.263680	2.582314	-2.163111
C	2.210919	1.791133	1.145829
C	1.687525	-1.617751	-2.390412
O	4.653851	-1.794660	-1.042877
O	-1.571526	-0.029373	0.093540
C	-2.746492	-0.354561	-0.486997
O	-2.861446	-0.617544	-1.662959
C	-3.861130	-0.383856	0.525950
C	-4.087964	-1.797642	1.115892
C	-4.611102	-2.781337	0.066533
C	-2.836608	-2.342699	1.810726
H	4.307300	-1.986537	0.983414
H	3.509607	0.412140	-0.689293
H	3.107947	-1.234786	2.671692
H	1.532991	-0.444627	2.780738
H	2.160516	-2.805580	0.831009
H	2.888986	-3.412791	-0.665608
H	2.956920	0.360216	3.427420
H	1.211467	1.248299	-1.208492
H	-0.678520	-0.219659	-1.751822
H	0.448509	-1.335898	0.859350
H	-1.277645	1.818016	-2.933784
H	-1.855579	3.479276	-2.317609
H	2.692264	2.358685	0.341665
H	0.209399	1.063477	1.584955
H	2.333469	2.364072	2.071450
H	1.851577	-2.595152	-2.855628
H	2.596461	-1.034510	-2.579637
H	0.847811	-1.136547	-2.896200
H	5.444656	-1.261308	-0.919213
H	-3.625115	0.315950	1.332072
H	-4.771573	-0.049925	0.021796

H	-4.867369	-1.669649	1.877954
H	-4.831225	-3.748701	0.529143
H	-5.527340	-2.411167	-0.403651
H	-3.874473	-2.943938	-0.726774
H	-3.066589	-3.279371	2.328005
H	-2.444086	-1.632606	2.545825
H	-2.040387	-2.550361	1.087010

ωB97XD energy = -1154.81959221 a.u.

(2*R*,6*R*,7*S*,8*R*)-**1**, Conf. J

C	-4.162286	-1.414930	-0.272899
C	-3.669333	0.007044	-0.287395
C	-3.024264	0.627907	-1.278334
C	-2.855122	0.056174	-2.661599
C	-1.825315	-1.895797	0.610303
C	-2.968967	-2.427652	-0.215415
C	-0.800568	1.695618	-0.755625
C	-0.437175	1.098394	0.633264
C	0.150461	-0.329075	0.595377
C	-0.813626	-1.291236	-0.023291
O	-0.142950	2.975668	-0.840979
C	0.632711	3.242102	0.230885
C	0.522002	2.112858	1.196899
O	1.285615	4.249388	0.325645
C	1.223496	2.083347	2.326734
C	-2.298308	1.926383	-1.013447
C	-2.021338	-1.879832	2.101652
O	-5.002491	-1.524281	0.869624
O	1.351817	-0.284591	-0.193609
C	2.517712	-0.600635	0.407386
O	2.600136	-0.984916	1.551935
C	3.687901	-0.383217	-0.518756
C	4.789512	-1.437640	-0.354833
C	4.294840	-2.817881	-0.795565
C	6.036212	-1.023055	-1.138388
H	-4.744802	-1.638741	-1.178285
H	-3.745263	0.498837	0.681451
H	-3.197139	0.777681	-3.411899
H	-3.411274	-0.872028	-2.808177
H	-2.628556	-2.604008	-1.241156
H	-3.348678	-3.382188	0.171916
H	-1.800508	-0.150623	-2.882996
H	-1.341412	1.043479	1.251434
H	0.421201	-0.620273	1.612319
H	-0.772003	-1.338161	-1.110075
H	1.171433	1.249598	3.019922
H	1.886759	2.909031	2.566441
H	-2.726648	2.445582	-0.149110
H	-0.368710	1.086397	-1.555478
H	-2.377461	2.600278	-1.873708
H	-2.802844	-1.162873	2.380084
H	-1.107441	-1.629544	2.644749
H	-2.364617	-2.859485	2.449915
H	-5.390631	-2.403170	0.891590
H	3.329902	-0.343621	-1.552491
H	4.080834	0.613535	-0.279385

H	5.041790	-1.486273	0.710657
H	5.075078	-3.572953	-0.657569
H	3.420374	-3.134859	-0.218553
H	4.021035	-2.810167	-1.857883
H	6.829248	-1.769467	-1.029096
H	6.428007	-0.061990	-0.789138
H	5.814054	-0.927392	-2.208113

ωB97XD energy = -1154.81943578 a.u.

(2*R*,6*R*,7*S*,8*S*)-**1**, Conf. A

C	2.208545	-3.162266	-0.306593
C	2.658358	-1.779261	-0.708772
C	3.263946	-0.876739	0.068846
C	3.783219	-1.180964	1.449849
C	0.083623	-1.894634	0.264121
C	0.928665	-3.104324	0.585815
C	2.180764	1.379028	0.325477
C	0.757549	1.210010	-0.288345
C	-0.228242	0.598449	0.735770
C	0.173827	-0.819607	1.058336
O	2.514718	2.776196	0.230939
C	1.542481	3.511353	-0.351848
C	0.417872	2.608490	-0.729017
O	1.639761	4.699491	-0.516292
C	-0.648668	3.068642	-1.379583
C	3.307372	0.575923	-0.346132
C	-0.665126	-1.961073	-1.040010
O	1.886065	-3.944659	-1.452696
O	-1.545555	0.688083	0.175525
C	-2.571762	0.761486	1.050931
O	-2.420962	0.806008	2.249123
C	-3.895930	0.758908	0.331960
C	-4.151584	-0.546272	-0.446162
C	-4.038576	-1.769076	0.467145
C	-5.521172	-0.484281	-1.124653
H	2.987655	-3.678512	0.271154
H	2.281700	-1.457265	-1.680111
H	3.869928	-2.253160	1.638143
H	3.124241	-0.766145	2.222957
H	1.245873	-3.063194	1.632330
H	0.390576	-4.045600	0.431693
H	4.769293	-0.728298	1.597941
H	0.804902	0.536382	-1.150489
H	-0.216238	1.199732	1.649953
H	0.741938	-0.917696	1.981200
H	-1.460153	2.414401	-1.676473
H	-0.710464	4.126645	-1.617063
H	3.217388	0.681382	-1.432841
H	2.163168	1.155548	1.397412
H	4.253515	1.039152	-0.046095
H	-1.094911	-1.004368	-1.333785
H	-1.483660	-2.685951	-0.952313
H	-0.011658	-2.334976	-1.834620
H	2.665122	-4.003744	-2.013702
H	-4.672083	0.908939	1.087593
H	-3.919462	1.609166	-0.359427
H	-3.384754	-0.626365	-1.225823
H	-3.043836	-1.851435	0.918404
H	-4.770490	-1.713496	1.281116
H	-4.228474	-2.689365	-0.094511
H	-6.320050	-0.394680	-0.379195

ωB97XD energy = -1154.81683060 a.u.

(2*R*,6*R*,7*S*,8*S*)-**1**, Conf. B

C	4.094440	-1.636245	0.368128
C	3.773741	-0.157684	0.317789
C	3.325785	0.561010	-0.719070
C	3.313222	0.093554	-2.148000
C	1.573765	-1.845496	0.687043
C	2.820914	-2.511839	0.159107
C	1.363780	1.741918	0.332783
C	0.145747	1.169195	-0.452466
C	-0.286758	-0.207488	0.102341
C	0.770936	-1.225478	-0.184719
O	0.972070	3.036402	0.825929
C	-0.304016	3.352798	0.519089
C	-0.888702	2.253831	-0.301990
O	-0.820847	4.380337	0.873566
C	-2.119852	2.348579	-0.799950
C	2.680070	1.907596	-0.440527
C	1.418730	-1.785593	2.181946
O	5.026321	-2.084656	-0.610026
O	-1.502919	-0.566457	-0.580903
C	-2.386064	-1.312814	0.110090
O	-2.214433	-1.657799	1.257555
C	-3.603706	-1.650985	-0.713707
C	-4.912105	-1.304657	0.014851
C	-5.024112	0.202349	0.259419
C	-6.109332	-1.823882	-0.782982
H	4.497344	-1.848621	1.368430
H	3.732089	0.314728	1.300163
H	3.716274	-0.913438	-2.258889
H	3.916393	0.772641	-2.763033
H	2.992171	-3.487743	0.625902
H	2.727369	-2.672736	-0.918668
H	2.297546	0.124586	-2.560637
H	0.402419	1.040989	-1.510221
H	-0.505625	-0.119605	1.168885
H	0.977767	-1.343715	-1.247788
H	-2.547263	1.571839	-1.422650
H	-2.714340	3.229669	-0.576018
H	2.487643	2.457067	-1.368938
H	1.557768	1.128385	1.218598
H	3.333326	2.533050	0.176059
H	1.620244	-2.769141	2.618594
H	0.415210	-1.484710	2.488141
H	2.143422	-1.088354	2.623392
H	5.821575	-1.548742	-0.537191
H	-3.549956	-1.138253	-1.679306
H	-3.562690	-2.729421	-0.907238
H	-4.888624	-1.812577	0.985821

H	-5.951159	0.441937	0.789022
H	-4.191352	0.580033	0.861715
H	-5.032386	0.749074	-0.691771
H	-7.047091	-1.612510	-0.259966

ωB97XD energy = -1154.81617638 a.u.

(2*R*,6*R*,7*S*,8*S*)-**1**, Conf. C

C	2.759766	-2.817992	-0.373862
C	2.917366	-1.454200	-0.976670
C	3.198759	-0.340948	-0.290177
C	3.706203	-0.340122	1.133507
C	0.595822	-1.956667	0.647339
C	1.250489	-3.088239	-0.112624
C	1.811732	1.762141	-0.051944
C	0.310959	1.545659	-0.409243
C	-0.363107	0.414781	0.389996
C	0.123897	-0.926204	-0.063958
O	2.052472	3.172143	-0.231769
C	0.905970	3.884808	-0.198171
C	-0.247138	2.936748	-0.203876
O	0.889669	5.088019	-0.186359
C	-1.490723	3.397426	-0.082994
C	2.894971	1.024911	-0.857955
C	0.640933	-2.042988	2.146230
O	3.188441	-3.864105	-1.234828
O	-1.777975	0.492841	0.097676
C	-2.613764	-0.059766	1.000390
O	-2.251878	-0.467117	2.080514
C	-4.025603	-0.109756	0.474759
C	-4.149881	-0.912221	-0.834753
C	-3.578273	-2.323752	-0.677264
C	-5.612363	-0.952009	-1.281175
H	3.292632	-2.884317	0.584112
H	2.541568	-1.365896	-1.996683
H	4.306055	-1.223838	1.361024
H	2.883666	-0.310300	1.857483
H	1.166093	-4.040231	0.421487
H	0.780299	-3.217816	-1.093041
H	4.328478	0.541865	1.316598
H	0.235335	1.298966	-1.477791
H	-0.224646	0.577790	1.461886
H	0.129574	-1.024688	-1.149480
H	-2.354521	2.746972	-0.110609
H	-1.638013	4.466173	0.042610
H	2.596533	0.961738	-1.909589
H	1.950745	1.564460	1.017968
H	3.787149	1.660140	-0.806398
H	0.272433	-1.143354	2.640008
H	1.659208	-2.256484	2.492431
H	0.011153	-2.875355	2.479564
H	4.109689	-3.711620	-1.463927
H	-4.644635	-0.554426	1.258989
H	-4.373037	0.917369	0.309872
H	-3.566371	-0.387968	-1.600978
H	-3.697102	-2.891756	-1.605213
H	-2.510903	-2.304637	-0.431644
H	-4.097520	-2.866636	0.121258
H	-5.712194	-1.472233	-2.238843

ωB97XD energy = -1154.81597232 a.u.

(2*R*,6*R*,7*S*,8*S*)-**1**, Conf. D

C	3.958502	-1.677525	-0.424535
C	3.471791	-0.362800	-0.959096
C	3.436277	0.806315	-0.309457
C	4.185331	1.125655	0.959486
C	1.618823	-1.831515	0.532267
C	2.727128	-2.587168	-0.165975
C	1.181731	1.802152	0.086079
C	-0.052165	1.085967	-0.551238
C	-0.355134	-0.247014	0.174300
C	0.657789	-1.278918	-0.215229
O	0.763471	3.141170	0.413542
C	-0.546856	3.357559	0.173076
C	-1.128328	2.136197	-0.451813
O	-1.089951	4.400569	0.429717
C	-2.388348	2.125030	-0.879824
C	2.475849	1.887014	-0.754036
C	1.781644	-1.619981	2.011074
O	4.780484	-2.390315	-1.339568
O	-1.657275	-0.701376	-0.248025
C	-2.305489	-1.509269	0.619168
O	-1.888066	-1.761304	1.726440
C	-3.581073	-2.063425	0.034392
C	-4.544295	-1.013056	-0.541153
C	-5.792299	-1.703077	-1.095896
C	-4.912960	0.036912	0.508799
H	4.494778	-1.536786	0.522390
H	2.907288	-0.449185	-1.888409
H	4.832631	1.993251	0.786960
H	4.812904	0.305861	1.312091
H	3.054312	-3.459795	0.408978
H	2.387937	-2.943097	-1.144259
H	3.504775	1.405685	1.772126
H	0.147292	0.861612	-1.605369
H	-0.392441	-0.076433	1.252261
H	0.647303	-1.498206	-1.283378
H	-2.817676	1.261622	-1.371893
H	-3.005062	3.006463	-0.730414
H	2.244535	1.802356	-1.820484
H	1.413601	1.325680	1.044852
H	2.902274	2.881055	-0.584057
H	0.894495	-1.187336	2.476461
H	2.640402	-0.967879	2.215319
H	1.983887	-2.576394	2.504204
H	5.532756	-1.836455	-1.567319
H	-3.296781	-2.772731	-0.752505
H	-4.069086	-2.629869	0.832780
H	-4.036260	-0.514352	-1.375552
H	-6.475188	-0.973068	-1.541350
H	-5.534394	-2.438858	-1.864558
H	-6.334713	-2.224184	-0.298200
H	-5.595967	0.783524	0.091280

ωB97XD energy = -1154.81592944 a.u.

(2*R*,6*R*,7*S*,8*S*)-**1**, Conf. E

C	-4.114427	-1.463613	-0.215824
C	-3.724894	-0.003307	-0.130437
C	-3.155945	0.647470	0.891791
C	-3.035888	0.112361	2.291694
C	-1.645183	-1.757021	-0.776618
C	-2.866351	-2.396975	-0.162397
C	-1.242540	1.794031	-0.281402
C	0.014888	1.137658	0.368175
C	0.333243	-0.221185	-0.296377
C	-0.743888	-1.203954	0.041766
O	-0.839357	3.092003	-0.755591
C	0.466695	3.350824	-0.534939
C	1.070236	2.197047	0.191612
O	0.993664	4.375121	-0.883974
C	2.331482	2.236543	0.614450
C	-2.477214	1.977696	0.613342
C	-1.621560	-1.641846	-2.275619
O	-4.974429	-1.920533	0.822139
O	1.589014	-0.683049	0.240888
C	2.274095	-1.554798	-0.529707
O	1.927919	-1.864389	-1.646924
C	3.491880	-2.095512	0.177302
C	4.440104	-1.024887	0.740901
C	5.618327	-1.696569	1.449068
C	4.922590	-0.076932	-0.359097
H	-4.612682	-1.612339	-1.184195
H	-3.748781	0.511877	-1.091781
H	-1.985542	0.073718	2.605198
H	-3.478874	-0.878326	2.396373
H	-3.120772	-3.343911	-0.650206
H	-2.682941	-2.608258	0.895126
H	-3.541533	0.790179	2.990182
H	-0.160044	0.960349	1.435382
H	0.452520	-0.081706	-1.373183
H	-0.863414	-1.357077	1.113730
H	2.772754	1.419762	1.172125
H	2.937613	3.110303	0.393495
H	-2.180497	2.477451	1.542357
H	-1.540152	1.228938	-1.170587
H	-3.153468	2.655885	0.083531
H	-1.899269	-2.599398	-2.727960
H	-0.637940	-1.365648	-2.659404
H	-2.355589	-0.902511	-2.623232
H	-5.750651	-1.353135	0.842696
H	3.137153	-2.738114	0.992388
H	4.017768	-2.729345	-0.542350
H	3.882455	-0.445048	1.486536
H	6.288384	-0.948958	1.884785
H	5.278471	-2.356562	2.253959
H	6.204071	-2.298801	0.744661
H	5.590210	0.688064	0.049925

ωB97XD energy = -1154.81587062 a.u.

(2*R*,6*R*,7*S*,8*S*)-**1**, Conf. F

C	3.878486	-1.951832	-0.284486
C	3.666153	-0.539031	-0.738910
C	3.431440	0.498645	0.071965
C	3.654729	0.461532	1.566481
C	1.398512	-1.979370	0.288617
C	2.532496	-2.725462	-0.378628
C	1.382207	1.975220	0.163655
C	0.141057	1.358028	-0.546747
C	-0.251248	-0.034637	-0.015216
C	0.737332	-1.073923	-0.441781
O	1.153932	3.398825	0.180239
C	-0.149854	3.701019	0.003639
C	-0.885438	2.455184	-0.364697
O	-0.578681	4.819341	0.121162
C	-2.208444	2.484875	-0.515761
C	2.779806	1.759836	-0.442852
C	1.192237	-2.245601	1.752736
O	4.788154	-2.674485	-1.103033
O	-1.528722	-0.348334	-0.619042
C	-2.329081	-1.195794	0.053006
O	-2.055828	-1.654935	1.139217
C	-3.594257	-1.489733	-0.715940
C	-4.846809	-1.471998	0.171315
C	-5.068659	-0.084999	0.780539
C	-6.066745	-1.918508	-0.636585
H	4.219483	-1.976654	0.759318
H	3.467330	-0.432024	-1.805768
H	4.472225	-0.205636	1.847622
H	2.759914	0.122869	2.101410
H	2.682400	-3.715547	0.064378
H	2.326980	-2.868744	-1.444365
H	3.895126	1.461306	1.942319
H	0.358840	1.261813	-1.619647
H	-0.388049	0.002702	1.068558
H	0.975811	-1.014933	-1.503595
H	-2.780548	1.616151	-0.811967
H	-2.727214	3.423009	-0.340427
H	2.713189	1.757301	-1.535742
H	1.382837	1.656515	1.213183
H	3.370169	2.635998	-0.150033
H	0.843090	-3.275242	1.888358
H	0.449022	-1.590959	2.209101
H	2.138472	-2.157185	2.299751
H	5.628023	-2.206506	-1.111391
H	-3.692284	-0.776903	-1.541581
H	-3.465113	-2.483680	-1.162295
H	-4.682073	-2.185721	0.986605
H	-5.957158	-0.078384	1.419375
H	-4.218122	0.228240	1.394414
H	-5.219296	0.665277	-0.005972
H	-6.961758	-1.944140	-0.007252

ωB97XD energy = -1154.81586066 a.u.

(2*R*,6*R*,7*S*,8*S*)-**1**, Conf. G

C	3.599737	-2.010942	-0.441978

C	3.303561	-0.579541	-0.817046
C	3.454407	0.501061	-0.045606
C	4.153425	0.485156	1.288434
C	1.167306	-1.935857	0.288280
C	2.514874	-2.575066	0.527533
C	1.429310	1.943609	0.335349
C	0.235209	1.112275	-0.225660
C	-0.283290	0.100090	0.823992
C	0.781260	-0.935863	1.090934
O	1.038068	3.328004	0.283321
C	-0.191305	3.507028	-0.247547
C	-0.747412	2.177777	-0.628989
O	-0.691246	4.594546	-0.372282
C	-1.911178	2.075667	-1.266515
C	2.766768	1.796643	-0.410174
C	0.455604	-2.391204	-0.958743
O	3.609572	-2.846262	-1.595998
O	-1.497086	-0.472333	0.316997
C	-2.314260	-1.044223	1.228279
O	-2.116079	-0.993060	2.419153
C	-3.458745	-1.773272	0.568441
C	-4.185999	-0.995557	-0.538249
C	-5.278914	-1.870330	-1.156198
C	-4.763879	0.317340	-0.005265
H	4.569735	-2.094352	0.067213
H	2.753411	-0.474656	-1.752829
H	4.679864	-0.452400	1.477307
H	3.442155	0.632681	2.110788
H	2.839932	-2.374977	1.553038
H	2.492822	-3.660781	0.385290
H	4.880184	1.302459	1.348727
H	0.561410	0.540826	-1.101074
H	-0.525394	0.629442	1.750923
H	1.393752	-0.722029	1.964610
H	-2.311810	1.116973	-1.573293
H	-2.479192	2.975533	-1.483886
H	2.581723	1.859118	-1.488165
H	1.581050	1.725862	1.397511
H	3.384458	2.657398	-0.131225
H	1.152113	-2.410587	-1.803350
H	-0.410447	-1.779014	-1.207703
H	0.113985	-3.424729	-0.823375
H	4.283180	-2.524679	-2.202603
H	-3.040754	-2.699385	0.152656
H	-4.155316	-2.055881	1.362999
H	-3.452852	-0.765180	-1.320858
H	-4.864882	-2.797143	-1.566920
H	-6.031951	-2.140019	-0.406613
H	-5.789711	-1.341451	-1.966958
H	-5.499580	0.122823	0.783610

ωB97XD energy = -1154.81574253 a.u.

(2*R*,6*R*,7*S*,8*S*)-**1**, Conf. H

C	2.998130	-2.642113	0.359193
C	3.269158	-1.179892	0.076428
C	2.926422	-0.464366	-1.001995
C	2.434036	-1.043083	-2.299551
C	0.716928	-1.755408	1.068054
C	1.472173	-2.931387	0.499177
C	1.841764	1.548732	0.072132
C	0.359569	1.443891	-0.400352
C	-0.437785	0.435725	0.455043
C	0.074764	-0.949519	0.216049
O	2.097763	2.940467	0.335674
C	1.008473	3.719135	0.161217
C	-0.123697	2.867410	-0.304355
O	1.021707	4.906102	0.359480
C	-1.311425	3.397383	-0.588598
C	2.926349	1.050679	-0.895148
C	0.888988	-1.482203	2.536451
O	3.463990	-3.545257	-0.637326
O	-1.807290	0.508363	0.016214
C	-2.743219	0.101034	0.899990
O	-2.497380	-0.159360	2.055145
C	-4.093742	-0.013856	0.241878
C	-4.106286	-1.067534	-0.883910
C	-3.628014	-2.429841	-0.374372
C	-5.505602	-1.164426	-1.493527
H	3.482212	-2.884283	1.315777
H	3.619840	-0.623962	0.947080
H	2.364305	-2.130741	-2.270889
H	3.119051	-0.764466	-3.109575
H	1.345777	-3.833679	1.106789
H	1.105521	-3.156427	-0.506466
H	1.455898	-0.624137	-2.564389
H	0.319220	1.107150	-1.442885
H	-0.392075	0.728040	1.506850
H	0.008895	-1.253619	-0.828157
H	-2.135419	2.792129	-0.947337
H	-1.459699	4.464854	-0.454575
H	2.776881	1.519959	-1.874173
H	1.963205	1.031090	1.029465
H	3.883697	1.413195	-0.507398
H	1.900000	-1.109714	2.749387
H	0.766670	-2.409798	3.104827
H	0.163558	-0.759625	2.914629
H	4.401034	-3.381477	-0.778861
H	-4.813978	-0.277321	1.021218
H	-4.371206	0.964135	-0.168038
H	-3.411170	-0.727456	-1.661119
H	-3.653342	-3.172871	-1.177554
H	-2.602602	-2.385825	0.008241
H	-4.271785	-2.787649	0.437548
H	-5.520020	-1.884499	-2.317599

ωB97XD energy = -1154.81572217 a.u.

(2*R*,6*R*,7*S*,8*S*)-**1**, Conf. I

C	3.819218	-1.920442	-0.302895
C	3.506903	-0.483588	-0.641383
C	3.521860	0.559592	0.193492
C	4.067007	0.499592	1.596315
C	1.319124	-1.968318	0.147258

C	2.655060	-2.576390	0.503354
C	1.415387	1.918693	0.417262
C	0.315624	1.078757	-0.300837
C	-0.259053	-0.015729	0.630295
C	0.808387	-1.030053	0.954911
O	0.985976	3.292303	0.377892
C	-0.198517	3.455470	-0.251054
C	-0.676446	2.126816	-0.728512
O	-0.721994	4.531820	-0.376921
C	-1.796358	2.011825	-1.439091
C	2.827868	1.846661	-0.187021
C	0.765553	-2.373758	-1.192949
O	3.999106	-2.692672	-1.486647
O	-1.391930	-0.588684	-0.039282
C	-2.355350	-1.116948	0.742093
O	-2.310454	-1.111512	1.950411
C	-3.487693	-1.689097	-0.073307
C	-4.838552	-1.049003	0.291037
C	-4.843711	0.448398	-0.026474
C	-5.973481	-1.769694	-0.438423
H	4.726702	-1.993682	0.312081
H	3.060392	-0.351068	-1.627527
H	3.263267	0.570140	2.339906
H	4.741900	1.342493	1.780728
H	2.854584	-2.425338	1.568632
H	2.693121	-3.652082	0.301473
H	4.614237	-0.423740	1.796421
H	0.745331	0.577748	-1.174960
H	-0.621387	0.447295	1.553454
H	1.310746	-0.849217	1.903186
H	-2.142984	1.053540	-1.807435
H	-2.382451	2.900834	-1.653427
H	2.752798	1.951309	-1.274897
H	1.460967	1.658045	1.479901
H	3.380473	2.713756	0.190859
H	1.552416	-2.340662	-1.953383
H	-0.080971	-1.765581	-1.509264
H	0.432089	-3.417621	-1.144943
H	4.721227	-2.311441	-1.994957
H	-3.278561	-1.560831	-1.140156
H	-3.521593	-2.763881	0.137548
H	-4.978053	-1.176271	1.370906
H	-4.052215	0.983045	0.509111
H	-4.697864	0.616279	-1.100674
H	-5.798609	0.902341	0.255027
H	-6.942854	-1.338646	-0.169960

ωB97XD energy = -1154.81570289 a.u.

(2*R*,6*R*,7*S*,8*S*)-**1**, Conf. J

C	3.120148	-2.646826	0.405483
C	3.381767	-1.164585	0.245198
C	3.171189	-0.393755	-0.828971
C	2.880333	-0.906609	-2.212033
C	0.743186	-1.857329	0.865427
C	1.598379	-2.976483	0.323917
C	1.908747	1.529105	0.213535
C	0.498961	1.420547	-0.443267
C	-0.368629	0.353629	0.260678
C	0.189487	-1.008281	-0.007014
O	2.090185	2.907403	0.587493
C	1.010912	3.672735	0.318713
C	-0.027727	2.827873	-0.337997
O	0.965200	4.845633	0.584537
C	-1.177770	3.353508	-0.754867
C	3.115167	1.112798	-0.640533
C	0.723473	-1.680159	2.358678
O	3.740054	-3.477472	-0.569959
O	-1.691919	0.452465	-0.301227
C	-2.710803	0.084008	0.499158
O	-2.561949	-0.274097	1.645955
C	-4.041564	0.174699	-0.204148
C	-4.835936	-1.137998	-0.100162
C	-6.227316	-0.958870	-0.709361
C	-4.081742	-2.290079	-0.769311
H	3.481004	-2.936108	1.402665
H	3.590829	-0.654253	1.186433
H	3.651510	-0.552381	-2.907020
H	1.927429	-0.508847	-2.582396
H	1.421030	-3.921002	0.849179
H	1.373287	-3.139348	-0.734078
H	2.856636	-1.995705	-2.256204
H	0.595594	1.138393	-1.498166
H	-0.432711	0.578542	1.327660
H	0.264866	-1.241174	-1.068580
H	-1.931470	2.756933	-1.255029
H	-1.365691	4.410238	-0.588547
H	3.069017	1.631974	-1.604504
H	1.927216	0.957426	1.147356
H	4.009764	1.473990	-0.123596
H	-0.069505	-1.007059	2.689472
H	1.685857	-1.293734	2.720524
H	0.568008	-2.647566	2.847076
H	4.681583	-3.281643	-0.578562
H	-4.602020	0.985389	0.276029
H	-3.885420	0.446281	-1.252897
H	-4.945620	-1.367664	0.966081
H	-6.814161	-1.877345	-0.610998
H	-6.779266	-0.151037	-0.217659
H	-6.157668	-0.720392	-1.777554
H	-4.645817	-3.224119	-0.685203

ωB97XD energy = -1154.81559694 a.u.

(2*R*,6*R*,7*S*,8*S*)-**1**, Conf. K

C	3.359307	-2.507171	0.360346
C	3.523293	-1.004729	0.274853
C	3.296183	-0.201361	-0.771779
C	3.086608	-0.668737	-2.185413
C	0.916482	-1.898299	0.755558
C	1.867486	-2.935604	0.210548
C	1.856594	1.575835	0.292955
C	0.491041	1.397847	-0.437271
C	-0.333816	0.245601	0.179827

C	0.331136	-1.062843	-0.109225
O	1.916648	2.943279	0.739146
C	0.797922	3.641434	0.449784
C	-0.138405	2.759654	-0.304710
O	0.650265	4.792306	0.769524
C	-1.295527	3.224795	-0.771072
C	3.127275	1.287007	-0.519331
C	0.832116	-1.774764	2.251989
O	4.066578	-3.247300	-0.628692
O	-1.630191	0.280036	-0.448030
C	-2.668457	-0.165719	0.286523
O	-2.556527	-0.568353	1.421909
C	-3.965433	-0.048920	-0.477101
C	-5.048509	-1.025513	-0.007662
C	-6.382522	-0.687884	-0.677271
C	-4.644409	-2.474981	-0.289415
H	3.707674	-2.817876	1.355489
H	3.665801	-0.524779	1.244118
H	2.119099	-0.323601	-2.570191
H	3.143961	-1.753540	-2.278393
H	1.736364	-3.908004	0.697165
H	1.691203	-3.075573	-0.859839
H	3.851292	-0.228388	-2.836986
H	0.656486	1.166815	-1.495844
H	-0.465966	0.422379	1.249512
H	0.458761	-1.254047	-1.174077
H	-1.971073	2.602153	-1.345683
H	-1.567111	4.257699	-0.573535
H	3.088755	1.847757	-1.460128
H	1.876438	0.962124	1.199785
H	3.969748	1.682311	0.056830
H	1.755361	-1.345486	2.663687
H	0.716670	-2.765924	2.702679
H	-0.011293	-1.161940	2.575157
H	4.992440	-2.989443	-0.593911
H	-4.306328	0.986705	-0.343172
H	-3.760935	-0.173803	-1.546283
H	-5.157336	-0.902718	1.075918
H	-7.170820	-1.364779	-0.333890
H	-6.697343	0.337119	-0.454349
H	-6.308589	-0.789114	-1.766959
H	-5.417926	-3.167729	0.056289

ωB97XD energy = -1154.81552727 a.u.

(2*R*,6*R*,7*S*,8*S*)-**1**, Conf. L

C	2.683570	-2.984512	-0.316530
C	3.024485	-1.547382	-0.625224
C	3.456200	-0.623972	0.238843
C	3.874063	-0.934534	1.652522
C	0.391447	-1.963359	0.078023
C	1.328075	-3.088488	0.449766
C	2.133927	1.508472	0.460758
C	0.799021	1.241008	-0.299360
C	-0.216137	0.470837	0.577879
C	0.309659	-0.905858	0.895131
O	2.344803	2.932876	0.448861
C	1.359705	3.605617	-0.184936
C	0.361538	2.625216	-0.699502
O	1.356676	4.804706	-0.287588
C	-0.689275	3.024350	-1.413331
C	3.392871	0.842761	-0.119784
C	-0.247497	-2.082093	-1.280290
O	2.550998	-3.744128	-1.514385
O	-1.455574	0.442479	-0.144426
C	-2.577667	0.274685	0.584165
O	-2.578529	0.172566	1.788263
C	-3.799977	0.275927	-0.302256
C	-5.012748	-0.422769	0.319624
C	-6.261204	-0.154602	-0.523635
C	-4.764245	-1.925410	0.473877
H	3.453875	-3.446494	0.316237
H	2.709526	-1.223007	-1.617768
H	4.007794	-2.004121	1.826398
H	3.130960	-0.576473	2.376184
H	1.542244	-3.048819	1.522145
H	0.907142	-4.074239	0.225340
H	4.816514	-0.430528	1.892025
H	0.996409	0.631394	-1.188036
H	-0.384807	1.022783	1.508135
H	0.815992	-0.969100	1.856199
H	-1.411994	2.319864	-1.807795
H	-0.829087	4.084724	-1.602248
H	3.400684	0.986442	-1.205773
H	2.031624	1.238089	1.517046
H	4.255109	1.379604	0.290444
H	0.490604	-2.416363	-2.016270
H	-0.713080	-1.157012	-1.617882
H	-1.022478	-2.857826	-1.248801
H	3.380742	-3.700599	-1.998910
H	-4.029944	1.330681	-0.505146
H	-3.538964	-0.175277	-1.266565
H	-5.163109	0.005177	1.317401
H	-6.461007	0.918074	-0.617278
H	-6.145785	-0.565668	-1.534094
H	-7.141970	-0.622989	-0.073793
H	-5.627022	-2.414976	0.936204

ωB97XD energy = -1154.81544160 a.u.

(2*R*,6*R*,7*S*,8*S*)-**1**, Conf. M

C	2.988898	-2.820714	-0.356643
C	3.231264	-1.412210	-0.810917
C	3.389458	-0.359695	-0.000470
C	3.649310	-0.475498	1.483834
C	0.668736	-2.038253	0.333025
C	1.460938	-3.111612	-0.379498
C	1.945366	1.708153	0.152634
C	0.545921	1.523661	-0.507944
C	-0.255621	0.345867	0.082451
C	0.306662	-0.963347	-0.376817
O	2.190830	3.128493	0.163963
C	1.052029	3.838128	0.015368
C	-0.055944	2.900231	-0.333073

O	1.012371	5.034810	0.136258
C	-1.294790	3.362746	-0.491747
C	3.173488	1.049248	-0.499322
C	0.462362	-2.226263	1.809148
O	3.571902	-3.792404	-1.214614
O	-1.604102	0.461887	-0.430086
C	-2.588295	-0.053554	0.330650
O	-2.401555	-0.547691	1.419422
C	-3.933211	0.113135	-0.335493
C	-4.988953	-0.887148	0.146038
C	-6.363911	-0.501432	-0.404081
C	-4.617728	-2.317764	-0.252910
H	3.353748	-2.966252	0.668957
H	3.033259	-1.239944	-1.869424
H	4.238895	0.377148	1.835979
H	4.193820	-1.386486	1.740704
H	1.300155	-4.099186	0.065079
H	1.169408	-3.172405	-1.433082
H	2.715631	-0.481147	2.058134
H	0.682497	1.338994	-1.582403
H	-0.298187	0.430870	1.171178
H	0.495964	-0.986377	-1.449893
H	-2.118080	2.725094	-0.784839
H	-1.477286	4.421112	-0.329230
H	3.073695	1.076682	-1.589302
H	1.880312	1.405394	1.204617
H	4.029064	1.679587	-0.229877
H	1.415357	-2.429099	2.312213
H	-0.175320	-3.101351	1.977105
H	-0.024875	-1.375079	2.286115
H	4.517619	-3.626338	-1.264154
H	-4.259068	1.139885	-0.119399
H	-3.799262	0.052016	-1.421289
H	-5.020478	-0.832989	1.240293
H	-7.131435	-1.196875	-0.050826
H	-6.653919	0.507488	-0.091866
H	-6.369209	-0.529816	-1.500544
H	-3.655659	-2.614168	0.174442

ωB97XD energy = -1154.81533612 a.u.

(2*R*,6*R*,7*S*,8*S*)-**1**, Conf. N

C	2.180314	-3.220649	-0.317915
C	2.722146	-1.852585	-0.629842
C	3.264490	-0.990559	0.233102
C	3.632314	-1.341532	1.651183
C	0.059216	-1.888953	0.143537
C	0.850518	-3.121571	0.509607
C	2.265416	1.312588	0.424759
C	0.893244	1.204308	-0.308794
C	-0.193865	0.612313	0.619240
C	0.135706	-0.822183	0.950208
O	2.664726	2.694521	0.365346
C	1.770319	3.473385	-0.280358
C	0.640545	2.621268	-0.750064
O	1.927065	4.658059	-0.423662
C	-0.356728	3.136245	-1.466152
C	3.415316	0.464854	-0.143367
C	-0.600115	-1.928556	-1.209181
O	1.981125	-3.861360	-1.572305
O	-1.455094	0.746897	-0.051944
C	-2.552743	0.773231	0.730435
O	-2.509495	0.756044	1.938692
C	-3.822335	0.805190	-0.082863
C	-4.764115	-0.348780	0.302959
C	-6.095177	-0.213493	-0.437376
C	-4.113038	-1.706970	0.028707
H	2.897669	-3.803000	0.277300
H	2.489987	-1.512338	-1.637964
H	3.527881	-2.408002	1.861197
H	3.010855	-0.800384	2.375317
H	1.113610	-3.090141	1.572013
H	0.277633	-4.043156	0.342846
H	4.671044	-1.057761	1.853525
H	0.987444	0.540589	-1.175036
H	-0.243166	1.200866	1.540483
H	0.645936	-0.945123	1.903259
H	-1.173782	2.525219	-1.831080
H	-0.353599	4.199302	-1.689011
H	3.445510	0.591524	-1.231030
H	2.141783	1.090723	1.490149
H	4.345140	0.878929	0.262334
H	0.130947	-2.203589	-1.977065
H	-1.079914	-0.988828	-1.477538
H	-1.364971	-2.714432	-1.218858
H	1.681919	-4.762086	-1.421421
H	-4.313116	1.764226	0.117636
H	-3.581833	0.763837	-1.150071
H	-4.950125	-0.265696	1.380052
H	-6.573069	0.749772	-0.231324
H	-5.949083	-0.293373	-1.521393
H	-6.789255	-1.004307	-0.136244
H	-4.780828	-2.521913	0.324819

ωB97XD energy = -1154.81529758 a.u.

(2*R*,6*R*,7*S*,8*S*)-**1**, Conf. O

C	3.627966	-2.205509	-0.234672
C	3.463096	-0.749574	-0.594672
C	3.575515	0.299285	0.225702
C	4.095477	0.204456	1.636316
C	1.127606	-1.997756	0.154338
C	2.386874	-2.730958	0.551643
C	1.612465	1.867298	0.403771
C	0.440381	1.147434	-0.330669
C	-0.258406	0.110254	0.580542
C	0.697550	-0.999501	0.936033
O	1.333079	3.278872	0.355655
C	0.176999	3.565488	-0.281402
C	-0.436190	2.294009	-0.760615
O	-0.228459	4.691004	-0.412530
C	-1.562504	2.300934	-1.470859
C	3.017995	1.644615	-0.179838
C	0.565253	-2.368796	-1.192386

O	3.756554	-3.007302	-1.405177
O	-1.415813	-0.350442	-0.131374
C	-2.437677	-0.827831	0.606859
O	-2.431325	-0.852985	1.815206
C	-3.555846	-1.333012	-0.272593
C	-4.947740	-1.086948	0.320970
C	-5.244758	0.411660	0.418538
C	-6.011367	-1.805154	-0.511777
H	4.509787	-2.358866	0.402344
H	3.039181	-0.588700	-1.586572
H	4.566178	-0.757813	1.847283
H	3.289575	0.341032	2.368502
H	2.576321	-2.584458	1.619389
H	2.319901	-3.807870	0.364351
H	4.831889	0.992594	1.826987
H	0.826502	0.610508	-1.204008
H	-0.605492	0.604347	1.493573
H	1.197640	-0.854063	1.891602
H	-2.009770	1.387520	-1.844337
H	-2.048691	3.249167	-1.680920
H	2.968283	1.742122	-1.269851
H	1.613929	1.606888	1.467535
H	3.651583	2.455729	0.195180
H	1.370705	-2.447440	-1.929561
H	-0.192652	-1.671298	-1.546607
H	0.107676	-3.363825	-1.131537
H	4.522860	-2.705147	-1.901779
H	-3.467434	-0.879545	-1.265826
H	-3.384384	-2.409909	-0.399920
H	-4.950833	-1.506398	1.333452
H	-4.511121	0.928228	1.044788
H	-5.234399	0.875354	-0.575762
H	-6.232890	0.583571	0.856259
H	-7.005293	-1.661701	-0.076831

ωB97XD energy = -1154.81526419 a.u.

(2*R*,6*R*,7*S*,8*S*)-**1**, Conf. P

C	3.962415	-1.682228	-0.410851
C	3.591453	-0.313736	-0.897428
C	3.331223	0.733434	-0.107095
C	3.681388	0.780945	1.362676
C	1.551468	-1.885366	0.381428
C	2.681966	-2.563566	-0.360656
C	1.170924	2.030608	0.111447
C	-0.072285	1.286527	-0.466138
C	-0.291701	-0.105657	0.158836
C	0.753153	-1.068415	-0.315694
O	0.827600	3.431090	0.104877
C	-0.506070	3.622594	0.026544
C	-1.162730	2.310003	-0.242512
O	-1.014923	4.706807	0.144805
C	-2.490107	2.228180	-0.298468
C	2.526189	1.908030	-0.607888
C	1.498300	-2.110292	1.865629
O	4.858055	-2.363972	-1.278595
O	-1.579840	-0.583371	-0.305605
C	-2.159729	-1.547755	0.439390
O	-1.731866	-1.902701	1.514333
C	-3.382674	-2.122463	-0.231984
C	-4.494803	-1.099136	-0.519337
C	-5.673504	-1.794252	-1.204416
C	-4.943207	-0.388980	0.760189
H	4.390929	-1.632087	0.599149
H	3.294897	-0.272168	-1.946078
H	4.582331	0.208286	1.593054
H	2.872539	0.382188	1.985805
H	2.949375	-3.521469	0.097123
H	2.398594	-2.761567	-1.399505
H	3.853608	1.814399	1.680313
H	0.065319	1.155777	-1.548440
H	-0.332358	-0.023726	1.247962
H	0.895686	-1.031698	-1.395625
H	-3.001747	1.303220	-0.522420
H	-3.073981	3.125937	-0.117047
H	2.368295	1.850142	-1.689779
H	1.287204	1.754063	1.166261
H	3.057154	2.844680	-0.401787
H	1.253283	-3.159771	2.063141
H	0.742783	-1.503767	2.365695
H	2.477384	-1.922719	2.322421
H	5.649959	-1.828229	-1.380163
H	-3.059533	-2.585646	-1.171519
H	-3.758577	-2.911507	0.425419
H	-4.093875	-0.354757	-1.218752
H	-6.460040	-1.073685	-1.448569
H	-5.364229	-2.284630	-2.133061
H	-6.108881	-2.556385	-0.547609
H	-5.722166	0.348397	0.542964

ωB97XD energy = -1154.81517990 a.u.

(2*R*,6*R*,7*S*,8*S*)-**1**, Conf. Q

C	1.917481	-3.360034	-0.286661
C	2.496725	-2.086446	-0.826633
C	2.984391	-1.090270	-0.078402
C	3.304868	-1.227458	1.392175
C	-0.040193	-1.956013	0.530588
C	0.371156	-3.219150	-0.192218
C	2.186781	1.305687	0.074265
C	0.733176	1.494726	-0.453264
C	-0.299182	0.587296	0.242465
C	-0.140328	-0.836086	-0.194796
O	2.810334	2.601329	-0.032579
C	1.896691	3.593025	-0.108679
C	0.543750	2.985201	-0.276797
O	2.197095	4.757325	-0.061574
C	-0.539013	3.760385	-0.293801
C	3.121395	0.308068	-0.631296
C	-0.187294	-2.053755	2.022253
O	2.145482	-4.480898	-1.129764
O	-1.603515	1.039824	-0.189660
C	-2.641683	0.750970	0.621174
O	-2.509196	0.274080	1.726326

C	-3.961271	1.059753	-0.036792
C	-4.601955	-0.196432	-0.678719
C	-3.694337	-0.824101	-1.740348
C	-5.021825	-1.228524	0.370293
H	2.307524	-3.566783	0.718917
H	2.278942	-1.901465	-1.879106
H	4.112326	-0.543511	1.672867
H	3.618674	-2.239614	1.655816
H	-0.019065	-4.115563	0.300590
H	-0.007977	-3.216455	-1.219531
H	2.440056	-0.979980	2.018888
H	0.715443	1.269167	-1.528899
H	-0.235739	0.709090	1.326839
H	-0.038557	-0.932644	-1.275603
H	-1.533717	3.362529	-0.442276
H	-0.411819	4.830523	-0.158391
H	2.938974	0.330320	-1.710707
H	2.143930	1.077688	1.146260
H	4.137738	0.682167	-0.460851
H	0.692273	-2.533062	2.468399
H	-1.048397	-2.687982	2.261441
H	-0.355057	-1.088849	2.501834
H	3.093720	-4.580282	-1.253634
H	-4.630240	1.455064	0.732441
H	-3.811538	1.823353	-0.805393
H	-5.508838	0.167129	-1.178310
H	-4.223091	-1.623880	-2.268281
H	-3.366454	-0.085581	-2.478773
H	-2.798976	-1.262235	-1.284989
H	-5.533837	-2.069157	-0.108823

ωB97XD energy = -1154.81516715 a.u.

(2*R*,6*R*,7*S*,8*S*)-**1**, Conf. R

C	2.227574	-3.139911	-0.320868
C	2.613970	-1.813612	-0.903579
C	3.038296	-0.761105	-0.195175
C	3.481253	-0.848056	1.247340
C	0.181512	-1.965766	0.632455
C	0.686266	-3.179902	-0.114712
C	1.982709	1.527408	0.016887
C	0.483078	1.545491	-0.402138
C	-0.385758	0.529380	0.361968
C	-0.089608	-0.869123	-0.084709
O	2.443484	2.885474	-0.132535
C	1.418807	3.765386	-0.137377
C	0.136134	3.004959	-0.207418
O	1.586074	4.956736	-0.108358
C	-1.027379	3.649497	-0.143873
C	2.970954	0.638834	-0.757447
C	0.139240	-2.067912	2.130261
O	2.524134	-4.235298	-1.176217
O	-1.758637	0.823898	0.013423
C	-2.706880	0.373077	0.859754
O	-2.457738	-0.099359	1.945872
C	-4.086324	0.500181	0.266941
C	-4.424051	-0.646631	-0.719444
C	-3.667187	-0.533536	-2.045341
C	-4.215553	-2.023080	-0.081857
H	2.709592	-3.290332	0.654593
H	2.298215	-1.664167	-1.936632
H	3.940155	-1.810995	1.481197
H	2.641762	-0.710272	1.938843
H	0.441811	-4.109991	0.408635
H	0.236194	-3.235028	-1.111389
H	4.215143	-0.066933	1.470521
H	0.412487	1.314399	-1.474415
H	-0.266474	0.666857	1.439967
H	-0.041449	-0.960577	-1.169848
H	-1.978122	3.139835	-0.222595
H	-1.015280	4.728000	-0.014850
H	2.708979	0.630054	-1.820502
H	2.046523	1.302315	1.088473
H	3.947335	1.128386	-0.662551
H	-0.628362	-2.793038	2.422664
H	-0.107631	-1.124314	2.617635
H	1.093333	-2.441453	2.521527
H	3.465692	-4.223362	-1.370465
H	-4.791331	0.481861	1.101184
H	-4.175897	1.461518	-0.249295
H	-5.493673	-0.529375	-0.934833
H	-4.025368	-1.290583	-2.750252
H	-3.806019	0.450453	-2.504913
H	-2.593584	-0.687352	-1.902053
H	-4.584469	-2.809995	-0.746934

ωB97XD energy = -1154.81504613 a.u.

(2*R*,6*R*,7*S*,8*S*)-**1**, Conf. S

C	2.460063	-3.057276	-0.196744
C	2.885826	-1.640134	-0.493024
C	3.287340	-0.723114	0.392089
C	3.585691	-1.032277	1.836004
C	0.197660	-1.926873	0.050403
C	1.052736	-3.096106	0.477457
C	2.045638	1.464533	0.498616
C	0.745881	1.222148	-0.328676
C	-0.342528	0.526678	0.525224
C	0.088800	-0.876337	0.874771
O	2.307986	2.879657	0.462337
C	1.389343	3.567220	-0.250955
C	0.389001	2.606912	-0.798104
O	1.438699	4.761583	-0.389482
C	-0.595891	3.017762	-1.594282
C	3.310697	0.739002	0.011747
C	-0.321850	-1.991459	-1.360054
O	2.373656	-3.822445	-1.394959
O	-1.572631	0.562307	-0.213948
C	-2.708940	0.690426	0.505383
O	-2.723234	0.787832	1.711048
C	-3.931134	0.675965	-0.377727
C	-4.716491	-0.654226	-0.287509
C	-3.836520	-1.848813	-0.663690
C	-5.372804	-0.854080	1.080265

H	3.165015	-3.545684	0.490264
H	2.666565	-1.315904	-1.510718
H	4.513222	-0.540917	2.148483
H	3.690241	-2.103175	2.022508
H	1.196381	-3.068605	1.561920
H	0.601612	-4.060204	0.220256
H	2.792731	-0.661384	2.497355
H	0.967025	0.574926	-1.184045
H	-0.497374	1.099468	1.444024
H	0.510047	-0.972059	1.873608
H	-1.315341	2.325367	-2.015479
H	-0.685225	4.075116	-1.825729
H	3.396247	0.864878	-1.073131
H	1.873811	1.227132	1.553845
H	4.166077	1.246584	0.470918
H	0.491103	-2.224674	-2.055676
H	-0.826684	-1.077488	-1.670174
H	-1.037385	-2.817627	-1.444602
H	3.236428	-3.825228	-1.820212
H	-4.574619	1.500137	-0.055587
H	-3.627798	0.852749	-1.413161
H	-5.514015	-0.571361	-1.036981
H	-4.426638	-2.770143	-0.688059
H	-3.376301	-1.712748	-1.647574
H	-3.029994	-1.989539	0.066102
H	-5.974277	-1.768945	1.080533

ωB97XD energy = -1154.81492423 a.u.

(2*R*,6*R*,7*S*,8*S*)-**1**, Conf. T

C	2.698718	-2.850538	0.478595
C	3.111831	-1.406726	0.289686
C	2.973201	-0.637706	-0.797299
C	2.619127	-1.142496	-2.168460
C	0.417558	-1.814002	0.929204
C	1.150698	-3.023442	0.403784
C	1.922981	1.422329	0.219754
C	0.504425	1.455163	-0.426431
C	-0.462338	0.491951	0.296520
C	-0.044305	-0.923007	0.045372
O	2.251450	2.778493	0.573468
C	1.255834	3.649638	0.303825
C	0.129344	2.911275	-0.335789
O	1.335662	4.823817	0.555837
C	-0.963554	3.548591	-0.750033
C	3.073295	0.869592	-0.635016
C	0.416721	-1.613376	2.419679
O	3.226764	-3.759265	-0.481232
O	-1.772225	0.711477	-0.260452
C	-2.815662	0.372911	0.525218
O	-2.690024	0.009619	1.673511
C	-4.125074	0.469796	-0.213006
C	-4.578667	-0.894749	-0.788318
C	-3.567605	-1.460089	-1.789909
C	-4.890303	-1.910928	0.312963
H	3.029446	-3.156760	1.481191
H	3.380771	-0.903892	1.219630
H	1.707146	-0.657968	-2.537970
H	2.485748	-2.224363	-2.191703
H	0.879037	-3.936011	0.944956
H	0.906655	-3.179047	-0.651163
H	3.415146	-0.879380	-2.875625
H	0.562254	1.151269	-1.478188
H	-0.496377	0.736231	1.360903
H	0.007470	-1.174992	-1.013276
H	-1.779808	3.028095	-1.236355
H	-1.041457	4.620720	-0.594937
H	3.074183	1.375369	-1.607137
H	1.887723	0.864433	1.161341
H	4.004029	1.144482	-0.128750
H	0.185811	-2.557182	2.923921
H	-0.319521	-0.873700	2.739405
H	1.408078	-1.299449	2.773587
H	4.184024	-3.667298	-0.489561
H	-4.873874	0.830366	0.497357
H	-4.028915	1.195141	-1.025814
H	-5.510542	-0.684121	-1.328359
H	-3.972787	-2.352891	-2.276044
H	-3.318330	-0.730235	-2.566773
H	-2.636090	-1.748116	-1.289664
H	-5.295413	-2.829343	-0.123773

ωB97XD energy = -1154.81483134 a.u.

(2*R*,6*R*,7*S*,8*S*)-**1**, Conf. U

C	3.993354	-1.636834	-0.295863
C	3.563509	-0.257022	-0.703583
C	3.453416	0.819967	0.083007
C	4.038193	0.959760	1.465554
C	1.553803	-1.897444	0.317711
C	2.752884	-2.570443	-0.310017
C	1.155831	1.772010	0.272677
C	0.070052	1.129527	-0.647303
C	-0.359400	-0.264539	-0.124204
C	0.686616	-1.272587	-0.485013
O	0.645478	3.054934	0.684521
C	-0.591885	3.309721	0.207166
C	-1.014028	2.174611	-0.659712
O	-1.192594	4.319104	0.469807
C	-2.158407	2.215326	-1.337962
C	2.563596	1.971978	-0.330503
C	1.526693	-1.834389	1.819035
O	4.927039	-2.220792	-1.195684
O	-1.595848	-0.613601	-0.773443
C	-2.403443	-1.460286	-0.100774
O	-2.138313	-1.890265	0.999643
C	-3.657633	-1.775661	-0.878887
C	-4.944086	-1.232778	-0.214390
C	-5.273090	-1.940174	1.102307
C	-4.884427	0.286154	-0.035484
H	4.415498	-1.627378	0.716914
H	3.115562	-0.211152	-1.696957
H	4.733658	1.807007	1.479083
H	4.582266	0.075737	1.801268

H	3.013584	-3.505414	0.196713
H	2.550637	-2.805741	-1.359890
H	3.264274	1.184234	2.209077
H	0.469133	0.999215	-1.659475
H	-0.545168	-0.214023	0.950902
H	0.811423	-1.385017	-1.562388
H	-2.460187	1.409392	-1.995975
H	-2.810641	3.076785	-1.227351
H	2.499258	2.064159	-1.419431
H	1.245740	1.183969	1.192745
H	2.949012	2.919140	0.060878
H	1.707804	-2.829112	2.239195
H	0.571764	-1.477581	2.209044
H	2.324440	-1.180278	2.192929
H	5.695718	-1.645802	-1.251497
H	-3.559677	-1.365677	-1.887549
H	-3.722635	-2.866015	-0.956923
H	-5.750106	-1.455844	-0.925336
H	-6.228581	-1.578496	1.495981
H	-5.352729	-3.022805	0.960635
H	-4.500178	-1.759931	1.854219
H	-5.826284	0.664198	0.373560

ωB97XD energy = -1154.81464588 a.u.

(2*R*,6*R*,7*S*,8*S*)-**1**, Conf. V

C	-2.139297	-3.212359	-0.591321
C	-2.799368	-1.858220	-0.466364
C	-2.960155	-1.032760	0.576091
C	-2.731363	-1.350571	2.031948
C	0.001484	-1.834710	-0.623383
C	-0.712204	-3.038206	-1.195636
C	-2.235183	1.144099	-0.492434
C	-0.828334	1.173164	0.169054
C	0.268659	0.704128	-0.814277
C	-0.015905	-0.690227	-1.318681
O	-2.631853	2.516115	-0.676972
C	-1.813852	3.380660	-0.034727
C	-0.691357	2.612156	0.579259
O	-2.016163	4.566519	-0.005884
C	0.224776	3.198948	1.345857
C	-3.328795	0.420225	0.305062
C	0.553245	-2.004842	0.764855
O	-2.010896	-3.952242	0.614637
O	1.515747	0.804509	-0.108926
C	2.627349	0.930688	-0.865613
O	2.601698	1.032547	-2.069512
C	3.870529	0.898335	-0.014158
C	4.063412	-0.452571	0.702441
C	4.042762	-1.618308	-0.288745
C	5.363103	-0.431984	1.509026
H	-2.713573	-3.812731	-1.312248
H	-3.026493	-1.444409	-1.450984
H	-3.665458	-1.202346	2.587800
H	-2.003893	-0.653276	2.465378
H	-0.818658	-2.922329	-2.278611
H	-0.170931	-3.971907	-1.009751
H	-2.373682	-2.363714	2.201386
H	-0.798392	0.508126	1.035329
H	0.308890	1.391295	-1.665082
H	-0.428018	-0.717613	-2.325376
H	1.035155	2.634930	1.794444
H	0.166105	4.268418	1.525495
H	-3.478744	0.954534	1.250185
H	-2.182007	0.714618	-1.495070
H	-4.264721	0.503162	-0.256632
H	0.949621	-1.078855	1.180337
H	1.366507	-2.740421	0.743394
H	-0.212490	-2.413365	1.429977
H	-2.891066	-4.195142	0.916533
H	4.717537	1.102451	-0.675014
H	3.810674	1.702144	0.728322
H	3.228852	-0.580706	1.402554
H	4.194532	-2.569450	0.231580
H	3.090751	-1.679016	-0.826644
H	4.839119	-1.509478	-1.034072
H	5.498180	-1.373486	2.050503

ωB97XD energy = -1154.81432576 a.u.

(2*R*,6*R*,7*S*,8*S*)-**1**, Conf. W

C	-4.012694	-1.619695	-0.421228
C	-3.680780	-0.143474	-0.466624
C	-3.307303	0.657169	0.539246
C	-3.409056	0.307970	1.998075
C	-1.480023	-1.871410	-0.538941
C	-2.768580	-2.479230	-0.041186
C	-1.259347	1.731623	-0.461265
C	-0.108243	1.217338	0.455593
C	0.353207	-0.192736	0.020409
C	-0.729923	-1.179798	0.325693
O	-0.813340	2.974167	-1.034615
C	0.432059	3.318292	-0.642331
C	0.932717	2.295635	0.319746
O	0.984796	4.312501	-1.035736
C	2.101059	2.441772	0.940016
C	-2.623849	1.971528	0.201044
C	-1.218732	-1.950344	-2.018055
O	-5.023397	-1.982627	0.512944
O	1.537679	-0.506401	0.778565
C	2.369362	-1.414892	0.227939
O	2.172072	-1.916476	-0.856606
C	3.556000	-1.706599	1.113954
C	4.901957	-1.235115	0.516569
C	5.296447	-2.011813	-0.741910
C	4.907442	0.274868	0.266918
H	-4.336478	-1.912625	-1.430014
H	-3.556564	0.245859	-1.478235
H	-3.845010	-0.678407	2.158198
H	-4.034766	1.047478	2.512466
H	-2.918658	-3.492441	-0.429245
H	-2.754396	-2.542022	1.050856
H	-2.424040	0.349758	2.478624
H	-0.448558	1.155675	1.495358

H	0.618970	-0.179443	-1.039396
H	-1.008125	-1.201211	1.378942
H	2.453922	1.716961	1.663755
H	2.719663	3.305643	0.714653
H	-2.488573	2.591637	1.094382
H	-1.397880	1.044077	-1.301577
H	-3.224358	2.552362	-0.506194
H	-0.183243	-1.713589	-2.268870
H	-1.880888	-1.272999	-2.573807
H	-1.426847	-2.962500	-2.379802
H	-5.806458	-1.454938	0.330487
H	3.396777	-1.235351	2.087435
H	3.586030	-2.791557	1.259149
H	5.650323	-1.449937	1.290320
H	6.298486	-1.715514	-1.068601
H	5.305115	-3.090540	-0.555697
H	4.596859	-1.823485	-1.560523
H	5.883526	0.601201	-0.104772

ωB97XD energy = -1154.81425653 a.u.

H	-0.620633	-0.874185	-2.188396
H	1.563370	2.437906	1.649150
H	0.992072	4.184696	1.310115
H	-3.161374	1.611703	1.343860
H	-2.076184	0.941289	-1.423285
H	-4.097448	1.214673	-0.091541
H	0.853912	-2.932932	1.069194
H	-0.506884	-2.044252	1.737619
H	0.957273	-1.174730	1.233162
H	-4.364071	-3.629283	-0.322820
H	4.824111	0.230670	-0.991190
H	4.146797	1.256970	0.284612
H	3.250831	-0.709900	1.460887
H	3.804641	-3.025580	0.716804
H	2.814262	-2.201337	-0.496818
H	4.555784	-2.380371	-0.752979
H	5.407815	-1.696852	2.202758
H	5.568398	0.053843	1.989032
H	6.213983	-1.052465	0.764822

ωB97XD energy = -1154.81819502 a.u.

(2*S*,6*R*,7*S*,8*S*)-**1**, Conf. A

C	-2.599239	-2.833224	-0.069081
C	-3.048060	-1.401819	-0.166327
C	-3.065471	-0.478431	0.803399
C	-2.898580	-0.755831	2.274562
C	-0.295347	-1.881912	-0.398037
C	-1.237705	-2.992974	-0.797746
C	-1.998146	1.440192	-0.455206
C	-0.579676	1.251213	0.153366
C	0.370152	0.545354	-0.843137
C	-0.159533	-0.822042	-1.204882
O	-2.143261	2.846320	-0.727854
C	-1.163825	3.585445	-0.157573
C	-0.176063	2.662328	0.474513
O	-1.151080	4.787796	-0.196026
C	0.852528	3.114728	1.187741
C	-3.164828	0.994184	0.438037
C	0.288144	-1.997866	0.984689
O	-3.491129	-3.733779	-0.712379
O	1.662274	0.507007	-0.218860
C	2.727617	0.405704	-1.043679
O	2.633033	0.388011	-2.247900
C	4.013670	0.315741	-0.261874
C	4.047486	-0.866544	0.723006
C	3.787299	-2.193378	0.005857
C	5.385596	-0.889621	1.464156
H	-2.473130	-3.128400	0.980476
H	-3.210553	-1.073832	-1.194502
H	-2.845057	-1.819399	2.513656
H	-1.997829	-0.270135	2.669836
H	-1.441937	-2.958949	-1.872489
H	-0.834979	-3.984914	-0.566072
H	-3.745887	-0.329436	2.823916
H	-0.627245	0.636461	1.055902
H	0.455168	1.148417	-1.752266

(2*S*,6*R*,7*S*,8*S*)-**1**, Conf. B

C	-2.433624	-2.989971	-0.113513
C	-2.977049	-1.596954	-0.266502
C	-3.135444	-0.668425	0.685381
C	-3.062130	-0.920240	2.168807
C	-0.188471	-1.873850	-0.282547
C	-1.013358	-3.055140	-0.736904
C	-2.118305	1.306551	-0.524379
C	-0.739573	1.240565	0.191975
C	0.334656	0.590972	-0.712548
C	-0.064222	-0.815521	-1.093303
O	-2.347577	2.690696	-0.845839
C	-1.469399	3.515470	-0.230200
C	-0.468575	2.685876	0.502944
O	-1.541366	4.713965	-0.306168
C	0.462886	3.231301	1.281449
C	-3.311284	0.789983	0.292331
C	0.287204	-1.931930	1.144556
O	-3.206015	-3.960718	-0.807669
O	1.571654	0.650149	0.016270
C	2.703026	0.606652	-0.718095
O	2.709186	0.568008	-1.926355
C	3.936803	0.576656	0.147097
C	4.697707	-0.753334	-0.015521
C	5.993617	-0.718096	0.795430
C	3.823873	-1.944157	0.386616
H	-2.364877	-3.264685	0.946762
H	-3.084144	-1.292260	-1.309006
H	-2.221970	-0.378876	2.621346
H	-2.964613	-1.976391	2.426289
H	-1.139036	-3.045383	-1.824190
H	-0.557887	-4.012571	-0.461905
H	-3.970656	-0.539010	2.649013
H	-0.808411	0.645139	1.106151
H	0.453081	1.188168	-1.621759

H	-0.440186	-0.911279	-2.109379
H	1.181021	2.621720	1.818777
H	0.513219	4.311384	1.382999
H	-3.413786	1.415248	1.186804
H	-2.085150	0.782238	-1.481488
H	-4.217524	0.939472	-0.303650
H	0.882027	-1.064680	1.429294
H	0.901765	-2.827598	1.291426
H	-0.562183	-2.018729	1.831856
H	-4.111102	-3.917774	-0.485648
H	4.578255	1.406017	-0.168179
H	3.662489	0.730298	1.195564
H	4.949850	-0.854296	-1.077786
H	6.554421	-1.649324	0.668870
H	6.639156	0.109480	0.483847
H	5.782598	-0.597668	1.864900
H	4.372875	-2.883946	0.272756
H	2.920572	-2.010279	-0.229235
H	3.513110	-1.859671	1.435228

ωB97XD energy = -1154.81769299 a.u.

(2*S*,6*R*,7*S*,8*S*)-**1**, Conf. C

C	-4.140049	-1.601535	0.141543
C	-3.782781	-0.143358	-0.002740
C	-3.297180	0.680602	0.930789
C	-3.238915	0.356600	2.397891
C	-1.650559	-1.888144	-0.381860
C	-2.868885	-2.499921	0.268343
C	-1.361370	1.706020	-0.320009
C	-0.129172	1.170179	0.467312
C	0.263465	-0.246029	-0.012796
C	-0.790073	-1.224542	0.398909
O	-0.955403	2.939221	-0.942286
C	0.330050	3.262018	-0.682655
C	0.912492	2.228200	0.220154
O	0.854773	4.247845	-1.131369
C	2.148418	2.345874	0.700694
C	-2.637447	1.976073	0.489685
C	-1.588643	-1.946357	-1.881954
O	-4.850530	-2.057411	-1.005301
O	1.510599	-0.575887	0.628408
C	2.371252	-1.336096	-0.076831
O	2.152566	-1.724409	-1.201947
C	3.631702	-1.629603	0.698309
C	4.897006	-1.273398	-0.099423
C	4.969990	0.230590	-0.374913
C	6.141194	-1.758197	0.646342
H	-4.752351	-1.773419	1.037797
H	-3.759680	0.189647	-1.041836
H	-3.734667	1.142800	2.978838
H	-2.200820	0.313765	2.749916
H	-3.125417	-3.470376	-0.168375
H	-2.669521	-2.652038	1.333627
H	-3.714000	-0.596527	2.640148
H	-0.356258	1.112181	1.537851
H	0.427145	-0.235391	-1.092362

H	-0.937717	-1.268153	1.478282
H	2.568104	1.612561	1.379243
H	2.755332	3.196779	0.405066
H	-2.390677	2.609590	1.349494
H	-1.606313	1.021178	-1.137924
H	-3.308211	2.554457	-0.153603
H	-0.650931	-1.559498	-2.284275
H	-2.428511	-1.399306	-2.326207
H	-1.694480	-2.984850	-2.212093
H	-5.642265	-1.521457	-1.111001
H	3.612514	-1.094964	1.653284
H	3.624213	-2.703952	0.916759
H	4.834902	-1.798462	-1.059575
H	5.863209	0.476222	-0.957390
H	4.099818	0.584496	-0.937396
H	5.019461	0.793815	0.565375
H	7.048651	-1.530621	0.078406
H	6.111007	-2.839841	0.813888
H	6.228462	-1.267348	1.623299

ωB97XD energy = -1154.81751408 a.u.

(2*S*,6*R*,7*S*,8*S*)-**1**, Conf. D

C	-2.938403	-2.788794	0.127469
C	-3.262663	-1.316673	0.186810
C	-3.002674	-0.459357	1.178495
C	-2.543857	-0.864606	2.552041
C	-0.690870	-1.895273	-0.705563
C	-1.404479	-3.036895	-0.021417
C	-1.944953	1.447814	-0.098556
C	-0.478352	1.443881	0.427659
C	0.378010	0.386528	-0.303438
C	-0.084211	-0.987368	0.067458
O	-2.229872	2.790486	-0.531744
C	-1.174690	3.622359	-0.398315
C	-0.040372	2.868434	0.208779
O	-1.215434	4.779324	-0.727206
C	1.110941	3.471586	0.496981
C	-3.044704	1.030490	0.888332
C	-0.862683	-1.780233	-2.193566
O	-3.561939	-3.392922	-1.000961
O	1.733800	0.567179	0.148970
C	2.707345	0.171374	-0.695211
O	2.495760	-0.233947	-1.815525
C	4.070607	0.263476	-0.060070
C	4.687188	-1.135511	0.137010
C	6.094950	-1.004883	0.719880
C	3.798933	-2.014672	1.021972
H	-3.266510	-3.304814	1.040689
H	-3.574619	-0.907266	-0.775541
H	-1.557133	-0.440927	2.774884
H	-2.480192	-1.947469	2.678706
H	-1.289524	-3.981531	-0.562543
H	-0.985599	-3.170840	0.980920
H	-3.231756	-0.472499	3.310184
H	-0.460925	1.210616	1.498689
H	0.345616	0.566236	-1.380513

H	-0.016961	-1.184192	1.137614
H	1.935367	2.940630	0.957928
H	1.229195	4.525776	0.263931
H	-2.942762	1.616672	1.808794
H	-2.019538	0.818168	-0.991394
H	-4.000770	1.309004	0.433924
H	-0.639055	-2.741168	-2.667077
H	-0.208804	-1.025749	-2.634159
H	-1.905340	-1.552052	-2.445325
H	-4.512434	-3.260102	-0.937055
H	4.707089	0.854824	-0.726062
H	3.996960	0.779789	0.902195
H	4.758486	-1.601450	-0.853065
H	6.563147	-1.987883	0.831135
H	6.739083	-0.395000	0.078053
H	6.063647	-0.535652	1.710587
H	4.254790	-2.998897	1.167981
H	2.809232	-2.168407	0.578789
H	3.660414	-1.556851	2.008979

ωB97XD energy = -1154.81738519 a.u.

(2*S*,6*R*,7*S*,8*S*)-**1**, Conf. E

C	-3.900861	-1.735566	0.012603
C	-3.649108	-0.274420	-0.232479
C	-3.298897	0.658960	0.661546
C	-3.346191	0.493029	2.157920
C	-1.400130	-1.955412	-0.147370
C	-2.719654	-2.562645	-0.562586
C	-1.410392	1.756286	-0.614697
C	-0.270479	1.026782	0.150492
C	0.314733	-0.137502	-0.684399
C	-0.754720	-1.154063	-1.003652
O	-0.887389	3.040739	-1.002816
C	0.291351	3.321120	-0.400994
C	0.710406	2.136239	0.403286
O	0.856004	4.374753	-0.537613
C	1.784462	2.162570	1.188633
C	-2.705453	1.974025	0.180833
C	-1.004466	-2.188256	1.286270
O	-5.064652	-2.210599	-0.651781
O	1.406336	-0.674831	0.080219
C	2.402951	-1.259565	-0.616326
O	2.407946	-1.347829	-1.822005
C	3.504623	-1.758734	0.284169
C	4.855757	-1.102190	-0.052641
C	4.813661	0.408090	0.191581
C	5.974972	-1.760467	0.755419
H	-3.985100	-1.938932	1.087764
H	-3.592922	-0.022217	-1.292860
H	-3.808344	-0.442342	2.478248
H	-3.916629	1.318198	2.599667
H	-2.824627	-2.556474	-1.652024
H	-2.827535	-3.598586	-0.223209
H	-2.340797	0.544419	2.594267
H	-0.644627	0.605083	1.086766
H	0.726287	0.257461	-1.618285

H	-1.137161	-1.089317	-2.019821
H	2.077704	1.299947	1.776616
H	2.390682	3.062300	1.240105
H	-2.480029	2.624980	1.033562
H	-1.644524	1.240268	-1.548022
H	-3.404925	2.521529	-0.459192
H	-0.888113	-3.263671	1.464264
H	-1.790697	-1.841622	1.966835
H	-0.068415	-1.699043	1.553735
H	-5.817388	-1.688462	-0.359458
H	3.242443	-1.578774	1.331589
H	3.576610	-2.841225	0.131653
H	5.043265	-1.276259	-1.118669
H	5.769241	0.871188	-0.072090
H	4.033783	0.897361	-0.401176
H	4.619141	0.622610	1.249789
H	6.943869	-1.314279	0.510974
H	6.037882	-2.834724	0.553895
H	5.809577	-1.626977	1.831408

ωB97XD energy = -1154.81714732 a.u.

(2*S*,6*R*,7*S*,8*S*)-**1**, Conf. F

C	-3.786211	-1.719328	0.117603
C	-3.567197	-0.243791	-0.065335
C	-3.134859	0.640376	0.842689
C	-3.040330	0.390611	2.325146
C	-1.310437	-1.904696	-0.285986
C	-2.660465	-2.497124	-0.615485
C	-1.372157	1.818109	-0.536213
C	-0.164949	1.059735	0.085532
C	0.347782	-0.048409	-0.865085
C	-0.748056	-1.048737	-1.147765
O	-0.890508	3.124480	-0.903146
C	0.331965	3.386568	-0.386616
C	0.823727	2.166806	0.318606
O	0.876482	4.451850	-0.513739
C	1.951357	2.170892	1.025001
C	-2.591048	1.983903	0.382894
C	-0.786335	-2.218172	1.090596
O	-5.005689	-2.169384	-0.457654
O	1.498469	-0.633347	-0.235008
C	2.349524	-1.297958	-1.046865
O	2.208015	-1.357201	-2.245293
C	3.458713	-1.960563	-0.267857
C	4.171901	-1.050846	0.744745
C	5.247191	-1.844258	1.490195
C	4.770233	0.179029	0.058418
H	-3.762370	-1.985248	1.182305
H	-3.615884	0.069386	-1.109802
H	-1.999087	0.428962	2.668683
H	-3.466262	-0.565824	2.633354
H	-2.863929	-2.421895	-1.688265
H	-2.733110	-3.553821	-0.335958
H	-3.572178	1.183862	2.863296
H	-0.452852	0.580509	1.024463
H	0.676132	0.403083	-1.806451

H	-1.218067	-0.929103	-2.121105
H	2.297397	1.286132	1.547522
H	2.548175	3.076686	1.078641
H	-2.292742	2.590331	1.246071
H	-1.688097	1.347313	-1.469111
H	-3.346981	2.558373	-0.162391
H	-0.654471	-3.301458	1.195564
H	-1.508562	-1.916656	1.858064
H	0.169290	-1.739010	1.301597
H	-5.729830	-1.673157	-0.065139
H	3.016699	-2.819286	0.253379
H	4.169572	-2.350759	-1.001937
H	3.426074	-0.716095	1.476082
H	5.745948	-1.218414	2.236650
H	4.820400	-2.711065	2.005497
H	6.012356	-2.208886	0.794870
H	5.266246	0.828992	0.786326
H	4.004567	0.773194	-0.451148
H	5.515313	-0.120211	-0.687635

ωB97XD energy = -1154.81714552 a.u.

(2*S*,6*R*,7*S*,8*S*)-**1**, Conf. G

C	-3.142891	-2.526407	0.210782
C	-3.306770	-1.030878	0.319715
C	-2.894122	-0.229768	1.306650
C	-2.388142	-0.717060	2.635985
C	-0.888557	-1.847017	-0.788888
C	-1.658612	-2.924771	-0.063197
C	-1.752426	1.593979	-0.025139
C	-0.254751	1.431117	0.372530
C	0.428304	0.312949	-0.444451
C	-0.139969	-1.015347	-0.055605
O	-1.935599	2.970438	-0.405603
C	-0.795211	3.690691	-0.344387
C	0.305024	2.810522	0.143976
O	-0.747106	4.855744	-0.642249
C	1.531848	3.288338	0.341657
C	-2.800540	1.263747	1.047306
C	-1.153155	-1.698108	-2.259773
O	-3.910828	-3.036451	-0.874186
O	1.826904	0.347100	-0.103983
C	2.681576	-0.162854	-1.015773
O	2.340932	-0.517669	-2.120300
C	4.083143	-0.236925	-0.464944
C	4.179421	-1.050977	0.838835
C	3.598379	-2.456195	0.660424
C	5.633887	-1.108250	1.309221
H	-3.450417	-3.029101	1.138509
H	-3.637231	-0.568776	-0.612077
H	-1.330613	-0.458911	2.772421
H	-2.487603	-1.797992	2.757188
H	-1.682618	-3.865307	-0.622882
H	-1.179841	-3.119256	0.901669
H	-2.936120	-0.231320	3.451548
H	-0.172090	1.170075	1.434248
H	0.325219	0.519917	-1.512035

H	-0.015347	-1.235477	1.004935
H	2.333905	2.663148	0.715781
H	1.734293	4.332364	0.121301
H	-2.557468	1.810579	1.965570
H	-1.966268	1.000993	-0.920348
H	-3.757139	1.654309	0.685447
H	-2.194591	-1.405421	-2.438492
H	-1.019052	-2.664497	-2.756040
H	-0.485340	-0.979528	-2.737411
H	-4.834480	-2.807692	-0.733678
H	4.710115	-0.680717	-1.243377
H	4.438084	0.785616	-0.287954
H	3.588320	-0.528598	1.600636
H	3.692956	-3.032022	1.586484
H	2.536900	-2.426039	0.391627
H	4.128854	-2.998428	-0.131003
H	5.713417	-1.637094	2.264178
H	6.053122	-0.105520	1.443874
H	6.258002	-1.639322	0.580896

ωB97XD energy = -1154.81710269 a.u.

(2*S*,6*R*,7*S*,8*S*)-**1**, Conf. H

C	-4.101154	-1.487646	0.284486
C	-3.711258	-0.032276	0.210385
C	-3.098404	0.701027	1.144632
C	-2.898776	0.263373	2.569133
C	-1.684142	-1.804621	-0.494483
C	-2.850757	-2.421385	0.240397
C	-1.267939	1.766765	-0.230804
C	0.031374	1.152873	0.375248
C	0.317106	-0.242659	-0.224414
C	-0.733001	-1.206743	0.231640
O	-0.902612	3.034279	-0.807374
C	0.411944	3.311583	-0.679337
C	1.066884	2.203702	0.074083
O	0.909013	4.316768	-1.116636
C	2.351922	2.271242	0.413503
C	-2.439607	2.005932	0.732580
C	-1.769512	-1.773383	-1.993940
O	-4.919861	-1.844466	-0.824102
O	1.602229	-0.667494	0.273768
C	2.252234	-1.586020	-0.472847
O	1.851278	-1.969843	-1.547416
C	3.510019	-2.071948	0.204261
C	4.481230	-0.959967	0.632701
C	5.701393	-1.574082	1.321930
C	4.896548	-0.095634	-0.559814
H	-4.639813	-1.706810	1.217219
H	-3.785659	0.380005	-0.797326
H	-3.304134	1.014167	3.257162
H	-1.831527	0.167436	2.803250
H	-3.176233	-3.361396	-0.216614
H	-2.554246	-2.633459	1.272426
H	-3.376625	-0.693814	2.788998
H	-0.076636	1.037160	1.459836
H	0.378332	-0.168534	-1.312435

H	-0.775765	-1.308043	1.316327
H	2.833133	1.489273	0.987727
H	2.937304	3.132709	0.105423
H	-2.080244	2.563344	1.605142
H	-1.620775	1.149298	-1.062651
H	-3.152893	2.651327	0.210113
H	-2.611071	-1.151584	-2.321875
H	-1.968766	-2.780874	-2.372543
H	-0.850182	-1.415319	-2.460066
H	-5.703127	-1.286256	-0.822285
H	3.204702	-2.651296	1.084327
H	3.998626	-2.757046	-0.494339
H	3.962184	-0.327540	1.363172
H	6.390171	-0.794367	1.661735
H	5.411223	-2.171334	2.192716
H	6.250705	-2.226384	0.633039
H	5.585153	0.695117	-0.245737
H	4.034788	0.382518	-1.036919
H	5.404382	-0.703036	-1.317785

ωB97XD energy = -1154.81708961 a.u.

(2*S*,6*R*,7*S*,8*S*)-**1**, Conf. I

C	-3.461107	-2.454967	0.170815
C	-3.568072	-0.953752	0.070837
C	-3.305631	-0.046740	1.016765
C	-3.078199	-0.376764	2.465862
C	-1.026495	-1.962798	-0.451382
C	-1.973597	-2.926603	0.223750
C	-1.836547	1.551102	-0.271558
C	-0.459070	1.396857	0.439276
C	0.317399	0.174577	-0.101846
C	-0.372692	-1.084488	0.317586
O	-1.858954	2.865546	-0.858669
C	-0.719378	3.557563	-0.644956
C	0.200528	2.725602	0.183104
O	-0.544430	4.668256	-1.074110
C	1.370473	3.202310	0.603292
C	-3.089525	1.398740	0.602523
C	-1.015715	-1.966377	-1.954060
O	-4.047468	-3.074765	-0.969176
O	1.633881	0.227079	0.480497
C	2.647325	-0.252707	-0.268064
O	2.496075	-0.721488	-1.372613
C	3.970715	-0.074292	0.436324
C	5.076054	-1.006501	-0.068307
C	6.423298	-0.587077	0.524331
C	4.760143	-2.467723	0.260085
H	-3.954595	-2.829026	1.078775
H	-3.698870	-0.602341	-0.954036
H	-3.248165	-1.431973	2.690741
H	-2.053059	-0.130170	2.768545
H	-1.943116	-3.921458	-0.231946
H	-1.691348	-3.029976	1.276170
H	-3.744175	0.219852	3.099601
H	-0.602567	1.254439	1.516486
H	0.420045	0.254556	-1.186174

H	-0.452360	-1.191115	1.399529
H	2.033760	2.616684	1.228931
H	1.667415	4.206260	0.313927
H	-3.000634	2.054431	1.476311
H	-1.905654	0.845769	-1.105787
H	-3.936779	1.762920	0.012724
H	-0.225447	-1.339675	-2.370895
H	-1.984302	-1.640876	-2.351837
H	-0.864934	-2.987249	-2.318971
H	-4.969190	-2.805810	-1.026613
H	4.257182	0.974637	0.279938
H	3.815981	-0.194676	1.514610
H	5.124341	-0.901976	-1.158282
H	7.227760	-1.228511	0.151596
H	6.670927	0.448395	0.267457
H	6.412205	-0.669683	1.618101
H	5.551363	-3.128216	-0.108153
H	3.819919	-2.785185	-0.199269
H	4.682031	-2.612839	1.344839

ωB97XD energy = -1154.81676363 a.u.

(2*S*,6*R*,7*S*,8*S*)-**1**, Conf. J

C	-2.923357	-2.756837	-0.032364
C	-3.279644	-1.313097	-0.250036
C	-3.317443	-0.332995	0.661523
C	-3.276801	-0.530838	2.154234
C	-0.540151	-1.960366	-0.170451
C	-1.511103	-3.028617	-0.616865
C	-2.033500	1.449280	-0.592287
C	-0.694541	1.240868	0.170673
C	0.314118	0.404086	-0.652061
C	-0.268989	-0.944440	-0.998595
O	-2.079593	2.837136	-0.971883
C	-1.111904	3.568235	-0.373782
C	-0.242293	2.652130	0.421257
O	-1.023097	4.760823	-0.505811
C	0.736699	3.112579	1.196492
C	-3.302679	1.117426	0.205660
C	-0.080669	-2.063371	1.259593
O	-3.800508	-3.646616	-0.710253
O	1.504055	0.323787	0.148065
C	2.664578	0.105920	-0.504938
O	2.739109	-0.009940	-1.705033
C	3.824573	0.071887	0.461022
C	5.061237	-0.652118	-0.079043
C	6.248550	-0.430807	0.860681
C	4.786714	-2.145031	-0.277116
H	-2.913749	-2.995913	1.038801
H	-3.345091	-1.042160	-1.305330
H	-2.366877	-0.097871	2.587986
H	-3.331434	-1.578504	2.455301
H	-1.610419	-3.032790	-1.706905
H	-1.197876	-4.031765	-0.307353
H	-4.120698	-0.005390	2.615926
H	-0.868226	0.707795	1.109070
H	0.564318	0.936118	-1.575181

H	-0.650547	-1.010259	-2.015092
H	1.359771	2.445883	1.782383
H	0.921562	4.181672	1.246191
H	-3.352278	1.790536	1.069461
H	-2.041304	0.887761	-1.528609
H	-4.166355	1.348634	-0.426428
H	-0.938347	-2.086199	1.941929
H	0.579223	-1.248616	1.555196
H	0.456624	-3.008111	1.403831
H	-4.698476	-3.476514	-0.411203
H	4.064392	1.119643	0.687221
H	3.486240	-0.376716	1.402443
H	5.297981	-0.214282	-1.055442
H	7.149534	-0.911163	0.467080
H	6.465232	0.634682	0.991733
H	6.049412	-0.858816	1.850818
H	5.672121	-2.651186	-0.674115
H	3.965004	-2.310065	-0.979497
H	4.530161	-2.621288	0.677528

ωB97XD energy = -1154.81651541 a.u.

(2*S*,6*R*,7*S*,8*S*)-**1**, Conf. K

C	-2.508892	-2.920631	-0.173387
C	-2.976100	-1.510872	-0.406506
C	-3.186699	-0.554299	0.506754
C	-3.263995	-0.771851	1.995266
C	-0.214069	-1.896210	-0.152549
C	-1.039007	-3.058095	-0.653676
C	-1.978172	1.354744	-0.635971
C	-0.676211	1.244483	0.207472
C	0.445536	0.534190	-0.587269
C	0.028442	-0.865724	-0.972286
O	-2.119367	2.742599	-0.992572
C	-1.266825	3.540284	-0.308900
C	-0.371474	2.682015	0.520969
O	-1.281744	4.739468	-0.403982
C	0.512875	3.200242	1.369902
C	-3.264192	0.899782	0.068741
C	0.124511	-1.932012	1.313400
O	-3.250822	-3.876098	-0.919915
O	1.625141	0.564653	0.232384
C	2.808711	0.600931	-0.419378
O	2.898600	0.642377	-1.624688
C	3.974215	0.556011	0.535693
C	4.661471	-0.830383	0.565668
C	3.679056	-1.934569	0.963844
C	5.365410	-1.161685	-0.752183
H	-2.556125	-3.171771	0.893994
H	-2.970647	-1.227667	-1.460516
H	-2.443789	-0.258722	2.512921
H	-3.242874	-1.824523	2.283244
H	-1.058976	-3.077291	-1.747871
H	-0.651211	-4.023099	-0.309510
H	-4.193109	-0.336831	2.381171
H	-0.857120	0.670443	1.119646
H	0.658442	1.105260	-1.495700

H	-0.253028	-0.974966	-2.017246
H	1.154181	2.569216	1.975442
H	0.603216	4.278620	1.460851
H	-3.430623	1.549999	0.935361
H	-1.875895	0.817222	-1.581053
H	-4.099777	1.068256	-0.618547
H	0.712037	-1.072572	1.634229
H	0.701485	-2.837394	1.535055
H	-0.787529	-1.985782	1.918739
H	-4.179748	-3.791739	-0.686042
H	4.694232	1.310849	0.206561
H	3.626686	0.815679	1.539311
H	5.426898	-0.758321	1.348905
H	4.198678	-2.891713	1.071002
H	3.185015	-1.708196	1.914387
H	2.899522	-2.062546	0.203095
H	5.896749	-2.114954	-0.666577
H	6.092858	-0.389058	-1.019814
H	4.648438	-1.240512	-1.574463

ωB97XD energy = -1154.81650181 a.u.

(2*S*,6*R*,7*S*,8*S*)-**1**, Conf. L

C	-3.453569	-2.327475	-0.109295
C	-3.527437	-0.839581	-0.306361
C	-3.399644	0.116418	0.622429
C	-3.422742	-0.110413	2.111533
C	-0.960798	-1.984212	-0.178613
C	-2.100232	-2.852294	-0.659692
C	-1.778821	1.651247	-0.573012
C	-0.513287	1.191947	0.205156
C	0.330272	0.183495	-0.611052
C	-0.491546	-1.025600	-0.985738
O	-1.568187	3.031522	-0.924696
C	-0.490999	3.565218	-0.304952
C	0.194649	2.491125	0.471900
O	-0.189795	4.725351	-0.409865
C	1.248087	2.746999	1.244112
C	-3.104094	1.545158	0.195210
C	-0.552504	-2.191124	1.255868
O	-4.461161	-3.025729	-0.829476
O	1.470845	-0.127551	0.204970
C	2.545736	-0.640917	-0.429887
O	2.593112	-0.802909	-1.625998
C	3.629279	-0.999801	0.558092
C	5.015045	-1.175075	-0.068919
C	5.544879	0.149144	-0.625127
C	5.983307	-1.763742	0.959998
H	-3.523897	-2.581055	0.956200
H	-3.516303	-0.545729	-1.357624
H	-3.687132	-1.131691	2.391018
H	-4.151896	0.564730	2.573874
H	-2.169194	-2.831219	-1.751831
H	-1.984764	-3.897781	-0.353796
H	-2.451205	0.124773	2.563648
H	-0.796045	0.698565	1.138758
H	0.694553	0.666610	-1.523264

H	-0.856434	-1.007733	-2.010270
H	1.738646	1.963026	1.810355
H	1.633653	3.760255	1.308046
H	-3.048330	2.202522	1.070666
H	-1.869574	1.116014	-1.520282
H	-3.895359	1.941819	-0.449290
H	-0.123672	-3.193800	1.372373
H	-1.423788	-2.146970	1.919082
H	0.186419	-1.467296	1.598581
H	-5.319775	-2.694240	-0.550836
H	3.649653	-0.240194	1.348598
H	3.305505	-1.932391	1.039711
H	4.914885	-1.881513	-0.901022
H	6.530869	0.010949	-1.079526
H	4.878139	0.554459	-1.390885
H	5.647334	0.892595	0.175343
H	6.971238	-1.920891	0.516469
H	5.628735	-2.726733	1.343064
H	6.105748	-1.085661	1.813449

ωB97XD energy = -1154.81637577 a.u.

(2*S*,6*R*,7*S*,8*S*)-**1**, Conf. M

C	-2.656218	-2.934397	0.088631
C	-3.082229	-1.487771	0.114871
C	-2.922681	-0.603367	1.103864
C	-2.490574	-0.960234	2.499150
C	-0.447562	-1.897663	-0.683635
C	-1.104814	-3.078758	-0.009853
C	-1.958018	1.357278	-0.166655
C	-0.514981	1.462826	0.411083
C	0.440256	0.461590	-0.274840
C	0.060889	-0.937541	0.097158
O	-2.320479	2.671509	-0.628718
C	-1.333607	3.578596	-0.464798
C	-0.171649	2.912459	0.190542
O	-1.444521	4.726989	-0.807365
C	0.924454	3.596755	0.510809
C	-3.058372	0.876138	0.790123
C	-0.565494	-1.819633	-2.179011
O	-3.201317	-3.595369	-1.048606
O	1.762851	0.742938	0.220720
C	2.790412	0.380602	-0.575845
O	2.640297	-0.056470	-1.694484
C	4.116918	0.554204	0.117555
C	4.637986	-0.772442	0.723280
C	3.650288	-1.366177	1.732023
C	5.007042	-1.793618	-0.355079
H	-2.976788	-3.459088	0.999581
H	-3.381938	-1.112325	-0.865147
H	-1.534811	-0.481947	2.746169
H	-2.374082	-2.036123	2.646453
H	-0.910206	-4.017516	-0.538392
H	-0.709309	-3.178056	1.005952
H	-3.222511	-0.593004	3.227753
H	-0.520993	1.241817	1.484885
H	0.434162	0.627624	-1.354575

H	0.096937	-1.115010	1.172334
H	1.766967	3.126294	1.003610
H	0.978028	4.654444	0.269992
H	-3.028411	1.480342	1.704219
H	-1.957600	0.713039	-1.052160
H	-4.016373	1.080356	0.301476
H	0.022793	-1.005943	-2.606082
H	-1.614325	-1.707809	-2.478578
H	-0.218678	-2.757004	-2.625309
H	-4.160173	-3.527240	-1.015646
H	4.831786	0.920445	-0.624362
H	4.013940	1.301451	0.909359
H	5.554827	-0.503988	1.263530
H	3.359728	-0.633103	2.491336
H	2.737580	-1.711520	1.232858
H	4.096381	-2.227027	2.239642
H	5.440418	-2.688499	0.102936
H	5.738287	-1.384004	-1.059035
H	4.127453	-2.096471	-0.931411

ωB97XD energy = -1154.81633199 a.u.

(2*S*,6*R*,7*S*,8*S*)-**1**, Conf. N

C	-4.052183	-1.606269	0.071563
C	-3.701919	-0.155806	-0.149465
C	-3.292934	0.736174	0.757911
C	-3.339862	0.512894	2.244002
C	-1.531560	-1.895396	-0.249828
C	-2.784400	-2.464786	0.373007
C	-1.288789	1.711020	-0.430991
C	-0.100096	1.245369	0.462536
C	0.334714	-0.191649	0.094351
C	-0.731374	-1.150997	0.521895
O	-0.861266	2.911152	-1.101720
C	0.399432	3.274091	-0.780631
C	0.936830	2.308326	0.219539
O	0.936901	4.241065	-1.254721
C	2.129612	2.482725	0.783346
C	-2.616833	2.009444	0.278917
C	-1.369298	-2.079874	-1.732119
O	-4.654682	-2.159200	-1.094018
O	1.557484	-0.463901	0.807702
C	2.364176	-1.398853	0.263495
O	2.116807	-1.954015	-0.783742
C	3.592922	-1.647352	1.104257
C	4.906997	-1.204777	0.420120
C	5.241873	-2.039261	-0.818349
C	4.896672	0.291658	0.098804
H	-4.738485	-1.723985	0.921994
H	-3.603890	0.103194	-1.204938
H	-3.878196	1.332741	2.733355
H	-2.329929	0.502346	2.672070
H	-2.998057	-3.477917	0.017458
H	-2.657451	-2.508827	1.459281
H	-3.827885	-0.425511	2.516218
H	-0.394090	1.248551	1.518397
H	0.543839	-0.250378	-0.976189

H	-0.945974	-1.102535	1.589735
H	2.507124	1.796219	1.531720
H	2.743755	3.327470	0.485159
H	-2.432080	2.698923	1.110728
H	-1.471228	0.974141	-1.219522
H	-3.251383	2.538120	-0.439597
H	-2.148353	-1.536271	-2.279863
H	-1.500919	-3.136459	-1.985654
H	-0.386984	-1.767514	-2.090108
H	-5.442189	-1.649375	-1.306330
H	3.479870	-1.131934	2.061634
H	3.632711	-2.724546	1.297343
H	5.692480	-1.381795	1.166358
H	6.219513	-1.748196	-1.215736
H	5.276351	-3.107239	-0.580183
H	4.494275	-1.900622	-1.603993
H	5.855447	0.601015	-0.328174
H	4.719615	0.891507	0.997448
H	4.116268	0.537968	-0.630775

ωB97XD) energy = -1154.81572141 a.u.

(2*S*,6*R*,7*S*,8*S*)-**1**, Conf. O

C	-3.844353	-1.714837	-0.046055
C	-3.579717	-0.268528	-0.356354
C	-3.279803	0.713462	0.503033
C	-3.412643	0.631750	2.001143
C	-1.336626	-1.930822	-0.033294
C	-2.626695	-2.567767	-0.494110
C	-1.318008	1.737010	-0.718345
C	-0.227332	1.061673	0.160399
C	0.412523	-0.144289	-0.567546
C	-0.638707	-1.178908	-0.892744
O	-0.770091	2.994685	-1.156364
C	0.363457	3.321161	-0.494024
C	0.725193	2.194819	0.415178
O	0.935213	4.366642	-0.660824
C	1.726553	2.286746	1.286301
C	-2.658398	2.000203	-0.017003
C	-1.029251	-2.083745	1.432797
O	-4.961789	-2.232626	-0.756393
O	1.455320	-0.636297	0.290430
C	2.391622	-1.411716	-0.299264
O	2.386474	-1.661935	-1.482464
C	3.424580	-1.896862	0.687854
C	4.795617	-1.198662	0.527988
C	5.473527	-1.525971	-0.804329
C	4.683874	0.312752	0.741027
H	-3.997793	-1.858641	1.031093
H	-3.462739	-0.075005	-1.424022
H	-2.434986	0.720999	2.490896
H	-3.883580	-0.289702	2.347800
H	-2.661254	-2.628834	-1.586363
H	-2.754166	-3.581818	-0.099565
H	-4.016127	1.473219	2.360805
H	-0.660463	0.689735	1.092033
H	0.878775	0.197857	-1.496911

H	-0.952312	-1.178901	-1.934056
H	1.972102	1.469626	1.955154
H	2.320340	3.195141	1.329729
H	-2.485644	2.702258	0.806936
H	-1.497682	1.162357	-1.629008
H	-3.317728	2.505265	-0.730390
H	-0.126732	-1.554307	1.737253
H	-0.893278	-3.146183	1.667061
H	-1.867798	-1.733489	2.045607
H	-5.732856	-1.699507	-0.542140
H	3.048854	-1.743295	1.703087
H	3.546042	-2.971537	0.520558
H	5.422118	-1.606104	1.331918
H	6.474918	-1.084899	-0.834727
H	5.573525	-2.606741	-0.945162
H	4.902189	-1.134385	-1.650465
H	5.669784	0.784300	0.688740
H	4.249499	0.545686	1.718697
H	4.053487	0.778347	-0.025551

ωB97XD energy = -1154.81549534 a.u.

(1*R*,5*S*,6*S*,7*S*,10*R*)-**2**, Conf. A

C	3.455426	0.016246	-0.232388
C	2.931714	1.391632	-0.673720
C	1.581558	1.656898	-0.056824
C	0.567761	0.581269	-0.397932
C	1.047906	-0.782358	0.186624
C	2.425758	-1.083169	-0.466600
C	-0.887608	0.924539	-0.055073
C	-1.847838	-0.194442	-0.473220
C	-1.404902	-1.532408	0.127742
C	0.041833	-1.870682	-0.232340
C	-3.325660	0.163171	-0.189979
C	-4.282845	-0.830132	-0.857330
C	-3.655447	0.296940	1.300880
O	-1.302311	2.096362	-0.746964
C	1.365699	2.687544	0.766564
O	3.007913	-2.289689	0.004852
H	0.587984	0.472177	-1.495624
C	1.175546	-0.745179	1.716706
H	3.715540	0.038344	0.831439
H	4.368771	-0.238675	-0.778223
H	3.646793	2.172914	-0.400605
H	2.834145	1.405734	-1.768253
H	2.253648	-1.165100	-1.554072
H	-0.975432	1.088543	1.030455
H	-1.749974	-0.272941	-1.567510
H	-2.058158	-2.332377	-0.234768
H	-1.522238	-1.513430	1.217433
H	0.108578	-2.013978	-1.321281
H	0.313354	-2.825001	0.237073
H	-3.489567	1.141410	-0.654186
H	-4.050427	-0.957291	-1.920397
H	-4.242740	-1.816862	-0.381998
H	-5.315915	-0.475565	-0.781103

H	-4.694299	0.617754	1.429155
H	-3.022827	1.037805	1.799154
H	-3.542799	-0.657499	1.827849
H	-0.618745	2.767202	-0.650597
H	0.416688	2.848258	1.271301
H	2.155781	3.399659	0.987389
H	2.475955	-3.033174	-0.291047
H	1.810161	0.077304	2.053721
H	0.202806	-0.617216	2.198797
H	1.614701	-1.680605	2.074133

ωB97XD energy = -737.554870274 a.u.

(1*R*,5*S*,6*S*,7*S*,10*R*)-**2**, Conf. B

C	3.405980	0.429614	-0.242817
C	2.665651	1.727129	-0.600009
C	1.301586	1.741162	0.042918
C	0.462098	0.541815	-0.357233
C	1.161618	-0.760214	0.138725
C	2.558712	-0.801790	-0.540989
C	-1.023398	0.632489	0.019990
C	-1.810062	-0.591794	-0.460357
C	-1.143775	-1.879721	0.040699
C	0.333013	-1.966221	-0.339948
C	-3.317838	-0.577876	-0.095210
C	-3.589190	-0.161589	1.355024
C	-4.170463	0.255188	-1.058214
O	-1.616779	1.777460	-0.581444
C	0.947376	2.671898	0.934710
O	3.328811	-1.929209	-0.151145
H	0.479972	0.503422	-1.459732
C	1.306085	-0.796503	1.667475
H	3.671043	0.430291	0.819993
H	4.341741	0.355056	-0.804803
H	3.253210	2.595716	-0.288569
H	2.548635	1.786886	-1.691103
H	2.383728	-0.841326	-1.630318
H	-1.114272	0.705887	1.115039
H	-1.737866	-0.587624	-1.558823
H	-1.673503	-2.739775	-0.383742
H	-1.257999	-1.961593	1.128632
H	0.408506	-2.034420	-1.435758
H	0.755703	-2.891992	0.071396
H	-3.649952	-1.621006	-0.199903
H	-2.977703	-0.723591	2.069660
H	-3.391706	0.906081	1.498441
H	-4.639208	-0.339375	1.608307
H	-3.929403	1.316972	-0.975088
H	-5.235105	0.117196	-0.838371
H	-4.000957	-0.047326	-2.097176
H	-1.029072	2.528860	-0.450688
H	-0.003410	2.653173	1.460828
H	1.625996	3.479946	1.192876
H	2.918510	-2.726524	-0.496440
H	1.908162	-1.660788	1.961254
H	1.790496	0.102349	2.055052
H	0.333848	-0.874025	2.161155

ωB97XD energy = -737.553303664 a.u.

(1*R*,5*S*,6*R*,7*R*,8*R*,10*R*)-**3**, Conf. A

C	4.268154	-1.033405	0.388942
C	4.241129	0.414278	0.902219
C	2.833937	0.879748	1.191079
C	1.912496	0.686477	0.008607
C	1.836389	-0.836821	-0.382140
C	3.278204	-1.250909	-0.756148
C	0.495129	1.206022	0.122519
C	-0.223235	1.096602	-1.222527
C	-0.432364	-0.353557	-1.617604
C	0.934724	-1.051058	-1.627760
O	0.381314	2.601741	0.460954
C	-0.775958	3.093805	-0.062841
C	-1.327513	2.093365	-1.037578
O	3.249305	-2.608841	-1.171367
C	2.469026	1.391202	2.366645
C	-2.542328	2.193765	-1.568770
O	-1.215393	4.171414	0.238446
H	2.377369	1.212426	-0.841990
C	1.329231	-1.699629	0.788890
O	-1.297030	-1.003246	-0.669004
C	-2.572631	-1.252785	-1.016946
O	-3.038661	-0.980431	-2.100941
C	-3.324527	-1.952146	0.089841
C	-2.996040	-1.501246	1.520554
C	-3.293886	-0.013469	1.721446
C	-3.778300	-2.356803	2.519808
H	4.029761	-1.729547	1.199860
H	5.279390	-1.281234	0.042013
H	4.672307	1.071883	0.133452
H	4.868986	0.511627	1.792291
H	3.586244	-0.619728	-1.608335
H	-0.054447	0.647382	0.890145
H	0.458152	1.516327	-1.979374
H	-0.896977	-0.429334	-2.603371
H	1.462981	-0.674044	-2.513552
H	0.793607	-2.122791	-1.787294
H	4.144619	-2.892364	-1.374459
H	3.188397	1.487137	3.175321
H	1.464954	1.755447	2.554637
H	-2.925404	1.451801	-2.262032
H	-3.178118	3.029459	-1.291296
H	0.262998	-1.549168	0.958756
H	1.852259	-1.470734	1.719875
H	1.484146	-2.755592	0.553570
H	-4.389888	-1.820520	-0.123175
H	-3.107971	-3.022852	-0.019952
H	-1.925889	-1.666855	1.690770
H	-4.356010	0.195564	1.546348
H	-3.056829	0.295993	2.744104
H	-2.713862	0.613872	1.038021
H	-3.557520	-3.422137	2.395507
H	-4.857943	-2.218875	2.386973

H	-3.531408	-2.078848	3.549059

ωB97XD energy = -1154.85459973 a.u.

(1*R*,5*S*,6*R*,7*R*,8*R*,10*R*)-**3**, Conf. B

C	4.443866	-0.690815	0.421276
C	4.322509	0.830776	0.592946
C	2.898846	1.248591	0.872539
C	1.933304	0.723674	-0.165291
C	1.966875	-0.849996	-0.199302
C	3.417280	-1.234656	-0.572958
C	0.484462	1.150773	-0.062092
C	-0.299682	0.683724	-1.288361
C	-0.399341	-0.829993	-1.338664
C	1.022518	-1.405889	-1.298260
O	0.263152	2.574930	-0.036742
C	-0.964402	2.843695	-0.562752
C	-1.478260	1.607539	-1.243376
O	3.483068	-2.649732	-0.672750
C	2.555397	1.987662	1.927271
C	-2.724602	1.487107	-1.690198
O	-1.483603	3.924377	-0.475157
H	2.301707	1.074654	-1.143821
C	1.597960	-1.457697	1.167894
O	-1.142976	-1.315452	-0.207750
C	-2.401967	-1.757587	-0.391392
O	-2.957014	-1.775839	-1.467997
C	-3.013889	-2.208211	0.911052
C	-3.679434	-1.064269	1.713683
C	-2.674330	-0.007994	2.181858
C	-4.836098	-0.420413	0.945905
H	4.308375	-1.194241	1.384105
H	5.452719	-0.941077	0.069040
H	4.652139	1.319309	-0.335520
H	4.989618	1.176429	1.387602
H	3.623615	-0.792567	-1.563573
H	0.035542	0.744517	0.851852
H	0.293370	0.972593	-2.170759
H	-0.910535	-1.159411	-2.246305
H	1.467003	-1.200450	-2.281231
H	0.969464	-2.493528	-1.206317
H	4.387276	-2.907331	-0.870742
H	3.305288	2.318468	2.640509
H	1.534506	2.309289	2.101949
H	-3.072372	0.577823	-2.170444
H	-3.421783	2.309772	-1.560339
H	1.823747	-2.527120	1.159131
H	0.534317	-1.344539	1.378927
H	2.153751	-0.991247	1.984188
H	-3.766503	-2.961128	0.664490
H	-2.234841	-2.671943	1.523344
H	-4.094791	-1.550461	2.605575
H	-2.305780	0.581615	1.336784
H	-3.149054	0.684471	2.883607
H	-1.811761	-0.462475	2.680591
H	-4.474512	0.099875	0.053047
H	-5.349876	0.313229	1.574685

H	-5.567725	-1.166918	0.622109

ωB97XD energy = -1154.85457756 a.u.

(1*R*,5*S*,6*R*,7*R*,8*R*,10*R*)-**3**, Conf. C

C	4.371190	-0.938323	0.563440
C	4.312186	0.561924	0.887821
C	2.894391	1.029043	1.114343
C	1.982740	0.668194	-0.036053
C	1.941116	-0.892732	-0.234507
C	3.394177	-1.318695	-0.549448
C	0.553209	1.164517	0.014109
C	-0.163189	0.872178	-1.304620
C	-0.332888	-0.621702	-1.520125
C	1.053028	-1.276852	-1.447768
O	0.405824	2.588569	0.177119
C	-0.763069	2.983914	-0.399243
C	-1.291239	1.857847	-1.241022
O	3.396932	-2.717958	-0.792700
C	2.513588	1.674752	2.216438
C	-2.507568	1.865651	-1.778176
O	-1.229282	4.079268	-0.231611
H	2.438279	1.096206	-0.944548
C	1.445972	-1.616451	1.032582
O	-1.175121	-1.180883	-0.496310
C	-2.452147	-1.484439	-0.793956
O	-2.941615	-1.334812	-1.891954
C	-3.190110	-2.004925	0.412071
C	-3.938138	-0.888823	1.174582
C	-4.822157	-1.516711	2.254518
C	-2.977905	0.135950	1.783601
H	4.141370	-1.529932	1.455663
H	5.389742	-1.207391	0.255672
H	4.731742	1.123385	0.040418
H	4.935522	0.786569	1.757914
H	3.693718	-0.791974	-1.472591
H	0.019249	0.693429	0.847950
H	0.507340	1.213427	-2.109093
H	-0.795873	-0.827192	-2.488170
H	1.576017	-0.993327	-2.370867
H	0.940972	-2.363373	-1.479953
H	4.299001	-3.004613	-0.957964
H	3.227741	1.888090	3.007090
H	1.500988	2.036685	2.357271
H	-2.874499	1.035516	-2.372945
H	-3.160789	2.715779	-1.604287
H	0.378296	-1.461420	1.189802
H	1.969471	-1.273025	1.927335
H	1.614309	-2.690553	0.920888
H	-3.911291	-2.742614	0.050540
H	-2.485548	-2.499117	1.089076
H	-4.586528	-0.373601	0.454248
H	-5.540236	-2.223417	1.826368
H	-4.212135	-2.056699	2.988587
H	-5.384788	-0.746242	2.790617
H	-2.410016	0.664649	1.014515
H	-2.264823	-0.352878	2.458593

H	-3.530268	0.884254	2.360003

ωB97XD energy = -1154.85432165 a.u.

(1*R*,5*S*,6*R*,7*R*,8*R*,10*R*)-**3**, Conf. D

C	3.773184	-1.969425	0.042433
C	4.231309	-0.626730	0.630477
C	3.060368	0.213855	1.080192
C	2.033391	0.403740	-0.012725
C	1.471284	-0.990049	-0.485946
C	2.687687	-1.782229	-1.017363
C	0.847461	1.298633	0.280573
C	0.024652	1.527050	-0.986360
C	-0.627622	0.247177	-1.466963
C	0.460155	-0.819028	-1.649920
O	1.167909	2.634725	0.718146
C	0.150054	3.470589	0.365224
C	-0.746123	2.751136	-0.602052
O	2.215642	-3.027759	-1.510344
C	2.960899	0.713148	2.312156
C	-1.946738	3.192594	-0.964344
O	0.059605	4.593442	0.784110
H	2.564073	0.844694	-0.872913
C	0.808944	-1.758930	0.673252
O	-1.592874	-0.182333	-0.490882
C	-2.894728	-0.198112	-0.832174
O	-3.307140	0.128290	-1.922898
C	-3.772257	-0.622642	0.320322
C	-3.183590	-1.662027	1.283805
C	-4.175542	-1.929153	2.418550
C	-2.818988	-2.958452	0.556506
H	3.389579	-2.622232	0.833314
H	4.630944	-2.484219	-0.408870
H	4.780832	-0.071268	-0.143563
H	4.927686	-0.792157	1.457233
H	3.113283	-1.201406	-1.854473
H	0.220955	0.852080	1.061842
H	0.734042	1.824415	-1.774464
H	-1.151262	0.403623	-2.412407
H	1.011884	-0.540067	-2.557744
H	-0.008584	-1.783438	-1.862026
H	2.964334	-3.546669	-1.815907
H	3.734665	0.523588	3.051097
H	2.133483	1.344136	2.617788
H	-2.571554	2.637412	-1.657987
H	-2.323603	4.125666	-0.556299
H	-0.154278	-1.322508	0.937222
H	1.432883	-1.762466	1.569864
H	0.631793	-2.793446	0.368362
H	-4.013770	0.297439	0.869052
H	-4.708869	-0.983051	-0.116673
H	-2.270610	-1.240890	1.720624
H	-4.421690	-1.009965	2.960374
H	-5.109256	-2.351269	2.028135
H	-3.762298	-2.643763	3.137144
H	-2.405561	-3.691493	1.256334
H	-2.070874	-2.787880	-0.223646

H	-3.706173	-3.402652	0.089683

ωB97XD energy = -1154.85413979 a.u.

(1*R*,5*S*,6*R*,7*R*,8*R*,10*R*)-**3**, Conf. E

C	4.433845	-1.284791	0.439337
C	4.530162	0.204386	0.802281
C	3.176707	0.798416	1.110386
C	2.178775	0.557720	0.001054
C	1.973202	-0.986597	-0.223893
C	3.365124	-1.552310	-0.620387
C	0.810707	1.195163	0.128198
C	0.007782	0.999293	-1.157554
C	-0.317786	-0.462848	-1.395009
C	0.996545	-1.253193	-1.398465
O	0.812061	2.623974	0.314151
C	-0.343073	3.138883	-0.194289
C	-1.019068	2.080946	-1.018914
O	3.311919	-2.927451	-0.964846
C	2.912599	1.449140	2.243470
C	-2.254963	2.205452	-1.493097
O	-0.690654	4.271425	0.008239
H	2.632184	0.955693	-0.922096
C	1.461779	-1.680331	1.052315
O	-1.177736	-0.951812	-0.347943
C	-2.467011	-1.206830	-0.639809
O	-2.956703	-1.031073	-1.733340
C	-3.207738	-1.755970	0.554401
C	-4.591630	-1.116265	0.739311
C	-5.370762	-1.855812	1.828291
C	-4.467248	0.373485	1.067439
H	4.205066	-1.865530	1.342860
H	5.394903	-1.647991	0.061725
H	4.959855	0.745029	-0.053283
H	5.214804	0.346397	1.643184
H	3.664906	-1.051047	-1.550504
H	0.270137	0.766670	0.980465
H	0.668126	1.295952	-1.987733
H	-0.836218	-0.598214	-2.346821
H	1.503283	-0.999750	-2.339010
H	0.779687	-2.323125	-1.451708
H	3.325694	-3.452500	-0.159104
H	3.679077	1.572238	3.003736
H	1.947822	1.904352	2.439359
H	-2.720921	1.422453	-2.083694
H	-2.821197	3.107091	-1.278368
H	0.438413	-1.387688	1.288188
H	2.083766	-1.454956	1.921395
H	1.442936	-2.765475	0.904140
H	-3.318401	-2.833734	0.381199
H	-2.596932	-1.629584	1.453801
H	-5.128059	-1.219935	-0.210933
H	-4.851601	-1.791646	2.792198
H	-6.365115	-1.417463	1.957928
H	-5.499039	-2.915380	1.583633
H	-3.926066	0.519778	2.010103
H	-5.454888	0.832299	1.175049

H	-3.931002	0.917928	0.283809

ωB97XD energy = -1154.85351123 a.u.

(1*R*,5*S*,6*R*,7*R*,8*R*,10*R*)-**3**, Conf. F

C	3.210072	-2.647372	0.029133
C	3.967779	-1.439693	0.597625
C	3.030579	-0.350175	1.058992
C	2.054990	0.062579	-0.019825
C	1.178932	-1.169662	-0.461226
C	2.174085	-2.231544	-1.007914
C	1.114699	1.211131	0.279237
C	0.342910	1.606768	-0.978026
C	-0.599287	0.516195	-1.432766
C	0.204817	-0.782963	-1.606229
O	1.746021	2.442122	0.686127
C	0.949752	3.488632	0.329813
C	-0.107434	2.985543	-0.611769
O	1.525643	-3.419155	-1.436679
C	3.069804	0.168091	2.286490
C	-1.173029	3.694085	-0.968752
O	1.133038	4.608578	0.724951
H	2.658740	0.358961	-0.894317
C	0.387583	-1.756661	0.719589
O	-1.638909	0.382115	-0.449973
C	-2.859226	0.009974	-0.889241
O	-3.101878	-0.220250	-2.052578
C	-3.848290	-0.077014	0.244331
C	-3.449617	-1.095141	1.328534
C	-4.502025	-1.111303	2.438419
C	-3.252076	-2.490687	0.733336
H	2.704919	-3.191695	0.833974
H	3.906602	-3.348931	-0.439244
H	4.618523	-1.028026	-0.187751
H	4.621434	-1.755060	1.415713
H	2.702372	-1.774883	-1.862861
H	0.418779	0.932717	1.079342
H	1.088973	1.715834	-1.781113
H	-1.071649	0.769696	-2.385125
H	0.778963	-0.657314	-2.535764
H	-0.505646	-1.598322	-1.780776
H	1.054734	-3.251740	-2.257199
H	3.793438	-0.187747	3.014616
H	2.412392	0.971797	2.599783
H	-1.921768	3.295557	-1.646753
H	-1.312068	4.697834	-0.579138
H	-0.447264	-1.113142	0.999910
H	1.015787	-1.891703	1.602426
H	-0.018914	-2.730788	0.434429
H	-3.939255	0.918514	0.693999
H	-4.813496	-0.344678	-0.194397
H	-2.499361	-0.763494	1.763901
H	-5.475368	-1.429229	2.046576
H	-4.217236	-1.809279	3.231632
H	-4.625900	-0.120998	2.888598
H	-2.479313	-2.497798	-0.041939
H	-4.181782	-2.855329	0.281621

H	-2.948056	-3.202760	1.506575

ωB97XD energy = -1154.85310905 a.u.

(1*R*,5*S*,6*R*,7*R*,8*R*,10*R*)-**3**, Conf. G

C	2.784719	-3.105465	-0.004160
C	3.779562	-2.049883	0.499430
C	3.072857	-0.809064	0.990140
C	2.127112	-0.237880	-0.041714
C	1.026445	-1.297468	-0.427971
C	1.787590	-2.515138	-1.001072
C	1.414863	1.055451	0.293673
C	0.652460	1.575762	-0.923435
C	-0.480719	0.657605	-1.321750
C	0.087665	-0.753115	-1.536920
O	2.270355	2.160851	0.650483
C	1.644104	3.327529	0.327891
C	0.460889	3.009383	-0.541616
O	0.826171	-3.476275	-1.411743
C	3.270409	-0.299825	2.206146
C	-0.493578	3.885624	-0.835264
O	2.040783	4.400197	0.697215
H	2.724712	-0.050702	-0.949394
C	0.197487	-1.727077	0.797760
O	-1.466975	0.684735	-0.274769
C	-2.754655	0.516803	-0.631265
O	-3.117163	0.384317	-1.778961
C	-3.673228	0.497970	0.563991
C	-4.468206	-0.817810	0.645800
C	-3.533758	-2.016984	0.822561
C	-5.491264	-0.741707	1.779846
H	2.232969	-3.539119	0.836395
H	3.330355	-3.927168	-0.485438
H	4.445933	-1.766568	-0.328343
H	4.412075	-2.469251	1.286774
H	2.345473	-2.161983	-1.886115
H	0.727572	0.902757	1.134054
H	1.361980	1.560480	-1.765594
H	-0.960760	0.995080	-2.243666
H	0.641797	-0.718901	-2.484601
H	-0.733773	-1.456752	-1.692894
H	1.282908	-4.250850	-1.750245
H	3.959607	-0.774062	2.899550
H	2.784026	0.609341	2.542150
H	-1.341224	3.616187	-1.458159
H	-0.438715	4.897606	-0.445962
H	-0.508061	-0.950310	1.094391
H	0.830145	-1.952311	1.659383
H	-0.378777	-2.621384	0.547109
H	-3.095109	0.655855	1.479528
H	-4.368021	1.337510	0.450338
H	-5.003276	-0.933306	-0.304100
H	-2.957727	-1.928328	1.751557
H	-4.103573	-2.950096	0.868291
H	-2.822639	-2.104855	-0.005226
H	-6.189556	0.089201	1.635671
H	-4.993265	-0.601168	2.746730

H	-6.074967	-1.665777	1.837573

ωB97XD energy = -1154.85307473 a.u.

(1*R*,5*S*,6*R*,7*R*,8*R*,10*R*)-**3**, Conf. H

C	4.242232	-1.300961	0.464965
C	4.405107	0.194978	0.771040
C	3.076407	0.853010	1.057089
C	2.067578	0.616416	-0.043607
C	1.807853	-0.926870	-0.228479
C	3.174197	-1.547684	-0.600828
C	0.719063	1.294616	0.076273
C	-0.093903	1.092654	-1.201838
C	-0.464371	-0.358956	-1.407932
C	0.823622	-1.194965	-1.397899
O	0.754034	2.728553	0.228967
C	-0.395560	3.255820	-0.277924
C	-1.100156	2.192063	-1.071277
O	2.974922	-2.934544	-0.830385
C	2.840252	1.550151	2.168533
C	-2.345630	2.310221	-1.518127
O	-0.719716	4.399157	-0.098288
H	2.528271	0.978657	-0.977653
C	1.274450	-1.573335	1.065125
O	-1.357305	-0.731958	-0.346373
C	-2.253989	-1.708551	-0.587257
O	-2.369104	-2.256519	-1.659719
C	-3.062326	-2.009203	0.649610
C	-3.777381	-0.778702	1.236147
C	-4.772388	-0.190868	0.233138
C	-4.473223	-1.152400	2.546057
H	3.971831	-1.848099	1.374023
H	5.198321	-1.712334	0.117009
H	4.856230	0.687511	-0.102697
H	5.094740	0.338461	1.607525
H	3.502366	-1.069164	-1.540263
H	0.172580	0.898422	0.939895
H	0.567601	1.360771	-2.040348
H	-0.983927	-0.510758	-2.357815
H	1.334868	-0.983778	-2.346787
H	0.569001	-2.257192	-1.422064
H	3.819916	-3.341510	-1.038950
H	3.612171	1.666950	2.924246
H	1.893329	2.046082	2.351961
H	-2.821343	1.518871	-2.089428
H	-2.913113	3.213207	-1.315289
H	0.243181	-1.278047	1.262326
H	1.872355	-1.296571	1.936420
H	1.297707	-2.660999	0.959115
H	-3.784537	-2.786851	0.386052
H	-2.373781	-2.423763	1.396461
H	-3.015490	-0.020174	1.451344
H	-5.260995	0.696606	0.646580
H	-4.280170	0.104914	-0.698705
H	-5.549605	-0.922190	-0.017199
H	-4.967868	-0.280274	2.984667
H	-3.760200	-1.539967	3.281180

H	-5.237088	-1.920911	2.377932

ωB97XD energy = -1154.85289376 a.u.

(1*R*,5*S*,6*R*,7*R*,8*R*,10*R*)-**3**, Conf. I

C	3.422584	-2.580390	-0.086247
C	4.201752	-1.308538	0.281286
C	3.287837	-0.226328	0.804448
C	2.147372	0.071169	-0.142523
C	1.274047	-1.220339	-0.369485
C	2.224887	-2.271997	-0.985249
C	1.209376	1.204153	0.216236
C	0.231801	1.476297	-0.925858
C	-0.709018	0.310358	-1.152448
C	0.132410	-0.951488	-1.384654
O	1.838947	2.483389	0.431907
C	0.944452	3.470207	0.139894
C	-0.224422	2.859288	-0.578336
O	1.464671	-3.439226	-1.263155
C	3.479970	0.378415	1.976629
C	-1.366953	3.500909	-0.802961
O	1.136758	4.622360	0.423094
H	2.602220	0.326123	-1.114191
C	0.688267	-1.752211	0.952376
O	-1.551902	0.137110	-0.000063
C	-2.871854	0.378197	-0.121534
O	-3.390305	0.775364	-1.142199
C	-3.605300	0.058254	1.155564
C	-4.181910	-1.378585	1.173638
C	-5.254529	-1.583284	0.101153
C	-3.085897	-2.442947	1.075169
H	3.067553	-3.083723	0.818937
H	4.088153	-3.283188	-0.603316
H	4.707468	-0.930100	-0.618862
H	4.981840	-1.539967	1.011830
H	2.601608	-1.852391	-1.934810
H	0.660150	0.963713	1.134430
H	0.834747	1.559205	-1.843988
H	-1.353463	0.491206	-2.015681
H	0.570657	-0.847467	-2.386340
H	-0.523511	-1.824774	-1.425288
H	2.049899	-4.117078	-1.611274
H	4.316702	0.103034	2.612769
H	2.836328	1.177305	2.328613
H	-2.196938	3.023686	-1.315484
H	-1.484826	4.524497	-0.460060
H	-0.103502	-1.102801	1.326325
H	1.450337	-1.835140	1.730190
H	0.252756	-2.740276	0.782564
H	-2.921067	0.186963	1.998670
H	-4.422466	0.778424	1.248625
H	-4.662171	-1.481543	2.155016
H	-5.711596	-2.572391	0.205489
H	-6.045751	-0.831416	0.179998
H	-4.830170	-1.509806	-0.905130
H	-2.590299	-2.418112	0.098486
H	-3.514026	-3.441832	1.203942

H	-2.315212	-2.300984	1.839277

ωB97XD energy = -1154.85268700 a.u.

(1*R*,5*S*,6*R*,7*R*,8*R*,10*R*)-**3**, Conf. J

C	3.908413	-2.209078	0.221947
C	4.480365	-0.832525	0.591104
C	3.392165	0.129103	1.003959
C	2.300773	0.241852	-0.036094
C	1.628822	-1.162209	-0.282085
C	2.757966	-2.095134	-0.778505
C	1.193467	1.243768	0.212300
C	0.283074	1.349592	-1.009681
C	-0.468648	0.064039	-1.270285
C	0.549937	-1.078733	-1.393610
O	1.623314	2.603818	0.427699
C	0.630216	3.449256	0.032504
C	-0.384835	2.662298	-0.747263
O	2.183062	-3.362256	-1.061219
C	3.409447	0.784960	2.164288
C	-1.587679	3.124822	-1.070248
O	0.640066	4.624323	0.284591
H	2.791878	0.531684	-0.979880
C	1.009617	-1.732079	1.008926
O	-1.383140	-0.137905	-0.179142
C	-2.522749	-0.807857	-0.435046
O	-2.830977	-1.202070	-1.536886
C	-3.320109	-1.018667	0.828612
C	-4.826092	-1.167190	0.590898
C	-5.520854	-1.601783	1.883066
C	-5.429621	0.133270	0.053572
H	3.550120	-2.724891	1.118860
H	4.700417	-2.833650	-0.210422
H	4.996469	-0.416247	-0.286208
H	5.228414	-0.931790	1.382621
H	3.151304	-1.659394	-1.713862
H	0.611162	0.952965	1.094608
H	0.942882	1.494673	-1.879540
H	-1.052702	0.123322	-2.192446
H	1.049341	-0.940545	-2.362043
H	0.021626	-2.032921	-1.459671
H	2.875035	-3.962017	-1.352043
H	4.223313	0.641717	2.869884
H	2.641766	1.499481	2.440517
H	-2.298859	2.520184	-1.624921
H	-1.880506	4.128597	-0.778088
H	0.105841	-1.190826	1.291248
H	1.704688	-1.681516	1.849865
H	0.738722	-2.778324	0.845217
H	-2.916460	-1.929785	1.290831
H	-3.107950	-0.199001	1.523832
H	-4.965726	-1.948658	-0.164949
H	-5.111618	-2.543988	2.262924
H	-5.401202	-0.842829	2.665854
H	-6.593482	-1.743266	1.718798
H	-6.503047	0.016861	-0.124819
H	-4.965371	0.428025	-0.892061

H	-5.296702	0.948634	0.775394

ωB97XD energy = -1154.85226055 a.u.

(1*R*,5*S*,6*R*,7*R*,8*S*,10*R*)-**3**, Conf. A

C	4.300725	-1.808885	-0.384257
C	4.679051	-0.360321	-0.723570
C	3.748852	0.624846	-0.058337
C	2.296075	0.342235	-0.367456
C	1.898137	-1.100074	0.121439
C	2.817981	-2.079932	-0.642012
C	1.267704	1.309181	0.177105
C	-0.133898	0.979154	-0.341220
C	-0.578803	-0.357236	0.207083
C	0.428603	-1.421287	-0.245081
O	1.455222	2.685859	-0.201045
C	0.247419	3.316095	-0.170076
C	-0.830554	2.274972	-0.058109
O	2.454622	-3.400970	-0.272397
C	4.184571	1.613327	0.722759
C	-2.093528	2.568200	0.234408
O	0.136372	4.510251	-0.244793
H	2.194792	0.348581	-1.464964
C	2.089585	-1.267958	1.641157
O	-1.876111	-0.648584	-0.340882
C	-2.655844	-1.508327	0.348151
O	-2.312319	-2.032929	1.382667
C	-3.991231	-1.694396	-0.327478
C	-4.809891	-0.392665	-0.413629
C	-6.116120	-0.642700	-1.169505
C	-5.085246	0.184888	0.977327
H	4.524193	-2.024041	0.666042
H	4.903808	-2.498091	-0.988812
H	4.611531	-0.222008	-1.812490
H	5.716936	-0.162647	-0.441556
H	2.618317	-1.936881	-1.718454
H	1.277815	1.280956	1.275098
H	-0.059756	0.881304	-1.435985
H	-0.673649	-0.327412	1.296547
H	0.335237	-1.512223	-1.335750
H	0.159613	-2.387998	0.185734
H	3.038476	-4.021641	-0.716177
H	5.246845	1.744537	0.909226
H	3.514323	2.336958	1.173662
H	-2.853397	1.798129	0.299741
H	-2.379514	3.600779	0.410169
H	3.044877	-0.863105	1.983037
H	2.045898	-2.329849	1.894231
H	1.303541	-0.765036	2.210101
H	-3.813274	-2.081072	-1.337225
H	-4.538167	-2.452655	0.239845
H	-4.216877	0.331737	-0.985926
H	-6.688312	0.284697	-1.271230
H	-5.929016	-1.035700	-2.174010
H	-6.742766	-1.365773	-0.634383
H	-4.163798	0.366061	1.541193

H	-5.696927	-0.505799	1.568785
H	-5.624558	1.134478	0.902963

ωB97XD energy = -1154.85360791 a.u.

(1*R*,5*S*,6*R*,7*R*,8*S*,10*R*)-**3**, Conf. B

C	2.993712	-3.102520	-0.297113
C	3.948706	-2.021774	-0.823417
C	3.644388	-0.672269	-0.218006
C	2.197917	-0.272228	-0.402431
C	1.244077	-1.329965	0.268507
C	1.532203	-2.675994	-0.433488
C	1.781245	1.094020	0.097285
C	0.337201	1.413753	-0.295961
C	-0.609020	0.472468	0.410692
C	-0.239738	-0.965524	0.027476
O	2.528924	2.204220	-0.433809
C	1.747279	3.320349	-0.383242
C	0.333948	2.898100	-0.094985
O	0.647938	-3.647962	0.102863
C	4.562892	0.056034	0.416756
C	-0.623665	3.746554	0.265368
O	2.182206	4.425125	-0.567614
H	1.994005	-0.297616	-1.485252
C	1.497379	-1.456358	1.782899
O	-1.941286	0.754068	-0.053115
C	-2.959920	0.385817	0.749975
O	-2.801698	-0.078152	1.856677
C	-4.299305	0.585567	0.088668
C	-4.999004	-0.765396	-0.160604
C	-6.389476	-0.532748	-0.753336
C	-4.157694	-1.668862	-1.066462
H	3.204996	-3.316746	0.755983
H	3.150760	-4.036538	-0.851344
H	3.834265	-1.947803	-1.914686
H	4.987289	-2.304109	-0.630056
H	1.308244	-2.534683	-1.505308
H	1.891826	1.137416	1.189184
H	0.246553	1.228577	-1.378078
H	-0.585176	0.623563	1.493766
H	-0.476281	-1.085836	-1.038338
H	-0.870259	-1.664966	0.581424
H	0.839121	-4.499845	-0.298234
H	5.580939	-0.308824	0.522221
H	4.349292	1.037504	0.825552
H	-1.634652	3.406083	0.461438
H	-0.400990	4.804370	0.365981
H	1.087144	-0.608835	2.337436
H	2.563088	-1.512965	2.016320
H	1.007921	-2.358605	2.156827
H	-4.912654	1.195910	0.759507
H	-4.170510	1.124163	-0.855043
H	-5.110441	-1.257562	0.812993
H	-6.319069	-0.037398	-1.729120
H	-6.911787	-1.483450	-0.898839
H	-7.005010	0.093921	-0.099766
H	-3.982479	-1.190255	-2.037412

H	-4.669696	-2.619164	-1.246141
H	-3.183863	-1.898835	-0.622526

ωB97XD energy = -1154.85327460 a.u.

(1*R*,5*S*,6*R*,7*R*,8*S*,10*R*)-**3**, Conf. C

C	3.263044	-2.854071	-0.404638
C	4.071866	-1.675417	-0.964721
C	3.654425	-0.367690	-0.336100
C	2.165748	-0.124260	-0.442374
C	1.367379	-1.280611	0.268572
C	1.759321	-2.584586	-0.462213
C	1.635816	1.189343	0.090360
C	0.146221	1.353764	-0.215971
C	-0.650126	0.315502	0.538408
C	-0.158554	-1.072740	0.112814
O	2.229741	2.373225	-0.475887
C	1.337637	3.399332	-0.374213
C	-0.002813	2.829009	-0.004919
O	1.013090	-3.647868	0.109425
C	4.519600	0.456228	0.255286
C	-1.020377	3.572325	0.418089
O	1.639434	4.544042	-0.579703
H	1.908808	-0.167342	-1.513311
C	1.716455	-1.386066	1.765593
O	-2.029741	0.470300	0.168304
C	-2.959770	0.011363	1.031900
O	-2.689236	-0.461788	2.111607
C	-4.346683	0.168607	0.462010
C	-4.543779	-0.584899	-0.866732
C	-4.250299	-2.078559	-0.705714
C	-5.962184	-0.352640	-1.390321
H	3.548180	-3.051753	0.634132
H	3.489186	-3.762648	-0.977131
H	3.901506	-1.610929	-2.049235
H	5.142044	-1.846879	-0.819776
H	1.465691	-2.463491	-1.519596
H	1.803084	1.244122	1.174444
H	0.012037	1.161320	-1.292321
H	-0.567546	0.460923	1.619364
H	-0.438951	-1.206180	-0.940900
H	-0.682093	-1.840331	0.687787
H	1.280064	-4.474078	-0.302005
H	5.575936	0.207201	0.307076
H	4.219439	1.408360	0.678872
H	-1.976459	3.128683	0.673277
H	-0.901617	4.647407	0.511837
H	1.249431	-0.590238	2.351023
H	2.793179	-1.327152	1.940289
H	1.347641	-2.337229	2.156650
H	-5.050286	-0.193496	1.216835
H	-4.532412	1.237741	0.305293
H	-3.833769	-0.169495	-1.591802
H	-3.217892	-2.260032	-0.388638
H	-4.913666	-2.526671	0.043096
H	-4.403980	-2.605812	-1.652368
H	-6.110035	-0.857135	-2.350208

H	-6.164831	0.713524	-1.535894
H	-6.706551	-0.745274	-0.687442

ωB97XD energy = -1154.85315857 a.u.

(1*R*,5*S*,6*R*,7*R*,8*S*,10*R*)-**3**, Conf. D

C	3.944914	-2.442507	-0.242268
C	4.630005	-1.107070	-0.564073
C	3.889439	0.056823	0.049214
C	2.426663	0.075286	-0.333721
C	1.716242	-1.250263	0.132755
C	2.453861	-2.407532	-0.578108
C	1.590652	1.235824	0.160767
C	0.181506	1.195605	-0.432569
C	-0.557726	-0.017585	0.079980
C	0.233642	-1.269674	-0.314057
O	2.071616	2.545242	-0.197345
C	1.015408	3.407391	-0.218771
C	-0.254319	2.604498	-0.170894
O	1.808175	-3.620954	-0.224379
C	4.480306	0.947413	0.846030
C	-1.446581	3.140481	0.070492
O	1.152354	4.599188	-0.286039
H	2.383860	0.092002	-1.434983
C	1.789565	-1.435064	1.660697
O	-1.849646	-0.035090	-0.551930
C	-2.839042	-0.695259	0.082725
O	-2.691808	-1.249769	1.147706
C	-4.114135	-0.658513	-0.724787
C	-5.382285	-0.913946	0.095135
C	-5.614141	0.197021	1.122604
C	-6.585174	-1.053332	-0.840824
H	4.065284	-2.683773	0.819208
H	4.423751	-3.250162	-0.810357
H	4.651299	-0.971679	-1.655222
H	5.669648	-1.122925	-0.225093
H	2.342644	-2.241828	-1.663906
H	1.536335	1.211489	1.257489
H	0.291757	1.083320	-1.522865
H	-0.712209	0.039097	1.161543
H	0.183117	-1.353936	-1.408072
H	-0.250334	-2.156082	0.101929
H	2.276480	-4.354623	-0.631143
H	5.536547	0.858818	1.084920
H	3.953037	1.800488	1.258734
H	-2.345706	2.534137	0.094323
H	-1.533979	4.208493	0.245038
H	1.089283	-0.781268	2.186548
H	2.787218	-1.222337	2.051866
H	1.526534	-2.465076	1.912298
H	-4.171465	0.302960	-1.248182
H	-4.004599	-1.423346	-1.505165
H	-5.244405	-1.857770	0.635120
H	-6.527972	0.009372	1.694904
H	-4.784621	0.264932	1.831679
H	-5.728792	1.167809	0.623850
H	-7.496065	-1.270948	-0.274668

H	-6.440239	-1.859668	-1.567701
H	-6.754746	-0.124001	-1.398156

ωB97XD energy = -1154.85299390 a.u.

(1*R*,5*S*,6*R*,7*R*,8*S*,10*R*)-**3**, Conf. E

C	3.452586	-2.867085	-0.336533
C	4.324964	-1.663693	-0.720669
C	3.825775	-0.392257	-0.078192
C	2.365321	-0.133232	-0.371366
C	1.476547	-1.322235	0.154741
C	1.969634	-2.588166	-0.582340
C	1.764590	1.151199	0.157554
C	0.334575	1.343199	-0.350177
C	-0.563580	0.269635	0.217266
C	-0.012289	-1.097209	-0.204087
O	2.431765	2.361974	-0.247489
C	1.532683	3.386538	-0.215934
C	0.154084	2.804541	-0.077441
O	1.156188	-3.677119	-0.173715
C	4.601954	0.390222	0.671805
C	-0.914818	3.529379	0.237058
O	1.859050	4.539249	-0.307414
H	2.257044	-0.113010	-1.468026
C	1.610402	-1.511064	1.678231
O	-1.873455	0.450928	-0.349362
C	-2.925623	0.011650	0.370224
O	-2.817791	-0.510719	1.455967
C	-4.224684	0.303560	-0.341989
C	-5.397608	-0.567705	0.116909
C	-6.702334	-0.047224	-0.490464
C	-5.170659	-2.037744	-0.244496
H	3.598112	-3.119372	0.719087
H	3.755877	-3.745053	-0.921016
H	4.294750	-1.537773	-1.812796
H	5.368465	-1.848162	-0.450754
H	1.820886	-2.411087	-1.661871
H	1.775473	1.140485	1.255709
H	0.358960	1.214263	-1.443937
H	-0.649090	0.355607	1.304248
H	-0.140770	-1.176220	-1.291949
H	-0.611561	-1.889738	0.250090
H	1.471228	-4.479407	-0.598219
H	5.642553	0.133058	0.849222
H	4.244897	1.317882	1.105750
H	-1.896995	3.078677	0.328103
H	-0.812324	4.596884	0.406379
H	1.060911	-0.748155	2.235669
H	2.651152	-1.465390	2.007480
H	1.194598	-2.481530	1.958733
H	-4.443821	1.364268	-0.158870
H	-4.061849	0.208270	-1.421426
H	-5.462472	-0.488780	1.208307
H	-7.554572	-0.643074	-0.149542
H	-6.888400	0.995826	-0.213218
H	-6.673354	-0.104265	-1.585477
H	-4.264447	-2.429700	0.225494

H	-5.078213	-2.160046	-1.330904
H	-6.012212	-2.652827	0.089236

ωB97XD energy = -1154.85286744 a.u.

(1*R*,5*S*,6*R*,7*R*,8*S*,10*R*)-**3**, Conf. F

C	2.787066	-3.193587	-0.266518
C	3.799084	-2.153009	-0.766292
C	3.542887	-0.796433	-0.154813
C	2.120814	-0.328946	-0.368287
C	1.103844	-1.347829	0.269108
C	1.349395	-2.701842	-0.433868
C	1.754724	1.050589	0.135102
C	0.336977	1.439809	-0.288792
C	-0.669345	0.534760	0.381796
C	-0.354279	-0.914028	-0.010210
O	2.565214	2.129242	-0.368320
C	1.834617	3.279707	-0.325917
C	0.397319	2.920848	-0.072316
O	0.410914	-3.636668	0.076022
C	4.478080	-0.116786	0.508886
C	-0.526636	3.810585	0.275927
O	2.323553	4.364683	-0.491603
H	1.941473	-0.334737	-1.455795
C	1.310863	-1.495335	1.788721
O	-1.973031	0.887670	-0.110514
C	-3.031666	0.546850	0.654415
O	-2.922787	0.046060	1.751353
C	-4.335350	0.836080	-0.044088
C	-4.936896	-0.424617	-0.713358
C	-3.983257	-1.034946	-1.744217
C	-5.383834	-1.466698	0.314191
H	2.965985	-3.420608	0.789862
H	2.915337	-4.131249	-0.821947
H	3.708789	-2.065999	-1.858847
H	4.820024	-2.483407	-0.555750
H	1.156746	-2.546143	-1.509781
H	1.841404	1.080006	1.229605
H	0.265136	1.270095	-1.374892
H	-0.666110	0.671262	1.467186
H	-0.561529	-1.009260	-1.084750
H	-1.033157	-1.591232	0.513223
H	0.575223	-4.494590	-0.324047
H	5.475762	-0.528390	0.634423
H	4.298132	0.869681	0.922055
H	-1.556785	3.518749	0.448690
H	-0.255402	4.855577	0.390870
H	2.366732	-1.598649	2.049280
H	0.774345	-2.378940	2.141933
H	0.922187	-0.635421	2.339588
H	-5.034447	1.214378	0.707031
H	-4.173118	1.608258	-0.800863
H	-5.828353	-0.069690	-1.245877
H	-3.634993	-0.287863	-2.464428
H	-3.102080	-1.469884	-1.259387
H	-4.482757	-1.836754	-2.296864
H	-4.533383	-1.846549	0.888674
H	-5.862548	-2.313929	-0.187245
H	-6.099941	-1.043622	1.025734

ωB97XD energy = -1154.85238320 a.u.

(1*R*,5*S*,6*R*,7*R*,8*S*,10*R*)-**3**, Conf. G

C	4.415910	-1.706729	-0.496066
C	4.665547	-0.264808	-0.959149
C	3.731465	0.704926	-0.276545
C	2.276933	0.316694	-0.429655
C	2.022078	-1.113196	0.172490
C	2.939171	-2.083052	-0.606119
C	1.250701	1.250834	0.170850
C	-0.184766	0.823250	-0.151026
C	-0.495620	-0.542236	0.429349
C	0.557031	-1.539845	-0.064382
O	1.305221	2.606383	-0.315822
C	0.077017	3.174856	-0.148298
C	-0.895440	2.091638	0.224577
O	2.695484	-3.396981	-0.126188
C	4.169079	1.767850	0.398790
C	-2.042353	2.347783	0.846592
O	-0.117563	4.351529	-0.293790
H	2.069355	0.251768	-1.510071
C	2.341264	-1.171269	1.678500
O	-1.751554	-1.016461	-0.095974
C	-2.814267	-1.108843	0.722086
O	-2.804665	-0.754732	1.881368
C	-4.022675	-1.670227	0.016882
C	-5.071929	-0.578802	-0.278274
C	-4.553027	0.435494	-1.300890
C	-6.371909	-1.224151	-0.761917
H	4.739984	-1.834949	0.542206
H	5.011455	-2.398023	-1.105680
H	4.498340	-0.205836	-2.044475
H	5.707660	0.015233	-0.782112
H	2.642436	-2.024390	-1.667859
H	1.389345	1.301095	1.259211
H	-0.248359	0.725091	-1.247035
H	-0.569687	-0.505411	1.519157
H	0.391012	-1.676668	-1.141093
H	0.384985	-2.510952	0.406784
H	3.277667	-4.009160	-0.583777
H	5.233245	1.970756	0.481445
H	3.496497	2.480687	0.862915
H	-2.703534	1.560332	1.191321
H	-2.317753	3.379354	1.045156
H	3.287622	-0.679593	1.916055
H	2.400770	-2.214922	1.996369
H	1.566515	-0.687887	2.279323
H	-3.719724	-2.160904	-0.912861
H	-4.456646	-2.419395	0.686127
H	-5.274707	-0.057838	0.666048
H	-4.342201	-0.056835	-2.257588
H	-5.298308	1.216255	-1.481689
H	-3.632392	0.925143	-0.970262
H	-6.209452	-1.769353	-1.699383

H	-7.136499	-0.463435	-0.947695
H	-6.768279	-1.929083	-0.024038

ωB97XD energy = -1154.85219585 a.u.

(1*R*,5*S*,6*R*,7*R*,8*S*,10*R*)-**3**, Conf. H

C	2.887308	-3.076334	-0.323927
C	3.825115	-1.985887	-0.861129
C	3.524951	-0.641911	-0.241365
C	2.072224	-0.250141	-0.391928
C	1.140440	-1.318453	0.292969
C	1.420703	-2.657409	-0.425359
C	1.658420	1.110551	0.125087
C	0.202757	1.422759	-0.229802
C	-0.716710	0.471217	0.498248
C	-0.350491	-0.960653	0.088959
O	2.385620	2.228245	-0.418341
C	1.598262	3.338958	-0.342653
C	0.195130	2.906113	-0.021870
O	0.553873	-3.638443	0.122571
C	4.452028	0.089061	0.377680
C	-0.759164	3.746973	0.364248
O	2.021272	4.447475	-0.532216
H	1.844936	-0.270142	-1.470280
C	1.428962	-1.454825	1.800125
O	-2.063904	0.755128	0.085332
C	-3.054656	0.329891	0.897954
O	-2.852929	-0.172538	1.980269
C	-4.412822	0.528701	0.275233
C	-4.778343	-0.573822	-0.750697
C	-4.005857	-0.441816	-2.065556
C	-4.624391	-1.976288	-0.156306
H	3.121886	-3.298257	0.722645
H	3.037815	-4.004763	-0.889209
H	3.689479	-1.905416	-1.949475
H	4.868870	-2.263040	-0.689391
H	1.172926	-2.509537	-1.491057
H	1.795026	1.149035	1.214238
H	0.084601	1.241886	-1.309992
H	-0.654646	0.611316	1.581400
H	-0.607292	-1.069444	-0.973613
H	-0.965512	-1.670636	0.646553
H	0.743356	-4.486986	-0.286338
H	5.474538	-0.269264	0.459643
H	4.240491	1.066890	0.796222
H	-1.762829	3.398586	0.582747
H	-0.540914	4.805839	0.463631
H	1.027318	-0.613986	2.370690
H	2.500006	-1.507111	2.008430
H	0.952839	-2.362462	2.178192
H	-5.136213	0.515722	1.093656
H	-4.449161	1.507060	-0.214159
H	-5.841301	-0.414436	-0.971837
H	-4.126111	0.554383	-2.503313
H	-2.935637	-0.612198	-1.915322
H	-4.366567	-1.177483	-2.791556
H	-3.570653	-2.213896	0.028782

H	-5.016969	-2.728575	-0.847398
H	-5.154922	-2.072139	0.795720

ωB97XD energy = -1154.85202117 a.u.

(1*R*,5*S*,6*R*,7*R*,8*S*,10*R*)-**3**, Conf. I

C	4.075116	-2.177630	-0.204601
C	4.660081	-0.777468	-0.439498
C	3.791507	0.297676	0.168341
C	2.353342	0.206529	-0.289414
C	1.739099	-1.189634	0.099682
C	2.604317	-2.252950	-0.613666
C	1.397898	1.273635	0.198209
C	0.023045	1.136016	-0.460570
C	-0.628372	-0.153229	-0.016158
C	0.286297	-1.317093	-0.417293
O	1.782203	2.629146	-0.098629
C	0.659769	3.401153	-0.145870
C	-0.539051	2.495861	-0.178543
O	2.048386	-3.529074	-0.336004
C	4.262640	1.206218	1.022701
C	-1.781200	2.924059	0.022168
O	0.699419	4.601816	-0.173738
H	2.362762	0.258772	-1.390305
C	1.760094	-1.425649	1.622293
O	-1.889819	-0.271969	-0.699015
C	-2.803967	-1.100643	-0.152041
O	-2.618270	-1.694023	0.887045
C	-4.060143	-1.176342	-0.986657
C	-5.312821	-0.623720	-0.269719
C	-5.762503	-1.501586	0.900562
C	-5.108643	0.827959	0.170189
H	4.165217	-2.453840	0.851314
H	4.647758	-2.916625	-0.779197
H	4.730988	-0.598901	-1.522265
H	5.676575	-0.719339	-0.040390
H	2.529951	-2.054783	-1.697242
H	1.296638	1.207801	1.289968
H	0.188610	1.068981	-1.547562
H	-0.825655	-0.146961	1.059880
H	0.292687	-1.360292	-1.514830
H	-0.136728	-2.257209	-0.057318
H	2.597194	-4.204321	-0.743367
H	5.308812	1.199823	1.316081
H	3.642533	1.996557	1.431562
H	-2.624189	2.243252	-0.012047
H	-1.964488	3.975480	0.221666
H	2.718254	-1.147999	2.067549
H	1.571441	-2.482401	1.825044
H	0.985428	-0.851748	2.137022
H	-3.896008	-0.634883	-1.921541
H	-4.219051	-2.233222	-1.226318
H	-6.107496	-0.637219	-1.026361
H	-5.943177	-2.531431	0.576177
H	-5.003953	-1.530269	1.687196
H	-6.691957	-1.112873	1.329182
H	-4.346790	0.893005	0.956716

H	-6.034293	1.244056	0.579067
H	-4.795428	1.460437	-0.667637

ωB97XD energy = -1154.85171890 a.u.

(1*R*,5*S*,6*R*,7*R*,8*S*,10*R*)-**3**, Conf. J

C	4.262647	-1.750173	-0.403554
C	4.607153	-0.298130	-0.762993
C	3.672701	0.675548	-0.086673
C	2.218302	0.365909	-0.362872
C	1.857634	-1.081170	0.139391
C	2.781472	-2.048574	-0.634678
C	1.187201	1.313810	0.208322
C	-0.226359	0.965211	-0.265922
C	-0.638145	-0.390342	0.260101
C	0.390932	-1.428083	-0.205294
O	1.338941	2.693645	-0.177564
C	0.123524	3.304101	-0.092114
C	-0.927194	2.243471	0.081532
O	2.446710	-3.373860	-0.252881
C	4.107677	1.677204	0.677956
C	-2.156640	2.508695	0.510507
O	-0.011612	4.495729	-0.164966
H	2.090482	0.367956	-1.457585
C	2.076049	-1.236718	1.656671
O	-1.914168	-0.721638	-0.318580
C	-2.699181	-1.581934	0.364424
O	-2.420884	-1.999013	1.465880
C	-3.943541	-1.951756	-0.408292
C	-4.994885	-0.824359	-0.532115
C	-5.330377	-0.221718	0.834353
C	-4.604972	0.247524	-1.553445
H	4.507334	-1.950827	0.644928
H	4.868217	-2.433893	-1.011860
H	4.514642	-0.170904	-1.851439
H	5.646762	-0.078811	-0.504277
H	2.561827	-1.914289	-1.708338
H	1.230759	1.287460	1.305503
H	-0.189336	0.897332	-1.364975
H	-0.746728	-0.380749	1.348068
H	0.284795	-1.517658	-1.294807
H	0.146351	-2.402269	0.224595
H	3.038098	-3.986298	-0.698125
H	5.171246	1.826715	0.841818
H	3.435346	2.393252	1.137624
H	-2.893113	1.725702	0.649521
H	-2.441510	3.533113	0.730035
H	1.283029	-0.755491	2.234761
H	3.023642	-0.801027	1.981863
H	2.069307	-2.298258	1.914998
H	-3.644413	-2.281704	-1.409022
H	-4.381010	-2.802397	0.119128
H	-5.900380	-1.317520	-0.907808
H	-4.461700	0.279945	1.275972
H	-6.130959	0.518876	0.745582
H	-5.651764	-0.991360	1.542800
H	-5.401105	0.993187	-1.645787

H	-4.440465	-0.194907	-2.541409
H	-3.685239	0.764827	-1.268698

ωB97XD energy = -1154.85129595 a.u.

(1*R*,5*S*,6*R*,7*R*,8*S*,10*R*)-**3**, Conf. K

C	4.293216	-1.782900	-0.540136
C	4.635853	-0.320967	-0.860260
C	3.729389	0.637055	-0.126686
C	2.266823	0.346037	-0.375490
C	1.905752	-1.107517	0.108671
C	2.802524	-2.073844	-0.713190
C	1.256813	1.299361	0.225294
C	-0.168396	0.959214	-0.214262
C	-0.569964	-0.395413	0.320952
C	0.426947	-1.435420	-0.200354
O	1.404804	2.680619	-0.155582
C	0.193417	3.295242	-0.033872
C	-0.858400	2.237214	0.152470
O	2.506278	-3.436455	-0.458925
C	4.189621	1.610947	0.658646
C	-2.084606	2.501162	0.592258
O	0.061800	4.487888	-0.091665
H	2.116970	0.363161	-1.467316
C	2.161221	-1.288080	1.616167
O	-1.879764	-0.698467	-0.189114
C	-2.678211	-1.477204	0.569411
O	-2.342341	-1.918849	1.645213
C	-4.026973	-1.699191	-0.068820
C	-4.892448	-0.420582	-0.175087
C	-4.515228	0.442710	-1.382320
C	-6.372770	-0.808103	-0.235493
H	4.592418	-2.009065	0.492118
H	4.857549	-2.457480	-1.191466
H	4.511420	-0.160268	-1.940745
H	5.684545	-0.116751	-0.626861
H	2.542051	-1.931487	-1.770526
H	1.326116	1.269039	1.320972
H	-0.160184	0.893354	-1.314115
H	-0.622641	-0.393255	1.413204
H	0.288251	-1.500936	-1.287682
H	0.184050	-2.417740	0.212149
H	2.949932	-3.710461	0.349391
H	5.257640	1.750349	0.800844
H	3.533112	2.315274	1.158116
H	-2.821210	1.717270	0.730233
H	-2.366484	3.524692	0.819775
H	3.168413	-0.980010	1.905882
H	2.016181	-2.336281	1.896271
H	1.462082	-0.711688	2.226772
H	-3.884392	-2.126216	-1.068738
H	-4.531307	-2.443707	0.550791
H	-4.740277	0.165796	0.742020
H	-3.454908	0.703494	-1.385588
H	-4.730999	-0.095200	-2.313348
H	-5.097537	1.369726	-1.389791
H	-6.681326	-1.354070	0.661432

H	-6.569677	-1.445642	-1.105682
H	-7.003475	0.081898	-0.324930

ωB97XD energy = -1154.85120326 a.u.

(1*R*,5*S*,6*R*,7*R*,8*S*,10*R*)-**3**, Conf. L

C	3.632449	-2.715404	-0.201621
C	4.465170	-1.455006	-0.475030
C	3.827748	-0.230145	0.135650
C	2.391385	-0.049647	-0.300645
C	1.527618	-1.300344	0.112203
C	2.167988	-2.518042	-0.591555
C	1.662796	1.184182	0.186654
C	0.280858	1.303254	-0.459044
C	-0.599023	0.164844	0.001822
C	0.071734	-1.155687	-0.392687
O	2.290972	2.442259	-0.125113
C	1.331772	3.410389	-0.165322
C	-0.015524	2.744568	-0.180943
O	1.385138	-3.661708	-0.284585
C	4.477026	0.574608	0.977029
C	-1.154104	3.396705	0.031656
O	1.594131	4.582411	-0.200485
H	2.393697	-0.010131	-1.402135
C	1.518485	-1.516122	1.637837
O	-1.865074	0.288713	-0.668127
C	-2.939839	-0.260373	-0.062365
O	-2.876200	-0.826752	1.004441
C	-4.175091	-0.048039	-0.908391
C	-5.508449	-0.507735	-0.302581
C	-5.571582	-2.025138	-0.102489
C	-5.851245	0.249678	0.984350
H	3.686448	-2.982173	0.859198
H	4.045393	-3.561019	-0.766013
H	4.541184	-1.306452	-1.561934
H	5.483785	-1.585444	-0.099120
H	2.117050	-2.326610	-1.677649
H	1.562998	1.146519	1.279825
H	0.420581	1.200175	-1.546826
H	-0.779980	0.218729	1.079231
H	0.058467	-1.211691	-1.489605
H	-0.519052	-1.994726	-0.018223
H	1.790089	-4.434947	-0.686099
H	5.508156	0.370854	1.252209
H	4.025864	1.468580	1.393179
H	-2.110745	2.886209	0.010193
H	-1.136355	4.464405	0.228099
H	2.514921	-1.411094	2.073279
H	1.142002	-2.517817	1.857371
H	0.865275	-0.804169	2.148460
H	-4.215424	1.020557	-1.151872
H	-3.992509	-0.558754	-1.862188
H	-6.265975	-0.242538	-1.052238
H	-4.850974	-2.350581	0.651998
H	-6.571597	-2.323469	0.228298
H	-5.356710	-2.556112	-1.036524
H	-6.862170	-0.003475	1.319041

H	-5.813767	1.333744	0.828004
H	-5.153418	-0.007245	1.785819

ωB97XD energy = -1154.85097809 a.u.

(1*R*,5*S*,6*R*,7*R*,8*S*,10*R*)-**3**, Conf. M

C	3.436864	-2.871608	-0.228047
C	4.337400	-1.667511	-0.537735
C	3.796343	-0.400801	0.079935
C	2.361268	-0.133992	-0.314034
C	1.434014	-1.324559	0.136145
C	1.975229	-2.586296	-0.573661
C	1.727628	1.147641	0.182630
C	0.337319	1.347733	-0.423225
C	-0.599935	0.271989	0.072974
C	-0.024764	-1.091690	-0.327207
O	2.424618	2.359760	-0.163023
C	1.528099	3.386629	-0.186776
C	0.141489	2.807893	-0.154242
O	1.133570	-3.676375	-0.230034
C	4.518574	0.369603	0.893579
C	-0.946511	3.534466	0.080915
O	1.863017	4.539116	-0.243821
H	2.331757	-0.103196	-1.415486
C	1.456085	-1.527354	1.663435
O	-1.871034	0.472672	-0.568934
C	-2.963118	-0.019346	0.053822
O	-2.908821	-0.616084	1.104558
C	-4.207867	0.320299	-0.734916
C	-5.482945	-0.450542	-0.362572
C	-5.313071	-1.961769	-0.547580
C	-5.999480	-0.106182	1.037870
H	3.507725	-3.134865	0.832592
H	3.778016	-3.744765	-0.798535
H	4.388566	-1.531666	-1.627823
H	5.357507	-1.857691	-0.192694
H	1.901616	-2.401661	-1.659640
H	1.660081	1.127116	1.278663
H	0.438536	1.225879	-1.513286
H	-0.750559	0.345202	1.154006
H	-0.074426	-1.154987	-1.422706
H	-0.657072	-1.887688	0.071994
H	1.475428	-4.476240	-0.638034
H	5.543297	0.106493	1.140980
H	4.133928	1.292947	1.312807
H	-1.933586	3.085406	0.091879
H	-0.854570	4.600878	0.262843
H	2.470243	-1.491713	2.068018
H	1.015221	-2.497547	1.903497
H	0.872209	-0.765751	2.186488
H	-4.369655	1.399069	-0.607548
H	-3.970550	0.177924	-1.794551
H	-6.238242	-0.112413	-1.084673
H	-4.955096	-2.200623	-1.555287
H	-4.600115	-2.366978	0.176320
H	-6.269342	-2.473978	-0.402504
H	-5.300510	-0.444733	1.806395

H	-6.966084	-0.588946	1.214558
H	-6.140865	0.974180	1.153904

ωB97XD energy = -1154.85090765 a.u.

(1*R*,5*S*,6*R*,7*R*,8*S*,10*R*)-**3**, Conf. N

C	4.228312	-1.814863	-0.607718
C	4.409294	-0.444981	-1.276063
C	3.626000	0.627121	-0.557811
C	2.162689	0.274066	-0.416001
C	1.993237	-1.070474	0.387528
C	2.751013	-2.154380	-0.412028
C	1.255644	1.302084	0.226309
C	-0.209812	0.870686	0.142992
C	-0.418764	-0.374830	0.980865
C	0.501085	-1.484220	0.457398
O	1.254706	2.604190	-0.390242
C	0.059712	3.207516	-0.132668
C	-0.883393	2.176694	0.423219
O	2.584819	-3.392185	0.264921
C	4.189192	1.744421	-0.097562
C	-2.025454	2.484640	1.030829
O	-0.139645	4.372074	-0.351166
H	1.775437	0.091153	-1.431038
C	2.575413	-0.963965	1.809918
O	-1.797399	-0.797978	1.061410
C	-2.496269	-1.087967	-0.050142
O	-2.047979	-1.028696	-1.174230
C	-3.909370	-1.506685	0.280260
C	-4.941718	-0.913068	-0.688091
C	-6.316095	-1.536815	-0.439467
C	-4.996001	0.612081	-0.566690
H	4.730589	-1.832918	0.365333
H	4.697229	-2.591976	-1.224399
H	4.048538	-0.506339	-2.313040
H	5.469873	-0.182342	-1.322658
H	2.270920	-2.217634	-1.403401
H	1.550370	1.454919	1.273441
H	-0.410911	0.615784	-0.905683
H	-0.196419	-0.149328	2.027770
H	0.159490	-1.762100	-0.544560
H	0.407664	-2.368131	1.093770
H	3.045426	-4.078904	-0.224156
H	5.252973	1.921914	-0.229577
H	3.617448	2.526435	0.390183
H	-2.679496	1.718245	1.433363
H	-2.319161	3.525003	1.132555
H	2.668512	-1.963822	2.240555
H	1.935699	-0.376342	2.473492
H	3.559423	-0.489408	1.814116
H	-3.929663	-2.602227	0.218673
H	-4.139364	-1.237591	1.316049
H	-4.619378	-1.165410	-1.704803
H	-6.667807	-1.319633	0.576398
H	-7.056091	-1.138329	-1.140437
H	-6.289382	-2.624745	-0.561586
H	-5.727534	1.031538	-1.264104

H	-4.027182	1.070540	-0.789537
H	-5.291522	0.910035	0.447269

ωB97XD energy = -1154.85081723 a.u.

(1*R*,5*S*,6*R*,7*R*,8*S*,10*R*)-**3**, Conf. O

C	4.348826	-1.630930	-0.443783
C	4.525189	-0.219238	-1.020027
C	3.621329	0.775107	-0.332194
C	2.170688	0.347555	-0.351260
C	1.995471	-1.042875	0.367469
C	2.878187	-2.046603	-0.408422
C	1.157713	1.299129	0.248149
C	-0.273606	0.808166	0.016730
C	-0.493377	-0.491341	0.764940
C	0.526289	-1.525423	0.272575
O	1.145590	2.627664	-0.310093
C	-0.093289	3.165030	-0.123984
C	-1.023836	2.068047	0.313702
O	2.712335	-3.325109	0.188501
C	4.080045	1.891093	0.234973
C	-2.209387	2.296067	0.870480
O	-0.331288	4.328162	-0.308501
H	1.892234	0.198792	-1.406691
C	2.435854	-0.988968	1.842696
O	-1.846394	-0.988805	0.694321
C	-2.421078	-1.278945	-0.489086
O	-1.881595	-1.135901	-1.564124
C	-3.829808	-1.779399	-0.290558
C	-4.805160	-0.668189	0.147344
C	-4.884046	0.452374	-0.892222
C	-6.186742	-1.270529	0.410833
H	4.759266	-1.682039	0.570259
H	4.911411	-2.350259	-1.052182
H	4.274785	-0.238529	-2.090613
H	5.570202	0.093726	-0.942708
H	2.495880	-2.078261	-1.442926
H	1.350410	1.416619	1.323386
H	-0.376470	0.607097	-1.057666
H	-0.375936	-0.314869	1.838100
H	0.296263	-1.757961	-0.771440
H	0.416935	-2.448947	0.846958
H	3.245690	-3.964087	-0.291324
H	5.141444	2.123069	0.218874
H	3.424932	2.619164	0.700675
H	-2.847261	1.481800	1.197406
H	-2.556308	3.315758	1.007262
H	3.394933	-0.480570	1.965624
H	2.529283	-2.006505	2.229864
H	1.713198	-0.461043	2.470227
H	-4.158320	-2.210282	-1.240563
H	-3.815184	-2.568993	0.467633
H	-4.430121	-0.250850	1.090912
H	-5.572531	1.236323	-0.561453
H	-3.910696	0.917856	-1.075521
H	-5.249905	0.062788	-1.849140
H	-6.144740	-2.048770	1.179629

H	-6.595169	-1.718489	-0.502681
H	-6.888983	-0.501407	0.747130

ωB97XD energy = -1154.85070486 a.u.

3-deoxy-1,2;5,6-di-*O*-isopropylidene-3-*C*-methylene-*α*-D-*ribo*-hexofuranose (**4**), Conf. A

C	-1.191829	-0.755905	-0.862184
O	-0.709663	0.353744	-1.609734
C	0.165646	1.143387	-0.810906
C	-0.340227	0.953241	0.598172
C	-1.008980	-0.403139	0.632449
O	-2.315784	-0.416657	1.164534
C	-3.239972	-0.334316	0.082868
O	-2.560102	-0.936197	-1.018523
C	-4.448296	-1.182158	0.424524
C	-3.609266	1.114144	-0.228593
C	-0.308187	1.831351	1.594199
C	1.613303	0.693846	-1.024682
C	2.678702	1.525654	-0.310513
O	3.689812	0.575688	-0.043723
C	3.046045	-0.659535	0.221788
O	1.803804	-0.608211	-0.488256
C	2.756051	-0.820065	1.710285
C	3.908263	-1.774053	-0.339347
H	0.069006	2.182101	-1.147539
H	1.795768	0.665488	-2.106772
H	-0.686174	-1.661239	-1.201402
H	-0.406629	-1.135043	1.174838
H	-4.131418	-2.207271	0.625041
H	-4.943947	-0.778006	1.310602
H	-5.155635	-1.179678	-0.408286
H	-4.308726	1.140427	-1.068024
H	-4.080703	1.569304	0.646456
H	-2.726905	1.696193	-0.497425
H	-0.755242	1.598901	2.555873
H	0.136592	2.815452	1.474458
H	2.293457	1.965607	0.619517
H	3.102413	2.313161	-0.935783
H	2.223385	-1.758724	1.886509
H	2.132568	0.003836	2.067402
H	3.692008	-0.832987	2.274815
H	4.877830	-1.788134	0.164995
H	3.417022	-2.738687	-0.190040
H	4.063284	-1.612348	-1.407987

ωB97XD energy = -883.305951357 a.u.

3-deoxy-1,2;5,6-di-*O*-isopropylidene-3-*C*-methylene-*α*-D-*ribo*-hexofuranose (**4**), Conf. B

C	1.955967	0.370095	-1.302827
O	0.673528	-0.227599	-1.380356
C	-0.198813	0.235074	-0.343120
C	0.456456	1.495451	0.170619
C	1.922277	1.316808	-0.097068
O	2.538279	0.554701	0.931942
C	3.190215	-0.592574	0.387251
O	2.974050	-0.532658	-1.020838
C	4.685315	-0.502925	0.642320
C	2.565350	-1.850367	0.973293
C	-0.113124	2.578035	0.690213
C	-1.591163	0.397377	-0.952831
C	-2.171366	-0.929427	-1.437682
O	-2.781202	-1.437130	-0.263804
C	-3.301442	-0.330115	0.450970
O	-2.526188	0.802698	0.033990
C	-3.096621	-0.577198	1.934893
C	-4.759428	-0.074771	0.087991
H	-0.251347	-0.521493	0.454072
H	-1.554354	1.158048	-1.738657
H	2.176999	0.824098	-2.270230
H	2.459338	2.257922	-0.252089
H	5.196804	-1.350971	0.179727
H	5.077438	0.423861	0.217301
H	4.880100	-0.513710	1.717632
H	3.056562	-2.736448	0.563369
H	2.678905	-1.849619	2.060470
H	1.503277	-1.896234	0.724376
H	0.493735	3.428962	0.986438
H	-1.185159	2.635344	0.843124
H	-1.416432	-1.640099	-1.774426
H	-2.911314	-0.770610	-2.233756
H	-2.033893	-0.720699	2.142033
H	-3.643513	-1.471161	2.245544
H	-3.459153	0.278595	2.509818
H	-4.864673	0.056230	-0.992021
H	-5.378100	-0.918578	0.404560
H	-5.114712	0.833676	0.581109

ωB97XD energy = -883.305403735 a.u.

3-deoxy-1,2;5,6-di-*O*-isopropylidene-3-*C*-methylene-*α*-D-*ribo*-hexofuranose (**4**), Conf. C

C	-1.890925	-1.415763	0.202576
O	-0.540476	-1.620572	-0.085293
C	0.211861	-0.433173	0.176805
C	-0.580697	0.297797	1.238165
C	-1.967565	-0.324758	1.283853
O	-3.029254	0.510771	0.880796
C	-3.198569	0.382320	-0.527173
O	-2.616280	-0.874733	-0.874795
C	-4.687060	0.335358	-0.821955
C	-2.497048	1.512165	-1.273257
C	-0.178169	1.305907	2.005585
C	1.621881	-0.873174	0.576644
C	2.343939	-1.601148	-0.556077
O	2.935145	-0.532184	-1.272770
C	3.318649	0.444434	-0.322345
O	2.450395	0.255004	0.803600
C	3.086303	1.817866	-0.926032
C	4.757259	0.234702	0.136263
H	0.283494	0.162675	-0.745048
H	1.567764	-1.463570	1.496347
H	-2.307136	-2.391952	0.457848

H	-2.207850	-0.707303	2.278177
H	-4.852015	0.207461	-1.894648
H	-5.139840	-0.503022	-0.288748
H	-5.161499	1.264939	-0.497487
H	-2.962488	2.467699	-1.018440
H	-1.440353	1.563459	-1.005612
H	-2.581415	1.350121	-2.350893
H	-0.854548	1.743911	2.734268
H	0.820408	1.719579	1.918210
H	1.673650	-2.141694	-1.224548
H	3.105466	-2.288424	-0.163528
H	3.696321	1.941223	-1.824382
H	3.355325	2.593965	-0.205063
H	2.033050	1.929567	-1.193568
H	5.005751	0.948239	0.926038
H	4.886920	-0.775512	0.532963
H	5.443595	0.377171	-0.702502

ωB97XD energy = -883.305394979 a.u.

3-deoxy-1,2;5,6-di-*O*-isopropylidene-3-*C*-methylene-*α*-D-*ribo*-hexofuranose (**4**), Conf. D

C	-1.218280	-0.545626	-1.035245
O	-0.709104	0.690983	-1.512544
C	0.183493	1.267578	-0.565884
C	-0.275134	0.736294	0.770759
C	-0.998186	-0.563890	0.494290
O	-2.297297	-0.660982	1.040111
C	-3.233549	-0.292966	0.031927
O	-2.595244	-0.640675	-1.196724
C	-4.470090	-1.154393	0.187729
C	-3.549219	1.200280	0.081032
C	-0.146633	1.314902	1.958519
C	1.626942	0.884912	-0.916399
C	2.705244	1.557146	-0.044690
O	3.162821	0.526216	0.802953
C	2.971111	-0.698728	0.121278
O	1.810359	-0.504242	-0.683155
C	2.686172	-1.776588	1.147377
C	4.166414	-1.024277	-0.771643
H	0.079798	2.356359	-0.640960
H	1.776045	1.099966	-1.981193
H	-0.752471	-1.361639	-1.590289
H	-0.420210	-1.427673	0.829592
H	-5.188915	-0.921976	-0.601720
H	-4.192786	-2.208171	0.123111
H	-4.936396	-0.963227	1.157421
H	-4.251164	1.453187	-0.717650
H	-3.997931	1.449005	1.046342
H	-2.645991	1.796775	-0.052998
H	-0.547377	0.845651	2.851640
H	0.344566	2.275872	2.079141
H	2.314272	2.361825	0.583312
H	3.521052	1.957611	-0.660289
H	1.851619	-1.462341	1.777951
H	3.565183	-1.937482	1.776205
H	2.434991	-2.714347	0.645569
H	4.325048	-0.232588	-1.509673
H	5.071274	-1.125546	-0.166939
H	3.987388	-1.959530	-1.308001

ωB97XD energy = -883.305310724 a.u.

3-deoxy-1,2;5,6-di-*O*-isopropylidene-3-*C*-methylene-*α*-D-*ribo*-hexofuranose (**4**), Conf. E

C	1.923510	-1.072153	-0.894897
O	0.576317	-1.366057	-0.668159
C	-0.114740	-0.203483	-0.208423
C	0.665300	0.959514	-0.780945
C	2.012821	0.425365	-1.237831
O	3.134412	0.893638	-0.523240
C	3.360246	0.031173	0.586585
O	2.724107	-1.204213	0.253583
C	4.855473	-0.200259	0.709314
C	2.757204	0.605158	1.863999
C	0.266787	2.223480	-0.878879
C	-1.559670	-0.331916	-0.665328
C	-2.244201	-1.609759	-0.184119
O	-3.593479	-1.203461	-0.064066
C	-3.592332	0.147252	0.363067
O	-2.336719	0.691577	-0.064760
C	-3.668207	0.244348	1.882442
C	-4.728783	0.869035	-0.337474
H	-0.106560	-0.178214	0.892032
H	-1.608049	-0.246897	-1.759381
H	2.269963	-1.773778	-1.655695
H	2.190181	0.623747	-2.296937
H	5.061873	-0.885973	1.534873
H	5.237444	-0.632397	-0.217821
H	5.364674	0.748050	0.898851
H	2.873805	-0.111574	2.680992
H	3.266342	1.535644	2.127402
H	1.695739	0.821174	1.731527
H	0.920702	2.976266	-1.309731
H	-0.708527	2.536382	-0.520480
H	-1.834439	-1.941647	0.780828
H	-2.188660	-2.430500	-0.898365
H	-2.852354	-0.322414	2.338962
H	-4.620510	-0.157221	2.238485
H	-3.579730	1.288048	2.195108
H	-5.685793	0.420071	-0.059623
H	-4.735282	1.924599	-0.054236
H	-4.598375	0.788229	-1.418714

ωB97XD energy = -883.304695831 a.u.

3-deoxy-1,2;5,6-di-*O*-isopropylidene-3-*C*-methylene-*α*-D-*ribo*-hexofuranose (**4**), Conf. F

C	1.970440	0.221776	-1.306214
O	0.695210	-0.394171	-1.229231
C	-0.113115	0.174404	-0.194128
C	0.553368	1.489811	0.130189
C	2.000666	1.297954	-0.213538
O	2.696170	0.661357	0.849280
C	3.330120	-0.533234	0.392805

O	3.020535	-0.633349	-0.995402
C	4.836907	-0.401524	0.535671
C	2.764862	-1.722853	1.155382
C	-0.014725	2.612156	0.557300
C	-1.538637	0.253804	-0.719160
C	-2.090633	-1.094689	-1.188600
O	-3.463068	-0.998438	-0.861685
C	-3.550741	-0.255279	0.338866
O	-2.418230	0.623300	0.331501
C	-3.463535	-1.165197	1.560974
C	-4.825216	0.566055	0.305935
H	-0.106963	-0.485470	0.687785
H	-1.589474	1.001615	-1.521472
H	2.118818	0.565734	-2.331069
H	2.511190	2.220008	-0.508769
H	5.331879	-1.287893	0.130787
H	5.182806	0.479760	-0.009292
H	5.101882	-0.295309	1.590535
H	3.255356	-2.643234	0.828542
H	2.931483	-1.589168	2.227238
H	1.693180	-1.815260	0.968145
H	0.579469	3.506198	0.722111
H	-1.078986	2.661503	0.764200
H	-1.608165	-1.929833	-0.660812
H	-1.995893	-1.252936	-2.262166
H	-4.316543	-1.848513	1.582422
H	-3.461659	-0.564636	2.474251
H	-2.541822	-1.752785	1.536949
H	-4.822667	1.208167	-0.577285
H	-5.695545	-0.094058	0.266824
H	-4.891839	1.187410	1.202490

ωB97XD energy = -883.304602007 a.u.

3-deoxy-1,2;5,6-di-*O*-isopropylidene-3-*C*-methylene-*α*-D-*ribo*-hexofuranose (**4**), Conf. G

C	1.225170	-0.781660	-0.783002
O	0.102270	-0.773823	0.060947
C	-0.166509	0.503810	0.630146
C	0.816638	1.461557	-0.005210
C	1.704222	0.675866	-0.948371
O	3.071279	0.650413	-0.600293
C	3.318500	-0.521123	0.171920
O	2.330515	-1.455724	-0.254988
C	4.685678	-1.057599	-0.203118
C	3.193274	-0.241981	1.666932
C	0.914129	2.768561	0.217676
C	-1.620961	0.907930	0.377493
C	-2.058357	0.851061	-1.093878
O	-3.383707	0.371010	-1.023032
C	-3.455634	-0.491016	0.101678
O	-2.515230	0.036635	1.039571
C	-3.066543	-1.918265	-0.268902
C	-4.848801	-0.388336	0.691008
H	-0.024112	0.438539	1.716318
H	-1.755124	1.918886	0.786057
H	0.935397	-1.299991	-1.699929
H	1.629621	1.044450	-1.974583
H	4.725440	-1.238431	-1.278826
H	5.456864	-0.332813	0.068941
H	4.876916	-1.994256	0.326061
H	2.207401	0.152931	1.916598
H	3.345089	-1.168633	2.225769
H	3.945379	0.490133	1.971335
H	1.662920	3.365456	-0.293990
H	0.273964	3.280149	0.930905
H	-1.419242	0.169884	-1.672122
H	-2.071631	1.829911	-1.574871
H	-2.050844	-1.944405	-0.669473
H	-3.765833	-2.314420	-1.010331
H	-3.091682	-2.550631	0.622362
H	-5.587142	-0.756119	-0.026077
H	-4.911795	-0.986268	1.603431
H	-5.067439	0.654720	0.928544

ωB97XD energy = -883.303517017 a.u.

3-deoxy-1,2;5,6-di-*O*-isopropylidene-3-*C*-methylene-*α*-D-*ribo*-hexofuranose (**4**), Conf. H

C	1.359297	-0.215722	-1.096204
O	0.202381	-0.507262	-0.334066
C	-0.122185	0.535924	0.586486
C	0.753994	1.702633	0.183608
C	1.915622	1.106018	-0.555137
O	2.930446	0.685622	0.344073
C	3.211866	-0.706007	0.168387
O	2.385313	-1.132643	-0.911847
C	4.661954	-0.884899	-0.244395
C	2.859748	-1.457918	1.444082
C	0.551017	3.001015	0.384268
C	-1.617235	0.834421	0.551483
C	-2.189003	1.106342	-0.848342
O	-3.449241	0.471117	-0.817384
C	-3.330095	-0.651201	0.041461
O	-2.365123	-0.270107	1.024321
C	-2.837541	-1.879913	-0.716381
C	-4.666485	-0.872595	0.722478
H	0.129546	0.199822	1.601861
H	-1.803498	1.681900	1.224694
H	1.093595	-0.231689	-2.155823
H	2.332263	1.765050	-1.323487
H	4.868893	-1.940996	-0.435212
H	4.859980	-0.313546	-1.154052
H	5.322459	-0.531914	0.551369
H	1.795671	-1.350521	1.661961
H	3.081856	-2.520940	1.321272
H	3.441937	-1.064310	2.281125
H	1.264816	3.734858	0.022547
H	-0.312317	3.378753	0.923434
H	-1.542824	0.679468	-1.626293
H	-2.344327	2.167220	-1.048897
H	-1.863633	-1.684975	-1.170048
H	-3.558366	-2.153188	-1.491737
H	-2.722991	-2.717042	-0.022776

H	-5.425035	-1.136779	-0.018597
H	-4.583915	-1.684436	1.449243
H	-4.970024	0.040856	1.237870

ωB97XD energy = -883.303403171 a.u.

3-deoxy-1,2;5,6-di-*O*-isopropylidene-3-*C*-methylene-*α*-D-*ribo*-hexofuranose (**4**), Conf. I

C	0.926439	-0.674188	-0.597053
O	0.080223	-0.549231	0.529179
C	-0.253949	0.804899	0.798479
C	0.797230	1.633244	0.097561
C	1.516408	0.727764	-0.878945
O	2.903157	0.583340	-0.653014
C	3.127489	-0.642297	0.040956
O	2.032462	-1.473959	-0.336554
C	4.404695	-1.263815	-0.486109
C	3.160175	-0.421693	1.550923
C	1.059508	2.920477	0.299642
C	-1.679683	1.153489	0.333519
C	-1.975786	0.901565	-1.148517
O	-2.466820	-0.419860	-1.159167
C	-3.165885	-0.622002	0.061280
O	-2.632984	0.338460	0.982355
C	-2.878740	-2.028073	0.550811
C	-4.653137	-0.340208	-0.119485
H	-0.224995	0.936129	1.885853
H	-1.865740	2.199613	0.604959
H	0.364812	-1.135755	-1.413325
H	1.381450	1.054030	-1.914363
H	4.574926	-2.230393	-0.005709
H	4.322511	-1.407445	-1.564977
H	5.252015	-0.607937	-0.271877
H	2.229141	0.023082	1.905183
H	3.296723	-1.379679	2.058831
H	3.988961	0.243361	1.807117
H	1.841121	3.427425	-0.257830
H	0.521996	3.500776	1.044751
H	-1.103104	0.952687	-1.803650
H	-2.727703	1.612239	-1.519142
H	-3.236506	-2.757406	-0.180956
H	-3.389757	-2.201391	1.501458
H	-1.802945	-2.144008	0.690593
H	-4.804700	0.666762	-0.518095
H	-5.089168	-1.064471	-0.812558
H	-5.165921	-0.412582	0.842981

ωB97XD energy = -883.303265994 a.u.

Methyl 5-deoxy-2,3-*O*-isopropylidene-*β*-D-*erythro*-pent-4-enofuranoside (**5**), Conf. A

C	-1.339882	-0.732260	0.333820
O	-1.286810	0.616426	0.782478
C	-0.629123	1.387533	-0.144541
C	0.209325	0.498829	-1.049048
C	-0.118826	-0.923911	-0.565480
C	-0.718289	2.712405	-0.166322
O	1.604449	0.615407	-0.871508
C	2.014999	-0.294561	0.140223
O	1.022938	-1.325894	0.154209
C	3.343604	-0.895908	-0.279120
C	2.078985	0.379780	1.506416
O	-2.464026	-0.966116	-0.449025
C	-3.693237	-0.791520	0.234291
H	-1.330448	-1.358524	1.233537
H	-0.006819	0.680290	-2.102320
H	-0.335808	-1.640790	-1.359767
H	-0.135872	3.274910	-0.885644
H	-1.340042	3.248804	0.540628
H	3.236722	-1.374397	-1.254649
H	4.103902	-0.113561	-0.343258
H	3.663563	-1.640637	0.453637
H	1.108307	0.796494	1.781598
H	2.372524	-0.353061	2.262657
H	2.816681	1.185958	1.486722
H	-3.834463	0.251914	0.532835
H	-3.736811	-1.433122	1.125072
H	-4.479184	-1.085175	-0.461707

ωB97XD energy = -652.084348640 a.u.

Methyl 5-deoxy-2,3-*O*-isopropylidene-*β*-D-*erythro*-pent-4-enofuranoside (**5**), Conf. B

C	-1.316799	-0.759426	0.343747
O	-1.460440	0.560002	0.849346
C	-0.873386	1.442419	-0.023916
C	0.240800	0.736430	-0.758109
C	-0.026994	-0.759114	-0.478308
C	-1.247806	2.711573	-0.132444
O	1.491279	0.990578	-0.137062
C	2.127728	-0.255530	0.105753
O	1.063001	-1.174902	0.311960
C	2.965384	-0.670623	-1.102260
C	2.931658	-0.150752	1.383935
O	-2.356777	-1.092410	-0.517456
C	-3.631185	-1.110000	0.101825
H	-1.278438	-1.425572	1.213279
H	0.273793	0.995467	-1.820274
H	-0.140519	-1.373051	-1.376009
H	-0.711298	3.373028	-0.801442
H	-2.060893	3.111490	0.461501
H	2.344391	-0.736032	-2.000809
H	3.751765	0.067035	-1.280444
H	3.422701	-1.647521	-0.926034
H	2.270601	0.142189	2.201486
H	3.393266	-1.113598	1.615576
H	3.716557	0.600537	1.268877
H	-3.905116	-0.116242	0.469621
H	-3.646346	-1.822957	0.937619
H	-4.343374	-1.430772	-0.658587

ωB97XD energy = -652.083100275 a.u.

Methyl 5-deoxy-2,3-*O*-isopropylidene-*β*-D-*erythro*-pent-4-enofuranoside (**5**), Conf. C

C	-1.249751	-0.770852	0.747729
O	-1.299095	0.632806	0.989108
C	-0.757161	1.344393	-0.048393
C	0.014221	0.420337	-0.976036
C	-0.192578	-0.971001	-0.356219
C	-0.898224	2.661119	-0.162649
O	1.411994	0.607561	-0.978043
C	1.981404	-0.224687	0.024144
O	1.068807	-1.315971	0.170200
C	3.300625	-0.761439	-0.496669
C	2.129357	0.515824	1.349741
O	-2.506464	-1.281114	0.441202
C	-3.167969	-0.686530	-0.662047
H	-0.948601	-1.260599	1.673891
H	-0.332661	0.514268	-2.007150
H	-0.507352	-1.746667	-1.058542
H	-0.420752	3.176428	-0.986917
H	-1.457839	3.235814	0.565650
H	3.138153	-1.273539	-1.446983
H	4.006965	0.058910	-0.645742
H	3.723801	-1.465653	0.223758
H	2.544754	-0.161106	2.100976
H	2.803067	1.367352	1.225268
H	1.165087	0.882944	1.707161
H	-4.120334	-1.206761	-0.763353
H	-2.602413	-0.808845	-1.596758
H	-3.353355	0.378208	-0.488594

ωB97XD energy = -652.080400985 a.u.

Bicyclomycin (**6**), Conf. A

N	-1.972387	1.334565	-0.390008
C	-2.455493	0.294171	0.515141
C	-1.301207	-0.120368	1.450295
N	-0.070487	-0.129909	0.910717
C	0.255325	0.322759	-0.429047
C	-0.719691	1.444762	-0.864075
O	-0.359227	2.336850	-1.623706
O	-1.562717	-0.414515	2.607620
C	-2.970793	-0.916084	-0.276210
O	-3.450242	0.874886	1.290974
C	-1.966207	-1.818576	-0.957884
C	-4.281505	-1.146915	-0.335733
C	-0.986745	-1.166756	-1.945688
O	0.241088	-0.714082	-1.383405
C	1.699234	0.887345	-0.473356
C	2.819286	0.009601	0.142271
C	2.776152	-1.468402	-0.272417
O	3.849231	-2.189308	0.293318
C	4.172579	0.627622	-0.213391
O	2.656621	-0.019839	1.570245
O	1.757330	2.111877	0.228520
H	1.915032	1.032559	-1.536667
H	-2.656073	2.013099	-0.702219
H	0.714356	-0.337726	1.527459
H	-3.306827	0.547431	2.195095

H	-2.533178	-2.570682	-1.514666
H	-1.377327	-2.367237	-0.212096
H	-4.674770	-1.981789	-0.908065
H	-4.985200	-0.514393	0.192814
H	-1.472575	-0.357286	-2.506146
H	-0.661679	-1.918253	-2.666985
H	2.868987	-1.553816	-1.357024
H	1.817964	-1.912956	0.016109
H	3.797371	-2.081013	1.249272
H	4.972305	0.057079	0.260560
H	4.218681	1.668362	0.115950
H	4.329803	0.601532	-1.296128
H	2.730886	0.886259	1.894039
H	1.300083	2.774336	-0.304225

ωB97XD energy = -1103.92528994 a.u.

Bicyclomycin (**6**), Conf. B

N	-1.523468	-0.811071	-1.243330
C	-2.535133	0.010030	-0.585353
C	-1.937159	1.403991	-0.328025
N	-0.620309	1.443164	-0.061853
C	0.286077	0.313637	-0.037049
C	-0.191807	-0.731397	-1.074804
O	0.581548	-1.466412	-1.676803
O	-2.671683	2.378561	-0.380979
C	-2.984141	-0.637777	0.730756
O	-3.582259	0.147326	-1.487500
C	-2.036587	-0.605816	1.908751
C	-4.188313	-1.201740	0.806835
C	-0.634752	-1.195449	1.699403
O	0.357248	-0.269694	1.254825
C	1.718488	0.879937	-0.376136
C	2.950809	0.157475	0.259289
C	3.014463	-1.375091	0.106018
O	3.257508	-1.796971	-1.210149
C	4.225590	0.762161	-0.323706
O	2.953685	0.485872	1.652102
O	1.773470	2.231179	0.042728
H	1.811628	0.871990	-1.465531
H	-1.865274	-1.523039	-1.877468
H	-0.179248	2.344176	0.103632
H	-3.880302	1.069352	-1.412668
H	-2.506381	-1.169860	2.720022
H	-1.918111	0.420623	2.278170
H	-4.518899	-1.688993	1.719197
H	-4.871124	-1.180462	-0.034670
H	-0.664241	-2.068282	1.034677
H	-0.243805	-1.529016	2.662371
H	2.108417	-1.839008	0.516249
H	3.861188	-1.700496	0.718629
H	2.401867	-1.789617	-1.661535
H	4.313016	0.505180	-1.380925
H	5.086884	0.344745	0.203846
H	4.239780	1.847883	-0.212215
H	2.160963	0.085745	2.034646
H	2.188739	2.215787	0.920173

ωB97XD energy = -1103.92482136 a.u.

Bicyclomycin (**6**), Conf. C

N	1.837877	-0.360192	1.419926
C	2.415984	0.450473	0.351486
C	1.332254	1.411699	-0.179027
N	0.072482	0.945655	-0.205664
C	-0.341577	-0.349086	0.298725
C	0.550005	-0.734709	1.513966
O	0.097672	-1.360365	2.464519
O	1.676416	2.528583	-0.539167
C	2.954125	-0.438876	-0.777059
O	3.417685	1.216924	0.934623
C	1.964469	-1.137189	-1.682458
C	4.270103	-0.564189	-0.941800
C	0.903501	-2.022699	-1.014107
O	-0.314623	-1.363850	-0.679776
C	-1.804107	-0.314712	0.800264
C	-2.956119	-0.196735	-0.263758
C	-2.629392	0.627616	-1.514864
O	-2.302729	1.972826	-1.206526
C	-3.405876	-1.591310	-0.695505
O	-4.056925	0.448957	0.367328
O	-1.941343	0.737539	1.742586
H	-1.945624	-1.272138	1.307861
H	2.462127	-0.626906	2.171072
H	-0.657721	1.593456	-0.523646
H	3.331892	2.102337	0.541043
H	2.539588	-1.774283	-2.361130
H	1.442993	-0.405917	-2.312935
H	4.675271	-1.206489	-1.718146
H	4.966549	-0.022277	-0.312743
H	1.314106	-2.538377	-0.136776
H	0.580820	-2.784935	-1.725444
H	-3.504331	0.595600	-2.175252
H	-1.775391	0.205552	-2.049079
H	-3.012563	2.318021	-0.651475
H	-2.591292	-2.125727	-1.189706
H	-4.255569	-1.510190	-1.378805
H	-3.728889	-2.164605	0.177343
H	-3.733001	0.813337	1.204040
H	-1.610910	0.418678	2.590071

ωB97XD energy = -1103.92382803 a.u.

Bicyclomycin (**6**), Conf. D

N	1.527854	-0.433839	1.424304
C	2.526371	0.190801	0.563331
C	1.924926	1.476482	-0.021885
N	0.604613	1.448978	-0.262598
C	-0.304565	0.340560	-0.012506
C	0.200294	-0.454896	1.215391
O	-0.559217	-1.109787	1.922248
O	2.661045	2.428502	-0.235940
C	2.937580	-0.766403	-0.565705
O	3.596105	0.537420	1.378253
C	1.977094	-0.985143	-1.714478
C	4.124794	-1.368094	-0.511111
C	0.569319	-1.503572	-1.379540
O	-0.415383	-0.503743	-1.135248
C	-1.711755	1.004610	0.212571
C	-3.035233	0.182633	0.015244
C	-3.209919	-1.149708	0.761101
O	-2.447596	-2.211116	0.235146
C	-4.192316	1.088406	0.449191
O	-3.187256	-0.040465	-1.381732
O	-1.797441	2.092516	-0.692536
H	-1.697271	1.426515	1.222761
H	1.881392	-1.026666	2.165865
H	0.171935	2.257986	-0.698647
H	3.900940	1.404231	1.061841
H	2.436148	-1.718544	-2.384093
H	1.866455	-0.062933	-2.298452
H	4.430431	-2.069090	-1.282020
H	4.819684	-1.165979	0.295789
H	0.601457	-2.221093	-0.548411
H	0.172015	-2.031048	-2.247830
H	-4.260043	-1.430454	0.636072
H	-3.011849	-1.005160	1.828080
H	-1.624975	-2.217108	0.739490
H	-4.185748	1.221601	1.535839
H	-5.137719	0.624948	0.158603
H	-4.127603	2.071112	-0.019124
H	-2.589893	-0.770389	-1.599342
H	-2.214236	1.725436	-1.489512

ωB97XD energy = -1103.92226264 a.u.

1α,4β-dihydroxy-8α-acetoxy-guaia-2,10(14),11(13)-triene-6,12-olide (**7**), Conf. A

C	2.834988	-2.136818	-0.344495
C	3.721085	-1.233258	0.061149
C	3.141289	0.156897	0.169333
C	1.736712	-0.009015	-0.495388
C	1.473437	-1.547485	-0.622594
C	0.629423	0.728105	0.225221
C	-0.760320	0.655779	-0.450464
C	-1.604190	-0.465862	0.154141
C	-1.047161	-1.871679	-0.113874
C	0.388294	-2.058521	0.324146
C	0.685165	-2.594999	1.508431
O	0.978185	2.127502	0.294162
C	-0.107422	2.919651	0.125164
C	-1.268898	2.063554	-0.261973
C	4.000103	1.207990	-0.533850
O	3.053481	0.451864	1.560612
C	-2.479721	2.593918	-0.419526
O	1.027635	-1.885534	-1.939242
H	1.790526	0.390021	-1.515413
O	-0.061105	4.113532	0.263443
O	-2.909126	-0.380690	-0.448808
O	-3.846429	-1.322121	1.373006
C	-3.952955	-0.844855	0.267497

C	-5.241636	-0.676364	-0.489362
H	3.011856	-3.199841	-0.473019
H	4.753967	-1.431182	0.329494
H	0.568266	0.373599	1.259122
H	-0.634237	0.457327	-1.523158
H	-1.715843	-0.305556	1.231438
H	-1.680961	-2.580822	0.423802
H	-1.140225	-2.071727	-1.184428
H	-0.102523	-2.927080	2.178719
H	1.708395	-2.701434	1.853343
H	3.514434	2.187925	-0.487796
H	4.978126	1.274215	-0.048408
H	4.149702	0.950345	-1.586860
H	2.822869	1.383886	1.656014
H	-2.618642	3.656171	-0.241628
H	-3.329242	1.997153	-0.727046
H	1.754702	-1.729443	-2.550057
H	-5.438695	0.387031	-0.652663
H	-6.057624	-1.116537	0.081516
H	-5.165128	-1.152717	-1.469695

ωB97XD energy = -1110.90058117 a.u.

Mexicanin (**8**), Conf. A

C	-1.338298	-0.585197	0.251378
C	-1.636991	0.769925	-0.452003
C	-3.123375	0.684149	-0.725512
C	-3.616497	-0.554800	-0.597371
C	-2.538570	-1.453990	-0.149136
C	-0.024233	-1.262485	-0.222016
C	1.119791	-0.265145	-0.463838
C	1.254967	0.930692	0.500892
C	0.425974	2.155140	0.133320
C	-1.107130	2.037694	0.249310
C	2.522129	-0.808056	-0.490821
C	3.414033	0.300498	-0.042031
O	2.642855	1.315486	0.423257
O	-2.557628	-2.669859	-0.075633
C	-1.732115	3.314587	-0.324059
H	-1.171493	0.754862	-1.451538
O	4.615080	0.363488	-0.068675
C	2.994728	-2.001534	-0.835446
C	-1.450450	-0.493091	1.791372
O	0.385853	-2.268215	0.676767
H	-3.698678	1.537476	-1.069182
H	-4.623903	-0.887721	-0.813036
H	-0.233071	-1.724798	-1.199734
H	0.941728	0.171312	-1.457314
H	1.079983	0.622157	1.535227
H	0.760866	2.987972	0.760235
H	0.682432	2.422341	-0.901782
H	-1.374021	1.988055	1.310631
H	-1.602887	3.363165	-1.412058
H	-2.800375	3.386385	-0.102774
H	-1.252086	4.198982	0.104829
H	4.065160	-2.180630	-0.802911
H	2.339543	-2.813516	-1.128970
H	-1.469239	-1.492675	2.227006
H	-0.619231	0.042582	2.247473
H	-2.378852	0.015084	2.069286
H	-0.318937	-2.928554	0.694651

ωB97XD energy = -883.096903969 a.u.

Neurolenin A (**9**), Conf. A

C	3.384325	0.372899	-1.232204
C	3.318683	-0.953264	-1.398106
C	3.014370	-1.974942	-0.338260
C	1.569823	-2.490069	-0.486321
C	2.159485	2.207317	0.125846
C	3.157765	1.039072	0.073906
C	0.472839	-1.425229	-0.586340
C	0.354055	-0.478412	0.633606
C	-0.113441	0.952178	0.307350
C	0.812388	1.839492	-0.525903
O	3.713848	0.696219	1.105439
C	4.005831	-3.141896	-0.414154
C	2.786607	3.404290	-0.611653
O	1.943708	2.548051	1.474007
H	0.267465	2.774811	-0.687762
O	-0.783108	-2.134785	-0.654406
C	-1.417724	-2.122223	0.538572
C	-0.692009	-1.190953	1.450594
C	-0.999168	-1.055074	2.737033
O	-2.406857	-2.773667	0.757700
O	-1.345384	0.805162	-0.421878
C	-2.305294	1.739285	-0.239692
C	-3.546697	1.402744	-1.024707
O	-2.155741	2.709851	0.466016
C	-4.121269	0.010598	-0.705869
C	-4.439516	-0.129355	0.783942
C	-5.362743	-0.247178	-1.561091
H	3.501768	1.017793	-2.099147
H	3.422641	-1.343662	-2.411870
H	3.125317	-1.509878	0.643085
H	1.489364	-3.123563	-1.378046
H	1.347435	-3.134472	0.373518
H	0.553347	-0.870674	-1.523489
H	1.296789	-0.406272	1.180970
H	-0.320321	1.463923	1.249918
H	0.983429	1.391652	-1.510606
H	3.773733	-3.890191	0.349472
H	5.029371	-2.793946	-0.250898
H	3.961072	-3.634317	-1.392220
H	2.868490	3.230444	-1.688774
H	3.779184	3.624447	-0.208502
H	2.148421	4.275743	-0.447559
H	2.639043	2.107192	1.986598
H	-0.460667	-0.367586	3.382288
H	-1.812242	-1.635482	3.162783
H	-4.282273	2.182612	-0.807346
H	-3.296197	1.460900	-2.090950

H	-3.364289	-0.735384	-0.969862
H	-3.548171	0.014016	1.403708
H	-5.188173	0.610381	1.092143
H	-4.826177	-1.128731	0.999953
H	-5.137983	-0.166409	-2.630024
H	-6.154668	0.474718	-1.327185
H	-5.757312	-1.250409	-1.374505

ωB97XD energy = -1230.05669807 a.u.

Neurolenin A (**9**), Conf. B

C	3.492652	0.664328	-1.096385
C	3.592234	-0.658631	-1.271738
C	3.329441	-1.722369	-0.242672
C	1.964632	-2.388566	-0.501904
C	1.979101	2.340474	0.172330
C	3.097605	1.285718	0.191100
C	0.773731	-1.441309	-0.672307
C	0.452002	-0.552012	0.554967
C	-0.139710	0.830660	0.221841
C	0.721560	1.825233	-0.555818
O	3.606253	0.989721	1.261098
C	4.447938	-2.770355	-0.251917
C	2.510693	3.598298	-0.537558
O	1.647094	2.659779	1.501762
H	0.080968	2.692866	-0.743924
O	-0.391478	-2.269396	-0.873141
C	-1.147079	-2.337744	0.243929
C	-0.589642	-1.388779	1.251978
C	-1.020060	-1.336955	2.508631
O	-2.104627	-3.062258	0.339935
O	-1.314518	0.560526	-0.565676
C	-2.405569	1.327925	-0.360239
C	-3.593367	0.826239	-1.140227
O	-2.410619	2.288236	0.375482
C	-4.808636	0.580284	-0.228181
C	-6.016659	0.172611	-1.073159
C	-4.498073	-0.474662	0.836845
H	3.595707	1.328476	-1.950486
H	3.809853	-1.021193	-2.277795
H	3.315486	-1.261124	0.746519
H	2.017103	-3.008573	-1.405197
H	1.750147	-3.069480	0.331281
H	0.878033	-0.850615	-1.584703
H	1.330549	-0.402712	1.187169
H	-0.445894	1.303963	1.157540
H	0.996910	1.412634	-1.532191
H	4.528368	-3.254618	-1.231857
H	4.249472	-3.547670	0.492010
H	5.411969	-2.311352	-0.016682
H	1.769868	4.392411	-0.417898
H	2.672532	3.432373	-1.606782
H	3.447179	3.930660	-0.080809
H	2.339270	2.274530	2.061488
H	-0.607826	-0.633526	3.225804
H	-1.811074	-2.005429	2.835318
H	-3.841706	1.592201	-1.883899
H	-3.320845	-0.090738	-1.671289
H	-5.034716	1.528079	0.274781
H	-6.253792	0.930418	-1.827462
H	-5.827350	-0.775193	-1.590686
H	-6.901311	0.036304	-0.443343
H	-3.679270	-0.157332	1.491523
H	-4.209135	-1.427768	0.380251
H	-5.374116	-0.652775	1.468535

ωB97XD energy = -1230.05571350 a.u.

Neurolenin A (**9**), Conf. C

C	2.999929	0.776700	-1.456093
C	3.087618	-0.530827	-1.727949
C	3.098232	-1.657814	-0.733093
C	1.723390	-2.353943	-0.701704
C	1.796512	2.336052	0.228555
C	2.909431	1.319944	-0.078518
C	0.515222	-1.433823	-0.511753
C	0.495370	-0.633318	0.814071
C	-0.198798	0.739080	0.721678
C	0.410830	1.804982	-0.188662
O	3.666600	0.989905	0.820868
C	4.201917	-2.666844	-1.069717
C	2.102269	3.638636	-0.531978
O	1.797002	2.590858	1.611967
H	-0.281747	2.652892	-0.161793
O	-0.659680	-2.272451	-0.481476
C	-1.136345	-2.410945	0.775334
C	-0.328649	-1.545203	1.684555
C	-0.404876	-1.616001	3.009767
O	-2.066842	-3.126504	1.044870
O	-1.516265	0.451643	0.223201
C	-2.567731	1.121077	0.741068
C	-3.870591	0.562135	0.226920
O	-2.450958	2.024902	1.535132
C	-3.879704	0.184497	-1.262350
C	-3.576133	1.398795	-2.143245
C	-5.227970	-0.438507	-1.628700
H	2.885662	1.487500	-2.270325
H	3.072411	-0.829899	-2.777498
H	3.311162	-1.251573	0.257610
H	1.566324	-2.910458	-1.633617
H	1.729857	-3.093771	0.108246
H	0.390265	-0.780996	-1.378255
H	1.498717	-0.489521	1.220595
H	-0.283209	1.153121	1.729120
H	0.442063	1.450075	-1.224535
H	5.185345	-2.189763	-1.045798
H	4.052756	-3.099145	-2.065778
H	4.203044	-3.486022	-0.344588
H	1.977964	3.523339	-1.612782
H	3.120226	3.979010	-0.322075
H	1.405637	4.404417	-0.182876
H	2.609873	2.198547	1.967108
H	0.194235	-0.975969	3.650286
H	-1.084420	-2.323432	3.475308

H	-4.647246	1.299282	0.452079
H	-4.080674	-0.331454	0.829239
H	-3.098379	-0.567159	-1.420559
H	-2.594744	1.829034	-1.916443
H	-4.329908	2.182158	-1.998218
H	-3.580977	1.122592	-3.202577
H	-6.045426	0.274175	-1.464683
H	-5.247164	-0.730128	-2.683589
H	-5.430027	-1.331720	-1.029211

ωB97XD energy = -1230.05511611 a.u.

Neurolenin A (**9**), Conf. D

C	2.991280	0.842589	-1.446914
C	3.106411	-0.459333	-1.735354
C	3.139305	-1.599069	-0.755484
C	1.779600	-2.324798	-0.735653
C	1.752274	2.354524	0.254881
C	2.886074	1.365080	-0.062460
C	0.551148	-1.434043	-0.535927
C	0.512628	-0.650378	0.799525
C	-0.209105	0.708868	0.724403
C	0.378579	1.799194	-0.170911
O	3.645621	1.036192	0.835419
C	4.264479	-2.579720	-1.104631
C	2.031109	3.671860	-0.490183
O	1.746290	2.594182	1.640920
H	-0.331948	2.631774	-0.133572
O	-0.604404	-2.299177	-0.516830
C	-1.079209	-2.463416	0.737555
C	-0.293607	-1.589208	1.658038
C	-0.373378	-1.674170	2.982212
O	-1.992940	-3.203803	0.997296
O	-1.521766	0.403836	0.223219
C	-2.586353	1.029470	0.769462
C	-3.876609	0.455959	0.241116
O	-2.486790	1.910138	1.591330
C	-3.906288	0.216034	-1.276622
C	-5.240799	-0.418745	-1.672494
C	-3.663151	1.516111	-2.047107
H	2.863618	1.561322	-2.252123
H	3.099153	-0.744938	-2.788729
H	3.343054	-1.201963	0.240802
H	1.635844	-2.873041	-1.674579
H	1.800953	-3.074347	0.065075
H	0.411879	-0.773505	-1.394379
H	1.512109	-0.490969	1.209694
H	-0.300545	1.107401	1.737390
H	0.418140	1.459720	-1.211605
H	4.125096	-3.001771	-2.106479
H	4.282456	-3.408451	-0.390561
H	5.237691	-2.082538	-1.073217
H	1.317845	4.418798	-0.133863
H	1.910939	3.566017	-1.572434
H	3.041189	4.031715	-0.274833
H	2.563635	2.209039	1.993711
H	0.209195	-1.027283	3.631038
H	-1.039312	-2.400410	3.438358
H	-4.030005	-0.497517	0.762758
H	-4.678279	1.134833	0.546613
H	-3.104305	-0.489934	-1.519298
H	-5.397085	-1.370526	-1.155038
H	-6.078147	0.244868	-1.424768
H	-5.275497	-0.611868	-2.749347
H	-2.686392	1.951112	-1.809216
H	-4.430966	2.260877	-1.805940
H	-3.694736	1.339781	-3.126991

ωB97XD energy = -1230.05508425 a.u.

Neurolenin A (**9**), Conf. E

C	3.361697	1.023362	-1.208270
C	3.629777	-0.270274	-1.423162
C	3.566952	-1.380690	-0.412120
C	2.287429	-2.215448	-0.614751
C	1.716540	2.468668	0.175536
C	2.959930	1.565999	0.112611
C	0.975137	-1.429222	-0.672513
C	0.632292	-0.618233	0.601679
C	-0.163210	0.679307	0.353141
C	0.490273	1.790238	-0.467285
O	3.558187	1.318742	1.148062
C	4.808774	-2.273819	-0.510099
C	2.029211	3.782919	-0.561443
O	1.436838	2.749307	1.525252
H	-0.277257	2.561863	-0.590748
O	-0.087660	-2.395162	-0.821611
C	-0.757491	-2.574999	0.336588
C	-0.239632	-1.599854	1.340507
C	-0.577494	-1.638079	2.625529
O	-1.630262	-3.395788	0.465418
O	-1.354106	0.287289	-0.353574
C	-2.555556	0.553214	0.211309
C	-3.654112	-0.173699	-0.521047
O	-2.691331	1.246561	1.191352
C	-5.051071	0.409136	-0.294636
C	-5.183190	1.794162	-0.933036
C	-6.110482	-0.551305	-0.840048
H	3.330856	1.710932	-2.049343
H	3.840248	-0.578045	-2.448826
H	3.550533	-0.944676	0.588468
H	2.362326	-2.793319	-1.543994
H	2.220804	-2.945021	0.202028
H	0.938008	-0.807658	-1.569711
H	1.527326	-0.368891	1.176375
H	-0.448913	1.092296	1.322672
H	0.742382	1.433780	-1.471796
H	5.718939	-1.699357	-0.317581
H	4.892821	-2.727631	-1.504214
H	4.755230	-3.082254	0.225055
H	2.127542	3.639013	-1.641394
H	2.949058	4.230481	-0.174407
H	1.207715	4.479298	-0.377110
H	2.194340	2.430234	2.040159

H	-0.201549	-0.909113	3.336756
H	-1.258043	-2.405017	2.982804
H	-3.406516	-0.209421	-1.588044
H	-3.604527	-1.212788	-0.166901
H	-5.195781	0.515292	0.786668
H	-4.449848	2.494838	-0.522911
H	-5.035330	1.733345	-2.018608
H	-6.179028	2.212205	-0.755178
H	-5.981891	-0.702706	-1.918716
H	-7.116825	-0.152848	-0.677062
H	-6.050588	-1.530718	-0.354569

ωB97XD energy = -1230.05473727 a.u.

Neurolenin A (**9**), Conf. F

C	3.436084	0.930632	-0.974316
C	3.677784	-0.373601	-1.151801
C	3.467622	-1.469859	-0.144348
C	2.191295	-2.264879	-0.480098
C	1.693820	2.427815	0.225369
C	2.913284	1.493824	0.294461
C	0.919579	-1.440657	-0.698780
C	0.450359	-0.605026	0.518527
C	-0.263521	0.713540	0.163195
C	0.533600	1.793532	-0.568226
O	3.395035	1.239588	1.387441
C	4.682317	-2.404152	-0.103418
C	2.127065	3.739133	-0.453876
O	1.269075	2.698147	1.539072
H	-0.178817	2.593446	-0.790835
O	-0.144349	-2.379347	-0.964306
C	-0.944819	-2.535112	0.111516
C	-0.531671	-1.553488	1.156246
C	-1.018386	-1.566447	2.393070
O	-1.832243	-3.349236	0.151103
O	-1.368961	0.323974	-0.673444
C	-2.502297	1.055626	-0.613045
C	-3.614405	0.405717	-1.397805
O	-2.585476	2.085808	0.017140
C	-4.815608	0.003227	-0.509743
C	-4.374808	-0.863697	0.673309
C	-5.631281	1.212377	-0.045204
H	3.518111	1.612596	-1.816607
H	3.985823	-0.700865	-2.146326
H	3.357607	-1.023517	0.845818
H	2.351072	-2.859621	-1.387666
H	2.007149	-2.979175	0.332203
H	1.006790	-0.829192	-1.599234
H	1.276531	-0.373064	1.194548
H	-0.660071	1.147206	1.084698
H	0.902515	1.413581	-1.527020
H	4.853802	-2.871195	-1.079998
H	4.525260	-3.202646	0.627966
H	5.585840	-1.857019	0.179040
H	3.014068	4.153237	0.034050
H	1.311729	4.459068	-0.351122
H	2.335682	3.601077	-1.518993
H	1.969771	2.375719	2.127683
H	-0.705939	-0.839330	3.136478
H	-1.754560	-2.312383	2.676799
H	-3.946739	1.122016	-2.157053
H	-3.213923	-0.475664	-1.904012
H	-5.457471	-0.609532	-1.155526
H	-3.799709	-1.736772	0.350092
H	-3.754320	-0.285455	1.368964
H	-5.246863	-1.219758	1.230735
H	-5.974483	1.810495	-0.896190
H	-5.036185	1.862297	0.602304
H	-6.513408	0.881944	0.512962

ωB97XD energy = -1230.05439208 a.u.

Neurolenin A (**9**), Conf. G

C	-2.730599	-1.445564	-1.351241
C	-3.093557	-0.238509	-1.801184
C	-3.411223	0.972648	-0.969068
C	-2.235409	1.968254	-1.001212
C	-1.290713	-2.444905	0.556189
C	-2.589112	-1.766105	0.089710
C	-0.851862	1.391851	-0.688793
C	-0.690805	0.777309	0.724614
C	0.300622	-0.398116	0.808140
C	-0.041041	-1.689190	0.063794
O	-3.444442	-1.504673	0.921078
C	-4.694913	1.645422	-1.468570
C	-1.274422	-3.883434	0.008681
O	-1.289215	-2.479322	1.962768
H	0.814765	-2.356192	0.210260
O	0.080399	2.491783	-0.745657
C	0.437806	2.901299	0.491966
C	-0.143972	1.954555	1.488906
C	-0.118591	2.176598	2.799289
O	1.119647	3.874374	0.685951
O	1.530735	0.106966	0.256246
C	2.693578	-0.364942	0.749228
C	3.878159	0.253061	0.054378
O	2.755239	-1.207539	1.615475
C	4.552738	-0.749602	-0.903729
C	3.587173	-1.223025	-1.993673
C	5.805329	-0.119773	-1.514843
H	-2.414755	-2.208343	-2.058083
H	-3.089576	-0.079241	-2.880843
H	-3.579836	0.659154	0.062897
H	-2.172806	2.441574	-1.988589
H	-2.446715	2.770641	-0.283311
H	-0.537978	0.694953	-1.468739
H	-1.647378	0.447614	1.136479
H	0.458608	-0.638645	1.861956
H	-0.120004	-1.498391	-1.011916
H	-5.545534	0.962350	-1.395677
H	-4.593590	1.958417	-2.514085
H	-4.918906	2.535061	-0.872324
H	-1.142621	-3.910741	-1.077194
H	-2.198935	-4.406011	0.270157

H	-0.438032	-4.411234	0.472967
H	-2.187086	-2.245512	2.246374
H	-0.545208	1.470893	3.505836
H	0.345364	3.078650	3.186475
H	3.560297	1.145317	-0.493241
H	4.590364	0.552209	0.828939
H	4.855277	-1.618986	-0.306623
H	2.718393	-1.739462	-1.572464
H	3.219081	-0.373403	-2.580746
H	4.086992	-1.916901	-2.676718
H	5.545252	0.761207	-2.113597
H	6.318709	-0.830687	-2.169913
H	6.511844	0.196010	-0.740662

ωB97XD energy = -1230.05425330 a.u.

Neurolenin A (**9**), Conf. H

C	2.882925	1.439256	-1.306953
C	3.277267	0.225817	-1.711353
C	3.540857	-0.970542	-0.840108
C	2.372342	-1.970952	-0.934625
C	1.319556	2.473518	0.480704
C	2.644793	1.785002	0.115452
C	0.970616	-1.394557	-0.716631
C	0.724959	-0.755540	0.673126
C	-0.270475	0.420051	0.678147
C	0.106352	1.698615	-0.070007
O	3.441055	1.536806	1.007402
C	4.857114	-1.647542	-1.239067
C	1.333925	3.896888	-0.104877
O	1.228698	2.545738	1.882734
H	-0.762199	2.360799	0.010676
O	0.045625	-2.498428	-0.809720
C	-0.389377	-2.883717	0.410567
C	0.131124	-1.918743	1.423577
C	0.022899	-2.115947	2.733730
O	-1.084389	-3.852089	0.580325
O	-1.468989	-0.091433	0.064979
C	-2.659164	0.288066	0.573285
C	-3.803781	-0.389197	-0.136550
O	-2.772470	1.079530	1.481051
C	-4.848019	0.619029	-0.642162
C	-4.235145	1.582958	-1.661793
C	-6.047286	-0.121131	-1.236404
H	2.614716	2.189618	-2.045937
H	3.348295	0.049780	-2.786019
H	3.639743	-0.638711	0.195148
H	2.374789	-2.456563	-1.917939
H	2.540042	-2.763654	-0.194665
H	0.701970	-0.712823	-1.526283
H	1.655172	-0.418471	1.135881
H	-0.486811	0.679600	1.716839
H	0.250234	1.489123	-1.135290
H	5.698877	-0.959761	-1.121258
H	4.828709	-1.981047	-2.282774
H	5.042026	-2.524349	-0.611415
H	1.266098	3.894788	-1.196890

H	2.240319	4.429220	0.197146
H	0.470981	4.434289	0.295284
H	2.104058	2.311204	2.229365
H	0.405000	-1.397088	3.452319
H	-0.465051	-3.010822	3.107879
H	-3.415500	-0.995163	-0.960486
H	-4.265187	-1.070767	0.587318
H	-5.186766	1.201363	0.222852
H	-3.407667	2.154858	-1.228533
H	-3.852288	1.036135	-2.531981
H	-4.981691	2.300260	-2.016626
H	-5.744654	-0.723674	-2.101291
H	-6.812509	0.585625	-1.572436
H	-6.505852	-0.792755	-0.503400

ωB97XD energy = -1230.05415525 a.u.

Neurolenin A (**9**), Conf. I

C	2.726545	1.282989	-1.336349
C	3.041754	0.039865	-1.719620
C	3.269708	-1.145483	-0.823364
C	2.038257	-2.071955	-0.841135
C	1.296339	2.456637	0.478025
C	2.564462	1.681659	0.083306
C	0.687148	-1.402229	-0.581048
C	0.535523	-0.728153	0.806233
C	-0.392210	0.501955	0.819683
C	0.018216	1.732733	0.010374
O	3.381380	1.413776	0.950587
C	4.522238	-1.917529	-1.253061
C	1.363006	3.854550	-0.162360
O	1.270579	2.583977	1.878821
H	-0.807509	2.445160	0.106498
O	-0.311969	-2.442719	-0.623163
C	-0.738123	-2.770669	0.615947
C	-0.102906	-1.840934	1.595988
C	-0.157728	-2.025562	2.911187
O	-1.509699	-3.672018	0.822522
O	-1.635254	0.021162	0.280474
C	-2.791314	0.550576	0.730729
C	-3.976369	-0.122134	0.086062
O	-2.836502	1.450573	1.537483
C	-4.168871	0.232677	-1.407189
C	-4.268519	1.746280	-1.614454
C	-3.106842	-0.384212	-2.322289
H	2.471229	2.026786	-2.086431
H	3.061887	-0.168379	-2.790749
H	3.431166	-0.790650	0.196216
H	1.966658	-2.578682	-1.811181
H	2.187895	-2.856659	-0.088836
H	0.433494	-0.716176	-1.392104
H	1.500585	-0.440711	1.229177
H	-0.548652	0.805974	1.857437
H	0.101351	1.479619	-1.052184
H	5.410517	-1.282324	-1.198363
H	4.424919	-2.286052	-2.280698
H	4.680027	-2.781303	-0.600450

H	1.242644	3.815591	-1.249125
H	2.311445	4.344412	0.075676
H	0.551777	4.455787	0.254795
H	2.144811	2.312386	2.199401
H	0.313030	-1.334626	3.604060
H	-0.689029	-2.881453	3.316146
H	-3.856254	-1.205603	0.191524
H	-4.856735	0.189996	0.652533
H	-5.135041	-0.210494	-1.679479
H	-5.033940	2.191839	-0.971602
H	-3.315633	2.238186	-1.384164
H	-4.516018	1.977230	-2.655334
H	-2.129454	0.081942	-2.164636
H	-3.385156	-0.239509	-3.371349
H	-2.990148	-1.456673	-2.140545

ωB97XD energy = -1230.05397232 a.u.

Neurolenin A (**9**), Conf. J

C	-2.793176	-1.616647	-1.157535
C	-3.311197	-0.443971	-1.542198
C	-3.600402	0.742465	-0.665849
C	-2.520077	1.823914	-0.861850
C	-1.033219	-2.510269	0.518950
C	-2.428285	-1.924405	0.246626
C	-1.068587	1.353408	-0.735889
C	-0.681024	0.771222	0.646048
C	0.394745	-0.330754	0.610395
C	0.077684	-1.643344	-0.105807
O	-3.173456	-1.721002	1.192396
C	-4.990472	1.313121	-0.968200
C	-0.974068	-3.930066	-0.072081
O	-0.842781	-2.577453	1.911048
H	1.000623	-2.232490	-0.070200
O	-0.232787	2.515214	-0.920844
C	0.258423	2.963032	0.256149
C	-0.119970	1.993297	1.325654
C	0.071750	2.230382	2.619511
O	0.895319	3.980383	0.352146
O	1.524738	0.246247	-0.068137
C	2.749639	0.099029	0.484508
C	3.804686	0.783925	-0.345611
O	2.948467	-0.527213	1.500185
C	4.624539	-0.209542	-1.202291
C	5.515980	-1.114586	-0.348547
C	3.726941	-1.031150	-2.132014
H	-2.521045	-2.354159	-1.907836
H	-3.472264	-0.288839	-2.610352
H	-3.593798	0.421688	0.377588
H	-2.629222	2.286957	-1.850075
H	-2.692369	2.617822	-0.124195
H	-0.811064	0.670716	-1.548232
H	-1.550830	0.383015	1.181015
H	0.679064	-0.559164	1.639779
H	-0.138789	-1.460277	-1.163747
H	-5.070817	1.624933	-2.015866
H	-5.189400	2.187642	-0.341563
H	-5.767228	0.569165	-0.771610
H	-0.048276	-4.399223	0.269138
H	-0.977644	-3.923073	-1.166093
H	-1.814431	-4.532005	0.285186
H	-1.703137	-2.390463	2.318537
H	-0.204853	1.505135	3.378613
H	0.524682	3.164472	2.937550
H	3.322246	1.521688	-0.991340
H	4.472640	1.306995	0.344454
H	5.275312	0.414784	-1.827521
H	6.180463	-0.525127	0.290915
H	4.918444	-1.759808	0.302725
H	6.135983	-1.751483	-0.987949
H	3.082949	-0.388892	-2.741179
H	3.080976	-1.706263	-1.558035
H	4.332308	-1.647526	-2.803884

ωB97XD energy = -1230.05324546 a.u.

Neurolenin A (**9**), Conf. K

C	3.449951	1.015571	-1.039847
C	3.731079	-0.280632	-1.219731
C	3.577942	-1.382448	-0.208326
C	2.318738	-2.215529	-0.517111
C	1.697692	2.475805	0.189649
C	2.938768	1.569271	0.237597
C	1.019319	-1.426359	-0.695947
C	0.565639	-0.606769	0.537290
C	-0.199087	0.691948	0.208799
C	0.529818	1.796901	-0.553695
O	3.445599	1.328189	1.322198
C	4.821120	-2.279207	-0.194529
C	2.076534	3.783631	-0.527637
O	1.307281	2.764701	1.509507
H	-0.220890	2.569796	-0.750338
O	-0.028985	-2.389793	-0.936092
C	-0.808250	-2.555007	0.153838
C	-0.378872	-1.578450	1.196444
C	-0.839190	-1.602843	2.443210
O	-1.698757	-3.365608	0.202315
O	-1.319387	0.300813	-0.604995
C	-2.570848	0.554293	-0.148225
C	-3.579778	-0.150632	-1.022588
O	-2.791139	1.231187	0.827694
C	-5.057426	0.152994	-0.742279
C	-5.502824	-0.330104	0.641688
C	-5.399745	1.629105	-0.966986
H	3.491779	1.695934	-1.886302
H	4.027388	-0.598953	-2.220734
H	3.476004	-0.939493	0.784184
H	2.473591	-2.799454	-1.432541
H	2.177765	-2.939735	0.295097
H	1.064766	-0.809432	-1.595937
H	1.404120	-0.357415	1.191625
H	-0.573525	1.109651	1.145487
H	0.870264	1.433462	-1.529377
H	4.985505	-2.741398	-1.174620

H	4.704261	-3.081473	0.540191
H	5.713677	-1.704885	0.068407
H	2.967334	4.228074	-0.074693
H	1.248246	4.486766	-0.412046
H	2.255826	3.631426	-1.596053
H	2.017935	2.441861	2.085976
H	-0.525550	-0.870889	3.180969
H	-1.557863	-2.360435	2.740385
H	-3.327361	0.082756	-2.063452
H	-3.383372	-1.224480	-0.904934
H	-5.612959	-0.431651	-1.487764
H	-5.276522	-1.392809	0.780043
H	-5.000682	0.233499	1.432305
H	-6.583185	-0.198238	0.760963
H	-4.895376	2.264427	-0.233443
H	-6.477962	1.790603	-0.868190
H	-5.103595	1.957347	-1.969907

ωB97XD energy = -1230.05310623 a.u.

Swinhoeisterol F (**10**), Conf. A

C	4.511172	-2.874739	0.205604
C	4.897861	-2.164519	-1.087687
C	4.749003	-0.663755	-0.908912
C	3.333276	-0.252811	-0.545326
C	2.844368	-0.978872	0.750152
C	3.083326	-2.499520	0.597566
C	3.124333	1.269752	-0.492612
C	1.871781	1.701906	0.307824
C	0.803118	0.626771	0.343609
C	1.315022	-0.748179	0.817026
C	-0.499277	0.956201	1.115500
C	-0.812440	-0.368775	1.853726
C	0.578677	-0.952010	2.147294
C	1.413274	3.073780	-0.182113
C	0.296162	3.182043	-1.202580
C	-1.083568	2.657701	-0.761782
C	-1.086075	1.176604	-0.323510
C	3.604797	-0.480847	1.989911
C	-0.529294	2.106428	2.116164
C	5.789661	0.160358	-1.043121
O	6.197552	-2.587926	-1.443502
O	1.993035	4.068613	0.214022
C	-2.376465	0.445834	-0.702220
C	-3.567191	1.008077	0.086845
C	-2.615257	0.426256	-2.216343
C	-4.905636	0.304540	-0.175094
C	-4.918550	-1.206065	0.108334
C	-4.683734	-1.493515	1.595329
C	-6.195697	-1.907992	-0.415484
C	-7.496575	-1.301425	0.121420
C	-6.228912	-1.974882	-1.946207
H	2.681801	-0.637944	-1.346226
H	2.174228	1.872226	1.345827
H	0.870005	-1.447645	0.098082
O	0.071123	0.517786	-0.903510
H	5.231964	-2.600023	0.983447
H	4.593200	-3.955689	0.055838
H	4.181531	-2.477391	-1.867775
H	2.805893	-3.003741	1.530607
H	2.404406	-2.883603	-0.175964
H	3.067254	1.645291	-1.520208
H	3.979732	1.766507	-0.029520
H	-1.406727	-0.201670	2.757471
H	-1.364473	-1.074254	1.227190
H	0.542050	-1.999631	2.460929
H	1.064545	-0.385250	2.951487
H	0.605538	2.612597	-2.085923
H	0.218094	4.237048	-1.475420
H	-1.748982	2.785213	-1.619667
H	-1.494488	3.287362	0.033348
H	3.352437	-1.086880	2.866031
H	3.384941	0.560232	2.236220
H	4.684985	-0.551649	1.835573
H	-0.103100	3.040234	1.745128
H	0.028657	1.836484	3.019199
H	-1.563281	2.309658	2.416019
H	5.706485	1.233846	-0.921522
H	6.778427	-0.229447	-1.261873
H	6.418162	-2.222853	-2.305582
H	-2.223532	-0.595854	-0.404747
H	-3.691511	2.071343	-0.157005
H	-3.334277	0.968042	1.158796
H	-3.341010	-0.349087	-2.479512
H	-1.684607	0.207375	-2.747536
H	-3.012259	1.377484	-2.587841
H	-5.199390	0.486803	-1.215061
H	-5.670235	0.789128	0.445048
H	-4.085573	-1.663876	-0.443954
H	-4.730148	-2.568483	1.798986
H	-3.701677	-1.138862	1.922175
H	-5.434722	-0.999251	2.222275
H	-6.140712	-2.943919	-0.051420
H	-8.353557	-1.919076	-0.166380
H	-7.495513	-1.224953	1.213221
H	-7.664368	-0.298924	-0.287539
H	-7.074898	-2.579923	-2.288632
H	-5.312753	-2.423915	-2.345266
H	-6.339184	-0.980920	-2.394306

ωB97XD energy = -1358.18121584 a.u.

Swinhoeisterol F (**10**), Conf. B

C	4.450064	-2.947857	0.164135
C	4.895767	-2.193122	-1.084043
C	4.772233	-0.698027	-0.846860
C	3.353874	-0.271179	-0.512795
C	2.803000	-1.042252	0.730551
C	3.016599	-2.559809	0.521170
C	3.176220	1.251591	-0.395659
C	1.906122	1.676132	0.380979
C	0.811719	0.627185	0.328098
C	1.277363	-0.780504	0.750811

C	-0.508616	0.950578	1.071780
C	-0.878552	-0.403792	1.725022
C	0.486722	-1.034177	2.040818
C	1.500161	3.081416	-0.056321
C	0.426536	3.264157	-1.110798
C	-0.979950	2.760357	-0.737231
C	-1.039750	1.259758	-0.372403
C	3.527697	-0.616383	2.018165
C	-0.547136	2.046411	2.131286
C	5.833787	0.107518	-0.913184
O	6.197911	-2.630226	-1.413476
O	2.085226	4.041092	0.412005
C	-2.340956	0.594405	-0.830646
C	-3.539941	1.175894	-0.067514
C	-2.514925	0.646586	-2.353011
C	-4.894655	0.539587	-0.404221
C	-4.992664	-0.977272	-0.176683
C	-4.727854	-1.338128	1.289304
C	-6.333537	-1.538877	-0.710390
C	-6.284920	-3.060274	-0.885988
C	-7.547310	-1.142073	0.137241
H	2.723475	-0.604470	-1.352963
H	2.174017	1.789545	1.435902
H	0.846710	-1.433992	-0.018270
O	0.117252	0.594241	-0.945229
H	5.147809	-2.720019	0.977475
H	4.516788	-4.023308	-0.027083
H	4.200452	-2.457363	-1.900315
H	2.695576	-3.096523	1.421570
H	2.358609	-2.897134	-0.291289
H	3.163844	1.676105	-1.405545
H	4.025581	1.705923	0.119306
H	-1.508058	-0.272698	2.610638
H	-1.415693	-1.062451	1.037634
H	0.414469	-2.095424	2.296891
H	0.954128	-0.521639	2.891008
H	0.751385	2.723348	-2.006362
H	0.385314	4.331412	-1.340893
H	-1.614477	2.948708	-1.607203
H	-1.396933	3.363123	0.075349
H	3.239035	-1.263132	2.852913
H	3.312563	0.413399	2.311167
H	4.611434	-0.694439	1.895309
H	-0.082370	2.986176	1.827789
H	-0.031829	1.713246	3.038380
H	-1.586287	2.262514	2.402900
H	5.769423	1.176982	-0.750994
H	6.820630	-0.295350	-1.116223
H	6.454272	-2.238440	-2.253646
H	-2.243523	-0.464077	-0.571282
H	-3.612948	2.251054	-0.276767
H	-3.351324	1.088317	1.010235
H	-3.255141	-0.089936	-2.679287
H	-1.570750	0.415296	-2.854134
H	-2.860881	1.626772	-2.699302
H	-5.140565	0.755049	-1.451936
H	-5.661361	1.044418	0.197122
H	-4.209194	-1.454869	-0.782833
H	-5.383305	-0.770337	1.959409
H	-4.889267	-2.402894	1.482046
H	-3.695746	-1.112699	1.571201
H	-6.471768	-1.102852	-1.710377
H	-7.202636	-3.426553	-1.357648
H	-5.440341	-3.361995	-1.514912
H	-6.187458	-3.573389	0.077337
H	-8.472706	-1.477136	-0.342206
H	-7.621243	-0.059040	0.275441
H	-7.505403	-1.607184	1.128385

ωB97XD energy = -1358.18106816 a.u.

Swinhoeisterol F (**10**), Conf. C

C	-4.991145	-2.503835	0.167892
C	-5.465395	-1.373976	1.075806
C	-5.038525	-0.037875	0.492877
C	-3.533623	0.080443	0.327487
C	-2.962486	-1.078440	-0.552429
C	-3.475232	-2.430062	-0.003022
C	-3.069651	1.469461	-0.141465
C	-1.646487	1.479768	-0.750398
C	-0.780853	0.349458	-0.228183
C	-1.427172	-1.043941	-0.358403
C	0.667640	0.267909	-0.770582
C	0.878471	-1.251209	-0.987434
C	-0.522254	-1.753109	-1.375039
C	-1.054075	2.881432	-0.622090
C	-0.114604	3.209816	0.522921
C	1.209133	2.424040	0.576354
C	1.034113	0.890888	0.623235
C	-3.407651	-0.939232	-2.017679
C	1.048153	1.011303	-2.046878
C	-5.934088	0.891920	0.156114
O	-6.859000	-1.518099	1.256499
O	-1.392640	3.741664	-1.414639
C	2.110798	0.198663	1.462352
C	3.479672	0.341501	0.784391
C	2.100650	0.655630	2.925865
C	4.544437	-0.599893	1.344424
C	5.936312	-0.461969	0.705258
C	6.906986	-1.439533	1.377967
C	5.918191	-0.581878	-0.839300
C	7.313564	-0.396515	-1.445308
C	5.282058	-1.880103	-1.347702
H	-3.097144	-0.082907	1.325991
H	-1.740844	1.324108	-1.829452
H	-1.233054	-1.508426	0.616641
O	-0.296025	0.585946	1.118231
H	-5.512248	-2.423608	-0.792474
H	-5.276624	-3.461724	0.613380
H	-4.954763	-1.489447	2.048240
H	-3.131314	-3.239259	-0.657834
H	-3.012334	-2.606832	0.977548
H	-3.124546	2.157100	0.709647

H	-3.741195	1.868672	-0.904873
H	1.634234	-1.452722	-1.752933
H	1.196506	-1.762554	-0.075259
H	-0.610310	-2.843056	-1.336853
H	-0.766495	-1.439948	-2.397965
H	-0.658495	3.014113	1.453692
H	0.094489	4.280355	0.460835
H	1.726726	2.759733	1.478656
H	1.855848	2.702868	-0.261582
H	-3.142468	-1.836389	-2.586319
H	-2.952643	-0.084712	-2.523383
H	-4.491510	-0.809779	-2.084452
H	0.753769	2.062136	-2.065810
H	0.581719	0.532662	-2.914668
H	2.133077	0.969560	-2.194103
H	-5.653659	1.852591	-0.259464
H	-6.996530	0.705235	0.272967
H	-7.156265	-0.874290	1.906244
H	1.852393	-0.865725	1.473140
H	3.829406	1.380529	0.867548
H	3.350763	0.143848	-0.286250
H	2.653956	-0.048550	3.553273
H	1.075970	0.704384	3.305673
H	2.565345	1.638988	3.056297
H	4.195153	-1.637263	1.242095
H	4.654098	-0.424869	2.421672
H	6.293286	0.555940	0.925351
H	7.938384	-1.286495	1.049225
H	6.886566	-1.307161	2.464752
H	6.637181	-2.481215	1.170862
H	5.305408	0.249973	-1.211140
H	7.248294	-0.280358	-2.531997
H	7.807479	0.494113	-1.041105
H	7.958245	-1.259255	-1.245782
H	5.291531	-1.905658	-2.442415
H	4.241068	-1.984363	-1.024858
H	5.831247	-2.760385	-0.995661

ωB97XD energy = -1358.18070027 a.u.

Swinhoeisterol F (**10**), Conf. D

C	-5.309811	-2.008472	0.070343
C	-5.652956	-0.770682	0.892822
C	-4.981911	0.447725	0.282297
C	-3.469877	0.318514	0.225428
C	-3.035325	-0.960336	-0.560543
C	-3.794074	-2.183691	0.004562
C	-2.755845	1.589909	-0.261978
C	-1.312839	1.343434	-0.767785
C	-0.677035	0.119898	-0.135955
C	-1.532035	-1.158368	-0.251324
C	0.773650	-0.222580	-0.556874
C	0.745565	-1.764370	-0.690495
C	-0.687143	-2.060218	-1.160672
C	-0.515120	2.640657	-0.652972
C	0.392332	2.875756	0.540500
C	1.563343	1.893720	0.727536
C	1.140659	0.412579	0.838291
C	-3.350895	-0.829214	-2.059590
C	1.327778	0.387501	-1.841372
C	-5.692913	1.485620	-0.161673
O	-7.059890	-0.684040	0.982557
O	-0.664862	3.504164	-1.498280
C	1.987784	-0.360873	1.865057
C	3.496251	-0.344034	1.575161
C	1.709105	0.146763	3.287118
C	3.930612	-1.179034	0.371882
C	5.447625	-1.262302	0.144007
C	5.730301	-2.177664	-1.053192
C	6.120321	0.127831	0.024396
C	7.636484	0.016213	-0.168891
C	5.505021	1.015766	-1.062661
H	-3.136065	0.140012	1.260219
H	-1.362437	1.151563	-1.843956
H	-1.481647	-1.590656	0.755938
O	-0.258091	0.353537	1.229690
H	-5.751046	-1.899098	-0.926496
H	-5.773075	-2.884489	0.534739
H	-5.226534	-0.911221	1.901918
H	-3.543256	-3.070426	-0.589422
H	-3.429515	-2.379639	1.022164
H	-2.753791	2.319045	0.555549
H	-3.300627	2.053666	-1.087359
H	1.510389	-2.127927	-1.382995
H	0.912746	-2.262809	0.267823
H	-0.950077	-3.118931	-1.076248
H	-0.808288	-1.775417	-2.213185
H	-0.236242	2.823295	1.436677
H	0.778919	3.893296	0.446286
H	2.072941	2.199463	1.645288
H	2.297442	2.015628	-0.075563
H	-3.201642	-1.786731	-2.568793
H	-2.726400	-0.089558	-2.565278
H	-4.391211	-0.528907	-2.212899
H	1.195840	1.467733	-1.922709
H	0.828760	-0.059737	-2.707831
H	2.397841	0.181381	-1.942941
H	-5.236178	2.366326	-0.597426
H	-6.777040	1.473057	-0.115941
H	-7.289413	0.032496	1.581676
H	1.646993	-1.401697	1.836782
H	4.014101	-0.725224	2.465649
H	3.828167	0.695391	1.461912
H	2.171908	1.122346	3.472541
H	2.127119	-0.552794	4.017714
H	0.635896	0.234011	3.471301
H	3.459920	-0.798987	-0.540069
H	3.546355	-2.199662	0.499288
H	5.887829	-1.738518	1.033555
H	6.799807	-2.355547	-1.193310
H	5.250619	-3.151490	-0.909446
H	5.334138	-1.752857	-1.982722
H	5.964412	0.633597	0.986373

H	8.114484	0.993002	-0.042537
H	8.080379	-0.672200	0.558977
H	7.889491	-0.344759	-1.171648
H	6.015636	1.983341	-1.100171
H	4.443452	1.212731	-0.881586
H	5.597327	0.558318	-2.054106

ωB97XD energy = -1358.18068677 a.u.

Swinhoeisterol F (**10**), Conf. E

C	-5.294977	-2.180643	0.307368
C	-5.596864	-1.016103	1.244589
C	-5.061153	0.274121	0.647796
C	-3.563680	0.233353	0.397824
C	-3.171441	-0.969879	-0.519272
C	-3.791452	-2.265460	0.053782
C	-2.982662	1.569869	-0.091945
C	-1.600904	1.436804	-0.777151
C	-0.833086	0.212687	-0.316484
C	-1.632534	-1.101680	-0.417951
C	0.563906	-0.015316	-0.945324
C	0.596085	-1.545094	-1.185324
C	-0.870622	-1.886870	-1.494609
C	-0.857471	2.766282	-0.667674
C	0.175566	2.970939	0.423853
C	1.410616	2.051617	0.384606
C	1.077437	0.544158	0.427815
C	-3.685539	-0.767366	-1.954593
C	0.951114	0.701149	-2.234863
C	-5.868906	1.298658	0.368447
O	-6.986411	-1.012753	1.499209
O	-1.147551	3.671730	-1.428332
C	2.121566	-0.270097	1.196364
C	3.458408	-0.265979	0.444306
C	2.250987	0.173670	2.658617
C	4.483642	-1.249865	1.010392
C	5.789561	-1.363891	0.199799
C	5.530463	-2.023356	-1.162698
C	6.537459	-0.018178	0.054185
C	6.754740	0.674761	1.403913
C	7.886213	-0.193351	-0.653701
H	-3.091155	0.014612	1.368906
H	-1.767981	1.308681	-1.851157
H	-1.431363	-1.598414	0.539488
O	-0.246071	0.375313	1.000564
H	-5.854993	-2.036101	-0.623083
H	-5.657512	-3.107123	0.763160
H	-5.051255	-1.195699	2.187891
H	-3.569821	-3.100240	-0.621401
H	-3.297038	-2.497438	1.006966
H	-2.921208	2.252793	0.762489
H	-3.648723	2.042965	-0.816965
H	1.281870	-1.817189	-1.993459
H	0.907219	-2.099365	-0.296154
H	-1.072179	-2.961819	-1.464103
H	-1.138034	-1.530112	-2.497162
H	-0.331160	2.809692	1.381859
H	0.491629	4.015317	0.367665
H	2.016246	2.316536	1.255189
H	2.028358	2.276383	-0.490443
H	-4.751664	-0.523963	-1.954599
H	-3.551619	-1.681198	-2.542214
H	-3.173542	0.040132	-2.482886
H	0.759180	1.775521	-2.233156
H	0.400829	0.278111	-3.082062
H	2.019127	0.557340	-2.432554
H	-5.510146	2.229588	-0.054296
H	-6.937440	1.222945	0.541096
H	-7.177987	-0.348897	2.168283
H	1.748987	-1.299807	1.221542
H	3.873237	0.750607	0.458340
H	3.271546	-0.508344	-0.609470
H	2.746622	-0.599310	3.252274
H	1.263762	0.346011	3.096941
H	2.841411	1.090731	2.762424
H	4.023529	-2.246271	1.060163
H	4.720009	-0.979607	2.045167
H	6.449816	-2.033963	0.771724
H	5.053822	-1.325411	-1.861200
H	6.455105	-2.373377	-1.628437
H	4.869768	-2.890274	-1.055430
H	5.918749	0.641652	-0.571813
H	7.336754	1.593038	1.275959
H	5.814361	0.949207	1.890526
H	7.309477	0.021564	2.088861
H	8.411762	0.764265	-0.724883
H	7.774755	-0.582340	-1.669300
H	8.528322	-0.885390	-0.094624

ωB97XD energy = -1358.18033768 a.u.

Swinhoeisterol F (**10**), Conf. F

C	4.368703	-2.973072	0.172247
C	4.772108	-2.271709	-1.120714
C	4.680981	-0.767127	-0.933953
C	3.284707	-0.304807	-0.557349
C	2.776913	-1.019730	0.737097
C	2.958424	-2.547364	0.575545
C	3.133818	1.224324	-0.493671
C	1.901904	1.697483	0.315965
C	0.794466	0.662192	0.351180
C	1.257656	-0.733308	0.814010
C	-0.490624	1.035070	1.132315
C	-0.848502	-0.281423	1.865632
C	0.521771	-0.917747	2.147302
C	1.491556	3.088537	-0.161958
C	0.375645	3.243028	-1.177437
C	-1.020685	2.767848	-0.732998
C	-1.076556	1.285420	-0.302028
C	3.561946	-0.557264	1.975419
C	-0.472686	2.179853	2.139503
C	5.751227	0.017395	-1.072431
O	6.052339	-2.741879	-1.488415
O	2.107836	4.059131	0.239042

C	-2.396086	0.606843	-0.677373
C	-3.560023	1.206711	0.123911
C	-2.645038	0.608707	-2.190157
C	-4.921259	0.547251	-0.135915
C	-4.989722	-0.972549	0.100143
C	-4.706897	-1.297494	1.571212
C	-6.339635	-1.556706	-0.383852
C	-6.510603	-1.421060	-1.902943
C	-6.510702	-3.029788	0.006999
H	2.613470	-0.659685	-1.355896
H	2.215929	1.849917	1.353268
H	0.783101	-1.411587	0.093802
O	0.051899	0.587023	-0.892344
H	5.104241	-2.728469	0.946368
H	4.409656	-4.055677	0.017464
H	4.038994	-2.553956	-1.896829
H	2.668422	-3.045699	1.507917
H	2.261083	-2.902616	-0.195357
H	3.085920	1.608586	-1.518519
H	4.009519	1.685481	-0.031676
H	-1.429833	-0.097174	2.774513
H	-1.431309	-0.962390	1.239915
H	0.447934	-1.965298	2.454467
H	1.033151	-0.374761	2.952242
H	0.660381	2.666667	-2.064666
H	0.335461	4.301490	-1.445255
H	-1.684284	2.924209	-1.587441
H	-1.404898	3.408419	0.066782
H	3.296599	-1.163248	2.847765
H	3.376096	0.487509	2.233251
H	4.638264	-0.660246	1.812521
H	-0.020848	3.101740	1.769348
H	0.085682	1.886729	3.035023
H	-1.496329	2.414452	2.451747
H	5.710216	1.092770	-0.945521
H	6.722575	-0.409056	-1.300446
H	6.279852	-2.381542	-2.350692
H	-2.281634	-0.442298	-0.388728
H	-3.652971	2.273717	-0.117751
H	-3.317525	1.160255	1.192957
H	-3.400714	-0.136654	-2.455484
H	-1.726612	0.359449	-2.729256
H	-3.007993	1.577318	-2.551450
H	-5.215231	0.774007	-1.164840
H	-5.670848	1.030060	0.506259
H	-4.208912	-1.456598	-0.506473
H	-4.593652	-2.371185	1.739586
H	-3.783252	-0.822538	1.912564
H	-5.522027	-0.937357	2.211553
H	-7.142785	-0.983606	0.104787
H	-7.455833	-1.868496	-2.226239
H	-6.511805	-0.381775	-2.240792
H	-5.700240	-1.944196	-2.426386
H	-7.426338	-3.439468	-0.431177
H	-6.575070	-3.169353	1.089140
H	-5.669045	-3.629114	-0.362831

ωB97XD energy = -1358.18027563 a.u.

Swinhoeisterol F (**10**), Conf. G

C	-5.425589	-1.879028	0.133886
C	-5.698045	-0.634672	0.972754
C	-4.990180	0.559708	0.356538
C	-3.486587	0.367495	0.263074
C	-3.126079	-0.920153	-0.545915
C	-3.920506	-2.115580	0.029552
C	-2.729960	1.613308	-0.226728
C	-1.312254	1.311194	-0.771889
C	-0.712832	0.053239	-0.172621
C	-1.624951	-1.185402	-0.279882
C	0.708793	-0.343052	-0.641347
C	0.611292	-1.880273	-0.791972
C	-0.845695	-2.111327	-1.223188
C	-0.454351	2.570097	-0.668011
C	0.491873	2.752973	0.504113
C	1.622545	1.719822	0.661179
C	1.143469	0.256069	0.753993
C	-3.474519	-0.760505	-2.034849
C	1.235338	0.254875	-1.943205
C	-5.666767	1.630633	-0.061945
O	-7.097754	-0.489929	1.094855
O	-0.587218	3.447596	-1.501520
C	1.991176	-0.568190	1.741938
C	3.504098	-0.503236	1.488901
C	1.676915	-0.165339	3.189130
C	3.950601	-0.992098	0.113308
C	5.465789	-1.181404	-0.056544
C	5.754634	-1.709764	-1.466404
C	6.278486	0.086039	0.304796
C	7.788593	-0.151268	0.197643
C	5.872332	1.323942	-0.502241
H	-3.136754	0.163277	1.287835
H	-1.399404	1.134843	-1.848186
H	-1.565786	-1.632357	0.720400
O	-0.245388	0.246542	1.182847
H	-5.885690	-1.744485	-0.851297
H	-5.912782	-2.739101	0.603637
H	-5.255675	-0.800714	1.971093
H	-3.720634	-3.007494	-0.575694
H	-3.539578	-2.333186	1.036709
H	-2.676698	2.330963	0.599237
H	-3.274404	2.110685	-1.032612
H	1.340778	-2.265942	-1.510580
H	0.782431	-2.397862	0.155496
H	-1.150574	-3.158942	-1.141042
H	-0.985732	-1.810581	-2.268710
H	-0.118342	2.719923	1.413977
H	0.920323	3.753652	0.410322
H	2.151966	1.989331	1.579434
H	2.355340	1.822018	-0.146331
H	-3.385029	-1.719477	-2.555234
H	-2.827389	-0.047710	-2.550716
H	-4.502051	-0.407368	-2.158946

H	0.617885	-0.089471	-2.780199
H	2.257566	-0.079083	-2.140045
H	1.231405	1.345788	-1.975573
H	-5.183632	2.495482	-0.501021
H	-6.749039	1.663442	0.009927
H	-7.283545	0.229237	1.705839
H	1.682629	-1.613833	1.636665
H	3.993747	-1.115104	2.258639
H	3.854977	0.522583	1.657685
H	2.196412	-0.835486	3.881231
H	0.605199	-0.224759	3.388365
H	2.010406	0.854447	3.411696
H	3.597780	-0.295702	-0.654630
H	3.459968	-1.950857	-0.099850
H	5.779724	-1.958212	0.657545
H	5.187970	-2.628062	-1.652917
H	5.461600	-0.982949	-2.232620
H	6.813000	-1.942701	-1.611446
H	6.068560	0.299616	1.361266
H	8.341617	0.683503	0.640197
H	8.085877	-1.066978	0.720832
H	8.109993	-0.240360	-0.845724
H	6.464959	2.191597	-0.194708
H	4.817667	1.580366	-0.359111
H	6.040132	1.175753	-1.574675

ωB97XD energy = -1358.18022925 a.u.

Swinhoeisterol F (**10**), Conf. H

C	-5.196479	-2.098900	-0.084114
C	-5.594795	-0.894555	0.762684
C	-4.940721	0.357566	0.204763
C	-3.424908	0.270347	0.191376
C	-2.932192	-0.974262	-0.615857
C	-3.675230	-2.233289	-0.110324
C	-2.732207	1.573763	-0.238270
C	-1.267164	1.379910	-0.699739
C	-0.620418	0.155576	-0.080260
C	-1.433930	-1.141259	-0.264869
C	0.852619	-0.130544	-0.461160
C	0.874764	-1.667628	-0.643363
C	-0.533142	-1.990996	-1.170787
C	-0.508220	2.692541	-0.520074
C	0.334129	2.918819	0.721350
C	1.527220	1.968168	0.932681
C	1.151256	0.471533	0.969724
C	-3.205153	-0.808261	-2.119759
C	1.414287	0.529585	-1.717608
C	-5.666766	1.386839	-0.234946
O	-7.005444	-0.847598	0.816002
O	-0.638497	3.571680	-1.352318
C	1.987402	-0.311100	1.999488
C	3.506048	-0.147564	1.845961
C	1.557387	0.043300	3.429206
C	4.064179	-0.519922	0.474150
C	5.598804	-0.531063	0.404220
C	6.181182	-1.777717	1.079295
C	6.129898	-0.367603	-1.040953
C	5.553450	-1.397499	-2.019402
C	5.913605	1.052413	-1.576008
H	-3.118034	0.071714	1.230843
H	-1.274429	1.217214	-1.781656
H	-1.404270	-1.602880	0.730035
O	-0.257753	0.354349	1.305472
H	-5.611956	-1.971776	-1.089889
H	-5.649405	-2.999380	0.342079
H	-5.191960	-1.052278	1.778846
H	-3.384195	-3.094419	-0.723361
H	-3.335605	-2.451528	0.911354
H	-2.778364	2.280414	0.597614
H	-3.262088	2.044189	-1.069616
H	1.671976	-1.986110	-1.321872
H	1.024460	-2.192497	0.303704
H	-0.766811	-3.058872	-1.125319
H	-0.627771	-1.679343	-2.218258
H	-0.333194	2.816236	1.584444
H	0.690288	3.950786	0.677773
H	1.977934	2.253976	1.887235
H	2.298184	2.143473	0.174900
H	-3.003767	-1.743561	-2.651809
H	-2.593769	-0.030520	-2.582378
H	-4.250942	-0.542182	-2.297105
H	1.331216	1.617717	-1.733173
H	0.877819	0.158113	-2.597567
H	2.468596	0.275377	-1.856082
H	-5.221808	2.290174	-0.635115
H	-6.751008	1.343347	-0.224206
H	-7.270100	-0.150427	1.423472
H	1.755448	-1.372412	1.860059
H	3.975874	-0.767925	2.618833
H	3.793779	0.887146	2.078676
H	1.796901	1.081957	3.683267
H	2.085017	-0.596476	4.143655
H	0.483625	-0.100921	3.563702
H	3.680188	0.196889	-0.255781
H	3.677646	-1.503108	0.173357
H	5.960535	0.343973	0.965185
H	5.848931	-2.693435	0.577451
H	7.275894	-1.761048	1.057792
H	5.874287	-1.849877	2.127144
H	7.215680	-0.530718	-0.990205
H	6.035465	-1.307216	-2.998075
H	5.700098	-2.424480	-1.671060
H	4.478477	-1.243385	-2.169344
H	4.849298	1.276945	-1.712793
H	6.397109	1.178263	-2.550208
H	6.328863	1.802246	-0.894207

ωB97XD energy = -1358.17986340 a.u.

Swinhoeisterol F (**10**), Conf. I

C	4.940020	-2.658471	0.032163
C	5.338919	-1.755349	-1.130086
C	5.021465	-0.309663	-0.787161

C	3.550829	-0.082162	-0.482781
C	3.054843	-1.016299	0.667671
C	3.456845	-2.473698	0.344613
C	3.189095	1.393741	-0.248035
C	1.850118	1.597805	0.501826
C	0.893459	0.435716	0.315738
C	1.509432	-0.939289	0.643177
C	-0.481071	0.536410	1.022730
C	-0.708638	-0.900262	1.555487
C	0.712955	-1.390269	1.875294
C	1.290762	2.978127	0.163431
C	0.230974	3.126124	-0.911331
C	-1.113915	2.421746	-0.650647
C	-0.994583	0.900283	-0.415065
C	3.677834	-0.617595	2.016060
C	-0.688204	1.529857	2.161071
C	5.975048	0.622667	-0.751627
O	6.697571	-2.003631	-1.426600
O	1.748483	3.956547	0.725493
C	-2.182422	0.116788	-0.979686
C	-3.460969	0.429355	-0.191723
C	-2.347102	0.306944	-2.492199
C	-4.645233	-0.461752	-0.576236
C	-5.814685	-0.403587	0.417692
C	-6.413001	1.005878	0.495503
C	-6.902159	-1.472302	0.147179
C	-7.560705	-1.344809	-1.230902
C	-6.380430	-2.897800	0.358577
H	2.994341	-0.412321	-1.374666
H	2.067320	1.652323	1.573091
H	1.184953	-1.571518	-0.192962
O	0.255961	0.434641	-0.987449
H	5.569913	-2.417313	0.895650
H	5.144791	-3.699093	-0.237654
H	4.715972	-2.033240	-1.998888
H	3.164103	-3.122580	1.178380
H	2.879685	-2.810860	-0.527351
H	3.163054	1.900185	-1.219084
H	3.959195	1.895819	0.341862
H	-1.373508	-0.912052	2.424569
H	-1.147716	-1.559570	0.802250
H	0.763679	-2.469925	2.045356
H	1.088178	-0.897494	2.780944
H	0.650690	2.715507	-1.836442
H	0.065608	4.197268	-1.048519
H	-1.732386	2.607494	-1.532354
H	-1.636410	2.893166	0.187549
H	3.481204	-1.383857	2.772805
H	3.292062	0.328285	2.402827
H	4.762017	-0.507578	1.924490
H	-0.341093	2.542700	1.949340
H	-0.159953	1.191084	3.058681
H	-1.753524	1.592182	2.409434
H	5.771308	1.660771	-0.517226
H	7.011167	0.364091	-0.943712
H	6.936281	-1.518243	-2.221871
H	-1.947430	-0.942468	-0.828968
H	-3.723801	1.485668	-0.332844
H	-3.257741	0.298673	0.880455
H	-2.813809	1.266350	-2.740526
H	-2.983516	-0.478853	-2.908702
H	-1.376800	0.251898	-2.994117
H	-4.283377	-1.495190	-0.652073
H	-5.003055	-0.184044	-1.575602
H	-5.403486	-0.636398	1.412281
H	-7.294403	1.023529	1.145482
H	-5.692513	1.723656	0.898711
H	-6.716832	1.367779	-0.493432
H	-7.683630	-1.302143	0.901702
H	-8.372580	-2.072316	-1.333290
H	-7.986705	-0.350281	-1.393839
H	-6.842527	-1.542567	-2.034408
H	-7.203936	-3.618712	0.328975
H	-5.880462	-2.999984	1.328042
H	-5.667336	-3.189095	-0.420310

ωB97XD energy = -1358.17975387 a.u.

Swinhoeisterol F (**10**), Conf. J

C	5.038636	-2.631135	0.003463
C	5.439192	-1.689005	-1.126740
C	5.094861	-0.258550	-0.747968
C	3.617161	-0.062274	-0.456466
C	3.120722	-1.035993	0.660500
C	3.548955	-2.477378	0.301622
C	3.230140	1.400730	-0.184308
C	1.876777	1.563296	0.549943
C	0.942062	0.391391	0.318568
C	1.574736	-0.981743	0.619164
C	-0.443912	0.452325	1.007797
C	-0.655163	-1.000623	1.501753
C	0.770281	-1.474091	1.829944
C	1.298734	2.942544	0.239782
C	0.254215	3.100380	-0.848543
C	-1.083637	2.369435	-0.626687
C	-0.942482	0.844592	-0.427870
C	3.720679	-0.665784	2.027235
C	-0.681516	1.414227	2.167229
C	6.033235	0.686975	-0.674673
O	6.805396	-1.908167	-1.411224
O	1.729519	3.913268	0.835419
C	-2.107618	0.056338	-1.031875
C	-3.407025	0.338491	-0.266287
C	-2.241274	0.274115	-2.543890
C	-4.566923	-0.570535	-0.678728
C	-5.786086	-0.499593	0.253521
C	-6.417586	0.897518	0.238899
C	-6.797552	-1.631909	-0.049466
C	-7.839886	-1.789051	1.062624
C	-7.490263	-1.488480	-1.409274
H	3.077038	-0.374677	-1.364694
H	2.077452	1.591675	1.625491
H	1.269482	-1.597619	-0.236233

O	0.323725	0.414635	-0.993363
H	5.653648	-2.406439	0.881949
H	5.262262	-3.660291	-0.293984
H	4.832632	-1.951325	-2.011850
H	3.255524	-3.154468	1.112308
H	2.988215	-2.797558	-0.587291
H	3.212207	1.935247	-1.140408
H	3.983461	1.895640	0.432697
H	-1.331979	-1.044455	2.360493
H	-1.072138	-1.648842	0.726626
H	0.837681	-2.556186	1.977246
H	1.124946	-0.994849	2.751033
H	0.694414	2.717007	-1.775757
H	0.074847	4.171862	-0.963740
H	-1.691040	2.567651	-1.513431
H	-1.626580	2.810990	0.214681
H	3.519735	-1.451687	2.762376
H	3.321361	0.267371	2.430813
H	4.805054	-0.544510	1.954276
H	-0.340956	2.435218	1.985967
H	-0.164253	1.058497	3.064652
H	-1.751312	1.459247	2.399448
H	5.810690	1.715124	-0.414596
H	7.075500	0.449414	-0.860707
H	7.047574	-1.395103	-2.187848
H	-1.861778	-1.002347	-0.895895
H	-3.686730	1.390471	-0.407227
H	-3.222784	0.205164	0.809074
H	-2.721645	1.229012	-2.783646
H	-2.850718	-0.515701	-2.991772
H	-1.258905	0.249282	-3.024143
H	-4.203521	-1.607928	-0.705182
H	-4.875518	-0.324951	-1.702527
H	-5.418279	-0.686019	1.274414
H	-7.322656	0.941527	0.851641
H	-5.724267	1.646395	0.632531
H	-6.684705	1.199309	-0.780354
H	-6.212772	-2.562899	-0.074693
H	-8.444040	-2.687407	0.899022
H	-7.364479	-1.878561	2.045435
H	-8.527506	-0.936852	1.095902
H	-8.128078	-2.356442	-1.605850
H	-6.772524	-1.414553	-2.231785
H	-8.130462	-0.599607	-1.436735

ωB97XD energy = -1358.17965149 a.u.

Swinhoeisterol F (**10**), Conf. K

C	-4.983116	-2.326093	0.476440
C	-5.486591	-1.005291	1.048915
C	-4.952153	0.147654	0.216595
C	-3.434421	0.192553	0.182829
C	-2.837620	-1.149990	-0.350199
C	-3.456430	-2.326862	0.439613
C	-2.864789	1.428376	-0.533287
C	-1.395725	1.264524	-0.994253
C	-0.630658	0.263678	-0.149942
C	-1.326400	-1.106461	-0.019984
C	0.856109	0.023417	-0.509162
C	1.012697	-1.508789	-0.354369
C	-0.367553	-2.052383	-0.755036
C	-0.757890	2.646328	-1.127243
C	0.100164	3.203125	-0.005769
C	1.375938	2.418474	0.352180
C	1.124781	0.945303	0.740895
C	-3.143049	-1.341404	-1.845146
C	1.355127	0.449338	-1.886987
C	-5.772336	0.988486	-0.415984
O	-6.895292	-1.073546	1.124619
O	-0.998948	3.315924	-2.115241
C	2.035392	0.482751	1.892062
C	3.540654	0.609156	1.609987
C	1.670132	1.213566	3.192408
C	4.088179	-0.379757	0.580631
C	5.595760	-0.251091	0.315634
C	6.420837	-0.595842	1.561649
C	6.058831	-1.071308	-0.914078
C	5.780965	-2.573415	-0.788757
C	5.480148	-0.535349	-2.228189
H	-3.103167	0.250734	1.232176
H	-1.405260	0.871566	-2.015488
H	-1.239595	-1.335291	1.049643
O	-0.264335	0.796261	1.144640
H	-5.415133	-2.462179	-0.521278
H	-5.347705	-3.146935	1.101750
H	-5.066947	-0.899518	2.065089
H	-3.090226	-3.272372	0.022840
H	-3.091098	-2.282092	1.474707
H	-2.958516	2.287715	0.139648
H	-3.446589	1.669636	-1.425690
H	1.826846	-1.900833	-0.971527
H	1.221785	-1.797419	0.678909
H	-0.507971	-3.102570	-0.482460
H	-0.504063	-1.974723	-1.840897
H	-0.530368	3.241881	0.889749
H	0.362760	4.225463	-0.288066
H	1.832302	2.946416	1.193814
H	2.104116	2.472123	-0.463321
H	-2.879790	-2.354684	-2.165521
H	-2.598084	-0.644577	-2.485711
H	-4.208204	-1.194643	-2.044625
H	1.088263	1.468861	-2.169886
H	0.939320	-0.214188	-2.652897
H	2.444767	0.371497	-1.947666
H	-5.415064	1.819802	-1.012223
H	-6.847817	0.852728	-0.367015
H	-7.220797	-0.287127	1.572643
H	1.815421	-0.576832	2.063495
H	4.058868	0.458740	2.564187
H	3.778471	1.636350	1.299948
H	1.999618	2.258426	3.183529
H	2.162268	0.727638	4.040665
H	0.591663	1.193688	3.365118

H	3.552449	-0.246199	-0.362009
H	3.863805	-1.400533	0.918569
H	5.796638	0.803494	0.071909
H	7.492067	-0.602012	1.333760
H	6.262744	0.131582	2.362911
H	6.155951	-1.583240	1.956624
H	7.148948	-0.941212	-0.969442
H	6.206708	-3.111980	-1.641568
H	6.215074	-2.998770	0.121048
H	4.704082	-2.776958	-0.777162
H	5.971878	-1.005712	-3.085935
H	5.617062	0.548064	-2.315723
H	4.409241	-0.749084	-2.317483

ωB97XD energy = -1358.17956162 a.u.

Swinhoeisterol F (**10**), Conf. L

C	4.528954	-2.768879	-0.082180
C	5.052511	-1.784323	-1.122676
C	4.791790	-0.361293	-0.659624
C	3.318263	-0.077551	-0.425206
C	2.701706	-1.079391	0.602577
C	3.042065	-2.523033	0.165197
C	3.017637	1.390154	-0.081276
C	1.649454	1.605026	0.610444
C	0.640918	0.524071	0.267545
C	1.167294	-0.907868	0.499040
C	-0.765848	0.640566	0.907559
C	-1.093321	-0.826755	1.272333
C	0.274722	-1.421170	1.636730
C	1.194327	3.041461	0.369153
C	0.220096	3.341491	-0.751558
C	-1.178614	2.715191	-0.607698
C	-1.179237	1.170763	-0.515863
C	3.261281	-0.844384	2.015406
C	-0.964723	1.529463	2.130797
C	5.789866	0.503867	-0.471818
O	6.412565	-2.085074	-1.358294
O	1.661349	3.936499	1.049847
C	-2.363833	0.548532	-1.278349
C	-3.740194	1.051972	-0.804704
C	-2.203420	0.764264	-2.790083
C	-4.285078	0.437716	0.489191
C	-4.589354	-1.071421	0.461327
C	-5.132359	-1.497906	1.829868
C	-5.488800	-1.498644	-0.722743
C	-5.698486	-3.016330	-0.759847
C	-6.835919	-0.770332	-0.767834
H	2.805248	-0.296643	-1.375514
H	1.806419	1.547319	1.691933
H	0.861455	-1.441960	-0.409367
O	0.074980	0.671887	-1.057791
H	5.116457	-2.654373	0.835375
H	4.690208	-3.788607	-0.445191
H	4.475580	-1.940385	-2.051384
H	2.664715	-3.227117	0.916017
H	2.501158	-2.742428	-0.765569
H	3.075478	1.977926	-1.004027
H	3.775030	1.796587	0.592680
H	-1.830842	-0.898795	2.077558
H	-1.488923	-1.378016	0.416139
H	0.257567	-2.512634	1.711084
H	0.615401	-1.031022	2.604091
H	0.662218	2.968033	-1.681590
H	0.130523	4.428421	-0.816205
H	-1.744834	3.030150	-1.488289
H	-1.699501	3.144720	0.253665
H	2.951281	-1.647908	2.691225
H	2.928482	0.098558	2.454871
H	4.354494	-0.822753	2.001301
H	-0.530130	2.526346	2.037910
H	-0.508685	1.063863	3.010888
H	-2.031428	1.655517	2.342916
H	5.627681	1.524594	-0.146492
H	6.820766	0.202007	-0.624902
H	6.728296	-1.545790	-2.089401
H	-2.312271	-0.533380	-1.120753
H	-4.459580	0.875241	-1.612775
H	-3.709803	2.141864	-0.687777
H	-2.920756	0.138058	-3.329993
H	-1.197029	0.497405	-3.121432
H	-2.398179	1.802358	-3.081178
H	-5.202338	0.974601	0.764934
H	-3.581533	0.622671	1.308833
H	-3.640210	-1.604136	0.318826
H	-5.267792	-2.580510	1.901016
H	-4.436288	-1.201201	2.621964
H	-6.094723	-1.021087	2.046975
H	-4.942696	-1.238190	-1.639723
H	-6.162776	-3.318315	-1.704181
H	-4.746960	-3.551619	-0.666035
H	-6.355990	-3.352518	0.049148
H	-7.421149	-1.103368	-1.631178
H	-6.715495	0.314277	-0.850356
H	-7.429877	-0.975797	0.129615

ωB97XD energy = -1358.17939029 a.u.

Swinhoeisterol F (**10**), Conf. M

C	-4.059859	0.898103	1.256860
C	-4.181292	0.929239	-0.263417
C	-3.623025	-0.355862	-0.849878
C	-2.165755	-0.583545	-0.489841
C	-1.955343	-0.583168	1.058211
C	-2.602007	0.686459	1.658868
C	-1.540369	-1.809138	-1.174208
C	-0.243671	-2.311035	-0.492105
C	0.473066	-1.218159	0.277050
C	-0.428041	-0.479702	1.285598
C	1.789315	-1.610754	0.994688
C	1.667598	-0.909584	2.374422
C	0.154649	-0.898351	2.640881
C	0.603377	-3.063159	-1.514899
C	1.736681	-2.348038	-2.224706

C	2.892611	-1.828251	-1.348365
C	2.462839	-0.864415	-0.215046
C	-2.598757	-1.820594	1.705524
C	2.121484	-3.081396	1.231440
C	-4.380217	-1.174500	-1.582348
O	-5.533176	1.183080	-0.584897
O	0.318056	-4.213159	-1.794445
C	3.563815	0.194202	0.033445
C	3.031688	1.583252	0.429500
C	4.617446	-0.354190	1.004777
C	2.651496	2.433658	-0.785441
C	1.891492	3.726269	-0.447460
C	1.934761	4.684235	-1.643909
C	0.451145	3.458912	0.058583
C	-0.163791	4.696608	0.720107
C	-0.483009	2.912637	-1.024558
H	-1.617243	0.299582	-0.849428
H	-0.521530	-3.072936	0.242728
H	-0.169501	0.575973	1.136707
O	1.181616	-0.286637	-0.578079
H	-4.707172	0.102533	1.642410
H	-4.433134	1.843333	1.663170
H	-3.553956	1.759014	-0.633073
H	-2.514260	0.652074	2.751200
H	-2.025024	1.561772	1.331069
H	-1.346469	-1.557309	-2.222745
H	-2.237467	-2.650196	-1.183142
H	2.229628	-1.443828	3.146551
H	2.037182	0.115471	2.362705
H	-0.128524	-0.223893	3.454542
H	-0.188041	-1.904380	2.913680
H	1.296201	-1.487144	-2.740017
H	2.127618	-3.039361	-2.975069
H	3.568521	-1.294413	-2.023240
H	3.470704	-2.663661	-0.940732
H	-2.600975	-1.727442	2.796316
H	-2.082482	-2.750843	1.457813
H	-3.635883	-1.934136	1.377849
H	1.460286	-3.494911	2.000644
H	3.149659	-3.173223	1.598261
H	2.025647	-3.720386	0.352094
H	-4.000005	-2.092077	-2.015705
H	-5.428055	-0.953567	-1.757045
H	-5.607680	1.308745	-1.535544
H	4.078754	0.340348	-0.925572
H	3.802340	2.109869	1.008001
H	2.164222	1.487631	1.083766
H	4.225878	-0.453137	2.020864
H	5.480275	0.317470	1.044404
H	4.978936	-1.338416	0.687155
H	2.054086	1.826204	-1.474328
H	3.572058	2.696088	-1.324858
H	2.425668	4.212460	0.383723
H	1.370239	5.603254	-1.459749
H	2.968414	4.970389	-1.863327
H	1.527595	4.212034	-2.544724
H	0.524687	2.689780	0.839112
H	-1.124399	4.450455	1.185563
H	0.493307	5.099608	1.498335
H	-0.350790	5.492891	-0.008872
H	-1.466895	2.695025	-0.595594
H	-0.098921	1.986243	-1.461823
H	-0.635438	3.642335	-1.827355

ωB97XD energy = -1358.17926740 a.u.

Swinhoeisterol F (**10**), Conf. N

C	-5.198512	-2.177962	0.206584
C	-5.631163	-0.881772	0.883445
C	-5.010158	0.301139	0.160814
C	-3.492590	0.253565	0.148764
C	-2.967611	-1.075562	-0.484823
C	-3.673988	-2.272504	0.193435
C	-2.839132	1.505011	-0.460249
C	-1.370265	1.290968	-0.900290
C	-0.687184	0.180479	-0.123811
C	-1.464264	-1.151240	-0.125579
C	0.792733	-0.114509	-0.469778
C	0.856295	-1.660585	-0.442379
C	-0.542654	-2.088877	-0.916658
C	-0.646398	2.635316	-0.905460
C	0.189184	3.050324	0.290494
C	1.411463	2.172527	0.618592
C	1.077465	0.685380	0.863459
C	-3.251604	-1.124495	-1.995302
C	1.336441	0.382970	-1.806344
C	-5.765250	1.243230	-0.406958
O	-7.042566	-0.866122	0.932261
O	-0.799092	3.388416	-1.849925
C	1.936050	0.075919	1.987166
C	3.449732	0.254878	1.805251
C	1.496084	0.613702	3.355355
C	4.014203	-0.292156	0.495640
C	5.549365	-0.324220	0.433030
C	6.116887	-1.477966	1.267389
C	6.046061	-0.323375	-1.033140
C	7.568643	-0.190634	-1.131641
C	5.561159	-1.530031	-1.844700
H	-3.175686	0.206919	1.203164
H	-1.375148	0.982555	-1.950033
H	-1.417729	-1.473810	0.922289
O	-0.327702	0.577094	1.219711
H	-5.616932	-2.200673	-0.805647
H	-5.626337	-3.025022	0.751663
H	-5.226376	-0.890268	1.911004
H	-3.359750	-3.201427	-0.296608
H	-3.327969	-2.339529	1.233988
H	-2.903612	2.316568	0.272712
H	-3.385735	1.841209	-1.344094
H	1.662160	-2.046433	-1.074264
H	1.018699	-2.050552	0.565961
H	-0.747063	-3.147446	-0.730513
H	-0.647730	-1.919647	-1.995371

H	-0.472493	3.040398	1.163960
H	0.512073	4.078283	0.109657
H	1.858447	2.599903	1.520488
H	2.172438	2.261962	-0.163634
H	-2.661096	-0.404761	-2.566480
H	-4.304187	-0.908904	-2.198903
H	-3.031223	-2.119084	-2.396245
H	1.220654	1.455440	-1.971323
H	0.812919	-0.121932	-2.625744
H	2.398309	0.143601	-1.909909
H	-5.346309	2.098866	-0.923305
H	-6.847760	1.171166	-0.383627
H	-7.327401	-0.102989	1.443652
H	1.731973	-1.000439	1.997729
H	3.934957	-0.242965	2.653744
H	3.713362	1.317740	1.895030
H	2.034396	0.088755	4.151012
H	0.425253	0.466796	3.508802
H	1.713324	1.681956	3.466606
H	3.647929	0.329093	-0.328442
H	3.617003	-1.301347	0.322800
H	5.913436	0.614832	0.877588
H	7.209343	-1.449661	1.310567
H	5.752320	-1.432912	2.298129
H	5.818324	-2.448761	0.856734
H	5.611871	0.575532	-1.494983
H	7.874506	-0.016487	-2.168326
H	7.935853	0.645905	-0.526973
H	8.073893	-1.100834	-0.790468
H	5.877829	-1.438861	-2.888751
H	4.469996	-1.619082	-1.837289
H	5.978196	-2.465876	-1.456551

ωB97XD energy = -1358.17924268 a.u.

Swinhoeisterol F (**10**), Conf. O

C	-4.426330	-2.774874	0.101731
C	-4.959958	-1.785813	1.132878
C	-4.723363	-0.365158	0.650147
C	-3.254947	-0.061678	0.408508
C	-2.626567	-1.066329	-0.609931
C	-2.944349	-2.509916	-0.155512
C	-2.977181	1.407182	0.048985
C	-1.615859	1.634006	-0.651758
C	-0.591221	0.571623	-0.301574
C	-1.094139	-0.871223	-0.513144
C	0.808378	0.703652	-0.953016
C	1.163006	-0.761104	-1.308121
C	-0.198586	-1.385454	-1.648584
C	-1.177194	3.079627	-0.431393
C	-0.199385	3.409405	0.678649
C	1.206495	2.797246	0.538964
C	1.219564	1.253798	0.458268
C	-3.193762	-0.854657	-2.023436
C	1.004127	1.582112	-2.183813
C	-5.735812	0.480869	0.452296
O	-6.314111	-2.104554	1.378751
O	-1.661683	3.959133	-1.120101
C	2.435108	0.635787	1.158986
C	3.722278	1.027534	0.418882
C	2.467706	0.971826	2.653838
C	5.043816	0.435797	0.932774
C	5.267471	-1.090436	0.815554
C	4.583541	-1.879141	1.942486
C	4.928452	-1.650043	-0.583832
C	5.707335	-0.931344	-1.691778
C	5.184874	-3.158416	-0.679466
H	-2.736362	-0.262946	1.359714
H	-1.777016	1.561136	-1.731740
H	-0.777350	-1.389132	0.400791
O	-0.020002	0.746017	1.020666
H	-5.019929	-2.680307	-0.814175
H	-4.570705	-3.792427	0.477809
H	-4.376294	-1.921231	2.060566
H	-2.560409	-3.216368	-0.900784
H	-2.396122	-2.711714	0.774917
H	-3.038401	2.002670	0.966530
H	-3.743432	1.796872	-0.624768
H	1.885742	-0.820653	-2.127906
H	1.589410	-1.299758	-0.457873
H	-0.162979	-2.477416	-1.707189
H	-0.552767	-1.015438	-2.619021
H	-0.633378	3.046182	1.616568
H	-0.123448	4.498307	0.725045
H	1.770390	3.121349	1.417458
H	1.723103	3.225222	-0.325562
H	-4.287112	-0.847960	-2.005730
H	-2.875155	-1.661159	-2.691650
H	-2.875285	0.088025	-2.474012
H	0.564352	2.577886	-2.102080
H	0.556638	1.106677	-3.063123
H	2.073752	1.707443	-2.385036
H	-5.590937	1.499221	0.111616
H	-6.761235	0.163862	0.611479
H	-6.635197	-1.559942	2.103552
H	2.301814	-0.445751	1.084496
H	3.824577	2.120332	0.461036
H	3.601675	0.780206	-0.643484
H	3.128887	0.285945	3.188822
H	1.469168	0.878406	3.090334
H	2.835204	1.986673	2.841608
H	5.186103	0.724117	1.981683
H	5.848947	0.939435	0.385005
H	6.350293	-1.237330	0.953630
H	3.504156	-1.976929	1.786219
H	4.994875	-2.888616	2.027083
H	4.739359	-1.387723	2.907921
H	3.856519	-1.487210	-0.759777
H	5.471241	-1.360690	-2.670807
H	5.486008	0.138514	-1.739356
H	6.787347	-1.041674	-1.533445
H	5.007337	-3.514490	-1.699462
H	4.536043	-3.734005	-0.014151

H	6.225964	-3.391643	-0.422803

ωB97XD energy = -1358.17913501 a.u.

Swinhoeisterol F (**10**), Conf. P

C	4.485157	-2.746202	-0.213343
C	5.002966	-1.722142	-1.218195
C	4.758205	-0.319164	-0.689858
C	3.288750	-0.036085	-0.430462
C	2.676812	-1.074940	0.562905
C	3.003114	-2.501503	0.062825
C	2.998902	1.418600	-0.026824
C	1.640423	1.610661	0.689917
C	0.621548	0.550681	0.314280
C	1.142112	-0.891641	0.483388
C	-0.775863	0.651306	0.977211
C	-1.108968	-0.828368	1.288967
C	0.260465	-1.444489	1.610680
C	1.189441	3.058230	0.520165
C	0.210889	3.412421	-0.580134
C	-1.190125	2.786915	-0.455756
C	-1.200198	1.240902	-0.415842
C	3.253142	-0.900384	1.977814
C	-0.971189	1.490467	2.234928
C	5.764947	0.528189	-0.470033
O	6.357895	-2.021499	-1.483193
O	1.662704	3.918526	1.240195
C	-2.424145	0.654962	-1.132368
C	-3.727299	1.159836	-0.485344
C	-2.369811	0.923991	-2.641389
C	-5.021578	0.466693	-0.957663
C	-5.602522	-0.588374	0.000755
C	-6.941451	-1.098192	-0.542806
C	-4.608948	-1.723317	0.335224
C	-5.090215	-2.589600	1.503551
C	-4.246279	-2.602834	-0.865443
H	2.765921	-0.215316	-1.383737
H	1.809655	1.503155	1.765770
H	0.822732	-1.388581	-0.441167
O	0.037752	0.751841	-0.998474
H	5.085490	-2.674760	0.700267
H	4.634438	-3.750641	-0.621471
H	4.413546	-1.833913	-2.145402
H	2.630648	-3.232994	0.789504
H	2.449684	-2.680083	-0.869221
H	3.047330	2.041578	-0.926801
H	3.765773	1.796646	0.652900
H	-1.832010	-0.925113	2.104827
H	-1.521989	-1.347820	0.420414
H	0.237335	-2.537978	1.641265
H	0.615237	-1.095418	2.588583
H	0.646491	3.078829	-1.528203
H	0.126272	4.501567	-0.596237
H	-1.754001	3.131293	-1.326683
H	-1.711562	3.189609	0.418216
H	2.952350	-1.733137	2.621696
H	2.924168	0.021950	2.461699
H	4.346051	-0.876127	1.951337
H	-0.546693	2.494453	2.175997
H	-0.506860	0.997400	3.095612
H	-2.039938	1.594576	2.451929
H	5.613967	1.533329	-0.094439
H	6.792023	0.224877	-0.644642
H	6.669742	-1.450095	-2.191229
H	-2.369635	-0.430260	-1.010062
H	-3.810745	2.235185	-0.682157
H	-3.646157	1.062203	0.606571
H	-2.590056	1.969589	-2.882529
H	-3.101505	0.309415	-3.173287
H	-1.380776	0.680107	-3.037738
H	-4.871232	0.011687	-1.944205
H	-5.796349	1.227576	-1.107585
H	-5.806049	-0.073229	0.952252
H	-7.403577	-1.829471	0.126768
H	-7.648386	-0.271031	-0.664078
H	-6.817530	-1.570122	-1.523875
H	-3.690884	-1.232670	0.675044
H	-4.304473	-3.285188	1.816978
H	-5.360456	-1.975054	2.369394
H	-5.965445	-3.189474	1.230638
H	-3.449651	-3.304579	-0.595928
H	-3.894312	-2.016480	-1.720085
H	-5.105071	-3.196162	-1.198026

ωB97XD energy = -1358.17890982 a.u.

Swinhoeisterol F (**10**), Conf. Q

C	-5.207057	-2.256953	-0.128335
C	-5.557889	-1.240153	0.952739
C	-5.024088	0.126400	0.559226
C	-3.517679	0.135629	0.369535
C	-3.069553	-0.920289	-0.692036
C	-3.694529	-2.290336	-0.338265
C	-2.931464	1.533547	0.109119
C	-1.527748	1.510806	-0.543960
C	-0.759835	0.243006	-0.222762
C	-1.535271	-1.051772	-0.538954
C	0.668621	0.113311	-0.807604
C	0.731005	-1.367899	-1.253196
C	-0.714605	-1.679232	-1.672728
C	-0.807602	2.819292	-0.227747
C	0.176904	2.886823	0.924045
C	1.423605	1.987067	0.830473
C	1.112353	0.484246	0.659651
C	-3.523661	-0.519069	-2.105131
C	1.067318	1.010788	-1.975970
C	-5.838390	1.169513	0.389572
O	-6.954674	-1.289102	1.157688
O	-1.081281	3.816455	-0.870760
C	2.095906	-0.404534	1.442700
C	3.573763	-0.171825	1.095371
C	1.870855	-0.251478	2.953893
C	3.995651	-0.657357	-0.292252
C	5.449710	-0.330940	-0.684341

C	5.621953	1.181146	-0.891256
C	6.498520	-0.906699	0.294427
C	6.305117	-2.409582	0.525583
C	7.929671	-0.640737	-0.187024
H	-3.084652	-0.220541	1.318288
H	-1.661050	1.531294	-1.629779
H	-1.370024	-1.670749	0.351860
O	-0.240823	0.231674	1.127338
H	-5.739379	-1.988767	-1.047491
H	-5.569596	-3.242712	0.179353
H	-5.038910	-1.543144	1.879256
H	-3.439378	-3.015850	-1.119398
H	-3.230775	-2.657579	0.587411
H	-2.899845	2.074590	1.061140
H	-3.576696	2.113539	-0.554390
H	1.456939	-1.522081	-2.057141
H	1.012622	-2.032919	-0.432572
H	-0.900354	-2.750435	-1.795184
H	-0.942383	-1.195709	-2.630880
H	-0.370554	2.605216	1.830638
H	0.483639	3.931434	1.016163
H	1.978152	2.142342	1.759952
H	2.085851	2.325732	0.026811
H	-4.594361	-0.297188	-2.119885
H	-3.341887	-1.332830	-2.814486
H	-3.009242	0.366318	-2.484770
H	0.883805	2.073981	-1.813132
H	0.506547	0.723331	-2.871988
H	2.130473	0.895481	-2.208394
H	-5.480277	2.154151	0.112829
H	-6.911029	1.058031	0.509268
H	-7.179090	-0.725740	1.904208
H	1.852080	-1.444972	1.199888
H	4.172136	-0.694371	1.851777
H	3.813829	0.892054	1.214663
H	2.430707	-1.023769	3.490672
H	0.813319	-0.356444	3.205696
H	2.219925	0.718057	3.326493
H	3.347364	-0.209840	-1.050444
H	3.821467	-1.738021	-0.359381
H	5.623779	-0.817769	-1.656591
H	5.656639	1.715408	0.065175
H	6.541487	1.416880	-1.432801
H	4.787677	1.590955	-1.471132
H	6.374611	-0.395479	1.260256
H	7.096031	-2.806129	1.170159
H	5.347848	-2.637968	1.003013
H	6.344913	-2.954808	-0.425640
H	8.657060	-1.085991	0.499410
H	8.152878	0.427282	-0.253547
H	8.092435	-1.082793	-1.178117

ωB97XD energy = -1358.17872217 a.u.

Swinhoeisterol F (**10**), Conf. R

C	4.242446	-2.902568	0.203986
C	4.721863	-2.142987	-1.028596
C	4.608052	-0.648544	-0.782310
C	3.187254	-0.209976	-0.474774
C	2.601810	-0.985742	0.749734
C	2.805403	-2.503584	0.533208
C	3.022839	1.313628	-0.347804
C	1.739719	1.744355	0.403792
C	0.637672	0.705979	0.316485
C	1.078818	-0.709334	0.739169
C	-0.696296	1.036257	1.031366
C	-1.094530	-0.317865	1.669344
C	0.257566	-0.964449	2.009823
C	1.356200	3.157973	-0.028653
C	0.303579	3.364274	-1.100741
C	-1.115125	2.861828	-0.774562
C	-1.189970	1.360012	-0.424329
C	3.302700	-0.576665	2.055921
C	-0.742420	2.125712	2.097647
C	5.678665	0.146787	-0.820210
O	6.026350	-2.590636	-1.333718
O	1.945872	4.106412	0.456417
C	-2.483118	0.703111	-0.912401
C	-3.701191	1.319624	-0.208568
C	-2.604634	0.715382	-2.439529
C	-4.997942	0.505374	-0.304121
C	-4.926030	-0.874488	0.391621
C	-6.165235	-1.110437	1.263354
C	-4.665282	-2.032135	-0.606151
C	-4.076615	-3.261460	0.092162
C	-5.903433	-2.415912	-1.422857
H	2.571965	-0.529422	-1.331440
H	1.985306	1.846106	1.465318
H	0.658205	-1.352677	-0.043915
O	-0.024445	0.690910	-0.973956
H	4.924653	-2.687445	1.033842
H	4.303169	-3.977184	0.006130
H	4.041478	-2.394109	-1.861461
H	2.459703	-3.043862	1.422258
H	2.161988	-2.828413	-0.295914
H	3.038149	1.747276	-1.353713
H	3.865083	1.753991	0.190457
H	-1.742571	-0.185193	2.541136
H	-1.624650	-0.967914	0.968669
H	0.169151	-2.026946	2.255618
H	0.711713	-0.463554	2.873938
H	0.648263	2.838609	-1.998062
H	0.274374	4.435512	-1.313661
H	-1.721345	3.059455	-1.662605
H	-1.558018	3.457228	0.029993
H	2.992833	-1.229108	2.878505
H	3.088639	0.451916	2.354030
H	4.388185	-0.661072	1.954216
H	-0.272364	3.066208	1.804756
H	-0.235847	1.784700	3.006797
H	-1.782493	2.344091	2.363209
H	5.621551	1.215632	-0.651291
H	6.665442	-0.264419	-1.006074

H	6.304313	-2.195451	-2.165368
H	-2.406949	-0.347427	-0.620519
H	-3.876920	2.324572	-0.614916
H	-3.468846	1.455060	0.856877
H	-2.839166	1.710968	-2.830998
H	-3.404424	0.044673	-2.767679
H	-1.673116	0.374051	-2.899563
H	-5.285690	0.383799	-1.355942
H	-5.796752	1.101608	0.152416
H	-4.060889	-0.850909	1.070355
H	-6.152604	-2.095302	1.740952
H	-6.217839	-0.358537	2.057420
H	-7.086840	-1.032321	0.676438
H	-3.909512	-1.676664	-1.317614
H	-3.844523	-4.048548	-0.632699
H	-3.151352	-3.010528	0.623148
H	-4.777703	-3.682495	0.821621
H	-5.635064	-3.123728	-2.213438
H	-6.366031	-1.544617	-1.898632
H	-6.661485	-2.896004	-0.794559

ωB97XD energy = -1358.17870532 a.u.

Swinhoeisterol F (**10**), Conf. S

C	4.949238	-2.678777	0.086940
C	5.386898	-1.759193	-1.047999
C	5.064521	-0.317592	-0.693075
C	3.586540	-0.088397	-0.428406
C	3.052153	-1.038420	0.691331
C	3.457944	-2.492275	0.357276
C	3.225144	1.385246	-0.180471
C	1.862923	1.584239	0.527658
C	0.908658	0.428467	0.294937
C	1.508524	-0.953690	0.622132
C	-0.486938	0.525333	0.960550
C	-0.736011	-0.917155	1.466898
C	0.673557	-1.416279	1.824268
C	1.320298	2.971374	0.189494
C	0.293890	3.137627	-0.914617
C	-1.061168	2.435787	-0.705129
C	-0.954351	0.910726	-0.487233
C	3.635480	-0.663303	2.064017
C	-0.724400	1.505077	2.104883
C	6.019656	0.610736	-0.616724
O	6.753071	-2.008442	-1.306997
O	1.765630	3.940617	0.776795
C	-2.125836	0.138920	-1.100056
C	-3.429279	0.449526	-0.352856
C	-2.240404	0.346470	-2.614885
C	-4.600396	-0.435753	-0.788014
C	-5.802207	-0.432564	0.171973
C	-6.303586	0.995539	0.417229
C	-6.936963	-1.358718	-0.330421
C	-6.452878	-2.790807	-0.592974
C	-8.123088	-1.399441	0.641631
H	3.055260	-0.400952	-1.341784
H	2.045448	1.623600	1.606025
H	1.205849	-1.573285	-0.231504
O	0.311778	0.448534	-1.027103
H	5.553225	-2.454490	0.973164
H	5.157888	-3.715865	-0.193271
H	4.789942	-2.021136	-1.939716
H	3.137485	-3.153267	1.171047
H	2.906331	-2.812709	-0.537229
H	3.234912	1.908352	-1.142950
H	3.977795	1.872181	0.443678
H	-1.428002	-0.937526	2.314297
H	-1.153425	-1.565168	0.691867
H	0.715323	-2.497954	1.983533
H	1.022436	-0.934861	2.746434
H	0.739807	2.737207	-1.831844
H	0.136948	4.211148	-1.042936
H	-1.650744	2.636462	-1.603228
H	-1.607728	2.897756	0.122922
H	3.410121	-1.438524	2.803349
H	3.243926	0.279811	2.451892
H	4.722690	-0.559374	2.008057
H	-0.362769	2.517650	1.917388
H	-0.227741	1.150560	3.014347
H	-1.796659	1.572502	2.319710
H	5.812533	1.646116	-0.373320
H	7.059955	0.351415	-0.783548
H	7.017195	-1.512654	-2.087663
H	-1.899786	-0.922888	-0.953626
H	-3.679260	1.508494	-0.493772
H	-3.263340	0.307776	0.724571
H	-2.694779	1.310636	-2.867647
H	-2.867133	-0.431523	-3.059837
H	-1.255229	0.291752	-3.086876
H	-4.221213	-1.459288	-0.886575
H	-4.944966	-0.135395	-1.787658
H	-5.454734	-0.840130	1.135888
H	-7.101557	1.026104	1.162513
H	-5.503477	1.644140	0.783764
H	-6.690561	1.433527	-0.511655
H	-7.297705	-0.943532	-1.284288
H	-7.294877	-3.437160	-0.860394
H	-5.728716	-2.847246	-1.409420
H	-5.983652	-3.211563	0.305357
H	-8.876343	-2.115274	0.297691
H	-8.617552	-0.430268	0.742248
H	-7.794446	-1.717050	1.639284

ωB97XD energy = -1358.17869127 a.u.

Swinhoeisterol F (**10**), Conf. T

C	-5.153820	-2.301363	0.058562
C	-5.532240	-1.191233	1.033372
C	-5.012365	0.139803	0.518073
C	-3.504031	0.153849	0.342943
C	-3.032682	-0.989934	-0.612520
C	-3.638956	-2.329974	-0.133473
C	-2.936351	1.529853	-0.043560
C	-1.524246	1.468555	-0.675859

C	-0.741643	0.244552	-0.239194
C	-1.497324	-1.083846	-0.442182
C	0.691248	0.085237	-0.805803
C	0.775918	-1.428646	-1.118064
C	-0.663153	-1.796981	-1.514026
C	-0.825535	2.810264	-0.468277
C	0.145845	2.990954	0.683496
C	1.407468	2.107854	0.677556
C	1.122274	0.591699	0.627522
C	-3.482581	-0.730490	-2.059513
C	1.072398	0.875900	-2.054098
C	-5.839762	1.148748	0.239735
O	-6.930772	-1.241445	1.225983
O	-1.105153	3.743878	-1.198046
C	2.115291	-0.213370	1.485846
C	3.596259	0.066804	1.191245
C	1.827274	-0.009141	2.979166
C	4.024286	-0.141380	-0.261165
C	5.544159	-0.106339	-0.507968
C	6.108249	1.287027	-0.195848
C	6.322236	-1.222865	0.226925
C	5.692088	-2.604031	0.016341
C	7.794730	-1.253883	-0.199921
H	-3.074232	-0.104320	1.324160
H	-1.646293	1.390374	-1.760425
H	-1.327433	-1.619092	0.500443
O	-0.229470	0.357753	1.108197
H	-5.679499	-2.131218	-0.887447
H	-5.505009	-3.258527	0.456411
H	-5.021291	-1.397230	1.990516
H	-3.364332	-3.122052	-0.839787
H	-3.178052	-2.597102	0.827086
H	-2.926551	2.161188	0.851857
H	-3.582064	2.031364	-0.767706
H	1.507178	-1.643488	-1.903049
H	1.059478	-2.016152	-0.240712
H	-0.832984	-2.877453	-1.541331
H	-0.893501	-1.403475	-2.511766
H	-0.406228	2.775296	1.605215
H	0.433964	4.044890	0.690743
H	1.947368	2.345821	1.598445
H	2.075257	2.392281	-0.142199
H	-3.279395	-1.604053	-2.687315
H	-2.980544	0.124982	-2.516665
H	-4.556815	-0.530925	-2.103139
H	0.932270	1.954589	-1.966874
H	0.461564	0.543486	-2.900604
H	2.117211	0.696625	-2.321191
H	-5.492838	2.107371	-0.127680
H	-6.912047	1.031746	0.357081
H	-7.172205	-0.614890	1.914630
H	1.933331	-1.273316	1.277619
H	4.178396	-0.595998	1.843198
H	3.846488	1.087566	1.507682
H	2.462258	-0.672867	3.574534
H	0.783256	-0.228656	3.210936
H	2.039325	1.017036	3.299915
H	3.576991	0.643809	-0.879569
H	3.610189	-1.086218	-0.631958
H	5.682901	-0.286459	-1.585393
H	6.176461	1.455198	0.885190
H	7.107149	1.427698	-0.616525
H	5.464658	2.068532	-0.614315
H	6.298389	-1.001597	1.303754
H	6.319305	-3.385953	0.456063
H	4.701487	-2.682159	0.474409
H	5.585919	-2.823633	-1.053420
H	8.326358	-2.066862	0.305147
H	8.315298	-0.323012	0.040048
H	7.881814	-1.420362	-1.280941

ωB97XD energy = -1358.17866918 a.u.

Swinhoeisterol F (**10**), Conf. U

C	-4.967629	-2.471250	0.089306
C	-5.426532	-1.377151	1.047554
C	-4.985174	-0.021267	0.523725
C	-3.479253	0.086296	0.358142
C	-2.927537	-1.037074	-0.578192
C	-3.451934	-2.406082	-0.086041
C	-2.999628	1.490398	-0.046323
C	-1.580443	1.512524	-0.664745
C	-0.725379	0.347246	-0.205466
C	-1.390614	-1.030283	-0.395096
C	0.717486	0.272324	-0.764088
C	0.906468	-1.237332	-1.054275
C	-0.503845	-1.701625	-1.452610
C	-0.968857	2.898929	-0.473544
C	-0.013567	3.157224	0.676619
C	1.300131	2.353717	0.677728
C	1.104422	0.822641	0.654096
C	-3.381516	-0.825219	-2.031848
C	1.098357	1.070232	-2.007050
C	-5.870849	0.932914	0.231976
O	-6.821336	-1.512947	1.225264
O	-1.304305	3.801848	-1.218391
C	2.179630	0.077035	1.449014
C	3.536784	0.218466	0.748259
C	2.204419	0.480212	2.928087
C	4.604843	-0.739714	1.271824
C	6.004067	-0.601697	0.646581
C	6.973878	-1.535300	1.383206
C	6.089038	-0.840663	-0.887611
C	5.243290	-2.025526	-1.367473
C	5.804756	0.414498	-1.723310
H	-3.041050	-0.129234	1.345829
H	-1.684718	1.410373	-1.749406
H	-1.194357	-1.541836	0.555583
O	-0.225386	0.512613	1.146507
H	-5.491806	-2.344261	-0.864321
H	-5.261687	-3.444505	0.494134
H	-4.915182	-1.541065	2.012631
H	-3.119805	-3.189053	-0.777735

H	-2.986025	-2.629833	0.883276
H	-3.039831	2.135842	0.838078
H	-3.670596	1.935349	-0.784444
H	1.652435	-1.411499	-1.836122
H	1.226531	-1.795302	-0.170601
H	-0.606457	-2.790844	-1.468177
H	-0.752010	-1.334344	-2.456347
H	-0.551778	2.918953	1.600963
H	0.207837	4.227081	0.668602
H	1.831811	2.639060	1.589022
H	1.941519	2.663267	-0.153433
H	-3.126873	-1.696045	-2.644633
H	-2.923834	0.049214	-2.499722
H	-4.464784	-0.685122	-2.084566
H	0.619589	0.638892	-2.892671
H	2.181578	1.021125	-2.164880
H	0.818469	2.124647	-1.973327
H	-5.580456	1.908227	-0.140581
H	-6.934984	0.752948	0.343848
H	-7.109268	-0.897503	1.905964
H	1.898515	-0.981506	1.424731
H	3.896549	1.253313	0.837620
H	3.381946	0.035789	-0.319543
H	2.702569	1.442828	3.086529
H	2.741870	-0.264003	3.522107
H	1.187170	0.548351	3.324713
H	4.250074	-1.772605	1.146738
H	4.716811	-0.590118	2.352712
H	6.341952	0.429214	0.828748
H	8.004075	-1.382893	1.045865
H	6.945827	-1.363313	2.464198
H	6.718261	-2.587530	1.210434
H	7.138149	-1.099662	-1.087458
H	5.449210	-2.238745	-2.421397
H	5.457128	-2.935979	-0.798037
H	4.170393	-1.821105	-1.278273
H	6.007708	0.222230	-2.782447
H	6.441633	1.247711	-1.407638
H	4.765241	0.744545	-1.644270

ωB97XD energy = -1358.17864465 a.u.

Lobatolide A (**11**), Conf. A

C	-2.274134	-1.291277	0.990475
C	-2.820220	-0.269822	1.931070
C	-1.995784	1.029702	1.741750
C	-1.756580	1.422128	0.273013
C	-0.324758	1.163015	-0.296585
C	-0.175139	0.077452	-1.394750
C	0.399752	-1.286681	-0.935915
C	-0.482303	-2.180862	-0.062581
O	-0.930504	-1.413815	1.064219
C	-1.781689	-2.571330	-0.797223
C	-2.851538	-1.993933	-0.009290
O	-1.980394	2.847068	0.178944
C	-0.977693	3.506129	-0.432950

C	0.078317	2.525689	-0.805193
C	0.241257	-3.427243	0.425064
C	-4.319773	-0.047283	1.737149
O	-1.804545	-3.222441	-1.826187
O	-0.997442	4.695860	-0.618445
C	1.160734	2.894371	-1.485988
O	-1.407251	-0.076499	-2.064356
O	1.610689	-1.064078	-0.201487
C	2.744340	-0.813396	-0.888903
O	2.770518	-0.675850	-2.090795
C	3.947739	-0.781186	0.020377
C	3.752031	-0.061789	1.364091
C	4.998916	-0.240902	2.232949
C	3.436521	1.421034	1.157954
H	-2.641958	-0.625816	2.953473
H	-2.545104	1.848502	2.214103
H	-1.038163	0.939331	2.261936
H	-2.500558	0.960435	-0.380402
H	0.339013	0.882391	0.528065
H	0.577430	0.451585	-2.096598
H	0.636713	-1.860341	-1.840555
H	-3.900420	-2.019785	-0.259826
H	0.541002	-4.030613	-0.435835
H	1.122413	-3.156163	1.008659
H	-0.431362	-4.023153	1.047104
H	-4.536881	0.364270	0.746400
H	-4.689837	0.661379	2.482187
H	-4.878172	-0.980835	1.850310
H	1.283636	3.935834	-1.766924
H	1.932962	2.189464	-1.781875
H	-1.281471	-0.581315	-2.874051
H	4.217869	-1.829883	0.201730
H	4.766935	-0.331509	-0.548386
H	2.902702	-0.529228	1.875555
H	5.210632	-1.299262	2.417108
H	5.878740	0.200758	1.750373
H	4.869543	0.250033	3.202330
H	3.291441	1.925526	2.118046
H	2.526166	1.568355	0.569436
H	4.258163	1.925195	0.635411

ωB97XD energy = -1304.05041576 a.u.

Lobatolide A (**11**), Conf. B

C	1.675904	-2.037947	-0.758151
C	2.673048	-1.518162	-1.739829
C	2.571162	0.029814	-1.757272
C	2.456623	0.674734	-0.365273
C	1.030390	1.150772	0.057744
C	0.385250	0.441997	1.280591
C	-0.737576	-0.568435	0.946473
C	-0.357372	-1.858174	0.215990
O	0.435561	-1.531440	-0.935606
C	0.580020	-2.719009	1.088763
C	1.821257	-2.801024	0.348062
O	3.296807	1.850266	-0.369268
C	2.649348	2.971262	0.001950

C	1.231912	2.625423	0.300172
C	-1.570487	-2.668706	-0.214944
C	4.089356	-2.004166	-1.432792
O	0.261313	-3.174149	2.172411
O	3.187605	4.046840	0.061164
C	0.357301	3.543042	0.703579
O	1.397352	-0.140661	2.070663
O	-1.712514	0.112452	0.147733
C	-2.684683	0.802068	0.786647
O	-2.735807	0.909768	1.989708
C	-3.655289	1.403752	-0.196741
C	-4.246186	0.384303	-1.186562
C	-4.977569	-0.740934	-0.450403
C	-5.175931	1.092748	-2.173216
H	2.381313	-1.878412	-2.734360
H	3.479524	0.419187	-2.224775
H	1.728691	0.340975	-2.381638
H	2.871191	0.018461	0.402931
H	0.345755	1.024721	-0.785076
H	-0.127345	1.221569	1.857386
H	-1.204883	-0.870130	1.891252
H	2.725962	-3.278250	0.690637
H	-1.242833	-3.578466	-0.724092
H	-2.145274	-2.955172	0.669930
H	-2.201120	-2.086720	-0.888786
H	4.445245	-1.613953	-0.473748
H	4.777405	-1.660509	-2.209112
H	4.139196	-3.096209	-1.396967
H	0.678174	4.573059	0.824766
H	-0.677908	3.300140	0.927451
H	1.031065	-0.429178	2.912691
H	-4.446966	1.883903	0.385273
H	-3.122652	2.186714	-0.750820
H	-3.412744	-0.051953	-1.750075
H	-5.381076	-1.469431	-1.160491
H	-4.315414	-1.279302	0.235842
H	-5.813225	-0.342306	0.136067
H	-4.652890	1.884739	-2.719201
H	-6.026510	1.547111	-1.651800
H	-5.574094	0.384648	-2.906456

ωB97XD energy = -1304.05008165 a.u.

C	-0.441927	2.382772	0.751582
C	0.370614	-3.446032	-0.714799
C	4.518150	0.589162	-1.403463
O	2.152936	-3.102982	1.736644
O	0.316045	4.690414	0.777240
C	-1.637218	2.556522	1.309985
O	1.296216	-0.038188	2.066724
O	-1.383413	-1.357707	-0.090889
C	-2.582204	-1.308698	0.527135
O	-2.702813	-1.230163	1.729405
C	-3.730735	-1.347578	-0.446647
C	-4.369937	0.040268	-0.665717
C	-3.449457	0.962474	-1.467945
C	-5.722990	-0.119322	-1.362247
H	3.063630	-0.141583	-2.810051
H	2.554852	2.250445	-1.978592
H	1.200208	1.154670	-2.228172
H	2.373465	1.226920	0.557026
H	-0.311323	0.784109	-0.654682
H	-0.737760	0.205989	1.925792
H	-0.421891	-2.081684	1.586886
H	4.195201	-1.538097	0.446254
H	0.075984	-4.134346	0.081536
H	-0.479680	-3.258669	-1.371994
H	1.176193	-3.908322	-1.290840
H	4.578370	0.977501	-0.381769
H	4.857199	1.376614	-2.081147
H	5.211621	-0.252173	-1.490315
H	-1.942157	3.553062	1.614610
H	-2.326856	1.734535	1.482923
H	1.171675	-0.596888	2.840410
H	-3.396414	-1.758819	-1.404027
H	-4.474230	-2.023975	-0.014794
H	-4.543577	0.484096	0.323155
H	-2.483865	1.109295	-0.977214
H	-3.260385	0.544280	-2.463541
H	-3.905387	1.948789	-1.595830
H	-5.601680	-0.594017	-2.343256
H	-6.195166	0.855223	-1.519260
H	-6.406680	-0.734964	-0.769482

ωB97XD energy = -1304.04997135 a.u.

Lobatolide A (**11**), Conf. C

C	2.606567	-0.962886	-0.926744
C	3.090464	0.176457	-1.760183
C	2.078439	1.340644	-1.603677
C	1.640057	1.613559	-0.154477
C	0.212429	1.120510	0.245905
C	0.120166	-0.026190	1.286862
C	-0.206354	-1.434079	0.727990
C	0.866000	-2.145700	-0.099566
O	1.305954	-1.260329	-1.139915
C	2.134797	-2.402602	0.740485
C	3.181814	-1.641066	0.091053
O	1.640244	3.048231	0.021717
C	0.492542	3.522417	0.542627

Lobatolide A (**11**), Conf. D

C	2.688047	-1.267877	-0.737064
C	3.460365	-0.257238	-1.518810
C	2.663174	1.073876	-1.513785
C	2.063579	1.449832	-0.148132
C	0.537558	1.170713	0.027472
C	0.132735	0.141731	1.120311
C	-0.336085	-1.233136	0.588091
C	0.688319	-2.125354	-0.115214
O	1.391186	-1.358768	-1.106133
C	1.788972	-2.557026	0.877099
C	3.016026	-1.994711	0.354654
O	2.242888	2.873458	0.023135
C	1.094147	3.529442	0.277513

C	-0.021225	2.543306	0.301488
C	0.053564	-3.342231	-0.770322
C	4.881696	-0.083442	-0.983077
O	1.571855	-3.213802	1.879383
O	1.045958	4.720772	0.446382
C	-1.279884	2.912003	0.523547
O	1.178148	0.015537	2.057423
O	-1.419747	-0.987022	-0.317841
C	-2.667167	-0.933513	0.203758
O	-2.896625	-1.080815	1.381305
C	-3.691710	-0.696308	-0.876908
C	-5.038799	-0.180070	-0.360902
C	-4.901308	1.210700	0.263624
C	-6.059618	-0.169226	-1.501132
H	3.512074	-0.606824	-2.557564
H	3.350500	1.873720	-1.802305
H	1.873438	1.039523	-2.269622
H	2.622984	0.982565	0.664989
H	0.127757	0.809189	-0.918832
H	-0.757685	0.549067	1.616171
H	-0.713452	-1.809940	1.440078
H	3.982255	-2.048947	0.830925
H	-0.441482	-3.946466	-0.005640
H	-0.674200	-3.034310	-1.522665
H	0.827422	-3.949839	-1.245925
H	4.876555	0.331805	0.029770
H	5.439877	0.603472	-1.623944
H	5.419324	-1.035562	-0.960407
H	-1.510211	3.957971	0.700785
H	-2.097232	2.196603	0.538016
H	0.879222	-0.497204	2.815203
H	-3.265076	-0.009635	-1.617567
H	-3.822587	-1.656770	-1.392867
H	-5.385666	-0.871876	0.415230
H	-4.221739	1.197503	1.120655
H	-4.526019	1.932011	-0.473536
H	-5.870528	1.574448	0.618140
H	-5.743389	0.509982	-2.302022
H	-7.035891	0.171528	-1.143295
H	-6.189859	-1.165713	-1.936264

ωB97XD energy = -1304.04990859 a.u.

Lobatolide A (**11**), Conf. E

C	-2.695533	-0.903331	0.843464
C	-3.202048	0.240245	1.657141
C	-2.153191	1.378486	1.560019
C	-1.627126	1.630947	0.135739
C	-0.176350	1.135774	-0.169504
C	-0.023512	-0.009551	-1.207031
C	0.207692	-1.429019	-0.633276
C	-0.935652	-2.118597	0.111143
O	-1.420452	-1.238604	1.136159
C	-2.150680	-2.324526	-0.817156
C	-3.218599	-1.546813	-0.224388
O	-1.624998	3.061786	-0.066663
C	-0.444684	3.536406	-0.505430

C	0.510630	2.400431	-0.625255
C	-0.513243	-3.440350	0.734526
C	-4.595059	0.691509	1.219614
O	-2.118055	-2.999488	-1.830599
O	-0.256440	4.702105	-0.742248
C	1.747732	2.584675	-1.077624
O	-1.113187	0.033735	-2.098562
O	1.325778	-1.346282	0.256245
C	2.559215	-1.599511	-0.243144
O	2.756317	-1.914323	-1.393543
C	3.609906	-1.375282	0.810012
C	4.017079	0.114699	0.884086
C	4.828480	0.374168	2.154376
C	4.798789	0.541754	-0.360616
H	-3.243372	-0.083003	2.704745
H	-2.626404	2.301759	1.904685
H	-1.318568	1.170484	2.235213
H	-2.315268	1.230156	-0.611978
H	0.292038	0.792452	0.757929
H	0.908087	0.197266	-1.751468
H	0.471003	-2.083809	-1.472253
H	-4.203165	-1.410588	-0.643593
H	-0.176658	-4.121211	-0.051476
H	0.291351	-3.283593	1.454861
H	-1.366187	-3.894881	1.244737
H	-4.583244	1.089012	0.199705
H	-4.953462	1.482914	1.882551
H	-5.313685	-0.132097	1.257710
H	2.068182	3.582478	-1.361493
H	2.458698	1.771030	-1.182016
H	-0.978562	-0.591726	-2.818068
H	3.212883	-1.696147	1.777219
H	4.481140	-1.987317	0.559550
H	3.094793	0.708908	0.945441
H	4.257064	0.122689	3.053508
H	5.747369	-0.223948	2.157233
H	5.115762	1.427987	2.220634
H	4.998722	1.617707	-0.339508
H	4.264668	0.308538	-1.287003
H	5.762025	0.020829	-0.403364

ωB97XD energy = -1304.04990489 a.u.

Lobatolide A (**11**), Conf. F

C	-2.540911	-1.208125	0.810649
C	-3.235428	-0.170081	1.627498
C	-2.392456	1.130232	1.551638
C	-1.884166	1.467381	0.138611
C	-0.368367	1.200280	-0.132475
C	-0.023489	0.120130	-1.195959
C	0.423911	-1.255169	-0.647648
C	-0.603054	-2.121815	0.086134
O	-1.230963	-1.337327	1.112262
C	-1.761769	-2.506637	-0.856545
C	-2.945222	-1.918559	-0.266530
O	-2.097176	2.881533	-0.067450
C	-0.985003	3.538708	-0.445810

C	0.139130	2.565405	-0.525795
C	0.018453	-3.364125	0.705209
C	-4.680632	0.045811	1.179621
O	-1.612626	-3.146244	-1.882727
O	-0.968463	4.722512	-0.666275
C	1.361114	2.945356	-0.888579
O	-1.095919	0.012929	-2.102087
O	1.524285	-0.977412	0.222129
C	2.732378	-1.526298	-0.058194
O	2.921515	-2.283365	-0.979258
C	3.770064	-1.033565	0.915282
C	4.032328	0.479768	0.779266
C	5.032685	0.935881	1.842257
C	4.525210	0.831846	-0.626409
H	-3.231511	-0.506625	2.671774
H	-3.024942	1.959214	1.880433
H	-1.551382	1.069547	2.247801
H	-2.487428	0.966071	-0.621224
H	0.118339	0.897733	0.798512
H	0.870953	0.487986	-1.719271
H	0.781420	-1.859083	-1.489682
H	-3.935238	-1.938610	-0.694552
H	0.485956	-3.965299	-0.078096
H	0.768471	-3.085510	1.448169
H	-0.756255	-3.960196	1.193813
H	-4.726260	0.453036	0.164506
H	-5.174267	0.756250	1.847260
H	-5.248711	-0.888582	1.200182
H	1.546565	3.986420	-1.134434
H	2.189266	2.246218	-0.950964
H	-0.882012	-0.614891	-2.800340
H	3.422561	-1.253048	1.930786
H	4.688499	-1.597467	0.731160
H	3.081116	0.996726	0.959132
H	4.669199	0.720616	2.852096
H	5.996642	0.430068	1.713226
H	5.208622	2.013420	1.769691
H	4.694038	1.909266	-0.720591
H	3.811052	0.533600	-1.402067
H	5.471436	0.323601	-0.843397

ωB97XD energy = -1304.04984034 a.u.

C	1.168362	2.666193	0.251231
C	-1.342213	-2.774128	-0.373389
C	4.347388	-1.833363	-1.214826
O	0.344201	-3.147085	2.141900
O	3.068654	4.173163	0.106329
C	0.235174	3.546947	0.602040
O	1.335362	-0.069218	2.056755
O	-1.645129	0.004119	-0.080766
C	-2.681397	0.664323	0.481471
O	-2.813406	0.793083	1.677277
C	-3.645212	1.180717	-0.553776
C	-5.007969	0.466648	-0.455607
C	-5.989080	1.078824	-1.456414
C	-4.862610	-1.040999	-0.676799
H	2.731367	-1.828824	-2.635270
H	3.657493	0.534440	-2.112120
H	1.925661	0.356505	-2.366468
H	2.912492	0.145277	0.486148
H	0.424106	1.023808	-0.877712
H	-0.234539	1.213459	1.722408
H	-1.212782	-0.921957	1.714383
H	2.904270	-3.156187	0.829588
H	-1.960989	-3.076930	0.475628
H	-1.954150	-2.236013	-1.098638
H	-0.933763	-3.672954	-0.841982
H	4.610955	-1.403559	-0.243260
H	5.068313	-1.467099	-1.949892
H	4.455228	-2.919709	-1.148450
H	0.504777	4.590297	0.733394
H	-0.799085	3.261164	0.773120
H	0.927090	-0.365847	2.876367
H	-3.781528	2.251379	-0.370160
H	-3.218669	1.050259	-1.552855
H	-5.391315	0.633265	0.558107
H	-6.116147	2.153002	-1.288083
H	-5.636939	0.936014	-2.484950
H	-6.972427	0.606447	-1.371410
H	-4.435140	-1.249151	-1.665122
H	-5.836752	-1.535659	-0.620248
H	-4.215079	-1.502967	0.075626

ωB97XD energy = -1304.04973692 a.u.

Lobatolide A (**11**), Conf. G

C	1.897120	-1.990092	-0.709185
C	2.931359	-1.434683	-1.631077
C	2.745908	0.104255	-1.688519
C	2.516202	0.767432	-0.319252
C	1.045805	1.182777	0.007459
C	0.352725	0.454550	1.191578
C	-0.695346	-0.614122	0.798055
C	-0.203911	-1.895615	0.123046
O	0.648825	-1.548059	-0.978466
C	0.712991	-2.694497	1.073083
C	2.003616	-2.728382	0.418125
O	3.299774	1.981090	-0.296653
C	2.582971	3.074016	0.027894

Lobatolide A (**11**), Conf. H

C	2.638692	-0.976120	-0.784202
C	3.178453	0.148267	-1.602737
C	2.140597	1.298624	-1.544951
C	1.606166	1.594657	-0.132379
C	0.143144	1.139282	0.177157
C	-0.045267	-0.006123	1.205275
C	-0.301965	-1.424606	0.638415
C	0.843338	-2.148617	-0.070966
O	1.363227	-1.291873	-1.097149
C	2.036661	-2.366690	0.882853
C	3.129873	-1.615863	0.300630
O	1.631437	3.030595	0.031744
C	0.471188	3.534660	0.491160

C	-0.500542	2.419046	0.655618
C	0.412308	-3.472619	-0.684480
C	4.567297	0.588847	-1.141911
O	1.973809	-3.034150	1.899688
O	0.311032	4.707704	0.712124
C	-1.711614	2.629068	1.164509
O	1.041095	0.008274	2.104304
O	-1.398064	-1.366110	-0.284281
C	-2.651854	-1.434906	0.217224
O	-2.885469	-1.426467	1.404856
C	-3.685797	-1.520775	-0.876983
C	-4.312155	-0.158119	-1.256348
C	-5.093896	0.462490	-0.096647
C	-3.273794	0.814972	-1.818932
H	3.238188	-0.190702	-2.644494
H	2.625299	2.207963	-1.909972
H	1.309574	1.080145	-2.221097
H	2.279146	1.198314	0.631685
H	-0.339962	0.823842	-0.753580
H	-0.972559	0.219239	1.744806
H	-0.593107	-2.058225	1.485318
H	4.108806	-1.491818	0.736458
H	0.053547	-4.138327	0.104782
H	-0.377076	-3.315503	-1.421029
H	1.268520	-3.945456	-1.172229
H	4.539734	0.997845	-0.126911
H	4.948179	1.368507	-1.806235
H	5.276786	-0.243425	-1.156212
H	-1.999260	3.634904	1.454843
H	-2.432449	1.829608	1.309017
H	0.860000	-0.570455	2.851854
H	-3.226097	-1.967017	-1.762857
H	-4.471034	-2.188778	-0.512359
H	-5.026231	-0.387720	-2.057140
H	-5.869105	-0.217259	0.269838
H	-4.439265	0.694573	0.749205
H	-5.575818	1.391506	-0.416072
H	-2.567624	1.132403	-1.045309
H	-3.760384	1.716472	-2.202735
H	-2.699338	0.362729	-2.633687

ωB97XD energy = -1304.04932334 a.u.

Lobatolide A (**11**), Conf. I

C	2.293028	-1.725182	-0.765248
C	3.250702	-0.947236	-1.605138
C	2.808735	0.539695	-1.597127
C	2.402651	1.082092	-0.216231
C	0.868664	1.199571	0.054881
C	0.277113	0.297027	1.172225
C	-0.555027	-0.910866	0.679152
C	0.176526	-2.048647	-0.035538
O	0.998038	-1.496518	-1.076113
C	1.182105	-2.727375	0.918743
C	2.483547	-2.496285	0.328325
O	2.938973	2.420771	-0.114743
C	2.014466	3.343869	0.210935

C	0.697304	2.665929	0.361897
C	-0.774911	-3.078137	-0.625696
C	4.696678	-1.126577	-1.142444
O	0.857373	-3.297014	1.945050
O	2.276350	4.511667	0.344733
C	-0.396689	3.336252	0.713701
O	1.306483	-0.097130	2.051628
O	-1.560989	-0.412472	-0.213468
C	-2.701125	0.067082	0.329588
O	-2.906587	0.091203	1.521315
C	-3.635137	0.589342	-0.732570
C	-5.117265	0.434258	-0.371775
C	-5.981939	1.174017	-1.394467
C	-5.509118	-1.043037	-0.285037
H	3.159514	-1.310215	-2.636592
H	3.653684	1.139028	-1.946867
H	1.992087	0.689527	-2.309165
H	2.879233	0.511856	0.584372
H	0.326391	0.970544	-0.866509
H	-0.449670	0.911856	1.716533
H	-1.046487	-1.355326	1.552506
H	3.428717	-2.779645	0.764051
H	-1.362653	-3.527197	0.179161
H	-1.443195	-2.612162	-1.351340
H	-0.202912	-3.868018	-1.118953
H	4.845244	-0.722116	-0.136034
H	5.371848	-0.596930	-1.818967
H	4.985344	-2.181494	-1.133539
H	-0.333672	4.404833	0.894665
H	-1.360875	2.851313	0.839751
H	0.928222	-0.500136	2.839625
H	-3.388417	1.651553	-0.864024
H	-3.407914	0.096708	-1.683839
H	-5.267875	0.890000	0.613590
H	-5.735056	2.240023	-1.436332
H	-5.842872	0.755396	-2.398610
H	-7.042895	1.084270	-1.142485
H	-6.566599	-1.149346	-0.025010
H	-4.926622	-1.570297	0.476559
H	-5.351544	-1.542844	-1.248815

ωB97XD energy = -1304.04926938 a.u.

Lobatolide A (**11**), Conf. J

C	-2.269907	-1.245723	0.846585
C	-2.833991	-0.224557	1.776816
C	-1.961076	1.051048	1.655516
C	-1.641161	1.462512	0.207403
C	-0.190001	1.177510	-0.297062
C	-0.012288	0.102610	-1.401264
C	0.500580	-1.284078	-0.936017
C	-0.450979	-2.169730	-0.127726
O	-0.935893	-1.408227	0.987767
C	-1.720458	-2.514047	-0.933122
C	-2.813515	-1.916254	-0.193196
O	-1.826753	2.893501	0.126620
C	-0.783430	3.536495	-0.432457

C	0.266558	2.536829	-0.768303
C	0.214808	-3.440378	0.379291
C	-4.313801	0.048329	1.510090
O	-1.708140	-3.150415	-1.971407
O	-0.768341	4.728440	-0.603530
C	1.388171	2.889246	-1.391760
O	-1.211502	-0.007266	-2.136483
O	1.668271	-1.113032	-0.123135
C	2.851837	-0.866176	-0.719184
O	2.961866	-0.672200	-1.908758
C	3.997117	-0.910767	0.264165
C	3.863586	-0.058112	1.544206
C	3.583107	1.409186	1.213223
C	2.853342	-0.606495	2.557337
H	-2.719592	-0.604889	2.799684
H	-2.506128	1.877878	2.118650
H	-1.032643	0.919037	2.218107
H	-2.363376	1.029447	-0.488809
H	0.427360	0.873097	0.554889
H	0.785412	0.466233	-2.056785
H	0.775447	-1.848636	-1.835760
H	-3.847959	-1.906840	-0.499014
H	0.551286	-4.037501	-0.472226
H	1.065279	-3.196354	1.017951
H	-0.505965	-4.029593	0.951740
H	-4.700735	0.754023	2.249405
H	-4.905674	-0.869133	1.574835
H	-4.466812	0.485169	0.518091
H	1.546254	3.931261	-1.652410
H	2.158815	2.171067	-1.658078
H	-1.055417	-0.495951	-2.950685
H	4.132132	-1.964032	0.541360
H	4.883279	-0.604799	-0.296771
H	4.853861	-0.110867	2.014461
H	4.317886	1.810486	0.507459
H	2.590306	1.531955	0.767099
H	3.611160	2.024464	2.117507
H	2.931194	-0.054595	3.499468
H	3.040514	-1.664144	2.772228
H	1.827060	-0.517884	2.191769

ωB97XD energy = -1304.04921604 a.u.

Lobatolide A (**11**), Conf. K

C	1.908095	-2.024166	-0.576934
C	3.008409	-1.505991	-1.442097
C	2.838326	0.030563	-1.566653
C	2.518197	0.745553	-0.242551
C	1.029632	1.171414	-0.030597
C	0.255355	0.484020	1.127822
C	-0.776138	-0.587413	0.694789
C	-0.246119	-1.892871	0.100302
O	0.685793	-1.583873	-0.947428
C	0.595933	-2.664016	1.139120
C	1.929099	-2.727276	0.577230
O	3.299589	1.960649	-0.216194
C	2.564400	3.063931	0.020578

C	1.138248	2.662340	0.169520
C	-1.348072	-2.784284	-0.451133
C	4.388805	-1.898176	-0.915571
O	0.149506	-3.079872	2.193100
O	3.045110	4.165993	0.088260
C	0.186820	3.553461	0.435151
O	1.174366	-0.022683	2.069859
O	-1.652161	0.007996	-0.272251
C	-2.679848	0.756459	0.188493
O	-2.873486	0.953425	1.367089
C	-3.531080	1.282573	-0.937986
C	-4.876402	0.529890	-1.075662
C	-4.661343	-0.967759	-1.306518
C	-5.812538	0.783144	0.107920
H	2.874696	-1.934090	-2.443578
H	3.779633	0.441598	-1.941714
H	2.068724	0.261327	-2.308575
H	2.859562	0.155646	0.610992
H	0.470426	0.981443	-0.950781
H	-0.355769	1.262690	1.598456
H	-1.361381	-0.861241	1.580828
H	2.795882	-3.147233	1.062940
H	-1.892942	-2.271646	-1.244866
H	-0.910819	-3.702988	-0.850192
H	-2.037810	-3.049649	0.354363
H	4.484032	-2.982362	-0.807924
H	4.587053	-1.439069	0.058139
H	5.161052	-1.558747	-1.610216
H	0.449819	4.600697	0.547692
H	-0.855792	3.272825	0.557005
H	0.711858	-0.280468	2.873577
H	-3.727523	2.338997	-0.731556
H	-2.970022	1.206154	-1.872662
H	-5.346719	0.946946	-1.974890
H	-5.613307	-1.465287	-1.513741
H	-3.989482	-1.152273	-2.151273
H	-4.227611	-1.444947	-0.419811
H	-5.988050	1.853410	0.254484
H	-5.394171	0.385984	1.037449
H	-6.779771	0.300835	-0.064954

ωB97XD energy = -1304.04862815 a.u.

Lobatolide A (**11**), Conf. L

C	2.652377	-1.365875	-0.648830
C	3.496252	-0.391418	-1.401487
C	2.738964	0.961238	-1.450902
C	2.104160	1.381954	-0.114209
C	0.560733	1.178317	0.008960
C	0.065719	0.155547	1.068246
C	-0.433165	-1.197989	0.506335
C	0.594212	-2.142076	-0.121002
O	1.373578	-1.413612	-1.082345
C	1.625892	-2.601477	0.931181
C	2.898235	-2.090961	0.465102
O	2.344578	2.797937	0.046147
C	1.224165	3.506312	0.285166

C	0.064157	2.572978	0.295655
C	-0.048123	-3.345160	-0.794339
C	4.893410	-0.252430	-0.796755
O	1.334388	-3.240178	1.926252
O	1.229443	4.698782	0.453066
C	-1.175174	2.998831	0.525105
O	1.061082	-0.018892	2.051762
O	-1.431558	-0.917657	-0.483485
C	-2.699326	-0.710836	-0.057177
O	-3.006301	-0.727797	1.112205
C	-3.612694	-0.480017	-1.237521
C	-4.980206	0.146038	-0.926127
C	-5.889440	-0.781865	-0.114605
C	-4.839582	1.521002	-0.265552
H	3.589098	-0.755321	-2.432425
H	3.457918	1.735672	-1.731260
H	1.974409	0.932062	-2.232244
H	2.608347	0.899538	0.726122
H	0.163101	0.857992	-0.957747
H	-0.828222	0.593734	1.527674
H	-0.899101	-1.747380	1.332891
H	3.837721	-2.173950	0.988536
H	-0.602065	-3.922358	-0.049446
H	-0.725331	-3.023450	-1.586958
H	0.727872	-3.985320	-1.221360
H	4.849880	0.174590	0.210258
H	5.502456	0.410780	-1.415993
H	5.401283	-1.219156	-0.736808
H	-1.354273	4.053209	0.711550
H	-2.026935	2.324357	0.539822
H	0.701074	-0.504993	2.800434
H	-3.061988	0.137049	-1.955743
H	-3.743939	-1.456950	-1.721427
H	-5.452185	0.297340	-1.905913
H	-6.009402	-1.751399	-0.610416
H	-5.481093	-0.955578	0.883768
H	-6.883784	-0.337258	-0.006299
H	-4.410088	1.429510	0.737158
H	-5.817640	2.001143	-0.165598
H	-4.202202	2.184799	-0.860848

ωB97XD energy = -1304.04831371 a.u.

Lobatolide A (**11**), Conf. M

C	-1.572586	-2.079889	0.671661
C	-2.596053	-1.594290	1.643660
C	-2.524954	-0.044989	1.688136
C	-2.396429	0.626362	0.310017
C	-0.969504	1.129257	-0.076610
C	-0.289498	0.452242	-1.298810
C	0.848009	-0.539220	-0.952826
C	0.474988	-1.851466	-0.262466
O	-0.341228	-1.564379	0.884075
C	-0.437707	-2.701028	-1.172611
C	-1.689358	-2.817522	-0.454871
O	-3.252249	1.790759	0.321196
C	-2.612559	2.927340	-0.014173

C	-1.185409	2.605633	-0.293018
C	1.688633	-2.663481	0.162806
C	-3.997480	-2.100764	1.302417
O	-0.095931	-3.124199	-2.262177
O	-3.163186	3.997241	-0.061090
C	-0.315896	3.542952	-0.660495
O	-1.275133	-0.133904	-2.117933
O	1.772146	0.162834	-0.110252
C	2.730118	0.904378	-0.713137
O	2.862016	0.953257	-1.914230
C	3.570048	1.669285	0.280439
C	4.342109	0.812183	1.309566
C	3.468294	0.338911	2.474351
C	5.072828	-0.349749	0.631518
H	-2.315966	-1.965358	2.637455
H	-3.449598	0.318433	2.144539
H	-1.701153	0.272272	2.333978
H	-2.788320	-0.020937	-0.477514
H	-0.299851	0.995688	0.776904
H	0.218772	1.251352	-1.852917
H	1.353511	-0.809054	-1.886899
H	-2.582500	-3.295424	-0.825670
H	2.278999	-2.918489	-0.721347
H	2.304936	-2.099301	0.863576
H	1.361870	-3.590192	0.641035
H	-4.343775	-1.699114	0.344570
H	-4.705162	-1.784215	2.072509
H	-4.025985	-3.192635	1.245309
H	-0.648323	4.571003	-0.766291
H	0.726465	3.318870	-0.870323
H	-0.884930	-0.415997	-2.951482
H	4.270181	2.255417	-0.318643
H	2.910223	2.367913	0.809216
H	5.101581	1.485843	1.726043
H	2.666918	-0.321746	2.134386
H	4.073547	-0.205524	3.206044
H	3.003697	1.187914	2.986777
H	5.705386	-0.880746	1.349113
H	5.708691	0.000137	-0.187842
H	4.363748	-1.075851	0.217431

ωB97XD energy = -1304.04795012 a.u.

Lobatolide A (**11**), Conf. N

C	-1.929050	-1.712107	0.881696
C	-2.685433	-0.908329	1.886409
C	-2.205469	0.562854	1.787047
C	-2.076637	1.098079	0.350293
C	-0.627761	1.180670	-0.226935
C	-0.282952	0.252101	-1.423944
C	0.611481	-0.966048	-1.079928
C	-0.001412	-2.088906	-0.240080
O	-0.593474	-1.512049	0.934118
C	-1.185994	-2.747932	-0.976690
C	-2.343924	-2.483177	-0.147770
O	-2.591995	2.448267	0.353388
C	-1.736995	3.348385	-0.170017

C	-0.496358	2.638958	-0.587293
C	1.018391	-3.139077	0.173976
C	-4.197985	-1.042499	1.711415
O	-1.077209	-3.332421	-2.039437
O	-1.997013	4.520848	-0.257557
C	0.510535	3.279217	-1.176338
O	-1.471822	-0.139751	-2.074028
O	1.757499	-0.494285	-0.360161
C	2.757440	0.088183	-1.047902
O	2.731567	0.239259	-2.248677
C	3.888759	0.550325	-0.159975
C	4.014394	-0.073980	1.239587
C	5.431760	0.161248	1.771840
C	2.979056	0.474023	2.227317
H	-2.409738	-1.275512	2.883040
H	-2.937748	1.188090	2.304985
H	-1.253031	0.680506	2.311985
H	-2.716609	0.536205	-0.333557
H	0.084398	0.957776	0.572024
H	0.330382	0.845128	-2.110429
H	0.943147	-1.416116	-2.023211
H	-3.361899	-2.746275	-0.388557
H	1.445176	-3.601901	-0.719496
H	0.526411	-3.915538	0.765138
H	1.813735	-2.686035	0.768066
H	-4.526330	-0.632951	0.750889
H	-4.713878	-0.494118	2.503582
H	-4.514433	-2.088121	1.764088
H	0.430664	4.346873	-1.356328
H	1.414517	2.770952	-1.501164
H	-1.265133	-0.530770	-2.928719
H	4.799084	0.384192	-0.743980
H	3.786688	1.641234	-0.072210
H	3.862906	-1.156739	1.143264
H	6.188557	-0.265478	1.105764
H	5.635972	1.234040	1.871503
H	5.556494	-0.293325	2.759344
H	3.098699	1.558081	2.344699
H	3.112740	0.017478	3.213193
H	1.957739	0.271453	1.898666

ωB97XD energy = -1304.04784854 a.u.

Lobatolide A (**11**), Conf. O

C	-1.420450	-1.978410	0.911065
C	-2.324789	-1.426939	1.963134
C	-2.338105	0.119422	1.823300
C	-2.401428	0.621799	0.370375
C	-1.054567	1.145298	-0.221322
C	-0.490988	0.388227	-1.458073
C	0.710686	-0.540647	-1.170610
C	0.470719	-1.792469	-0.318017
O	-0.194631	-1.410532	0.897829
C	-0.528015	-2.740977	-1.012364
C	-1.660957	-2.824708	-0.115393
O	-3.326672	1.730344	0.331550
C	-2.787174	2.858115	-0.170334

C	-1.367575	2.586584	-0.529083
C	1.753748	-2.535764	0.022533
C	-3.728242	-2.027409	1.882742
O	-0.332423	-3.246035	-2.103391
O	-3.404877	3.886058	-0.278475
C	-0.573105	3.534675	-1.018329
O	-1.544767	-0.284497	-2.106944
O	1.680246	0.300521	-0.539429
C	2.948867	0.311139	-1.015278
O	3.302765	-0.335199	-1.972051
C	3.838885	1.195187	-0.182171
C	4.234933	0.557730	1.171905
C	5.481268	1.260898	1.716514
C	3.099379	0.606174	2.198862
H	-1.888078	-1.666627	2.940405
H	-3.224664	0.494879	2.341295
H	-1.465639	0.547239	2.325460
H	-2.828073	-0.139063	-0.285950
H	-0.283361	1.110922	0.551498
H	-0.074742	1.156266	-2.124677
H	1.113723	-0.896768	-2.125676
H	-2.576639	-3.360615	-0.310288
H	2.260801	-2.829910	-0.898848
H	2.418139	-1.906011	0.617994
H	1.515627	-3.432951	0.599496
H	-4.221801	-1.759983	0.942844
H	-4.342614	-1.648200	2.703023
H	-3.700699	-3.118270	1.957595
H	-0.964709	4.534687	-1.176902
H	0.466720	3.341444	-1.266239
H	-1.224351	-0.710239	-2.909030
H	4.734102	1.379984	-0.779757
H	3.337079	2.151965	0.002260
H	4.497063	-0.492433	0.983555
H	5.786999	0.822316	2.671217
H	6.322414	1.180029	1.021162
H	5.282177	2.325774	1.886152
H	2.194192	0.107091	1.845402
H	2.839408	1.647285	2.425642
H	3.411238	0.128364	3.132905

ωB97XD energy = -1304.04775851 a.u.

3-methylenecyclopent-1-ene (**12**), Conf. A

C	-1.532490	-0.606618	-0.000018
C	-0.097939	-1.193970	0.000018
C	0.835251	0.010070	0.000005
C	-0.012461	1.207429	0.000008
C	-1.311559	0.885040	0.000002
C	2.172388	-0.016115	-0.000012
H	-2.108694	-0.921047	0.878054
H	-2.108641	-0.921028	-0.878131
H	0.081813	-1.818402	0.879949
H	0.081843	-1.818456	-0.879868
H	0.389019	2.215268	0.000014
H	-2.128950	1.598710	0.000001

H	2.722550	-0.952377	-0.000019
H	2.751919	0.902313	-0.000020

ωB97XD energy = -233.356186215 a.u.

(*S*)-4-isopropyl-1-methyl-3-methylenecyclohex-1-ene (**13**), Conf. A

C	0.784587	0.117595	0.719106
C	-0.042546	1.334180	0.350008
C	-1.356402	1.057864	-0.249167
C	-1.938791	-0.152407	-0.239013
C	-1.282666	-1.326385	0.446055
C	-0.157816	-0.896685	1.389041
C	1.533268	-0.457507	-0.515324
C	2.554951	0.542823	-1.063868
C	2.229892	-1.784116	-0.194021
C	0.385066	2.589285	0.542959
C	-3.265029	-0.414047	-0.892842
H	1.547520	0.420132	1.449317
H	-1.871160	1.896181	-0.716320
H	-2.043069	-1.889166	1.002333
H	-0.904454	-2.021266	-0.319129
H	-0.599983	-0.418890	2.271321
H	0.394212	-1.769385	1.749588
H	0.791937	-0.642845	-1.304538
H	2.085719	1.487233	-1.349236
H	3.323004	0.761428	-0.311029
H	3.058763	0.133001	-1.945228
H	2.826249	-2.118802	-1.048805
H	1.520936	-2.582631	0.042424
H	2.908938	-1.670592	0.660387
H	-0.221349	3.444577	0.256395
H	1.352877	2.794226	0.991268
H	-3.660967	0.479342	-1.382663
H	-3.174864	-1.206439	-1.646508
H	-4.001391	-0.759712	-0.157180

ωB97XD energy = -429.902877726 a.u.

(*S*)-4-isopropyl-1-methyl-3-methylenecyclohex-1-ene (**13**), Conf. B

C	-0.625953	-0.097196	-0.410835
C	0.174537	1.185808	-0.222308
C	1.631714	1.013578	-0.111472
C	2.248436	-0.173282	-0.003271
C	1.454356	-1.452805	0.070257
C	-0.002503	-1.204753	0.453815
C	-2.151602	0.042713	-0.223292
C	-2.905580	-1.153898	-0.814151
C	-2.561274	0.268559	1.236274
C	-0.348658	2.420528	-0.207176
C	3.743197	-0.305401	0.051136
H	-0.463624	-0.389436	-1.461475
H	2.229339	1.923860	-0.135264
H	1.508678	-1.963636	-0.903113
H	1.925161	-2.132906	0.791537
H	-0.571662	-2.134338	0.354541
H	-0.047328	-0.909604	1.509146
H	-2.465714	0.919237	-0.803287
H	-3.986624	-0.991726	-0.752210
H	-2.649413	-1.307965	-1.867847
H	-2.686013	-2.081461	-0.274146
H	-2.399459	-0.635390	1.833928
H	-3.625813	0.515978	1.302355
H	-1.993521	1.083490	1.694428
H	0.295075	3.290541	-0.110822
H	-1.412441	2.612216	-0.288342
H	4.242590	0.659923	-0.065398
H	4.060729	-0.747119	1.003636
H	4.100020	-0.974786	-0.741527

ωB97XD energy = -429.900736923 a.u.

(*S*)-4-isopropyl-1-methyl-3-methylenecyclohex-1-ene (**13**), Conf. C

C	0.826578	-0.032425	0.860443
C	0.232610	1.221675	0.248036
C	-1.124785	1.113211	-0.301750
C	-1.925967	0.049496	-0.131603
C	-1.490760	-1.136715	0.690659
C	-0.279679	-0.828626	1.573263
C	1.664433	-0.872719	-0.151722
C	1.046694	-1.031360	-1.546414
C	3.077547	-0.296072	-0.293942
C	0.906662	2.379704	0.192105
C	-3.291264	-0.035211	-0.751277
H	1.532097	0.283252	1.638556
H	-1.485169	1.962909	-0.880251
H	-2.329717	-1.464515	1.318093
H	-1.283240	-1.983209	0.020790
H	-0.621929	-0.232656	2.428121
H	0.136891	-1.756601	1.981357
H	1.758972	-1.873777	0.293314
H	1.692557	-1.659208	-2.169318
H	0.057031	-1.491541	-1.524227
H	0.949566	-0.058406	-2.039484
H	3.578919	-0.213657	0.676332
H	3.044058	0.701659	-0.745650
H	3.693330	-0.931833	-0.938726
H	0.474761	3.263260	-0.270459
H	1.901361	2.478971	0.616447
H	-3.522972	0.850191	-1.349208
H	-3.364317	-0.916330	-1.401288
H	-4.064612	-0.144256	0.018802

ωB97XD = -429.900265174 a.u.

(*S*)-4-isopropyl-1-methyl-3-methylenecyclohex-1-ene (**13**), Conf. D

C	-0.881583	0.383756	-0.720385
C	0.112043	1.427084	-0.241697
C	1.435257	0.954591	0.180888
C	1.865918	-0.309625	0.042966
C	1.014054	-1.358750	-0.624158
C	-0.136345	-0.756809	-1.433953

C	-1.863721	-0.030062	0.415643
C	-2.869740	-1.080087	-0.064048
C	-1.216411	-0.466745	1.734015
C	-0.213636	2.725577	-0.166770
C	3.208460	-0.758553	0.544222
H	-1.516364	0.860181	-1.478814
H	2.090058	1.691825	0.643854
H	1.648178	-1.965503	-1.283952
H	0.640633	-2.055666	0.138231
H	0.283364	-0.341193	-2.358182
H	-0.836680	-1.540435	-1.738259
H	-2.426514	0.887167	0.636676
H	-3.671907	-1.204909	0.670562
H	-3.327060	-0.795127	-1.018091
H	-2.395303	-2.059011	-0.197217
H	-0.494797	0.270951	2.094038
H	-0.701916	-1.427571	1.637609
H	-1.987665	-0.587188	2.502336
H	0.487593	3.465738	0.209040
H	-1.190740	3.083599	-0.479966
H	3.750721	0.053721	1.035521
H	3.096218	-1.579049	1.264056
H	3.826404	-1.139801	-0.277782

ωB97XD = -429.899998873 energy

Table S39. Cartesian coordinates and energies of the low-energy conformers calculated at the ωB97XD/6-31+G(d,p) SMD/$CHCl_3$ level.

(2*S*,6*R*,7*S*,8*R*)-**1**, Conf. A

C	-4.251428	-0.715675	-0.619466
C	-3.288624	0.356915	-1.046384
C	-2.934759	1.456131	-0.366894
C	-3.628035	1.984455	0.860926
C	-2.282654	-1.730204	0.558653
C	-3.469935	-2.021961	-0.327255
C	-0.432128	1.328870	-0.703460
C	-0.147751	0.690575	0.681867
C	0.080858	-0.837404	0.634201
C	-1.083337	-1.539055	-0.003327
O	0.725477	2.144358	-1.017444
C	1.565122	2.267631	0.024144
C	1.065915	1.440963	1.155132
O	2.554664	2.962992	-0.026608
C	1.670036	1.404821	2.340165
C	-1.680526	2.218723	-0.768996
C	-2.587672	-1.450273	2.004390
O	-5.194092	-1.041073	-1.640570
O	1.251835	-1.059341	-0.172894
C	2.226563	-1.860040	0.292226
O	2.165834	-2.438033	1.358159
C	3.375091	-1.927265	-0.678751
C	4.066206	-0.565735	-0.893030
C	4.672903	-0.041906	0.409523
C	5.131907	-0.693429	-1.981922
H	-4.788539	-0.418074	0.288951
H	-2.717739	0.096557	-1.939676
H	-4.537397	1.438868	1.120118
H	-3.900914	3.034464	0.699519
H	-3.136854	-2.427533	-1.288689
H	-4.156685	-2.744657	0.126929
H	-2.958024	1.972543	1.729615
H	-0.992198	0.851756	1.356429
H	0.283523	-1.191584	1.647703
H	-0.965779	-1.730394	-1.068343
H	1.297086	0.786230	3.151599
H	2.562950	1.996279	2.521353
H	-1.531285	3.072519	-0.098503
H	-0.475815	0.571761	-1.487387
H	-1.763945	2.618416	-1.785317
H	-1.691056	-1.388919	2.626191
H	-3.233736	-2.233332	2.415840
H	-3.133020	-0.503365	2.106236
H	-5.700015	-0.246958	-1.847407
H	4.091499	-2.660013	-0.296527
H	2.982703	-2.294419	-1.633981
H	3.307254	0.147434	-1.236368
H	5.143485	0.933732	0.253124
H	3.917497	0.077972	1.193579
H	5.437201	-0.733657	0.785201
H	5.618409	0.271245	-2.159885
H	4.698160	-1.033428	-2.928599
H	5.906979	-1.411481	-1.687300

ωB97XD energy = -1154.85285053 a.u.

(2*S*,6*R*,7*S*,8*R*)-**1**, Conf. B

C	-4.285924	-0.961682	-0.533989
C	-3.417404	0.165628	-1.017446
C	-3.117113	1.300729	-0.371789
C	-3.799308	1.803543	0.872650
C	-2.211292	-1.794865	0.614336
C	-3.402102	-2.196979	-0.223011
C	-0.623432	1.341029	-0.788052
C	-0.244440	0.764973	0.601554
C	0.088925	-0.744713	0.586345
C	-1.045490	-1.540963	0.008579
O	0.459102	2.229405	-1.165287
C	1.317232	2.451183	-0.155803
C	0.924523	1.617615	1.011817
O	2.242537	3.224956	-0.256911
C	1.570476	1.661238	2.174445
C	-1.933866	2.137948	-0.834425
C	-2.490858	-1.500069	2.061852
O	-5.225604	-1.390077	-1.519068
O	1.242104	-0.920828	-0.259827
C	2.325922	-1.544177	0.233290
O	2.397999	-1.968738	1.368659
C	3.421058	-1.656335	-0.794954
C	4.760153	-1.085596	-0.296764
C	5.848707	-1.334817	-1.341485
C	4.636294	0.405359	0.023033
H	-4.822261	-0.675980	0.378502
H	-2.862418	-0.073267	-1.926755
H	-4.651030	1.194394	1.181228
H	-4.161649	2.824802	0.702510
H	-3.070358	-2.611921	-1.180881
H	-4.021163	-2.949884	0.277020
H	-3.095870	1.863544	1.712134
H	-1.074330	0.882400	1.303013
H	0.351061	-1.052505	1.601034
H	-0.948401	-1.756311	-1.053858
H	1.276876	1.037495	3.014031
H	2.417350	2.328589	2.307783
H	-1.825098	3.020275	-0.193861
H	-0.638334	0.560889	-1.550239
H	-2.076257	2.499566	-1.858326
H	-3.171081	-0.644130	2.156363
H	-1.588705	-1.278232	2.637138
H	-2.991558	-2.353942	2.531928
H	-5.796428	-0.644030	-1.735777
H	3.533726	-2.724463	-1.015618
H	3.112076	-1.151545	-1.714992
H	5.029312	-1.619401	0.622631

H	6.813444	-0.949978	-0.994189
H	5.968961	-2.403471	-1.550451
H	5.608436	-0.829825	-2.285133
H	5.593128	0.811118	0.368052
H	3.897825	0.591945	0.809989
H	4.331273	0.971809	-0.864903

ωB97XD energy = -1154.85173206 a.u.

(2*S*,6*R*,7*S*,8*R*)-**1**, Conf. C

C	-4.321551	-1.007037	-0.478981
C	-3.489313	0.148885	-0.959057
C	-3.187983	1.273032	-0.295015
C	-3.842110	1.735055	0.979959
C	-2.197223	-1.832838	0.574549
C	-3.404448	-2.233439	-0.238987
C	-0.711618	1.362377	-0.790768
C	-0.273297	0.767144	0.573582
C	0.076604	-0.739432	0.519962
C	-1.052892	-1.541821	-0.055487
O	0.344302	2.271377	-1.193523
C	1.248403	2.472835	-0.221308
C	0.909452	1.618405	0.947822
O	2.171324	3.245499	-0.351701
C	1.613104	1.639261	2.077165
C	-2.033546	2.141140	-0.773894
C	-2.439796	-1.572824	2.035749
O	-5.289440	-1.422667	-1.441854
O	1.223926	-0.875375	-0.340446
C	2.357433	-1.395055	0.163052
O	2.454486	-1.820853	1.295423
C	3.458321	-1.403519	-0.865811
C	4.852151	-1.168157	-0.270381
C	5.918635	-1.367109	-1.348887
C	4.958508	0.226589	0.350785
H	-4.828674	-0.759072	0.460882
H	-2.959329	-0.056665	-1.891112
H	-3.113797	1.799357	1.797679
H	-4.666314	1.096610	1.303557
H	-3.094802	-2.608664	-1.220472
H	-3.991825	-3.015796	0.254163
H	-4.237872	2.748474	0.839963
H	-1.075137	0.868400	1.309580
H	0.355293	-1.066205	1.524255
H	-0.978267	-1.730057	-1.124699
H	1.361920	0.999397	2.918199
H	2.465339	2.305174	2.179571
H	-1.916311	3.007496	-0.113234
H	-0.744720	0.595453	-1.565663
H	-2.214711	2.527180	-1.782550
H	-2.973426	-2.415249	2.489137
H	-3.072650	-0.686305	2.169156
H	-1.516932	-1.414043	2.599180
H	-5.878025	-0.679620	-1.617617
H	3.416541	-2.388032	-1.349969
H	3.233410	-0.657950	-1.635215
H	5.010020	-1.914427	0.517553

H	6.922292	-1.241599	-0.928956
H	5.860627	-2.367357	-1.792035
H	5.800373	-0.632643	-2.155164
H	5.948678	0.381209	0.792459
H	4.215679	0.376576	1.141503
H	4.802839	1.003574	-0.407640

ωB97XD energy = -1154.85154715 a.u.

(2*S*,6*R*,7*S*,8*R*)-**1**, Conf. D

C	-3.774514	-1.596454	-0.422025
C	-3.094711	-0.441517	-1.101809
C	-3.105046	0.846159	-0.731827
C	-4.024976	1.448316	0.297460
C	-1.727840	-1.687340	1.025247
C	-2.703449	-2.513314	0.221620
C	-0.638414	1.338272	-0.980223
C	-0.313861	1.194215	0.530934
C	0.293271	-0.175221	0.918417
C	-0.574480	-1.310544	0.460924
O	0.287714	2.324934	-1.501482
C	0.995820	2.928836	-0.533808
C	0.638548	2.331148	0.780762
O	1.789482	3.811338	-0.773404
C	1.153560	2.764095	1.928946
C	-2.065583	1.800368	-1.302017
C	-2.233984	-1.154145	2.337038
O	-4.499005	-2.419648	-1.335632
O	1.564882	-0.279651	0.252077
C	2.676118	-0.468211	0.984734
O	2.672762	-0.571069	2.194471
C	3.897502	-0.529617	0.107087
C	3.816647	-1.614276	-0.983350
C	5.088211	-1.588029	-1.831985
C	3.589130	-2.998258	-0.371485
H	-4.452997	-1.242370	0.362891
H	-2.396592	-0.748890	-1.882420
H	-3.460986	1.852367	1.147114
H	-4.559995	2.296943	-0.145800
H	-2.185669	-3.032340	-0.592079
H	-3.212152	-3.263969	0.836204
H	-4.768588	0.747564	0.682330
H	-1.223859	1.307381	1.125701
H	0.464966	-0.184308	1.997082
H	-0.307566	-1.721808	-0.510887
H	0.892913	2.307636	2.879408
H	1.862625	3.587117	1.937666
H	-2.206212	2.803266	-0.883011
H	-0.415602	0.418263	-1.522466
H	-2.159540	1.889488	-2.389494
H	-1.447331	-0.698022	2.943130
H	-2.692704	-1.957657	2.923220
H	-3.011832	-0.397858	2.171896
H	-5.183923	-1.880839	-1.747996
H	4.017669	0.455239	-0.360015
H	4.758948	-0.706226	0.757098
H	2.964120	-1.376525	-1.630438

H	5.037739	-2.338080	-2.628272
H	5.237025	-0.608396	-2.298971
H	5.971823	-1.808935	-1.220519
H	3.562578	-3.766575	-1.151183
H	2.641536	-3.052106	0.176878
H	4.395211	-3.254482	0.326842

ωB97XD energy = -1154.85112748 a.u.

(2*S*,6*R*,7*S*,8*R*)-**1**, Conf. E

C	-3.714319	-1.461765	-0.505159
C	-2.912086	-0.404630	-1.210879
C	-2.892118	0.911774	-0.960812
C	-3.870794	1.646432	-0.083706
C	-1.833178	-1.498318	1.160146
C	-2.766486	-2.353554	0.337114
C	-0.395308	1.274272	-0.968241
C	-0.259735	1.248362	0.576992
C	0.271055	-0.095174	1.124774
C	-0.608556	-1.234237	0.691469
O	0.637286	2.172455	-1.448077
C	1.237109	2.844253	-0.451623
C	0.674360	2.394192	0.850091
O	2.093820	3.674012	-0.659425
C	1.003249	2.959659	2.008891
C	-1.749766	1.761234	-1.499183
C	-2.437064	-0.820546	2.358966
O	-4.376108	-2.341912	-1.413243
O	1.584000	-0.250082	0.558547
C	2.476149	-1.016511	1.205281
O	2.235720	-1.565722	2.261006
C	3.788551	-1.068399	0.466972
C	3.655358	-1.402637	-1.028636
C	5.025380	-1.332310	-1.703264
C	3.011420	-2.775397	-1.231982
H	-4.454366	-1.007342	0.164019
H	-2.158583	-0.811997	-1.887536
H	-4.308981	2.481259	-0.643920
H	-3.369471	2.089668	0.785786
H	-2.195918	-2.976618	-0.359856
H	-3.377160	-3.014188	0.962284
H	-4.688544	1.019297	0.276377
H	-1.231420	1.410997	1.048996
H	0.364869	-0.021645	2.212024
H	-0.269410	-1.747803	-0.206479
H	0.576567	2.622873	2.949355
H	1.717898	3.777340	2.037491
H	-1.885170	2.807241	-1.202129
H	-0.157635	0.302668	-1.403130
H	-1.714615	1.738464	-2.593598
H	-3.177332	-0.073334	2.046283
H	-1.694848	-0.317625	2.983853
H	-2.966762	-1.549635	2.981707
H	-4.990106	-1.821708	-1.944003
H	4.259352	-0.084154	0.584198
H	4.420343	-1.803544	0.973922
H	3.008561	-0.643988	-1.484198

H	4.939315	-1.536017	-2.775863
H	5.479619	-0.342668	-1.584125
H	5.712791	-2.072818	-1.275994
H	2.888016	-2.992290	-2.298208
H	2.021740	-2.836492	-0.764909
H	3.634182	-3.566168	-0.796081

ωB97XD energy = -1154.85094993 a.u.

(2*S*,6*R*,7*S*,8*R*)-**1**, Conf. F

C	-4.073744	-1.500149	-0.482011
C	-3.438598	-0.244294	-1.009487
C	-3.336708	0.942216	-0.395196
C	-4.076174	1.341963	0.854144
C	-1.877096	-1.914472	0.656615
C	-2.968917	-2.542779	-0.176040
C	-0.899402	1.425711	-0.840295
C	-0.417240	0.990253	0.569231
C	0.173565	-0.440463	0.618383
C	-0.783154	-1.443684	0.046121
O	0.005078	2.474043	-1.272748
C	0.844261	2.863964	-0.300233
C	0.601021	2.042022	0.915279
O	1.651176	3.751756	-0.464704
C	1.248754	2.246626	2.059702
C	-2.334143	1.966145	-0.907271
C	-2.206041	-1.662751	2.102488
O	-4.942775	-2.113884	-1.433330
O	1.367603	-0.439132	-0.188452
C	2.558137	-0.635189	0.407725
O	2.681803	-0.923341	1.579833
C	3.689786	-0.401061	-0.558735
C	4.961069	-1.193351	-0.237530
C	4.736453	-2.696101	-0.422298
C	6.116692	-0.701313	-1.110747
H	-4.630225	-1.298123	0.440673
H	-2.868065	-0.399560	-1.927095
H	-4.803486	0.599240	1.187210
H	-4.612421	2.281884	0.676088
H	-2.566875	-2.888228	-1.134683
H	-3.429908	-3.399633	0.327579
H	-3.382223	1.537826	1.680828
H	-1.249471	1.004394	1.277403
H	0.451288	-0.663826	1.650652
H	-0.647431	-1.645188	-1.014858
H	1.066573	1.632757	2.936779
H	1.986804	3.039901	2.138681
H	-2.389063	2.882911	-0.309178
H	-0.776332	0.621912	-1.567583
H	-2.543555	2.244380	-1.945530
H	-1.336314	-1.356558	2.689027
H	-2.623259	-2.565791	2.561124
H	-2.968180	-0.878748	2.195253
H	-5.635188	-1.482387	-1.659548
H	3.341668	-0.610078	-1.575734
H	3.891315	0.678192	-0.513516
H	5.214617	-1.008180	0.812888

H	5.641313	-3.258545	-0.168233
H	3.926159	-3.066662	0.214247
H	4.482775	-2.924084	-1.465217
H	7.038151	-1.246937	-0.881320
H	6.310355	0.365571	-0.954825
H	5.896782	-0.851728	-2.174975

ωB97XD energy = -1154.85072800 a.u.

(2*S*,6*R*,7*S*,8*R*)-**1**, Conf. G

C	-3.739890	-1.781627	-0.371468
C	-3.192141	-0.551181	-1.038263
C	-3.242670	0.711521	-0.592959
C	-4.092385	1.197416	0.551339
C	-1.597864	-1.802132	0.939809
C	-2.565876	-2.649560	0.149140
C	-0.838685	1.382345	-0.974619
C	-0.411073	1.198432	0.506115
C	0.305037	-0.142103	0.794232
C	-0.514666	-1.309561	0.327456
O	-0.015918	2.452392	-1.503806
C	0.703177	3.068011	-0.551704
C	0.472124	2.390945	0.752334
O	1.410820	4.019648	-0.795643
C	1.020168	2.817601	1.887358
C	-2.311340	1.753953	-1.193334
C	-2.045553	-1.392563	2.315487
O	-4.471402	-2.611529	-1.273104
O	1.543569	-0.134932	0.058138
C	2.697788	-0.297823	0.726772
O	2.761509	-0.454600	1.929673
C	3.885389	-0.295233	-0.196623
C	4.517842	-1.697691	-0.308936
C	3.511052	-2.723164	-0.835532
C	5.758718	-1.634147	-1.199399
H	-4.382107	-1.512471	0.475444
H	-2.551186	-0.769724	-1.894540
H	-4.709553	2.039883	0.216050
H	-3.473000	1.578963	1.372406
H	-2.068409	-3.085269	-0.724018
H	-2.979805	-3.468032	0.748269
H	-4.760629	0.433406	0.953076
H	-1.286334	1.221526	1.160580
H	0.534951	-0.187563	1.861021
H	-0.290475	-1.642359	-0.684452
H	0.850554	2.307993	2.831394
H	1.662174	3.694142	1.890820
H	-2.492784	2.732725	-0.735018
H	-0.588733	0.503352	-1.570345
H	-2.478082	1.865330	-2.269941
H	-1.256291	-0.901569	2.890052
H	-2.384495	-2.267158	2.881304
H	-2.896740	-0.702950	2.255508
H	-5.214066	-2.101668	-1.616578
H	3.582515	0.059669	-1.186140
H	4.617394	0.406536	0.215571
H	4.828021	-2.002149	0.698487

H	3.982412	-3.705224	-0.946877
H	2.657932	-2.842571	-0.158325
H	3.123408	-2.421620	-1.816303
H	6.250507	-2.611271	-1.249794
H	6.487277	-0.909078	-0.820625
H	5.492003	-1.341335	-2.222324

ωB97XD energy = -1154.85069198 a.u.

(2*S*,6*R*,7*S*,8*R*)-**1**, Conf. H

C	-4.216141	-0.629296	-0.576820
C	-3.242965	0.447729	-0.965123
C	-2.842528	1.491490	-0.227114
C	-3.486380	1.955413	1.052600
C	-2.246430	-1.746631	0.502943
C	-3.453200	-1.963576	-0.378671
C	-0.343573	1.355173	-0.630659
C	-0.036647	0.631870	0.708014
C	0.136104	-0.900758	0.590233
C	-1.056011	-1.530384	-0.068874
O	0.806037	2.194054	-0.910405
C	1.665396	2.251259	0.121150
C	1.201032	1.333824	1.195006
O	2.647292	2.958729	0.099932
C	1.849435	1.192618	2.348530
C	-1.587724	2.254806	-0.622275
C	-2.521282	-1.562644	1.969832
O	-5.187793	-0.878528	-1.592295
O	1.287743	-1.176732	-0.232292
C	2.311351	-1.873998	0.295066
O	2.347622	-2.229395	1.455874
C	3.382393	-2.171570	-0.723894
C	4.228307	-0.952147	-1.162134
C	3.493829	-0.034546	-2.140225
C	4.769454	-0.179096	0.041958
H	-4.727248	-0.374471	0.359027
H	-2.706541	0.235185	-1.891784
H	-4.388293	1.399916	1.316949
H	-3.759164	3.013619	0.956994
H	-3.143879	-2.315056	-1.369041
H	-4.145320	-2.700953	0.042712
H	-2.784580	1.895603	1.893542
H	-0.860458	0.780456	1.411323
H	0.323967	-1.299282	1.589246
H	-0.962342	-1.651260	-1.146557
H	1.504586	0.505330	3.115336
H	2.753329	1.761832	2.546275
H	-1.411113	3.073460	0.084515
H	-0.398222	0.647837	-1.459910
H	-1.690004	2.704178	-1.615804
H	-3.167948	-2.365040	2.341152
H	-3.053954	-0.619534	2.145592
H	-1.612367	-1.551469	2.576453
H	-5.682395	-0.064695	-1.741959
H	4.035184	-2.918422	-0.266315
H	2.905953	-2.620798	-1.601866
H	5.084943	-1.383156	-1.695144

H	4.152474	0.775681	-2.470044
H	3.164037	-0.585608	-3.028037
H	2.610486	0.420798	-1.685728
H	5.457257	0.607968	-0.283140
H	5.307763	-0.838468	0.731562
H	3.960119	0.304111	0.600869

ωB97XD energy = -1154.85055201 a.u.

(2*S*,6*R*,7*S*,8*R*)-**1**, Conf. I

C	-4.163994	-1.022269	-0.413516
C	-3.361109	0.149652	-0.904769
C	-3.079176	1.281792	-0.245863
C	-3.729492	1.728239	1.036679
C	-2.011716	-1.803192	0.620809
C	-3.220830	-2.229547	-0.177249
C	-0.610391	1.429440	-0.764246
C	-0.159982	0.829404	0.593214
C	0.245714	-0.658568	0.514908
C	-0.883185	-1.491741	-0.026179
O	0.426358	2.366171	-1.152443
C	1.303429	2.600455	-0.161862
C	0.971945	1.731374	0.999388
O	2.195448	3.411056	-0.270959
C	1.625130	1.798135	2.156649
C	-1.950578	2.176864	-0.736834
C	-2.238974	-1.550373	2.085203
O	-5.131696	-1.460335	-1.366678
O	1.363877	-0.729048	-0.389306
C	2.250370	-1.725880	-0.219413
O	2.165343	-2.537831	0.679981
C	3.338470	-1.692765	-1.260434
C	4.611139	-0.940234	-0.801968
C	4.331767	0.532851	-0.500937
C	5.297629	-1.628446	0.378875
H	-4.667909	-0.781043	0.529708
H	-2.836737	-0.045630	-1.842178
H	-4.536145	1.071555	1.367792
H	-4.148197	2.733160	0.902651
H	-2.914847	-2.603872	-1.160199
H	-3.787988	-3.020698	0.325602
H	-2.994453	1.806590	1.847300
H	-0.973518	0.878181	1.320669
H	0.575441	-0.984810	1.505132
H	-0.823715	-1.683224	-1.096124
H	1.367518	1.155804	2.994185
H	2.440167	2.504567	2.287500
H	-1.848755	3.046581	-0.077837
H	-0.624454	0.671295	-1.548200
H	-2.149930	2.557856	-1.743917
H	-2.921674	-0.703311	2.229624
H	-1.316247	-1.337641	2.630686
H	-2.713874	-2.421344	2.550333
H	-5.734934	-0.728946	-1.541692
H	3.595086	-2.731498	-1.486762
H	2.946377	-1.221843	-2.165913
H	5.292065	-0.987707	-1.660921

H	5.267617	1.069548	-0.313061
H	3.822663	1.026752	-1.334746
H	3.703043	0.644629	0.389512
H	6.252320	-1.140090	0.601161
H	5.498218	-2.684041	0.166574
H	4.681218	-1.582054	1.283631

ωB97XD energy = -1154.85039085 a.u.

(2*S*,6*R*,7*S*,8*R*)-**1**, Conf. J

C	-4.253201	-0.607180	-0.714490
C	-3.282531	0.498448	-1.018364
C	-2.888051	1.481402	-0.198136
C	-3.538317	1.831497	1.114349
C	-2.312853	-1.779613	0.368609
C	-3.483359	-1.944417	-0.571800
C	-0.380723	1.381409	-0.540735
C	-0.096916	0.603469	0.773149
C	0.056014	-0.928247	0.601995
C	-1.105035	-1.514259	-0.143029
O	0.768217	2.238105	-0.763686
C	1.628971	2.227332	0.266983
C	1.152645	1.261773	1.292401
O	2.618673	2.924182	0.284624
C	1.806540	1.051846	2.432238
C	-1.631001	2.272079	-0.524548
C	-2.642014	-1.722072	1.834680
O	-5.198644	-0.808037	-1.764409
O	1.252550	-1.178325	-0.162007
C	2.284110	-1.801099	0.435927
O	2.238395	-2.226657	1.572937
C	3.491486	-1.891657	-0.458043
C	4.141273	-0.518010	-0.759751
C	5.631498	-0.711553	-1.048187
C	3.457842	0.199406	-1.925147
H	-4.787350	-0.409046	0.222122
H	-2.740936	0.359075	-1.956078
H	-3.759756	2.905303	1.136817
H	-2.862353	1.639017	1.956814
H	-3.134083	-2.230463	-1.569877
H	-4.184885	-2.711009	-0.224638
H	-4.470489	1.293380	1.295958
H	-0.923160	0.742305	1.475496
H	0.183251	-1.370011	1.591935
H	-0.974943	-1.555265	-1.223047
H	1.456318	0.334372	3.167954
H	2.721084	1.596976	2.648879
H	-1.478497	3.061102	0.220515
H	-0.414075	0.708678	-1.399694
H	-1.711661	2.760745	-1.501206
H	-3.244281	-0.834170	2.063534
H	-1.754753	-1.698048	2.472145
H	-3.241762	-2.592902	2.122258
H	-5.695815	0.009384	-1.882576
H	4.202739	-2.539047	0.059486
H	3.207713	-2.378803	-1.398436
H	4.050251	0.107477	0.138195

H	6.108713	0.246181	-1.280876
H	6.151012	-1.148476	-0.188827
H	5.778242	-1.378293	-1.907100
H	3.887012	1.195642	-2.071294
H	2.384328	0.316206	-1.762363
H	3.596325	-0.367768	-2.854376

ωB97XD energy = -1154.85028579 a.u.

(2*S*,6*R*,7*S*,8*R*)-**1**, Conf. K

C	-4.238457	-0.958752	-0.425089
C	-3.423833	0.219992	-0.879571
C	-3.088439	1.305108	-0.168912
C	-3.674240	1.689893	1.163947
C	-2.084664	-1.832419	0.526548
C	-3.314283	-2.194085	-0.271776
C	-0.625482	1.445118	-0.736625
C	-0.160788	0.773621	0.582232
C	0.189201	-0.724530	0.446146
C	-0.963850	-1.499628	-0.123957
O	0.409592	2.397232	-1.090886
C	1.311679	2.564596	-0.109419
C	1.012301	1.616876	0.997388
O	2.203831	3.378700	-0.188625
C	1.736239	1.564859	2.112703
C	-1.958531	2.202132	-0.652075
C	-2.279582	-1.662344	2.007553
O	-5.236771	-1.331306	-1.374232
O	1.304186	-0.826241	-0.461020
C	2.309132	-1.663359	-0.145904
O	2.342644	-2.299622	0.888355
C	3.362577	-1.705213	-1.222696
C	4.648506	-0.923995	-0.865487
C	4.359657	0.557810	-0.622592
C	5.406733	-1.542806	0.310086
H	-4.715526	-0.754017	0.540576
H	-2.936975	0.065963	-1.844575
H	-4.488096	1.038780	1.488432
H	-4.063805	2.713615	1.109048
H	-3.032874	-2.518552	-1.279534
H	-3.887042	-3.001200	0.198106
H	-2.905607	1.695082	1.946602
H	-0.955120	0.825918	1.331050
H	0.502854	-1.097182	1.423878
H	-0.928221	-1.628497	-1.204156
H	1.512686	0.856656	2.905501
H	2.576781	2.238616	2.252977
H	-1.830086	3.044470	0.037197
H	-0.654758	0.727056	-1.557621
H	-2.174709	2.623329	-1.639402
H	-2.766787	-2.547797	2.430673
H	-2.938077	-0.809924	2.215293
H	-1.341872	-1.503333	2.545531
H	-5.822964	-0.577486	-1.506707
H	3.610389	-2.759060	-1.383738
H	2.939154	-1.302553	-2.146202
H	5.284379	-1.004167	-1.756092

H	5.293325	1.116622	-0.500698
H	3.806577	1.004135	-1.455336
H	3.770099	0.702811	0.289806
H	6.364316	-1.030910	0.455015
H	5.613606	-2.604694	0.138835
H	4.837252	-1.461017	1.241810

ωB97XD energy = -1154.85022746 a.u.

(2*S*,6*R*,7*S*,8*R*)-**1**, Conf. L

C	-4.432894	-0.363314	0.024220
C	-3.484496	0.809981	0.180170
C	-2.724475	1.394283	-0.755042
C	-2.821615	1.123826	-2.232179
C	-2.366342	-1.735985	0.466658
C	-3.666807	-1.679310	-0.294697
C	-0.263591	1.525199	-0.292496
C	-0.089223	0.559319	0.910610
C	0.019186	-0.930277	0.519090
C	-1.236848	-1.383554	-0.158909
O	0.818842	2.487903	-0.203216
C	1.637438	2.258401	0.835940
C	1.143185	1.082570	1.597810
O	2.606319	2.950470	1.055355
C	1.761388	0.623559	2.683312
C	-1.586945	2.302574	-0.337791
C	-2.474986	-1.952839	1.950870
O	-5.408614	-0.219499	-1.004666
O	1.125472	-1.049997	-0.393928
C	2.128728	-1.899097	-0.109449
O	2.140603	-2.621563	0.866043
C	3.209535	-1.813705	-1.153743
C	3.921586	-0.445045	-1.172102
C	4.633805	-0.171106	0.153549
C	4.900719	-0.389661	-2.344802
H	-4.948921	-0.493822	0.984958
H	-3.296690	1.088527	1.217445
H	-3.616227	0.418431	-2.474919
H	-1.872931	0.733845	-2.622571
H	-3.472717	-1.690562	-1.371569
H	-4.321143	-2.525539	-0.057663
H	-3.017106	2.059801	-2.769755
H	-0.956013	0.640467	1.575013
H	0.244176	-1.512731	1.414828
H	-1.267523	-1.201088	-1.231744
H	1.394873	-0.242445	3.226808
H	2.659154	1.111622	3.051573
H	-1.770074	2.745794	0.646943
H	-0.102970	1.002375	-1.238188
H	-1.463354	3.119033	-1.058060
H	-2.922535	-1.075599	2.437829
H	-1.509355	-2.140357	2.426312
H	-3.132550	-2.801721	2.166863
H	-5.898804	0.594996	-0.844385
H	3.930399	-2.611576	-0.954456
H	2.748037	-1.998488	-2.130091
H	3.159427	0.329538	-1.324407

H	5.116195	0.811171	0.138207
H	3.939572	-0.184594	1.000163
H	5.406156	-0.927000	0.341665
H	5.405174	0.581359	-2.383569
H	4.388929	-0.540716	-3.301553
H	5.671757	-1.164151	-2.248497

ωB97XD energy = -1154.85013252 a.u.

(2*S*,6*R*,7*S*,8*R*)-**1**, Conf. M

C	-3.674093	-1.773540	-0.280308
C	-3.170449	-0.518115	-0.934977
C	-3.207295	0.729596	-0.448082
C	-4.001427	1.172842	0.751846
C	-1.470240	-1.811699	0.924281
C	-2.468340	-2.644392	0.155602
C	-0.831530	1.429473	-0.930320
C	-0.325934	1.212061	0.520915
C	0.411537	-0.130564	0.736313
C	-0.423021	-1.289571	0.274948
O	-0.041068	2.515437	-1.476549
C	0.730469	3.107052	-0.550598
C	0.564106	2.403042	0.748929
O	1.428374	4.061683	-0.810564
C	1.160116	2.812135	1.865996
C	-2.315163	1.797959	-1.062092
C	-1.852678	-1.450951	2.333070
O	-4.442084	-2.583149	-1.170117
O	1.611598	-0.091264	-0.057738
C	2.801439	-0.251821	0.549292
O	2.924266	-0.426185	1.745064
C	3.934082	-0.210169	-0.441207
C	4.334885	-1.611747	-0.963282
C	4.902667	-2.503633	0.142532
C	3.185876	-2.309056	-1.695897
H	-4.275812	-1.536844	0.605047
H	-2.573689	-0.703920	-1.830043
H	-4.635840	0.388547	1.168834
H	-4.647582	2.013524	0.470859
H	-2.009285	-3.048222	-0.753314
H	-2.844777	-3.485322	0.748306
H	-3.344124	1.543533	1.547971
H	-1.164575	1.213393	1.221751
H	0.692718	-0.204547	1.789051
H	-0.247866	-1.587069	-0.757410
H	1.033067	2.285491	2.807356
H	1.798136	3.691527	1.857543
H	-2.479426	2.760678	-0.565010
H	-0.609379	0.566089	-1.558813
H	-2.536377	1.940667	-2.125108
H	-2.144696	-2.348474	2.889299
H	-2.719503	-0.777992	2.337897
H	-1.044123	-0.960439	2.880474
H	-5.200268	-2.067381	-1.467709
H	3.643084	0.422340	-1.284230
H	4.790400	0.246607	0.061927
H	5.134613	-1.425393	-1.690544

H	5.274961	-3.442921	-0.280402
H	5.732630	-2.015636	0.664774
H	4.139142	-2.752342	0.887825
H	3.540012	-3.233563	-2.163852
H	2.762281	-1.672327	-2.479783
H	2.377539	-2.575602	-1.005232

ωB97XD energy = -1154.84957433 a.u.

(2*S*,6*R*,7*S*,8*R*)-**1**, Conf. N

C	-4.445738	-0.587116	0.074600
C	-3.585165	0.661420	0.083742
C	-2.855723	1.172486	-0.916567
C	-2.923769	0.714248	-2.348550
C	-2.286028	-1.754578	0.651768
C	-3.588409	-1.873346	-0.098713
C	-0.407125	1.547627	-0.489610
C	-0.161896	0.731627	0.808465
C	0.038708	-0.782832	0.587527
C	-1.187842	-1.389404	-0.020750
O	0.583649	2.607885	-0.496367
C	1.406845	2.562203	0.562727
C	1.030882	1.412003	1.425097
O	2.293329	3.371150	0.721918
C	1.711910	1.095255	2.523571
C	-1.790012	2.204245	-0.616997
C	-2.373537	-1.818509	2.152174
O	-5.432009	-0.633807	-0.954175
O	1.143205	-0.954255	-0.322866
C	2.203266	-1.684120	0.064346
O	2.302786	-2.196034	1.161193
C	3.241936	-1.782722	-1.021712
C	4.559085	-1.078319	-0.643406
C	5.592381	-1.297109	-1.749269
C	4.336302	0.412466	-0.382303
H	-4.949834	-0.642027	1.048734
H	-3.433295	1.086802	1.075870
H	-1.949819	0.344629	-2.692721
H	-3.180959	1.560763	-2.997250
H	-3.394368	-1.993931	-1.168766
H	-4.181518	-2.731185	0.237081
H	-3.668788	-0.066884	-2.499636
H	-1.030307	0.829157	1.469059
H	0.301650	-1.241559	1.542396
H	-1.236758	-1.323328	-1.106299
H	1.442825	0.243125	3.140888
H	2.569596	1.689718	2.825741
H	-2.007161	2.740121	0.313627
H	-0.188718	0.943978	-1.373913
H	-1.730251	2.942335	-1.424564
H	-3.011028	-2.652750	2.463379
H	-2.830182	-0.903246	2.553701
H	-1.398342	-1.940754	2.630265
H	-5.976872	0.158478	-0.885109
H	3.429686	-2.849283	-1.185483
H	2.844423	-1.356243	-1.947513
H	4.933276	-1.541180	0.278190

H	6.548058	-0.832480	-1.485228
H	5.772774	-2.363117	-1.925495
H	5.253439	-0.851083	-2.692414
H	5.284501	0.915443	-0.166418
H	3.675009	0.580067	0.473616
H	3.888859	0.901768	-1.256208

ωB97XD energy = -1154.84939525 a.u.

(2*S*,6*R*,7*S*,8*R*)-**1**, Conf. O

C	-3.962722	-1.612734	-0.444915
C	-3.366897	-0.367638	-1.029981
C	-3.319311	0.851931	-0.479144
C	-4.102478	1.295632	0.727723
C	-1.769194	-1.867023	0.770051
C	-2.813546	-2.586687	-0.047397
C	-0.892633	1.410973	-0.902160
C	-0.424130	1.080876	0.540108
C	0.221726	-0.317853	0.691449
C	-0.677118	-1.393316	0.158251
O	-0.023135	2.470202	-1.377982
C	0.778550	2.950987	-0.414782
C	0.546133	2.189857	0.841866
O	1.550304	3.862379	-0.614895
C	1.169641	2.480657	1.980888
C	-2.346377	1.884061	-1.030950
C	-2.148712	-1.536090	2.187248
O	-4.806597	-2.217935	-1.422147
O	1.439306	-0.312069	-0.079666
C	2.610189	-0.493335	0.555872
O	2.698052	-0.756574	1.737946
C	3.782455	-0.305194	-0.370603
C	4.830103	-1.421468	-0.247359
C	4.259432	-2.760807	-0.719958
C	6.084771	-1.046776	-1.037050
H	-4.551437	-1.379032	0.449618
H	-2.772866	-0.549980	-1.927111
H	-3.437890	1.578422	1.553558
H	-4.804389	0.543540	1.092647
H	-2.373830	-2.971379	-0.974128
H	-3.245882	-3.432938	0.501414
H	-4.678127	2.194517	0.475329
H	-1.271481	1.100832	1.229937
H	0.477688	-0.467667	1.742459
H	-0.504417	-1.655010	-0.884183
H	0.998779	1.909824	2.888857
H	1.876846	3.304253	2.024039
H	-2.453098	2.831827	-0.491330
H	-0.719915	0.571332	-1.576703
H	-2.543393	2.090354	-2.088374
H	-2.543883	-2.423497	2.693453
H	-2.942194	-0.778530	2.205732
H	-1.308943	-1.156288	2.774022
H	-5.148634	-3.044512	-1.061644
H	3.425718	-0.219589	-1.401345
H	4.230038	0.658734	-0.097504
H	5.098439	-1.512751	0.811812
H	4.998897	-3.560608	-0.606682
H	3.369532	-3.049237	-0.148860
H	3.978708	-2.710178	-1.779286
H	6.844346	-1.831845	-0.958828
H	6.525104	-0.114377	-0.667167
H	5.853561	-0.910560	-2.100733

ωB97XD energy = -1154.84938455 a.u.

(2*R*,6*R*,7*S*,8*R*)-**1**, Conf. A

C	4.434891	-0.232376	0.444967
C	3.478283	0.882239	0.096985
C	2.647893	1.519900	0.929493
C	2.657035	1.340448	2.423941
C	2.439413	-1.690079	-0.148846
C	3.684095	-1.573933	0.696291
C	0.216469	1.545623	0.312519
C	0.128378	0.509788	-0.841148
C	0.038098	-0.958340	-0.372518
C	1.266142	-1.341450	0.394508
O	-0.884002	2.470324	0.113768
C	-1.640986	2.162051	-0.951362
C	-1.081796	0.956895	-1.615649
O	-2.610997	2.817804	-1.259345
C	-1.637152	0.412752	-2.695716
C	1.516728	2.359581	0.377275
C	2.641944	-1.970219	-1.612160
O	5.356851	-0.455579	-0.621847
O	-1.113028	-1.056179	0.487166
C	-2.101320	-1.913991	0.177143
O	-2.051615	-2.687756	-0.756821
C	-3.253372	-1.760108	1.134102
C	-3.968656	-0.400544	0.985816
C	-4.624244	-0.263708	-0.389344
C	-4.998719	-0.232524	2.102988
H	4.997958	0.003255	1.357797
H	3.366820	1.051641	-0.974600
H	3.514976	0.762934	2.775342
H	1.749890	0.825716	2.765394
H	3.409128	-1.606427	1.755296
H	4.395752	-2.384375	0.505924
H	2.673131	2.314765	2.925595
H	1.024996	0.579695	-1.466014
H	-0.125080	-1.596094	-1.243425
H	1.227763	-1.115145	1.458888
H	-1.224672	-0.476575	-3.163658
H	-2.527730	0.851694	-3.136402
H	1.751114	2.731036	-0.626490
H	0.022503	1.070383	1.277040
H	1.331659	3.225691	1.022478
H	3.111957	-1.110592	-2.106087
H	1.709785	-2.197517	-2.135383
H	3.326288	-2.814235	-1.746930
H	5.847327	0.360217	-0.775345
H	-3.956186	-2.577844	0.951391
H	-2.861208	-1.858666	2.152256

H	-3.214302	0.389456	1.092681
H	-5.101428	0.715603	-0.495084
H	-3.896811	-0.366092	-1.201184
H	-5.393010	-1.033685	-0.529296
H	-5.504986	0.734666	2.018257
H	-4.528581	-0.283202	3.090965
H	-5.763472	-1.017224	2.051518

ωB97XD energy = -1154.85241085 a.u.

(2*R*,6*R*,7*S*,8*R*)-**1**, Conf. B

C	-4.443453	-0.569528	-0.392501
C	-3.585842	0.659622	-0.213652
C	-2.808203	1.240208	-1.134376
C	-2.792725	0.852544	-2.588815
C	-2.328825	-1.764571	0.363042
C	-3.579113	-1.862319	-0.476292
C	-0.388614	1.575421	-0.540073
C	-0.220536	0.708177	0.736655
C	-0.002533	-0.794974	0.464930
C	-1.192254	-1.380397	-0.231966
O	0.609987	2.624562	-0.454310
C	1.370741	2.533774	0.648132
C	0.933580	1.360943	1.448455
O	2.255363	3.325877	0.884081
C	1.538819	1.002890	2.578176
C	-1.759451	2.245212	-0.712182
C	-2.502981	-1.877588	1.852155
O	-5.336100	-0.725285	0.710323
O	1.150934	-0.918313	-0.392072
C	2.188025	-1.672007	0.010275
O	2.221291	-2.258392	1.073302
C	3.293321	-1.696205	-1.012551
C	4.602995	-1.087142	-0.476362
C	5.706816	-1.243709	-1.522648
C	4.413000	0.379564	-0.085043
H	-5.029665	-0.508906	-1.319053
H	-3.495232	0.989747	0.822081
H	-3.602483	0.169531	-2.855211
H	-1.847363	0.363101	-2.855712
H	-3.302432	-2.005560	-1.525459
H	-4.217726	-2.700701	-0.177555
H	-2.874903	1.743555	-3.221798
H	-1.127419	0.780022	1.346653
H	0.215025	-1.293072	1.411623
H	-1.175458	-1.279942	-1.316120
H	1.221821	0.133247	3.146552
H	2.379200	1.578196	2.956050
H	-2.034062	2.729155	0.231586
H	-0.131945	1.004867	-1.436209
H	-1.650388	3.027177	-1.472055
H	-3.027070	-0.997139	2.244377
H	-1.552945	-1.975364	2.383374
H	-3.125387	-2.743477	2.100036
H	-5.889365	0.062256	0.767331
H	3.458999	-2.747115	-1.274197
H	2.973282	-1.163913	-1.913076

H	4.889831	-1.651793	0.419405
H	6.653135	-0.837211	-1.150547
H	5.870123	-2.296403	-1.778033
H	5.451678	-0.706563	-2.444414
H	5.354345	0.814071	0.267018
H	3.680242	0.491577	0.720754
H	4.067775	0.973575	-0.940153

ωB97XD energy = -1154.85159901 a.u.

(2*R*,6*R*,7*S*,8*R*)-**1**, Conf. C

C	4.445615	-0.730459	0.310906
C	3.651207	0.540313	0.131414
C	2.946134	1.186152	1.066826
C	2.975995	0.838879	2.531141
C	2.247297	-1.820974	-0.352937
C	3.513201	-1.969005	0.454705
C	0.526267	1.635539	0.555200
C	0.255034	0.771688	-0.706069
C	-0.018896	-0.719023	-0.418233
C	1.154078	-1.351985	0.262452
O	-0.421350	2.733449	0.508033
C	-1.246363	2.667696	-0.548580
C	-0.905957	1.471605	-1.361363
O	-2.110176	3.494055	-0.740528
C	-1.591474	1.134733	-2.451092
C	1.935021	2.236739	0.663680
C	2.373699	-1.988100	-1.841900
O	5.289576	-0.960857	-0.816855
O	-1.170207	-0.783257	0.447642
C	-2.221826	-1.528952	0.069404
O	-2.251524	-2.174558	-0.958620
C	-3.337887	-1.476297	1.079901
C	-4.709577	-1.217528	0.436649
C	-5.811245	-1.353903	1.487922
C	-4.754195	0.157247	-0.233884
H	5.065816	-0.682945	1.215820
H	3.533899	0.846272	-0.908962
H	2.007453	0.443551	2.863350
H	3.170430	1.736369	3.129774
H	3.258341	-2.067228	1.514544
H	4.093500	-2.851162	0.163112
H	3.737402	0.094979	2.775865
H	1.132199	0.798588	-1.362049
H	-0.268454	-1.213189	-1.358374
H	1.176799	-1.221956	1.343557
H	-1.349218	0.248369	-3.030503
H	-2.422643	1.749152	-2.785389
H	2.197890	2.691569	-0.297664
H	0.281543	1.082104	1.465285
H	1.893940	3.034554	1.413468
H	2.946649	-1.157668	-2.272959
H	1.407412	-2.038486	-2.349856
H	2.930678	-2.900939	-2.076714
H	5.878546	-0.203746	-0.915108
H	-3.343397	-2.450515	1.584224
H	-3.115223	-0.713989	1.832463

H	-4.865600	-1.984326	-0.331805
H	-6.798378	-1.213965	1.034766
H	-5.794565	-2.342002	1.960514
H	-5.696499	-0.599696	2.276418
H	-5.732998	0.334967	-0.691586
H	-3.999498	0.248953	-1.022834
H	-4.573338	0.955543	0.496430

ωB97XD energy = -1154.85133029 a.u.

(2*R*,6*R*,7*S*,8*R*)-**1**, Conf. D

C	4.380219	-0.261229	0.355892
C	3.425511	0.860541	0.025681
C	2.609696	1.499913	0.871001
C	2.635732	1.319124	2.364927
C	2.359102	-1.699291	-0.195516
C	3.624703	-1.597891	0.619483
C	0.159389	1.584129	0.295873
C	0.024355	0.526429	-0.833478
C	-0.041106	-0.940555	-0.359546
C	1.207079	-1.322734	0.373301
O	-0.899699	2.551840	0.073546
C	-1.690876	2.233696	-0.963771
C	-1.206446	0.971334	-1.578018
O	-2.634172	2.921339	-1.284864
C	-1.835495	0.387985	-2.595495
C	1.485233	2.357635	0.335916
C	2.523503	-1.995904	-1.660087
O	5.280602	-0.490482	-0.727998
O	-1.162200	-1.079620	0.536886
C	-2.139497	-1.949958	0.225786
O	-2.145536	-2.612444	-0.792169
C	-3.206552	-2.004006	1.289560
C	-4.172433	-0.794762	1.295773
C	-3.531496	0.482669	1.839550
C	-4.796189	-0.560305	-0.081296
H	4.961640	-0.029834	1.258193
H	3.300692	1.034858	-1.043626
H	3.471809	0.703179	2.703386
H	1.711576	0.846839	2.721545
H	3.375852	-1.634287	1.684721
H	4.324160	-2.413374	0.407059
H	2.704736	2.292121	2.865134
H	0.898530	0.588586	-1.491723
H	-0.218255	-1.573425	-1.230593
H	1.200571	-1.085221	1.436003
H	-1.484156	-0.543927	-3.028235
H	-2.729393	0.838100	-3.017882
H	1.709398	2.717012	-0.674607
H	-0.050133	1.138271	1.271937
H	1.333199	3.232515	0.977960
H	3.020236	-1.158862	-2.166499
H	1.573931	-2.186658	-2.166074
H	3.169712	-2.868670	-1.800932
H	5.774743	0.321469	-0.889820
H	-3.774207	-2.919555	1.107713
H	-2.719537	-2.086041	2.266701

H	-4.974680	-1.081663	1.986957
H	-2.740680	0.846970	1.178411
H	-4.280638	1.276384	1.926622
H	-3.090720	0.320545	2.829311
H	-5.260502	-1.471180	-0.474297
H	-4.042560	-0.228596	-0.805305
H	-5.563880	0.218450	-0.028485

ωB97XD energy = -1154.85102641 a.u.

(2*R*,6*R*,7*S*,8*R*)-**1**, Conf. E

C	-4.039570	-1.171154	-0.370667
C	-3.415430	0.185808	-0.590668
C	-2.589938	0.541226	-1.581377
C	-2.274345	-0.329500	-2.767907
C	-1.921047	-1.570477	0.973422
C	-2.987986	-2.209823	0.119982
C	-0.374183	1.546081	-0.940479
C	-0.270574	1.248140	0.580791
C	0.183441	-0.184939	0.927548
C	-0.775579	-1.199362	0.387697
O	0.423430	2.735471	-1.176436
C	1.048548	3.173380	-0.072800
C	0.690045	2.298300	1.073344
O	1.776725	4.140651	-0.088854
C	1.210545	2.465241	2.286919
C	-1.786776	1.818688	-1.476496
C	-2.316898	-1.186449	2.371956
O	-5.061837	-1.098696	0.622806
O	1.475219	-0.370115	0.318555
C	2.498474	-0.802304	1.073395
O	2.391918	-1.086617	2.249104
C	3.774601	-0.860265	0.275474
C	3.661998	-1.659086	-1.034652
C	4.988028	-1.600656	-1.794303
C	3.241728	-3.105568	-0.766031
H	-4.474438	-1.560884	-1.300449
H	-3.548644	0.876353	0.243172
H	-2.904792	-1.220073	-2.821626
H	-1.229989	-0.666004	-2.742062
H	-2.525403	-2.654065	-0.766705
H	-3.530252	-2.999741	0.651036
H	-2.399418	0.233363	-3.700038
H	-1.252749	1.382964	1.047598
H	0.297411	-0.262590	2.010206
H	-0.595437	-1.488074	-0.646744
H	0.956246	1.809443	3.114717
H	1.915633	3.270043	2.474825
H	-2.279204	2.540291	-0.815566
H	0.105719	0.756424	-1.524561
H	-1.678525	2.285281	-2.461995
H	-1.470074	-0.860001	2.980404
H	-2.795765	-2.032175	2.876570
H	-3.059983	-0.379656	2.351063
H	-5.733438	-0.475043	0.323357
H	4.058551	0.175730	0.053102
H	4.545090	-1.289079	0.922517

H	2.891284	-1.183634	-1.652746
H	4.912535	-2.133505	-2.748094
H	5.279245	-0.566770	-2.009385
H	5.794467	-2.065703	-1.214243
H	3.162102	-3.666365	-1.703281
H	2.269164	-3.162342	-0.263758
H	3.976531	-3.613431	-0.129586

ωB97XD energy = -1154.85066408 a.u.

(2*R*,6*R*,7*S*,8*R*)-**1**, Conf. F

C	-3.914641	-1.612357	-0.233020
C	-3.503703	-0.170835	-0.410231
C	-2.832876	0.352929	-1.442050
C	-2.526140	-0.396454	-2.710482
C	-1.648898	-1.778475	0.908207
C	-2.692754	-2.516493	0.107304
C	-0.723871	1.632406	-0.925260
C	-0.439629	1.264105	0.556369
C	0.230725	-0.106367	0.775309
C	-0.625497	-1.216309	0.252155
O	-0.136807	2.943394	-1.136308
C	0.510968	3.407190	-0.056100
C	0.401536	2.414168	1.043303
O	1.074098	4.479035	-0.056643
C	1.010845	2.577747	2.215186
C	-2.205269	1.723315	-1.318987
C	-1.961720	-1.524426	2.357205
O	-4.849993	-1.740964	0.837463
O	1.486510	-0.086170	0.067507
C	2.594060	-0.471410	0.722480
O	2.593321	-0.848866	1.877189
C	3.822580	-0.398810	-0.143877
C	4.417711	-1.795458	-0.408854
C	3.440266	-2.676577	-1.189630
C	5.749428	-1.659071	-1.146771
H	-4.368229	-2.009026	-1.150913
H	-3.645774	0.442255	0.480810
H	-2.795945	0.207342	-3.584723
H	-3.056995	-1.348526	-2.778594
H	-2.254914	-2.850541	-0.838449
H	-3.076143	-3.397916	0.632044
H	-1.453207	-0.609728	-2.795738
H	-1.386964	1.230631	1.106189
H	0.437624	-0.220718	1.840514
H	-0.503236	-1.421749	-0.810159
H	0.942233	1.835364	3.005415
H	1.600805	3.469766	2.405573
H	-2.728583	2.328569	-0.570661
H	-0.188863	0.961917	-1.603123
H	-2.257359	2.253728	-2.276338
H	-2.761861	-0.779402	2.451452
H	-1.096768	-1.169220	2.923089
H	-2.332131	-2.437424	2.833971
H	-5.623791	-1.204229	0.630821
H	3.584767	0.097558	-1.089315
H	4.553782	0.214407	0.393300

H	4.606895	-2.264779	0.564335
H	3.871996	-3.666763	-1.368809
H	2.497703	-2.820818	-0.649698
H	3.206611	-2.228224	-2.163130
H	6.204604	-2.641279	-1.312489
H	6.459801	-1.050649	-0.576757
H	5.608197	-1.184891	-2.125816

ωB97XD energy = -1154.85050085 a.u.

(2*R*,6*R*,7*S*,8*R*)-**1**, Conf. G

C	-4.383991	-0.601519	-0.227432
C	-3.544598	0.630371	0.009493
C	-2.857664	1.324158	-0.905173
C	-2.955705	1.084816	-2.387718
C	-2.200575	-1.795252	0.293058
C	-3.496443	-1.856418	-0.477895
C	-0.406197	1.662594	-0.454746
C	-0.123803	0.705570	0.734242
C	0.104422	-0.765987	0.336594
C	-1.114590	-1.322690	-0.331616
O	0.578901	2.724270	-0.364459
C	1.425123	2.567029	0.665541
C	1.066664	1.331184	1.408847
O	2.316391	3.354928	0.889995
C	1.761033	0.904114	2.460586
C	-1.797716	2.312316	-0.469253
C	-2.280195	-2.051132	1.772538
O	-5.201821	-0.879985	0.908947
O	1.213289	-0.795008	-0.583621
C	2.157403	-1.739037	-0.419382
O	2.136718	-2.550404	0.484345
C	3.223349	-1.655111	-1.480795
C	4.486119	-0.878711	-1.035344
C	4.175516	0.579355	-0.693622
C	5.216682	-1.570258	0.117211
H	-5.027764	-0.476165	-1.107820
H	-3.376899	0.854099	1.063792
H	-2.029642	0.643980	-2.778644
H	-3.100713	2.033428	-2.917267
H	-3.280397	-1.899686	-1.549887
H	-4.094645	-2.736568	-0.217901
H	-3.776753	0.415662	-2.654943
H	-0.983370	0.704025	1.412745
H	0.382014	-1.329468	1.229340
H	-1.169721	-1.127376	-1.401734
H	1.500071	-0.010063	2.986291
H	2.619205	1.467223	2.816337
H	-2.010719	2.707300	0.530132
H	-0.211273	1.167857	-1.409533
H	-1.757420	3.158714	-1.163828
H	-2.855622	-2.961225	1.970683
H	-2.811152	-1.233454	2.276105
H	-1.298341	-2.161132	2.239840
H	-5.769045	-0.116903	1.069294
H	3.502735	-2.682568	-1.731480
H	2.800417	-1.180286	-2.369966

H	5.148211	-0.891481	-1.910152
H	3.574775	0.647137	0.219297
H	5.100774	1.138179	-0.519235
H	3.626588	1.079234	-1.498854
H	5.433659	-2.618280	-0.115482
H	4.621913	-1.549908	1.037190
H	6.166271	-1.065027	0.323208

ωB97XD energy = -1154.85037625 a.u.

(2*R*,6*R*,7*S*,8*R*)-**1**, Conf. H

C	-4.207732	-1.359030	-0.324304
C	-3.679977	0.054578	-0.282708
C	-3.036972	0.700950	-1.261521
C	-2.884658	0.169417	-2.661375
C	-1.895935	-1.901250	0.582336
C	-3.052612	-2.400577	-0.248053
C	-0.787754	1.683153	-0.712421
C	-0.438026	1.047107	0.660688
C	0.121832	-0.389337	0.584819
C	-0.867576	-1.317781	-0.046682
O	-0.080496	2.949440	-0.766783
C	0.687608	3.166016	0.311769
C	0.541070	2.026565	1.253722
O	1.367443	4.161662	0.425705
C	1.226808	1.949337	2.391756
C	-2.275715	1.971723	-0.957337
C	-2.094184	-1.886572	2.072395
O	-5.076750	-1.605088	0.781114
O	1.314389	-0.346264	-0.224232
C	2.489316	-0.696439	0.329123
O	2.598208	-1.122523	1.459813
C	3.628624	-0.444715	-0.624950
C	4.866771	-1.307414	-0.362508
C	4.581872	-2.783757	-0.648910
C	6.041893	-0.805324	-1.203259
H	-4.755624	-1.547411	-1.256761
H	-3.705924	0.509297	0.708305
H	-3.208568	0.921196	-3.390401
H	-3.458567	-0.743396	-2.834802
H	-2.710972	-2.588796	-1.270573
H	-3.477615	-3.330579	0.144747
H	-1.833649	-0.052924	-2.885776
H	-1.342538	0.994001	1.277108
H	0.399093	-0.704579	1.592096
H	-0.835768	-1.343970	-1.135040
H	1.125449	1.103881	3.065892
H	1.917267	2.740213	2.670778
H	-2.685311	2.474038	-0.074124
H	-0.377110	1.088296	-1.532381
H	-2.339782	2.668683	-1.800475
H	-2.857933	-1.149069	2.348791
H	-1.179079	-1.655367	2.623189
H	-2.465386	-2.857422	2.417154
H	-5.822525	-0.996741	0.722645
H	3.267872	-0.574537	-1.651110
H	3.873521	0.620524	-0.513780

H	5.127229	-1.203932	0.697353
H	5.460217	-3.399352	-0.427122
H	3.750826	-3.161283	-0.044437
H	4.329125	-2.931270	-1.706479
H	6.943258	-1.394585	-1.004202
H	6.271240	0.243730	-0.986109
H	5.820988	-0.886348	-2.274896

ωB97XD energy = -1154.84993550 a.u.

(2*R*,6*R*,7*S*,8*R*)-**1**, Conf. I

C	3.782012	-1.632179	0.107166
C	3.404032	-0.182665	0.291316
C	2.784333	0.360614	1.344943
C	2.509248	-0.374898	2.628622
C	1.476628	-1.758527	-0.958818
C	2.533810	-2.514366	-0.192292
C	0.682644	1.671569	0.893928
C	0.342675	1.303728	-0.576042
C	-0.369898	-0.051242	-0.756164
C	0.488192	-1.176838	-0.267601
O	0.117409	2.989016	1.123372
C	-0.555142	3.462195	0.062551
C	-0.486804	2.470766	-1.041674
O	-1.105906	4.540367	0.080857
C	-1.113226	2.649722	-2.202209
C	2.177836	1.741983	1.235811
C	1.742686	-1.512196	-2.418187
O	4.681594	-1.781924	-0.990933
O	-1.580285	-0.000047	0.021836
C	-2.734221	-0.387826	-0.549119
O	-2.821608	-0.741360	-1.707868
C	-3.872829	-0.353868	0.435060
C	-4.130541	-1.731432	1.094423
C	-4.646719	-2.763744	0.090689
C	-2.900744	-2.253865	1.841359
H	4.255803	-2.034710	1.012274
H	3.522263	0.422631	-0.608431
H	3.032796	-1.331545	2.689111
H	1.437113	-0.577057	2.746773
H	2.121305	-2.839190	0.767991
H	2.883856	-3.403021	-0.728076
H	2.810126	0.233425	3.489357
H	1.269941	1.242779	-1.156560
H	-0.638466	-0.165313	-1.807969
H	0.402005	-1.379394	0.798759
H	-1.070527	1.908868	-2.995601
H	-1.690498	3.552549	-2.379925
H	2.685383	2.333702	0.466032
H	0.164026	1.008158	1.590951
H	2.272373	2.275754	2.188070
H	2.062654	-2.436288	-2.910070
H	2.564442	-0.795342	-2.541679
H	0.869529	-1.125044	-2.949423
H	5.470242	-1.257146	-0.811370
H	-3.650416	0.385232	1.208989
H	-4.768071	-0.037968	-0.107234

H	-4.922466	-1.552211	1.832000
H	-3.891108	-2.995987	-0.667327
H	-4.906257	-3.696626	0.602457
H	-5.540667	-2.402366	-0.428414
H	-2.512502	-1.512061	2.547654
H	-2.093454	-2.513293	1.146260
H	-3.152900	-3.158543	2.404723

ωB97XD energy = -1154.84941384 a.u.

(2*R*,6*R*,7*S*,8*S*)-**1**, Conf. A

C	4.218595	-1.520843	-0.349823
C	3.651070	-0.219250	-0.838544
C	3.524459	0.913088	-0.134386
C	4.194450	1.205603	1.183108
C	1.868009	-1.896765	0.493125
C	3.062988	-2.541948	-0.172806
C	1.183651	1.692621	0.166360
C	0.098101	0.893517	-0.614058
C	-0.202462	-0.469417	0.073235
C	0.876909	-1.441643	-0.282866
O	0.579959	2.963123	0.519729
C	-0.688359	3.075944	0.097198
C	-1.058608	1.856477	-0.667259
O	-1.355416	4.059762	0.326034
C	-2.216291	1.768563	-1.320373
C	2.516911	1.959286	-0.561364
C	1.985169	-1.669924	1.973510
O	5.127070	-2.116730	-1.275297
O	-1.445769	-0.981536	-0.446493
C	-2.491591	-1.096319	0.391159
O	-2.448933	-0.792250	1.565360
C	-3.687790	-1.693235	-0.304888
C	-5.032411	-1.203539	0.245551
C	-5.224541	0.293505	-0.007070
C	-6.173657	-2.014576	-0.370283
H	4.720539	-1.385081	0.614628
H	3.123831	-0.292696	-1.790741
H	4.770585	2.134602	1.096143
H	4.875419	0.418894	1.512300
H	3.437827	-3.397244	0.399518
H	2.792169	-2.896758	-1.172855
H	3.458153	1.373197	1.978719
H	0.446926	0.683567	-1.630340
H	-0.317352	-0.321031	1.147237
H	0.919956	-1.662212	-1.349813
H	-2.468112	0.903329	-1.924003
H	-2.936700	2.579730	-1.262997
H	2.361461	1.956956	-1.644793
H	1.387746	1.201693	1.122475
H	2.855766	2.959368	-0.271817
H	1.089856	-1.227885	2.415603
H	2.836957	-1.016683	2.197989
H	2.176769	-2.622346	2.480940
H	5.870121	-1.514940	-1.398263
H	-3.610210	-1.501786	-1.380379

H	-3.597863	-2.779561	-0.170454
H	-5.030761	-1.372592	1.328711
H	-6.179808	0.637663	0.403143
H	-4.432248	0.887466	0.460309
H	-5.226063	0.509224	-1.083100
H	-7.139331	-1.696008	0.036102
H	-6.059575	-3.085123	-0.167504
H	-6.208963	-1.879560	-1.458576

ωB97XD energy = -1154.84705266 a.u.

(2*R*,6*R*,7*S*,8*S*)-**1**, Conf. B

C	4.061498	-1.650582	-0.335071
C	3.587913	-0.308344	-0.813390
C	3.516983	0.819046	-0.094002
C	4.180134	1.051806	1.238768
C	1.674246	-1.881945	0.452890
C	2.837410	-2.595101	-0.199966
C	1.224188	1.746914	0.178191
C	0.092918	1.042056	-0.629805
C	-0.295320	-0.317940	0.006763
C	0.737106	-1.345093	-0.336997
O	0.710831	3.053521	0.545002
C	-0.549810	3.255413	0.134245
C	-1.006680	2.073460	-0.640265
O	-1.149364	4.277663	0.382894
C	-2.187814	2.063773	-1.256170
C	2.585303	1.932340	-0.523862
C	1.773912	-1.683089	1.937851
O	4.949204	-2.289682	-1.251638
O	-1.557089	-0.706782	-0.574676
C	-2.405832	-1.408805	0.191798
O	-2.153092	-1.747346	1.330495
C	-3.700150	-1.707557	-0.519165
C	-4.921366	-1.230072	0.286834
C	-4.915609	0.291728	0.451207
C	-6.210406	-1.705439	-0.383351
H	4.549661	-1.563080	0.641858
H	3.074712	-0.334792	-1.775525
H	4.766594	0.201401	1.590586
H	3.445789	1.302152	2.013737
H	3.141470	-3.483293	0.364444
H	2.564661	-2.913958	-1.211506
H	4.853746	1.914075	1.162266
H	0.429096	0.851261	-1.654448
H	-0.439573	-0.193531	1.081528
H	0.791591	-1.547282	-1.407119
H	-2.510498	1.223625	-1.859907
H	-2.853895	2.917112	-1.162141
H	2.448619	1.954203	-1.609556
H	1.378330	1.228169	1.129280
H	2.983630	2.904285	-0.214628
H	1.900142	-2.651444	2.435436
H	0.897497	-1.195900	2.369487
H	2.658470	-1.083670	2.185442
H	5.735280	-1.739187	-1.343260
H	-3.691579	-1.247743	-1.511986

H	-3.747310	-2.794752	-0.651730
H	-4.858194	-1.687454	1.281527
H	-5.768510	0.619314	1.054858
H	-4.005989	0.649006	0.947005
H	-4.986413	0.789448	-0.523956
H	-7.086075	-1.401061	0.199813
H	-6.233274	-2.796317	-0.479966
H	-6.313638	-1.274983	-1.387188

ωB97XD energy = -1154.84689976 a.u.

(2*R*,6*R*,7*S*,8*S*)-**1**, Conf. C

C	2.410286	-3.036034	-0.327232
C	2.763324	-1.627687	-0.738796
C	3.318662	-0.687355	0.032983
C	3.869926	-0.952586	1.408581
C	0.222792	-1.898462	0.291709
C	1.146528	-3.056100	0.588186
C	2.094901	1.483977	0.311216
C	0.674683	1.220369	-0.269684
C	-0.241634	0.563098	0.791517
C	0.251399	-0.829267	1.099934
O	2.321393	2.912659	0.207088
C	1.294853	3.563887	-0.361419
C	0.236140	2.586463	-0.726027
O	1.309118	4.763351	-0.525553
C	-0.859308	2.956526	-1.387538
C	3.260100	0.765134	-0.384893
C	-0.529519	-1.989071	-1.007986
O	2.125076	-3.842595	-1.470553
O	-1.583822	0.590456	0.277242
C	-2.584475	0.518175	1.170358
O	-2.401176	0.448585	2.368538
C	-3.929287	0.543263	0.493352
C	-4.136718	-0.615068	-0.500080
C	-3.976532	-1.970551	0.190819
C	-5.511806	-0.490681	-1.157101
H	3.230152	-3.499330	0.236956
H	2.358664	-1.329837	-1.706491
H	4.018700	-2.017351	1.602630
H	3.197570	-0.568507	2.185975
H	1.483556	-2.998672	1.627472
H	0.657150	-4.025758	0.444413
H	4.831378	-0.444356	1.541875
H	0.743420	0.539195	-1.123275
H	-0.224433	1.169620	1.701671
H	0.841726	-0.893995	2.011739
H	-1.616412	2.238952	-1.683433
H	-1.010131	4.001370	-1.645141
H	3.141296	0.862737	-1.469471
H	2.118779	1.271167	1.383368
H	4.180819	1.285255	-0.098087
H	-1.079345	-1.079961	-1.249871
H	-1.246462	-2.817567	-0.963931
H	0.157621	-2.225587	-1.827589
H	2.911451	-3.866346	-2.027994
H	-4.690768	0.510130	1.277605

H	-4.022741	1.501711	-0.030914
H	-3.371430	-0.531182	-1.281309
H	-2.987494	-2.084376	0.648528
H	-4.726040	-2.092029	0.982339
H	-4.107402	-2.788072	-0.525909
H	-6.309229	-0.566827	-0.407916
H	-5.665762	-1.287607	-1.892314
H	-5.625232	0.469384	-1.672395

ωB97XD energy = -1154.84677159 a.u.

(2*R*,6*R*,7*S*,8*S*)-**1**, Conf. D

C	3.970446	-1.657297	-0.433766
C	3.457743	-0.357392	-0.980641
C	3.441209	0.825035	-0.352495
C	4.226920	1.173002	0.885186
C	1.655173	-1.805516	0.577698
C	2.756300	-2.572215	-0.121624
C	1.180899	1.767729	0.084056
C	-0.052549	1.047219	-0.543810
C	-0.358913	-0.273209	0.206411
C	0.657783	-1.309322	-0.163499
O	0.737322	3.100708	0.444507
C	-0.563424	3.305257	0.191886
C	-1.131265	2.097098	-0.459614
O	-1.113938	4.348761	0.464614
C	-2.378707	2.088049	-0.925091
C	2.453672	1.888841	-0.781485
C	1.872098	-1.523929	2.036772
O	4.777515	-2.380288	-1.362021
O	-1.658985	-0.730887	-0.229583
C	-2.335707	-1.527649	0.616111
O	-1.942039	-1.794077	1.733695
C	-3.610680	-2.057238	0.011603
C	-4.554682	-0.982082	-0.551737
C	-5.820224	-1.642599	-1.100021
C	-4.894181	0.068926	0.506408
H	4.536285	-1.494851	0.490654
H	2.857589	-0.463943	-1.885398
H	4.878874	0.368344	1.229586
H	3.568569	1.457433	1.714945
H	3.103728	-3.421067	0.477115
H	2.392606	-2.960758	-1.078903
H	4.855448	2.047432	0.677642
H	0.146984	0.805415	-1.593146
H	-0.406536	-0.082271	1.280175
H	0.623785	-1.572935	-1.220990
H	-2.786615	1.231954	-1.447967
H	-3.013908	2.958548	-0.786482
H	2.201293	1.801385	-1.842616
H	1.436837	1.289800	1.034149
H	2.869120	2.888433	-0.616420
H	2.030121	-2.464214	2.576863
H	1.034588	-1.003639	2.505197
H	2.777418	-0.921304	2.180140
H	5.552680	-1.844846	-1.566242
H	-3.327101	-2.755581	-0.785314

H	-4.116129	-2.630495	0.794218
H	-4.044110	-0.488739	-1.387187
H	-6.490575	-0.895384	-1.537671
H	-5.582931	-2.379127	-1.875332
H	-6.368889	-2.157252	-0.301739
H	-5.557437	0.836994	0.094667
H	-3.998422	0.573553	0.885433
H	-5.403738	-0.392884	1.360837

ωB97XD energy = -1154.84653295 a.u.

(2*R*,6*R*,7*S*,8*S*)-**1**, Conf. E

C	2.982054	-2.693231	-0.328690
C	3.017341	-1.312834	-0.917692
C	3.464749	-0.199530	-0.323100
C	4.318613	-0.153384	0.917160
C	0.804152	-1.898240	0.677819
C	1.509423	-3.054150	0.003762
C	1.775750	1.599873	0.043485
C	0.356170	1.365286	-0.555936
C	-0.428529	0.300452	0.250444
C	0.083775	-1.066293	-0.083276
O	1.886761	3.026483	0.282778
C	0.760148	3.694722	-0.003569
C	-0.247918	2.747268	-0.546174
O	0.661924	4.889318	0.167941
C	-1.433317	3.176810	-0.976635
C	2.988227	1.155455	-0.801295
C	1.120092	-1.688242	2.130632
O	3.437142	-3.700580	-1.230923
O	-1.806738	0.386486	-0.168140
C	-2.752209	0.072486	0.731448
O	-2.507362	-0.231358	1.881794
C	-4.134394	0.121835	0.138003
C	-4.750743	-1.286503	0.011378
C	-6.174562	-1.175785	-0.534252
C	-3.887862	-2.196648	-0.865767
H	3.571636	-2.738131	0.593992
H	2.428250	-1.204838	-1.829588
H	5.240675	0.399340	0.700001
H	4.600908	-1.139923	1.288730
H	1.503419	-3.954971	0.627127
H	1.015852	-3.295173	-0.943710
H	3.820152	0.385350	1.732001
H	0.443340	1.010470	-1.588738
H	-0.376936	0.531136	1.316091
H	-0.059945	-1.324295	-1.132817
H	-2.165789	2.504624	-1.407555
H	-1.681566	4.232009	-0.900007
H	2.721521	1.139018	-1.862636
H	1.836075	1.139503	1.034327
H	3.772397	1.907426	-0.663269
H	0.570609	-0.857402	2.576974
H	2.192597	-1.505131	2.269340
H	0.880423	-2.595428	2.697118
H	4.360729	-3.521454	-1.441112
H	-4.753078	0.735572	0.800506

H	-4.096558	0.600581	-0.845048
H	-4.797431	-1.719807	1.017965
H	-6.645246	-2.162423	-0.598588
H	-6.800580	-0.545450	0.106821
H	-6.174390	-0.739192	-1.540627
H	-4.354876	-3.180786	-0.977360
H	-2.891600	-2.351536	-0.436525
H	-3.760164	-1.767956	-1.867483

ωB97XD energy = -1154.84641382 a.u.

(2*R*,6*R*,7*S*,8*S*)-**1**, Conf. F

C	3.043201	-2.571027	-0.376532
C	2.962839	-1.204639	-0.993533
C	3.418164	-0.060128	-0.468027
C	4.380130	0.053735	0.685469
C	0.910834	-1.852406	0.774826
C	1.623016	-2.994841	0.084965
C	1.662134	1.641054	-0.002869
C	0.225269	1.326543	-0.517196
C	-0.470614	0.261503	0.367131
C	0.082009	-1.093307	0.048718
O	1.705155	3.070460	0.239511
C	0.537461	3.679988	-0.012521
C	-0.438177	2.680155	-0.519073
O	0.384207	4.868826	0.158169
C	-1.651046	3.044578	-0.932259
C	2.840400	1.262409	-0.924924
C	1.340724	-1.557301	2.183236
O	3.471402	-3.576397	-1.294587
O	-1.868409	0.271432	0.015611
C	-2.759383	-0.042457	0.969966
O	-2.455519	-0.240117	2.128839
C	-4.155176	-0.119460	0.410543
C	-4.304561	-1.179533	-0.698375
C	-3.864674	-2.560566	-0.207401
C	-5.749593	-1.203717	-1.197394
H	3.710517	-2.566177	0.492665
H	2.287949	-1.142970	-1.848523
H	4.720608	-0.909440	1.069288
H	3.942304	0.616254	1.518797
H	1.713309	-3.870847	0.736198
H	1.070785	-3.294928	-0.812101
H	5.264083	0.617701	0.363665
H	0.272494	0.935225	-1.538869
H	-0.373765	0.532975	1.419923
H	-0.134154	-1.403335	-0.974105
H	-2.357384	2.329903	-1.338803
H	-1.950330	4.087325	-0.868401
H	2.505326	1.221759	-1.966040
H	1.809439	1.181699	0.979605
H	3.589820	2.056578	-0.843317
H	2.386517	-1.226442	2.204159
H	1.285162	-2.468427	2.789230
H	0.729896	-0.792506	2.667208
H	4.364917	-3.359043	-1.584085
H	-4.828434	-0.344492	1.242450

H	-4.419241	0.868607	0.015739
H	-3.654944	-0.886912	-1.532293
H	-3.989489	-3.310407	-0.995794
H	-2.810428	-2.571694	0.092828
H	-4.461672	-2.875889	0.657061
H	-5.867410	-1.928060	-2.010291
H	-6.061503	-0.222810	-1.572769
H	-6.436814	-1.490795	-0.392258

ωB97XD energy = -1154.84633108 a.u.

(2*R*,6*R*,7*S*,8*S*)-**1**, Conf. G

C	3.512953	-2.289597	-0.349886
C	3.345812	-0.877941	-0.832788
C	3.565334	0.240660	-0.130017
C	4.308064	0.323843	1.178022
C	1.154128	-1.966246	0.495030
C	2.111374	-2.931612	-0.167967
C	1.550307	1.668844	0.171312
C	0.283176	1.221409	-0.616293
C	-0.405053	0.005245	0.066180
C	0.343977	-1.240800	-0.285140
O	1.341796	3.060440	0.523157
C	0.165727	3.539922	0.091691
C	-0.545105	2.478472	-0.668592
O	-0.182502	4.678182	0.311305
C	-1.685125	2.730493	-1.310103
C	2.906876	1.537656	-0.550328
C	1.327712	-1.777868	1.975541
O	4.204919	-3.120154	-1.282086
O	-1.738972	-0.127453	-0.463837
C	-2.778965	0.054792	0.367079
O	-2.661268	0.334903	1.543197
C	-4.098572	-0.157258	-0.325738
C	-4.855057	-1.369208	0.252809
C	-6.226506	-1.489883	-0.412165
C	-4.046490	-2.658476	0.089499
H	4.038271	-2.309733	0.611266
H	2.810346	-0.790705	-1.779191
H	5.149809	1.018662	1.071318
H	4.704101	-0.633997	1.519825
H	2.222979	-3.858447	0.404900
H	1.747826	-3.193926	-1.167146
H	3.672095	0.731302	1.973177
H	0.559474	0.919974	-1.631811
H	-0.479710	0.181222	1.139583
H	0.321810	-1.465928	-1.351633
H	-2.184783	1.978896	-1.911476
H	-2.136813	3.716300	-1.241698
H	2.762753	1.590134	-1.633972
H	1.597007	1.139877	1.127983
H	3.521800	2.393001	-0.251500
H	0.582604	-1.112865	2.417318
H	2.322534	-1.373208	2.197777
H	1.260090	-2.746108	2.484337
H	5.094132	-2.765813	-1.396789
H	-4.689743	0.752291	-0.175526

H	-3.935650	-0.293343	-1.399035
H	-5.003547	-1.184983	1.323693
H	-6.793757	-2.321706	0.018248
H	-6.815420	-0.575716	-0.281276
H	-6.124083	-1.675699	-1.488424
H	-4.595914	-3.514263	0.495115
H	-3.084469	-2.608403	0.611788
H	-3.842941	-2.858837	-0.969603

ωB97XD energy = -1154.84617687 a.u.

(2*R*,6*R*,7*S*,8*S*)-**1**, Conf. H

C	3.575768	-2.020130	-0.452058
C	3.278042	-0.590451	-0.831263
C	3.452958	0.492098	-0.065868
C	4.171593	0.476687	1.256818
C	1.148935	-1.937294	0.302446
C	2.498178	-2.578533	0.528655
C	1.439565	1.932614	0.337208
C	0.239162	1.113570	-0.222327
C	-0.283039	0.112302	0.836217
C	0.778114	-0.927263	1.101328
O	1.040728	3.326165	0.298535
C	-0.181684	3.503011	-0.226260
C	-0.736672	2.184679	-0.629656
O	-0.681292	4.600677	-0.333105
C	-1.888320	2.088021	-1.291164
C	2.766531	1.792044	-0.423223
C	0.417775	-2.397449	-0.930944
O	3.584283	-2.860571	-1.606653
O	-1.505737	-0.453517	0.335805
C	-2.309485	-1.050373	1.231122
O	-2.092975	-1.040735	2.425870
C	-3.463870	-1.760870	0.573312
C	-4.171596	-0.986195	-0.547814
C	-5.275424	-1.853576	-1.154999
C	-4.733106	0.341498	-0.035893
H	4.548094	-2.101815	0.050477
H	2.710991	-0.483945	-1.756866
H	4.705436	-0.459023	1.436428
H	3.471471	0.621545	2.089640
H	2.836488	-2.374395	1.548862
H	2.468422	-3.665482	0.393377
H	4.896161	1.297434	1.306300
H	0.560181	0.537678	-1.096035
H	-0.516091	0.652892	1.758243
H	1.410780	-0.696630	1.956398
H	-2.276747	1.133689	-1.627127
H	-2.463279	2.983023	-1.512897
H	2.569922	1.857008	-1.498888
H	1.597601	1.716006	1.396994
H	3.393434	2.645916	-0.143304
H	1.116964	-2.488223	-1.768616
H	-0.404995	-1.742883	-1.217218
H	0.006867	-3.401101	-0.762726
H	4.263075	-2.538772	-2.211153
H	-3.056032	-2.698739	0.173210

H	-4.172484	-2.024199	1.364098
H	-3.433407	-0.778145	-1.331441
H	-4.873004	-2.791507	-1.553355
H	-6.033471	-2.104147	-0.403011
H	-5.778153	-1.328661	-1.974350
H	-5.482850	0.169230	0.745842
H	-5.215443	0.898830	-0.846048
H	-3.950527	0.981913	0.385705

ωB97XD energy = -1154.84617597 a.u.

(2*R*,6*R*,7*S*,8*S*)-**1**, Conf. I

C	2.376031	-3.112433	-0.281938
C	2.811829	-1.713371	-0.642342
C	3.351993	-0.809564	0.182221
C	3.796106	-1.117739	1.587606
C	0.183821	-1.911808	0.181836
C	1.051943	-3.094689	0.542434
C	2.192326	1.402467	0.401910
C	0.818568	1.210301	-0.305855
C	-0.214892	0.572507	0.653708
C	0.203016	-0.838895	0.985327
O	2.484336	2.821891	0.353153
C	1.537006	3.528069	-0.282888
C	0.477941	2.603529	-0.765377
O	1.612243	4.729525	-0.412084
C	-0.535866	3.034218	-1.514633
C	3.382370	0.650576	-0.211705
C	-0.491925	-1.992441	-1.159816
O	2.140001	-3.889746	-1.456122
O	-1.497668	0.650492	0.009566
C	-2.583231	0.614561	0.798237
O	-2.523595	0.533693	2.008365
C	-3.863844	0.686547	0.008910
C	-4.773529	-0.527639	0.269375
C	-6.120369	-0.327411	-0.425926
C	-4.103555	-1.826575	-0.184392
H	3.135286	-3.616984	0.329735
H	2.485173	-1.383169	-1.629048
H	3.878491	-2.190381	1.777203
H	3.094851	-0.706718	2.325224
H	1.318018	-3.043790	1.602463
H	0.547179	-4.051090	0.366633
H	4.771182	-0.660313	1.789131
H	0.933930	0.542769	-1.165382
H	-0.266765	1.167016	1.570538
H	0.741789	-0.921011	1.927256
H	-1.290657	2.355675	-1.896271
H	-0.619775	4.089785	-1.758548
H	3.355640	0.770556	-1.300266
H	2.115339	1.166648	1.467071
H	4.295381	1.129380	0.159013
H	0.246650	-2.205928	-1.940810
H	-1.039731	-1.087583	-1.420611
H	-1.194539	-2.834186	-1.169409
H	2.959660	-3.930714	-1.962192
H	-4.375909	1.604068	0.320479

H	-3.637704	0.769762	-1.058366
H	-4.945816	-0.586766	1.350736
H	-6.618441	0.583653	-0.077218
H	-5.992066	-0.247833	-1.512512
H	-6.788939	-1.172090	-0.229097
H	-4.751618	-2.686579	0.014687
H	-3.156173	-1.999904	0.337986
H	-3.897410	-1.802368	-1.261603

ωB97XD energy = -1154.84616207 a.u.

(2*R*,6*R*,7*S*,8*S*)-**1**, Conf. J

C	3.820760	-1.881477	-0.321979
C	3.495311	-0.444608	-0.647804
C	3.504681	0.588767	0.200968
C	4.039768	0.513361	1.605859
C	1.319177	-1.963950	0.137780
C	2.666855	-2.553328	0.484236
C	1.389676	1.915680	0.438245
C	0.305352	1.072713	-0.294876
C	-0.269937	-0.020815	0.637697
C	0.803937	-1.032322	0.952111
O	0.927649	3.289749	0.413243
C	-0.240856	3.435482	-0.230629
C	-0.686956	2.113153	-0.741689
O	-0.779300	4.513950	-0.344627
C	-1.773268	1.988077	-1.502130
C	2.801418	1.876566	-0.164790
C	0.764777	-2.366711	-1.201809
O	4.009348	-2.640789	-1.516728
O	-1.411400	-0.586530	-0.028173
C	-2.327141	-1.201001	0.736582
O	-2.231137	-1.295284	1.943519
C	-3.483128	-1.727699	-0.071765
C	-4.805163	-1.026205	0.295592
C	-4.749125	0.471406	-0.013285
C	-5.967377	-1.695181	-0.438681
H	4.730738	-1.952246	0.288260
H	3.049653	-0.301948	-1.632906
H	3.227918	0.554722	2.343199
H	4.696408	1.366210	1.812251
H	2.870347	-2.398672	1.547973
H	2.715239	-3.629401	0.284540
H	4.602970	-0.403345	1.793636
H	0.748840	0.573579	-1.162115
H	-0.623706	0.445957	1.561815
H	1.318776	-0.839830	1.891324
H	-2.078471	1.029499	-1.905943
H	-2.376755	2.861183	-1.735412
H	2.726645	1.996287	-1.251039
H	1.434321	1.651608	1.498423
H	3.347848	2.738822	0.233166
H	1.519961	-2.219760	-1.982191
H	-0.142609	-1.828557	-1.473936
H	0.537929	-3.440107	-1.197004
H	4.738604	-2.250067	-2.012075
H	-3.275600	-1.607755	-1.139338

H	-3.565326	-2.798352	0.144145
H	-4.956202	-1.153528	1.374361
H	-3.932588	0.972299	0.519068
H	-4.605041	0.642142	-1.087127
H	-5.683118	0.961400	0.280608
H	-6.918708	-1.223707	-0.170944
H	-6.037379	-2.760346	-0.193204
H	-5.844539	-1.606313	-1.525187

ωB97XD energy = -1154.84603782 a.u.

(2*R*,6*R*,7*S*,8*S*)-**1**, Conf. K

C	4.070422	-1.506166	-0.403152
C	3.534539	-0.176663	-0.851403
C	3.441421	0.937569	-0.113967
C	4.121750	1.172054	1.209749
C	1.716176	-1.840609	0.447856
C	2.889532	-2.500568	-0.241368
C	1.120599	1.765058	0.211914
C	0.020541	1.012705	-0.595306
C	-0.323352	-0.349695	0.063354
C	0.733809	-1.342028	-0.310743
O	0.542499	3.038046	0.600087
C	-0.714968	3.198422	0.160411
C	-1.104849	2.012701	-0.644459
O	-1.359400	4.192784	0.409233
C	-2.245591	1.978207	-1.330770
C	2.461425	2.023698	-0.504457
C	1.843489	-1.652832	1.932230
O	4.954358	-2.100237	-1.353318
O	-1.595307	-0.772351	-0.467865
C	-2.305690	-1.645460	0.266581
O	-1.914744	-2.095426	1.324503
C	-3.634948	-1.949529	-0.369924
C	-4.642641	-0.787907	-0.231267
C	-5.956510	-1.173494	-0.911701
C	-4.866086	-0.401206	1.231525
H	4.584698	-1.409916	0.559438
H	3.001028	-0.207984	-1.802422
H	4.724617	2.086181	1.149752
H	4.780325	0.356889	1.514339
H	3.245280	-3.379127	0.307520
H	2.602095	-2.823685	-1.247588
H	3.391588	1.336755	2.011615
H	0.376482	0.813143	-1.611121
H	-0.434132	-0.222872	1.142253
H	0.765509	-1.537787	-1.383173
H	-2.501887	1.136773	-1.964671
H	-2.944227	2.808377	-1.270397
H	2.307855	2.065761	-1.587395
H	1.312460	1.244285	1.154595
H	2.825814	3.002971	-0.176927
H	0.969902	-1.178895	2.383678
H	2.726783	-1.048218	2.170753
H	1.986545	-2.625184	2.417712
H	5.715452	-1.518545	-1.461826
H	-3.475126	-2.168032	-1.430645

H	-4.035391	-2.844518	0.114852
H	-4.227020	0.082546	-0.752739
H	-6.678399	-0.351589	-0.860976
H	-5.801493	-1.420216	-1.967736
H	-6.408191	-2.045859	-0.423836
H	-5.618222	0.390808	1.307776
H	-3.948306	-0.029765	1.700206
H	-5.217689	-1.260375	1.815232

ωB97XD energy = -1154.84591610 a.u.

(2*R*,6*R*,7*S*,8*S*)-**1**, Conf. L

C	2.679985	-2.955061	-0.320528
C	2.928139	-1.597793	-0.906653
C	3.283041	-0.512953	-0.207034
C	3.786328	-0.554153	1.216582
C	0.526837	-1.969557	0.596626
C	1.151247	-3.147811	-0.117645
C	1.958014	1.623213	0.005106
C	0.483143	1.513444	-0.482390
C	-0.323326	0.430206	0.262507
C	0.107261	-0.941946	-0.151536
O	2.285120	3.035588	-0.068065
C	1.188172	3.803509	-0.133254
C	-0.007904	2.935461	-0.312115
O	1.245227	5.011129	-0.070304
C	-1.226032	3.474585	-0.345507
C	3.058691	0.873574	-0.764592
C	0.554419	-2.012645	2.097424
O	3.092574	-4.018437	-1.177128
O	-1.702921	0.599880	-0.150062
C	-2.660972	0.207277	0.703743
O	-2.432953	-0.198844	1.825562
C	-4.034899	0.316485	0.097117
C	-4.681842	-1.070574	-0.092697
C	-6.095609	-0.908135	-0.650970
C	-3.828322	-1.964140	-0.995811
H	3.176275	-3.056737	0.652021
H	2.560142	-1.469164	-1.925266
H	4.496024	0.261675	1.391272
H	4.293685	-1.492414	1.452183
H	0.994879	-4.082089	0.432375
H	0.709084	-3.267307	-1.112595
H	2.973722	-0.432261	1.942109
H	0.481007	1.268359	-1.552733
H	-0.262247	0.593926	1.340258
H	0.131136	-1.064966	-1.234263
H	-2.121102	2.888279	-0.507754
H	-1.332069	4.547637	-0.210161
H	2.810829	0.840687	-1.830448
H	2.003426	1.368841	1.069257
H	3.971144	1.470415	-0.650613
H	1.554579	-2.280422	2.457810
H	-0.128112	-2.793550	2.453349
H	0.251837	-1.073602	2.563887
H	4.041778	-3.935798	-1.323185
H	-4.647557	0.917880	0.776858

H	-3.972937	0.833905	-0.865017
H	-4.749727	-1.541346	0.895646
H	-6.585893	-1.881579	-0.756816
H	-6.716600	-0.287520	0.004307
H	-6.072443	-0.435928	-1.640768
H	-2.835699	-2.146473	-0.569050
H	-3.691279	-1.505275	-1.982756
H	-4.308473	-2.937622	-1.140165

ωB97XD energy = -1154.84586835 a.u.

(2*R*,6*R*,7*S*,8*S*)-**1**, Conf. M

C	3.009317	-2.608429	-0.393753
C	3.012547	-1.245357	-1.019357
C	3.277966	-0.104150	-0.371641
C	3.906246	-0.039197	1.000570
C	0.820831	-1.911166	0.688225
C	1.545104	-3.008373	-0.059627
C	1.672678	1.810270	-0.052743
C	0.197465	1.486934	-0.433655
C	-0.394693	0.329898	0.398279
C	0.185338	-0.980476	-0.033703
O	1.788161	3.255701	-0.122327
C	0.591043	3.859255	-0.129931
C	-0.475763	2.831340	-0.267052
O	0.477889	5.062330	-0.055749
C	-1.758512	3.191781	-0.288827
C	2.803795	1.224161	-0.915902
C	0.989698	-1.901013	2.180450
O	3.497661	-3.626522	-1.264980
O	-1.813299	0.307160	0.110331
C	-2.625768	-0.244444	1.025729
O	-2.244192	-0.622817	2.114585
C	-4.044156	-0.322407	0.524226
C	-4.182723	-1.090964	-0.803269
C	-3.606684	-2.503940	-0.686796
C	-5.650961	-1.125802	-1.227580
H	3.592277	-2.610715	0.535294
H	2.538359	-1.195034	-2.000276
H	4.575554	-0.880488	1.194472
H	3.149242	-0.034825	1.792989
H	1.568471	-3.940781	0.514724
H	1.044614	-3.213097	-1.012095
H	4.487104	0.882867	1.108480
H	0.155069	1.201793	-1.492782
H	-0.261645	0.528963	1.463778
H	0.128496	-1.129821	-1.111930
H	-2.558762	2.476620	-0.429725
H	-2.015433	4.240841	-0.168681
H	2.469993	1.135463	-1.954740
H	1.836647	1.560131	1.000423
H	3.621689	1.953238	-0.886606
H	0.604711	-0.996427	2.653717
H	2.045534	-2.019785	2.450135
H	0.457093	-2.755167	2.615155
H	4.415883	-3.425343	-1.478328
H	-4.639380	-0.802740	1.306063

H	-4.416146	0.701867	0.398665
H	-3.614472	-0.547460	-1.567676
H	-3.728095	-3.048549	-1.629068
H	-2.536833	-2.490003	-0.448243
H	-4.119253	-3.070045	0.100589
H	-5.765006	-1.631527	-2.192175
H	-6.064629	-0.116108	-1.326698
H	-6.257471	-1.668204	-0.492194

ωB97XD energy = -1154.84586682 a.u.

(2*R*,6*R*,7*S*,8*S*)-**1**, Conf. N

C	4.134780	-1.583246	0.344871
C	3.792882	-0.109568	0.287357
C	3.308019	0.594936	-0.743326
C	3.253634	0.117827	-2.166970
C	1.623843	-1.824447	0.708553
C	2.872171	-2.480815	0.168300
C	1.346597	1.732640	0.330188
C	0.135242	1.140674	-0.446425
C	-0.280186	-0.235036	0.126570
C	0.787806	-1.242673	-0.160388
O	0.919745	3.022630	0.835970
C	-0.354265	3.307960	0.528262
C	-0.914433	2.212767	-0.306387
O	-0.890674	4.328561	0.897958
C	-2.135936	2.295042	-0.831612
C	2.648938	1.934178	-0.454412
C	1.513072	-1.731951	2.204264
O	5.063161	-2.027592	-0.643531
O	-1.496632	-0.607550	-0.555882
C	-2.387259	-1.347602	0.122702
O	-2.219813	-1.706505	1.270786
C	-3.605062	-1.669896	-0.704440
C	-4.914062	-1.299742	0.012975
C	-5.002490	0.207829	0.261909
C	-6.111464	-1.790937	-0.801390
H	4.559088	-1.778411	1.338511
H	3.769609	0.369859	1.266820
H	3.631713	-0.898781	-2.281437
H	3.858071	0.778454	-2.801664
H	3.064242	-3.445279	0.650969
H	2.757604	-2.659846	-0.904817
H	2.229588	0.167546	-2.555807
H	0.391858	1.002712	-1.502044
H	-0.496508	-0.138610	1.192159
H	0.979151	-1.375611	-1.224736
H	-2.532806	1.521217	-1.478323
H	-2.759110	3.159675	-0.619746
H	2.437150	2.481891	-1.379505
H	1.560702	1.130197	1.217561
H	3.301224	2.562714	0.160418
H	1.652756	-2.722558	2.651357
H	0.548915	-1.345444	2.540229
H	2.302004	-1.087970	2.613836
H	5.860654	-1.491256	-0.567812
H	-3.539236	-1.161054	-1.670913

H	-3.579479	-2.749791	-0.892883
H	-4.917323	-1.812473	0.982243
H	-5.927710	0.459257	0.790773
H	-4.166569	0.573125	0.869153
H	-4.999778	0.758769	-0.686807
H	-7.050928	-1.568545	-0.284456
H	-6.066312	-2.872917	-0.966846
H	-6.145821	-1.300854	-1.782260

ωB97XD energy = -1154.84553303 a.u.

(2*R*,6*R*,7*S*,8*S*)-**1**, Conf. O

C	-4.139531	-1.436337	-0.181976
C	-3.742077	0.020814	-0.075799
C	-3.130723	0.647757	0.937718
C	-2.949449	0.084576	2.318537
C	-1.689645	-1.723399	-0.815783
C	-2.895193	-2.374763	-0.179959
C	-1.241264	1.778597	-0.267545
C	0.019586	1.110529	0.356107
C	0.330653	-0.234195	-0.343432
C	-0.745596	-1.220743	-0.011184
O	-0.820940	3.078953	-0.750952
C	0.481000	3.319154	-0.536472
C	1.077480	2.170910	0.195536
O	1.012793	4.344401	-0.899827
C	2.329408	2.210260	0.647496
C	-2.450177	1.977829	0.655541
C	-1.736999	-1.548410	-2.307386
O	-4.982744	-1.913364	0.865345
O	1.587969	-0.708656	0.187642
C	2.300590	-1.542106	-0.590071
O	1.983426	-1.820178	-1.728878
C	3.504875	-2.097882	0.125043
C	4.422820	-1.041870	0.761882
C	5.582825	-1.732037	1.480674
C	4.934139	-0.049108	-0.283602
H	-4.661558	-1.561327	-1.139770
H	-3.796642	0.555332	-1.025056
H	-1.885598	0.032246	2.578725
H	-3.391961	-0.906371	2.426307
H	-3.165548	-3.308396	-0.685492
H	-2.679097	-2.609417	0.866758
H	-3.416076	0.750462	3.055288
H	-0.148696	0.909101	1.418994
H	0.448676	-0.072039	-1.416325
H	-0.828948	-1.413465	1.057956
H	2.752833	1.396893	1.224639
H	2.952705	3.076568	0.443355
H	-2.125074	2.464804	1.581581
H	-1.562608	1.230212	-1.156820
H	-3.135656	2.662275	0.145356
H	-1.927740	-2.514112	-2.789015
H	-0.812006	-1.144177	-2.722899
H	-2.561298	-0.883680	-2.596393
H	-5.767710	-1.354657	0.897221
H	3.131664	-2.778675	0.900268

H	4.061677	-2.695024	-0.602900
H	3.837142	-0.495689	1.510848
H	6.230800	-0.996725	1.969140
H	5.221785	-2.426230	2.247367
H	6.197427	-2.302354	0.773694
H	5.564069	0.717296	0.180325
H	4.113056	0.463902	-0.797458
H	5.534021	-0.563443	-1.044287

ωB97XD energy = -1154.84539924 a.u.

(2*R*,6*R*,7*S*,8*S*)-**1**, Conf. P

C	2.805698	-2.747140	-0.316583
C	2.897119	-1.361724	-0.887779
C	3.380899	-0.273341	-0.275743
C	4.222149	-0.271980	0.974021
C	0.641673	-1.890838	0.669285
C	1.317758	-3.062545	-0.007337
C	1.745071	1.570507	0.090281
C	0.331426	1.389073	-0.540878
C	-0.510116	0.352775	0.245850
C	-0.042998	-1.029529	-0.091977
O	1.892686	2.988302	0.359832
C	0.798501	3.697931	0.047748
C	-0.221579	2.791822	-0.540803
O	0.734282	4.892586	0.234396
C	-1.373977	3.265039	-1.012836
C	2.958846	1.104400	-0.740535
C	0.949929	-1.701098	2.126619
O	3.238661	-3.758638	-1.225209
O	-1.874716	0.498119	-0.195699
C	-2.849731	0.187145	0.674911
O	-2.636204	-0.140406	1.825036
C	-4.212561	0.280888	0.041949
C	-4.702861	-1.069411	-0.536898
C	-3.789073	-1.588503	-1.649299
C	-4.901009	-2.127144	0.550248
H	3.381101	-2.823125	0.612857
H	2.322787	-1.220522	-1.804514
H	4.455313	-1.271695	1.344542
H	3.738619	0.285331	1.785384
H	1.273851	-3.969079	0.606130
H	0.829635	-3.277591	-0.963824
H	5.170329	0.240181	0.770583
H	0.427469	1.032296	-1.572068
H	-0.466823	0.574325	1.314044
H	-0.181151	-1.272958	-1.145762
H	-2.110414	2.620063	-1.477634
H	-1.590793	4.327137	-0.936731
H	2.712488	1.115207	-1.806888
H	1.773579	1.089689	1.072685
H	3.763400	1.828393	-0.574212
H	0.690944	-2.609272	2.682786
H	0.411147	-0.865056	2.576332
H	2.024277	-1.536858	2.275055
H	4.169805	-3.606972	-1.423307
H	-4.908839	0.616283	0.815213

H	-4.185988	1.029276	-0.754996
H	-5.682875	-0.851344	-0.979092
H	-4.229368	-2.472871	-2.121836
H	-3.630142	-0.833350	-2.426722
H	-2.808109	-1.877456	-1.255113
H	-5.348877	-3.030840	0.123255
H	-5.561564	-1.765186	1.345260
H	-3.948460	-2.411673	1.010260

ωB97XD energy = -1154.84516610 a.u.

(2*R*,6*R*,7*S*,8*S*)-**1**, Conf. Q

C	3.237883	-2.456607	0.323047
C	3.397311	-0.978273	0.039800
C	2.980136	-0.281958	-1.025486
C	2.495033	-0.880227	-2.315621
C	0.911978	-1.734046	1.066320
C	1.740265	-2.856511	0.488536
C	1.749246	1.613003	0.077657
C	0.283390	1.399503	-0.399235
C	-0.441770	0.342938	0.465283
C	0.165995	-1.003690	0.228764
O	1.883034	3.026648	0.371030
C	0.743644	3.709001	0.184107
C	-0.306669	2.783617	-0.317138
O	0.664430	4.897323	0.401991
C	-1.519931	3.225317	-0.645124
C	2.868906	1.228655	-0.898172
C	1.133764	-1.421127	2.519116
O	3.758643	-3.325484	-0.681741
O	-1.813803	0.324317	0.018147
C	-2.753509	-0.041098	0.905959
O	-2.517192	-0.251871	2.078063
C	-4.106457	-0.158621	0.255681
C	-4.145681	-1.219014	-0.862329
C	-3.701539	-2.588409	-0.343391
C	-5.549550	-1.286150	-1.464326
H	3.754541	-2.662514	1.269799
H	3.723362	-0.403007	0.907217
H	2.484159	-1.970680	-2.293069
H	3.147737	-0.562787	-3.138756
H	1.690117	-3.759244	1.107462
H	1.368949	-3.112245	-0.508247
H	1.490509	-0.515664	-2.559655
H	0.271198	1.049129	-1.436731
H	-0.419120	0.644556	1.514465
H	0.090492	-1.328787	-0.808595
H	-2.281586	2.563643	-1.041364
H	-1.762590	4.276971	-0.518946
H	2.681029	1.698844	-1.869891
H	1.916139	1.100016	1.029141
H	3.797237	1.652577	-0.501585
H	2.148902	-1.037126	2.684009
H	1.041647	-2.335696	3.115194
H	0.424759	-0.689610	2.912023
H	4.688316	-3.106997	-0.813594
H	-4.826095	-0.407534	1.040554

H	-4.374465	0.821103	-0.156472
H	-3.446966	-0.903717	-1.646643
H	-3.741729	-3.337070	-1.141510
H	-2.674391	-2.568714	0.038767
H	-4.354445	-2.926991	0.470381
H	-5.585886	-2.013173	-2.282324
H	-5.864036	-0.314938	-1.861933
H	-6.283354	-1.595628	-0.710305

ωB97XD energy = -1154.84501690 a.u.

(2*R*,6*R*,7*S*,8*S*)-**1**, Conf. R

C	3.219747	-2.556040	0.405204
C	3.430631	-1.071387	0.199534
C	3.140019	-0.327749	-0.875828
C	2.786826	-0.870430	-2.231526
C	0.844001	-1.812374	0.940280
C	1.706368	-2.929656	0.402909
C	1.840445	1.550799	0.176730
C	0.424992	1.391277	-0.450462
C	-0.401991	0.316301	0.292373
C	0.199692	-1.033966	0.062252
O	1.972127	2.947800	0.542015
C	0.869466	3.663356	0.276264
C	-0.144278	2.784168	-0.365113
O	0.791697	4.842842	0.538129
C	-1.309940	3.267948	-0.791691
C	3.040979	1.179198	-0.703252
C	0.925340	-1.560724	2.419611
O	3.824970	-3.395438	-0.577203
O	-1.724500	0.338854	-0.287720
C	-2.773042	0.154183	0.529933
O	-2.671346	0.031109	1.734122
C	-4.074988	0.093668	-0.223808
C	-4.727381	-1.297849	-0.104927
C	-6.089070	-1.294106	-0.799335
C	-3.817848	-2.387477	-0.677179
H	3.635750	-2.809195	1.389116
H	3.670209	-0.536252	1.119384
H	3.511552	-0.512774	-2.973891
H	1.805973	-0.502672	-2.556042
H	1.577570	-3.855395	0.974552
H	1.434540	-3.136635	-0.636496
H	2.784907	-1.960736	-2.256399
H	0.510078	1.087616	-1.499265
H	-0.481570	0.575519	1.349478
H	0.224320	-1.317019	-0.989602
H	-2.041365	2.644550	-1.293218
H	-1.543275	4.318652	-0.642286
H	2.951004	1.685153	-1.670900
H	1.903178	0.996248	1.117431
H	3.935611	1.571200	-0.208917
H	0.204598	-0.818744	2.768773
H	1.930542	-1.220799	2.701298
H	0.744014	-2.492683	2.966531
H	4.763156	-3.177709	-0.619402
H	-4.739318	0.849281	0.208661

H	-3.906267	0.339723	-1.276504
H	-4.881788	-1.499424	0.962005
H	-6.582260	-2.265860	-0.690032
H	-6.752595	-0.531113	-0.377808
H	-5.979636	-1.092817	-1.872165
H	-4.293572	-3.369979	-0.592236
H	-2.858561	-2.440689	-0.150135
H	-3.610180	-2.203627	-1.738489

ωB97XD energy = -1154.84497158 a.u.

(2*R*,6*R*,7*S*,8*S*)-**1**, Conf. S

C	3.980581	-1.632338	-0.323694
C	3.533526	-0.261178	-0.742522
C	3.460715	0.829367	0.031406
C	4.096984	0.988645	1.387477
C	1.566969	-1.876707	0.380127
C	2.744574	-2.569283	-0.269358
C	1.162956	1.744380	0.281172
C	0.069076	1.098316	-0.621567
C	-0.380093	-0.278165	-0.065327
C	0.656893	-1.301182	-0.413536
O	0.631566	3.022944	0.714665
C	-0.588302	3.278640	0.218163
C	-1.001507	2.157475	-0.663406
O	-1.188245	4.296164	0.484659
C	-2.122228	2.212528	-1.380560
C	2.548492	1.973234	-0.358206
C	1.621519	-1.743578	1.874844
O	4.886184	-2.231801	-1.249784
O	-1.619339	-0.616353	-0.721256
C	-2.421402	-1.496530	-0.097265
O	-2.150761	-1.988621	0.979683
C	-3.677399	-1.774960	-0.883062
C	-4.953384	-1.193899	-0.228738
C	-5.299719	-1.869211	1.099432
C	-4.867893	0.326108	-0.073601
H	4.444030	-1.602308	0.668569
H	3.039414	-0.231626	-1.714517
H	4.776643	1.849344	1.369276
H	4.671388	0.117622	1.707478
H	3.023141	-3.486219	0.261302
H	2.500424	-2.837259	-1.302748
H	3.346410	1.203773	2.157649
H	0.467109	0.937154	-1.628636
H	-0.570682	-0.203245	1.007246
H	0.744452	-1.458358	-1.489144
H	-2.403891	1.416347	-2.059665
H	-2.778715	3.074318	-1.296736
H	2.450618	2.068290	-1.444158
H	1.281775	1.161900	1.199581
H	2.937281	2.920684	0.029225
H	1.716683	-2.733921	2.334760
H	0.739569	-1.259279	2.298485
H	2.506534	-1.170259	2.177274
H	5.679418	-1.685686	-1.291642
H	-3.563645	-1.366890	-1.890591

H	-3.774416	-2.862685	-0.961382
H	-5.761261	-1.415954	-0.936989
H	-6.266289	-1.507462	1.467040
H	-5.365361	-2.956899	0.988457
H	-4.546590	-1.657177	1.864474
H	-5.804754	0.724787	0.329636
H	-4.681759	0.817357	-1.034349
H	-4.064901	0.615024	0.615704

ωB97XD energy = -1154.84491923 a.u.

(2*R*,6*R*,7*S*,8*S*)-**1**, Conf. T

C	3.464502	-2.413644	0.358041
C	3.574196	-0.908262	0.243891
C	3.287872	-0.125395	-0.804505
C	3.044774	-0.611382	-2.205222
C	1.014530	-1.873489	0.799977
C	1.985115	-2.895366	0.257588
C	1.799603	1.591382	0.268666
C	0.437235	1.361740	-0.448257
C	-0.345330	0.194529	0.198229
C	0.360474	-1.096469	-0.072098
O	1.800510	2.970587	0.715904
C	0.659613	3.613654	0.426951
C	-0.238634	2.703448	-0.332116
O	0.469714	4.763581	0.754673
C	-1.402615	3.127539	-0.821972
C	3.071413	1.358802	-0.556176
C	0.993724	-1.699764	2.292473
O	4.176296	-3.149678	-0.635628
O	-1.643261	0.163785	-0.434856
C	-2.708516	-0.116848	0.333065
O	-2.634141	-0.337105	1.524679
C	-3.990844	-0.084318	-0.459217
C	-5.001572	-1.152063	-0.019387
C	-6.342679	-0.923395	-0.717689
C	-4.469559	-2.560314	-0.296308
H	3.850814	-2.692732	1.346960
H	3.725429	-0.410982	1.202867
H	2.064574	-0.279556	-2.568156
H	3.107279	-1.697183	-2.286818
H	1.896737	-3.855037	0.778638
H	1.782481	-3.069022	-0.803419
H	3.788479	-0.175582	-2.884612
H	0.601513	1.117718	-1.503049
H	-0.484914	0.389801	1.262499
H	0.463028	-1.316695	-1.134246
H	-2.044639	2.484623	-1.413439
H	-1.724259	4.149173	-0.638867
H	2.998641	1.913012	-1.498330
H	1.854179	0.991245	1.181455
H	3.906495	1.781903	0.011306
H	1.950984	-1.301172	2.653066
H	0.854739	-2.671671	2.778824
H	0.198617	-1.034672	2.635042
H	5.098013	-2.867867	-0.614448
H	-4.415757	0.917152	-0.309325

H	-3.761375	-0.181886	-1.525186
H	-5.149554	-1.044549	1.061484
H	-7.079997	-1.666289	-0.396039
H	-6.747807	0.069665	-0.494472
H	-6.237200	-1.007758	-1.806474
H	-5.182305	-3.318721	0.044188
H	-3.519865	-2.743828	0.217690
H	-4.307090	-2.710765	-1.370926

ωB97XD energy = -1154.84475134 a.u.

(2*R*,6*R*,7*S*,8*S*)-**1**, Conf. U

C	2.092509	-3.264418	-0.287007
C	2.585234	-1.961824	-0.843270
C	3.043838	-0.941098	-0.108289
C	3.422561	-1.054724	1.348818
C	0.083161	-1.959657	0.566974
C	0.544630	-3.209729	-0.149379
C	2.097678	1.396193	0.058810
C	0.633976	1.513692	-0.458245
C	-0.341158	0.555993	0.252288
C	-0.105773	-0.860100	-0.172431
O	2.646839	2.736019	-0.043870
C	1.683772	3.665741	-0.120673
C	0.364968	2.992499	-0.277444
O	1.929752	4.850251	-0.079314
C	-0.759133	3.708056	-0.273082
C	3.080631	0.462827	-0.666229
C	-0.002900	-2.040364	2.063698
O	2.363635	-4.373913	-1.141086
O	-1.672723	0.924592	-0.187398
C	-2.694184	0.652803	0.642738
O	-2.539287	0.264272	1.783257
C	-4.028712	0.863068	-0.020593
C	-4.581789	-0.431966	-0.667987
C	-3.694429	-0.937526	-1.807857
C	-4.829704	-1.534155	0.363803
H	2.524401	-3.452760	0.703318
H	2.318715	-1.782814	-1.885654
H	4.235693	-0.359494	1.584236
H	3.753877	-2.059989	1.618305
H	0.217764	-4.115676	0.372173
H	0.132901	-3.240346	-1.163933
H	2.583794	-0.799726	2.006714
H	0.618700	1.285748	-1.532932
H	-0.281327	0.696620	1.333575
H	-0.023377	-0.964286	-1.253957
H	-1.735309	3.261259	-0.409627
H	-0.698895	4.783941	-0.131805
H	2.874483	0.471177	-1.741312
H	2.079653	1.167492	1.129825
H	4.075962	0.896362	-0.512724
H	0.909737	-2.483283	2.479901
H	-0.830986	-2.700550	2.348094
H	-0.174075	-1.073178	2.539366
H	3.319169	-4.448287	-1.243647
H	-4.725579	1.208581	0.747641

H	-3.934770	1.637551	-0.787164
H	-5.549813	-0.143292	-1.095509
H	-4.181302	-1.770504	-2.326252
H	-3.493445	-0.151752	-2.543821
H	-2.731238	-1.299962	-1.431086
H	-5.327601	-2.389012	-0.106218
H	-5.463855	-1.181800	1.184148
H	-3.890050	-1.893659	0.797780

ωB97XD energy = -1154.84468114 a.u.

(2*S*,6*R*,7*S*,8*S*)-**1**, Conf. A

C	-2.637269	-2.801024	-0.060164
C	-3.069615	-1.364834	-0.157764
C	-3.075073	-0.444995	0.816674
C	-2.908660	-0.727217	2.285802
C	-0.323673	-1.882584	-0.405463
C	-1.284526	-2.980780	-0.798751
C	-1.987589	1.451090	-0.448264
C	-0.567847	1.250721	0.148956
C	0.363925	0.537501	-0.859780
C	-0.183641	-0.823964	-1.214624
O	-2.110390	2.869663	-0.723801
C	-1.116561	3.585595	-0.171072
C	-0.145561	2.657642	0.469527
O	-1.084655	4.793996	-0.231406
C	0.878985	3.095538	1.198877
C	-3.153645	1.031020	0.454645
C	0.267694	-2.007833	0.971875
O	-3.551797	-3.693368	-0.695315
O	1.665047	0.494082	-0.248038
C	2.722030	0.340187	-1.062824
O	2.622534	0.261963	-2.270015
C	4.011302	0.282563	-0.287194
C	4.056770	-0.870140	0.732701
C	3.826329	-2.221027	0.052574
C	5.389756	-0.848800	1.481624
H	-2.510676	-3.097995	0.987227
H	-3.224893	-1.029206	-1.184753
H	-2.861891	-1.791774	2.523223
H	-2.005236	-0.246783	2.681207
H	-1.490728	-2.941318	-1.873393
H	-0.890218	-3.976061	-0.565822
H	-3.753352	-0.295995	2.836832
H	-0.613300	0.638474	1.052510
H	0.440267	1.142822	-1.767830
H	-0.665628	-0.864152	-2.188615
H	1.569818	2.409842	1.678217
H	1.041114	4.162040	1.329250
H	-3.129805	1.644112	1.362816
H	-2.082446	0.961183	-1.418368
H	-4.086741	1.265477	-0.068603
H	0.824883	-2.948567	1.052728
H	-0.525888	-2.051018	1.726998
H	0.943360	-1.190811	1.224317
H	-4.405976	-3.613012	-0.255731

H	4.822610	0.180125	-1.013500
H	4.136236	1.240822	0.230528
H	3.252487	-0.706990	1.460670
H	3.857416	-3.033092	0.786433
H	2.854624	-2.266169	-0.451842
H	4.601094	-2.414523	-0.699113
H	5.420396	-1.637458	2.240604
H	5.550381	0.110597	1.985517
H	6.227391	-1.013231	0.792954

ωB97XD energy = -1154.84905998 a.u.

(2*S*,6*R*,7*S*,8*S*)-**1**, Conf. B

C	-3.801218	-1.683917	0.112248
C	-3.573887	-0.207869	-0.051121
C	-3.116664	0.651429	0.869946
C	-2.989657	0.361599	2.341939
C	-1.330200	-1.898255	-0.310048
C	-2.692119	-2.463126	-0.643212
C	-1.364597	1.828372	-0.510428
C	-0.150822	1.066084	0.088134
C	0.341647	-0.044256	-0.871134
C	-0.769856	-1.024709	-1.156834
O	-0.873282	3.142952	-0.876590
C	0.358569	3.381431	-0.396218
C	0.851953	2.165330	0.304235
O	0.912727	4.446553	-0.549686
C	1.985911	2.159792	1.001967
C	-2.565109	1.999269	0.428037
C	-0.794051	-2.268902	1.046132
O	-5.037350	-2.113262	-0.456741
O	1.485141	-0.646310	-0.238767
C	2.328484	-1.334178	-1.025922
O	2.188541	-1.422948	-2.228537
C	3.432584	-1.984992	-0.233831
C	4.158073	-1.046685	0.744267
C	5.232762	-1.823572	1.505966
C	4.759220	0.155457	0.013525
H	-3.773184	-1.965905	1.171086
H	-3.634608	0.128300	-1.087907
H	-3.452127	-0.581350	2.640130
H	-3.466475	1.165556	2.915188
H	-2.894863	-2.362922	-1.714559
H	-2.774914	-3.523708	-0.380473
H	-1.937756	0.341121	2.652984
H	-0.421480	0.590843	1.033478
H	0.676157	0.410359	-1.808227
H	-1.261108	-0.863868	-2.113789
H	2.319124	1.275846	1.534952
H	2.602429	3.053103	1.051745
H	-2.246965	2.584146	1.298666
H	-1.695148	1.375422	-1.446108
H	-3.323925	2.587820	-0.098462
H	-0.573436	-3.343504	1.069519
H	-1.547425	-2.090601	1.821813
H	0.117229	-1.733124	1.312235
H	-5.753539	-1.653558	-0.003906

H	2.981534	-2.819870	0.317294
H	4.139268	-2.406475	-0.954639
H	3.420635	-0.682586	1.469559
H	5.738348	-1.179087	2.232677
H	4.802145	-2.672245	2.048548
H	5.992880	-2.213917	0.818381
H	5.252271	0.834206	0.717478
H	3.995966	0.732023	-0.521497
H	5.506596	-0.171700	-0.719489

ωB97XD energy = -1154.84843384 a.u.

(2*S*,6*R*,7*S*,8*S*)-**1**, Conf. C

C	-2.552084	-2.913200	-0.110916
C	-3.047577	-1.502358	-0.262554
C	-3.170684	-0.573686	0.695762
C	-3.101187	-0.834743	2.176761
C	-0.268862	-1.888665	-0.302032
C	-1.146183	-3.032773	-0.755582
C	-2.078603	1.352943	-0.513190
C	-0.701838	1.239648	0.196271
C	0.339663	0.558796	-0.724212
C	-0.113431	-0.829873	-1.108290
O	-2.248908	2.756016	-0.837429
C	-1.335118	3.534953	-0.234865
C	-0.377159	2.674363	0.510312
O	-1.355498	4.741329	-0.331105
C	0.559551	3.182119	1.308893
C	-3.288648	0.893420	0.308068
C	0.219072	-1.978558	1.117903
O	-3.376149	-3.860780	-0.788150
O	1.588022	0.577439	-0.007250
C	2.715318	0.517301	-0.734227
O	2.722528	0.469053	-1.947470
C	3.949598	0.496699	0.127807
C	4.711592	-0.836454	-0.005577
C	6.005910	-0.779653	0.805752
C	3.840115	-2.018325	0.424594
H	-2.478221	-3.187243	0.947805
H	-3.142094	-1.186546	-1.303112
H	-2.232381	-0.338241	2.626754
H	-3.057106	-1.895979	2.429480
H	-1.280317	-3.006110	-1.842030
H	-0.723053	-4.007542	-0.489072
H	-3.987119	-0.410274	2.664197
H	-0.784657	0.646949	1.110308
H	0.463683	1.159259	-1.630027
H	-0.516841	-0.898172	-2.116116
H	1.235472	2.545192	1.869506
H	0.660407	4.258376	1.418657
H	-3.357776	1.517553	1.206324
H	-2.071753	0.835473	-1.473207
H	-4.190409	1.080338	-0.284709
H	0.834449	-1.129616	1.414438
H	0.811110	-2.892260	1.247467
H	-0.627504	-2.055499	1.810569
H	-4.260289	-3.818782	-0.406125

H	4.590286	1.320653	-0.204128
H	3.676524	0.670885	1.172900
H	4.969494	-0.964281	-1.063866
H	6.567306	-1.714184	0.702968
H	6.652136	0.040007	0.473299
H	5.794759	-0.631488	1.872033
H	4.389021	-2.960538	0.325318
H	2.933698	-2.099243	-0.185511
H	3.534537	-1.914856	1.473030

ωB97XD energy = -1154.84841382 a.u.

(2*S*,6*R*,7*S*,8*S*)-**1**, Conf. D

C	-3.900610	-1.715949	0.017849
C	-3.644128	-0.257040	-0.234630
C	-3.294754	0.676789	0.660571
C	-3.350320	0.514153	2.155849
C	-1.402387	-1.946113	-0.143238
C	-2.724647	-2.550740	-0.555743
C	-1.400079	1.748864	-0.615721
C	-0.261926	1.021518	0.150060
C	0.319655	-0.139862	-0.691943
C	-0.752195	-1.154996	-1.007110
O	-0.858173	3.034512	-1.013260
C	0.318420	3.299317	-0.420144
C	0.721961	2.128881	0.404433
O	0.896465	4.350419	-0.581848
C	1.775496	2.160596	1.218038
C	-2.689733	1.987370	0.178528
C	-1.015964	-2.166627	1.293412
O	-5.073662	-2.187373	-0.644612
O	1.418263	-0.677026	0.068383
C	2.391800	-1.300844	-0.613558
O	2.373702	-1.440625	-1.819658
C	3.502817	-1.778492	0.283781
C	4.841744	-1.091043	-0.047690
C	4.770874	0.418322	0.193175
C	5.966997	-1.725908	0.769513
H	-3.988752	-1.916239	1.091796
H	-3.576979	-0.008029	-1.295235
H	-3.819650	-0.418230	2.475112
H	-3.918963	1.344016	2.592509
H	-2.824035	-2.547655	-1.646185
H	-2.831891	-3.584334	-0.208036
H	-2.347318	0.562799	2.597515
H	-0.634237	0.600637	1.086425
H	0.725078	0.261349	-1.625251
H	-1.138323	-1.087702	-2.021804
H	2.039983	1.309397	1.836154
H	2.394271	3.051714	1.279255
H	-2.458136	2.634352	1.032281
H	-1.635665	1.237116	-1.549775
H	-3.385489	2.537006	-0.464207
H	-0.869072	-3.238377	1.474323
H	-1.821815	-1.844428	1.962779
H	-0.098898	-1.649913	1.576896
H	-5.829247	-1.693239	-0.306351

H	3.237001	-1.606130	1.331155
H	3.599073	-2.858329	0.126911
H	5.044540	-1.263755	-1.111519
H	5.717601	0.897514	-0.076791
H	3.980977	0.894294	-0.398801
H	4.576866	0.633451	1.251196
H	6.929576	-1.261025	0.531964
H	6.052181	-2.799260	0.568651
H	5.790443	-1.595420	1.844402

ωB97XD energy = -1154.84813547 a.u.

(2*S*,6*R*,7*S*,8*S*)-**1**, Conf. E

C	-4.152933	-1.576966	0.169430
C	-3.791240	-0.119594	0.030639
C	-3.271984	0.687228	0.962543
C	-3.160969	0.339159	2.420079
C	-1.676391	-1.862454	-0.422542
C	-2.884517	-2.485533	0.236803
C	-1.354118	1.697096	-0.310660
C	-0.118220	1.150839	0.458970
C	0.269135	-0.258990	-0.045574
C	-0.784730	-1.240544	0.359310
O	-0.932643	2.934484	-0.938028
C	0.349008	3.238695	-0.682900
C	0.926288	2.209822	0.221522
O	0.878283	4.224966	-1.144498
C	2.155410	2.327817	0.721283
C	-2.613081	1.982850	0.516523
C	-1.662794	-1.856021	-1.924509
O	-4.918875	-2.009574	-0.955309
O	1.518062	-0.594748	0.596519
C	2.372172	-1.376986	-0.081793
O	2.145374	-1.803217	-1.196223
C	3.634370	-1.651683	0.694025
C	4.896369	-1.270277	-0.099533
C	4.938065	0.232786	-0.385465
C	6.145876	-1.718989	0.658705
H	-4.730758	-1.757300	1.085353
H	-3.798723	0.230389	-1.003088
H	-3.599531	1.133905	3.035006
H	-2.109802	0.252371	2.721383
H	-3.151873	-3.442961	-0.222783
H	-2.663187	-2.670223	1.292290
H	-3.660070	-0.600123	2.670111
H	-0.339327	1.077136	1.528935
H	0.432809	-0.231845	-1.124301
H	-0.910836	-1.314144	1.439505
H	2.559467	1.600038	1.415673
H	2.778344	3.173014	0.441070
H	-2.347504	2.609725	1.375218
H	-1.616003	1.028219	-1.135051
H	-3.291911	2.563441	-0.116781
H	-0.744815	-1.440448	-2.344840
H	-2.516001	-1.291135	-2.318130
H	-1.774347	-2.879337	-2.299784
H	-5.713337	-1.465835	-1.009827

H	3.606009	-1.118780	1.649324
H	3.644745	-2.726875	0.908139
H	4.858640	-1.804627	-1.056349
H	5.825387	0.490917	-0.972889
H	4.060412	0.568120	-0.949634
H	4.978826	0.804507	0.550027
H	7.051278	-1.479103	0.091056
H	6.138338	-2.799413	0.839368
H	6.217885	-1.214465	1.630180

ωB97XD energy = -1154.84752794 a.u.

(2*S*,6*R*,7*S*,8*S*)-**1**, Conf. F

C	-4.109849	-1.476776	0.311519
C	-3.716779	-0.022065	0.254212
C	-3.068276	0.684678	1.186099
C	-2.816170	0.210946	2.589345
C	-1.715165	-1.771204	-0.548913
C	-2.866294	-2.412784	0.190835
C	-1.267973	1.751073	-0.211285
C	0.038411	1.131474	0.366308
C	0.320251	-0.252648	-0.262947
C	-0.732033	-1.222938	0.175671
O	-0.896929	3.026366	-0.791982
C	0.411877	3.292452	-0.673203
C	1.071916	2.188091	0.072337
O	0.904006	4.304494	-1.119971
C	2.355609	2.256576	0.419756
C	-2.413514	1.994375	0.778892
C	-1.855588	-1.661331	-2.040058
O	-4.987267	-1.800988	-0.767183
O	1.602850	-0.687057	0.243809
C	2.269298	-1.596459	-0.488238
O	1.883722	-1.986124	-1.571623
C	3.519964	-2.075540	0.202914
C	4.481239	-0.956962	0.638705
C	5.701864	-1.566222	1.329751
C	4.896376	-0.087921	-0.549702
H	-4.608952	-1.715988	1.259709
H	-3.821612	0.415495	-0.739958
H	-1.741187	0.106655	2.780652
H	-3.290139	-0.749680	2.804383
H	-3.204166	-3.333239	-0.297234
H	-2.543272	-2.668967	1.204483
H	-3.191896	0.946807	3.310533
H	-0.059296	0.995743	1.448657
H	0.392716	-0.157441	-1.348001
H	-0.750682	-1.369280	1.255751
H	2.832049	1.475579	0.999969
H	2.952918	3.114785	0.123868
H	-2.029463	2.534039	1.651654
H	-1.640024	1.150770	-1.045570
H	-3.137364	2.647635	0.280525
H	-2.008056	-2.656590	-2.472578
H	-0.984168	-1.213492	-2.521358
H	-2.742347	-1.072135	-2.302306
H	-5.771801	-1.244519	-0.698648

H	3.204813	-2.653317	1.080603
H	4.023223	-2.760104	-0.485821
H	3.957064	-0.328464	1.368443
H	6.383644	-0.783096	1.678054
H	5.411545	-2.169944	2.196690
H	6.260158	-2.211688	0.640991
H	5.570678	0.713420	-0.229628
H	4.033506	0.380447	-1.036419
H	5.420080	-0.687854	-1.303840

ωB97XD energy = -1154.84729492 a.u.

(2*S*,6*R*,7*S*,8*S*)-**1**, Conf. G

C	-3.067491	-2.695173	0.156629
C	-3.334830	-1.212414	0.224848
C	-3.007981	-0.368579	1.209695
C	-2.515297	-0.789593	2.565650
C	-0.817043	-1.869075	-0.745553
C	-1.551814	-2.998451	-0.061675
C	-1.894352	1.481615	-0.087476
C	-0.425021	1.422583	0.421254
C	0.386711	0.343759	-0.331486
C	-0.127897	-1.016133	0.022627
O	-2.122256	2.844799	-0.526164
C	-1.038368	3.623470	-0.394123
C	0.064697	2.832790	0.214159
O	-1.038798	4.786512	-0.730558
C	1.233378	3.394708	0.518791
C	-2.996878	1.121929	0.916037
C	-1.061395	-1.702360	-2.217937
O	-3.773516	-3.280766	-0.937625
O	1.748089	0.468010	0.134044
C	2.728757	0.090611	-0.701206
O	2.530269	-0.285924	-1.838624
C	4.085304	0.172011	-0.053284
C	4.712755	-1.225257	0.125998
C	6.112044	-1.088076	0.726392
C	3.826148	-2.128138	0.987107
H	-3.370849	-3.196999	1.084554
H	-3.662517	-0.786510	-0.725282
H	-1.499361	-0.417743	2.745705
H	-2.505967	-1.873894	2.698768
H	-1.491170	-3.931862	-0.631506
H	-1.101863	-3.175264	0.919770
H	-3.149372	-0.356032	3.348463
H	-0.405814	1.175146	1.487896
H	0.365228	0.544890	-1.404555
H	-0.030290	-1.243246	1.084285
H	2.032869	2.836497	0.992543
H	1.400276	4.445366	0.297186
H	-2.855278	1.704333	1.833093
H	-2.011634	0.863008	-0.981680
H	-3.948225	1.433369	0.472526
H	-0.840786	-2.640228	-2.739356
H	-0.455019	-0.913338	-2.666901
H	-2.118646	-1.484393	-2.410651
H	-4.714810	-3.107412	-0.820304

H	4.722259	0.780739	-0.703461
H	4.001093	0.671364	0.916693
H	4.803922	-1.677208	-0.869204
H	6.590749	-2.067944	0.826257
H	6.755967	-0.461113	0.099987
H	6.065843	-0.634910	1.724339
H	4.292624	-3.109330	1.124148
H	2.843760	-2.291812	0.529907
H	3.668146	-1.687897	1.979498

ωB97XD energy = -1154.84724162 a.u.

(2*S*,6*R*,7*S*,8*S*)-**1**, Conf. H

C	-3.161098	-2.541488	0.218136
C	-3.335255	-1.047303	0.321036
C	-2.911451	-0.242400	1.301493
C	-2.374732	-0.721363	2.620576
C	-0.920947	-1.832992	-0.804543
C	-1.682127	-2.930768	-0.098243
C	-1.769079	1.564898	-0.030064
C	-0.279749	1.410664	0.391654
C	0.426580	0.301837	-0.420677
C	-0.144456	-1.034022	-0.062077
O	-1.941159	2.947917	-0.430601
C	-0.807341	3.659635	-0.349931
C	0.280536	2.792892	0.176581
O	-0.758952	4.827566	-0.665065
C	1.496919	3.279099	0.418501
C	-2.828709	1.250676	1.032641
C	-1.227417	-1.632478	-2.260831
O	-3.968185	-3.067758	-0.835588
O	1.814719	0.332263	-0.026637
C	2.734720	-0.030946	-0.935628
O	2.469662	-0.269833	-2.095956
C	4.107277	-0.109860	-0.321517
C	4.200813	-1.159123	0.804305
C	3.772032	-2.542615	0.310354
C	5.622611	-1.188804	1.366263
H	-3.434191	-3.038096	1.158302
H	-3.686340	-0.587551	-0.604573
H	-1.319059	-0.447225	2.737877
H	-2.457465	-1.803888	2.743784
H	-1.712078	-3.853551	-0.687568
H	-1.186699	-3.156188	0.850991
H	-2.914252	-0.241754	3.446110
H	-0.216368	1.144150	1.452214
H	0.358846	0.525185	-1.487062
H	-0.003742	-1.286767	0.988896
H	2.287284	2.664090	0.833778
H	1.713298	4.321889	0.202707
H	-2.596430	1.805592	1.948398
H	-1.974660	0.972841	-0.926204
H	-3.784796	1.626656	0.653669
H	-2.283919	-1.376222	-2.402999
H	-1.063414	-2.569955	-2.803801
H	-0.613878	-0.858692	-2.726261
H	-4.888727	-2.844339	-0.655322

H	4.811436	-0.350137	-1.122909
H	4.363795	0.879302	0.074983
H	3.517729	-0.851461	1.605349
H	3.854035	-3.283710	1.112495
H	2.733052	-2.549394	-0.039094
H	4.406202	-2.873348	-0.521209
H	5.699614	-1.906349	2.189945
H	5.925578	-0.206683	1.745617
H	6.341708	-1.488886	0.594496

ωB97XD energy = -1154.84715224 a.u.

(2*S*,6*R*,7*S*,8*S*)-**1**, Conf. I

C	-2.555199	-2.887005	-0.179576
C	-3.006395	-1.469029	-0.391293
C	-3.189206	-0.523338	0.540313
C	-3.244457	-0.759516	2.025742
C	-0.246984	-1.898881	-0.190177
C	-1.098707	-3.042254	-0.691320
C	-1.973237	1.369192	-0.608999
C	-0.654878	1.245879	0.204004
C	0.442262	0.526323	-0.618001
C	-0.000047	-0.864817	-1.005804
O	-2.099407	2.771139	-0.959802
C	-1.219148	3.544715	-0.302937
C	-0.326397	2.680126	0.515699
O	-1.215795	4.750108	-0.412856
C	0.568536	3.183787	1.363499
C	-3.249767	0.938730	0.123160
C	0.107022	-1.956039	1.270256
O	-3.332925	-3.826433	-0.920429
O	1.637176	0.542937	0.184691
C	2.814219	0.558103	-0.466370
O	2.900643	0.568962	-1.677574
C	3.985005	0.549515	0.481274
C	4.664872	-0.836755	0.589773
C	3.683253	-1.907531	1.069268
C	5.351003	-1.260231	-0.709740
H	-2.587021	-3.149517	0.884019
H	-3.006982	-1.167276	-1.440150
H	-3.234790	-1.816400	2.299623
H	-4.160759	-0.315282	2.432808
H	-1.134897	-3.043152	-1.785789
H	-0.716533	-4.014978	-0.362353
H	-2.407929	-0.264004	2.534313
H	-0.820069	0.676287	1.121357
H	0.642691	1.102185	-1.525737
H	-0.309750	-0.955363	-2.044656
H	1.197718	2.542764	1.972102
H	0.682690	4.259527	1.464704
H	-3.379182	1.577678	1.003995
H	-1.901517	0.841460	-1.561214
H	-4.099240	1.129925	-0.541295
H	0.682393	-2.866578	1.475992
H	-0.800772	-2.016656	1.881476
H	0.696430	-1.101751	1.601649
H	-4.245937	-3.767896	-0.616544

H	4.708290	1.281201	0.109319
H	3.645757	0.866243	1.470967
H	5.439787	-0.716910	1.357212
H	4.200325	-2.858237	1.235970
H	3.198191	-1.617129	2.006993
H	2.898939	-2.084714	0.323615
H	5.899236	-2.196531	-0.559385
H	6.062477	-0.501712	-1.052991
H	4.622434	-1.422733	-1.510867

ωB97XD energy = -1154.84685633 a.u.

H	4.452009	0.864954	0.283243
H	3.797441	-0.240933	1.497589
H	5.128060	-1.075041	-1.127905
H	7.053865	-1.786668	0.291034
H	6.763708	-0.047797	0.451576
H	6.237514	-1.157639	1.730289
H	5.111054	-3.378738	-0.167445
H	3.464097	-2.758836	-0.330181
H	4.246851	-2.784600	1.260593

ωB97XD energy = -1154.84669958 a.u.

(2*S*,6*R*,7*S*,8*S*)-**1**, Conf. J

C	-3.618615	-2.295968	0.179558
C	-3.630362	-0.790932	0.087290
C	-3.287938	0.088739	1.034664
C	-3.039077	-0.265203	2.473800
C	-1.172350	-1.946709	-0.500423
C	-2.164323	-2.865546	0.174675
C	-1.729222	1.581436	-0.253308
C	-0.367665	1.328796	0.454854
C	0.334881	0.075301	-0.122304
C	-0.427616	-1.148446	0.275645
O	-1.647569	2.905658	-0.838259
C	-0.472239	3.508045	-0.604085
C	0.375971	2.619367	0.234435
O	-0.219989	4.612394	-1.031055
C	1.558777	3.019186	0.698735
C	-2.986187	1.520750	0.622678
C	-1.224827	-1.891495	-2.000895
O	-4.297532	-2.870036	-0.937947
O	1.650058	0.014129	0.470829
C	2.711035	-0.125117	-0.340961
O	2.626357	-0.218341	-1.548456
C	4.006884	-0.127800	0.429320
C	4.985260	-1.210907	-0.049538
C	6.336809	-1.038129	0.644232
C	4.416596	-2.611466	0.190561
H	-4.100538	-2.640984	1.103451
H	-3.763401	-0.420950	-0.930893
H	-3.254068	-1.312869	2.696864
H	-1.995587	-0.068443	2.748732
H	-2.208414	-3.847271	-0.308799
H	-1.862895	-3.017373	1.215478
H	-3.658601	0.358617	3.129191
H	-0.521809	1.163674	1.526276
H	0.447578	0.180201	-1.202048
H	-0.486865	-1.286151	1.355227
H	2.161887	2.391959	1.346086
H	1.939835	4.002065	0.435356
H	-2.848642	2.164704	1.498466
H	-1.851470	0.897318	-1.097299
H	-3.812216	1.935259	0.035442
H	-0.449586	-1.256506	-2.434618
H	-2.202330	-1.530425	-2.342081
H	-1.108537	-2.899534	-2.414171
H	-5.204299	-2.541842	-0.943964

(2*S*,6*R*,7*S*,8*S*)-**1**, Conf. K

C	-3.839735	-1.688760	-0.042201
C	-3.571536	-0.243099	-0.351023
C	-3.264883	0.733714	0.513242
C	-3.388685	0.644646	2.010530
C	-1.336060	-1.926231	-0.047417
C	-2.634296	-2.547398	-0.509218
C	-1.305472	1.738939	-0.712205
C	-0.213030	1.062885	0.160263
C	0.419050	-0.141836	-0.577748
C	-0.640465	-1.167107	-0.903780
O	-0.741079	2.999122	-1.156503
C	0.395135	3.306600	-0.508136
C	0.745399	2.192656	0.413422
O	0.981425	4.348350	-0.698641
C	1.731831	2.288605	1.302092
C	-2.636568	2.019228	-0.005012
C	-1.026227	-2.101784	1.414321
O	-4.975729	-2.195146	-0.742471
O	1.465259	-0.640558	0.276565
C	2.366946	-1.462037	-0.289672
O	2.329510	-1.770996	-1.463371
C	3.412554	-1.925330	0.691275
C	4.767870	-1.195980	0.527390
C	5.435238	-1.483311	-0.818485
C	4.633988	0.308929	0.768089
H	-3.985150	-1.837465	1.033880
H	-3.453354	-0.044314	-1.417723
H	-2.406813	0.723036	2.493541
H	-3.867011	-0.274012	2.355000
H	-2.670025	-2.594198	-1.602699
H	-2.764053	-3.564276	-0.121874
H	-3.981301	1.490851	2.378725
H	-0.640682	0.693035	1.094449
H	0.882804	0.207843	-1.505438
H	-0.970449	-1.141640	-1.939848
H	1.953273	1.481807	1.991959
H	2.337535	3.189266	1.354355
H	-2.453368	2.714460	0.822232
H	-1.491332	1.172315	-1.625502
H	-3.296108	2.528831	-0.715383
H	-0.845144	-3.163317	1.624000
H	-1.883557	-1.805058	2.029491
H	-0.149741	-1.541821	1.740988
H	-5.748101	-1.686945	-0.469452

H	3.039270	-1.779429	1.708333
H	3.557610	-2.996701	0.523162
H	5.411385	-1.608179	1.314560
H	6.438917	-1.045614	-0.842472
H	5.530788	-2.559671	-0.996377
H	4.863716	-1.057829	-1.649527
H	5.613073	0.796130	0.713567
H	4.206775	0.518399	1.754340
H	3.989635	0.778347	0.014722

ωB97XD energy = -1154.84644806 a.u.

(2*S*,6*R*,7*S*,8*S*)-**1**, Conf. L

C	-3.493259	-2.272794	-0.077745
C	-3.549280	-0.783572	-0.275457
C	-3.381244	0.166473	0.653426
C	-3.371183	-0.067032	2.140260
C	-0.989607	-1.973430	-0.195079
C	-2.153763	-2.817146	-0.660729
C	-1.751232	1.665743	-0.560923
C	-0.483539	1.188816	0.199494
C	0.330675	0.175039	-0.638559
C	-0.517411	-1.017887	-1.006192
O	-1.510343	3.049617	-0.918961
C	-0.418411	3.552327	-0.319755
C	0.247659	2.476745	0.463975
O	-0.091022	4.710519	-0.448585
C	1.299141	2.718036	1.244591
C	-3.067222	1.591614	0.222997
C	-0.565398	-2.199158	1.230989
O	-4.597726	-2.958541	-0.660350
O	1.480769	-0.163302	0.157945
C	2.516573	-0.739475	-0.474283
O	2.548095	-0.910608	-1.675080
C	3.583169	-1.171692	0.498428
C	5.000518	-1.154188	-0.083976
C	5.439026	0.271585	-0.425443
C	5.974861	-1.806920	0.897654
H	-3.544624	-2.523878	0.983009
H	-3.548772	-0.478047	-1.325176
H	-2.383017	0.143153	2.568200
H	-3.658180	-1.081486	2.422875
H	-2.226039	-2.787989	-1.754576
H	-2.051420	-3.865170	-0.357986
H	-4.071723	0.624936	2.622616
H	-0.761947	0.696264	1.134067
H	0.684215	0.663234	-1.551719
H	-0.904809	-0.979942	-2.021938
H	1.767949	1.931406	1.825996
H	1.708613	3.722224	1.313533
H	-2.988709	2.244929	1.099228
H	-1.863652	1.139801	-1.510132
H	-3.860260	1.998641	-0.413361
H	-0.097705	-3.187645	1.321248
H	-1.434337	-2.205102	1.898232
H	0.146900	-1.457900	1.593318
H	-4.629807	-2.748488	-1.601699

H	3.523526	-0.550651	1.398635
H	3.310633	-2.192072	0.801352
H	4.990294	-1.744466	-1.007739
H	6.447370	0.276238	-0.852378
H	4.768176	0.738180	-1.154253
H	5.454082	0.899136	0.474689
H	6.990225	-1.818618	0.487736
H	5.689512	-2.841342	1.118772
H	6.004876	-1.255303	1.845669

ωB97XD energy = -1154.84615219 a.u.

(2*S*,6*R*,7*S*,8*S*)-**1**, Conf. M

C	-2.811627	-2.819445	0.107687
C	-3.167323	-1.354511	0.149361
C	-2.929857	-0.483427	1.136153
C	-2.462683	-0.860984	2.513920
C	-0.580939	-1.874429	-0.725019
C	-1.273987	-3.035573	-0.050993
C	-1.886434	1.414993	-0.146714
C	-0.435350	1.451449	0.411704
C	0.463279	0.416218	-0.301255
C	0.018851	-0.967490	0.055980
O	-2.178937	2.754557	-0.617336
C	-1.151773	3.601527	-0.453282
C	-0.025298	2.886499	0.203257
O	-1.212215	4.759231	-0.802432
C	1.098252	3.518414	0.538664
C	-2.999122	1.002374	0.824611
C	-0.770129	-1.745548	-2.209140
O	-3.441184	-3.454568	-1.005639
O	1.800437	0.624609	0.200470
C	2.823726	0.287784	-0.603084
O	2.674192	-0.081597	-1.750480
C	4.149829	0.403181	0.100117
C	4.621564	-0.940947	0.708840
C	3.650814	-1.467776	1.768331
C	4.889236	-2.000418	-0.361965
H	-3.119595	-3.329913	1.029453
H	-3.482868	-0.957717	-0.817384
H	-1.483137	-0.417178	2.729116
H	-2.381891	-1.941068	2.656357
H	-1.137636	-3.971753	-0.602757
H	-0.851243	-3.172503	0.948845
H	-3.156172	-0.468696	3.267727
H	-0.437099	1.217525	1.481371
H	0.461887	0.602895	-1.376994
H	0.081161	-1.170645	1.125113
H	1.912034	3.009968	1.043263
H	1.214085	4.574325	0.309805
H	-2.924777	1.602747	1.738102
H	-1.933551	0.775287	-1.032548
H	-3.951824	1.249782	0.345189
H	-0.467463	-2.674599	-2.704497
H	-0.195425	-0.926065	-2.645017
H	-1.829621	-1.597872	-2.450278
H	-4.394906	-3.340038	-0.921416

H	4.882625	0.742805	-0.636807
H	4.072937	1.152992	0.892409
H	5.572839	-0.711787	1.204698
H	4.071034	-2.349392	2.263633
H	3.441661	-0.714642	2.535614
H	2.696480	-1.764494	1.318124
H	5.325231	-2.896900	0.091798
H	5.585629	-1.634096	-1.123769
H	3.965082	-2.298171	-0.868852

ωB97XD energy = -1154.84605016 a.u.

(1*R*,5*S*,6*S*,7*S*,10*R*)-**2**, Conf. A

C	3.457263	0.014533	-0.232780
C	2.935023	1.391198	-0.671879
C	1.585630	1.654777	-0.053032
C	0.570777	0.582693	-0.402477
C	1.046135	-0.783477	0.183303
C	2.426697	-1.084769	-0.464149
C	-0.886285	0.927769	-0.065163
C	-1.849801	-0.190549	-0.478257
C	-1.405373	-1.529189	0.120982
C	0.039627	-1.868082	-0.243525
C	-3.327890	0.164217	-0.186559
C	-4.287093	-0.834058	-0.843086
C	-3.649464	0.301842	1.305453
O	-1.295124	2.100775	-0.770000
C	1.372628	2.677159	0.782180
O	3.007525	-2.293757	0.015063
H	0.599156	0.472558	-1.499237
C	1.161603	-0.746320	1.713640
H	3.721824	0.039879	0.830566
H	4.368796	-0.236490	-0.784548
H	3.651107	2.170398	-0.395236
H	2.836637	1.405726	-1.766040
H	2.257489	-1.176158	-1.549718
H	-0.978991	1.105840	1.016164
H	-1.757157	-0.273839	-1.572767
H	-2.059624	-2.327660	-0.243482
H	-1.520411	-1.511340	1.210982
H	0.107660	-2.002786	-1.332859
H	0.309551	-2.826691	0.217360
H	-3.502204	1.140196	-0.653200
H	-4.247326	-1.816579	-0.358893
H	-5.320509	-0.477414	-0.770471
H	-4.056471	-0.972956	-1.905751
H	-4.687415	0.625580	1.440006
H	-3.012495	1.041894	1.800871
H	-3.534830	-0.651707	1.833950
H	-0.641150	2.792736	-0.619195
H	0.423886	2.836897	1.289316
H	2.165467	3.385526	1.009963
H	2.467573	-3.037032	-0.274248
H	1.531621	-1.707099	2.084151
H	1.847110	0.032165	2.057067
H	0.194652	-0.554244	2.186841

ωB97XD energy = -737.573101461 a.u.

(1*R*,5*S*,6*S*,7*S*,10*R*)-**2**, Conf. B

C	3.409284	0.427086	-0.240900
C	2.671034	1.725021	-0.599638
C	1.308831	1.739680	0.046868
C	0.466012	0.544535	-0.359227
C	1.159580	-0.762627	0.134690
C	2.560848	-0.803987	-0.536442
C	-1.021158	0.637980	0.013894
C	-1.811865	-0.583318	-0.467735
C	-1.145327	-1.875988	0.022987
C	0.330138	-1.961422	-0.360662
C	-3.318479	-0.573273	-0.094752
C	-3.585225	-0.179344	1.362182
C	-4.182188	0.267680	-1.040355
O	-1.607695	1.788535	-0.594954
C	0.961471	2.665119	0.947668
O	3.328758	-1.935098	-0.138106
H	0.490991	0.506197	-1.460945
C	1.287236	-0.806436	1.663982
H	3.676108	0.433674	0.822055
H	4.343702	0.354918	-0.806662
H	3.261040	2.592240	-0.288714
H	2.551674	1.781425	-1.690470
H	2.391715	-0.852081	-1.624923
H	-1.118056	0.718551	1.106696
H	-1.744380	-0.576182	-1.566288
H	-1.676477	-2.731320	-0.409652
H	-1.257524	-1.965386	1.110430
H	0.407433	-2.012720	-1.456581
H	0.750996	-2.894648	0.035555
H	-3.649878	-1.614938	-0.211035
H	-2.972185	-0.754463	2.065153
H	-3.386004	0.885428	1.527737
H	-4.635260	-0.360370	1.616908
H	-3.969567	1.333854	-0.928397
H	-5.245917	0.102814	-0.831449
H	-4.002478	-0.001362	-2.087500
H	-1.040452	2.547714	-0.416406
H	0.012255	2.650000	1.477923
H	1.645308	3.469344	1.207896
H	2.897160	-2.734122	-0.459038
H	1.827846	-1.707295	1.969207
H	1.824836	0.059479	2.058047
H	0.307463	-0.822488	2.149566

ωB97XD energy = -737.571120275 a.u.

(1*R*,5*S*,6*R*,7*R*,8*R*,10*R*)-**3**, Conf. A

C	3.389482	-2.459176	0.007670
C	4.051201	-1.212103	0.612772
C	3.023448	-0.213969	1.087208
C	2.046540	0.154815	-0.005448
C	1.251825	-1.121655	-0.479082
C	2.321872	-2.106581	-1.028195

C	1.037156	1.243806	0.284141
C	0.260564	1.604147	-0.979512
C	-0.609568	0.461313	-1.450541
C	0.277226	-0.777781	-1.635986
O	1.596385	2.516813	0.700183
C	0.751170	3.508322	0.331179
C	-0.276738	2.949507	-0.604785
O	1.744066	-3.279195	-1.587346
C	2.985013	0.253258	2.336419
C	-1.397318	3.581117	-0.940412
O	0.886121	4.644175	0.720943
H	2.645584	0.489067	-0.867456
C	0.478270	-1.768339	0.682848
O	-1.640315	0.249490	-0.465149
C	-2.871494	-0.075468	-0.889684
O	-3.158373	-0.214449	-2.062084
C	-3.833054	-0.227374	0.260605
C	-3.375353	-1.243755	1.321637
C	-4.403088	-1.315042	2.451463
C	-3.140417	-2.621626	0.700485
H	2.936108	-3.056900	0.808848
H	4.144767	-3.089128	-0.472790
H	4.670518	-0.732806	-0.158451
H	4.718748	-1.498978	1.430364
H	2.809727	-1.611836	-1.877846
H	0.352835	0.937892	1.082620
H	1.006449	1.762181	-1.773163
H	-1.094229	0.701999	-2.398659
H	0.861385	-0.591591	-2.546417
H	-0.353747	-1.644917	-1.849693
H	1.476088	-3.867649	-0.871861
H	3.714389	-0.073231	3.073817
H	2.247472	0.979281	2.664884
H	-2.122233	3.129935	-1.612148
H	-1.613301	4.569334	-0.544342
H	-0.333941	-1.128751	1.031297
H	1.120678	-1.984017	1.539815
H	0.016505	-2.705277	0.354054
H	-3.954005	0.759440	0.723584
H	-4.797374	-0.524683	-0.161029
H	-2.429382	-0.884818	1.743538
H	-4.076596	-2.014836	3.227906
H	-4.550646	-0.336409	2.920987
H	-5.374286	-1.660336	2.076371
H	-2.388911	-2.587337	-0.095878
H	-4.067544	-3.017094	0.268118
H	-2.788542	-3.333708	1.454467

ωB97XD energy = -1154.88226567 a.u.

(1*R*,5*S*,6*R*,7*R*,8*R*,10*R*)-**3**, Conf. B

C	4.265695	-1.259219	0.432457
C	4.396169	0.233665	0.768801
C	3.054126	0.852517	1.073508
C	2.056876	0.625478	-0.038974
C	1.806379	-0.918208	-0.237672
C	3.184954	-1.529334	-0.615363

C	0.707331	1.300993	0.074299
C	-0.105942	1.100788	-1.202614
C	-0.467056	-0.352143	-1.413046
C	0.828073	-1.174461	-1.412765
O	0.741471	2.744961	0.216805
C	-0.403577	3.260783	-0.289851
C	-1.119132	2.194380	-1.061957
O	3.098406	-2.917921	-0.909140
C	2.791040	1.494839	2.213160
C	-2.378714	2.295592	-1.474839
O	-0.715753	4.416132	-0.122133
H	2.525123	0.990451	-0.966693
C	1.267186	-1.575461	1.046210
O	-1.353903	-0.731685	-0.344363
C	-2.205643	-1.746804	-0.545685
O	-2.274520	-2.361922	-1.591162
C	-3.036333	-2.010626	0.682949
C	-3.785039	-0.768354	1.199444
C	-4.777287	-0.249555	0.156968
C	-4.489346	-1.093795	2.517029
H	4.036360	-1.820903	1.346959
H	5.217815	-1.643650	0.052865
H	4.829282	0.752368	-0.098086
H	5.086468	0.372906	1.605621
H	3.493641	-1.068229	-1.562230
H	0.157892	0.924280	0.943244
H	0.550825	1.373456	-2.042118
H	-0.990658	-0.497815	-2.361152
H	1.337911	-0.937268	-2.355387
H	0.582036	-2.238235	-1.456789
H	3.015523	-3.406319	-0.081700
H	3.554995	1.602064	2.979579
H	1.825160	1.946801	2.417449
H	-2.858565	1.494227	-2.028695
H	-2.960036	3.188706	-1.263989
H	1.102645	-2.645504	0.878319
H	0.305252	-1.156383	1.345550
H	1.950498	-1.459821	1.890747
H	-3.740373	-2.811795	0.440756
H	-2.355955	-2.380367	1.460057
H	-3.042103	0.015628	1.388365
H	-5.550883	-0.998942	-0.050523
H	-5.273500	0.659789	0.511201
H	-4.285436	-0.009039	-0.792015
H	-3.776123	-1.430881	3.277071
H	-5.235182	-1.886444	2.379968
H	-5.006788	-0.212054	2.909218

ωB97XD energy = -1154.88212999 a.u.

(1*R*,5*S*,6*R*,7*R*,8*R*,10*R*)-**3**, Conf. C

C	4.452420	-0.671249	0.389649
C	4.310640	0.844241	0.596959
C	2.883367	1.230249	0.899033
C	1.925697	0.725256	-0.155070
C	1.960512	-0.849328	-0.212191
C	3.420376	-1.224752	-0.594201

C	0.478118	1.155061	-0.059554
C	-0.298952	0.704258	-1.294444
C	-0.405060	-0.807563	-1.351684
C	1.014903	-1.386172	-1.318431
O	0.257929	2.588538	-0.030790
C	-0.958342	2.856362	-0.561411
C	-1.476552	1.629114	-1.245876
O	3.591122	-2.624668	-0.777922
C	2.527434	1.909725	1.990810
C	-2.727451	1.503494	-1.678902
O	-1.468133	3.948846	-0.475522
H	2.304877	1.084522	-1.125090
C	1.587551	-1.471865	1.145372
O	-1.147783	-1.288623	-0.214026
C	-2.385447	-1.778933	-0.386130
O	-2.933493	-1.856572	-1.468115
C	-2.992645	-2.211795	0.923227
C	-3.673939	-1.065015	1.708971
C	-2.682770	-0.001737	2.188374
C	-4.822451	-0.431155	0.922096
H	4.347860	-1.183860	1.354553
H	5.452274	-0.905208	0.010546
H	4.621759	1.354658	-0.325219
H	4.980467	1.180841	1.393458
H	3.614773	-0.792796	-1.584294
H	0.015046	0.759091	0.850317
H	0.299390	0.994715	-2.171526
H	-0.917154	-1.132708	-2.259119
H	1.456833	-1.164665	-2.298028
H	0.953618	-2.475372	-1.245368
H	3.632735	-3.048370	0.087420
H	3.272656	2.224524	2.717290
H	1.497179	2.186553	2.193195
H	-3.069936	0.596372	-2.167271
H	-3.439539	2.312735	-1.542544
H	0.555753	-1.251445	1.422186
H	2.231493	-1.117713	1.953387
H	1.667544	-2.563012	1.091579
H	-3.735816	-2.978685	0.691793
H	-2.209201	-2.656537	1.543841
H	-4.100192	-1.549137	2.596512
H	-3.179743	0.700230	2.866176
H	-1.838748	-0.449857	2.724039
H	-2.283891	0.576493	1.348372
H	-5.541966	-1.184374	0.584191
H	-4.452732	0.098998	0.037255
H	-5.356922	0.294662	1.543774

ωB97XD energy = -1154.88198224 a.u.

(1*R*,5*S*,6*R*,7*R*,8*R*,10*R*)-**3**, Conf. D

C	2.885083	-3.046372	-0.010820
C	3.829223	-1.963560	0.531914
C	3.063308	-0.761888	1.028804
C	2.131194	-0.204859	-0.022276
C	1.062033	-1.286946	-0.436175
C	1.870027	-2.486320	-1.007359

C	1.390406	1.074534	0.299529
C	0.636512	1.582828	-0.926576
C	-0.470922	0.642822	-1.344655
C	0.133497	-0.752594	-1.557098
O	2.228265	2.204339	0.658332
C	1.585498	3.348779	0.324678
C	0.412044	3.012817	-0.544425
O	1.028300	-3.515646	-1.511251
C	3.189555	-0.282407	2.267697
C	-0.563814	3.867402	-0.835187
O	1.974445	4.432708	0.690773
H	2.742407	-0.008316	-0.917532
C	0.219924	-1.738061	0.770080
O	-1.476113	0.650058	-0.310298
C	-2.749427	0.427783	-0.668615
O	-3.094930	0.231298	-1.816993
C	-3.687131	0.453159	0.509867
C	-4.480831	-0.858438	0.648642
C	-3.549205	-2.041254	0.920052
C	-5.530695	-0.715281	1.750929
H	2.353481	-3.520540	0.824363
H	3.461699	-3.832134	-0.509336
H	4.497108	-1.640468	-0.279012
H	4.460712	-2.374239	1.324841
H	2.410124	-2.119899	-1.889729
H	0.704506	0.926122	1.140376
H	1.355883	1.581830	-1.759217
H	-0.938795	0.977908	-2.272645
H	0.705556	-0.692760	-2.491803
H	-0.670067	-1.471928	-1.735717
H	0.679187	-4.024554	-0.770199
H	3.870232	-0.747782	2.976554
H	2.642171	0.588583	2.614995
H	-1.403349	3.577838	-1.460762
H	-0.543415	4.881312	-0.445663
H	-0.429433	-0.940271	1.133550
H	0.838700	-2.069744	1.607032
H	-0.437713	-2.564269	0.480272
H	-3.125890	0.658891	1.426111
H	-4.381977	1.284149	0.343101
H	-4.996769	-1.034970	-0.302789
H	-2.995871	-1.895475	1.855913
H	-4.118008	-2.972902	1.007695
H	-2.820164	-2.176401	0.113419
H	-6.222640	0.106760	1.538451
H	-5.056470	-0.517698	2.720175
H	-6.118611	-1.633952	1.849009

ωB97XD energy = -1154.88193270 a.u.

(1*R*,5*S*,6*R*,7*R*,8*R*,10*R*)-**3**, Conf. E

C	4.308179	-1.010271	0.433312
C	4.264824	0.442628	0.929878
C	2.852769	0.898414	1.206368
C	1.943683	0.686338	0.017857
C	1.868726	-0.847280	-0.337698
C	3.322805	-1.261997	-0.702663

C	0.528418	1.212503	0.108292
C	-0.183982	1.071653	-1.235358
C	-0.401221	-0.385506	-1.588268
C	0.965085	-1.086029	-1.578296
O	0.415536	2.628834	0.399095
C	-0.729987	3.100582	-0.146177
C	-1.289258	2.070566	-1.078249
O	3.431256	-2.639709	-1.041283
C	2.467536	1.399451	2.381581
C	-2.516597	2.128648	-1.586567
O	-1.156697	4.201785	0.110593
H	2.420525	1.187224	-0.840152
C	1.362622	-1.687554	0.847093
O	-1.263706	-1.000974	-0.613965
C	-2.501030	-1.383722	-0.964997
O	-2.965282	-1.221271	-2.075782
C	-3.203640	-2.067751	0.179418
C	-3.171323	-1.291126	1.506944
C	-3.881741	0.057230	1.376765
C	-3.795868	-2.136304	2.617607
H	4.083850	-1.697366	1.256523
H	5.314890	-1.253867	0.079667
H	4.693735	1.094079	0.155200
H	4.886115	0.552991	1.823130
H	3.625793	-0.653620	-1.570258
H	-0.028570	0.692935	0.896080
H	0.500296	1.466795	-2.001196
H	-0.864819	-0.489113	-2.571186
H	1.490295	-0.729565	-2.474859
H	0.798677	-2.160461	-1.709092
H	3.020156	-2.789065	-1.899374
H	3.177164	1.503056	3.198990
H	1.451371	1.733733	2.567922
H	-2.892823	1.355843	-2.250381
H	-3.176022	2.954806	-1.335494
H	1.485418	-2.749970	0.616503
H	0.303683	-1.512970	1.044432
H	1.913195	-1.475596	1.766203
H	-4.235484	-2.253218	-0.132633
H	-2.713829	-3.040708	0.311971
H	-2.120983	-1.108055	1.763376
H	-3.429553	0.684563	0.601003
H	-4.938734	-0.084901	1.120372
H	-3.836631	0.612378	2.319589
H	-3.277431	-3.094532	2.732223
H	-4.850993	-2.346611	2.403989
H	-3.748145	-1.610939	3.577131

ωB97XD energy = -1154.88165091 a.u.

(1*R*,5*S*,6*R*,7*R*,8*R*,10*R*)-**3**, Conf. F

C	4.411732	-1.332315	0.417801
C	4.529327	0.151074	0.797886
C	3.182723	0.750052	1.122098
C	2.184582	0.543305	0.006231
C	1.948360	-0.996272	-0.234174
C	3.331762	-1.580715	-0.635806

C	0.829171	1.204698	0.137449
C	0.022051	1.037462	-1.148331
C	-0.331566	-0.414101	-1.400680
C	0.967485	-1.227824	-1.412840
O	0.856810	2.643143	0.324430
C	-0.281561	3.173850	-0.181257
C	-0.987291	2.133650	-0.996017
O	3.259422	-2.960431	-0.973067
C	2.918927	1.361947	2.278174
C	-2.232438	2.267720	-1.443633
O	-0.595420	4.323902	0.017487
H	2.648581	0.937839	-0.911637
C	1.423034	-1.694051	1.033182
O	-1.202546	-0.893114	-0.354972
C	-2.473830	-1.203130	-0.652290
O	-2.956761	-1.076773	-1.759946
C	-3.209828	-1.749615	0.544069
C	-4.598734	-1.120442	0.733762
C	-5.359781	-1.857676	1.836428
C	-4.485868	0.372276	1.050760
H	4.187340	-1.922678	1.315353
H	5.366915	-1.697035	0.026661
H	4.956824	0.698730	-0.053857
H	5.219749	0.271645	1.637545
H	3.632238	-1.086724	-1.568651
H	0.280257	0.797633	0.993236
H	0.685162	1.327950	-1.977139
H	-0.848656	-0.529568	-2.354922
H	1.477583	-0.967386	-2.349159
H	0.724764	-2.291562	-1.479742
H	3.193696	-3.476120	-0.160834
H	3.684975	1.455707	3.044214
H	1.950493	1.801343	2.497064
H	-2.709981	1.489933	-2.032328
H	-2.802195	3.165471	-1.220298
H	0.442911	-1.317676	1.329625
H	2.095602	-1.567916	1.884808
H	1.297667	-2.766249	0.846790
H	-3.311497	-2.828586	0.372593
H	-2.600394	-1.613655	1.442382
H	-5.147445	-1.237458	-0.208297
H	-4.831594	-1.781286	2.794908
H	-6.357678	-1.427449	1.972078
H	-5.481043	-2.920695	1.601373
H	-3.933145	0.529760	1.985283
H	-5.476984	0.823246	1.167673
H	-3.965736	0.919605	0.256929

ωB97XD energy = -1154.88160085 a.u.

(1*R*,5*S*,6*R*,7*R*,8*R*,10*R*)-**3**, Conf. G

C	4.382171	-0.963462	0.522459
C	4.303037	0.512167	0.944445
C	2.878513	0.944759	1.191714
C	1.986483	0.656196	0.007002
C	1.937244	-0.894210	-0.268360
C	3.399388	-1.308127	-0.597950

C	0.564231	1.170820	0.052848
C	-0.134771	0.942136	-1.285571
C	-0.326489	-0.539497	-1.550077
C	1.052802	-1.209654	-1.503466
O	0.437141	2.600175	0.260089
C	-0.706998	3.028665	-0.322449
C	-1.246550	1.944282	-1.203762
O	3.506019	-2.678380	-0.963257
C	2.469343	1.485081	2.341035
C	-2.461660	1.980511	-1.742942
O	-1.147983	4.137226	-0.128795
H	2.465815	1.116118	-0.871938
C	1.428437	-1.672671	0.958327
O	-1.168193	-1.122262	-0.534602
C	-2.440608	-1.433827	-0.821911
O	-2.950622	-1.251620	-1.909889
C	-3.150403	-2.021498	0.368648
C	-4.009532	-0.982607	1.122220
C	-4.803296	-1.690878	2.221453
C	-3.162298	0.150267	1.705599
H	4.182721	-1.604253	1.390651
H	5.394333	-1.200181	0.179205
H	4.718340	1.130535	0.136402
H	4.918984	0.684274	1.831668
H	3.693260	-0.761340	-1.502885
H	0.003883	0.694471	0.864552
H	0.553981	1.294996	-2.067628
H	-0.792951	-0.709316	-2.522372
H	1.579744	-0.880716	-2.408159
H	0.927044	-2.291693	-1.594011
H	3.449762	-3.218646	-0.166532
H	3.167715	1.643789	3.159349
H	1.442789	1.799304	2.503962
H	-2.829058	1.172604	-2.367840
H	-3.119220	2.823774	-1.550405
H	0.395386	-1.420677	1.201072
H	2.032903	-1.484564	1.848271
H	1.442697	-2.748466	0.751767
H	-3.796610	-2.820871	-0.004890
H	-2.415952	-2.453633	1.055510
H	-4.719480	-0.554053	0.403216
H	-5.448736	-0.983593	2.752552
H	-5.438257	-2.483591	1.811647
H	-4.127468	-2.143753	2.957440
H	-3.791606	0.848123	2.267819
H	-2.652559	0.723013	0.925202
H	-2.402621	-0.243480	2.392446

ωB97XD energy = -1154.88157830 a.u.

(1*R*,5*S*,6*R*,7*R*,8*R*,10*R*)-**3**, Conf. H

C	4.267375	-1.027402	0.371578
C	4.232491	0.411531	0.909476
C	2.820726	0.852637	1.211658
C	1.906295	0.682408	0.020328
C	1.827752	-0.837823	-0.390593
C	3.272469	-1.239943	-0.770301

C	0.490945	1.204878	0.131421
C	-0.218580	1.117277	-1.218838
C	-0.437913	-0.327069	-1.625184
C	0.924115	-1.033212	-1.638479
O	0.378343	2.607252	0.480824
C	-0.762421	3.104568	-0.050498
C	-1.318094	2.119173	-1.031920
O	3.260639	-2.597978	-1.196201
C	2.442557	1.306404	2.408148
C	-2.534162	2.223167	-1.560175
O	-1.187904	4.194534	0.252951
H	2.380637	1.212733	-0.820755
C	1.318533	-1.714067	0.767612
O	-1.310332	-0.973349	-0.676901
C	-2.574915	-1.248440	-1.024695
O	-3.039018	-1.007170	-2.121806
C	-3.329466	-1.941484	0.081608
C	-2.984734	-1.518747	1.516727
C	-3.256614	-0.030381	1.744937
C	-3.778595	-2.376802	2.503909
H	4.044292	-1.737258	1.175549
H	5.276767	-1.258509	0.010457
H	4.652738	1.085287	0.149395
H	4.862467	0.496547	1.799397
H	3.571908	-0.602048	-1.618447
H	-0.067550	0.653818	0.896153
H	0.469835	1.536351	-1.968183
H	-0.898095	-0.393629	-2.612700
H	1.452526	-0.649379	-2.520508
H	0.769070	-2.101877	-1.807669
H	4.164295	-2.860230	-1.402966
H	3.158131	1.381222	3.223552
H	1.426566	1.628541	2.615473
H	-2.911988	1.488296	-2.264252
H	-3.181528	3.050913	-1.283974
H	0.265177	-1.524751	0.979189
H	1.878473	-1.544853	1.690062
H	1.415734	-2.769163	0.497296
H	-4.395037	-1.790948	-0.118893
H	-3.130087	-3.013719	-0.047186
H	-1.917287	-1.706217	1.680615
H	-4.314921	0.202036	1.572659
H	-3.013727	0.255305	2.773997
H	-2.663138	0.599790	1.074755
H	-3.573203	-3.443635	2.362752
H	-4.856870	-2.221911	2.375306
H	-3.525595	-2.119064	3.537665

ωB97XD energy = -1154.88147706 a.u.

(1*R*,5*S*,6*R*,7*R*,8*R*,10*R*)-**3**,Conf. I

C	4.200622	-1.653984	0.338087
C	4.496753	-0.193664	0.711822
C	3.236915	0.552922	1.074803
C	2.191770	0.463077	-0.012581
C	1.773533	-1.039440	-0.243947
C	3.069257	-1.778332	-0.683326

C	0.927197	1.276364	0.154666
C	0.070471	1.202434	-1.105937
C	-0.458030	-0.195291	-1.335903
C	0.737088	-1.157851	-1.391388
O	1.123444	2.702949	0.334896
C	0.032931	3.356098	-0.131965
C	-0.808501	2.399947	-0.920401
O	2.830430	-3.140068	-1.015390
C	3.074679	1.183582	2.239349
C	-2.052598	2.651918	-1.314201
O	-0.147117	4.533097	0.074746
H	2.672372	0.802133	-0.943930
C	1.209322	-1.673814	1.040447
O	-1.349105	-0.508488	-0.246466
C	-2.352631	-1.368606	-0.469593
O	-2.578028	-1.865833	-1.555418
C	-3.162263	-1.628255	0.773622
C	-4.613923	-1.130322	0.638984
C	-5.410659	-1.519641	1.884460
C	-4.661673	0.380663	0.402113
H	3.939738	-2.218545	1.242497
H	5.096870	-2.121740	-0.081513
H	4.958164	0.302627	-0.153540
H	5.220928	-0.153361	1.530618
H	3.397658	-1.321410	-1.625534
H	0.358304	0.937280	1.026634
H	0.736464	1.418360	-1.954735
H	-1.021162	-0.258960	-2.269706
H	1.240630	-0.958662	-2.346083
H	0.370502	-2.186099	-1.447241
H	2.724195	-3.645551	-0.201032
H	3.866357	1.187004	2.984628
H	2.166377	1.724552	2.487093
H	-2.621275	1.924546	-1.886018
H	-2.530876	3.596942	-1.073086
H	0.283500	-1.192924	1.359242
H	1.914051	-1.617755	1.873261
H	0.968010	-2.727652	0.863208
H	-3.160229	-2.711767	0.934477
H	-2.681095	-1.151609	1.632972
H	-5.058813	-1.632345	-0.228935
H	-5.395355	-2.602876	2.046788
H	-4.997960	-1.037891	2.779287
H	-6.456242	-1.208623	1.789387
H	-5.696915	0.731311	0.336534
H	-4.160539	0.661604	-0.530330
H	-4.172201	0.921336	1.221455

ωB97XD energy = -1154.88138391 a.u.

C	1.034922	1.278345	0.182198
C	0.152498	1.289229	-1.064307
C	-0.483009	-0.060505	-1.309446
C	0.637127	-1.106476	-1.400600
O	1.343622	2.682789	0.377847
C	0.297902	3.424489	-0.057982
C	-0.628925	2.546959	-0.842464
O	2.575551	-3.250323	-1.087176
C	3.194032	0.985471	2.232999
C	-1.860225	2.899147	-1.198501
O	0.211636	4.608106	0.169841
H	2.723577	0.689942	-0.949303
C	1.098274	-1.699448	1.015566
O	-1.388687	-0.314357	-0.216534
C	-2.376214	-1.200421	-0.407015
O	-2.566821	-1.768906	-1.463918
C	-3.203862	-1.398579	0.836020
C	-4.703050	-1.156592	0.591701
C	-5.494168	-1.497047	1.855173
C	-4.964879	0.283952	0.147161
H	3.788348	-2.460124	1.166173
H	4.928217	-2.423077	-0.175654
H	4.975162	0.003949	-0.197119
H	5.222254	-0.502891	1.474437
H	3.272618	-1.470316	-1.675461
H	0.457413	0.972355	1.060858
H	0.819551	1.469523	-1.920770
H	-1.059814	-0.065172	-2.237709
H	1.140576	-0.922775	-2.358490
H	0.198041	-2.104301	-1.473238
H	2.441540	-3.760935	-0.280049
H	3.992026	0.918436	2.968574
H	2.333296	1.591559	2.499248
H	-2.501029	2.227507	-1.762084
H	-2.256234	3.874498	-0.930355
H	0.224496	-1.146806	1.364056
H	1.819919	-1.721792	1.835126
H	0.762533	-2.724319	0.823046
H	-3.044767	-2.435113	1.156162
H	-2.836906	-0.739951	1.628748
H	-5.022898	-1.831614	-0.211219
H	-5.332747	-2.536848	2.159402
H	-5.196131	-0.850198	2.689545
H	-6.567577	-1.356649	1.691245
H	-6.034897	0.453458	-0.011912
H	-4.453480	0.517407	-0.793452
H	-4.618834	0.997370	0.905144

ωB97XD energy = -1154.88130041 a.u.

(1*R*,5*S*,6*R*,7*R*,8*R*,10*R*)-**3**, Conf. J

C	4.076199	-1.898486	0.268177
C	4.487460	-0.472669	0.664822
C	3.293020	0.361498	1.057840
C	2.230613	0.372693	-0.016794
C	1.693541	-1.088119	-0.266111
C	2.922633	-1.917039	-0.736008

(1*R*,5*S*,6*R*,7*R*,8*R*,10*R*)-**3**, Conf. K

C	4.275195	-1.125746	0.337375
C	4.359504	0.382679	0.615656
C	3.006371	0.961423	0.947922
C	1.981172	0.655358	-0.118957
C	1.778528	-0.903404	-0.239921
C	3.162357	-1.480817	-0.650333

C	0.616623	1.295617	0.011308
C	-0.236544	1.003360	-1.220898
C	-0.561179	-0.468751	-1.333009
C	0.761208	-1.246805	-1.359359
O	0.615069	2.745244	0.076761
C	-0.559041	3.203804	-0.417874
C	-1.271480	2.079084	-1.105510
O	3.115832	-2.883438	-0.878907
C	2.756222	1.634321	2.072640
C	-2.548201	2.124776	-1.472670
O	-0.896304	4.358532	-0.302703
H	2.405756	0.992911	-1.077373
C	1.319878	-1.522405	1.093681
O	-1.360800	-0.827027	-0.189918
C	-2.191732	-1.874300	-0.290077
O	-2.357662	-2.494369	-1.322128
C	-2.870696	-2.178757	1.020832
C	-3.900884	-1.122892	1.485113
C	-3.262656	0.177681	1.977976
C	-4.948574	-0.850831	0.404169
H	4.108763	-1.660840	1.280845
H	5.225122	-1.486919	-0.069332
H	4.740753	0.886223	-0.283860
H	5.073377	0.577548	1.421040
H	3.412855	-1.051488	-1.628713
H	0.110396	0.950650	0.918731
H	0.381530	1.243649	-2.099181
H	-1.133101	-0.683870	-2.238590
H	1.220859	-1.037976	-2.333722
H	0.551029	-2.319287	-1.342090
H	3.088046	-3.335826	-0.027557
H	3.539871	1.799520	2.808278
H	1.780822	2.057370	2.293562
H	-3.027159	1.281359	-1.961515
H	-3.145002	3.013403	-1.287999
H	0.377041	-1.096746	1.440693
H	2.053500	-1.380655	1.890621
H	1.151089	-2.597383	0.967167
H	-3.369691	-3.142276	0.894274
H	-2.093886	-2.291416	1.785726
H	-4.409466	-1.586095	2.339599
H	-4.024201	0.824959	2.426193
H	-2.495397	-0.014583	2.736350
H	-2.794927	0.729437	1.157247
H	-4.498681	-0.360029	-0.467750
H	-5.732203	-0.187569	0.784726
H	-5.422603	-1.775981	0.059407

ωB97XD energy = -1154.88082913 a.u.

(1*R*,5*S*,6*R*,7*R*,8*R*,10*R*)-**3**, Conf. L

C	3.446109	-2.570195	-0.118092
C	4.205339	-1.298263	0.289542
C	3.270760	-0.245067	0.832238
C	2.144415	0.064672	-0.126588
C	1.272740	-1.224449	-0.377649
C	2.238544	-2.267276	-1.006068

C	1.208228	1.198332	0.228592
C	0.243836	1.482208	-0.920363
C	-0.700615	0.322383	-1.159727
C	0.139742	-0.938741	-1.396766
O	1.847202	2.483293	0.445955
C	0.967447	3.466544	0.140834
C	-0.205403	2.868504	-0.574379
O	1.571673	-3.464497	-1.385987
C	3.425035	0.317154	2.032398
C	-1.345506	3.516246	-0.795979
O	1.180158	4.624191	0.414716
H	2.610500	0.321478	-1.091029
C	0.675353	-1.768928	0.932280
O	-1.551632	0.146579	-0.010205
C	-2.870586	0.370850	-0.130535
O	-3.396257	0.748931	-1.159087
C	-3.603068	0.067559	1.149749
C	-4.204565	-1.359223	1.173129
C	-5.285887	-1.551578	0.108024
C	-3.125890	-2.439897	1.067363
H	3.112208	-3.101728	0.782219
H	4.113220	-3.247258	-0.660926
H	4.709158	-0.890442	-0.597845
H	4.982024	-1.539512	1.020729
H	2.599269	-1.843652	-1.952490
H	0.658709	0.973581	1.149243
H	0.854308	1.560370	-1.832818
H	-1.335020	0.509571	-2.027887
H	0.585264	-0.819831	-2.392571
H	-0.523131	-1.806139	-1.454748
H	1.414759	-3.997501	-0.597877
H	4.252368	0.032423	2.678054
H	2.751344	1.081778	2.407217
H	-2.174764	3.043520	-1.314013
H	-1.471915	4.539653	-0.453750
H	1.439192	-1.956470	1.690411
H	0.142320	-2.706755	0.741988
H	-0.054501	-1.080783	1.360669
H	-2.914213	0.185789	1.990375
H	-4.407331	0.801984	1.246297
H	-4.679916	-1.452618	2.157203
H	-5.766426	-2.528625	0.226305
H	-6.061173	-0.781531	0.180220
H	-4.864583	-1.504514	-0.901910
H	-2.629974	-2.415506	0.090080
H	-3.570087	-3.433511	1.188512
H	-2.356929	-2.316766	1.837196

ωB97XD energy = -1154.88054135 a.u.

(1*R*,5*S*,6*R*,7*R*,8*R*,10*R*)-**3**, Conf. M

C	4.191131	-1.582016	0.250493
C	4.480315	-0.102238	0.544573
C	3.227505	0.644001	0.932441
C	2.132919	0.488064	-0.097626
C	1.727253	-1.029273	-0.233344
C	3.010956	-1.769717	-0.704371

C	0.866952	1.294560	0.091724
C	-0.057869	1.139560	-1.112804
C	-0.576191	-0.275939	-1.235334
C	0.634194	-1.218360	-1.317197
O	1.054991	2.730892	0.180408
C	-0.065487	3.347111	-0.264736
C	-0.936840	2.340969	-0.951828
O	2.778229	-3.148645	-0.960976
C	3.112178	1.329348	2.071408
C	-2.197065	2.569713	-1.306149
O	-0.247012	4.532736	-0.116488
H	2.562909	0.785459	-1.067262
C	1.239288	-1.609642	1.107139
O	-1.388327	-0.543333	-0.075268
C	-2.302098	-1.523318	-0.148029
O	-2.524117	-2.149798	-1.165530
C	-3.002852	-1.730329	1.170257
C	-4.349644	-0.976609	1.283581
C	-4.174637	0.537511	1.153745
C	-5.393055	-1.499830	0.294947
H	3.988512	-2.108971	1.191492
H	5.073023	-2.053905	-0.194208
H	4.893468	0.361284	-0.362384
H	5.240339	-0.014988	1.326411
H	3.282936	-1.352076	-1.682102
H	0.351317	1.000546	1.011801
H	0.556114	1.311271	-2.009570
H	-1.196106	-0.398036	-2.126744
H	1.081783	-1.052720	-2.305465
H	0.287391	-2.254494	-1.307036
H	2.711366	-3.614222	-0.119043
H	3.937800	1.377164	2.777422
H	2.209508	1.871286	2.337056
H	-2.788226	1.809734	-1.808597
H	-2.667131	3.527060	-1.100291
H	0.348353	-1.097052	1.472446
H	1.998076	-1.543678	1.890328
H	0.965212	-2.662983	0.981563
H	-3.181237	-2.804171	1.275085
H	-2.337827	-1.405896	1.975210
H	-4.710188	-1.189843	2.297305
H	-5.118864	1.051161	1.362923
H	-3.419065	0.919978	1.848196
H	-3.865690	0.815299	0.139885
H	-5.101346	-1.298693	-0.741599
H	-6.359105	-1.013893	0.469479
H	-5.535168	-2.580829	0.397182

ωB97XD energy = -1154.88024381 a.u.

(1*R*,5*S*,6*R*,7*R*,8*R*,10*R*)-**3**, Conf. N

C	3.721549	-1.985263	0.180114
C	4.130272	-0.637586	0.793591
C	2.925166	0.189163	1.169685
C	1.977564	0.375192	0.007245
C	1.428855	-1.021298	-0.475643
C	2.676178	-1.832378	-0.925658

C	0.795044	1.298051	0.203480
C	0.037872	1.493090	-1.107155
C	-0.600848	0.211491	-1.590461
C	0.490868	-0.860732	-1.700076
O	1.123413	2.657173	0.594489
C	0.146029	3.486167	0.155911
C	-0.734226	2.737858	-0.798262
O	2.330080	-3.096986	-1.476054
C	2.723015	0.663791	2.400201
C	-1.930721	3.159663	-1.195395
O	0.076979	4.639984	0.508024
H	2.570278	0.784547	-0.826237
C	0.690099	-1.758215	0.656075
O	-1.628911	-0.146762	-0.645611
C	-2.697625	-0.819978	-1.091720
O	-2.833424	-1.153562	-2.252777
C	-3.716612	-1.084981	-0.011395
C	-3.168853	-1.549317	1.354354
C	-2.697135	-0.382143	2.225784
C	-4.246117	-2.353912	2.086628
H	3.327444	-2.642280	0.966048
H	4.599278	-2.484897	-0.241879
H	4.718415	-0.078052	0.052842
H	4.774497	-0.798839	1.662700
H	3.131190	-1.286813	-1.762338
H	0.126365	0.912743	0.980203
H	0.788112	1.758186	-1.866864
H	-1.070719	0.350601	-2.566535
H	1.094183	-0.587034	-2.575264
H	0.029755	-1.823452	-1.934152
H	2.101112	-3.697762	-0.757246
H	3.442002	0.473926	3.193505
H	1.855720	1.263801	2.659027
H	-2.541388	2.570572	-1.873870
H	-2.329929	4.105999	-0.841685
H	-0.185850	-1.204233	0.995990
H	1.327894	-1.927866	1.526594
H	0.326947	-2.728316	0.299216
H	-4.293665	-0.159182	0.119135
H	-4.400302	-1.830612	-0.424705
H	-2.316783	-2.216402	1.171047
H	-3.545745	0.257412	2.499719
H	-1.963205	0.241693	1.710613
H	-2.243108	-0.750753	3.151901
H	-5.145446	-1.746735	2.247033
H	-4.538413	-3.244109	1.519455
H	-3.885986	-2.682917	3.067369

ωB97XD energy = -1154.87965734 a.u.

(1*R*,5*S*,6*R*,7*R*,8*S*,10*R*)-**3**, Conf. A

C	4.304548	-1.798485	-0.441506
C	4.675792	-0.337951	-0.737121
C	3.748792	0.621058	-0.031650
C	2.297275	0.345603	-0.351006
C	1.899331	-1.102673	0.121064

C	2.818083	-2.074962	-0.670044
C	1.266764	1.311036	0.190223
C	-0.132156	0.983461	-0.333243
C	-0.576191	-0.352080	0.218896
C	0.428572	-1.412383	-0.247027
O	1.452297	2.693610	-0.202511
C	0.249079	3.314366	-0.186017
C	-0.829830	2.281604	-0.057932
O	2.505754	-3.439310	-0.422741
C	4.183121	1.571599	0.797992
C	-2.091057	2.576137	0.243995
O	0.144294	4.513670	-0.287273
H	2.203066	0.354147	-1.448210
C	2.094624	-1.287994	1.636223
O	-1.876877	-0.642288	-0.327602
C	-2.660615	-1.501577	0.346223
O	-2.320901	-2.036403	1.382568
C	-3.989583	-1.688611	-0.336921
C	-4.820418	-0.392479	-0.409362
C	-6.121137	-0.650269	-1.170734
C	-5.104717	0.167051	0.986100
H	4.564435	-2.039220	0.597675
H	4.886244	-2.469468	-1.081483
H	4.595451	-0.167590	-1.819913
H	5.715648	-0.148263	-0.456631
H	2.594842	-1.930964	-1.735016
H	1.272033	1.303153	1.286982
H	-0.052248	0.882374	-1.426874
H	-0.665932	-0.320439	1.308166
H	0.336094	-1.485751	-1.338548
H	0.154503	-2.385068	0.167811
H	2.882486	-3.697078	0.426778
H	5.246013	1.696587	0.990486
H	3.511977	2.263509	1.297679
H	-2.849283	1.804637	0.324667
H	-2.387082	3.606922	0.417750
H	1.402047	-0.678485	2.222779
H	3.104571	-1.026878	1.961054
H	1.900169	-2.329478	1.912817
H	-3.800409	-2.058297	-1.350943
H	-4.536192	-2.459484	0.213615
H	-4.235675	0.346310	-0.971414
H	-6.742069	-1.386502	-0.646153
H	-6.703519	0.272317	-1.265508
H	-5.926615	-1.030473	-2.179287
H	-4.185010	0.362069	1.549514
H	-5.706095	-0.537501	1.573126
H	-5.657654	1.109985	0.920632

ωB97XD energy = -1154.88260409 a.u.

(1*R*,5*S*,6*R*,7*R*,8*S*,10*R*)-**3**, Conf. B

C	3.302854	-2.823794	-0.453339
C	4.098302	-1.620097	-0.979280
C	3.663942	-0.336487	-0.315474
C	2.174062	-0.107749	-0.432926
C	1.385325	-1.278614	0.267263

C	1.794648	-2.576582	-0.482772
C	1.625733	1.198509	0.096419
C	0.136176	1.346437	-0.213428
C	-0.647217	0.297246	0.539920
C	-0.140241	-1.081173	0.102656
O	2.201291	2.395481	-0.485579
C	1.296281	3.398867	-0.390994
C	-0.030443	2.820483	0.000222
O	1.077924	-3.716332	-0.027617
C	4.511886	0.473383	0.321409
C	-1.047189	3.551020	0.449045
O	1.585835	4.548428	-0.623353
H	1.923472	-0.155532	-1.504172
C	1.725542	-1.383936	1.764733
O	-2.030234	0.436410	0.166360
C	-2.965596	0.003465	1.027990
O	-2.703312	-0.445896	2.125476
C	-4.349374	0.155463	0.453875
C	-4.549384	-0.614538	-0.865641
C	-4.276677	-2.109490	-0.684135
C	-5.962233	-0.371096	-1.396985
H	3.615146	-3.049176	0.574783
H	3.522013	-3.711063	-1.055757
H	3.924054	-1.528462	-2.060534
H	5.170171	-1.784177	-0.837167
H	1.480348	-2.455367	-1.527217
H	1.796645	1.275271	1.176997
H	0.007793	1.153066	-1.289964
H	-0.564553	0.442669	1.620069
H	-0.408615	-1.204675	-0.954653
H	-0.664098	-1.859818	0.663102
H	1.462887	-4.015766	0.804326
H	5.570829	0.233845	0.381034
H	4.194247	1.401652	0.786252
H	-1.991803	3.095969	0.729322
H	-0.947728	4.628227	0.548692
H	1.339809	-0.535577	2.336164
H	2.801987	-1.431139	1.945238
H	1.263300	-2.278677	2.194772
H	-5.058554	-0.193575	1.209634
H	-4.528153	1.223882	0.284840
H	-3.833045	-0.218984	-1.595544
H	-4.947923	-2.540608	0.068532
H	-4.434574	-2.646871	-1.625102
H	-3.246142	-2.303126	-0.365042
H	-6.150797	0.696147	-1.556874
H	-6.715492	-0.745705	-0.693126
H	-6.111643	-0.885055	-2.352228

ωB97XD energy = -1154.88237978 a.u.

(1*R*,5*S*,6*R*,7*R*,8*S*,10*R*)-**3**, Conf. C

C	3.113750	-3.031885	-0.337709
C	4.042596	-1.903855	-0.810540
C	3.676464	-0.584878	-0.175083
C	2.222889	-0.230850	-0.391652
C	1.292147	-1.327238	0.252105

C	1.638305	-2.656678	-0.473876
C	1.753596	1.118879	0.102598
C	0.306528	1.394103	-0.306734
C	-0.612089	0.418582	0.390079
C	-0.196825	-1.001386	-0.010564
O	2.468229	2.260268	-0.434759
C	1.649228	3.338663	-0.397100
C	0.253685	2.877568	-0.099794
O	0.794988	-3.729452	-0.077214
C	4.543810	0.151260	0.522414
C	-0.724774	3.693447	0.281915
O	2.052787	4.458547	-0.602794
H	2.040089	-0.261965	-1.476980
C	1.523087	-1.459208	1.768061
O	-1.953180	0.663285	-0.076606
C	-2.969560	0.342730	0.740800
O	-2.810630	-0.075286	1.870636
C	-4.310059	0.536982	0.084395
C	-5.027779	-0.810766	-0.135528
C	-6.414911	-0.568843	-0.730585
C	-4.202186	-1.744652	-1.023530
H	3.332271	-3.272426	0.710999
H	3.298229	-3.940028	-0.920362
H	3.945541	-1.803522	-1.900721
H	5.085698	-2.157313	-0.600687
H	1.411639	-2.509125	-1.537529
H	1.859125	1.183672	1.192276
H	0.234057	1.209475	-1.390057
H	-0.593348	0.562678	1.473273
H	-0.407783	-1.111356	-1.082127
H	-0.819797	-1.726966	0.518825
H	1.092173	-4.063246	0.777260
H	5.571621	-0.178315	0.654111
H	4.274073	1.102769	0.970479
H	-1.722317	3.321480	0.493187
H	-0.541305	4.758511	0.391902
H	1.157288	-0.587609	2.317086
H	2.579123	-1.580928	2.019942
H	0.973055	-2.321636	2.158858
H	-4.912298	1.168327	0.745965
H	-4.182530	1.054526	-0.870951
H	-5.148819	-1.283958	0.846625
H	-6.339945	-0.088501	-1.713812
H	-6.949216	-1.515772	-0.860786
H	-7.022278	0.074851	-0.084915
H	-3.232709	-1.986658	-0.574416
H	-4.015675	-1.288231	-2.003297
H	-4.730918	-2.689481	-1.186299

ωB97XD energy = -1154.88218026 a.u.

(1*R*,5*S*,6*R*,7*R*,8*S*,10*R*)-**3**, Conf. D

C	4.078903	-2.279954	-0.304245
C	4.681546	-0.896533	-0.590891
C	3.878338	0.203383	0.059156
C	2.417184	0.146247	-0.324416
C	1.785304	-1.221390	0.139124

C	2.581310	-2.329578	-0.604785
C	1.519467	1.260571	0.166329
C	0.114340	1.138449	-0.421893
C	-0.551760	-0.108268	0.110032
C	0.302179	-1.315442	-0.292100
O	1.921679	2.599211	-0.217977
C	0.821412	3.389047	-0.247890
C	-0.400387	2.523199	-0.172092
O	2.052951	-3.628485	-0.374973
C	4.412298	1.093544	0.897613
C	-1.617190	2.992512	0.089009
O	0.896364	4.590772	-0.345777
H	2.372397	0.154478	-1.424587
C	1.884362	-1.411158	1.662909
O	-1.845307	-0.207039	-0.516655
C	-2.813213	-0.871958	0.135311
O	-2.659687	-1.362680	1.235269
C	-4.076480	-0.950484	-0.682777
C	-5.358735	-0.881151	0.155609
C	-5.502943	0.486049	0.828232
C	-6.571772	-1.191867	-0.722911
H	4.248815	-2.542397	0.748065
H	4.581131	-3.040708	-0.910243
H	4.681615	-0.731880	-1.677493
H	5.723238	-0.861213	-0.259066
H	2.433623	-2.160928	-1.679069
H	1.473226	1.260525	1.262135
H	0.228745	1.019928	-1.510798
H	-0.698992	-0.047459	1.191685
H	0.247378	-1.399116	-1.385270
H	-0.134134	-2.226869	0.124350
H	2.336140	-3.934011	0.494628
H	5.471710	1.059911	1.140208
H	3.831423	1.888619	1.354972
H	-2.479524	2.335171	0.138756
H	-1.771802	4.054142	0.259976
H	2.898891	-1.257634	2.037919
H	1.563634	-2.421515	1.938198
H	1.232422	-0.724063	2.208584
H	-4.063833	-0.163076	-1.443205
H	-4.027208	-1.909361	-1.215546
H	-5.289797	-1.646629	0.937394
H	-4.670352	0.693278	1.509366
H	-5.538551	1.285077	0.077172
H	-6.426955	0.536710	1.413784
H	-6.485398	-2.179421	-1.189137
H	-6.676050	-0.449514	-1.524102
H	-7.494774	-1.177567	-0.133720

ωB97XD energy = -1154.88198936 a.u.

(1*R*,5*S*,6*R*,7*R*,8*S*,10*R*)-**3**, Conf. E

C	3.413477	-2.875311	-0.366533
C	4.282079	-1.669135	-0.752719
C	3.794134	-0.404950	-0.089690
C	2.333091	-0.140565	-0.372184
C	1.447049	-1.321090	0.180999

C	1.923138	-2.598195	-0.565304
C	1.746985	1.156511	0.139094
C	0.309195	1.344559	-0.342500
C	-0.575214	0.286498	0.273492
C	-0.045902	-1.086587	-0.152136
O	2.407273	2.362748	-0.321547
C	1.512668	3.379816	-0.297228
C	0.139249	2.812870	-0.096835
O	1.140142	-3.738933	-0.241397
C	4.571642	0.358149	0.680907
C	-0.914885	3.546585	0.248806
O	1.845552	4.531790	-0.443740
H	2.210444	-0.143650	-1.466440
C	1.604508	-1.493678	1.702056
O	-1.904675	0.468814	-0.251400
C	-2.941923	0.105058	0.520086
O	-2.812391	-0.376300	1.627561
C	-4.262255	0.404143	-0.141885
C	-5.303849	-0.707884	0.046237
C	-6.659044	-0.243035	-0.488424
C	-4.856595	-2.001792	-0.637675
H	3.598834	-3.138847	0.682912
H	3.689153	-3.745935	-0.970149
H	4.229957	-1.533772	-1.842104
H	5.329039	-1.859172	-0.500283
H	1.737687	-2.432548	-1.634249
H	1.788164	1.189707	1.234521
H	0.310282	1.190372	-1.432857
H	-0.619499	0.391364	1.360456
H	-0.193619	-1.170625	-1.236629
H	-0.644723	-1.871521	0.316987
H	1.422634	-4.081102	0.614761
H	5.612604	0.096057	0.854396
H	4.213195	1.270229	1.148401
H	-1.894266	3.100948	0.390965
H	-0.812329	4.617628	0.398971
H	1.169579	-0.659605	2.258877
H	2.650552	-1.575784	2.006682
H	1.075698	-2.393485	2.033755
H	-4.626697	1.335193	0.311712
H	-4.097710	0.603599	-1.205344
H	-5.400711	-0.898137	1.121579
H	-6.603034	-0.027082	-1.562538
H	-7.419997	-1.017268	-0.344144
H	-7.001153	0.664481	0.020910
H	-4.715270	-1.843536	-1.714020
H	-5.607860	-2.788264	-0.510051
H	-3.913450	-2.375989	-0.224272

ωB97XD energy = -1154.88195470 a.u.

(1*R*,5*S*,6*R*,7*R*,8*S*,10*R*)-**3**, Conf. F

C	2.896109	-3.121479	-0.323130
C	3.876344	-2.032263	-0.782531
C	3.564812	-0.703873	-0.137618
C	2.130634	-0.283825	-0.363635
C	1.146020	-1.341416	0.264172

C	1.439305	-2.681711	-0.465104
C	1.717801	1.082380	0.135807
C	0.287875	1.424670	-0.282703
C	-0.680654	0.485782	0.396869
C	-0.323553	-0.948015	-0.011894
O	2.487317	2.193628	-0.388654
C	1.717105	3.307234	-0.351639
C	0.300085	2.907780	-0.066303
O	0.548948	-3.718734	-0.076487
C	4.458700	-0.015581	0.574939
C	-0.642929	3.764547	0.314896
O	2.171920	4.408938	-0.548571
H	1.956437	-0.299899	-1.450685
C	1.356454	-1.492645	1.781000
O	-2.002259	0.799776	-0.079549
C	-3.044284	0.479045	0.707141
O	-2.915081	0.008926	1.820235
C	-4.361334	0.754309	0.032863
C	-4.933668	-0.485071	-0.699446
C	-4.022655	-0.961684	-1.832801
C	-5.260628	-1.629904	0.260947
H	3.097621	-3.378080	0.724938
H	3.044065	-4.033253	-0.910453
H	3.790492	-1.919485	-1.872393
H	4.905763	-2.333348	-0.568312
H	1.224718	-2.520847	-1.529341
H	1.816976	1.134773	1.226763
H	0.216537	1.250877	-1.367827
H	-0.665507	0.619623	1.481667
H	-0.525073	-1.038036	-1.087176
H	-0.984983	-1.649959	0.502345
H	0.826439	-4.066969	0.778881
H	5.468553	-0.395112	0.711246
H	4.230547	0.942410	1.032238
H	-1.657642	3.437338	0.518328
H	-0.412755	4.819691	0.433037
H	2.401788	-1.676769	2.039677
H	0.752931	-2.322588	2.163064
H	1.039086	-0.603472	2.332042
H	-5.064665	1.072767	0.807080
H	-4.233215	1.568700	-0.685364
H	-5.874260	-0.138957	-1.145236
H	-3.768733	-0.146635	-2.518622
H	-3.086546	-1.378070	-1.443701
H	-4.517572	-1.749459	-2.410526
H	-5.763816	-2.442121	-0.274635
H	-5.919676	-1.299624	1.070756
H	-4.353515	-2.043449	0.715428

ωB97XD energy = -1154.88113553 a.u.

(1*R*,5*S*,6*R*,7*R*,8*S*,10*R*)-**3**, Conf. G

C	4.354646	-1.724909	-0.570292
C	4.652013	-0.255906	-0.905033
C	3.729715	0.679136	-0.162413
C	2.272496	0.344250	-0.387198
C	1.962759	-1.113249	0.124944

C	2.870081	-2.059039	-0.709588
C	1.245871	1.274664	0.217992
C	-0.176467	0.893024	-0.193642
C	-0.531380	-0.466168	0.365215
C	0.488334	-1.483319	-0.153917
O	1.343545	2.660582	-0.198638
C	0.122376	3.233378	-0.074554
C	-0.894199	2.158264	0.169416
O	2.624339	-3.431479	-0.432482
C	4.173118	1.664300	0.620671
C	-2.106967	2.399701	0.658789
O	-0.041308	4.426030	-0.174669
H	2.105817	0.336286	-1.475592
C	2.252633	-1.267648	1.628086
O	-1.824091	-0.849385	-0.144489
C	-2.750336	-1.312576	0.710574
O	-2.572499	-1.397136	1.910089
C	-4.032548	-1.683637	0.014136
C	-4.949982	-0.467051	-0.256354
C	-4.455639	0.385837	-1.426525
C	-6.376894	-0.957251	-0.512230
H	4.681382	-1.940461	0.455193
H	4.924789	-2.383107	-1.233502
H	4.505299	-0.104944	-1.983746
H	5.697851	-0.023122	-0.685094
H	2.580762	-1.934363	-1.760883
H	1.335835	1.279018	1.310968
H	-0.182895	0.813785	-1.292160
H	-0.581162	-0.445461	1.455933
H	0.336298	-1.571678	-1.237322
H	0.274614	-2.463202	0.281943
H	3.067410	-3.668026	0.390795
H	5.239415	1.833708	0.749715
H	3.504382	2.341053	1.143690
H	-2.815957	1.602906	0.858895
H	-2.413468	3.418008	0.881071
H	1.567000	-0.680376	2.245018
H	3.266286	-0.956450	1.891195
H	2.121877	-2.312186	1.929947
H	-3.806446	-2.187375	-0.931764
H	-4.547533	-2.388612	0.670984
H	-4.963750	0.151374	0.651782
H	-5.091095	1.268926	-1.552059
H	-3.427528	0.728825	-1.285639
H	-4.489705	-0.188827	-2.360340
H	-7.044550	-0.115931	-0.726163
H	-6.776973	-1.494062	0.354214
H	-6.405623	-1.635400	-1.374045

ωB97XD energy = -1154.88035008 a.u.

(1*R*,5*S*,6*R*,7*R*,8*S*,10*R*)-**3**, Conf. H

C	4.275912	-1.725596	-0.469241
C	4.603274	-0.260837	-0.793747
C	3.671647	0.685336	-0.077149
C	2.218097	0.371753	-0.351451
C	1.870087	-1.081993	0.143539

C	2.792611	-2.039137	-0.661100
C	1.183437	1.313432	0.221476
C	-0.229428	0.955522	-0.243326
C	-0.628420	-0.400653	0.291008
C	0.401888	-1.429425	-0.189847
O	1.317521	2.699012	-0.186290
C	0.101988	3.290578	-0.104289
C	-0.938176	2.230786	0.101787
O	2.517835	-3.408368	-0.396194
C	4.105610	1.659582	0.724785
C	-2.160043	2.487873	0.558759
O	-0.039571	4.485808	-0.207398
H	2.088623	0.370497	-1.445025
C	2.103035	-1.246597	1.655447
O	-1.904992	-0.744541	-0.284558
C	-2.716403	-1.562057	0.408126
O	-2.475099	-1.932927	1.539862
C	-3.935507	-1.960928	-0.386442
C	-4.983956	-0.841363	-0.585283
C	-5.396790	-0.220275	0.750189
C	-4.545919	0.220603	-1.596071
H	4.563671	-1.944501	0.567225
H	4.859884	-2.391332	-1.112632
H	4.490411	-0.107550	-1.876227
H	5.645144	-0.042024	-0.543182
H	2.542204	-1.908545	-1.721760
H	1.233721	1.312442	1.317031
H	-0.195163	0.883378	-1.341759
H	-0.729003	-0.386918	1.378906
H	0.288839	-1.508216	-1.278873
H	0.162213	-2.409261	0.231395
H	2.925787	-3.652069	0.443018
H	5.170008	1.812001	0.886564
H	3.432213	2.344342	1.231102
H	-2.883300	1.697854	0.730114
H	-2.458762	3.508863	0.779280
H	1.396903	-0.660212	2.249647
H	3.107890	-0.942385	1.958131
H	1.955500	-2.292334	1.944932
H	-3.601544	-2.326420	-1.363391
H	-4.393722	-2.793128	0.153003
H	-5.861983	-1.347988	-1.004192
H	-4.558906	0.306468	1.222901
H	-6.203910	0.505880	0.608700
H	-5.745166	-0.982223	1.455591
H	-5.355372	0.937970	-1.769400
H	-4.288053	-0.234053	-2.558709
H	-3.671354	0.777970	-1.248778

ωB97XD energy = -1154.88030367 a.u.

(1*R*,5*S*,6*R*,7*R*,8*S*,10*R*)-**3**, Conf. I

C	4.393243	-1.703204	-0.544666
C	4.660356	-0.238515	-0.919397
C	3.725808	0.701469	-0.198077
C	2.272352	0.331372	-0.394924
C	2.005410	-1.118163	0.157505

C	2.918342	-2.061548	-0.674725
C	1.235699	1.253307	0.205301
C	-0.186925	0.837370	-0.178245
C	-0.503248	-0.515224	0.424818
C	0.532833	-1.522964	-0.081363
O	1.307942	2.628568	-0.247033
C	0.080000	3.186251	-0.135640
C	-0.921283	2.106250	0.145655
O	2.785911	-3.425939	-0.296169
C	4.158733	1.716445	0.552182
C	-2.138236	2.364105	0.616107
O	-0.101767	4.372884	-0.271227
H	2.089587	0.294833	-1.480215
C	2.330733	-1.238805	1.655738
O	-1.783002	-0.981025	-0.047814
C	-2.795908	-1.113744	0.824469
O	-2.713856	-0.809848	1.998355
C	-4.043226	-1.646366	0.172381
C	-4.982191	-0.520036	-0.316850
C	-4.406856	0.220650	-1.525664
C	-6.354450	-1.110926	-0.644311
H	4.718330	-1.894737	0.483969
H	4.972950	-2.367040	-1.193416
H	4.507243	-0.117450	-2.001224
H	5.702553	0.019219	-0.710382
H	2.624078	-1.949314	-1.730575
H	1.341123	1.283277	1.296790
H	-0.204663	0.722234	-1.273594
H	-0.534626	-0.461993	1.515057
H	0.360890	-1.652001	-1.158651
H	0.336819	-2.485957	0.402178
H	1.947096	-3.767277	-0.623671
H	5.223393	1.905706	0.666586
H	3.484496	2.395505	1.064771
H	-2.849408	1.580480	0.852879
H	-2.443647	3.391851	0.792051
H	3.295987	-0.792532	1.905626
H	2.359108	-2.295314	1.937748
H	1.577696	-0.751641	2.281870
H	-3.776830	-2.292815	-0.669724
H	-4.559097	-2.245760	0.927133
H	-5.107416	0.194828	0.507295
H	-3.435561	0.676302	-1.312185
H	-4.273733	-0.467202	-2.369692
H	-5.085735	1.018129	-1.845351
H	-7.040805	-0.331267	-0.990823
H	-6.275863	-1.864295	-1.437957
H	-6.802903	-1.589244	0.232838

ωB97XD energy = -1154.88013975 a.u.

(1*R*,5*S*,6*R*,7*R*,8*S*,10*R*)-**3**, Conf. J

C	4.089322	-2.152325	-0.256933
C	4.662419	-0.736961	-0.424182
C	3.777530	0.302495	0.219316
C	2.348934	0.216591	-0.267521
C	1.735834	-1.189637	0.087240

C	2.615032	-2.235261	-0.652846
C	1.383214	1.277349	0.211901
C	0.016753	1.141607	-0.462822
C	-0.630488	-0.151279	-0.020167
C	0.288317	-1.303565	-0.445264
O	1.768448	2.640898	-0.090808
C	0.650959	3.401953	-0.160690
C	-0.548602	2.503283	-0.190254
O	2.122123	-3.560582	-0.509994
C	4.221768	1.168938	1.131918
C	-1.790714	2.935001	0.006936
O	0.698053	4.608175	-0.211308
H	2.378792	0.277644	-1.366708
C	1.743224	-1.455361	1.602785
O	-1.901308	-0.271154	-0.690187
C	-2.797257	-1.124117	-0.159498
O	-2.586986	-1.747907	0.861636
C	-4.063133	-1.201337	-0.974810
C	-5.305758	-0.636566	-0.247854
C	-5.733515	-1.484877	0.951260
C	-5.101223	0.824796	0.155633
H	4.204517	-2.474079	0.786155
H	4.654697	-2.858632	-0.872969
H	4.738323	-0.513692	-1.497601
H	5.674264	-0.688422	-0.011655
H	2.525137	-2.025845	-1.726297
H	1.270628	1.227156	1.301523
H	0.196234	1.074296	-1.546963
H	-0.814806	-0.150387	1.057744
H	0.307224	-1.314306	-1.542966
H	-0.138885	-2.253675	-0.117536
H	2.346275	-3.885424	0.369992
H	5.262625	1.158720	1.446405
H	3.581730	1.922117	1.581412
H	-2.635327	2.255231	-0.021434
H	-1.981819	3.986493	0.202191
H	2.735977	-1.335788	2.042739
H	1.394669	-2.473382	1.805990
H	1.070881	-0.787169	2.146633
H	-3.912805	-0.667681	-1.916304
H	-4.227842	-2.259746	-1.203304
H	-6.112060	-0.673770	-0.990784
H	-5.895081	-2.529846	0.665391
H	-4.976199	-1.468712	1.740768
H	-6.669605	-1.100791	1.371226
H	-4.326812	0.913437	0.928028
H	-6.023289	1.245724	0.569653
H	-4.805769	1.440719	-0.700936

ωB97XD energy = -1154.88005096 a.u.

3-deoxy-1,2;5,6-di-*O*-isopropylidene-3-*C*-methylene-*α*-D-*ribo*-hexofuranose (**4**), Conf. A

C	-1.191829	-0.755905	-0.862184
O	-0.709663	0.353744	-1.609734
C	0.165646	1.143387	-0.810906

C	-0.340227	0.953241	0.598172
C	-1.008980	-0.403139	0.632449
O	-2.315784	-0.416657	1.164534
C	-3.239972	-0.334316	0.082868
O	-2.560102	-0.936197	-1.018523
C	-4.448296	-1.182158	0.424524
C	-3.609266	1.114144	-0.228593
C	-0.308187	1.831351	1.594199
C	1.613303	0.693846	-1.024682
C	2.678702	1.525654	-0.310513
O	3.689812	0.575688	-0.043723
C	3.046045	-0.659535	0.221788
O	1.803804	-0.608211	-0.488256
C	2.756051	-0.820065	1.710285
C	3.908263	-1.774053	-0.339347
H	0.069006	2.182101	-1.147539
H	1.795768	0.665488	-2.106772
H	-0.686174	-1.661239	-1.201402
H	-0.406629	-1.135043	1.174838
H	-4.131418	-2.207271	0.625041
H	-4.943947	-0.778006	1.310602
H	-5.155635	-1.179678	-0.408286
H	-4.308726	1.140427	-1.068024
H	-4.080703	1.569304	0.646456
H	-2.726905	1.696193	-0.497425
H	-0.755242	1.598901	2.555873
H	0.136592	2.815452	1.474458
H	2.293457	1.965607	0.619517
H	3.102413	2.313161	-0.935783
H	2.223385	-1.758724	1.886509
H	2.132568	0.003836	2.067402
H	3.692008	-0.832987	2.274815
H	4.877830	-1.788134	0.164995
H	3.417022	-2.738687	-0.190040
H	4.063284	-1.612348	-1.407987

ωB97XD energy = -883.305951357 a.u.

3-deoxy-1,2;5,6-di-*O*-isopropylidene-3-*C*-methylene-*α*-D-*ribo*-hexofuranose (**4**), Conf. B

C	1.955967	0.370095	-1.302827
O	0.673528	-0.227599	-1.380356
C	-0.198813	0.235074	-0.343120
C	0.456456	1.495451	0.170619
C	1.922277	1.316808	-0.097068
O	2.538279	0.554701	0.931942
C	3.190215	-0.592574	0.387251
O	2.974050	-0.532658	-1.020838
C	4.685315	-0.502925	0.642320
C	2.565350	-1.850367	0.973293
C	-0.113124	2.578035	0.690213
C	-1.591163	0.397377	-0.952831
C	-2.171366	-0.929427	-1.437682
O	-2.781202	-1.437130	-0.263804
C	-3.301442	-0.330115	0.450970
O	-2.526188	0.802698	0.033990
C	-3.096621	-0.577198	1.934893
C	-4.759428	-0.074771	0.087991
H	-0.251347	-0.521493	0.454072
H	-1.554354	1.158048	-1.738657
H	2.176999	0.824098	-2.270230
H	2.459338	2.257922	-0.252089
H	5.196804	-1.350971	0.179727
H	5.077438	0.423861	0.217301
H	4.880100	-0.513710	1.717632
H	3.056562	-2.736448	0.563369
H	2.678905	-1.849619	2.060470
H	1.503277	-1.896234	0.724376
H	0.493735	3.428962	0.986438
H	-1.185159	2.635344	0.843124
H	-1.416432	-1.640099	-1.774426
H	-2.911314	-0.770610	-2.233756
H	-2.033893	-0.720699	2.142033
H	-3.643513	-1.471161	2.245544
H	-3.459153	0.278595	2.509818
H	-4.864673	0.056230	-0.992021
H	-5.378100	-0.918578	0.404560
H	-5.114712	0.833676	0.581109

ωB97XD energy = -883.305403735 a.u.

3-deoxy-1,2;5,6-di-*O*-isopropylidene-3-*C*-methylene-*α*-D-*ribo*-hexofuranose (**4**), Conf. C

C	-1.890925	-1.415763	0.202576
O	-0.540476	-1.620572	-0.085293
C	0.211861	-0.433173	0.176805
C	-0.580697	0.297797	1.238165
C	-1.967565	-0.324758	1.283853
O	-3.029254	0.510771	0.880796
C	-3.198569	0.382320	-0.527173
O	-2.616280	-0.874733	-0.874795
C	-4.687060	0.335358	-0.821955
C	-2.497048	1.512165	-1.273257
C	-0.178169	1.305907	2.005585
C	1.621881	-0.873174	0.576644
C	2.343939	-1.601148	-0.556077
O	2.935145	-0.532184	-1.272770
C	3.318649	0.444434	-0.322345
O	2.450395	0.255004	0.803600
C	3.086303	1.817866	-0.926032
C	4.757259	0.234702	0.136263
H	0.283494	0.162675	-0.745048
H	1.567764	-1.463570	1.496347
H	-2.307136	-2.391952	0.457848
H	-2.207850	-0.707303	2.278177
H	-4.852015	0.207461	-1.894648
H	-5.139840	-0.503022	-0.288748
H	-5.161499	1.264939	-0.497487
H	-2.962488	2.467699	-1.018440
H	-1.440353	1.563459	-1.005612
H	-2.581415	1.350121	-2.350893
H	-0.854548	1.743911	2.734268
H	0.820408	1.719579	1.918210
H	1.673650	-2.141694	-1.224548

H	3.105466	-2.288424	-0.163528
H	3.696321	1.941223	-1.824382
H	3.355325	2.593965	-0.205063
H	2.033050	1.929567	-1.193568
H	5.005751	0.948239	0.926038
H	4.886920	-0.775512	0.532963
H	5.443595	0.377171	-0.702502

ωB97XD energy = -883.305394979 a.u.

3-deoxy-1,2;5,6-di-*O*-isopropylidene-3-*C*-methylene-*α*-D-*ribo*-hexofuranose (**4**), Conf. D

C	-1.218280	-0.545626	-1.035245
O	-0.709104	0.690983	-1.512544
C	0.183493	1.267578	-0.565884
C	-0.275134	0.736294	0.770759
C	-0.998186	-0.563890	0.494290
O	-2.297297	-0.660982	1.040111
C	-3.233549	-0.292966	0.031927
O	-2.595244	-0.640675	-1.196724
C	-4.470090	-1.154393	0.187729
C	-3.549219	1.200280	0.081032
C	-0.146633	1.314902	1.958519
C	1.626942	0.884912	-0.916399
C	2.705244	1.557146	-0.044690
O	3.162821	0.526216	0.802953
C	2.971111	-0.698728	0.121278
O	1.810359	-0.504242	-0.683155
C	2.686172	-1.776588	1.147377
C	4.166414	-1.024277	-0.771643
H	0.079798	2.356359	-0.640960
H	1.776045	1.099966	-1.981193
H	-0.752471	-1.361639	-1.590289
H	-0.420210	-1.427673	0.829592
H	-5.188915	-0.921976	-0.601720
H	-4.192786	-2.208171	0.123111
H	-4.936396	-0.963227	1.157421
H	-4.251164	1.453187	-0.717650
H	-3.997931	1.449005	1.046342
H	-2.645991	1.796775	-0.052998
H	-0.547377	0.845651	2.851640
H	0.344566	2.275872	2.079141
H	2.314272	2.361825	0.583312
H	3.521052	1.957611	-0.660289
H	1.851619	-1.462341	1.777951
H	3.565183	-1.937482	1.776205
H	2.434991	-2.714347	0.645569
H	4.325048	-0.232588	-1.509673
H	5.071274	-1.125546	-0.166939
H	3.987388	-1.959530	-1.308001

ωB97XD energy = -883.305310724 a.u.

3-deoxy-1,2;5,6-di-*O*-isopropylidene-3-*C*-methylene-*α*-D-*ribo*-hexofuranose (**4**), Conf. E

C	1.923510	-1.072153	-0.894897
O	0.576317	-1.366057	-0.668159
C	-0.114740	-0.203483	-0.208423
C	0.665300	0.959514	-0.780945
C	2.012821	0.425365	-1.237831
O	3.134412	0.893638	-0.523240
C	3.360246	0.031173	0.586585
O	2.724107	-1.204213	0.253583
C	4.855473	-0.200259	0.709314
C	2.757204	0.605158	1.863999
C	0.266787	2.223480	-0.878879
C	-1.559670	-0.331916	-0.665328
C	-2.244201	-1.609759	-0.184119
O	-3.593479	-1.203461	-0.064066
C	-3.592332	0.147252	0.363067
O	-2.336719	0.691577	-0.064760
C	-3.668207	0.244348	1.882442
C	-4.728783	0.869035	-0.337474
H	-0.106560	-0.178214	0.892032
H	-1.608049	-0.246897	-1.759381
H	2.269963	-1.773778	-1.655695
H	2.190181	0.623747	-2.296937
H	5.061873	-0.885973	1.534873
H	5.237444	-0.632397	-0.217821
H	5.364674	0.748050	0.898851
H	2.873805	-0.111574	2.680992
H	3.266342	1.535644	2.127402
H	1.695739	0.821174	1.731527
H	0.920702	2.976266	-1.309731
H	-0.708527	2.536382	-0.520480
H	-1.834439	-1.941647	0.780828
H	-2.188660	-2.430500	-0.898365
H	-2.852354	-0.322414	2.338962
H	-4.620510	-0.157221	2.238485
H	-3.579730	1.288048	2.195108
H	-5.685793	0.420071	-0.059623
H	-4.735282	1.924599	-0.054236
H	-4.598375	0.788229	-1.418714

ωB97XD energy = -883.304695831 a.u.

3-deoxy-1,2;5,6-di-*O*-isopropylidene-3-*C*-methylene-*α*-D-*ribo*-hexofuranose (**4**), Conf. F

C	1.970440	0.221776	-1.306214
O	0.695210	-0.394171	-1.229231
C	-0.113115	0.174404	-0.194128
C	0.553368	1.489811	0.130189
C	2.000666	1.297954	-0.213538
O	2.696170	0.661357	0.849280
C	3.330120	-0.533234	0.392805
O	3.020535	-0.633349	-0.995402
C	4.836907	-0.401524	0.535671
C	2.764862	-1.722853	1.155382
C	-0.014725	2.612156	0.557300
C	-1.538637	0.253804	-0.719160
C	-2.090633	-1.094689	-1.188600
O	-3.463068	-0.998438	-0.861685
C	-3.550741	-0.255279	0.338866
O	-2.418230	0.623300	0.331501
C	-3.463535	-1.165197	1.560974

C	-4.825216	0.566055	0.305935
H	-0.106963	-0.485470	0.687785
H	-1.589474	1.001615	-1.521472
H	2.118818	0.565734	-2.331069
H	2.511190	2.220008	-0.508769
H	5.331879	-1.287893	0.130787
H	5.182806	0.479760	-0.009292
H	5.101882	-0.295309	1.590535
H	3.255356	-2.643234	0.828542
H	2.931483	-1.589168	2.227238
H	1.693180	-1.815260	0.968145
H	0.579469	3.506198	0.722111
H	-1.078986	2.661503	0.764200
H	-1.608165	-1.929833	-0.660812
H	-1.995893	-1.252936	-2.262166
H	-4.316543	-1.848513	1.582422
H	-3.461659	-0.564636	2.474251
H	-2.541822	-1.752785	1.536949
H	-4.822667	1.208167	-0.577285
H	-5.695545	-0.094058	0.266824
H	-4.891839	1.187410	1.202490

ωB97XD energy = -883.304602007 a.u.

3-deoxy-1,2;5,6-di-*O*-isopropylidene-3-*C*-methylene-*α*-D-*ribo*-hexofuranose (**4**), Conf. G

C	1.225170	-0.781660	-0.783002
O	0.102270	-0.773823	0.060947
C	-0.166509	0.503810	0.630146
C	0.816638	1.461557	-0.005210
C	1.704222	0.675866	-0.948371
O	3.071279	0.650413	-0.600293
C	3.318500	-0.521123	0.171920
O	2.330515	-1.455724	-0.254988
C	4.685678	-1.057599	-0.203118
C	3.193274	-0.241981	1.666932
C	0.914129	2.768561	0.217676
C	-1.620961	0.907930	0.377493
C	-2.058357	0.851061	-1.093878
O	-3.383707	0.371010	-1.023032
C	-3.455634	-0.491016	0.101678
O	-2.515230	0.036635	1.039571
C	-3.066543	-1.918265	-0.268902
C	-4.848801	-0.388336	0.691008
H	-0.024112	0.438539	1.716318
H	-1.755124	1.918886	0.786057
H	0.935397	-1.299991	-1.699929
H	1.629621	1.044450	-1.974583
H	4.725440	-1.238431	-1.278826
H	5.456864	-0.332813	0.068941
H	4.876916	-1.994256	0.326061
H	2.207401	0.152931	1.916598
H	3.345089	-1.168633	2.225769
H	3.945379	0.490133	1.971335
H	1.662920	3.365456	-0.293990
H	0.273964	3.280149	0.930905
H	-1.419242	0.169884	-1.672122
H	-2.071631	1.829911	-1.574871
H	-2.050844	-1.944405	-0.669473
H	-3.765833	-2.314420	-1.010331
H	-3.091682	-2.550631	0.622362
H	-5.587142	-0.756119	-0.026077
H	-4.911795	-0.986268	1.603431
H	-5.067439	0.654720	0.928544

ωB97XD energy = -883.303517017 a.u.

3-deoxy-1,2;5,6-di-*O*-isopropylidene-3-*C*-methylene-*α*-D-*ribo*-hexofuranose (**4**), Conf. H

C	1.359297	-0.215722	-1.096204
O	0.202381	-0.507262	-0.334066
C	-0.122185	0.535924	0.586486
C	0.753994	1.702633	0.183608
C	1.915622	1.106018	-0.555137
O	2.930446	0.685622	0.344073
C	3.211866	-0.706007	0.168387
O	2.385313	-1.132643	-0.911847
C	4.661954	-0.884899	-0.244395
C	2.859748	-1.457918	1.444082
C	0.551017	3.001015	0.384268
C	-1.617235	0.834421	0.551483
C	-2.189003	1.106342	-0.848342
O	-3.449241	0.471117	-0.817384
C	-3.330095	-0.651201	0.041461
O	-2.365123	-0.270107	1.024321
C	-2.837541	-1.879913	-0.716381
C	-4.666485	-0.872595	0.722478
H	0.129546	0.199822	1.601861
H	-1.803498	1.681900	1.224694
H	1.093595	-0.231689	-2.155823
H	2.332263	1.765050	-1.323487
H	4.868893	-1.940996	-0.435212
H	4.859980	-0.313546	-1.154052
H	5.322459	-0.531914	0.551369
H	1.795671	-1.350521	1.661961
H	3.081856	-2.520940	1.321272
H	3.441937	-1.064310	2.281125
H	1.264816	3.734858	0.022547
H	-0.312317	3.378753	0.923434
H	-1.542824	0.679468	-1.626293
H	-2.344327	2.167220	-1.048897
H	-1.863633	-1.684975	-1.170048
H	-3.558366	-2.153188	-1.491737
H	-2.722991	-2.717042	-0.022776
H	-5.425035	-1.136779	-0.018597
H	-4.583915	-1.684436	1.449243
H	-4.970024	0.040856	1.237870

ωB97XD energy = -883.303403171 a.u.

3-deoxy-1,2;5,6-di-*O*-isopropylidene-3-*C*-methylene-*α*-D-*ribo*-hexofuranose (**4**), Conf. I

C	0.926439	-0.674188	-0.597053
O	0.080223	-0.549231	0.529179
C	-0.253949	0.804899	0.798479

C	0.797230	1.633244	0.097561
C	1.516408	0.727764	-0.878945
O	2.903157	0.583340	-0.653014
C	3.127489	-0.642297	0.040956
O	2.032462	-1.473959	-0.336554
C	4.404695	-1.263815	-0.486109
C	3.160175	-0.421693	1.550923
C	1.059508	2.920477	0.299642
C	-1.679683	1.153489	0.333519
C	-1.975786	0.901565	-1.148517
O	-2.466820	-0.419860	-1.159167
C	-3.165885	-0.622002	0.061280
O	-2.632984	0.338460	0.982355
C	-2.878740	-2.028073	0.550811
C	-4.653137	-0.340208	-0.119485
H	-0.224995	0.936129	1.885853
H	-1.865740	2.199613	0.604959
H	0.364812	-1.135755	-1.413325
H	1.381450	1.054030	-1.914363
H	4.574926	-2.230393	-0.005709
H	4.322511	-1.407445	-1.564977
H	5.252015	-0.607937	-0.271877
H	2.229141	0.023082	1.905183
H	3.296723	-1.379679	2.058831
H	3.988961	0.243361	1.807117
H	1.841121	3.427425	-0.257830
H	0.521996	3.500776	1.044751
H	-1.103104	0.952687	-1.803650
H	-2.727703	1.612239	-1.519142
H	-3.236506	-2.757406	-0.180956
H	-3.389757	-2.201391	1.501458
H	-1.802945	-2.144008	0.690593
H	-4.804700	0.666762	-0.518095
H	-5.089168	-1.064471	-0.812558
H	-5.165921	-0.412582	0.842981

ωB97XD energy = -883.303265994 a.u.

Methyl 5-deoxy-2,3-*O*-isopropylidene-*β*-D-*erythro*-pent-4-enofuranoside (**5**), Conf. A

C	-1.339882	-0.732260	0.333820
O	-1.286810	0.616426	0.782478
C	-0.629123	1.387533	-0.144541
C	0.209325	0.498829	-1.049048
C	-0.118826	-0.923911	-0.565480
C	-0.718289	2.712405	-0.166322
O	1.604449	0.615407	-0.871508
C	2.014999	-0.294561	0.140223
O	1.022938	-1.325894	0.154209
C	3.343604	-0.895908	-0.279120
C	2.078985	0.379780	1.506416
O	-2.464026	-0.966116	-0.449025
C	-3.693237	-0.791520	0.234291
H	-1.330448	-1.358524	1.233537
H	-0.006819	0.680290	-2.102320
H	-0.335808	-1.640790	-1.359767
H	-0.135872	3.274910	-0.885644
H	-1.340042	3.248804	0.540628
H	3.236722	-1.374397	-1.254649
H	4.103902	-0.113561	-0.343258
H	3.663563	-1.640637	0.453637
H	1.108307	0.796494	1.781598
H	2.372524	-0.353061	2.262657
H	2.816681	1.185958	1.486722
H	-3.834463	0.251914	0.532835
H	-3.736811	-1.433122	1.125072
H	-4.479184	-1.085175	-0.461707

ωB97XD energy = -652.084348640 a.u.

Methyl 5-deoxy-2,3-*O*-isopropylidene-*β*-D-*erythro*-pent-4-enofuranoside (**5**), Conf. B

C	-1.316799	-0.759426	0.343747
O	-1.460440	0.560002	0.849346
C	-0.873386	1.442419	-0.023916
C	0.240800	0.736430	-0.758109
C	-0.026994	-0.759114	-0.478308
C	-1.247806	2.711573	-0.132444
O	1.491279	0.990578	-0.137062
C	2.127728	-0.255530	0.105753
O	1.063001	-1.174902	0.311960
C	2.965384	-0.670623	-1.102260
C	2.931658	-0.150752	1.383935
O	-2.356777	-1.092410	-0.517456
C	-3.631185	-1.110000	0.101825
H	-1.278438	-1.425572	1.213279
H	0.273793	0.995467	-1.820274
H	-0.140519	-1.373051	-1.376009
H	-0.711298	3.373028	-0.801442
H	-2.060893	3.111490	0.461501
H	2.344391	-0.736032	-2.000809
H	3.751765	0.067035	-1.280444
H	3.422701	-1.647521	-0.926034
H	2.270601	0.142189	2.201486
H	3.393266	-1.113598	1.615576
H	3.716557	0.600537	1.268877
H	-3.905116	-0.116242	0.469621
H	-3.646346	-1.822957	0.937619
H	-4.343374	-1.430772	-0.658587

ωB97XD energy = -652.083100275 a.u.

Methyl 5-deoxy-2,3-*O*-isopropylidene-*β*-D-*erythro*-pent-4-enofuranoside (**5**), Conf. C

C	-1.249751	-0.770852	0.747729
O	-1.299095	0.632806	0.989108
C	-0.757161	1.344393	-0.048393
C	0.014221	0.420337	-0.976036
C	-0.192578	-0.971001	-0.356219
C	-0.898224	2.661119	-0.162649
O	1.411994	0.607561	-0.978043
C	1.981404	-0.224687	0.024144
O	1.068807	-1.315971	0.170200
C	3.300625	-0.761439	-0.496669

C	2.129357	0.515824	1.349741
O	-2.506464	-1.281114	0.441202
C	-3.167969	-0.686530	-0.662047
H	-0.948601	-1.260599	1.673891
H	-0.332661	0.514268	-2.007150
H	-0.507352	-1.746667	-1.058542
H	-0.420752	3.176428	-0.986917
H	-1.457839	3.235814	0.565650
H	3.138153	-1.273539	-1.446983
H	4.006965	0.058910	-0.645742
H	3.723801	-1.465653	0.223758
H	2.544754	-0.161106	2.100976
H	2.803067	1.367352	1.225268
H	1.165087	0.882944	1.707161
H	-4.120334	-1.206761	-0.763353
H	-2.602413	-0.808845	-1.596758
H	-3.353355	0.378208	-0.488594

ωB97XD energy = -652.080400985 a.u.

Bicyclomycin (**6**), Conf. A

N	-1.972387	1.334565	-0.390008
C	-2.455493	0.294171	0.515141
C	-1.301207	-0.120368	1.450295
N	-0.070487	-0.129909	0.910717
C	0.255325	0.322759	-0.429047
C	-0.719691	1.444762	-0.864075
O	-0.359227	2.336850	-1.623706
O	-1.562717	-0.414515	2.607620
C	-2.970793	-0.916084	-0.276210
O	-3.450242	0.874886	1.290974
C	-1.966207	-1.818576	-0.957884
C	-4.281505	-1.146915	-0.335733
C	-0.986745	-1.166756	-1.945688
O	0.241088	-0.714082	-1.383405
C	1.699234	0.887345	-0.473356
C	2.819286	0.009601	0.142271
C	2.776152	-1.468402	-0.272417
O	3.849231	-2.189308	0.293318
C	4.172579	0.627622	-0.213391
O	2.656621	-0.019839	1.570245
O	1.757330	2.111877	0.228520
H	1.915032	1.032559	-1.536667
H	-2.656073	2.013099	-0.702219
H	0.714356	-0.337726	1.527459
H	-3.306827	0.547431	2.195095
H	-2.533178	-2.570682	-1.514666
H	-1.377327	-2.367237	-0.212096
H	-4.674770	-1.981789	-0.908065
H	-4.985200	-0.514393	0.192814
H	-1.472575	-0.357286	-2.506146
H	-0.661679	-1.918253	-2.666985
H	2.868987	-1.553816	-1.357024
H	1.817964	-1.912956	0.016109
H	3.797371	-2.081013	1.249272
H	4.972305	0.057079	0.260560
H	4.218681	1.668362	0.115950
H	4.329803	0.601532	-1.296128
H	2.730886	0.886259	1.894039
H	1.300083	2.774336	-0.304225

ωB97XD energy = -1103.92528994 a.u.

Bicyclomycin (**6**), Conf. B

N	-1.523468	-0.811071	-1.243330
C	-2.535133	0.010030	-0.585353
C	-1.937159	1.403991	-0.328025
N	-0.620309	1.443164	-0.061853
C	0.286077	0.313637	-0.037049
C	-0.191807	-0.731397	-1.074804
O	0.581548	-1.466412	-1.676803
O	-2.671683	2.378561	-0.380979
C	-2.984141	-0.637777	0.730756
O	-3.582259	0.147326	-1.487500
C	-2.036587	-0.605816	1.908751
C	-4.188313	-1.201740	0.806835
C	-0.634752	-1.195449	1.699403
O	0.357248	-0.269694	1.254825
C	1.718488	0.879937	-0.376136
C	2.950809	0.157475	0.259289
C	3.014463	-1.375091	0.106018
O	3.257508	-1.796971	-1.210149
C	4.225590	0.762161	-0.323706
O	2.953685	0.485872	1.652102
O	1.773470	2.231179	0.042728
H	1.811628	0.871990	-1.465531
H	-1.865274	-1.523039	-1.877468
H	-0.179248	2.344176	0.103632
H	-3.880302	1.069352	-1.412668
H	-2.506381	-1.169860	2.720022
H	-1.918111	0.420623	2.278170
H	-4.518899	-1.688993	1.719197
H	-4.871124	-1.180462	-0.034670
H	-0.664241	-2.068282	1.034677
H	-0.243805	-1.529016	2.662371
H	2.108417	-1.839008	0.516249
H	3.861188	-1.700496	0.718629
H	2.401867	-1.789617	-1.661535
H	4.313016	0.505180	-1.380925
H	5.086884	0.344745	0.203846
H	4.239780	1.847883	-0.212215
H	2.160963	0.085745	2.034646
H	2.188739	2.215787	0.920173

ωB97XD energy = -1103.92482136 a.u.

Bicyclomycin (**6**), Conf. C

N	1.837877	-0.360192	1.419926
C	2.415984	0.450473	0.351486
C	1.332254	1.411699	-0.179027
N	0.072482	0.945655	-0.205664
C	-0.341577	-0.349086	0.298725
C	0.550005	-0.734709	1.513966
O	0.097672	-1.360365	2.464519

O	1.676416	2.528583	-0.539167
C	2.954125	-0.438876	-0.777059
O	3.417685	1.216924	0.934623
C	1.964469	-1.137189	-1.682458
C	4.270103	-0.564189	-0.941800
C	0.903501	-2.022699	-1.014107
O	-0.314623	-1.363850	-0.679776
C	-1.804107	-0.314712	0.800264
C	-2.956119	-0.196735	-0.263758
C	-2.629392	0.627616	-1.514864
O	-2.302729	1.972826	-1.206526
C	-3.405876	-1.591310	-0.695505
O	-4.056925	0.448957	0.367328
O	-1.941343	0.737539	1.742586
H	-1.945624	-1.272138	1.307861
H	2.462127	-0.626906	2.171072
H	-0.657721	1.593456	-0.523646
H	3.331892	2.102337	0.541043
H	2.539588	-1.774283	-2.361130
H	1.442993	-0.405917	-2.312935
H	4.675271	-1.206489	-1.718146
H	4.966549	-0.022277	-0.312743
H	1.314106	-2.538377	-0.136776
H	0.580820	-2.784935	-1.725444
H	-3.504331	0.595600	-2.175252
H	-1.775391	0.205552	-2.049079
H	-3.012563	2.318021	-0.651475
H	-2.591292	-2.125727	-1.189706
H	-4.255569	-1.510190	-1.378805
H	-3.728889	-2.164605	0.177343
H	-3.733001	0.813337	1.204040
H	-1.610910	0.418678	2.590071

ωB97XD energy = -1103.92382803 a.u.

Bicyclomycin (**6**), Conf. D

N	1.527854	-0.433839	1.424304
C	2.526371	0.190801	0.563331
C	1.924926	1.476482	-0.021885
N	0.604613	1.448978	-0.262598
C	-0.304565	0.340560	-0.012506
C	0.200294	-0.454896	1.215391
O	-0.559217	-1.109787	1.922248
O	2.661045	2.428502	-0.235940
C	2.937580	-0.766403	-0.565705
O	3.596105	0.537420	1.378253
C	1.977094	-0.985143	-1.714478
C	4.124794	-1.368094	-0.511111
C	0.569319	-1.503572	-1.379540
O	-0.415383	-0.503743	-1.135248
C	-1.711755	1.004610	0.212571
C	-3.035233	0.182633	0.015244
C	-3.209919	-1.149708	0.761101
O	-2.447596	-2.211116	0.235146
C	-4.192316	1.088406	0.449191
O	-3.187256	-0.040465	-1.381732
O	-1.797441	2.092516	-0.692536
H	-1.697271	1.426515	1.222761
H	1.881392	-1.026666	2.165865
H	0.171935	2.257986	-0.698647
H	3.900940	1.404231	1.061841
H	2.436148	-1.718544	-2.384093
H	1.866455	-0.062933	-2.298452
H	4.430431	-2.069090	-1.282020
H	4.819684	-1.165979	0.295789
H	0.601457	-2.221093	-0.548411
H	0.172015	-2.031048	-2.247830
H	-4.260043	-1.430454	0.636072
H	-3.011849	-1.005160	1.828080
H	-1.624975	-2.217108	0.739490
H	-4.185748	1.221601	1.535839
H	-5.137719	0.624948	0.158603
H	-4.127603	2.071112	-0.019124
H	-2.589893	-0.770389	-1.599342
H	-2.214236	1.725436	-1.489512

ωB97XD energy = -1103.92226264 a.u.

1*α*,4*β*-dihydroxy-8*α*-acetoxy-guaia-2,10(14),11(13)-triene-6,12-olide (**7**), Conf. A

C	2.870531	-2.097305	-0.392665
C	3.747445	-1.190699	0.026131
C	3.144454	0.184479	0.183125
C	1.743368	0.019217	-0.489561
C	1.496632	-1.521280	-0.639420
C	0.619060	0.735327	0.226110
C	-0.767162	0.633113	-0.452820
C	-1.589249	-0.498415	0.163184
C	-1.013176	-1.897559	-0.095765
C	0.432817	-2.065306	0.315979
C	0.755463	-2.622209	1.484707
O	0.933420	2.148603	0.287119
C	-0.163148	2.906372	0.110273
C	-1.306841	2.031260	-0.273061
C	3.990460	1.272415	-0.473528
O	3.048076	0.425369	1.589574
C	-2.528846	2.536028	-0.434159
O	1.036670	-1.844900	-1.954317
H	1.805081	0.429498	-1.504232
O	-0.137471	4.108549	0.240880
O	-2.894854	-0.447563	-0.445435
O	-3.859806	-1.257500	1.422304
C	-3.949762	-0.854055	0.281332
C	-5.225642	-0.723003	-0.495924
H	3.064045	-3.153156	-0.555205
H	4.788630	-1.377565	0.271766
H	0.559712	0.394964	1.264347
H	-0.635270	0.432566	-1.523960
H	-1.702316	-0.326798	1.237690
H	-1.627125	-2.612395	0.458080
H	-1.124526	-2.109334	-1.162380
H	-0.017586	-2.976380	2.161861
H	1.785411	-2.730777	1.810554

H	3.502084	2.248452	-0.384602
H	4.971212	1.325440	0.009503
H	4.138674	1.062031	-1.537034
H	2.893416	1.368155	1.729827
H	-2.698088	3.595199	-0.260576
H	-3.369174	1.925018	-0.741052
H	1.729522	-1.605682	-2.581850
H	-5.434846	0.336537	-0.674330
H	-6.047648	-1.165404	0.065748
H	-5.127911	-1.211123	-1.468788

ωB97XD energy = -1110.92647146 a.u.

1*α*,4*β*-dihydroxy-8*α*-acetoxy-guaia-2,10(14),11(13)-triene-6,12-olide (**7**), Conf. B

C	2.126627	-2.186932	0.802917
C	3.088125	-1.356521	1.192203
C	3.110789	-0.076818	0.394630
C	1.736400	-0.121389	-0.372395
C	1.350375	-1.639049	-0.383993
C	0.732970	0.846162	0.246358
C	-0.708595	0.781620	-0.285936
C	-1.587595	-0.127921	0.562263
C	-0.896461	-1.461324	0.881514
C	-0.131201	-1.998859	-0.304622
C	-0.728898	-2.756109	-1.226598
O	1.191988	2.187326	-0.081717
C	0.162213	3.021029	-0.323307
C	-1.100468	2.233168	-0.331853
C	4.293853	-0.027115	-0.572845
O	3.215693	0.985783	1.337444
C	-2.298928	2.811650	-0.345993
O	1.925093	-2.143365	-1.593923
H	1.858767	0.184582	-1.416577
O	0.325894	4.205542	-0.501435
O	-2.773119	-0.354523	-0.226998
O	-3.983578	-0.784953	1.622064
C	-3.904535	-0.690533	0.414378
C	-5.025786	-0.924352	-0.553271
H	1.939515	-3.182205	1.195837
H	3.826688	-1.546249	1.964970
H	0.744938	0.782255	1.338803
H	-0.691571	0.383447	-1.308841
H	-1.878439	0.370694	1.490588
H	-0.226471	-1.302348	1.733296
H	-1.654049	-2.175315	1.213605
H	-1.773316	-3.035013	-1.116519
H	-0.211317	-3.103316	-2.114450
H	4.221347	-0.829230	-1.312084
H	4.300280	0.931210	-1.104980
H	5.236601	-0.126420	-0.026153
H	3.211467	1.827443	0.864462
H	-2.380696	3.894762	-0.363996
H	-3.216403	2.232344	-0.338506
H	2.050608	-3.095774	-1.501848
H	-4.745292	-1.710453	-1.259735
H	-5.215148	-0.013960	-1.129238
H	-5.926586	-1.213181	-0.012817

ωB97XD energy = -1110.92216307 a.u.

Mexicanin (**8**), Conf. A

C	-1.344907	-0.591792	0.257996
C	-1.623169	0.765630	-0.454980
C	-3.103108	0.689220	-0.750899
C	-3.605819	-0.548157	-0.630844
C	-2.543736	-1.449394	-0.165971
C	-0.017017	-1.272471	-0.176854
C	1.106140	-0.264369	-0.456713
C	1.247517	0.921546	0.517047
C	0.437333	2.155050	0.147261
C	-1.097570	2.036056	0.247011
C	2.508874	-0.803443	-0.522688
C	3.403473	0.285037	-0.043036
O	2.653276	1.285609	0.454776
O	-2.575841	-2.669687	-0.094569
C	-1.714449	3.310122	-0.339660
H	-1.148558	0.741279	-1.449045
O	4.612056	0.346789	-0.074448
C	2.971816	-1.982685	-0.927782
C	-1.490905	-0.488586	1.794859
O	0.422627	-2.217815	0.778635
H	-3.668823	1.545346	-1.103665
H	-4.612449	-0.873084	-0.865363
H	-0.206443	-1.790372	-1.128141
H	0.894132	0.170477	-1.442487
H	1.067433	0.614864	1.549926
H	0.760781	2.981643	0.788544
H	0.704080	2.425675	-0.884038
H	-1.373702	1.991791	1.305801
H	-1.579551	3.351319	-1.427466
H	-2.784020	3.385938	-0.124751
H	-1.233937	4.195895	0.087789
H	4.040040	-2.180819	-0.922373
H	2.306795	-2.773284	-1.259324
H	-1.554163	-1.483718	2.239482
H	-0.654771	0.025751	2.268328
H	-2.409558	0.048772	2.048946
H	-0.251070	-2.907234	0.834191

ωB97XD energy = -883.121954139 a.u.

Neurolenin A (**9**), Conf. A

C	3.410665	0.384811	-1.221947
C	3.357034	-0.945297	-1.365886
C	3.048787	-1.954910	-0.295675
C	1.614017	-2.491749	-0.467448
C	2.157051	2.220386	0.102213
C	3.179553	1.073200	0.069257
C	0.510129	-1.438518	-0.590229
C	0.345759	-0.503417	0.631639
C	-0.105120	0.932907	0.312542
C	0.813236	1.816079	-0.533887

O	3.758623	0.773024	1.104181
C	4.051505	-3.112726	-0.337251
C	2.757823	3.408459	-0.668143
O	1.944322	2.592997	1.447070
H	0.259240	2.742170	-0.718452
O	-0.743770	-2.166857	-0.688016
C	-1.401433	-2.164427	0.484510
C	-0.723282	-1.222219	1.415642
C	-1.083921	-1.073550	2.687330
O	-2.385220	-2.843590	0.674234
O	-1.349533	0.807557	-0.409882
C	-2.320049	1.709168	-0.178073
C	-3.548867	1.415536	-0.996017
O	-2.196679	2.634020	0.598741
C	-4.160283	0.031261	-0.708215
C	-4.504962	-0.125205	0.773903
C	-5.394420	-0.179728	-1.585454
H	3.519721	1.016681	-2.099647
H	3.467219	-1.348975	-2.373410
H	3.129520	-1.478937	0.683949
H	1.563022	-3.124732	-1.361629
H	1.385931	-3.134837	0.391832
H	0.601056	-0.884334	-1.525725
H	1.271026	-0.440712	1.208894
H	-0.302855	1.436063	1.260967
H	0.992493	1.353642	-1.509565
H	3.812279	-3.851495	0.434198
H	5.069916	-2.753611	-0.161192
H	4.029298	-3.619452	-1.308806
H	2.840419	3.205645	-1.739764
H	3.748739	3.656433	-0.275663
H	2.103382	4.273110	-0.528699
H	2.648459	2.174179	1.968225
H	-0.577146	-0.372740	3.344521
H	-1.904134	-1.656135	3.097277
H	-4.276932	2.203458	-0.782822
H	-3.272272	1.482859	-2.054741
H	-3.416597	-0.728116	-0.974472
H	-3.617142	-0.045597	1.411217
H	-5.218105	0.646249	1.090189
H	-4.955728	-1.104128	0.963099
H	-5.153063	-0.079387	-2.649393
H	-6.174303	0.553636	-1.345563
H	-5.814727	-1.178513	-1.428310

ωB97XD energy = -1230.08645640 a.u.

Neurolenin A (**9**), Conf. B

C	3.533497	0.668840	-1.080227
C	3.631119	-0.656838	-1.243447
C	3.350873	-1.717357	-0.215754
C	1.995889	-2.393901	-0.501383
C	1.994494	2.358804	0.131404
C	3.120061	1.312465	0.188155
C	0.803997	-1.449992	-0.677696
C	0.465356	-0.565663	0.546949
C	-0.112084	0.824059	0.220959
C	0.739002	1.811464	-0.576494
O	3.623427	1.051147	1.272340
C	4.474490	-2.758465	-0.197217
C	2.513633	3.591504	-0.626462
O	1.663564	2.729803	1.452494
H	0.092762	2.671652	-0.782213
O	-0.365196	-2.287911	-0.881874
C	-1.119139	-2.358609	0.228177
C	-0.588946	-1.397871	1.233468
C	-1.052190	-1.319514	2.477774
O	-2.067105	-3.106286	0.316110
O	-1.307530	0.575873	-0.551477
C	-2.424753	1.257513	-0.245292
C	-3.594943	0.815901	-1.084409
O	-2.465011	2.126194	0.601562
C	-4.872325	0.609029	-0.255651
C	-6.043116	0.281202	-1.182979
C	-4.677450	-0.487744	0.794133
H	3.650988	1.323381	-1.939983
H	3.865773	-1.023825	-2.243833
H	3.308350	-1.256186	0.772883
H	2.070079	-2.999470	-1.412619
H	1.775694	-3.083521	0.323000
H	0.906122	-0.865443	-1.593421
H	1.333683	-0.426700	1.194809
H	-0.400354	1.292855	1.163699
H	1.018979	1.387663	-1.546129
H	4.584201	-3.241206	-1.175205
H	4.262606	-3.537262	0.542209
H	5.430153	-2.293435	0.062828
H	1.769471	4.387039	-0.533470
H	2.667434	3.386606	-1.689712
H	3.453637	3.944761	-0.191515
H	2.340072	2.340227	2.030065
H	-0.655300	-0.601068	3.189299
H	-1.847105	-1.981495	2.809683
H	-3.759225	1.601184	-1.833116
H	-3.331259	-0.101851	-1.619022
H	-5.089604	1.551237	0.261447
H	-6.196314	1.068518	-1.929603
H	-5.866809	-0.660810	-1.716666
H	-6.972697	0.172587	-0.614367
H	-3.873999	-0.239879	1.496530
H	-4.426236	-1.443535	0.319618
H	-5.593092	-0.631551	1.377522

ωB97XD energy = -1230.08563229 a.u.

Neurolenin A (**9**), Conf. C

C	2.975631	1.063332	-1.392359
C	3.187496	-0.217666	-1.721179
C	3.283124	-1.388315	-0.782801
C	1.981769	-2.213569	-0.824657
C	1.593691	2.413382	0.333497
C	2.810287	1.532660	0.003188
C	0.686507	-1.426912	-0.616959
C	0.550585	-0.713154	0.748609

C	-0.247003	0.603503	0.720496
C	0.272604	1.764843	-0.126185
O	3.589221	1.250478	0.903673
C	4.482141	-2.271365	-1.143951
C	1.774060	3.766882	-0.373282
O	1.555940	2.617833	1.729428
H	-0.496990	2.542923	-0.074787
O	-0.400998	-2.389740	-0.658051
C	-0.871561	-2.652478	0.572467
C	-0.212027	-1.740530	1.546903
C	-0.355820	-1.852343	2.864413
O	-1.708362	-3.504205	0.773728
O	-1.546624	0.259472	0.194805
C	-2.647568	0.673439	0.846296
C	-3.896180	0.126520	0.206549
O	-2.614228	1.382265	1.830839
C	-3.987285	0.366624	-1.310325
C	-5.264788	-0.271857	-1.856498
C	-3.934404	1.860436	-1.638200
H	2.806189	1.796919	-2.176383
H	3.218232	-0.464109	-2.783729
H	3.432430	-1.022009	0.235176
H	1.901092	-2.726352	-1.790518
H	2.045436	-2.991196	-0.053295
H	0.506617	-0.747510	-1.451434
H	1.526017	-0.512738	1.196623
H	-0.366439	0.944232	1.750255
H	0.353907	1.470085	-1.177412
H	5.417895	-1.709237	-1.069531
H	4.394523	-2.658569	-2.165609
H	4.545466	-3.126331	-0.463139
H	2.753602	4.196357	-0.142624
H	1.003165	4.450823	-0.008471
H	1.671262	3.678480	-1.458422
H	2.388988	2.269265	2.086620
H	0.132679	-1.165189	3.549245
H	-0.974920	-2.639260	3.286131
H	-3.913461	-0.951596	0.409417
H	-4.750529	0.578993	0.718192
H	-3.126733	-0.123691	-1.780023
H	-5.290094	-1.348256	-1.655372
H	-6.153865	0.178877	-1.398371
H	-5.339051	-0.129225	-2.939763
H	-3.001525	2.321288	-1.293749
H	-4.766476	2.393274	-1.161879
H	-4.004563	2.024325	-2.718661

ωB97XD energy = -1230.08543291 a.u.

Neurolenin A (**9**), Conf. D

C	2.986482	0.942549	-1.420801
C	3.143969	-0.352164	-1.725633
C	3.201534	-1.506784	-0.764269
C	1.868777	-2.281431	-0.778880
C	1.674995	2.381693	0.285800
C	2.852060	1.445357	-0.034037
C	0.609298	-1.438488	-0.572248

C	0.521955	-0.691917	0.779794
C	-0.226534	0.653045	0.735459
C	0.325666	1.773130	-0.145878
O	3.625460	1.148509	0.866616
C	4.363378	-2.442050	-1.115856
C	1.899323	3.709490	-0.455696
O	1.659558	2.621471	1.676625
H	-0.413174	2.580806	-0.107439
O	-0.518691	-2.354386	-0.578521
C	-0.983578	-2.570496	0.663364
C	-0.263614	-1.675175	1.610214
C	-0.368324	-1.774027	2.932397
O	-1.855978	-3.377973	0.893339
O	-1.546074	0.337133	0.243056
C	-2.610518	0.934570	0.803893
C	-3.901597	0.388705	0.253235
O	-2.518758	1.783630	1.666638
C	-3.945492	0.250579	-1.277128
C	-3.720074	1.601473	-1.959414
C	-5.279938	-0.363862	-1.701858
H	2.840749	1.667283	-2.217698
H	3.155571	-0.620566	-2.783251
H	3.374076	-1.126543	0.244779
H	1.757752	-2.806935	-1.734841
H	1.909385	-3.047254	0.005473
H	0.447221	-0.770026	-1.419074
H	1.510551	-0.519005	1.209089
H	-0.316279	1.021947	1.759088
H	0.385170	1.447151	-1.189192
H	5.319273	-1.912333	-1.063315
H	4.251720	-2.850050	-2.127033
H	4.402878	-3.282047	-0.414958
H	1.780422	3.598983	-1.537138
H	2.896786	4.107120	-0.245433
H	1.158312	4.429882	-0.099374
H	2.479305	2.244032	2.035186
H	0.174249	-1.107185	3.596276
H	-1.006461	-2.531492	3.378987
H	-4.705876	1.041262	0.605619
H	-4.043958	-0.595813	0.717415
H	-3.142085	-0.431201	-1.578041
H	-2.745550	2.030965	-1.700662
H	-4.493124	2.321015	-1.663159
H	-3.756902	1.497468	-3.048946
H	-6.118405	0.284100	-1.417848
H	-5.316782	-0.502026	-2.787659
H	-5.435569	-1.341797	-1.233630

ωB97XD energy = -1230.08543240 a.u.

Neurolenin A (**9**), Conf. E

C	3.043960	1.351240	-1.245632
C	3.413764	0.112755	-1.596980
C	3.558487	-1.081894	-0.695961
C	2.360965	-2.036543	-0.873195
C	1.391647	2.491517	0.385242
C	2.714984	1.746590	0.143217

C	0.977337	-1.398891	-0.733439
C	0.684495	-0.745613	0.637447
C	-0.250977	0.477052	0.596956
C	0.186335	1.715621	-0.183720
O	3.435985	1.503967	1.101647
C	4.869497	-1.818585	-0.990685
C	1.478524	3.869477	-0.290326
O	1.222252	2.664852	1.775697
H	-0.666129	2.403138	-0.165226
O	0.002470	-2.468470	-0.864353
C	-0.504789	-2.819519	0.329573
C	0.000629	-1.875871	1.364143
C	-0.190200	-2.050610	2.668661
O	-1.256658	-3.759492	0.459359
O	-1.474704	0.012271	-0.014675
C	-2.646083	0.278196	0.589260
C	-3.802729	-0.370288	-0.123983
O	-2.743581	0.959948	1.588896
C	-4.852252	0.654727	-0.588359
C	-4.253237	1.644792	-1.590056
C	-6.058736	-0.070483	-1.184889
H	2.865859	2.095468	-2.017354
H	3.556290	-0.084783	-2.660493
H	3.590832	-0.749241	0.343859
H	2.408771	-2.510158	-1.860944
H	2.453393	-2.838010	-0.129622
H	0.778218	-0.718451	-1.562905
H	1.604807	-0.452326	1.145991
H	-0.464383	0.767174	1.627190
H	0.368708	1.467160	-1.233989
H	5.730904	-1.168687	-0.809567
H	4.907666	-2.156114	-2.032810
H	4.966395	-2.698545	-0.346898
H	1.475027	3.790813	-1.381160
H	2.382092	4.398722	0.026996
H	0.608122	4.456626	0.015091
H	2.048131	2.377769	2.198095
H	0.186920	-1.338820	3.397455
H	-0.734498	-2.916558	3.034907
H	-3.431485	-0.950253	-0.973870
H	-4.256726	-1.068112	0.588811
H	-5.183954	1.213267	0.295363
H	-3.421305	2.210030	-1.155034
H	-3.878259	1.121379	-2.478251
H	-5.005948	2.369127	-1.918367
H	-5.769816	-0.647192	-2.072240
H	-6.831112	0.644416	-1.487321
H	-6.504344	-0.764465	-0.463922

ωB97XD energy = -1230.08490772 a.u.

Neurolenin A (**9**), Conf. F

C	3.345474	1.100247	-1.189997
C	3.642165	-0.186932	-1.412626
C	3.588265	-1.311466	-0.417023
C	2.336251	-2.177169	-0.658337
C	1.648109	2.490203	0.186189
C	2.920121	1.627534	0.127215
C	1.006550	-1.422587	-0.705574
C	0.639537	-0.652405	0.584380
C	-0.176276	0.637571	0.371989
C	0.439335	1.770846	-0.447895
O	3.524206	1.403367	1.167614
C	4.851709	-2.173847	-0.501063
C	1.916694	3.806444	-0.560916
O	1.365721	2.777189	1.539141
H	-0.350286	2.521190	-0.564619
O	-0.037899	-2.420605	-0.867863
C	-0.675983	-2.652376	0.291436
C	-0.203852	-1.671715	1.306562
C	-0.550353	-1.729460	2.589245
O	-1.500699	-3.531038	0.410342
O	-1.384082	0.243677	-0.315821
C	-2.573685	0.472914	0.273715
C	-3.686937	-0.207528	-0.478923
O	-2.701214	1.107919	1.298845
C	-5.059738	0.448154	-0.302145
C	-5.105018	1.823347	-0.972115
C	-6.149792	-0.468781	-0.859938
H	3.308353	1.792407	-2.027011
H	3.869767	-0.478436	-2.439071
H	3.537157	-0.894076	0.590612
H	2.441460	-2.721612	-1.604335
H	2.281717	-2.928179	0.139693
H	0.947936	-0.790568	-1.593148
H	1.528739	-0.398668	1.165887
H	-0.446227	1.025555	1.355365
H	0.699014	1.427224	-1.454558
H	5.746086	-1.578097	-0.295075
H	4.960347	-2.622701	-1.495147
H	4.808002	-2.983846	0.233946
H	2.016711	3.654186	-1.639017
H	2.825489	4.283907	-0.182639
H	1.073982	4.479952	-0.383136
H	2.120768	2.455342	2.058156
H	-0.199266	-0.993489	3.306609
H	-1.203020	-2.520721	2.947494
H	-3.416009	-0.275854	-1.537889
H	-3.705752	-1.237830	-0.097807
H	-5.232835	0.580733	0.772231
H	-4.345120	2.498913	-0.564807
H	-4.935676	1.731971	-2.052491
H	-6.081541	2.297245	-0.825554
H	-5.998176	-0.651272	-1.931051
H	-7.139441	-0.016672	-0.734918
H	-6.155916	-1.438647	-0.350850

ωB97XD energy = -1230.08482561 a.u.

Neurolenin A (**9**), Conf. G

C	-2.680735	-1.582218	-1.223051
C	-3.137302	-0.395745	-1.644954
C	-3.454522	0.806786	-0.799822
C	-2.341765	1.866296	-0.932812

C	-1.043271	-2.496827	0.560761
C	-2.412018	-1.896849	0.198812
C	-0.914535	1.369818	-0.692747
C	-0.639246	0.788182	0.714550
C	0.410157	-0.337462	0.757827
C	0.115093	-1.639890	0.013452
O	-3.219002	-1.696648	1.096395
C	-4.805652	1.404512	-1.206857
C	-0.957032	-3.911484	-0.034313
O	-0.949351	-2.576344	1.966717
H	1.023613	-2.246742	0.089839
O	-0.040732	2.525050	-0.808013
C	0.365363	2.956267	0.398074
C	-0.109206	2.000581	1.436066
C	-0.022795	2.236174	2.742178
O	1.016349	3.967773	0.535630
O	1.597814	0.228904	0.166409
C	2.799470	-0.093777	0.674805
C	3.913038	0.585780	-0.076589
O	2.942321	-0.851856	1.612633
C	4.375460	-0.199157	-1.328327
C	4.937744	-1.575707	-0.968714
C	3.281570	-0.312047	-2.393161
H	-2.379386	-2.329101	-1.952447
H	-3.224731	-0.245543	-2.722015
H	-3.526854	0.504538	0.247093
H	-2.371476	2.309605	-1.935265
H	-2.554311	2.673615	-0.220822
H	-0.601833	0.687814	-1.485009
H	-1.553071	0.421187	1.186015
H	0.613418	-0.566020	1.806211
H	-0.046473	-1.444486	-1.051456
H	-5.612068	0.680023	-1.057987
H	-4.804238	1.702463	-2.261730
H	-5.030234	2.291253	-0.605394
H	-0.881980	-3.893750	-1.125477
H	-1.828778	-4.507178	0.253653
H	-0.061025	-4.394105	0.364874
H	-1.831289	-2.369827	2.317724
H	-0.380919	1.516490	3.472814
H	0.414365	3.160759	3.108657
H	3.577660	1.582807	-0.376697
H	4.751944	0.689041	0.616037
H	5.192772	0.398751	-1.749889
H	5.719608	-1.504069	-0.205292
H	4.156653	-2.240543	-0.581779
H	5.370985	-2.053698	-1.853815
H	2.463893	-0.959838	-2.057767
H	3.691733	-0.743959	-3.312360
H	2.853944	0.666088	-2.636939

ωB97XD energy = -1230.08412676 a.u.

Neurolenin A (**9**), Conf. H

C	3.440184	0.932238	-0.977305
C	3.679964	-0.374513	-1.144653
C	3.469019	-1.465679	-0.132152
C	2.197610	-2.267618	-0.473547
C	1.693596	2.429858	0.209730
C	2.924016	1.510619	0.284912
C	0.923555	-1.448647	-0.694939
C	0.435648	-0.625764	0.521562
C	-0.265142	0.702981	0.183355
C	0.530303	1.775733	-0.562570
O	3.424569	1.290028	1.379305
C	4.684126	-2.397930	-0.082986
C	2.113036	3.728737	-0.498242
O	1.277380	2.727025	1.525373
H	-0.180431	2.573747	-0.797566
O	-0.139194	-2.402200	-0.963656
C	-0.921224	-2.579574	0.114545
C	-0.546689	-1.580796	1.151895
C	-1.063281	-1.577491	2.377382
O	-1.777134	-3.434989	0.155639
O	-1.397369	0.344449	-0.640237
C	-2.506536	1.101893	-0.562269
C	-3.624145	0.544468	-1.405016
O	-2.574333	2.103011	0.122065
C	-4.826661	0.053742	-0.564466
C	-4.393046	-0.964941	0.492182
C	-5.622022	1.200067	0.062724
H	3.515500	1.605961	-1.826906
H	3.984479	-0.708099	-2.137943
H	3.348371	-1.019999	0.857413
H	2.367556	-2.853779	-1.384665
H	2.016746	-2.984514	0.337077
H	1.006491	-0.841253	-1.597619
H	1.255886	-0.402405	1.207427
H	-0.640820	1.128147	1.116786
H	0.896529	1.385434	-1.517352
H	4.861205	-2.866702	-1.057837
H	4.526467	-3.194529	0.651259
H	5.586706	-1.848888	0.201897
H	1.292224	4.446252	-0.415303
H	2.323687	3.567087	-1.559226
H	2.998838	4.159789	-0.021612
H	1.981418	2.415540	2.117423
H	-0.775360	-0.832884	3.113774
H	-1.793803	-2.327121	2.668025
H	-3.952460	1.339303	-2.083253
H	-3.234361	-0.281321	-2.004793
H	-5.483012	-0.458593	-1.278803
H	-3.825111	-1.790540	0.052141
H	-3.765621	-0.495658	1.260420
H	-5.266670	-1.389340	0.998067
H	-5.947382	1.921415	-0.695036
H	-5.026798	1.740271	0.805272
H	-6.515480	0.809801	0.562170

ωB97XD energy = -1230.08384465 a.u.

Neurolenin A (**9**), Conf. I

C	2.880363	1.557796	-1.134895
C	3.364969	0.363955	-1.501469

C	3.588057	-0.832184	-0.618299
C	2.479238	-1.879121	-0.845493
C	1.103721	2.514823	0.483791
C	2.490129	1.891316	0.254704
C	1.044328	-1.359086	-0.741454
C	0.649214	-0.769891	0.633481
C	-0.383185	0.372285	0.582330
C	-0.017107	1.664974	-0.146397
O	3.211291	1.692898	1.222954
C	4.964701	-1.450360	-0.882677
C	1.096852	3.918783	-0.141300
O	0.884986	2.624120	1.874064
H	-0.923351	2.280977	-0.141423
O	0.166848	-2.500955	-0.935770
C	-0.349915	-2.926780	0.229074
C	0.034022	-1.971847	1.304392
C	-0.197525	-2.193397	2.595134
O	-1.021633	-3.931110	0.307467
O	-1.534858	-0.164445	-0.101888
C	-2.741557	-0.103585	0.492102
C	-3.803009	-0.745104	-0.361956
O	-2.929084	0.419639	1.571293
C	-4.618259	0.278832	-1.187689
C	-5.484334	1.179789	-0.305581
C	-3.727273	1.106119	-2.117099
H	2.652356	2.299207	-1.896138
H	3.547049	0.200998	-2.564776
H	3.559099	-0.518772	0.427365
H	2.595245	-2.332826	-1.837152
H	2.612888	-2.681559	-0.109077
H	0.817018	-0.675098	-1.560545
H	1.521893	-0.416576	1.186817
H	-0.665932	0.611852	1.608954
H	0.219043	1.462155	-1.196002
H	5.064622	-1.760874	-1.929111
H	5.115169	-2.332566	-0.252378
H	5.761784	-0.734305	-0.661033
H	0.171401	4.421044	0.152794
H	1.137393	3.881393	-1.233480
H	1.942381	4.508038	0.226317
H	1.726618	2.409774	2.308389
H	0.088279	-1.471669	3.354711
H	-0.685790	-3.108493	2.918231
H	-3.326892	-1.462099	-1.035562
H	-4.475298	-1.288460	0.307783
H	-5.286828	-0.327070	-1.811596
H	-6.136644	0.588995	0.346242
H	-4.869980	1.824063	0.332732
H	-6.117320	1.824119	-0.925399
H	-3.100746	0.467893	-2.749297
H	-3.065142	1.768079	-1.545903
H	-4.339290	1.736709	-2.770610

ωB97XD energy = -1230.08381885 a.u.

Swinhoeisterol F (**10**), Conf. A

C	4.483940	-2.885889	0.194225
C	4.916839	-2.168420	-1.085830
C	4.734499	-0.665756	-0.938064
C	3.324427	-0.257671	-0.538980
C	2.821969	-0.998277	0.739821
C	3.049705	-2.515484	0.560278
C	3.129515	1.266207	-0.460793
C	1.870283	1.694755	0.334593
C	0.793440	0.626953	0.345129
C	1.293101	-0.758995	0.799205
C	-0.511751	0.951740	1.113357
C	-0.839047	-0.381489	1.830981
C	0.545431	-0.982835	2.119993
C	1.431235	3.074746	-0.142673
C	0.308416	3.221429	-1.147709
C	-1.076620	2.688776	-0.733560
C	-1.090354	1.201239	-0.323946
C	3.573325	-0.525769	1.994548
C	-0.538106	2.085622	2.131128
C	5.740100	0.180157	-1.177395
O	6.221418	-2.554388	-1.481266
O	2.047929	4.056607	0.245482
C	-2.381124	0.481984	-0.723159
C	-3.570795	1.017418	0.086357
C	-2.628483	0.511203	-2.235432
C	-4.903029	0.300292	-0.172015
C	-4.907555	-1.206445	0.134612
C	-4.726601	-1.465755	1.633734
C	-6.158331	-1.930933	-0.422584
C	-7.482181	-1.310098	0.034735
C	-6.121530	-2.048470	-1.949619
H	2.672394	-0.624849	-1.347726
H	2.163013	1.845343	1.378291
H	0.851284	-1.445648	0.066467
O	0.069740	0.546364	-0.910517
H	5.177974	-2.622004	1.003375
H	4.567114	-3.966221	0.036930
H	4.265406	-2.504186	-1.903729
H	2.749063	-3.038378	1.475596
H	2.389377	-2.876912	-0.239216
H	3.086531	1.662761	-1.481165
H	3.986370	1.742137	0.022437
H	-1.430649	-0.218636	2.737068
H	-1.400201	-1.071687	1.195395
H	0.496387	-2.037355	2.408169
H	1.030359	-0.437687	2.939321
H	0.623364	2.685164	-2.050050
H	0.230788	4.284354	-1.389959
H	-1.728467	2.836588	-1.598225
H	-1.495551	3.302854	0.069196
H	3.332981	-1.164269	2.851266
H	3.335120	0.502280	2.276242
H	4.656171	-0.570628	1.841910
H	-0.117225	3.027788	1.773798
H	0.025097	1.802475	3.027074
H	-1.570268	2.285127	2.439716

H	5.636243	1.255407	-1.082905
H	6.706827	-0.186318	-1.509015
H	6.824740	-2.387883	-0.746485
H	-2.225164	-0.567976	-0.459245
H	-3.709527	2.082354	-0.142613
H	-3.326506	0.964371	1.154966
H	-3.351741	-0.259497	-2.519682
H	-1.702363	0.312745	-2.784439
H	-3.029357	1.472988	-2.574787
H	-5.194725	0.465505	-1.215583
H	-5.673911	0.787148	0.439034
H	-4.049525	-1.663477	-0.378211
H	-4.745343	-2.539552	1.851907
H	-3.770781	-1.074875	1.996955
H	-5.519892	-0.988504	2.221489
H	-6.119707	-2.953845	-0.021691
H	-8.325593	-1.940854	-0.267373
H	-7.529919	-1.196062	1.122824
H	-7.636447	-0.322181	-0.414463
H	-6.960457	-2.653458	-2.311800
H	-5.195666	-2.525857	-2.290304
H	-6.194615	-1.068651	-2.435982

ωB97XD energy = -1358.21103127 a.u.

Swinhoeisterol F (**10**), Conf. B

C	4.451903	-2.949667	0.152609
C	4.904159	-2.186453	-1.088023
C	4.777999	-0.692265	-0.844880
C	3.359235	-0.268849	-0.504972
C	2.802709	-1.050546	0.728777
C	3.016683	-2.565747	0.506525
C	3.184665	1.253033	-0.373092
C	1.905311	1.670248	0.395954
C	0.810839	0.621478	0.328075
C	1.276421	-0.788915	0.743487
C	-0.513317	0.938059	1.067454
C	-0.885097	-0.421554	1.709017
C	0.479125	-1.052338	2.027508
C	1.509022	3.076568	-0.037147
C	0.430106	3.279798	-1.077870
C	-0.977464	2.763678	-0.725684
C	-1.040539	1.261175	-0.374886
C	3.519222	-0.635477	2.023650
C	-0.556379	2.024009	2.135628
C	5.834997	0.120201	-0.920542
O	6.213832	-2.617161	-1.413282
O	2.122312	4.030945	0.418691
C	-2.342033	0.601218	-0.842042
C	-3.540804	1.177342	-0.073476
C	-2.520839	0.672037	-2.362787
C	-4.896149	0.542112	-0.411205
C	-5.000068	-0.973734	-0.177910
C	-4.738252	-1.329489	1.289517
C	-6.343202	-1.532895	-0.710611
C	-6.297031	-3.053836	-0.887622
C	-7.554146	-1.137629	0.141168
H	2.734079	-0.596029	-1.350909
H	2.163387	1.772109	1.454414
H	0.851598	-1.440171	-0.030531
O	0.121599	0.599846	-0.950387
H	5.143227	-2.729677	0.974476
H	4.514701	-4.024339	-0.047147
H	4.219516	-2.446790	-1.912378
H	2.694314	-3.109891	1.401756
H	2.363748	-2.895775	-0.312479
H	3.183644	1.690088	-1.377826
H	4.030157	1.696647	0.157978
H	-1.517109	-0.295564	2.593281
H	-1.420148	-1.075909	1.015799
H	0.406426	-2.116154	2.272919
H	0.940237	-0.545020	2.883981
H	0.765935	2.765776	-1.985587
H	0.381088	4.351210	-1.288483
H	-1.598626	2.960286	-1.603294
H	-1.405808	3.357373	0.087355
H	3.229080	-1.291994	2.850535
H	3.301049	0.390834	2.327332
H	4.604838	-0.710533	1.908523
H	-0.103366	2.972293	1.838768
H	-0.034548	1.687482	3.037909
H	-1.595876	2.230681	2.413336
H	5.766739	1.189368	-0.752117
H	6.822287	-0.271942	-1.145775
H	6.464327	-2.234283	-2.261974
H	-2.246931	-0.459913	-0.593086
H	-3.615070	2.253230	-0.278921
H	-3.349870	1.084679	1.003227
H	-3.257847	-0.065112	-2.696327
H	-1.579215	0.451584	-2.875757
H	-2.871626	1.654826	-2.696956
H	-5.138851	0.753621	-1.460660
H	-5.662790	1.051485	0.186876
H	-4.216656	-1.456328	-0.779898
H	-5.385201	-0.750927	1.959479
H	-4.910496	-2.392073	1.488896
H	-3.702491	-1.114257	1.567828
H	-6.483853	-1.094503	-1.709256
H	-7.218326	-3.420334	-1.353931
H	-5.457408	-3.356248	-1.523973
H	-6.191665	-3.568773	0.074510
H	-8.482857	-1.463618	-0.340125
H	-7.623200	-0.054952	0.289227
H	-7.514935	-1.610889	1.129085

ωB97XD energy = -1358.21102367 a.u.

Swinhoeisterol F (**10**), Conf. C

C	-4.999388	-2.502771	0.165238
C	-5.469560	-1.378905	1.082566
C	-5.041443	-0.036850	0.513317
C	-3.537455	0.077278	0.329188
C	-2.973886	-1.079163	-0.558209
C	-3.484041	-2.430813	-0.008228

C	-3.078724	1.467026	-0.142562
C	-1.654571	1.477401	-0.753782
C	-0.787758	0.344577	-0.238103
C	-1.436843	-1.047222	-0.373253
C	0.659193	0.262882	-0.784319
C	0.865641	-1.255349	-1.010626
C	-0.537186	-1.751126	-1.398128
C	-1.066238	2.877044	-0.618990
C	-0.108386	3.205113	0.506722
C	1.210760	2.412236	0.565858
C	1.034128	0.880341	0.609249
C	-3.427370	-0.934733	-2.019962
C	1.037589	1.011567	-2.056968
C	-5.933658	0.909848	0.212063
O	-6.867638	-1.516912	1.264313
O	-1.436209	3.749831	-1.391293
C	2.112273	0.184398	1.444217
C	3.480483	0.328525	0.763746
C	2.109350	0.642755	2.907336
C	4.555197	-0.590357	1.343126
C	5.950189	-0.441091	0.712149
C	6.930538	-1.392751	1.406994
C	5.947611	-0.585066	-0.830512
C	7.344364	-0.384757	-1.428096
C	5.340388	-1.902625	-1.323060
H	-3.094831	-0.089680	1.323923
H	-1.751790	1.321493	-1.832425
H	-1.240068	-1.520145	0.597011
O	-0.298208	0.577325	1.109106
H	-5.519012	-2.417856	-0.796318
H	-5.281646	-3.464131	0.606586
H	-4.962677	-1.503654	2.053921
H	-3.143283	-3.239006	-0.665519
H	-3.020639	-2.607052	0.971799
H	-3.134314	2.157750	0.706215
H	-3.753370	1.858342	-0.907741
H	1.617367	-1.451482	-1.781324
H	1.185539	-1.773190	-0.102667
H	-0.627740	-2.841150	-1.365302
H	-0.782205	-1.428996	-2.417987
H	-0.652837	3.028214	1.441456
H	0.111827	4.273348	0.437696
H	1.722584	2.747282	1.471603
H	1.861498	2.690435	-0.268739
H	-3.182195	-1.837684	-2.589164
H	-2.962652	-0.089128	-2.532534
H	-4.510232	-0.789125	-2.081709
H	0.748766	2.064729	-2.069460
H	0.566774	0.539327	-2.926133
H	2.122233	0.969258	-2.207372
H	-5.650335	1.875525	-0.192090
H	-6.996378	0.739281	0.355844
H	-7.155014	-0.897024	1.944499
H	1.854614	-0.880257	1.452856
H	3.817707	1.372786	0.830776
H	3.353447	0.110218	-0.303076
H	2.647239	-0.071815	3.537250
H	1.086441	0.712414	3.291430
H	2.591126	1.618119	3.037771
H	4.221590	-1.634006	1.253129
H	4.656649	-0.396929	2.418123
H	6.289759	0.585139	0.920112
H	7.964779	-1.218821	1.095267
H	6.890640	-1.252810	2.493052
H	6.689353	-2.442595	1.203175
H	5.321391	0.228272	-1.220140
H	7.286464	-0.285459	-2.517748
H	7.821349	0.520699	-1.034927
H	8.003543	-1.232935	-1.211152
H	5.352676	-1.942311	-2.418098
H	4.300110	-2.023395	-1.002669
H	5.905012	-2.768431	-0.958043

ωB97XD energy = -1358.21099966 a.u.

Swinhoeisterol F (**10**), Conf. D

C	-5.225703	-2.250695	0.301944
C	-5.613658	-1.067590	1.190577
C	-5.065836	0.229484	0.615332
C	-3.564243	0.205261	0.372733
C	-3.117962	-1.012072	-0.495621
C	-3.713180	-2.302355	0.109481
C	-3.009356	1.542628	-0.146308
C	-1.607820	1.427104	-0.798263
C	-0.814865	0.240342	-0.284147
C	-1.578413	-1.097580	-0.354758
C	0.597927	0.027910	-0.882063
C	0.676798	-1.508297	-1.064458
C	-0.773728	-1.902914	-1.384505
C	-0.911319	2.781198	-0.712908
C	0.115812	3.052752	0.365536
C	1.371730	2.161877	0.379688
C	1.077401	0.650586	0.475599
C	-3.599160	-0.873069	-1.949342
C	0.985328	0.706729	-2.190265
C	-5.864555	1.275092	0.385698
O	-7.007633	-1.042900	1.443034
O	-1.247231	3.666399	-1.486530
C	2.128118	-0.112104	1.286676
C	3.460953	-0.154858	0.528074
C	2.269954	0.412044	2.720176
C	4.485609	-1.102473	1.155419
C	5.722796	-1.393712	0.283605
C	5.335148	-2.223249	-0.949098
C	6.515156	-0.122634	-0.101065
C	6.878418	0.724268	1.123370
C	7.787790	-0.462167	-0.885352
H	-3.107882	0.027360	1.359678
H	-1.749978	1.257472	-1.870011
H	-1.389783	-1.556790	0.623599
O	-0.253237	0.467044	1.036030
H	-5.741226	-2.156396	-0.663357
H	-5.580154	-3.174775	0.770165

H	-5.152957	-1.214979	2.176469
H	-3.441675	-3.157710	-0.520086
H	-3.250671	-2.474854	1.090542
H	-2.986364	2.254760	0.685857
H	-3.674762	1.968202	-0.901339
H	1.381930	-1.788320	-1.853042
H	0.990815	-2.020293	-0.151156
H	-0.946189	-2.980835	-1.310693
H	-1.030873	-1.595381	-2.405904
H	-0.398623	2.929418	1.325379
H	0.412596	4.100250	0.268761
H	1.955758	2.477659	1.247779
H	1.995180	2.367978	-0.495508
H	-4.668781	-0.645111	-1.990054
H	-3.440452	-1.807915	-2.497286
H	-3.084519	-0.081050	-2.498587
H	0.766928	1.776073	-2.231387
H	0.459099	0.238610	-3.029375
H	2.059739	0.584947	-2.367792
H	-5.501146	2.212507	-0.020710
H	-6.922248	1.228207	0.626446
H	-7.471071	-1.044999	0.596401
H	1.756346	-1.139220	1.370531
H	3.878994	0.858918	0.468266
H	3.263453	-0.468467	-0.504277
H	2.774935	-0.326679	3.349696
H	1.288109	0.604647	3.164717
H	2.856333	1.336456	2.769822
H	3.991451	-2.060619	1.367968
H	4.805427	-0.707986	2.126189
H	6.394413	-2.013138	0.897925
H	4.842735	-1.609133	-1.712759
H	6.206461	-2.691129	-1.416140
H	4.643126	-3.026619	-0.672418
H	5.874545	0.486125	-0.756149
H	7.492157	1.583205	0.830596
H	5.995207	1.115221	1.638129
H	7.456020	0.134746	1.846940
H	8.357747	0.446831	-1.107654
H	7.572528	-0.952896	-1.838989
H	8.437294	-1.127679	-0.301983

ωB97XD energy = -1358.21082835 a.u.

Swinhoeisterol F (**10**), Conf. E

C	-5.307832	-2.006029	0.083000
C	-5.682949	-0.759036	0.885597
C	-4.979130	0.464473	0.318914
C	-3.468291	0.313414	0.226079
C	-3.035830	-0.974621	-0.540826
C	-3.793105	-2.185072	0.047424
C	-2.759374	1.577476	-0.288579
C	-1.312809	1.323960	-0.786072
C	-0.675595	0.110354	-0.135643
C	-1.531610	-1.169131	-0.230588
C	0.773998	-0.238882	-0.553676
C	0.745808	-1.782265	-0.665886
C	-0.688473	-2.086279	-1.126197
C	-0.522835	2.624296	-0.685861
C	0.404369	2.877670	0.484427
C	1.570600	1.894529	0.694133
C	1.147106	0.417284	0.830393
C	-3.351535	-0.868160	-2.041616
C	1.323303	0.352017	-1.848134
C	-5.662401	1.561438	-0.018260
O	-7.088111	-0.615257	0.997131
O	-0.706043	3.491662	-1.527934
C	1.995177	-0.341594	1.867855
C	3.504349	-0.327516	1.578114
C	1.722333	0.188608	3.282749
C	3.940170	-1.172305	0.382366
C	5.457826	-1.254744	0.155167
C	5.741732	-2.180321	-1.033508
C	6.127823	0.136044	0.021107
C	7.642009	0.025368	-0.184711
C	5.501183	1.015251	-1.065983
H	-3.127281	0.151165	1.261198
H	-1.358736	1.113251	-1.858882
H	-1.482675	-1.588183	0.782088
O	-0.253895	0.367178	1.226262
H	-5.717224	-1.912300	-0.931882
H	-5.780627	-2.879204	0.544609
H	-5.333263	-0.897068	1.917442
H	-3.534060	-3.086489	-0.520119
H	-3.445460	-2.351084	1.075754
H	-2.760757	2.325990	0.511363
H	-3.308215	2.015662	-1.125889
H	1.506250	-2.152712	-1.359724
H	0.919250	-2.268472	0.297643
H	-0.950362	-3.143387	-1.019643
H	-0.811785	-1.820642	-2.183116
H	-0.221039	2.858019	1.384803
H	0.800073	3.889605	0.366311
H	2.074949	2.219586	1.607960
H	2.307872	1.998585	-0.108169
H	-3.222480	-1.838381	-2.532975
H	-2.714020	-0.151627	-2.564442
H	-4.386740	-0.551520	-2.203333
H	1.203706	1.433378	-1.940382
H	0.813329	-0.099112	-2.706538
H	2.390803	0.136051	-1.956727
H	-5.185147	2.449175	-0.418452
H	-6.737255	1.613211	0.125922
H	-7.465784	-0.610824	0.108846
H	1.654429	-1.382764	1.854794
H	4.019833	-0.702439	2.472658
H	3.835618	0.711016	1.456115
H	2.138781	-0.501935	4.023657
H	0.649991	0.282063	3.474358
H	2.187358	1.166110	3.453615
H	3.469763	-0.799199	-0.532496
H	3.558669	-2.192561	0.519223
H	5.900529	-1.721747	1.048255

H	6.811478	-2.365630	-1.167643
H	5.257449	-3.151872	-0.884232
H	5.353128	-1.762789	-1.970087
H	5.978630	0.648431	0.980673
H	8.120699	1.004067	-0.068995
H	8.094607	-0.656279	0.545077
H	7.888927	-0.343083	-1.186887
H	6.017908	1.979687	-1.122411
H	4.444036	1.222831	-0.869046
H	5.573138	0.547382	-2.054673

ωB97XD energy = -1358.21035886 a.u.

Swinhoeisterol F (**10**), Conf. F

C	4.935429	-2.658220	0.009214
C	5.375974	-1.739473	-1.131342
C	5.019126	-0.294317	-0.817635
C	3.550628	-0.082079	-0.481376
C	3.048052	-1.034934	0.646956
C	3.446633	-2.483578	0.291713
C	3.194285	1.390404	-0.216299
C	1.848265	1.582541	0.527509
C	0.889094	0.426322	0.315414
C	1.502425	-0.955205	0.620039
C	-0.486628	0.516662	1.020754
C	-0.717248	-0.928566	1.528500
C	0.703100	-1.424533	1.843589
C	1.299573	2.968426	0.206114
C	0.231282	3.145296	-0.851416
C	-1.114712	2.433640	-0.616372
C	-0.999829	0.908888	-0.409461
C	3.663761	-0.664666	2.006298
C	-0.692256	1.489530	2.175857
C	5.937753	0.673009	-0.882233
O	6.740257	-1.936932	-1.459636
O	1.788015	3.940484	0.764248
C	-2.189045	0.136717	-0.987789
C	-3.465534	0.436763	-0.190419
C	-2.360594	0.355006	-2.495330
C	-4.648131	-0.455343	-0.579077
C	-5.816540	-0.406485	0.417462
C	-6.415850	1.001342	0.510020
C	-6.905286	-1.472866	0.139077
C	-7.571852	-1.327058	-1.232843
C	-6.381305	-2.900315	0.326841
H	2.995515	-0.393510	-1.380871
H	2.057718	1.613373	1.601315
H	1.180885	-1.575323	-0.225935
O	0.254073	0.452790	-0.990379
H	5.533321	-2.431334	0.902250
H	5.148824	-3.695431	-0.269418
H	4.826619	-2.028159	-2.037496
H	3.134546	-3.155215	1.099795
H	2.890924	-2.791252	-0.604309
H	3.177764	1.920416	-1.174829
H	3.964778	1.869907	0.392747
H	-1.383372	-0.951954	2.396304
H	-1.155526	-1.575289	0.763832
H	0.752620	-2.506925	1.996566
H	1.076111	-0.943520	2.756261
H	0.655635	2.765160	-1.787773
H	0.061389	4.219060	-0.963176
H	-1.722260	2.640062	-1.500983
H	-1.644249	2.888396	0.226148
H	3.476235	-1.452599	2.743404
H	3.266243	0.265977	2.417872
H	4.747836	-0.538787	1.923416
H	-0.351319	2.508194	1.979328
H	-0.159377	1.137439	3.065850
H	-1.756722	1.545570	2.429291
H	5.710792	1.712528	-0.672803
H	6.958343	0.450847	-1.178488
H	7.268583	-1.802396	-0.663105
H	-1.956418	-0.925454	-0.855156
H	-3.732149	1.493512	-0.319601
H	-3.256090	0.294858	0.878908
H	-2.998503	-0.423878	-2.923770
H	-1.394423	0.308671	-3.008012
H	-2.826671	1.319234	-2.725878
H	-4.283289	-1.487395	-0.659953
H	-5.008240	-0.172795	-1.576313
H	-5.403061	-0.648892	1.408649
H	-6.713080	1.378802	-0.475529
H	-7.302741	1.010469	1.154059
H	-5.699518	1.714884	0.929670
H	-7.682878	-1.314186	0.900346
H	-8.383503	-2.055049	-1.342105
H	-8.002946	-0.331100	-1.378296
H	-6.859130	-1.509415	-2.045378
H	-7.205020	-3.622374	0.299722
H	-5.870155	-3.014195	1.289979
H	-5.676207	-3.183098	-0.462991

ωB97XD energy = -1358.21019031 a.u.

Swinhoeisterol F (**10**), Conf. G

C	4.363465	-2.972690	0.157626
C	4.803540	-2.269806	-1.127959
C	4.670984	-0.761786	-0.979397
C	3.280698	-0.307513	-0.561201
C	2.770323	-1.033215	0.722877
C	2.946253	-2.556798	0.540156
C	3.137858	1.221881	-0.476696
C	1.902311	1.689535	0.334188
C	0.790803	0.657884	0.356757
C	1.251052	-0.744479	0.801986
C	-0.491891	1.024199	1.144079
C	-0.853448	-0.299283	1.862856
C	0.514236	-0.946150	2.132505
C	1.503735	3.084640	-0.134228
C	0.374415	3.269560	-1.124786
C	-1.021887	2.785150	-0.690469
C	-1.081993	1.297417	-0.284108
C	3.552448	-0.586338	1.968368

C	-0.464676	2.155626	2.164410
C	5.700608	0.050152	-1.233707
O	6.090722	-2.696870	-1.538148
O	2.157009	4.044718	0.248478
C	-2.403512	0.626406	-0.668068
C	-3.561288	1.194664	0.165092
C	-2.673799	0.679105	-2.176166
C	-4.924042	0.539850	-0.101822
C	-4.989743	-0.986984	0.088260
C	-4.692774	-1.354946	1.546098
C	-6.344876	-1.558331	-0.398556
C	-6.541974	-1.365657	-1.907965
C	-6.505094	-3.046277	-0.064045
H	2.606263	-0.650642	-1.362200
H	2.211978	1.828019	1.374673
H	0.777548	-1.415175	0.074326
O	0.046488	0.604121	-0.888506
H	5.074839	-2.730394	0.958449
H	4.410282	-4.055233	0.000237
H	4.132215	-2.585538	-1.937641
H	2.639471	-3.070340	1.458696
H	2.265692	-2.896918	-0.251706
H	3.096946	1.623522	-1.495169
H	4.015207	1.667365	-0.001414
H	-1.427811	-0.120091	2.777038
H	-1.444864	-0.968827	1.232662
H	0.434569	-1.999054	2.419595
H	1.027706	-0.418735	2.946236
H	0.658143	2.721591	-2.030491
H	0.330172	4.334320	-1.367411
H	-1.681021	2.958991	-1.544561
H	-1.406460	3.411556	0.119845
H	3.303952	-1.218542	2.827487
H	3.348660	0.447988	2.254042
H	4.631346	-0.663645	1.801748
H	-0.022140	3.085302	1.800577
H	0.106200	1.853355	3.049169
H	-1.484390	2.385381	2.492781
H	5.634374	1.128432	-1.139021
H	6.649541	-0.349635	-1.577860
H	6.706582	-2.551569	-0.809274
H	-2.281493	-0.431115	-0.414899
H	-3.660522	2.268519	-0.041825
H	-3.306956	1.113245	1.228834
H	-3.424075	-0.066375	-2.457213
H	-1.763296	0.459193	-2.742841
H	-3.051825	1.655477	-2.499255
H	-5.228655	0.797540	-1.120505
H	-5.666793	1.001950	0.563746
H	-4.214096	-1.451107	-0.539715
H	-4.566521	-2.432373	1.682902
H	-3.770822	-0.881387	1.895394
H	-5.505067	-1.023214	2.206014
H	-7.142613	-1.007966	0.124067
H	-7.494531	-1.799901	-2.230469
H	-6.549479	-0.313869	-2.206749
H	-5.743318	-1.869348	-2.467973
H	-7.429147	-3.440694	-0.500753
H	-6.549489	-3.230810	1.012981
H	-5.670445	-3.629899	-0.473651

ωB97XD energy = -1358.21017261 a.u.

Swinhoeisterol F (**10**), Conf. H

C	5.023979	-2.638895	-0.036618
C	5.476675	-1.669307	-1.129280
C	5.096288	-0.241920	-0.764998
C	3.618522	-0.060696	-0.451224
C	3.106422	-1.062791	0.629204
C	3.528273	-2.491652	0.224081
C	3.236921	1.396218	-0.138313
C	1.874517	1.543661	0.586400
C	0.934932	0.383808	0.313828
C	1.560880	-1.000226	0.577440
C	-0.454396	0.429510	0.997063
C	-0.674310	-1.037197	1.444308
C	0.746723	-1.524971	1.768142
C	1.312188	2.933485	0.307855
C	0.259234	3.136150	-0.760702
C	-1.080550	2.397752	-0.578958
C	-0.947658	0.868426	-0.426376
C	3.692621	-0.740675	2.013554
C	-0.693035	1.354527	2.184699
C	6.005120	0.736724	-0.769960
O	6.849308	-1.838378	-1.437775
O	1.777068	3.890127	0.910849
C	-2.115209	0.102720	-1.055310
C	-3.413890	0.369234	-0.281496
C	-2.251505	0.363544	-2.559766
C	-4.566213	-0.551753	-0.689828
C	-5.789270	-0.477501	0.238474
C	-6.449316	0.904721	0.175330
C	-6.776615	-1.640150	-0.028365
C	-7.807684	-1.792069	1.094292
C	-7.480721	-1.547845	-1.386187
H	3.085604	-0.344277	-1.373073
H	2.064215	1.536151	1.664322
H	1.261954	-1.591570	-0.297041
O	0.322387	0.451070	-1.001199
H	5.601793	-2.443052	0.876666
H	5.254201	-3.661356	-0.353762
H	4.948812	-1.926613	-2.057389
H	3.208991	-3.198544	0.998546
H	2.992866	-2.768588	-0.693988
H	3.231780	1.961072	-1.076875
H	3.988506	1.862522	0.503589
H	-1.357576	-1.103576	2.296375
H	-1.087457	-1.659531	0.646262
H	0.809433	-2.611341	1.883571
H	1.096094	-1.071695	2.704159
H	0.705282	2.799615	-1.703469
H	0.076008	4.211094	-0.833782
H	-1.673575	2.627748	-1.467761

H	-1.633368	2.813322	0.268789
H	3.489980	-1.554484	2.717716
H	3.284997	0.174687	2.448620
H	4.778018	-0.611236	1.959045
H	-0.364604	2.384721	2.031840
H	-0.168739	0.976541	3.069127
H	-1.761895	1.385943	2.423413
H	5.762588	1.764803	-0.524312
H	7.034232	0.536330	-1.051996
H	7.360116	-1.735163	-0.625371
H	-1.875632	-0.960569	-0.947326
H	-3.704756	1.418810	-0.416998
H	-3.222517	0.232015	0.791832
H	-2.866045	-0.411220	-3.027912
H	-1.272256	0.346814	-3.048737
H	-2.726112	1.327645	-2.773414
H	-4.194354	-1.586326	-0.701426
H	-4.873492	-0.320314	-1.717506
H	-5.420442	-0.621889	1.265528
H	-7.364502	0.949759	0.774400
H	-5.776443	1.678973	0.556984
H	-6.708148	1.172991	-0.855868
H	-6.170734	-2.557886	-0.032981
H	-8.385806	-2.713744	0.963990
H	-7.323985	-1.838239	2.076775
H	-8.521127	-0.960144	1.107501
H	-8.092063	-2.440065	-1.561390
H	-6.769012	-1.470182	-2.214493
H	-8.148466	-0.679502	-1.430921

ωB97XD energy = -1358.21011003 a.u.

Swinhoeisterol F (**10**), Conf. I

C	-5.428804	-1.869255	0.145085
C	-5.732326	-0.613084	0.963509
C	-4.986697	0.583659	0.392797
C	-3.486204	0.366439	0.264978
C	-3.129822	-0.931500	-0.524514
C	-3.924685	-2.112582	0.073345
C	-2.731159	1.603318	-0.250990
C	-1.311439	1.289187	-0.789267
C	-0.712219	0.040284	-0.170237
C	-1.628737	-1.197103	-0.255177
C	0.706716	-0.368291	-0.636340
C	0.604673	-1.907355	-0.759905
C	-0.853840	-2.142368	-1.182849
C	-0.458091	2.549522	-0.703224
C	0.510851	2.748912	0.443241
C	1.634583	1.711866	0.622558
C	1.150971	0.253412	0.745956
C	-3.477381	-0.796984	-2.016100
C	1.230067	0.205008	-1.949590
C	-5.628091	1.713161	0.082068
O	-7.127173	-0.408575	1.104943
O	-0.622633	3.431211	-1.534108
C	2.000879	-0.557797	1.743618
C	3.514013	-0.491798	1.489274
C	1.691525	-0.139373	3.187017
C	3.960048	-0.982829	0.114530
C	5.475932	-1.171327	-0.053085
C	5.766642	-1.702303	-1.461178
C	6.286445	0.098711	0.305973
C	7.796718	-0.132717	0.193185
C	5.872436	1.336022	-0.497155
H	-3.130477	0.176908	1.290542
H	-1.398279	1.091850	-1.861845
H	-1.572660	-1.629198	0.751569
O	-0.238968	0.257332	1.181022
H	-5.856670	-1.752086	-0.859659
H	-5.927628	-2.724724	0.612506
H	-5.367831	-0.774330	1.986858
H	-3.716979	-3.020440	-0.504756
H	-3.561083	-2.299546	1.092534
H	-2.677682	2.339892	0.558277
H	-3.279239	2.078342	-1.068616
H	1.330488	-2.305969	-1.475255
H	0.777603	-2.409285	0.195613
H	-1.160476	-3.187574	-1.078045
H	-0.995074	-1.861169	-2.233372
H	-0.093133	2.752037	1.358428
H	0.950096	3.742390	0.322804
H	2.163452	2.001702	1.534778
H	2.366961	1.791264	-0.187511
H	-3.410745	-1.768907	-2.516424
H	-2.816249	-0.110762	-2.550456
H	-4.497802	-0.424527	-2.150568
H	1.231274	1.295806	-2.001698
H	0.610198	-0.153193	-2.779288
H	2.252331	-0.131692	-2.143039
H	-5.122397	2.584224	-0.319604
H	-6.696257	1.809713	0.250615
H	-7.521458	-0.377901	0.224386
H	1.695671	-1.605482	1.650347
H	4.002628	-1.103615	2.259607
H	3.864026	0.534673	1.655323
H	2.236838	-0.784669	3.883884
H	0.624627	-0.227075	3.405870
H	1.996924	0.893340	3.391440
H	3.607234	-0.286699	-0.653466
H	3.469930	-1.942353	-0.096555
H	5.790689	-1.946220	0.662908
H	6.826187	-1.934014	-1.606032
H	5.202521	-2.623495	-1.645242
H	5.472416	-0.979470	-2.231283
H	6.078645	0.311732	1.362842
H	8.348773	0.701618	0.639832
H	8.100406	-1.049650	0.711808
H	8.118229	-0.215650	-0.851220
H	6.469488	2.204110	-0.196196
H	4.819577	1.594202	-0.340325
H	6.027087	1.189122	-1.572311

ωB97XD energy = -1358.20997778 a.u.

Swinhoeisterol F (**10**), Conf. J

C	-5.206513	-2.098570	-0.059649
C	-5.644228	-0.879373	0.753961
C	-4.951562	0.374877	0.243892
C	-3.434962	0.264455	0.202581
C	-2.942703	-0.993936	-0.577700
C	-3.687017	-2.236884	-0.042642
C	-2.742379	1.558560	-0.256947
C	-1.277860	1.351805	-0.719871
C	-0.626367	0.143542	-0.073524
C	-1.444473	-1.155750	-0.221533
C	0.843057	-0.154436	-0.457932
C	0.862893	-1.695716	-0.597519
C	-0.548375	-2.032762	-1.106151
C	-0.526202	2.669873	-0.576620
C	0.345882	2.930152	0.632727
C	1.534313	1.979467	0.867143
C	1.157808	0.486175	0.951388
C	-3.212560	-0.861217	-2.085511
C	1.394351	0.471107	-1.734804
C	-5.650772	1.462105	-0.092349
O	-7.056079	-0.773376	0.812262
O	-0.697830	3.542256	-1.415971
C	2.006626	-0.267881	1.992626
C	3.523178	-0.094057	1.825062
C	1.589319	0.118076	3.417714
C	4.086210	-0.515056	0.468406
C	5.622830	-0.512482	0.410022
C	6.211577	-1.738219	1.116433
C	6.169003	-0.374756	-1.032477
C	5.610661	-1.428894	-1.994841
C	5.947244	1.031395	-1.599098
H	-3.126932	0.085570	1.245198
H	-1.290433	1.157798	-1.796466
H	-1.418511	-1.593783	0.783910
O	-0.251158	0.379623	1.304621
H	-5.580574	-1.994964	-1.087135
H	-5.672038	-2.993040	0.367062
H	-5.333120	-1.032245	1.796055
H	-3.383197	-3.118535	-0.618952
H	-3.372780	-2.416182	0.994301
H	-2.783766	2.284643	0.562460
H	-3.277692	2.006905	-1.097600
H	1.653764	-2.032624	-1.274418
H	1.020266	-2.194970	0.362077
H	-0.782454	-3.098453	-1.024318
H	-0.647782	-1.753030	-2.161846
H	-0.312765	2.871822	1.507237
H	0.712772	3.956314	0.549639
H	1.988547	2.296358	1.809806
H	2.301422	2.126545	0.099794
H	-3.046346	-1.817395	-2.593030
H	-2.575375	-0.119801	-2.572510
H	-4.249009	-0.564113	-2.273511
H	1.311466	1.559022	-1.779497
H	0.853378	0.074500	-2.601115
H	2.449375	0.218645	-1.872472
H	-5.182742	2.371437	-0.452904
H	-6.731053	1.482846	0.014151
H	-7.397974	-0.755689	-0.090189
H	1.782650	-1.334039	1.879780
H	4.001611	-0.678664	2.620342
H	3.798351	0.952336	2.016410
H	1.809109	1.168516	3.640644
H	2.139927	-0.491297	4.142327
H	0.521187	-0.047017	3.578040
H	3.697049	0.167675	-0.291393
H	3.712220	-1.514438	0.207774
H	5.971193	0.377239	0.956187
H	5.909766	-2.668042	0.620411
H	7.306849	-1.701309	1.121894
H	5.880941	-1.801440	2.158345
H	7.255257	-0.529238	-0.966414
H	6.110286	-1.363899	-2.967529
H	5.751979	-2.447463	-1.618490
H	4.538260	-1.280046	-2.167931
H	4.881929	1.245851	-1.747513
H	6.438452	1.141330	-2.572395
H	6.353214	1.798731	-0.930222

ωB97XD energy = -1358.20957274 a.u.

Swinhoeisterol F (**10**), Conf. K

C	-4.991205	-2.302993	0.536423
C	-5.515095	-0.970640	1.074614
C	-4.949788	0.186455	0.265085
C	-3.430900	0.193376	0.182826
C	-2.845022	-1.166447	-0.309427
C	-3.465752	-2.310278	0.521721
C	-2.867780	1.396113	-0.592326
C	-1.395516	1.214353	-1.042329
C	-0.632128	0.243897	-0.160724
C	-1.332285	-1.118755	0.015820
C	0.854569	-0.012112	-0.509104
C	1.005482	-1.539110	-0.301473
C	-0.376035	-2.091733	-0.685680
C	-0.762684	2.590022	-1.219475
C	0.104733	3.185100	-0.130065
C	1.376807	2.411634	0.263954
C	1.126415	0.954259	0.706123
C	-3.153041	-1.406069	-1.796823
C	1.358905	0.363044	-1.898828
C	-5.752311	1.107691	-0.274728
O	-6.928296	-0.968581	1.173533
O	-1.026255	3.235700	-2.223798
C	2.038826	0.533421	1.872556
C	3.543095	0.662783	1.584023
C	1.674508	1.307807	3.147245
C	4.098961	-0.364785	0.597249
C	5.580451	-0.166625	0.244028
C	6.481261	-0.273126	1.479540
C	6.058728	-1.106901	-0.890523
C	5.964474	-2.595554	-0.539731

C	5.342805	-0.832305	-2.217440
H	-3.082023	0.288692	1.223635
H	-1.401659	0.781872	-2.047204
H	-1.247826	-1.314603	1.091996
O	-0.265743	0.822513	1.115209
H	-5.396476	-2.465012	-0.471453
H	-5.366855	-3.111688	1.171852
H	-5.162252	-0.857116	2.108302
H	-3.096203	-3.272028	0.147119
H	-3.114952	-2.220823	1.558491
H	-2.965737	2.289536	0.034069
H	-3.453491	1.582749	-1.496116
H	1.816573	-1.954704	-0.906876
H	1.214085	-1.793630	0.741101
H	-0.519677	-3.130831	-0.374881
H	-0.509842	-2.050297	-1.773761
H	-0.529373	3.267725	0.760342
H	0.376141	4.194221	-0.450382
H	1.825839	2.972648	1.087734
H	2.108281	2.436038	-0.549410
H	-2.920505	-2.439085	-2.076635
H	-2.585160	-0.752411	-2.462790
H	-4.213314	-1.236357	-2.009270
H	1.102645	1.375847	-2.216248
H	0.939259	-0.322197	-2.643650
H	2.448583	0.275826	-1.955002
H	-5.378579	1.947868	-0.849532
H	-6.826998	1.061653	-0.126919
H	-7.296079	-1.172043	0.304567
H	1.826099	-0.521696	2.077738
H	4.064265	0.556428	2.542697
H	3.768243	1.678754	1.230328
H	2.173800	0.856736	4.011053
H	0.597103	1.288153	3.332114
H	1.994397	2.354816	3.098404
H	3.513666	-0.330476	-0.324112
H	3.952234	-1.368348	1.018874
H	5.686316	0.856302	-0.149307
H	7.539921	-0.238208	1.197589
H	6.303047	0.548675	2.179690
H	6.310575	-1.210126	2.023000
H	7.122064	-0.874294	-1.044063
H	6.399037	-3.203952	-1.340671
H	6.500540	-2.836391	0.383953
H	4.922302	-2.913218	-0.417559
H	5.846684	-1.347982	-3.042547
H	5.328655	0.238758	-2.451335
H	4.306138	-1.187966	-2.199681

ωB97XD energy = -1358.20943355 a.u.

Swinhoeisterol F (**10**), Conf. L

C	4.923285	-2.693509	0.049282
C	5.409955	-1.752618	-1.053735
C	5.058411	-0.311394	-0.717047
C	3.581876	-0.091113	-0.423465
C	3.033847	-1.063951	0.666086
C	3.428230	-2.508367	0.289134
C	3.231793	1.378875	-0.137870
C	1.861956	1.570444	0.561750
C	0.900964	0.426796	0.296201
C	1.490942	-0.966608	0.592910
C	-0.496883	0.515749	0.957708
C	-0.756388	-0.936747	1.428694
C	0.648559	-1.450912	1.781354
C	1.337554	2.967080	0.244890
C	0.301985	3.172182	-0.840033
C	-1.056470	2.468430	-0.661051
C	-0.959510	0.939290	-0.480698
C	3.608479	-0.732708	2.053157
C	-0.732072	1.468781	2.123732
C	5.988697	0.646835	-0.727475
O	6.781590	-1.958015	-1.343981
O	1.820934	3.925386	0.830499
C	-2.132216	0.186132	-1.114652
C	-3.437153	0.481812	-0.362593
C	-2.246603	0.429389	-2.623796
C	-4.598617	-0.415855	-0.800027
C	-5.799938	-0.427471	0.160837
C	-6.330875	0.991962	0.391118
C	-6.917956	-1.381283	-0.329415
C	-6.404043	-2.801582	-0.596417
C	-8.092615	-1.449229	0.654598
H	3.052740	-0.376544	-1.346925
H	2.035588	1.581054	1.642392
H	1.189488	-1.566552	-0.274794
O	0.309586	0.484753	-1.028779
H	5.494694	-2.495245	0.966124
H	5.134194	-3.726056	-0.247921
H	4.886282	-2.013011	-1.983356
H	3.083411	-3.195363	1.070570
H	2.897912	-2.787839	-0.631135
H	3.255953	1.930021	-1.084219
H	3.984581	1.835611	0.509455
H	-1.452293	-0.971269	2.272314
H	-1.173412	-1.564236	0.636671
H	0.684002	-2.536231	1.916553
H	0.995162	-0.988967	2.714152
H	0.751666	2.806057	-1.770067
H	0.144624	4.249318	-0.936906
H	-1.633894	2.697032	-1.560380
H	-1.608646	2.911068	0.173298
H	3.383467	-1.533703	2.765218
H	3.209751	0.194459	2.471416
H	4.696443	-0.621477	2.010903
H	-0.366726	2.485051	1.960469
H	-0.239026	1.090466	3.025828
H	-1.804301	1.538247	2.337878
H	5.765213	1.683596	-0.501175
H	7.016493	0.420843	-0.994548
H	7.285442	-1.853993	-0.527340
H	-1.911044	-0.879533	-0.990866
H	-3.697648	1.538937	-0.497584

H	-3.267630	0.335005	0.713362
H	-2.876504	-0.336238	-3.086812
H	-1.263499	0.380792	-3.102832
H	-2.695149	1.401630	-2.855898
H	-4.206597	-1.434679	-0.897540
H	-4.946711	-0.119228	-1.799584
H	-5.443058	-0.816510	1.128861
H	-7.125235	1.015195	1.141678
H	-5.543086	1.663113	0.745523
H	-6.732938	1.411832	-0.540280
H	-7.298548	-0.976008	-1.279893
H	-7.235480	-3.469564	-0.846404
H	-5.695153	-2.843763	-1.427728
H	-5.906110	-3.209994	0.292659
H	-8.837279	-2.176287	0.313046
H	-8.604811	-0.489752	0.766559
H	-7.750375	-1.766732	1.648292

ωB97XD energy = -1358.20916217 a.u.

Swinhoeisterol F (**10**), Conf. M

C	4.530892	-2.767043	-0.089552
C	5.087556	-1.785348	-1.121854
C	4.790250	-0.351802	-0.708538
C	3.317483	-0.084139	-0.436109
C	2.701355	-1.091403	0.582971
C	3.039990	-2.528102	0.129364
C	3.021459	1.380688	-0.074709
C	1.653326	1.590286	0.622361
C	0.639977	0.516803	0.269073
C	1.166448	-0.918567	0.480717
C	-0.763636	0.626389	0.916476
C	-1.093603	-0.845443	1.260739
C	0.274478	-1.447665	1.611816
C	1.211188	3.030286	0.396306
C	0.218149	3.356709	-0.695383
C	-1.179294	2.725992	-0.560485
C	-1.184470	1.181275	-0.494671
C	3.257291	-0.870251	1.999577
C	-0.953320	1.494975	2.154298
C	5.761907	0.561792	-0.635293
O	6.456422	-2.034767	-1.390054
O	1.720331	3.918842	1.064290
C	-2.375819	0.574721	-1.259222
C	-3.747736	1.067338	-0.760086
C	-2.237257	0.829392	-2.766906
C	-4.287491	0.416561	0.517918
C	-4.600025	-1.089552	0.445420
C	-5.138724	-1.555167	1.802465
C	-5.509829	-1.475872	-0.745358
C	-5.727345	-2.990185	-0.829522
C	-6.853114	-0.740373	-0.756881
H	2.800534	-0.295015	-1.386091
H	1.811730	1.515434	1.702282
H	0.863218	-1.443463	-0.433587
O	0.068000	0.686590	-1.052547
H	5.087957	-2.651748	0.849869
H	4.702302	-3.788138	-0.445696
H	4.578563	-1.967749	-2.077768
H	2.647487	-3.242944	0.861926
H	2.519914	-2.729296	-0.816866
H	3.078102	1.981476	-0.989010
H	3.782753	1.771827	0.604699
H	-1.825962	-0.924209	2.069959
H	-1.498076	-1.383210	0.399861
H	0.255649	-2.540394	1.666846
H	0.617120	-1.072247	2.584353
H	0.658218	3.013811	-1.638381
H	0.122923	4.444792	-0.734832
H	-1.743800	3.058137	-1.435658
H	-1.698226	3.141428	0.308474
H	2.977375	-1.699820	2.657545
H	2.894214	0.049325	2.464082
H	4.350002	-0.811357	1.988342
H	-0.521500	2.494929	2.074707
H	-0.490754	1.015295	3.023648
H	-2.018564	1.618733	2.375631
H	5.577100	1.592322	-0.352950
H	6.785942	0.306324	-0.890176
H	6.940059	-2.009801	-0.555231
H	-2.321167	-0.510260	-1.125254
H	-4.473116	0.915795	-1.567998
H	-3.711416	2.153039	-0.610704
H	-1.235693	0.576531	-3.125650
H	-2.441456	1.873462	-3.029678
H	-2.957746	0.211854	-3.313504
H	-5.200482	0.950410	0.813741
H	-3.577774	0.571973	1.338306
H	-3.653855	-1.621408	0.281110
H	-5.273953	-2.640102	1.843855
H	-4.440106	-1.282083	2.601603
H	-6.101467	-1.086041	2.036952
H	-4.969652	-1.187545	-1.657313
H	-6.198815	-3.259730	-1.780985
H	-4.777146	-3.532857	-0.760093
H	-6.381309	-3.351898	-0.027939
H	-7.447080	-1.041401	-1.627229
H	-6.727228	0.346078	-0.806364
H	-7.443376	-0.969353	0.138055

ωB97XD energy = -1358.20910034 a.u.

Swinhoeisterol F (**10**), Conf. N

C	-5.219507	-2.168903	0.162837
C	-5.677345	-0.886491	0.859494
C	-5.022263	0.326951	0.217456
C	-3.504117	0.248639	0.164010
C	-2.988588	-1.069679	-0.492087
C	-3.697045	-2.269994	0.173027
C	-2.850324	1.505075	-0.435436
C	-1.380440	1.291323	-0.879228
C	-0.697832	0.167368	-0.121019
C	-1.483981	-1.159259	-0.138945
C	0.778156	-0.131651	-0.480241

C	0.831803	-1.678577	-0.473656
C	-0.571775	-2.090958	-0.947455
C	-0.660364	2.634603	-0.864659
C	0.201055	3.028789	0.315361
C	1.417006	2.137237	0.629274
C	1.079816	0.649620	0.858891
C	-3.276056	-1.094953	-2.002170
C	1.320265	0.378973	-1.810886
C	-5.752741	1.358928	-0.213093
O	-7.090408	-0.808220	0.929473
O	-0.848049	3.416922	-1.785406
C	1.949821	0.021495	1.963586
C	3.461880	0.218100	1.780726
C	1.519415	0.527958	3.346282
C	4.041182	-0.282899	0.457672
C	5.578010	-0.349767	0.444879
C	6.083717	-1.584272	1.198954
C	6.137811	-0.240274	-0.993928
C	7.666055	-0.148794	-1.015233
C	5.655703	-1.358491	-1.924269
H	-3.177883	0.183934	1.214393
H	-1.386873	0.993108	-1.931914
H	-1.437928	-1.500497	0.902846
O	-0.325778	0.543395	1.226739
H	-5.607243	-2.175711	-0.864616
H	-5.658995	-3.028103	0.680076
H	-5.348180	-0.929584	1.906402
H	-3.378028	-3.196883	-0.317735
H	-3.367188	-2.338527	1.218277
H	-2.911371	2.312074	0.302863
H	-3.401286	1.843648	-1.316583
H	1.630561	-2.060570	-1.116619
H	0.996728	-2.083918	0.528134
H	-0.781952	-3.150448	-0.772769
H	-0.679707	-1.904653	-2.022842
H	-0.454921	3.027856	1.193645
H	0.537379	4.054037	0.141131
H	1.868040	2.554810	1.533375
H	2.174753	2.229262	-0.155445
H	-3.080044	-2.089964	-2.415443
H	-2.671091	-0.383208	-2.568396
H	-4.324371	-0.854495	-2.204792
H	1.202146	1.452811	-1.968874
H	0.802576	-0.122825	-2.636181
H	2.384736	0.149579	-1.912123
H	-5.312672	2.238671	-0.669496
H	-6.831736	1.363954	-0.091970
H	-7.445238	-0.881702	0.034907
H	1.755780	-1.056544	1.952317
H	3.950895	-0.301236	2.614370
H	3.715220	1.280100	1.902642
H	2.079162	0.000313	4.126090
H	0.454337	0.355990	3.518877
H	1.715693	1.598879	3.473677
H	3.712873	0.386121	-0.345145
H	3.628005	-1.273896	0.227079
H	5.946799	0.537973	0.981304
H	7.172250	-1.576031	1.312552
H	5.655042	-1.633118	2.205390
H	5.804927	-2.509215	0.681200
H	5.748782	0.706766	-1.395890
H	8.025426	0.114502	-2.016261
H	8.030393	0.613944	-0.317115
H	8.131033	-1.102985	-0.741971
H	6.037844	-1.202171	-2.939177
H	4.563092	-1.398777	-1.987184
H	6.010200	-2.340637	-1.590319

ωB97XD energy = -1358.20900976 a.u.

Swinhoeisterol F (**10**), Conf. O

C	-4.439489	-2.765240	0.109121
C	-5.007309	-1.775639	1.127772
C	-4.726310	-0.344555	0.694942
C	-3.256848	-0.063430	0.417621
C	-2.629607	-1.078455	-0.587239
C	-2.951401	-2.512298	-0.113027
C	-2.978685	1.399938	0.035116
C	-1.614504	1.615025	-0.667637
C	-0.589002	0.558498	-0.301270
C	-1.096486	-0.886607	-0.488751
C	0.809526	0.676270	-0.957370
C	1.162121	-0.795701	-1.284292
C	-0.200463	-1.424059	-1.613071
C	-1.185428	3.063220	-0.466874
C	-0.190734	3.420569	0.614224
C	1.212264	2.799278	0.489310
C	1.226703	1.255557	0.440070
C	-3.189103	-0.883720	-2.006446
C	1.002902	1.527398	-2.206215
C	-5.708326	0.557002	0.610920
O	-6.373432	-2.036747	1.399023
O	-1.706972	3.934692	-1.147668
C	2.446353	0.653181	1.147837
C	3.729590	1.036667	0.395772
C	2.487576	1.016180	2.635672
C	5.055348	0.458518	0.915059
C	5.286142	-1.068585	0.824173
C	4.607430	-1.839599	1.965947
C	4.946896	-1.653181	-0.565407
C	5.719554	-0.949206	-1.686564
C	5.211062	-3.161167	-0.637847
H	-2.737233	-0.254573	1.370328
H	-1.773827	1.522009	-1.745995
H	-0.786193	-1.392914	0.433581
O	-0.013710	0.757414	1.017455
H	-4.997602	-2.669570	-0.831858
H	-4.599322	-3.783035	0.479791
H	-4.496684	-1.939270	2.086202
H	-2.550586	-3.232658	-0.835646
H	-2.429288	-2.694317	0.835938
H	-3.040496	2.013072	0.940812
H	-3.745879	1.772545	-0.648063

H	1.882661	-0.870300	-2.104539
H	1.590298	-1.318855	-0.425114
H	-0.166716	-2.517249	-1.647510
H	-0.552806	-1.072146	-2.590818
H	-0.625917	3.093968	1.565242
H	-0.105783	4.510114	0.630703
H	1.772703	3.142534	1.362511
H	1.730546	3.208965	-0.382614
H	-4.282146	-0.831220	-1.994217
H	-2.905519	-1.722243	-2.651409
H	-2.832184	0.030242	-2.486624
H	0.572624	2.529294	-2.142851
H	0.546911	1.037266	-3.073336
H	2.072068	1.642971	-2.416244
H	-5.535612	1.585646	0.314504
H	-6.729007	0.293269	0.870836
H	-6.856789	-2.028630	0.563691
H	2.318146	-0.430144	1.091301
H	3.828399	2.130349	0.419399
H	3.604751	0.770231	-0.661285
H	3.163661	0.348694	3.176314
H	1.495911	0.916503	3.088498
H	2.840153	2.039877	2.804336
H	5.200530	0.764382	1.958743
H	5.855523	0.956106	0.354106
H	6.369804	-1.209032	0.962956
H	3.526409	-1.938032	1.820058
H	5.017641	-2.849285	2.062801
H	4.771690	-1.334663	2.923704
H	3.873759	-1.497337	-0.740500
H	5.479912	-1.392996	-2.659157
H	5.495412	0.119691	-1.749752
H	6.801361	-1.053281	-1.532397
H	5.030725	-3.534966	-1.651810
H	4.567694	-3.731446	0.038293
H	6.254560	-3.387598	-0.382655

ωB97XD energy = -1358.20894598 a.u.

Swinhoeisterol F (**10**), Conf. P

C	4.504823	-2.726992	-0.232640
C	5.060770	-1.691844	-1.212283
C	4.766105	-0.282689	-0.720455
C	3.293356	-0.028796	-0.434486
C	2.678958	-1.088018	0.531858
C	3.014658	-2.498828	0.001175
C	2.996203	1.415395	0.002217
C	1.630946	1.584808	0.715420
C	0.615866	0.532577	0.308168
C	1.143670	-0.910885	0.443941
C	-0.784385	0.609937	0.968279
C	-1.112461	-0.879087	1.238320
C	0.257974	-1.497256	1.550809
C	1.184983	3.034028	0.577120
C	0.197511	3.420975	-0.498801
C	-1.200363	2.783792	-0.403240
C	-1.208617	1.238128	-0.407419
C	3.240220	-0.943418	1.956509
C	-0.988148	1.411245	2.247880
C	5.739437	0.623380	-0.594787
O	6.428850	-1.928273	-1.495879
O	1.684197	3.881592	1.303233
C	-2.431492	0.672695	-1.142718
C	-3.736811	1.172063	-0.494822
C	-2.371131	0.974456	-2.644540
C	-5.026108	0.472560	-0.972677
C	-5.613401	-0.574188	-0.008634
C	-6.952650	-1.083525	-0.551174
C	-4.622771	-1.710086	0.332080
C	-5.094762	-2.556598	1.517647
C	-4.278010	-2.608478	-0.859121
H	2.776549	-0.191104	-1.394000
H	1.793328	1.447227	1.788471
H	0.836670	-1.389503	-0.494110
O	0.034501	0.767242	-1.001799
H	5.063265	-2.663393	0.710843
H	4.674181	-3.727439	-0.644118
H	4.549886	-1.822037	-2.175652
H	2.622882	-3.250433	0.696361
H	2.492595	-2.650217	-0.953099
H	3.046975	2.063262	-0.879842
H	3.759343	1.773533	0.697579
H	-1.836903	-0.998638	2.049716
H	-1.521657	-1.376925	0.355074
H	0.239733	-2.591429	1.549869
H	0.605431	-1.171820	2.539262
H	0.640716	3.129717	-1.457522
H	0.102432	4.509612	-0.478861
H	-1.756106	3.153091	-1.268953
H	-1.729509	3.160489	0.477223
H	2.975277	-1.814543	2.565360
H	2.867624	-0.058351	2.477166
H	4.332039	-0.869349	1.943079
H	-0.522157	0.898117	3.096184
H	-2.058232	1.502524	2.464661
H	-0.574136	2.421487	2.218680
H	5.557098	1.635973	-0.252122
H	6.763256	0.380601	-0.862621
H	6.914616	-1.943680	-0.662054
H	-2.387349	-0.415422	-1.040671
H	-3.824633	2.247350	-0.689602
H	-3.656286	1.070436	0.596540
H	-2.546503	2.034116	-2.861048
H	-3.131285	0.403938	-3.186769
H	-1.396282	0.697702	-3.056455
H	-4.864754	0.005053	-1.951758
H	-5.800596	1.230959	-1.137399
H	-5.816693	-0.053207	0.939587
H	-7.411529	-1.820422	0.115837
H	-7.663091	-0.257110	-0.664584
H	-6.832561	-1.550440	-1.535713
H	-3.697992	-1.219726	0.652058
H	-4.311132	-3.257280	1.827514

H	-5.342645	-1.928880	2.381620
H	-5.982248	-3.149922	1.268429
H	-3.475561	-3.305337	-0.591680
H	-3.939901	-2.035789	-1.729071
H	-5.140759	-3.209396	-1.169588

ωB97XD energy = -1358.20883026 a.u.

Swinhoeisterol F (**10**), Conf. Q

C	-4.980333	-2.466811	0.089695
C	-5.436018	-1.375992	1.053027
C	-4.990438	-0.016807	0.540436
C	-3.485071	0.085052	0.358133
C	-2.939262	-1.040301	-0.578602
C	-3.464497	-2.406917	-0.082838
C	-3.009235	1.487095	-0.057303
C	-1.586588	1.505039	-0.672744
C	-0.731989	0.339511	-0.211519
C	-1.401337	-1.036592	-0.399490
C	0.709671	0.260343	-0.772200
C	0.893738	-1.250143	-1.061106
C	-0.518092	-1.709607	-1.458916
C	-0.979646	2.890262	-0.482347
C	-0.006809	3.156027	0.646958
C	1.301248	2.343760	0.660565
C	1.104476	0.814046	0.641698
C	-3.396035	-0.828499	-2.030957
C	1.090753	1.054378	-2.015982
C	-5.870207	0.954159	0.282588
O	-6.835765	-1.503527	1.229008
O	-1.345874	3.800794	-1.211543
C	2.182958	0.070846	1.435285
C	3.539411	0.214590	0.731304
C	2.213694	0.480814	2.912327
C	4.617941	-0.723507	1.271288
C	6.020804	-0.570553	0.656195
C	6.999513	-1.473591	1.418163
C	6.126866	-0.834445	-0.872663
C	5.314430	-2.047795	-1.336186
C	5.814245	0.395112	-1.734961
H	-3.042670	-0.128556	1.343979
H	-1.689933	1.396582	-1.756765
H	-1.206155	-1.551410	0.549544
O	-0.227549	0.507783	1.140594
H	-5.499403	-2.335508	-0.866961
H	-5.274964	-3.442006	0.490874
H	-4.930790	-1.546624	2.018257
H	-3.134442	-3.191863	-0.773011
H	-3.001884	-2.627358	0.888434
H	-3.053976	2.142979	0.819216
H	-3.681142	1.917842	-0.803388
H	1.637579	-1.424611	-1.844655
H	1.212792	-1.808830	-0.177320
H	-0.624387	-2.798568	-1.473097
H	-0.764605	-1.339499	-2.461863
H	-0.546266	2.946134	1.577890
H	0.227463	4.223269	0.623003
H	1.825021	2.632603	1.575306
H	1.948765	2.647675	-0.167529
H	-3.157083	-1.706280	-2.640575
H	-2.928480	0.036643	-2.506891
H	-4.478253	-0.674981	-2.082721
H	0.606442	0.626317	-2.900422
H	2.173557	0.999842	-2.175373
H	0.820528	2.112001	-1.981837
H	-5.574374	1.932600	-0.079960
H	-6.934933	0.791840	0.420851
H	-7.115921	-0.907798	1.933357
H	1.905247	-0.988579	1.412649
H	3.887682	1.254333	0.807996
H	3.386262	0.014714	-0.333783
H	2.724914	1.437425	3.066999
H	2.738538	-0.268993	3.511471
H	1.198579	0.566867	3.313671
H	4.280052	-1.762934	1.153771
H	4.721571	-0.560423	2.351207
H	6.338235	0.469120	0.824314
H	8.031756	-1.304335	1.092747
H	6.954892	-1.285966	2.496768
H	6.769204	-2.534147	1.257862
H	7.184200	-1.069201	-1.059049
H	5.537915	-2.280534	-2.383232
H	5.540823	-2.941304	-0.744176
H	4.236262	-1.863277	-1.265228
H	6.049072	0.194045	-2.786678
H	6.410593	1.259594	-1.421663
H	4.760075	0.683905	-1.687572

ωB97XD energy = -1358.20876856 a.u.

Lobatolide A (**11**), Conf. A

C	1.637593	-2.040734	-0.760667
C	2.644251	-1.545789	-1.744379
C	2.595649	0.005665	-1.750361
C	2.484004	0.638134	-0.354668
C	1.067163	1.142653	0.061399
C	0.408171	0.458193	1.291841
C	-0.734934	-0.532420	0.966266
C	-0.388424	-1.828599	0.226204
O	0.410062	-1.506688	-0.925566
C	0.531801	-2.725493	1.076651
C	1.768144	-2.819062	0.340243
O	3.349467	1.806155	-0.337621
C	2.713176	2.931789	0.009344
C	1.288913	2.618358	0.286103
C	-1.621514	-2.602819	-0.213969
C	4.044041	-2.082233	-1.449607
O	0.199822	-3.217449	2.146126
O	3.279588	4.000915	0.067075
C	0.417383	3.553655	0.656499
O	1.401486	-0.140345	2.095257
O	-1.695855	0.173982	0.171608
C	-2.707121	0.811358	0.796237

O	-2.813137	0.857849	2.003473
C	-3.651331	1.433831	-0.196221
C	-4.238116	0.418597	-1.195016
C	-4.988663	-0.700117	-0.469727
C	-5.148318	1.135704	-2.192067
H	2.333246	-1.887532	-2.739262
H	3.524938	0.359402	-2.205158
H	1.774941	0.355641	-2.382696
H	2.891923	-0.028021	0.407924
H	0.383640	1.021857	-0.782357
H	-0.088266	1.253778	1.860018
H	-1.202388	-0.824033	1.912845
H	2.662916	-3.325343	0.669650
H	-1.318994	-3.523155	-0.720864
H	-2.215989	-2.869657	0.664009
H	-2.230766	-2.003975	-0.893190
H	4.418207	-1.715770	-0.487466
H	4.737809	-1.753018	-2.227536
H	4.056507	-3.176063	-1.428105
H	0.737553	4.585213	0.771780
H	-0.625943	3.323419	0.856223
H	1.044454	-0.301833	2.976615
H	-4.449722	1.920470	0.370857
H	-3.099226	2.208921	-0.741310
H	-3.402013	-0.025657	-1.748419
H	-5.379373	-1.430458	-1.186063
H	-4.343970	-1.240356	0.232690
H	-5.835817	-0.295478	0.096881
H	-4.609417	1.918715	-2.736750
H	-5.998145	1.604456	-1.681118
H	-5.548777	0.429089	-2.926645

ωB97XD energy = -1304.08193562 a.u.

Lobatolide A (**11**), Conf. B

C	-2.535592	-1.166438	0.843882
C	-3.197363	-0.117145	1.671686
C	-2.349015	1.177986	1.562161
C	-1.862105	1.485996	0.137116
C	-0.357637	1.193210	-0.160491
C	-0.049274	0.109677	-1.231071
C	0.382837	-1.275462	-0.694485
C	-0.634438	-2.123963	0.076141
O	-1.221889	-1.313638	1.108379
C	-1.829630	-2.516125	-0.813340
C	-2.980536	-1.887627	-0.212976
O	-2.050573	2.909737	-0.086766
C	-0.933199	3.536718	-0.473607
C	0.171767	2.549446	-0.561912
C	-0.008193	-3.359826	0.703327
C	-4.652715	0.104272	1.264321
O	-1.732993	-3.219616	-1.809345
O	-0.906573	4.726293	-0.700497
C	1.399011	2.901840	-0.937734
O	-1.136760	0.012836	-2.123483
O	1.505414	-1.030029	0.159128
C	2.724671	-1.487462	-0.201265

O	2.927253	-2.105553	-1.223616
C	3.757603	-1.108602	0.824015
C	4.032417	0.409463	0.859724
C	4.954322	0.741269	2.033084
C	4.627748	0.896302	-0.462588
H	-3.163400	-0.445787	2.717484
H	-2.978930	2.010050	1.888252
H	-1.497154	1.130264	2.245848
H	-2.497890	0.997233	-0.603444
H	0.137155	0.886086	0.764078
H	0.840685	0.459301	-1.770713
H	0.706393	-1.878235	-1.549622
H	-3.985887	-1.902208	-0.606307
H	0.441708	-3.980218	-0.076085
H	0.758590	-3.075968	1.427048
H	-0.776630	-3.945057	1.215545
H	-4.727230	0.500636	0.246056
H	-5.119794	0.824736	1.941031
H	-5.226454	-0.825970	1.313626
H	1.616438	3.936048	-1.188927
H	2.210170	2.182879	-1.005798
H	-0.864901	-0.474556	-2.910409
H	3.397095	-1.437866	1.804411
H	4.676850	-1.652098	0.589295
H	3.074820	0.919852	1.024105
H	4.514708	0.427991	2.985933
H	5.922278	0.237254	1.924271
H	5.141723	1.818702	2.085853
H	4.789802	1.978969	-0.440230
H	3.979640	0.671216	-1.317408
H	5.595099	0.415332	-0.650210

ωB97XD energy = -1304.08181714 a.u.

Lobatolide A (**11**), Conf. C

C	-2.194545	-1.390938	0.994477
C	-2.784671	-0.409296	1.949109
C	-2.034860	0.934445	1.753390
C	-1.825757	1.327418	0.281739
C	-0.378647	1.179734	-0.286463
C	-0.155847	0.115148	-1.393044
C	0.463604	-1.234896	-0.951146
C	-0.372250	-2.182960	-0.086470
O	-0.848651	-1.447658	1.051962
C	-1.658767	-2.636890	-0.802462
C	-2.745378	-2.113382	-0.010179
O	-2.164635	2.736172	0.164707
C	-1.213841	3.459953	-0.437645
C	-0.077400	2.572771	-0.789441
C	0.419185	-3.392408	0.389132
C	-4.295167	-0.265686	1.773058
O	-1.667056	-3.308962	-1.824381
O	-1.336109	4.649557	-0.630975
C	0.987509	3.029739	-1.444660
O	-1.359417	-0.075991	-2.104685
O	1.659402	-0.962621	-0.209111
C	2.811937	-0.764943	-0.874949

O	2.874916	-0.719341	-2.086004
C	3.988601	-0.671420	0.061039
C	3.748158	0.098780	1.368397
C	4.981848	-0.013272	2.265119
C	3.402405	1.562753	1.092203
H	-2.573066	-0.759632	2.966470
H	-2.633539	1.716432	2.227989
H	-1.071814	0.905609	2.269811
H	-2.535185	0.810247	-0.367575
H	0.306136	0.947756	0.535062
H	0.602339	0.531238	-2.066293
H	0.725731	-1.783205	-1.863301
H	-3.796014	-2.194119	-0.245269
H	0.777613	-3.958108	-0.474968
H	1.272180	-3.080514	0.994547
H	-0.224191	-4.042119	0.988645
H	-4.545209	0.141540	0.787657
H	-4.690886	0.417774	2.529152
H	-4.804342	-1.227271	1.886799
H	1.042644	4.076688	-1.728937
H	1.822005	2.390305	-1.718591
H	-1.168633	-0.493056	-2.953237
H	4.266333	-1.709280	0.289203
H	4.817195	-0.231797	-0.502116
H	2.901744	-0.367077	1.885972
H	5.216294	-1.058953	2.492405
H	5.859937	0.432543	1.782160
H	4.821623	0.508784	3.214177
H	3.217155	2.102900	2.026541
H	2.504144	1.659865	0.474037
H	4.225504	2.066293	0.570328

ωB97XD energy = -1304.08172550 a.u.

Lobatolide A (**11**), Conf. D

C	1.912091	-1.965695	-0.722768
C	2.940851	-1.403546	-1.645641
C	2.760940	0.137420	-1.685979
C	2.517233	0.777435	-0.310094
C	1.044586	1.182429	0.010754
C	0.357810	0.457784	1.202290
C	-0.680081	-0.624477	0.819024
C	-0.185990	-1.896788	0.124889
O	0.662065	-1.526479	-0.975434
C	0.740077	-2.711790	1.048351
C	2.026235	-2.720831	0.395972
O	3.294521	2.004496	-0.257286
C	2.562377	3.082432	0.050553
C	1.148950	2.670763	0.239374
C	-1.321429	-2.769079	-0.388038
C	4.357600	-1.810447	-1.243415
O	0.375713	-3.214753	2.102030
O	3.049252	4.187743	0.141868
C	0.192467	3.543607	0.547338
O	1.338479	-0.052911	2.078871
O	-1.643003	-0.006889	-0.046771
C	-2.703878	0.605784	0.515610

O	-2.866700	0.676486	1.715891
C	-3.646715	1.161112	-0.516489
C	-5.021720	0.465984	-0.464607
C	-5.968995	1.122372	-1.468975
C	-4.893389	-1.036275	-0.725714
H	2.731241	-1.786050	-2.651736
H	3.681400	0.562911	-2.095164
H	1.951204	0.406110	-2.369864
H	2.921793	0.151123	0.487413
H	0.426940	1.011937	-0.874149
H	-0.236929	1.216523	1.724503
H	-1.182158	-0.942622	1.738964
H	2.931127	-3.155653	0.792683
H	-1.960934	-3.065939	0.447558
H	-1.918452	-2.229393	-1.125066
H	-0.912429	-3.671406	-0.850725
H	4.630191	-1.391950	-0.268815
H	5.072607	-1.438485	-1.982054
H	4.463801	-2.898074	-1.191519
H	0.429595	4.595559	0.677291
H	-0.842392	3.238777	0.680901
H	0.935684	-0.236730	2.935855
H	-3.768401	2.228766	-0.304990
H	-3.206683	1.053287	-1.512208
H	-5.429022	0.610413	0.543377
H	-6.083377	2.193658	-1.271478
H	-5.594604	1.005010	-2.493268
H	-6.961927	0.663788	-1.420290
H	-4.442504	-1.224163	-1.707802
H	-5.876748	-1.517116	-0.709340
H	-4.275743	-1.530983	0.031959

ωB97XD energy = -1304.08159898 a.u.

Lobatolide A (**11**), Conf. E

C	2.583889	-1.384464	-0.748157
C	3.402902	-0.441230	-1.563654
C	2.699288	0.942638	-1.548665
C	2.152968	1.351950	-0.172154
C	0.612583	1.200328	0.025616
C	0.141695	0.214251	1.131631
C	-0.403741	-1.142993	0.630070
C	0.551103	-2.114149	-0.070285
O	1.279127	-1.405926	-1.088596
C	1.647167	-2.603241	0.896247
C	2.889283	-2.112608	0.352690
O	2.448840	2.761951	0.017721
C	1.363993	3.498044	0.290030
C	0.170348	2.615850	0.304050
C	-0.175070	-3.293400	-0.698046
C	4.847206	-0.362934	-1.072346
O	1.420888	-3.264286	1.900198
O	1.426925	4.692627	0.480288
C	-1.057341	3.085146	0.513885
O	1.168268	0.046781	2.084398
O	-1.475424	-0.837336	-0.271679
C	-2.741678	-0.911542	0.191636

O	-3.010905	-1.215283	1.333352
C	-3.736862	-0.605562	-0.895506
C	-5.059855	-0.028597	-0.379163
C	-4.849945	1.341786	0.269577
C	-6.070273	0.057683	-1.524092
H	3.396022	-0.803372	-2.598784
H	3.438426	1.688531	-1.852843
H	1.895019	0.964943	-2.288926
H	2.692738	0.840609	0.626722
H	0.160548	0.876208	-0.914437
H	-0.729813	0.679977	1.609924
H	-0.808901	-1.676683	1.495941
H	3.863142	-2.219499	0.805943
H	-0.726634	-3.835587	0.074933
H	-0.873239	-2.951335	-1.464479
H	0.548345	-3.975823	-1.152361
H	4.903632	0.057343	-0.062502
H	5.429240	0.281272	-1.736816
H	5.320857	-1.348937	-1.060466
H	-1.218269	4.143867	0.695062
H	-1.927397	2.433422	0.508535
H	0.802767	-0.359892	2.879218
H	-3.272723	0.067468	-1.624484
H	-3.913914	-1.557063	-1.414950
H	-5.451130	-0.715867	0.379945
H	-4.153875	1.287683	1.113220
H	-4.454971	2.059898	-0.460259
H	-5.795598	1.741457	0.650328
H	-5.716113	0.737822	-2.308573
H	-7.032675	0.436472	-1.164420
H	-6.243193	-0.922876	-1.981104

ωB97XD energy = -1304.08140712 a.u.

O	-2.800286	-1.308733	1.734478
C	-3.808412	-1.223890	-0.454518
C	-4.375426	0.199050	-0.659169
C	-3.412165	1.082382	-1.453517
C	-5.732840	0.111387	-1.358616
H	3.033738	-0.307891	-2.817217
H	2.721368	2.120926	-1.971581
H	1.285053	1.140624	-2.238856
H	2.452613	1.078924	0.547910
H	-0.253108	0.847317	-0.675821
H	-0.778556	0.313866	1.879721
H	-0.539528	-1.997546	1.608479
H	4.096313	-1.746837	0.445318
H	-0.217033	-4.077168	0.095458
H	-0.657539	-3.182643	-1.380864
H	0.939545	-3.956055	-1.245399
H	4.647685	0.718887	-0.411013
H	4.939406	1.076515	-2.119692
H	5.180140	-0.566754	-1.511392
H	-1.739557	3.747374	1.505382
H	-2.247626	1.966668	1.336274
H	1.014497	-0.513076	2.920008
H	-3.484321	-1.634378	-1.415813
H	-4.589954	-1.868954	-0.043637
H	-4.529905	0.644921	0.332083
H	-2.432657	1.160426	-0.974676
H	-3.258447	0.674944	-2.460100
H	-3.812491	2.096270	-1.556085
H	-5.632987	-0.361305	-2.343355
H	-6.157850	1.109523	-1.507491
H	-6.447019	-0.475675	-0.771886

ωB97XD energy = -1304.08114554 a.u.

Lobatolide A (**11**), Conf. F

C	2.538565	-1.081658	-0.924923
C	3.095503	0.013309	-1.770333
C	2.171297	1.248810	-1.608036
C	1.749442	1.528116	-0.156531
C	0.284296	1.149060	0.227877
C	0.092350	0.024210	1.280115
C	-0.293209	-1.375381	0.740408
C	0.734314	-2.156321	-0.083406
O	1.224674	-1.302968	-1.130714
C	1.990833	-2.487955	0.745227
C	3.074136	-1.786797	0.100049
O	1.865260	2.961555	0.056591
C	0.750478	3.514780	0.549307
C	-0.282843	2.462807	0.712391
C	0.157844	-3.423555	-0.696672
C	4.550888	0.324255	-1.428067
O	1.982826	-3.212059	1.730871
O	0.674187	4.697677	0.799120
C	-1.488232	2.733211	1.208408
O	1.233354	-0.039506	2.108684
O	-1.466318	-1.240395	-0.075948
C	-2.670378	-1.264729	0.528268

Lobatolide A (**11**), Conf. G

C	2.568470	-1.094874	-0.781719
C	3.181553	-0.014547	-1.606019
C	2.230410	1.209176	-1.540151
C	1.707790	1.517045	-0.127171
C	0.213737	1.166594	0.164603
C	-0.065991	0.047987	1.203069
C	-0.388842	-1.364707	0.657523
C	0.709036	-2.157185	-0.056218
O	1.275611	-1.328413	-1.083149
C	1.896086	-2.456595	0.879383
C	3.024235	-1.767451	0.302307
O	1.833932	2.952048	0.069796
C	0.703992	3.527854	0.495973
C	-0.352172	2.493083	0.618632
C	0.192248	-3.446562	-0.676893
C	4.602215	0.321061	-1.156220
O	1.811932	-3.151470	1.882461
O	0.632249	4.715048	0.726152
C	-1.569309	2.792465	1.066547
O	0.991102	0.012930	2.137387
O	-1.485473	-1.249559	-0.258744
C	-2.740287	-1.385604	0.219054

O	-2.983692	-1.489479	1.403432
C	-3.763660	-1.391901	-0.885628
C	-4.312384	0.008402	-1.249217
C	-5.026389	0.672219	-0.070430
C	-3.236568	0.923306	-1.837014
H	3.206180	-0.358555	-2.647047
H	2.789722	2.081579	-1.888166
H	1.391247	1.068705	-2.226466
H	2.351697	1.070276	0.633121
H	-0.276616	0.876974	-0.769814
H	-0.994675	0.334029	1.711387
H	-0.705260	-1.972270	1.512837
H	4.015309	-1.709538	0.726673
H	-0.233973	-4.083767	0.102532
H	-0.571559	-3.233002	-1.426325
H	1.017980	-3.983107	-1.152221
H	4.613192	0.732973	-0.141486
H	5.035438	1.067399	-1.827456
H	5.247096	-0.562682	-1.173930
H	-1.807997	3.814708	1.345786
H	-2.351683	2.046237	1.170039
H	0.707816	-0.465969	2.925414
H	-3.319026	-1.846344	-1.775137
H	-4.588697	-2.022398	-0.543941
H	-5.057810	-0.177125	-2.031985
H	-5.821241	0.033088	0.327768
H	-4.333158	0.886234	0.750921
H	-5.476054	1.620593	-0.382008
H	-2.493454	1.201997	-1.083405
H	-3.685729	1.849515	-2.210204
H	-2.709077	0.443007	-2.668005

ωB97XD energy = -1304.08056237 a.u.

Lobatolide A (**11**), Conf. H

C	1.894860	-2.008747	-0.593143
C	2.991068	-1.495014	-1.465568
C	2.850728	0.047641	-1.563808
C	2.522935	0.736021	-0.229756
C	1.035590	1.161084	-0.028031
C	0.266197	0.494891	1.147346
C	-0.770433	-0.578181	0.732880
C	-0.252267	-1.880732	0.116149
O	0.671047	-1.560173	-0.939526
C	0.600832	-2.676907	1.123521
C	1.925182	-2.728067	0.553932
O	3.304988	1.959648	-0.165620
C	2.560435	3.053858	0.039157
C	1.133162	2.657451	0.134359
C	-1.362590	-2.756365	-0.442731
C	4.371062	-1.916519	-0.963939
O	0.162543	-3.139968	2.167193
O	3.048173	4.159514	0.121327
C	0.158002	3.543970	0.319789
O	1.181348	-0.007778	2.096593
O	-1.659883	0.027981	-0.215832
C	-2.694827	0.755034	0.255503

O	-2.907381	0.904067	1.440959
C	-3.527585	1.327335	-0.860050
C	-4.845668	0.549709	-1.093168
C	-4.584884	-0.921446	-1.421115
C	-5.824974	0.694284	0.072435
H	2.836329	-1.905130	-2.470822
H	3.807872	0.440763	-1.917797
H	2.098010	0.312756	-2.311412
H	2.866098	0.135637	0.615095
H	0.479062	0.949497	-0.943682
H	-0.338457	1.283382	1.609775
H	-1.337846	-0.857112	1.627506
H	2.796682	-3.164054	1.018581
H	-1.884345	-2.242426	-1.251267
H	-0.939465	-3.688308	-0.827791
H	-2.075157	-2.998288	0.350241
H	4.448953	-3.002992	-0.863572
H	4.595167	-1.464602	0.008051
H	5.135498	-1.588010	-1.672940
H	0.385431	4.601520	0.416899
H	-0.884444	3.241874	0.382607
H	0.725863	-0.147102	2.935280
H	-3.761304	2.362620	-0.594054
H	-2.936457	1.327299	-1.779264
H	-5.299027	1.018800	-1.974702
H	-5.517028	-1.424225	-1.698431
H	-3.881400	-1.029554	-2.253615
H	-4.169471	-1.452075	-0.555944
H	-6.026515	1.747583	0.294500
H	-5.434316	0.228906	0.983457
H	-6.777347	0.211110	-0.170016

ωB97XD energy = -1304.08015527 a.u.

Lobatolide A (**11**), Conf. I

C	-2.227404	-1.337566	0.846592
C	-2.837416	-0.347085	1.778669
C	-2.011029	0.959637	1.660293
C	-1.703499	1.374379	0.211591
C	-0.233018	1.193857	-0.284412
C	0.028419	0.119396	-1.371846
C	0.562115	-1.258173	-0.902006
C	-0.368320	-2.188691	-0.118752
O	-0.893125	-1.454947	0.998440
C	-1.618655	-2.577638	-0.930683
C	-2.735687	-2.017512	-0.208746
O	-1.992274	2.795548	0.109476
C	-1.002847	3.495902	-0.456738
C	0.116370	2.578302	-0.783846
C	0.342867	-3.434515	0.387809
C	-4.324279	-0.131357	1.506664
O	-1.582840	-3.235717	-1.961181
O	-1.083924	4.690469	-0.641083
C	1.202552	3.009760	-1.421178
O	-1.125719	-0.018780	-2.171999
O	1.707261	-1.047447	-0.065937
C	2.916658	-0.896846	-0.635965

O	3.071176	-0.824873	-1.837731
C	4.025890	-0.870856	0.385023
C	3.899322	0.169533	1.519898
C	3.646263	1.571727	0.965135
C	2.869413	-0.197912	2.590919
H	-2.710312	-0.725551	2.800146
H	-2.592762	1.761731	2.122299
H	-1.080815	0.865941	2.226562
H	-2.391944	0.894209	-0.487191
H	0.408689	0.958796	0.571269
H	0.851731	0.506506	-1.982817
H	0.873583	-1.802672	-1.800796
H	-3.769322	-2.046412	-0.519167
H	0.760733	-3.987878	-0.457356
H	1.146887	-3.162392	1.073886
H	-0.367791	-4.080894	0.910017
H	-4.738716	0.557179	2.247945
H	-4.880029	-1.071687	1.570325
H	-4.492367	0.300652	0.514417
H	1.285783	4.055673	-1.702480
H	2.026478	2.352432	-1.683316
H	-0.889958	-0.436077	-3.009079
H	4.085840	-1.877825	0.816316
H	4.948013	-0.695069	-0.173705
H	4.883657	0.171451	2.003731
H	4.383786	1.844829	0.202579
H	2.650328	1.639627	0.513052
H	3.691174	2.321039	1.762037
H	2.926294	0.512805	3.422458
H	3.050358	-1.200912	2.993030
H	1.849950	-0.175582	2.195426

ωB97XD energy = -1304.08011691 a.u.

Lobatolide A (**11**), Conf. J

C	-1.553473	-2.059219	0.700989
C	-2.576332	-1.568999	1.670260
C	-2.532193	-0.017521	1.681402
C	-2.403913	0.620079	0.289491
C	-0.981013	1.122716	-0.105720
C	-0.305884	0.441839	-1.329745
C	0.837729	-0.541791	-0.984405
C	0.487033	-1.841049	-0.254840
O	-0.327195	-1.529302	0.888981
C	-0.421685	-2.731853	-1.124704
C	-1.668591	-2.829422	-0.407184
O	-3.265671	1.790875	0.268023
C	-2.620951	2.917036	-0.061188
C	-1.194402	2.600451	-0.322612
C	1.715175	-2.618179	0.191634
C	-3.970578	-2.106559	1.351607
O	-0.075457	-3.214989	-2.193645
O	-3.182392	3.988844	-0.117232
C	-0.314008	3.534696	-0.673857
O	-1.287313	-0.154403	-2.147781
O	1.763283	0.196074	-0.174509
C	2.802783	0.805860	-0.781082

O	3.029973	0.692635	-1.967330
C	3.605076	1.652692	0.173497
C	4.264225	0.902451	1.353113
C	3.281299	0.544742	2.470274
C	5.046876	-0.320210	0.869412
H	-2.280719	-1.912499	2.669141
H	-3.468385	0.332017	2.125189
H	-1.720812	0.332785	2.325588
H	-2.804323	-0.041680	-0.480907
H	-0.309448	0.995406	0.746275
H	0.198412	1.239687	-1.888994
H	1.329590	-0.829467	-1.919086
H	-2.559380	-3.330399	-0.755009
H	2.330014	-2.859770	-0.679587
H	2.306316	-2.033324	0.897725
H	1.409269	-3.551462	0.672111
H	-4.330872	-1.735910	0.385749
H	-4.676343	-1.781716	2.120508
H	-3.981173	-3.200261	1.324863
H	-0.626694	4.569039	-0.784413
H	0.730471	3.299942	-0.862260
H	-0.911107	-0.335115	-3.017468
H	4.374643	2.138117	-0.430565
H	2.938670	2.431739	0.562508
H	4.984693	1.617183	1.770065
H	2.532793	-0.178731	2.136214
H	3.816469	0.109485	3.320879
H	2.750000	1.433615	2.828211
H	5.589233	-0.785166	1.699102
H	5.775248	-0.048122	0.098157
H	4.378585	-1.079853	0.445901

ωB97XD energy = -1304.07966183 a.u.

Lobatolide A (**11**), Conf. K

C	-1.502087	-1.959575	0.894880
C	-2.401880	-1.393926	1.941587
C	-2.362900	0.153791	1.822840
C	-2.391250	0.670363	0.375711
C	-1.028236	1.165544	-0.199787
C	-0.469080	0.402901	-1.434302
C	0.693122	-0.576242	-1.145714
C	0.409530	-1.830748	-0.310411
O	-0.260112	-1.434156	0.899772
C	-0.605296	-2.755294	-1.010478
C	-1.755396	-2.794174	-0.141393
O	-3.287584	1.813999	0.334590
C	-2.712993	2.919225	-0.156358
C	-1.303371	2.616754	-0.509525
C	1.669820	-2.606816	0.041522
C	-3.822416	-1.944306	1.832356
O	-0.397436	-3.303426	-2.083930
O	-3.313676	3.965755	-0.262010
C	-0.480235	3.541642	-0.998035
O	-1.530066	-0.224466	-2.120018
O	1.683340	0.220593	-0.485622
C	2.931805	0.284403	-0.994204

O	3.274053	-0.320660	-1.987412
C	3.824090	1.171383	-0.170028
C	4.330561	0.497891	1.128818
C	5.562802	1.250884	1.635523
C	3.254097	0.435561	2.214391
H	-1.986544	-1.660911	2.920537
H	-3.246040	0.543084	2.336552
H	-1.485974	0.551668	2.340695
H	-2.842402	-0.066027	-0.291611
H	-0.268598	1.115560	0.583274
H	-0.009091	1.160712	-2.081322
H	1.091498	-0.924738	-2.104114
H	-2.684783	-3.304967	-0.343652
H	2.175843	-2.920041	-0.874781
H	2.350208	-1.995839	0.638624
H	1.405624	-3.497694	0.618079
H	-4.296009	-1.640377	0.892801
H	-4.430678	-1.560102	2.655543
H	-3.834574	-3.037003	1.885279
H	-0.831582	4.556381	-1.161633
H	0.554694	3.316782	-1.241575
H	-1.234711	-0.483581	-3.000940
H	4.675120	1.428462	-0.804854
H	3.286986	2.092130	0.083983
H	4.639125	-0.526074	0.878764
H	5.946073	0.794911	2.554346
H	6.367745	1.243370	0.893435
H	5.317149	2.296795	1.857596
H	2.355257	-0.088948	1.880636
H	2.955913	1.447806	2.514732
H	3.637464	-0.078054	3.102277

ωB97XD energy = -1304.07947075 a.u.

Lobatolide A (**11**), Conf. L

C	2.604834	-1.421432	-0.653972
C	3.480521	-0.481960	-1.413313
C	2.774883	0.898710	-1.459786
C	2.158154	1.328036	-0.119588
C	0.609001	1.188491	0.002482
C	0.074783	0.195632	1.072299
C	-0.462298	-1.149858	0.527575
C	0.526283	-2.132464	-0.107164
O	1.323575	-1.427587	-1.074521
C	1.554999	-2.635512	0.924488
C	2.834076	-2.157877	0.459460
O	2.452881	2.739153	0.064959
C	1.362505	3.482335	0.291555
C	0.165127	2.605091	0.274907
C	-0.167332	-3.304147	-0.784550
C	4.883375	-0.393713	-0.814694
O	1.260351	-3.298025	1.909501
O	1.424217	4.678573	0.471739
C	-1.064000	3.079795	0.464049
O	1.051062	0.003230	2.072950
O	-1.457684	-0.838934	-0.455748
C	-2.735734	-0.682122	-0.043394

O	-3.060280	-0.766348	1.120894
C	-3.635215	-0.425869	-1.225888
C	-4.996763	0.218612	-0.925659
C	-5.932382	-0.695182	-0.129563
C	-4.844005	1.590209	-0.262098
H	3.553573	-0.850803	-2.443573
H	3.527198	1.641665	-1.737686
H	2.009260	0.905799	-2.240177
H	2.650597	0.823339	0.713848
H	0.200748	0.878999	-0.962581
H	-0.811024	0.665858	1.515854
H	-0.937307	-1.677812	1.361519
H	3.777497	-2.276913	0.970540
H	-0.771070	-3.845255	-0.050938
H	-0.813772	-2.953312	-1.591229
H	0.578335	-3.990363	-1.195225
H	4.860933	0.039644	0.190878
H	5.512721	0.243381	-1.441943
H	5.356456	-1.378072	-0.752451
H	-1.222378	4.138980	0.644798
H	-1.937941	2.433456	0.445741
H	0.634471	-0.377408	2.855306
H	-3.072110	0.187992	-1.936472
H	-3.776297	-1.400140	-1.713143
H	-5.454919	0.378079	-1.910154
H	-6.053632	-1.665656	-0.624079
H	-5.549752	-0.872699	0.879088
H	-6.924251	-0.238885	-0.041996
H	-5.818525	2.079346	-0.161187
H	-4.201886	2.249588	-0.857388
H	-4.414761	1.500043	0.741584

ωB97XD energy = -1304.07915646 a.u.

3-methylenecyclopent-1-ene (**12**), Conf A

C	-1.531570	-0.607359	-0.000018
C	-0.097887	-1.194205	0.000018
C	0.834517	0.010371	0.000005
C	-0.013028	1.209391	0.000009
C	-1.312528	0.883354	0.000002
C	2.173458	-0.015971	-0.000012
H	-2.108363	-0.919971	0.878340
H	-2.108308	-0.919951	-0.878421
H	0.083029	-1.818443	0.879986
H	0.083061	-1.818498	-0.879903
H	0.387811	2.218235	0.000014
H	-2.131565	1.596152	0.000001
H	2.722473	-0.954106	-0.000020
H	2.754095	0.903097	-0.000021

ωB97XD energy = -233.364666717 a.u.

(*S*)-4-isopropyl-1-methyl-3-methylenecyclohex-1-ene (**13**), Conf. A

C	0.782550	0.112096	0.719612
C	-0.043925	1.331173	0.352394

C	-1.361893	1.061320	-0.243369
C	-1.945823	-0.149970	-0.238612
C	-1.285855	-1.329599	0.432077
C	-0.161151	-0.908352	1.378334
C	1.540619	-0.454513	-0.514415
C	2.566238	0.548630	-1.050049
C	2.235674	-1.782618	-0.197444
C	0.390134	2.586446	0.540804
C	-3.276229	-0.405444	-0.884932
H	1.541458	0.411812	1.454768
H	-1.877881	1.904002	-0.702435
H	-2.045101	-1.900205	0.981570
H	-0.907061	-2.012580	-0.342924
H	-0.602664	-0.441376	2.267411
H	0.391076	-1.784765	1.729059
H	0.805736	-0.637436	-1.310182
H	2.100184	1.492033	-1.346694
H	3.325366	0.772950	-0.289446
H	3.082688	0.140093	-1.925359
H	2.835823	-2.111707	-1.052704
H	1.525172	-2.583303	0.028224
H	2.911371	-1.677410	0.661061
H	-0.214201	3.444380	0.253447
H	1.361847	2.789128	0.983637
H	-3.674117	0.489508	-1.371608
H	-3.193517	-1.198397	-1.639368
H	-4.008218	-0.751737	-0.144868

ωB97XD energy = -429.913680817 a.u.

(*S*)-4-isopropyl-1-methyl-3-methylenecyclohex-1-ene (**13**), Conf. B

C	-0.625600	-0.098912	-0.408684
C	0.174703	1.185208	-0.223027
C	1.633511	1.015752	-0.112861
C	2.250574	-0.172620	-0.003220
C	1.456512	-1.451835	0.071339
C	-0.000648	-1.205965	0.455775
C	-2.152026	0.042145	-0.225065
C	-2.903123	-1.157234	-0.812284
C	-2.569081	0.271201	1.231544
C	-0.350706	2.420732	-0.206574
C	3.744778	-0.303920	0.052200
H	-0.462772	-0.393554	-1.458433
H	2.230904	1.926738	-0.138291
H	1.510857	-1.960594	-0.902865
H	1.929876	-2.131123	0.791349
H	-0.567748	-2.136304	0.352445
H	-0.047461	-0.915120	1.512489
H	-2.464474	0.916749	-0.808758
H	-3.984916	-0.991583	-0.761870
H	-2.639432	-1.320774	-1.863280
H	-2.690794	-2.082014	-0.263767
H	-2.410014	-0.630228	1.834270
H	-3.634718	0.518593	1.290809
H	-2.008242	1.088976	1.694570
H	0.292778	3.292365	-0.111223
H	-1.415987	2.610444	-0.284645
H	4.245613	0.660939	-0.068668
H	4.061253	-0.741955	1.007014
H	4.101908	-0.978878	-0.736003

ωB97XD energy = -429.911869437 a.u.

(*S*)-4-isopropyl-1-methyl-3-methylenecyclohex-1-ene (**13**), Conf. C

C	0.823437	-0.035047	0.861008
C	0.229868	1.219812	0.247128
C	-1.127503	1.115084	-0.306315
C	-1.932287	0.052370	-0.134882
C	-1.500065	-1.135776	0.685675
C	-0.284184	-0.837969	1.565229
C	1.676448	-0.868889	-0.144921
C	1.064079	-1.054310	-1.537935
C	3.080700	-0.273404	-0.291646
C	0.906424	2.378296	0.192310
C	-3.299155	-0.027524	-0.750665
H	1.522743	0.281736	1.644078
H	-1.484915	1.965771	-0.886254
H	-2.339258	-1.458283	1.315482
H	-1.302738	-1.981123	0.011994
H	-0.622448	-0.252615	2.429191
H	0.132346	-1.771047	1.961543
H	1.786376	-1.863308	0.310154
H	1.725475	-1.672482	-2.155447
H	0.088129	-1.543952	-1.512928
H	0.944228	-0.090705	-2.045658
H	3.576218	-0.163399	0.679456
H	3.039905	0.714576	-0.764885
H	3.709659	-0.914524	-0.919457
H	0.476178	3.262984	-0.272244
H	1.901615	2.476378	0.617697
H	-3.519339	0.843398	-1.374625
H	-3.388177	-0.927798	-1.372071
H	-4.072566	-0.101467	0.024048

ωB97XD energy = -429.910925178 a.u.

(*S*)-4-isopropyl-1-methyl-3-methylenecyclohex-1-ene (**13**), Conf. D

C	-0.878436	0.379228	-0.719575
C	0.114567	1.424724	-0.241687
C	1.440496	0.956735	0.180991
C	1.874218	-0.308096	0.043339
C	1.022538	-1.361481	-0.616257
C	-0.133649	-0.767854	-1.424028
C	-1.868654	-0.026700	0.413230
C	-2.883291	-1.064386	-0.074037
C	-1.232596	-0.476665	1.732165
C	-0.213351	2.724533	-0.168209
C	3.219348	-0.750971	0.541606
H	-1.509844	0.853144	-1.481795
H	2.094865	1.696651	0.641400
H	1.656782	-1.968218	-1.275529
H	0.656435	-2.055337	0.152096

H	0.279093	-0.363476	-2.356745
H	-0.835052	-1.555892	-1.714052
H	-2.425163	0.894213	0.635107
H	-3.688296	-1.185398	0.659087
H	-3.338710	-0.767223	-1.025753
H	-2.420369	-2.047934	-0.215031
H	-0.517156	0.258399	2.112370
H	-0.715107	-1.435842	1.632393
H	-2.010730	-0.604936	2.493253
H	0.487793	3.466143	0.208116
H	-1.191271	3.081900	-0.482514
H	3.763322	0.065203	1.025851
H	3.111904	-1.567862	1.266791
H	3.834368	-1.137934	-0.280274

ωB97XD energy = -429.910719744 a.u.

Table S40. Cartesian coordinates and energies of the low-energy conformers calculated at the CAM-B3LYP/TZVP PCM (solvent: MeCN) level.

(2*S*,6*R*,7*S*,8*R*)-**1**, Conf. A

C	-3.956291	-1.594450	-0.433462
C	-3.376646	-0.330803	-0.989098
C	-3.339301	0.867882	-0.413733
C	-4.118690	1.268152	0.805595
C	-1.751744	-1.901034	0.737765
C	-2.818066	-2.579369	-0.082838
C	-0.927793	1.472918	-0.853957
C	-0.428563	1.086607	0.560109
C	0.214800	-0.311937	0.640464
C	-0.713136	-1.360570	0.109961
O	-0.081848	2.570399	-1.292568
C	0.725567	3.018220	-0.319691
C	0.520910	2.199032	0.900474
O	1.470955	3.947773	-0.486587
C	1.106467	2.485231	2.049871
C	-2.386530	1.925589	-0.940887
C	-2.041937	-1.733725	2.198799
O	-4.782788	-2.278040	-1.380259
O	1.405758	-0.261160	-0.174639
C	2.401864	-1.113537	0.113511
O	2.360359	-1.879756	1.043348
C	3.526286	-1.003974	-0.878669
C	4.885505	-1.436473	-0.333569
C	5.892093	-1.553983	-1.473175
C	5.385149	-0.473822	0.739520
H	-4.538356	-1.390823	0.466874
H	-2.794464	-0.485319	-1.894709
H	-3.457940	1.519105	1.639315
H	-4.815821	0.505077	1.142169
H	-2.398960	-2.934780	-1.025677
H	-3.251749	-3.435294	0.437575
H	-4.691048	2.173133	0.587206
H	-1.262609	1.071692	1.258750
H	0.512420	-0.500563	1.670004
H	-0.602186	-1.544571	-0.953434
H	0.941222	1.895134	2.942008
H	1.776121	3.332564	2.122828
H	-2.498574	2.847493	-0.367028
H	-0.748920	0.670872	-1.563891
H	-2.603292	2.170357	-1.982240
H	-2.247981	-2.706328	2.650781
H	-2.934023	-1.122057	2.355632
H	-1.220949	-1.274383	2.745809
H	-5.519326	-1.700604	-1.607916
H	3.240794	-1.637931	-1.724130
H	3.562834	0.017916	-1.259781
H	4.761193	-2.422474	0.120249
H	6.864226	-1.878189	-1.099003
H	5.562410	-2.275003	-2.223270
H	6.031341	-0.591067	-1.971060
H	6.343754	-0.805823	1.141090
H	4.684668	-0.398628	1.572176
H	5.526064	0.527038	0.323380

CAM-B3LYP energy = -1154.93136299 a.u.

(2*S*,6*R*,7*S*,8*R*)-**1**, Conf. B

C	-3.643379	-1.943626	-0.454573
C	-3.179001	-0.660705	-1.071020
C	-3.327061	0.571625	-0.592935
C	-4.236237	0.957855	0.537814
C	-1.515746	-1.883842	0.884910
C	-2.425207	-2.748931	0.050915
C	-0.980249	1.445085	-0.917536
C	-0.548836	1.227637	0.553744
C	0.247304	-0.070917	0.791956
C	-0.504578	-1.263356	0.287058
O	-0.244093	2.608401	-1.384591
C	0.430723	3.220042	-0.400125
C	0.236564	2.469317	0.864753
O	1.070336	4.221044	-0.591100
C	0.700837	2.904214	2.022871
C	-2.471997	1.698958	-1.142377
C	-1.936919	-1.649918	2.304246
O	-4.306491	-2.792594	-1.395976
O	1.482289	0.067313	0.056324
C	2.565476	-0.582818	0.510101
O	2.547608	-1.281307	1.492473
C	3.775295	-0.283975	-0.331601
C	4.882983	-1.329988	-0.236450
C	4.455466	-2.652699	-0.865388
C	6.156335	-0.802475	-0.889546
H	-4.313303	-1.750823	0.385008
H	-2.518411	-0.806159	-1.922792
H	-4.860843	0.140095	0.888127
H	-4.894304	1.767143	0.211775
H	-1.894353	-3.114869	-0.829502
H	-2.791372	-3.613303	0.607876
H	-3.673556	1.347097	1.390138
H	-1.425950	1.163784	1.194572
H	0.485027	-0.145003	1.851349
H	-0.289983	-1.509029	-0.747582
H	0.542470	2.361660	2.945890
H	1.259459	3.830126	2.071838
H	-2.740390	2.640705	-0.659921
H	-0.647796	0.624835	-1.546890
H	-2.634284	1.832680	-2.213364
H	-2.051669	-2.605610	2.820255
H	-2.909233	-1.152897	2.349714
H	-1.226197	-1.046525	2.865531
H	-5.090266	-2.332489	-1.715093
H	3.456585	-0.143255	-1.366015
H	4.144076	0.688397	0.009363
H	5.085270	-1.502868	0.823168

H	5.244489	-3.400021	-0.768172
H	3.558249	-3.053478	-0.392518
H	4.248820	-2.522846	-1.930882
H	6.961198	-1.535188	-0.815411
H	6.495474	0.119755	-0.414543
H	5.992978	-0.593669	-1.949846

CAM-B3LYP energy = -1154.93129627 a.u.

(2*S*,6*R*,7*S*,8*R*)-**1**, Conf. C

C	-4.161768	-0.914152	-0.557048
C	-3.299268	0.211801	-1.036316
C	-3.021755	1.348711	-0.403964
C	-3.738477	1.861315	0.811548
C	-2.115976	-1.751363	0.645143
C	-3.281246	-2.141015	-0.227332
C	-0.523170	1.422872	-0.751051
C	-0.173756	0.868805	0.651758
C	0.155147	-0.637037	0.663757
C	-0.964023	-1.432612	0.065188
O	0.560540	2.323892	-1.107353
C	1.407423	2.537353	-0.089461
C	0.978170	1.731362	1.079738
O	2.346407	3.283474	-0.186257
C	1.559277	1.835060	2.261843
C	-1.841604	2.191335	-0.852550
C	-2.406076	-1.583076	2.106194
O	-5.085193	-1.357544	-1.555977
O	1.346560	-0.795742	-0.136016
C	2.131628	-1.858064	0.098868
O	1.891799	-2.673013	0.955624
C	3.317257	-1.887916	-0.823120
C	4.241170	-0.669346	-0.686947
C	4.801419	-0.547905	0.726271
C	5.362927	-0.756464	-1.715896
H	-4.716016	-0.624686	0.337275
H	-2.734242	-0.030737	-1.933528
H	-4.596346	1.256920	1.095069
H	-4.092278	2.877456	0.620137
H	-2.923800	-2.543299	-1.176626
H	-3.909573	-2.898587	0.244594
H	-3.068486	1.929006	1.672444
H	-1.017275	0.997104	1.326657
H	0.384035	-0.938790	1.684225
H	-0.862154	-1.595598	-1.002538
H	1.229561	1.259066	3.116807
H	2.392539	2.511859	2.401001
H	-1.768871	3.088944	-0.235568
H	-0.498383	0.635290	-1.498160
H	-1.961275	2.523652	-1.885277
H	-1.520465	-1.344762	2.692149
H	-2.839557	-2.501913	2.506929
H	-3.142228	-0.792841	2.273898
H	-5.669139	-0.622842	-1.773033
H	3.868311	-2.804679	-0.613557
H	2.941097	-1.945488	-1.846939
H	3.648563	0.222940	-0.901287

H	5.446090	0.328034	0.810209
H	4.010474	-0.447170	1.471751
H	5.395523	-1.428199	0.983977
H	6.014300	0.116375	-1.652354
H	4.968148	-0.809100	-2.731897
H	5.976753	-1.644467	-1.546271

CAM-B3LYP energy = -1154.93088597 a.u.

(2*S*,6*R*,7*S*,8*R*)-**1**, Conf. D

C	-3.581360	-1.709232	-0.482294
C	-2.933037	-0.550226	-1.173154
C	-3.022789	0.738939	-0.857974
C	-4.017585	1.329531	0.099198
C	-1.627273	-1.651669	1.102298
C	-2.510389	-2.538240	0.262765
C	-0.583990	1.380533	-0.977818
C	-0.352037	1.308296	0.551348
C	0.294029	-0.006911	1.028787
C	-0.500177	-1.190726	0.570837
O	0.306965	2.417928	-1.470470
C	0.906582	3.094354	-0.479493
C	0.493292	2.518893	0.824160
O	1.650141	4.015458	-0.694374
C	0.846514	3.057252	1.978019
C	-2.009461	1.721704	-1.416215
C	-2.185904	-1.212544	2.422088
O	-4.199983	-2.614428	-1.401469
O	1.611211	-0.052986	0.439408
C	2.571261	-0.733911	1.083987
O	2.389465	-1.277789	2.145502
C	3.862652	-0.728211	0.317542
C	3.783084	-1.490377	-1.014085
C	5.106536	-1.362695	-1.760077
C	3.413097	-2.954626	-0.801629
H	-4.327436	-1.366074	0.236437
H	-2.194945	-0.849091	-1.914065
H	-3.523960	1.778950	0.964723
H	-4.559007	2.138879	-0.396942
H	-1.916936	-3.051092	-0.495921
H	-3.017920	-3.295680	0.862860
H	-4.748509	0.610633	0.460370
H	-1.302154	1.381495	1.076051
H	0.403216	0.029488	2.110877
H	-0.192028	-1.576332	-0.395248
H	0.528956	2.643682	2.926471
H	1.472973	3.939826	1.997326
H	-2.244057	2.732399	-1.077020
H	-0.258087	0.466993	-1.466127
H	-2.033248	1.738273	-2.507244
H	-1.489680	-0.606679	2.998705
H	-2.452772	-2.085333	3.021880
H	-3.103514	-0.634096	2.288366
H	-4.893011	-2.138657	-1.871706
H	4.137266	0.310459	0.123950
H	4.625398	-1.170076	0.958621
H	3.002824	-1.020237	-1.617609

H	5.057336	-1.872439	-2.723408
H	5.361117	-0.317645	-1.944016
H	5.920521	-1.812145	-1.185958
H	3.356133	-3.478270	-1.756833
H	2.447143	-3.065541	-0.305824
H	4.163712	-3.457956	-0.187249

CAM-B3LYP energy = -1154.93086664 a.u.

(2*S*,6*R*,7*S*,8*R*)-**1**, Conf. E

C	-3.962652	-1.596505	-0.431949
C	-3.381429	-0.332388	-0.987631
C	-3.341733	0.866770	-0.414942
C	-4.119213	1.267493	0.805212
C	-1.750258	-1.897011	0.742355
C	-2.815778	-2.579939	-0.075372
C	-0.930531	1.471977	-0.856864
C	-0.430963	1.090714	0.558314
C	0.214913	-0.306455	0.642749
C	-0.710311	-1.358762	0.114654
O	-0.084935	2.568175	-1.299378
C	0.721596	3.020298	-0.327694
C	0.516601	2.205714	0.895510
O	1.466524	3.949635	-0.497718
C	1.100208	2.497327	2.044536
C	-2.389476	1.923631	-0.944990
C	-2.045872	-1.721003	2.201335
O	-4.885926	-2.223982	-1.322848
O	1.406449	-0.255854	-0.171449
C	2.402388	-1.108045	0.118200
O	2.359855	-1.873726	1.048397
C	3.527571	-0.998899	-0.873157
C	4.884561	-1.440903	-0.330296
C	5.890094	-1.559908	-1.470702
C	5.390001	-0.485039	0.746141
H	-4.545931	-1.392940	0.462742
H	-2.795485	-0.483650	-1.892690
H	-3.457589	1.528427	1.635133
H	-4.810031	0.501302	1.147775
H	-2.387245	-2.942615	-1.012945
H	-3.248844	-3.434545	0.447492
H	-4.699884	2.166451	0.583860
H	-1.265051	1.076293	1.256887
H	0.512066	-0.491722	1.673066
H	-0.595430	-1.548567	-0.947380
H	0.934490	1.910619	2.938818
H	1.768644	3.345833	2.114987
H	-2.501950	2.846873	-0.373400
H	-0.751306	0.667508	-1.564023
H	-2.606469	2.165943	-1.986857
H	-2.251456	-2.691031	2.658974
H	-2.939498	-1.109972	2.351677
H	-1.227682	-1.256427	2.748054
H	-4.432999	-2.416720	-2.152087
H	3.238993	-1.626757	-1.722115
H	3.568984	0.024823	-1.248927
H	4.754941	-2.427860	0.119948

H	6.860502	-1.890943	-1.098036
H	5.556113	-2.276356	-2.223282
H	6.034624	-0.596007	-1.965153
H	6.346687	-0.824083	1.146407
H	4.690028	-0.408685	1.579105
H	5.536756	0.516416	0.333482

CAM-B3LYP energy = -1154.93052753 a.u.

(2*S*,6*R*,7*S*,8*R*)-**1**, Conf. F

C	-3.970609	-1.577642	-0.438833
C	-3.389744	-0.318400	-0.994539
C	-3.337552	0.875572	-0.412386
C	-4.106052	1.276069	0.813709
C	-1.756395	-1.893118	0.732632
C	-2.823000	-2.565248	-0.091963
C	-0.924415	1.475653	-0.855106
C	-0.424946	1.090087	0.558990
C	0.215011	-0.309837	0.639549
C	-0.715140	-1.355202	0.106791
O	-0.077024	2.571622	-1.295137
C	0.731902	3.018814	-0.323300
C	0.526958	2.200885	0.897683
O	1.478724	3.947024	-0.491412
C	1.114069	2.486694	2.046380
C	-2.382427	1.929962	-0.942265
C	-2.048741	-1.726451	2.193322
O	-4.831641	-2.155059	-1.422779
O	1.407095	-0.261601	-0.174044
C	2.400016	-1.117739	0.113719
O	2.354656	-1.885860	1.041841
C	3.526340	-1.009573	-0.876471
C	4.883521	-1.446543	-0.329874
C	5.891817	-1.564198	-1.467948
C	5.383682	-0.487338	0.746074
H	-4.546807	-1.371560	0.464411
H	-2.817059	-0.472611	-1.906014
H	-3.439148	1.522007	1.643982
H	-4.675767	2.184105	0.601198
H	-2.404346	-2.915016	-1.037039
H	-3.250103	-3.426807	0.427924
H	-4.805014	0.515459	1.152007
H	-1.258405	1.077584	1.258304
H	0.510840	-0.500102	1.669320
H	-0.603349	-1.538482	-0.956614
H	0.948487	1.897383	2.938981
H	1.785361	3.332819	2.118286
H	-2.492631	2.853491	-0.370697
H	-0.747020	0.672665	-1.564376
H	-2.599256	2.172394	-1.984113
H	-1.226533	-1.271497	2.742170
H	-2.259859	-2.698570	2.644066
H	-2.938136	-1.110806	2.349537
H	-5.173275	-2.988719	-1.080555
H	3.240571	-1.641423	-1.723418
H	3.566199	0.012823	-1.255888
H	4.756008	-2.433172	0.121705

H	6.862468	-1.891643	-1.092750
H	5.561630	-2.282776	-2.220160
H	6.034367	-0.600581	-1.963530
H	6.340762	-0.822536	1.148631
H	4.681947	-0.412120	1.577676
H	5.527805	0.514011	0.332228

CAM-B3LYP energy = -1154.93047800 a.u.

(2*S*,6*R*,7*S*,8*R*)-**1**, Conf. G

C	-3.658692	-1.934908	-0.454818
C	-3.184840	-0.654175	-1.071138
C	-3.327201	0.579810	-0.597427
C	-4.236621	0.971687	0.530896
C	-1.524580	-1.879277	0.888207
C	-2.437042	-2.744053	0.057179
C	-0.976124	1.441927	-0.919971
C	-0.548461	1.225937	0.552456
C	0.245443	-0.073560	0.793277
C	-0.508154	-1.266289	0.291216
O	-0.233404	2.600373	-1.388492
C	0.440278	3.212625	-0.403503
C	0.238916	2.466440	0.862955
O	1.084226	4.210624	-0.595373
C	0.699606	2.903744	2.021592
C	-2.466224	1.702157	-1.147916
C	-1.951596	-1.634406	2.304013
O	-4.434181	-2.731960	-1.350958
O	1.480263	0.061648	0.057020
C	2.564134	-0.585761	0.513230
O	2.546636	-1.281745	1.497333
C	3.773957	-0.286853	-0.328420
C	4.882751	-1.331491	-0.231630
C	4.456895	-2.655429	-0.859103
C	6.155657	-0.803293	-0.885057
H	-4.327888	-1.738313	0.379234
H	-2.517806	-0.799431	-1.919483
H	-3.674815	1.370909	1.379138
H	-4.858266	0.154622	0.888090
H	-1.898221	-3.117840	-0.816874
H	-2.807399	-3.605151	0.616221
H	-4.898806	1.775004	0.198456
H	-1.426894	1.164421	1.191662
H	0.483340	-0.145789	1.852804
H	-0.289315	-1.519075	-0.740868
H	0.535908	2.364612	2.945680
H	1.260527	3.828304	2.069901
H	-2.731249	2.645971	-0.667693
H	-0.646385	0.618559	-1.546725
H	-2.626267	1.834786	-2.219361
H	-2.073947	-2.586151	2.825497
H	-2.921220	-1.131582	2.342354
H	-1.240247	-1.031369	2.864831
H	-3.904500	-2.922432	-2.133897
H	3.455552	-0.147592	-1.363121
H	4.141556	0.686302	0.011604
H	5.084951	-1.502827	0.828251

H	5.246735	-3.401732	-0.760785
H	3.560002	-3.056676	-0.385985
H	4.250433	-2.527048	-1.924816
H	6.961370	-1.534951	-0.809816
H	6.493587	0.119923	-0.411113
H	5.992329	-0.595969	-1.945655

CAM-B3LYP energy = -1154.93044824 a.u.

(2*S*,6*R*,7*S*,8*R*)-**1**, Conf. H

C	-3.661909	-1.925906	-0.463986
C	-3.194448	-0.646958	-1.078238
C	-3.326065	0.582700	-0.590684
C	-4.223431	0.971008	0.548712
C	-1.524175	-1.876655	0.879027
C	-2.435058	-2.734407	0.039692
C	-0.976309	1.448062	-0.917655
C	-0.544540	1.230994	0.553447
C	0.246580	-0.070556	0.791414
C	-0.509533	-1.259053	0.283781
O	-0.236370	2.608262	-1.386688
C	0.441557	3.218518	-0.403550
C	0.245610	2.470075	0.862463
O	1.084737	4.216917	-0.596198
C	0.712048	2.904929	2.019720
C	-2.467138	1.706163	-1.142192
C	-1.946881	-1.644288	2.298152
O	-4.367118	-2.675616	-1.455572
O	1.482590	0.063673	0.056839
C	2.563764	-0.589030	0.511645
O	2.543367	-1.287411	1.494048
C	3.774950	-0.292859	-0.329033
C	4.880992	-1.340393	-0.232057
C	4.452227	-2.662986	-0.860397
C	6.155650	-0.815148	-0.884462
H	-4.328264	-1.730624	0.377576
H	-2.543590	-0.793394	-1.937084
H	-3.652644	1.357633	1.396782
H	-4.848328	0.155096	0.902708
H	-1.904763	-3.095136	-0.843118
H	-2.795642	-3.603726	0.595852
H	-4.881435	1.782786	0.228772
H	-1.421190	1.170953	1.195160
H	0.483105	-0.146920	1.850921
H	-0.294558	-1.503865	-0.750969
H	0.552242	2.364292	2.943611
H	1.273952	3.828942	2.067041
H	-2.732296	2.649725	-0.661528
H	-0.647308	0.625794	-1.546190
H	-2.629302	1.838073	-2.213381
H	-2.067066	-2.600518	2.811982
H	-2.916604	-1.142367	2.343486
H	-1.233932	-1.045783	2.861853
H	-4.627390	-3.521018	-1.073070
H	3.457509	-0.152361	-1.363871
H	4.144837	0.679233	0.011541
H	5.082126	-1.512738	0.827868

H	5.240193	-3.411262	-0.761988
H	3.554076	-3.062215	-0.387991
H	4.246663	-2.533603	-1.926157
H	6.959460	-1.548883	-0.809025
H	6.495639	0.107021	-0.409945
H	5.993510	-0.606993	-1.945078

CAM-B3LYP energy = -1154.93039748 a.u.

(2*S*,6*R*,7*S*,8*R*)-**1**, Conf. I

C	-4.171143	-0.906137	-0.556142
C	-3.302930	0.216595	-1.036312
C	-3.020675	1.353724	-0.407800
C	-3.735398	1.870153	0.807025
C	-2.119641	-1.744686	0.651203
C	-3.286543	-2.136338	-0.218167
C	-0.521898	1.420528	-0.754747
C	-0.173912	0.870561	0.649910
C	0.153823	-0.635516	0.666349
C	-0.965826	-1.432871	0.070898
O	0.564331	2.317193	-1.114319
C	1.410919	2.533104	-0.096633
C	0.978939	1.733010	1.075596
O	2.351702	3.276604	-0.195904
C	1.558915	1.840620	2.257901
C	-1.838308	2.191989	-0.858997
C	-2.412365	-1.563524	2.110261
O	-5.180104	-1.273111	-1.498255
O	1.344781	-0.797374	-0.133378
C	2.129481	-1.859419	0.104313
O	1.889073	-2.672219	0.962908
C	3.315371	-1.891693	-0.817263
C	4.239912	-0.673368	-0.682999
C	4.800052	-0.549925	0.730090
C	5.361739	-0.762709	-1.711677
H	-4.725437	-0.613039	0.332062
H	-2.733079	-0.026085	-1.931915
H	-4.096039	2.882815	0.609945
H	-3.062770	1.947732	1.664991
H	-2.922822	-2.550794	-1.161497
H	-3.916735	-2.890019	0.257246
H	-4.589622	1.263626	1.096998
H	-1.017579	1.001754	1.324024
H	0.383000	-0.934166	1.687704
H	-0.861769	-1.604399	-0.995352
H	1.227162	1.268804	3.114877
H	2.393264	2.516466	2.395162
H	-1.763545	3.091248	-0.244707
H	-0.499121	0.630104	-1.498974
H	-1.956923	2.521700	-1.892667
H	-2.850487	-2.477212	2.517571
H	-3.145209	-0.768703	2.270610
H	-1.526923	-1.324239	2.696029
H	-4.753471	-1.539124	-2.321204
H	3.865844	-2.808372	-0.605840
H	2.939528	-1.950878	-1.841109
H	3.647789	0.218870	-0.898854

H	5.445299	0.325720	0.812623
H	4.009078	-0.447380	1.475291
H	5.393533	-1.430178	0.989354
H	6.013458	0.109964	-1.649504
H	4.967064	-0.816862	-2.727641
H	5.975176	-1.650677	-1.540484

CAM-B3LYP energy = -1154.93005497 a.u.

(2*S*,6*R*,7*S*,8*R*)-**1**, Conf. J

C	-3.592414	-1.704456	-0.484665
C	-2.938857	-0.546289	-1.174476
C	-3.024029	0.742930	-0.860840
C	-4.017518	1.335528	0.096180
C	-1.631015	-1.648596	1.102159
C	-2.516448	-2.535326	0.265111
C	-0.583663	1.379152	-0.980528
C	-0.352668	1.308493	0.548733
C	0.292384	-0.006568	1.028063
C	-0.501912	-1.191008	0.571732
O	0.309990	2.413700	-1.474202
C	0.909475	3.091055	-0.483700
C	0.493533	2.518707	0.820493
O	1.654891	4.010453	-0.699357
C	0.845563	3.059023	1.973808
C	-2.008223	1.722958	-1.419686
C	-2.192892	-1.203124	2.418522
O	-4.324636	-2.544330	-1.378253
O	1.609862	-0.054311	0.439622
C	2.569903	-0.732461	1.087289
O	2.387393	-1.273598	2.150061
C	3.862225	-0.727466	0.322407
C	3.784883	-1.492112	-1.007905
C	5.109449	-1.365508	-1.752101
C	3.414939	-2.956053	-0.793238
H	-4.335380	-1.359834	0.230489
H	-2.196329	-0.842070	-1.913787
H	-3.523126	1.790972	0.958094
H	-4.562898	2.140458	-0.402815
H	-1.915836	-3.054214	-0.485906
H	-3.025873	-3.290437	0.866351
H	-4.746326	0.616745	0.461940
H	-1.303041	1.383047	1.072761
H	0.400971	0.031210	2.110186
H	-0.191344	-1.580418	-0.392149
H	0.526004	2.647808	2.922610
H	1.472985	3.940937	1.992262
H	-2.240704	2.734367	-1.081230
H	-0.259526	0.464200	-1.467453
H	-2.031784	1.739129	-2.510722
H	-1.497653	-0.595239	2.994147
H	-2.462061	-2.072896	3.021629
H	-3.109511	-0.624180	2.280332
H	-3.722355	-2.880966	-2.052030
H	4.136452	0.311023	0.127280
H	4.624419	-1.167648	0.965295
H	3.005434	-1.023268	-1.613490

H	5.061857	-1.876998	-2.714590
H	5.364095	-0.320741	-1.937523
H	5.922639	-1.813744	-1.175917
H	3.359671	-3.481581	-1.747515
H	2.448235	-3.066222	-0.298698
H	4.164682	-3.458010	-0.176683

CAM-B3LYP energy = -1154.93003552 a.u.

(2*S*,6*R*,7*S*,8*R*)-**1**, Conf. K

C	-4.173844	-0.896281	-0.559218
C	-3.313212	0.226731	-1.038710
C	-3.021754	1.354898	-0.398660
C	-3.725510	1.863085	0.826191
C	-2.118005	-1.743595	0.638993
C	-3.282526	-2.127007	-0.236605
C	-0.523383	1.425557	-0.753119
C	-0.170518	0.872174	0.648983
C	0.155493	-0.634192	0.660568
C	-0.964958	-1.426288	0.060151
O	0.560317	2.325355	-1.113021
C	1.410621	2.538074	-0.097910
C	0.983848	1.733335	1.073101
O	2.350435	3.282754	-0.197927
C	1.568555	1.837152	2.253414
C	-1.841359	2.194843	-0.852004
C	-2.408782	-1.577865	2.100240
O	-5.107686	-1.225969	-1.589816
O	1.347277	-0.795329	-0.138122
C	2.129267	-1.859901	0.096824
O	1.885798	-2.675344	0.952115
C	3.316800	-1.891153	-0.822597
C	4.246349	-0.677791	-0.677619
C	4.804164	-0.566783	0.737398
C	5.369932	-0.764730	-1.704603
H	-4.720730	-0.607946	0.339733
H	-2.759320	-0.011247	-1.943837
H	-4.077359	2.881401	0.642961
H	-3.048624	1.923919	1.682155
H	-2.924441	-2.521313	-1.188917
H	-3.904231	-2.893392	0.233527
H	-4.583653	1.260440	1.112542
H	-1.011857	1.002056	1.326206
H	0.382589	-0.937278	1.681050
H	-0.862924	-1.587570	-1.007807
H	1.240676	1.262215	3.109783
H	2.403109	2.512971	2.389559
H	-1.765758	3.093647	-0.237135
H	-0.501474	0.637495	-1.499825
H	-1.963701	2.524995	-1.885069
H	-2.846027	-2.495928	2.498715
H	-3.141580	-0.785016	2.269896
H	-1.522415	-1.344642	2.687103
H	-5.633052	-1.979714	-1.298719
H	3.862894	-2.811614	-0.616344
H	2.942998	-1.941281	-1.847642
H	3.658499	0.218497	-0.888357

H	5.453033	0.305450	0.827350
H	4.012237	-0.466107	1.481836
H	5.393315	-1.451416	0.991584
H	6.025397	0.104561	-1.634835
H	4.977140	-0.809839	-2.721728
H	5.979048	-1.656638	-1.538520

CAM-B3LYP energy = -1154.92999862 a.u.

(2*S*,6*R*,7*S*,8*R*)-**1**, Conf. L

C	-3.601503	-1.686865	-0.493018
C	-2.950134	-0.532644	-1.182101
C	-3.023023	0.753728	-0.854626
C	-4.006075	1.347351	0.112709
C	-1.633472	-1.650046	1.088609
C	-2.520012	-2.525755	0.241846
C	-0.580756	1.384111	-0.977810
C	-0.347389	1.307717	0.550816
C	0.293309	-0.011555	1.024095
C	-0.504727	-1.190015	0.559470
O	0.313758	2.419192	-1.469175
C	0.917104	3.091019	-0.477387
C	0.503001	2.514271	0.825486
O	1.664072	4.009701	-0.690934
C	0.859196	3.048827	1.980201
C	-2.005259	1.731284	-1.414178
C	-2.190356	-1.217041	2.411190
O	-4.259480	-2.490294	-1.475019
O	1.611403	-0.060342	0.437058
C	2.569608	-0.741382	1.084223
O	2.384979	-1.285437	2.145190
C	3.863138	-0.735459	0.321401
C	3.787438	-1.497243	-1.010643
C	5.113107	-1.369327	-1.752646
C	3.416812	-2.961522	-0.799571
H	-4.336151	-1.339216	0.234901
H	-2.222219	-0.833305	-1.932059
H	-4.739538	0.631937	0.475608
H	-4.545825	2.161703	-0.377042
H	-1.929805	-3.030330	-0.524818
H	-3.020360	-3.290928	0.841121
H	-3.503902	1.790795	0.976337
H	-1.296574	1.383638	1.076703
H	0.400599	0.019795	2.106521
H	-0.198419	-1.570840	-0.409057
H	0.540816	2.634362	2.927987
H	1.488947	3.929026	2.000922
H	-2.234713	2.743197	-1.075128
H	-0.258990	0.470384	-1.468498
H	-2.030456	1.747388	-2.505150
H	-1.490583	-0.619428	2.992093
H	-2.462657	-2.092207	3.005093
H	-3.104231	-0.631948	2.281324
H	-4.643124	-3.258001	-1.036558
H	4.138207	0.303298	0.128903
H	4.624119	-1.177440	0.964504
H	3.008951	-1.026948	-1.616332

H	5.066815	-1.878810	-2.716256
H	5.368163	-0.324207	-1.935537
H	5.925401	-1.818858	-1.176195
H	3.363086	-3.485076	-1.755012
H	2.449176	-3.072398	-0.307029
H	4.165372	-3.464876	-0.182699

CAM-B3LYP energy = -1154.92998489 a.u.

(2*S*,6*R*,7*S*,8*R*)-**1**, Conf. M

C	-4.092654	-1.198547	-0.375711
C	-3.389722	0.022723	-0.881579
C	-3.168935	1.161228	-0.230050
C	-3.821259	1.559714	1.062349
C	-1.880732	-1.863372	0.620847
C	-3.071587	-2.340145	-0.170102
C	-0.726414	1.504428	-0.773515
C	-0.212089	0.940559	0.573941
C	0.262786	-0.523593	0.499604
C	-0.821181	-1.406891	-0.037598
O	0.227306	2.527895	-1.169499
C	1.126336	2.792240	-0.209727
C	0.876414	1.903164	0.950957
O	1.972774	3.636885	-0.343316
C	1.535718	2.023765	2.089534
C	-2.121342	2.132442	-0.743209
C	-2.060678	-1.773904	2.106309
O	-5.052896	-1.701757	-1.309576
O	1.392154	-0.540140	-0.400615
C	2.272093	-1.548524	-0.286946
O	2.171024	-2.409713	0.552285
C	3.370801	-1.460553	-1.309730
C	4.725613	-1.000322	-0.735691
C	4.637706	0.387909	-0.110423
C	5.330285	-2.009807	0.234271
H	-4.591247	-0.994234	0.573182
H	-2.886095	-0.133051	-1.832963
H	-4.585524	0.861591	1.394219
H	-4.290509	2.539367	0.942338
H	-2.757993	-2.672310	-1.161162
H	-3.580437	-3.172923	0.318924
H	-3.087306	1.669829	1.864721
H	-1.007760	0.957731	1.315483
H	0.603780	-0.832526	1.486084
H	-0.798823	-1.518930	-1.116412
H	1.335462	1.384110	2.939361
H	2.303727	2.779035	2.196486
H	-2.089816	3.017409	-0.104878
H	-0.678256	0.752089	-1.555197
H	-2.359480	2.476008	-1.751423
H	-2.861437	-1.076063	2.364243
H	-1.159167	-1.455332	2.626094
H	-2.355402	-2.746946	2.505134
H	-5.723554	-1.024005	-1.446538
H	3.484606	-2.455817	-1.741575
H	3.060989	-0.778002	-2.099357
H	5.389337	-0.935003	-1.601882

H	5.627476	0.733677	0.191011
H	4.224219	1.116635	-0.809397
H	4.005007	0.384636	0.780272
H	6.329655	-1.690780	0.534626
H	5.414619	-2.997490	-0.222225
H	4.724090	-2.109170	1.135437

CAM-B3LYP energy = -1154.92879666 a.u.

(2*S*,6*R*,7*S*,8*R*)-**1**, Conf. N

C	-3.397125	-2.021798	-0.324003
C	-2.967773	-0.762500	-1.010732
C	-3.169872	0.490306	-0.611922
C	-4.112650	0.909792	0.478791
C	-1.295682	-1.808569	1.040954
C	-2.160431	-2.752317	0.245706
C	-0.849691	1.431435	-0.947032
C	-0.440461	1.313260	0.541854
C	0.399132	0.062126	0.867216
C	-0.300798	-1.183658	0.420551
O	-0.148972	2.594984	-1.465139
C	0.480357	3.287253	-0.504103
C	0.288721	2.600935	0.797266
O	1.084179	4.300999	-0.739185
C	0.709533	3.118938	1.937674
C	-2.345354	1.613558	-1.214157
C	-1.743823	-1.514606	2.440658
O	-4.015880	-2.948801	-1.221167
O	1.642465	0.204804	0.146473
C	2.744676	-0.358508	0.668060
O	2.736859	-0.966037	1.710759
C	3.955377	-0.137522	-0.195311
C	4.392716	-1.387572	-0.985444
C	4.907240	-2.504771	-0.084009
C	3.293560	-1.888353	-1.917025
H	-4.086192	-1.802526	0.493336
H	-2.287368	-0.934696	-1.841648
H	-4.715406	0.092318	0.866000
H	-4.791699	1.675134	0.094848
H	-1.602209	-3.149780	-0.603528
H	-2.505505	-3.595378	0.847032
H	-3.579490	1.367364	1.316085
H	-1.327076	1.248216	1.169100
H	0.620041	0.056395	1.932701
H	-0.066739	-1.476188	-0.597307
H	0.552380	2.623121	2.886839
H	1.229689	4.068239	1.944722
H	-2.658363	2.571909	-0.795544
H	-0.472840	0.591383	-1.522944
H	-2.490332	1.675578	-2.294160
H	-1.068844	-0.847737	2.973464
H	-1.820589	-2.443963	3.009192
H	-2.738764	-1.062310	2.448381
H	-4.808770	-2.536049	-1.580009
H	3.748564	0.676129	-0.888462
H	4.767355	0.170488	0.464524
H	5.227596	-1.050345	-1.605289

H	5.292301	-3.328341	-0.687515
H	5.714309	-2.154139	0.561501
H	4.115259	-2.897709	0.554695
H	3.666584	-2.697234	-2.546682
H	2.928477	-1.093634	-2.569507
H	2.442525	-2.278416	-1.353709

CAM-B3LYP energy = -1154.92878529 a.u.

(2*S*,6*R*,7*S*,8*R*)-**1**, Conf. O

C	-4.148740	-1.356570	0.238596
C	-3.651423	0.059513	0.066502
C	-3.103022	0.628098	-1.002970
C	-3.072477	0.025256	-2.376942
C	-1.770640	-1.827300	0.942111
C	-2.979464	-2.375031	0.232329
C	-0.842411	1.673252	-0.693650
C	-0.383127	1.159448	0.694784
C	0.195494	-0.265813	0.701388
C	-0.822808	-1.244904	0.215072
O	-0.155539	2.938017	-0.902670
C	0.668364	3.257581	0.104335
C	0.590710	2.210072	1.150412
O	1.332539	4.260926	0.091291
C	1.291516	2.281874	2.268247
C	-2.344694	1.925866	-0.852395
C	-1.832802	-1.802102	2.439794
O	-5.062019	-1.807448	-0.762867
O	1.340810	-0.250363	-0.177923
C	2.314588	-1.147826	0.040405
O	2.296950	-1.924436	0.962402
C	3.379280	-1.071369	-1.018802
C	4.745411	-1.588164	-0.574923
C	5.667280	-1.730362	-1.781670
C	5.366539	-0.680616	0.482543
H	-4.646205	-1.410309	1.211169
H	-3.609316	0.617043	0.997849
H	-3.568117	-0.939659	-2.414089
H	-2.046413	-0.089549	-2.735782
H	-2.737379	-2.574111	-0.811834
H	-3.331888	-3.305909	0.680556
H	-3.571303	0.694417	-3.083139
H	-1.240426	1.137445	1.368110
H	0.538566	-0.497932	1.707110
H	-0.885567	-1.316397	-0.865586
H	1.231261	1.522733	3.037234
H	1.954864	3.119901	2.439350
H	-2.699090	2.490998	0.011279
H	-0.482485	1.019344	-1.485283
H	-2.477545	2.551235	-1.737464
H	-0.892617	-1.504354	2.899608
H	-2.607786	-1.112946	2.788366
H	-2.098846	-2.789993	2.820635
H	-5.818272	-1.210734	-0.771015
H	3.006664	-1.666735	-1.858292
H	3.445785	-0.043003	-1.377993
H	4.597949	-2.577289	-0.135099

H	6.642461	-2.115772	-1.480551
H	5.250105	-2.412969	-2.524148
H	5.826720	-0.763784	-2.266293
H	6.331302	-1.071382	0.809530
H	4.728565	-0.591498	1.362655
H	5.531634	0.322219	0.080242

CAM-B3LYP energy = -1154.92873763 a.u.

(2*S*,6*R*,7*S*,8*R*)-**1**, Conf. P

C	-3.693349	-1.919836	-0.347610
C	-3.274321	-0.607453	-0.933791
C	-3.376613	0.598784	-0.382500
C	-4.184885	0.925932	0.839893
C	-1.451891	-1.935053	0.793455
C	-2.441900	-2.754716	0.006762
C	-1.061651	1.481716	-0.869347
C	-0.500477	1.179361	0.541553
C	0.311891	-0.127832	0.626609
C	-0.493651	-1.291386	0.135950
O	-0.360851	2.667388	-1.334566
C	0.399843	3.223042	-0.379884
C	0.310999	2.403501	0.853708
O	1.025945	4.232765	-0.569980
C	0.870141	2.775355	1.991715
C	-2.565150	1.753175	-0.941922
C	-1.740836	-1.764092	2.254345
O	-4.441561	-2.715970	-1.271568
O	1.465698	0.055414	-0.219874
C	2.572483	-0.658819	0.054047
O	2.640789	-1.419675	0.985530
C	3.651804	-0.353879	-0.950519
C	4.993705	-1.064517	-0.766031
C	5.689278	-0.677996	0.536933
C	4.884437	-2.579048	-0.924146
H	-4.286924	-1.770240	0.555833
H	-2.687469	-0.709785	-1.843863
H	-4.860662	1.754740	0.615065
H	-3.549060	1.264761	1.661949
H	-1.997001	-3.080995	-0.934698
H	-2.763797	-3.642898	0.553537
H	-4.784983	0.092482	1.196082
H	-1.315944	1.071292	1.253653
H	0.654140	-0.259641	1.651390
H	-0.376197	-1.488044	-0.924462
H	0.786810	2.182288	2.893279
H	1.432982	3.698410	2.045679
H	-2.779884	2.665548	-0.382264
H	-0.794743	0.697412	-1.571403
H	-2.823620	1.950645	-1.983790
H	-1.831042	-2.741462	2.733207
H	-2.693760	-1.251677	2.409523
H	-0.969739	-1.201745	2.777320
H	-5.245140	-2.236284	-1.499548
H	3.235884	-0.584799	-1.934512
H	3.793853	0.729532	-0.939605
H	5.617446	-0.697905	-1.586691

H	6.693386	-1.103895	0.572756
H	5.784288	0.406148	0.626525
H	5.136836	-1.043679	1.402163
H	5.875630	-3.035449	-0.909553
H	4.410779	-2.843133	-1.871978
H	4.298625	-3.018439	-0.117264

CAM-B3LYP energy = -1154.92872925 a.u.

H	-4.630249	-2.530645	0.130168
H	-6.740223	-1.962721	1.325986
H	-5.413968	-2.215672	2.463188
H	-5.948035	-0.577242	2.076701
H	-6.282193	-1.062617	-1.001056
H	-4.643586	-0.634010	-1.486875
H	-5.505224	0.361991	-0.308059

CAM-B3LYP energy = -1154.93114223 a.u.

(2*R*,6*R*,7*S*,8*R*)-**1**, Conf. A

C	4.136118	-1.396726	0.285066
C	3.635252	0.019213	0.229780
C	3.069041	0.717397	1.207144
C	3.004296	0.266137	2.636852
C	1.813494	-1.913036	-0.609757
C	2.967112	-2.416857	0.217007
C	0.821455	1.713110	0.686793
C	0.423759	1.091566	-0.676271
C	-0.154653	-0.331184	-0.598989
C	0.832115	-1.269788	0.015628
O	0.122757	2.986345	0.767922
C	-0.655961	3.223744	-0.296089
C	-0.528719	2.100279	-1.254751
O	-1.322322	4.221437	-0.389103
C	-1.175607	2.084065	-2.406765
C	2.314880	1.985622	0.888337
C	1.960821	-2.008210	-2.097937
O	5.002503	-1.667229	-0.820921
O	-1.344471	-0.249204	0.216206
C	-2.318209	-1.147473	0.001742
O	-2.261986	-1.982716	-0.865931
C	-3.439342	-0.989914	0.991465
C	-4.786481	-1.515178	0.501571
C	-5.777398	-1.572913	1.659469
C	-5.332116	-0.664470	-0.641513
H	4.681596	-1.586622	1.213026
H	3.620143	0.432848	-0.774982
H	1.974912	0.066182	2.946417
H	3.379645	1.052485	3.295650
H	2.627680	-2.595981	1.237337
H	3.374724	-3.353182	-0.168004
H	3.584338	-0.635723	2.820153
H	1.309231	1.020600	-1.308649
H	-0.447118	-0.642895	-1.598975
H	0.831969	-1.259423	1.100977
H	-1.077604	1.269656	-3.112808
H	-1.831227	2.903388	-2.672132
H	2.710268	2.461520	-0.010786
H	0.430106	1.120085	1.510952
H	2.407070	2.698661	1.709894
H	2.813582	-1.415967	-2.438106
H	1.074947	-1.677563	-2.636593
H	2.168267	-3.041010	-2.385778
H	5.734457	-1.041313	-0.793611
H	-3.125668	-1.536132	1.886614
H	-3.508832	0.059934	1.281259

(2*R*,6*R*,7*S*,8*R*)-**1**, Conf. B

C	-4.145872	-1.385217	-0.271234
C	-3.647636	0.028037	-0.231091
C	-3.065649	0.708897	-1.210478
C	-2.985468	0.237613	-2.633096
C	-1.811064	-1.909264	0.595567
C	-2.964944	-2.403816	-0.234265
C	-0.822593	1.717235	-0.694026
C	-0.427885	1.097454	0.670532
C	0.153814	-0.324121	0.595353
C	-0.829007	-1.262471	-0.025311
O	-0.129564	2.993872	-0.771987
C	0.645287	3.233512	0.294284
C	0.519877	2.108858	1.251893
O	1.307521	4.233745	0.389873
C	1.164080	2.094141	2.405429
C	-2.316303	1.982349	-0.901657
C	-1.958241	-2.012114	2.083430
O	-5.010917	-1.536624	0.856082
O	1.347702	-0.239511	-0.213590
C	2.318336	-1.141193	0.000385
O	2.256223	-1.980903	0.863346
C	3.444017	-0.980680	-0.983719
C	4.785589	-1.522146	-0.496487
C	5.778397	-1.576556	-1.652948
C	5.336221	-0.687681	0.656165
H	-4.715200	-1.579555	-1.184186
H	-3.654974	0.456890	0.766601
H	-3.555563	-0.672454	-2.807206
H	-1.952514	0.044501	-2.934708
H	-2.633015	-2.556363	-1.261914
H	-3.352720	-3.359420	0.127864
H	-3.365691	1.010380	-3.305178
H	-1.315834	1.024443	1.299266
H	0.441290	-0.636290	1.596672
H	-0.826611	-1.246979	-1.110452
H	1.067228	1.278849	3.110612
H	1.816195	2.915557	2.672955
H	-2.715342	2.464615	-0.007543
H	-0.424714	1.126350	-1.516589
H	-2.408960	2.688130	-1.729524
H	-2.187585	-3.042081	2.364731
H	-2.796296	-1.402950	2.430078
H	-1.063628	-1.705175	2.621713
H	-5.374869	-2.428049	0.845297
H	3.128766	-1.513251	-1.886498
H	3.522374	0.072069	-1.260575

H	4.620141	-2.539969	-0.135657
H	6.737349	-1.977408	-1.321393
H	5.411450	-2.208415	-2.463678
H	5.957951	-0.578261	-2.060037
H	6.281903	-1.097874	1.013754
H	4.646104	-0.659339	1.500267
H	5.519295	0.340399	0.333178

CAM-B3LYP energy = -1154.93113404 a.u.

(2*R*,6*R*,7*S*,8*R*)-**1**, Conf. C

C	-3.829573	-1.778339	-0.319753
C	-3.505830	-0.313216	-0.411562
C	-2.924217	0.328171	-1.418404
C	-2.653324	-0.281228	-2.762677
C	-1.568242	-1.877522	0.837148
C	-2.563741	-2.623758	-0.011523
C	-0.879128	1.667010	-0.835267
C	-0.558639	1.264348	0.626767
C	0.192304	-0.068311	0.784791
C	-0.609730	-1.194586	0.218855
O	-0.333140	3.003374	-1.013504
C	0.292168	3.462560	0.078871
C	0.198218	2.449776	1.157109
O	0.820419	4.543443	0.104588
C	0.714081	2.650378	2.356936
C	-2.364331	1.714270	-1.206218
C	-1.856004	-1.807504	2.306105
O	-4.768988	-2.020019	0.731943
O	1.436885	0.072426	0.065562
C	2.490242	-0.649609	0.478040
O	2.439964	-1.410200	1.412106
C	3.715728	-0.345972	-0.339316
C	4.772073	-1.447890	-0.324669
C	4.281650	-2.699687	-1.046174
C	6.068928	-0.935656	-0.942731
H	-4.249637	-2.149410	-1.258114
H	-3.647043	0.219162	0.525293
H	-3.056925	0.357106	-3.552002
H	-3.088973	-1.272032	-2.872008
H	-2.102184	-2.883134	-0.964466
H	-2.898165	-3.549329	0.460160
H	-1.580121	-0.368694	-2.952650
H	-1.492172	1.149256	1.178127
H	0.420284	-0.216254	1.837998
H	-0.500802	-1.316395	-0.853925
H	0.637750	1.915368	3.147778
H	1.234410	3.573743	2.576678
H	-2.907870	2.239534	-0.418837
H	-0.333443	1.040069	-1.537774
H	-2.452665	2.303559	-2.120958
H	-2.802890	-1.293659	2.487564
H	-1.075792	-1.300757	2.870695
H	-1.969540	-2.815202	2.711400
H	-5.561972	-1.501957	0.555918
H	3.407667	-0.111703	-1.359853
H	4.130144	0.578432	0.074774

H	4.966390	-1.706653	0.718830
H	5.035166	-3.487891	-1.008703
H	3.367990	-3.092158	-0.598457
H	4.078262	-2.482675	-2.098073
H	6.838934	-1.708202	-0.921911
H	6.450200	-0.065473	-0.405533
H	5.916077	-0.646248	-1.985492

CAM-B3LYP energy = -1154.93099040 a.u.

(2*R*,6*R*,7*S*,8*R*)-**1**, Conf. D

C	-3.845550	-1.761654	-0.311850
C	-3.520507	-0.301799	-0.418587
C	-2.915253	0.321686	-1.421849
C	-2.622320	-0.307706	-2.752399
C	-1.568989	-1.875044	0.820573
C	-2.566266	-2.610072	-0.033692
C	-0.875608	1.671137	-0.842840
C	-0.560672	1.268270	0.620145
C	0.189829	-0.064338	0.780172
C	-0.609901	-1.188296	0.207254
O	-0.336150	3.010879	-1.016370
C	0.284651	3.470009	0.078490
C	0.192756	2.454171	1.154162
O	0.808235	4.553105	0.108120
C	0.707530	2.653157	2.354718
C	-2.358968	1.710718	-1.221048
C	-1.855773	-1.815202	2.290256
O	-4.800066	-1.881838	0.744923
O	1.438397	0.077331	0.067914
C	2.487707	-0.649347	0.482203
O	2.431100	-1.413281	1.413236
C	3.717700	-0.346709	-0.328773
C	4.769407	-1.453095	-0.315313
C	4.276234	-2.699741	-1.043835
C	6.070442	-0.943661	-0.926855
H	-4.291042	-2.137473	-1.236935
H	-3.688570	0.243227	0.505599
H	-3.015396	0.318020	-3.557038
H	-3.056433	-1.300160	-2.853189
H	-2.114078	-2.845103	-0.998024
H	-2.878081	-3.554881	0.418681
H	-1.546658	-0.397340	-2.926421
H	-1.496864	1.152060	1.166964
H	0.411790	-0.214547	1.834330
H	-0.500431	-1.303056	-0.866084
H	0.633126	1.915834	3.143581
H	1.225030	3.577487	2.577068
H	-2.906970	2.242157	-0.440917
H	-0.322530	1.048346	-1.543279
H	-2.444589	2.291300	-2.141710
H	-2.794559	-1.288738	2.477058
H	-1.067573	-1.325617	2.858901
H	-1.983558	-2.824939	2.686229
H	-5.057710	-2.806525	0.821196
H	3.414712	-0.106279	-1.349390
H	4.134342	0.573920	0.091422

H	4.959170	-1.717134	0.727696
H	5.026345	-3.491229	-1.007228
H	3.359438	-3.090337	-0.600903
H	4.077309	-2.477379	-2.095474
H	6.837235	-1.719408	-0.906491
H	6.453374	-0.077196	-0.384858
H	5.922341	-0.649487	-1.968962

CAM-B3LYP energy = -1154.93098183 a.u.

(2*R*,6*R*,7*S*,8*R*)-**1**, Conf. E

C	-4.321782	-0.613522	-0.399347
C	-3.506545	0.634261	-0.204554
C	-2.776424	1.275606	-1.109545
C	-2.792374	0.968807	-2.578380
C	-2.198967	-1.746999	0.418608
C	-3.423215	-1.878890	-0.448774
C	-0.369459	1.662116	-0.502027
C	-0.154150	0.829633	0.787203
C	0.082752	-0.672659	0.557968
C	-1.084390	-1.288501	-0.142966
O	0.608292	2.737797	-0.455011
C	1.398478	2.678958	0.625253
C	0.993132	1.526115	1.464386
O	2.277000	3.478597	0.817307
C	1.591840	1.245481	2.608310
C	-1.754238	2.294883	-0.666817
C	-2.390545	-1.960626	1.889280
O	-5.241932	-0.786409	0.682461
O	1.270716	-0.783422	-0.255774
C	2.014806	-1.893765	-0.143437
O	1.745365	-2.790804	0.617475
C	3.197957	-1.865720	-1.068646
C	4.167913	-0.705571	-0.803516
C	4.738871	-0.767902	0.609277
C	5.280278	-0.715450	-1.846003
H	-4.884073	-0.575437	-1.335895
H	-3.413269	0.930400	0.836911
H	-2.977071	1.881383	-3.149762
H	-3.552969	0.239612	-2.848821
H	-3.116018	-2.022367	-1.484818
H	-4.043904	-2.730136	-0.163617
H	-1.827290	0.581449	-2.915916
H	-1.045429	0.900885	1.411070
H	0.281138	-1.145112	1.517220
H	-1.064562	-1.164912	-1.221064
H	1.291717	0.409873	3.227242
H	2.413123	1.858982	2.955700
H	-2.045525	2.757028	0.278096
H	-0.111340	1.081329	-1.385184
H	-1.662341	3.088797	-1.410601
H	-3.092882	-1.228862	2.295187
H	-1.462482	-1.898393	2.454088
H	-2.832058	-2.943415	2.067743
H	-5.811228	-0.010257	0.723857
H	3.714173	-2.820051	-0.964559
H	2.818477	-1.795745	-2.090493

H	3.607745	0.226500	-0.908633
H	5.419293	0.066167	0.786284
H	3.955676	-0.720706	1.368167
H	5.297547	-1.694942	0.760346
H	5.964027	0.120020	-1.689121
H	4.878610	-0.636997	-2.857605
H	5.861768	-1.638687	-1.785137

CAM-B3LYP energy = -1154.93063255 a.u.

(2*R*,6*R*,7*S*,8*R*)-**1**, Conf. F

C	4.333863	-0.592459	0.376256
C	3.518777	0.652838	0.195900
C	2.779897	1.275500	1.105849
C	2.792814	0.949951	2.570780
C	2.199623	-1.740835	-0.399373
C	3.425199	-1.856928	0.466395
C	0.370637	1.667522	0.509543
C	0.154401	0.834013	-0.778641
C	-0.081473	-0.668118	-0.547426
C	1.084825	-1.278472	0.158894
O	-0.605408	2.744812	0.460956
C	-1.396275	2.684932	-0.618718
C	-0.992827	1.530077	-1.456140
O	-2.273986	3.485272	-0.811728
C	-1.592643	1.248099	-2.599144
C	1.756450	2.297871	0.673538
C	2.387766	-1.969125	-1.868482
O	5.219336	-0.656319	-0.743524
O	-1.272376	-0.779946	0.261923
C	-2.012084	-1.893139	0.149004
O	-1.735328	-2.791662	-0.607561
C	-3.200955	-1.866092	1.066799
C	-4.175495	-0.712861	0.788133
C	-4.735552	-0.785941	-0.628491
C	-5.295556	-0.723630	1.822350
H	4.925358	-0.549893	1.294852
H	3.437805	0.965579	-0.840956
H	3.553262	0.217383	2.832638
H	1.827365	0.559837	2.903926
H	3.123139	-1.967974	1.508485
H	4.031355	-2.729228	0.208426
H	2.978838	1.855180	3.153390
H	1.045843	0.904536	-1.402413
H	-0.275065	-1.142997	-1.506416
H	1.065051	-1.144718	1.235644
H	-1.293747	0.411111	-3.216816
H	-2.413547	1.861823	-2.947031
H	2.044611	2.765507	-0.269631
H	0.111262	1.088278	1.393382
H	1.667635	3.087524	1.422336
H	1.454093	-1.938798	-2.426670
H	2.853195	-2.942462	-2.037535
H	3.066893	-1.224250	-2.289675
H	5.782027	-1.431553	-0.645557
H	-3.711345	-2.823793	0.965108
H	-2.828606	-1.787661	2.090632

H	-3.621374	0.222910	0.892400
H	-5.419583	0.043136	-0.814801
H	-3.947102	-0.737931	-1.381881
H	-5.287611	-1.717050	-0.778868
H	-5.982640	0.107263	1.656080
H	-4.901882	-0.637874	2.836494
H	-5.871479	-1.650362	1.761780

CAM-B3LYP energy = -1154.93061277 a.u.

(2*R*,6*R*,7*S*,8*R*)-**1**, Conf. G

C	3.846330	-1.363476	0.415189
C	3.316238	0.026356	0.630752
C	2.537514	0.453262	1.618041
C	2.197107	-0.357142	2.834358
C	1.771180	-1.581361	-1.035740
C	2.752792	-2.307841	-0.154452
C	0.406280	1.593532	0.930136
C	0.309905	1.354088	-0.598459
C	-0.220848	-0.029619	-1.009041
C	0.659280	-1.113319	-0.476739
O	-0.336025	2.816443	1.193458
C	-0.895899	3.335425	0.092553
C	-0.545635	2.494083	-1.076413
O	-1.562893	4.336421	0.127235
C	-0.956556	2.784865	-2.298096
C	1.819073	1.775056	1.492455
C	2.220656	-1.276461	-2.432110
O	4.927582	-1.349297	-0.521683
O	-1.547856	-0.144850	-0.450836
C	-2.441153	-0.914409	-1.090803
O	-2.195311	-1.485438	-2.124973
C	-3.752265	-0.965230	-0.359520
C	-3.658158	-1.632313	1.020752
C	-5.012859	-1.566977	1.717071
C	-3.164430	-3.071430	0.915120
H	4.199625	-1.800742	1.352686
H	3.491947	0.691091	-0.210868
H	2.762519	-1.284609	2.893905
H	1.134770	-0.614854	2.857627
H	2.221744	-2.743520	0.691984
H	3.263051	-3.116267	-0.681018
H	2.396185	0.221976	3.738918
H	1.305528	1.435386	-1.035564
H	-0.301984	-0.065079	-2.092947
H	0.441688	-1.392829	0.549116
H	-0.696893	2.176819	-3.154873
H	-1.575763	3.656097	-2.468708
H	2.370101	2.460226	0.845768
H	-0.122313	0.815621	1.477584
H	1.720859	2.248352	2.471358
H	3.107273	-0.638370	-2.418082
H	1.454382	-0.788288	-3.031077
H	2.512780	-2.198890	-2.938518
H	5.614989	-0.765127	-0.183257
H	-4.113635	0.058551	-0.243223
H	-4.457224	-1.505813	-0.991016

H	-2.939463	-1.063041	1.615215
H	-4.957231	-2.009999	2.712495
H	-5.355971	-0.536702	1.824894
H	-5.767921	-2.115993	1.149066
H	-3.096686	-3.527630	1.903670
H	-2.176711	-3.132071	0.454606
H	-3.850935	-3.673066	0.314343

CAM-B3LYP energy = -1154.93052764 a.u.

(2*R*,6*R*,7*S*,8*R*)-**1**, Conf. H

C	3.863047	-1.345046	0.412855
C	3.332690	0.039457	0.635221
C	2.540396	0.454395	1.615913
C	2.191965	-0.366113	2.823329
C	1.766691	-1.590136	-1.008258
C	2.749076	-2.300081	-0.116277
C	0.409318	1.599220	0.932584
C	0.314053	1.353370	-0.594940
C	-0.219200	-0.031119	-0.999575
C	0.656179	-1.111937	-0.454782
O	-0.327564	2.826948	1.189266
C	-0.884501	3.342630	0.085407
C	-0.537340	2.493750	-1.079125
O	-1.547197	4.346698	0.114463
C	-0.947231	2.779548	-2.302328
C	1.822152	1.776738	1.495580
C	2.212828	-1.307080	-2.410480
O	4.937583	-1.216944	-0.520389
O	-1.549364	-0.139975	-0.447481
C	-2.441384	-0.909647	-1.089034
O	-2.192251	-1.484281	-2.120467
C	-3.755298	-0.956863	-0.362681
C	-3.668420	-1.631299	1.014617
C	-5.025225	-1.564069	1.706622
C	-3.179965	-3.071819	0.903684
H	4.248256	-1.776062	1.341032
H	3.524531	0.711181	-0.196251
H	2.386563	0.205798	3.733497
H	2.758573	-1.293220	2.878954
H	2.224523	-2.702383	0.751210
H	3.233859	-3.138717	-0.622618
H	1.129812	-0.624841	2.840038
H	1.310847	1.429587	-1.030501
H	-0.294565	-0.073539	-2.083614
H	0.438400	-1.376800	0.574739
H	-0.689901	2.165900	-3.155796
H	-1.563130	3.652204	-2.477600
H	2.373524	2.463881	0.851273
H	-0.123827	0.826477	1.482936
H	1.724359	2.246726	2.476197
H	3.083747	-0.647644	-2.410479
H	1.435898	-0.851231	-3.021168
H	2.526842	-2.233775	-2.895514
H	5.320509	-2.087981	-0.668572
H	-4.112112	0.067984	-0.242300
H	-4.460827	-1.491207	-0.998831

H	-2.949522	-1.067757	1.614311
H	-4.974688	-2.012093	2.700075
H	-5.364756	-0.533017	1.818230
H	-5.780406	-2.107450	1.133381
H	-3.118334	-3.533250	1.890203
H	-2.190537	-3.134081	0.447101
H	-3.866283	-3.667652	0.296936

CAM-B3LYP energy = -1154.93051689 a.u.

(2*R*,6*R*,7*S*,8*R*)-**1**, Conf. I

C	-4.143994	-1.411790	-0.276207
C	-3.653393	0.008926	-0.235428
C	-3.066002	0.687852	-1.213606
C	-2.975251	0.208173	-2.632774
C	-1.802401	-1.904121	0.612387
C	-2.954449	-2.421518	-0.206538
C	-0.828948	1.710140	-0.701580
C	-0.431064	1.097506	0.665191
C	0.159269	-0.320905	0.595882
C	-0.818780	-1.269081	-0.017466
O	-0.142947	2.989706	-0.784476
C	0.630358	3.237904	0.281214
C	0.510725	2.116425	1.243266
O	1.286941	4.242027	0.373032
C	1.153930	2.110014	2.397397
C	-2.324287	1.966649	-0.909403
C	-1.944869	-1.960555	2.105613
O	-5.111256	-1.651843	0.746445
O	1.351904	-0.234158	-0.212782
C	2.323689	-1.135355	0.001818
O	2.260139	-1.975776	0.863761
C	3.450678	-0.972257	-0.980032
C	4.790490	-1.519232	-0.494048
C	5.784140	-1.570683	-1.649933
C	5.342278	-0.691540	0.662911
H	-4.684006	-1.614390	-1.199833
H	-3.667964	0.455205	0.756349
H	-1.939440	0.021053	-2.927788
H	-3.357571	0.974506	-3.310876
H	-2.614969	-2.598123	-1.227721
H	-3.348938	-3.363211	0.179477
H	-3.537571	-0.706866	-2.805508
H	-1.318752	1.022691	1.294110
H	0.447260	-0.626465	1.599504
H	-0.808241	-1.276967	-1.102564
H	1.061257	1.297175	3.105966
H	1.801189	2.936204	2.661968
H	-2.725687	2.450249	-0.017041
H	-0.428532	1.117994	-1.521975
H	-2.421193	2.668611	-1.739960
H	-2.335461	-2.930991	2.414784
H	-2.645961	-1.202863	2.471640
H	-1.001728	-1.794686	2.623407
H	-4.724933	-1.454414	1.606364
H	3.135243	-1.499240	-1.885999
H	3.531558	0.081784	-1.251246

H	4.622279	-2.538334	-0.138151
H	6.741755	-1.975557	-1.319418
H	5.416318	-2.197778	-2.463951
H	5.966602	-0.570932	-2.052108
H	6.286648	-1.105812	1.019245
H	4.651637	-0.665351	1.506637
H	5.528242	0.337563	0.344864

CAM-B3LYP energy = -1154.92901160 a.u.

(2*R*,6*R*,7*S*,8*R*)-**1**, Conf. J

C	-3.852891	-1.773566	-0.315774
C	-3.527267	-0.309487	-0.422343
C	-2.913726	0.310869	-1.423070
C	-2.614584	-0.323791	-2.749633
C	-1.576308	-1.864849	0.837313
C	-2.575500	-2.615797	-0.001225
C	-0.874502	1.661673	-0.847694
C	-0.559388	1.262246	0.616224
C	0.192181	-0.069632	0.778507
C	-0.609667	-1.197113	0.215303
O	-0.335505	3.000646	-1.024591
C	0.285992	3.462519	0.068979
C	0.194506	2.449325	1.147146
O	0.809435	4.545586	0.095704
C	0.709471	2.651140	2.347111
C	-2.357910	1.700417	-1.225909
C	-1.861966	-1.753434	2.306670
O	-4.893272	-2.000985	0.635641
O	1.437500	0.069568	0.062264
C	2.489164	-0.654386	0.477147
O	2.434260	-1.416392	1.409627
C	3.717061	-0.349710	-0.335890
C	4.779707	-1.445209	-0.309075
C	4.301000	-2.703919	-1.026428
C	6.076886	-0.928436	-0.922724
H	-4.265361	-2.152768	-1.249520
H	-3.698185	0.250991	0.493971
H	-1.538157	-0.406377	-2.921360
H	-3.010690	0.295712	-3.557517
H	-2.115148	-2.879154	-0.954179
H	-2.905213	-3.540874	0.474925
H	-3.041273	-1.319669	-2.847347
H	-1.495258	1.148462	1.164152
H	0.416483	-0.215965	1.833139
H	-0.490915	-1.335475	-0.854162
H	0.635194	1.915881	3.137902
H	1.227139	3.575961	2.566978
H	-2.906144	2.234294	-0.447590
H	-0.321528	1.036792	-1.546383
H	-2.443462	2.278013	-2.148389
H	-2.668555	-1.040131	2.506542
H	-0.996854	-1.420929	2.877865
H	-2.182402	-2.716120	2.707173
H	-4.636745	-1.632843	1.487962
H	3.412432	-0.123955	-1.359436
H	4.123855	0.580088	0.073637

H	4.969795	-1.697030	0.736899
H	5.059195	-3.487169	-0.980508
H	3.387333	-3.099620	-0.581640
H	4.102200	-2.493832	-2.080602
H	6.851333	-1.696242	-0.893606
H	6.449982	-0.053166	-0.388064
H	5.928049	-0.645431	-1.967816

CAM-B3LYP energy = -1154.92887689 a.u.

(2*R*,6*R*,7*S*,8*S*)-**1**, Conf. A

C	3.710189	-2.141560	-0.293006
C	3.505241	-0.747972	-0.791810
C	3.603865	0.374535	-0.086526
C	4.269353	0.504738	1.253977
C	1.297653	-1.961321	0.444206
C	2.334871	-2.845217	-0.204229
C	1.534701	1.748919	0.150347
C	0.266286	1.258513	-0.607111
C	-0.314826	-0.031542	0.013562
C	0.545385	-1.198540	-0.342489
O	1.310830	3.154163	0.447065
C	0.083021	3.567566	0.112135
C	-0.639459	2.462328	-0.561761
O	-0.296404	4.686926	0.339568
C	-1.846697	2.655072	-1.067103
C	2.894959	1.621254	-0.556904
C	1.306757	-1.916424	1.941291
O	4.499559	-2.944133	-1.175422
O	-1.623632	-0.222581	-0.575119
C	-2.527436	-0.920865	0.128554
O	-2.324011	-1.324970	1.246477
C	-3.783306	-1.153354	-0.665948
C	-5.038633	-1.338056	0.183397
C	-5.414382	-0.048429	0.906644
C	-6.189793	-1.830853	-0.687399
H	4.172003	-2.136849	0.695398
H	3.032377	-0.696536	-1.769018
H	4.733359	-0.416757	1.595599
H	3.570253	0.836438	2.025240
H	2.460928	-3.785323	0.335530
H	2.036426	-3.082027	-1.226625
H	5.047900	1.269356	1.194677
H	0.520928	1.040891	-1.645568
H	-0.441016	0.093134	1.086219
H	0.631712	-1.337716	-1.416412
H	-2.378276	1.883176	-1.601661
H	-2.324111	3.619720	-0.948564
H	2.756821	1.617808	-1.638462
H	1.586121	1.261675	1.123379
H	3.473428	2.509411	-0.297543
H	1.182981	-2.925721	2.340008
H	0.515939	-1.293086	2.351622
H	2.265006	-1.548501	2.316292
H	5.377212	-2.551494	-1.233132
H	-3.909844	-0.336062	-1.377887

H	-3.596139	-2.052897	-1.260876
H	-4.821597	-2.102202	0.933272
H	-6.296120	-0.197813	1.531583
H	-4.606981	0.303022	1.550208
H	-5.644570	0.742649	0.188312
H	-7.089392	-1.983632	-0.089438
H	-5.944703	-2.776085	-1.174928
H	-6.427883	-1.102561	-1.466736

CAM-B3LYP energy = -1154.92812578 a.u.

(2*R*,6*R*,7*S*,8*S*)-**1**, Conf. B

C	3.179337	-2.612625	-0.356304
C	3.213401	-1.211153	-0.874226
C	3.572781	-0.120783	-0.203952
C	4.338363	-0.105038	1.088443
C	0.895293	-1.957816	0.510600
C	1.703027	-3.036314	-0.169278
C	1.815476	1.619078	0.123506
C	0.436361	1.380900	-0.556916
C	-0.348811	0.233658	0.117622
C	0.251109	-1.081866	-0.253828
O	1.880964	3.040817	0.418954
C	0.740309	3.683333	0.141927
C	-0.215712	2.737553	-0.482028
O	0.596144	4.855089	0.376132
C	-1.388278	3.158005	-0.927498
C	3.083921	1.233618	-0.657489
C	1.009906	-1.884296	2.002249
O	3.743191	-3.562223	-1.265248
O	-1.697872	0.293175	-0.403523
C	-2.688364	-0.179829	0.367527
O	-2.506557	-0.636191	1.468859
C	-4.027101	-0.018653	-0.299994
C	-5.089574	-1.006111	0.176865
C	-6.460752	-0.590404	-0.345729
C	-4.753369	-2.432295	-0.248263
H	3.695547	-2.685770	0.602133
H	2.694569	-1.079404	-1.819995
H	5.243230	0.494385	0.961256
H	4.638886	-1.094372	1.423397
H	1.678975	-3.973467	0.389483
H	1.302978	-3.228875	-1.165850
H	3.768668	0.364942	1.893619
H	0.584495	1.110803	-1.603631
H	-0.391295	0.392572	1.192279
H	0.240317	-1.255398	-1.326204
H	-2.085956	2.501071	-1.423517
H	-1.664232	4.196968	-0.797311
H	2.884214	1.247734	-1.729192
H	1.828531	1.129820	1.096660
H	3.832641	1.999132	-0.447753
H	0.376466	-1.116393	2.439873
H	2.042027	-1.692696	2.306103
H	0.729395	-2.844472	2.440719
H	4.673995	-3.345679	-1.385448
H	-4.345845	1.006761	-0.087922

H	-3.892111	-0.081323	-1.381109
H	-5.110465	-0.968419	1.268437
H	-7.232264	-1.279103	0.001584
H	-6.728368	0.412762	-0.009189
H	-6.478904	-0.594169	-1.438524
H	-5.504784	-3.132981	0.118972
H	-3.784700	-2.751137	0.138407
H	-4.726902	-2.512401	-1.338074

CAM-B3LYP energy = -1154.92801902 a.u.

(2*R*,6*R*,7*S*,8*S*)-**1**, Conf. C

C	3.086698	-2.538496	-0.392632
C	3.007780	-1.177299	-1.004427
C	3.409258	-0.033726	-0.458219
C	4.329591	0.089732	0.722804
C	0.919948	-1.878041	0.737370
C	1.663645	-2.981045	0.024614
C	1.660692	1.690053	-0.018804
C	0.210579	1.366410	-0.481191
C	-0.432161	0.254409	0.377605
C	0.153078	-1.069621	0.012742
O	1.717216	3.131723	0.157445
C	0.526696	3.722329	-0.000413
C	-0.471945	2.708176	-0.415168
O	0.374566	4.904356	0.167695
C	-1.707810	3.063555	-0.725457
C	2.823705	1.273266	-0.935422
C	1.225224	-1.705036	2.193517
O	3.550627	-3.538189	-1.304429
O	-1.843825	0.245739	0.058496
C	-2.691968	-0.212316	0.990589
O	-2.335929	-0.569037	2.087247
C	-4.111972	-0.212482	0.498475
C	-4.345105	-1.108871	-0.725671
C	-3.989044	-2.561662	-0.428082
C	-5.791699	-0.981466	-1.189857
H	3.730411	-2.532388	0.488244
H	2.371219	-1.126872	-1.883841
H	4.705502	-0.866653	1.076452
H	3.848857	0.594193	1.564170
H	1.747234	-3.877026	0.642077
H	1.137745	-3.252681	-0.891885
H	5.189022	0.704980	0.444975
H	0.223071	1.021300	-1.516191
H	-0.325760	0.488870	1.433953
H	0.006751	-1.313722	-1.035657
H	-2.441926	2.352734	-1.072320
H	-2.000740	4.102082	-0.635475
H	2.482145	1.203836	-1.968546
H	1.822998	1.279905	0.977184
H	3.570433	2.067234	-0.883821
H	0.639541	-0.916182	2.659199
H	2.284320	-1.484939	2.348567
H	1.020275	-2.637095	2.724827
H	4.449532	-3.311477	-1.565803
H	-4.741871	-0.535089	1.327464

H	-4.381057	0.817123	0.251894
H	-3.694855	-0.751575	-1.527621
H	-4.153657	-3.186124	-1.307265
H	-2.943511	-2.673481	-0.134688
H	-4.608284	-2.953577	0.382614
H	-5.968177	-1.592569	-2.076165
H	-6.040813	0.051755	-1.437485
H	-6.480767	-1.316521	-0.410729

CAM-B3LYP energy = -1154.92758824 a.u.

(2*R*,6*R*,7*S*,8*S*)-**1**, Conf. D

C	3.717823	-2.140933	-0.295269
C	3.513858	-0.750915	-0.794318
C	3.603554	0.370438	-0.087306
C	4.263311	0.502174	1.255819
C	1.294623	-1.955404	0.442208
C	2.329973	-2.838546	-0.209379
C	1.536894	1.752846	0.146779
C	0.265041	1.266377	-0.607087
C	-0.315890	-0.023324	0.013599
C	0.543496	-1.190380	-0.343624
O	1.320225	3.160335	0.438829
C	0.091863	3.576732	0.109850
C	-0.638006	2.472052	-0.557119
O	-0.282809	4.697877	0.336463
C	-1.848608	2.666811	-1.053581
C	2.895488	1.616257	-0.561622
C	1.304652	-1.912606	1.939342
O	4.565547	-2.848500	-1.201643
O	-1.625255	-0.213763	-0.574289
C	-2.527313	-0.915752	0.127963
O	-2.322455	-1.322466	1.244669
C	-3.782913	-1.148658	-0.666858
C	-5.036457	-1.346506	0.182073
C	-5.419010	-0.064111	0.914536
C	-6.185410	-1.839829	-0.691366
H	4.176058	-2.134931	0.694548
H	3.046627	-0.700481	-1.774024
H	4.727645	-0.418738	1.598592
H	3.561552	0.833401	2.024836
H	2.445129	-3.782603	0.328865
H	2.031303	-3.069247	-1.233051
H	5.041453	1.267382	1.198745
H	0.515974	1.049916	-1.646752
H	-0.441208	0.101058	1.086424
H	0.629876	-1.328297	-1.417702
H	-2.386055	1.895411	-1.582919
H	-2.322752	3.632799	-0.932891
H	2.755686	1.608046	-1.642939
H	1.586802	1.268789	1.121480
H	3.477165	2.503945	-0.307827
H	0.515278	-1.288092	2.350657
H	2.263725	-1.546947	2.314330
H	1.178883	-2.922029	2.337184
H	4.647317	-3.759602	-0.898320
H	-3.914384	-0.326497	-1.372310

H	-3.591534	-2.042377	-1.269198
H	-4.814525	-2.114616	0.926446
H	-6.299643	-0.222691	1.538767
H	-4.613218	0.287192	1.560174
H	-5.653818	0.730686	0.201822
H	-7.083830	-2.001495	-0.093974
H	-5.935407	-2.780450	-1.185288
H	-6.427928	-1.107729	-1.465753

CAM-B3LYP energy = -1154.92725217 a.u.

(2*R*,6*R*,7*S*,8*S*)-**1**, Conf. E

C	3.717456	-2.144312	-0.289251
C	3.510259	-0.750439	-0.789355
C	3.606651	0.373375	-0.086946
C	4.271280	0.504685	1.253651
C	1.296310	-1.959222	0.447034
C	2.333031	-2.845492	-0.198956
C	1.537319	1.748639	0.148734
C	0.268039	1.259431	-0.607992
C	-0.315090	-0.028912	0.014366
C	0.542760	-1.198095	-0.340205
O	1.315172	3.154201	0.445132
C	0.088034	3.569208	0.109690
C	-0.635847	2.464678	-0.563875
O	-0.289886	4.689193	0.336470
C	-1.842618	2.658882	-1.069751
C	2.897029	1.619051	-0.559174
C	1.310200	-1.908841	1.943969
O	4.606268	-2.919261	-1.093814
O	-1.624196	-0.218489	-0.573921
C	-2.528456	-0.916181	0.129933
O	-2.325004	-1.320488	1.247732
C	-3.784416	-1.147597	-0.664720
C	-5.038868	-1.339542	0.184226
C	-5.417870	-0.054219	0.913398
C	-6.188960	-1.831496	-0.688486
H	4.178341	-2.138258	0.695168
H	3.033133	-0.695828	-1.765559
H	5.055176	1.263593	1.191735
H	4.730139	-0.418080	1.598916
H	2.458360	-3.785162	0.341527
H	2.024075	-3.086117	-1.218907
H	3.573881	0.844253	2.023000
H	0.522135	1.040182	-1.646244
H	-0.440843	0.097341	1.086902
H	0.625816	-1.340649	-1.414034
H	-2.375199	1.887422	-1.603951
H	-2.318638	3.624311	-0.951990
H	2.758211	1.613777	-1.640664
H	1.588725	1.261718	1.121910
H	3.475990	2.507555	-0.302232
H	0.520773	-1.283718	2.354201
H	2.269331	-1.539264	2.315004
H	1.187494	-2.916486	2.347099
H	4.264345	-2.946085	-1.995041
H	-3.913222	-0.326896	-1.372356

H	-3.595465	-2.043484	-1.264596
H	-4.819561	-2.106495	0.930566
H	-6.299156	-0.208718	1.537728
H	-4.611279	0.296328	1.558443
H	-5.650166	0.739506	0.198670
H	-7.088050	-1.989164	-0.091038
H	-5.941564	-2.773974	-1.180174
H	-6.429067	-1.100438	-1.464611

CAM-B3LYP energy = -1154.92723616 a.u.

(2*R*,6*R*,7*S*,8*S*)-**1**, Conf. F

C	3.185142	-2.616443	-0.355451
C	3.213824	-1.214427	-0.875225
C	3.575642	-0.122057	-0.210672
C	4.347700	-0.103672	1.077501
C	0.897624	-1.953584	0.520542
C	1.701850	-3.036417	-0.156749
C	1.817036	1.616103	0.120514
C	0.436463	1.378676	-0.557104
C	-0.350005	0.235136	0.122283
C	0.246922	-1.083001	-0.244678
O	1.883234	3.037466	0.417463
C	0.743537	3.681315	0.139555
C	-0.213188	2.736550	-0.484860
O	0.600543	4.853253	0.373505
C	-1.384538	3.158269	-0.932307
C	3.083237	1.231094	-0.664350
C	1.026339	-1.868709	2.010482
O	3.852505	-3.557474	-1.196376
O	-1.698898	0.295147	-0.398735
C	-2.689924	-0.176468	0.372498
O	-2.508625	-0.632042	1.474219
C	-4.028355	-0.014954	-0.295547
C	-5.089974	-1.005154	0.177537
C	-6.460963	-0.590351	-0.346308
C	-4.750925	-2.429834	-0.250356
H	3.705971	-2.687350	0.596085
H	2.685644	-1.078411	-1.816499
H	5.259310	0.483110	0.939823
H	4.639110	-1.093207	1.419999
H	1.681072	-3.970775	0.406634
H	1.284909	-3.235012	-1.146694
H	3.787964	0.381233	1.880785
H	0.582176	1.105044	-1.603228
H	-0.391587	0.398082	1.196407
H	0.227252	-1.263738	-1.315823
H	-2.082616	2.501732	-1.428330
H	-1.659152	4.197771	-0.803635
H	2.879948	1.243796	-1.735413
H	1.832561	1.125677	1.093052
H	3.831940	1.997634	-0.458562
H	0.751630	-2.825732	2.459291
H	0.396122	-1.098164	2.448137
H	2.060842	-1.672679	2.303127
H	3.452695	-3.528242	-2.073394
H	-4.348532	1.009419	-0.080635

H	-3.892539	-0.074360	-1.376734
H	-5.112617	-0.969988	1.269158
H	-7.231995	-1.280974	-0.001770
H	-6.730622	0.411668	-0.008003
H	-6.477343	-0.591724	-1.439141
H	-5.502033	-3.132458	0.113780
H	-3.782554	-2.748258	0.137436
H	-4.722300	-2.507319	-1.340308

CAM-B3LYP energy = -1154.92713667 a.u.

(2*R*,6*R*,7*S*,8*S*)-**1**, Conf. G

C	3.193360	-2.609289	-0.361197
C	3.226895	-1.210200	-0.875679
C	3.570009	-0.120239	-0.197636
C	4.321774	-0.104048	1.102861
C	0.895871	-1.956016	0.501853
C	1.706293	-3.028977	-0.182820
C	1.814572	1.626340	0.122544
C	0.435021	1.388186	-0.556606
C	-0.347014	0.237587	0.115238
C	0.255202	-1.075512	-0.260598
O	1.883927	3.049867	0.409061
C	0.741543	3.691542	0.137592
C	-0.218530	2.744020	-0.477615
O	0.598856	4.864027	0.369138
C	-1.394758	3.163293	-0.914502
C	3.082400	1.233334	-0.655041
C	1.005197	-1.890942	1.994274
O	3.836425	-3.473404	-1.299594
O	-1.696928	0.295600	-0.404250
C	-2.685431	-0.181642	0.366703
O	-2.501370	-0.640032	1.466836
C	-4.025225	-0.022265	-0.299151
C	-5.086062	-1.010649	0.179355
C	-6.458329	-0.596461	-0.341631
C	-4.749086	-2.436670	-0.245693
H	3.702926	-2.681865	0.600589
H	2.719715	-1.079642	-1.827688
H	3.741604	0.358806	1.904644
H	5.224200	0.501268	0.986801
H	1.672145	-3.970257	0.371307
H	1.310329	-3.212789	-1.182610
H	4.625681	-1.092927	1.436106
H	0.582558	1.121723	-1.604397
H	-0.388698	0.393286	1.190407
H	0.248925	-1.243494	-1.333883
H	-2.095786	2.505630	-1.404719
H	-1.670162	4.202258	-0.783170
H	2.884120	1.244375	-1.727059
H	1.825434	1.143306	1.098806
H	3.832847	1.997771	-0.447316
H	2.036056	-1.700373	2.302885
H	0.723431	-2.853500	2.426746
H	0.369648	-1.125730	2.433510
H	3.763635	-4.380389	-0.981931
H	-4.344864	1.002887	-0.087151

H	-3.891458	-0.085187	-1.380415
H	-5.105647	-0.972620	1.270940
H	-7.228730	-1.285758	0.006965
H	-6.726471	0.406579	-0.005138
H	-6.477911	-0.600670	-1.434399
H	-5.499439	-3.137879	0.122727
H	-3.779663	-2.754478	0.139923
H	-4.723912	-2.517149	-1.335507

CAM-B3LYP energy = -1154.92713017 a.u.

(2*R*,6*R*,7*S*,8*S*)-**1**, Conf. H

C	3.099853	-2.532932	-0.399629
C	3.015896	-1.175920	-1.011593
C	3.407034	-0.030751	-0.462871
C	4.324282	0.097933	0.719953
C	0.924394	-1.871706	0.737495
C	1.667910	-2.972150	0.021296
C	1.657453	1.694678	-0.023679
C	0.205471	1.371676	-0.479957
C	-0.432573	0.258278	0.379879
C	0.155231	-1.064194	0.014022
O	1.716425	3.137018	0.147233
C	0.525098	3.727782	-0.003246
C	-0.476989	2.713239	-0.408931
O	0.374562	4.910215	0.163559
C	-1.715548	3.068444	-0.708522
C	2.816806	1.273282	-0.942536
C	1.232480	-1.698600	2.193021
O	3.629822	-3.450612	-1.357709
O	-1.844764	0.246090	0.062745
C	-2.689960	-0.219200	0.993880
O	-2.331154	-0.579196	2.088576
C	-4.110573	-0.222738	0.503434
C	-4.341232	-1.112394	-0.726075
C	-3.979687	-2.565663	-0.437623
C	-5.788537	-0.987441	-1.188732
H	3.743443	-2.522783	0.481012
H	2.382474	-1.129572	-1.893211
H	5.181116	0.717250	0.443163
H	4.704851	-0.856675	1.073442
H	1.745904	-3.869184	0.640684
H	1.140616	-3.241267	-0.895115
H	3.840137	0.599958	1.560790
H	0.213572	1.028197	-1.515580
H	-0.325105	0.493087	1.436084
H	0.007947	-1.308406	-1.034214
H	-2.452653	2.357492	-1.048653
H	-2.007555	4.107041	-0.616421
H	2.471307	1.198898	-1.974006
H	1.822105	1.287913	0.973309
H	3.563229	2.067970	-0.897853
H	2.291651	-1.477575	2.345986
H	1.029098	-2.630548	2.725171
H	0.647189	-0.909983	2.659583
H	3.629937	-4.333175	-0.970569
H	-4.738047	-0.553202	1.331175

H	-4.384550	0.807198	0.263677
H	-3.692765	-0.747794	-1.526163
H	-4.142077	-3.185236	-1.320679
H	-2.933689	-2.675346	-0.145061
H	-4.597346	-2.964978	0.370669
H	-5.963248	-1.593766	-2.078667
H	-6.041506	0.046352	-1.429967
H	-6.475996	-1.329725	-0.411332

CAM-B3LYP energy = -1154.92673130 a.u.

(2*R*,6*R*,7*S*,8*S*)-**1**, Conf. I

C	3.102489	-2.534941	-0.395428
C	3.017922	-1.170618	-1.002144
C	3.414458	-0.026842	-0.454215
C	4.331986	0.095908	0.728754
C	0.924731	-1.879483	0.733585
C	1.672438	-2.980249	0.021485
C	1.659579	1.691557	-0.016286
C	0.211753	1.363565	-0.482997
C	-0.430762	0.251020	0.375583
C	0.154663	-1.072802	0.010198
O	1.710950	3.133205	0.161513
C	0.519659	3.720622	-0.002168
C	-0.474321	2.703713	-0.421307
O	0.363559	4.902271	0.164875
C	-1.709438	3.055770	-0.738257
C	2.826250	1.279512	-0.930145
C	1.233946	-1.704245	2.188711
O	3.687221	-3.512430	-1.255995
O	-1.842548	0.241990	0.057600
C	-2.690251	-0.212616	0.991857
O	-2.333281	-0.567984	2.088634
C	-4.110946	-0.210788	0.501808
C	-4.348182	-1.110653	-0.718991
C	-3.995300	-2.563327	-0.417070
C	-5.795201	-0.981174	-1.181283
H	3.743669	-2.529296	0.482357
H	2.379029	-1.113476	-1.880898
H	4.702875	-0.861088	1.086171
H	3.851943	0.606220	1.566969
H	1.758351	-3.876670	0.637824
H	1.138290	-3.253850	-0.891460
H	5.195656	0.705104	0.450748
H	0.228298	1.017038	-1.517478
H	-0.323738	0.485062	1.431936
H	0.006046	-1.317652	-1.037801
H	-2.440083	2.342870	-1.088208
H	-2.005411	4.093638	-0.650642
H	2.488142	1.211747	-1.964554
H	1.820704	1.280998	0.979716
H	3.570853	2.075185	-0.874919
H	1.034840	-2.636674	2.721528
H	0.646168	-0.917596	2.655434
H	2.292343	-1.478826	2.340755
H	3.175786	-3.551784	-2.072515
H	-4.740464	-0.529099	1.332733

H	-4.377716	0.818675	0.252194
H	-3.698323	-0.757636	-1.523168
H	-4.162898	-3.190370	-1.293852
H	-2.949604	-2.676774	-0.124869
H	-4.614222	-2.950915	0.395934
H	-5.974721	-1.594928	-2.065144
H	-6.042070	0.051809	-1.432112
H	-6.483823	-1.311708	-0.399838

CAM-B3LYP energy = -1154.92672677 a.u.

(2*R*,6*R*,7*S*,8*S*)-**1**, Conf. J

C	3.982461	-1.659065	-0.382189
C	3.531464	-0.313944	-0.853723
C	3.485125	0.807372	-0.140982
C	4.181307	1.034922	1.170590
C	1.603148	-1.878413	0.447156
C	2.747506	-2.580711	-0.241685
C	1.223293	1.792968	0.180067
C	0.048439	1.102934	-0.571832
C	-0.325485	-0.250024	0.077431
C	0.692874	-1.272746	-0.308397
O	0.753908	3.122565	0.533494
C	-0.518913	3.338148	0.178889
C	-1.027315	2.155585	-0.555427
O	-1.086060	4.368478	0.434307
C	-2.210779	2.175672	-1.145005
C	2.564442	1.928246	-0.560790
C	1.672301	-1.804591	1.941579
O	4.852557	-2.315840	-1.308318
O	-1.615117	-0.637208	-0.453013
C	-2.333848	-1.518506	0.260446
O	-1.971754	-1.949967	1.327886
C	-3.627480	-1.869137	-0.417755
C	-4.719262	-0.799934	-0.238083
C	-5.954537	-1.195482	-1.039555
C	-5.066783	-0.580277	1.230262
H	4.481955	-1.583875	0.584871
H	3.012002	-0.333559	-1.808196
H	4.829158	1.910868	1.085095
H	4.796341	0.195294	1.483487
H	3.048450	-3.484160	0.291554
H	2.453559	-2.868833	-1.252129
H	3.473778	1.252979	1.974144
H	0.338389	0.904141	-1.604081
H	-0.424673	-0.134132	1.153913
H	0.750470	-1.413685	-1.384252
H	-2.571831	1.344771	-1.731766
H	-2.842784	3.049558	-1.048730
H	2.401009	1.930655	-1.638808
H	1.390931	1.295531	1.134111
H	2.989035	2.894657	-0.284914
H	0.797487	-1.333154	2.382854
H	2.560125	-1.257093	2.267303
H	1.758519	-2.811761	2.355263
H	5.652705	-1.786631	-1.394966
H	-3.435216	-2.013158	-1.481398

H	-3.970147	-2.814615	0.003489
H	-4.337550	0.139654	-0.644700
H	-6.730878	-0.434007	-0.952622
H	-5.720028	-1.319403	-2.097998
H	-6.368403	-2.138075	-0.673080
H	-5.843149	0.179763	1.328497
H	-4.204214	-0.251379	1.811125
H	-5.441060	-1.502691	1.681107

CAM-B3LYP energy = -1154.92658615 a.u.

(2*R*,6*R*,7*S*,8*S*)-**1**, Conf. K

C	3.918042	-1.718240	-0.422354
C	3.455134	-0.400339	-0.954038
C	3.462844	0.764871	-0.314171
C	4.243786	1.069826	0.932131
C	1.587438	-1.841953	0.559722
C	2.680552	-2.605921	-0.147421
C	1.235032	1.791729	0.099675
C	-0.009999	1.100291	-0.529888
C	-0.350198	-0.219246	0.199112
C	0.644738	-1.267341	-0.180307
O	0.824550	3.146157	0.430190
C	-0.469828	3.374702	0.177765
C	-1.062833	2.173744	-0.456739
O	-0.993682	4.427134	0.435338
C	-2.299053	2.193484	-0.924711
C	2.520003	1.862918	-0.744226
C	1.742759	-1.679938	2.040556
O	4.720105	-2.450529	-1.353292
O	-1.660200	-0.642816	-0.254927
C	-2.338034	-1.481737	0.545540
O	-1.957050	-1.784057	1.650095
C	-3.592749	-2.004150	-0.097492
C	-4.613535	-0.933080	-0.500363
C	-5.821434	-1.597821	-1.151872
C	-5.032559	-0.079568	0.691411
H	4.477181	-1.588619	0.505552
H	2.874008	-0.475281	-1.869395
H	4.874034	0.246576	1.258117
H	3.590876	1.345450	1.763789
H	3.000236	-3.477945	0.425730
H	2.322512	-2.954329	-1.117339
H	4.888643	1.932755	0.748277
H	0.189995	0.860852	-1.574962
H	-0.402440	-0.049575	1.271584
H	0.641675	-1.472653	-1.247162
H	-2.729717	1.348081	-1.437940
H	-2.904668	3.082395	-0.801255
H	2.279100	1.790944	-1.805016
H	1.462628	1.328696	1.058563
H	2.967282	2.842523	-0.569053
H	1.830357	-2.661686	2.511101
H	0.906303	-1.159495	2.500889
H	2.660216	-1.136797	2.280244
H	5.520101	-1.942128	-1.524454
H	-3.294776	-2.571816	-0.982298

H	-4.043317	-2.700210	0.610180
H	-4.144474	-0.285978	-1.244076
H	-6.544527	-0.849228	-1.478539
H	-5.529763	-2.188311	-2.021959
H	-6.324701	-2.263267	-0.446304
H	-5.758984	0.675475	0.387802
H	-4.183739	0.440371	1.139249
H	-5.494465	-0.697293	1.465469

CAM-B3LYP energy = -1154.92648677 a.u.

(2*R*,6*R*,7*S*,8*S*)-**1**, Conf. L

C	2.773785	-2.916373	-0.273702
C	3.050818	-1.480262	-0.613755
C	3.504326	-0.533928	0.199064
C	4.040825	-0.790412	1.577028
C	0.443075	-1.975955	0.096262
C	1.418575	-3.060565	0.476270
C	2.093423	1.526512	0.426845
C	0.755116	1.240463	-0.310921
C	-0.226051	0.444046	0.576500
C	0.341179	-0.913004	0.891026
O	2.252608	2.970823	0.437057
C	1.243710	3.610438	-0.168533
C	0.279840	2.614987	-0.695827
O	1.203445	4.811034	-0.241413
C	-0.764743	2.988464	-1.416388
C	3.359624	0.918248	-0.184166
C	-0.211593	-2.140996	-1.243502
O	2.697168	-3.713577	-1.459878
O	-1.480833	0.390277	-0.127872
C	-2.594811	0.210872	0.598440
O	-2.585345	0.080835	1.796945
C	-3.823253	0.238742	-0.269141
C	-5.024084	-0.499293	0.317598
C	-6.276652	-0.185341	-0.493924
C	-4.776517	-2.003502	0.378818
H	3.552018	-3.327335	0.373717
H	2.674738	-1.184792	-1.589324
H	4.158182	-1.850224	1.791805
H	3.388311	-0.369002	2.346222
H	1.622841	-3.009944	1.545518
H	1.032330	-4.057856	0.257473
H	5.013927	-0.308691	1.696420
H	0.948414	0.647080	-1.204522
H	-0.392117	0.990451	1.504688
H	0.869598	-0.944999	1.837859
H	-1.459295	2.274573	-1.833432
H	-0.938107	4.041120	-1.601676
H	3.327622	1.039125	-1.268215
H	2.020074	1.245146	1.476198
H	4.205929	1.496787	0.191246
H	0.532931	-2.408860	-1.994642
H	-0.751651	-1.257788	-1.568183
H	-0.918521	-2.974664	-1.202875
H	3.530060	-3.627229	-1.936514
H	-4.064963	1.296529	-0.412790

H	-3.566664	-0.153292	-1.254923
H	-5.172347	-0.133202	1.336130
H	-6.485753	0.885814	-0.506282
H	-6.163684	-0.517573	-1.529008
H	-7.146574	-0.693284	-0.075161
H	-5.629861	-2.518446	0.822734
H	-3.896124	-2.243857	0.975940
H	-4.627202	-2.409570	-0.625055

CAM-B3LYP energy = -1154.92588686 a.u.

(2*R*,6*R*,7*S*,8*S*)-**1**, Conf. M

C	3.496095	-2.348558	-0.182226
C	3.416961	-0.893806	-0.547118
C	3.598945	0.147824	0.255525
C	4.140322	0.052364	1.652285
C	0.996897	-2.014803	0.129428
C	2.204736	-2.812420	0.550112
C	1.717812	1.799977	0.403728
C	0.513255	1.167933	-0.349580
C	-0.259632	0.173469	0.544091
C	0.618261	-0.995622	0.897290
O	1.509934	3.237750	0.371404
C	0.388263	3.587367	-0.271713
C	-0.279731	2.368640	-0.787812
O	0.049344	4.736907	-0.381438
C	-1.360499	2.449051	-1.546151
C	3.111167	1.512860	-0.163864
C	0.432559	-2.363803	-1.216047
O	3.641970	-3.158924	-1.352745
O	-1.451368	-0.199337	-0.173059
C	-2.492148	-0.650423	0.544392
O	-2.481168	-0.721067	1.747773
C	-3.625605	-1.078324	-0.346622
C	-4.995961	-1.060008	0.325576
C	-5.440650	0.364283	0.643589
C	-6.019445	-1.766953	-0.557010
H	4.339029	-2.543324	0.485181
H	3.006858	-0.715038	-1.537290
H	3.378261	0.301314	2.395683
H	4.954813	0.767225	1.789249
H	2.367164	-2.690786	1.620793
H	2.085038	-3.878917	0.350543
H	4.516563	-0.940233	1.889834
H	0.873227	0.618212	-1.218850
H	-0.571280	0.680706	1.456641
H	1.120531	-0.877627	1.851464
H	-1.838866	1.573021	-1.958115
H	-1.786853	3.418883	-1.769822
H	3.080944	1.603640	-1.250890
H	1.690933	1.536967	1.460122
H	3.778110	2.288865	0.216932
H	1.233513	-2.422909	-1.954781
H	-0.323455	-1.664890	-1.558635
H	-0.018331	-3.359259	-1.174503
H	4.439533	-2.880830	-1.816250
H	-3.622216	-0.458843	-1.245287

H	-3.379795	-2.093545	-0.674078
H	-4.911338	-1.611314	1.264951
H	-4.728521	0.873440	1.293926
H	-5.541665	0.950132	-0.273703
H	-6.408807	0.364007	1.146630
H	-7.000636	-1.776469	-0.080225
H	-5.730174	-2.800902	-0.753203
H	-6.121888	-1.258445	-1.519029

CAM-B3LYP energy = -1154.92587072 a.u.

(2*R*,6*R*,7*S*,8*S*)-**1**, Conf. N

C	3.490792	-2.357442	-0.206276
C	3.426773	-0.902437	-0.556840
C	3.592338	0.128357	0.261996
C	4.107676	0.014235	1.667351
C	0.993529	-2.007848	0.134349
C	2.201438	-2.803129	0.555479
C	1.727042	1.800757	0.407395
C	0.520464	1.174741	-0.347814
C	-0.258161	0.184091	0.544890
C	0.615453	-0.987218	0.900777
O	1.531280	3.240121	0.366528
C	0.411926	3.595251	-0.277407
C	-0.265826	2.379349	-0.787588
O	0.081629	4.746835	-0.392245
C	-1.348426	2.464890	-1.542774
C	3.120669	1.500331	-0.152207
C	0.429073	-2.357829	-1.210921
O	3.655847	-3.065022	-1.436977
O	-1.450149	-0.185193	-0.173714
C	-2.491447	-0.637029	0.542651
O	-2.480958	-0.709439	1.745906
C	-3.624290	-1.063466	-0.349825
C	-4.993493	-1.059437	0.324821
C	-5.447310	0.359353	0.654302
C	-6.013720	-1.766752	-0.561234
H	4.343765	-2.572970	0.442309
H	3.046079	-0.714652	-1.556289
H	4.477081	-0.982665	1.897967
H	3.334951	0.256615	2.401717
H	2.380858	-2.660132	1.621386
H	2.064834	-3.874994	0.389482
H	4.922790	0.723750	1.826801
H	0.879596	0.623062	-1.216275
H	-0.569580	0.693295	1.456433
H	1.115877	-0.869348	1.855755
H	-1.834204	1.590955	-1.950437
H	-1.768604	3.437019	-1.768159
H	3.098192	1.597029	-1.238923
H	1.692826	1.543729	1.465101
H	3.793165	2.267680	0.236599
H	1.227888	-2.402946	-1.952979
H	-0.336575	-1.666458	-1.547085
H	-0.009205	-3.359076	-1.172561
H	3.713592	-4.006596	-1.243315
H	-3.626070	-0.435885	-1.242906

H	-3.372941	-2.074067	-0.687277
H	-4.903500	-1.617191	1.259894
H	-4.737514	0.868309	1.307301
H	-5.553778	0.951348	-0.258422
H	-6.414634	0.348751	1.158842
H	-6.994009	-1.786370	-0.082901
H	-5.717847	-2.797280	-0.765458
H	-6.121226	-1.251948	-1.519342

CAM-B3LYP energy = -1154.92579317 a.u.

(2*R*,6*R*,7*S*,8*S*)-**1**, Conf. O

C	2.759279	-2.929041	-0.303231
C	3.053601	-1.497103	-0.630285
C	3.489845	-0.558892	0.200102
C	3.994872	-0.829646	1.587479
C	0.437932	-1.971132	0.108378
C	1.416405	-3.052311	0.486499
C	2.105386	1.520485	0.428698
C	0.765113	1.242297	-0.308266
C	-0.220477	0.453781	0.581128
C	0.341758	-0.904382	0.898939
O	2.278448	2.963360	0.429291
C	1.273332	3.608707	-0.176288
C	0.299002	2.619227	-0.695799
O	1.243282	4.809267	-0.255016
C	-0.745865	2.998939	-1.412679
C	3.366193	0.896671	-0.176891
C	-0.225407	-2.141472	-1.226445
O	2.714832	-3.629492	-1.547968
O	-1.475521	0.404864	-0.122977
C	-2.590086	0.228970	0.603213
O	-2.581188	0.098333	1.801658
C	-3.818516	0.261241	-0.264251
C	-5.017523	-0.484487	0.316603
C	-6.270510	-0.168688	-0.493548
C	-4.765390	-1.988333	0.367432
H	3.544927	-3.364567	0.319364
H	2.707688	-1.199083	-1.615527
H	4.099088	-1.892225	1.795700
H	3.331781	-0.408524	2.347619
H	1.650490	-2.979762	1.548874
H	1.010689	-4.052021	0.310237
H	4.970137	-0.358575	1.729949
H	0.954798	0.645500	-1.200502
H	-0.383619	1.003487	1.507900
H	0.874366	-0.933035	1.843388
H	-1.448314	2.289507	-1.824033
H	-0.911300	4.052397	-1.600624
H	3.341913	1.022410	-1.260602
H	2.028262	1.247100	1.479855
H	4.218063	1.462613	0.205450
H	0.516787	-2.394024	-1.985077
H	-0.781469	-1.265247	-1.542405
H	-0.918713	-2.986408	-1.183305
H	2.547569	-4.560887	-1.369552
H	-4.062882	1.319592	-0.398861

H	-3.561240	-0.122105	-1.253227
H	-5.167481	-0.125983	1.337572
H	-6.482747	0.901901	-0.498721
H	-6.155862	-0.493495	-1.530798
H	-7.139187	-0.682043	-0.078831
H	-5.617375	-2.508964	0.807288
H	-3.884583	-2.230246	0.963373
H	-4.614277	-2.386953	-0.639148

CAM-B3LYP energy = -1154.92578279 a.u.

(2*R*,6*R*,7*S*,8*S*)-**1**, Conf. P

C	3.996692	-1.649639	-0.376862
C	3.540377	-0.304892	-0.847498
C	3.485353	0.815207	-0.134531
C	4.175092	1.042574	1.180231
C	1.607093	-1.877560	0.442157
C	2.755456	-2.575900	-0.244103
C	1.219459	1.794470	0.180358
C	0.048202	1.101948	-0.574925
C	-0.323980	-0.252270	0.072941
C	0.695535	-1.273635	-0.313467
O	0.746022	3.122790	0.533065
C	-0.526344	3.335565	0.175196
C	-1.030129	2.152036	-0.560756
O	-1.096536	4.364515	0.429339
C	-2.212338	2.169500	-1.152921
C	2.562299	1.933287	-0.556625
C	1.677080	-1.801832	1.936530
O	4.972411	-2.255807	-1.224502
O	-1.613317	-0.640513	-0.457168
C	-2.331497	-1.521751	0.256999
O	-1.967606	-1.954472	1.323297
C	-3.627158	-1.869847	-0.418563
C	-4.718537	-0.801699	-0.229602
C	-5.956278	-1.193015	-1.029343
C	-5.061597	-0.590096	1.240982
H	4.488359	-1.574229	0.589716
H	3.019775	-0.320858	-1.802702
H	4.782222	0.199831	1.500227
H	3.464755	1.270489	1.978488
H	3.057033	-3.480276	0.287029
H	2.455880	-2.866356	-1.253881
H	4.831125	1.912397	1.094479
H	0.341241	0.904481	-1.606566
H	-0.422954	-0.137440	1.149560
H	0.752799	-1.414569	-1.389426
H	-2.570304	1.337912	-1.740595
H	-2.846522	3.041934	-1.057761
H	2.401974	1.935801	-1.635135
H	1.385551	1.296980	1.134636
H	2.983571	2.900776	-0.279536
H	1.770697	-2.807765	2.351508
H	0.799282	-1.335941	2.377712
H	2.560906	-1.247313	2.261216
H	4.609023	-2.325985	-2.114842
H	-3.438674	-2.008516	-1.483568

H	-3.968147	-2.817432	-0.000680
H	-4.338145	0.140147	-0.632269
H	-5.725116	-1.311141	-2.089182
H	-6.368891	-2.137621	-0.666665
H	-6.732403	-0.432107	-0.935789
H	-5.434770	-1.514915	1.687790
H	-5.837497	0.169554	1.345710
H	-4.197243	-0.264653	1.821098

CAM-B3LYP energy = -1154.92571047 a.u.

(2*R*,6*R*,7*S*,8*S*)-**1**, Conf. Q

C	3.995855	-1.649164	-0.382837
C	3.545222	-0.308125	-0.855272
C	3.484246	0.809282	-0.138779
C	4.169139	1.037320	1.178617
C	1.604136	-1.871855	0.442014
C	2.749220	-2.569841	-0.249263
C	1.221322	1.799881	0.175628
C	0.044206	1.110801	-0.573281
C	-0.325427	-0.243768	0.074260
C	0.695167	-1.263441	-0.313202
O	0.756490	3.132841	0.522845
C	-0.518475	3.347669	0.175872
C	-1.032456	2.162534	-0.550519
O	-1.083386	4.379380	0.430768
C	-2.221061	2.180510	-1.129769
C	2.563361	1.927758	-0.564476
C	1.671423	-1.803267	1.936758
O	4.911216	-2.200588	-1.331117
O	-1.614313	-0.633769	-0.456359
C	-2.330319	-1.518309	0.255814
O	-1.966802	-1.950317	1.322562
C	-3.623014	-1.871724	-0.422771
C	-4.721282	-0.810985	-0.232079
C	-5.954339	-1.206126	-1.037202
C	-5.069892	-0.608141	1.238418
H	4.488202	-1.573014	0.587555
H	3.035809	-0.327900	-1.814880
H	4.784154	0.198863	1.494674
H	3.455650	1.253189	1.977411
H	3.041783	-3.479379	0.281335
H	2.456301	-2.850581	-1.262047
H	4.815937	1.914559	1.098412
H	0.330337	0.914895	-1.607205
H	-0.424538	-0.129505	1.150943
H	0.755029	-1.400464	-1.389423
H	-2.586914	1.347937	-1.711083
H	-2.852422	3.054497	-1.030300
H	2.401075	1.924617	-1.642659
H	1.386087	1.306019	1.132028
H	2.988987	2.895218	-0.293770
H	2.558355	-1.256154	2.265428
H	1.757541	-2.811676	2.347476
H	0.795684	-1.333744	2.378233
H	5.157505	-3.085954	-1.040723
H	-3.432071	-2.005779	-1.487909

H	-3.959040	-2.822694	-0.008531
H	-4.345602	0.134999	-0.629434
H	-6.735376	-0.450414	-0.942404
H	-5.719397	-1.317917	-2.096895
H	-6.362204	-2.154909	-0.680090
H	-5.850357	0.146662	1.344308
H	-4.209103	-0.280686	1.822660
H	-5.439254	-1.537128	1.679707

CAM-B3LYP energy = -1154.92570134 a.u.

(2*R*,6*R*,7*S*,8*S*)-**1**, Conf. R

C	3.930338	-1.713102	-0.416670
C	3.463429	-0.396853	-0.951805
C	3.464818	0.769477	-0.315080
C	4.242609	1.077759	0.932144
C	1.589061	-1.839844	0.559172
C	2.684803	-2.602922	-0.144975
C	1.234264	1.792008	0.096410
C	-0.010764	1.100063	-0.532554
C	-0.350146	-0.219439	0.196893
C	0.645234	-1.267528	-0.181431
O	0.823335	3.146173	0.427334
C	-0.471302	3.374132	0.175744
C	-1.064036	2.173057	-0.458836
O	-0.995588	4.426182	0.433911
C	-2.300485	2.192361	-0.926199
C	2.518880	1.863712	-0.748059
C	1.746026	-1.673648	2.039436
O	4.841985	-2.396347	-1.276991
O	-1.659831	-0.643771	-0.257181
C	-2.336983	-1.483564	0.543050
O	-1.955155	-1.786660	1.647072
C	-3.592241	-2.005185	-0.099533
C	-4.615424	-0.933469	-0.494755
C	-5.824390	-1.596575	-1.145934
C	-5.032113	-0.085636	0.701879
H	4.481922	-1.580436	0.510635
H	2.879177	-0.471220	-1.866678
H	4.891475	1.937082	0.745590
H	4.870033	0.254421	1.263450
H	3.004143	-3.475054	0.428034
H	2.319481	-2.955420	-1.112321
H	3.588430	1.360158	1.760495
H	0.188763	0.860588	-1.577715
H	-0.402601	-0.049257	1.269287
H	0.641096	-1.475083	-1.247939
H	-2.731015	1.346933	-1.439494
H	-2.906449	3.080952	-0.802156
H	2.277969	1.788295	-1.808621
H	1.462476	1.329025	1.055155
H	2.963894	2.844883	-0.576104
H	0.908720	-1.154197	2.499282
H	2.661996	-1.127033	2.276854
H	1.836887	-2.653802	2.512610
H	4.424079	-2.518787	-2.137283
H	-3.295754	-2.568190	-0.987780

H	-4.040163	-2.705088	0.606033
H	-4.149144	-0.282491	-1.236829
H	-6.549215	-0.847256	-1.467063
H	-5.534574	-2.182667	-2.019606
H	-6.324945	-2.265823	-0.442043
H	-5.760033	0.670009	0.403428
H	-4.182648	0.433172	1.149802
H	-5.491430	-0.707149	1.474441

CAM-B3LYP energy = -1154.92561704 a.u.

(2*R*,6*R*,7*S*,8*S*)-**1**, Conf. S

C	3.931593	-1.707173	-0.423227
C	3.467647	-0.395110	-0.958763
C	3.461387	0.768517	-0.317401
C	4.234192	1.078694	0.932649
C	1.588872	-1.834470	0.555807
C	2.682481	-2.594408	-0.154193
C	1.231497	1.797131	0.092498
C	-0.016949	1.106495	-0.530850
C	-0.351417	-0.214268	0.197755
C	0.645954	-1.259121	-0.183674
O	0.825571	3.154420	0.417384
C	-0.470669	3.382640	0.174910
C	-1.069887	2.179333	-0.449407
O	-0.991788	4.436509	0.432226
C	-2.311482	2.197232	-0.903034
C	2.515199	1.861196	-0.753709
C	1.744206	-1.675006	2.036935
O	4.780918	-2.337750	-1.383464
O	-1.660925	-0.641764	-0.254548
C	-2.334214	-1.484488	0.545766
O	-1.950520	-1.786573	1.649446
C	-3.587365	-2.011811	-0.096368
C	-4.612380	-0.945073	-0.499827
C	-5.817470	-1.614913	-1.151307
C	-5.035078	-0.092919	0.691639
H	4.482299	-1.573420	0.508865
H	2.893875	-0.473842	-1.878183
H	4.868372	0.259261	1.260653
H	3.576691	1.350841	1.761794
H	2.993745	-3.472336	0.417356
H	2.325339	-2.935866	-1.126868
H	4.875016	1.945071	0.750911
H	0.176960	0.869221	-1.577598
H	-0.402503	-0.045357	1.270415
H	0.643786	-1.462146	-1.250955
H	-2.747984	1.350264	-1.408604
H	-2.915690	3.086479	-0.775146
H	2.272859	1.780363	-1.813519
H	1.459033	1.337838	1.053201
H	2.961853	2.842676	-0.587552
H	1.834821	-2.657208	2.505977
H	0.906213	-1.157891	2.498202
H	2.659892	-1.129269	2.277339
H	5.033457	-3.204940	-1.047144
H	-3.287441	-2.579080	-0.980794

H	-4.035030	-2.709120	0.611930
H	-4.145860	-0.296404	-1.243811
H	-6.543580	-0.869385	-1.478268
H	-5.523257	-2.204440	-2.021189
H	-6.318079	-2.282214	-0.445600
H	-5.763927	0.659604	0.387594
H	-4.188333	0.429924	1.140026
H	-5.495287	-0.712227	1.465436

CAM-B3LYP energy = -1154.92561480 a.u.

(2*R*,6*R*,7*S*,8*S*)-**1**, Conf. T

C	2.727362	-2.835968	-0.303005
C	2.898915	-1.460242	-0.861333
C	3.375320	-0.395734	-0.223327
C	4.155260	-0.428092	1.060379
C	0.550332	-1.923979	0.609829
C	1.219688	-3.094240	-0.068142
C	1.836547	1.544896	0.089090
C	0.414822	1.428165	-0.534549
C	-0.454323	0.385733	0.203981
C	-0.009640	-0.993388	-0.156460
O	2.065063	2.963115	0.311817
C	0.984607	3.710286	0.056973
C	-0.092433	2.846372	-0.483090
O	0.973125	4.899003	0.245287
C	-1.236072	3.370592	-0.892087
C	3.028309	0.990548	-0.709623
C	0.702228	-1.842463	2.097653
O	3.160817	-3.864022	-1.197944
O	-1.813948	0.572173	-0.256967
C	-2.808125	0.235578	0.579776
O	-2.615195	-0.165819	1.701854
C	-4.161922	0.415434	-0.049164
C	-4.804146	-0.904597	-0.522958
C	-3.947249	-1.616217	-1.565233
C	-5.159310	-1.834101	0.632629
H	3.257893	-2.940241	0.644577
H	2.385584	-1.301805	-1.806190
H	5.115016	0.072217	0.909274
H	4.357463	-1.435070	1.415563
H	1.112803	-4.012856	0.511305
H	0.773118	-3.262247	-1.049277
H	3.647295	0.115454	1.860393
H	0.495672	1.115573	-1.576959
H	-0.431832	0.572578	1.274780
H	-0.062578	-1.181142	-1.225153
H	-2.020623	2.772897	-1.329571
H	-1.397481	4.436557	-0.791656
H	2.800120	0.993926	-1.775732
H	1.832021	1.102082	1.084350
H	3.864956	1.671660	-0.545506
H	0.327028	-2.759987	2.556252
H	0.164129	-1.003923	2.533089
H	1.754751	-1.760728	2.379969
H	4.105803	-3.752716	-1.347474
H	-4.805698	0.878633	0.699116

H	-4.071801	1.095460	-0.894870
H	-5.737944	-0.605397	-1.006182
H	-4.473708	-2.485665	-1.961237
H	-3.705745	-0.957754	-2.401144
H	-3.007064	-1.968520	-1.134551
H	-5.695429	-2.708674	0.260683
H	-5.797008	-1.333335	1.363037
H	-4.266667	-2.184084	1.152709

CAM-B3LYP energy = -1154.92553407 a.u.

(2*R*,6*R*,7*S*,8*S*)-**1**, Conf. U

C	3.808050	-2.037725	0.389137
C	3.690273	-0.536006	0.316775
C	3.383836	0.237256	-0.720046
C	3.355427	-0.206492	-2.151171
C	1.292408	-1.893508	0.738010
C	2.428103	-2.727826	0.205072
C	1.609458	1.688704	0.326267
C	0.317454	1.283834	-0.435952
C	-0.304807	-0.003191	0.145779
C	0.598866	-1.159196	-0.126354
O	1.399590	3.049904	0.789449
C	0.191885	3.529292	0.466986
C	-0.544975	2.511801	-0.321193
O	-0.164262	4.631831	0.792542
C	-1.734395	2.775638	-0.835962
C	2.927244	1.658968	-0.448496
C	1.138997	-1.849849	2.227572
O	4.667757	-2.632177	-0.585200
O	-1.562057	-0.192859	-0.545791
C	-2.512724	-0.906493	0.076472
O	-2.391691	-1.326155	1.200581
C	-3.703397	-1.133988	-0.814029
C	-5.015576	-1.349083	-0.063535
C	-5.461802	-0.077190	0.651223
C	-6.092631	-1.841376	-1.024606
H	4.188025	-2.285632	1.384081
H	3.681895	-0.067291	1.297493
H	3.633199	-1.250289	-2.263786
H	4.049672	0.400438	-2.739022
H	2.467377	-3.709768	0.680273
H	2.297818	-2.879826	-0.866495
H	2.366751	-0.049634	-2.589620
H	0.546762	1.096215	-1.485706
H	-0.513559	0.128546	1.204920
H	0.799496	-1.297639	-1.184395
H	-2.269255	2.060059	-1.442093
H	-2.194401	3.738389	-0.652021
H	2.811788	2.217582	-1.379081
H	1.714623	1.089528	1.228550
H	3.664404	2.189108	0.156547
H	1.046723	-2.865780	2.617671
H	0.265819	-1.285887	2.546478
H	2.022623	-1.413640	2.701461
H	5.545783	-2.249092	-0.483278
H	-3.785837	-0.303121	-1.516697

H	-3.462647	-2.018904	-1.411607
H	-4.843858	-2.123243	0.687902
H	-6.384535	-0.249411	1.207351
H	-4.709083	0.274183	1.358048
H	-5.650674	0.723208	-0.068833
H	-7.031863	-2.014628	-0.497356
H	-5.799827	-2.775668	-1.506822
H	-6.282088	-1.103564	-1.808318

CAM-B3LYP energy = -1154.92544501 a.u.

(2*R*,6*R*,7*S*,8*S*)-**1**, Conf. V

C	3.343922	-2.505761	0.344449
C	3.503491	-1.010250	0.233117
C	3.288802	-0.215417	-0.810538
C	3.102332	-0.676135	-2.224444
C	0.919546	-1.889759	0.805589
C	1.852748	-2.928978	0.241042
C	1.873305	1.561402	0.279509
C	0.489385	1.390970	-0.405079
C	-0.330188	0.253139	0.239581
C	0.327857	-1.055682	-0.044016
O	1.945292	2.945973	0.714095
C	0.831216	3.636116	0.440386
C	-0.123496	2.759991	-0.280979
O	0.704073	4.791291	0.753149
C	-1.270301	3.231411	-0.741604
C	3.117606	1.271719	-0.560308
C	0.854221	-1.791483	2.299183
O	4.030838	-3.270309	-0.648385
O	-1.633653	0.291325	-0.388282
C	-2.677108	-0.183818	0.308164
O	-2.576959	-0.627105	1.425141
C	-3.961171	-0.042189	-0.463156
C	-5.059009	-1.015327	-0.040591
C	-6.387205	-0.608933	-0.670653
C	-4.698955	-2.452275	-0.405128
H	3.719164	-2.799563	1.328625
H	3.635023	-0.528004	1.198261
H	3.863316	-0.219798	-2.863613
H	2.137055	-0.348915	-2.618920
H	1.732714	-3.894011	0.737004
H	1.643059	-3.069075	-0.819362
H	3.178325	-1.755341	-2.319241
H	0.622916	1.145596	-1.459535
H	-0.455926	0.440222	1.303386
H	0.442877	-1.247717	-1.106507
H	-1.960950	2.617212	-1.299664
H	-1.533517	4.266131	-0.561371
H	3.054657	1.822202	-1.500679
H	1.916248	0.970212	1.192277
H	3.972624	1.668708	-0.010880
H	0.108081	-1.079368	2.643348
H	1.822761	-1.504791	2.718131
H	0.609818	-2.768449	2.721711
H	4.967892	-3.052495	-0.597816
H	-4.292650	0.989034	-0.305649

H	-3.744383	-0.135063	-1.528778
H	-5.158489	-0.950885	1.045429
H	-7.184638	-1.287417	-0.364163
H	-6.674497	0.402572	-0.378045
H	-6.326168	-0.638293	-1.761527
H	-5.477365	-3.141969	-0.075227
H	-3.761618	-2.762854	0.057820
H	-4.595040	-2.559723	-1.487926

CAM-B3LYP energy = -1154.92543703 a.u.

H	3.571125	-0.455956	1.380954
H	3.317495	-2.112249	0.881444
H	4.961298	-1.761789	-0.997053
H	6.506134	0.175937	-0.886490
H	4.851409	0.724777	-1.142123
H	5.586273	0.847715	0.460862
H	6.970347	-1.920635	0.466470
H	5.636935	-2.880611	1.111861
H	6.030628	-1.316416	1.831160

CAM-B3LYP energy = -1154.92784840 a.u.

(2*S*,6*R*,7*S*,8*S*)-**1**, Conf. A

C	-3.531833	-2.223358	-0.107740
C	-3.553036	-0.737726	-0.287337
C	-3.399914	0.195450	0.647942
C	-3.457884	-0.041545	2.128930
C	-1.031412	-1.995127	-0.204209
C	-2.216204	-2.793502	-0.687135
C	-1.735938	1.674706	-0.539270
C	-0.472112	1.178981	0.210251
C	0.317499	0.139651	-0.616254
C	-0.552962	-1.030815	-0.985860
O	-1.478716	3.061808	-0.888705
C	-0.362731	3.537314	-0.316535
C	0.289740	2.450415	0.455028
O	-0.013147	4.680318	-0.453518
C	1.344836	2.674734	1.219840
C	-3.049456	1.614370	0.239399
C	-0.594178	-2.281784	1.201621
O	-4.585872	-2.876337	-0.820844
O	1.463515	-0.214774	0.181801
C	2.534177	-0.715039	-0.455273
O	2.587322	-0.840003	-1.652945
C	3.607310	-1.121639	0.516655
C	5.011640	-1.170819	-0.079533
C	5.514224	0.225147	-0.434750
C	5.965566	-1.864271	0.887668
H	-3.599235	-2.488061	0.948652
H	-3.524411	-0.431525	-1.330658
H	-3.753566	-1.053818	2.392752
H	-4.178924	0.646361	2.577397
H	-2.293536	-2.739884	-1.773924
H	-2.143176	-3.845730	-0.405268
H	-2.496625	0.169477	2.604520
H	-0.749508	0.707286	1.150901
H	0.682920	0.609049	-1.529273
H	-0.956551	-0.959585	-1.989784
H	1.805369	1.889057	1.801443
H	1.769733	3.668972	1.276323
H	-2.962796	2.249289	1.123271
H	-1.854399	1.159144	-1.487868
H	-3.829437	2.048420	-0.388880
H	-0.214097	-3.305042	1.262331
H	-1.440303	-2.227679	1.889600
H	0.183954	-1.611316	1.552072
H	-5.425869	-2.564114	-0.467659

(2*S*,6*R*,7*S*,8*S*)-**1**, Conf. B

C	-2.900298	-2.750672	-0.044967
C	-3.261011	-1.311839	-0.244367
C	-3.309134	-0.352564	0.675734
C	-3.288434	-0.571514	2.160571
C	-0.518161	-1.964106	-0.195985
C	-1.499162	-3.018790	-0.642906
C	-2.043563	1.451020	-0.557739
C	-0.690755	1.253963	0.175060
C	0.300897	0.413688	-0.659562
C	-0.286984	-0.929157	-0.999153
O	-2.109534	2.859193	-0.910631
C	-1.124062	3.575308	-0.349535
C	-0.232327	2.664768	0.410817
O	-1.044271	4.767799	-0.487437
C	0.756119	3.124631	1.159244
C	-3.297685	1.102093	0.243273
C	0.000299	-2.120207	1.202758
O	-3.790564	-3.638342	-0.727329
O	1.511134	0.339329	0.118800
C	2.660266	0.113482	-0.536252
O	2.715782	-0.048076	-1.729644
C	3.838046	0.134243	0.399053
C	5.033023	-0.686903	-0.079872
C	6.252499	-0.387103	0.785497
C	4.716974	-2.179527	-0.080217
H	-2.888903	-3.004172	1.016152
H	-3.319067	-1.025081	-1.291857
H	-2.389316	-0.146630	2.614219
H	-3.348815	-1.619410	2.443820
H	-1.604937	-3.003987	-1.728539
H	-1.184771	-4.021914	-0.348042
H	-4.135656	-0.051686	2.614531
H	-0.844535	0.732207	1.117498
H	0.533791	0.941674	-1.584051
H	-0.714428	-0.967949	-1.994809
H	1.393113	2.466392	1.732441
H	0.944139	4.189731	1.209533
H	-3.345115	1.754075	1.117673
H	-2.057584	0.919274	-1.504650
H	-4.165469	1.337122	-0.375778
H	-0.820877	-2.272785	1.905739
H	0.593134	-1.274454	1.536084
H	0.624877	-3.015739	1.260778
H	-4.672618	-3.521187	-0.358500

H	4.121390	1.186584	0.500244
H	3.509664	-0.192165	1.387302
H	5.254375	-0.381775	-1.105209
H	7.119059	-0.954695	0.443401
H	6.511052	0.672854	0.756144
H	6.066816	-0.658741	1.827709
H	5.566697	-2.754898	-0.450746
H	3.858721	-2.410597	-0.712435
H	4.495622	-2.526176	0.932541

CAM-B3LYP energy = -1154.92782917 a.u.

(2*S*,6*R*,7*S*,8*S*)-**1**, Conf. C

C	-3.841831	-2.023162	0.129713
C	-3.687275	-0.535128	0.007942
C	-3.363174	0.337131	0.954604
C	-3.321502	0.025540	2.419697
C	-1.354307	-1.952970	-0.456796
C	-2.460997	-2.737774	0.200321
C	-1.613161	1.659164	-0.294492
C	-0.296124	1.288409	0.440218
C	0.287239	-0.043215	-0.077433
C	-0.610824	-1.167517	0.317108
O	-1.400361	2.973884	-0.875289
C	-0.175060	3.456943	-0.634359
C	0.574893	2.492809	0.206843
O	0.184481	4.523968	-1.059293
C	1.782086	2.779259	0.665190
C	-2.890988	1.719087	0.541564
C	-1.281463	-2.016021	-1.951404
O	-4.531617	-2.549477	-1.008422
O	1.572666	-0.199335	0.569731
C	2.498406	-0.943850	-0.053040
O	2.333357	-1.421021	-1.148376
C	3.725928	-1.125736	0.796997
C	5.015094	-1.311153	-0.000616
C	5.392994	-0.035261	-0.746768
C	6.142591	-1.757969	0.923963
H	-4.396020	-2.293095	1.032156
H	-3.681753	-0.195760	-1.024960
H	-3.916720	0.754376	2.974969
H	-2.302677	0.097100	2.809462
H	-2.586761	-3.723619	-0.250437
H	-2.217432	-2.883005	1.252614
H	-3.700169	-0.968285	2.650037
H	-0.484303	1.173820	1.508484
H	0.449385	0.011226	-1.151241
H	-0.754326	-1.232510	1.392062
H	2.326875	2.104688	1.308496
H	2.247231	3.718979	0.395272
H	-2.719165	2.349140	1.416228
H	-1.771783	0.993499	-1.140277
H	-3.649145	2.214834	-0.067170
H	-0.446105	-1.452039	-2.359746
H	-2.207023	-1.650979	-2.402231
H	-1.174945	-3.055996	-2.267999
H	-5.387203	-2.111308	-1.073998

H	3.810031	-0.284715	1.486959
H	3.533822	-2.011544	1.410637
H	4.838176	-2.099871	-0.735530
H	6.298032	-0.185398	-1.337250
H	4.602251	0.283636	-1.427197
H	5.584264	0.779698	-0.043895
H	7.065804	-1.909952	0.362935
H	5.896959	-2.694080	1.428476
H	6.339356	-1.004505	1.690820

CAM-B3LYP energy = -1154.92778537 a.u.

(2*S*,6*R*,7*S*,8*S*)-**1**, Conf. D

C	-3.344399	-2.524854	0.185327
C	-3.485778	-1.032598	0.103720
C	-3.270083	-0.135981	1.058546
C	-3.073373	-0.467846	2.506450
C	-0.954409	-1.962479	-0.529873
C	-1.850265	-2.960541	0.158686
C	-1.886267	1.525525	-0.241420
C	-0.480170	1.402911	0.406383
C	0.314159	0.216556	-0.179196
C	-0.328683	-1.066901	0.228525
O	-1.966902	2.868286	-0.790913
C	-0.843360	3.573234	-0.608803
C	0.129570	2.755683	0.155905
O	-0.722026	4.697295	-1.021329
C	1.287772	3.260142	0.547831
C	-3.097701	1.319736	0.666826
C	-0.962260	-1.975260	-2.027467
O	-3.986740	-3.150155	-0.930264
O	1.641420	0.300279	0.392366
C	2.661963	-0.206988	-0.315222
O	2.523704	-0.718486	-1.398465
C	3.973038	-0.006499	0.395243
C	5.071474	-0.981003	-0.022073
C	6.414626	-0.522635	0.536592
C	4.751872	-2.404123	0.425502
H	-3.782167	-2.915840	1.107250
H	-3.612368	-0.675946	-0.915595
H	-3.234716	-1.522110	2.722465
H	-2.065061	-0.206656	2.838164
H	-1.811468	-3.941728	-0.317308
H	-1.522355	-3.079399	1.191227
H	-3.762152	0.115953	3.121730
H	-0.576963	1.238763	1.480464
H	0.398414	0.319883	-1.258248
H	-0.394320	-1.182111	1.306780
H	1.992127	2.691780	1.136557
H	1.546695	4.276268	0.277978
H	-2.994971	1.951062	1.551260
H	-1.965780	0.859389	-1.098128
H	-3.972819	1.673713	0.119154
H	-0.276522	-1.251601	-2.461768
H	-1.966528	-1.783670	-2.412206
H	-0.679662	-2.967531	-2.386215
H	-4.913073	-2.884972	-0.934695

H	4.278722	1.021121	0.174722
H	3.799861	-0.049748	1.472015
H	5.129105	-0.968232	-1.112941
H	7.212633	-1.201162	0.231788
H	6.671404	0.478563	0.185996
H	6.395210	-0.501186	1.629172
H	5.530600	-3.096021	0.100933
H	3.804313	-2.753492	0.013958
H	4.689844	-2.459320	1.515434

CAM-B3LYP energy = -1154.92769101 a.u.

(2*S*,6*R*,7*S*,8*S*)-**1**, Conf. E

C	-3.857757	-2.014195	0.103445
C	-3.706411	-0.527541	0.005040
C	-3.360809	0.327482	0.958653
C	-3.298005	-0.005558	2.418405
C	-1.358001	-1.948328	-0.432548
C	-2.469149	-2.719561	0.230029
C	-1.616296	1.662979	-0.282994
C	-0.293483	1.296249	0.443788
C	0.286359	-0.038365	-0.069754
C	-0.611507	-1.159027	0.334719
O	-1.412553	2.981086	-0.859887
C	-0.185184	3.464705	-0.632330
C	0.575725	2.499654	0.197994
O	0.168471	4.533086	-1.058967
C	1.789714	2.785414	0.638536
C	-2.891917	1.713886	0.557413
C	-1.287170	-2.021750	-1.926788
O	-4.548813	-2.420565	-1.079718
O	1.574665	-0.192044	0.572685
C	2.495546	-0.943794	-0.048410
O	2.323788	-1.430098	-1.138708
C	3.727393	-1.120602	0.796464
C	5.011760	-1.315029	-0.006757
C	5.387206	-0.046310	-0.766261
C	6.143801	-1.755380	0.915362
H	-4.449394	-2.300915	0.977053
H	-3.727619	-0.173488	-1.021952
H	-2.278367	0.082319	2.802103
H	-3.654598	-1.010370	2.636622
H	-2.565911	-3.727602	-0.181287
H	-2.248966	-2.823061	1.292955
H	-3.904708	0.701571	2.989350
H	-0.474110	1.187983	1.514114
H	0.443844	0.010450	-1.144520
H	-0.752756	-1.215696	1.410224
H	2.343763	2.110710	1.273670
H	2.251051	3.724979	0.361617
H	-2.719234	2.337282	1.436691
H	-1.775623	0.999340	-1.130469
H	-3.651813	2.213308	-0.046030
H	-1.194593	-3.064940	-2.237366
H	-0.444245	-1.471473	-2.338179
H	-2.208484	-1.647156	-2.378192
H	-4.681675	-3.373573	-1.044728

H	3.816788	-0.273743	1.478606
H	3.537247	-2.000723	1.418824
H	4.829554	-2.109912	-0.733679
H	6.289062	-0.202702	-1.359981
H	4.593317	0.267282	-1.445493
H	5.582872	0.774689	-0.071674
H	7.063664	-1.913484	0.350540
H	5.899730	-2.686757	1.429320
H	6.345858	-0.995616	1.674589

CAM-B3LYP energy = -1154.92768187 a.u.

(2*S*,6*R*,7*S*,8*S*)-**1**, Conf. F

C	-3.351078	-2.527916	0.165028
C	-3.498390	-1.038826	0.106531
C	-3.262062	-0.154315	1.066720
C	-3.042567	-0.502278	2.507661
C	-0.950077	-1.957775	-0.516160
C	-1.845980	-2.950665	0.177258
C	-1.892860	1.523742	-0.227690
C	-0.480856	1.410170	0.409256
C	0.314198	0.224940	-0.177151
C	-0.323276	-1.058788	0.237459
O	-1.987623	2.867863	-0.772108
C	-0.865211	3.577736	-0.604037
C	0.120830	2.764724	0.148743
O	-0.753977	4.702431	-1.017848
C	1.282262	3.274201	0.524332
C	-3.097171	1.305544	0.687306
C	-0.960616	-1.974710	-2.013795
O	-4.033439	-3.039550	-0.981817
O	1.644145	0.314087	0.387642
C	2.661376	-0.197709	-0.321326
O	2.518218	-0.715163	-1.401142
C	3.975869	0.005443	0.382048
C	5.067155	-0.981309	-0.025468
C	6.414644	-0.524375	0.523781
C	4.739757	-2.396504	0.441297
H	-3.811977	-2.940920	1.066297
H	-3.653821	-0.673391	-0.904913
H	-3.197683	-1.559746	2.712626
H	-3.726809	0.070004	3.138735
H	-1.773020	-3.944623	-0.271069
H	-1.538979	-3.042418	1.219558
H	-2.031773	-0.240395	2.830803
H	-0.568845	1.250431	1.484798
H	0.392629	0.325614	-1.256907
H	-0.384423	-1.170163	1.316187
H	1.996711	2.709672	1.104484
H	1.533427	4.290885	0.249326
H	-2.991198	1.929953	1.576273
H	-1.973529	0.859664	-1.086037
H	-3.976759	1.660972	0.147873
H	-0.261475	-1.265008	-2.449759
H	-1.961467	-1.764934	-2.397718
H	-0.696849	-2.972840	-2.370667
H	-3.988566	-4.001156	-0.961304

H	4.286874	1.027928	0.145694
H	3.806348	-0.021729	1.459876
H	5.121913	-0.982988	-1.116549
H	7.207533	-1.211741	0.225488
H	6.676735	0.470653	0.159841
H	6.398338	-0.489004	1.616047
H	5.513430	-3.097388	0.124010
H	3.789116	-2.745420	0.036449
H	4.679830	-2.437229	1.531979

CAM-B3LYP energy = -1154.92760000 a.u.

(2*S*,6*R*,7*S*,8*S*)-**1**, Conf. G

C	-3.278925	-2.404362	0.261738
C	-3.362566	-0.907479	0.332539
C	-2.956333	-0.108331	1.311653
C	-2.543073	-0.575604	2.674241
C	-1.014013	-1.849619	-0.791071
C	-1.832129	-2.882170	-0.057975
C	-1.718012	1.614975	-0.052249
C	-0.235328	1.387955	0.348863
C	0.404097	0.238390	-0.457892
C	-0.226537	-1.057069	-0.069700
O	-1.820062	3.003025	-0.469897
C	-0.650854	3.652461	-0.407325
C	0.389183	2.738423	0.123991
O	-0.542878	4.804647	-0.737676
C	1.614781	3.168062	0.374331
C	-2.780427	1.370166	1.018648
C	-1.270648	-1.710197	-2.259985
O	-4.127220	-2.904874	-0.776717
O	1.804742	0.226773	-0.094865
C	2.678297	-0.279257	-0.977236
O	2.354823	-0.682672	-2.067708
C	4.081223	-0.266575	-0.438377
C	4.265729	-1.109843	0.831108
C	3.914990	-2.573233	0.583367
C	5.694292	-0.965975	1.343678
H	-3.573364	-2.864815	1.208093
H	-3.635876	-0.455549	-0.617820
H	-1.482428	-0.380390	2.853693
H	-2.718515	-1.638763	2.826261
H	-1.920055	-3.810562	-0.624661
H	-1.344029	-3.116635	0.887849
H	-3.092938	-0.025962	3.441938
H	-0.170395	1.126009	1.405673
H	0.328694	0.441557	-1.523487
H	-0.118875	-1.276968	0.988793
H	2.372869	2.525596	0.796528
H	1.876202	4.195895	0.156187
H	-2.511435	1.917586	1.923981
H	-1.962253	1.030000	-0.936428
H	-3.713040	1.799455	0.648575
H	-0.631423	-0.969677	-2.734852
H	-2.313823	-1.446228	-2.447992
H	-1.102324	-2.669806	-2.753818
H	-5.028153	-2.612389	-0.600176

H	4.736575	-0.628456	-1.230653
H	4.348572	0.771312	-0.227179
H	3.587499	-0.716491	1.591979
H	4.042901	-3.159256	1.494556
H	2.881071	-2.694877	0.254765
H	4.563198	-3.001824	-0.185009
H	5.834403	-1.537871	2.262077
H	5.938182	0.076524	1.555006
H	6.411020	-1.336907	0.606942

CAM-B3LYP energy = -1154.92727282 a.u.

(2*S*,6*R*,7*S*,8*S*)-**1**, Conf. H

C	-2.703739	-2.777799	-0.064480
C	-3.111966	-1.340330	-0.151244
C	-3.105941	-0.433241	0.821697
C	-2.962307	-0.730468	2.285868
C	-0.372371	-1.905886	-0.391864
C	-1.356015	-2.973810	-0.797914
C	-1.994434	1.458344	-0.427449
C	-0.573262	1.261012	0.162286
C	0.349145	0.506152	-0.819945
C	-0.226101	-0.839019	-1.173299
O	-2.120086	2.879427	-0.704785
C	-1.104714	3.592836	-0.195536
C	-0.130410	2.669437	0.437001
O	-1.063230	4.792301	-0.278756
C	0.906090	3.115146	1.126551
C	-3.159467	1.042341	0.470201
C	0.251185	-2.087328	0.959722
O	-3.631082	-3.651870	-0.713977
O	1.645985	0.440664	-0.192825
C	2.717564	0.340760	-0.995106
O	2.634423	0.289336	-2.197678
C	3.999655	0.307390	-0.212583
C	4.141088	-0.924628	0.693039
C	4.088573	-2.218745	-0.112100
C	5.434408	-0.828591	1.494526
H	-2.591447	-3.087425	0.975651
H	-3.261419	-0.998930	-1.172925
H	-2.963795	-1.793569	2.512945
H	-2.046762	-0.294383	2.693550
H	-1.560167	-2.915344	-1.867874
H	-0.986257	-3.977371	-0.578478
H	-3.789990	-0.269757	2.830894
H	-0.621658	0.681596	1.081742
H	0.456454	1.092047	-1.732034
H	-0.721542	-0.854954	-2.137496
H	1.602587	2.443866	1.608343
H	1.074322	4.180510	1.221439
H	-3.133292	1.645534	1.379887
H	-2.088308	0.970085	-1.393108
H	-4.086630	1.291653	-0.049243
H	0.903196	-2.964761	0.944414
H	-0.510470	-2.283562	1.716598
H	0.844645	-1.234551	1.273425
H	-4.478566	-3.580183	-0.261858

H	4.818458	0.340083	-0.931248
H	4.045758	1.212514	0.396569
H	3.303787	-0.919793	1.394766
H	4.181880	-3.084547	0.544905
H	3.150966	-2.320383	-0.661647
H	4.906396	-2.256894	-0.835891
H	5.536564	-1.682150	2.166204
H	5.462835	0.080502	2.097502
H	6.302488	-0.820130	0.830914

CAM-B3LYP energy = -1154.92726565 a.u.

(2*S*,6*R*,7*S*,8*S*)-**1**, Conf. I

C	-3.308200	-2.388996	0.247380
C	-3.384846	-0.896416	0.339745
C	-2.951727	-0.112793	1.318789
C	-2.518928	-0.598474	2.668882
C	-1.019812	-1.849953	-0.765065
C	-1.844245	-2.867157	-0.020281
C	-1.716757	1.617768	-0.038621
C	-0.230150	1.393720	0.350014
C	0.402386	0.238243	-0.453537
C	-0.228596	-1.052887	-0.052550
O	-1.825307	3.006914	-0.451748
C	-0.655395	3.656087	-0.407201
C	0.392214	2.742768	0.110436
O	-0.551960	4.807902	-0.740541
C	1.621910	3.172549	0.339651
C	-2.771668	1.367648	1.038629
C	-1.277402	-1.724025	-2.235037
O	-4.188096	-2.766707	-0.813605
O	1.805401	0.227282	-0.099103
C	2.672085	-0.290162	-0.981474
O	2.340640	-0.705210	-2.065205
C	4.078599	-0.273625	-0.452016
C	4.270385	-1.104354	0.824645
C	3.917352	-2.569886	0.593459
C	5.702074	-0.956190	1.327154
H	-3.642023	-2.866099	1.172644
H	-3.682005	-0.434602	-0.597838
H	-1.457553	-0.401054	2.840568
H	-2.689086	-1.664577	2.806929
H	-1.894868	-3.818305	-0.556105
H	-1.380965	-3.064725	0.946822
H	-3.063277	-0.063533	3.450902
H	-0.155768	1.139870	1.408276
H	0.320538	0.434667	-1.519933
H	-0.119120	-1.262812	1.007557
H	2.386366	2.531283	0.751999
H	1.880103	4.199506	0.113678
H	-2.493831	1.906085	1.946689
H	-1.966437	1.034282	-0.922413
H	-3.704977	1.803974	0.678841
H	-0.628386	-0.997714	-2.718457
H	-2.317352	-1.448581	-2.423542
H	-1.122596	-2.691256	-2.718491
H	-4.185250	-3.726841	-0.886451

H	4.728446	-0.644110	-1.244850
H	4.348285	0.766043	-0.252918
H	3.596916	-0.703108	1.585623
H	4.049746	-3.146919	1.509742
H	2.881569	-2.694024	0.271695
H	4.561060	-3.006615	-0.174114
H	5.847563	-1.519095	2.250260
H	5.947639	0.088219	1.526769
H	6.414170	-1.334612	0.589735

CAM-B3LYP energy = -1154.92716303 a.u.

(2*S*,6*R*,7*S*,8*S*)-**1**, Conf. J

C	-3.544440	-2.216585	-0.095899
C	-3.561632	-0.729489	-0.273263
C	-3.395715	0.202431	0.660221
C	-3.440738	-0.037044	2.141081
C	-1.037894	-1.993353	-0.212538
C	-2.227791	-2.787000	-0.690665
C	-1.734769	1.677728	-0.535361
C	-0.468856	1.178843	0.208160
C	0.316189	0.138558	-0.621657
C	-0.558201	-1.028463	-0.992920
O	-1.475510	3.064015	-0.886448
C	-0.356191	3.536935	-0.318576
C	0.296375	2.448638	0.450970
O	-0.004254	4.678995	-0.457249
C	1.354095	2.670776	1.212780
C	-3.044495	1.620713	0.249921
C	-0.598270	-2.285110	1.191609
O	-4.671723	-2.865161	-0.684899
O	1.460935	-0.220927	0.175945
C	2.531479	-0.721711	-0.460807
O	2.586525	-0.843160	-1.658765
C	3.601759	-1.133866	0.511974
C	5.008922	-1.172766	-0.078333
C	5.507528	0.228026	-0.419897
C	5.961133	-1.870258	0.887662
H	-3.601607	-2.480880	0.957063
H	-3.537917	-0.415613	-1.315676
H	-2.476992	0.177173	2.610067
H	-3.731787	-1.050466	2.405760
H	-2.302210	-2.730683	-1.779167
H	-2.156299	-3.840623	-0.414013
H	-4.161427	0.647077	2.595847
H	-0.743179	0.706808	1.149507
H	0.682941	0.608410	-1.533915
H	-0.963311	-0.953442	-1.996055
H	1.814592	1.884229	1.793231
H	1.781152	3.664155	1.268059
H	-2.951829	2.255479	1.133251
H	-1.859290	1.162320	-1.483276
H	-3.826709	2.056541	-0.374301
H	-0.216131	-3.307877	1.247393
H	-1.442927	-2.235337	1.881600
H	0.179197	-1.614796	1.543657
H	-4.687321	-2.664663	-1.627926

H	3.559649	-0.476625	1.382383	H	4.121778	1.188174	0.494361
H	3.313794	-2.128991	0.865808	H	3.509852	-0.187629	1.385859
H	4.965023	-1.756502	-1.000803	H	5.252631	-0.385545	-1.107371
H	6.501526	0.186364	-0.867772	H	7.118224	-0.954660	0.441591
H	4.845818	0.730993	-1.125982	H	6.511210	0.674130	0.749781
H	5.573371	0.843522	0.481056	H	6.067145	-0.653957	1.825765
H	6.967971	-1.919758	0.470540	H	5.564260	-2.756905	-0.445722
H	5.635179	-2.889456	1.102591	H	3.856305	-2.412505	-0.707423
H	6.020021	-1.329473	1.835628	H	4.494173	-2.523302	0.937503

CAM-B3LYP energy = -1154.92704207 a.u.

CAM-B3LYP energy = -1154.92703326 a.u.

(2*S*,6*R*,7*S*,8*S*)-**1**, Conf. K

C	-2.908334	-2.752580	-0.036262
C	-3.265288	-1.311703	-0.235098
C	-3.308950	-0.350931	0.682756
C	-3.283402	-0.569448	2.167386
C	-0.519937	-1.963537	-0.195221
C	-1.501416	-3.019164	-0.638740
C	-2.044692	1.450530	-0.555301
C	-0.690832	1.254043	0.175379
C	0.299833	0.413460	-0.660148
C	-0.287698	-0.929576	-0.999543
O	-2.110620	2.858092	-0.910535
C	-1.124255	3.574776	-0.351625
C	-0.232036	2.665090	0.409162
O	-1.044092	4.766976	-0.491685
C	0.757104	3.125694	1.156210
C	-3.297302	1.103361	0.248714
C	-0.003646	-2.117417	1.204603
O	-3.863287	-3.649851	-0.603535
O	1.510558	0.339225	0.117496
C	2.659339	0.113061	-0.538019
O	2.714110	-0.049936	-1.731270
C	3.837715	0.135721	0.396451
C	5.031923	-0.687470	-0.080948
C	6.252185	-0.385610	0.782589
C	4.715084	-2.179908	-0.076458
H	-2.896345	-3.005110	1.021004
H	-3.323329	-1.018951	-1.282024
H	-2.385707	-0.139724	2.619255
H	-3.338948	-1.617582	2.450862
H	-1.598667	-3.004797	-1.726707
H	-1.187279	-4.022291	-0.343996
H	-4.132628	-0.054460	2.623117
H	-0.843101	0.733066	1.118462
H	0.532068	0.941408	-1.584825
H	-0.713285	-0.969523	-1.996034
H	1.394409	2.468044	1.729739
H	0.945365	4.190819	1.205037
H	-3.342190	1.756125	1.122645
H	-2.060513	0.917343	-1.501398
H	-4.166412	1.338724	-0.368329
H	0.622907	-3.011420	1.264468
H	-0.825239	-2.270847	1.906847
H	0.586874	-1.270095	1.538017
H	-3.929278	-3.474952	-1.549586

(2*S*,6*R*,7*S*,8*S*)-**1**, Conf. L

C	-3.564047	-2.193597	-0.097646
C	-3.576909	-0.710647	-0.271717
C	-3.389980	0.215380	0.663447
C	-3.420453	-0.026495	2.144411
C	-1.052650	-1.989140	-0.217280
C	-2.250686	-2.766938	-0.699866
C	-1.725242	1.685535	-0.533361
C	-0.460199	1.180749	0.207688
C	0.316420	0.134962	-0.622771
C	-0.568399	-1.023810	-0.994338
O	-1.461033	3.071423	-0.882948
C	-0.337372	3.538326	-0.319054
C	0.312600	2.446426	0.447661
O	0.019824	4.678730	-0.458269
C	1.374204	2.663106	1.205623
C	-3.034212	1.632456	0.253064
C	-0.609260	-2.294146	1.182797
O	-4.694755	-2.743427	-0.776305
O	1.458576	-0.233962	0.174216
C	2.527352	-0.737091	-0.463539
O	2.582428	-0.855636	-1.661809
C	3.596369	-1.155715	0.507919
C	5.005348	-1.180800	-0.079038
C	5.496941	0.226262	-0.404697
C	5.958962	-1.882971	0.882118
H	-3.617070	-2.460146	0.958878
H	-3.569607	-0.400338	-1.314014
H	-2.449342	0.176024	2.603204
H	-3.719219	-1.037359	2.410058
H	-2.337350	-2.697475	-1.785058
H	-2.179087	-3.824945	-0.433385
H	-4.128266	0.664880	2.608342
H	-0.734651	0.710999	1.150122
H	0.686570	0.602247	-1.534941
H	-0.978261	-0.940343	-1.994771
H	1.832794	1.874269	1.784473
H	1.806514	3.654294	1.259341
H	-2.938886	2.267145	1.136161
H	-1.853515	1.171815	-1.481672
H	-3.815456	2.070174	-0.370991
H	-0.232778	-3.319520	1.229562
H	-1.451153	-2.244641	1.876091
H	0.173471	-1.630907	1.536723
H	-4.657414	-3.703841	-0.706154

H	3.548868	-0.509016	1.385836
H	3.312436	-2.156519	0.848724
H	4.967034	-1.755120	-1.007633
H	6.492079	0.194800	-0.850851
H	4.834050	0.733156	-1.106910
H	5.557487	0.832645	0.502780
H	6.967019	-1.922883	0.466920
H	5.637937	-2.906012	1.085921
H	6.012742	-1.351507	1.835639

CAM-B3LYP energy = -1154.92699682 a.u.

(2*S*,6*R*,7*S*,8*S*)-**1**, Conf. M

C	-2.924917	-2.739452	-0.038793
C	-3.278894	-1.302436	-0.237571
C	-3.309739	-0.342111	0.681089
C	-3.278473	-0.558815	2.166008
C	-0.530577	-1.962067	-0.199081
C	-1.519805	-3.007745	-0.647665
C	-2.040190	1.456186	-0.556843
C	-0.687099	1.256293	0.174404
C	0.301031	0.412031	-0.660099
C	-0.293654	-0.927318	-1.001073
O	-2.102387	2.864148	-0.911693
C	-1.114218	3.578109	-0.352717
C	-0.224275	2.665983	0.407914
O	-1.030995	4.770193	-0.492261
C	0.766355	3.123858	1.154673
C	-3.294026	1.111874	0.246173
C	-0.012374	-2.123600	1.199172
O	-3.905320	-3.546531	-0.694124
O	1.510062	0.331707	0.119469
C	2.659563	0.103713	-0.534322
O	2.716291	-0.057153	-1.727719
C	3.835727	0.121807	0.403074
C	5.035208	-0.690425	-0.079449
C	6.251996	-0.390273	0.789624
C	4.726592	-2.184554	-0.089862
H	-2.907489	-2.990529	1.022615
H	-3.346238	-1.017301	-1.284747
H	-2.374611	-0.137198	2.613207
H	-3.342074	-1.606157	2.450555
H	-1.630533	-2.985408	-1.732658
H	-1.202117	-4.013878	-0.360886
H	-4.120500	-0.035048	2.625078
H	-0.841026	0.735998	1.117573
H	0.537217	0.939370	-1.584122
H	-0.721917	-0.962453	-1.996525
H	1.401958	2.464456	1.728100
H	0.957621	4.188453	1.203401
H	-3.338647	1.765376	1.119573
H	-2.057233	0.922996	-1.502882
H	-4.161864	1.348230	-0.372242
H	0.607020	-3.022857	1.255709
H	-0.833807	-2.271890	1.902637
H	0.585769	-1.281839	1.533120
H	-3.648434	-4.471475	-0.609017

H	4.114149	1.174571	0.513271
H	3.506598	-0.213397	1.388188
H	5.256549	-0.377625	-1.102483
H	7.121800	-0.951479	0.445239
H	6.505469	0.671083	0.767439
H	6.066083	-0.669443	1.829806
H	5.579544	-2.753267	-0.463242
H	3.870119	-2.415681	-0.724430
H	4.506020	-2.538855	0.920420

CAM-B3LYP energy = -1154.92697350 a.u.

(2*S*,6*R*,7*S*,8*S*)-**1**, Conf. N

C	-3.793183	-1.728638	0.064465
C	-3.577910	-0.257602	-0.106437
C	-3.185560	0.618162	0.814568
C	-3.151456	0.366809	2.293989
C	-1.310556	-1.920478	-0.270185
C	-2.652848	-2.499634	-0.640494
C	-1.400190	1.806068	-0.512970
C	-0.183991	1.081041	0.121946
C	0.342873	-0.043071	-0.797205
C	-0.745888	-1.040748	-1.092968
O	-0.931086	3.131839	-0.879630
C	0.287678	3.398232	-0.387115
C	0.792083	2.202782	0.333230
O	0.820097	4.465561	-0.543582
C	1.903342	2.238802	1.048095
C	-2.631204	1.962374	0.379234
C	-0.801146	-2.298907	1.089036
O	-5.004770	-2.180878	-0.546616
O	1.489512	-0.618422	-0.138303
C	2.341222	-1.321564	-0.902287
O	2.208491	-1.434097	-2.096127
C	3.444263	-1.946521	-0.095560
C	4.323505	-0.946986	0.666492
C	5.379523	-1.699938	1.468134
C	4.967052	0.064902	-0.275373
H	-3.805123	-1.998991	1.121370
H	-3.590226	0.059254	-1.146736
H	-3.593941	-0.583967	2.580391
H	-3.698651	1.160461	2.808649
H	-2.821588	-2.412378	-1.714738
H	-2.732252	-3.554618	-0.370790
H	-2.129570	0.398669	2.680491
H	-0.464424	0.626738	1.069803
H	0.686314	0.391947	-1.735466
H	-1.216751	-0.887391	-2.057405
H	2.251676	1.380137	1.603372
H	2.489126	3.148184	1.093666
H	-2.358125	2.557309	1.252982
H	-1.688763	1.338266	-1.449507
H	-3.375397	2.536080	-0.176194
H	-0.587403	-3.370907	1.110448
H	-1.561194	-2.122276	1.852700
H	0.104520	-1.770217	1.369053
H	-5.743258	-1.742584	-0.110294

H	2.982650	-2.638193	0.613174
H	4.052747	-2.530290	-0.786288
H	3.683801	-0.408837	1.369154
H	5.994592	-1.006578	2.043452
H	4.923172	-2.404906	2.164936
H	6.041297	-2.262142	0.804790
H	5.589088	0.767388	0.280982
H	4.221523	0.645846	-0.821497
H	5.602561	-0.438550	-1.008002

CAM-B3LYP energy = -1154.92675554 a.u.

H	4.817009	0.333594	-0.938125
H	4.047940	1.212683	0.387307
H	3.302790	-0.914440	1.394571
H	4.175873	-3.084040	0.551912
H	3.145155	-2.322430	-0.656406
H	4.900535	-2.262956	-0.832713
H	5.535016	-1.677940	2.166564
H	5.464484	0.084545	2.091137
H	6.301082	-0.822514	0.827118

CAM-B3LYP energy = -1154.92648280 a.u.

(2*S*,6*R*,7*S*,8*S*)-**1**, Conf. O

C	-2.712276	-2.779001	-0.054367
C	-3.116757	-1.339372	-0.141249
C	-3.105125	-0.430397	0.829073
C	-2.955063	-0.726643	2.292624
C	-0.374963	-1.905722	-0.390683
C	-1.359271	-2.974459	-0.792684
C	-1.994533	1.458700	-0.424631
C	-0.572560	1.261009	0.162695
C	0.347872	0.505321	-0.820806
C	-0.227739	-0.839856	-1.173527
O	-2.119460	2.879466	-0.703880
C	-1.102874	3.592753	-0.196809
C	-0.128472	2.669357	0.435610
O	-1.060531	4.792052	-0.281725
C	0.909017	3.115102	1.123636
C	-3.158242	1.044831	0.475698
C	0.247211	-2.085280	0.961853
O	-3.693828	-3.671165	-0.582499
O	1.645560	0.439276	-0.195390
C	2.716043	0.338505	-0.999028
O	2.631228	0.286496	-2.201470
C	3.999150	0.305243	-0.218214
C	4.139322	-0.923498	0.691992
C	4.083546	-2.220514	-0.108219
C	5.433710	-0.826803	1.491694
H	-2.599339	-3.086386	0.982181
H	-3.268386	-0.992043	-1.161779
H	-2.040540	-0.286148	2.697789
H	-2.952114	-1.789717	2.519948
H	-1.554181	-2.917702	-1.866034
H	-0.990527	-3.977987	-0.571893
H	-3.783540	-0.270243	2.840048
H	-0.619574	0.682287	1.082609
H	0.454210	1.091018	-1.733105
H	-0.721542	-0.856885	-2.138622
H	1.605589	2.443875	1.605401
H	1.077985	4.180454	1.217297
H	-3.129833	1.649129	1.384547
H	-2.090470	0.969304	-1.389559
H	-4.086255	1.294348	-0.042107
H	0.900143	-2.962021	0.948160
H	-0.514584	-2.281117	1.718645
H	0.839620	-1.231630	1.275109
H	-3.848294	-3.449695	-1.508228

(2*S*,6*R*,7*S*,8*S*)-**1**, Conf. P

C	-2.723549	-2.768970	-0.056912
C	-3.126018	-1.333518	-0.143378
C	-3.104707	-0.425320	0.827263
C	-2.950386	-0.720238	2.290748
C	-0.381160	-1.903882	-0.394779
C	-1.372475	-2.963988	-0.801209
C	-1.991403	1.462984	-0.425815
C	-0.569964	1.263442	0.162148
C	0.349260	0.506135	-0.820964
C	-0.231407	-0.836395	-1.175001
O	-2.114542	2.884202	-0.704200
C	-1.097234	3.595948	-0.196668
C	-0.123980	2.671068	0.435421
O	-1.053351	4.795281	-0.280850
C	0.914106	3.115122	1.123648
C	-3.155887	1.049883	0.473495
C	0.243909	-2.090745	0.955401
O	-3.739852	-3.566916	-0.666909
O	1.646183	0.435781	-0.194511
C	2.717373	0.335705	-0.997309
O	2.633781	0.285892	-2.199922
C	3.999754	0.300455	-0.215348
C	4.138615	-0.929746	0.693044
C	4.082787	-2.225465	-0.109256
C	5.432463	-0.834909	1.493845
H	-2.605224	-3.075836	0.983131
H	-3.284910	-0.993521	-1.163887
H	-2.031222	-0.285507	2.691650
H	-2.953321	-1.783056	2.519111
H	-1.581287	-2.898781	-1.869863
H	-0.999784	-3.969871	-0.589683
H	-3.773425	-0.257152	2.840764
H	-0.618056	0.684616	1.081952
H	0.458117	1.092155	-1.732762
H	-0.728506	-0.849028	-2.138391
H	1.609668	2.442703	1.605221
H	1.084618	4.180188	1.217749
H	-3.128000	1.654424	1.382206
H	-2.087755	0.974032	-1.390899
H	-4.083189	1.299611	-0.045438
H	-0.517112	-2.283973	1.713615
H	0.842174	-1.241281	1.268991
H	0.891712	-2.971277	0.937782
H	-3.456871	-4.488032	-0.652753

H	4.818275	0.329556	-0.934479
H	4.048472	1.206912	0.391645
H	3.301582	-0.921375	1.395046
H	4.174392	-3.090082	0.549552
H	3.144676	-2.326074	-0.658159
H	4.900187	-2.267067	-0.833335
H	5.532917	-1.687151	2.167451
H	5.463203	0.075482	2.094746
H	6.300323	-0.829939	0.829911

CAM-B3LYP energy = -1154.92640253 a.u.

(2*S*,6*R*,7*S*,8*S*)-**1**, Conf. Q

C	-4.043370	-1.552328	0.306162
C	-3.674859	-0.100170	0.218317
C	-3.116956	0.662164	1.150917
C	-2.968817	0.274145	2.590725
C	-1.643994	-1.820954	-0.542993
C	-2.788332	-2.466757	0.196563
C	-1.313834	1.750514	-0.235896
C	-0.000873	1.161616	0.350498
C	0.317737	-0.217235	-0.264440
C	-0.711691	-1.204481	0.177333
O	-0.963208	3.036606	-0.813487
C	0.336982	3.327767	-0.684041
C	1.009698	2.238831	0.064931
O	0.810174	4.343656	-1.122553
C	2.277472	2.339673	0.426002
C	-2.475918	1.972148	0.731657
C	-1.732654	-1.806538	-2.037892
O	-4.922903	-1.911937	-0.764146
O	1.613120	-0.617108	0.249498
C	2.291045	-1.538471	-0.453596
O	1.925059	-1.940526	-1.530987
C	3.523476	-2.015943	0.263725
C	4.527281	-0.919282	0.636580
C	5.699268	-1.532144	1.395757
C	5.008780	-0.158934	-0.594263
H	-4.533532	-1.781758	1.255641
H	-3.725733	0.286394	-0.796648
H	-3.380597	1.055995	3.233403
H	-1.915765	0.170727	2.865682
H	-3.108823	-3.395023	-0.279206
H	-2.465527	-2.707925	1.209169
H	-3.471117	-0.661299	2.829224
H	-0.100036	1.029107	1.428535
H	0.389549	-0.136600	-1.346318
H	-0.757658	-1.305522	1.258098
H	2.768485	1.576389	1.009726
H	2.849732	3.212613	0.138355
H	-2.120168	2.527873	1.601109
H	-1.657551	1.145222	-1.071736
H	-3.202358	2.605717	0.219905
H	-2.626604	-1.272935	-2.368869
H	-1.829998	-2.829319	-2.408677
H	-0.861856	-1.357705	-2.509876
H	-5.704625	-1.350604	-0.716052

H	3.191883	-2.527723	1.170688
H	3.998522	-2.755461	-0.381074
H	4.023055	-0.218780	1.305936
H	6.408465	-0.760665	1.698898
H	5.363043	-2.053488	2.293480
H	6.234270	-2.250081	0.769373
H	5.716473	0.622037	-0.312855
H	4.184953	0.318594	-1.127825
H	5.512940	-0.833030	-1.290961

CAM-B3LYP energy = -1154.92637423 a.u.

(2*S*,6*R*,7*S*,8*S*)-**1**, Conf. R

C	-4.063590	-1.532662	0.281810
C	-3.691744	-0.083934	0.213765
C	-3.119379	0.660262	1.151169
C	-2.965062	0.253646	2.585317
C	-1.647516	-1.817630	-0.516552
C	-2.796021	-2.445662	0.229205
C	-1.312838	1.754923	-0.225949
C	0.003230	1.167132	0.355229
C	0.316902	-0.215021	-0.254623
C	-0.712718	-1.197140	0.197316
O	-0.965997	3.043540	-0.800708
C	0.335368	3.333002	-0.681838
C	1.013356	2.242167	0.059647
O	0.806050	4.349215	-1.122471
C	2.284976	2.340540	0.407691
C	-2.474322	1.971897	0.743569
C	-1.736187	-1.817771	-2.011534
O	-4.932676	-1.772073	-0.827355
O	1.614304	-0.614672	0.254949
C	2.285912	-1.542137	-0.446023
O	1.912814	-1.950230	-1.518732
C	3.521479	-2.018092	0.266892
C	4.530892	-0.921851	0.625482
C	5.707326	-1.533281	1.378951
C	5.004719	-0.169746	-0.613374
H	-4.595927	-1.767287	1.207509
H	-3.757971	0.316935	-0.793990
H	-1.911381	0.159649	2.860683
H	-3.457375	-0.690497	2.810701
H	-3.089285	-3.402193	-0.210395
H	-2.489959	-2.642125	1.257148
H	-3.387310	1.021376	3.238357
H	-0.090178	1.039929	1.434481
H	0.383391	-0.139682	-1.337229
H	-0.757394	-1.288085	1.278822
H	2.780713	1.576112	0.985823
H	2.855612	3.212713	0.114550
H	-2.117550	2.519894	1.617523
H	-1.657375	1.151101	-1.062690
H	-3.198427	2.611511	0.236137
H	-0.857903	-1.387841	-2.487188
H	-2.621968	-1.274047	-2.347469
H	-1.849178	-2.842776	-2.371798
H	-5.213129	-2.692924	-0.803360

H	3.194378	-2.522507	1.179586
H	3.990205	-2.763498	-0.375761
H	4.033693	-0.216238	1.294755
H	6.420566	-0.761781	1.672402
H	5.376928	-2.048996	2.282056
H	6.235845	-2.255768	0.752297
H	5.716517	0.611004	-0.341884
H	4.178033	0.306918	-1.143274
H	5.501784	-0.848993	-1.310155

CAM-B3LYP energy = -1154.92627309 a.u.

(2*S*,6*R*,7*S*,8*S*)-**1**, Conf. S

C	-4.084993	-1.541452	0.237197
C	-3.708048	-0.098500	0.066870
C	-3.226519	0.737753	0.978503
C	-3.191547	0.462040	2.451076
C	-1.633423	-1.869790	-0.412838
C	-2.828030	-2.457547	0.294352
C	-1.320743	1.724436	-0.340523
C	-0.068269	1.169910	0.391233
C	0.301174	-0.236265	-0.127473
C	-0.745652	-1.203323	0.319386
O	-0.907104	2.965040	-0.974329
C	0.375026	3.269102	-0.734866
C	0.963452	2.236909	0.152240
O	0.893586	4.253936	-1.192421
C	2.177455	2.374842	0.657291
C	-2.555376	2.016339	0.511246
C	-1.623202	-1.966600	-1.907241
O	-4.880653	-1.984369	-0.866919
O	1.576193	-0.578611	0.467263
C	2.300889	-1.531684	-0.140189
O	1.960930	-2.056502	-1.172486
C	3.569235	-1.838884	0.604035
C	4.669048	-0.782022	0.406697
C	5.885942	-1.152449	1.247466
C	5.046878	-0.619638	-1.061580
H	-4.646925	-1.699841	1.161018
H	-3.680232	0.209066	-0.975607
H	-2.163302	0.390758	2.815342
H	-3.706997	-0.457717	2.720424
H	-3.116596	-3.423162	-0.124253
H	-2.579497	-2.611788	1.344214
H	-3.656208	1.286170	2.997707
H	-0.264878	1.085944	1.460327
H	0.423562	-0.216716	-1.207963
H	-0.861185	-1.226419	1.399533
H	2.593866	1.654495	1.345324
H	2.778825	3.233786	0.387432
H	-2.269261	2.639278	1.360687
H	-1.602465	1.065527	-1.158888
H	-3.238623	2.604559	-0.103745
H	-1.698532	-3.014725	-2.205604
H	-0.720367	-1.554177	-2.351213
H	-2.491497	-1.461471	-2.336191
H	-5.660871	-1.421763	-0.923458

H	3.337216	-1.924157	1.666318
H	3.925367	-2.806392	0.249357
H	4.284207	0.173506	0.770184
H	6.666370	-0.396334	1.150967
H	5.628727	-1.239264	2.304347
H	6.304240	-2.108113	0.922346
H	5.832468	0.129026	-1.172724
H	4.198975	-0.303684	-1.670836
H	5.420215	-1.561410	-1.471202

CAM-B3LYP energy = -1154.92626392 a.u.

(2*S*,6*R*,7*S*,8*S*)-**1**, Conf. T

C	-4.100350	-1.540326	0.206235
C	-3.729728	-0.098102	0.047754
C	-3.242110	0.729108	0.963320
C	-3.203997	0.443510	2.434022
C	-1.630190	-1.867561	-0.381895
C	-2.829733	-2.443213	0.324340
C	-1.331374	1.725245	-0.341870
C	-0.077109	1.183387	0.396421
C	0.299010	-0.227303	-0.104730
C	-0.747204	-1.191859	0.347810
O	-0.924813	2.967021	-0.978353
C	0.356443	3.277295	-0.743037
C	0.951563	2.251023	0.146532
O	0.869924	4.262515	-1.205622
C	2.168551	2.393754	0.643284
C	-2.572812	2.010911	0.502321
C	-1.609635	-1.981490	-1.875078
O	-4.882132	-1.877942	-0.941594
O	1.572153	-0.555317	0.501190
C	2.304954	-1.514884	-0.085707
O	1.968214	-2.067160	-1.104643
C	3.580232	-1.785550	0.660734
C	4.694327	-0.770617	0.349231
C	5.906611	-1.063086	1.226682
C	5.074904	-0.774004	-1.127281
H	-4.700155	-1.704047	1.105465
H	-3.715601	0.217627	-0.991877
H	-3.674528	1.260937	2.985793
H	-2.175950	0.376084	2.799294
H	-3.086577	-3.435030	-0.056044
H	-2.602079	-2.551784	1.385170
H	-3.715400	-0.480517	2.696787
H	-0.274105	1.110985	1.466378
H	0.427097	-0.220007	-1.184776
H	-0.868202	-1.202689	1.427345
H	2.591239	1.677598	1.331925
H	2.766095	3.252698	0.364979
H	-2.295306	2.634818	1.353956
H	-1.604654	1.060779	-1.158745
H	-3.253969	2.596441	-0.117472
H	-2.465508	-1.466625	-2.316966
H	-1.699291	-3.031390	-2.163148
H	-0.696045	-1.588622	-2.314766
H	-5.158563	-2.797027	-0.864095

H	3.368801	-1.772331	1.730210
H	3.912896	-2.786748	0.385168
H	4.322300	0.224202	0.605931
H	6.699589	-0.336501	1.044014
H	5.650132	-1.024135	2.286551
H	6.307992	-2.056718	1.012919
H	5.863027	-0.044560	-1.319181
H	4.229049	-0.527058	-1.769746
H	5.446822	-1.756743	-1.427420

CAM-B3LYP energy = -1154.92614292 a.u.

(2*S*,6*R*,7*S*,8*S*)-**1**, Conf. U

C	-3.453764	-2.172174	0.332974
C	-3.594216	-0.693201	0.148929
C	-3.274370	0.249148	1.031664
C	-3.051842	0.016389	2.497030
C	-1.272858	-1.843700	-1.019330
C	-2.459880	-2.732676	-0.705211
C	-1.708716	1.642313	-0.328883
C	-0.380184	1.267271	0.381045
C	0.262621	-0.001657	-0.220410
C	-0.650759	-1.171380	-0.055912
O	-1.522471	2.977287	-0.868832
C	-0.316427	3.487554	-0.586392
C	0.450556	2.511331	0.226188
O	0.016751	4.581364	-0.961253
C	1.640791	2.810735	0.719049
C	-2.974619	1.647207	0.530606
C	-0.957926	-1.715548	-2.480348
O	-4.673677	-2.891067	0.114051
O	1.486558	-0.208323	0.524919
C	2.482005	-0.871401	-0.083024
O	2.425560	-1.236780	-1.230939
C	3.632543	-1.117963	0.854093
C	4.981085	-1.292014	0.159695
C	5.443122	0.008011	-0.491636
C	6.018623	-1.802662	1.154052
H	-3.090589	-2.393004	1.337751
H	-3.783005	-0.389603	-0.879032
H	-3.309624	-0.991173	2.813975
H	-3.668781	0.713795	3.068677
H	-3.028780	-2.875472	-1.626263
H	-2.136175	-3.726947	-0.388425
H	-2.017581	0.211629	2.791239
H	-0.558898	1.073477	1.437859
H	0.521931	0.174397	-1.261176
H	-0.932126	-1.354081	0.975811
H	2.193662	2.121227	1.339762
H	2.083489	3.774894	0.503184
H	-2.842691	2.343063	1.361111
H	-1.869412	0.998473	-1.190634
H	-3.789769	2.026564	-0.087876
H	-1.750160	-1.169904	-3.000982
H	-0.916473	-2.708777	-2.933106
H	-0.008073	-1.222013	-2.669098
H	-5.317090	-2.592980	0.765943

H	3.671207	-0.311270	1.588251
H	3.375055	-2.025627	1.409083
H	4.855592	-2.042918	-0.623833
H	6.394161	-0.134473	-1.007012
H	4.720611	0.372283	-1.222935
H	5.585672	0.786907	0.261899
H	6.983723	-1.947193	0.666343
H	5.716353	-2.755771	1.591534
H	6.161279	-1.088863	1.969296

CAM-B3LYP energy = -1154.92612196 a.u.

(2*S*,6*R*,7*S*,8*S*)-**1**, Conf. V

C	-2.821607	-2.681629	0.350272
C	-3.299932	-1.269052	0.222719
C	-3.145306	-0.296696	1.117401
C	-2.788540	-0.503633	2.560168
C	-0.849381	-1.837349	-1.096456
C	-1.784365	-2.979964	-0.752616
C	-2.010176	1.446874	-0.268379
C	-0.593628	1.362798	0.362050
C	0.282151	0.282849	-0.310452
C	-0.338766	-1.064688	-0.143126
O	-2.159023	2.803789	-0.763785
C	-1.082150	3.563999	-0.525304
C	-0.070448	2.764641	0.208695
O	-1.023179	4.713813	-0.875248
C	1.050055	3.312066	0.649317
C	-3.196889	1.142619	0.648286
C	-0.656938	-1.607940	-2.566231
O	-3.858984	-3.652337	0.164951
O	1.557820	0.338027	0.372766
C	2.645937	-0.061062	-0.302802
O	2.607869	-0.447484	-1.444562
C	3.893015	0.084054	0.526498
C	5.022858	-0.862905	0.128506
C	6.315235	-0.457384	0.829364
C	4.665914	-2.313762	0.436695
H	-2.364812	-2.837364	1.328697
H	-3.612554	-0.995983	-0.783450
H	-1.807719	-0.088180	2.804074
H	-2.796635	-1.549662	2.856244
H	-2.356035	-3.231790	-1.648257
H	-1.226060	-3.879531	-0.482785
H	-3.509357	0.026192	3.187436
H	-0.665709	1.111800	1.419475
H	0.442495	0.535250	-1.355551
H	-0.513455	-1.329760	0.894244
H	1.778808	2.750668	1.215150
H	1.252413	4.356043	0.445846
H	-3.184536	1.832880	1.493602
H	-2.069974	0.807891	-1.146611
H	-4.107938	1.337875	0.080112
H	-0.411086	-2.553630	-3.054576
H	0.138950	-0.901053	-2.786179
H	-1.582983	-1.252131	-3.026840
H	-4.519057	-3.519395	0.853736

H	4.214844	1.123093	0.404143
H	3.633813	-0.036357	1.579624
H	5.170424	-0.766743	-0.949606
H	7.135434	-1.116083	0.540257
H	6.600615	0.565853	0.578709
H	6.205599	-0.519101	1.915054
H	5.467537	-2.984607	0.124045
H	3.754886	-2.622659	-0.077343
H	4.513825	-2.453005	1.510130

CAM-B3LYP energy = -1154.92600892 a.u.

(2*S*,6*R*,7*S*,8*S*)-**1**, Conf. W

C	-3.802188	-1.725788	0.074030
C	-3.583299	-0.254066	-0.094750
C	-3.183126	0.620114	0.823474
C	-3.140530	0.365663	2.301944
C	-1.313256	-1.917435	-0.270513
C	-2.656428	-2.495939	-0.638278
C	-1.400720	1.806563	-0.509079
C	-0.183108	1.081735	0.123013
C	0.342251	-0.042136	-0.797289
C	-0.746907	-1.039089	-1.093818
O	-0.932098	3.131711	-0.878493
C	0.287810	3.398381	-0.388820
C	0.793373	2.203597	0.331845
O	0.820194	4.465338	-0.547764
C	1.905997	2.240095	1.044552
C	-2.629193	1.964019	0.386408
C	-0.805516	-2.294660	1.089732
O	-5.068864	-2.164192	-0.417732
O	1.488533	-0.618745	-0.138815
C	2.339269	-1.322880	-0.902980
O	2.205992	-1.435375	-2.096766
C	3.441882	-1.948788	-0.096425
C	4.323073	-0.949668	0.663965
C	5.378238	-1.703208	1.466170
C	4.967776	0.059991	-0.279500
H	-3.812915	-1.997410	1.126523
H	-3.596692	0.069406	-1.134224
H	-2.117433	0.403672	2.684545
H	-3.576685	-0.588109	2.588212
H	-2.817918	-2.409691	-1.715262
H	-2.736244	-3.551413	-0.371062
H	-3.691266	1.154305	2.820523
H	-0.461245	0.627407	1.071492
H	0.685856	0.393293	-1.735300
H	-1.215869	-0.886680	-2.059404
H	2.255281	1.381899	1.599958
H	2.491973	3.149447	1.088197
H	-2.353360	2.558883	1.259298
H	-1.692052	1.337929	-1.444379
H	-3.374476	2.538211	-0.167011
H	-1.565828	-2.117503	1.852939
H	0.099774	-1.765595	1.370180
H	-0.591440	-3.366550	1.112204
H	-5.136001	-1.931982	-1.351245

H	2.979734	-2.638977	0.613392
H	4.049053	-2.534147	-0.786961
H	3.684502	-0.409626	1.366208
H	5.994560	-1.010030	2.040364
H	4.921069	-2.406556	2.164071
H	6.038920	-2.267281	0.803330
H	5.591039	0.762300	0.275696
H	4.222921	0.641298	-0.826159
H	5.602295	-0.445391	-1.011658

CAM-B3LYP energy = -1154.92596561 a.u.

(2*S*,6*R*,7*S*,8*S*)-**1**, Conf. X

C	-3.810255	-1.711282	0.069377
C	-3.592044	-0.243796	-0.098817
C	-3.181687	0.624616	0.820245
C	-3.133515	0.368540	2.298411
C	-1.318421	-1.911512	-0.271138
C	-2.663503	-2.481121	-0.644038
C	-1.396146	1.810348	-0.509657
C	-0.179617	1.084524	0.123485
C	0.343534	-0.040921	-0.795788
C	-0.749127	-1.033564	-1.092830
O	-0.926285	3.135662	-0.877475
C	0.293805	3.400785	-0.387766
C	0.798499	2.204987	0.331884
O	0.827226	4.467411	-0.545778
C	1.911899	2.239598	1.043478
C	-2.625865	1.967934	0.383747
C	-0.811375	-2.295167	1.087521
O	-5.068564	-2.056400	-0.513191
O	1.487497	-0.620929	-0.136279
C	2.336623	-1.327751	-0.899762
O	2.203166	-1.441111	-2.093442
C	3.438129	-1.954961	-0.092710
C	4.323784	-0.956093	0.662862
C	5.378446	-1.710263	1.465130
C	4.969448	0.048994	-0.284821
H	-3.819176	-1.983091	1.125749
H	-3.616180	0.077272	-1.137456
H	-3.575731	-0.582097	2.585629
H	-3.675028	1.161472	2.820147
H	-2.832053	-2.385727	-1.717561
H	-2.739910	-3.539896	-0.382349
H	-2.108371	0.397717	2.676270
H	-0.458634	0.631201	1.072187
H	0.689263	0.393179	-1.733632
H	-1.218760	-0.877324	-2.057371
H	2.260597	1.380581	1.597991
H	2.499158	3.148126	1.087029
H	-2.351431	2.563103	1.256889
H	-1.686782	1.342336	-1.445463
H	-3.370007	2.541874	-0.171422
H	0.094705	-1.768533	1.370139
H	-0.598623	-3.367471	1.105628
H	-1.571771	-2.120137	1.851058
H	-5.183562	-3.011373	-0.453088

H	2.974875	-2.641206	0.620172
H	4.042349	-2.544512	-0.782281
H	3.688212	-0.412123	1.364814
H	5.997763	-1.017157	2.036181
H	4.920735	-2.410414	2.165884
H	6.036311	-2.277962	0.802588
H	5.595693	0.751180	0.267160
H	4.225178	0.630784	-0.831752
H	5.601189	-0.460230	-1.016720

CAM-B3LYP energy = -1154.92589601 a.u.

(2*S*,6*R*,7*S*,8*S*)-**1**, Conf. Y

C	-3.865659	-2.015342	0.131854
C	-3.712289	-0.527043	0.007840
C	-3.356154	0.338080	0.949360
C	-3.280693	0.017767	2.411478
C	-1.362495	-1.939724	-0.457724
C	-2.471817	-2.728364	0.189295
C	-1.606182	1.661932	-0.293993
C	-0.292415	1.288677	0.445672
C	0.289114	-0.044087	-0.070983
C	-0.611946	-1.166909	0.321763
O	-1.389976	2.977366	-0.871052
C	-0.164062	3.457763	-0.626771
C	0.582103	2.490989	0.214670
O	0.198340	4.524638	-1.049341
C	1.789106	2.773845	0.675695
C	-2.889077	1.720599	0.534516
C	-1.285134	-1.975019	-1.954833
O	-4.689720	-2.536035	-0.911835
O	1.573859	-0.204320	0.574836
C	2.494992	-0.953412	-0.050042
O	2.323210	-1.432102	-1.143674
C	3.725665	-1.137605	0.794580
C	5.011170	-1.321751	-0.009410
C	5.385653	-0.044459	-0.754896
C	6.142867	-1.770454	0.909074
H	-4.399346	-2.282160	1.042434
H	-3.744009	-0.170828	-1.020100
H	-2.257595	0.107377	2.784993
H	-3.636778	-0.983861	2.643760
H	-2.595094	-3.711803	-0.267933
H	-2.226470	-2.883741	1.240187
H	-3.880670	0.731943	2.980570
H	-0.483937	1.175263	1.513360
H	0.450550	0.010739	-1.145020
H	-0.748815	-1.242197	1.396687
H	2.330917	2.097208	1.319350
H	2.257126	3.712697	0.407760
H	-2.724928	2.355153	1.407339
H	-1.761093	0.997950	-1.142017
H	-3.645386	2.209940	-0.081483
H	-1.319212	-3.008380	-2.304537
H	-0.375231	-1.518569	-2.337700
H	-2.134067	-1.459342	-2.413709
H	-4.314016	-2.298630	-1.766569

H	3.813135	-0.298104	1.485917
H	3.535917	-2.024820	1.406899
H	4.830690	-2.109073	-0.744945
H	6.287893	-0.193541	-1.349894
H	4.591795	0.275838	-1.431060
H	5.580263	0.769142	-0.051380
H	7.063415	-1.921415	0.343414
H	5.899484	-2.707542	1.412856
H	6.343265	-1.018501	1.676453

CAM-B3LYP energy = -1154.92580550 a.u.

References

1. Lee, K.H.; Min, Y.D.; Choi, S.Z.; Kwon, H.C.; Cho, O.R.; Lee, K.C.; Lee, K.R. *Arch. Pharm. Res.* **2004**, *27*, 1016-1019.
2. Vasas, A.; Lajter, I.; Kúsz, N.; Király, S.B.; Kovács, T.; Kurtán, T.; Bózsity, N.; Nagy, N.; Schelz, Z.; Zupkó, I.; Krupitza, G.; Frisch, R.; Mándi, A.; Hohmann, J. *Pharmaceutics* **2021**, *13*, 2088.
3. Raghavendra, S.; Tadiparthi, K.; Yadav, J.S. *Carbohydr. Res.* **2017**, *442*, 17-19.
4. Doboszewski, B.; Herdewijn, P. *Tetrahedron* **2008**, *64*, 5551- 5562.
5. Ivanova, N.A.; Valiullina, Z.R.; Shitikova, O.V.; Miftakhov, M.S. *Russ. J. Org. Chem.* **2007**, *43*, 742-764.
6. Kohn, H.; Abuzar, S.; Korp, J.D.; Zektzer, A.S.; Martin, G.E. *J. Heterocyclic. Chem.* **1988**, *25*, 1511-1517.
7. Błoszyk, E.; Dudek, A.; Kosturkiewicz, Z.; Rychłewska, U.; Daniewski, W.M.; Gumulka, M.; Nawrot, J.; Buděšínský, M.; Vašíčková, S.; Holub, M. *Collect. Czech. Chem. Commun.* **1989**, *54*, 1903-1918.
8. Manchand, P.S.; Blount, J.F. *J. Org. Chem.* **1978**, *43*, 4352-4354.
9. Li, J.; Tang, H.; Kurtán, T.; Mándi, A.; Zhuang, C.L.; Su, L.; Zheng, G.L.; Zhang, W. *J. Nat. Prod.* **2018**, *81*, 1645-1650.
10. Kobayashi, S.; Lu, C.; Hoye, T.R.; Hillmyer, M.A. *J. Am. Chem. Soc.* **2009**, *131*, 7960-7961.
11. Nishida, T.; Satoh, K.; Kamigaito, M. *Molecules* **2020**, *25*, 5890.
12. CHESHIRE CCAT, The Chemical Shift Repository for computed NMR scaling factors, http://cheshirenmr.info/index.htm. (condition as of Oct. 5. 2018 was considered)
13. a) Rablen, P.R.; Pearlman, S.A.; Finkbiner, J. *J. Phys. Chem. A* 1999, **103**, 7357-7363. b) Jain, R.J.; Bally, T.; Rablen, P. R. *J. Org. Chem.* **2009**, *74*, 4017-4023. c) Lodewyk, M.W.; Siebert, M.R.; Tantillo, D.J. *Chem. Rev.* **2012**, *112*, 1839-1862.
14. Konstantinov, I.A.; Broadbelt, L.J. *J. Phys. Chem. A* **2011**, *115*, 12364-12372.
15. Pierens, G.K. *J. Comput. Chem.* **2014**, *35*, 1388-1394.
16. Qiu, S.; de Gussem, E.; Tehrani, K.A.; Sergeyev, S.; Bultinck, P.; Herrebout, W. *J. Med. Chem.* **2013**, *56*, 8903–8914.
